# A cryptic START domain regulates deeply conserved transcription factors

**DOI:** 10.1093/plcell/koag267

**Published:** 2026-09-07

**Authors:** Courtney E Dresden, Ekaterina P Andrianova, Brian J Smith, Nicole I Callery, Dominic Kolonay, Ashton S Holub, Ricardo A Urquidi Camacho, Sarah G Choudury, Isabella J Higgins, Igor B Zhulin, Aman Y Husbands

**Affiliations:** Department of Biology, University of Pennsylvania, 415 S. University Ave, Philadelphia, PA 19104, United States; Molecular, Cellular, and Developmental Biology, The Ohio State University, 111 Biological Sciences Building, 484 W. 12th Avenue, Columbus, OH 43215, United States; Department of Microbiology, The Ohio State University, 105 Biological Sciences Building, 484 W. 12th Avenue, Columbus, OH 43215, United States; Department of Molecular Genetics, 105 Biological Sciences Building, 484 W. 12th Avenue, The Ohio State University, Columbus, OH 43215, United States; Department of Biology, University of Pennsylvania, 415 S. University Ave, Philadelphia, PA 19104, United States; Molecular, Cellular, and Developmental Biology, The Ohio State University, 111 Biological Sciences Building, 484 W. 12th Avenue, Columbus, OH 43215, United States; Department of Biology, University of Pennsylvania, 415 S. University Ave, Philadelphia, PA 19104, United States; Department of Molecular Genetics, 105 Biological Sciences Building, 484 W. 12th Avenue, The Ohio State University, Columbus, OH 43215, United States; Department of Biology, University of Pennsylvania, 415 S. University Ave, Philadelphia, PA 19104, United States; Department of Biology, University of Pennsylvania, 415 S. University Ave, Philadelphia, PA 19104, United States; Department of Biology, University of Pennsylvania, 415 S. University Ave, Philadelphia, PA 19104, United States; Department of Microbiology, The Ohio State University, 105 Biological Sciences Building, 484 W. 12th Avenue, Columbus, OH 43215, United States; Department of Biology, University of Pennsylvania, 415 S. University Ave, Philadelphia, PA 19104, United States; Molecular, Cellular, and Developmental Biology, The Ohio State University, 111 Biological Sciences Building, 484 W. 12th Avenue, Columbus, OH 43215, United States; Department of Molecular Genetics, 105 Biological Sciences Building, 484 W. 12th Avenue, The Ohio State University, Columbus, OH 43215, United States; Epigenetics Institute, University of Pennsylvania, 3400 Civic Center Boulevard, Philadelphia, PA 19104, United States

## Abstract

Transcription factors (TFs) integrate a diverse array of inputs to achieve the exquisite control of gene expression necessary for life. In plants, this is exemplified by the deeply conserved CLASS III HOMEODOMAIN LEUCINE ZIPPER (HD-Zip III) family of TFs. HD-Zip III activity is controlled by inputs at transcriptional, post-transcriptional, and post-translational levels. As part of their multidomain architecture, HD-Zip III TFs contain a StAR-related lipid transfer (START) domain, a ubiquitously distributed evolutionary module that binds various types of lipophilic ligands. Here, we show that HD-Zip III and HD-Zip IV proteins contain an additional cryptic, deeply conserved START domain which we term the disorder-containing START domain (dSTART). The dSTART domain is required for HD-Zip III developmental function, promoting homodimerization and controlling subcellular localization and DNA-binding competence. The dSTART domain also helps discriminate responsive from nonresponsive binding sites across the HD-Zip III shared genetic network. Finally, we identify candidate ligands of the dSTART domain including several species of phosphatidylglycerol. The identification and functional characterization of a cryptic START domain provides new mechanistic insights into a deeply conserved family of TFs with roles in nearly all aspects of plant development.

## Introduction

In eukaryotes, highly complex processes unfold in precise stereotypical ways (Kong et al. 2025). This is accomplished in large part by transcription factors (TFs) which integrate numerous genetic and environmental inputs to coordinate gene expression (Karin 1990). To achieve the required precision, these important regulators are often controlled by multiple distinct mechanisms (Latchman 1997; Pierre-Jerome et al. 2018). For instance, TFs are preferred targets of microRNAs in both plants and animals (Croft et al. 2012; Samad et al. 2017), and post-transcriptional regulation of TF activity is essential for organismal growth and development (Bartel 2004; Hobert 2008). At the protein level, TF activity is modulated by numerous inputs including interacting partners, various types of post-translational modifications, and, in some cases, binding of small-molecule ligands (Calkhoven and Ab 1996; vom Baur et al. 1996; Kornberg 1999; Chi et al. 2013; Filtz et al. 2014; Skelly et al. 2016). Understanding how highly regulated TFs integrate a diverse array of inputs to produce stable transcriptional outcomes is a perennial challenge, and mapping the full suite of inputs converging on a given TF is an essential prerequisite.

An excellent example of highly regulated TFs are the plant-specific members of the deeply conserved CLASS III HOMEODOMAIN LEUCINE ZIPPER (HD-Zip III) family. HD-Zip III proteins arose in the streptophyte lineage sometime after its divergence from chlorophytes over 700 million years ago, then proliferated throughout land plant evolution (Schrick et al. 2004; Romani et al. 2018; Wang et al. 2020; Morris et al. 2018). The HD-Zip III family in *Arabidopsis thaliana* has 5 paralogs—*REVOLUTA* (*REV*), *PHABULOSA* (*PHB*), *PHAVOLUTA* (*PHV*), *CORONA* (*CNA*), and *ATHB8*—that collectively impact nearly all aspects of development through functionally redundant and functionally divergent activities. Examples of redundancy include *PHB*, *PHV*, *REV*, and *CNA* promoting dorsal identity in lateral organs and xylem cell fate in the vasculature (Prigge et al. 2005). An example of functional divergence is *REV* and *CNA*, whose activities promote and antagonize the stem cell niche, respectively (Prigge et al. 2005). In keeping with their critical roles in development, HD-Zip III activity is regulated by the integration of multiple inputs. One such input is microRNA 166 (miR166), which spatially restricts HD-Zip III transcript accumulation (Rhoades et al. 2002; Kim et al. 2005). Loss of miR166 regulation leads to a wide range of pleiotropic HD-Zip III-dependent phenotypes including enlarged meristems, shortened roots, mispatterned vasculature, dorsalized leaves, and floral patterning defects (Skopelitis et al. 2017; Zhu et al. 2011; Merelo et al. 2016; Singh et al. 2017; Sakaguchi and Watanabe 2012; Roodbarkelari and Groot 2017). Other inputs into HD-Zip III activity include interacting proteins from several TF and chromatin remodeler classes (Chandler et al. 2007; Meng et al. 2017; Zhang et al. 2017; Husbands et al. 2023), as well as members of the LITTLE ZIPPER (ZPR) family of microProteins (Wenkel et al. 2007; Kim et al. 2008; Husbands et al. 2016). The latter are directly transactivated by HD-Zip III TFs and negatively regulate HD-Zip III activity through the formation of heteromeric complexes lacking DNA-binding capacity (Wenkel et al. 2007; Husbands et al. 2016).

HD-Zip III protein architecture consists of a DNA binding homeodomain (HD), a leucine zipper dimerization domain (LZ), and a C-terminal Per/Arn/Sim-like MEKHLA domain (Ponting and Aravind 1999; Mukherjee and Burglin 2006; Sessa et al. 2018). These proteins also contain a StAR-related transfer (START) domain suggesting additional levels of regulation. START domains are part of the StARkin domain superfamily, named for their kinship to the steroidogenic acute regulatory (StAR) protein (Wong and Levine 2016). StARkin domains are present throughout the tree of life and are defined by a conserved α/β helix grip fold structure that binds a variety of ligands, including sterols, phospholipids, and isoprenoids (Alpy and Tomasetto 2005; Radauer et al. 2008; Roderick et al. 2002; Tsujishita and Hurley 2000). Ligand binding triggers conformational changes which often directly modulate the activity of StARkin-containing multidomain proteins via a broad set of regulatory mechanisms (Dresden et al. 2021). The presence of a ligand-binding domain in HD-Zip III proteins led to the long-standing hypothesis that their activity is controlled by lipid ligands, perhaps analogous to nuclear receptors in metazoan systems (Schrick et al. 2004).

Supporting this, several types of phospholipids were recently identified as ligands for the HD-Zip III START domain including multiple species of phosphatidylcholine (PC). Mutations that prevent PC-binding abolish DNA-binding competence and hence HD-Zip III transcriptional output (Husbands et al. 2023). The START domain also promotes HD-Zip III homodimerization and increases the potency of target transactivation. These findings propose a model in which the START domain converts HD-Zip III proteins into transcriptionally potent, DNA-binding-competent TFs upon binding of their phospholipid ligand (Husbands et al. 2023). START-dependent effects on homodimerization have also been reported for the evolutionarily related HD-Zip IV family of TFs (Mukherjee et al. 2022; Nagata and Mitsutomo 2021). However, the HD-Zip IV START domain does not seem to affect DNA-binding competence (Mukherjee et al. 2022), instead impacting HD-Zip IV activity through modulation of subcellular localization and protein stability by ceramide and lyso-PC ligands (Iida et al. 2019; Nagata et al. 2021; Mukherjee et al. 2022; Schrick et al. 2023; Vijaya et al. 2024; Wojciechowska et al. 2024). These findings exemplify the flexibility of the START module, as even evolutionarily related sets of proteins can use dramatically different regulatory mechanisms.

The START domain also contributes to functional divergence of HD-Zip III paralogs. A recent study found that HD-Zip III TFs do not generate their paralog-specific transcriptional outcomes by binding to distinct sites in the genome (Holub et al. 2024). Instead, functional divergence results from differential usage of shared binding sites, with hundreds of uniquely regulated genes emerging from a commonly bound genetic network. After binding to a given site, several unknown factors determine whether an HD-Zip III TF produces a change in gene expression. If the site is considered “responsive” to the paralog, then transcription is either promoted or inhibited. If the site is considered “nonresponsive” to the paralog, then gene expression remains unchanged. Discrimination between responsive and nonresponsive binding sites depends in part on the START domain, however the regulatory properties driving this discrimination are unknown (Holub et al. 2024).

Downstream of the HD-Zip III START domain is a region with no known structural features, sometimes referred to as the HD-START-associated or START-adjacent domain (SAD or STAD; McConnell et al. 2001; Mukherjee and Burglin 2006). Here, we show that this region contains a cryptic START domain. This domain is present in all HD-Zip III proteins, as well as members of the HD-Zip IV subfamily, indicating a deep shared ancestry. Subsequent predictive modeling of HD-Zip III and HD-Zip IV multimers using AlphaFold2 similarly recognizes this cryptic domain and suggests a surprising possible mode of ligand binding (Vijaya et al. 2024; see Discussion). In addition to amino acids and structural features common to members of the START-like domain superfamily, the newly identified START domain possesses unique defining features. These include a highly conserved LPSGF motif as well as long insertions between 3 of its secondary structure elements. These insertions likely prevented its identification as a functional START-like domain using standard domain-detection tools. One insertion has properties of an intrinsically disordered region (IDR) prompting us to name this new domain the disorder-containing START domain (dSTART). Genetic and molecular analyses using PHB as a representative HD-Zip III TF reveal the dSTART domain is required for HD-Zip III developmental function, and controls subcellular localization and DNA-binding competence. In addition, genomic assays propose a role for the dSTART domain in helping HD-Zip III TFs discriminate between responsive and nonresponsive binding sites across their shared genetic network. Finally, we identify candidate phospholipid ligands of the dSTART domain. Our findings provide a wealth of new mechanistic insights into a deeply conserved, essential family of TFs with roles in nearly all aspects of plant development.

## Results

### HD-Zip III TFs contain a cryptic START domain

**Figure 1:**
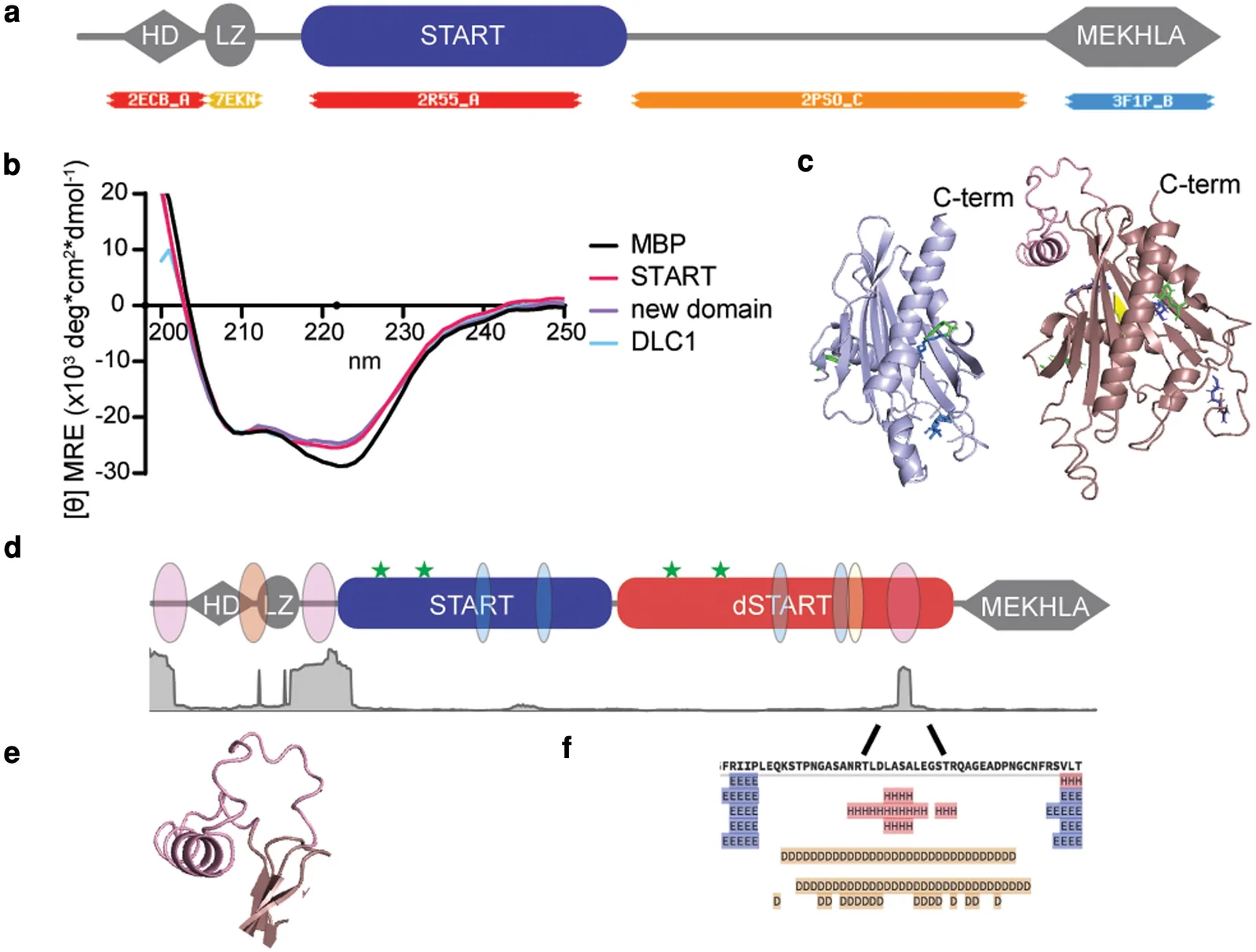
HD-Zip III proteins contain a cryptic, deeply conserved START domain defined by unique structural motifs and features. a) Classically defined HD-Zip III protein architecture. HHpred analyses show the region between the START and MEKHLA domains has homology to the StARkin protein STARD13 while the START domain resembles human STARD5. b) Circular dichroism of MBP-START, MBP-new domain, and MBP-DLC1 shows all 3 recombinant proteins have broadly similar secondary structure profiles. c) Homology-guided modeling using I-TASSER shows the PHB START domain (light blue) and new domain (light red) each adopt the helix-grip fold structure characteristic of START-like domains. Regions of interest include highly conserved tryptophan (green) and arginine or glutamine residues (blue), an LPSGF motif (yellow), and 3 largely unstructured loops or insertions (pink). d) PHB architecture (upper) with DEPICTER readout (lower; gray graph). Two regions of intrinsic disorder flank the HD and LZ, and a third falls within the disorder-containing START (dSTART) domain (pink ovals). Other regions of interest include highly conserved tryptophan residues (green stars), SDmut motifs (blue ovals), the LPSGF motif (yellow), and the nuclear localization signal (NLS) in the HD-LZ (orange oval). See Figure S10a for cartoon and full description of constructs used to test these features. e) I-TASSER homology model of the dSTART domain focusing on region of disorder predicted by DEPICTER (pink). f) Quick2D analysis indicates this region is predicted as both ordered and disordered. Predictions of order occur at an RTLDLASALE amino acid motif.

Genetic analyses have long suggested a regulatory role for the region downstream of the HD-Zip III START domain (Prigge et al. 2005). This region is sometimes referred to as the HD-START-associated domain (McConnell et al. 2001; Mukherjee and Burglin 2006), despite the lack of evidence of structural domain(s) (Fig. 1a). This hindered the molecular identification of new HD-Zip III regulatory inputs, prompting us to examine it more carefully for structural features that may have been missed by previous analyses. All *Arabidopsis* HD-Zip III paralogs (REV, PHB, PHV, CNA, and ATHB8) have similar architectures, and we selected PHB as a representative HD-Zip III protein. A PSI-BLAST search confirmed the 4 established domains of PHB: HD (Pfam ID PF00046), LZ (or bZIP_1; Pfam ID PF00170), START (Pfam ID PF01852), and MEKHLA (Pfam ID PF08670). By contrast, no domain was recognized within the ∼250 amino acid sequence separating the START and MEKHLA domains (residues 385 to 702). Searches initiated with this sequence against the Pfam and Conserved Domain Databases (CDD) also found no similarity to any known domain. We then conducted a more sensitive profile–profile matching search using HHpred (Soding et al. 2005). HHpred analyses identified several proteins from *Mus musculus* and *Homo sapiens* with high confidence support (>90% probability; PDB IDs of 5 best hits: 2PSO, 2MOU, 6L1M, 5I9J, 2R55; Fig. 1a), all of which contain a StARkin domain from the START-like domain superfamily (Pfam ID PF01852).

We next built a multiple sequence alignment (MSA) comparing the newly discovered domain (residues 399 to 681) with the PHB START domain (residues 168 to 384) and the mammalian START domains returned by HHpred (Figure S1). Consistent with MSAs of other distantly related StARkin domains (Ponting and Aravind 1999; Iyer et al. 2001), relatively few amino acids are universally conserved. Importantly, these include 2 conserved tryptophan residues—W427 and W494—common to nearly all START domains (Ponting and Aravind 1999). These residues sit at the base of the first 2 α-helices and their human analogs are proposed to be important for N-terminal helix alignment, structural stability, and/or lipid ligand loading (Roostaee et al. 2009; Thorsell et al. 2011). Mapping of secondary structure elements onto the MSA shows good agreement between the newly discovered domain and both mammalian and plant START sequences (Figure S1). To experimentally validate this prediction, we produced recombinant protein in *E. coli* and performed circular dichroism. These analyses confirm the new domain adopts a similar secondary structure profile as experimentally validated START domains from PHB and STARD12 (deleted in liver cancer-1; Thorsell et al. 2011; Husbands et al. 2023; Fig. 1b). Finally, homology modeling with iTASSER (Yang and Zhang 2015; Yang et al. 2015; Zheng et al. 2021)—and later AlphaFold2 (Senior et al. 2020)—predicts this new domain adopts the helix-grip fold structure characteristic of START-like domains (Fig. 1c). Taken together, our computational and experimental data indicate that the uncharacterized region between the START domain and MEHKLA domain of HD-Zip III proteins contains a cryptic START domain.

### The cryptic START domain is deeply conserved and has unique structural features

To determine the extent of its conservation and any potentially important structural features, we built an MSA of the new START domain using HD-Zip III sequences from across the plant kingdom. The domain is present in *Chlorokybus atmophyticus*, which belongs to one of the earliest-diverging extant streptophyte lineages in which an HD-Zip III protein has been identified (Figure S2). It is also present in members of the evolutionarily related HD-Zip IV subfamily, indicating a deep ancestry that predates the divergence of the 2 HD-Zip subfamilies (Figure S3). Our MSAs also reveal structural features unique to the new START domain. First is a 5 amino acid motif—LPSGF—which is absolutely conserved in >90% of the 2,800 protein sequences included in our analysis (Fig. 1c and d; Doc S1; see Methods). These residues partially overlap the seventh β-strand and are predicted to line the interior of the hydrophobic START binding pocket (Fig. 1c). The LPSGF motif is not present in other START domains, and no other motifs within the new START domain approach this level of conservation (Doc S1). Thus, LPSGF appears to be a major determinant of this new subfamily. Second, the new START domain is 30 to 80 amino acids larger than other described START domains. In HD-Zip III proteins, these additional residues manifest as 3 large insertions between the β1-β2, β7-β8, and α3-β2 secondary structure elements (Fig. 1c; Figures S1 and S2). By contrast, HD-Zip IV proteins only possess the α3-β2 insertion (Figure S4). These insertions are likely the reason this new START domain was not recognized by previous generations of domain discovery tools such as Pfam or CDD. Interestingly, the α3-β2 insertion is predicted to be an IDR (Fig. 1d; Figure S4). We thus renamed the cryptic START domain *disorder-containing START* (*dSTART*).

The internal IDR of the HD-Zip III dSTART domain has additional properties of interest. For instance, despite its prediction as an IDR, homology models reveal a small, weakly supported α-helix (Fig. 1e). To investigate this possibility, the PHB dSTART sequence was run through Quick2D which integrates results from secondary structure, transmembrane, and disorder prediction packages (Biegert et al. 2006). Interestingly, this package finds support for both intrinsic disorder and a small α-helix (RTLDLASALE) within the PHB dSTART IDR (Fig. 1f). This property is reminiscent of regions that undergo signal-dependent transitions between ordered and disordered states (Kopp and Postle 2020). In addition, our MSA shows minimal conservation of the IDR across plant evolution, with the notable exception of the predicted α-helical region (Figure S2). Similar analyses with the HD-Zip IV dSTART domain find only intrinsic disorder (Figure S4), suggesting the RTLDLASALE α-helix may have HD-Zip III-specific regulatory roles. Taken together, our analyses identify a new deeply conserved START domain with structural features that distinguish it from other START-like domains.

### START and dSTART domains have distinct patterns of evolutionary conservation

**Figure 2:**
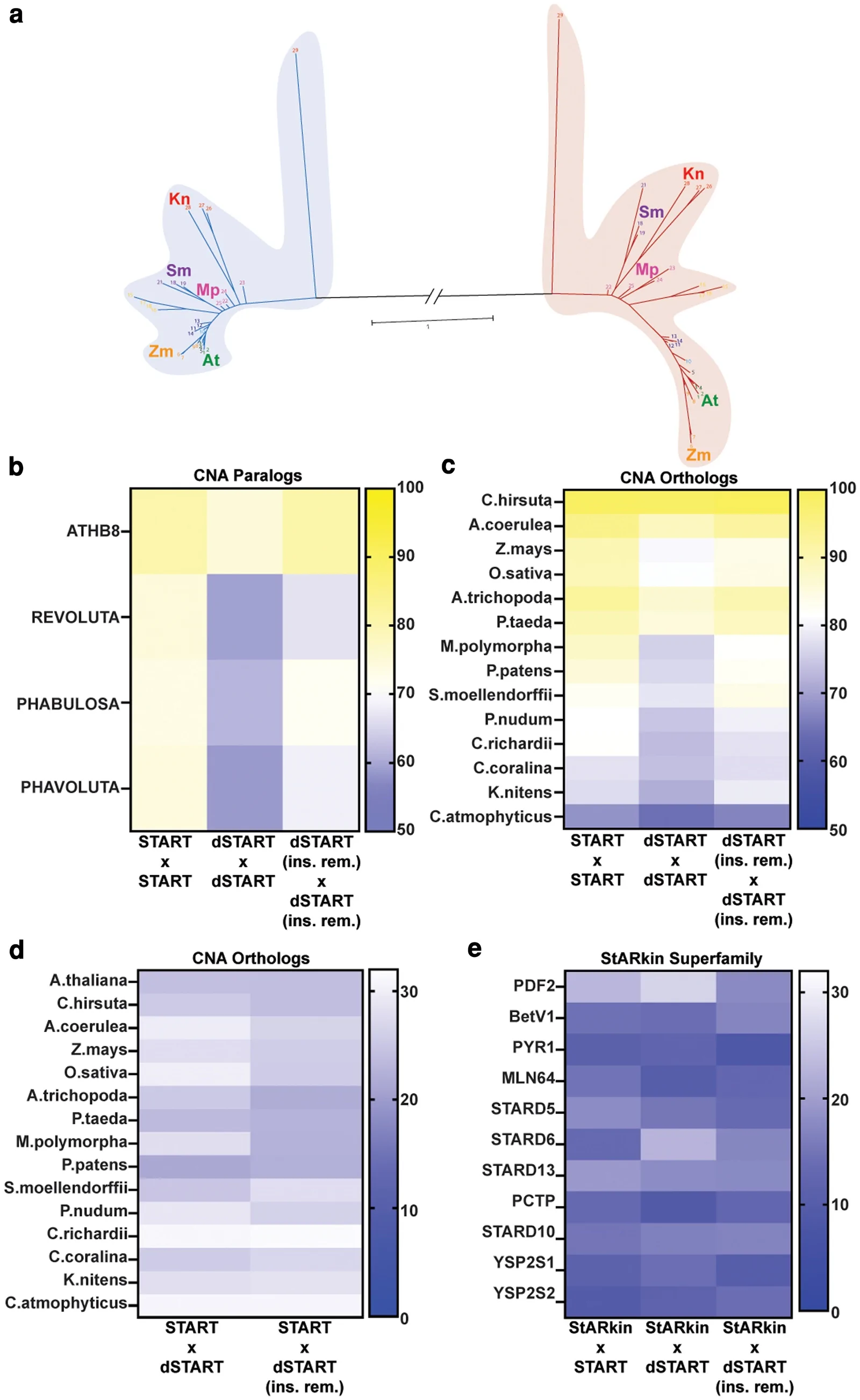
The START and dSTART domains have distinct patterns of evolutionary conservation. a) Maximum-likelihood unrooted phylogenetic tree showing that START (blue) and dSTART (red) domains sampled from across the plant kingdom cluster into distinct subclades. Numbers represent species (see Table S1a for species list) and their colors represent clades (light green: Brassicaceae, black: basal dicot, dark green: eudicots, orange: monocots, light blue: early-diverging angiosperms, dark blue: gymnosperms, pink: bryophytes, purple: lycophytes, yellow: ferns, and red: charophytes). Scale bar indicates expected number of amino acid substitutions per site over evolutionary time. Note: Tree was clipped to facilitate visualization. The full version and corresponding branch-support metrics can be found in Figure S6. b) Percent identity matrices comparing START, dSTART, and dSTART (no insertions) sequences of *A. thaliana* CNA with its paralogs. The dSTART domain is less conserved than the START domain, and this is only partly explained by its multiple insertions. c) Percent identity matrices comparing START, dSTART, and dSTART (no insertions) sequences of *A. thaliana* CNA with its orthologs in extant plant species similarly indicate weaker conservation of the dSTART domain. d) Percent identity matrices comparing dSTART and dSTART (no insertions) sequences of CNA orthologs with their respective START domains. Percent identities of START-dSTART pairs range from 21% to 31%, and removal of dSTART insertions usually lowers this conservation. e) Percent identity matrices comparing START, dSTART, and dSTART (no insertions) sequences of CNA with other members of the StARkin superfamily. Percent identities of START-dSTART pairs range from 10% to 22% and removal of dSTART insertions has variable effects on this conservation.

The START and dSTART domains are both present in the earliest-identified HD-Zip III protein indicating a deep conservation. To begin to map the evolutionary relationship of these 2 domains, we first constructed phylogenetic trees of the START and dSTART domains using sequences from CNA and orthologs across the plant kingdom (Fig. 2a; Figure S5). CNA was selected because recent analyses indicate the ancestral HD-Zip III gene most closely resembles CNA (Holub et al. 2024). This phylogeny shows START and dSTART form distinct subclusters supporting a deep evolutionary divergence between the domains (Fig. 2a). We then analyzed their patterns of conservation across the plant lineage by creating a series of percent identity matrices using sequences from HD-Zip III paralogs in *A. thaliana*, HD-Zip III orthologs in other plant species, and StARkin domains from nonplant species.

We first compared the *Arabidopsis* CNA sequence to its 4 paralogs, which fall into 2 evolutionary subclades: the REV clade (PHB, PHV, and REV) and the CNA clade (CNA and ATHB8) (Figure S7a; Table S1b; Prigge et al. 2005). The CNA START domain is relatively well conserved between paralogs, ranging from 73% identity for PHB to 80% identity for ATHB8 (Fig. 2b; Table S1b). By contrast, conservation is weaker for the CNA dSTART domain, particularly for members of the REV clade, with CNA and REV dSTART domains sharing only 62% identity (Fig. 2b; Table S1b). We hypothesized this weaker conservation may result from the 3 dSTART insertions, which vary in both size and amino acid composition (Figure S7b). To test this possibility, we removed the insertions from dSTART sequences. This qualitatively improved MSA alignments and increased percent identities between the paralogs (Fig. 2b; Figure S7c, Table S1b). However, these identities remained lower than those of the START domain suggesting divergence of the dSTART domain across the HD-Zip III subclades is only partially explained by these structural features.

We then determined the conservation patterns of the HD-Zip III START and dSTART domains across plant evolution by comparing CNA orthologs from several extant species (Figure S8a, Table S1c). Like the HD-Zip III paralogs, the START domain was more highly conserved than the dSTART domain in all ortholog comparisons (Fig. 2c; Table S1c). Conservation of dSTART between the orthologs was also increased by removing its insertions but rarely to levels seen with the START domain (Fig. 2c; Figure S8b and c, Table S1c). Thus, analyses with HD-Zip III paralogs and CNA orthologs both support a weaker evolutionary conservation of the dSTART domain which is only partially explained by its insertions. Interestingly, this pattern is reversed in an HD-Zip IV protein, whose dSTART domain consistently shows higher conservation than their START domain (Figure S9, Table S1d).

Finally, we used these percent identity matrices to investigate potential evolutionary origins of the START and dSTART domains. The START and dSTART phylogeny points to a deep evolutionary divergence (Fig. 2a) but does not distinguish between capture of 2 distinct domains versus duplication of a single ancestral domain. Both events are common mechanisms of domain architecture evolution (Forslund and Sonnhammer 2012). To distinguish between these possibilities, we compared START-dSTART pairs within CNA orthologs of extant plant species. Depending on the species, a given CNA START domain shares 21% to 31% (*µ* = 27%) identity with its adjacent dSTART domain (Fig. 2d; Table S1e). By contrast, similar comparisons find only 10% to 22% (*µ* = 14%) identity between other members of the StARkin superfamily and the CNA START or dSTART domain (Fig. 2e; Table S1f). These findings support the idea of an ancient duplication, rather than capture of 2 distinct START-like domains by the HD-Zip III or HD-Zip IV antecedent. Nevertheless, other scenarios, including transfers or rearrangements between the HD-Zip III and HD-Zip IV sub-families remain possible.

Taken together, our evolutionary analyses reveal distinct patterns of START and dSTART domain conservation within and between the HD-Zip subfamilies. In addition, the insertions that define the dSTART domain contribute to, but do not fully explain, the increased divergence of the dSTART domain in HD-Zip III proteins.

### START-like-defining residues within the dSTART domain are required for full PHB function

**Figure 3:**
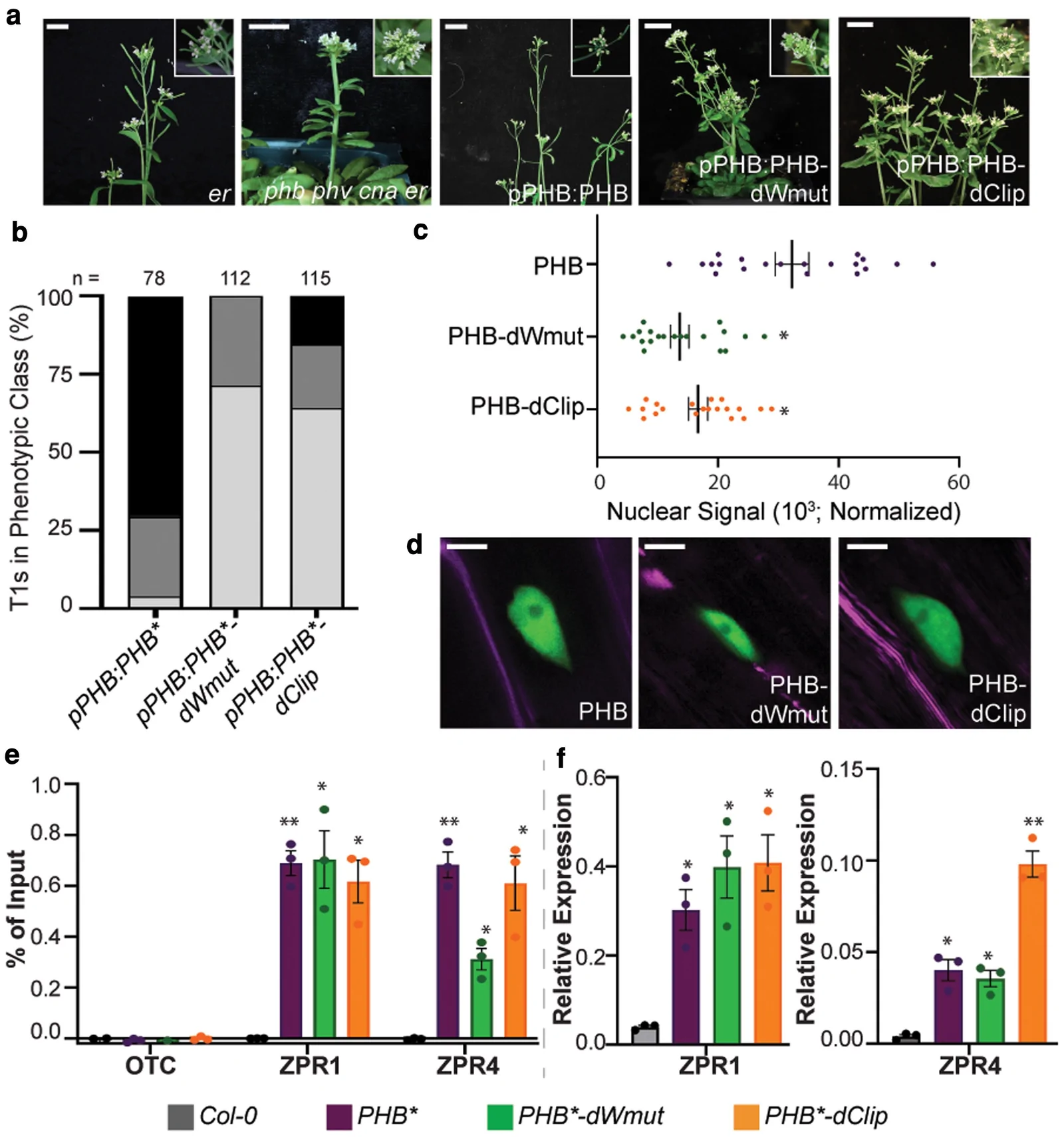
The dSTART domain promotes PHB homodimerization. a) In contrast to *er* single mutants, *phb phv cna er* quadruple mutants have fasciated stems, larger inflorescences, and much shorter siliques (Prigge et al. 2005). These phenotypes are fully complemented by the *pPHB:PHB* transgene, unaffected by the *pPHB:PHB-dWmut* transgene, and partially complemented by the *pPHB:PHB-dClip* transgene. b) Phenotypic scoring of primary transformants (*n* above bar) carrying miR166-insensitive (*) constructs. Ectopic PHB conditions strong gain-of-function phenotypes, while PHB-dWmut and PHB-dClip are dosage-sensitive hypomorphic variants. Categories range from severe (black) to moderate (dark gray) to mild/wildtype (light gray) phenotypes (following Husbands et al. 2023; category images in Figure S11a). c) Quantification of BiFC imaging showing PHB-dWmut and PHB-dClip have weaker signal than PHB. d) Confocal images of root cells demonstrating that PHB, PHB-dWmut, and PHB-dClip are nuclear localized. Note: all PHB variants have an mCitrine YFP-3xFLAG fusion at their C-terminus. Green = YFP, purple = propidium iodide. e) ChIP assay demonstrating PHB, PHB-dWmut, and PHB-dClip bind regulatory regions of *ZPR1* and *ZPR4*. All variants show significant enrichment over the *ORNITHINE TRANSCARBAMYLASE* (*OTC*) negative control locus. f) RT-qPCR assay demonstrating *ZPR1* and *ZPR4* are robustly transactivated by PHB, PHB-dWmut, and PHB-dClip in 24-h estradiol-induced lines. *n* = 3 biological replicates. *P-value ≤ 0.01, **P-value ≤ 0.001, 2-way paired Student's *t*-test against OTC (for ChIP) or Col-0 (for RT-qPCR). Additional statistical comparisons are available in Table S2a.

Next, we assessed dSTART-dependent effects on HD-Zip III developmental and molecular function using PHB as a representative member. We first tested the impact of the 2 tryptophan residues that are highly conserved across the START-like domain superfamily by mutagenizing them to alanines (dWmut; Fig. 1c; Figure S10a; Ponting and Aravind 1999). These mutations are predicted to minimally affect protein folding (Figure S10b), and circular dichroism confirmed wildtype and dWmut dSTART domains purified from *E. coli* have similar secondary structure profiles (Figure S10g). To assess potential regulatory roles in development, we repurposed 2 genetic assays previously used to characterize the START domains of PHB and CNA (Husbands et al. 2023; Holub et al. 2024). The first involves complementation of a *phb phv cna er* loss-of-function mutant with YFP-tagged *pPHB:PHB* reporter constructs. Tryptophan residues in the dSTART domain of *pPHB:PHB* were replaced with alanine residues to create *pPHB:PHB-dWmut*. As expected, *phb phv cna er* mutants transformed with *pPHB:PHB* were indistinguishable from *er* plants (Fig. 3a). By contrast, the *pPHB:PHB-dWmut* construct failed to complement the *phb phv cna er* mutant phenotype (Fig. 3a). This suggests PHB activity is either reduced or lost upon mutation of these tryptophan residues. To distinguish between these possibilities, we used the second genetic assay which involves transforming wildtype plants with miR166-resistant YFP-tagged pPHB:PHB* reporter constructs. This reporter conditions strong gain-of-function phenotypes owing to both misexpression and elevated dosage of PHB, providing a more sensitive readout of PHB activity than complementation assays (McConnel and Barton 1998; Husbands et al. 2023). Here, we similarly replaced tryptophan residues in pPHB:PHB* with alanine residues and transformed the resulting pPHB:PHB*-dWmut construct into wildtype plants. As expected, over 90% of pPHB:PHB* primary transformants show strong phenotypes consistent with ectopic HD-Zip III activity, including dorsalized leaves and enlarged meristems (Fig. 3b; Figure S11a). By contrast, the majority of pPHB:PHB*-dWmut seedlings were indistinguishable from wildtype, with ∼30% showing weak PHB gain-of-function phenotypes (Fig. 3b; Figure S11a). Thus, PHB-dWmut appears to be a hypomorphic variant whose activity can be partially restored through elevated dosage.

To test whether PHB-dWmut shows defects in known START-directed regulatory mechanisms (Dresden et al. 2021), we first cloned PHB* and PHB*-dWmut coding sequences into a bimolecular fluorescence complementation (BiFC) system. Localization of PHB-dWmut in infiltrated tobacco leaves was strictly nuclear indicating these conserved residues do not impact subcellular localization (Figure S12a). Interestingly, fluorescence intensity of PHB-dWmut was markedly lower than PHB (Fig. 3c), despite comparable accumulation (Figure S12b), suggesting reduced formation of PHB-dWmut dimers. We then cloned the PHB*-dWmut coding sequence into an established estradiol-inducible overexpression system (Husbands et al. 2023; Holub et al. 2024). Confocal imaging and western blotting of 24-h estradiol-induced lines similarly found PHB and PHB-dWmut accumulate solely in the nucleus, and to comparable levels (Fig. 3d; Figure S13). dSTART tryptophan residues thus do not control PHB activity through subcellular localization or protein stability, but rather by inhibiting productive self-association. The estradiol-inducible system also presents the opportunity to test whether PHB-dWmut dimers retain transcriptional functionality. Using chromatin immunoprecipitation (ChIP), we assayed PHB-dWmut occupancy at 2 known HD-Zip III targets, *ZPR1* and *ZPR4* (Wenkel et al. 2007; Kim et al. 2008; Husbands et al. 2016, 2023). PHB-dWmut ChIP signal intensities at *ZPR1* and *ZPR4* were indistinguishable or slightly lower than PHB, respectively (Fig. 3e). This indicates DNA-binding competency is not severely impacted in this variant. Target activation is unaffected, as *ZPR1* and *ZPR4* transcripts were induced to comparable levels as PHB (Fig. 3f). These findings suggest that the PHB-dWmut dimers that do form are functional. Supporting this, daily application of estradiol to PHB*- and PHB*-dWmut-inducible lines produced strong gain-of-function phenotypes with equal frequency (Figure S11b). Taken together, subcellular localization, protein stability, DNA-binding competence, and transactivation potential are largely unaffected in PHB-dWmut. Instead, its hypomorphic, dose-dependent activity likely results from compromised homodimerization.

### The dSTART α3-β2 IDR-containing insertion is required for full PHB function

The dSTART domain is defined by unique structural features including an IDR-containing insertion between its α3 and β2 secondary structure elements. To test whether this 41-amino-acid-long feature contributes to PHB dSTART regulation, we clipped 36 amino acids out of its center, creating an α3-β2 linker matching those of canonical START domains (dClip; Figure S10a; Ponting and Aravind 1999). This truncation is predicted to minimally affect protein folding at other regions of the dSTART domain (Figure S10c), and circular dichroism confirmed wildtype and dClip dSTART domains purified from *E. coli* have similar secondary structure profiles (Figure S10g). Amino acids were clipped from both *pPHB:PHB* and pPHB:PHB* reporters to create *pPHB:PHB-dClip* and pPHB:PHB*-dClip, respectively. In contrast to *pPHB:PHB*, the *pPHB:PHB-dClip* transgene only partially complemented the *phb phv cna er* quadruple mutant phenotype (Fig. 3a). Further, severe gain-of-function phenotypes were observed much less frequently in pPHB:PHB*-dClip primary transformants compared to those transformed with the pPHB:PHB* transgene (Fig. 3b). Thus, the α3-β2 insertion is required for full dSTART activity, but its effects appear to be weaker than those of the conserved dSTART tryptophan residues. The PHB*-dClip cassette was then cloned into the BiFC and estradiol-induction systems and tested for defects in START-directed regulatory mechanisms. Subcellular localization and protein stability of PHB-dClip were indistinguishable from PHB in both systems, ruling them out as regulatory mechanisms (Fig. 3c and d; Figures S11–S13). BiFC also revealed weaker fluorescence of PHB-dClip than PHB, suggesting this variant similarly forms fewer dimers (Fig. 3c; Figure S12). Critically, this reduction was less severe than PHB-dWmut, in line with the greater residual developmental activity of PHB-dClip. In estradiol-induced lines, PHB-dClip showed comparable ChIP signal to PHB at *ZPR1* and *ZPR4* loci (Fig. 3e), normal (or possibly elevated) transactivation of these targets (Fig. 3f), and strong gain-of-function phenotypes in response to daily application of estradiol (Figure S11b). Thus, like PHB-dWmut, PHB-dClip dimers that do form largely retain functionality. Taken together, the dSTART α3-β2 IDR-containing insertion is required for full PHB developmental function, and its removal creates a dosage-sensitive hypomorphic variant with compromised homodimerization.

### Motifs within the dSTART domain control PHB subcellular localization

**Figure 4:**
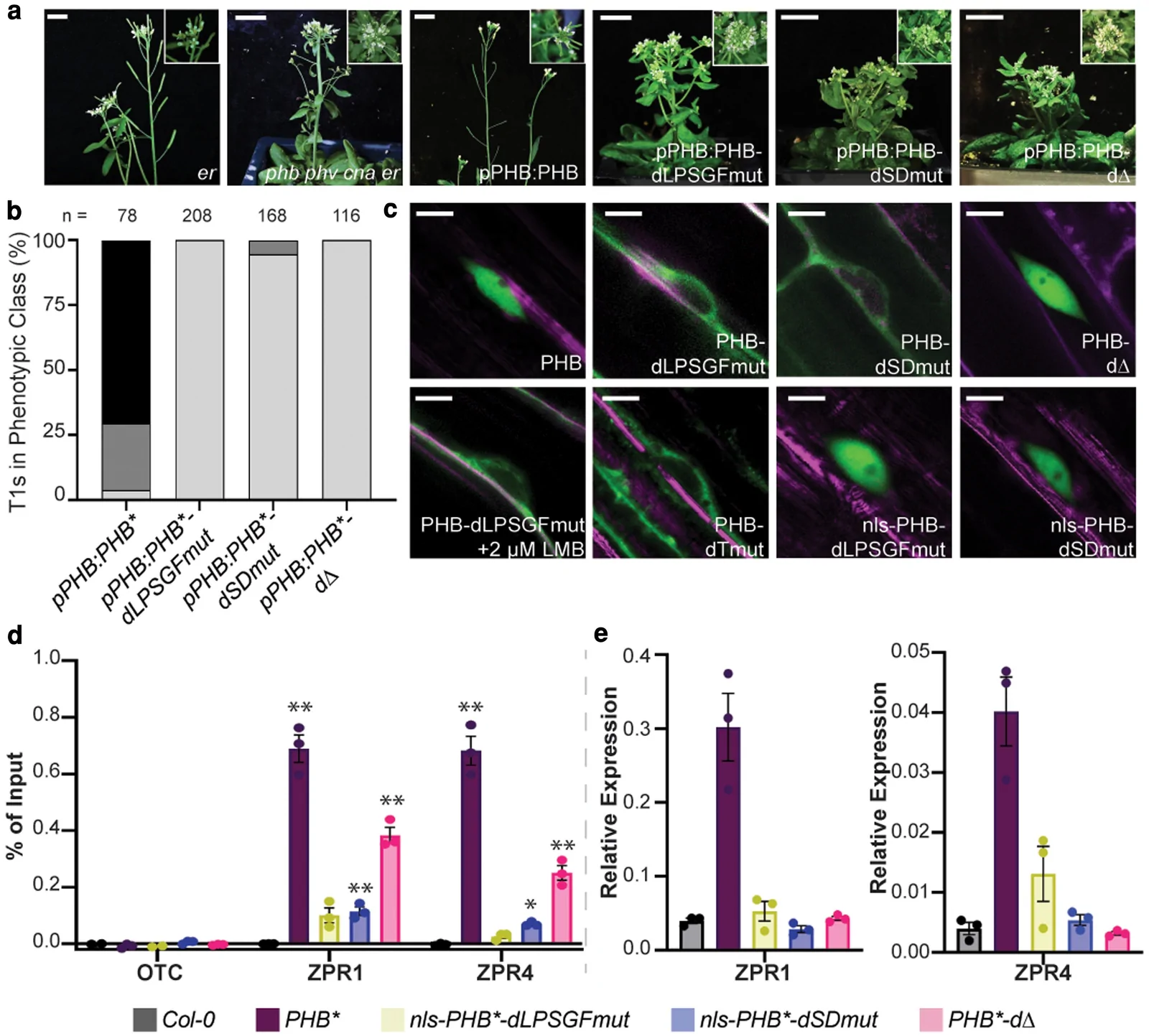
The dSTART domain controls PHB subcellular localization, DNA-binding competence, and transactivation potential. a) *phb phv cna er* pleiotropic phenotypes are fully complemented by the *pPHB:PHB* transgene and unaffected by *pPHB:PHB-dLPSGFmut; pPHB:PHB-dSDmut*, and *pPHB:PHB-dΔ* transgenes. b) Phenotypic scoring of primary transformants (*n* above bar) carrying miR166-insensitive (*) constructs. Ectopic PHB conditions strong gain-of-function phenotypes, while PHB-dLPSGFmut, PHB-dSDmut, and PHB-dΔ primary transformants resemble the wildtype. Categories range from severe (black) to moderate (dark gray) to mild/wildtype (light gray) phenotypes (following Husbands et al. 2023; category images in Figure S11a). c) Confocal images of root cells demonstrating that PHB-dLPSGFmut, PHB-dSDmut, and PHB-dTmut localize to the cytoplasm. Application of 2 µM Leptomycin B (LMB) fails to condition nuclear localization of PHB-dLPSGFmut. nls-PHB-dLPSGFmut and nls-PHB-dSDmut both localize to the nucleus. Note: all PHB variants have an mCitrine YFP-3xFLAG fusion at their C-terminus. Green = YFP, purple = propidium iodide. d) Binding to regulatory regions of *ZPR1* and *ZPR4* is strongly reduced for nls-PHB-dLPSGFmut and nls-PHB-dSDmut, while binding of PHB-dΔ is reduced by approximately half. e) Except for PHB, all nuclear-localized variants fail to upregulate *ZPR1* and *ZPR4* in 24-h estradiol-induced lines. *n* = 3 biological replicates. *P-value ≤ 0.01, **P-value ≤ 0.001, 2-way paired Student's *t*-test against OTC (for ChIP) or Col-0 (for RT-qPCR). Additional statistical comparisons in Table S2a.

The second defining feature of the dSTART domain is the highly conserved LPSGF motif lining the interior of its hydrophobic binding pocket (Fig. 1d). As the SGF residues within this motif show the strongest conservation (Doc S1), we tested their contribution to dSTART activity by mutating them into the physicochemically similar amino acids TAY (dLPSGFmut; Figure S10a; Castro-Chavez 2010). These mutations are predicted to minimally affect protein folding (Figure S10c), and circular dichroism confirmed wildtype and dLPSGF dSTART domains purified from *E. coli* have similar secondary structure profiles (Figure S10g). Mutations were then introduced into both *pPHB:PHB* and pPHB:PHB* reporters to create *pPHB:PHB-dLPSGFmut* and pPHB:PHB*-dLPSGFmut, respectively. Neither construct showed detectable developmental activity, with *pPHB:PHB-dLPSGFmut* failing to complement the *phb phv cna* er quadruple mutant (Fig. 4a), and pPHB:PHB*-dLPSGFmut failing to condition any gain-of-function phenotypes (Fig. 4b; Figure S11a), suggesting SGF-to-TAY mutations severely affect PHB function.

We next tested whether canonical START-directed regulatory mechanisms were affected by these mutations. PHB-dLPSGFmut showed no obvious defects in protein stability (Figure S13), but, remarkably, appears to be completely excluded from the nucleus (Fig. 4c). Several mechanisms could account for this phenotype. First, LPSGF may be a nuclear localization signal (NLS) directly mediating contact with import machinery. Second, the motif could contribute to cytoplasmic retention. For instance, if LPSGF is required to uncouple PHB from cytoplasmic retention factors, its mutation could create a dominant negative variant unable to enter the nucleus. Third, LPSGF may indirectly regulate subcellular localization, acting as part of an allosteric relay that gates access to the N-terminal HD-Zip III NLS. In this scenario, LPSGF mutations would prevent effective unmasking of the NLS, blocking nuclear import.

To distinguish between these 3 possibilities, we first analyzed the dSTART sequence using various localization–prediction tools, including NLS and nuclear export signal (NES) classifiers. No NLS sequences were predicted in the dSTART domain, arguing against the first hypothesis that LPSGF directly mediates contact with the nuclear import machinery. Our analyses also uncovered multiple putative NES-like sequences (Figure S14). Surprisingly, these include LPSGF, as well as DAPLL and RDMYLLQ, 2 motifs analogous to the amino acid stretches previously shown to control activity of the PHB START domain (Husbands et al. 2023). As mutating a bona fide NES should result in nuclear localization not nuclear exclusion, we considered the possibility that these NES-like signatures may be false positives, a known issue for short hydrophobic amino acid patches (Xu et al. 2012; Huang et al. 2017). Supporting this, the application of leptomycin B (LMB)—a potent inhibitor of nuclear export—did not result in nuclear localization of PHB-LPSGFmut (Fig. 4c; Figure S15). This suggests cytoplasmic retention or impaired access to the endogenous NLS as likelier models to explain the PHB-LPSGFmut cellular phenotype.

Cytoplasmic retention sequences are variable and context-dependent in nature. As such, they are not captured by traditional localization–prediction tools. However, these motifs often cluster with NES sequences (Yang et al. 1998; Patenaude et al. 2009; Zhu et al. 2022) or even act as NES motifs, themselves (Prigent et al. 2000). We therefore considered the possibility that—rather than dLPSGFmut behaving as a dominant negative—LPSGF, DAPLL, and RDMYLLQ instead act as redundant cytoplasmic retention points. This model makes 3 testable predictions. First, mutation of DAPLL and RDMYLLQ should phenocopy PHB-dLPSGFmut, as such a variant would possess a functional retention motif (LPSGF) but be unable to relieve LPSGF-mediated cytoplasmic retention. Second, complete removal of the dSTART domain—and thus all 3 cytoplasmic retention sequences—should restore nuclear localization. Third, simultaneous mutation of the 3 motifs should disrupt the contact with cytoplasmic retention factors and similarly restore nuclear localization. To test the first prediction, we mutagenized DAPLL and RDMYLLQ into AVVAA and GAVVGAG, respectively (dSDmut), following previous work (Figure S10a, Husbands et al. 2023). After confirming a broadly similar secondary structure profile for dSDmut (Figure S10e and g), these mutations were introduced into both *pPHB:PHB* and pPHB:PHB* reporters, to create *pPHB:PHB-dSDmut* and pPHB:PHB*-dSDmut, respectively. Neither construct had detectable effects on development (Fig. 4a and b; Figure S11a), and PHB-dSDmut is completely retained in the cytoplasm, mirroring the accumulation pattern of PHB-dLPSGFmut and satisfying the first prediction (Fig. 4c). To test the second prediction, we removed the dSTART domain from both *pPHB:PHB* and pPHB:PHB* reporters, to create *pPHB:PHB-dΔ* and pPHB:PHB*-dΔ, respectively (Figure S10a). Neither construct showed detectable developmental activity (Fig. 4a and b; Figure S11a), suggesting compromised function of PHB-dΔ. More importantly, the accumulation pattern of PHB-dΔ is strictly nuclear, satisfying the second prediction (Fig. 4c). To test the third prediction, we created a triple mutant carrying both dLPSGFmut and dSDmut sets of mutations (dTmut). After confirming a broadly similar secondary structure profile for dTmut (Figure S10f and h), *PHB-dTmut*-expressing lines were examined by confocal imaging. Contrary to model expectations, PHB-dTmut remained excluded from the nucleus (Fig. 4c). Thus, our data do not support a model in which LPSGF, DAPLL, and RDMYLLQ function as redundant cytoplasmic retention motifs.

Three testable predictions similarly emerge if the dSTART domain regulates functional exposure of the HD-Zip III NLS. First, both PHB-SDmut and PHB-dTmut should be cytoplasmically localized, as the allosteric relay is compromised in either case. Second, deletion of the dSTART domain should restore nuclear localization, as this would eliminate masking entirely and render the NLS constitutively accessible. Third, the addition of an exogenous NLS should bypass the regulatory role of the dSTART domain and restore nuclear accumulation in the motif mutants. The first 2 predictions are satisfied by existing data (Fig. 4c). To test the third prediction, we fused a *NLS* tag to the N-termini of *PHB-dLPSGFmut* and *PHB-dSDmut* cassettes. Cassettes were cloned into the estradiol-inducible system, and confocal imaging revealed that both nls-PHB-dLPSGFmut and nls-PHB-dSDmut indeed accumulate in the nucleus (Fig. 4c). Taken together, our findings support a model in which 3 conserved motifs within the dSTART domain control HD-Zip III subcellular localization via allosteric regulation of their NLS.

### Motifs within the dSTART domain control PHB DNA-binding competence

StARkin domains impact biological processes through a diverse set of regulatory mechanisms, and a given StARkin domain often uses multiple independent mechanisms (reviewed in Dresden et al. 2021). For instance, the START domain of PHB impacts homodimerization, transactivation potential, and DNA-binding competence (Husbands et al. 2023). If the HD-Zip III NLS is shielded from exposure in PHB-dLPSGFmut and PHB-dSDmut, these variants should also have compromised transcriptional functionality, given the substantial overlap between the NLS and the DNA-binding HD (Fig. 1d). The nls-PHB-dLPSGFmut and nls-PHB-dSDmut variants present an opportunity to test this hypothesis. We first assayed the DNA-binding capabilities of these proteins by measuring occupancy at *ZPR1* and *ZPR4* loci. Strikingly, ChIP signal intensities were sharply reduced for nls-PHB-dLPSGFmut and nls-PHB-dSDmut relative to PHB, particularly at the *ZPR4* locus (Fig. 4d). This reduction is not explained by differences in protein abundance between PHB, nls-PHB-dLPSGFmut, and nls-PHB-dSDmut (Figure S13), indicating these mutations compromise DNA binding. In addition, both variants fail to upregulate *ZPR1* and *ZPR4* targets (Fig. 4e), suggesting this reduced binding capacity has functional consequences. These findings propose the LPSGF, DAPLL, and RDMYLLQ motifs contribute to HD-Zip III DNA-binding competence and target regulation, lending additional support to a model in which the dSTART domain regulates functional exposure of the HD-Zip III NLS.

### The dSTART domain facilitates the differential usage of shared binding sites

Functional divergence of 2 HD-Zip III paralogs—CNA and PHB—results from differential usage of shared binding sites, with hundreds of uniquely regulated targets emerging from a commonly bound genetic network (Holub et al. 2024), reviewed in (Higgins et al. 2025). Deletions, chimeras, and evolutionarily guided amino acid substitutions show that START domains partially license this phenomenon by helping to distinguish responsive from nonresponsive binding sites (Holub et al. 2024). Given their evolutionary relatedness (Fig. 2), it is conceivable the dSTART domain also contributes to the differential usage of shared binding sites by HD-Zip III TFs.

**Figure 5:**
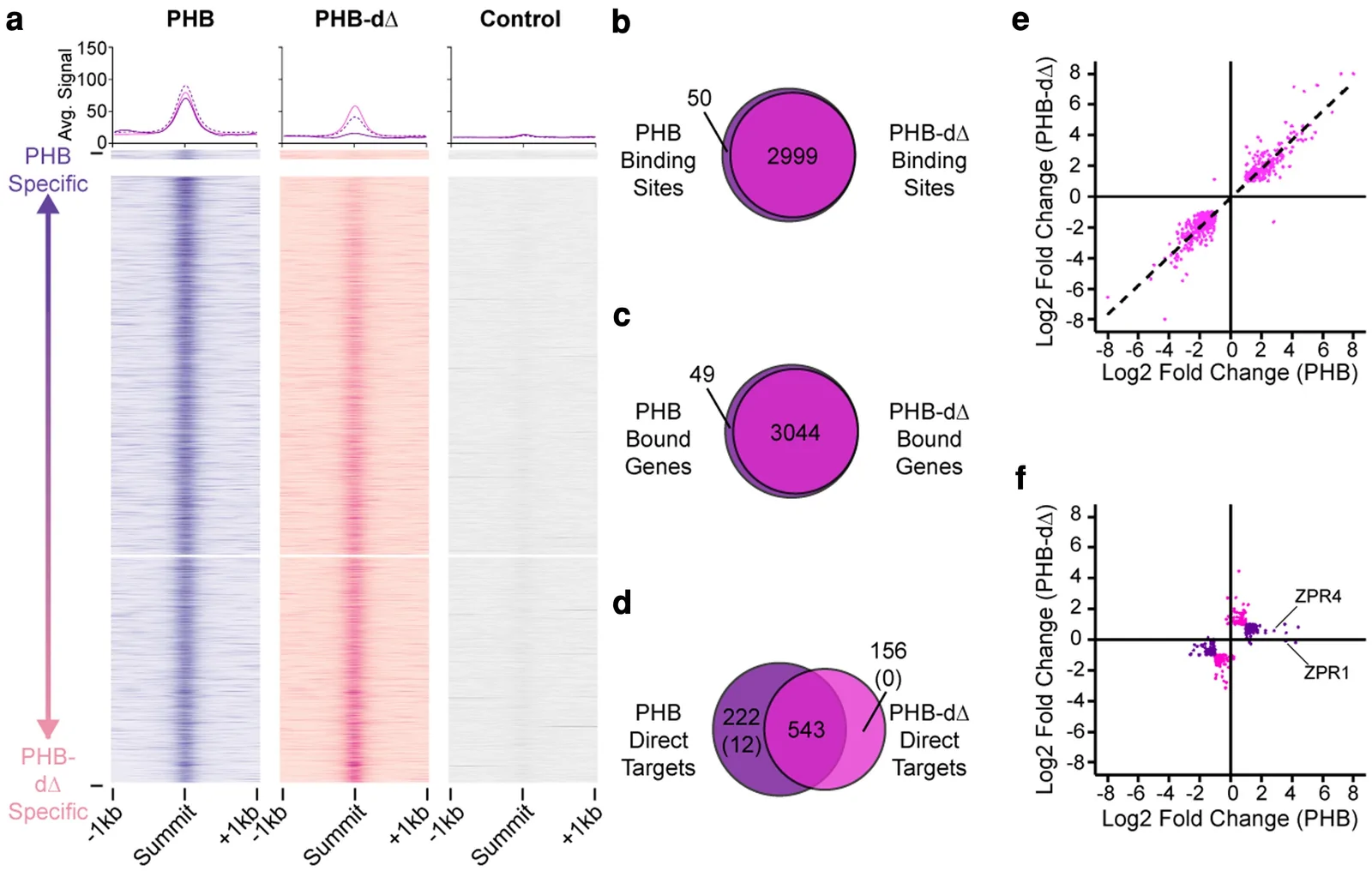
The dSTART domain helps distinguish responsive from nonresponsive PHB binding sites. a) Histograms and heatmaps of ChIP-seq signal intensities compared to a nontransgenic control (gray). Histogram lines and colors delineate 4 categories of binding sites: PHB uniquely bound (purple, solid); mutually bound—PHB higher affinity (purple, dashed); mutually bound—equal affinities (magenta); and mutually bound—PHB-dΔ higher affinity (pink, dashed). Heatmaps are separated into 2 major categories: PHB uniquely bound (upper) and mutually bound (lower). Mutually bound heatmaps contain two further subcategories: mutually bound—PHB higher affinity (upper); and mutually bound—equal affinities (lower). b) Venn diagram of sites bound by PHB and PHB-dΔ. c) Venn diagram of genes bound by PHB and PHB-dΔ. d) Venn diagram of direct targets of PHB and PHB-dΔ, ie bound in ChIP-seq and differentially expressed in RNA-Seq. Numbers in parentheses correspond to direct targets bound specifically by PHB or PHB-dΔ. e) Scatterplot showing differential expression of genes bound and regulated by both PHB and PHB-dΔ. f) Scatterplot showing differential expression of genes bound by both paralogs but uniquely regulated by PHB (purple) or PHB-dΔ (pink).

To test this, we used 24 h-estradiol-induced lines to determine how loss of the dSTART domain affects binding and regulation of PHB targets genome wide. ChIP-seq identified 3,049 and 2,999 sites bound by PHB and PHB-dΔ (Fig. 5a and b; Table S2b), corresponding to 3,093 and 3,044 bound genes, respectively (Fig. 5c). The binding profiles of PHB and PHB-dΔ are virtually identical, with ∼98% of genes bound by PHB also bound by PHB-dΔ (3,044 out of 3,093). However, clear differences were observed in ChIP signal intensities across their 2,999 mutually bound sites. ChIP signal intensities fell into 3 categories: higher signal for PHB (1,863 out of 2,999), higher signal for PHB-dΔ (1 out of 2,999), and equivalent signal for both variants (1,135 out of 2,999; Fig. 5a and b). Thus, like the PHB and CNA START domains (Holub et al. 2024), one function of the dSTART domain may be to increase DNA binding affinities in a site-specific manner.

We then tested how loss of the dSTART domain impacts regulation of PHB targets by performing RNA-seq on the same 24 h-estradiol-induced lines. After induction, 1,758 and 1,707 genes were differentially expressed in PHB and PHB-dΔ lines, respectively (Table S2c, d). Genes that were also bound in ChIP assays were called as direct targets. This corresponds to 777 and 699 high-confidence direct targets of PHB and PHB-dΔ, respectively, which fall into 3 categories: PHB-specific, PHB-dΔ-specific, and mutually regulated (Fig. 5d; Table S2e). Several mechanistic conclusions can be drawn from these data. First, mutual targets of PHB and PHB-dΔ are almost all regulated in the same direction (Fig. 5e). This indicates the dSTART domain does not control the valence of PHB target regulation. Second, the magnitudes of gene expression changes induced by PHB and PHB-dΔ are similar, demonstrating that deletion of the dSTART domain does not impair transcriptional potency. This differs from the START domain whose loss reduces transcriptional potency of both CNA and PHB (Husbands et al. 2023; Holub et al. 2024). Related to this, the majority of PHB and PHB-dΔ direct targets fall into the mutually regulated category, despite the reduced binding affinity of PHB-dΔ (Fig. 5d). This indicates that the effects of PHB-dΔ hypomorphic DNA binding can be overcome by increasing dosage. Third, and most importantly, despite having virtually identical binding profiles, PHB and PHB-dΔ each have hundreds of uniquely regulated targets (Fig. 5d and f). Specifically, 27% of PHB-specific targets (210 out of 777) and 22% of PHB-dΔ-specific targets (156 out of 699) are also bound by the other variant. These PHB-specific targets include *ZPR1* and *ZPR4*, which remain bound by PHB-dΔ, but are not upregulated by this variant (Fig. 5f). As transcriptional potency is unaffected in PHB-dΔ (Fig. 5e), the dSTART domain thus helps discriminate between responsive and nonresponsive binding sites. Taken together, these and previous findings (Holub et al. 2024) demonstrate the differential usage of shared binding sites by HD-Zip III TFs is mediated by both their START and dSTART domains.

### The dSTART domain binds phosphatidylglycerol

**Figure 6:**
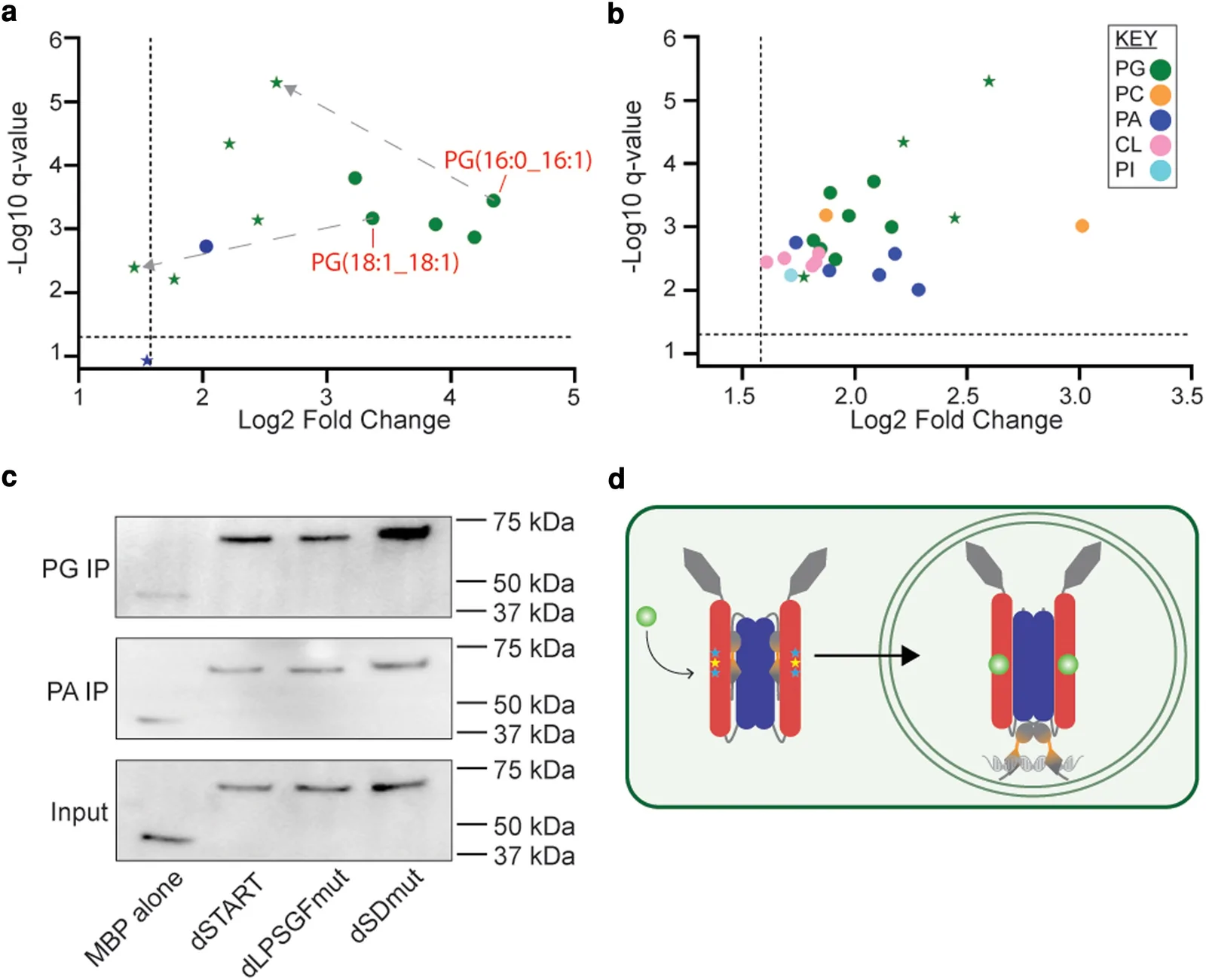
The dSTART domain binds multiple species of phosphatidylglycerol (PG) and phosphatidic acid (PA). a) LC-MS analysis of lipids bound by recombinant dSTART protein in *E. coli*. Five species of PG (green circles) and one of PA (blue circle) are significantly enriched (≥3-fold enrichment, *q* ≤ 0.01) over the negative control. After incubation with plant liposomes, these lipid species decrease in abundance (light green or blue stars). To illustrate this, 2 lipids, before and after liposome incubation, are labeled with red text and gray arrows. b) Twenty new lipid species are significantly enriched (≥3-fold enrichment, *q* ≤ 0.01) after incubation with plant liposomes including: PG (green circles), PA (blue circles), cardiolipins (CL; pink circles), PC (orange circles), and phosphatidylinositol (PI; purple circle). Four of the 5 bacterial-bound species of PG highlighted in Fig. 6a remain significantly enriched after liposome incubation (green stars). c) dSTART, dLPSGFmut, and dSDmut proteins bind to PG-conjugated beads (PG 18:1_18:1) but not to PA-conjugated beads (PA 16:0_6:0). d) Working model of dSTART regulation. Binding of a ligand and/or protein partner (green circle) unmasks the HD-Zip III (purple) NLS (yellow), promoting nuclear localization and DNA-binding competence across a shared network of binding sites. This allosteric relief requires the LPSGF (yellow star), DAPLL and RDMYLLQ (blue stars) motifs.

A core property of StARkin domains is their binding to hydrophobic ligands (Tsujishita and Hurley 2000; Roderick et al. 2002; Radauer et al. 2008), which can have important regulatory consequences (reviewed in Dresden et al. 2021). For instance, the PHB START domain binds to multiple species of PC, and binding of PC is required for PHB DNA-binding competence (Husbands et al. 2023). This prompted us to test whether the dSTART domain similarly binds to lipophilic ligands. To do this, we used a tandem-purification strategy to obtain highly pure GFP-tagged recombinant dSTART protein from *E. coli* (Figure S16a; see Methods). GFP-tagged PC-transfer protein (PC-TP) and GFP alone were purified in parallel as controls (Figure S16a). Three lines of evidence support functionality of the recombinant proteins. First, isolated proteins were strongly fluorescent, consistent with a properly folded GFP tag. Second, circular dichroism returned similar secondary structure profiles for dSTART and PC-TP, and these profiles are distinct from the GFP negative control (Figure S10i). Third, liquid-chromatography mass spectrometry (LC-MS) confirmed binding of expected bacterial lipids. Here, lipids were extracted from GFP and dSTART proteins, subjected to LC-MS, and queried against a panel of over 1,200 lipids (Table S3). Multiple phosphatidylglycerol (PG) species were highly enriched across the 4 dSTART replicates (≥3-fold enrichment, *q* ≤ 0.01; Fig. 6a), and these species match previously reported “fortuitous bacterial ligands” bound by other recombinant phospholipid binding proteins, including PC-TP (de Brouwer et al. 2001), steroidogenic factor 1 (Sablin et al. 2009), liver receptor homolog-1 (Zhai et al. 2017), and the HD-Zip III START domain (Husbands et al. 2023).

After confirming functionality, GFP and dSTART proteins were incubated with liposomes generated from *Arabidopsis* total lipid extracts, repurified by affinity chromatography, and subjected to LC-MS. Consistent with efficient exchange for endogenous plant ligands, most bacterial ligands were strongly depleted after liposome incubation (Fig. 6a), and 20 new lipids were enriched specifically in dSTART samples (≥3-fold enrichment, *q* ≤ .01; Fig. 6b). Seven of these new lipids are PGs and 5 are members of the phosphatidic acid (PA) class. Also enriched were 5 species of cardiolipin—phospholipid dimers comprised of monomers of PG and PA (Gonzalvez et al. 2008). Thus, out of the 20 identified plant-enriched lipids, 17 of them have either PG or PA as part of their structure (Fig. 6b). To independently confirm binding, we incubated recombinant MBP-tagged dSTART protein with PG- or PA-conjugated beads. dSTART showed little-to-no binding of PA-conjugated beads, largely resembling the MBP negative control (Fig. 6c). By contrast, binding to PG-beads was much stronger, mirroring its greater recovery in LC-MS analyses. Finally, as DAPLL and RDMYLLQ are analogous to the amino acid stretches involved in PC-binding at the PHB START domain (Husbands et al. 2023), we tested the possibility that DAPLL, RDMYLLQ, and/or LPSGF residues mediate ligand binding at the dSTART domain using recombinant dLPSGFmut, dSDmut, and dTmut proteins. Interestingly, all 3 proteins retain PG-binding capacity (Fig. 6c; Figure S17). These residues may thus mediate the response induced by ligand binding, rather than binding itself, a phenomenon that has been observed for other START domains (Baker et al. 2007). A nonmutually exclusive explanation is that these residues do directly participate in ligand binding but redundantly with many others, as seen for START domains such as PC-TP (Roderick et al. 2002). Taken together, our in vitro analyses propose PG phospholipids as candidate ligands of the dSTART domain.

## Discussion

The StARkin domain is a ubiquitously distributed evolutionary module that uses a diverse set of regulatory mechanisms to impact a wide range of biological processes (Dresden et al. 2021). Here we demonstrate that HD-Zip III and HD-Zip IV TFs contain a cryptic START-like domain defined by multiple unique features including a conserved LPSGF amino acid motif and an insertion between its α3 and β2 secondary structure elements predicted to be intrinsically disordered. This new domain, which we have termed the dSTART domain, is present in the earliest-diverging known sequence of HD-Zip III and HD-Zip IV TFs, indicating its origin predates the divergence of streptophyte algae over 700 million years ago (Schrick et al. 2004; Morris et al. 2018; Romani et al. 2018; Wang et al. 2020). Using PHB as a representative member, we show the dSTART domain is required for full HD-Zip III developmental function. Evolutionarily guided mutagenesis identified multiple distinct dSTART-regulated mechanisms, including promotive effects on PHB nuclear localization and DNA binding competence. Genomic analyses indicate the dSTART domain also contributes to the differential usage of shared binding sites phenomenon previously observed for PHB and CNA (Holub et al. 2024). Finally, we identify members of the PG phospholipid class as candidate ligands of the PHB dSTART domain. Our findings propose a model in which the HD-Zip III dSTART domain promotes homodimerization, enables nuclear import, promotes occupancy across a shared network architecture, and helps to generate paralog-specific transcriptional outcomes (Fig. 6d).

Some regulatory mechanisms are shared by the START and dSTART domains. For instance, both domains show promotive effects on DNA-binding competence or affinity. Specifically, mutations preventing PC-binding by the START domain abolish binding of PHB to *ZPR3* and *ZPR4* loci (Husbands et al. 2023), while nls-PHB-dLPSGFmut and nls-PHB-dSDmut show sharply reduced binding at their *ZPR1* and *ZPR4* targets. Similarly, deletion of either domain reduces ChIP signal across hundreds of loci, an effect which is stronger for the dSTART domain (Fig. 5; Holub et al. 2024). START and dSTART domains also play roles in mediating the differential usage of shared binding sites phenomenon, as loss of either domain drives the interconversion of hundreds of responsive and nonresponsive binding sites (Holub et al. 2024). Logical next steps include determining whether each domain licenses its own unique subset of shared sites as well as testing for genes whose expression depends on allostery between the domains. The underlying drivers of this understudied mechanism of TF functional divergence remain completely unknown in plants and animals. However, to date, all TFs shown to exhibit differential usage of shared binding sites contain ligand binding domains (Kulik et al. 2021; Holub et al. 2024). It is therefore tempting to speculate that ligands may be involved in mediating binding site discrimination. An alternate, though not mutually exclusive mechanism could be protein partners whose interaction is directly or indirectly controlled by the domains. Direct interactions between proteins and StARkin domains are well-documented (Alpy et al. 2005; Kanno et al. 2007; Ma et al. 2009; Park et al. 2009; Du et al. 2012; Miyakawa et al. 2013; Prashek et al. 2017; Carrat et al. 2020; Tugaeva et al. 2020; Apprill et al. 2026), making the dSTART α3-β2 insertion—a potential IDR-to-α-helix switch—particularly intriguing.

Other regulatory mechanisms used by the START and dSTART domains are distinct. For instance, the START domain increases PHB transcriptional potency, which does not seem to be the case for the dSTART domain (Husbands et al. 2023). Further, mutation, deletion, and sequence analyses show no evidence of START-dependent effects on subcellular localization (Husbands et al. 2023). By contrast, the dSTART domain contains 3 sequences—LPSGF, DAPLL, and RDMYLLQ—required for translocation to the nucleus. One attractive hypothesis is that interaction with a ligand and/or protein partner triggers allosteric changes that unmask the N-terminal HD-Zip III NLS (Fig. 6d). Indeed, numerous TFs undergo ligand- or interaction partner-dependent conformational changes that expose their NLS to promote nuclear translocation (Picard and Yamamoto 1987; Lyck et al. 1997; Scharf et al. 1998; Xu et al. 2000; McBride et al. 2002; Ni et al. 2013). As many HD-Zip III NLS residues are contained within the DNA-binding HD, dSTART-mediated occlusion of the NLS would also explain the impaired DNA-binding capacity of nls-PHB-dLPSGFmut and nls-PHB-dSDmut. A key next step is direct structural demonstration of NLS occlusion using purified PHB and PHB-dLPSGFmut or PHB-dSDmut. Finally, the dSTART-specific α3-β2 insertion, as well as its conserved tryptophan residues, contribute to dSTART function not via localization, stability, DNA binding, or transactivation, but rather by promoting HD-Zip III homodimerization.

In addition to their distinct sets of regulatory mechanisms, the START and dSTART domains have distinct lipid binding profiles. For instance, unlike the PHB START domain, no species of phosphatidylethanolamine were significantly enriched in bacterial or plant dSTART samples (Table S3; Husbands et al. 2023). Further, only 2 species of PC were recovered after incubation of dSTART with plant liposomes (Fig. 6b), and neither match previously identified START ligands (Husbands et al. 2023). Finally, out of the 20 plant-enriched dSTART lipids, only PG(34:1) was also significantly enriched in START samples (Husbands et al. 2023). Thus, the START and dSTART domains both bind to phospholipid ligands but appear to have markedly different class and species preferences. We note that mutating LPSGF, DAPLL, and RDMYLLQ is insufficient to abolish binding of PG by the dSTART domain (Fig. 6c). Identifying PG-contact points, perhaps via ligand-docking software or artificial neural networks, then validating them using quantitative biochemical approaches is thus a priority. One caveat to consider is that our MS approach did not prioritize sterols and, as such, their candidacy as dSTART ligands cannot be formally excluded.

A remarkable 85% of the dSTART ligands recovered after incubation with *Arabidopsis* liposomes contained either PG or PA. Links between PA- or PG-binding and TF subcellular localization have been noted in other systems, including plants. For instance, binding of PA triggers nuclear translocation of the R2R3 MYB TF WEREWOLF (WER; Yao et al. 2013). Mutations that prevent PA binding led to cytoplasmic accumulation of WER and the specification of hair cells in place of nonhair cells. Interestingly, when driven to the nucleus by the addition of an NLS tag, these mutant WER variants were fully functional (Yao et al. 2013). This distinguishes them from PHB-dLPSGFmut and PHB-dSDmut and highlights the fact that multiple regulatory mechanisms are controlled by the LPSGF, DAPLL, and RDMYLLQ motifs. In another example, the mobile florigen molecule FLOWERING LOCUS T (FT) binds to PG, and, at low temperatures, this binding anchors FT to chloroplast membranes (Susila et al. 2021). As temperatures increase, binding is disrupted, releasing FT from the membrane and allowing it to traffic to the shoot apical meristem to promote the floral transition. These examples illustrate phospholipids can act as environmental sensors, and that spatial regulation of their intracellular and intercellular accumulation is an efficient way to pattern TF activity. In our experiments, we did not observe overt non-nuclear accumulation of wildtype PHB protein, suggesting PA and PG are not limiting under the conditions and cell types we tested. Careful examination of HD-Zip III subcellular localization across a wide range of developmental and environmental contexts is thus an important next step.

Artificial neural networks like AlphaFold2 are an increasingly important tool to predict function from structure. For instance, elegant work using AlphaFold2 multimeric models of full-length HD-Zip III and HD-Zip IV proteins suggests different modes of action for their dSTART domains (Vijaya et al. 2024). For some HD-Zip IV proteins, homodimerization is proposed to complete the lipid binding pocket of each dSTART monomer. Binding of dSTART ligands would therefore be stabilized by an interface formed by both dSTART domains. This intriguing scenario would not have emerged from homology modeling of the dSTART domain in isolation. By contrast, HD-Zip III multimers are not predicted to homodimerize via either of their START domains (Vijaya et al. 2024). In addition to highlighting the power of modeling to inform biological hypotheses, these findings nicely illustrate the dissimilarities between the HD-Zip III and HD-Zip IV subfamilies, despite their evolutionary relatedness. For instance, HD-Zip IV START domains can bind to sphingolipids (ie very long-chain fatty acid-containing ceramides (VLCFA-Cers; Nagata et al. 2021; Vijaya et al. 2024) while HD-Zip III StARkin domains seem to prefer phospholipids (ie PC or PG; this study and Husbands et al. 2023). In addition, DNA-binding activity is not affected by the HD-Zip IV START domain (Mukherjee et al. 2022), whereas this is a key regulatory feature of both HD-Zip III START domains (Husbands et al. 2023; Holub et al. 2024). Further, proteasome-dependent protein turnover is a major regulatory control point for HD-Zip IV START domains, which careful experiments indicate is intricately tied to binding of their VLCFA-Cers and lyso-PC ligands (Iida et al. 2019; Nagata et al. 2021; Mukherjee et al. 2022; Wojciechowska et al. 2024). By contrast, there is no evidence this regulatory mechanism is affected by either HD-Zip III START domain. Finally, evolutionary conservation patterns also differ between the subfamilies, with the HD-Zip III START and HD-Zip IV dSTART domains showing higher conservation than their adjacent StARkin domain. The significance of this is unclear but may reflect different selective pressures on distinct regulatory properties.

In summary, we report the identification and functional characterization of a cryptic, deeply conserved START-like domain contained within key regulators of plant development. Our findings reinforce the flexible and diverse nature of the StARkin regulatory module and open new research directions. These include testing for allostery between the START and dSTART domains, identifying drivers of dSTART-mediated effects on subcellular localization and differential usage of shared binding sites, and functionally characterizing the HD-Zip IV dSTART domain whose suite of regulatory mechanisms remain unknown.

## Methods

### Bioinformatics tools and databases

PSI-BLAST searches against the RefSeq database at NCBI were carried out with default parameters. Protein domain architectures were obtained from the Pfam (El-Gebali et al. 2019), Conserved Domain Database (Marchler-Bauer et al. 2002), and TREND (Gumerov and Zhulin 2020) servers. Profile to profile searches for domain identification were carried out in HHpred (Soding et al. 2005). Note: Fig. 1a was generated by combining results from a search using full-length PHB sequence and a search with just the dSTART region. To collect homologous sequences, full-length sequence of *A. thaliana* PHB protein was used as a query for BLASTP searches against the genomes of the representative set of Viridiplantae species available in NCBI RefSeq and NCBI nonredundant databases. Additionally, the 1KP database was scanned for the HD-Zip III proteins in Chlorophyta lineage. UniProt accessions for HD-Zip III paralogs are as follows: PHB (ATHB14; O04291), PHV (ATHB9; O04292), REV (Q9SE43), CNA (ATHB15; Q9ZU11), and ATHB8 (Q39123).

### Homology modeling, phylogenetic tree construction, pairwise similarity studies, and MSAs

Modeling of the dSTART domain was performed by I-TASSER (https://zhanggroup.org/I-TASSER) and AlphaFold2 (https://alphafold.ebi.ac.uk) using default parameters. Models were visualized in Pymol. For minimal red/blue MSAs, START and dSTART paralog or ortholog amino acid sequences were aligned using Multiple Alignment using Fast Fourier Transform (MAFFT; https://mafft.cbrc.jp/alignment/server). Sequence boundaries were defined by BLAST as described above. MSAs were constructed using G-INS-I with default parameters in MAFFT (Katoh et al. 2002). Sequence alignments were edited in Jalview (Waterhouse et al. 2009) or in R. Aligned residues were then colored using the R “msa” package with the following criteria: identical in >82% of species (blue), not identical but sharing physicochemical properties (red), no similarity (white). The file was exported in .tex format and formatted via TeXworks. Alignments for phylogenetic trees were created via MAFFT using G-INS-I with default parameters, creating alignments for CNA orthologous START sequences alone, CNA orthologous dSTART sequences alone, and for CNA START and dSTART orthologous sequences together. Note: START and dSTART sequences were chosen using these MAFFT alignments as a guide. MAFFT output as .fasta files were then fed into IQ-TREE (https://iqtree.github.io/) command line (Wong et al. 2026) with ModelFinder (CNA orthologous START sequences alone model: Q.PLANT+G4, CNA orthologous dSTART sequences alone model: Q.PLANT+F+G4, CNA START, and dSTART orthologous sequences together model: Q.PLANT+F+G4), UltraFast Bootstrap (Hoang et al. 2018), and SH-aLRT support. Newick format trees from IQ-TREE were then visualized in the Interactive Tree of Life server (Letunic and Bork 2024) and recreated in Adobe Illustrator for visualization purposes. Newick files for START alone, dSTART alone, and START/dSTART sequences together are in Table S1a. Pairwise comparisons between different START domains done via MAFFT and the percent identity matrix readout. Values were input into Microsoft Excel and colored through the heatmap function. Secondary structure prediction from amino acid sequences were carried out using Ali2D and Quick2D from the MPI Bioinformatics Toolkit (Zimmermann et al. 2018). DEPICTER was also used to identify regions of intrinsic disorder.

### Plant materials and growth conditions

*Arabidopsis thaliana* (Col-0 ecotype) seedlings were grown on soil at 22 °C in long day conditions on soil or on 1% agarose Murashige and Skoog medium plates (pH 5.7). Twenty-four-hour estradiol inductions were performed by spraying 7-d-old or 10-d-old seedlings on plates with 50 µM β-estradiol in 1% DMSO and 0.005% Silwet. A minimum of 50 independent transformants were screened per construct to identify lines with representative behavior. The *phb-13 phv-11 cna-2 er-2* line (Prigge et al. 2005) was obtained from the Arabidopsis Biological Resource Center.

### Molecular biology and plant transformations

The pPHB:PHB and pPHB:PHB* constructs have been described previously and were used as templates to create the complementation and gain-of-function/overexpression constructs, respectively. In brief, these constructs are the native promoter of *PHB* (∼4.5 kb) driving a *PHB* coding sequence with an *mCitrine-YFP-3xFLAG* fusion at its C-terminus. Gain-of-function constructs were created as follows. Using Gibson assembly (NEB), to create pPHB:PHB*-dΔ, the 867 bp dSTART domain (1,191 to 2,058 bp from the start codon) was removed through overlapping primers containing linker regions upstream and downstream of the dSTART domain. To create pPHB:PHB*-dWmut, we synthesized a mutated dSTART domain variant (GeneArt), which made amino acid changes W425A and W492A by substituting the W codon with GCG. To create the pPHB:PHB*-dClip construct, overlapping primers were made to remove amino acids 605 to 645 (1,819 to 1,926 bp from the start codon). Similarly, the pPHB:PHB*-dSDmut construct was created through a synthesized mutated dSTART domain variant (Twist Bioscience), which made amino acid changes of RDMYLLQ and DAPLL to GAVVGAG and AVVAA, respectively. Finally, the pPHB:PHB*-dLPSGFmut construct was made by substituting the SGF amino acids (positions 599 to 601) with TAY (ACCGCATAC). All constructs were then placed into the pB7GW binary vector via Gateway LR reactions (VIB Ghent; Invitrogen). Complementation assays were similarly constructed but using miR166-sensitive *pPHB:PHB* as a template.

For the estradiol-induction constructs, the coding sequence regions of all miR166-resistant constructs were reamplified and cloned into pCR8-GW entry vector using Gibson assembly. Gateway LR reactions were then performed to place the coding regions into the *pUBQ10:XVE oLexA* system described in Husbands et al. (2023).

All constructs, both native and inducible, were transformed into plants using *Agrobacterium tumefaciens*-mediated floral dip transformation. A minimum of 20 transformants were analyzed for both native and inducible constructs. Cloning primers and synthesized sequences can be found in Table S4a.

### Confocal imaging and western blotting

Induced seedlings (5 d old) were incubated in 0.5 µg/mL propidium iodide solution for 10 s to stain the root cell plasma membranes and then washed in water to remove excess propidium iodide, as described previously (Husbands et al. 2023). Full seedlings were wet mounted to a glass slide with a coverslip. Roots were examined under a Leica Stellaris 5 using a Leica C plan 40× objective. The mCitrine YFP tag and propidium iodide were excited using the 526 nm wavelength, and emission was captured between 490 and 550 nm or 570 and 620 nm, respectively.

For LMB (Sigma) treatment, LMB was diluted in water to either 20 nM or 2 µM. Roots were then flooded with LMB solution post-estradiol induction. Subcellular localization was imaged 4 h after LMB treatment as above.

For western blotting, 3 replicates of lysates from induced seedlings tagged with both mCitrine and a 3×-FLAG tag were separated on an SDS-PAGE gel, blotted to nitrocellulose membrane, blocked with 5% milk fat in PBST for 1 h at room temperature, then probed with 1:1,000 anti-FLAG (Millipore) or 1:1,000 anti-Actin (Millipore). Proteins were visualized using 1:5,000 HRP-conjugated secondary antibody (Jackson) and SuperSignal West Pico PLUS substrate (Pierce). Western blots were quantified via Fiji/ImageJ (Schindelin et al. 2012).

### ChIP and qPCR

Ten-day-old seedlings were grown densely on MS plates and sprayed with estradiol to induce expression. Twenty-four hours later, seedlings were collected off the plates and placed in 1× PBS containing 2% formaldehyde (ThermoFisher). Seedlings were crosslinked in a vacuum chamber for 15 min at 20 to 25 mmHg vacuum pressure. Pressure was released, tubes containing the seedlings were shaken to redistribute both seedlings and formaldehyde, then vacuum was reapplied for a further 15 min. Crosslinking was quenched by adding 2 mL of 2 M glycine, shaking, then vacuuming for 5 additional minutes. Seedlings were rinsed 3 times with water, dried with paper towels, then weighed and separated into 0.5 g aliquots for flash freezing in liquid nitrogen.

Seedlings were ground under liquid nitrogen using a ceramic mortar and pestle, and the powdered tissue was transferred to a 50 mL conical tube on ice. About 8.5 mL of nuclear isolation buffer (10 mM HEPES pH 8.0, 1 M sucrose, 5 mM KCl, 5 mM MgCl_2_, 0.6% Triton, 0.4 mM PMSF, 1× cOmplete EDTA free protease inhibitor) was added to the powder and the tubes were rotated for 15 min at 4 °C. The homogenized solution was then filtered through 2 layers of Miracloth into a fresh conical tube and centrifuged at 3,000 × *g* for 15 min at 4 °C. Nuclear pellets were resuspended in 1 mL of nuclear wash buffer (10 mM Tris pH 8.0, 250 mM sucrose, 10 mM MgCl_2_, 1 mM EDTA, 1% Triton, 1 mM PMSF, 1× cOmplete EDTA free protease inhibitor) and transferred to a 1.5 mL Eppendorf tube. Tubes were centrifuged for 10 min at 12,000 × *g* at 4 °C and the supernatant was discarded. Pellets were resuspended in 1 mL of nuclear lysis buffer (20 mM Tris pH 8.0, 2 mM EDTA, 0.01% SDS, 1 mM PMSF, 1× cOmplete EDTA free protease inhibitor) and transferred to a 1 mL Covaris milliTUBE with AFA Fiber. Samples were sonicated in a Covaris S220 (150W peak power, 20% duty factor, 200 cycles/burst for 6 min). The sonicated chromatin was transferred to a fresh 1.5 mL Eppendorf tube and centrifuged for 10 min at 12,000 rpm at 4 °C to pellet the insoluble debris. Soluble chromatin fractions (920 µL) were moved to a fresh 1.5 mL Eppendorf tube and 30 µL of 5 M NaCl and 20 µL of 30% Triton were added to the fractions. Four hundred and twenty microliter of sonicated chromatin was added to each IP tube and 42 µL of sonicated chromatin was put aside at −20 °C for a 10% input control. 2 µL of either M2 mouse monoclonal anti-FLAG (ThermoFisher) or mouse IgG (Cell Signaling Technologies) negative control antibodies were added to their respective tubes and rotated slowly overnight at 4 °C.

Forty microliter of Protein A/G magnetic beads (Invitrogen) were equilibrated with the nuclear lysis buffer and then added to each IP tube and rotated slowly for 2 h at 4 °C. Beads were then washed twice, with 5-min rotations for each wash, at 4 °C. The wash buffers are as follows, in order of wash: low salt wash buffer (150 mM NaCl, 0.1% SDS, 1% Triton, 2 mM EDTA, 20 mM Tris pH 8.0), high salt wash buffer (500 mM NaCl, 0.1% SDS, 1% Triton, 2 mM EDTA, 20 mM Tris pH 8.0), LiCl wash buffer (250 mM LiCl, 1% IGEPAL, 0.1% SDS, 1 mM EDTA, 10 mM Tris pH 8.0), and TE buffer (10 mM Tris pH 8.0, 1 mM EDTA, 0.1% IGEPAL). Bound protein was eluted from beads by rotating with 250 µL of elution buffer (1% SDS, 0.1 M NaHCO_3_) at 65 °C for 15 min. The eluted protein–DNA complexes were removed from the beads and added to a new tube containing 10 µL of 5 M NaCl. 250 µL of elution buffer and 10 µL of 5 M NaCl was also added to the input control tubes. All tubes were placed at 65 °C overnight to reverse the crosslinking. The next day, 10 µL of 0.5 M EDTA, 20 µL of 1 M Tris pH 7.0, 1 µL of 20 mg/mL proteinase K, and 1 µL Rnase (50 ng/µL) were added to each tube and incubated at 45 °C for 1 h. Samples were then cleaned up with the Zymo DNA Clean and Concentrator-25 kit and eluted in 100 µL of the kit's elution buffer. Recovered DNA was quantified using qPCR with 10 µL SsoAdvanced Universal SYBR Green Supermix (Bio-Rad), 1 µL of combined 10 µM primers, 5 µL dH_2_O, and 4 µL of DNA with 2 technical replicates for each tested target (OTC, ZPR1, ZPR4). Data were plotted using Prism graphing software (GraphPad). A Student's *t*-test was used to calculate statistical significance compared to the OTC negative control (all statistics in Table S2a).

### ChIP-seq and bioinformatics

ChIPseq libraries were constructed using the NEBNext Ultra II DNA library kit and single-end sequenced using the Illumina NextSeq1000 located on-site at the Penn Epigenetics Institute. All analyses were performed as described in Holub et al. (2024), however key parameters include: peak calling using a combination of Model-based Analysis of ChIP-Seq (MACS2: *q* < .001) (Zhang et al. 2008; Fu et al. 2022) and IDR threshold (<0.01) (Stephens 2017), and quantification of ChIP signal intensities using Diffbind (*q* < 0.05) (Stark and Brown 2011). Diffbind outputs and mapping statistics can be found in Table S2b, S2f.

### RNA-seq and bioinformatics

Total RNA from 24-h induced seedlings was isolated via the Direct-zol RNA Miniprep kit (Zymo). Libraries were then made via the QuantSeq 3′ mRNA-Seq V2 Library Prep Kit FWD with UDI (Lexogen) and PCR Add-on and Reamplification V2 kits (Lexogen). Libraries were then sequenced using the Illumina NextSeq1000 located on-site at the Penn Epigenetics Institute. All analyses were performed as described in Holub et al. (2024), however key parameters include: carefully timed inductions to minimize circadian effects, application of the lfcShrink function using “ashr” (Dobin et al. 2013) and calling of differentially expressed genes via DESeq2 (≥2-fold or ≤−2-fold change in levels, and a *q*-value ≤0.1). DEseq2 comparisons and mapping statistics can be found in Table S2c, S2d, S2f. Proposed direct targets can be found in Table S2e.

### qRT-PCR assays

Total RNA was extracted from 7-d-old induced *Arabidopsis* seedlings via RNAzol RT (Sigma-Aldrich) extraction methods. RNA was quantified via nanodrop and converted into cDNA via SuperScriptIV Reverse Transcriptase (ThermoFisher), using 500 ng of total RNA and oligo(dT)_18_ primer. The cDNA was diluted 1:1 with Ultra-Pure H_2_O (Invitrogen) before use in qRT-PCR reactions. At least 3 biological replicates and 2 technical replicates were used for relative quantification. ΔCt values were normalized to ACTIN2 and a Student's *t*-test was used to calculate statistical significance (all statistics in Table S2a).

### BiFC assays

Wild type dSTART (1,191 to 2,058 bp from the start codon) domain or mutant versions of the dSTART domain were cloned downstream of the CaMV 35S promoter fused to either an N-terminal Venus protein with Myc tag or a C-terminal Venus protein with HA tag in the pVS1 vector backbone (Addgene) via Gibson cloning. Agrobacterium were then transformed and grown overnight in 50 mL of LB-Kanamycin at 28 °C. Cultures were spun down at 4,200 rpm for 15 min and then resuspended in 2 to 5 mL of infiltration media (10 mM MgCl_2_, 10 mM MES [Fisher, BP300-100], 100 µM acetosyringone) and left to sit at room temperature for at least 2 h. Resuspended pellets were then diluted in 10 mL of infiltration media to an OD of 1, with both N-terminal Venus and C-terminal Venus versions of each variant combined with its respective partner. Using a needless syringe, samples were infiltrated into the abaxial side of 25 to 30 d old *Nicotiana benthamiana*. Fluorescent signal was observed under fluorescent microscope (Leica) at least 20 h post-infiltration. Nuclear signal representing interaction events were imaged using the Leica LasX program and tissue samples were collected from the areas imaged. Quantification of nuclear signal was done via Fiji/ImageJ (Schindelin et al. 2012).

Collected tissue samples were then processed for western blotting. Lysates containing both Myc and HA tags were separated on an SDS-PAGE gel, blotted to nitrocellulose membrane, blocked with 5% milk fat in PBST for 1 h at room temperature, then probed with either 1:1,000 anti-HA (Cell Signaling Technology) or 1:500 anti-Myc (Cell Signaling Technology). Proteins were visualized using 1:5,000 HRP-conjugated secondary antibody (Jackson) and SuperSignal West Pico PLUS substrate (Pierce). Western blots were quantified via Fiji/ImageJ (Schindelin et al. 2012).

### Protein purification, circular dichroism, and PG/PA-conjugated bead assays

Wild type dSTART (1,191 to 2,058 bp from the start codon) domain or mutant versions of the dSTART domain were cloned downstream of mEGFP in the pASG vector backbone (ThermoFisher) or downstream of MBP in a modified pET28b vector (Laha et al. 2015) via Gibson cloning. BL21 bacteria were transformed and grown to an OD of ∼0.4 to 0.6 at 37 °C and induced overnight at 16 °C with 100 ng/mL anhydrotetracycline for mEGFP-tagged proteins or with 100 µM IPTG for MBP-tagged proteins. For mEGFP-tagged proteins, bacteria were lysed via sonication (100 mM Tris, pH 8.0, 200 mM NaCl, 2× HALT protease inhibitor) and purified on a Ni^2+^ column (Qiagen). Proteins were eluted using 250 mM imidazole diluted in lysis buffer. This protein was then doubly purified by running it over a StrepTactin Sepharose (IBA) column, washing, and eluting using 2.5 mM desthiobiotin diluted in lysis buffer. For MBP-tagged proteins, bacteria were lysed (100 mM Tris, pH 8.0, 200 mM NaCl, 2× HALT protease inhibitor) and then purified on an Amylose resin column (NEB) and eluted using 10 mM maltose diluted in lysis buffer. All proteins were concentrated on a 30 MWCO column (Amersham) into detergent-free buffer (1× PBS, pH 7.4, 5% glycerol) compatible for circular dichroism or IP LC-MS analyses. Proteins were also quantified via the Lowry Protein Assay (Creative Proteomics) and on a Coomassie-stained SDS-PAGE gel containing control concentrations of BSA (250, 500, and 1,000 ng).

Circular dichroism was performed using a Jasco J-815 Spectrometer (OSU Biophysical Interaction & Characterization Facility; University of Pennsylvania Biological Chemistry Resources Center [BCRC]) as previously described (Chandler et al. 2007). In brief, 0.1 mg/mL of purified MBP-tagged proteins in 1× PBS, pH 7.4, 5% glycerol were analyzed in 1 mm width quartz cuvettes (Sigma), with scans taken every 1 nm from 190 to 250 nm at a controlled temperature of 4 °C. Buffer alone (1× PBS, pH 7.4, 5% glycerol) was also scanned and subtracted as background. CD values (mdeg) were corrected and converted to mean residue ellipticity (MRE) in Excel (Microsoft). MRE was calculated as [*θ*]MRE = (*θ* × MRW)/(10 × *c* × *l*), where *θ* is the blank-corrected ellipticity in mdeg, MRW is the mean residue weight (110 Da), *c* is the protein concentration in mg/mL, and *l* is the path length (0.1 cm). Values were divided by 10^3^ for plotting in Prism (GraphPad); *y* axis units are therefore 10^3^ deg cm^2^ dmol^−1^.

PG-conjugated beads and PA-conjugated beads were obtained from Echelon Biosciences (Cat. No. P-B0PG and P-B0PA, respectively). About 0.9 nmol of purified MBP-tagged dSTART domain, its mutant versions (3 replicates of MBP-dLPSGFmut and MBP-dSDmut, and 1 replicate of MBP-dTmut), or an MBP alone control were incubated with 0.25 nmols of beads equilibrated in Buffer System 1 (10 mM HEPES, pH 7.4, 150 mM NaCl, supplemented with 0.3% IGEPAL). Mixtures were gently rotated at room temperature for 1 h, then transferred to 4 °C overnight for complete capture. Mixtures were gently washed 3 times using 500 µL of Buffer System 1, eluted in 1× Laemmli buffer, and separated from the beads after a 30 s spin at maximum speed. Bound proteins and 1:500 diluted inputs were separated on an SDS-PAGE gel. Proteins were detected via western blotting using a 1:5,000 dilution of HRP-conjugated antibody targeting the 6×-His tag at the C-terminus of all recombinant proteins (ThermoFisher).

### Liposome production, lipid immunoprecipitations, and mass spectrometry

Total lipids from 15 g of 12-d-old *Arabidopsis* seedling tissues were extracted using the Bligh-Dyer extraction method (Bligh and Dyer 1959). Lipids were dried under argon and resuspended in 100% ethanol to 100 mg/mL. Liposomes were generated by rapid injection of these ethanol-suspended lipids into water. Liposomes were then diluted 1:10 into IP buffer (1× PBS pH 7.4, 150 mM NaCl, 5% glycerol) and briefly sonicated. To identify lipids bound by the dSTART domain in plants, 100 mg of protein (mEGFP control and mEGFP-dSTART) was incubated with 6 mg of liposomes in IP buffer (1× PBS, pH 7.4, 150 mM NaCl, 5% glycerol) for 30 min at room temperature. Proteins were then re-purified using StrepTactin affinity chromatography, extensively washed to remove any liposome contamination, and eluted using 2.5 mM desthiobiotin. Lipids were then extracted using Bligh-Dyer and dried under argon. To identify lipids bound by the dSTART domain in bacteria, lipids were extracted from recombinant proteins immediately after purification from bacteria. Dried lipids were sent to Creative Proteomics for UPLC-MS analysis. A full list of identified lipids can be found in Table S3.

Samples were extracted in 400 μL of isopropyl alcohol:MeOH (1:1, v/v), centrifuged for 15 min at 12,000 rpm at 4 °C, then the supernatant was transferred for LC-MS analysis. Creative Proteomics uses an ACQUITY UPLC combined with AB SCIEX 5500 for ESI− and ESI+ modes. For ESI−, the LC system is comprised of ACQUITY UPLC BEH Amide (100 × 2.1 mm × 1.7 μm). Flow rate of the mobile phase is 0.1 mL/min, the column temperature is maintained at 45 °C, and the sample manager temperature is set at 4 °C. Mass spectrometry parameters in ESI− mode is listed as follows: Curtain Gas 35 arb, Collision GAS 9 arb, IonSpray voltage 4.5 KV, Temperature 450 °C, IonSource Gas1 50 arb and IonSource Gas2 50 arb. For ESI+, the LC system is comprised of ACQUITY UPLC BEH C18 (100 × 2.1 mm × 1.7 μm). Flow rate of the mobile phase is 0.3 mL/min, the column temperature is maintained at 45 °C, and the sample manager temperature is set at 4 °C. Mass spectrometry parameters in ESI+ mode is listed as follows: Curtain Gas 35 arb, Collision GAS 7 arb, IonSpray voltage 5.2 KV, Temperature 450 °C, IonSource Gas1 55 arb, and IonSource Gas2 55 arb.

## Supplementary Material

koag267_Supplementary_Data

## Data Availability

All relevant data generated in this study are deposited to public repositories and are publicly released. Raw and processed data of ChIP-seq and RNA-seq data are deposited at the NCBI Gene Expression Omnibus (https://www.ncbi.nlm.nih.gov/geo/query/acc.cgi?acc=GSE304240 and https://www.ncbi.nlm.nih.gov/geo/query/acc.cgi?&acc=GSE304241, respectively). Relevant R codes are available at github.com/HusbandsLab.
