# Supplementary material for: A cryptic START domain regulates deeply conserved transcription factors
Source: Plant Cell. 2026 Sep 7;38(9):koag267. doi: 10.1093/plcell/koag267 (PMC13590234; doi:10.1093/plcell/koag267)
Supplement: Supplementary file 1 [file koag267_supplementary_data.zip › Dresden et al 2025, Supp Figs (final revision).docx]

Supplemental Information

**A cryptic START domain regulates deeply conserved transcription factors**

Courtney E. Dresden*^1,2^*, Ekaterina P. Andrianova*^3,†^*, Brian J. Smith*^4,†^*, Nicole I. Callery*^1,†^*, Dominic Kolonay*^2^*, Ashton S. Holub*^1,4^*, Ricardo Urquidi Camacho*^1^*, Sarah G. Choudury*^1^*, Isabella J. Higgins*^1^*, Igor B. Zhulin*^3^*, and Aman Y. Husbands*^1,2,4,5,^**

^1^ Department of Biology, University of Pennsylvania, 415 S. University Ave, Philadelphia, PA, 19104, USA

^2^ Molecular, Cellular, and Developmental Biology, the Ohio State University, Columbus, OH 43215, USA.

^3^ Department of Microbiology, The Ohio State University, Columbus, OH 43215, USA.

^4^ Department of Molecular Genetics, The Ohio State University, Columbus, OH 43215, USA.

^5^ Epigenetics Institute, University of Pennsylvania, Philadelphia PA 19104 USA

*^†^* These authors contributed equally.

* Corresponding author. Email: [ayh@upenn.edu](mailto:ayh@upenn.edu)

**Supplemental Figure 1. Multiple sequence alignment of uncharacterized region downstream of the START domain contains START-like domain secondary structure features and patterns.** Secondary structure predictions for multiple START domains made by Quick2D. Predicted α-helices are highlighted in red, and β-sheets are highlighted in blue. Bolded residues represent highly conserved tryptophan (W) residues that define START-like domains. START-like domains from the representative HD-Zip III TF PHB both share similar secondary structure patterns with mammalian START domains. Mammalian START domains are the top hits of the HHpred analysis.

**Supplemental Figure 2. The ancestral-most HD-Zip III protein contains both START-like domains.** Multiple sequence alignment of CNA orthologs reveals a deep conservation of the dSTART domain. The dSTART domain is present in the earliest-diverging organism known to contain an HD-Zip III protein, *Chlorokybus atmophyticus*. Regions of interest include the LPSGF (yellow bar), RTLDLA (blue bar) motifs, and loop regions (pink bars). Blue highlighted amino acids show identity across 82% of the sequences compared. Red highlighted amino acids are similar in physicochemical properties, but not identical, across 82% of the sequences compared. Non-highlighted amino acids share no identity or similarity across 82% of the sequences compared.

**Supplemental Figure 3. Multiple sequence alignment of dSTART domains from select *A. thaliana* HD-Zip III and HD-Zip IV proteins.** Highly conserved residues (bold and red) are called from a multiple sequence alignment of more than 2800 HD-Zip III and HD-Zip IV sequences (see **Supp Doc 1**). The dSTART domain is found in both HD-Zip III and HD-Zip IV proteins, indicating a deep ancestry that predates the divergence of the two HD-Zip subfamilies. The HD-Zip IV dSTART domain also contains the highly conserved stretch of amino acids (LPSGF) that defines the dSTART domain.

**Supplemental Figure 4. Structural and evolutionary analyses of a representative HD-Zip IV family member PDF2. a**, PDF2 architecture (upper) with DEPICTER readout (lower; gray graph). Region near dSTART end also has predicted intrinsic disorder. **b**, Percent identity matrix comparing PDF2 START, dSTART, and dSTART (no insertions) sequences with orthologs in extant plant species showing an opposite pattern of conservation than HD-Zip III TFs. **c**, I-TASSER homology model of PDF2 dSTART domain with intrinsic disorder region (IDR) protruding as a large loop. **d**, Quick2D calls the IDR as disordered but does not predict any structures, in contrast to HD-Zip III TFs (**Figs. 1d,f**).

**Supplemental Figure 5. Phylogenetic trees of CNA and CNA ortholog START and dSTART domains across evolution. a**, Rectangular phylogenetic tree of CNA START sequences from extant plant orthologs. **b**, Rectangular phylogenetic tree of CNA dSTART sequences from extant plant orthologs. Both trees contain UltraFast Bootstrap/SH-aLRT values (produced by IQ-TREE analysis) displayed at nodes**.**

**Supplemental Figure 6. Phylogenetic trees of CNA and CNA ortholog START and dSTART sequences together. a**, Unclipped version of unrooted tree in **Fig. 2a**. Scale bar indicates expected number of amino acid substitutions per site over evolutionary time. **b**, Rectangular phylogenetic tree version of panel **a** containing UltraFast Bootstrap/SH-aLRT values (produced by IQ-TREE analysis) displayed at nodes**.**

**Supplemental Figure 7. Multiple sequence alignments of START and dSTART domains in *A. thaliana* HD-Zip III paralogs. a,** Multiple sequence alignment showing conservation of HD-Zip III START domains. **b,** Multiple sequence alignment showing conservation of HD-Zip III dSTART domains. Note sequence conservation is lower for their insertions except at the RTLDLASXL stretch of the α3-β2 insertion. Pink bars represent dSTART-specific insertions. **c,** Multiple sequence alignment showing conservation of HD-Zip III dSTART domains after removal of the three dSTART-specific insertions. Red highlighted amino acids are similar in physicochemical properties, but not identical, across 82% of the sequences compared. Non-highlighted amino acids share no identity or similarity across 82% of the sequences compared.

**Supplemental Figure 8. Multiple sequence alignments of START and dSTART domains from CNA orthologs across plant evolution.** **a,** Multiple sequence alignment showing conservation of CNA ortholog START domains. **b,** Multiple sequence alignment showing conservation of CNA ortholog dSTART domains. Note sequence conservation is lower for their insertions except at the RTLDLASXL stretch of the α3-β2 insertion. Pink bars represent dSTART-specific insertions. **c,** Multiple sequence alignment showing conservation of CNA ortholog dSTART domains after removal of the three dSTART-specific insertions. Red highlighted amino acids are similar in physicochemical properties, but not identical, across 82% of the sequences compared. Non-highlighted amino acids share no identity or similarity across 82% of the sequences compared.

**Supplemental Figure 9. Multiple sequence alignments of START and dSTART domains from PDF2 orthologs across plant evolution.** **a,** Multiple sequence alignment showing conservation of PDF2 ortholog START domains. **b,** Multiple sequence alignment showing conservation of PDF2 ortholog dSTART domains. Note sequence conservation is lower for their insertions. Pink bar represents dSTART-specific insertions. **c,** Multiple sequence alignment showing conservation of PDF2 ortholog dSTART domains after removal of the three dSTART-specific insertions. Red highlighted amino acids are similar in physicochemical properties, but not identical, across 82% of the sequences compared. Non-highlighted amino acids share no identity or similarity across 82% of the sequences compared.

**Supplemental Figure 10. Cartoon models of PHB mutations and deletions, as well as I-TASSER homology models and circular dichroism of PHB dSTART mutants. a**, Cartoon models of all dSTART mutations and deletions. Names and positions of altered or deleted residues are noted. **b**, W-to-A (dWmut; RMSD=0.667), **c**, clipped IDR (dClip; RMSD=1.011), **d**, SGF-to-TAY (dLPSGFmut; RMSD=1.864), **e**, DAPLL and RDMYLLQ to AVVAA and GAVVGAG, respectively (dSDmut; RMSD=2.398), and **f**, combined dLPSGFmut and dSDmut (dTmut; RMSD=2.301). **g**, Circular dichroism of recombinant purified MBP-tagged START domains shows near identical secondary structure profiles. Note the shoulder at ~215nm consistent with the numerous helices within these proteins. **h**, Circular dichroism of recombinant purified MBP-tagged dTmut (isolated and processed independently of proteins in panel **g**) shows a near identical secondary structure profile to dSTART. Note the shoulder at ~215nm consistent with the numerous helices within these proteins. **i**, Circular dichroism of recombinant doubly-purified GFP-tagged START domains. GFP-dSTART and GFP-PC-TP have similar secondary structures that are distinct from GFP. Note the small shoulder at ~215nm in the START proteins consistent with the presence of their three α-helices.

**Supplemental Figure 11. Phenotypes and scoring of primary transformants from modified *pPHB:PHB** reporters and *pUBQ:PHB** estradiol-inducible lines. a,** Phenotypic scoring of primary transformants (*n* above bar) carrying miR166-insensitive (*) constructs. On the right are representative examples of severe (black), moderate (dark grey), and mild/wildtype (light grey) phenotypes. All constructs were transformed into wildtype Col-0, and categorization followed ref [31]. Note: data in bar graph was split and reused in **Fig. 3b** and **Fig. 4b**. **b**, Three-day-old miR166-insensitive (*) constructs were induced with estradiol daily for five days, then phenotypically scored. On the right are representative examples of severe (black), moderate (dark grey), and mild/wildtype (light grey) phenotypes.

**Supplemental Figure 12. Bimolecular Fluorescence Complementation (BiFC) assay of PHB and selected mutants. a**, Representative images of *Nicotiana benthamiana* infiltrated with *PHB, PHB-dWmut,* or *PHB-dClip* BiFC constructs showing reduced signal intensity for the mutated variants. **b**, Anti-HA and anti-Myc western blots of identical samples in panel **a** showing relatively equal accumulation of N-Venus and C-Venus tagged PHB variants. Anti-H3 blot used as a loading control. Quantification of the blots is found in **Table S1j**.

**Supplemental Figure 13. Steady state protein levels are not affected by the dSTART domain.** Representative anti-FLAG Western blot of estradiol-induced lines showing PHB variants accumulate to similar or higher levels. Note: all PHB variants have an mCitrine YFP-3xFLAG fusion at their C-terminus. Anti-actin blot used as a loading control. Three westerns were performed, then quantified. Quantification data is in **Table S1j**.

**Supplemental Figure 14. LocNES identifies three putative nuclear export signal (NES) sequences in the dSTART domain. a,** Amino acid stretches containing LSPGF (blue) or SDmut (RDMYLLQ, DAPLL; black) motifs emerge as top hits in the LocNES NES prediction software [114]. **b**, LPSGF, or **c**, SDmut mutations abolish their predictions as NES sequences. Note each set of mutations does not prevent the other motif(s) from being called as an NES suggesting independent contributions. **d**, dSTART proteins mutated for all three motifs show no NES and/or cytoplasmic retentions sequences.

**Supplemental Figure 15. Leptomycin B treatment does not result in nuclear localization of PHB-dLPSGFmut. a,** 24-hour estradiol-induced seedlings showing non-nuclear accumulation of PHB-dLPSGFmut. **b,c** 4-hour incubation with (**b**) 20nM or (**c**) 2uM leptomycin B failed to induce nuclear localization of PHB-dLPSGFmut, suggesting LPSGF does not function as a canonical NES. Green = YFP, purple = propidium iodide.

**Supplemental Figure 16. Recombinant START domain proteins used in LC-MS and lipid-conjugated bead assays. a,** Double purification of GFP alone (lanes 1 and 2), GFP-tagged dSTART (lanes 3 and 4), and GFP-tagged PC-TP proteins (lanes 5 and 6) in *E coli*. Lanes 1, 3, and 5 are eluates from the first 6x-His-tag purification step using nickel affinity purification. Lanes 2, 4, and 6 are eluates from the second Twin-Strep-tag purification using Strep-Tactin XT. Note PC-TP samples were run on the same gel and intervening lanes were not included in the figure for clarity. **b**, Amylose resin-purified MBP alone (lane 1), MBP-dSTART (lane 2), MBP-dWmut (lane 3), MBP-dClip (lane 4), MBP-dLPSGFmut (lane 5), and MBP-dSDmut (lane 6). To assist with quantification, 250ng (lane 7), 500ng (lane 8), and 1ug (lane 9) of bovine serum albumin (BSA) was run out on the same gel. GFP or MBP alone negative controls are marked with a black asterisk. Approximate expected sizes of tagged START variants are marked with a red asterisk.

**Supplemental Figure 17. PHB-dTmut binds phosphatidylglycerol (PG). a**, Amylose resin-purified MBP alone (lane 1), MBP-dSTART (lane 2), and MBP-dTmut (lane 3). To assist with quantification, 250ng (lane 4), 500ng (lane 5), and 1ug (lane 6) of bovine serum albumin (BSA) was run out on the same gel. **b**, dSTART and dTmut proteins bind to PG-conjugated beads.
