# Supplementary material for: A cryptic START domain regulates deeply conserved transcription factors
Source: Plant Cell. 2026 Sep 7;38(9):koag267. doi: 10.1093/plcell/koag267 (PMC13590234; doi:10.1093/plcell/koag267)
Supplement: Supplementary file 1 [file koag267_supplementary_data.zip › Supp Doc 1.docx]

```
CLUSTAL

XP_020582785.1/1-284 -------PAVLRTFSQRLSRGFNDA-ING--FSDDG-----------WS--FL-GY----
XP_020682623.1/1-284 -------PAVLRTFSQRLSRGFNDA-ING--FSDDG-----------WS--LL-GY----
XP_020682624.1/1-284 -------PAVLRTFSQRLSRGFNDA-ING--FSDDG-----------WS--LL-GY----
XP_006846015.1/1-286 -------PAVLRTFSQRLSRGFNDA-VNG--FADDG-----------WS--LM-SS----
NP_001142394.1/1-289 -------PAVLRTFSQRLSRGFNDA-VSG--FPDDG-----------WS-CLL-NT----
NP_001146215.1/1-288 -------PAVLRTFSQRLSRGFNDA-VSG--FPDDG-----------WS-SLL-SS----
XP_020405718.1/1-288 -------PAVLRTFSQRLSRGFNDA-VSG--FPDDG-----------WS-SLL-SS----
XP_002443548.1/1-287 -------PAVLRTFSQRLSRGFNDA-VSG--FPDDG-----------WS-SLL-SS----
XP_025808876.1/1-287 -------PAVLRTFSQRLSRGFNDA-VSG--FPDDG-----------WS-PLL-SS----
XP_004963232.1/1-287 -------PAVLRTFSQRLSRGFNDA-VSG--FPDDG-----------WS-PLL-SS----
XP_020192001.1/1-285 -------PAVLRTFSQRLSRGFNDA-VNG--FLDDG-----------WS--LM-SS----
XP_003562005.1/1-285 -------PAVLRTFSQRLSRGFNDA-VNG--FLDDG-----------WS--LM-SS----
XP_004982339.1/1-286 -------PAVLRTFSQRLSRGFNDA-VNG--FPDDG-----------WS--LM-SS----
XP_025796542.1/1-286 -------PAVLRTFSQRLSRGFNDA-VNG--FPDDG-----------WS--LM-SS----
NP_001347456.1/1-286 -------PAVLRTFSQRLSRGFNDA-VNG--FPDDG-----------WS--LM-SS----
NP_001352464.1/1-286 -------PAVLRTFSQRLSRGFNDA-VNG--FPDDG-----------WS--LM-SS----
XP_002464180.1/1-286 -------PAVLRTFSQRLSRGFNDA-VNG--FPDDG-----------WS--LM-SS----
XP_015689987.1/1-286 -------PAVLRTFSQRLSRGFNDA-VNG--FPDDG-----------WS--PM-SS----
XP_015632484.1/1-286 -------PAVLRTFSQRLSRGFNDA-VNG--FPDDG-----------WS--LM-SS----
XP_015620816.1/1-285 -------PAVFRTFSQRLSRGFNDA-VSG--FPDDG-----------WS--LL-SS----
XP_006664738.1/1-285 -------PAVFRTFSQRLSRGFNDA-VSG--FPDDG-----------WS--LL-SS----
XP_020190954.1/1-286 -------PAVLRTFSQRLSRGFNDA-VSG--FPDDG-----------WS--LL-TS----
XP_020163543.1/1-286 -------PAVLRTFSQRLSRGFNDA-VSG--FPDDG-----------WS--LL-TS----
XP_003577453.1/1-286 -------PAVLRTFSQRLSRGFNDA-VSG--FPDDG-----------WS--LL-SS----
XP_020575104.1/1-285 -------PTVLRTFSQRMNRGFNDA-ING--FPDDG-----------WS--LM-GS----
XP_020696902.1/1-286 -------PTVLRTFSQRMSRGFNDA-VNG--FPDDG-----------WS--LM-GS----
XP_020578108.1/1-284 -------PAVLRTFSQRLSRGFNDA-ING--FMDDG-----------WS--LM-SS----
XP_020698923.1/1-284 -------PAVLRTFSQRLSRGFNDA-ING--FLDDG-----------WS--LM-SG----
XP_020096610.1/1-279 -------PAVLRTFSHRLCRGFNDA-VNG--FTEDG-----------WS--LM-SN----
XP_009412508.1/1-287 -------PAVLRNFIRRLSRGFNDA-VNG--FADDG-----------WS--LM-GS----
XP_020271156.1/1-284 -------PAVLRTFSQRLSKGFNDA-VNG--FTDDG-----------WS--LL-GS----
XP_020273236.1/1-287 -------PAVLRTFSQRLSKGFNDA-VNG--FTDDG-----------WS--LL-GS----
XP_020092733.1/1-287 -------PAVLRNFSQRLSRGFNDA-VNG--FADDG-----------WS--LI-NS----
XP_019701934.1/1-288 -------PAVLRTFSQRLSRGFNDA-VNG--FTDDG-----------WS--LM-GS----
XP_010905421.1/1-288 -------PAVLRTFSQRLSRGFNDA-VNG--FTDDG-----------WS--LM-GS----
XP_010905420.1/1-289 -------PAVLRTFSQRLSRGFNDA-VNG--FTDDG-----------WS--LM-GS----
XP_010923678.1/1-288 -------PAVLRTFSQRLSRGFNDA-VNG--FVDDG-----------WS--LM-GS----
XP_008794452.1/1-288 -------PAVLRTFSQRLSRGFNDA-VNG--FADDG-----------WS--LM-GS----
XP_009397391.1/1-288 -------PAVLRTFSQRLSRGFNDA-VNG--FADDG-----------WS--LL-GS----
XP_009408551.1/1-288 -------PAVLRTFSQRLSRGFNDA-VNG--FADDG-----------WL--LL-GS----
XP_009399045.1/1-288 -------PAVLRTFSQRLSRGFNDA-VNG--FVDDG-----------WS--LL-GS----
XP_009388964.1/1-288 -------PAVLRAFSQRLSRGFNDA-VNG--FSDDG-----------WS--LL-GS----
XP_009397717.1/1-288 -------PAVLRAFSQRLSRGFNDA-VNG--FADDG-----------WS--LL-GS----
XP_018488876.1/1-280 -------PAVLRTFSQRLCRGFNDA-VNG--FGDDG-----------WS-PMS-S-----
XP_013587486.1/1-280 -------PAVLRTFSQRLCRGFNDA-VNG--FADDG-----------WS-PMS-S-----
XP_009115139.1/1-280 -------PAVLRTFSQRLCRGFNDA-VNG--FADDG-----------WS-PMS-S-----
XP_013662459.1/1-280 -------PAVLRTFSQRLCRGFNDA-VNG--FADDG-----------WS-PMS-S-----
XP_013707293.1/1-280 -------PAVLRTFSQRLCRGFNDA-VNG--FADDG-----------WS-PMS-S-----
XP_006415465.2/1-280 -------PAVLRTFSQRLCRGFNDA-VNG--FADDG-----------WS-PMS-S-----
XP_019099288.1/1-280 -------PAVLRTFSQRLCRGFNDA-VNG--FADDG-----------WS-PMS-S-----
NP_174337.1/1-280    -------PAVLRTFSQRLCRGFNDA-VNG--FVDDG-----------WS-PMS-S-----
XP_020867051.1/1-280 -------PAVLRTFSQRLCRGFNDA-VNG--FVDDG-----------WS-PMS-S-----
XP_020867052.1/1-280 -------PAVLRTFSQRLCRGFNDA-VNG--FVDDG-----------WS-PMS-S-----
XP_010460847.1/1-280 -------PAVLRTFSQRLCRGFNDA-VNG--FADDG-----------WS-PMS-S-----
XP_019094482.1/1-280 -------PAVLRTFSQRLCRGFNDA-VNG--FADDG-----------WS-PMS-S-----
XP_006306764.1/1-280 -------PAVLRTFSQRLCRGFNDA-VNG--FADDG-----------WS-PMS-S-----
XP_010538268.1/1-285 -------PAVLRTFSQRLSRGFNDA-VNG--FSADG-----------WS-PIV-S-----
XP_010519070.1/1-284 -------PAVLRTFSQRLSRGFNDA-VNG--FADDG-----------WS-PMG-S-----
XP_018436356.1/1-280 -------PAVLRTFSQKLCRGFNDA-VNG--FVDDG-----------WS-PLG-S-----
XP_013637594.1/1-286 -------PAVLRTFSQKLCRGFNDA-VNG--FVDDG-----------WS-PLG-SN----
XP_013692700.1/1-286 -------PAVLRTFSQKLCRGFNDA-VNG--FVDDG-----------WS-PLG-SN----
XP_009143747.1/1-286 -------PAVLRTFSQKLCRGFNDA-VNG--FVDDG-----------WS-PLG-SN----
XP_018484893.1/1-285 -------PAVLRTFSQRLSRGFNDA-VNG--FVDDG-----------WS-PMG-S-----
XP_013637264.1/1-280 -------PAVLRTFSQRLSRGFNDA-VNG--FVDDG-----------WS-PMG-S-----
XP_013637263.1/1-280 -------PAVLRTFSQRLSRGFNDA-VNG--FVDDG-----------WS-PMG-S-----
XP_022557131.1/1-280 -------PAVLRTFSQRLSRGFNDA-VNG--FVDDG-----------WS-PMG-S-----
XP_013729051.1/1-280 -------PAVLRTFSQRLSRGFNDA-VNG--FVDDG-----------WS-PMG-S-----
XP_013744548.1/1-280 -------PAVLRTFSQRLSRGFNDA-VNG--FVDDG-----------WS-PMG-S-----
XP_009141396.1/1-280 -------PAVLRTFSQRLSRGFNDA-VNG--FVDDG-----------WS-PLG-S-----
XP_006410621.1/1-284 -------PAVLRTFSQRLCRGFNDA-VNG--FVDDG-----------WT-PMG-S-----
NP_181018.1/1-284    -------PAVLRTFSQRLCRGFNDA-VNG--FVDDG-----------WS-PMG-S-----
XP_020884308.1/1-284 -------PAVLRTFSQRLCRGFNDA-VNG--FVDDG-----------WS-PMG-S-----
XP_006293359.1/1-284 -------PAVLRTFSQRLCRGFNDA-VNG--FGDDG-----------WS-PMG-S-----
XP_010516626.1/1-284 -------PAVLRTFSQRLCRGFNDA-VNG--FVDDG-----------WT-PMG-S-----
XP_010509720.1/1-284 -------PAVLRTFSQRLCRGFNDA-VNG--FVDDG-----------WT-PMG-S-----
XP_010504942.1/1-284 -------PAVLRTFSQRLCRGFNDA-VNG--FVDDG-----------WT-PMG-S-----
XP_017223336.1/1-283 -------PAVLRAFRQRLCRGFNDA-ING--FVDDG-----------WS-TMN-S-----
XP_016559371.1/1-285 -------PAALRAFSQRLCRGFNNA-VSG--FIDDG-----------WT-IMD-N-----
XP_016559374.1/1-285 -------PAALRAFSQRLCRGFNNA-VSG--FIDDG-----------WT-IMD-N-----
XP_012830207.1/1-281 -------PAVLRSLSQRLCRGFNDA-VNG--IVDDG-----------WS-IIS-S-----
XP_012827547.1/1-287 -------PAVLRSLSQRLCRGFNDA-VNG--FVDDG-----------WS-IMG-S-----
XP_022866806.1/1-284 -------PAVLRALSQKLCRGFNDA-VNG--FIDDG-----------WS-IMG-S-----
XP_022866805.1/1-285 -------PAVLRALSQKLCRGFNDA-VNG--FIDDG-----------WS-IMG-S-----
XP_023001017.1/1-285 -------PAVLRTFSQRLSRGFNDA-VNG--FVDDG-----------WS--LM-DS----
XP_023001016.1/1-285 -------PAVLRTFSQRLSRGFNDA-VNG--FVDDG-----------WS--LM-DS----
XP_023519837.1/1-285 -------PAVLRTFSQRLSRGFNDA-VNG--FVDDG-----------WS--LM-DS----
XP_023519836.1/1-285 -------PAVLRTFSQRLSRGFNDA-VNG--FVDDG-----------WS--LM-DS----
XP_022927139.1/1-285 -------PAVLRTFSQRLSRGFNDA-VNG--FVDDG-----------WS--LM-DS----
XP_022927138.1/1-285 -------PAVLRTFSQRLSRGFNDA-VNG--FVDDG-----------WS--LM-DS----
XP_022139982.1/1-285 -------PAVLRTFSQRLCRGFNDA-VNG--FVDDG-----------WS--LM-DS----
XP_004135550.1/1-285 -------PAVLRTFSQRLCRGFNDA-VNG--FVDDG-----------WS--LM-DS----
XP_008445207.1/1-285 -------PAVLRTFSQRLCRGFNDA-VNG--FVDDG-----------WS--LM-DS----
XP_017230337.1/1-286 -------PAVLRTFSQRLCRGFNDA-VNG--FLDDG-----------WS--IL-GS----
XP_017230338.1/1-286 -------PAVLRTFSQRLCRGFNDA-VNG--FLDDG-----------WS--IL-GS----
XP_010316959.1/1-285 -------PAVLRAFSQRLCRGFNNA-VSG--FIDDG-----------WT--IM-GS----
XP_004233170.1/1-285 -------PAVLRAFSQRLCRGFNNA-VSG--FIDDG-----------WT--IM-GS----
XP_015066839.1/1-285 -------PAVLRAFSQRLCRGFNNA-VSG--FIDDG-----------WT--IM-GS----
XP_015066842.1/1-285 -------PAVLRAFSQRLCRGFNNA-VSG--FIDDG-----------WT--IM-GS----
XP_015066843.1/1-285 -------PAVLRAFSQRLCRGFNNA-VSG--FIDDG-----------WT--IM-GS----
XP_006353018.1/1-285 -------PAVLRAFSQRLCRGFNNA-VSG--FIDDG-----------WT--IM-GS----
XP_006353020.1/1-285 -------PAVLRAFSQRLCRGFNNA-VSG--FIDDG-----------WT--IM-GS----
XP_019178964.1/1-287 -------PAVLRALSQRLCRGFNDA-VNG--FVDDG-----------WT--VL-GS----
XP_019178963.1/1-287 -------PAVLRALSQRLCRGFNDA-VNG--FVDDG-----------WT--VL-GS----
XP_019228489.1/1-285 -------PAVLRALSQRLCRGFNDA-VNG--FVDDG-----------WT--VM-DS----
XP_019228488.1/1-285 -------PAVLRALSQRLCRGFNDA-VNG--FVDDG-----------WT--VM-DS----
NP_001289498.1/1-285 -------PAVLRALSQRLCRGFNDA-VNG--FVDDG-----------WT--VM-DS----
XP_009777911.1/1-285 -------PAVLRALSQRLCRGFNDA-VNG--FVDDG-----------WT--VM-DS----
XP_009612342.1/1-285 -------PAVLRALSQRLCRGFNDA-VNG--FVDDG-----------WT--VM-DS----
XP_009612341.1/1-285 -------PAVLRALSQRLCRGFNDA-VNG--FVDDG-----------WT--VM-DS----
XP_006363649.1/1-287 -------PAVLRALSQRLCRGFNDA-VSG--FVDDG-----------WT--IM-DS----
XP_015066689.1/1-287 -------PAVLRALSQRLCRGFNDA-VNG--FVDDG-----------WT--IM-DS----
XP_010315636.1/1-287 -------PAVLRALSQRLCRGFNDA-VNG--FVDDG-----------WT--IT-DS----
XP_022879368.1/1-285 -------PVVLRALSQRLCRGFNDA-VNG--FVDDG-----------WS--IL-GG----
XP_020554026.1/1-285 -------PTVLRALSQRLCRGFNDA-VNG--FVDDG-----------WS--IM-GS----
XP_011096678.1/1-285 -------PTVLRALSQRLCRGFNDA-VNG--FVDDG-----------WS--IM-GS----
XP_011085207.1/1-285 -------PAVLRTLSQRLCRGFNDA-VNG--FTDDG-----------WS--IM-GS----
XP_023003193.1/1-285 -------PAVLRTFGQKLCRGFNDA-VNG--FVDDG-----------WS--AM-SS----
XP_022926723.1/1-285 -------PAVLRTFGQKLCRGFNDA-VNG--FADDG-----------WS--AM-SS----
XP_023518237.1/1-285 -------PAVLRTFGQKLCRGFNDA-VNG--FADDG-----------WS--AM-SS----
XP_022146537.1/1-286 -------PAVLRTFSQKLCRGFNDA-VNG--FADDG-----------WS--PL-GS----
XP_011658319.1/1-286 -------PAVLRTFSQKLCRGFNDA-VNG--FADDG-----------WS--PM-GS----
XP_008439554.1/1-286 -------PAVLRTFSQKLCRGFNDA-VNG--FADDG-----------WS--PM-GS----
XP_022978372.1/1-286 -------PAVLRTFSQKLCRGFNDA-VNG--FADDG-----------WS--PM-GS----
XP_023543833.1/1-286 -------PAVLRTFSQKLCRGFNDA-VNG--FADDG-----------WS--PM-GS----
XP_022926042.1/1-286 -------PAVLRTFSQKLCRGFNDA-VNG--FADDG-----------WS--PM-GS----
XP_010247499.1/1-286 -------PAVLRTFSQRLCRGFNDA-ING--FSDDG-----------WS--LM-GS----
XP_016717556.1/1-285 -------PAVLRTFSQRLCRGFNDA-VNG--FVDDG-----------WS--LM-GS----
XP_012455108.1/1-285 -------PAVLRTFSQRLCRGFNDA-VNG--FVDDG-----------WS--LM-GS----
XP_016717554.1/1-287 -------PAVLRTFSQRLCRGFNDA-VNG--FVDDG-----------WS--LM-GS----
XP_012455107.1/1-287 -------PAVLRTFSQRLCRGFNDA-VNG--FVDDG-----------WS--LM-GS----
XP_016711412.1/1-287 -------PAVLRTFSQRLCRGFNDA-VNG--FVDDG-----------WS--LM-GS----
XP_017649973.1/1-287 -------PAVLRTFSQRLCRGFNDA-VNG--FVDDG-----------WS--LM-GS----
XP_022716449.1/1-285 -------PAVLRTFSQRLCRGFNDA-VNG--FMDDG-----------WS--LM-GG----
XP_022724664.1/1-287 -------PAVLRTFSQRLCRGFNDA-VNG--FADDG-----------WS--LM-GS----
XP_021298192.1/1-285 -------PAVLRTFSQRLCRGFNDA-VNG--FADDG-----------WS--LM-GS----
XP_021298193.1/1-294 -------PAVLRTFSQRLCRGFNDA-VNG--FADDG-----------WS--LM-GS----
XP_021298191.1/1-294 -------PAVLRTFSQRLCRGFNDA-VNG--FADDG-----------WS--LM-GS----
XP_007022874.2/1-285 -------PAVLRTFSQRLCRGFNDA-VNG--FADDG-----------WS--LM-GS----
XP_017979205.1/1-294 -------PAVLRTFSQRLCRGFNDA-VNG--FADDG-----------WS--LM-GS----
XP_017979204.1/1-294 -------PAVLRTFSQRLCRGFNDA-VNG--FADDG-----------WS--LM-GS----
XP_017236680.1/1-286 -------PAVLRTFSQRLCRGFNDA-VNG--FLDDG-----------WS--IL-DS----
XP_010051959.1/1-285 -------PAVLRTFSQKLCRGFNDA-VNG--FVDDG-----------WS--VL-SS----
XP_010051960.1/1-285 -------PAVLRTFSQKLCRGFNDA-VNG--FVDDG-----------WS--VL-SS----
XP_015882975.1/1-284 -------PAVLRTFSQRLCRGFNDA-VNG--FADDG-----------WS--LM-GS----
XP_015882797.1/1-284 -------PAVLRTFSQRLCRGFNDA-VNG--FADDG-----------WS--LM-GS----
XP_010098570.1/1-284 -------PAVLRTFSQRLCRGFNDA-VNG--FVDDG-----------WS--LL-GS----
XP_011012084.1/1-284 -------PAVLRTFSQRLCRGFNDA-VNG--FTDSG-----------WS--LL-DG----
XP_011012083.1/1-284 -------PAVLRTFSQRLCRGFNDA-VNG--FTDSG-----------WS--LL-DG----
XP_006377667.2/1-284 -------PAVLRTFSQRLCRGFNDA-VNG--FTDSG-----------WS--LL-DG----
XP_002281868.2/1-285 -------PAVLRTFSQRLCRGFNDA-VNG--FADDG-----------WS--LM-GS----
XP_018847863.1/1-285 -------PAVLRTFSQRLCRGFNDA-VNG--FADDG-----------WS--LL-GS----
XP_018847862.1/1-285 -------PAVLRTFSQRLCRGFNDA-VNG--FADDG-----------WS--LL-GS----
XP_023919942.1/1-285 -------PAVLRTFSQRLCRGFNDA-VNG--FADDG-----------WS--LM-GS----
XP_023919941.1/1-285 -------PAVLRTFSQRLCRGFNDA-VNG--FADDG-----------WS--LM-GS----
XP_018828101.1/1-285 -------PAVLRTFSQRLCRGFNDA-VNG--FVDDG-----------WS--LL-GS----
XP_018828100.1/1-285 -------PAVLRTFSQRLCRGFNDA-VNG--FVDDG-----------WS--LL-GS----
XP_002298892.1/1-285 -------PAVLRTFSQRLCRGFNDA-VNG--FTDDG-----------WS--LL-GS----
XP_011038887.1/1-285 -------PAVLRTFSQRLCRGFNDA-VNG--FTDDG-----------WS--LL-GS----
XP_011038886.1/1-285 -------PAVLRTFSQRLCRGFNDA-VNG--FTDDG-----------WS--LL-GS----
XP_021657817.1/1-285 -------PTVLRTFSQRLCRGFNDA-VNG--FADDG-----------WS--LL-GG----
XP_021657818.1/1-285 -------PTVLRTFSQRLCRGFNDA-VNG--FADDG-----------WS--LL-GG----
XP_015577248.1/1-260 -------PAVLRTFSQRLCRGFNDA-VNG--FSDDG-----------WS--LL-GG----
XP_021616343.1/1-285 -------PAVLRTFSQRLCRGFNDA-VNG--FADDG-----------WS--LL-GG----
XP_021645103.1/1-285 -------PAVLRTFSQRLCRGFNDA-VNG--FADDG-----------WS--LL-GG----
XP_021592762.1/1-285 -------PAVLRTFSQRLCRGFNDA-VNG--FADDG-----------WS--LL-GG----
XP_012075627.1/1-285 -------PAVLRTFSQRLCRGFNDA-VNG--FADDG-----------WS--LL-GG----
XP_008372224.1/1-285 -------PAVLRTFCQRLCSGFNDA-VNG--FADDG-----------WT--LM-GS----
XP_009362238.1/1-285 -------PAVLRTFCQRLCSGFNDA-VNG--FADDG-----------WT--LM-GS----
XP_009344405.1/1-284 -------PAVLRTFCQRLCSGFNDA-VNG--FADDG-----------WT--LM-SS----
XP_008349150.1/1-284 -------PAVLRTFCQRLCSGFNDA-VNG--FADDG-----------WT--LM-GS----
XP_007214622.1/1-285 -------PAVLRTFCQRLCRGFNDA-VNG--FADDG-----------WS--LM-GS----
XP_021812393.1/1-285 -------PAVLRTFCQRLCRGFNDA-VNG--FADDG-----------WS--LM-GS----
XP_024161164.1/1-285 -------PAVLRTFSQRLCRGFNDA-VNG--FADDG-----------WS--LL-GS----
XP_004293989.1/1-285 -------PAVLRTFSQRLCRGFNDA-VNG--FADDG-----------WS--LL-GS----
XP_019419795.1/1-282 -------PAVLRTLSQRLCRGFNDA-VNG--FVDDG-----------WS--LL-GN----
XP_019426301.1/1-282 -------PAVLRTLSQRLCRGFNDA-VNG--FVDDG-----------WS--LM-SN----
XP_003524993.1/1-285 -------PAVLRTFSQRLCKGFNDA-VNG--FVDDG-----------WS--LM-GN----
XP_003532788.1/1-285 -------PAVLRTFSQRLCKGFNDA-VNG--FVDDG-----------WS--LM-GN----
XP_020215009.1/1-285 -------PAVLRTFSQRLCRGFNDA-VNG--FSDDG-----------WS--LM-GN----
XP_017409410.1/1-285 -------PAVLRTFSQRLCKGFNDA-VNG--FVDDG-----------WS--PI-GN----
XP_017409418.1/1-285 -------PAVLRTFSQRLCKGFNDA-VNG--FVDDG-----------WS--PI-GN----
XP_014509941.1/1-285 -------PAVLRTFSQRLCKGFNDA-VNG--FVDDG-----------WS--LI-GN----
XP_007159548.1/1-285 -------PAVLRTFSQRLCKGFNDA-VNG--FVDDG-----------WS--LI-GN----
XP_024047818.1/1-288 -------PAVLRTFSQRLSRGFNDA-ING--FLDDG-----------WS--LL-SS----
XP_006448915.2/1-288 -------PAVLRTFSQRLSRGFNDA-ING--FLDDG-----------WS--LL-SS----
XP_006468302.1/1-288 -------PAVLRTFSQRLSRGFNDA-ING--FLDDG-----------WS--LL-SS----
XP_006468303.1/1-287 -------PAVLRTFSQRLSRGFNDA-ING--FLDDG-----------WS--LL-SS----
XP_024047817.1/1-287 -------PAVLRTFSQRLSRGFNDA-ING--FLDDG-----------WS--LL-SS----
XP_004504217.1/1-285 -------PAVLRTFSQRLSKGFNDA-ING--FVDDG-----------WS--LI-GN----
XP_003594520.2/1-281 -------PAVLRTFSQRLCRGFNDA-VNG--FVDDG-----------WS--LL-GN----
XP_024632601.1/1-281 -------PAVLRTFSQRLCRGFNDA-VNG--FVDDG-----------WS--LL-GN----
XP_004486450.1/1-285 -------PAVLRTFSQRLCRGFNDA-VNG--FVDDG-----------WS--LL-GN----
XP_007147487.1/1-285 -------PAVLRTFSQRLCRGFNDA-VNG--FVDDG-----------WS--LM-GT----
XP_017437412.1/1-285 -------PAVLRTFSQRLCRGFNDA-VNG--FVDDG-----------WS--LM-GT----
XP_014518680.1/1-285 -------PAVLRTFSQRLCRGFNDA-VNG--FVDDG-----------WS--LM-GT----
XP_003546255.1/1-285 -------PAVLRTFSQRLCRGFNDA-VNG--FVDDG-----------WS--LM-GT----
XP_020233213.1/1-285 -------PAVLRTFSQRLCRGFNDA-VNG--FVDDG-----------WS--LM-GT----
XP_003535078.1/1-285 -------PAVLRTFSQRLCRGFNDA-VNG--FVDDG-----------WS--LM-GT----
XP_014617344.1/1-285 -------PAVLRTFSQRLCRGFNDA-VNG--FVDDG-----------WS--LM-GT----
XP_015959291.1/1-283 -------PAVLRTFSQRLCRGFNDA-VNG--FVEDG-----------WS--LL-GN----
XP_016197724.1/1-283 -------PAVLRTFSQRLCRGFNDA-VNG--FVEDG-----------WS--LL-GN----
XP_025694278.1/1-283 -------PAVLRTFSQRLCRGFNDA-VNG--FVEDG-----------WS--LL-GN----
XP_006846777.1/1-281 -------PAVLRTFSQRLSRGFNDA-VNG--FADDG-----------WS--LM-SS----
XP_003574071.1/1-280 -------PAVLRTFSQRLSRGFNDA-ISG--FNDDG-----------WS--VM-GG----
XP_020174778.1/1-278 -------PAVLRTFSQRLSRGFNDA-ISG--FNDDG-----------WS--VM-AG----
XP_006661867.1/1-281 -------PAVLRTFSQRLSRGFNDA-ISG--FNDDG-----------WS--VM-GG----
XP_015614901.1/1-281 -------PAVLRTFSQRLSRGFNDA-ISG--FNDDG-----------WS--VM-GG----
XP_004982838.1/1-282 -------PAVLRTFSQRLSRGFNDA-ISG--FNDDG-----------WS--VM-GG----
XP_025796396.1/1-282 -------PAVLRTFSQRLSRGFNDA-ISG--FNDDG-----------WS--VM-GG----
NP_001316502.1/1-284 -------PAVLRTFSQRLSRGFNDA-ISG--FNDDG-----------WS--VM-CG----
XP_021306737.1/1-282 -------PAVLRTFSQRLSRGFNDA-ISG--FNDDG-----------WS--IM-AG----
XP_021306733.1/1-293 -------PAVLRTFSQRLSRGFNDA-ISG--FNDDG-----------WS--IM-AG----
XP_020162053.1/1-281 -------PAVLRTFSQRLSRGFNDA-ISG--FNDDG-----------WS--IM-AG----
XP_006649248.1/1-282 -------PAVLRTFSQRLSRGFNDA-ISG--FNDDG-----------WS--IM-GG----
XP_015632709.1/1-282 -------PAVLRTFSQRLSRGFNDA-ISG--FNDDG-----------WS--IM-GG----
NP_001296150.1/1-282 -------PAVLRTFSQRLSRGFNDA-ISG--FNDDG-----------WS--VM-GG----
XP_008665074.1/1-283 -------PAVLRTFSQRLSRGFNDA-ISG--FNDDG-----------WS--VM-GG----
NP_001105994.1/1-283 -------PAVLRTFSQRLSRGFNDA-ISG--FNDDG-----------WS--VM-GG----
XP_008660404.1/1-283 -------PAVLRTFSQRLSRGFNDA-ISG--FNDDG-----------WS--VM-GG----
XP_002468669.1/1-283 -------PAVLRTFSQRLSRGFNDA-ISG--FNDDG-----------WS--VM-GG----
XP_021309716.1/1-285 -------PAVLRTFSQRLSRGFNDA-ISG--FNDDG-----------WS--VM-GG----
XP_004986037.1/1-283 -------PAVLRTFSQRLSRGFNDA-ISG--FNDDG-----------WS--IM-GG----
XP_025795001.1/1-283 -------PAVLRTFSQRLSRGFNDA-ISG--FNDDG-----------WS--IM-GG----
XP_020093893.1/1-283 -------PAVLRTFSQRLSRGFNDA-ING--FNDDG-----------WS--LM-DS----
XP_020093894.1/1-282 -------PAVLRTFSQRLSRGFNDA-ING--FNDDG-----------WS--LM-DS----
XP_020259754.1/1-283 -------PAVLRTFSQKLSRGFNDA-INS--FVDDG-----------WS--VM-GC----
XP_009401955.1/1-282 -------PAILRTFGQRLNRGFNDA-ING--FTDDG-----------WS--LM-GS----
XP_008791541.1/1-283 -------PAVLRTFSQRLSRGFNDA-ING--FVDDG-----------WS--LM-GG----
XP_017698637.1/1-283 -------PAVLRTFSQRLSRGFNDA-ING--FVDDG-----------WS--LM-GG----
XP_010920422.1/1-283 -------PAVLRTFSQRLSRGFNDA-ING--FVDDG-----------WS--LM-GG----
XP_010920423.1/1-283 -------PAVLRTFSQRLSRGFNDA-ING--FVDDG-----------WS--LM-GG----
XP_010920419.1/1-283 -------PAVLRTFSQRLSRGFNDA-ING--FVDDG-----------WS--LM-GG----
XP_010920426.1/1-283 -------PAVLRTFSQRLSRGFNDA-ING--FVDDG-----------WS--LM-GG----
XP_019709326.1/1-287 -------PAVLRTFSQRLSRGFNDA-VNG--FVDDG-----------WS--LM-GG----
XP_008785281.1/1-283 -------PAVLRTFSQRLSRGFNDA-VNG--FVDDG-----------WS--VM-GS----
XP_008462593.1/1-288 -------PAVLRTFSQRLSRGFNDA-VNG--FNDDG-----------WS--LV-NC----
XP_022969629.1/1-286 -------PAVLRTFSQRLSRGFNEA-VNG--FNDDG-----------WS--LM-NY----
XP_023536970.1/1-286 -------PAVLRTFSQRLSRGFNEA-VNG--FNDDG-----------WS--LM-NC----
XP_022937855.1/1-288 -------PAVLRTFSQRLSRGFNEA-VNG--FNDDG-----------WS--LM-NC----
XP_022937858.1/1-288 -------PAVLRTFSQRLSRGFNEA-VNG--FNDDG-----------WS--LM-NC----
XP_012827869.1/1-285 -------PAVLRTFSQKLSRGFNDA-ING--FNDDG-----------WS--LL-NC----
XP_022874815.1/1-279 -------PAVLRTFSQRLIRGFNDA-ING--FSDDS-----------WS--IL-NF----
XP_022874814.1/1-283 -------PAVLRTFSQRLIRGFNDA-ING--FSDDS-----------WS--IL-NF----
XP_018434000.1/1-280 -------PAILRTFSQRLSRGFNDA-VNG--FNDDG-----------WS--IA-HC----
XP_013642496.1/1-279 -------PAVLRTFSQRLSRGFNDA-VNG--FNDDG-----------WS--IM-HC----
XP_009150087.1/1-279 -------PAVLRTFSQRLSRGFNDA-VNG--FNDDG-----------WS--IM-HC----
XP_009150088.1/1-279 -------PAVLRTFSQRLSRGFNDA-VNG--FNDDG-----------WS--IM-HC----
XP_022563181.1/1-279 -------PAVLRTFSQRLSRGFNDA-VNG--FNDDG-----------WS--IM-HC----
XP_013675790.1/1-280 -------PAVLRTFSQRLSRGFNDA-VNG--FNDDG-----------WS--IM-HC----
XP_013623090.1/1-279 -------PAVLRTFSQRLSRGFNDA-VNG--FNDDG-----------WS--IM-HC----
XP_013623089.1/1-280 -------PAVLRTFSQRLSRGFNDA-VNG--FNDDG-----------WS--IM-HC----
XP_020885782.1/1-285 -------PAVLRTFSQRLSRGFNDA-VNG--FGDDG-----------WS--TM-HC----
XP_006280004.1/1-285 -------PAVLRTFSQRLSRGFNDA-VNG--FGDDG-----------WS--TM-HC----
XP_019088563.1/1-285 -------PAVLRTFSQRLSRGFNDA-VNG--FGDDG-----------WS--TM-PC----
XP_010483719.1/1-285 -------PAVLRTFSQRLSRGFNDA-VNG--FGDDG-----------WS--TL-PC----
XP_010456679.1/1-285 -------PAVLRTFSQRLSRGFNDA-VNG--FGDDG-----------WS--TM-PC----
XP_006400821.1/1-284 -------PAVLRTFSQRLSRGFNDA-VNG--FGDDG-----------WS--TM-HC----
NP_200877.1/1-284    -------PAVLRTFSQRLSRGFNDA-VNG--FGDDG-----------WS--TM-HC----
XP_013611543.1/1-282 -------PAVLRTFSQRLSRGFNDA-VNG--FGDDG-----------WS--TM-HC----
XP_009120605.1/1-284 -------PAVLRTFSQRLSRGFNDA-VNG--FGDDG-----------WS--TM-HC----
XP_013667152.1/1-284 -------PAVLRTFSQRLSRGFNDA-VNG--FGDDG-----------WS--TM-HC----
XP_018446535.1/1-284 -------PAVLRTFSQRLSRGFNDA-VNG--FGDDG-----------WS--TM-HC----
XP_018454374.1/1-284 -------PAVLRTFSQRLSRGFNDA-VNG--FGDDG-----------WS--TM-HC----
XP_009126588.1/1-285 -------PAVLRTFSQRLSRGFNDA-VNG--FGDDG-----------WS--TM-HC----
XP_013680521.1/1-285 -------PAVLRTFSQRLSRGFNDA-VNG--FGDDG-----------WS--TM-HC----
XP_013612427.1/1-285 -------PAVLRTFSQRLSRGFNDA-VNG--FGDDG-----------WS--TM-HC----
XP_010535608.1/1-283 -------PAVLRAFSQRLSRGFNDA-VNG--FNDDG-----------WS--VM-HC----
XP_010520372.1/1-276 -------PAVLRIFSQRLSRGFNDA-VNG--FSDDG-----------WS--VM-HC----
XP_004487103.1/1-286 -------PAVLRTFSQRMSRGFNDA-VNG--FNDDG-----------WS--VL-NC----
XP_003597252.1/1-283 -------PAVLRTFSQRLSRGFNDA-VNG--FNDNG-----------WS--VL-NC----
XP_024455683.1/1-286 -------PAVLRTFNQRLSRGFNDA-ING--FNDDG-----------WS--LM-NA----
XP_022026352.1/1-288 -------PAVLRTLSQRLSRGFNDA-ING--FTDDG-----------WS--LM-NC----
XP_022000486.1/1-288 -------PAVLRTLTQRLSRGFNDA-VNG--FSDDG-----------WS--LM-NC----
XP_016717477.1/1-281 -------PAVLRTFSQRLSRGFNEA-ING--FNEDG-----------WS--IM-NC----
XP_017643014.1/1-281 -------PAVLRTFSQRLSRGFNEA-ING--FNEDG-----------WS--IM-NC----
XP_016724213.1/1-281 -------PAVLRTFSQRLSRGFNEA-ING--FNEDG-----------WS--IM-NC----
XP_012462938.1/1-281 -------PAVLRTFSQRLSRGFNEA-ING--FNEDG-----------WS--IM-NC----
XP_012471509.1/1-292 -------PAVLRTFSQRLSRGFNDA-ING--FNDDG-----------WS--IM-NC----
XP_017642742.1/1-292 -------PAVLRTFSQRLSRGFNDA-ING--FNDDG-----------WS--IM-NC----
XP_016755876.1/1-292 -------PAVLRTFSQRLSRGFNDA-ING--FNDDS-----------WS--IM-NC----
XP_012471510.1/1-286 -------PAVLRTFSQRLSRGFNDA-ING--FNDDG-----------WS--IM-NC----
XP_016753711.1/1-286 -------PAVLRTFSQRLSRGFNDA-ING--FNDDG-----------WS--IM-NC----
XP_017642744.1/1-286 -------PAVLRTFSQRLSRGFNDA-ING--FNDDG-----------WS--IM-NC----
XP_016755877.1/1-286 -------PAVLRTFSQRLSRGFNDA-ING--FNDDS-----------WS--IM-NC----
XP_016750468.1/1-286 -------PAVLRTFSQRLSRGFNDA-ING--FNDDG-----------WS--IM-NC----
XP_016688657.1/1-286 -------PAVLRTFSQRLSRGFNDA-ING--FNDDG-----------WS--IM-NC----
XP_012443272.1/1-286 -------PAVLRTFSQRLSRGFNDA-ING--FNDDG-----------WS--IM-NC----
XP_017603001.1/1-286 -------PAVLRTFSQRLSRGFNDA-ING--FNDDG-----------WS--IM-NC----
XP_022755632.1/1-285 -------PAVLRTFSQRLSRGFNDA-ING--FNDDG-----------WS--IM-NC----
XP_022755633.1/1-285 -------PAVLRTFSQRLSRGFNDA-ING--FNDDG-----------WS--IM-NC----
XP_022729815.1/1-286 -------PAVLRTFSQRLSRGFNDA-ING--FNDDG-----------WS--IM-NC----
XP_022729816.1/1-286 -------PAVLRTFSQRLSRGFNDA-ING--FNDDG-----------WS--IM-NC----
XP_022752857.1/1-286 -------PAVLRTFSQRLSRGFNDA-ING--FNDDG-----------WS--IM-NC----
XP_022752856.1/1-286 -------PAVLRTFSQRLSRGFNDA-ING--FNDDG-----------WS--IM-NC----
XP_021277981.1/1-286 -------PAVLRTFSQRLSRGFNDA-ING--FNDDG-----------WS--IM-NC----
XP_017983554.1/1-286 -------PAVLRTFSQRLSRGFNDA-ING--FNDDG-----------WS--IM-NC----
XP_011089912.1/1-283 -------PAVLRTFSQRLSRGFNDA-ING--FNDDG-----------WS--LL-NC----
XP_007208090.1/1-286 -------PAVLRTFSQRLIRGFNDA-VNG--FNDDG-----------WS--LI-NC----
XP_008225704.1/1-286 -------PAVLRTFSQRLIRGFNDA-VNG--FSDDG-----------WS--LI-NC----
XP_021830853.1/1-286 -------PAVLRTFSQRLIRGFNDA-VNG--FNDDG-----------WS--LI-NC----
XP_008349754.1/1-286 -------PAVLRTFSQRLIRGFNDA-VNG--FNDDG-----------WS--LV-NC----
NP_001315783.1/1-288 -------PAVLRTFSQRLIRGFNDA-VNG--FNDDG-----------WS--LV-NC----
XP_009367069.1/1-286 -------PAVLRTFSQRLIRGFNDA-VNG--FNDDG-----------WS--LV-NC----
XP_008344872.1/1-286 -------PAVLRTFSQRLIRGFNDA-VNG--FNDDG-----------WS--LV-NC----
XP_015952399.1/1-286 -------PAVLRTFSQRLSRGFNDA-VNG--FNDDG-----------WT--VL-NC----
XP_020982118.1/1-286 -------PAVLRTFSQRLSRGFNDA-VNG--FNDDG-----------WT--VL-NC----
XP_025685402.1/1-286 -------PAVLRTFSQRLSRGFNDA-VNG--FNDDG-----------WT--VL-NC----
XP_016187430.1/1-286 -------PAVLRTFSQRLSRGFNDA-VNG--FNDDG-----------WT--VL-NC----
XP_020214107.1/1-284 -------PAVLRTFSQRLSRGFNDA-VNG--FNDDG-----------WT--VL-NC----
XP_006592258.1/1-284 -------PAVLRTFSQRLSRGFNDA-VNG--FNDDG-----------WT--VL-NC----
XP_007132262.1/1-285 -------PAVLRTFSQRLSRGFNDA-VNG--FNDDG-----------WS--VL-NC----
XP_003538150.1/1-285 -------PAVLRTFSQRLSRGFNDA-VNG--FNDDG-----------WT--VL-NC----
XP_017433493.1/1-285 -------PAVLRTFSQRLSRGFNDA-VNG--FNDDG-----------WT--VL-NC----
XP_014494018.1/1-285 -------PAVLRTFSQRLSRGFNDA-VNG--FNDDG-----------WT--VL-NC----
XP_010089983.2/1-286 -------PAVLRTFSQRLIRGFNDA-VNG--FNDDG-----------WS--IL-NV----
XP_006378974.2/1-285 -------PAVLRTFSQRLSRGFNDA-ING--FNDDG-----------WS--LM-NS----
XP_011010051.1/1-285 -------PAVLRTFSQRLSRGFNDA-ING--FNDDG-----------WS--LM-NS----
XP_011010050.1/1-285 -------PAVLRTFSQRLSRGFNDA-ING--FNDDG-----------WS--LM-NS----
XP_021905657.1/1-285 -------PAVLRTFSQRLSRGFNDA-ING--FNDDG-----------WT--IM-HC----
XP_021610391.1/1-286 -------PAVLRTFSQKLSRGFNDA-VNG--FNDDG-----------WS--LM-SC----
XP_002529946.1/1-286 -------PAVLRTFSQRLSRGFNDA-ING--FNDDG-----------WS--LM-SC----
XP_021658728.1/1-286 -------PAVLRTFSQRLSRGFNDA-ING--FNDDG-----------WS--LM-SC----
XP_012064816.1/1-286 -------PAVLRTFSQRLSRGFNDA-ING--FNDDG-----------WS--LM-SC----
XP_021628343.1/1-286 -------PAVLRTFSQRLSRGFNDA-ING--FNDDG-----------WS--LM-SC----
XP_021657859.1/1-286 -------PAVLRTFSQRLSRGFNDA-ING--FNDDG-----------WS--LT-SC----
XP_015874442.1/1-285 -------PAVLRTFSQRLIRGFNDA-VNG--FNDDG-----------WS--LM-SC----
XP_023926843.1/1-286 -------PAVLRTFSQRLSRGFNDA-VNG--FNDDG-----------WS--LI-NS----
XP_018819529.1/1-286 -------PAVLRTFSQRLSRGFNDA-VNG--FNDDG-----------WS--LM-NG----
XP_010651164.1/1-286 -------PAVLRTFSQRLSRGFNDA-ING--FNDDG-----------WS--LM-SC----
XP_024034672.1/1-286 -------PAVLRTFSQRLSRGFNDA-VNG--FNDDG-----------WS--LM-TC----
XP_006488084.1/1-286 -------PAVLRTFSQRLSRGFNDA-VNG--FNDDG-----------WS--LM-TC----
XP_024034673.1/1-286 -------PAVLRTFSQRLSRGFNDA-VNG--FNDDG-----------WS--LM-TC----
XP_010264677.1/1-287 -------PAVLRTFSQRLSRGFNDA-ING--FNDDG-----------WS--LM-SC----
XP_010253680.1/1-288 -------PAVLRTFSQRLSRGFNDA-ING--FNDDG-----------WS--LM-SC----
XP_022005829.1/1-282 -------PAILRTLSQRLSRGFNDA-ING--FNDDG-----------WS--LM-NC----
XP_023751537.1/1-278 -------PAVLRTLSQRLSRGFNDA-ING--FNDDG-----------WS--LM-NC----
XP_024971452.1/1-283 -------PAVLRTLSQRLSRGFNDA-ING--FNDDG-----------WS--LM-NC----
XP_024971444.1/1-287 -------PAVLRTLSQRLSRGFNDA-ING--FNDDG-----------WS--LM-NC----
XP_024985647.1/1-287 -------PAVLRTLSQRLSRGFNDA-ING--FSDDG-----------WS--LM-NC----
XP_023749448.1/1-288 -------PAVLRTLSQRLSRGFNDA-ING--FSDDG-----------WS--LM-NC----
XP_010689538.1/1-281 -------PAVLRTFSQRLNRGFNDA-ING--FTDDG-----------WT--ML-NC----
XP_021858740.1/1-280 -------PAVLRTFSQRLSRGFNDA-ING--FPDDG-----------WT--LL-TC----
XP_021773317.1/1-281 -------PAVLRTFSQRLSRGFNDA-ING--FPDDG-----------WT--LL-TC----
XP_021745941.1/1-281 -------PAVLRTFSQRLSRGFNDA-ING--FPDDG-----------WT--LL-TC----
XP_016729039.1/1-286 -------PAVLRTFSQRLSRGFNDA-ING--FNDDG-----------WS--IM-NC----
XP_017623828.1/1-286 -------PAVLRTFSQRLSRGFNDA-ING--FNDDG-----------WS--IM-NC----
XP_012476406.1/1-286 -------PAVLRTFSQRLSRGFNDA-ING--FNDDG-----------WS--IM-NC----
XP_016754776.1/1-286 -------PAVLITFSQRLSRGFNDA-ING--FNDDG-----------WS--IM-NC----
XP_022152944.1/1-286 -------PAVLRTFSQRLSRGFNDA-VNG--FNDDG-----------WS--LI-NC----
XP_011040884.1/1-286 -------PAVLRTFNQRLSRGFNDA-ING--FNDDG-----------WS--LM-NA----
XP_010043753.1/1-286 -------PAVLRTFSQRLSRGFNDA-VNG--FNDGG-----------WS--LM-NG----
XP_024158223.1/1-285 -------PAVLRTFSQRLIRGFNDA-VNG--FNDDG-----------WS--LI-NC----
XP_004291573.1/1-285 -------PAVLRTFSQRLIRGFNDA-VNG--FNDDG-----------WS--LI-NC----
XP_018808765.1/1-285 -------PAVLRTFSQRLSRGFNDA-VNG--FNDDG-----------WS--LM-NC----
XP_023512455.1/1-283 -------PAVLRTFSQRLSRGFNDA-VNG--FNDNG-----------WS--LI-NC----
XP_023512456.1/1-283 -------PAVLRTFSQRLSRGFNDA-VNG--FNDNG-----------WS--LI-NC----
XP_022944186.1/1-283 -------PAVLRTFSQRLSRGFNDA-VNG--FNDNG-----------WS--LI-NC----
XP_022944185.1/1-283 -------PAVLRTFSQRLSRGFNDA-VNG--FNDNG-----------WS--LI-NC----
XP_022985614.1/1-283 -------PAVLRTFSQRLSRGFNDA-VNG--FNDNG-----------WS--LI-NC----
XP_022985615.1/1-283 -------PAVLRTFSQRLSRGFNDA-VNG--FNDNG-----------WS--LI-NC----
XP_011656996.1/1-283 -------PAVLRTFSQRLSRGFNDA-VNG--FNDNG-----------WS--LI-NC----
XP_011656997.1/1-283 -------PAVLRTFSQRLSRGFNDA-VNG--FNDNG-----------WS--LI-NC----
XP_008463945.1/1-283 -------PAVLRTFSQRLSRGFNDA-VNG--FNDNG-----------WS--LI-NC----
XP_008463946.1/1-283 -------PAVLRTFSQRLSRGFNDA-VNG--FNDNG-----------WS--LI-NC----
XP_022140363.1/1-286 -------PAVLRTFSQRLSRGFNDA-VNG--FNDNG-----------WS--LI-NC----
XP_022995706.1/1-286 -------PAVLRTFSQRLSRGFNDL-VNG--FNDNG-----------WS--LI-NC----
XP_023534373.1/1-286 -------PAVLRTFSQRLSRGFNDL-VNG--FNDNG-----------WS--LI-NC----
XP_022958713.1/1-286 -------PAVLRTFSQRLSRGFNDL-VNG--FNDNG-----------WS--LI-NC----
XP_025614062.1/1-285 -------PAVLRTFSQRLSRGFNDA-VNG--FNDDG-----------WS--VL-NC----
XP_025614063.1/1-285 -------PAVLRTFSQRLSRGFNDA-VNG--FNDDG-----------WS--VL-NC----
XP_016170485.1/1-285 -------PAVLRTFSQRLSRGFNDA-VNG--FNDDG-----------WS--VL-NC----
XP_016170486.1/1-285 -------PAVLRTFSQRLSRGFNDA-VNG--FNDDG-----------WS--VL-NC----
XP_015935757.1/1-285 -------PAVLRTFSQRLSRGFNDA-VNG--FNDDG-----------WS--VL-NC----
XP_015935758.1/1-285 -------PAVLRTFSQRLSRGFNDA-VNG--FNDDG-----------WS--VL-NC----
XP_004505999.1/1-286 -------PAVLRTFSQRLSRGFNDA-VNG--FNDDG-----------WS--IL-NC----
XP_013456006.1/1-286 -------PAVLRTFSQRLSRGFNDA-VNG--FSDDG-----------WS--IL-NC----
XP_019453966.1/1-285 -------PAVLRTFSQRLSRGFNDA-VNG--FNDDG-----------WT--VV-NC----
XP_019448740.1/1-279 -------PAVLRTFSQRLSRGFNDA-VNG--FNDDG-----------WT--IV-NC----
XP_019448741.1/1-279 -------PAVLRTFSQRLSRGFNDA-VNG--FNDDG-----------WT--IV-NC----
XP_019458095.1/1-286 -------PAVLRTFSQRLSRGFNNA-VNG--FNDDG-----------WS--VL-NC----
XP_019447870.1/1-286 -------PAVLRTFSQRLSRGFNHA-VNG--FNDDG-----------WS--VV-NC----
XP_012838629.1/1-280 -------PAVLRTFSQRLIRGFNDA-ING--FNDDG-----------WS--IL-SS----
XP_012838630.1/1-280 -------PAVLRTFSQRLIRGFNDA-ING--FNDDG-----------WS--IL-SS----
XP_012838627.1/1-280 -------PAVLRTFSQRLIRGFNDA-ING--FNDDG-----------WS--IL-SS----
XP_012838628.1/1-280 -------PAVLRTFSQRLIRGFNDA-ING--FNDDG-----------WS--IL-SS----
XP_017214964.1/1-286 -------PAVLRTFSQRLSRGFNDA-ING--FSDDG-----------WS--VM-NC----
XP_017234817.1/1-286 -------PAVLRTFSQRLSRGFNEA-ING--FNDDG-----------WS--VI-NC----
XP_019178153.1/1-282 -------PAVLRTFSQRLNRGFNDA-ING--FNDDG-----------WS--VL-SG----
XP_019166562.1/1-283 -------PAVLRTFSQRLSRGFNDA-ING--FNDDG-----------WS--VL-SG----
XP_011089294.1/1-282 -------PAVLRTFSQRLSRGFNDA-ING--FNDDG-----------WS--IL-NC----
XP_022892122.1/1-274 ----------------RLSRGFNDA-INS--FNDDG-----------WS--LL-NS----
XP_022883393.1/1-279 -------PAVLRTFSQRLSRGFNDA-INS--FNDDG-----------WS--LL-NS----
XP_022883398.1/1-279 -------PAVLRTFSQRLSRGFNDA-INS--FNDDG-----------WS--LL-NS----
XP_022883384.1/1-283 -------PAVLRTFSQRLSRGFNDA-INS--FNDDG-----------WS--LL-NS----
XP_022883389.1/1-283 -------PAVLRTFSQRLSRGFNDA-INS--FNDDG-----------WS--LL-NS----
XP_009776589.1/1-283 -------PAVLRTFSQRLSRGFNDA-ING--FSDDG-----------WS--LL-SS----
NP_001312504.1/1-283 -------PAVLRTFSQRLSRGFNDA-ING--FSDDG-----------WS--LL-SS----
XP_016472209.1/1-283 -------PAVLRTFSQRLSRGFNDA-ING--FSDDG-----------WS--LL-SS----
XP_019255429.1/1-283 -------PAVLRTFSQRLSRGFNDA-ING--FSDDG-----------WS--LL-SS----
XP_009624234.1/1-283 -------PAVLRTFSQRLSRGFNDA-ING--FSDDG-----------WS--LL-SS----
XP_016504967.1/1-283 -------PAVLRTFSQRLSRGFNDA-ING--FSDDG-----------WS--LL-SS----
XP_016547131.1/1-283 -------PAVLRTFSQRLCRGFNDA-ING--FSDDG-----------WS--ML-SS----
XP_004251138.1/1-283 -------PAVLRTFSQRLCRGFNDA-ING--FGDDG-----------WS--ML-SS----
XP_015058512.1/1-283 -------PAVLRTFSQRLCRGFNDA-ING--FGDDG-----------WS--ML-SS----
XP_006340193.1/1-283 -------PAVLRTFSQRLCRGFNDA-ING--FGDDG-----------WS--ML-SS----
XP_004235650.1/1-283 -------PAALRALSQRLSRGFNEA-LNG--IADEG-----------WS--ML-DS----
XP_006343047.1/1-283 -------PAALRALSQRLSRGFNEA-LNG--IADEG-----------WS--ML-DS----
XP_009774805.1/1-285 -------PAALRALSQRLSRGFNEA-LNG--LTDEG-----------WS--ML-GS----
XP_016498616.1/1-285 -------PAALRALSQRLSRGFNEA-LNG--LTDEG-----------WS--ML-GS----
XP_016434103.1/1-285 -------PAALRALSQRLSRGFNEA-LNG--LTDEG-----------WS--ML-GS----
XP_009593967.1/1-285 -------PAALRALSQRLSRGFNEA-LNG--LTDEG-----------WS--ML-GS----
XP_004510297.1/1-285 -------PAALRALSQRLSRGFNEA-LNG--FTDEG-----------WT--MM-GN----
XP_004510296.1/1-285 -------PAALRALSQRLSRGFNEA-LNG--FTDEG-----------WT--MM-GN----
XP_003627005.1/1-284 -------PAALRALSQRLSRGFNEA-LNG--FTDEG-----------WT--MM-GN----
XP_003531653.1/1-284 -------PSALRALSQRLSRGFNEA-LNG--FTDEG-----------WT--TI-GN----
XP_006585576.1/1-288 -------PSALRALSQRLSRGFNEA-LNG--FTDEG-----------WT--TI-GN----
XP_014634618.1/1-288 -------PSALRALSQRLSRGFNEA-LNG--FTDEG-----------WT--TI-GN----
XP_014634619.1/1-288 -------PSALRALSQRLSRGFNEA-LNG--FTDEG-----------WT--TI-GN----
XP_003531652.1/1-285 -------PAALRALSQRLSRGFNEA-LNG--FTDEG-----------WT--TI-SN----
XP_003530109.1/1-284 -------PAALRALSQRLSRGFNEA-LNG--FTDEG-----------WT--TI-GN----
XP_007135643.1/1-285 -------PAALRALSQRLSRGFNEA-LNG--FTDEG-----------WT--TI-GN----
XP_003530112.1/1-285 -------PAALRALSQRLSRGFNEA-LNG--FTDEG-----------WT--TI-GN----
XP_020226142.1/1-285 -------PAALRALSQRLSRGFNEA-LNG--FTDEG-----------WT--TI-GN----
XP_014516466.1/1-285 -------PAALRALSQRLSRGFNEA-LNG--FTDEG-----------WT--TI-GN----
XP_017407594.1/1-285 -------PAALRALSQRLSRGFNEA-LNG--FTDEG-----------WT--TI-GN----
XP_019448178.1/1-285 -------PAALRALGQRLSRGFNEA-LNG--FTDEG-----------WS--TI-AN----
XP_019429328.1/1-285 -------PAALRALSQRLCRGFNEA-LNG--FTDEG-----------WS--TI-GN----
XP_004486776.1/1-285 -------PAALRALGQRLSRGFNEA-LNG--FTDEG-----------WS--MI-GN----
XP_003597690.1/1-286 -------PAALRALGQRLSRGFNEA-LNG--FTDEG-----------WS--MI-GN----
XP_015932679.1/1-285 -------PAALRALGQRLSRGFNEA-LNG--FTDEG-----------WS--TI-GN----
XP_025674177.1/1-285 -------PAALRALGQRLSRGFNEA-LNG--FTDEG-----------WS--TI-GN----
XP_025610204.1/1-285 -------PAALRALGQRLSRGFNEA-LNG--FTDEG-----------WS--TI-GN----
XP_020226055.1/1-285 -------PAALRALGQRLSRGFNEA-ING--FTDEG-----------WS--MI-GN----
XP_007150614.1/1-285 -------PAALRALGQRLSRGFNEA-ING--FTDEG-----------WS--MI-GN----
XP_014501306.1/1-285 -------PAALRALGQRLSRGFNEA-ING--FTDEG-----------WS--MI-GN----
XP_017425270.1/1-285 -------PAALRALGQRLSRGFNEA-ING--FTDEG-----------WS--MI-GN----
XP_021836126.1/1-285 -------PAALRALSQRLNRGFNEA-LNG--FTDEG-----------WS--MI-GN----
XP_010691423.1/1-284 -------PAALRALSQRLSRGFNEA-LNG--FTDEG-----------WS--MI-GN----
XP_010691424.1/1-284 -------PAALRALSQRLSRGFNEA-LNG--FTDEG-----------WS--MI-GN----
XP_021765330.1/1-284 -------PAALRALSQRLSRGFNEA-LNG--FTDEG-----------WS--MI-GN----
XP_021749885.1/1-284 -------PAALRALSQRLSRGFNEA-LNG--FTDEG-----------WS--MI-GN----
XP_010267183.1/1-285 -------PAALRALSQRLSRGFNEA-LNG--FTDEG-----------WS--MM-GN----
XP_017258627.1/1-285 -------PVALRALSQRLSRGFNEA-LNG--FTDEG-----------WS--LM-GT----
XP_023000315.1/1-284 -------PAALRALGQRLTRGFNEA-LNG--FNDEG-----------WS--VM-GN----
XP_023000313.1/1-284 -------PAALRALGQRLTRGFNEA-LNG--FNDEG-----------WS--VM-GN----
XP_023514554.1/1-284 -------PAALRALGQRLTRGFNEA-LNG--FNDEG-----------WS--VM-GN----
XP_023514552.1/1-284 -------PAALRALGQRLTRGFNEA-LNG--FNDEG-----------WS--VM-GN----
XP_022964458.1/1-284 -------PAALRALGQRLTRGFNEA-LNG--FNDEG-----------WS--VM-GN----
XP_022964454.1/1-284 -------PAALRALGQRLTRGFNEA-LNG--FNDEG-----------WS--VM-GN----
XP_022147627.1/1-284 -------PAALRALGQRLTRGFNEA-LNG--FNDEG-----------WS--VM-GN----
XP_022147619.1/1-284 -------PAALRALGQRLTRGFNEA-LNG--FNDEG-----------WS--VM-GN----
XP_008462437.1/1-284 -------PAALRSLGQRLTRGFNEA-LNG--FNDEG-----------WS--VM-GN----
XP_004141659.1/1-284 -------PAALRSLGQRLTRGFNEA-LNG--FNDEG-----------WS--VM-GN----
XP_008462436.1/1-284 -------PAALRSLGQRLTRGFNEA-LNG--FNDEG-----------WS--VM-GN----
XP_011659682.1/1-284 -------PAALRSLGQRLTRGFNEA-LNG--FNDEG-----------WS--VM-GN----
XP_022992111.1/1-284 -------PAALRSLGQRLTRGFNEA-LNG--FNDEG-----------WS--VM-GN----
XP_023548534.1/1-284 -------PAALRSLGQRLTRGFNEA-LNG--FNDEG-----------WS--VM-GN----
XP_022953371.1/1-284 -------PAALRSLGQRLTRGFNEA-LNG--FNDEG-----------WS--VM-GN----
XP_002304217.1/1-285 -------PAALRALSQRLSRGFNEA-LNG--FSDEG-----------WS--MI-GN----
XP_011013041.1/1-285 -------PAALRALSQRLSRGFNEA-LNG--FSDEG-----------WS--MI-GN----
XP_011013042.1/1-285 -------PAALRALSQRLSRGFNEA-LNG--FSDEG-----------WS--MI-GN----
XP_011046759.1/1-284 -------PAALRALSQRLSRGFNEA-LNG--FSDEG-----------WS--MI-GN----
XP_024464085.1/1-284 -------PAALRALSQRLSRGFNEA-LNG--FSDEG-----------WS--MI-GN----
XP_024183498.1/1-286 -------PAALRALSQRLSRGFNEA-LNG--FTDEG-----------WS--LM-GN----
XP_004302866.1/1-286 -------PAALRALSQRLSRGFNEA-LNG--FTDEG-----------WS--LM-GN----
NP_001280898.1/1-285 -------PAALRALSQRLSRGFNDA-LNG--FTDEG-----------WS--MM-GN----
XP_007214960.1/1-285 -------PAALRALSQRLSRGFNEA-LNG--FTDEG-----------WS--MM-GN----
XP_008228437.1/1-285 -------PAALRALSQRLSRGFNEA-LNG--FTDEG-----------WS--MM-GN----
XP_021818763.1/1-285 -------PAALRALSQRLSRGFNEA-LNG--FTDEG-----------WS--MM-GN----
XP_022723515.1/1-285 -------PAALRALSQRLSRGFNEA-LNG--FTDEG-----------WS--MM-GN----
XP_016688508.1/1-285 -------PAALRALSQRLSRGFNDA-VNG--FTDEG-----------WS--MM-GN----
XP_012443274.1/1-285 -------PAALRALSQRLSRGFNDA-VNG--FTDEG-----------WS--MM-GN----
XP_022721457.1/1-285 -------PAALRALSQRLSRGFNEA-VNG--FTDEG-----------WS--MM-GN----
XP_022715018.1/1-285 -------PAALRALSQRLSRGFNEA-LNG--FADEG-----------WS--MM-GN----
XP_017979082.1/1-285 -------PAALRALSQRLSRGFNEA-LNG--FTDEG-----------WS--MM-GN----
XP_021293768.1/1-285 -------PAALRALSQRLSRGFNEA-LNG--FTDEG-----------WS--MM-GN----
XP_010062518.1/1-285 -------PAALRALSQRLSRGFNEA-LNG--FTDEG-----------WS--IM-GN----
XP_006426893.1/1-285 -------PAALRALSQRLSRGFNEA-VNG--FTDEG-----------WT--VM-GN----
XP_021610318.1/1-285 -------PAALRALSQRLSRGFNEA-LNG--FTDEG-----------WS--VM-GN----
XP_010098130.1/1-285 -------PAALRVLSQRLSRGFNEA-LNG--FTDEG-----------WS--MM-GS----
XP_021635282.1/1-296 -------PAALRALSQRLSRGFNEA-LNG--FTDEG-----------WS--MM-GN----
XP_002284003.2/1-285 -------PAALRALSQRLSRGFNEA-LNG--FTDEG-----------WS--MM-GN----
XP_002515977.1/1-285 -------PAALRALSQRLSRGFNEA-LNG--FTDEG-----------WS--MM-GN----
XP_021643260.1/1-285 -------PAALRSLSQRLSRGFNEA-LNG--FTDEG-----------WS--MM-GN----
XP_021628194.1/1-285 -------PAALRALSQRLSRGFNEA-LNG--FTDEG-----------WS--MM-GN----
XP_015894591.1/1-285 -------PAALRALSQRLSRGFNEA-LNG--FTDEG-----------WS--MM-GN----
XP_012069098.1/1-285 -------PAALRALSQRLSRGFNEA-LNG--FTDEG-----------WS--MM-GN----
XP_018847857.1/1-285 -------PAALRALSQRLSRGFNEA-LNG--FTDEG-----------WS--MM-GN----
XP_023898511.1/1-285 -------PAALRALSQRLSRGFNEA-LNG--FTDEG-----------WS--MM-GN----
XP_010545673.1/1-285 -------PAALRALSQRLSRGFNEA-VNG--FTDEG-----------WS--VI-G-----
XP_010545670.1/1-286 -------PAALRALSQRLSRGFNEA-VNG--FTDEG-----------WS--VI-G-----
XP_010535179.1/1-285 -------PAALRALSQRLSRGFNEA-VNG--FADEG-----------WS--VI-G-----
XP_018436929.1/1-283 -------PAALRALSQRLSRGFNES-VNS--FTDEG-----------WS--VI-G-----
XP_013592012.1/1-283 -------PAALRALSQRLSRGFNEA-VNG--FTDEG-----------WS--VM-G-----
XP_013640375.1/1-284 -------PAALRALSQRLSRGFNEA-VNG--FTDEG-----------WS--VI-G-----
XP_013640376.1/1-283 -------PAALRALSQRLSRGFNEA-VNG--FTDEG-----------WS--VI-G-----
XP_009147709.1/1-283 -------PAALRALSQRLSRGFNEA-VNG--FTDEG-----------WS--VI-G-----
XP_006306297.1/1-281 -------PAALRALSQRLSRGFNEA-VNG--FTDEG-----------WS--VI-G-----
XP_010462051.1/1-281 -------PAALRALSQRLSRGFNEA-VNG--FTDEG-----------WS--VI-G-----
XP_010479714.1/1-281 -------PAALRALSQRLSRGFNEA-VNG--FTDEG-----------WS--VI-G-----
XP_010462052.1/1-281 -------PAALRALSQRLSRGFNEA-VNG--FTDEG-----------WS--VI-G-----
XP_010500807.1/1-281 -------PAALRALSQRLSRGFNEA-VNG--FTDEG-----------WS--VI-G-----
NP_849795.1/1-282    -------PAALRALSQRLSRGFNEA-VNG--FTDEG-----------WS--VI-G-----
NP_001077702.1/1-281 -------PAALRALSQRLSRGFNEA-VNG--FTDEG-----------WS--VI-G-----
NP_175627.1/1-281    -------PAALRALSQRLSRGFNEA-VNG--FTDEG-----------WS--VI-G-----
XP_020868586.1/1-281 -------PAALRALSQRLSRGFNEA-VNG--FTDEG-----------WS--VI-G-----
XP_006392953.1/1-282 -------PAALRALSQRLSRGFNEA-VNG--FTDEG-----------WS--VI-G-----
XP_018460807.1/1-282 -------PAALRALSQRLSRGFNEA-VNG--FTDEG-----------WS--VI-G-----
XP_013621760.1/1-283 -------PAALRALSQRLSRGFNEA-VNG--FTDEG-----------WS--VI-G-----
XP_018451524.1/1-282 -------PAALRALSQRLSRGFNEA-VNG--FTDEG-----------WS--VI-G-----
XP_009107148.1/1-282 -------PAALRALSQRLSRGFNEA-VNG--FTDEG-----------WS--VI-G-----
XP_009107147.1/1-283 -------PAALRALSQRLSRGFNEA-VNG--FTDEG-----------WS--VI-G-----
XP_009600774.1/1-284 -------PAALRALSKKLSRGFNEA-LNG--FSSEG-----------WS--ML-DN----
XP_016459079.1/1-284 -------PAALRALSKKLSRGFNEA-LNG--FSSEG-----------WS--ML-DN----
XP_019236029.1/1-285 -------PAALRALSKKLSRGFNEA-LNG--FSSEG-----------WS--ML-DN----
XP_009771098.1/1-285 -------PAALRALSKKLSRGFNEA-LNG--FSSEG-----------WS--MR-DN----
XP_016550962.1/1-285 -------PAALRALSKRLSRGFNEA-LNG--FSSEG-----------WS--MQ-DN----
XP_016550960.1/1-285 -------PAALRALSKRLSRGFNEA-LNG--FSSEG-----------WS--MQ-DN----
XP_016550961.1/1-285 -------PAALRALSKRLSRGFNEA-LNG--FSSEG-----------WS--MQ-DN----
XP_006359469.1/1-285 -------PAALRALSKRLNRGFNEA-LNG--FSSEG-----------WS--ML-DN----
XP_004252354.1/1-285 -------PAALRALSKRLNRGFNEA-LNG--FSSEG-----------WS--ML-DN----
XP_015061298.1/1-285 -------PAALRALSKRLNRGFNEA-LNG--FSSEG-----------WS--ML-DN----
XP_019198775.1/1-285 -------PAALRALRQRLSRGFNEA-VNG--FTDEG-----------WS--LL-GN----
XP_019194412.1/1-285 -------PAALRALSHRLSRGFNEA-LNG--FTDEG-----------WS--LL-GN----
XP_016562656.1/1-283 -------PAALRALGQRLSRGFNEA-LNG--LADEG-----------WS--ML-DS----
XP_019267274.1/1-285 -------PAALRALSQRLNRGFNEA-LNG--LTDEG-----------WS--ML-GS----
XP_019267275.1/1-282 -------PAALRALS---QRGFNEA-LNG--LTDEG-----------WS--ML-GS----
XP_022889649.1/1-285 -------PAALRALSQRLTRGFNEV-LNG--FSDEG-----------WS--LV-GN----
XP_022889648.1/1-285 -------PAALRALSQRLTRGFNEV-LNG--FSDEG-----------WS--LV-GN----
XP_012852689.1/1-281 -------PAALRALSHRLSRGFNEA-LNG--FGDDG-----------WS--LI-GN----
XP_012856468.1/1-279 -------PAALRALSHRLSRGFNEA-LNG--FNDEG-----------WS--LL-GN----
XP_011080516.1/1-285 -------PAALRALSHRLSRGFNEA-LNG--LSDEG-----------WS--LI-AN----
XP_011072422.1/1-285 -------PAALRALSHRLGRGFNEA-LNG--FSDDG-----------WS--LV-GN----
XP_022851148.1/1-285 -------PAALRALGHRLSRGFNEA-LNG--FSDEG-----------WS--LM-GS----
XP_022844668.1/1-285 -------PAALRALSHRLSRGFNEA-LNG--FSDDG-----------WS--LM-GN----
XP_006853643.2/1-288 -------PAALRALSQRLSKGFNEA-LNG--FTDDG-----------WS--LL-GN----
XP_010249545.1/1-285 -------PAALRALSQRLSRGFNEA-LNG--FTDEG-----------WS--MM-GN----
XP_010249539.1/1-285 -------PAALRALSQRLSRGFNEA-LNG--FTDEG-----------WS--MM-GN----
XP_010915409.1/1-284 -------PAALRALSQRLSRGFNEA-LNG--FADDG-----------WS--MI-SG----
XP_008791633.1/1-284 -------PAALRALSQRLSRGFNEA-LNG--FADDG-----------WS--MI-GS----
XP_017235966.1/1-285 -------PAALRALSQKLSRGFNEA-ING--FADEG-----------WS--LM-GS----
XP_023761873.1/1-288 -------PAALRSLSQRLSRGFNEA-VNG--FTDEG-----------WS--LM-GN----
XP_021997125.1/1-286 -------PAALRALSQRLSRGFNEA-VSG--FTDEG-----------WS--LT-SN----
XP_021990052.1/1-285 -------PAALRALSQRLSRGFNEA-VNG--FTDEG-----------WS--LT-SN----
XP_024979339.1/1-285 -------PAALRALSQRLSRGFNEA-VNG--FTDEG-----------WS--LM-GT----
XP_024979341.1/1-290 -------PAALRALSQRLSRGFNEA-VNG--FTDEG-----------WS--LM-GT----
XP_024979340.1/1-291 -------PAALRALSQRLSRGFNEA-VNG--FTDEG-----------WS--LM-GT----
XP_024979342.1/1-291 -------PAALRALSQRLSRGFNEA-VNG--FTDEG-----------WS--LM-GT----
XP_022016208.1/1-285 -------PAALRALSQRLSRGFNEA-VNG--FTDEG-----------WL--LI-NN----
XP_022016207.1/1-311 -------PAALRALSQRLSRGFNEA-VNG--FTDEG-----------WL--LI-NN----
XP_022009819.1/1-285 -------PAELRALSQRLSRGFNEA-LNG--FTTEG-----------WS--LM-AT----
XP_022009815.1/1-285 -------PAELRALSQRLSRGFNEA-LNG--FTTEG-----------WS--LM-AT----
XP_022009809.1/1-285 -------PAELRALSQRLSRGFNEA-LNG--FTTEG-----------WS--LM-AT----
XP_023759500.1/1-286 -------PAALRGLSQRLSRGFNEA-LNG--FPDEG-----------WS--LM-GN----
XP_023751740.1/1-285 -------PAALRALSQRLSRGFNEA-LNG--FTDEG-----------WS--LI-AN----
XP_024971803.1/1-285 -------PAALRALSQRLSRGFNEA-LNG--FSDEG-----------WS--LM-GN----
XP_022033649.1/1-285 -------PAALRALSQRLSRGFNEA-LNG--FNDEG-----------WS--LM-AN----
XP_024972195.1/1-285 -------PAALRALSQRLSRGFNEA-LNG--FTDEG-----------WS--LM-AN----
XP_024972196.1/1-289 -------PAALRALSQRLSRGFNEA-LNG--FTDEG-----------WS--LM-AN----
XP_020248539.1/1-284 -------PAALRALSQRLSRGFNEA-LNG--FADDG-----------WS--II-NS----
XP_020269197.1/1-284 -------PAALRTLSQRLCRGFNEA-LNG--FSDDG-----------WS--II-NT----
XP_020269196.1/1-284 -------PAALRTLSQRLCRGFNEA-LNG--FSDDG-----------WS--II-NT----
XP_009399813.1/1-284 -------PAALRALSQRLSRGFNEA-VNG--FVDDG-----------WS--MV-SS----
XP_009399812.1/1-284 -------PAALRALSQRLSRGFNEA-VNG--FVDDG-----------WS--MV-SS----
XP_009413853.1/1-284 -------PAALRALSQRLSRGFNEA-VNG--FADDG-----------WS--MV-TT----
XP_024985513.1/1-286 -------PAALRAIGQRMSKGFNEA-ING--FTDEG-----------WS--LM-ES----
XP_023729871.1/1-286 -------PSALRSLGQRMSRGFNEA-ING--FSDEG-----------WS--MM-ES----
XP_022000974.1/1-282 -------PAALRALGQRMSRGFNEA-ING--FSDEG-----------WT--MM-ES----
XP_022023495.1/1-282 -------PAALRALAQRMSRGFNEA-ING--FTDEG-----------WT--MM-ES----
XP_010526102.1/1-286 -------PAALRALSQRLSKGFNEA-VNG--FSDEG-----------WS--MI-ES----
XP_019086035.1/1-282 -------PAALRALSQRLSKGFNEA-VNG--FSDEG-----------WS--ML-ES----
XP_010437837.1/1-288 -------PAALRALSQRLSKGFNEA-VNG--FSDEG-----------WS--ML-ES----
XP_010437838.1/1-288 -------PAALRALSQRLSKGFNEA-VNG--FSDEG-----------WS--ML-ES----
XP_022549566.1/1-283 -------PAALRALSQRLSKGFNEA-VNG--FSDEG-----------WS--ML-ES----
XP_013670651.1/1-283 -------PAALRALSQRLSKGFNEA-VNG--FSDEG-----------WS--ML-ES----
XP_020874560.1/1-283 -------PAALRALSQRLSKGFNEA-VNG--FSDEG-----------WS--IL-ES----
XP_020874561.1/1-283 -------PAALRALSQRLSKGFNEA-VNG--FSDEG-----------WS--IL-ES----
XP_020874562.1/1-283 -------PAALRALSQRLSKGFNEA-VNG--FSDEG-----------WS--IL-ES----
NP_195014.1/1-283    -------PAALRALSQRLSKGFNEA-VNG--FSDEG-----------WS--IL-ES----
XP_006412419.1/1-283 -------PAALRALSQRLSKGFNEA-VNG--FSDEG-----------WS--ML-ES----
XP_006412418.1/1-283 -------PAALRALSQRLSKGFNEA-VNG--FSDEG-----------WS--ML-ES----
XP_018471819.1/1-283 -------PAALRALSQRLSKGFNEA-VNG--FSDEG-----------WS--ML-ES----
XP_009125299.1/1-283 -------PAALRALSQRLSKGFNEA-VNG--FSDEG-----------WS--ML-ES----
XP_010432643.1/1-283 -------PAALRALSQRLSKGFNEA-VNG--FSDEG-----------WS--ML-ES----
XP_010432644.1/1-283 -------PAALRALSQRLSKGFNEA-VNG--FSDEG-----------WS--ML-ES----
XP_006283109.1/1-283 -------PAALRALSQRLSKGFNEA-VNG--FSDEG-----------WS--ML-ES----
XP_010437839.1/1-283 -------PAALRALSQRLSKGFNEA-VNG--FSDEG-----------WS--ML-ES----
XP_010437840.1/1-283 -------PAALRALSQRLSKGFNEA-VNG--FSDEG-----------WS--ML-ES----
XP_010447325.1/1-283 -------PAALRALSQRLSKGFNEA-VNG--FSDEG-----------WS--ML-ES----
XP_010447326.1/1-283 -------PAALRALSQRLSKGFNEA-VNG--FSDEG-----------WS--ML-ES----
XP_013622315.1/1-283 -------PAALRALSQRLSKGFNEA-VNG--FSDEG-----------WS--ML-ER----
XP_013656273.1/1-283 -------PAALRALSQRLSKGFNEA-VNG--FSDEG-----------WS--MV-ES----
XP_009108824.1/1-283 -------PAALRALSQRLSKGFNEA-VNG--FSDEG-----------WS--ML-ES----
XP_013730326.1/1-283 -------PAALRALSQRLSKGFNEA-VNG--FSDEG-----------WS--ML-ES----
XP_013730325.1/1-283 -------PAALRALSQRLSKGFNEA-VNG--FSDEG-----------WS--ML-ES----
XP_018447492.1/1-283 -------PAALRALSQRLSKGFNEA-VNG--FSDEG-----------WS--ML-ES----
XP_019415259.1/1-283 -------PAALRALSQRLSKGFNEA-VNG--FADDG-----------WS--ML-ES----
XP_022980940.1/1-286 -------PTALRALSQRLSRGFNEA-VNG--FTDEG-----------WS--LL-EN----
XP_023523539.1/1-286 -------PAALRALSQRLSRGFNEA-VNG--FTDEG-----------WS--LL-EN----
XP_022941184.1/1-286 -------PAALRALSQRLSRGFNEA-VNG--FTDEG-----------WS--LL-EN----
XP_022134366.1/1-286 -------PAALRALSQRLSRGFNEA-VNG--FTDEG-----------WS--LL-ES----
XP_004136224.1/1-286 -------PAALRALSQKLSRGFNEA-VNG--FTDEG-----------WS--LL-EN----
XP_008466070.1/1-286 -------PAALRALSQKLSRGFNEA-VNG--FTDEG-----------WS--LL-EN----
XP_022976315.1/1-286 -------PAALRALSQKLSRGFNEA-VNG--FTDEG-----------WS--LL-EN----
XP_022937345.1/1-286 -------PAALRSLSQKLSRGFNEA-VNG--FTDEG-----------WS--LL-EN----
XP_023534956.1/1-286 -------PAALRALSQKLSRGFNEA-VNG--FTDEG-----------WS--LL-EN----
XP_023535295.1/1-286 -------PAALRALSQKLSRGFNEA-VNG--FTDEG-----------WS--LL-EN----
XP_019175908.1/1-285 -------PAALRALSQRLSKGFNEA-VNG--FTDEG-----------WS--MI-ES----
XP_019175907.1/1-285 -------PAALRALSQRLSKGFNEA-VNG--FTDEG-----------WS--MI-ES----
XP_009626659.1/1-285 -------PAALRALGQRLSKGFNEA-VNG--FTDEG-----------WS--ML-ES----
XP_019266312.1/1-285 -------PAALRALGQRLSKGFNEA-VNG--FTDEG-----------WS--ML-ES----
XP_009781184.1/1-285 -------PAALRALGQRLSKGFNEA-VNG--FTDEG-----------WS--ML-ES----
XP_016488110.1/1-285 -------PAALRALGQRLSKGFNEA-VNG--FTDEG-----------WS--ML-ES----
XP_016537761.1/1-285 -------PAALRALGQRLSKGFNEA-VNG--FTDEG-----------WS--ML-ES----
XP_006359741.1/1-285 -------PAALRALGQRLSKGFNEA-VNG--FTDEG-----------WS--ML-ES----
XP_010325078.1/1-288 -------PAALRALGQRLSKGFNEA-VNG--FTDEG-----------WS--ML-ES----
XP_004245155.1/1-285 -------PAALRALGQRLSKGFNEA-VNG--FTDEG-----------WS--ML-ES----
XP_015085523.1/1-285 -------PAALRALGQRLSKGFNEA-VNG--FTDEG-----------WS--ML-ES----
XP_022868182.1/1-285 -------PAALRALAQRLSRGFNEA-VNG--FTDES-----------WS--MI-ES----
XP_022868181.1/1-285 -------PAALRALAQRLSRGFNEA-VNG--FTDES-----------WS--MI-ES----
XP_022854559.1/1-285 -------PAALRALGQRLSRGFNEA-VNG--FTDEA-----------WS--MV-ES----
XP_011081776.1/1-285 -------PAALRALSQRLSRGFNEA-VNG--FTDEG-----------WS--MI-ES----
XP_012857948.1/1-282 -------PAALRALGQRLSRGFNEA-VNG--FADEG-----------WS--MI-ES----
XP_021847759.1/1-284 -------PAALRALSQRLSKGFNEA-VNG--FMDEG-----------WS--MM-ES----
XP_010668539.1/1-284 -------PAALRALSQRLSKGFNEA-VNG--FMDEG-----------WS--MM-DS----
XP_021739137.1/1-284 -------PAALRALSQRLSKGFNEA-VNG--FMDEG-----------WS--MM-DS----
XP_021737291.1/1-284 -------PAALRALSQRLSKGFNEA-VNG--FMDEG-----------WS--MM-DS----
XP_010047286.1/1-285 -------PAALRALAIYLDRGFNEA-VNG--FMDDG-----------WS--ML-ES----
XP_003603630.2/1-285 -------PAALRALSQRLSKGFNEA-VNG--FTDDG-----------WS--ML-DS----
XP_004501146.1/1-286 -------PTALRALSQRLSKGFNEA-VNG--FTDDG-----------WS--ML-ES----
XP_012571693.1/1-286 -------PTALRALSQRLSKGFNEA-VNG--FTDDG-----------WS--ML-ES----
XP_012571692.1/1-286 -------PTALRALSQRLSKGFNEA-VNG--FTDDG-----------WS--ML-ES----
XP_019417530.1/1-285 -------PVALRALSQRLSKGFNEA-VNG--FADDG-----------WS--ML-ES----
XP_019417531.1/1-285 -------PVALRALSQRLSKGFNEA-VNG--FADDG-----------WS--ML-ES----
XP_016180268.1/1-286 -------PAALRALSQRLSKGFNEA-VNG--FTDDG-----------WS--LL-ES----
XP_025623723.1/1-286 -------PAALRALSQRLSKGFNEA-VNG--FTDDG-----------WS--LL-ES----
XP_015945672.1/1-286 -------PAALRALSQRLSKGFNEA-VNG--FTDDG-----------WS--LL-ES----
XP_007137231.1/1-287 -------PAALRALSQRLSKGFNEA-VNG--FADDG-----------WS--ML-ES----
XP_022636494.1/1-293 -------PAALRALSQRLSKGFNEA-VNG--FADDG-----------WS--ML-ES----
XP_017409217.1/1-287 -------PAALRALSQRLSKGFNEA-VNG--FADDG-----------WS--ML-ES----
XP_014501941.1/1-287 -------PAALRALSQRLSKGFNEA-VNG--FADDG-----------WS--ML-ES----
XP_003526496.1/1-286 -------PAALRALSQRLSKGFNEA-VNG--FADDG-----------WS--ML-ES----
XP_003522716.1/1-286 -------PAALRALSQRLSKGFNEA-VNG--FADDG-----------WS--ML-ES----
XP_020217689.1/1-286 -------PAALRALSQRLSKGFNEA-VNG--FADDG-----------WS--ML-ES----
XP_016683069.1/1-283 -------PAALRALSHKLSKGFNEA-VNG--FTDEG-----------WS--ML-ES----
XP_016677926.1/1-283 -------PAALRALSHKLSKGFNEA-VNG--FTDEG-----------WS--ML-ES----
XP_017643110.1/1-283 -------PAALRALSHKLSKGFNEA-VNG--FTDEG-----------WS--ML-ES----
XP_017643111.1/1-283 -------PAALRALSHKLSKGFNEA-VNG--FTDEG-----------WS--ML-ES----
XP_012451775.1/1-283 -------PAALRALSHKLSKGFNEA-VNG--FTDEG-----------WS--ML-ES----
XP_016744766.1/1-285 -------PAALRALSQKLSKGFNEA-VNG--FTDEG-----------WS--ML-ES----
XP_017643921.1/1-285 -------PAALRALSQKLSKGFNEA-VNG--FTDEG-----------WS--ML-ES----
XP_012436119.1/1-285 -------PAALRALSQKLSKGFNEA-VNG--FTDEG-----------WS--ML-ES----
XP_016681632.1/1-285 -------PAALRALSQKLSKGFNEA-VNG--FTDEG-----------WS--ML-ES----
XP_016689618.1/1-285 -------PAALRALSQKLNKGFNEA-VNG--FADEG-----------WS--ML-ES----
XP_016689617.1/1-285 -------PAALRALSQKLNKGFNEA-VNG--FADEG-----------WS--ML-ES----
XP_012445947.1/1-285 -------PAALRALSQKLNKGFNEA-VNG--FADEG-----------WS--ML-ES----
XP_012445944.1/1-285 -------PAALRALSQKLNKGFNEA-VNG--FADEG-----------WS--ML-ES----
XP_012445945.1/1-285 -------PAALRALSQKLNKGFNEA-VNG--FADEG-----------WS--ML-ES----
XP_017614652.1/1-285 -------PAALRALSQKLSKGFNEA-VNG--FADEG-----------WS--ML-ES----
XP_016732732.1/1-285 -------PAALRALSQKLSKGFNEA-VNG--FADEG-----------WS--ML-ES----
XP_016732731.1/1-285 -------PAALRALSQKLSKGFNEA-VNG--FADEG-----------WS--ML-ES----
XP_022754596.1/1-285 -------PAALRAVSQKLSKGFNEA-VNG--FTDEG-----------WS--ML-ES----
XP_022750159.1/1-285 -------PAALRALSQKLSRGFNEA-VNG--FTDEG-----------WS--ML-ES----
XP_022729737.1/1-285 -------PAALRALSQKLSKGFNEA-VNG--FTDDG-----------WS--ML-ES----
XP_022729738.1/1-285 -------PAALRALSQKLSKGFNEA-VNG--FTDDG-----------WS--ML-ES----
XP_021276132.1/1-285 -------PAALRSLSQKLSKGFNEA-VNG--FTDEG-----------WS--ML-ES----
XP_017983822.1/1-285 -------PAALRALSQKLSKGFNEA-VNG--FTDEG-----------WS--ML-ES----
XP_002283717.3/1-285 -------PAALRALGQRLTKGFNEA-VNG--FTDEG-----------WS--MM-ES----
XP_024446213.1/1-284 -------PAALRALSQRLSKGFNEA-VNG--FADEG-----------WS--LL-ES----
XP_024446215.1/1-284 -------PAALRALSQRLSKGFNEA-VNG--FADEG-----------WS--LL-ES----
XP_011006428.1/1-284 -------PAALRALSQRLSKGFNEA-VNG--FADEG-----------WP--LL-ES----
XP_024459734.1/1-284 -------PAALRALSQRLSKGFNEA-VNG--FADEG-----------WS--ML-ES----
XP_011019595.1/1-284 -------PAALRALSQRLSKGFNEA-VNG--FADEG-----------WS--LL-ES----
XP_015573174.1/1-284 -------PAALRALSQRLSKGFNEA-ING--FTDEG-----------WS--ML-ES----
XP_012077376.1/1-284 -------PAALRALSQRLSKGFNEA-VNG--FNDEG-----------WT--ML-ES----
XP_021608403.1/1-284 -------PAALRALSQRLSKGFNEA-VNG--FTDEG-----------WS--ML-ES----
XP_021608402.1/1-284 -------PAALRALSQRLSKGFNEA-VNG--FTDEG-----------WS--ML-ES----
XP_021597909.1/1-284 -------PAALRALSQRLSKGFNEA-VNG--FTDDG-----------WS--ML-ES----
XP_021642839.1/1-284 -------PAALRALSQRLSKGFNEA-VNG--FTDDG-----------WS--ML-ES----
XP_021689146.1/1-284 -------PAALRALSQRLSKGFNEA-VNG--FTDEG-----------WS--ML-ES----
XP_018805752.1/1-285 -------PAALRALSQRLSKGFNEA-VNG--FTVEG-----------WS--ML-ES----
XP_023918167.1/1-285 -------PAALRALSQRLSKGFNEA-VNG--FTDEG-----------WS--ML-ES----
XP_018845499.1/1-285 -------PAALRALSQRLSKGFNEA-VNG--FTDEG-----------WS--ML-ES----
XP_018845500.1/1-285 -------PAALRALSQRLSKGFNEA-VNG--FTDEG-----------WS--ML-ES----
NP_001280750.1/1-285 -------PAALRALSQRLSKGFNEA-VNG--FTDEG-----------WS--VL-ES----
NP_001280796.1/1-285 -------PAALRALSQRLSKGFNEA-VNG--FTDEG-----------WS--VL-ES----
XP_009367440.1/1-285 -------PAALRALSQRLSKGFNEA-VNG--FTDEG-----------WS--VL-ES----
XP_017182108.1/1-285 -------PAALRALSQRLSKGFNEA-VNG--FTDEG-----------WS--VL-ES----
XP_004291167.1/1-285 -------PAALRALSQRLSKGFNEA-VNG--FTDEG-----------WS--IL-ES----
XP_024162655.1/1-285 -------PAALRALSQRLSKGFNEA-VNG--FTDEG-----------WS--IL-ES----
XP_021819449.1/1-284 -------PAALRGLSQRLSKGFNEA-VNG--FTDEG-----------WS--IL-ES----
XP_007225281.1/1-285 -------PAALRALSQRLSKGFNEA-VNG--FTDEG-----------WS--IL-ES----
XP_008220092.1/1-285 -------PAALRALSQRLSKGFNEA-VNG--FTDEG-----------WS--IL-ES----
XP_006450706.1/1-285 -------PAALRALSQRLSRGFNEA-LNG--FTDEG-----------WS--ML-ES----
XP_006476026.1/1-285 -------PAALRALSQRLSRGFNEA-LNG--FTDEG-----------WS--ML-ES----
XP_024930942.1/1-285 -------PAALRALSQRLSKGFNEA-VNG--FTDEG-----------WS--ML-ES----
XP_010102406.2/1-285 -------PAALRALSQKLSKGFNEA-VNG--FTDEG-----------WT--ML-ES----
XP_024540686.1/1-258 -------PSVLRSLSRRMARGFNDA-VNG--FPDDG-----------WC--TM-GG----
XP_024399220.1/1-283 -------PAVIRTLSQRLTKGFNDA-VNG--FGDDG-----------WV--PM-ES----
XP_024399219.1/1-283 -------PAVIRTLSQRLTKGFNDA-VNG--FGDDG-----------WV--PM-ES----
XP_024399215.1/1-283 -------PAVIRTLSQRLTKGFNDA-VNG--FGDDG-----------WV--PM-ES----
XP_024390746.1/1-284 -------PAVLRTLCQRLTKGFNNA-VNG--FPDDG-----------WE--AT-IS----
XP_024390752.1/1-284 -------PAVLRTLCQRLTKGFNNA-VNG--FPDDG-----------WE--AT-IS----
XP_024373354.1/1-283 -------PAVLRILGQRLTKGFNDA-VNG--FGDDG-----------WE--TT-VT----
XP_024373357.1/1-283 -------PAVLRILGQRLTKGFNDA-VNG--FGDDG-----------WE--TT-VT----
XP_024370817.1/1-284 -------PVVLRTLSQRLAKGFKNA-VNG--FGDDG-----------WV--ST-VS----
XP_024370815.1/1-284 -------PVVLRTLSQRLAKGFKNA-VNG--FGDDG-----------WV--ST-VS----
XP_024370814.1/1-284 -------PVVLRTLSQRLAKGFKNA-VNG--FGDDG-----------WV--ST-VS----
XP_024386785.1/1-285 -------PAVLRTLGQRLAKGFKNA-VNG--FADDG-----------WV--ST-VS----
XP_024386782.1/1-285 -------PAVLRTLGQRLAKGFKNA-VNG--FADDG-----------WV--ST-VS----
XP_024386791.1/1-285 -------PAVLRTLGQRLAKGFKNA-VNG--FADDG-----------WV--ST-VS----
XP_002961227.1/1-277 --------AAWRGVSRRIARGFNEA-VNS--FADDG-----------WM--IT-DA----
XP_002969458.1/1-277 --------AAWRGVSRRIARGFNEA-VNS--FADDG-----------WM--IT-DA----
XP_024533017.1/1-265 -------PAALRAFSHRIARGFNDA-VNA--FAEDG-----------WT--SD-GG----
XP_011076983.1/1-261 -------PAFLRSFSQRLSRGFNDA-ITG--FTEDG-----------WT--LL-DA----
XP_011076980.1/1-264 -------PAFLRSFSQRLSRGFNDA-ITG--FTEDG-----------WT--LL-DA----
XP_011076981.1/1-263 -------PAFLRSFSQRLSRGFNDA-ITG--FTEDG-----------WT--LL-DA----
XP_011084330.1/1-264 -------PAFLRSVSRRLCRGFNDA-VNC--FSEDG-----------WT--LM-NV----
XP_011084333.1/1-264 -------PAFLRSVSRRLCRGFNDA-VNC--FSEDG-----------WT--LM-NV----
XP_011084331.1/1-264 -------PAFLRSVSRRLCRGFNDA-VNC--FSEDG-----------WT--LM-NV----
XP_012834933.1/1-260 -------PAFLRSFSQRLSRGFNDA-VNC--FSEDG-----------WT--LM-NG----
XP_012834932.1/1-260 -------PAFLRSFSQRLSRGFNDA-VNC--FSEDG-----------WT--LM-NG----
XP_012834931.1/1-260 -------PAFLRSFSQRLSRGFNDA-VNC--FSEDG-----------WT--LM-NG----
XP_012834934.1/1-260 -------PAFLRSFSQRLSRGFNDA-VNC--FSEDG-----------WT--LM-NG----
XP_002274194.1/1-284 -------PAVLRAFRQKLSRGFNDA-ING--FNDDG-----------WS--LM-QI----
XP_019079956.1/1-284 -------PAVLRAFRQKLSRGFNDA-ING--FNDDG-----------WS--LM-QI----
XP_023759583.1/1-283 -------PAALRALGQRMSRGFNEA-VNG--FADEG-----------WS--VT-ES----
XP_020087984.1/1-275 -------PAALRALSQRLGRGFSEA-ING--FADEG-----------WS--AA-SN----
XP_015693848.1/1-289 -------PAALRALSQKLTRGFNEA-LNG--LADDG-----------WS--VI-ES----
XP_015624690.1/1-290 -------PAALRALSQKLTRGFNEA-LNG--LADDG-----------WS--VI-ES----
XP_008655743.1/1-292 -------PASLRALSQKLTRGFNEA-LNG--LADDG-----------WS--VV-GS----
XP_020183693.1/1-281 -------PAALRALSQKLTRGLNET-LGG--LADDG-----------WS--VI-ES----
XP_020183687.1/1-281 -------PAALRALSQKLTRGLNET-LGG--LADDG-----------WS--VI-ES----
XP_003565084.1/1-284 -------PAALRALSQKLTRGFNEA-LCG--LTDDG-----------WS--AI-ES----
XP_002454995.1/1-286 -------PAALRALSQKLTRGFNEA-LNG--LADDG-----------WS--VV-ES----
XP_012701181.1/1-286 -------PAALRALSQKLTRGFNEA-LNG--LADDG-----------WS--VI-ES----
XP_012701180.1/1-286 -------PAALRALSQKLTRGFNEA-LNG--LADDG-----------WS--VI-ES----
XP_012701179.1/1-286 -------PAALRALSQKLTRGFNEA-LNG--LADDG-----------WS--VI-ES----
XP_025816436.1/1-287 -------PAALRALSQKLTRGFNEA-LNG--LADDG-----------WS--VI-ES----
XP_018726848.1/1-227 ---------SMLKLAERMVISFWAG-VSA--S---TAHT--------WT-TLSGT-----
XP_018726849.1/1-227 ---------SMLKLAERMVISFWAG-VSA--S---TAHT--------WT-TLSGT-----
XP_019193052.1/1-216 ---------SILKLAERMVISFCSG-VSS--ST--VAHE--------WT-VLSGT-----
XP_019193050.1/1-216 ---------SILKLAERMVISFCSG-VSS--ST--VAHE--------WT-VLSGT-----
XP_021834047.1/1-194 ---------SMLKLAERMVISFYAG-VSA--S---TTHT--------WT-TLSRT-----
XP_010522330.1/1-229 ---------SMLKLAERMVISFCSG-VSA--S---TAHT--------WT-TLSGT-----
XP_009802524.1/1-219 -----------LKLAERMVISYCSG-VSA--T---AAHQ--------WT-TLSG-SG---
XP_009802526.1/1-219 -----------LKLAERMVISYCSG-VSA--T---AAHQ--------WT-TLSG-SG---
XP_009802525.1/1-219 -----------LKLAERMVISYCSG-VSA--T---AAHQ--------WT-TLSG-SG---
XP_009802527.1/1-219 -----------LKLAERMVISYCSG-VSA--T---AAHQ--------WT-TLSG-SG---
XP_019250148.1/1-219 -----------LKLAERMVISYCSG-VSA--T---AAHQ--------WT-TLSG-SG---
XP_019250149.1/1-219 -----------LKLAERMVISYCSG-VSA--T---AAHQ--------WT-TLSG-SG---
XP_016449923.1/1-219 -----------LKLVERMVISYCSG-VSA--T---EAHQ--------WT-TLSG-SG---
XP_009629760.1/1-219 -----------LKLAERMVISYCSG-VSA--T---AAHQ--------WT-TLSG-SG---
XP_009629762.1/1-219 -----------LKLAERMVISYCSG-VSA--T---AAHQ--------WT-TLSG-SG---
XP_010234287.2/1-230 ---------SMLRLAERMTASFCGG-VAA--S---ATHQ--------WT-TLSGS-----
XP_021806177.1/1-204 ---------SILKLAKRMVISFYAG-VSA--S---TTHT--------WT-TLSRT-----
XP_016580642.1/1-229 ---------SMMKLAERMVNSFGSG-VSA--S---VGNH--------WT-TLATGSG---
XP_008667763.1/1-266 ---------SMLKLAERMVTSFCGG-VTA--S---AAHQ--------WT-TLSG-SG---
XP_006352040.1/1-227 ---------SMMKLAERMVNSFSSG-VSA--T---VGNH--------WT-TLSQGNG---
XP_009611638.1/1-223 ---------SMLKLAERMVNSFCSG-MSA--T---AGNQ--------WS-TLS-GSG---
XP_009611623.1/1-223 ---------SMLKLAERMVNSFCSG-MSA--T---AGNQ--------WS-TLS-GSG---
XP_016510654.1/1-223 ---------SMLKLAERMVNSFCSG-MSA--T---AGNQ--------WS-TLS-GSG---
XP_009766038.1/1-223 ---------SMLKLAERMVNSFCSG-VSA--T---AGNQ--------WT-TLS-GSG---
XP_020210648.1/1-236 PAGDLCGRKSMLNLAERMMLSFCNG-VGA--S---TAHV--------WT-PLTI------
XP_007143210.1/1-219 ---------SMMRLSERMMMSFCTA-VGA--S---TANT--------WT-LLTV------
XP_014491869.1/1-226 ----------MMRLAERMMLSFCTA-VGA--S---TANA--------WT-LLTE------
XP_022633652.1/1-226 ----------MMRLAERMMLSFCTA-VGA--S---TANA--------WT-LLTE------
XP_017412341.1/1-227 ---------SMMRLAERMMLSFCTA-VGA--S---TANA--------WT-LITD------
XP_022011155.1/1-226 ---------SMLKLAQRMVLSYCCG-VGA--S---SAHT--------WT-TLSGT-----
XP_003555330.2/1-228 ---------SMMKLAERMVLSFCTG-VGA--S---TANA--------WT-PLPS------
XP_014628518.1/1-229 ---------SMMKLAERMVLSFCTG-VGA--S---TANA--------WT-PLPS------
XP_017647009.1/1-225 ---------SILKLAERMVNSFCTG-VGA--S---TAHA--------WT-TLT-------
XP_016711277.1/1-225 ---------SILKLAERMVNSFCTG-VGA--S---TAHA--------WT-TLT-------
XP_012457027.1/1-225 ---------SILKLAERMVNSFCTG-VGA--S---TAHA--------WT-TLT-------
XP_016723454.1/1-225 ---------SILKLAERMVNSFCTG-VGA--S---TAHA--------WT-TLT-------
XP_020978973.1/1-214 ---------SMLKLAERMVMSFCTG-VGA--S---TAHA--------WT-TLSS------
XP_016202355.1/1-214 ---------SMLKLAERMVMSFCTG-VGA--S---TAHA--------WT-TLSS------
XP_015964876.1/1-214 ---------SMLKLAERMVMSFCTG-VGA--S---TAHA--------WT-TLSS------
XP_025699645.1/1-214 ---------SMLKLAERMVMSFCTG-VGA--S---TAHA--------WT-TLSS------
XP_008441375.1/1-228 -----------MKLAERMVRSFCSG-VGA--A---TAHN--------WT-TLSTI-----
XP_004154226.2/1-228 -----------MKLAERMVRSFCSG-VGA--A---TAHN--------WT-TLSTI-----
XP_023519981.1/1-226 -----------MKLAERMVRSFCSG-VGA--A---SAHN--------WT-TLSTI-----
XP_023001231.1/1-226 -----------MKLAERMVRSFCSG-VGA--A---SAHN--------WT-TLSTI-----
XP_022933178.1/1-226 -----------MKLAERMVRSFCSG-VGA--A---SAHN--------WT-TLSTI-----
XP_004511340.1/1-228 ---------SMLNLAERMVTSFSTS-IGD--S---TIHA--------WT-SLPSN-----
XP_002979015.2/1-232 ---------SMLKLAQRMTNNFCAG-VSA--S---TVHT--------WT-TLSGS-----
XP_002988402.1/1-232 ---------SMLKLAQRMTNNFCAG-VSA--S---TVHT--------WT-TLSGS-----
XP_024390340.1/1-238 ---------SMLKLAQRMTSNFCAG-VSA--S---TAHT--------WT-TLSGS-----
XP_024368074.1/1-235 ---------SMLKLAQRMTNNFCAG-VSA--S---TVHT--------WT-TLSGS-----
XP_024401280.1/1-235 ---------SMLKLAQRMTNNFCAG-VSA--S---TVHT--------WT-TLSGS-----
XP_024394840.1/1-235 ---------SMLKLAQRMTNNFCAG-VSA--S---TVHT--------WT-TLSGS-----
XP_024394838.1/1-235 ---------SMLKLAQRMTNNFCAG-VSA--S---TVHT--------WT-TLSGS-----
XP_017256650.1/1-227 ---------SMLKLAERMVISFSAG-VSG--S---TAHK--------WT-TLSGN-----
XP_019186546.1/1-227 ---------SILKLAERMVMSFCAG-VGA--S---TSQT--------WT-TLSGS-----
XP_024988375.1/1-226 ---------SMLKLAERMVLGFCTG-VGA--S---TAHT--------WT-TLSGS-----
XP_017231769.1/1-226 ---------SMLKLAERMVLSFCSG-VGS--S---TEHT--------WT-TLSGS-----
XP_009108529.1/1-235 ---------SMLKLAERMVMSFCSG-VGA--S---TAHA--------WS-TLAAT-----
XP_018469534.1/1-236 ---------SMLKLAERMVVSFCSG-VGA--S---TANA--------WT-TLTTT-----
XP_018467466.1/1-236 ---------SMLKLAERMVMSFCRG-VGA--S---TANA--------WT-TLTTT-----
XP_022543253.1/1-234 ---------SMLKLAERMVMSFFSG-VGA--S---TAHA--------WT-TLATT-----
XP_009135181.1/1-234 ---------SMLKLAERMVMSFFSG-VGA--S---TAHA--------WT-TLATT-----
XP_013642975.1/1-234 ---------SMLKLAERMVMSFFSG-VGA--S---TAHA--------WT-TLATT-----
XP_009135189.1/1-234 ---------SMLKLAERMVMSFFSG-VGA--S---TAHA--------WT-TLATT-----
XP_018515487.1/1-234 ---------SMLKLAERMVMSFFSG-VGA--S---TAHA--------WT-TLATT-----
XP_013623861.1/1-234 ---------SMLKLAERMARSFCSG-VGA--S---TAHA--------WT-TLATT-----
XP_013623905.1/1-234 ---------SMLKLAERMARSFCSG-VGA--S---TAHA--------WT-TLATT-----
XP_013623953.1/1-236 ---------SMLKLAERMARSFCSG-VGA--S---TAHA--------WT-TLATT-----
XP_013623587.1/1-236 ---------SMLKLAERMARSFCSG-VGA--S---TAHA--------WT-TLATT-----
XP_013623995.1/1-236 ---------SMLKLAERMARSFCSG-VGA--S---TAHA--------WT-TLATT-----
XP_013623820.1/1-236 ---------SMLKLAERMARSFCSG-VGA--S---TAHA--------WT-TLATT-----
XP_022926381.1/1-227 ---------SMLKLAERMVTSFCAG-VGA--S---SIHA--------WT-ALPAA-----
XP_023517485.1/1-227 ---------SMLKLAERMVTSFCAG-VGA--S---SIHA--------WT-ALPAA-----
XP_023002930.1/1-227 ---------SMLKLAERMVTSFCAG-VGA--S---SIHA--------WT-ALPAA-----
XP_004134971.1/1-229 ---------SMLKLAERMVTSFCAG-VGA--S---SVHA--------WT-ALPAA-----
XP_008439968.1/1-229 ---------SMLKLAERMVTSFCAG-VGA--S---SVHA--------WT-ALPAA-----
XP_022950176.1/1-227 ---------SMLKLAERMVTSFCAG-VGA--S---SVHA--------WT-ALPIA-----
XP_022979090.1/1-227 ---------SMLKLAERMVTSFCAG-VGA--S---SVHA--------WT-ALPIA-----
XP_023543388.1/1-249 ---------SMLKLAERMVTSFCAG-VGA--S---SVHA--------WT-ALPIA-----
XP_023543387.1/1-227 ---------SMLKLAERMVTSFCAG-VGA--S---SVHA--------WT-ALPIA-----
XP_009134323.1/1-228 ---------SMLKLAESM------X-IGA--S---TAHA--------WT-TMSST-----
XP_019176002.1/1-227 ---------SMLKLAERMVMSFCAG-VGA--S---TAHT--------WT-TL--SGS---
XP_015070655.1/1-221 ---------SMLKLAERMVMSFCAG-VGA--S---TAHT--------WT-TL--SGS---
XP_015070652.1/1-221 ---------SMLKLAERMVMSFCAG-VGA--S---TAHT--------WT-TL--SGS---
XP_025885589.1/1-221 ---------SMLKLAERMVMSFCAG-VGA--S---TAHT--------WT-TL--SGS---
XP_019422236.1/1-227 ---------SMLNLAERMVMSFCTG-VGA--S---TSHA--------WT-TL--SAT---
XP_007157166.1/1-226 ---------SMLKLAERMVISYCTG-VGA--S---TAHA--------WT-TL--SAT---
XP_003538765.1/1-227 ---------SMMKLAERMVMSYCTG-VGA--S---TAHA--------WT-TL--SAT---
XP_003517589.1/1-227 ---------SMMKLAERMVMSYCTG-VGA--S---TAHA--------WT-TL--SAT---
XP_006573890.1/1-227 ---------SMMKLAERMVMSYCTG-VGA--S---TAHA--------WT-TL--SAT---
XP_011000687.1/1-226 ---------SILKLAERMVISFCTG-VGA--S---TAHT--------WT-TL--SAT---
XP_024436817.1/1-226 ---------SILKLAERMVMSFCTG-VGA--S---TAHT--------WT-TL--SAT---
XP_024436820.1/1-226 ---------SILKLAERMVMSFCTG-VGA--S---TAHT--------WT-TL--SAT---
XP_002518520.2/1-226 ---------SMLKLAERMVMSFCSG-VGA--S---TAHA--------WT-TL--SAT---
XP_015574304.1/1-226 ---------SMLKLAERMVMSFCSG-VGA--S---TAHA--------WT-TL--SAT---
XP_021642058.1/1-227 ---------SMLKLAERMVMSFCSG-VGA--S---TAHA--------WT-TL--SPT---
XP_012091395.1/1-226 ---------SMLKLAERMVMSFCSG-VGA--S---TAHA--------WT-TL--SAT---
XP_021635824.1/1-226 ---------SMLKLAERMVMSFCSG-VGA--S---TAHA--------WT-TL--SPT---
XP_021596887.1/1-226 ---------SMLKLAERMVMSFCSG-VGA--S---TAHA--------WT-TL--SPS---
XP_010057969.1/1-228 ---------SMLKLAERMVLSFCSG-VGA--S---TAHA--------WT-TL--SAA---
XP_006413738.1/1-245 ---------SMLKLAERMVMSFCSG-VGA--S---TAHV--------WT-TL--TTT---
XP_013630219.1/1-235 ---------SMLKLAERMVMSFCSG-VGA--S---TAHA--------WT-TL--AAT---
XP_013656136.1/1-235 ---------SMLKLAERMVMSFCSG-VGA--S---TAHA--------WT-TL--AAT---
XP_013685757.1/1-235 ---------SMLKLAERMVMSFCSG-VGA--S---TAHA--------WT-TL--AAT---
XP_013696499.1/1-240 ---------SMLKLAERMVMSFCSG-VGA--S---NAHA--------WT-TL--ATT---
XP_009137213.1/1-240 ---------SMLKLAERMVMSFCSG-VGA--S---NAHA--------WT-TL--ATT---
XP_013598423.1/1-241 ---------SMLKLAERMVMSFCSG-VGA--S---NAHA--------WT-TL--ATA---
XP_013717184.1/1-241 ---------SMLKLAERMVMSFCSG-VGA--S---NAHA--------WT-TL--ATA---
XP_018480642.1/1-241 ---------SMLKLAERMVMSFCSG-VGA--S---NAHA--------WT-TL--ATT---
XP_018477464.1/1-241 ---------SMLKLAERMVMSFCSG-VGA--S---NAHA--------WT-TL--ATT---
NP_193906.2/1-248    ---------SMLKLAERMVMSFCTG-VGA--S---TAHA--------WT-TL--STT---
XP_020873301.1/1-248 ---------SMLKLAERMVMSFCSG-VGA--S---TAHA--------WT-TL--STT---
XP_006285476.1/1-249 ---------SMLKLAERMVMSFCSG-VGA--S---TAHA--------WT-TL--STT---
XP_010434132.1/1-252 ---------SMLKLAERMVMSFCSG-VGA--S---TAHA--------WT-TL--STT---
XP_010439429.1/1-252 ---------SMLKLAERMVMSFCSG-VGA--S---TAHA--------WT-TL--STT---
XP_010449009.1/1-254 ---------SMLKLAERMVMSFCSG-VGA--S---TAHA--------WT-TL--STT---
XP_010432053.1/1-234 ---------SMLKLAERMVMSFCSG-VGA--S---TAHA--------WT-TM--STT---
XP_020876375.1/1-234 ---------SMLKLAERMVMSFCSG-VGA--S---TAHA--------WT-TM--STT---
XP_023636874.1/1-234 ---------SMLKLAERMVMSFCSG-VGA--S---TAHA--------WT-TM--STT---
XP_010422328.1/1-234 ---------SMLKLAERMVMSFCSG-VGA--S---TAHA--------WT-TM--STT---
XP_010455798.1/1-234 ---------SMLKLAERMVMSFCSG-VGA--S---TAHA--------WT-TM--STT---
NP_567274.1/1-235    ---------SMLKLAERMVMSFCSG-VGA--S---TAHA--------WT-TM--STT---
XP_024009884.1/1-236 ---------SMLKLAERMVMSFCSG-VGA--S---TAHA--------WT-TM--SST---
XP_018452346.1/1-236 ---------SMLKLAERMVMSFCSG-VGA--S---TAHA--------WT-TM--SST---
XP_013611160.1/1-236 ---------SMLKLAERMVMSFCTG-VGA--S---TAHA--------WT-TM--SST---
XP_009114536.1/1-235 ---------SMLKLAERMVMSFCSG-VGA--S---TAHA--------WT-TM--SST---
XP_013662000.1/1-235 ---------SMLKLAERMVMSFCSG-VGA--S---TAHA--------WT-TM--SST---
XP_018472664.1/1-233 ---------SMLKLAERMVMSFCSG-VGA--S---TAHA--------WT-TM--SST---
XP_022555047.1/1-234 ---------SMLKLAERMVMSFCSG-VGA--S---TAHA--------WT-TM--SST---
XP_013735469.1/1-234 ---------SMLKLAERMVMSFCSG-VGA--S---TAHA--------WT-TM--SST---
XP_013629800.1/1-234 ---------SMLKLAERMVMSFCSG-VGA--S---TAHA--------WT-TM--SST---
XP_013735464.1/1-234 ---------SMLKLAERMVMSFCSG-VGA--S---TAHA--------WT-TM--SST---
XP_022571893.1/1-234 ---------SMLKLAERMVMSFCSG-VGA--S---TAHA--------WT-TM--SST---
XP_010538642.1/1-236 ---------SMLKLAERMVMSFCSG-VGA--S---TAHA--------WT-TL--SAT---
XP_019057303.1/1-229 ---------SMLKLAERMAMSFCSG-VGA--S---TAHA--------WT-TL--SAT---
XP_016545426.1/1-226 ---------SMLKLAERMVMSFCAG-VGA--S---TAHT--------WT-TL--SGS---
XP_009793614.1/1-227 -----------LKLAERMVMSFCAG-VGA--S---TAHT--------WT-TL--SGS---
XP_016508926.1/1-227 -----------LKLAERMVMSFCAG-VGA--S---TAHT--------WT-TL--SGS---
XP_016560096.1/1-235 ----PAGRKSMLKLAERMVRSFCAG-VGT--S---TTHT--------WT-TL--SGS---
XP_021993721.1/1-227 ---------SMLKLSERMVLSFCSG-VGA--S---TTHT--------WT-TL--SGS---
XP_022036928.1/1-226 ---------SMMKLAERMVLSFCSG-VGA--S---TAHT--------WT-AL--SGS---
XP_023742870.1/1-226 ---------SMLKLAERMVLSFCSG-VGA--S---TAHT--------WT-TL--SGS---
XP_006352605.1/1-223 ---------SMLKLAERMVMSFCAG-VGA--S---TAHT--------WT-TL--SGS---
XP_010327237.1/1-223 ---------SMLKLAERMVMSFCAG-VGA--S---TAHT--------WT-TL--SGS---
XP_015055984.1/1-223 ---------SMLKLAERMVMSFCAG-VGA--S---TAHT--------WT-TL--SGS---
XP_009609606.1/1-229 ---------SMLKLAERMVMSFCAG-VGA--S---TAHT--------WT-TL--SGS---
XP_009609608.1/1-229 ---------SMLKLAERMVMSFCAG-VGA--S---TAHT--------WT-TL--SGS---
XP_018628742.1/1-229 ---------SMLKLAERMVMSFCAG-VGA--S---TAHT--------WT-TL--SGS---
XP_019237831.1/1-229 ---------SMLKLAERMVMSFCAG-VGA--S---TAHT--------WT-TL--SGS---
XP_018849836.1/1-227 ---------SMLKLAERMVMSFCTG-VGA--S---TAHA--------WT-TL--SAT---
XP_007214541.1/1-230 ---------SMLKLAQRMVMSFCTG-VGA--S---TAHA--------WT-TL--SAT---
XP_021821565.1/1-230 ---------SMLKLAQRMVMSFCTG-VGA--S---TAHA--------WT-TL--SAT---
XP_008224792.1/1-230 ---------SMLKLAQRMVMSFCTG-VGA--S---TAHA--------WT-TL--SAT---
XP_024198966.1/1-227 ---------SMLKLAQRMVLSFCTG-VGA--S---TAHA--------WT-TL--SAT---
XP_004293268.1/1-227 ---------SMLKLAQRMVLSFCTG-VGA--S---TAHA--------WT-TL--SAT---
XP_009364098.1/1-227 ---------SMLKLAQRMVISFCTG-VGA--S---TAHA--------WT-TL--SAT---
XP_008371114.1/1-227 ---------SMLKLAQRMVMSFCTG-VGA--S---TAHA--------WT-TL--SAT---
XP_008384034.1/1-227 ---------SMLKLAQRLVMSFCTG-VGA--S---TAHA--------WT-TL--SAT---
XP_009340946.1/1-227 ---------SMLKLAQRMVMSFCTG-VGA--S---TAHA--------WT-TL--SAT---
XP_009357415.1/1-227 ---------SMLKLAQRMVMSFCTG-VGA--S---TAHA--------WT-TL--SAT---
XP_011037708.1/1-226 ---------SMLKLAERMVMSFCTG-VGA--S---TAHA--------WT-TL--SAT---
XP_024454819.1/1-226 ---------SMLKLAERMVMSFCTG-VGA--S---TAHA--------WT-TL--SAT---
XP_015884145.1/1-226 ---------SMLKLAERMVRSFCTG-VGA--S---TAHA--------WT-TL--SAT---
XP_024024693.1/1-228 ---------SMLKLAERMVMSFCVG-VGA--S---TAHA--------WT-TL--SAT---
XP_024024692.1/1-228 ---------SMLKLAERMVMSFCVG-VGA--S---TAHA--------WT-TL--SAT---
XP_018820351.1/1-226 ---------SMLKLAERMVMSFCTG-VGA--S---TAHA--------WT-TL--SAT---
XP_023904570.1/1-226 ---------SMLKLAERMAVSFCTG-VGA--S---TAHA--------WT-TL--SAA---
XP_023904569.1/1-226 ---------SMLKLAERMAVSFCTG-VGA--S---TAHA--------WT-TL--SAA---
XP_006449076.1/1-226 ---------SMLKLAERMVTSFCTG-VGA--S---TAHA--------WT-TL--SAT---
XP_006468235.1/1-226 ---------SMLKLAERMVTSFCTG-VGA--S---TAHA--------WT-TL--SAT---
XP_022721034.1/1-226 ---------SMLKLAERMVTSFCTG-VGA--S---TAHA--------WT-TL--SAT---
XP_022721035.1/1-226 ---------SMLKLAERMVTSFCTG-VGA--S---TAHA--------WT-TL--SAT---
XP_022724746.1/1-226 ---------SMLKLAERMVTSFCTG-VGA--S---TAHA--------WT-TL--STT---
XP_007026002.1/1-226 ---------SMLKLAERMVTSFCTG-VGA--S---TAHA--------WT-TL--SAT---
XP_021294392.1/1-226 ---------SMLKLAERMVTSFCTG-VGA--S---TAHA--------WT-TL--SAT---
XP_022777405.1/1-226 ---------SMLKLAERMVTSFCTG-VGA--S---TAHA--------WT-TL--SAT---
XP_017615807.1/1-226 ---------SMLKLAERMVTSFCTG-VGA--S---TAHA--------WT-TL--SAT---
XP_016753649.1/1-226 ---------SMLKLAERMVTSFCTG-VGA--S---TAHA--------WT-SL--SAT---
XP_016683607.1/1-226 ---------SMLKLAERMVTSFCTG-VGA--S---TAHA--------WT-TL--SAT---
XP_012451363.1/1-226 ---------SMLKLAERMVTSFCTG-VGA--S---TAHA--------WT-TL--SAT---
XP_002266688.1/1-226 ---------SMLKLAERMVMSFCAG-VGA--S---TTHT--------WT-TL--SGS---
XP_021853797.1/1-224 ---------SMLKLAERMVMSFCAG-VGA--S---TAHT--------WT-TL--SGS---
XP_021719656.1/1-227 ---------SMLKLAERMVMSFCAG-VGA--S---TAHT--------WT-TL--SGS---
XP_021766927.1/1-227 ---------SMLKLAERMVMSFCAG-VGA--S---TAHT--------WT-TL--SGS---
XP_010665524.1/1-227 ---------SMLKLAERMVMSFCAG-VGA--S---TAHT--------WT-TL--SGS---
XP_009605864.1/1-234 ----PAGRKSMLKLAERMVMSFCAG-VGA--S---TTHT--------WT-TL--SGS---
XP_019223778.1/1-233 ----PAGRKSMLKLAERMVMSFCAG-VGA--S---TTHT--------WT-TL--SGS---
XP_016440385.1/1-233 ----PAGRKSMLKLAERMVMSFCAG-VGA--S---TTHT--------WT-TL--SGS---
XP_009790281.1/1-233 ----PAGRKSMLKLAERMVMSFCAG-VGA--S---TTHT--------WT-TL--SGS---
XP_016570434.1/1-222 ---------SMLKLAERMVMSFCAG-VGA--S---TAHT--------WT-TL--SGS---
XP_016486479.1/1-229 ---------SMLKLAERMVMSFCAG-VGA--S---TAHT--------WT-TL--SGS---
XP_009597904.1/1-229 ---------SMLKLAERMVMSFCAG-VGA--S---TAHT--------WT-TL--SGS---
XP_009799795.1/1-229 ---------SMLKLAERMVMSFCAG-VGA--S---TAHT--------WT-TL--SGS---
XP_019249652.1/1-229 ---------SMLKLAERMVMSFCAG-VGA--S---TAHT--------WT-TL--SGS---
XP_006343296.1/1-221 ---------SMLKLAERLVMSFCAG-VGA--S---TAHT--------WT-TL--SGS---
XP_020217012.1/1-226 ---------SMLKLAERMVISYCTG-VGA--S---TAHA--------WT-TL--SAT---
XP_017406677.1/1-226 ---------SMLKLAERMVISYCTG-VGA--S---TAHA--------WT-TL--SAT---
XP_014519998.1/1-226 ---------SMLKLAERMVISYCTG-VGA--S---TAHA--------WT-TL--SAT---
XP_014519997.1/1-226 ---------SMLKLAERMVISYCTG-VGA--S---TAHA--------WT-TL--SAT---
XP_012841999.1/1-239 ---------SMLKLAERMLMSFCSG-VGA--S---TAHT--------WT-TL--SGS---
XP_024984347.1/1-227 ---------SMLKLAERMVLSFCSG-VGA--S---TTHT--------WT-TL--SGS---
XP_023751202.1/1-227 ---------SMLKLAERMVLSFCSG-VGA--S---TTHT--------WT-TL--SGS---
XP_023751208.1/1-227 ---------SMLKLAERMVLSFCSG-VGA--S---TTHT--------WT-TL--SGS---
XP_022041933.1/1-227 ---------SMLKLSERMVLSFCSG-VGA--S---TTHT--------WT-TL--SGS---
XP_024988632.1/1-226 ---------SMLKLAERMVLSFCSG-VGA--S---TAHT--------WT-TL--SGS---
XP_022018253.1/1-226 ---------SMLKLAERMVLSFCSG-VGA--S---TAHT--------WT-TL--SGS---
XP_023771293.1/1-226 ---------SMLKLAERMVLSFCSG-VGA--S---TAHT--------WT-TL--SGS---
XP_019170324.1/1-226 ---------SMLKLAERMVMSFCAG-VGA--S---TAHT--------WT-TL--SGS---
XP_017230122.1/1-226 ---------SMLKLAERMVLSFCTG-VGA--S---TAHT--------WT-TL--SGS---
XP_011096101.1/1-226 ---------SMLKLAERMVMSFCTG-VGA--S---TAHT--------WT-TL--SGS---
XP_012848972.1/1-227 ---------SMLKLAERMVMSFCTG-VGA--S---TAHT--------WT-TL--SGS---
XP_022876975.1/1-227 ---------SMLKLAERMVMSFCSG-VGA--S---TAHT--------WT-TL--SGS---
XP_022863158.1/1-227 ---------SMLKLAERMVMSFCTG-VGA--S---TAHT--------WT-TM--SGS---
XP_022877991.1/1-227 ---------SMLKLAERMVMSFCTG-VGA--S---TAHT--------WT-TM--SGS---
XP_019223745.1/1-223 ---------SMLKLAERMVNSFCSG-VSA--T---AGNQ--------WT-TLS-GSG---
XP_021800220.1/1-221 ---------SLXKLAERMVISFCAG-VSA--S---TTHT--------WT-TL--SGT---
XP_021684589.1/1-235 ---------SMLKLAERMAISFFGG-VSA--S---TAHT--------WT-TL--SGT---
XP_021684519.1/1-235 ---------SMLKLAERMAISFFGG-VSA--S---TAHT--------WT-TL--SGT---
XP_021684722.1/1-235 ---------SMLKLAERMAISFFGG-VSA--S---TAHT--------WT-TL--SGT---
XP_021633498.1/1-230 ---------SMLKLAERMAKSFCSG-VSA--S---TAHT--------WT-TL--SGT---
XP_009420270.1/1-216 ----------MLRLAERMVTSFCGG-VSA--S---TAHQ--------WT-TV--SGN---
XP_003573403.1/1-232 ---------SMLKLAERMVASFCGG-VTA--S---VAHQ--------WT-TL--SGS---
XP_006659894.1/1-229 ---------SMLKLAERMVASFCGG-VTA--S---VAHQ--------WT-TL--SGS---
XP_015650022.1/1-263 ---------SMLKLAERMVASFCGG-VTA--S---VAHQ--------WT-TL--SGS---
XP_015650023.1/1-230 ---------SMLKLAERMVASFCGG-VTA--S---VAHQ--------WT-TL--SGS---
XP_020407343.1/1-231 ---------SMLKLAERMVASFCGG-VTA--S---AAHQ--------WT-TL--SGS---
NP_001105127.2/1-231 ---------SMLKLAERMVASFCGG-VTA--S---AAHQ--------WT-TL--SGS---
XP_008661805.1/1-239 ---------SMLKLAERMVASFCGG-VTA--S---AAHQ--------WT-TL--SGS---
NP_001145750.1/1-239 ---------SMLKLAERMVASFCGG-VTA--S---AAHQ--------WT-TL--SGS---
XP_025821536.1/1-230 ---------SMLKLAERMVASFCGG-VTA--S---AAHQ--------WT-TL--SGS---
XP_002443965.1/1-229 ---------SMLKLAERMVASFCGG-VTA--S---AAHQ--------WT-TL--SGS---
XP_004972697.1/1-229 ---------SMLKLAERMVASFCGG-VTA--S---AAHQ--------WT-TL--SGS---
XP_008667765.1/1-230 ---------SMLKLAERMVTSFCGG-VTA--S---AAHQ--------WT-TL--SGS---
NP_001347891.1/1-231 ---------SMLKLAERMVTSFCGG-VTA--S---AAHQ--------WT-TL--SGS---
XP_015636340.1/1-229 ---------SMLKLAERMVVSFCGG-VTA--S---VAHQ--------WT-TL--SGS---
XP_015636338.1/1-229 ---------SMLKLAERMVVSFCGG-VTA--S---VAHQ--------WT-TL--SGS---
XP_006652859.1/1-228 ---------SMLKLAERMVMSFCGG-VTA--S---VAHQ--------WT-TL--SGS---
XP_003580598.1/1-233 ---------SMLKLAERMVASFCGG-VTA--S---VAHQ--------WT-RL--SGS---
XP_020199198.1/1-227 ---------SMLKLAERMVASFCGG-VTA--S---VAHQ--------WT-TL--SGS---
XP_020400772.1/1-230 ---------SMLKLAERMVMSFCGG-VTA--S---AAHQ--------WT-TL--SGS---
NP_001334663.1/1-230 ---------SMLKLAERMVMSFCGG-VTA--S---AAHQ--------WT-TL--SGS---
XP_008662197.1/1-270 ---------SMLKLAERMVMSFCGG-VTA--S---AAHQ--------WT-TL--SGS---
XP_008662196.1/1-270 ---------SMLKLAERMVMSFCGG-VTA--S---AAHQ--------WT-TL--SGS---
XP_025823505.1/1-227 ---------SMLKLAERMVMSFCGG-VTA--S---AAHQ--------WT-TL--SGS---
XP_004976894.1/1-227 ---------SMLKLAERMVMSFCGG-VTA--S---AAHQ--------WT-TL--SGS---
XP_002448563.2/1-227 ---------SMLKLAERMVMSFCGG-VTA--S---AAHQ--------WT-TL--SGS---
XP_019414154.1/1-227 ---------SMLKLAERMVMSFCGG-VSA--S---TTHT--------WT-TLQ-TGS---
XP_006348085.1/1-234 ----PAGRKSMLKLAERMVMSFCAG-VGA--S---TTHI--------WT-TL--SGS---
XP_004232734.1/1-234 ----PAGRKSMLKLAERMVMSFCAG-VGA--S---TTHI--------WT-TL--SGS---
XP_015066898.1/1-234 ----PAGRKSMLKLAERMVMSFCAG-VGA--S---TTHI--------WT-TL--SGS---
XP_003605900.1/1-232 ---------SMLKLAERMVISFCGG-VSA--S---TAHT--------WT-TL--SGT---
XP_022988693.1/1-229 ---------SMMKLAERMVMSFCGG-VSA--S---TAYT--------WT-TL--SGT---
XP_022930609.1/1-229 ---------SMLKLAERMVMSFCGG-VSA--S---TAHT--------WT-TL--SGT---
XP_023530197.1/1-229 ---------SMLKLAERMVMSFCGG-VSA--S---TAHT--------WT-TL--SGT---
XP_016188415.1/1-237 ---------SMLKLAERMVMSFCGG-VSA--S---TAHT--------WT-TL--NGT---
XP_011628991.1/1-236 ---------SMLKLAERMVVSFCAG-VSA--S---TAHT--------WT-TL--SGS---
XP_006826512.2/1-236 ---------SMLKLAERMVVSFCAG-VSA--S---TAHT--------WT-TL--SGS---
XP_020177398.1/1-220 ---------SMLKLAERMTASFCGG-VTA--S---TTHQ--------WT-TL--SGS---
XP_015695486.1/1-219 ---------SMLKLAERMVASFCGG-VTA--S---TTHQ--------WT-TL--SGS---
XP_015648539.1/1-217 ---------SMLKLAERMVASFCGG-VTA--S---TTHQ--------WT-TL--SGS---
XP_020401599.1/1-218 ---------SMMRLAERMVASFCGG-VTA--S---TTHQ--------WT-TL--SGS---
XP_020401598.1/1-218 ---------SMMRLAERMVASFCGG-VTA--S---TTHQ--------WT-TL--SGS---
XP_020401597.1/1-218 ---------SMMRLAERMVASFCGG-VTA--S---TTHQ--------WT-TL--SGS---
XP_021319954.1/1-239 ---------SMLRLAERMVTSFCGG-VTA--S---TTHQ--------WT-KL--SGS---
XP_002445014.2/1-239 ---------SMLRLAERMVTSFCGG-VTA--S---TTHQ--------WT-KL--SGS---
XP_025819913.1/1-218 ---------SMLRLAERMVASFCGG-VTA--S---TTHQ--------WT-TL--SGS---
XP_004972909.1/1-218 ---------SMLRLAERMVASFCGG-VTA--S---TTHQ--------WT-TL--SGS---
XP_020100432.1/1-229 ---------SMLKLAERMVISFCGG-VTA--S---TTHQ--------WT-TL--SGS---
XP_009385059.1/1-228 ---------SMLKLAERMVISFCGG-VGG--S---ASHQ--------WT-TL--SGS---
XP_018678797.1/1-226 ---------SMLKLAERMVMSFCGG-VSA--S---TTHQ--------WT-TL--SGS---
XP_009412012.1/1-233 ---------SMLKLAERMVISFCGG-VSA--S---TTHQ--------WT-TL--SGS---
XP_018685149.1/1-236 ---------SMLKLAERMVISFCGG-VSA--S---TTHQ--------WT-TL--SGS---
XP_020591278.1/1-230 ---------SMLKLAERMVLSFCGG-VSA--S---TTHQ--------WT-TL--SGS---
XP_020702932.1/1-229 ---------SMLKLAERMVLSFCGG-VSA--S---TTHQ--------WT-TL--SGS---
XP_020702933.1/1-230 ---------SMLKLAERMVLSFCGG-VSA--S---TTHQ--------WT-TL--SGS---
XP_020702931.1/1-230 ---------SMLKLAERMVLSFCGG-VSA--S---TTHQ--------WT-TL--SGS---
XP_020692781.1/1-229 ---------SMLKLAERMVISFCGG-VSA--S---TTHQ--------WT-TL--SGS---
XP_020692780.1/1-229 ---------SMLKLAERMVISFCGG-VSA--S---TTHQ--------WT-TL--SGS---
XP_020591591.1/1-229 ---------SMLKLAERMVISFCGG-VSA--S---TTHQ--------WT-TL--SGS---
XP_020591590.1/1-229 ---------SMLKLAERMVISFCGG-VSA--S---TTHQ--------WT-TL--SGS---
XP_020109359.1/1-227 ---------SMLKLAERMVISFCGA-VTA--S---TTHQ--------WT-TL--SGS---
XP_020270114.1/1-229 ---------SMLKLAERMVISFCGG-VSA--S---TTHQ--------WT-TL--SGS---
XP_010908855.1/1-227 ---------SMLKLAERMVISFCGG-VTA--S---TTHQ--------WT-TL--SGS---
XP_008808987.1/1-227 ---------SMLKLAERMVISFCGG-VTA--S---TTHQ--------WT-TL--SGS---
XP_008784552.1/1-223 ---------SMLKLAERMVMSFCGG-VTA--S---TTHQ--------WT-TL--SGS---
XP_010931820.1/1-223 ---------SMLKLAERMVMSFCGG-VTA--S---TTHQ--------WT-TL--SGS---
XP_003540308.2/1-226 ---------SMLKLAERMVISFCAG-VSA--S---TAHT--------WT-TLS--GT---
XP_006592818.1/1-226 ---------SMLKLAERMVISFCAG-VSA--S---TAHT--------WT-TLS--GT---
XP_006592816.1/1-226 ---------SMLKLAERMVISFCAG-VSA--S---TAHT--------WT-TLS--GT---
XP_003543387.1/1-226 ---------SMLKLAERMVISFCAG-VSA--S---TAHT--------WT-TLS--GT---
XP_020223651.1/1-226 ---------SMLKLAERMVISFCAG-VSA--S---TAHT--------WT-TLS--GT---
XP_020223648.1/1-226 ---------SMLKLAERMVISFCAG-VSA--S---TAHT--------WT-TLS--GT---
XP_007149968.1/1-226 ---------SMLKLAERMVISFCAG-VSA--S---TAHT--------WT-TLS--GT---
XP_017425582.1/1-226 ---------SMLKLAERMVISFCAG-VSA--S---TAHT--------WT-TLS--GT---
XP_014501634.1/1-226 ---------SMLKLAERMVISFCAG-VSA--S---TAHT--------WT-TLS--GT---
XP_022776632.1/1-226 ---------SMLKLAERMAISFCAG-VSA--S---TAHT--------WT-TLS--GT---
XP_022776631.1/1-226 ---------SMLKLAERMAISFCAG-VSA--S---TAHT--------WT-TLS--GT---
XP_022776629.1/1-226 ---------SMLKLAERMAISFCAG-VSA--S---TAHT--------WT-TLS--GT---
XP_022733641.1/1-226 ---------SMLKLAERMVISFCAG-VSA--S---TAHT--------WT-TLS--GT---
XP_022733640.1/1-226 ---------SMLKLAERMVISFCAG-VSA--S---TAHT--------WT-TLS--GT---
XP_022733638.1/1-226 ---------SMLKLAERMVISFCAG-VSA--S---TAHT--------WT-TLS--GT---
XP_007045612.1/1-226 ---------SMLKLAERMVISFCAG-VSA--S---TAHT--------WT-TLS--GT---
XP_007045611.1/1-226 ---------SMLKLAERMVISFCAG-VSA--S---TAHT--------WT-TLS--GT---
XP_017971478.1/1-226 ---------SMLKLAERMVISFCAG-VSA--S---TAHT--------WT-TLS--GT---
XP_021292769.1/1-226 ---------SMLKLAERMVISFCAG-VSA--S---TAHT--------WT-TLS--GT---
XP_021292767.1/1-226 ---------SMLKLAERMVISFCAG-VSA--S---TAHT--------WT-TLS--GT---
XP_021292765.1/1-226 ---------SMLKLAERMVISFCAG-VSA--S---TAHT--------WT-TLS--GT---
XP_010657309.1/1-226 ---------SMLKLAERMVISFCAG-VSA--S---TAHT--------WT-TLS--GS---
XP_010657313.1/1-226 ---------SMLKLAERMVISFCAG-VSA--S---TAHT--------WT-TLS--GS---
XP_010657310.1/1-226 ---------SMLKLAERMVISFCAG-VSA--S---TAHT--------WT-TLS--GS---
XP_010657311.1/1-226 ---------SMLKLAERMVISFCAG-VSA--S---TAHT--------WT-TLS--GS---
XP_012086347.1/1-228 ---------SMLKLAERMVISFCAG-VSA--S---TAHT--------WT-TLS--GT---
XP_012086344.1/1-228 ---------SMLKLAERMVISFCAG-VSA--S---TAHT--------WT-TLS--GT---
XP_024029967.1/1-226 ---------SMLKLAERMVISFCAG-VSA--S---TTHT--------WT-TLS--GT---
XP_010109517.1/1-226 ---------SMLKLAERMVISFCAG-VSA--S---TTHT--------WT-TLS--GT---
XP_006469697.1/1-226 ---------SMLKLAERMVISFCAG-VSA--S---TAHT--------WT-TLS--GT---
XP_024046029.1/1-226 ---------SMLKLAERMVISFCAG-VSA--S---TAHT--------WT-TLS--GT---
XP_020234199.1/1-226 ---------SMMKLAERMVISFCAG-VSA--S---TAHT--------WT-TLS--GT---
XP_020234201.1/1-226 ---------SMMKLAERMVISFCAG-VSA--S---TAHT--------WT-TLS--GT---
XP_021630856.1/1-226 ---------SMLKLAERMVISFFAG-VSA--S---TAHT--------WT-TLS--GT---
XP_021630853.1/1-226 ---------SMLKLAERMVISFFAG-VSA--S---TAHT--------WT-TLS--GT---
XP_021907367.1/1-221 ---------SMLKLAERMVNSFCAG-VSA--S---TAHT--------WT-TLS--GT---
XP_010275996.1/1-221 ---------SMLKLAERMVVSFCAG-VSA--S---TAHT--------WT-TLS--GS---
XP_020870784.1/1-223 ---------SMLKLAERMVISFCAG-VSA--S---TAHT--------WT-TLS--GT---
NP_172015.1/1-223    ---------SMLKLAERMVISFCAG-VSA--S---TAHT--------WT-TLS--GT---
XP_006418050.1/1-223 ---------SMLKLAERMVISFCAG-VSA--S---TAHT--------WT-TLS--GT---
XP_018447753.1/1-223 ---------SMLKLSERMVISFCAG-VSA--S---TAHT--------WT-TLS--GT---
XP_010457605.1/1-218 ---------SMLKLAERMVISFCAG-VSA--S---TAHT--------WT-TLS--GT---
XP_023632765.1/1-218 ---------SMLKLAERMVISFCAG-VSA--S---TAHT--------WT-TLS--GT---
XP_010484829.1/1-218 ---------SMLKLAERMVISFCAG-VSA--S---TAHT--------WT-TLS--GT---
XP_010475208.1/1-218 ---------SMLKLAERMVISFCAG-VSA--S---TAHT--------WT-TLS--GT---
XP_018487097.1/1-220 ---------SMLKLAERMVISFCAG-VSA--S---TAHT--------WT-TLS--GT---
XP_013664795.1/1-220 ---------SMLKLAERMVISFCAG-VSA--S---TAHT--------WT-TLS--GT---
XP_013716253.1/1-220 ---------SMLKLAERMVISFCAG-VSA--S---TAHT--------WT-TLS--GT---
XP_009118607.1/1-220 ---------SMLKLAERMVISFCAG-VSA--S---TAHT--------WT-TLS--GT---
XP_013605632.1/1-220 ---------SMLKLAERMVISFCAG-VSA--S---TAHT--------WT-TLS--GT---
XP_010475204.1/1-220 ---------SMLKLAERMVISFCAG-VSA--S---TAHT--------WT-TLS--GT---
XP_010457604.1/1-220 ---------SMLKLAERMVISFCAG-VSA--S---TAHT--------WT-TLS--GT---
XP_023632762.1/1-220 ---------SMLKLAERMVISFCAG-VSA--S---TAHT--------WT-TLS--GT---
XP_010484823.1/1-220 ---------SMLKLAERMVISFCAG-VSA--S---TAHT--------WT-TLS--GT---
XP_022558401.1/1-220 ---------SMLKLAERMVISFCAG-VSA--S---TAHT--------WT-TLS--GT---
XP_013586641.1/1-220 ---------SMLKLAERMVISFCAG-VSA--S---TAHT--------WT-TLS--GT---
XP_022558399.1/1-220 ---------SMLKLAERMVISFCAG-VSA--S---TAHT--------WT-TLS--GT---
XP_022558400.1/1-220 ---------SMLKLAERMVISFCAG-VSA--S---TAHT--------WT-TLS--GT---
XP_013586642.1/1-220 ---------SMLKLAERMVISFCAG-VSA--S---TAHT--------WT-TLS--GT---
XP_013696832.1/1-220 ---------SMLKLAERMVISFCAG-VSA--S---TAHT--------WT-TLS--GT---
XP_022548878.1/1-220 ---------SMLKLAERMVISFCAG-VSA--S---TAHT--------WT-TLS--GT---
XP_013666552.1/1-220 ---------SMLKLAERMVISFCAG-VSA--S---TAHT--------WT-TLS--GT---
XP_009119177.1/1-220 ---------SMLKLAERMVISFCAG-VSA--S---TAHT--------WT-TLS--GT---
XP_020964430.1/1-225 ---------SMLKLAERMVISFCAG-VSA--S---TAHT--------WT-TLS--GT---
XP_015936069.1/1-225 ---------SMLKLAERMVISFCAG-VSA--S---TAHT--------WT-TLS--GT---
XP_020964429.1/1-225 ---------SMLKLAERMVISFCAG-VSA--S---TAHT--------WT-TLS--GT---
XP_015936068.1/1-225 ---------SMLKLAERMVISFCAG-VSA--S---TAHT--------WT-TLS--GT---
XP_015577880.1/1-228 ---------SMLKLAERMVISFCAG-VSA--S---TAHT--------WT-TLS--GT---
XP_002524172.2/1-228 ---------SMLKLAERMVISFCAG-VSA--S---TAHT--------WT-TLS--GT---
XP_007132557.1/1-221 ---------SMMKLAERMVISFCAG-VSA--S---TAHT--------WT-TLS--GT---
XP_017433694.1/1-221 ---------SMMKLAERMVISFCAG-VSA--S---TAHT--------WT-TLS--GT---
XP_014494419.1/1-221 ---------SMMKLAERMVISFCAG-VSA--S---TAHT--------WT-TLS--GT---
XP_022634310.1/1-221 ---------SMMKLAERMVISFCAG-VSA--S---TAHT--------WT-TLS--GT---
XP_010267735.1/1-225 ---------SMLKLAERMVISFCSG-VSA--S---TSHT--------WT-TLS--GS---
XP_010267734.1/1-225 ---------SMLKLAERMVISFCSG-VSA--S---TSHT--------WT-TLS--GS---
XP_024185199.1/1-224 ---------SMLKLAERMVISFCAG-VSA--S---TTHT--------WT-TLS--GT---
XP_024185197.1/1-224 ---------SMLKLAERMVISFCAG-VSA--S---TTHT--------WT-TLS--GT---
XP_009342246.1/1-222 ---------SMLKLAERMVISFCSG-VSA--S---TTHT--------WT-TLS--GT---
XP_009342244.1/1-222 ---------SMLKLAERMVISFCSG-VSA--S---TTHT--------WT-TLS--GT---
XP_009342240.1/1-222 ---------SMLKLAERMVISFCSG-VSA--S---TTHT--------WT-TLS--GT---
XP_020416503.1/1-221 ---------SMLKLAERMVISFCAG-VSA--S---TTHT--------WT-TLS--GT---
XP_021822854.1/1-221 ---------SMLKLAERMVISFCAG-VSA--S---TTHT--------WT-TLS--GT---
XP_007217584.1/1-221 ---------SMLKLAERMVISFCAG-VSA--S---TTHT--------WT-TLS--GT---
XP_021822852.1/1-221 ---------SMLKLAERMVISFCAG-VSA--S---TTHT--------WT-TLS--GT---
XP_018730806.1/1-228 ---------SMLKLAERMVISFCAG-VSA--S---TAHT--------WT-TLS--GT---
XP_010051064.1/1-228 ---------SMLKLAERMVISFCAG-VSA--S---TAHT--------WT-TLS--GT---
XP_015881130.2/1-229 ---------SMLKLAERMVISFCAG-VSA--S---TTHT--------WT-TLS--GT---
XP_023534533.1/1-219 ---------SMLKLAERMVISFCAG-VSA--S---TTHT--------WT-TLS--GT---
XP_022947095.1/1-219 ---------SMLKLAERMVISFCAG-VSA--S---TTHT--------WT-TLS--GT---
XP_022947096.1/1-219 ---------SMLKLAERMVISFCAG-VSA--S---TTHT--------WT-TLS--GT---
XP_022970950.1/1-219 ---------SMLKLAERMVISFCAG-VSA--S---TTHT--------WT-TLS--GT---
XP_022970951.1/1-219 ---------SMLKLAERMVISFCAG-VSA--S---TTHT--------WT-TLS--GT---
XP_022140692.1/1-217 ---------SMLKLAERMVISFCAG-VSA--S---TTHT--------WT-TLS--GT---
XP_022140691.1/1-217 ---------SMLKLAERMVISFCAG-VSA--S---TTHT--------WT-TLS--GT---
XP_008455853.1/1-220 ---------SMLKLAERMVISFCAG-VSA--S---TTHT--------WT-TLS--GT---
XP_011650012.1/1-220 ---------SMLKLAERMVISFCAG-VSA--S---TTHT--------WT-TLS--GT---
XP_008455850.1/1-220 ---------SMLKLAERMVISFCAG-VSA--S---TTHT--------WT-TLS--GT---
XP_011650011.1/1-220 ---------SMLKLAERMVISFCAG-VSA--S---TTHT--------WT-TLS--GT---
XP_023513403.1/1-220 ---------SMLKLAERMVISFCAG-VSA--S---TTHT--------WT-TLS--GT---
XP_022944761.1/1-220 ---------SMLKLAERMVISFCAG-VSA--S---TTHT--------WT-TLS--GT---
XP_023513401.1/1-220 ---------SMLKLAERMVISFCAG-VSA--S---TTHT--------WT-TLS--GT---
XP_022944759.1/1-220 ---------SMLKLAERMVISFCAG-VSA--S---TTHT--------WT-TLS--GT---
XP_022986270.1/1-220 ---------SMLKLAERMVISFCAG-VSA--S---TTHT--------WT-TLS--GT---
XP_022986268.1/1-220 ---------SMLKLAERMVISFCAG-VSA--S---TTHT--------WT-TLS--GT---
XP_022136006.1/1-224 ---------SMLKLAERMVMSFCGG-VSA--S---TTHT--------WT-TLS--GT---
XP_008455423.1/1-229 ---------SMLKLAERMVMSFCGG-VSA--S---TTHT--------WT-TLS--GT---
XP_011658704.1/1-229 ---------SMLKLAERMVMSFCGG-VSA--S---TTHT--------WT-TLS--GT---
XP_004144488.1/1-229 ---------SMLKLAERMVMSFCGG-VSA--S---TTHT--------WT-TLS--GT---
XP_022968990.1/1-229 ---------SMLKLAERMVMSFCGG-VSA--S---TTHT--------WT-TLS--GT---
XP_023554403.1/1-229 ---------SMLKLAERMVMSFCGG-VSA--S---TTHT--------WT-TLS--GT---
XP_022952948.1/1-229 ---------SMLKLAERMVMSFCGG-VSA--S---TTHT--------WT-TLS--GT---
XP_019447751.1/1-228 ---------SMLKLAERMVISFCAG-VSA--S---TAHT--------WT-TLSGSGT---
XP_019447749.1/1-228 ---------SMLKLAERMVISFCAG-VSA--S---TAHT--------WT-TLSGSGT---
XP_004515074.1/1-228 ---------SMMKLAERMVMSFCGG-VSA--S---TAHT--------WT-TL--AGT---
XP_012463792.1/1-235 ---------SMLKLAERMVMSFCAG-VSA--S---TAHT--------WT-TL--SGT---
XP_012463790.1/1-235 ---------SMLKLAERMVMSFCAG-VSA--S---TAHT--------WT-TL--SGT---
XP_016678783.1/1-235 ---------SMLKLAERMVMSFCAG-VSA--S---TAHT--------WT-TL--SGT---
XP_016678782.1/1-235 ---------SMLKLAERMVMSFCAG-VSA--S---TAHT--------WT-TL--SGT---
XP_017631648.1/1-235 ---------SMLKLAERMVMSFCAG-VSA--S---TAHT--------WT-TL--SGT---
XP_016732503.1/1-235 ---------SMLKLAERMVMSFCAG-VSA--S---TAHT--------WT-TL--SGT---
XP_017631647.1/1-235 ---------SMLKLAERMVMSFCAG-VSA--S---TAHT--------WT-TL--SGT---
XP_012438430.1/1-229 ---------SMLKLAERMVISFCAG-VGA--S---TAHT--------WT-TL--SGT---
XP_012438427.1/1-229 ---------SMLKLAERMVISFCAG-VGA--S---TAHT--------WT-TL--SGT---
XP_012438429.1/1-229 ---------SMLKLAERMVISFCAG-VGA--S---TAHT--------WT-TL--SGT---
XP_017616162.1/1-229 ---------SMLKLAERMVISFCAG-VGA--S---TAHT--------WT-TL--SGT---
XP_017616160.1/1-229 ---------SMLKLAERMVISFCAG-VGA--S---TAHT--------WT-TL--SGT---
XP_017616161.1/1-229 ---------SMLKLAERMVISFCAG-VGA--S---TAHT--------WT-TL--SGT---
XP_016726347.1/1-229 ---------SMLKLAERMVISFCAG-VGA--S---TAHT--------WT-TL--SGT---
XP_016726344.1/1-229 ---------SMLKLAERMVISFCAG-VGA--S---TAHT--------WT-TL--SGT---
XP_016726345.1/1-229 ---------SMLKLAERMVISFCAG-VGA--S---TAHT--------WT-TL--SGT---
XP_019448933.1/1-227 ---------SMLKLAERMVISFCAG-VSA--S---TTHT--------WT-TLQ-TGT---
XP_004487400.1/1-228 ---------SMLKLAERMCISFCAG-VSA--S---TAHT--------WT-TL--SGT---
XP_003596999.2/1-227 ---------SMLKLAERMCISFCAG-VSA--S---TAHT--------WT-TL--SGT---
XP_024631890.1/1-227 ---------SMLKLAERMCISFCAG-VSA--S---TAHT--------WT-TL--SGT---
XP_024631891.1/1-227 ---------SMLKLAERMCISFCAG-VSA--S---TAHT--------WT-TL--SGT---
XP_004287935.1/1-224 ---------SMLKLAERMVISFCAG-VSA--S---TTHT--------WT-TL--SGT---
XP_018808969.1/1-228 ---------SMLKLAERMVISFCAG-VSA--S---TTHT--------WT-TL--SGT---
XP_018808967.1/1-228 ---------SMLKLAERMVISFCAG-VSA--S---TTHT--------WT-TL--SGT---
XP_018817598.1/1-227 ---------SMLKLAERMVISFCAG-VSA--S---TAHT--------WT-TL--SGT---
XP_018817597.1/1-227 ---------SMLKLAERMVISFCAG-VSA--S---TAHT--------WT-TL--SGT---
XP_018817594.1/1-227 ---------SMLKLAERMVISFCAG-VSA--S---TAHT--------WT-TL--SGT---
XP_018817599.1/1-227 ---------SMLKLAERMVISFCAG-VSA--S---TAHT--------WT-TL--SGT---
XP_023915056.1/1-227 ---------SMLKLAERMVISFCAG-VSA--S---TTHT--------WT-TL--SGT---
XP_023915054.1/1-227 ---------SMLKLAERMVISFCAG-VSA--S---TTHT--------WT-TL--SGT---
XP_023915053.1/1-227 ---------SMLKLAERMVISFCAG-VSA--S---TTHT--------WT-TL--SGT---
XP_021685023.1/1-228 ---------SMLKLAERMAISFFGG-VSA--S---TAHT--------WT-TL--SGT---
XP_021684657.1/1-228 ---------SMLKLAERMAISFFGG-VSA--S---TAHT--------WT-TL--SGT---
XP_021685089.1/1-228 ---------SMLKLAERMAISFFGG-VSA--S---TAHT--------WT-TL--SGT---
XP_015383009.1/1-229 ---------SMLKLAERMVISFCAG-VSA--S---TAHT--------WT-TL--SGT---
XP_015383007.1/1-229 ---------SMLKLAERMVISFCAG-VSA--S---TAHT--------WT-TL--SGT---
XP_020539441.1/1-235 ---------SMLKLAERMVISFCAG-VSA--S---TAHT--------WT-TL--SGT---
XP_020539439.1/1-235 ---------SMLKLAERMVISFCAG-VSA--S---TAHT--------WT-TL--SGT---
XP_015577881.1/1-233 ---------SMLKLAERMVISFCAG-VSA--S---TAHT--------WT-TL--SGT---
XP_015577879.1/1-233 ---------SMLKLAERMVISFCAG-VSA--S---TAHT--------WT-TL--SGT---
XP_015577877.1/1-233 ---------SMLKLAERMVISFCAG-VSA--S---TAHT--------WT-TL--SGT---
XP_021630854.1/1-231 ---------SMLKLAERMVISFFAG-VSA--S---TAHT--------WT-TL--SGT---
XP_021630849.1/1-231 ---------SMLKLAERMVISFFAG-VSA--S---TAHT--------WT-TL--SGT---
XP_021652390.1/1-229 ---------SMLKLAERMVISFFAG-VSA--S---TAHT--------WT-TL--SGT---
XP_021652388.1/1-229 ---------SMLKLAERMVISFFAG-VSA--S---TAHT--------WT-TL--SGT---
XP_021652389.1/1-234 ---------SMLKLAERMVISFFAG-VSA--S---TAHT--------WT-TL--SGT---
XP_021652386.1/1-234 ---------SMLKLAERMVISFFAG-VSA--S---TAHT--------WT-TL--SGT---
XP_006592380.1/1-228 ---------SMMKLAERMVISFCAG-VSA--S---TAHT--------WT-TL--SGT---
XP_003539872.2/1-228 ---------SMMKLAERMVISFCAG-VSA--S---TAHT--------WT-TL--SGT---
XP_025984806.1/1-230 ---------SMMKLAERMVISFCAG-VSA--S---TAHT--------WT-TL--SGT---
XP_014632311.1/1-230 ---------SMMKLAERMVISFCAG-VSA--S---TAHT--------WT-TL--SGT---
XP_024450362.1/1-227 ---------SMMKLAERMVISFCAG-VSA--S---TAHT--------WT-TL--SGT---
XP_002302880.2/1-227 ---------SMMKLAERMVISFCAG-VSA--S---TAHT--------WT-TL--SGT---
XP_024450361.1/1-227 ---------SMMKLAERMVISFCAG-VSA--S---TAHT--------WT-TL--SGT---
XP_024450360.1/1-227 ---------SMMKLAERMVISFCAG-VSA--S---TAHT--------WT-TL--SGT---
XP_011011493.1/1-227 ---------SMMKLAERMVISFCAG-VSA--S---TAHT--------WT-TL--SGT---
XP_011011491.1/1-227 ---------SMMKLAERMVISFCAG-VSA--S---TAHT--------WT-TL--SGT---
XP_011011492.1/1-227 ---------SMMKLAERMVISFCAG-VSA--S---TAHT--------WT-TL--SGT---
XP_011011489.1/1-227 ---------SMMKLAERMVISFCAG-VSA--S---TAHT--------WT-TL--SGT---
XP_011011488.1/1-227 ---------SMMKLAERMVISFCAG-VSA--S---TAHT--------WT-TL--SGT---
XP_011005116.1/1-225 ---------SMMKLAERMVISFCAG-VSA--S---TAHT--------WT-TL--SGT---
XP_011005113.1/1-225 ---------SMMKLAERMVISFCAG-VSA--S---TAHT--------WT-TL--SGT---
XP_011005112.1/1-225 ---------SMMKLAERMVISFCAG-VSA--S---TAHT--------WT-TL--SGT---
XP_024440391.1/1-225 ---------SMMKLAERMVISFCAG-VSA--S---TAHT--------WT-TL--SGT---
XP_002320458.2/1-225 ---------SMMKLAERMVISFCAG-VSA--S---TAHT--------WT-TL--SGT---
XP_024440389.1/1-225 ---------SMMKLAERMVISFCAG-VSA--S---TAHT--------WT-TL--SGT---
XP_019453767.1/1-227 ---------SMLKLAERMVISFCAG-VSA--S---TAHT--------WT-TLQ-SGT---
XP_012478857.1/1-227 ---------SMLKLAERMVISFCGG-VSA--S---TAHT--------WT-TL--SGT---
XP_012478855.1/1-227 ---------SMLKLAERMVISFCGG-VSA--S---TAHT--------WT-TL--SGT---
XP_012478856.1/1-227 ---------SMLKLAERMVISFCGG-VSA--S---TAHT--------WT-TL--SGT---
XP_012478851.1/1-227 ---------SMLKLAERMVISFCGG-VSA--S---TAHT--------WT-TL--SGT---
XP_016724999.1/1-227 ---------SMLKLAERMVISFCGG-VSA--S---TAHT--------WT-TL--SGT---
XP_017638204.1/1-227 ---------SMLKLAERMVISFCGG-VSA--S---TAHT--------WT-TL--SGT---
XP_017638203.1/1-227 ---------SMLKLAERMVISFCGG-VSA--S---TAHT--------WT-TL--SGT---
XP_016667113.1/1-227 ---------SMLKLAERMVISFCGG-VSA--S---TAHT--------WT-TL--SGT---
XP_016667112.1/1-227 ---------SMLKLAERMVISFCGG-VSA--S---TAHT--------WT-TL--SGT---
XP_015881131.2/1-227 ---------SMLKLAERMVISFCAG-VSA--S---TTHT--------WT-TL--SGT---
XP_008348131.1/1-222 ---------SMLKLAERMVISFCAG-VSA--S---TTHT--------WT-TL--SGT---
XP_008348133.1/1-220 ---------SMLKLAERMVISFCAG-VSA--S---TTHT--------WT-TL--SGT---
XP_018502305.1/1-220 ---------SMLKLAERMVISFCAG-VSA--S---TTHT--------WT-TL--SGT---
XP_009342243.1/1-220 ---------SMLKLAERMVISFCSG-VSA--S---TTHT--------WT-TL--SGT---
XP_017179321.1/1-222 ---------SMLKLAERMVISFCSG-VSA--S---TTHT--------WT-TL--SGT---
XP_017179320.1/1-222 ---------SMLKLAERMVISFCSG-VSA--S---TTHT--------WT-TL--SGT---
XP_008341986.1/1-222 ---------SMLKLAERMVISFCSG-VSA--S---TTHT--------WT-TL--SGT---
XP_017179319.1/1-220 ---------SMLKLAERMVISFCSG-VSA--S---TTHT--------WT-TL--SGT---
XP_024185198.1/1-222 ---------SMLKLAERMVISFCAG-VSA--S---TTHT--------WT-TL--SGT---
XP_011463209.1/1-222 ---------SMLKLAERMVISFCAG-VSA--S---TTHT--------WT-TL--SGT---
XP_020416501.1/1-219 ---------SMLKLAERMVISFCAG-VSA--S---TTHT--------WT-TL--SGT---
XP_021822853.1/1-219 ---------SMLKLAERMVISFCAG-VSA--S---TTHT--------WT-TL--SGT---
XP_008230079.1/1-219 ---------SMLKLAERMVISFCAG-VSA--S---TTHT--------WT-TL--PGT---
XP_020870786.1/1-221 ---------SMLKLAERMVISFCAG-VSA--S---TAHT--------WT-TL--SGT---
NP_001117231.1/1-221 ---------SMLKLAERMVISFCAG-VSA--S---TAHT--------WT-TL--SGT---
XP_018817596.1/1-225 ---------SMLKLAERMVISFCAG-VSA--S---TAHT--------WT-TL--SGT---
XP_019079350.1/1-224 ---------SMLKLAERMVISFCAG-VSA--S---TAHT--------WT-TL--SGS---
XP_022733639.1/1-224 ---------SMLKLAERMVISFCAG-VSA--S---TAHT--------WT-TL--SGT---
XP_021292768.1/1-224 ---------SMLKLAERMVISFCAG-VSA--S---TAHT--------WT-TL--SGT---
XP_021292766.1/1-224 ---------SMLKLAERMVISFCAG-VSA--S---TAHT--------WT-TL--SGT---
XP_017971480.1/1-224 ---------SMLKLAERMVISFCAG-VSA--S---TAHT--------WT-TL--SGT---
XP_018721484.1/1-222 ---------SMMKLAERMVISFCAG-VSA--S---TAQT--------WT-TL--SGT---
XP_018721483.1/1-222 ---------SMMKLAERMVISFCAG-VSA--S---TAQT--------WT-TL--SGT---
XP_018721482.1/1-222 ---------SMMKLAERMVISFCAG-VSA--S---TAQT--------WT-TL--SGT---
XP_025687365.1/1-237 ---------SMLKLAERMVISFCGG-VSA--S---TAHT--------WT-TL--NGT---
XP_006851770.2/1-243 ----PAGRRSMLKLAQRMTNNFCAG-VSA--S---TVHK--------WN-KISMG-----
XP_003623826.1/1-230 ---------SMLKLAHRMTNNFCAG-VCA--S---TVHK--------WN-KLNAG-----
XP_004492678.1/1-230 ---------SMLKLAQRMTNNFCAG-VCA--S---TVHQ--------WN-KLNVG-----
XP_004492677.1/1-230 ---------SMLKLAQRMTNNFCAG-VCA--S---TVHQ--------WN-KLNVG-----
XP_012847897.1/1-236 ---------SMLKLAQRMTNNFCAG-VCA--S---SVHK--------WN-KLRTE-----
XP_012843673.1/1-229 ---------SMLKLAQRMTNNFCAG-VCA--S---SVHK--------WN-KLRTE-----
XP_021279608.1/1-239 ---------SMLKLAQRMTDNFCAG-VCA--S---TLHK--------WN-KLNAG-----
XP_025641241.1/1-241 ---------SMLKLAQRMTNNFCAG-VCA--S---TVHK--------WN-KLNNTG----
XP_025641246.1/1-241 ---------SMLKLAQRMTNNFCAG-VCA--S---TVHK--------WN-KLNNTG----
XP_020991942.1/1-241 ---------SMLKLAQRMTNNFCAG-VCA--S---TVHK--------WN-KLNNTG----
XP_025632115.1/1-241 ---------SMLKLAQRMTNNFCAG-VCA--S---TVHK--------WN-KLNNTG----
XP_025632116.1/1-241 ---------SMLKLAQRMTNNFCAG-VCA--S---TVHK--------WN-KLNNTG----
XP_017442222.1/1-246 ---------SMVKLAQRMTNNFCAG-VCA--S---TVHK--------WN-KLNP-G----
XP_017442225.1/1-246 ---------SMVKLAQRMTNNFCAG-VCA--S---TVHK--------WN-KLNP-G----
XP_006576359.1/1-244 ---------SMVKLAQRMTNNFCAG-VCA--S---TVHK--------WN-KLNAAA----
XP_003521284.1/1-244 ---------SMVKLAQRMTNNFCAG-VCA--S---TVHK--------WN-KLNAAA----
XP_024174849.1/1-237 ---------SMLKLAQRMTDNFCAG-VCA--S---TVHK--------WN-KLHAG-----
XP_024174854.1/1-237 ---------SMLKLAQRMTDNFCAG-VCA--S---TVHK--------WN-KLHAG-----
XP_004306832.1/1-241 ---------SMLKLAQRMTDNFCAG-VCA--S---TVHK--------WN-KLNAG-----
XP_011469062.1/1-241 ---------SMLKLAQRMTDNFCAG-VCA--S---TVHK--------WN-KLNAG-----
XP_009340632.2/1-243 ---------SMLKLAHRMTDNFCAG-VCA--S---TVHK--------WT-KLNAG-----
XP_008385703.1/1-243 ---------SMLKLAQRMTDNFCAG-VCA--S---TVHK--------WT-KLNAG-----
XP_008385704.1/1-243 ---------SMLKLAQRMTDNFCAG-VCA--S---TVHK--------WT-KLNAG-----
XP_008232741.1/1-243 ---------SMLKLAQRMTDNFCAG-VCA--S---TVHK--------WN-KLNAR-----
XP_008232742.1/1-243 ---------SMLKLAQRMTDNFCAG-VCA--S---TVHK--------WN-KLNAR-----
XP_007220256.1/1-243 ---------SMLKLAQRMTDNFCAG-VCA--S---TVHK--------WN-KLNAR-----
XP_020413144.1/1-243 ---------SMLKLAQRMTDNFCAG-VCA--S---TVHK--------WN-KLNAR-----
XP_021817103.1/1-243 ---------SMLKLAQRMTDNFCAG-VCA--S---TVHK--------WN-KLNAR-----
XP_021817105.1/1-243 ---------SMLKLAQRMTDNFCAG-VCA--S---TVHK--------WN-KLNAR-----
XP_022872559.1/1-230 ---------SMLKLAQRMTNSFCAG-VCV--S---TAHK--------WN-NLCIG-----
XP_016575742.1/1-238 ----PSGRRSMLKLAQRMTNNFCAG-VCA--S---TVHK--------WN-K---------
XP_017608938.1/1-232 ---------SMLKLAQRMTGNFCAG-VCA--S---TVHK--------WN-KLNAG-----
XP_017608947.1/1-232 ---------SMLKLAQRMTGNFCAG-VCA--S---TVHK--------WN-KLNAG-----
XP_010255501.1/1-245 ----PSGRRSMLKLAQRMTDNFCAG-VCA--S---AVHK--------WN-KLCTG-----
XP_011023407.1/1-238 ---------SMLKLAQRMTDNFCAG-VCA--S---TVHK--------WN-KLNAG-----
XP_011023404.1/1-238 ---------SMLKLAQRMTDNFCAG-VCA--S---TVHK--------WN-KLNAG-----
XP_011023405.1/1-238 ---------SMLKLAQRMTDNFCAG-VCA--S---TVHK--------WN-KLNAG-----
XP_019152252.1/1-243 ----PSGRRSMLKLAQRMTNNFCAG-VCA--S---SVHQ--------WN-KLCAG-----
XP_022870029.1/1-235 ----PSGRRSMLKLAQRMTDNFCAG-VCA--S---TVHE--------WN-KFSAG-----
XP_017699285.1/1-225 ---------SMLKLAQRMTDNFCAG-VCA--S---SAHK--------WS-KLSL-G----
XP_008795402.1/1-225 ---------SMLKLAQRMTDNFCAG-VCA--S---SAHK--------WS-KLSL-G----
XP_010933869.1/1-224 ---------SMLKLAQRMTDNFCAG-VCA--S---SAHK--------WS-KLSL-G----
XP_008799528.1/1-222 ---------SMLKLAQRMTDNFCAG-VCA--S---SAHK--------WS-KLSV-G----
XP_010919252.1/1-223 ---------SMLKLAQRMTDNFCAG-VCA--S---SAHK--------WS-KLSV-G----
XP_010919250.1/1-223 ---------SMLKLAQRMTDNFCAG-VCA--S---SAHK--------WS-KLSV-G----
XP_007139955.1/1-229 ---------SMLKLAQRMTNNFCAG-VCA--S---TVHK--------WN-KLNA-G----
XP_017418814.1/1-230 ---------SMLKLAQRMTNNFCAG-VCA--S---TVHK--------WN-KLNA-G----
XP_014490274.1/1-230 ---------SMLKLAQRMTNNFCAG-VCA--S---TVHK--------WN-KLNA-G----
XP_003534596.1/1-229 ---------SMLKLAQRMTNNFCAG-VCA--S---TVHK--------WN-KLNA-G----
XP_006587871.1/1-229 ---------SMLKLAQRMTNNFCAG-VCA--S---TVHK--------WN-KLNA-G----
XP_003552359.1/1-228 ---------SMLKLAHRMTNNFCSG-VCA--S---TVHK--------WN-KLNA-G----
XP_015961616.1/1-228 ---------SMLKLAQRMTNNFCAG-VCA--S---TVHK--------WN-KLNA-G----
XP_015961617.1/1-228 ---------SMLKLAQRMTNNFCAG-VCA--S---TVHK--------WN-KLNA-G----
XP_025696060.1/1-228 ---------SMLKLAQRMTNNFCAG-VCA--S---TVHK--------WN-KLNA-G----
XP_025696061.1/1-228 ---------SMLKLAQRMTNNFCAG-VCA--S---TVHK--------WN-KLNA-G----
XP_016198931.1/1-228 ---------SMLKLAQRMTNNFCAG-VCA--S---TVHK--------WN-KLNA-G----
XP_016198932.1/1-228 ---------SMLKLAQRMTNNFCAG-VCA--S---TVHK--------WN-KLNA-G----
XP_025649261.1/1-228 ---------SMLKLAQRMTNNFCAG-VCA--S---TVHK--------WN-KLNA-G----
XP_025649262.1/1-228 ---------SMLKLAQRMTNNFCAG-VCA--S---TVHK--------WN-KLNA-G----
XP_021849370.1/1-228 ---------SMLKLAQRMTNNFCAG-VCA--S---SVHK--------WN-KLNI-G----
XP_021767325.1/1-228 ---------SMLKLAQRMTNNFCAG-VCA--S---TVHK--------WN-KLNI-G----
XP_021748881.1/1-228 ---------SMLKLAQRMTNNFCAG-VCA--S---TVHK--------WN-KLNI-G----
XP_019461451.1/1-231 ---------SMLKLAQRMTNNFCAG-VCA--S---TVHK--------WN-KLN-PG----
XP_012574211.1/1-233 ---------SMLKLAQRMTNNFCAG-VCA--S---TVHK--------WN-KLS-PG----
XP_004510857.1/1-233 ---------SMLKLAQRMTNNFCAG-VCA--S---TVHK--------WN-KLS-PG----
XP_020237185.1/1-234 ---------SMLKLAQRMTNNFCAG-VCA--S---TVHK--------WN-KLS-PG----
XP_020237261.1/1-234 ---------SMLKLAQRMTNNFCAG-VCA--S---TVHK--------WN-KLS-PG----
XP_019456357.1/1-238 ---------SMLKLAQRMTKNFCAG-VCA--S---TVHK--------WN-KLN-PG----
XP_013444922.1/1-235 ---------SMLKLAQRMTNNFCAG-VCA--S---TVHK--------WN-KLN-PG----
XP_003528880.1/1-238 ---------SMMKLAQRMTNNFCAG-VCA--S---TVHK--------WN-KLN-AG----
XP_006583340.1/1-238 ---------SMMKLAQRMTNNFCAG-VCA--S---TVHK--------WN-KLN-AG----
XP_014516480.1/1-238 ---------SMVKLAQRMTNNFCAG-VCA--S---TVHK--------WN-KLN-PG----
XP_014516483.1/1-238 ---------SMVKLAQRMTNNFCAG-VCA--S---TVHK--------WN-KLN-PG----
XP_007134961.1/1-238 ---------SMVKLAQRMTNNFCAG-VCA--S---TVHK--------WN-KLN-PG----
XP_010671720.1/1-229 ---------SMLKLAQRMTNNFCAG-VCA--S---TVHK--------WN-KLN-VG----
XP_012482732.1/1-232 ---------SMLKLAQRMTGNFCAG-VCA--S---TVHK--------WN-KLN-AG----
XP_012482742.1/1-232 ---------SMLKLAQRMTGNFCAG-VCA--S---TVHK--------WN-KLN-AG----
XP_016745638.1/1-232 ---------SMLKLAQRMTGNFCAG-VCA--S---TVHK--------WN-KLN-VG----
XP_016745639.1/1-232 ---------SMLKLAQRMTGNFCAG-VCA--S---TVHK--------WN-KLN-VG----
XP_010278578.1/1-242 ----PSGRRSMLKLAQRMTDNFCAG-VCA--S---AVHK--------WN-KLC-AG----
XP_010278577.1/1-242 ----PSGRRSMLKLAQRMTDNFCAG-VCA--S---AVHK--------WN-KLC-AG----
XP_006445143.1/1-238 ---------SMLKLAQRMTDNFCAG-VCA--S---TVHK--------WN-KLN-AG----
XP_006445141.1/1-238 ---------SMLKLAQRMTDNFCAG-VCA--S---TVHK--------WN-KLN-AG----
XP_022770361.1/1-238 ---------SMLKLAQRMTDNFCAG-VCA--S---TVHK--------WN-KLN-AG----
XP_022757219.1/1-237 ---------SMLKLAQRMTDNFCAG-VCA--S---TVHK--------WN-KLN-AG----
XP_022757220.1/1-237 ---------SMLKLAQRMTDNFCAG-VCA--S---TVHK--------WN-KLN-AG----
NP_001314420.1/1-237 ---------SMLKLAQRMTDNFCAG-VCA--S---TVHK--------WN-KLN-AG----
XP_016735217.1/1-237 ---------SMLKLAQRMTDNFCAG-VCA--S---TVHK--------WN-KLN-AG----
XP_012437660.1/1-237 ---------SMLKLAQRMTDNFCAG-VCA--S---TVHK--------WN-KLN-AG----
XP_012437661.1/1-237 ---------SMLKLAQRMTDNFCAG-VCA--S---TVHK--------WN-KLN-AG----
XP_017615505.1/1-237 ---------SMLKLAQRMTDNFCAG-VCA--S---TVHK--------WN-KLN-AG----
XP_017615506.1/1-237 ---------SMLKLAQRMTDNFCAG-VCA--S---TVHK--------WN-KLN-AG----
XP_016723220.1/1-237 ---------SMLKLAQRMTDNFCAG-VCA--S---TVHK--------WN-KPN-AG----
XP_016723229.1/1-237 ---------SMLKLAQRMTDNFCAG-VCA--S---TVHK--------WN-KPN-AG----
XP_021904309.1/1-244 ---------SMLKLAQRMTDNFCAG-VCA--S---TVHK--------WN-KLS-VG----
XP_021904311.1/1-244 ---------SMLKLAQRMTDNFCAG-VCA--S---TVHK--------WN-KLS-VG----
XP_007051912.2/1-241 ---------SMLKLAQRMTDNFCAG-VCA--S---TLHK--------WN-KLNNAG----
XP_007051913.2/1-241 ---------SMLKLAQRMTDNFCAG-VCA--S---TLHK--------WN-KLNNAG----
XP_012489878.1/1-239 ---------SMLKLAQRMTDNFCAG-VCA--S---TVHK--------WN-KLN-VG----
XP_016748231.1/1-239 ---------SMLKLAQRMTDNFCAG-VCA--S---TVHK--------WN-KLN-VG----
XP_017631289.1/1-239 ---------SMLKLAQRMTDNFCAG-VCA--S---TVHK--------WN-KLN-VG----
XP_023006686.1/1-236 ---------SMLKLAQRMTANFCAG-VCA--S---TVHK--------WN-KLNA-G----
XP_022958954.1/1-236 ---------SMLKLAQRMTANFCAG-VCA--S---TVHK--------WN-KLNA-G----
XP_023548045.1/1-236 ---------SMLKLAQRMTANFCAG-VCA--S---TVHK--------WN-KLNA-G----
XP_008460172.1/1-241 ---------SMLKLAQRMTANFCAG-VCA--S---TVHK--------WN-KLNA-G----
XP_004144982.1/1-241 ---------SMLKLAQRMTANFCAG-VCA--S---TVHK--------WN-KLNA-G----
XP_023514722.1/1-242 ---------SMLKLAQRMTANFCAG-VCA--S---TVHK--------WN-KLNA-G----
XP_022999814.1/1-242 ---------SMLKLAQRMTANFCAG-VCA--S---TVHK--------WN-KLNA-G----
XP_022964504.1/1-242 ---------SMLKLAQRMTANFCAG-VCA--S---TVHK--------WN-KLNA-G----
XP_010053664.1/1-245 ----PSGRRSMLKLAQRMTANFCAG-VCA--S---TVHK--------WN-KLS-GG----
XP_019189515.1/1-235 ---------SMLKLAQRMTNNFCAG-VCA--S---TVHK--------WN-KLCA-G----
XP_023730775.1/1-234 ---------SMLKLAQRMTDNFCAG-VCA--S---TVYK--------WN-KLCA-N----
XP_023730774.1/1-234 ---------SMLKLAQRMTDNFCAG-VCA--S---TVYK--------WN-KLCA-N----
XP_022020543.1/1-230 ---------SMLKLAQRMTDNFCAG-VCA--S---TVYK--------WN-KLCA-T----
XP_024962572.1/1-236 ---------SMLKLAQRMTDNFCAG-VCA--S---TVYK--------WN-KLCA-N----
XP_022017696.1/1-236 ---------SMLKLAQRMTDNFCAG-VCA--S---TVYK--------WN-KLRA-N----
XP_024018220.1/1-245 ---------SMLKLAQRMTDNFCAG-VCA--S---TVHK--------WN-KLNATG----
XP_011093166.1/1-234 ---------SMLKLAQRMTNNFFAG-VCA--S---TVHK--------WN-KLR-TE----
XP_011093167.1/1-234 ---------SMLKLAQRMTNNFFAG-VCA--S---TVHK--------WN-KLR-TE----
XP_006339457.1/1-238 ----PSGRRSMLKLAQRMTNNFCAG-VCA--S---TVHK--------WN-KLC-AG----
XP_015059298.1/1-238 ----PSGRRSMLKLAQRMTNNFCAG-VCA--S---TVHK--------WN-KLC-AG----
NP_001234657.2/1-238 ----PSGRRSMLKLAQRMTNNFCAG-VCA--S---TVHK--------WN-KLC-AG----
XP_009602857.1/1-241 ----PSGRRSMLKLAQRMTNNFCAG-VCA--S---TVHK--------WN-KLC-AG----
XP_009602856.1/1-241 ----PSGRRSMLKLAQRMTNNFCAG-VCA--S---TVHK--------WN-KLC-AG----
XP_009782444.1/1-245 ----PSGRRSMLKLAQRMTNNFCAG-VCA--S---TVHK--------WN-KLC-AG----
XP_009782443.1/1-245 ----PSGRRSMLKLAQRMTNNFCAG-VCA--S---TVHK--------WN-KLC-AG----
XP_019259406.1/1-245 ----PSGRRSMLKLAQRMTNNFCAG-VCA--S---TVHK--------WN-KLC-AG----
XP_015867375.1/1-242 ---------SMLKLAQRMTDNFCAG-VCA--S---TVHK--------WT-KLN-AG----
XP_015867374.1/1-242 ---------SMLKLAQRMTDNFCAG-VCA--S---TVHK--------WT-KLN-AG----
XP_015888672.1/1-242 ---------SMLKLAQRMTDNFCAG-VCA--S---TVHK--------WT-KLN-AG----
XP_018810322.1/1-233 ---------SMLKLAQRMTDNFCAG-VCA--S---TVHK--------WN-KLH-AD----
XP_018859074.1/1-233 ---------SMLKLAQRMTDNFCAG-VCA--S---TVHK--------WN-KLH-AD----
XP_017231243.1/1-235 ---------SMLKLAQRMTNNFCAG-VCA--S---TVHK--------WN-KLT-AG----
XP_017231244.1/1-235 ---------SMLKLAQRMTNNFCAG-VCA--S---TVHK--------WN-KLT-AG----
XP_017231245.1/1-235 ---------SMLKLAQRMTNNFCAG-VCA--S---TVHK--------WN-KLT-AG----
XP_017236322.1/1-235 ---------SMLKLAQRMTDNFCAG-VCA--S---TVHK--------WN-KLS-AG----
XP_002301331.2/1-238 ---------SMLKLAQRMTDNFCAG-VCA--S---TVHK--------WN-KLN-AG----
XP_024451706.1/1-238 ---------SMLKLAQRMTDNFCAG-VCA--S---TVHK--------WN-KLN-AG----
XP_002320755.1/1-241 ---------SMLKLAQRMTANFCAG-VCA--S---TVHK--------WN-KLN-AG----
XP_011035097.1/1-241 ---------SMLKLAQRMTANFCAG-VCA--S---TVHK--------WN-KLN-AG----
XP_018813891.1/1-238 ---------SMLKLAQRMTDNFCAG-VCA--S---TVHK--------WN-KLQ-AG----
XP_012083470.1/1-239 ---------SMLKLAQRMTDNFCAG-VCA--S---TVHK--------WN-KLN-AG----
XP_021651338.1/1-240 ---------SMLKLAQRMTDNFCAG-VCA--S---TVHK--------WN-KLN-AG----
XP_021613538.1/1-241 ---------SMLKLAQRMTDNFCAG-VCA--S---TVHK--------WN-KLN-AG----
XP_021686862.1/1-241 ---------SMLKLAQRMTDNFCAG-VCA--S---TVHK--------WN-KLN-AG----
XP_021686863.1/1-241 ---------SMLKLAQRMTDNFCAG-VCA--S---TVHK--------WN-KLN-AG----
XP_021612685.1/1-241 ---------SMLKLAQRMTDNFCAG-VCA--S---TVHK--------WN-KLN-AG----
XP_023879166.1/1-242 ---------SMLKLAQRMTDNFCAG-VCA--S---TVHK--------WN-KLN-AG----
XP_002272264.2/1-234 ---------SMLKLAQRMTDNFCAG-VCA--S---TVHK--------WN-KLC-AG----
XP_010661562.1/1-234 ---------SMLKLAQRMTDNFCAG-VCA--S---TVHK--------WN-KLC-AG----
XP_002511801.1/1-237 ---------SMLKLAQRMTDNFCAG-VCA--S---TVHK--------WN-KLN-AG----
XP_015584500.1/1-237 ---------SMLKLAQRMTDNFCAG-VCA--S---TVHK--------WN-KLN-AG----
XP_022141257.1/1-238 ---------SMLKLSQRMVDNFCSG-ICT--S---TLHK--------WD-KLVVG-----
XP_023885612.1/1-244 ----PAGKRSMLKLAQRMTDKFCSG-VCA--S---TTHK--------WD-NLRLG-----
XP_023885611.1/1-244 ----PAGKRSMLKLAQRMTDKFCSG-VCA--S---TTHK--------WD-NLRLG-----
XP_018830842.1/1-239 --------SSMLKLAQRMTDNFCSG-VCA--S---TARN--------WD-NLHVG-----
XP_018830843.1/1-239 --------SSMLKLAQRMTDNFCSG-VCA--S---TARN--------WD-NLHVG-----
XP_018830840.1/1-239 --------SSMLKLAQRMTDNFCSG-VCA--S---TARN--------WD-NLHVG-----
XP_018830841.1/1-239 --------SSMLKLAQRMTDNFCSG-VCA--S---TARN--------WD-NLHVG-----
XP_012090293.1/1-240 ---------SMLKLAQRMVDNFCCG-VSA--S---SVRK--------WD-KLLVG-----
XP_012090292.1/1-240 ---------SMLKLAQRMVDNFCCG-VSA--S---SVRK--------WD-KLLVG-----
XP_021614581.1/1-239 ---------SMLKLARRMVDNFCSG-VCA--S---SVHK--------WD-KLLVG-----
XP_021614580.1/1-239 ---------SMLKLARRMVDNFCSG-VCA--S---SVHK--------WD-KLLVG-----
XP_015581247.1/1-240 ---------SMLKLAQRMVDNFCSG-VCA--S---SLRK--------WD-KLLVG-----
XP_002510822.1/1-240 ---------SMLKLAQRMVDNFCSG-VCA--S---SLRK--------WD-KLLVG-----
XP_021648646.1/1-240 ---------SMVKLAQRMVDNFCSG-VCA--S---SLRK--------WD-KLLVG-----
XP_021634593.1/1-234 ---------SMVKLAQRMVENFCSG-VCA--S---SARK--------WD-KLTVE-----
XP_021634592.1/1-234 ---------SMVKLAQRMVENFCSG-VCA--S---SARK--------WD-KLTVE-----
XP_006490348.1/1-238 ---------SMLKLAQRMTYNFCSG-VCA--S---SVRK--------WD-KLCVG-----
XP_006490346.1/1-238 ---------SMLKLAQRMTYNFCSG-VCA--S---SVRK--------WD-KLCVG-----
XP_024958654.1/1-238 ---------SMLKLAQRMTYNFCSG-VCA--S---SVRK--------WD-KLCVG-----
XP_006490345.1/1-238 ---------SMLKLAQRMTYNFCSG-VCA--S---SVRK--------WD-KLCVG-----
XP_006421879.2/1-238 ---------SMLKLAQRMTYNFCSG-VCA--S---SVRK--------WD-KLCVG-----
XP_021892212.1/1-221 ---------SMLKLAQRMTYNFCTG-VCA--S---SVRK--------WD-KLQIG-----
XP_021892211.1/1-221 ---------SMLKLAQRMTYNFCTG-VCA--S---SVRK--------WD-KLQIG-----
XP_021892210.1/1-221 ---------SMLKLAQRMTYNFCTG-VCA--S---SVRK--------WD-KLQIG-----
XP_022765148.1/1-244 ----PAGRKSMIKLAQRMTNNFCSG-VCA--S---SMHK--------WD-KLSVG-----
XP_022765158.1/1-244 ----PAGRKSMIKLAQRMTNNFCSG-VCA--S---SMHK--------WD-KLSVG-----
XP_022765120.1/1-244 ----PAGRKSMIKLAQRMTNNFCSG-VCA--S---SMHK--------WD-KLSVG-----
XP_022765114.1/1-244 ----PAGRKSMIKLAQRMTNNFCSG-VCA--S---SMHK--------WD-KLSVG-----
XP_022736693.1/1-237 -----------LKLAQRMTCNFCSG-VCA--S---SMHK--------WD-RLSVG-----
XP_022736692.1/1-237 -----------LKLAQRMTCNFCSG-VCA--S---SMHK--------WD-RLSVG-----
XP_022736689.1/1-237 -----------LKLAQRMTCNFCSG-VCA--S---SMHK--------WD-RLSVG-----
XP_022734775.1/1-244 ----PAGRKSMLKLAQRMTYNFCAG-VCT--S---SVHK--------WD-KLSVG-----
XP_016743353.1/1-244 ----PAGRKSMIKLAQRMTYNFCAG-VCA--S---SIHK--------WD-KLSVG-----
XP_012474605.1/1-244 ----PAGRKSMIKLAQRMTYNFCAG-VCA--S---SIHK--------WD-KLSVG-----
XP_012474606.1/1-244 ----PAGRKSMIKLAQRMTYNFCAG-VCA--S---SIHK--------WD-KLSVG-----
XP_016743352.1/1-244 ----PAGRKSMIKLAQRMTYNFCAG-VCA--S---SIHK--------WD-KLSVG-----
XP_016743351.1/1-244 ----PAGRKSMIKLAQRMTYNFCAG-VCA--S---SIHK--------WD-KLSVG-----
XP_012474604.1/1-244 ----PAGRKSMIKLAQRMTYNFCAG-VCA--S---SIHK--------WD-KLSVG-----
XP_012474603.1/1-244 ----PAGRKSMIKLAQRMTYNFCAG-VCA--S---SIHK--------WD-KLSVG-----
XP_017646297.1/1-244 ----PAGRKSMIKLAQRMTYNFCAG-VCA--S---SVHK--------WD-KLSVG-----
XP_016736315.1/1-244 ----PAGRKSMIKLAQRMTYNFCAG-VCA--S---SVHK--------WD-KLSVG-----
XP_017646296.1/1-244 ----PAGRKSMIKLAQRMTYNFCAG-VCA--S---SVHK--------WD-KLSVG-----
XP_017646298.1/1-244 ----PAGRKSMIKLAQRMTYNFCAG-VCA--S---SVHK--------WD-KLSVG-----
XP_016736306.1/1-244 ----PAGRKSMIKLAQRMTYNFCAG-VCA--S---SVHK--------WD-KLSVG-----
XP_017646295.1/1-244 ----PAGRKSMIKLAQRMTYNFCAG-VCA--S---SVHK--------WD-KLSVG-----
XP_017972896.1/1-244 ----PAGRKNMLKLAQRMTYNFCAG-VCA--S---SVHK--------WD-KLSVG-----
XP_017972895.1/1-244 ----PAGRKNMLKLAQRMTYNFCAG-VCA--S---SVHK--------WD-KLSVG-----
XP_017972893.1/1-244 ----PAGRKNMLKLAQRMTYNFCAG-VCA--S---SVHK--------WD-KLSVG-----
XP_021274823.1/1-244 ----PAGRKNMLKLAQRMTYNFCAG-VCA--S---SVHK--------WD-KLSVG-----
XP_024536343.1/1-231 ---------SMLKLAQRMTNNFCAG-VSA--S---TVHT--------WT-TLSGS-----
XP_006366174.1/1-244 ----PSGRRSMLKLAQRMTNNFCSG-VCA--S---SIHK--------WN-KLNCVG----
XP_020219711.1/1-212 ---------SMLKLAQRMTNNFCAG-VCA--S---TVHK--------WN-KLNA------
XP_022897056.1/1-222 ---------SMLKLAQRMTNSFCAG-VCV--S---TANK--------WN-KLCI------
NP_001281015.1/1-243 ---------SMLKLAQRMTDNFCAG-VCA--S---TVHK--------WT-KLNA------
XP_008383260.1/1-243 ---------SMLKLAQRMTDNFCAG-VCA--S---TVHK--------WT-KLNA------
XP_022896229.1/1-235 ----PSGRRSMLKLAHRMTDNFCAG-VCP--S---TVHE--------WN-SFST------
XP_022896230.1/1-235 ----PSGRRSMLKLAHRMTDNFCAG-VCP--S---TVHE--------WN-SFST------
XP_025816451.1/1-225 ---------SMLKLARRMTENFCAG-VSA--S---SARE--------WS-KLDGAT----
XP_004968001.1/1-225 ---------SMLKLARRMTENFCAG-VSA--S---SARE--------WS-KLDGAT----
XP_004968002.1/1-225 ---------SMLKLARRMTENFCAG-VSA--S---SARE--------WS-KLDGAT----
XP_002457484.1/1-226 ---------SMLKLARRMTENFCAG-VSA--S---SARE--------WS-KLDGAA----
XP_009400446.1/1-224 ----PSGRRSMLKLAQRMTDNFCAG-VCA--S---SAHQ--------WN-KLRG-G----
XP_018676535.1/1-225 ----PSGRRSMLKLAQRMTDNFCAG-VCA--S---SARE--------WS-KLGG-A----
XP_009401771.1/1-232 ----PSGRRSMLKLAQRMTDNFCAG-VCA--S---SARE--------WK-KLGG-G----
XP_009384534.1/1-228 ----PSGRRSMLKLAQRMTDNFCAG-VCA--S---SARE--------WN-KLGG-G----
XP_020672447.1/1-217 ---------SMLKLAQRMTDNFCAG-VCA--S---SARK--------WS-RLRNMG----
XP_020582116.1/1-218 ---------SMLKLAQRMTDNFCAG-VCA--S---SARK--------WS-RLRNMG----
XP_020675647.1/1-231 ----PAGRRSMLKLAQRMTDNFCAG-VCA--S---SARK--------WS-HLANMG----
XP_020675648.1/1-231 ----PAGRRSMLKLAQRMTDNFCAG-VCA--S---SARK--------WS-HLANMG----
XP_020585632.1/1-228 ---------SMLKLAQRMTDNFCAG-VCA--S---SARK--------WS-RLANMG----
XP_016176402.1/1-228 ---------SMLKLAQRMTSNFCSG-VCA--S---SARK--------WD-SLHIGT----
XP_016176403.1/1-228 ---------SMLKLAQRMTSNFCSG-VCA--S---SARK--------WD-SLHIGT----
XP_016176400.1/1-228 ---------SMLKLAQRMTSNFCSG-VCA--S---SARK--------WD-SLHIGT----
XP_016176401.1/1-228 ---------SMLKLAQRMTSNFCSG-VCA--S---SARK--------WD-SLHIGT----
XP_010523042.1/1-245 ---------SMLKLAQRMTYNFCSG-ISA--P---SVQS--------WS-KLTVGH----
XP_010523041.1/1-240 ---------SMLKLAQRMTYNFCSG-ISA--P---SVQS--------WS-KLTVGH----
XP_020878511.1/1-224 ---------SMLKLAQRMTFNFCSG-ISA--P---SVHS--------WS-KLTVGN----
NP_567183.2/1-228    ---------SMLKLAQRMTFNFCSG-ISA--P---SVHN--------WS-KLTVGN----
XP_006287095.1/1-228 ---------SMLKLAQRMTFNFCSG-ISA--P---SVHC--------WS-KLTVGN----
XP_010456250.1/1-228 ---------SMLKLAQRMTFNFCSG-ISA--P---SVHN--------WS-KLTVGN----
XP_010419627.1/1-228 ---------SMLKLAQRMTFNFCSG-ISA--P---SVHN--------WS-KLTVGN----
XP_010427345.1/1-228 ---------SMLKLAQRMTFNFCSG-ISA--P---SVHN--------WS-KLTVGN----
XP_009111469.1/1-229 ---------SMLKLAQRMTINFCSG-ISA--P---SVHS--------WS-KLTVGN----
XP_018487039.1/1-229 ---------SMLKLAQRMTSNFCSG-ISA--P---SVHS--------WS-KLTVGN----
XP_018453129.1/1-229 ---------SMLKLAQRMTSNFCSG-ISA--P---SVHS--------WS-KLTVGN----
XP_013612299.1/1-229 ---------SMLKLAQRMTINFCSG-ISA--P---SVHS--------WS-KLTVGN----
XP_006396268.1/1-225 ---------SMLKLAQRMTFNFCSG-ISA--P---SVHS--------WS-KLTVGN----
XP_018475317.1/1-221 ---------SMLKLAQRMTVNFCSG-ISA--P---SVHS--------WS-KLTVEN----
XP_013629750.1/1-221 ---------SMLKLAQRMTVNFCSG-ISA--P---SVHS--------WS-KLTVEN----
XP_009134533.1/1-221 ---------SMLKLAQRMTVNFCSG-ISA--P---SVHS--------WS-KLTVEN----
XP_020531377.1/1-244 ----PAGRRSMLKLAQRMTGRFCAG-VCG--S---HVHD--------WA-HLSGSG----
XP_006857866.1/1-244 ----PAGRRSMLKLAQRMTGRFCAG-VCG--S---HVHD--------WA-HLSGSG----
XP_018434263.1/1-219 ---------SMLKLAQRMTENFCRG-VCA--S---SSQK--------WS-KLDIGN----
XP_018434260.1/1-219 ---------SMLKLAQRMTENFCRG-VCA--S---SSQK--------WS-KLDIGN----
XP_018434262.1/1-219 ---------SMLKLAQRMTENFCRG-VCA--S---SSQK--------WS-KLDIGN----
XP_013688926.1/1-219 ---------SMLRLAQRMTENFCRG-VCA--S---SSHK--------WS-KLDIGN----
XP_013637558.1/1-219 ---------SMLRLAQRMTENFCRG-VCA--S---SSHK--------WS-KLDIGN----
XP_013637556.1/1-219 ---------SMLRLAQRMTENFCRG-VCA--S---SSHK--------WS-KLDIGN----
XP_013637557.1/1-219 ---------SMLRLAQRMTENFCRG-VCA--S---SSHK--------WS-KLDIGN----
XP_009143168.1/1-219 ---------SMLRLAQRMTENFCRG-VCA--S---SSQK--------WS-KLDIGN----
XP_009143167.1/1-219 ---------SMLRLAQRMTENFCRG-VCA--S---SSQK--------WS-KLDIGN----
XP_009143166.1/1-219 ---------SMLRLAQRMTENFCRG-VCA--S---SSQK--------WS-KLDIGN----
XP_013692686.1/1-219 ---------SMLRLAQRMTENFCRG-VCA--S---SSQK--------WS-KLDIGN----
XP_022574820.1/1-219 ---------SMLRLAQRMTENFCRG-VCA--S---SSQK--------WS-KLDIGN----
XP_013692684.1/1-219 ---------SMLRLAQRMTENFCRG-VCA--S---SSQK--------WS-KLDIGN----
XP_013748127.1/1-219 ---------SMLRLAQRMTENFCRG-VCA--S---SSQK--------WS-KLDIGN----
XP_013692685.1/1-219 ---------SMLRLAQRMTENFCRG-VCA--S---SSQK--------WS-KLDIGN----
XP_013748124.1/1-219 ---------SMLRLAQRMTENFCRG-VCA--S---SSQK--------WS-KLDIGN----
XP_013748126.1/1-219 ---------SMLRLAQRMTENFCRG-VCA--S---SSQK--------WS-KLDIGN----
XP_022574821.1/1-219 ---------SMLRLAQRMTENFCRG-VCA--S---SSQK--------WS-KLDIGN----
XP_006290613.1/1-229 ---------SMLKLAKRMTDNFCGG-VCA--S---SLQK--------WS-KLNVGN----
XP_013592301.1/1-230 ---------SMLKLARRMTDNFCGG-VCA--S---SLQK--------WS-KLNVGN----
XP_018442581.1/1-230 ---------SMLKLAKRMTDNFCGG-VCA--S---SLQK--------WS-KLNVGN----
XP_013750213.1/1-232 ---------SMLKLAKRMTDNFCGG-VCA--S---SLQK--------WS-KLNVGN----
XP_013648430.1/1-232 ---------SMLKLAKRMTDNFCGG-VCA--S---SLQK--------WS-KLNVGN----
XP_009104292.1/1-232 ---------SMLKLAKRMTDNFCGG-VCA--S---SLQK--------WS-KLNVGN----
XP_019058514.1/1-223 ---------SMLKLAQRMTDNFCGG-VCA--S---SAQK--------WS-QLNVGN----
XP_010523728.1/1-220 ---------SMLKLAQRMTENFCGG-VCA--S---SAQK--------WS-KLNVGN----
XP_024514927.1/1-242 ---------SMLFLAQRMTSNYCAG-VSA--S---IVHT--------WT-TLSGA-----
XP_002985242.1/1-242 ---------SMLFLAQRMTSNYCAG-VSA--S---IVHT--------WT-TLSGA-----
XP_024530500.1/1-242 ---------SMLFLAQRMTSNYCAG-VSA--S---IVHT--------WT-TLSGA-----
XP_024530499.1/1-242 ---------SMLFLAQRMTSNYCAG-VSA--S---IVHT--------WT-TLSGA-----
XP_024530501.1/1-242 ---------SMLFLAQRMTSNYCAG-VSA--S---IVHT--------WT-TLSGA-----
XP_010107411.1/1-242 ----PSGKRSMLKLAQRMVDNFCAG-VTA--S---TVRK--------WD-KLCVG-----
XP_024028635.1/1-242 ----PSGKRSMLKLAQRMVDNFCAG-VTA--S---TVRK--------WD-KLCVG-----
XP_015877999.1/1-244 ---------SMLKLAQRMVDNFCTG-VCA--S---TVGK--------WD-KLHVG-----
XP_019427294.1/1-247 ---------SMLKLAQRMTSSFYSG-VCA--S---SARK--------WE-SLSMG-----
XP_019427292.1/1-247 ---------SMLKLAQRMTSSFYSG-VCA--S---SARK--------WE-SLSMG-----
XP_019452814.1/1-239 ---------SMLKLAQRMTSNFCSG-VCA--S---SARK--------WE-SLPVG-----
XP_019452815.1/1-239 ---------SMLKLAQRMTSNFCSG-VCA--S---SARK--------WE-SLPVG-----
XP_003592295.2/1-235 ---------SMLKLAQRMTNNFCSG-VCA--S---SARK--------WD-SLQMG-----
XP_024625328.1/1-235 ---------SMLKLAQRMTNNFCSG-VCA--S---SARK--------WD-SLQMG-----
XP_012570175.1/1-236 ---------SMLKLAQRMTSNFCSG-VCA--S---SARK--------WD-TLQMG-----
XP_012570176.1/1-236 ---------SMLKLAQRMTSNFCSG-VCA--S---SARK--------WD-TLQMG-----
XP_004496593.1/1-236 ---------SMLKLAQRMTSNFCSG-VCA--S---SARK--------WD-TLQMG-----
XP_025620154.1/1-234 ---------SMLKLAQRMTSNFCSG-VCA--S---SARK--------WD-SLHIG-----
XP_015941562.1/1-234 ---------SMLKLAQRMTSNFCSG-VCA--S---SARK--------WD-SLHIG-----
XP_025620153.1/1-234 ---------SMLKLAQRMTSNFCSG-VCA--S---SARK--------WD-SLHIG-----
XP_020239673.1/1-232 ---------SMLKLAQRMTSNFCSG-VCA--S---SARK--------WD-SLHIG-----
XP_020239648.1/1-232 ---------SMLKLAQRMTSNFCSG-VCA--S---SARK--------WD-SLHIG-----
XP_007143377.1/1-235 ---------SMLKLAQRMTGNFCSG-VCA--S---SARK--------WD-MLHIG-----
XP_007143378.1/1-235 ---------SMLKLAQRMTGNFCSG-VCA--S---SARK--------WD-MLHIG-----
XP_017415467.1/1-234 ---------SMLKLAQRMTSNFCSG-VCA--S---SARK--------WD-MLHIG-----
XP_017415466.1/1-234 ---------SMLKLAQRMTSNFCSG-VCA--S---SARK--------WD-MLHIG-----
XP_014514421.1/1-236 ---------SMLKLAQRMTSNFCSG-VCA--S---SARK--------WD-MLHIG-----
XP_014514419.1/1-236 ---------SMLKLAQRMTSNFCSG-VCA--S---SARK--------WD-MLHIG-----
XP_006606097.1/1-235 ---------SMLKLAQRMTSNFCSG-VCA--S---SARK--------WD-SLHIG-----
XP_006606096.1/1-235 ---------SMLKLAQRMTSNFCSG-VCA--S---SARK--------WD-SLHIG-----
XP_003536477.1/1-234 ---------SMLKLAQRMTSNFCSG-VCA--S---SARK--------WD-SLHIG-----
XP_006589557.1/1-234 ---------SMLKLAQRMTSNFCSG-VCA--S---SARK--------WD-SLHIG-----
XP_006402516.1/1-230 ---------SMLKLAKRMTDNFCGG-VCA--S---SLQK--------WS-KLNVG-----
NP_191674.1/1-237    ---------SMLKLAKRMTDNFCGG-VCA--S---SLQK--------WS-KLNVG-----
XP_002876596.1/1-239 ---------SMLKLAKRMTDNFCGG-VCA--S---SLQK--------WS-KLNVG-----
XP_010413451.1/1-238 ---------SMLKLAKRMTDNFCGG-VCA--S---SLQK--------WS-KLNVG-----
XP_010413452.1/1-238 ---------SMLKLAKRMTDNFCGG-VCA--S---SLQK--------WS-KLNVG-----
XP_010413453.1/1-238 ---------SMLKLAKRMTDNFCGG-VCA--S---SLQK--------WS-KLNVG-----
XP_010469036.1/1-242 ---------SMLKLAKRMTDNFCGG-VCA--S---SLQK--------WS-KLNVG-----
XP_010469037.1/1-242 ---------SMLKLAKRMTDNFCGG-VCA--S---SLQK--------WS-KLNVG-----
XP_010469038.1/1-242 ---------SMLKLAKRMTDNFCGG-VCA--S---SLQK--------WS-KLNVG-----
XP_010512448.1/1-238 ---------SMLKLAKRMTENFCGG-VCA--S---SLQK--------WS-KLNVG-----
XP_010512449.1/1-238 ---------SMLKLAKRMTENFCGG-VCA--S---SLQK--------WS-KLNVG-----
XP_006397757.1/1-219 ---------SMLKLAQRMTENFCRG-VCA--S---SSQK--------WS-RLEVG-----
XP_024010837.1/1-220 ---------SMLKLAQRMTENFCRG-VCA--S---SSQK--------WS-RLEVG-----
XP_023640300.1/1-220 ---------SMLKLAQRMTQKFCRG-VCA--S---SSQK--------WS-KLEMG-----
XP_006293718.1/1-220 ---------SMLKLAQRMTQKFCRG-VCA--S---SSQK--------WS-KLEMG-----
XP_010506553.1/1-219 ---------SMLKLAQRMTQKFCRG-VCA--S---SSQK--------WS-KLEIG-----
XP_010508114.1/1-219 ---------SMLKLAQRMTQKFCRG-VCA--S---SSQK--------WS-KLEIG-----
XP_010508115.1/1-219 ---------SMLKLAQRMTQKFCRG-VCA--S---SSQK--------WS-KLEIG-----
XP_022147276.1/1-219 ---------SMLRLAQRMTANFCAG-VCA--S---SVYK--------WN-TLNAGG----
XP_023527464.1/1-222 ----------MVKLAQRMTANFCTG-VCA--S---TVYK--------WN-KLNTGN----
XP_022980233.1/1-222 ----------MVKLAQRMTANFCTG-VCA--S---TVYK--------WN-KLNTGN----
XP_022980231.1/1-222 ----------MVKLAQRMTANFCTG-VCA--S---TVYK--------WN-KLNTGN----
XP_022924366.1/1-223 ---------SMVKLAQRMTANFCTG-VCA--S---TVYK--------WN-KLNTGN----
XP_022924364.1/1-223 ---------SMVKLAQRMTANFCTG-VCA--S---TVYK--------WN-KLNTGN----
XP_023527467.1/1-221 ----------MVKLAQRMTANFCTG-VCA--S---TVYK--------WN-KLNTGN----
XP_023527463.1/1-221 ----------MVKLAQRMTANFCTG-VCA--S---TVYK--------WN-KLNTGN----
XP_023527465.1/1-221 ----------MVKLAQRMTANFCTG-VCA--S---TVYK--------WN-KLNTGN----
XP_022980232.1/1-221 ----------MVKLAQRMTANFCTG-VCA--S---TVYK--------WN-KLNTGN----
XP_022924365.1/1-222 ---------SMVKLAQRMTANFCTG-VCA--S---TVYK--------WN-KLNTGN----
XP_009412599.1/1-224 ---------SMLKLAQRMTNAFCAG-VCT--S---SAQE--------WS-KLATE-----
XP_018677084.1/1-231 ----PSGRRSMLKLAQRMTDNFCAG-VCA--S---SAHE--------WN-NLSGG-----
XP_020114860.1/1-246 ---------SMLKLAQRMTDSFCAG-VCA--S---AAQN--------WR-RLDEW-----
XP_020114857.1/1-246 ---------SMLKLAQRMTDSFCAG-VCA--S---AAQN--------WR-RLDEW-----
XP_020114859.1/1-246 ---------SMLKLAQRMTDSFCAG-VCA--S---AAQN--------WR-RLDEW-----
XP_020084803.1/1-234 ---------SMLKLAQRMTDNFCAG-VSA--S---SAHK--------WT-KLVGAAAVAA
XP_015625616.1/1-226 ---------SMLKLARRMTENFCAG-VSA--S---SARE--------WS-KLDGATG---
XP_006647677.1/1-226 ---------SMLKLARRMTENFCAG-VSA--S---SARE--------WS-KLDGATG---
NP_001105493.1/1-226 ---------SMLKLARRMTENFCAG-VSA--S---SARE--------WS-KLDGAAG---
XP_020178635.1/1-225 ---------SMLKLARRMTENFCAG-VSA--S---SARE--------WS-KLDGATG---
XP_003570085.1/1-223 ---------SMLKLARRMTDNFCAG-VSA--S---SARE--------WS-KLDGATG---
XP_020247871.1/1-226 -----------MKLSQRMVKIFCSN-ISS--AS--N-QS--------WA-ALS-ES----
XP_020247870.1/1-226 -----------MKLSQRMVKIFCSN-ISS--AS--N-QS--------WA-ALS-ES----
XP_024004764.1/1-211 ----------LMRLSQRMVRIFCLN-LNG--SY--G-R------------ALS-ES----
XP_018449374.1/1-217 ---------MLMKLSQSMVRTFCLT-VSN--SY--G-Q------------ALS-ES----
XP_013602839.1/1-215 ----------LMKLSQSMVRTYCLT-ISN--SY--G-Q------------ALS-ES----
XP_013655966.1/1-215 ----------LMKLSESMVRTYCLT-ISN--SY--G-Q------------ALS-ES----
XP_009108342.1/1-215 ----------LMKLSQSMVRTYCLT-ISN--SY--G-Q------------ALS-ES----
XP_013655964.1/1-215 ----------LMKLSESMVRTYCLT-ISN--SY--G-Q------------ALS-ES----
XP_020872468.1/1-222 --PSVEARKNLMNLSQRMVRTFCLN-ISN--SY--G-Q------------A----S----
NP_193506.2/1-221    --PSVEARKNLMKLSQRMVKTFCLN-IIN--SH--G-Q------------A----P----
XP_023633855.1/1-222 --PSVEARRNLIKLSQTMVKTFCLN-ISN--SY--G-Q------------G----S----
XP_010449572.1/1-221 --PSVAARKNLMKLSQRMVKTFCLN-ISN--SY--G-Q------------A----S----
XP_010439957.1/1-223 --PSVAARKNLMKLSQRMVKTFCLN-ISN--SY--G-Q------------A----S----
XP_010439959.1/1-223 --PSVAARKNLMKLSQRMVKTFCLN-ISN--SY--G-Q------------A----S----
XP_010439958.1/1-223 --PSVAARKNLMKLSQRMVKTFCLN-ISN--SY--G-Q------------A----S----
XP_006854056.1/1-234 ---------SLMRLAQRMMRIFCIN-MSG--SG--G-QS--------WT-TLT-GG----
XP_020529072.1/1-234 ---------SLMRLAQRMMRIFCIN-MSG--SG--G-QS--------WT-TLT-GG----
NP_199499.3/1-224    ----------IMRLSQRLVKTFCVN-IST--AY--G-QS--------WT-ALS-ET----
XP_020871346.1/1-224 ----------IMRLSQRLVKTFCVN-MST--AY--G-QS--------WT-ALS-ET----
XP_006281890.1/1-224 ----------IMRLSQRMVRTFCVN-IST--AY--G-QS--------WT-ALS-ET----
XP_010481400.1/1-224 ----------MMRLSQRMVRTFCVN-IST--AY--G-QS--------WT-ALS-ET----
XP_010494885.1/1-224 ----------MMRLSQRMVRTFCVN-IST--AY--G-QS--------WT-ALS-ET----
XP_018455882.1/1-227 ----------MMRLSQRMVKTFCVN-IST--SY--G-QS--------WT-ALS-ET----
XP_013713146.1/1-227 ----------MMRLSQRMIKTFCVN-IST--SY--G-QS--------WT-ALS-ET----
XP_013607209.1/1-227 ----------MMRLSQRMIKTFCVN-IST--SY--G-QS--------WT-ALS-ET----
XP_009114317.1/1-227 ----------MMRLSQRMMKTFCVN-IST--SY--G-QS--------WT-ALS-ET----
XP_013661958.1/1-227 ----------MMRLSQRMMKTFCVN-IST--SY--G-QS--------WT-ALS-ET----
XP_006398384.1/1-227 ----------MMRLSQRMMRTFCVN-IST--SY--G-QS--------WT-ALS-ET----
XP_024011152.1/1-227 ----------MMRLSQRMMRTFCVN-IST--SY--G-QS--------WT-ALS-ET----
XP_018482944.1/1-227 ----------MMRLSQRMLKTFCVN-IST--SN--G-QS--------WT-ALS-ET----
XP_009101472.1/1-227 ----------MMRLSQRMMKTFCVN-IST--SY--G-QS--------WT-ALS-ET----
XP_013693979.1/1-227 ----------MMRLSQRMMKTFCVN-IST--SY--G-QS--------WT-ALS-ET----
XP_013615089.1/1-227 ----------MMRLSQRMVKTFCVN-IST--SY--G-QS--------WT-ALS-ET----
XP_013704013.1/1-227 ----------MMRLSQRMVKTFCVN-IST--SY--G-QS--------WT-ALS-ET----
XP_009373477.1/1-246 ----------MMNLAQRMIRTFCVS-MST--SN--G-QS--------WT-AIS-DS----
XP_006662631.2/1-256 ----------MMKLSQRMITTFCAN-ISA--SG--S-QS--------WT-ALS-DS----
XP_015613461.1/1-259 ----------MMKLSQRMITTFCAN-ISA--SG--T-QS--------WT-ALS-DS----
NP_001337805.1/1-240 ----------MMKLSQRMITSFSAN-ISA--SG--S-QS--------WT-SLS-ET----
XP_020163828.1/1-239 ----------MMKLSQRMITAFCAN-ISA--SG--S-QS--------WT-ALS-DS----
XP_010235052.1/1-238 ----------MMRLSQRMITAFCAN-ISA--SG--S-QS--------WT-ALS-DS----
XP_025798256.1/1-238 ----------MMKLSQRMITTFCAN-ISA--SG--S-QS--------WT-SLS-ES----
XP_004983555.1/1-238 ----------MMKLSQRMITTFCAN-ISA--SG--S-QS--------WT-ALS-ES----
XP_002464880.2/1-238 ----------MMKLSQRMITTFSAN-ISA--SG--S-QS--------WT-ALS-ET----
XP_020262583.1/1-231 ----------MMKLSHRMVKTFCAN-ISS--AS--N-QS--------WT-ALS-DS----
XP_020677410.1/1-231 ----------IMKLSHRMVKTFCAG-ITS--SG--G-QS--------WT-ALS-DS----
XP_020577604.1/1-229 ------------KLSHRMVRTFYAS-ITS--SG--G-QS--------WT-ALS-DS----
XP_020577606.1/1-229 ------------KLSHRMVRTFYAS-ITS--SG--G-QS--------WT-ALS-DS----
XP_020577607.1/1-229 ------------KLSHRMVRTFYAS-ITS--SG--G-QS--------WT-ALS-DS----
XP_019709148.1/1-245 ----------MMKLSQRMIRTFCAS-ISA--SE--V-QS--------WT-ALS-DS----
XP_008803567.1/1-241 ----------MMKLSQRMIRIFCAS-ICA--SG--V-QS--------WT-ALS-DS----
XP_008803570.2/1-241 ----------MMKLSQRMIRIFCAS-ICA--SG--V-QS--------WT-ALS-DS----
XP_018681950.1/1-231 ----------MMRLSQRMITTFCTG-VYA--SG--M-QS--------WT-ALS-DS----
XP_018681951.1/1-231 ----------MMRLSQRMITTFCTG-VYA--SG--M-QS--------WT-ALS-DS----
XP_010919976.1/1-237 ----------MMKLSQRMIRTFCAN-ISA--AG--V-QS--------WT-ALS-DS----
XP_008800084.1/1-237 ----------MMKLSQRMIRTLCAN-IST--TG--V-QS--------WT-ALS-DS----
XP_018679997.1/1-229 ----------MMKLSQRMMRTFCAS-VHA--SG--M-QS--------WT-ALS-ES----
XP_009397149.1/1-229 ----------MMKLSQRMMRTFCAS-VHA--SG--M-QS--------WT-ALS-ES----
XP_024995523.1/1-235 ----------LMNLAQRMVRMFCLN-ITG--SC--G-QS--------WT-ALS-DS----
XP_023736252.1/1-233 ----------LMNLAQRMVRMFCLN-ITG--SC--G-QS--------WT-ALS-DS----
XP_023736253.1/1-233 ----------LMNLAQRMVRMFCLN-ITG--SC--G-QS--------WT-ALS-DS----
XP_022022041.1/1-231 ----------LMNVAQRMVRMFCLH-ITG--SC--G-QS--------WT-ALS-DS----
XP_021969857.1/1-226 ----------LMNVAQRMVRMFSLH-ITG--SC--G-QS--------WT-ALS-DS----
XP_010245213.1/1-236 ----------MMKLAHRMIRTFCIN-VST--SG--G-HS--------WT-ALS-DS----
XP_010245210.1/1-236 ----------MMKLAHRMIRTFCIN-VST--SG--G-HS--------WT-ALS-DS----
XP_010276832.1/1-235 ----------LMKLAQRMIKTFCIS-IST--TG--G-QS--------WT-ALS-DT----
XP_010276837.1/1-235 ----------LMKLAQRMIKTFCIS-IST--TG--G-QS--------WT-ALS-DT----
XP_019155823.1/1-230 ----------MMNLAQRMIKIFCVN-IST--SM--G-QS--------WT-SLS-DS----
XP_022881662.1/1-238 ----------LMNLAQRMIRTFCLN-IST--CC--G-QS--------WT-AIS-DS----
XP_022877113.1/1-234 ----------LMNLAQRMIRTFCLN-IST--CC--G-QS--------WT-ALS-DS----
XP_016450789.1/1-246 ---------SLMNLAQRMIKTFCMN-IST--SC--G-QS--------WT-ALS-DS----
XP_009597691.1/1-246 ---------SLMNLAQRMIKTFCMN-IST--SC--G-QS--------WT-ALS-DS----
XP_009786380.1/1-246 ---------SLMNLAQRMIKTFCMN-IST--SC--G-QS--------WT-ALS-DS----
XP_016494823.1/1-246 ---------SLMNLAQRMIKTFCMN-IST--SC--G-QS--------WT-ALS-DS----
XP_016494822.1/1-246 ---------SLMNLAQRMIKTFCMN-IST--SC--G-QS--------WT-ALS-DS----
XP_019230103.1/1-246 ---------SLMNLAQRMIKTFCMN-IST--SC--G-QS--------WT-ALS-DS----
XP_016573090.1/1-250 ---------SLMNLSQRMIKTFCMN-IST--SC--G-QS--------WT-ALS-DS----
XP_016573102.1/1-250 ---------SLMNLSQRMIKTFCMN-IST--SC--G-QS--------WT-ALS-DS----
XP_006352911.1/1-250 ---------SLMNLAQRMIKTFCMN-IST--CC--G-QS--------WT-ALS-DS----
XP_015166604.1/1-250 ---------SLMNLAQRMIKTFCMN-IST--CC--G-QS--------WT-ALS-DS----
XP_015085651.1/1-250 ---------SLMNLAQRMIKTFCMN-IST--CC--G-QS--------WT-ALS-DS----
XP_015085654.1/1-250 ---------SLMNLAQRMIKTFCMN-IST--CC--G-QS--------WT-ALS-DS----
XP_004245919.1/1-250 ---------SLMNLAQRMIKTFCMN-IST--CC--G-QS--------WT-ALS-DS----
XP_025888165.1/1-250 ---------SLMNLAQRMIKTFCMN-IST--CC--G-QS--------WT-ALS-DS----
XP_011070327.1/1-236 ---------SLMNLAQRMTRTFCLN-IST--SY--G-QS--------WT-ALS-DC----
XP_011070329.1/1-236 ---------SLMNLAQRMTRTFCLN-IST--SY--G-QS--------WT-ALS-DC----
XP_012843107.1/1-236 ----------LMNLAQRMIRTFSLN-IST--CY--G-QS--------WT-ALS-DS----
XP_019423575.1/1-234 ----------LMSLAQRMIRTFSLN-MST--SS--G-QS--------WT-AIS-DS----
XP_019423571.1/1-234 ----------LMSLAQRMIRTFSLN-MST--SS--G-QS--------WT-AIS-DS----
XP_019435331.1/1-234 ----------LMSLAQRMTRTLSLN-MST--SS--G-QS--------WT-AIS-DS----
XP_003612811.1/1-239 ---------SLMKLANRMIKTFSLN-MTT--CG--G-QS--------WT-AIS-DS----
XP_020978415.1/1-227 ----------LMKLAQRMIRTFSLN-MST--SS--G-QS--------WT-AIS-DS----
XP_015963259.1/1-227 ----------LMKLAQRMIRTFSLN-MST--SS--G-QS--------WT-AIS-DS----
XP_025697880.1/1-227 ----------LMKLAQRMIRTFSLN-MST--SS--G-QS--------WT-AIS-DS----
XP_025697882.1/1-227 ----------LMKLAQRMIRTFSLN-MST--SS--G-QS--------WT-AIS-DS----
XP_025697881.1/1-227 ----------LMKLAQRMIRTFSLN-MST--SS--G-QS--------WT-AIS-DS----
XP_020217931.1/1-233 ----------MMKLAQRMIKTFGLN-MST--SG--G-QS--------WT-AIS-DS----
XP_007158253.1/1-232 ----------LMKLAQRMIKTFGLN-MST--SG--G-QS--------WT-AIS-DS----
XP_017427665.1/1-232 ----------LMKLAQRMIKTFGLN-MST--SG--G-QS--------WT-AIS-DS----
XP_017427666.1/1-232 ----------LMKLAQRMIKTFGLN-MST--SG--G-QS--------WT-AIS-DS----
XP_014521371.1/1-232 ----------LMKLAQRMIKTFGLN-MST--SG--G-QS--------WT-AIS-DS----
XP_014521373.1/1-232 ----------LMKLAQRMIKTFGLN-MST--SG--G-QS--------WT-AIS-DS----
XP_006587604.1/1-242 ----------LMKLAQRMIKTFSLN-MST--SG--G-QS--------WT-AIS-DS----
NP_001352051.1/1-233 ----------LMKLAQRMIKTFSLN-MST--SG--G-QS--------WT-AIS-DS----
XP_018840870.1/1-239 ----------LMKLAQRMVRTFCVN-IST--SS--G-LI--------WT-ALS-DS----
XP_018852246.1/1-240 ----------LMKLAQRMIRTFCIN-TST--SS--G-LI--------WT-ALS-DY----
XP_010053633.1/1-234 ----------LMRLAQRMIRTFSTN-IST--SG--G-QS--------WT-ALS-DS----
XP_021675948.1/1-236 ----------LMRLAQRMIRTFSVN-VST--CG--G-QS--------WT-ALS-DS----
XP_012081064.1/1-228 ----------LMRLAQRMIRTFCVN-VST--CT--G-QS--------WT-ALS-DS----
XP_011028126.1/1-235 ----------MMRLAQRMIRIFSLN-ISS--SS--G-QS--------WT-GLS-DS----
XP_002303433.2/1-235 ----------MMRLAQRMIRIFSLN-ISS--SS--G-QS--------WT-GLS-DS----
XP_015897558.1/1-247 ----------LMKLAQRMIRTFCVN-MST--SS--N-QS--------WT-ALS-DS----
XP_002277673.1/1-238 ----------LMNLAQRMIRTFSVN-IST--SS--G-QS--------WT-ALS-DS----
XP_022996123.1/1-236 ----------LMKLAQRMTRTFSLN-IST--SG--G-QS--------WT-ALS-DS----
XP_023534245.1/1-236 ----------LMKLAQRMTRTFSLN-IST--SG--G-QS--------WT-ALS-DS----
XP_022931244.1/1-236 ----------LMKLAQRMTRTFSLN-IST--SG--G-QS--------WT-ALS-DS----
XP_022931242.1/1-236 ----------LMKLAQRMTRTFSLN-IST--SG--G-QS--------WT-ALS-DS----
XP_022159691.1/1-235 ----------LMKLAQRMIRTFSLN-IST--SG--G-QS--------WT-ALS-DS----
XP_011652640.1/1-232 ----------LMKLAQRMIRTFSVN-IST--SG--G-QS--------WT-ALS-DS----
XP_011652639.1/1-232 ----------LMKLAQRMIRTFSVN-IST--SG--G-QS--------WT-ALS-DS----
XP_008466007.1/1-232 ----------LMKLAQRMIRTFSVN-IST--SG--G-QS--------WT-ALS-DS----
XP_023511589.1/1-230 ----------LMKLAQRMIRTFSVN-IST--SG--G-QS--------WT-ALS-ES----
XP_022963906.1/1-230 ----------LMKLAQRMIRTFSVN-IST--SG--G-QS--------WT-ALS-ES----
XP_023511591.1/1-230 ----------LMKLAQRMIRTFSVN-IST--SG--G-QS--------WT-ALS-ES----
XP_022963908.1/1-230 ----------LMKLAQRMIRTFSVN-IST--SG--G-QS--------WT-ALS-ES----
XP_022967184.1/1-230 ----------LMKLAQRMIRTFSVN-IST--SG--G-QS--------WT-ALS-ES----
XP_022967186.1/1-230 ----------LMKLAQRMIRTFSVN-IST--SG--G-QS--------WT-ALS-ES----
XP_010518669.1/1-225 ----------LMRLAQRMIRTFCVN-IST--SY--G-QS--------WT-ALS-ES----
XP_010677365.1/1-235 -----------MKLAQRMIRTFCVN-IST--SG--G-QS--------WT-ALS-DS----
XP_010677364.1/1-235 -----------MKLAQRMIRTFCVN-IST--SG--G-QS--------WT-ALS-DS----
XP_021852084.1/1-226 -----------MKLSQRMIRTFSVN-IST--SG--G-QS--------WT-ALS-ES----
XP_021852076.1/1-226 -----------MKLSQRMIRTFSVN-IST--SG--G-QS--------WT-ALS-ES----
XP_021765591.1/1-233 -----------MKLAQRMIRTLSVN-IST--SG--G-QS--------WT-ALS-ES----
XP_021768254.1/1-233 -----------MKLAQRMIRTLSVN-IST--SG--G-QS--------WT-ALS-ES----
XP_017232690.1/1-221 ----------LMNLAQRMIRTFCVS-LSS--SS--G-QS--------WT-ALS-DC----
XP_017232692.1/1-221 ----------LMNLAQRMIRTFCVS-LSS--SS--G-QS--------WT-ALS-DC----
XP_024021697.1/1-248 ----------MMNLAQRMIKTFCAN-IST--SG--G-QS--------WT-ALS-DS----
XP_010096931.1/1-248 ----------MMNLAQRMIKTFCAN-IST--SG--G-QS--------WT-ALS-DS----
XP_024021696.1/1-248 ----------MMNLAQRMIKTFCAN-IST--SG--G-QS--------WT-ALS-DS----
XP_017621310.1/1-230 ----------LMRLSQRMIRTFCVN-ISS--CS--G-QV--------WT-AVP-DS----
XP_016731150.1/1-230 ----------LMRLSQRMIRTFCVN-ISS--CS--G-QV--------WT-AVP-DS----
XP_012472199.1/1-230 ----------LMRLSQRMIRTFCVN-ISS--CS--G-QV--------WT-AVP-DS----
XP_016727305.1/1-230 ----------LMRLSQRMIRTFCVN-ISS--CS--G-QV--------WT-AVP-DS----
XP_016731145.1/1-230 ----------LMRLSQRMIRTFCVN-ISS--CS--G-QV--------WT-AVP-DS----
XP_012472200.1/1-230 ----------LMRLSQRMIRTFCVN-ISS--CS--G-QV--------WT-AVP-DS----
XP_012472201.1/1-230 ----------LMRLSQRMIRTFCVN-ISS--CS--G-QV--------WT-AVP-DS----
NP_001314348.1/1-230 ----------LMRLSQRMIRTFCVN-ISS--CS--G-QV--------WT-AVP-DS----
XP_016727300.1/1-230 ----------LMRLSQRMIRTFCVN-ISS--CS--G-QV--------WT-AVP-DS----
XP_022738572.1/1-235 ----------LMRLAQRMIRTFCVN-IST--SS--G-RL--------WT-ALP-DS----
XP_021297648.1/1-235 ----------LMRLAQRLIRTFCVN-IST--SS--G-QL--------WT-ALP-DS----
XP_017973778.1/1-235 ----------LMRLAQRMIRTFCVN-IST--SS--G-QL--------WT-ALP-DS----
XP_017973780.1/1-235 ----------LMRLAQRMIRTFCVN-IST--SS--G-QL--------WT-ALP-DS----
XP_022717458.1/1-235 ----------LMRLAQRMIRTFCVN-IST--SS--G-QL--------WT-ALP-DS----
XP_022717453.1/1-235 ----------LMRLAQRMIRTFCVN-IST--SS--G-QL--------WT-ALP-DS----
XP_022717437.1/1-235 ----------LMRLAQRMIRTFCVN-IST--SS--G-QL--------WT-ALP-DS----
XP_022756982.1/1-232 ----------LMRLAQRMIRTFCVN-IST--SS--G-QL--------WT-ALP-DS----
XP_004300207.1/1-230 ----------LMRLAQRMIRTFSVN-MST--SG--G-QA--------WT-ALS-DS----
XP_024169578.1/1-230 ----------LMRLAQRMIRTFSVN-MST--SG--G-QA--------WT-ALS-DS----
XP_008238183.1/1-249 ----------LMKLSQRMIRTFCVN-MST--SN--G-QS--------WT-ALS-DS----
XP_016651445.1/1-249 ----------LMKLSQRMIRTFCVN-MST--SN--G-QS--------WT-ALS-DS----
XP_020418924.1/1-249 ----------LMKLSQRMIRTFCVN-MST--SN--G-QS--------WT-ALS-DS----
XP_021804146.1/1-249 ----------LMKLSQRMIRTFCVN-MST--SN--G-QS--------WT-ALS-DS----
XP_008238184.1/1-248 ----------LMKLSQRMIRTFCVN-MST--SN--G-QS--------WT-ALS-DS----
XP_016651446.1/1-248 ----------LMKLSQRMIRTFCVN-MST--SN--G-QS--------WT-ALS-DS----
XP_007209851.1/1-248 ----------LMKLSQRMIRTFCVN-MST--SN--G-QS--------WT-ALS-DS----
XP_021804154.1/1-248 ----------LMKLSQRMIRTFCVN-MST--SN--G-QS--------WT-ALS-DS----
XP_017179076.1/1-239 ----------MMKLAQRMLRTVCVN-MST--SN--G-LS--------WT-DLS-DS----
XP_008356445.1/1-246 ----------MMKLAQRMIRTFCVS-MST--SN--G-QS--------WT-AIS-DS----
XP_008356446.1/1-240 ----------MMKLAQRMIRTFCVS-MST--SN--G-QS--------WT-AIS-DS----
XP_008356447.1/1-239 ----------MMKLAQRMIRTFCVS-MST--SN--G-QS--------WT-AIS-DS----
XP_009373485.1/1-239 ----------MMNLAQRMIRTFCVS-MST--SN--G-QS--------WT-AIS-DS----
XP_009373484.1/1-240 ----------MMNLAQRMIRTFCVS-MST--SN--G-QS--------WT-AIS-DS----
XP_004512486.1/1-245 ----------LMKLAHRMIKTFSLN-MTT--CG--G-QS--------WT-AIS-DL----
XP_023898631.1/1-242 ----------LMKLAQRMIRTFCVN-ISS--SS--G-LM--------WT-ALS-DS----
XP_024465375.1/1-238 ----------MMRLAQRMIRTFSLN-IST--SS--G-QS--------WT-ALP-DS----
XP_011018502.1/1-238 ----------MMRLAQRMIRTFSLN-IST--SS--G-QS--------WT-ALP-DS----
XP_011018503.1/1-238 ----------MMRLAQRMIRTFSLN-IST--SS--G-QS--------WT-ALP-DS----
XP_021893615.1/1-233 ----------LMRLAQRMIRTFSVN-MSC--CS--G-QS--------WT-ALS-ES----
XP_024952893.1/1-247 ----------LMRLSQRMIRTFSVN-IST--AS--G-QS--------WT-ALS-DS----
XP_006471522.1/1-248 ----------LMRLSQRMIRTFSVN-IST--AS--G-QS--------WT-ALS-DS----
XP_006432745.2/1-248 ----------LMRLSQRMIRTFSVN-IST--AS--G-QS--------WT-ALS-DS----
XP_021640807.1/1-241 ----------LMRLAQRMIRTFCVN-VST--CG--G-QS--------WT-ALS-ES----
XP_002518976.1/1-243 ----------LMRLAQRMIRTFCMN-IST--CS--G-QS--------WT-ALS-DS----
XP_021598537.1/1-237 ----------LMRLAQRMIRTFSMN-VST--CG--G-QS--------WT-ALS-DS----
XP_021598535.1/1-239 ----------LMRLAQRMIRTFSMN-VST--CG--G-QS--------WT-ALS-DS----
XP_006659280.1/1-226 -----------LHLVKQMTSSFTA--LCA--SM--S-KA---------------------
XP_025801379.1/1-254 --------AGMLKLAQQMMASFYAT-VSG--PI--A-QSQATISINDWF-GSI-GT----
XP_023156326.1/1-229 ---------GVLELAQRMVADFYSA-VSG--PV--T-QPS--SSIDEWY-GSA-GA----
NP_001349156.1/1-229 ---------GVLELAQRMVADFYSA-VSG--PV--T-QPS--SSIDEWY-GSA-GA----
XP_021310588.1/1-246 ---------GILELAKRMMAVFYSA-VSG--PV--T-QTS-TSNLYEWP-ASA-GT----
XP_020404516.1/1-224 ----------LLELAQRMMAIFYKT-TSG--LP--T-VEP-GNIVTRWG-RGCMGT----
XP_022684239.1/1-229 ---------GVLDLAQRMMVSFYTA-VSK--PV--T-PGP-SNIVNEWH-GSC-DI----
XP_020404892.1/1-233 -----------MELARRMTASFYAA-VSG--PV--T-VPA-TSSVDEWR-VSS-GS----
XP_022684238.1/1-237 ---------GVLELAQRMTASFYAA-ISG--PV--T-IAS-TNITNQWC-VSS-GT----
XP_025825251.1/1-229 ---------GVLELARRMKESFYAA-VSG--PV--T-IAS-TNIVDQWC-VSS-GT----
XP_020183090.1/1-242 -----------LELARRMTESFYVA-ISA--PR----GEA-WRKVADWR-GR--------
XP_020194819.1/1-240 -----------LEVAHRMTLKFYEA-ICG--PG----TQP-WTSVDERR-GSC-GV----
XP_024314575.1/1-244 ----PAGKKGILELAQRMTMNFYVA-MCG--LP--A-GQP-W---NDWN-GGI-GV----
XP_024314342.1/1-235 --------------SQQMKMNFYKA-MCD--SA----GRS-WSTVDEWA-GDC-GV----
XP_024959674.1/1-192 --------SSLLRLAHRMSQTCISN-MNV--ST--K-KN--W------N-LVH-SL----
XP_019196338.1/1-215 ----------MLKLSQRMVTCYARN-LIP--CK--E-NP--W------L-PLP-IP----
XP_006646400.1/1-226 ----------LMKLADDLLASYASS-VSA--TG--A-GT--W------T-VVI-GA----
XP_015632244.1/1-226 ----------LMKLADDLLASFAGG-IAA--TG--G-GT--W------T-VVI-GA----
XP_024314625.1/1-231 ----------LMKLADSLVVSYARS-MAN--LP--V-GA--W------T-TLC-GS----
XP_020164122.1/1-228 ----------LMKIADGLLADYASG-IAA--VP--G-SG--W------T-ILR-GA----
XP_025814677.1/1-221 ---------AIMKLADNLLANYTGS-IAG--LP--A-DD--W------S-VQC-GE----
XP_022682466.1/1-225 ---------AVMKLADNLLADYTSS-ISG--LP--V-DA--W------S-VQC-GE----
XP_002456467.1/1-226 ----------IMKLADNLLASFTSS-VTA--YP--E-DA--W------N-FQC-GL----
XP_008674944.1/1-228 ----------IMKLADNLLADYTSS-VAA--FP--D-DA--W------T-VQC-GV----
XP_010523539.1/1-228 ----------LVNLSRRMVSVFCAG-VCG--IT--G-QN--W------K-PVG-TD----
XP_020873847.1/1-203 ----------LLEVSRHMVHLFCSG-TCG--VI--G-YQ--W------R-RLG-A-----
NP_001190248.1/1-203 ----------LLNLSSFMVNVFCSG-VCG--IT--G-QR--W------N-RLN-TV----
NP_196343.2/1-203    ----------LLNLSSFMVNVFCSG-VCG--IT--G-QR--W------N-RLN-TV----
XP_020876233.1/1-195 ----------LLNLSSFMVNAFCTG-VCG--IT--G-QS--W------N-RFD-TV----
XP_010423235.1/1-188 ----------LLNLSTWMVSIFCTG-VCG--TT--R-QK--W------K-PLS-TI----
XP_010452682.1/1-218 ----------LLNLSTWMVSIFCTG-VCG--TT--R-QK--W------K-RLS-TI----
XP_010491330.1/1-188 ----------LLNLSTWMVFIFCTG-VCG--TT--R-QK--W------K-RLD-TI----
XP_006413300.2/1-224 ------------KLAQRMTVNYYSG-ITD--SS--P-RK--W------R-IIN-VE----
XP_022008499.1/1-216 ----------LRSLAQRMRRMFCAT-VCL--TG--W-ER--W------N-LVA--N----
XP_022008502.1/1-215 ----------LRSLAQRMRRMFCAT-VCL--TG--W-ER--W------N-LVA--N----
XP_022007635.1/1-226 ---------SLKALAQRMTSTFCGG-ICL--TD--G-QQ--W------D-SVV-DH----
XP_022008501.1/1-224 ---------SLIGLAKRMTSIYCGG-VCL--TD--G-QR--W------D-LVA-DH----
XP_021986984.1/1-226 ---------SLRGLAQRMMRIFCGG-VCL--TD--G-QQ--W------D-LVA-DH----
XP_021996009.1/1-224 ---------SLRGLAQRMMSIFCGG-VCL--TD--G-QQ--W------D-LVA-DH----
XP_021996011.1/1-224 ---------SLRGLAQRMMSIFCGG-VCL--TD--G-QQ--W------D-LVA-DH----
XP_022026050.1/1-224 ---------SLRGLAQRMMSIFCGG-VCL--TD--G-QQ--W------D-LVA-DH----
XP_022026083.1/1-224 ---------SLRGLAQRMMSIFCGG-VCL--TD--G-QQ--W------D-LVA-DH----
XP_021722389.1/1-209 ----------LLRLAERMRREFYGA-TSA--TE--G-NG--Y------K-PIS-IK----
XP_021847315.1/1-219 ---------GMLRLAERMRREFYGA-TSG--TK--G-NG--Y------K-PIS-IK----
XP_020224300.1/1-216 ------------KFSDRMVQGFCRV-LHE--SR--H-EG--F-------------I----
XP_021765248.1/1-224 ---------SMMMLSRRMIKKYCST-FSL--FE--D-AN--T-------------Y----
XP_004510020.1/1-219 -----------MNLSNRMVHNFCRD-LNT--CG--S-GH--I------P-MQL-FG----
XP_004487953.1/1-231 -----------MKLSNEMVQNFCRV-LNT--QS--S-V------------LHI-FE----
XP_008244376.1/1-218 -----------LKFSARMVKSFFMD-IGA--SR--E-NK--W------M-PFP-MS----
XP_008244380.1/1-218 -----------LKFSARMVKSFFMD-IGA--SR--E-NK--W------M-PFP-MS----
XP_012569676.1/1-224 -----------MNFANRMVKMFCEC-LTM--SN--Q-MK--F------T-QKT-LN----
XP_019418393.1/1-229 ---------GIIKVCHRMVKIFCEM-LNM--SK--L-QQ--V------I-TRK-T-----
XP_019440118.1/1-223 ---------SLIKLSHRMMKNFTEM-LSM--SK--K-SS--Q--------QII-M-----
XP_014517363.2/1-226 ----------MIKFSHGMLKNFYGI-LNI--SS--K-TE--C------L-EHL-AE----
XP_012569675.1/1-231 ---------SIMQLSNRMVKDLCGV-LSM--LT--K--P--E------F-PEN-FI----
XP_004486570.1/1-223 -----------MQLANEVVKDFSQI-LAM--SS--K-LD--N------I-PKY-FI----
XP_003594569.3/1-220 -----------MKLAHQVLKEFSKN-LTM--SS--K--S--D------L-PQY-IA----
XP_024632692.1/1-220 -----------MKLAHQVLKEFSKN-LTM--SS--K--S--D------L-PQY-IA----
XP_025887127.1/1-229 ----------MMKSAVDPTDMFCTG-VCG--T---K-HN--------WE-LIQ-PA----
XP_015078684.1/1-230 ---------GIRKMAQRMTDMFCTG-VCG--T---K-HN--------WE-IIQ-PA----
XP_025887126.1/1-230 ---------GIRKMAQRMTDMFCTG-VCG--T---K-HN--------WE-LIQ-PA----
XP_015078401.1/1-220 ------------KLAQRMTHNFCAG-VCG--T---T-HQ--------WQ-IVQ-LA----
XP_015158871.1/1-217 -------------LAQRMTHNFCAG-VCG--T---T-HQ--------WQ-IIQ-LA----
XP_015078778.1/1-220 ---------GMGTLSQRMTRSFCAV-ICA--T---S-HK--------WI-TIQ-KE----
XP_010321992.1/1-216 -------------LSQRMTRSFCAV-ICA--T---S-HK--------WI-TIQ-KE----
XP_004240735.1/1-216 -------------LSQRMTRSFCAV-ICA--T---S-HK--------WI-TIQ-KE----
XP_015169793.1/1-213 ---------GIRMLAQSMTRKFCAA-VCA--T---T-HK--------WE-IVQ-LE----
XP_006285631.1/1-212 ----------LMKLTKRMTRNYYTG-MTS--ST--V-GK--------WE-MIE-VE----
XP_023635531.1/1-198 ------------------------G-MTS--ST--V-GK--------WE-MIE-VE----
NP_567722.1/1-217    ------------KLAQRMTLNYYRG-ITS--PS--V-DK--------WQ-KIQ-VE----
XP_020873337.1/1-219 ------------KLAQRMTLNYYTG-ITS--SS--G-NK--------WE-KIQ-VE----
XP_020873338.1/1-219 ------------KLAQRMTLNYYTG-ITS--SS--G-NK--------WE-KIQ-VE----
XP_023000325.1/1-225 -----------LRLAQRMTSSIYQA-MGA--SS--S-HT--------WT-KVQ-SK----
XP_023000326.1/1-214 -----------LRLAQRMTSSIYQA-MGA--SS--S-HT--------WT-KVQ-SK----
XP_022963997.1/1-214 -----------LRLAQRMTSSIYQA-MGA--SS--S-HT--------WT-KVQ-SK----
XP_023513617.1/1-208 -----------LRLAQRMTSSVYQA-MGA--SS--S-HT--------WT-KVQ-SK----
XP_021312124.1/1-225 -----------LKLAQRMTSSLYRA-IGA--SR--V-RS--------WR-KRL-NY----
XP_012701492.1/1-243 ----PAGRRSVLKLAHRMTSSLCRV-IGG--SR--D-LA--------WS-RPA-SN----
XP_025815339.1/1-229 -----------LKLAHRMASSLCRV-IGG--SR--D-LA--------WS-GVA-DAS---
XP_015644459.1/1-237 -----------LKLAHRMTSSLCRT-TGG--SC--D-MA--------WR-RAP-KG----
XP_024314016.1/1-228 -----------LKLAHRMTASLCRS-IGG--SR--G-LA--------WS-RVT-RA----
XP_020168333.1/1-225 -----------LKLAHRMTSSLCRV-MGG--SR--G-LA--------WS-RAP-RA----
XP_013589242.1/1-206 ------------KLAQRMTINYYLC-ITA--SP--A-SI--------WK-SND-SE----
XP_022548456.1/1-204 ------------KLAQRMTINYYLC-ITA--SP--A-SI--------WK-SND-SE----
XP_024632691.1/1-227 ------------KLADRMVKMFCEC-LTM--PG--Q-VE--------LN-HLT-LD----
XP_017256377.1/1-220 ---------SLLQLSQRISRSYNEA-VRG--ST--E-SH--------WQ-PLP-TT----
XP_009632057.1/1-224 ----------LMKLAERMVRSYNAI-FSS--CN--E-NQ--------WM-PLR-IQ----
XP_016502932.1/1-224 ----------LMKLAERMVRSYNAI-FSS--CN--E-NQ--------WM-PLR-IQ----
XP_019258997.1/1-221 ----------LVKLAERMVRSYNAI-FSS--CS--E-NQ--------WM-PLR-IQ----
XP_009792862.1/1-224 ----------LMKLAERMVRSYNAI-FSS--CN--E-NQ--------WM-PLK-IQ----
XP_016544784.1/1-213 ----------LMKLAERMIKSYDEI-FSS--CD--E-NQ--------WM-PLS-IH----
XP_015089689.1/1-213 ----------LMKLAERTIKSFNSI-YSS--CN--E-NQ--------WM-PLS-IH----
XP_025883585.1/1-213 ----------LMKLAERTIKSFNSI-YSS--CN--E-NQ--------WM-PLS-IQ----
XP_011095790.1/1-221 ---------GLLKLAQRMVRQFNSN-ICS--NV--E-SV--------WR-PLP-VP----
XP_020553953.1/1-221 ---------GLLKLAQRMVRQFNSN-ICS--NV--E-SV--------WR-PLP-VP----
XP_022857758.1/1-208 ---------SLLNLAERMVRSFNAN-ICS--NQ--E-NK--------WR-QVP-IE----
XP_004487739.1/1-224 -----------LRLADRMMRSFCAN-LT----A--S-NP--------WM-RLA-PF----
XP_003596663.1/1-221 -----------LKLADRMMRRFCAN-LGS--TT--K-NP--------WI-RLA-PL----
XP_003596665.1/1-227 ---------SLLKLAERMRRRFCAN-LSS--TT--N-NP--------WM-RLD-PV----
XP_020201972.1/1-216 --------ASLMKLADRMMKMFCAN-VSN--TV--S-NP--------WM-KID-GY----
XP_025980734.1/1-220 -----------LKLGDRMMKTFCAN-VSA--TA--D-NP--------WM-KIT-SF----
XP_007149715.1/1-218 ---------SLLKLADRMMKTFCRN-MSS--TA--D-NP--------WT-QMA-------
XP_017423636.1/1-218 ---------SLLQLADRMMKTFCGN-MSS--TA--D-NP--------WT-QMA-------
XP_022631965.1/1-218 ---------SLLQLADRMMKTFCGN-VSS--TA--D-NP--------WT-QMA-------
XP_014522034.2/1-218 ---------SLLQLADRMMKTFCGN-VSS--TA--D-NP--------WT-QMA-------
XP_025669173.1/1-223 -----------LNLADRLVRSFCGD-INA--SM--T-NQ--------WM-PLI-SS----
XP_020963786.1/1-223 -----------LNLADRLVRSFCGD-INA--SM--T-NQ--------WM-PLI-SS----
XP_025614692.1/1-223 -----------LNLADRLVRTFCGD-INA--SM--T-NQ--------WM-PLI-SS----
XP_015936351.1/1-223 -----------LNLADRLVRTFCGD-INA--SM--T-NQ--------WM-PLI-SS----
XP_019451675.1/1-226 ---------SLLNVADRMMRTFVSD-INT--ST--R-NS--------WI-QIP-II----
XP_019465428.1/1-219 -----------LNMGDRMMRTFCSD-INA--ST--R-NP--------WK-QIP-SF----
XP_004295798.2/1-220 ---------SLLKLTERMMRSFVAD-ISA--ST--E-NK--------WR-TLP-VF----
XP_021807338.1/1-219 -----------LKFSERMVKSFFMD-ISA--SR--E-NK--------WM-PFP-VS----
XP_021807340.1/1-219 -----------LKFSERMVKSFFMD-ISA--SR--E-NK--------WM-PFP-VS----
XP_020417197.1/1-219 -----------LKFSERMVKSFFMD-ISA--SR--E-NK--------WM-PFP-VS----
XP_020417200.1/1-219 -----------LKFSERMVKSFFMD-ISA--SR--E-NK--------WM-PFP-VS----
XP_009333923.1/1-206 ---------SLLKLSERMVRSFYIDNISG--SA--E-NK--------WM-PLP-LGW---
XP_017184363.1/1-206 ---------SLLKLSERMVRSFYIDNIXG--SA--E-NK--------WM-PLP-LGW---
XP_021892911.1/1-224 ----PAGQTSILKLAERMLKFFCHD-INA--SA--L-NP--------WK-ALP-IE----
XP_002278971.2/1-210 -----------LCLAERMMRKFWAN-LSD--SS--E-NT--------WR-PVP-LK----
XP_010644412.1/1-211 ----------LLTLADRMMRSFWRN-LSA--SR--K-NQ--------WM-TVP-LS----
XP_024450660.1/1-210 -----------LRLARRMMRSFYHD-NSA--ST--D-NF--------WV-RIH-LC----
XP_002522867.2/1-214 ----------LIMLAERMMTKFVLD-LSG--ST--N-NL--------WM-PFP-VT----
XP_020534605.1/1-220 ----------LLRLTERMMRSFCVD-ISA--ST--S-NF--------WM-PLP-IS----
XP_021685850.1/1-217 ----------LLRLAERMMRSFCTD-LSA--CS--H-NL--------WM-PLP-VS----
XP_021594464.1/1-220 ----------LLRLAERMMRSFCAD-FSA--CS--N-NL--------WI-PMP-VS----
XP_018731177.1/1-223 ----------LLKLAERMMRTYLKE-VNW--ST--S-NK--------WS-PLL-AT----
XP_024018593.1/1-217 ---------SLLKLSERMVRRFCSE-LSA--SA--T-NK--------WR-ALP-RS----
XP_022139420.1/1-219 -----------LKLAERMTRSFYSN-VST--SK--D-SL--------WM-KIP-IS----
XP_022981426.1/1-220 -----------LKLAERMTRTFYKV-VSG--SK--E-NP--------WM-KIP-IP----
XP_022981427.1/1-220 -----------LKLAERMTRTFYKV-VSG--SK--E-NP--------WM-KIP-IP----
XP_022941489.1/1-220 -----------LKLAERMTRTFYNV-VSG--SK--E-NP--------WM-KIP-IP----
XP_022941488.1/1-220 -----------LKLAERMTRTFYNV-VSG--SK--E-NP--------WM-KIP-IP----
XP_023525599.1/1-220 -----------LKLAERMTRTFYNV-VSG--SK--E-NP--------WM-KIP-IP----
XP_023525597.1/1-220 -----------LKLAERMTRTFYNV-VSG--SK--E-NP--------WM-KIP-IP----
XP_008454681.2/1-220 -----------LKLAERMTKSFYRN-VSS--SK--E-NP--------WV-KIP-FP----
XP_011648553.1/1-223 --------SSVLKLAERMTKSFYRN-VSI--SK--E-NP--------WI-KIP-FP----
XP_018822340.1/1-211 ---------SLVKLSERMMRNFCAD-ISA--ST--T-NI--------WT-PLH-IL----
XP_018822367.1/1-211 ---------SLVKLSERMMRNFCAD-ISA--ST--T-NI--------WT-PLH-IL----
XP_023876961.1/1-210 ---------GLLKLSERMMRTFCID-VCA--SA--S-NL--------WM-PIP-RY----
XP_023876962.1/1-210 ---------GLLKLSERMMRTFCID-VCA--SA--S-NL--------WM-PIP-RY----
XP_024034242.1/1-215 ---------SLLKLAERMTRSFWGD-ISA--CN--E-NQ--------WM-PLP-IR----
XP_015381349.2/1-215 ---------SLLKLAERMTRSFWGD-ISA--CN--E-NQ--------WM-PLP-IR----
XP_022717683.1/1-216 ---------SLLKLAERMTRNFFMN-ISS--CT--Q-NI--------WM-QLP-KN----
XP_016729588.1/1-216 ---------SLLKLAEKMTKNFFNN-INS--CS--E-NV--------WS-GLP-QN----
XP_016729589.1/1-216 ---------SLLKLAEKMTKNFFNN-INS--CS--E-NV--------WS-GLP-QN----
XP_016702140.1/1-208 -----------------MTKNFFNN-INS--CS--E-NV--------WS-GLP-QN----
XP_012460158.1/1-216 ---------SLLKLAEKMTKNFFNN-INS--CS--E-NV--------WS-GLP-QN----
XP_012460145.1/1-216 ---------SLLKLAEKMTKNFFNN-INS--CS--E-NV--------WS-GLP-QN----
XP_017245631.1/1-197 ----------LMKLSQRMINGFCSS-INP--AD--G-QQDMISKIDDLE-----------
XP_004956045.3/1-247 ---------GILKLAQQMMASFYTA-VSG--PV--T-QTQATSNINEWF-GSM-GT----
XP_014660176.1/1-240 ---------GIMELAQRMMASFYSA-VSG--PV--T-QP--SSSIDEWH-GSN-GT----
XP_017239609.1/1-213 ---------SLLKLADKMVNNFIFG-IAG--LN--T-GR--------WT-QVH-GN----
XP_023740378.1/1-206 --------SCLKGLARRMTSNFCAG-VCL--TG--G-QR--------WN-LVS-DA----
XP_020095129.1/1-235 ----------LLKQAENMMKSFCGA-ISG--SP--E-HE--------WT-VEA-GI----
XP_020258373.1/1-212 -----------LKLAERMVLSFYAG-LAC--SL--A-HG--------WV-NEF-GD----
XP_008812148.1/1-175 ---------SILRLVERMVVGFCGG-VSG--SR--A-QD--------WS-HIS-GV----
XP_010930674.1/1-230 ----------ILRLVERMVVGFCGG-VSG--SR--A-QG--------WS-HIS-GI----
XP_010931877.1/1-230 ----------ILRLVERMVVGFCGG-VSG--SR--A-QG--------WS-HIS-GI----
XP_009380061.1/1-225 ---------SMMKLAHRLVMDFCAS-F--SASV--G-NK--------W-TMLS-GI----
XP_010939971.1/1-229 ---------SMMKLAQRMANNFCAS-I--TASNNHG-HK--------W-TNPS-GL----
XP_019710626.1/1-218 ---------SMMKLAQRMANNFCAS-I--TASNNHG-HK--------W-TNPS-GL----
XP_008787891.1/1-229 ---------SMMMLAQRLVNNFCAS-I--TASNNHG-HK--------W-TNPS-GL----
XP_008787892.1/1-218 ---------SMMMLAQRLVNNFCAS-I--TASNNHG-HK--------W-TNPS-GL----
XP_008799024.1/1-230 ---------SMMKLAQRMVNNFCAC-V--TASNKHG-HK--------W-TNPS-GL----
XP_010936845.1/1-230 ---------SMMTLAQRMVNNFCAR-I--TASNNHG-HV--------W-TNPS-GF----
XP_003570542.1/1-223 ---------SMMRLSERMVSSFCGS-L--SASQ--A-HR--------W-TTLS-GPG---
XP_004964922.1/1-229 ---------SMMKLSQRMVSSFCAS-L--SSSA--L-QR--------W-TPLS-GT----
XP_002438026.1/1-230 ---------SMMKLSQRMVGSFCAS-L--SSSP--L-QR--------W-TLLS-GT----
XP_020399989.1/1-232 ---------SMMRLSQRMVSSFCAS-L--SSSP--L-QR--------W-TLLS-GT----
XP_008649207.1/1-222 ---------SMTKLSQRMVSSFCAS-L--SSSP--L-QR--------W-TLLS-GT----
XP_020256822.1/1-228 ---------SMMKLSQRMLNIFCNS-L--CPSN--G-HQ--------W-NTLS-GL----
XP_009403550.1/1-228 ---------SMMKLAQRMVNNFCAN-V--GASS--D-QK--------W-TNVS-GS----
XP_020582158.1/1-232 ---------SMMRLSQRMVSNFCST-L--GSYN--G-YR--------W-TAPA-GL----
XP_020699118.1/1-240 ---------SMMRLSQRMVSNFCST-L--GSSN--G-YR--------W-IAPA-GV----
XP_020600375.1/1-232 ---------SMMRLAQRMVSNFCST-L--GSSN--G-HR--------W-TAPA-GL----
XP_020600376.1/1-232 ---------SMMRLAQRMVSNFCST-L--GSSN--G-HR--------W-TAPA-GL----
XP_020684394.1/1-232 ---------SMMRLSQRMVSNFCST-L--GSSN--G-HR--------W-TAPA-GL----
XP_017257147.1/1-219 ---------SLMQLSQRIVNSFCSS-I--NSVN--G-QQ--------V--MLS-GM----
XP_010244428.1/1-230 ---------SMMRLAQRMVNSFCAN-I--SASS--G--R--------W-TTLS-GL----
XP_010244355.1/1-230 ---------SMMRLAQRMVNSFCAN-I--SASS--G--R--------W-TTLS-GL----
XP_017240757.1/1-236 ---------SMMKLSQRMVNNFCSI-I--NLPN--S-QQ--------W-TALF-GT----
XP_017245021.1/1-233 ---------SMMKLAQRMVNNFCSI-I--NLPN--S-QQ--------W-TALS-GT----
XP_012829269.1/1-233 ---------SMIGLAQRMVNIFCSC-I--NPCN--G-QQ--------W-TTIS-GQ----
XP_022889938.1/1-236 ---------SMMKLAQRMVNNFCVS-I--NPSN--G-EQ--------W-TTVS-GS----
XP_022897471.1/1-232 ---------SMTKLAQRMINNFCAS-I--NPSN--G-ER--------W-MTLS-GS----
XP_009606250.1/1-235 ---------SMMKLAQRMVSNFCAS-I--NPSN--G-HQ--------W-NAIS-GL----
XP_016468230.1/1-235 ---------SMMKLAQRMVSNFCAS-I--NPSN--G-HQ--------W-NAIS-GL----
XP_019250773.1/1-235 ---------SMMKLAQRMVSNFCAS-I--NPSN--G-HQ--------W-NNIS-GL----
XP_009778533.1/1-235 ---------SMMKLAQRMVSNFCAS-I--NPSN--G-HQ--------W-NAIS-GL----
XP_016451582.1/1-235 ---------SMMKLAQRMVSNFCAS-I--NPSN--G-HQ--------W-NAIS-GL----
XP_016578176.1/1-240 ---------SMMKVSQRMVSSFCAS-I--NPSN--G-HQ--------W-NNIS-TT----
XP_006347370.1/1-231 ---------SMMKVAQRMVSSFCAS-I--NPSN--G-HQ--------W-NNIS-TL----
XP_004241474.1/1-230 ---------SMMKVAERMVSSFCAS-I--NPSN--G-HQ--------W-NNIS-TL----
XP_015077748.1/1-230 ---------SMMKVAERMVSSFCAS-I--NPSN--G-HQ--------W-NNIS-TL----
XP_022037776.1/1-241 ---------SMMNLAQRMVNNFCSS-I--NPSN--G-HQ--------W-TSLS-GM----
XP_024992062.1/1-237 ---------SMMKLSQRMVNNFCSS-I--NPSN--G-HQ--------W-TSLS-GL----
XP_023772270.1/1-245 ---------SMMKLSQRMVRNLCSG-I--NPSN--G-HQ--------W-TSVS-GL----
XP_012855673.1/1-237 ---------SMTRLAQRMVSNFCSS-I--NPSN--G-QQ--------W-TSVS-GM----
XP_022858328.1/1-236 ---------SMMKLAQRMVNNFCAS-L--NPSN--G-QQ--------W-RTLS-GS----
XP_011072039.1/1-232 ---------SMMKLAQRMVNNFCSS-I--NPAS--G-QQ--------W-TTLS-GL----
XP_006306921.1/1-215 ---------SIMRLAHRMVSNFCLS-V--GTSN--N-TR--------S-TVVS-GL----
NP_564041.2/1-215    ---------SIMRLAHRMVSNFCLS-V--GTSN--N-TR--------S-TVVS-GL----
XP_020866823.1/1-216 ---------SIMRLAHRMVNNFCLS-V--GTSN--N-TR--------S-TVVS-GL----
XP_010476956.1/1-215 ---------SIMRLAHRMVSNFCFS-V--GTSH--N-TR--------S-TVVS-GL----
XP_010498167.1/1-217 ---------SIMRLAHRMVSNFCLS-V--GTSH--N-TR--------S-TVVS-GL----
XP_019091528.1/1-217 ---------SIMRLAHRMVSNFCLS-V--GTSH--N-TR--------S-TVVS-GL----
XP_013613976.1/1-227 ---------SIMRLAQRMVSNFCLS-V--GTSN--N-TR--------T-PVFS-GL----
XP_018432674.1/1-227 ---------SIMRLAQRMVSNFCLS-V--GTSN--N-TR--------S-TVVS-GL----
XP_018432667.1/1-227 ---------SIMRLAQRMVSNFCLS-V--GTSN--N-TR--------S-TVVS-GL----
XP_013641731.1/1-225 ---------SIMRLSQRMVSNFCLS-V--GTSN--S-TR--------S-TVVS-GL----
XP_009149243.1/1-227 ---------SIMRLAQRMVSNFCLS-V--GTSN--S-TR--------S-TVVS-GL----
XP_006416656.2/1-224 ---------SIMRLSQRMVSNFCLS-V--GTSN--N-NR--------S-TVIS-GL----
XP_018451633.1/1-224 -----------MKLAQRMVSNFCLS-V--GTSN--N-TR--------S-TVVS-GM----
XP_018451632.1/1-225 -----------MKLAQRMVSNFCLS-V--GTSN--N-TR--------S-TVVS-GM----
XP_018451634.1/1-195 -----------MKLAQRMVSNFCLS-V--GTSN--N-TR--------S-TVVS-GM----
XP_013690958.1/1-228 ---------SIMKLAQRMVSNFCLS-V--GTSN--N-TR--------S-TVVS-GM----
XP_009110376.1/1-227 ---------SIMKLAQRMVSNFCLS-V--GTSN--N-TR--------S-TVVS-GM----
XP_009110377.1/1-227 ---------SIMKLAQRMVSNFCLS-V--GTSN--N-TR--------S-TVVS-GM----
XP_013601964.1/1-227 -----------MKLAQRMVSNFCLS-V--GTSN--N-TR--------SSTVVS-GM----
XP_013653874.1/1-227 -----------MKLAQRMVNNFCLS-V--GTSN--N-TR--------SSTVVS-GM----
XP_018445004.1/1-237 ---------SMMRLAHRMVSNYCIS-V--SRSN--N-TR--------S-TVVA-EL----
XP_018445005.1/1-237 ---------SMMRLAHRMVSNYCIS-V--SRSN--N-TR--------S-TVVA-EL----
XP_013648946.1/1-228 ---------SMMRLAHRMVSNYCIS-V--SRSN--N-TR--------S-TVVA-EL----
XP_009104812.1/1-228 ---------SMMRLAHRMVSNYCIS-V--SRSN--N-TR--------S-TVVA-EL----
XP_013588523.1/1-228 ---------SMMRLAHRMVSNYCIS-V--SRSN--N-TR--------S-TVVA-EL----
XP_013699248.1/1-228 ---------SMMRLAHRMVSNYCIS-V--SRSN--N-TR--------S-TVVA-EL----
XP_018457296.1/1-228 ---------SMMRLAHRMVSNYCIS-V--SRSN--N-TH--------S-TIVA-EL----
XP_009105998.1/1-230 ---------SMMRLAHRMVSNYCIS-V--SRSN--N-TH--------S-TVVA-EL----
NP_001302850.1/1-231 ---------SMMRLAHRMVSNYCIS-V--SRSN--N-TH--------S-TVVA-EL----
XP_013698656.1/1-231 ---------SMMRLAHRMVSNYCLS-V--SRSN--N-TH--------S-TVVA-EL----
XP_013649619.1/1-231 ---------SMMRLAHRMVSNYCIS-V--SRSN--N-TH--------S-TVVA-EL----
XP_013592873.1/1-231 ---------SMMRLAHRMVSNYCIS-V--SRSN--N-TH--------S-AVVA-EL----
XP_006390565.1/1-233 ---------SMMRLAQRMVSNYCLS-V--SRSN--N-TR--------S-TVVA-EL----
XP_006301097.1/1-230 ---------SMMRLAQRMISNYCLS-V--SRSN--N-TR--------S-TVVS-EL----
XP_010428260.1/1-231 ---------SMMRLAQRMISNYCLS-V--SRSN--N-TR--------S-TVVS-EL----
XP_010471396.1/1-230 ---------SMMRLAQRMISNYCLS-V--SRSK--N-IR--------S-TVVS-EL----
XP_010416135.1/1-230 ---------SMMRLAQRMISNYCLS-V--SRSN--N-TR--------S-TVVS-EL----
NP_177479.1/1-227    ---------SMMRLAQRMISNYCLS-V--SRSN--N-TR--------S-TVVS-EL----
XP_020890987.1/1-228 ---------SMMRLAQRMISNYCLS-V--SRSN--N-TR--------S-TVVS-EL----
XP_010537520.1/1-232 ---------SMMRLAQRMVSNFCLS-A--GSSN--N-SR--------S-TVVS-GL----
XP_010540042.1/1-226 ---------SMTRLAQRMVRNFCLS-V--STSN--N-SS--------S-VTVS-GL----
XP_010540046.1/1-196 ---------SMTRLAQRMVRNFCLS-V--STSN--N-SS--------S-VTVS-GL----
XP_019432660.1/1-235 --------SSMMKLSQKMVTNFCAN-V--GTSN--E-QR--------W-TTVS-GL----
XP_019420678.1/1-233 ---------SMLKLSQRMVTSFCAS-V--GTSN--G-QN--------W-TTTF-GS----
XP_013452816.1/1-243 --------SSMMKLAQKMVTSFCAS-I--SASA--S-HR--------W-TTLS-SL----
XP_020215886.1/1-221 ---------SMMKLAQRMVTNFCAS-V--STSS--G-HR--------W-TTLS-GL----
XP_020215888.1/1-221 ---------SMMKLAQRMVTNFCAS-V--STSS--G-HR--------W-TTLS-GL----
XP_007160572.1/1-230 ---------SMMKLAQRMVSNFCAS-I--GTSS--G-RR--------W-TTIS-GF----
XP_017424661.1/1-212 ---------SMMKLAQRMVTNFCGS-I--GTAS--G-RR--------W-TTIS-GL----
XP_017424660.1/1-230 ---------SMMKLAQRMVTNFCGS-I--GTAS--G-RR--------W-TTIS-GL----
XP_014508329.1/1-230 ---------SMMKLAQRMVTNFCGS-I--GTAN--G-RR--------W-TTIS-GL----
XP_022632295.1/1-230 ---------SMMKLAQRMVTNFCGS-I--GTAN--G-RR--------W-TTIS-GL----
XP_019225984.1/1-234 ---------SMMKLAKRMVSNFCAS-I--NPSN--G-HQ--------W-NTIS---G---
XP_016441514.1/1-233 ---------SMMKLAKRMVSNFCAS-I--NPSN--G-HQ--------W-NTIS---G---
XP_009587298.1/1-233 ---------SMMKLAKRMVSNFCAS-I--NPSN--G-HQ--------W-NTIS---G---
XP_016441515.1/1-233 ---------SMMKLAKRMVSNFCAS-I--NPSN--G-HQ--------W-NTIS---G---
XP_009786509.1/1-233 ---------SMMKLAKRMVSNFCAS-I--NPSN--G-HQ--------W-NTNS---G---
XP_016563952.1/1-234 ---------SMMKLAQRMVRNFCTS-V--NPSN--G-HQ--------W-STIS---G---
XP_004235382.1/1-234 ---------SMMKLARRMVSNFCAS-V--NPSN--G-HQ--------W-NTIS---G---
XP_015068940.1/1-234 ---------SMMKLARRMVSNFCAS-V--NPSN--G-HQ--------W-NTIS---G---
XP_006364383.1/1-234 ---------SMMKLARRMVSNFCAS-V--NPSN--G-HQ--------W-NTIS---G---
XP_019167409.1/1-245 ---------SMMKLAQRMVNNFCGS-V--NPSN--G-HH--------W-TTLS---G---
XP_011086973.1/1-238 ---------SMMKLAERMVNNFCSS-I--NPSN--G-QQ--------W-TTLS---V---
XP_010268321.1/1-230 ---------SMMKLAQRMINSFCAS-I--SASN--G-HR--------W-TTLS---G---
XP_010268322.1/1-230 ---------SMMKLAQRMINSFCAS-I--SASN--G-HR--------W-TTLS---G---
XP_010069492.1/1-236 ---------SMMKLAQRMVNNFCSS-I--STSSSRQ-QR--------W-TTLS---G---
XP_002527933.1/1-229 ---------SMMKLAQRMANSFCSS-I--STSN--S-HR--------W-TAIS---G---
XP_021678368.1/1-229 ---------SMMKLAQRMVNSFCAS-I--STSN--S-HR--------W-TTVS---G---
XP_021678369.1/1-229 ---------SMMKLAQRMVNSFCAS-I--STSN--S-HR--------W-TTVS---G---
XP_021602738.1/1-229 ---------SMMKLAQRMVQSFCAS-I--STSN--S-HR--------W-TTVS---G---
XP_012079215.1/1-230 ---------SMMKLAQRMVNSFCAS-I--STSN--R-HR--------W-TTVS---G---
XP_021687701.1/1-229 ---------SMMKLAQRMVNSFCAS-I--STSN--S-HR--------W-TTVS---G---
XP_006374212.1/1-231 ---------SMMKLAQRMVSSFCSS-I--STSN--S-HR--------W-STLS---G---
XP_011035552.1/1-231 ---------SMMKLAQRMVSSFCSS-I--STSN--S-HR--------W-STLS---G---
XP_002317722.3/1-231 ---------SMMKLGQRMVSSFCSS-I--STSN--S-HR--------W-STLS---G---
XP_011000766.1/1-231 ---------SMMKLGQRMVSSFCSS-I--STSN--S-HR--------W-STLS---G---
XP_021907827.1/1-230 ---------SMMKLAQRMVNNFCTS-I--STSS--S-HR--------W-TTLS---G---
XP_017635401.1/1-230 ---------SIMKLAQRMVNNFCTS-V--GTSN--S-HR--------S-TTLS---G---
XP_016686699.1/1-230 ---------SMMKLAQRMVNNFCTS-V--GTSN--S-HR--------S-TTLS---G---
XP_012440361.1/1-230 ---------SMMKLAQRMVNNFCTS-V--GTSN--S-HR--------S-TTLS---G---
NP_001313868.1/1-230 ---------SMMKLAQRMVNNFCTS-V--GTSN--S-HR--------S-TTLS---G---
XP_023919022.1/1-230 ---------SMMKLAQRMINNFCSS-I--STSN--G-HR--------W-TTLS---G---
XP_006478112.1/1-232 ---------SMMKLAQRMVSSFCTS-I--STSN--R-HR--------G-TTIS---G---
XP_024042646.1/1-232 ---------SMMKLAQRMVSSFCTS-I--STSN--R-HR--------G-TTIS---G---
XP_022715403.1/1-231 ---------GMMKLAQRMVNHFCTS-V--STSN--S-HR--------W-TTLS---G---
XP_022767009.1/1-255 ---------SMMKLAQRMVNNFCTS-I--STSN--S-HR--------W-TTLS---G---
XP_022737050.1/1-231 ---------SMMKLAQRMVNNFCTS-I--STSN--S-HR--------W-TTLL---G---
XP_017973835.1/1-231 ---------SMMKLAQRMVNNFCTS-I--STSN--S-HR--------W-TTLS---G---
XP_015967008.1/1-238 ----------MMRLAQRMVTNFCAS-I--GTSA--G-HR--------W-TTLS---G---
XP_025701434.1/1-239 ---------SMMRLAQRMVTNFCAS-I--GTSA--G-HR--------W-TTLS---G---
XP_020980110.1/1-239 ---------SMMRLAQRMVTNFCAS-I--GTSA--G-HR--------W-TTLS---G---
XP_019434881.1/1-231 -----------MKLAQRMVTNFCAN-I--STSN--G-QR--------W-TILS---G---
XP_019458275.1/1-234 ---------SMMKLAQRMVTNFCAS-I--STSN--G-QR--------W-TILS---G---
XP_019458276.1/1-234 ---------SMMKLAQRMVTNFCAS-I--STSN--G-QR--------W-TILS---G---
XP_006584922.1/1-234 ---------SMMKLAQRMVTNFCAN-I--STSS--G-HR--------W-TTLS---G---
XP_003524332.1/1-231 ---------SMMKLAQRMVTDFCAS-I--STSS--G-HR--------W-TTLS---G---
XP_004515249.1/1-229 -----------------MVTNFCAS-I--SASA--S-HR--------W-TTLS---A---
XP_019431381.1/1-229 ---------SMMKLAQRMITNFSAS-I--STSC--S-NR--------W-TTLS---G---
XP_019436771.1/1-231 ---------SMMKLAQRMITNFCAS-I--STSA--S-HK--------W-TTLS---G---
XP_019430342.1/1-233 ---------SMMKLAQRMITNFCAS-I--STSA--S-HR--------W-TTVS---G---
XP_006587436.1/1-240 ---------SMMKLAQRMVTNFCAS-I--SASA--G-HR--------W-TTLS-GSG---
XP_003549189.1/1-236 ---------SMMKLAQRMVTNFCAS-I--SSSA--G-HR--------W-TTLS-GSG---
XP_020221986.1/1-232 ---------SMMKLAQRMVTNFCAS-I--SASS--G-HR--------W-TTLS---G---
XP_007152598.1/1-234 ---------SMMKLAQRMVTNFCAS-I--SASA--G-HR--------W-TTLS-GSG---
XP_017437542.1/1-234 ---------SMMKLAQRMVTNFCAS-I--SASA--G-HR--------W-TTLS-GSG---
XP_014505752.1/1-234 ---------SMMKLAQRMVTNFCAS-I--SASA--G-HR--------W-TTLS-GSG---
XP_015881991.1/1-234 ---------SMMKLAQRMVNNFCAS-I--NPSN--G-HQ--------W-TTLS---G---
XP_004299483.1/1-232 ---------SMMKLAQRMVNNFCAS-I--STSN--G-HR--------W-TTIS---G---
XP_024174647.1/1-232 ---------SMMKLAQRMVNNFCAS-I--STSN--G-HR--------W-TTIS---G---
XP_008375041.1/1-227 ---------SMMKLAQRMVNNFCAS-I--SASN--G-HR--------W-TTLS---G---
XP_017188172.1/1-199 ---------SMMKLAQRMVNNFCAS-I--SASN--G-HR--------W-TTLS---G---
XP_009355964.1/1-227 ---------SMMKLAQRMVNNFCAS-I--SASN--R-HR--------W-TTLS---G---
XP_009355965.1/1-199 ---------SMMKLAQRMVNNFCAS-I--SASN--R-HR--------W-TTLS---G---
XP_023523434.1/1-228 ---------SMMKLAQRMVNNFCAS-I--STSH--G-HR--------W-TTLS---G---
XP_022949138.1/1-227 ---------SMMKLAQRMVNNFCAS-I--TTSH--G-HR--------W-TTLS---G---
XP_022998050.1/1-227 ---------SMMKLARRMVNNFCAS-I--STSH--G-HR--------W-TTLS---G---
XP_002271012.1/1-231 ---------SMMKLAQRMVNNFCAS-I--STSN--G-HR--------W-TTLS---G---
XP_010663934.1/1-231 ---------SMMKLAQRMVNNFCAS-I--STSN--G-HR--------W-TTLS---G---
XP_010099679.1/1-237 ---------SMMKLAQRMVNNFCAS-I--STSN--G-HR--------W-TTLS---G---
XP_018823334.1/1-236 ---------SMMKLAQRMVNNFCAS-I--STSN--S-HR--------W-TTLS---G---
XP_023001145.1/1-228 ---------SMMKLAQRMVNNFCAS-I--SSSH--G-HR--------W-TTLS---G---
XP_023519889.1/1-228 ---------SMMKLAQRMVNNFCAS-I--SSSH--G-HR--------W-TTLS---G---
XP_022924191.1/1-228 ---------SMMKLAQRMVNNFCAS-I--SSSH--G-HR--------W-TTLS---G---
XP_018849090.1/1-235 ---------SMMKLAQRMVNNFCAS-I--STSN--G-HR--------W-TTLS---G---
XP_008240378.1/1-228 ---------SMMKLAQRMVNNFCAS-I--STSN--G-HR--------W-TTLS---G---
XP_007210460.2/1-228 ---------SMMKLAQRMVNNFCAS-I--STSN--G-HR--------W-TTLS---G---
XP_021801797.1/1-228 ---------SMMKLAQRMVNNFCAS-I--STSN--G-HR--------W-TTLS---G---
XP_022136988.1/1-228 ---------SMMKLAQRMVNNFCAS-I--STSH--G-HR--------W-TTLS---G---
XP_016898817.1/1-228 ---------SMMKLAQRMVNNFCAS-I--STSH--G-HR--------W-TTLS---G---
XP_004137568.1/1-228 ---------SMMKLAQRMVNNFCAS-I--STSH--G-HR--------W-TTLS---G---
XP_015642098.1/1-238 ---------SMMKLSQRMVNSFCSS-L--GASQ--M-HQ--------W-TTLS-GSN---
XP_020186741.1/1-220 ---------SMMRLSQRMVGSFCAS-L--SASQ--Q-HR--------W-TTLS-GPG---
XP_025828617.1/1-229 ---------SMMKLSQRMVSGFCAS-L--SASQ--L-HR--------W-TTLS-GPN---
XP_004954072.1/1-226 ---------SMMKLSQRMVSSFCAS-L--SSSQ--L-HR--------W-TMLS-GPN---
XP_002452898.1/1-231 ---------SMMKLSQRMVSSFCAS-L--SASQ--L-HR--------W-TTLS-GPN---
NP_001142912.2/1-230 ---------SMMKLSQRMVSSFCAS-L--SASQ--L-HR--------W-TTLS-GPS---
XP_020089796.1/1-231 ---------SMMKLSQRMVNNFCAS-L--SSSQ--I-QR--------W-TMLS-GLS---
XP_020089795.1/1-241 ---------SMMKLSQRMVNNFCAS-L--SSSQ--I-QR--------W-TMLS-GLS---
XP_019708739.1/1-228 ---------SMMKLSQRMINNFCAS-L--SASN--G-NR--------W-TTLS-GLN---
XP_008789232.1/1-228 ---------SMMKLSQRMINNFCAS-L--SASN--G-NR--------W-TTLS-GLN---
XP_008789231.1/1-228 ---------SMMKLSQRMINNFCAS-L--SASN--G-NR--------W-TTLS-GLN---
XP_008789233.1/1-200 ---------SMMKLSQRMINNFCAS-L--SASN--G-NR--------W-TTLS-GLN---
XP_019704584.1/1-228 ---------SMMKLSQRMINNFCSS-L--SAST--G-NR--------W-TTLS-GLN---
XP_010917295.1/1-228 ---------SMMKLSQRMINNFCSS-L--SAST--G-NR--------W-TTLS-GLN---
XP_008806630.1/1-228 ---------SMMKLSQRMINNFCYS-L--SAST--G-NR--------W-TTLS-GLN---
XP_009388746.1/1-226 ---------SMMKLAQRMLSSFCAN-V--GASN--G-HQ--------W-NTIS-GLN---
XP_018675467.1/1-228 ---------SMMKLAQRMVTNFCAN-V--GASN--G-HK--------W-TTLS-GVN---
XP_018675466.1/1-228 ---------SMMKLAQRMVTNFCAN-V--GASN--G-HK--------W-TTLS-GVN---
XP_009409108.1/1-228 ---------SMMKLAQRMVNNFCAN-V--GAAN--G-HK--------W-TTLS-GLN---
XP_024625408.1/1-220 ----------MMKLTQRMVTNFCES-I--NGSA--S-HR--------WT-TLS-TLN---
XP_010550261.1/1-219 ---------SMMKLGQRMLKNFTGM-L--SMSG--K-LD--------FP-QLS-EVN---
XP_018816719.1/1-228 -----------MKLSHRMVKNFCGI-L--SMSG--K-LD--------FPHQLS-ELY---
XP_008365756.2/1-218 ---------SLMKLSQRMVKNFCGM-L--SMAG--N-LD--------XP-QLS-EVD---
XP_008381522.1/1-218 ---------SLMKLSQRMVKNFCGM-L--SMAG--N-LD--------XP-QLS-EVD---
XP_018506060.1/1-218 ---------SLMKLSQRMVKNFCGM-L--SMEG--K-LD--------FP-QLS-EVD---
XP_008362571.1/1-218 ---------SLMKLSQRMVKNFCGM-L--SMEG--K-LD--------FP-QLS-EVE---
XP_023870350.1/1-229 ---------SIMKLSHRMVKNFCGM-L--SMTG--K-LD--------FP-QLS-EVN---
XP_016649213.1/1-222 -----------MKLSHRMVKNFCGM-L--SMAG--K-LD--------FP-QLS-EVH---
XP_020415996.1/1-222 -----------MKLSHRMVKNFCGM-L--SMAG--K-LD--------FP-QLS-EVH---
XP_021823813.1/1-222 -----------MKLSHRMVKNFCGM-L--SMAG--K-LD--------FP-QLS-EVH---
XP_009367126.1/1-222 -----------MKLSQRMVKNFCGM-L--SMAV--K-LD--------FP-QLS-EVN---
XP_008348615.1/1-222 -----------MKLSQRMVKNFCGM-L--SMAG--K-LD--------FP-QLS-EVN---
XP_017179578.1/1-222 -----------MKLSQRMVKNFCGM-L--SMAG--K-ID--------FP-QSS-EVN---
XP_009370612.1/1-224 ---------SVMMLSQRMVKNFCGM-L--SMAG--K-LD--------FP-QSS-EVN---
XP_012845089.1/1-228 ----------LMKLSHRMIKNFCEV-L--GMSD--R-LD--------FP-YLS-ELN---
XP_012845088.1/1-228 ----------LMKLSHRMIKNFCEV-L--GMSD--R-LD--------FP-YLS-ELN---
XP_012845087.1/1-228 ----------LMKLSHRMIKNFCEV-L--GMSD--R-LD--------FP-YLS-ELN---
XP_011085930.1/1-227 ----------LMKLSHRMMNNFCEA-L--SMSD--K-LD--------FP-HLS-EFK---
XP_020551334.1/1-222 ---------------QRMCERFAFS-M--GLTT--T-HK--------HE-LEG-EFK---
XP_021297998.1/1-224 ---------SIMRLAHRMVKSFCSI-L--NMSG--E-LD--------FP-QLS-EEN---
XP_017974480.1/1-224 ---------SIMRLAHRMVKSFCSI-L--NMSG--E-LD--------FP-QLS-EEN---
XP_024448785.1/1-229 ------------NLAHRMVKIFFAS-L--GMSG--K-LD--------FP-QLS-EVH---
XP_024455385.1/1-229 ------------NLAHRMVKIFFAS-L--GMSG--K-LD--------FP-QLS-EVH---
XP_011005937.1/1-269 ------------NLAHRMVKIFFAS-L--GMSG--K-LD--------FP-QFS-EVN---
XP_011038413.1/1-229 ------------NLAHRMVKIFSSS-L--GMSG--K-LD--------FR-QLS-EGN---
XP_018731153.1/1-226 -----------MKLAHRMVKNFSAG-L--SMSG--K-LD--------FP-HLS-EVN---
XP_024190515.1/1-228 --------SSIMKLSHRMVRNFCGM-L--SMSG--K-LD--------FP-QLS-EAN---
XP_012074722.2/1-231 ----------MMKLSHRMIKDFCAM-L--SMSG--K-LD--------FP-QLS-EVN---
XP_010089514.2/1-229 -----------MNLSHRMVKNFCGT-L--NMSG--K-LD--------FP-QLA-EVT---
XP_024949330.1/1-235 ---------SLMKLAHRMVKNFCSM-L--SMSG--K-LD--------FP-QLS-EAN---
XP_006421085.1/1-235 ---------SLMKLAHRMVKNFCSM-L--SMSG--K-LD--------FP-QLS-EVN---
XP_002268272.1/1-229 ---------SIMKLGHRMVKDFCGV-L--SMSG--K-LD--------FP-QLS-EVD---
XP_004140733.2/1-228 ---------SIMKLSHRMVKNFCGI-L--SMSG--K-ID--------FP-QLS-EVN---
XP_008457114.1/1-228 ---------SIMKLSHRMVKNFCGI-L--SMSG--K-LD--------FP-QLS-EVN---
XP_008457113.1/1-229 ---------SIMKLSHRMVKNFCGI-L--SMSG--K-LD--------FP-QLS-EVN---
XP_011651208.1/1-229 ---------SIMKLSHRMVKNFCGI-L--SMSG--K-ID--------FP-QLS-EVN---
XP_022143367.1/1-228 ---------SIMKLSHRMVKNFCGV-L--SMSG--K-LD--------FP-QLS-EVN---
XP_022935490.1/1-228 ---------SIMKLSHRMVKNFCGV-L--SMSG--K-LD--------FP-QLS-EVN---
XP_022984101.1/1-228 ---------SIMKLSHRMVKNFCGV-L--SMSG--K-LD--------FP-QLS-EVN---
XP_022935488.1/1-229 ---------SIMKLSHRMVKNFCGV-L--SMSG--K-LD--------FP-QLS-EVN---
XP_022984098.1/1-229 ---------SIMKLSHRMVKNFCGV-L--SMSG--K-LD--------FP-QLS-EVN---
XP_022984102.1/1-229 ---------SIMKLSHRMVKNFCGV-L--SMSG--K-LD--------FP-QLS-EVN---
XP_015579295.1/1-234 ---------SLMKLSHRMVRSFCAM-L--SMSG--K-LD--------FP-QLS-EVN---
XP_002526595.2/1-234 ---------SLMKLSHRMVRSFCAM-L--SMSG--K-LD--------FP-QLS-EVN---
XP_015579294.1/1-234 ---------SLMKLSHRMVRSFCAM-L--SMSG--K-LD--------FP-QLS-EVN---
XP_021643520.1/1-232 ---------SIMKLAHRMVKNFCSM-L--SMSG--K-LD--------FP-QLS-EVK---
XP_021605433.1/1-231 ---------SIMKLGHRIVKNFCST-L--SMSD--K-LD--------FP-QLS-EVN---
XP_023739098.1/1-228 ---------SMMNLAQRMVNNFCAI-I--SPSD--D-HQ--------WS-CVS-DFD---
XP_024966239.1/1-227 -----------MNLAQRMVNNFCTS-I--VPSS--G-HL--------GT-SVS-DFD---
XP_025813558.1/1-232 -----------MKLSQQMVGLFCAS-L--SSSP--L-QR--------WV-LLP-GTT---
XP_020150479.1/1-234 ---------SMMALSQRMVSSFCGG-M--TASP--Q-HQ--------WT-TMP-GAA---
XP_003564122.2/1-224 ---------SMMKLSQRMVANFCGA-L--TANQ--L-IV--------WT-TIP-GGA---
XP_006585049.2/1-225 -----------MHLAHRMVKTFCRT-L--DVSD--C-EN--------FP-YLT-RMM---
XP_007143783.1/1-226 ----------IMRLTRRMVKTFCES-L--CMQD--N-TS--------FP-HLT-RMN---
XP_007158859.1/1-226 ----------IMRLTRRMVKCYCES-L--CMKD--N-TN--------FP-HLT-RMN---
XP_020230815.1/1-227 ---------GIMKLSLQMVKNFYGF-L--NMSS--N-TN--------FP-QHL-TEG---
XP_006586851.1/1-227 ---------SMMKFSHQMVKSFYGI-L--NMSS--I-TD--------FP-QHL-ADE---
XP_021888058.1/1-234 ---------SILKLCQRMIKDFCAS-M--CISGDNK-LD--------FI-HTC-ESN---
XP_020886111.1/1-226 -----------MKLGERMVKNFNEM-L--TMSG--K-VD--------FP-QQS-------
XP_020886110.1/1-228 -----------MKLGERMVKNFNEM-L--TMSG--K-VD--------FP-QQS-------
XP_011469296.1/1-224 ------------NLSHRMVKNFCGM-L--SMSG--K-LD--------FP-QLS-EMK---
XP_009367128.2/1-218 ---------SLMTLSQRMVKNFCGM-L--SMAG--N-LD--------FP-QLS-EVD---
XP_008348673.1/1-198 ---------SLMKLSQRMVKNFCGM-L--SMVG--N-LD--------LP-QLS-EVD---
XP_012832506.1/1-227 -----------MNLSQRMVESFFEV-L--CMSD--K-LD--------FP-NPS-EYQ---
XP_012832508.1/1-227 -----------MNLSQRMVESFFEV-L--CMSD--K-LD--------FP-NPS-EYQ---
XP_012832516.1/1-227 -----------MTLSQRMVESFFEV-L--CMSD--K-LD--------FP-NPL-EYH---
XP_012832518.1/1-227 -----------MTLSQRMVESFFEV-L--CMSD--K-LD--------FP-NPL-EYH---
XP_012835569.1/1-227 -----------MTLSQRMVESFFEV-L--CMSD--K-LD--------FP-NPS-EYQ---
XP_012835571.1/1-226 -----------MTLSQRMVESFFEV-L--CMSD--K-LD--------FP-NPS-EYQ---
XP_019172066.1/1-226 -----------MQLSQRMVKSFCEV-L--NMGE--R-VD--------FP-QSS-EMN---
XP_009631364.2/1-226 -----------MQICQRMVMSFCEI-L--SMGD--R-LD--------FP-TSS-EMT---
XP_016435660.1/1-229 -----------MQISQRMVKSFCEI-L--SMGD--R-LD--------FP-TSS-EMT---
XP_009771801.1/1-226 -----------MQISQRMVKSFCEI-L--SMGD--R-LD--------FP-TSS-EMT---
XP_016493115.1/1-226 -----------MQISQRMVKSFCEI-L--SMGD--R-LD--------FP-TSS-EMT---
XP_019248352.1/1-226 -----------MQISQRMVKSFCEI-L--SMGD--S-LD--------FP-TSS-EMT---
XP_025691463.1/1-225 -----------MEFSERMVKLFCGS-L--DMTG--N-ME--------LQ-Y---GKN---
XP_020995109.1/1-225 -----------MEFSERMVKLFCGS-L--DMTG--N-ME--------LQ-Y---GKN---
XP_016190415.2/1-225 -----------MEFSERMVKLFCGS-L--DMSG--N-ME--------LQ-Y---GKN---
XP_004486568.1/1-232 ---------GVMKFAHRMVKMFCEC-L--TMTG--Q-LE--------FQ-HLS-LDS---
XP_024632687.1/1-226 ------------KLAQRMVKMFCES-L--TMPG--Q-LE--------LN-HLT-LAS---
XP_020977993.1/1-241 -----------MRLGHRLVRLFSEN-L--NMSG--K-VE--------FP-QLS-VEY---
XP_025647253.1/1-241 -----------MRLGHRLVRLFSEN-L--NMSG--K-VE--------FP-QLS-VEY---
XP_020997095.1/1-240 -----------MRLGQRLVRLFSEN-L--NMSG--K-VE--------FP-QLS-VEY---
XP_025692389.1/1-228 -----------MRLGQRLVRLFSEN-L--NMSG--K-VE--------FP-QLS-VEY---
XP_019418389.1/1-226 ---------SIMNLSHRMVKIFCEN-L--IISD--K-LE--------FP-QLS-SER---
XP_007147460.1/1-227 ---------SIMNFAHRMLKIFCES-I--TMAD--G-LD--------FP-LSD-LAN---
XP_014517364.1/1-227 ---------SIMNFSHRMLKIFCES-I--TMAD--G-LD--------FP-LCD-LVN---
XP_017435438.1/1-226 ---------SIMNFSHRMLKIFCES-I--TMAD--G-LD--------FP-LCD-LAN---
XP_017435439.1/1-217 ---------SIMNFSHRMLKIFCES-I--TMAD--G-LD--------FP-LCD-LAN---
XP_025981664.1/1-227 -----------MNFCHRMIKVFCES-L--TMSG--N-LD--------FP-LLK-MEN---
XP_006586853.1/1-227 -----------MNFSHRMIKVFCES-L--TMSG--N-LD--------FP-HMN-MEN---
XP_020230831.1/1-226 -----------MNFSHRMIKIFCES-L--TMSG--K-LD--------LP-QSN-MEN---
XP_004489663.1/1-217 -----------MRLSHRMVRMFCEC-L--TTQS--N-VE--------FP-STI-QDS---
XP_018489833.1/1-227 -----------MKIGERMLKIFNEM-L--IMSG--K-VE--------FP-QQS-K-----
XP_013584487.1/1-219 -----------MKLGERMLKIFNEM-L--IMSG--K-VE--------FP-QQS-K-----
XP_009130351.1/1-219 -----------MKLGERMLKIFNEM-L--IMSG--K-VE--------FP-QQS-K-----
XP_013747906.2/1-219 -----------MKLGERMLKIFNEM-L--IMSG--K-VE--------FP-QQS-K-----
XP_013747661.1/1-219 -----------MKLGERMLKIFNEM-L--IMSG--K-VE--------FP-QQS-K-----
XP_013619678.1/1-219 -----------MKLGERMVKIFNEM-L--IMSG--K-VE--------FP-QQS-K-----
XP_022551084.1/1-219 -----------MKLGERMVKIFNEM-L--IMSG--K-VE--------FP-QQS-K-----
XP_009126392.1/1-219 -----------MKLGERMVKIFSEM-L--IMSG--K-IE--------FP-QQS-K-----
XP_018437612.1/1-222 -----------MKLGERMVKNLNEM-L--TMSG--K-VD--------FP-QQS-K-----
XP_013591191.1/1-225 -----------MKLGERMVKNFNEM-L--AMSG--K-VD--------FP-QQS-K-----
XP_022567005.1/1-225 -----------MKLGERMVKNFNEM-L--AMSG--K-VD--------FP-QQS-K-----
XP_022547796.1/1-225 -----------MKLGERMVMNFNEM-L--TMSG--K-VD--------FP-QQS-K-----
XP_009119205.1/1-225 -----------MKLGERMVMNFNEM-L--TMSG--K-VD--------FP-QQS-K-----
XP_009119199.1/1-225 -----------MKLGERMVMNFNEM-L--TMSG--K-VD--------FP-QQS-K-----
XP_006408322.1/1-232 -----------MKLGERMLKNFNDM-L--TMSG--K-VD--------FP-QHS-K-----
XP_023642295.1/1-229 -----------MKLGGRMIQDFNGM-L--TMSG--K-VD--------FP-KQS-K-----
XP_023642294.1/1-229 -----------MKLGGRMIQDFNGM-L--TMSG--K-VD--------FP-KQS-K-----
NP_186976.2/1-228    -----------MKLGERMVKNFNEM-L--TMSG--K-ID--------FP-QQS-K-----
XP_010507998.1/1-229 -----------LKLGERMIMNFNEM-L--TMSG--K-VD--------FP-QQS-K-----
XP_010485661.1/1-229 -----------LKLGERMIMNFNEM-L--TMSG--K-VD--------LP-QQS-K-----
XP_010450732.1/1-230 -----------LKLGERMIMNFNEM-L--TMSG--K-VD--------FP-QQS-K-----
XP_010463776.1/1-230 -----------LKLGERMIMNFNEM-L--TMSG--K-VD--------FP-QQS-K-----
XP_010463774.1/1-230 -----------LKLGERMIMNFNEM-L--TMSG--K-VD--------FP-QQS-K-----
XP_019092960.1/1-229 -----------LKLGERMIMNFNEM-L--TMSG--K-VD--------FP-QQS-K-----
XP_022551077.1/1-228 ---------SLMNLGERMLKNFAWI-L--KMSG--N-YD--------FS-QLS-ETN---
XP_013619236.1/1-228 ---------SLMNLGERMLKNFAWI-L--KMSG--N-YD--------FS-QLS-ETN---
XP_009126177.1/1-226 -----------MNLGERMLKNFAWI-L--KMSG--N-YD--------FS-QLS-ETN---
XP_013652421.1/1-226 -----------MNLGERMLKNFAWI-L--KMSG--N-YD--------FS-QLS-ETN---
XP_013652420.1/1-226 -----------MNLGERMLKNFAWI-L--KMSG--N-YD--------FS-QLS-ETN---
XP_006400265.1/1-228 -----------MRLGEKMSKNFAWI-L--KMSG--K-FD--------FS-QLS-ETN---
XP_013590801.1/1-226 -----------MDLGERMLKNFAWI-L--NMPE--K-SE--------FS-QQS-ATN---
XP_013651910.1/1-222 ----------LMDLGERMLKNFAWI-L--NMPE--K-SD--------FS-QQS-ATN---
XP_009103958.1/1-222 ----------LMDLGERMLKNFAWI-L--NMPE--K-SD--------FS-QQS-ATN---
XP_018444485.1/1-226 -----------MKLGERMLKNFAWI-L--KMPG--K-AE--------FP-QQN-VTN---
XP_009121397.1/1-226 -----------MDLGERMLKNFAWI-L--KMPE--K-SG--------FS-QQT-ATN---
XP_013659428.1/1-226 -----------MDLGERMLKNFAWI-L--KMPE--K-SG--------FS-QQT-ATN---
XP_022565333.1/1-226 -----------MDLGERMSKNFAWI-L--KMPE--K-SG--------FS-QQT-ATN---
XP_013610327.1/1-226 -----------MDLGERMSKNFAWI-L--KMPE--K-SG--------FS-QQT-ATN---
XP_006286655.1/1-225 -----------MSLGERMSKNFAWM-I--NMVQ--K-LD--------FS-QQS-ETN---
XP_010492677.1/1-224 -----------LKLGERMLKDFAWM-I--KMED--K-LD--------FA-QQS-ETN---
XP_010420445.1/1-224 -----------MKLGERMLKDFAWM-I--KMEK--K-LD--------FS-QQS-ETN---
XP_010453918.1/1-224 -----------MKLGERMLKDFAWM-I--NMEE--K-LD--------FS-QQS-ETN---
XP_006303739.1/1-224 ---------SIMNLGERMLKNFAWI-M--KMAT--K-LD--------FS-RPS-DAD---
XP_020876074.1/1-222 -----------MQLGERMLRNFAWI-M--KMTD--K-QD--------FS-QQS-ETN---
NP_197234.1/1-222    -----------MHLGERMLRNFAWM-M--KMVN--K-LD--------FS-PQS-ETN---
NP_174724.1/1-221    -----------MSLGERMLKNFAWI-M--KMSD--K-LD--------LP-QQS-GAN---
XP_020869876.1/1-222 -----------MNLGERMLKNFAWI-M--KMSD--K-LD--------FS-QHC-EAN---
XP_019091098.1/1-222 -----------MNLGERMLKNFAWI-M--KMSD--K-HD--------FS-QQC-DTN---
XP_010478910.1/1-223 -----------MNLGERMLKNFAWI-M--KMSD--K-QD--------FS-QQC-DTN---
XP_010501781.1/1-221 -----------MNLGERMLKNFAWI-M--KRSD--K-HD--------FS-QQC-DTN---
XP_015089790.1/1-222 ------------QISQRMVKSFCEI-L--SMTQ--K-LD--------FS-TSS-K-L---
XP_015089784.1/1-222 ------------QISQRMVKSFCEI-L--SMTE--K-LD--------FS-TSS-K-L---
XP_016549855.1/1-224 -----------MQTSQRMVKRFFEI-L--SMTD--R-LD--------FL-TSS-K-L---
XP_015057403.1/1-221 ------------QLSQRMVKSFFEI-L--SMTD--K-LD--------FP-TSP-Q-L---
XP_019066399.1/1-221 ------------QLSQRMVKNFFEI-L--SMTD--K-LD--------FP-ASP-Q-L---
XP_015166059.1/1-216 ------------QISQRMVKSFFEI-L--SMTD--N-LD--------FP-TSS-R-L---
XP_019071012.2/1-216 ------------QISQRMVKNFFEI-L--SMTD--N-LD--------FP-TSS-R-L---
XP_015086709.1/1-216 ------------QISQRMVKNFFEI-L--SMTD--N-LD--------FP-TSS-R-L---
XP_015087432.1/1-219 --------------SQRMVKSFFEI-L--SMTD--YDGD--------FP-TSS-Q-V---
XP_004247342.2/1-219 --------------SQRMVKSFFEI-L--SMTD--YDGD--------FP-TSS-Q-V---
XP_025888717.1/1-219 --------------SQRMVKSFFEI-L--SMTD--YDGD--------FP-TSS-Q-V---
XP_015087599.1/1-221 ------------KVSQRMVKSFFEI-L--SMAD--K-ID--------FP-TSS-Q-L---
XP_019071156.1/1-221 ------------KVSQRMVKSFFEI-L--SMAD--K-LD--------FP-TSS-Q-P---
XP_019070762.2/1-220 --------------SQRMVKKFFEI-L--SMTD--NHGD--------FS-ISP-Q-L---
XP_015087071.1/1-220 --------------SQRMVKSFFEI-L--SMTD--NHGD--------FS-ISP-Q-L---
XP_025888845.1/1-217 -----------------MVKSFFEI-L--CMTD--NHGD--------FS-ISP-Q-L---
XP_015170367.1/1-232 ----PEGLDNVMQISLRMVKRFCEI-L--SMTG--N-LV--------YP-TSS-Q-L---
XP_025886268.1/1-232 ----------IMQISLRMVKRFCEI-L--SMTE--K-LD--------FP-TSS-K-F---
XP_015072491.1/1-224 -----------MQISLRMVKRFCEI-L--SMTE--K-LD--------FP-TSS-K-F---
XP_010327374.2/1-219 -----------MNISQRMVKRFCEI-L--SMTD--K-LD--------FS-TSL-L-L---
XP_015089749.1/1-219 -----------MSISQRMVKRFCEI-L--NMTD--K-LD--------FS-TSL-L-L---
XP_015170971.1/1-209 ----------LMNLSQRMVKSFCEI-L--NMTD--K-LD--------FP-TSS-Q-L---
XP_025887726.1/1-223 ----------LMNLSQRMVKSFFEI-L--SMKD--G-LE--------FS-TSS-Q-L---
XP_006586358.1/1-227 ----------IMHLTHRMVKMFCGN-L--DMQ---DNPN--------FP-NLT-RMN---
XP_014617351.1/1-228 ---------SMMKFSHQMVKSFYGI-L--NMS---SITD--------FP-QHL-ADE---
XP_007147461.1/1-227 ----------MMRFSDQMVKNFYGI-L--NMVS--TKRD--------FP-EHL-AEE---
XP_017435408.1/1-226 ----------MMKFSHQMVKNFYEI-L--NIS---SKTE--------YI-EHL-AEE---
XP_017215828.1/1-223 ---------SILELAQRMTTSFYRA-LGA--SN--Y-HT--------WN-TAT-SK----
XP_012856110.1/1-229 ---------SILTLSQRMSWSFCRA-IGG--SR--R-IL--------WK-KIV-SK----
XP_023738891.1/1-226 ------------KLAERMTWSFSRA-LGG--SS--H-HT--------WK-KIP-SK----
XP_022017964.1/1-227 ---------SILKLAQRMTWSFGRA-LGG--SS--D-RT--------WK-KIP-SK----
XP_022154996.1/1-226 -----------LRLAQRMTSSFYQA-FGA--SI--S-HS--------WP-KVP-TK----
XP_008462169.1/1-225 -----------LRLAQRMTSSIYQA-IGA--SS--S-HT--------WT-KVQ-SK----
XP_004141841.1/1-226 -----------LRLAQRMTSSIYQA-IGA--SN--S-HT--------WT-KVQ-SK----
XP_020098514.1/1-228 ---------SILKLAHRMSLSFCHN-IGA--SR--Y-NT--------WQ-KVS--K----
XP_020098513.1/1-228 ---------SILKLAHRMSLSFCHN-IGA--SR--Y-NT--------WQ-KVS--K----
XP_020263950.1/1-229 ---------SILTLAERMCISFCHA-MGG--SR--N-RA--------WT-RVS-TK----
XP_020263948.1/1-255 ---------SILTLAERMCISFCHA-MGG--SR--N-RA--------WT-RVS-TK----
XP_009415234.2/1-232 ---------SILKLGQRMTSCFCQN-IGA--SG--H-HK--------WT-KVS-TK----
XP_008789333.1/1-230 ---------SLLKFAQRMTSSFCQA-IAA--SR--N-RT--------WT-KAS-IR----
XP_010920420.1/1-235 ----PAGRKSILKFSQRMTSSFCLA-IGA--SR--N-RT--------WT-KAS-IR----
XP_010920411.1/1-235 ----PAGRKSILKFSQRMTSSFCLA-IGA--SR--N-RT--------WT-KAS-IR----
XP_010920404.1/1-235 ----PAGRKSILKFSQRMTSSFCLA-IGA--SR--N-RT--------WT-KAS-IR----
XP_010521515.1/1-219 --------------------GFYRA-IGA--SS--Y-HQ--------WS-RIT-TK----
XP_013652056.1/1-227 -----------LKMAQRMTQSFYRA-IAA--SS--Y-HQ--------WN-KIT-TK----
XP_009104428.1/1-227 -----------LKMAQRMTQSFYRA-IAA--SS--Y-HQ--------WN-KIT-TK----
XP_018445476.1/1-226 -----------LKMAQRMTQSFYRA-IAA--SS--Y-HQ--------WN-KIT-TK----
XP_013590784.1/1-226 -----------LKMAQRMTQSFYRA-IAA--SS--Y-HQ--------WN-KIT-TK----
XP_013680822.1/1-226 -----------LKMAQRMTQSFYRA-IAA--SS--Y-HQ--------WN-KIT-TK----
XP_006389720.2/1-226 -----------LKMAQRMTQSFYRA-IAA--SS--X-HQ--------WT-KIT-TK----
XP_023642973.1/1-226 -----------LKMAQRMTQSFYRA-IAA--SS--Y-HQ--------WT-KIT-TK----
XP_020891640.1/1-226 -----------LKMAQRMTQSFYRA-IAA--SS--Y-HQ--------WT-KIT-TK----
NP_001320267.1/1-226 -----------LKMAQRMTQSFYRA-IAA--SS--Y-HQ--------WT-KIT-TK----
NP_565223.1/1-226    -----------LKMAQRMTQSFYRA-IAA--SS--Y-HQ--------WT-KIT-TK----
NP_001185443.1/1-226 -----------LKMAQRMTQSFYRA-IAA--SS--Y-HQ--------WT-KIT-TK----
XP_010473021.1/1-226 -----------LKMAQRMTQSFYRA-IAA--SS--Y-HQ--------WT-KIT-TK----
XP_010430051.1/1-226 -----------LKMAQRMTQSFYRA-IAA--SS--Y-HQ--------WT-KIT-TK----
XP_025617667.1/1-241 ---------SILKLGERMRWSFCHA-IGA--SS--F-HT--------WT-KVT-SK----
XP_015938429.1/1-242 ---------SILKLGERMRWSFCHA-IGA--SS--F-HT--------WT-KVT-SK----
XP_016176999.1/1-241 ---------SILKLGERMRWSFCHA-IGA--SS--F-HT--------WT-KVT-SK----
XP_021862846.1/1-225 ---------SILKLAHRMTWSFCRA-IGA--ST--F-NT--------WT-KIT-TK----
XP_022979290.1/1-231 -----------LKLAQRMSSSFSQA-IAA--SS--Y-QT--------WT-KVV-GK----
XP_022955725.1/1-233 -----------LKLAQRMSSSFSQA-IAA--SS--Y-QT--------WT-KVV-GK----
XP_022998610.1/1-231 -----------LKLAQRMSSSFSQA-VAA--SS--Y-QT--------WT-KVV-GK----
XP_022998611.1/1-227 -----------LKLAQRMSSSFSQA-VAA--SS--Y-QT--------WT-KVV-GK----
XP_023523626.1/1-229 -----------LKLAERMSSSFSQA-VAA--SS--Y-QT--------WT-KVV-GK----
XP_022948296.1/1-231 ---------SILKLAERMSSSFSQA-VAA--SS--Y-QT--------WT-KVV-GK----
XP_023523628.1/1-225 -----------LKLAERMSSSFSQA-VAA--SS--Y-QT--------WT-KVV-GK----
XP_022948295.1/1-227 ---------SILKLAERMSSSFSQA-VAA--SS--Y-QT--------WT-KVV-GK----
XP_010249693.1/1-228 ---------SILKLAQRMTWSFCRA-VGA--SS--F-HT--------WT-KIS-TK----
XP_010254081.1/1-230 ---------SMLKLAQRMTGSFCRA-VGA--SS--F-NT--------WA-KIS-AK----
XP_003529763.1/1-227 ---------SILKLAQRMTWSFCHA-VGA--SS--F-HT--------WT-KVT-SK----
XP_003533048.1/1-226 ---------SILKLAQRMTWSFCHA-IGA--SS--F-HT--------WT-MVT-SK----
XP_014634625.1/1-226 ---------SILKLAQRMTWSFCHA-IGA--SS--F-HT--------WT-MVT-SK----
XP_007150635.1/1-226 ---------SILKLAQRMTWSFCHA-IGA--SS--F-HT--------WT-KVT-SK----
XP_014498860.1/1-226 ---------SILKLAQRMTWSFCHA-IGA--SS--F-HT--------WT-KVT-SK----
XP_017425661.1/1-226 ---------SILKLAQRMTWSFCHA-IGA--SS--F-HT--------WT-KVT-SK----
XP_020207725.1/1-226 ---------SILKLAQRMTWSFCHA-IGA--SS--F-HT--------WT-KVT-SK----
XP_003546423.2/1-226 ---------SILKLAQRMTWSFCHA-IGA--SS--F-HT--------WT-KFT-SK----
XP_003543638.1/1-226 ---------SILKLAQRMTWSFCHA-IGA--SS--I-HA--------WT-KVT-SK----
XP_019457980.1/1-226 ---------SILKLAQRMTWNFCHA-IGA--SS--F-HK--------WT-KVT-SK----
XP_003597736.1/1-226 ---------SILKLAQRMTWSFCQA-ISA--SS--F-HT--------WT-KVT-SK----
XP_004486751.1/1-226 ---------SILKLAQRMTWSFCHA-IGA--SS--L-HT--------WT-KVT-SK----
XP_016169615.1/1-226 ---------SILKLAQRMTWGFCHA-VGA--SS--F-HT--------WT-KVT-SK----
XP_025611006.1/1-226 ---------SILKLAQRMTWGFCHA-VGA--SS--F-HT--------WT-KVT-SK----
XP_020982478.1/1-227 ---------SILKLAQRMTWGFCHA-VGA--SS--F-HT--------WT-KVT-SK----
XP_021892835.1/1-225 ---------SILKLAQRMTWSFCHA-LGA--SS--Y-HT--------WN-KVS-SK----
XP_012442963.1/1-227 ---------SILKLAQRMTWSFCHS-IGA--SS--Y-HT--------WN-KVS-TK----
NP_001314068.1/1-227 ---------SILKLAQRMTWSFCHS-IGA--SS--Y-HT--------WN-KVS-TK----
XP_016684379.1/1-227 ---------SILKLAQRMTWSFCHS-IGA--SS--Y-HT--------WN-KVS-TK----
XP_016730082.1/1-227 ---------SILKLAQRMTWSFCHS-IGA--SS--Y-HT--------WN-KVS-TK----
NP_001316938.1/1-227 ---------SILKLAQRMTWSFCHS-IGA--SS--Y-HT--------WN-KVS-TK----
XP_017606636.1/1-227 ---------SILKLAQRMTWSFCHS-IGA--SS--Y-HT--------WN-KVS-TK----
XP_022714992.1/1-229 ---------SILKLAQRMTRSFCYA-IGA--SS--Y-HT--------WN-KVS-GK----
XP_022721436.1/1-227 ---------SILKLAQRMTFSFCHS-IGA--SS--Y-HT--------WN-KVT-SK----
XP_022721435.1/1-227 ---------SILKLAQRMTFSFCHS-IGA--SS--Y-HT--------WN-KVT-SK----
XP_007024189.1/1-227 ---------SILKLAQRMTWSFCHA-IGA--SS--Y-NT--------WN-KVP-SK----
XP_021298731.1/1-227 ---------SILKLAQRMTWSFCHA-IGA--SS--Y-HT--------WN-KVP-SK----
XP_004302894.1/1-228 ---------SILKMAQRMTSSFCRA-IGA--SS--Y-HT--------WT-KIT-SK----
XP_011466687.1/1-235 ---------SILKMAQRMTSSFCRA-IGA--SS--Y-HT--------WT-KIT-SK----
XP_024180978.1/1-228 ---------SILKMAQRMTSSFCRA-IGA--SS--Y-HT--------WT-KIS-SK----
XP_024180977.1/1-235 ---------SILKMAQRMTSSFCRA-IGA--SS--Y-HT--------WT-KIS-SK----
XP_009353605.1/1-226 ---------SILKLAQRMTTSFCRA-IGA--SS--Y-HT--------WT-KIS-SK----
XP_008380909.1/1-226 ---------SILKLAQRMTTSFCRA-IGA--SS--Y-HT--------WT-KIS-SK----
NP_001280927.1/1-227 ---------SILKLAQRMTASFCRA-IGA--SS--Y-HT--------WT-KIS-SK----
XP_009338062.1/1-226 ---------SILKLAQRMTASFCRA-IGA--SS--Y-HT--------WT-KIS-SK----
XP_009338046.1/1-226 ---------SILKLAQRMTASFCRA-IGA--SS--Y-HT--------WT-KIS-SK----
XP_021829136.1/1-226 ---------SILKLAQRMTWSFCRA-IGA--SS--Y-HT--------WT-KIS-SK----
XP_008228560.1/1-226 ---------SILKLAQRMTWSFCRA-IGA--SS--Y-HT--------WT-KIS-SK----
XP_007217160.1/1-226 ---------SILKLAQRMTWSFCRA-IGA--SS--Y-HT--------WT-KIS-SK----
XP_022891611.1/1-228 ---------SILKLAQRMTWSFCRA-LGA--SS--Y-NT--------WS-KIP-SK----
XP_002304207.2/1-228 ---------SILKLAQRMTWSFCRA-IGA--SS--Y-HT--------WS-KVS-SK----
XP_011010522.1/1-228 ---------SILKLAQRMTWSFCRA-LGA--SS--Y-HI--------WS-KVS-SK----
XP_011009828.1/1-227 ---------SILKLAQRMTWSFCRA-ICA--SS--Y-HT--------WN-KVS-SK----
XP_024462179.1/1-228 ---------SILKLAQRMTWSFCRA-ICA--SS--Y-HT--------WN-KVS-SK----
XP_018837483.1/1-226 ---------SILKLAQRMTWSFCHA-IGA--SS--F-HT--------WT-KVS-SK----
XP_018856342.1/1-226 ---------SILKLAQRMTCSFCHA-IGA--SS--F-HT--------WT-KVS-SK----
XP_018856994.1/1-226 ---------SILKLAQRMTCSFCHA-IGA--SS--F-HT--------WT-KVS-SK----
XP_018858360.1/1-226 ---------SILKLAQRMTCSFCHA-IGA--SS--F-HT--------WT-KVS-SK----
XP_015867693.1/1-226 ---------SILKLAQRMTSSFCRA-IGA--SS--Y-NI--------WT-KVS-GK----
XP_010062490.1/1-226 ---------SILRLAQRLTQSFCQA-IGA--SS--Y-HS--------WT-KVP-TK----
XP_010098082.1/1-228 ---------SILKLAQRMTWSFCRA-IAA--SS--Y-NT--------WT-KVS-SK----
XP_002284502.1/1-228 ---------SILKLAQRMTWSFCRA-LGA--SS--F-NS--------WT-KVS-SK----
XP_023875609.1/1-226 ---------SILKLAQRMTWNFCRA-IGA--SS--Y-HT--------WT-KVS-SK----
XP_002516023.1/1-226 ---------SILKLAQRMTWSFCRA-IGA--SS--Y-HT--------WN-RVT-SK----
XP_021652755.1/1-228 ---------SILKLAHRMTWSFCRA-IGA--SS--Y-HT--------WN-KVT-SK----
XP_021654904.1/1-226 ---------SILKLAQRMTRSFCRA-IGA--SS--F-HT--------WN-KVT-SK----
XP_021611342.1/1-229 ---------SILKLAQRMTWSFCRA-IGA--SS--F-HT--------WN-KVT-SK----
XP_012073376.1/1-226 ---------SILKLAQRMTWSFCRA-IGA--SS--Y-HT--------WN-KVT-SK----
XP_006465622.1/1-226 ---------SILKLAQRMTWSFCRA-IGA--SS--Y-HV--------WN-KVT-SK----
XP_006426950.1/1-226 ---------SILKLAQRMTWSFCRA-IGA--SS--Y-HV--------WN-KVT-SK----
XP_009392419.1/1-229 ---------SILKLGQRMTSIFCQN-IGA--SG--H-RT--------WT-KVS-TK----
XP_020518333.1/1-227 ---------SILKLAQRMTSSFCRG-LGA--SS--L-NS--------WT-KLS--K----
XP_018629167.1/1-227 ---------SILKLAQRMTWSFCHA-IGA--SS--Y-NK--------WD-KIH-SK----
XP_009611250.1/1-227 ---------SILKLAQRMTWSFCHA-IGA--SS--Y-NK--------WD-KIH-SK----
XP_009611249.1/1-227 ---------SILKLAQRMTWSFCHA-IGA--SS--Y-NK--------WD-KIH-SK----
XP_009611248.1/1-227 ---------SILKLAQRMTWSFCHA-IGA--SS--Y-NK--------WD-KIH-SK----
XP_016479156.1/1-227 ---------SILKLAQRMTWSFCHA-IGA--SS--Y-NK--------WD-KIH-SK----
XP_016479155.1/1-227 ---------SILKLAQRMTWSFCHA-IGA--SS--Y-NK--------WD-KIH-SK----
XP_016479154.1/1-227 ---------SILKLAQRMTWSFCHA-IGA--SS--Y-NK--------WD-KIH-SK----
XP_009778213.1/1-229 ---------SILTLAQRMTWNFYRM-LGA--SS--Y-NT--------WN-KIP-SK----
XP_016501674.1/1-229 ---------SILTLAQRMTWNFYRM-LGA--SS--Y-NT--------WN-KIP-SK----
XP_019224885.1/1-229 ---------SILTLAQRMTWSFYRM-LGA--SS--Y-NT--------WN-KIP-SK----
XP_019224886.1/1-229 ---------SILTLAQRMTWSFYRM-LGA--SS--Y-NT--------WN-KIP-SK----
XP_009592772.1/1-229 ---------SILTLAQRMTWSFYRM-LGA--SS--Y-NT--------WN-KIP-SK----
XP_016496403.1/1-229 ---------SILTLAQRMTWSFYRM-LGA--SS--Y-NT--------WN-KIP-SK----
XP_016562690.1/1-228 ---------SILTLAQRMTWSFYRM-LGA--SS--Y-NT--------WN-KVP-SK----
XP_006343080.1/1-229 ---------SILTLAQRMTWGFYRV-LGA--SS--Y-NT--------WN-KVP-SK----
XP_004235676.1/1-229 ---------SILTLAQRMTRGFYRV-LGA--SS--Y-NT--------WN-KIP-SK----
XP_020227921.1/1-228 ---------SILKLAQRMTWSFCHA-VGA--SS--F-HT--------WT-KVT-SK----
XP_007135673.1/1-226 ---------SILKLAQRMTWSFCHA-VGA--SS--F-HT--------WT-KFT-SK----
XP_014515030.1/1-226 ---------SILKLAQRMTWSFCHA-VGA--SS--F-HT--------WT-KFT-SK----
XP_017407449.1/1-226 ---------SILKLAQRMTWSFCHA-IGA--SS--F-HT--------WT-KFT-SK----
XP_010691342.1/1-224 ---------SILKLAQRMTWSFCRA-VGA--SN--F-NS--------WT-KIT-TK----
XP_021731998.1/1-222 -----------LKLAQRMTWSFCRA-LGA--ST--S-NS--------WT-KIT-TK----
XP_021736870.1/1-224 ---------SILKLAQRMTWSFCRA-LGA--ST--S-NS--------WT-KIT-TK----
XP_024969955.1/1-229 ---------SILKLAQRMTWSFGRA-LGA--SS--H-CT--------WK-KIL-SK----
XP_024969954.1/1-229 ---------SILKLAQRMTWSFGRA-LGA--SS--H-CT--------WK-KIL-SK----
XP_024969953.1/1-229 ---------SILKLAQRMTWSFGRA-LGA--SS--H-CT--------WK-KIL-SK----
XP_021984354.1/1-227 ---------SILKLAQRMTSSFSRA-LGA--SS--Y-HT--------WK-KIP-SK----
XP_017232370.1/1-228 ---------SILKLAQRMTRSFYRA-LGA--SN--Y-QS--------WN-KAT-SK----
XP_017216573.1/1-228 ---------SILKLAQRMTKSFYRA-LGA--SN--Y-HT--------WN-KTT-SK----
XP_017248777.1/1-227 ---------SILNLAQRMTKSFYRA-LGA--SS--R-NT--------WN-KAP-SK----
XP_021627148.1/1-226 ---------SILKLAQRMTWSFCRA-IGA--SS--Y-HS--------WN-KVT-SK----
XP_021627146.1/1-226 ---------SILKLAQRMTWSFCRA-IGA--SS--Y-HS--------WN-KVT-SK----
XP_021627145.1/1-226 ---------SILKLAQRMTWSFCRA-IGA--SS--Y-HS--------WN-KVT-SK----
XP_019196965.1/1-228 ---------SILTLAQRMCLAFFRA-LGA--SS--Y-NT--------WN-KIP-TK----
XP_019196967.1/1-228 ---------SILTLAQRMCLAFFRA-LGA--SS--Y-NT--------WN-KIP-TK----
XP_022848789.1/1-228 ---------SILKLAQRMTWSYCRA-LGA--SS--Y-NT--------WS-KIP-SK----
XP_011072373.1/1-228 ---------SILKLAHRMTWSFCRA-LGG--SS--H-NS--------WN-KIT-SK----
XP_021907286.1/1-215 --------AGMLKLAERMVSMYFNG-VSA--SN--N-TG--------WA-KLL-------
XP_020885309.1/1-217 ---------SMLKIAERVARSFFNG-LTS--SM--G-S---------T---FS-------
XP_020885307.1/1-217 ---------SMLKIAERVARSFFNG-LTS--SM--G-S---------T---FS-------
NP_180796.2/1-218    ---------SMLKIAERIARTFFAG-MTN--AT--G-ST--------I---FS-------
XP_006296541.1/1-217 ---------SILQLVERIARGFFIG-LKN--SM--G-T---------T---FS-------
XP_010510028.1/1-215 ---------SILKLAERVSRNFFVG-LTS--SM--G-T---------T---FS-------
XP_010414006.1/1-214 ---------SILKLAERVTRSFFVG-LTS--SM--G-T---------T---FS-------
XP_010469602.1/1-219 ---------SILKLAERVARSFFVG-LTS--SM--G-T---------T---FS-------
XP_010417818.1/1-218 ---------SILKLAHRIAKTFFIG-LTN--SM--G-S---------T---CS-------
XP_010510869.1/1-220 ---------SILKLAERVARSFFIG-LTN--SM--G-T---------P---IS-------
XP_010510870.1/1-218 ---------SILKLAERVARSFFIG-LTN--SM--G-T---------P---IS-------
XP_018484769.1/1-219 ---------SLMKIAERMTRSFLSG-VAN--SA--G-DT--------WF-KLP-------
XP_018484754.1/1-219 ---------SLMKIAERMTRSFLSG-VAN--SA--G-DT--------WF-KLP-------
XP_013634882.1/1-219 ---------SLLKIAERMTRSFLTG-VAT--SN--G-DI--------WF-RLS-------
XP_018513778.1/1-219 ---------SLLKIAERMTRSFLSG-VAT--SN--G-DI--------WF-GLS-------
XP_009141217.1/1-219 ---------SLLKIAERMTRSFLSG-VAT--SN--G-DI--------WF-GLS-------
XP_013747219.1/1-219 ---------SLLKIAERMTRSFLSG-VAT--SN--G-DI--------WF-GLS-------
XP_024003849.1/1-217 ----------ILKLAERMGRSFFSG-VTA--ST--A-DL--------WC-NLS-------
XP_018437853.1/1-211 ---------SLLKLVERMSRGFFDG-VTA--ST--A-DV--------WC-NLP-------
XP_009143975.1/1-211 ---------SLLELVERMSRGFFDG-VTT--SN--A-DI--------WM-NLG-------
XP_009143974.1/1-211 ---------SLLELVERMSRGFFDG-VTT--SN--A-DI--------WM-NLG-------
XP_022575029.1/1-211 ---------SLLELVERMSRGFFDG-VTT--SN--A-DI--------WM-NLG-------
XP_013748684.1/1-211 ---------SLLELVERMSRGFFDG-VTT--SN--A-DI--------WM-NLG-------
XP_013694194.1/1-211 ---------SLLNLVERMSRGFFDG-VTT--SN--A-DI--------WM-NLG-------
XP_013634261.1/1-211 ---------SLLKLVERMSRGFFDG-VTT--ST--A-DI--------WW-NLA-------
XP_013741430.1/1-210 ---------SLLKLVERMSRGFFDG-VTT--ST--A-DI--------WW-K-G-------
XP_017254373.1/1-221 ---------SLLKLAERMVLTYCTT-AGV--ST--A-HM--------WT-TYC-------
XP_017254240.1/1-222 -----------LKLSKRMMLSYCNS-FGV--SA--A-HT--------CN-TLC-------
XP_017221582.1/1-224 ---------SMLKLAERMVLGYCNG-VGV--ST--E-HM--------WT-MFC-------
XP_017221584.1/1-224 ---------SMLKLAERMVLGYCNG-VGV--ST--E-HM--------WT-MFC-------
XP_015941575.1/1-215 ---------GLVKLAERMVMDFLSG-VGA--ST--V-EN--------WA-IVT-------
XP_025620358.1/1-215 ---------GLVKLAERMVMDFLSG-VGA--ST--V-EN--------WA-IVT-------
XP_016176493.1/1-215 ---------GLVKLAERMVMDFLSG-VGA--ST--V-EN--------WA-IVT-------
XP_003610661.2/1-228 ---------SILNLAERLVASFSTS-IGS--ST--T-HA--------WT-KVP-------
XP_004496853.1/1-220 ---------SLMRLTDRMKVYFSTV-AGS--SI--P-NC--------WE-VLT-------
XP_006353342.1/1-230 -------------LARRVTRSFCVG-VCA--T---Y-YD--------WE-SIQ-------
XP_015070591.1/1-229 -------------LARRVIRHFCIG-VCA--T---Y-YN--------WE-SIQ-------
XP_019068356.1/1-229 -------------LARRVTRHFCNG-VCA--T---Y-YK--------WE-SIQ-------
XP_004234441.1/1-229 -------------LARRVTRHFCNG-VCA--T---Y-YK--------WE-SIQ-------
XP_024926606.1/1-217 ---------SLLKLSERMMRSFFAD-VGG--GT--L-NN--------WT-SLP-------
XP_024525400.1/1-231 -----PARQSLLKLSQRMTTNFCAG-VGA--P---S-SQ--------WT-TLS-------
XP_002965186.2/1-231 -----PARQSLLKLSQRMTTNFCAG-VGA--P---S-SQ--------WT-TLS-------
XP_002962272.2/1-231 -----PARQSLLKLSQRMTTNFCAG-VGA--P---S-SQ--------WT-TLS-------
XP_019441536.1/1-226 ------------KLAERMKWSFLTG-VGS--ST--A-DI--------WT-TIS-------
XP_019441537.1/1-226 ------------KLAERMKWSFLTG-VGS--ST--A-DI--------WT-TIS-------
XP_019441535.1/1-226 ------------KLAERMKWSFLTG-VGS--ST--A-DI--------WT-TIS-------
XP_019451758.1/1-229 ---------SILKLAERMLMSFFTG-VGS--ST--S-DV--------WS-ALS-------
XP_012850835.1/1-238 ---------SLLKLAERMIGRFCNG-VNA--SI--N-NT--------WT-TLS-------
XP_020552079.1/1-225 ---------SILKLGERMVTRFCSG-VNA--ST--G-NS--------WT-TLS-------
XP_020552078.1/1-225 ---------SILKLGERMVTRFCSG-VNA--ST--G-NS--------WT-TLS-------
XP_020552080.1/1-223 ---------SILKLGERMVTRFCSG-VNA--ST--G-NS--------WT-TLS-------
XP_009408807.2/1-226 ----------ILKLAERMVVSFCSG-VSG--SV--A-HH--------WT-NIS-------
XP_006342404.1/1-225 -----------LKLGERMIIDYISG-VSG--TT--T-HQ--------WT-TFT-RS----
XP_025887998.1/1-218 -----------LKLGERMIINYISG-VSG--TK--T-HK--------WT-TFT-GS----
XP_015081481.1/1-225 -----------LKLGERMIINYISG-VSG--TK--T-HK--------WT-TFT-ES----
XP_022000230.1/1-232 ---PAVGKRSLTNLAQRMTANFCAG-VCA--TG--G-HK--------WD-VIS-------
XP_022000229.1/1-232 ---PAVGKRSLTNLAQRMTANFCAG-VCA--TG--G-HK--------WD-VIS-------
XP_022037790.1/1-222 -------------LAQRMTANFCAG-VCA--TG--G-HK--------WE-VVS-------
XP_022037789.1/1-222 -------------LAQRMTANFCAG-VCA--TG--G-HK--------WE-VVS-------
XP_024991925.1/1-226 -------------LAQRMTANFCAG-VCA--TG--G-HK--------WE-VIG-------
XP_023760005.1/1-229 ---------SLTALAQRMTSNFCAG-VCA--TG--G-HK--------WE-VIG-------
XP_023760001.1/1-229 ---------SLTALAQRMTSNFCAG-VCA--TG--G-HK--------WE-VIG-------
XP_017251723.1/1-236 ---------SLAKLAQRMNRIFCAG-VCA--T---G-NK--------WE-VVQ-LK----
XP_017243321.1/1-239 --------ASMAKLAQRMTRNFCAG-VCA--T---V-HN--------WE-VVQ-VG----
XP_017243319.1/1-239 --------ASMAKLAQRMTRNFCAG-VCA--T---V-HN--------WE-VVQ-VG----
XP_017243320.1/1-239 --------ASMAKLAQRMTRNFCAG-VCA--T---V-HN--------WE-VVQ-VG----
XP_017255563.1/1-234 ---------SMAKLAQRMTRNFCAG-VCA--T---K-HK--------WE-VVQ-VG----
XP_017255562.1/1-234 ---------SMAKLAQRMTRNFCAG-VCA--T---K-HK--------WE-VVQ-VG----
XP_012852237.1/1-226 ---------SMVKLAQRMTRNFCSV-ICS--T---V-HK--------WE-VVQ-SS----
XP_012852238.1/1-226 ---------SMVKLAQRMTRNFCSV-ICS--T---V-HK--------WE-VVQ-SS----
XP_012852236.1/1-226 ---------SMVKLAQRMTRNFCSV-ICS--T---V-HK--------WE-VVQ-SS----
XP_012852235.1/1-226 ---------SMVKLAQRMTRNFCSV-ICS--T---V-HK--------WE-VVQ-SS----
XP_011076208.1/1-227 -------------LAQRMTRSFCTG-VCA--T---V-HK--------WE-VVQ-SS----
XP_019200484.1/1-228 -------------LAQRMTRSFCAV-VCG--N---I-YK--------WE-PIQ-TG----
XP_019200481.1/1-228 -------------LAQRMTRSFCAV-VCG--N---I-YK--------WE-PIQ-TG----
XP_022879034.1/1-223 -----------------MTRSFCNS-VCA--T---V-HK--------WE-LVQ-SG----
XP_018623115.1/1-238 ---------SMAMLAQRMTRNFCAG-VCA--T---I-HK--------WE-SIQ-QA----
XP_018623116.1/1-238 ---------SMAMLAQRMTRNFCAG-VCA--T---I-HK--------WE-SIQ-QA----
XP_016449095.1/1-238 ---------SMAMLAQRMTCNFCAG-VCA--T---I-HK--------WE-SIQ-QA----
XP_009771835.1/1-238 ---------SMAMLAQRMTRNFCAG-VCA--T---I-HK--------WE-SIQ-QA----
XP_009771833.1/1-238 ---------SMAMLAQRMTRNFCAG-VCA--T---I-HK--------WE-SIQ-QA----
XP_019248454.1/1-225 ---------SMAMLAQRMTRNFCAG-VCA--T---I-HK--------WE-SIQ-QA----
XP_016564677.1/1-235 ----PSGRRSIAMLARRVTCNFCAG-VCG--T---F-YK--------WE-PIQ-SG----
XP_016564676.1/1-235 ----PSGRRSIAMLARRVTCNFCAG-VCG--T---F-YK--------WE-PIQ-SG----
XP_009606534.1/1-237 ----PSGRRSIAMLARRITRNFCGG-VCA--T---F-YK--------WE-PIQ-TG----
XP_016457239.1/1-237 ----PSGRRSIAMLARRITRNFCGG-VCA--T---F-YK--------WE-PIQ-TG----
XP_009606533.1/1-237 ----PSGRRSIAMLARRITRNFCGG-VCA--T---F-YK--------WE-PIQ-TG----
XP_009606532.1/1-237 ----PSGRRSIAMLARRITRNFCGG-VCA--T---F-YK--------WE-PIQ-TG----
XP_016457238.1/1-237 ----PSGRRSIAMLARRITRNFCGG-VCA--T---F-YK--------WE-PIQ-TG----
XP_016468064.1/1-237 ----PSGRRSIAMLARRITRNFCGG-VCA--T---F-YK--------WE-PIQ-TG----
XP_009774404.1/1-237 ----PSGRRSIAMLARRITRNFCGG-VCA--T---F-YK--------WE-PIQ-TG----
XP_016468062.1/1-237 ----PSGRRSIAMLARRITRNFCGG-VCA--T---F-YK--------WE-PIQ-TG----
XP_016468061.1/1-237 ----PSGRRSIAMLARRITRNFCGG-VCA--T---F-YK--------WE-PIQ-TG----
XP_009774402.1/1-237 ----PSGRRSIAMLARRITRNFCGG-VCA--T---F-YK--------WE-PIQ-TG----
XP_009774401.1/1-237 ----PSGRRSIAMLARRITRNFCGG-VCA--T---F-YK--------WE-PIQ-TG----
XP_019251817.1/1-237 ----PSGRRSIAMLARRITRNFCGG-VCA--T---F-YK--------WE-PIQ-TG----
XP_019251815.1/1-237 ----PSGRRSIAMLARRITRNFCGG-VCA--T---F-YK--------WE-PIQ-TG----
XP_019251816.1/1-237 ----PSGRRSIAMLARRITRNFCGG-VCA--T---F-YK--------WE-PIQ-TG----
XP_011470395.1/1-233 ---------SMLRLAQRMMQSFCSG-VCG--SL--V-HQ--------WD-KLC-ID----
XP_004309219.2/1-233 ---------SMLRLAQRMMQSFCSG-VCG--SL--V-HQ--------WD-KLC-ID----
XP_024180881.1/1-233 ---------SMLRLAQRMMQSFCSG-VCG--SS--V-RQ--------WD-KLC-ID----
XP_024180911.1/1-233 ---------SMLRLAQRMMQSFCSG-VCG--SS--V-RQ--------WD-KLC-ID----
XP_019450525.1/1-216 ---------GMMKLAQRITNSFFTG-LCA--SA--A-HQ--------WE-SLC-FD----
XP_019465255.1/1-230 ---------SMVKLAQRITNKFFSG-VCF--ST--A-HQ--------WE-PLH-FD----
XP_014502466.1/1-227 -----------LKLAQRMNEYFCSG-ICA--SS--A-RK--------WD-VVH-IG----
XP_017438850.1/1-222 -----------LKLAQRMNEYFCSG-ICA--SS--A-RK--------WD-VLH-IG----
XP_006599623.1/1-221 ----------MMKLAQRMTECFCSG-ICA--SS--A-CK--------WD-ILH-IG----
XP_006599622.1/1-221 ----------MMKLAQRMTECFCSG-ICA--SS--A-CK--------WD-ILH-IG----
XP_006599621.1/1-221 ----------MMKLAQRMTECFCSG-ICA--SS--A-CK--------WD-ILH-IG----
XP_004515730.1/1-240 ---------SMLKLAQRMTDYFWSG-VCP--ST--A-CK--------WD-ILH-IS----
XP_013442370.1/1-239 ---------SMLKLAQRMSDYFWSG-ICP--ST--A-CK--------WD-ILP-IS----
XP_004252035.1/1-225 -----------LKLAERMINCFSSG-VSA--TV--G-NQ--------WT-TVS-AG----
XP_015060726.1/1-225 -----------LKLAERMINIFSSG-VSA--TV--G-NQ--------WT-TVS-AG----
XP_006589608.1/1-229 ---------SMMKLAERMVLSFSTG-VGA--ST--A-NA--------WT-PLP-L-----
XP_006589605.1/1-230 ---------SMMKLAERMVLSFSTG-VGA--ST--A-NA--------WT-PLP-L-----
XP_006849468.1/1-231 ---------SMMKLAQRMINNFCSS-ISA--SS--S-HR--------WT-ALS-GLN---
XP_020526173.1/1-231 ---------SMMKLAQRMINNFCSS-ISA--SS--S-HR--------WT-ALS-GLN---
XP_020109515.1/1-228 ---------SMMKLSQRMVNNFCAS-LSA--SH--M-HR--------WT-TLS-GLN---
NP_001310555.1/1-224 ---------SMMKLSQRMVNSFCAS-LSA--SQ--L-HR--------WT-TLS-GPN---
XP_010544375.1/1-225 ---------GMMKLAQRMTYKFCSG-ISAP-PS--V-HG--------WR-KLT-V-----
NP_001314001.1/1-230 ---------SMLKLAQRMAYNFSAG-IGA--SS--V-NK--------WD-KLN-V-----
XP_016732454.1/1-230 ---------SMLKLAQRMAYNFSAG-IGA--SS--V-NK--------WD-KLN-V-----
XP_016732453.1/1-230 ---------SMLKLAQRMAYNFSAG-IGA--SS--V-NK--------WD-KLN-V-----
XP_012484067.1/1-230 ---------SMLKLAQRMAYNFSAG-IGA--SS--V-NK--------WD-NLN-V-----
XP_012484066.1/1-230 ---------SMLKLAQRMAYNFSAG-IGA--SS--V-NK--------WD-NLN-V-----
XP_017631791.1/1-230 ---------SMLKLAQRMAYNFSAG-IGA--SS--V-NK--------WD-KLN-V-----
XP_017631789.1/1-230 ---------SMLKLAQRMAYNFSAG-IGA--SS--V-NK--------WD-KLN-V-----
XP_016728129.1/1-230 ---------SMLKLAQRMAYNFSAG-IGA--SS--V-NK--------WD-KLN-V-----
XP_016728128.1/1-230 ---------SMLKLAQRMAYNFSAG-IGA--SS--V-NK--------WD-KLN-V-----
XP_021671684.1/1-240 ---------SMVKLAQRMVDNFCSG-VCA--SS--L-HK--------WD-KLL-V-----
XP_021671683.1/1-240 ---------SMVKLAQRMVDNFCSG-VCA--SS--L-HK--------WD-KLL-V-----
XP_021671685.1/1-240 ---------SMVKLAQRMVDNFCSG-VCA--SS--L-HK--------WD-KLL-V-----
XP_002318231.3/1-240 ---------SMLKLARRMVDNFCSG-VCA--SS--L-HN--------WG-NLV-A-----
XP_024438602.1/1-240 ---------SMLKLARRMVDNFCSG-VCA--SS--L-HN--------WG-NLV-A-----
XP_024442055.1/1-239 ---------SMLKLARRMVDSFCSG-VCA--ST--L-HN--------WG-NLV-V-----
XP_002322460.1/1-239 ---------SMLKLARRMVDSFCSG-VCA--ST--L-HN--------WG-NLV-V-----
XP_011048682.1/1-239 ---------SMLKLARRMVDSFCSG-VCA--ST--L-HN--------WA-NLV-V-----
XP_011048684.1/1-239 ---------SMLKLARRMVDSFCSG-VCA--ST--L-HN--------WA-NLV-V-----
XP_011048683.1/1-239 ---------SMLKLARRMVDSFCSG-VCA--ST--L-HN--------WA-NLV-V-----
XP_019081404.1/1-234 ---------SLLQLTNRMRDNFCAG-VCA--ST--V-RM--------WN-KLH-V-----
XP_003634081.1/1-234 ---------SLLQLTNRMRDNFCAG-VCA--ST--V-RM--------WN-KLH-V-----
XP_008377249.1/1-231 ---------SMLKLAQRMVDNFCAG-ISA--SS--V-RK--------WD-KLC-F-----
XP_017189459.1/1-231 ---------SMLKLAQRMVDNFCAG-ISA--SS--V-RK--------WD-KLC-F-----
XP_008377244.1/1-231 ---------SMLKLAQRMVDNFCAG-ISA--SS--V-RK--------WD-KLC-F-----
XP_009371302.1/1-231 ---------SMLKLAQRMVDNFCAG-ISA--SS--V-RK--------WD-KLC-F-----
XP_009371299.1/1-231 ---------SMLKLAQRMVDNFCAG-ISA--SS--V-RK--------WD-KLC-F-----
XP_008376545.1/1-231 ---------SMLKLAQRMVDNFCAG-ISA--SS--V-RK--------WD-KLC-F-----
XP_008376544.1/1-231 ---------SMLKLAQRMVDNFCAG-ISA--SS--V-RK--------WD-KLC-F-----
XP_017188609.1/1-231 ---------SMLKLAQRMVDNFCAG-ISA--SS--V-RK--------WD-KLC-F-----
XP_022945426.1/1-238 ---------SMLKLSQRMVDNFCSG-ICT--ST--L-HK--------WD-KLV-V-----
XP_022945425.1/1-238 ---------SMLKLSQRMVDNFCSG-ICT--ST--L-HK--------WD-KLV-V-----
XP_023541229.1/1-238 ---------SMLKLSQRMVDNFCSG-ICT--ST--L-QK--------WD-KLV-V-----
XP_023541228.1/1-238 ---------SMLKLSQRMVDNFCSG-ICT--ST--L-QK--------WD-KLV-V-----
XP_022968414.1/1-238 ---------SMLKLSQRMVDNFCSG-ICT--ST--L-HK--------WD-KLV-V-----
XP_022968413.1/1-238 ---------SMLKLSQRMVDNFCSG-ICT--ST--L-HK--------WD-KLV-V-----
XP_023552381.1/1-232 ---------SMLKLSQRMVDKFCSG-LCS--SS--L-HK--------WD-KLV-V-----
XP_022922479.1/1-232 ---------SMLKLSQRMVDKFCSG-LCS--SS--L-HK--------WD-KLV-V-----
XP_023552380.1/1-232 ---------SMLKLSQRMVDKFCSG-LCS--SS--L-HK--------WD-KLV-V-----
XP_022922478.1/1-232 ---------SMLKLSQRMVDKFCSG-LCS--SS--L-HK--------WD-KLV-V-----
XP_022984644.1/1-232 ---------SMLKLSQRMVDKFCSG-LCS--SS--L-HK--------WD-KLV-V-----
XP_022984643.1/1-232 ---------SMLKLSQRMVDKFCSG-LCS--SS--L-HK--------WD-KLV-V-----
XP_008439185.1/1-242 ----PSGRRSMLKLSQRMVDNFCSG-VCS--ST--L-HK--------WD-KLV-V-----
XP_008439184.1/1-242 ----PSGRRSMLKLSQRMVDNFCSG-VCS--ST--L-HK--------WD-KLV-V-----
XP_011651140.1/1-242 ----PSGRRSMLKLSQRMVDNFCSG-VCS--ST--L-HK--------WD-KLV-V-----
XP_004140784.1/1-242 ----PSGRRSMLKLSQRMVDNFCSG-VCS--ST--L-HK--------WD-KLV-V-----
XP_009391954.1/1-234 ---------SMLKLARRMTNAFCAG-VCA--SP--A-HG--------WS-KLA-S-----
XP_003581542.2/1-227 ---------SLLKLAQRMMESFFSG-VSA--SS--A-LE--------WS-KLD-GFT---
XP_006652658.1/1-224 ---------SMLKLARRMTKNFCAG-VST--SS--A-RE--------WS-KLD-GLT---
XP_015637147.1/1-225 ---------SMLKLARRMTDNFCAG-VST--SS--T-RE--------WS-KLV-GLT---
XP_025823430.1/1-225 ---------SLLRLAQRMMENFCAG-VSA--S---S-AE--------WN-KLD-GLT---
XP_004978185.1/1-225 ---------SLLKLAHRMMENFGAG-VSG--S---S-AE--------WS-KLD-GLT---
XP_021317935.1/1-228 ---------SLLKLARRMMENFCAG-MSA--SS--S-CE--------WS-ILD-GLT---
NP_001336434.1/1-226 ---------SLLKLARRMMENFCAG-MGA--SS--S-RE--------WS-MLD-GFT---
XP_019709552.1/1-234 ---------SMLRLARRMTDSFCAG-VCA--WA--T-HG--------WR-ELT-A-----
XP_015696802.1/1-257 ---------SMLKLAQRMTDNFCAG-VCA--SA--A-QK--------WR-RLD-EWRGGE
XP_015612621.1/1-258 ---------SMLKLAQRMTDNFCAG-VCA--SA--A-QK--------WR-RLD-EWRGEG
XP_004957413.1/1-253 ---------SMLKLAQRMTDNFCAG-VCA--SA--A-QK--------WR-RLD-EWRGEG
XP_025801167.1/1-251 ---------SMLKLAQRMTDNFCAG-VCA--SA--A-QK--------WR-RLD-EWRGGE
NP_001105125.2/1-256 ---------SMLKLAQRMTDNFCAG-VCA--SA--A-QK--------WR-RLD-EWRS--
XP_002462701.1/1-258 ---------SMLKLAQRMTDNFCAG-VCA--SA--A-QK--------WR-RLD-EWRGVG
XP_020262629.1/1-222 ----PAGRRSMLKLAQRMMDNFCAG-VCA--SS--S-RK--------WR-RFV-SV----
XP_020699782.1/1-235 ---------SMLRLAGRMTENFCAG-VCA--WA--E-RK--------WR-ELR-SV----
XP_020699776.1/1-235 ---------SMLRLAGRMTENFCAG-VCA--WA--E-RK--------WR-ELR-SV----
XP_020573166.1/1-235 ---------SMMRLAGRMTENFCAG-VCA--WA--E-RK--------WR-ELR-SA----
XP_020187432.1/1-228 ---------SLLRLAERMMDNFCAG-VSA--SS--A--E--------WS-KLD-DV----
XP_025656795.1/1-233 ----PAGRRSMLKLAQRMANNFFSG-ICL--SS--S-QK--------WD-THH-LG----
XP_025656792.1/1-233 ----PAGRRSMLKLAQRMANNFFSG-ICL--SS--S-QK--------WD-THH-LG----
XP_025656794.1/1-233 ----PAGRRSMLKLAQRMANNFFSG-ICL--SS--S-QK--------WD-THH-LG----
XP_020979736.1/1-233 ----PAGRRSMLKLAQRMANNFFSG-ICL--SS--S-QK--------WD-THH-LG----
XP_020999151.1/1-233 ----PAGRRSMLKLAQRMANNFFSG-ICL--SS--S-QK--------WD-THH-LG----
XP_015967451.1/1-233 ----PAGRRSMLKLAQRMANNFFSG-ICL--SS--S-QK--------WD-THH-LG----
XP_020999150.1/1-233 ----PAGRRSMLKLAQRMANNFFSG-ICL--SS--S-QK--------WD-THH-LG----
XP_025701970.1/1-233 ----PAGRRSMLKLAQRMANNFFSG-ICL--SS--S-QK--------WD-THH-LG----
XP_025701968.1/1-233 ----PAGRRSMLKLAQRMANNFFSG-ICL--SS--S-QK--------WD-THH-LG----
XP_025701969.1/1-233 ----PAGRRSMLKLAQRMANNFFSG-ICL--SS--S-QK--------WD-THH-LG----
XP_020221720.1/1-222 ---------SMLKLAQRMAEYFCSG-ICA--SS--A-RK--------WD-ILP-IG----
XP_007152327.1/1-222 ---------SIMKLAQRMTECFCSG-ICA--SS--A-RK--------WD-VLH-IG----
XP_006587345.1/1-222 ----------MLKLAQRMTEYFCSG-ICA--SS--V-RK--------WE-ILN-IG----
XP_006587344.1/1-222 ----------MLKLAQRMTEYFCSG-ICA--SS--V-RK--------WE-ILN-IG----
XP_010035104.1/1-239 ----PSGRRNMLKLAQRMTDNFCSG-VCA--SP--L-HN--------WE-VLH-LG----
XP_021765162.1/1-234 ---------SILKLMRRISNNFSSG-LCA--SS--A-CR--------WE-KLQ-VG----
XP_021765160.1/1-234 ---------SILKLMRRISNNFSSG-LCA--SS--A-CR--------WE-KLQ-VG----
XP_021730123.1/1-234 ---------SILKLMRRISNNFSSG-LCA--SS--A-CR--------WE-KLQ-VG----
XP_021842103.1/1-232 ---------SILKLMRRISNNFCSG-LCA--SS--A-CR--------WE-KLQ-VG----
XP_021842106.1/1-232 ---------SILKLMRRISNNFCSG-LCA--SS--A-CR--------WE-KLQ-VG----
XP_021842105.1/1-232 ---------SILKLMRRISNNFCSG-LCA--SS--A-CR--------WE-KLQ-VG----
XP_021842104.1/1-232 ---------SILKLMRRISNNFCSG-LCA--SS--A-CR--------WE-KLQ-VG----
XP_010693471.1/1-235 ---------SILKLMRRISNNFCSG-LCA--SS--A-CR--------WE-KLQ-VG----
XP_019107938.1/1-235 ---------SILKLMRRISNNFCSG-LCA--SS--A-CR--------WE-KLQ-VG----
XP_010693469.1/1-235 ---------SILKLMRRISNNFCSG-LCA--SS--A-CR--------WE-KLQ-VG----
XP_019107937.1/1-235 ---------SILKLMRRISNNFCSG-LCA--SS--A-CR--------WE-KLQ-VG----
XP_020163116.1/1-258 ----PAGRRCMLRLAQRMADNFCAG-VCA--TA--A-QK--------WR-RLD-EWRVEG
XP_020163109.1/1-258 ----PAGRRCMLRLAQRMADNFCAG-VCA--TA--A-QK--------WR-RLD-EWRVEG
XP_003578475.1/1-267 ---------SMLKLAQRMADNFCAG-VCA--TA--A-QK--------WR-RLD-EWRVEG
XP_011012369.1/1-242 ---------SMLKLARRMVDNFCSG-VCA--SS--L-HK--------WG-NPV-AG----
XP_011012370.1/1-242 ---------SMLKLARRMVDNFCSG-VCA--SS--L-HK--------WG-NPV-AG----
XP_008234278.1/1-230 ---------SMLKLAQRMIDNFCAG-VSA--SS--V-RN--------WD-KLC-VN----
XP_021827117.1/1-230 ---------SMLKLAQRMIDNFCAG-VSA--SS--V-RK--------WD-KLC-VN----
XP_021827116.1/1-230 ---------SMLKLAQRMIDNFCAG-VSA--SS--V-RK--------WD-KLC-VN----
XP_020413581.1/1-230 ---------SMLKLAQRMIDNFCAG-VSA--SS--V-RK--------WD-KLC-VN----
XP_007219555.2/1-230 ---------SMLKLAQRMIDNFCAG-VSA--SS--V-RK--------WD-KLC-VN----
XP_018456926.1/1-230 ------------TLAQRMKLSFYSG-ITA--SP--I-HK--------WE-KLV-AE----
XP_013613235.1/1-226 -----------LTLAQRMRSNFYSG-ITA--SP--I-HK--------WE-KLV-AE----
XP_013686466.1/1-227 -----------LTLAQRMKRNFYSG-ITG--SP--I-HK--------WE-KLV-AE----
XP_009106897.1/1-227 -----------LTLAQRMKRNFYSG-ITG--SP--I-HK--------WE-KLV-AE----
XP_006401857.1/1-229 ---------SILTLAQRMKRNFYSG-ITA--SS--I-HK--------WE-KLL-AE----
XP_020871109.1/1-230 ---------SILKLAQRMKLNFYSG-ITA--SC--I-HK--------WE-KLL-AE----
NP_001332093.1/1-229 ---------SILKLAQRMKLNFYSG-ITA--SC--I-HK--------WE-KLL-AE----
NP_200030.1/1-229    ---------SILKLAQRMKLNFYSG-ITA--SC--I-HK--------WE-KLL-AE----
XP_006282099.1/1-225 ---------SILKLAQRMKLNFYSG-ITA--SS--V-HK--------WE-KLN-AE----
XP_019095386.1/1-229 ---------SILKLAQRMKVNFYSG-ITA--SS--V-HK--------WE-KLN-AE----
XP_019095387.1/1-229 ---------SILKLAQRMKVNFYSG-ITA--SS--V-HK--------WE-KLN-AE----
XP_019091174.1/1-228 ---------SILKLAQRMKVNFYSG-ITA--SS--V-HK--------WE-KLN-AE----
XP_019091179.1/1-228 ---------SILKLAQRMKVNFYSG-ITA--SS--V-HK--------WE-KLN-AE----
XP_019087173.1/1-228 ---------SILKLAQRMKLNFYSG-ITA--SS--V-HK--------WE-RLN-AE----
XP_010555654.1/1-234 ----PAGEKSILKLAQRMAVNFYAG-ISR--SS--V-RK--------WD-KLL-VA----
XP_010555656.1/1-234 ----PAGEKSILKLAQRMAVNFYAG-ISR--SS--V-RK--------WD-KLL-VA----
XP_010555655.1/1-234 ----PAGEKSILKLAQRMAVNFYAG-ISR--SS--V-RK--------WD-KLL-VA----
XP_010537199.1/1-242 ----PAGAKSVLKLAQRMGFNFFAG-VSG--SS--V-RK--------WE-KLP-VA----
XP_010537197.1/1-242 ----PAGAKSVLKLAQRMGFNFFAG-VSG--SS--V-RK--------WE-KLP-VA----
XP_010537198.1/1-241 ----PAGAKSVLKLAQRMGFNFFAG-VSG--SS--V-RK--------WE-KLP-VA----
Con_95%              ...........hthupRh..sF....l....................a............
Con_85%              ..........hhphupRhs.sFsts.ls.......t...........W...h........
Con_75%              .........shhpLupRhstsFstu.lsu......s...........Ws..l..t.....
Con_65%              .........uhhpLupRMspsFssu.lsu..o...s..t........Ws..l..ss....
Con_55%              .........uhhKLAQRMscuFCsu.Vsu..Ss..s.pp........Wo.pL..ss....


XP_020582785.1/1-284 ---------------DG--V-EDVTIT-------INS------SPSKLFGSH--SRF--A
XP_020682623.1/1-284 ---------------DG--G-EDVTIN-------INS------SPSKLFGSH--SRS--A
XP_020682624.1/1-284 ---------------DG--G-EDVTIN-------INS------SPSKLFGSH--SRS--A
XP_006846015.1/1-286 ---------------DG--V-EDVTIV-------LNT------NPSKLFAAN--VNS-AA
NP_001142394.1/1-289 ---------------DG--A-EDITVT-------INS------SPNKLVGSH--VSA-SP
NP_001146215.1/1-288 ---------------DG--A-EDISIT-------INS------SPNKLIGSD--VSP-SP
XP_020405718.1/1-288 ---------------DG--A-EDISIT-------INS------SPNKLIGSD--VSP-SP
XP_002443548.1/1-287 ---------------DG--A-EDITIS-------INS------SPNKLIGSH--VSP-SP
XP_025808876.1/1-287 ---------------DG--P-EDITIT-------VNS------SPNKLVGSH--VSP-SP
XP_004963232.1/1-287 ---------------DG--P-EDITIT-------VNS------SPNKLVGSH--VSP-SP
XP_020192001.1/1-285 ---------------DR--A-EDVTIA-------INS------SPNKLAGSH--VNP-SQ
XP_003562005.1/1-285 ---------------DG--A-EDVTIA-------INS------SPNKLVGSH--VNS-SQ
XP_004982339.1/1-286 ---------------DG--A-EDVTIA-------INS------SPNKLVGSH--VNS-SQ
XP_025796542.1/1-286 ---------------DG--S-EDVTIA-------INS------SPNKLVGSH--VNS-SQ
NP_001347456.1/1-286 ---------------DG--A-EDVTIA-------INS------SPNKLIGPH--VNS-SQ
NP_001352464.1/1-286 ---------------DG--A-EDVTIA-------INS------SPNKLIGPH--VNS-SQ
XP_002464180.1/1-286 ---------------DG--A-EDVTIA-------INS------SPNKLIGPH--VNS-SQ
XP_015689987.1/1-286 ---------------DG--A-EDVTIA-------FNS------SPNKLVGSH--VNS-SQ
XP_015632484.1/1-286 ---------------DG--A-EDVTIA-------FNS------SPNKLVGSH--VNS-SQ
XP_015620816.1/1-285 ---------------DG--S-EDITIS-------VNS------SPNKLVGSH--VSP-NP
XP_006664738.1/1-285 ---------------DG--S-EDITIS-------VNS------SPNKLVGSH--VSP-NP
XP_020190954.1/1-286 ---------------DG--A-EDITIT-------VNS------SPNKLVGSH--ISP-SP
XP_020163543.1/1-286 ---------------DG--A-EDITIT-------VNS------SPNKLVGSH--ISP-SP
XP_003577453.1/1-286 ---------------DG--S-EDITIT-------VNS------SPNKLAGSH--VSP-SP
XP_020575104.1/1-285 ---------------DG--V-EDVTIA-------INS------YPNKLC-GH--SGS--S
XP_020696902.1/1-286 ---------------DG--V-EDVTIA-------INT------FPNKLLGGH--SSS--S
XP_020578108.1/1-284 ---------------DG--G-DDVTIT-------INS------SPSKLLGSV--NSS---
XP_020698923.1/1-284 ---------------DG--G-DDVTIT-------VNS------SPSKLLGSV--NSS---
XP_020096610.1/1-279 ---------------DG--V-DDVAIA-------INS------SPNKLLGSS--------
XP_009412508.1/1-287 ---------------DG--V-EDVTIA-------INS------SPNKFHRSH--FNS-ST
XP_020271156.1/1-284 ---------------DG--V-EDVTIM-------INS------SPDKLLGS-----S--S
XP_020273236.1/1-287 ---------------DG--V-EDVTIM-------INS------SPNKLFGSH--ANS--S
XP_020092733.1/1-287 ---------------DG--V-EDVTIA-------INA------SPNKLFGSN--ISC-SP
XP_019701934.1/1-288 ---------------DG--V-EDVTIT-------INS------SPNKLLGSH--VNS-ST
XP_010905421.1/1-288 ---------------DG--V-EDVTIT-------INS------SPNKLLGSH--VNS-ST
XP_010905420.1/1-289 ---------------DG--V-EDVTIT-------INS------SPNKLLGSH--VNS-ST
XP_010923678.1/1-288 ---------------DG--V-EDVTIA-------INS------SPNKLLGSH--VNS-ST
XP_008794452.1/1-288 ---------------DG--V-EDVTIA-------INA------SPNKLLGSH--VNS-ST
XP_009397391.1/1-288 ---------------DG--V-DDVTIA-------VNS------SPNKLLGSH--VNT-SA
XP_009408551.1/1-288 ---------------DG--V-EDVTIA-------INS------SSNKLIGSH--VNS-SA
XP_009399045.1/1-288 ---------------DG--V-EDVTVA-------INS------SPNKILSSH--ANT-SA
XP_009388964.1/1-288 ---------------DG--V-EDVTIA-------INS------SPNKLLGSH--ANP-SA
XP_009397717.1/1-288 ---------------DG--V-EDVTIA-------INS------SPNKLFGSH--VNP-SA
XP_018488876.1/1-280 ---------------DG--G-EDITIM-------INS------SSSKFAG-S--QYG-NS
XP_013587486.1/1-280 ---------------DG--G-EDITIM-------INS------SSSKFSG-S--QYG-NS
XP_009115139.1/1-280 ---------------DG--G-EDITIM-------INS------SSSKFAG-S--QYG-NS
XP_013662459.1/1-280 ---------------DG--G-EDITIM-------INS------SSSKFAG-S--QYG-NS
XP_013707293.1/1-280 ---------------DG--G-EDITIM-------INS------SSSKFSG-S--QYG-NS
XP_006415465.2/1-280 ---------------DG--G-EDITIM-------INS------SSAKFAG-S--QYG-NS
XP_019099288.1/1-280 ---------------DG--A-EDITIM-------INS------SSAKFAG-S--QYG-SS
NP_174337.1/1-280    ---------------DG--G-EDITIM-------INS------SSAKFAG-S--QYG-SS
XP_020867051.1/1-280 ---------------DG--A-EDITIM-------INS------SSAKFAG-S--QYG-SS
XP_020867052.1/1-280 ---------------DG--A-EDITIM-------INS------SSAKFAG-S--QYG-SS
XP_010460847.1/1-280 ---------------DG--A-EDITIM-------INS------SSAKFAG-S--QYG-SS
XP_019094482.1/1-280 ---------------DG--A-EDITIM-------INS------SSAKFAG-S--QYG-SS
XP_006306764.1/1-280 ---------------DG--A-EDITIM-------INS------SSAKFAG-S--QYG-SS
XP_010538268.1/1-285 ---------------NG--S-DDVTIM-------INS------SPGKFVG-N--QFG-NS
XP_010519070.1/1-284 ---------------DG--A-EDVTIM-------INS------SPGKYVS-S--QFG-SA
XP_018436356.1/1-280 ---------------DG--A-EDITVM-------INL------SPGKYGG--------SQ
XP_013637594.1/1-286 ---------------DG--A-EDISVM-------INL------SPGKFGGPS--HYG-SS
XP_013692700.1/1-286 ---------------DG--A-EDISVM-------INL------SPGKFGGPS--HYG-SS
XP_009143747.1/1-286 ---------------DG--A-EDISVM-------INL------SPGKFGGPS--HYG-SS
XP_018484893.1/1-285 ---------------DG--G-EDITVM-------INL------SPGKFGGSQ--YSN-NS
XP_013637264.1/1-280 ---------------DG--A-EDITLM-------INL------SPGKFGG--------NS
XP_013637263.1/1-280 ---------------DG--A-EDITLM-------INL------SPGKFGG--------NS
XP_022557131.1/1-280 ---------------DG--A-EDITLM-------INL------SPGKFGG--------NS
XP_013729051.1/1-280 ---------------DG--A-EDITLM-------INL------SPGKFGG--------NS
XP_013744548.1/1-280 ---------------DG--A-EDITLM-------INL------SPGKFGG--------NS
XP_009141396.1/1-280 ---------------DG--A-EDITLM-------INL------SPGKFGG--------NS
XP_006410621.1/1-284 ---------------DG--A-EDITVM-------INL------SPGKFGGSQ--YG--SS
NP_181018.1/1-284    ---------------DG--A-EDVTVM-------INL------SPGKFGGSQ--YG--NS
XP_020884308.1/1-284 ---------------DG--A-EDITVM-------INL------SPGKLCGSQ--YG--NS
XP_006293359.1/1-284 ---------------DG--A-EDITVM-------INL------SPGKFGGAQ--YG--NS
XP_010516626.1/1-284 ---------------DG--A-EDITVM-------INL------SPGKFGGAQ--YG--SS
XP_010509720.1/1-284 ---------------DG--A-EDITVM-------INL------SPGKFGGAQ--YG--NS
XP_010504942.1/1-284 ---------------DG--A-EDITVM-------INL------SPGKFGGAQ--YG--NS
XP_017223336.1/1-283 ---------------DG--L-EDVTVA-------INM------SAGKFLSSQ--YNA-FS
XP_016559371.1/1-285 ---------------DG--L-EDVTIA-------VNS------SPNKFLDAQ--YNT-LS
XP_016559374.1/1-285 ---------------DG--L-EDVTIA-------VNS------SPNKFLDAQ--YNT-LS
XP_012830207.1/1-281 ---------------DG--A-EDVTIA-------INL------SPSKFLASH--SNT-LS
XP_012827547.1/1-287 ---------------DG--V-EDVTIA-------VNS------SPSKFTGSQ--YNN-LS
XP_022866806.1/1-284 ---------------DG--V-DDITIA-------INS------SPSKFLGSQ--SNS-LS
XP_022866805.1/1-285 ---------------DG--V-DDITIA-------INS------SPSKFLGSQ--SNS-LS
XP_023001017.1/1-285 ---------------DG--V-EDVTVV-------VNS------SPNKFIGSQ--YNQ--S
XP_023001016.1/1-285 ---------------DG--V-EDVTVV-------VNS------SPNKFIGSQ--YNQ--S
XP_023519837.1/1-285 ---------------DG--V-EDVTVV-------VNS------SPNKFIGSQ--YNQ--S
XP_023519836.1/1-285 ---------------DG--V-EDVTVV-------VNS------SPNKFIGSQ--YNQ--S
XP_022927139.1/1-285 ---------------DG--V-EDVTVV-------VNS------SPNKFIGSQ--YNQ--S
XP_022927138.1/1-285 ---------------DG--V-EDVTVV-------VNS------SPNKFIGSQ--YNQ--S
XP_022139982.1/1-285 ---------------DG--V-EDVTVV-------INS------SPNKFLGLQ--YNT--S
XP_004135550.1/1-285 ---------------DG--V-EDVTVV-------INS------SPNKFLGSQ--YNT--S
XP_008445207.1/1-285 ---------------DG--V-EDVTVV-------INS------SPNKFLGSQ--YNT--S
XP_017230337.1/1-286 ---------------DG--V-EDVTIA-------INS------NPGKFVGSQ--YNNTLS
XP_017230338.1/1-286 ---------------DG--V-EDVTIA-------INS------NPGKFVGSQ--YNNTLS
XP_010316959.1/1-285 ---------------DG--V-DDVTIA-------VNS------SPSKFLDAQ--YN-TLS
XP_004233170.1/1-285 ---------------DG--V-DDVTIA-------VNS------SPSKFLDAQ--YN-TLS
XP_015066839.1/1-285 ---------------DG--V-DDVTIA-------VNS------SPSKFLDAQ--YN-TLS
XP_015066842.1/1-285 ---------------DG--V-DDVTIA-------VNS------SPSKFLDAQ--YN-TLS
XP_015066843.1/1-285 ---------------DG--V-DDVTIA-------VNS------SPSKFLDAQ--YN-TLS
XP_006353018.1/1-285 ---------------DG--V-DDVTIA-------VNS------SPSKFLDAQ--YN-TLS
XP_006353020.1/1-285 ---------------DG--V-DDVTIA-------VNS------SPSKFLDAQ--YN-TLS
XP_019178964.1/1-287 ---------------DG--V-EDVTIA-------INS------SPSKFLGSQ--YN-NLS
XP_019178963.1/1-287 ---------------DG--V-EDVTIA-------INS------SPSKFLGSQ--YN-NLS
XP_019228489.1/1-285 ---------------DG--V-EDVTIA-------INS------SSTKFLGSQ--YN-TLS
XP_019228488.1/1-285 ---------------DG--V-EDVTIA-------INS------SSTKFLGSQ--YN-TLS
NP_001289498.1/1-285 ---------------DG--V-EDVTVA-------INS------SSTKFLGSQ--YN-TLS
XP_009777911.1/1-285 ---------------DG--V-EDVTVA-------INS------SSTKFLGSQ--YN-TLS
XP_009612342.1/1-285 ---------------DG--V-EDVTIA-------INS------SSTKFLGSQ--YN-TLS
XP_009612341.1/1-285 ---------------DG--V-EDVTIA-------INS------SSTKFLGSQ--YN-TLS
XP_006363649.1/1-287 ---------------DG--V-EDVTIA-------INS------SSSKFLGSQ--YN-TLS
XP_015066689.1/1-287 ---------------DG--V-EDVTIA-------INS------SSSKFLGSQ--YN-TLS
XP_010315636.1/1-287 ---------------DG--V-EDVTIA-------INS------SSSKFLGSQ--YN-TLS
XP_022879368.1/1-285 ---------------DG--V-EDVTIA-------INS------SPSKFLGSQ--HN-SLS
XP_020554026.1/1-285 ---------------DG--V-EDVTIA-------INS------SPSKFLGSQ--YN-NLS
XP_011096678.1/1-285 ---------------DG--V-EDVTIA-------INS------SPSKFLGSQ--YN-NLS
XP_011085207.1/1-285 ---------------DG--G-EDVTIA-------INL------SPSKFLGSQ--FN-TLC
XP_023003193.1/1-285 ---------------DG--L-EDVTIV-------INS------SANKLPGSQ--YKTS--
XP_022926723.1/1-285 ---------------DG--L-EDVTIV-------INS------SANKLPGSQ--YKTS--
XP_023518237.1/1-285 ---------------DG--L-EDVTIV-------INS------SANKLPGSQ--YKTS--
XP_022146537.1/1-286 ---------------DG--L-EDVTIV-------INS------SPNKFPGSQ--YNMS--
XP_011658319.1/1-286 ---------------DG--V-EDVTIL-------INT------SANKFSGSQ--YNTS--
XP_008439554.1/1-286 ---------------DG--V-EDVTIL-------INT------SANKFSGSQ--YNTS--
XP_022978372.1/1-286 ---------------DG--L-EDVTIV-------INS------SANMFPGSQ--YNTS--
XP_023543833.1/1-286 ---------------DG--L-EDVTIV-------INS------SANVFPGSQ--YNTS--
XP_022926042.1/1-286 ---------------DG--L-EDVTIV-------INS------SANVFPGSQ--YNTS--
XP_010247499.1/1-286 ---------------DG--V-EDVTIA-------INS------TPSKLLGCQ--VNSS-T
XP_016717556.1/1-285 ---------------DG--V-EDVTIM-------INS------SPGKFFGSQ--YNTS--
XP_012455108.1/1-285 ---------------DG--V-EDVTIM-------INS------SPGKFFGSQ--YNTS--
XP_016717554.1/1-287 ---------------DG--V-EDVTIM-------INS------SPGKFFGSQ--YNTS--
XP_012455107.1/1-287 ---------------DG--V-EDVTIM-------INS------SPGKFFGSQ--YNTS--
XP_016711412.1/1-287 ---------------DG--V-EDVTIM-------INS------SPGKFFGSQ--YNTS--
XP_017649973.1/1-287 ---------------DG--V-EDVTIM-------INS------SPGKFFGSQ--YNTS--
XP_022716449.1/1-285 ---------------DG--V-EDVTIM-------INS------SPGKFLGSQ--YNT---
XP_022724664.1/1-287 ---------------DG--V-EDVTIM-------INS------SGGKFLGSQ--YNTS--
XP_021298192.1/1-285 ---------------DG--V-EDVTIM-------INS------SPGKFLGSQ--YNTS--
XP_021298193.1/1-294 ---------------DG--V-EDVTIM-------INS------SPGKFLGSQ--YNTS--
XP_021298191.1/1-294 ---------------DG--V-EDVTIM-------INS------SPGKFLGSQ--YNTS--
XP_007022874.2/1-285 ---------------DG--V-EDVTIM-------INS------SPGKFLGSQ--YNTS--
XP_017979205.1/1-294 ---------------DG--V-EDVTIM-------INS------SPGKFLGSQ--YNTS--
XP_017979204.1/1-294 ---------------DG--V-EDVTIM-------INS------SPGKFLGSQ--YNTS--
XP_017236680.1/1-286 ---------------DG--V-EDVTIA-------INS------NPEKFIGSQ--YNNSLS
XP_010051959.1/1-285 ---------------DG--V-EDVTIA-------VNS------SPNKFLGSQ--YNAT--
XP_010051960.1/1-285 ---------------DG--V-EDVTIA-------VNS------SPNKFLGSQ--YNAT--
XP_015882975.1/1-284 ---------------DG--V-EDVTIV-------INS------SPNKFLGSQ--HNTS--
XP_015882797.1/1-284 ---------------DG--V-EDVTIV-------INS------SPNKFLGSQ--HNTS--
XP_010098570.1/1-284 ---------------DG--A-EDVTIV-------INS------SSNKFLGSQ--YNAS--
XP_011012084.1/1-284 ---------------DG--G-DDVTIV-------INS------SPTKFLGSQ--YNAS--
XP_011012083.1/1-284 ---------------DG--G-DDVTIV-------INS------SPTKFLGSQ--YNAS--
XP_006377667.2/1-284 ---------------DG--G-DDVTIV-------INS------SPTKFLGSQ--YNAS--
XP_002281868.2/1-285 ---------------DG--V-EDVTIV-------INS------SPSKFLGPQ--YNST--
XP_018847863.1/1-285 ---------------DG--V-EDVTIM-------VNS------SPSKFLGSQ--YSTS--
XP_018847862.1/1-285 ---------------DG--V-EDVTIM-------VNS------SPSKFLGSQ--YSTS--
XP_023919942.1/1-285 ---------------DG--V-EDVTIM-------INS------SPNKFLGSQ--YSTS--
XP_023919941.1/1-285 ---------------DG--V-EDVTIM-------INS------SPNKFLGSQ--YSTS--
XP_018828101.1/1-285 ---------------DG--V-EDVTIM-------INS------SPNKFLNSQ--YSTS--
XP_018828100.1/1-285 ---------------DG--V-EDVTIM-------INS------SPNKFLNSQ--YSTS--
XP_002298892.1/1-285 ---------------DG--G-DDVTIV-------INS------SPNKFLGSQ--YNAS--
XP_011038887.1/1-285 ---------------DG--G-DDVTIV-------INS------SPNKFLGSQ--YNAS--
XP_011038886.1/1-285 ---------------DG--G-DDVTIV-------INS------SPNKFLGSQ--YNAS--
XP_021657817.1/1-285 ---------------DG--V-EDVTIV-------INS------SPNKFLGSQ--YNAS--
XP_021657818.1/1-285 ---------------DG--V-EDVTIV-------INS------SPNKFLGSQ--YNAS--
XP_015577248.1/1-260 ---------------DG--V-EDVTIV-------INS------TPNKFLGSQ--YTTS--
XP_021616343.1/1-285 ---------------DG--M-EDVTIA-------INS------SPNKFLGTQ--YNAS--
XP_021645103.1/1-285 ---------------DG--V-EDVTIV-------INS------SPNKILGSQ--YNAS--
XP_021592762.1/1-285 ---------------DG--V-EDVTIA-------INS------SPNKFLGSQ--YNAS--
XP_012075627.1/1-285 ---------------DG--V-EDVTIV-------INS------SPNKFLGSQ--YNAS--
XP_008372224.1/1-285 ---------------DG--V-EDVTIA-------INS------SPNKVLGSQ--YNTS--
XP_009362238.1/1-285 ---------------DG--V-EDVTIA-------INS------SPNKVLGSQ--YNTS--
XP_009344405.1/1-284 ---------------DG--V-EDVTIA-------INS------SP-KVLGSQ--YNTS--
XP_008349150.1/1-284 ---------------DG--V-EDVTIA-------INS------SP-KVLGSQ--YNTS--
XP_007214622.1/1-285 ---------------DG--V-EDVTIT-------INS------SPNKFLGSQ--YNAS--
XP_021812393.1/1-285 ---------------DG--V-EDVTIT-------INS------SPNKFLGSQ--YNAS--
XP_024161164.1/1-285 ---------------DG--A-EDVTIA-------INS------SPNKFLGSQ--YNAS--
XP_004293989.1/1-285 ---------------DG--A-EDVTIA-------INS------SPNKFLGSQ--YNTS--
XP_019419795.1/1-282 ---------------DG--V-DDVTIA-------VNS------TPNKFFGSQ--YNSS--
XP_019426301.1/1-282 ---------------DG--V-DDVTIA-------INT------SPNKYVASQ--YSSS--
XP_003524993.1/1-285 ---------------DG--V-EDVTIA-------INS------SPNKFFGSH--YNTS--
XP_003532788.1/1-285 ---------------DG--V-EDVTIG-------INS------SPNKFFSSH--YNTS--
XP_020215009.1/1-285 ---------------DG--V-EDVTIA-------INS------SPNKFFGSH--YNAS--
XP_017409410.1/1-285 ---------------DG--V-EDVTIA-------INS------SPNKSFGSH--YNTS--
XP_017409418.1/1-285 ---------------DG--V-EDVTIA-------INS------SPNKSFGSH--YNTS--
XP_014509941.1/1-285 ---------------DG--V-EDVTIA-------INS------SPNKSFGSH--YNAS--
XP_007159548.1/1-285 ---------------DG--V-EDVTIA-------INS------SPNKFYGSH--YNTS--
XP_024047818.1/1-288 ---------------DG--G-EDVTIA-------INS------SPNKFLGSQ--YNWS--
XP_006448915.2/1-288 ---------------DG--G-EDVTIA-------INS------SPNKFLGSQ--YNWS--
XP_006468302.1/1-288 ---------------DG--G-EDVTVA-------INS------SPNKFLGSQ--YNWS--
XP_006468303.1/1-287 ---------------DG--G-EDVTVA-------INS------SPNKFLGSQ--YNWS--
XP_024047817.1/1-287 ---------------DG--G-EDVTIA-------INS------SPNKFLGSQ--YNWS--
XP_004504217.1/1-285 ---------------DG--V-EDVTIT-------INS------SPNKYFGSH--YNAS--
XP_003594520.2/1-281 ---------------DG--V-EDVTIA-------VNS------SPNKFLGSN--YS-S--
XP_024632601.1/1-281 ---------------DG--V-EDVTIA-------VNS------SPNKFLGSN--YS-S--
XP_004486450.1/1-285 ---------------DG--V-EDVTIT-------VNS------SPNKFLGSN--YNSS--
XP_007147487.1/1-285 ---------------DG--V-EDVTIA-------INS------SPNKFLGSN--YNAS--
XP_017437412.1/1-285 ---------------DG--V-EDVTIA-------INS------SPNKFLGSN--YNAS--
XP_014518680.1/1-285 ---------------DG--V-EDVTIA-------VNS------SPNKFLGSN--YNAS--
XP_003546255.1/1-285 ---------------DG--V-EDVTIA-------INS------SPNKFLGSN--YNAS--
XP_020233213.1/1-285 ---------------DG--V-EDVTIA-------INS------SPNKFLGSN--YNAS--
XP_003535078.1/1-285 ---------------DG--V-EDVTIA-------INS------SPNKFLGSN--YNAS--
XP_014617344.1/1-285 ---------------DG--V-EDVTIA-------INS------SPNKFLGSN--YNAS--
XP_015959291.1/1-283 ---------------DG--V-EDVTIA-------INS------SPNKFLGSQ--YNSS--
XP_016197724.1/1-283 ---------------DG--V-EDVTIA-------INS------SPNKFLGSQ--YNSS--
XP_025694278.1/1-283 ---------------DG--V-EDVTIA-------INS------SPNKFLGSQ--YNSS--
XP_006846777.1/1-281 ---------------DG--V-EDVSVV-------INT------SPNK-------------
XP_003574071.1/1-280 ---------------DG--I-EDVIIA-------CNS------K-KIRSNT----AAPSA
XP_020174778.1/1-278 ---------------DG--I-EDVIIA-------CNS------K-KIRSNN----TAPNA
XP_006661867.1/1-281 ---------------DG--I-EDVIIA-------CNS------K-KVRNTS----TIANA
XP_015614901.1/1-281 ---------------DG--I-EDVIIA-------CNA------K-KVRNTS----TSANA
XP_004982838.1/1-282 ---------------DG--T-EDVIIA-------CNS------K-KTRNSS----NPASA
XP_025796396.1/1-282 ---------------DG--I-EDVIIT-------CNS------K-KIRNSS----NPASA
NP_001316502.1/1-284 ---------------DG--M-EDVIIA-------CNS------K-KIRSSS----NPATG
XP_021306737.1/1-282 ---------------DG--I-EDVIIA-------CNS------K-KIRSGS----NPATA
XP_021306733.1/1-293 ---------------DG--I-EDVIIA-------CNS------K-KIRSGS----NPATA
XP_020162053.1/1-281 ---------------DG--I-EDVVVA-------CNS------TKKIRNVS----NGVNA
XP_006649248.1/1-282 ---------------DG--V-EDVVIA-------CNS------TKKIKTGS----NACIA
XP_015632709.1/1-282 ---------------DG--V-EDVVIA-------CNS------TKKIRSNS----NAGIA
NP_001296150.1/1-282 ---------------DG--I-EDVVIA-------CNS------TKKIRNTS----NAGIT
XP_008665074.1/1-283 ---------------DG--I-EDVVVA-------CNS------TKKIRNNS----NAGIT
NP_001105994.1/1-283 ---------------DG--I-EDVVVA-------CNS------TKKIRNNS----NAGIT
XP_008660404.1/1-283 ---------------DG--I-EDVVVA-------CNS------TKKIRNNS----NAGIT
XP_002468669.1/1-283 ---------------DG--I-EDVVVA-------CNS------TKKIRNNS----NAGIT
XP_021309716.1/1-285 ---------------DG--I-EDVVVA-------CNS------TKKIRNNS----NAGIT
XP_004986037.1/1-283 ---------------DG--I-EDVVVA-------CNS------TKKIRNNS----NVGIT
XP_025795001.1/1-283 ---------------DG--I-EDVVVA-------CNS------SKKIRNNS----SVGIA
XP_020093893.1/1-283 ---------------DG--V-EDIVIA-------TNS------TKKIRTDA----NSANA
XP_020093894.1/1-282 ---------------DG--V-EDIVIA-------TNS------TKKIRTDA----NSANA
XP_020259754.1/1-283 ---------------DG--V-EDVVVT-------TNP------MKKTNSNA----NPANA
XP_009401955.1/1-282 ---------------DG--M-DDIVIA-------TNS------TKKTGAN-----ASSNA
XP_008791541.1/1-283 ---------------DG--V-EDVVVA-------TNT------SKKISAQA----NSANA
XP_017698637.1/1-283 ---------------DG--V-EDVVVA-------TNT------SKKISAQA----NSANA
XP_010920422.1/1-283 ---------------DG--V-EDVVVA-------TNT------SKKISAQA----NSANA
XP_010920423.1/1-283 ---------------DG--V-EDVVVA-------TNT------SKKISAQA----NSANA
XP_010920419.1/1-283 ---------------DG--V-EDVVVA-------TNT------SKKISAQA----NSANA
XP_010920426.1/1-283 ---------------DG--V-EDVVVA-------TNT------SKKISAQA----NSANA
XP_019709326.1/1-287 ---------------DG--M-EDVIVA-------TNT------SKKISTQA----NSANA
XP_008785281.1/1-283 ---------------DV--M-EDVIVA-------TNT------SKKINTQA----NSADG
XP_008462593.1/1-288 ---------------DG--A-DDVILA-------INS------SKNLNTSY----DP-VN
XP_022969629.1/1-286 ---------------DG--A-EDVSLA-------INS------SKNVNTSY----DP-VN
XP_023536970.1/1-286 ---------------DG--A-EDVSLA-------INS------SKNVNTSY----DP-VN
XP_022937855.1/1-288 ---------------DG--A-EDVSLA-------INS------SKNVNTSY----DP-VN
XP_022937858.1/1-288 ---------------DG--A-EDVSLA-------INS------SKNVNTSY----DP-VN
XP_012827869.1/1-285 ---------------NDN-A-EDVIIA-------VTS------AKNLTSSS----NT-I-
XP_022874815.1/1-279 ---------------DG--A-EDVVVA-------VNS------SKNLSTGT----NA-V-
XP_022874814.1/1-283 ---------------DG--A-EDVVVA-------VNS------SKNLSTGT----NA-V-
XP_018434000.1/1-280 ---------------NG--A-EDITVA-------VNS------TKHLNRFS----DS-L-
XP_013642496.1/1-279 ---------------NG--A-EDITVA-------VNS------TKHLNSFS----DP-L-
XP_009150087.1/1-279 ---------------NG--A-EDITVA-------VNS------TKHLNSFS----DP-L-
XP_009150088.1/1-279 ---------------NG--A-EDITVA-------VNS------TKHLNSFS----DP-L-
XP_022563181.1/1-279 ---------------NG--A-EDITVA-------VNS------TKNLNSFS----DP-L-
XP_013675790.1/1-280 ---------------NG--A-EDITVA-------VNS------TKNLNSFS----DP-L-
XP_013623090.1/1-279 ---------------NG--A-EDITVA-------VNS------TKHLNSFS----DP-L-
XP_013623089.1/1-280 ---------------NG--A-EDITVA-------VNS------TKHLNSFS----DP-L-
XP_020885782.1/1-285 ---------------DG--A-EDIIVA-------INS------TKHLNNIS----NS-L-
XP_006280004.1/1-285 ---------------DG--A-EDIIVA-------INS------TKHLNNIS----NS-L-
XP_019088563.1/1-285 ---------------DG--A-EDIIVA-------INS------TKHLNNIS----NS-L-
XP_010483719.1/1-285 ---------------DG--A-EDIIVA-------INS------TKHLNNIS----NS-L-
XP_010456679.1/1-285 ---------------DG--A-EDIIVA-------INS------TKHLNNIS----NS-L-
XP_006400821.1/1-284 ---------------DG--A-EDIIVA-------INS------TKHLNNIS----NS-L-
NP_200877.1/1-284    ---------------DG--A-EDIIVA-------INS------TKHLNNIS----NS-L-
XP_013611543.1/1-282 ---------------DG--A-EDIIVA-------INS------TKHLNNMS----NS-L-
XP_009120605.1/1-284 ---------------DG--A-EDIIVA-------INS------TKHLNNMS----NS-L-
XP_013667152.1/1-284 ---------------DG--A-EDIIVA-------INS------TKHLNNMS----NS-L-
XP_018446535.1/1-284 ---------------DG--A-EDIIVA-------INS------TKHLNNMS----NS-L-
XP_018454374.1/1-284 ---------------DG--A-EDIIVA-------INS------TKHLSNIS----SS-L-
XP_009126588.1/1-285 ---------------DG--A-EDIILA-------INS------TKHLSNIS----SS-L-
XP_013680521.1/1-285 ---------------DG--S-EDIIVA-------INS------TKHLSNIS----SS-L-
XP_013612427.1/1-285 ---------------DG--S-EDIIVA-------INF------TKHLSNIS----SS-L-
XP_010535608.1/1-283 ---------------DG--A-EDIIVT-------VNS------TKHLDNVS----NP-L-
XP_010520372.1/1-276 ---------------DG--V-EDIIVA-------VNS------TNHSNDIS----NS-L-
XP_004487103.1/1-286 ---------------DG--L-EDVTIA-------VNS------IKNLSGTS----NP-AS
XP_003597252.1/1-283 ---------------DG--A-EGVTIS-------VNS------IKNLSGTS----NP-AS
XP_024455683.1/1-286 ---------------DG--A-EDVIIA-------VNS------TKNLIGAN----NS-AH
XP_022026352.1/1-288 ---------------DG--A-EDVIIA-------VNS------TKNLNNSM----NP-SN
XP_022000486.1/1-288 ---------------DG--A-EDVIVA-------VNS------TKNLNNSM----NP-SA
XP_016717477.1/1-281 ---------------DG--T-EDVIIA-------INS------GKSLSNSS----NL-TT
XP_017643014.1/1-281 ---------------DG--T-EDVIIA-------INS------GKSLSNSS----NL-TT
XP_016724213.1/1-281 ---------------DG--T-EDVIIA-------INS------SKSLSNSS----NL-ST
XP_012462938.1/1-281 ---------------DG--T-EDVIIA-------INS------SKSLSNSS----NL-ST
XP_012471509.1/1-292 ---------------DG--N-EDVIIA-------INS------SKSFSCTS----NP-NN
XP_017642742.1/1-292 ---------------DG--N-EDVIIA-------INS------SKSFSCTS----NP-NN
XP_016755876.1/1-292 ---------------DG--N-EDVIIA-------INS------SKSFSCTS----NP-NN
XP_012471510.1/1-286 ---------------DG--N-EDVIIA-------INS------SKSFSCTS----NP-NN
XP_016753711.1/1-286 ---------------DG--N-EDVIIA-------INS------SKSFSCTS----NP-NN
XP_017642744.1/1-286 ---------------DG--N-EDVIIA-------INS------SKSFSCTS----NP-NN
XP_016755877.1/1-286 ---------------DG--N-EDVIIA-------INS------SKSFSCTS----NP-NN
XP_016750468.1/1-286 ---------------DG--T-EDVIIA-------INS------SKSFSSTS----NP-AN
XP_016688657.1/1-286 ---------------DG--T-EDVIIA-------VNS------IKSFSSTS----NP-AN
XP_012443272.1/1-286 ---------------DG--T-EDVIIA-------INS------IKSFSSTS----NP-AN
XP_017603001.1/1-286 ---------------DG--T-EDVIIA-------INS------SKSFSSTS----NP-AN
XP_022755632.1/1-285 ---------------DG--T-EDVIIA-------INS------SKSLSSTS----NP-AN
XP_022755633.1/1-285 ---------------DG--T-EDVIIA-------INS------SKSLSSTS----NP-AN
XP_022729815.1/1-286 ---------------DG--T-EDVIIA-------VNS------SKRLSSTS----NP-AN
XP_022729816.1/1-286 ---------------DG--T-EDVIIA-------VNS------SKRLSSTS----NP-AN
XP_022752857.1/1-286 ---------------DG--T-EDVIIA-------INL------SKSLSSTS----NP-AS
XP_022752856.1/1-286 ---------------DG--T-EDVIIA-------INL------SKSLSSTS----NP-AS
XP_021277981.1/1-286 ---------------DG--A-EDVIIA-------INS------SKNLSSTS----NP-AN
XP_017983554.1/1-286 ---------------DG--A-EDVIIA-------INS------SKNLSSSS----NP-AN
XP_011089912.1/1-283 ---------------DG--A-EDVIVA-------INS------AKNLTTGS--------S
XP_007208090.1/1-286 ---------------DG--A-EDVIIA-------VNS------TKNLTTSN----PA-NS
XP_008225704.1/1-286 ---------------DG--A-EDVIIA-------VNS------TKNLTTSN----PA-NS
XP_021830853.1/1-286 ---------------DG--A-EDVIIA-------VNS------TKNLTTSN----PA-NS
XP_008349754.1/1-286 ---------------DG--A-DDVIIA-------VNS------TKNLTSTS----NH-AN
NP_001315783.1/1-288 ---------------DGDGA-DDVIIA-------VNS------TKNLTSTS----NH-AN
XP_009367069.1/1-286 ---------------DG--A-EDVIIT-------VNS------TKNLTSTS----NH-AN
XP_008344872.1/1-286 ---------------DG--A-EDVIIT-------VNS------TKNLTSTS----NH-AN
XP_015952399.1/1-286 ---------------DD--A-EDVIIA-------VNS------TKNLSGTS----NP-AS
XP_020982118.1/1-286 ---------------DG--A-EDVIIA-------VNS------TKNLSGTS----NP-AS
XP_025685402.1/1-286 ---------------DG--A-EDVIIA-------VNS------TKNLSGTS----NP-AS
XP_016187430.1/1-286 ---------------DG--A-EDVIIA-------VNS------TKNLSGTS----NP-AS
XP_020214107.1/1-284 ---------------DG--A-EDVIIA-------VNS------TKNLSGTS----NP-AS
XP_006592258.1/1-284 ---------------DG--A-EDVFIA-------VNS------TKNLSGTS----NP-AS
XP_007132262.1/1-285 ---------------DG--A-EDVIIA-------INS------TKNLSGTS----NP-AS
XP_003538150.1/1-285 ---------------DG--A-EDVIIA-------VNS------TKNLSGTS----NP-AS
XP_017433493.1/1-285 ---------------DG--A-EDVIIA-------VNS------TKNLSGTS----NP-AS
XP_014494018.1/1-285 ---------------DG--A-EDVIIA-------VNS------TKNLSGTS----NP-AS
XP_010089983.2/1-286 ---------------DG--A-EDVTIA-------VNS------AKNLSTIS----NP-PN
XP_006378974.2/1-285 ---------------DG--A-EDVIIA-------VNT------TKNLISAN----NP-AH
XP_011010051.1/1-285 ---------------DG--A-EDVIIA-------VNT------TKNLISAN----NP-AH
XP_011010050.1/1-285 ---------------DG--A-EDVIIA-------VNT------TKNLISAN----NP-AH
XP_021905657.1/1-285 ---------------DG--A-EDVILA-------VNS------TKNLSTSA----NH-AN
XP_021610391.1/1-286 ---------------DG--A-EDVIIA-------VNS------TKNLSSTS----NT-SN
XP_002529946.1/1-286 ---------------DG--A-EDVIVT-------INS------TKNLSSTS----NA-AN
XP_021658728.1/1-286 ---------------DG--A-EDIIIA-------VNS------TKNLGSTS----NP-SN
XP_012064816.1/1-286 ---------------DG--A-EDVIIA-------VNS------TKNLGIST----NP-AN
XP_021628343.1/1-286 ---------------DG--A-EDVIIA-------VNS------TKNLSSTS----NP-AN
XP_021657859.1/1-286 ---------------DG--A-EDVIIA-------VNS------TKNLSSTS----NP-AN
XP_015874442.1/1-285 ---------------DG--S-EDVTIT-------VNS------AKNLSAAS----NS-AN
XP_023926843.1/1-286 ---------------DG--A-EDVIIA-------VNS------TKNLSATS----NP-AN
XP_018819529.1/1-286 ---------------DG--A-EDVIIA-------VNS------TKNLSTTS----NP-AN
XP_010651164.1/1-286 ---------------DG--A-EDVIIA-------VNS------TKNLNTTS----NP-AN
XP_024034672.1/1-286 ---------------DG--A-EDVIIA-------VNS------TKSLSTTS----NP-TN
XP_006488084.1/1-286 ---------------DG--A-EDVIIA-------VNS------TKSLSTTS----NP-TN
XP_024034673.1/1-286 ---------------DG--A-EDVIIA-------VNS------TKSLSTTS----NP-TN
XP_010264677.1/1-287 ---------------DG--A-EDVIVA-------INT------TKNMSTIA----NA-SN
XP_010253680.1/1-288 ---------------DG--A-EDVIIS-------INS------AKNVGTIA----NPAAN
XP_022005829.1/1-282 ---------------DG--A-EDVIVS-------INS------TKNIGNST----NS-SN
XP_023751537.1/1-278 ---------------DG--A-EDVIVA-------INS------TKN------------SN
XP_024971452.1/1-283 ---------------DG--A-EDVIVA-------INS------TKNLSTST----NP-SN
XP_024971444.1/1-287 ---------------DG--A-EDVIVA-------INS------TKNLSTST----NP-SN
XP_024985647.1/1-287 ---------------DG--A-EDVIIA-------VNS------TKNLNNSM----NP-SN
XP_023749448.1/1-288 ---------------DG--V-EDVIIA-------VNS------SKNLNNSM----NP-SN
XP_010689538.1/1-281 ---------------DG--A-EDVIIA-------CNT------AKTFMATL----NA-AS
XP_021858740.1/1-280 ---------------DG--A-EDVIIS-------CNS------AKNFMATL----NA-AS
XP_021773317.1/1-281 ---------------DG--A-EDVIIA-------CNS------AKTFMATL----NA-AS
XP_021745941.1/1-281 ---------------DG--A-EDVIIA-------CNS------AKTFMATL----NA-AS
XP_016729039.1/1-286 ---------------DG--A-EDVIIA-------INS------NKSLNSTP----NP-VN
XP_017623828.1/1-286 ---------------DG--A-EDVIIA-------INS------NKSLNSTP----NP-VN
XP_012476406.1/1-286 ---------------DG--A-EDVIIA-------INS------NKSLNSTP----NA-VN
XP_016754776.1/1-286 ---------------DG--A-EDVIIA-------INS------NKSLNSPP----NA-VN
XP_022152944.1/1-286 ---------------DG--A-EDVTLA-------INS------SKNLNTSY----DP-VN
XP_011040884.1/1-286 ---------------DG--A-EDVIIA-------VNS------TKNLIGAN----NS-AH
XP_010043753.1/1-286 ---------------DG--A-EDVMIA-------VTF------SKKLNTTS----NP-AN
XP_024158223.1/1-285 ---------------DG--A-EDIIMA-------VNS------TKNLTT-T----NP-AN
XP_004291573.1/1-285 ---------------DG--A-EDIIMA-------VNS------TKNLTT-T----NP-AN
XP_018808765.1/1-285 ---------------DG--A-EDVIIS-------VNS------TKNLSTTS----NP-DN
XP_023512455.1/1-283 ---------------EG--A-EDVVLA-------VNS------TKNFGTTS----NP-AN
XP_023512456.1/1-283 ---------------EG--A-EDVVLA-------VNS------TKNFGTTS----NP-AN
XP_022944186.1/1-283 ---------------EG--A-EDVVLA-------VNS------TKNFGTTS----NP-AN
XP_022944185.1/1-283 ---------------EG--A-EDVVLA-------VNS------TKNFGTTS----NP-AN
XP_022985614.1/1-283 ---------------EG--A-EDVVLA-------VNS------TKNFGTTS----NP-AN
XP_022985615.1/1-283 ---------------EG--A-EDVVLA-------VNS------TKNFGTTS----NP-AN
XP_011656996.1/1-283 ---------------EG--A-EDVVLT-------VNS------TKNFGTTS----NP-AN
XP_011656997.1/1-283 ---------------EG--A-EDVVLT-------VNS------TKNFGTTS----NP-AN
XP_008463945.1/1-283 ---------------EG--A-EDVVLT-------VNS------TKNFGTTS----NP-AN
XP_008463946.1/1-283 ---------------EG--A-EDVVLT-------VNS------TKNFGTTS----NP-AN
XP_022140363.1/1-286 ---------------DG--A-EDVVLA-------VNS------TKNLGTTS----NP-AN
XP_022995706.1/1-286 ---------------EG--A-EDVVLA-------VNS------TKNFGTTS----NP-AN
XP_023534373.1/1-286 ---------------EG--A-EDVVLA-------VNS------TKNFGTTS----NP-VN
XP_022958713.1/1-286 ---------------EG--A-EDVVLA-------VNS------TKNFGTTS----NP-AN
XP_025614062.1/1-285 ---------------DG--A-EDVIIA-------VSS------TKNFSGTS----NP-AS
XP_025614063.1/1-285 ---------------DG--A-EDVIIA-------VSS------TKNFSGTS----NP-AS
XP_016170485.1/1-285 ---------------DG--A-EDVIIA-------VSS------TKNFSGTS----NP-AS
XP_016170486.1/1-285 ---------------DG--A-EDVIIA-------VSS------TKNFSGTS----NP-AS
XP_015935757.1/1-285 ---------------DG--A-EDVIIA-------VSS------TKNFSGTS----NP-AS
XP_015935758.1/1-285 ---------------DG--A-EDVIIA-------VSS------TKNFSGTS----NP-AS
XP_004505999.1/1-286 ---------------DG--A-EDVIIA-------VNS------TKNLSGTS----NP-AS
XP_013456006.1/1-286 ---------------DG--A-EDVIIA-------VNS------TKNLSSSS----NP-AS
XP_019453966.1/1-285 ---------------DG--A-EDVIIA-------VNS------AKNLNGTS----NP-SS
XP_019448740.1/1-279 ---------------DG--A-EDVIIA-------VNS------AKNLNGIS----NS-AS
XP_019448741.1/1-279 ---------------DG--A-EDVIIA-------VNS------AKNLNGIS----NS-AS
XP_019458095.1/1-286 ---------------DG--V-EDVIIA-------VNS------TMNSNGTS----NP-AS
XP_019447870.1/1-286 ---------------DG--A-EDIIIS-------VNS------TKNLSGTS----NL-AT
XP_012838629.1/1-280 ---------------EG--A-EDVTIA-------INS------AKNPTTSS---------
XP_012838630.1/1-280 ---------------EG--A-EDVTIA-------INS------AKNPTTSS---------
XP_012838627.1/1-280 ---------------EG--A-EDVTIA-------INS------AKNPTTSS---------
XP_012838628.1/1-280 ---------------EG--A-EDVTIA-------INS------AKNPTTSS---------
XP_017214964.1/1-286 ---------------EG--S-EDVIVA-------ENS------LKNLFSTS----NQ-SH
XP_017234817.1/1-286 ---------------EG--S-EDVIVA-------VNS------AKNLVSTS----DL-SS
XP_019178153.1/1-282 ---------------DG--A-EDVTVA-------INS------TKNLGTTS--------N
XP_019166562.1/1-283 ---------------DG--A-EDVTVA-------INS------TKNLAT-S--------N
XP_011089294.1/1-282 ---------------DG--A-EDVVVA-------INS------AKSVTASS--------D
XP_022892122.1/1-274 ---------------DG--A-EDVVVA-------INS------SKNLSTGS--------N
XP_022883393.1/1-279 ---------------DG--A-EDVVVA-------INS------SKNLSAGS--------N
XP_022883398.1/1-279 ---------------DG--A-EDVVVA-------INS------SKNLSAGS--------N
XP_022883384.1/1-283 ---------------DG--A-EDVVVA-------INS------SKNLSAGS--------N
XP_022883389.1/1-283 ---------------DG--A-EDVVVA-------INS------SKNLSAGS--------N
XP_009776589.1/1-283 ---------------DG--G-EDVIVA-------VNS------RKNIATTS--------V
NP_001312504.1/1-283 ---------------DG--G-EDVIVA-------VNS------RKNIATTS--------V
XP_016472209.1/1-283 ---------------DG--G-EDVIVA-------VNS------RKNIATTS--------V
XP_019255429.1/1-283 ---------------DG--G-EDVIVA-------VNS------RKNIATTS--------I
XP_009624234.1/1-283 ---------------DG--G-EDVIVA-------VNS------RKNIATTS--------V
XP_016504967.1/1-283 ---------------DG--G-EDVIVA-------VNS------RKNIATTS--------V
XP_016547131.1/1-283 ---------------DG--A-EDVIVA-------VNS------RKNLATTS--------V
XP_004251138.1/1-283 ---------------DG--A-EDVIVA-------VNS------RKNLATTS--------I
XP_015058512.1/1-283 ---------------DG--A-EDVIVA-------VNS------RKNLATTS--------I
XP_006340193.1/1-283 ---------------DG--A-EDVIVA-------VNS------RKNLATTS--------V
XP_004235650.1/1-283 ---------------DG--M-DDVTIL-------VNS------SPDKLMG-L-NLPFA-N
XP_006343047.1/1-283 ---------------DG--M-DDVTIL-------VNS------SPDKLMG-L-NLPFA-N
XP_009774805.1/1-285 ---------------DG--M-DDITIL-------VNS------SPDKLMG-L-NLSFA-N
XP_016498616.1/1-285 ---------------DG--M-DDITIL-------VNS------SPDKLMG-L-NLSFA-N
XP_016434103.1/1-285 ---------------DG--M-DDITIL-------VNS------SPDKLMG-L-NLSFA-N
XP_009593967.1/1-285 ---------------DG--M-DDITIL-------VNS------SPDKLMG-L-NLSFA-N
XP_004510297.1/1-285 ---------------DG--V-DDVTIL-------VNS------SPEKLMG-L-NLSFG-N
XP_004510296.1/1-285 ---------------DG--V-DDVTIL-------VNS------SPEKLMG-L-NLSFG-N
XP_003627005.1/1-284 ---------------DG--V-DDVTIL-------VNS------SPDKLMG-L-NLSFG-N
XP_003531653.1/1-284 ---------------DG--V-DDVTIL-------VNS------SPDKLMG-L-NLSFA-N
XP_006585576.1/1-288 ---------------DG--V-DDVTIL-------VNS------SPDKLMG-L-NLSFA-N
XP_014634618.1/1-288 ---------------DG--V-DDVTIL-------VNS------SPDKLMG-L-NLSFA-N
XP_014634619.1/1-288 ---------------DG--V-DDVTIL-------VNS------SPDKLMG-L-NLSFA-N
XP_003531652.1/1-285 ---------------DG--V-DDVTIL-------VNS------SPDKLMG-L-NLSFA-N
XP_003530109.1/1-284 ---------------DG--V-DDVTIL-------VNS------SPDKLMG-L-NLSFA-N
XP_007135643.1/1-285 ---------------DG--V-DDVTIL-------VNS------SPDKLMG-L-NLPFT-N
XP_003530112.1/1-285 ---------------DG--V-DDVTIL-------VNS------SPDKLMG-L-NLSFA-N
XP_020226142.1/1-285 ---------------DG--V-DDVTIL-------VNS------SPDKLMG-L-NLSFA-N
XP_014516466.1/1-285 ---------------DG--V-DDVTIL-------VNS------SPDKLMG-L-NLPFA-N
XP_017407594.1/1-285 ---------------DG--V-DDVTIL-------VNS------SPDKLMG-L-NLPFA-N
XP_019448178.1/1-285 ---------------DG--V-DDVTVL-------VNS------SPDKLMG-L-NLSFA-G
XP_019429328.1/1-285 ---------------DG--A-DDVTIL-------VNS------SPEKLMG-L-NLSFA-N
XP_004486776.1/1-285 ---------------DG--A-DDVTIL-------VNS------SPDKLMG-L-NLSFA-N
XP_003597690.1/1-286 ---------------DG--V-DDVTIL-------VNS------SPDKLMG-L-NLSFA-N
XP_015932679.1/1-285 ---------------DG--V-DDVTIL-------VNS------SPDKLMG-L-NLSFA-N
XP_025674177.1/1-285 ---------------DG--V-DDVTIL-------VNS------SPDKLMG-L-NLSFA-N
XP_025610204.1/1-285 ---------------DG--V-DDVTIL-------VNS------SPDKLMG-L-NLSFA-N
XP_020226055.1/1-285 ---------------DG--V-DDVTVL-------VNS------APDKLMG-L-NLSFA-N
XP_007150614.1/1-285 ---------------DG--V-DDVTVL-------VNS------SPDKLMG-L-NLSFA-N
XP_014501306.1/1-285 ---------------DG--V-DDVTVL-------VNS------SPDKLMG-L-NLSFA-N
XP_017425270.1/1-285 ---------------DG--V-DDVTVL-------VNS------SPDKLMG-L-NLSFA-N
XP_021836126.1/1-285 ---------------DG--I-DDVTVL-------VNS------SPDKLMA-L-NLTFA-N
XP_010691423.1/1-284 ---------------DG--I-DDVTVL-------VNS------SPDKLMA-L-NLTYA-N
XP_010691424.1/1-284 ---------------DG--I-DDVTVL-------VNS------SPDKLMA-L-NLTYA-N
XP_021765330.1/1-284 ---------------DG--I-DDVTVL-------VNS------SPDKLMA-L-NLTFA-N
XP_021749885.1/1-284 ---------------DG--I-DDVTVL-------VNS------SPDKLMA-L-NLTFA-N
XP_010267183.1/1-285 ---------------DG--M-DDVTIL-------VNS------SPSKVMG-V-NLTFG-S
XP_017258627.1/1-285 ---------------EG--M-DDVTIL-------VNS------SPDKLMG-L-NLSFN-N
XP_023000315.1/1-284 ---------------DG--M-DDVTIL-------VNS------SPDKLMG-L-NISFA-N
XP_023000313.1/1-284 ---------------DG--M-DDVTIL-------VNS------SPDKLMG-L-NISFA-N
XP_023514554.1/1-284 ---------------DG--M-DDVTIL-------VNS------SPDKLMG-L-NISFA-N
XP_023514552.1/1-284 ---------------DG--M-DDVTIL-------VNS------SPDKLMG-L-NISFA-N
XP_022964458.1/1-284 ---------------DG--M-DDVTIL-------INS------SPDKLMG-L-NISFA-N
XP_022964454.1/1-284 ---------------DG--M-DDVTIL-------INS------SPDKLMG-L-NISFA-N
XP_022147627.1/1-284 ---------------DG--M-DDVTIL-------VNS------SPDKLMG-L-NISFA-N
XP_022147619.1/1-284 ---------------DG--M-DDVTIL-------VNS------SPDKLMG-L-NISFA-N
XP_008462437.1/1-284 ---------------DG--M-DDVTIL-------VNS------SPDKLMG-L-NISFA-N
XP_004141659.1/1-284 ---------------DG--M-DDVTIL-------VNS------SPDKLMG-L-NISFA-N
XP_008462436.1/1-284 ---------------DG--M-DDVTIL-------VNS------SPDKLMG-L-NISFA-N
XP_011659682.1/1-284 ---------------DG--M-DDVTIL-------VNS------SPDKLMG-L-NISFA-N
XP_022992111.1/1-284 ---------------DG--M-DDVTIL-------VNS------SPDKLMG-L-NISFA-N
XP_023548534.1/1-284 ---------------DG--M-DDVTIL-------VNS------SPDKLMG-L-NISFA-N
XP_022953371.1/1-284 ---------------DG--M-DDVTIL-------VNS------SPDKLMG-L-NISFA-N
XP_002304217.1/1-285 ---------------DG--M-DDVTIL-------VNS------SPDKLMG-S-NLSFT-N
XP_011013041.1/1-285 ---------------DG--M-DDVTIL-------VNS------SPDKLMG-S-NLSFT-N
XP_011013042.1/1-285 ---------------DG--M-DDVTIL-------VNS------SPDKLMG-S-NLSFT-N
XP_011046759.1/1-284 ---------------DG--M-DDVTIL-------VNS------SPDKLMG-L-NLSFS-S
XP_024464085.1/1-284 ---------------DG--M-DDVTIL-------VNS------SPDKLMG-L-NLSFS-N
XP_024183498.1/1-286 ---------------DG--M-DDVTIL-------INS------SPDKLMG-L-NLSFG-N
XP_004302866.1/1-286 ---------------DG--M-DDVTIL-------INS------SPDKLMG-L-NLSFG-N
NP_001280898.1/1-285 ---------------DG--M-DDVTIL-------INS------SPDKLMG-L-NLSFG-N
XP_007214960.1/1-285 ---------------DG--M-DDVTIL-------INS------SPDKLMG-L-NLSFA-N
XP_008228437.1/1-285 ---------------DG--M-DDVTIL-------INS------SPDKLMG-L-NLSFA-N
XP_021818763.1/1-285 ---------------DG--M-DDVTIL-------INS------SPDKLMG-L-NLSFA-S
XP_022723515.1/1-285 ---------------DG--M-DDITIL-------VNS------SPDKLMG-L-NLSFA-N
XP_016688508.1/1-285 ---------------DG--M-DDVTIL-------VNS------SPDKLMG-L-NLSFA-N
XP_012443274.1/1-285 ---------------DG--M-DDVTIL-------VNS------SPDKLMG-L-NLSFA-N
XP_022721457.1/1-285 ---------------DG--V-DDVTIL-------VNS------SPDKLMG-L-NLSFA-N
XP_022715018.1/1-285 ---------------DG--M-DDVTIL-------VNS------SPDKLMG-L-NLSFG-N
XP_017979082.1/1-285 ---------------DG--M-DDVTIL-------VNS------SPDKLMG-L-NLSFA-N
XP_021293768.1/1-285 ---------------DG--M-DDVTIL-------VNS------SPEKLMG-L-NLSFA-N
XP_010062518.1/1-285 ---------------DG--I-DDVTIL-------VNS------SPDKLMG-L-NLSFS-N
XP_006426893.1/1-285 ---------------DG--M-DDVTVL-------VNS------SPDKLMG-L-NLSFA-N
XP_021610318.1/1-285 ---------------DG--M-DDVTIL-------VNS------SPEKLMG-L-NLSFS-N
XP_010098130.1/1-285 ---------------DG--M-DDVTIL-------VNS------SPDKLMG-L-NLSFA-N
XP_021635282.1/1-296 ---------------DG--M-DDVTIL-------VNS------SPEKLMG-L-NLSFT-N
XP_002284003.2/1-285 ---------------DG--I-DDVTIL-------VNS------SPEKLTG-L-NLSFA-N
XP_002515977.1/1-285 ---------------DG--M-DDVTIL-------VNS------SPEKLMG-L-NLSFS-N
XP_021643260.1/1-285 ---------------DG--M-DDVTVL-------VNS------SPEKLMG-L-NLSFT-N
XP_021628194.1/1-285 ---------------DG--M-DDVTIL-------VNS------SPEKLMG-L-NLPFT-N
XP_015894591.1/1-285 ---------------DG--M-DDVTIL-------VNS------SPDKLMG-L-NLSFA-N
XP_012069098.1/1-285 ---------------DG--M-DDVTIL-------VNS------SPEKLMG-L-NLSFT-N
XP_018847857.1/1-285 ---------------DG--M-DDVTIL-------VNS------SPDKLMG-L-NLPFS-N
XP_023898511.1/1-285 ---------------DG--M-DDVTIL-------VNS------SPDKLMG-L-NLPFA-T
XP_010545673.1/1-285 ---------------DG--M-DDVTIL-------VNS------SPDKLMD-L-NLTFA-N
XP_010545670.1/1-286 ---------------DG--M-DDVTIL-------VNS------SPDKLMD-L-NLTFA-N
XP_010535179.1/1-285 ---------------DG--M-DDVTIL-------VNS------SPDKLMG-L-NLAFG-N
XP_018436929.1/1-283 ---------------DS--M-DDVTIT-------VNS------SPDKLMG-L-NLTFS-N
XP_013592012.1/1-283 ---------------DS--M-DDVTIT-------VNS------SPDKLMG-L-NLAFS-N
XP_013640375.1/1-284 ---------------DS--M-DDVTIT-------VNS------SPDKLMG-L-NLTFS-N
XP_013640376.1/1-283 ---------------DS--M-DDVTIT-------VNS------SPDKLMG-L-NLTFS-N
XP_009147709.1/1-283 ---------------DS--M-DDVTIT-------VNS------SPDKLMG-L-NLTFS-N
XP_006306297.1/1-281 ---------------DS--M-DDVTIT-------VNS------SPDKLMG-L-NLTFS-N
XP_010462051.1/1-281 ---------------DS--M-DDVTIT-------VNS------SPDKLMG-L-NLTFS-N
XP_010479714.1/1-281 ---------------DS--M-DDVTIT-------VNS------SPDKLMG-L-NLTFS-N
XP_010462052.1/1-281 ---------------DS--M-DDVTIT-------VNS------SPDKLMG-L-NLTFS-N
XP_010500807.1/1-281 ---------------DS--M-DDVTIT-------VNS------SPDKLMG-L-NLAFS-N
NP_849795.1/1-282    ---------------DS--M-DDVTIT-------VNS------SPDKLMG-L-NLTFA-N
NP_001077702.1/1-281 ---------------DS--M-DDVTIT-------VNS------SPDKLMG-L-NLTFA-N
NP_175627.1/1-281    ---------------DS--M-DDVTIT-------VNS------SPDKLMG-L-NLTFA-N
XP_020868586.1/1-281 ---------------DS--M-DDVTIT-------VNS------SPDKLMG-I-NLTFS-N
XP_006392953.1/1-282 ---------------DN--M-DDVTIT-------VNS------SPDKLMG-S-NLTFS-N
XP_018460807.1/1-282 ---------------DS--M-DDVIIT-------VNS------SPDKLMG-L-NLTFS-N
XP_013621760.1/1-283 ---------------DS--M-DDVTIT-------VNS------SADKLMG-L-NLTFS-N
XP_018451524.1/1-282 ---------------DS--M-DDVTIT-------VNS------SPDKLMG-L-NLTFS-N
XP_009107148.1/1-282 ---------------DS--M-DDVTIT-------VNS------SPDKLMG-L-NLTFS-N
XP_009107147.1/1-283 ---------------DS--M-DDVTIT-------VNS------SPDKLMG-L-NLTFS-N
XP_009600774.1/1-284 ---------------DG--M-DDVTIL-------VNS------SPDKLMG-L-NLSFA-D
XP_016459079.1/1-284 ---------------DG--M-DDVTIL-------VNS------SPDKLMG-L-NLSFA-D
XP_019236029.1/1-285 ---------------DG--M-DDVTIL-------VNS------SPDKFMG-L-NLSFA-D
XP_009771098.1/1-285 ---------------DG--M-DDVTIL-------VNS------SPDKLMG-L-NLSFA-D
XP_016550962.1/1-285 ---------------DG--I-DDVAIL-------VNS------SPDRRMG-L-NLSFS-D
XP_016550960.1/1-285 ---------------DG--I-DDVAIL-------VNS------SPDRRMG-L-NLSFS-D
XP_016550961.1/1-285 ---------------DG--I-DDVAIL-------VNS------SPDRRMG-L-NLSFS-D
XP_006359469.1/1-285 ---------------DG--M-DDVTIL-------VNS------SPDKLMG-L-NLSFS-D
XP_004252354.1/1-285 ---------------DG--M-DDVTIL-------VNS------SPDKLMG-L-NLSFS-D
XP_015061298.1/1-285 ---------------DG--M-DDVTIL-------VNS------SPDKLMG-L-NLSFS-D
XP_019198775.1/1-285 ---------------DG--I-DDVTVL-------VNS------SPYKLMG-L-DLTFG-N
XP_019194412.1/1-285 ---------------DG--M-DDVTIL-------VNS------SPDKLMG-L-NLSYT-N
XP_016562656.1/1-283 ---------------DG--M-DDVAIL-------VNS------SPDKLMG-L-NLSFA-N
XP_019267274.1/1-285 ---------------DG--M-DDITLL-------VNS------SPDKLMG-L-NLSFT-N
XP_019267275.1/1-282 ---------------DG--M-DDITLL-------VNS------SPDKLMG-L-NLSFT-N
XP_022889649.1/1-285 ---------------DG--I-DDVTVL-------VNS------NPNKLMG-L-NVSFV-N
XP_022889648.1/1-285 ---------------DG--I-DDVTVL-------VNS------NPNKLMG-L-NVSFV-N
XP_012852689.1/1-281 ---------------DG--M-DDVTIL-------VNS------NPEKLMG-L-NISFT-N
XP_012856468.1/1-279 ---------------DG--M-DDITIL-------VNS------NPDKLIT-L-NTPFT-N
XP_011080516.1/1-285 ---------------DG--I-DDVTVL-------VNS------NPEKLMG-L-NISFA-N
XP_011072422.1/1-285 ---------------DG--M-DDITIL-------VNS------NPDKLMG-L-NISFA-S
XP_022851148.1/1-285 ---------------DG--M-DDVTIV-------VNS------NPEKLMG-L-NLSFT-N
XP_022844668.1/1-285 ---------------DG--M-DDVTIV-------VNS------NPEKLMG-L-NLSFT-N
XP_006853643.2/1-288 ---------------DG--M-DDVTIL-------VNS------SPAKIMA-A-NLTST-N
XP_010249545.1/1-285 ---------------DG--L-DDVTIL-------LNS------SPGKIMG-A-NLAFA-N
XP_010249539.1/1-285 ---------------DG--L-DDVTIL-------LNS------SPGKIMG-A-NLAFA-N
XP_010915409.1/1-284 ---------------DG--T-DDVTIL-------VNS------SPSRLMG-L-NLGFT-N
XP_008791633.1/1-284 ---------------DG--T-DDVTIL-------VNS------SPSRMMG-L-NLGFT-N
XP_017235966.1/1-285 ---------------DG--M-DDVTIL-------VNS------SPDKLLG-L-NMSFS-N
XP_023761873.1/1-288 ---------------DG--S-DDVTIL-------VNS------SPEKLMG-L-NLSFS-N
XP_021997125.1/1-286 ---------------DG--M-DDVTIH-------VNS------SPEKLMG-L-NLSFS-N
XP_021990052.1/1-285 ---------------DG--V-DDVTIL-------VNS------SPEKLMG-L-NLSFS-N
XP_024979339.1/1-285 ---------------DG--M-DDVTIL-------VNS------SPEKLMG-L-NLSFS-N
XP_024979341.1/1-290 ---------------DG--M-DDVTIL-------VNS------SPEKLMG-L-NLSFS-N
XP_024979340.1/1-291 ---------------DG--M-DDVTIL-------VNS------SPEKLMG-L-NLSFS-N
XP_024979342.1/1-291 ---------------DG--M-DDVTIL-------VNS------SPEKLMG-L-NLSFS-N
XP_022016208.1/1-285 ---------------DG--I-DDVTIL-------VNS------SPEKLTG-L-NPSFS-N
XP_022016207.1/1-311 ---------------DG--I-DDVTIL-------VNS------SPEKLTG-L-NPSFS-N
XP_022009819.1/1-285 ---------------DG--V-DDVTVL-------VNS------SPEKLMG-L-ILPTP-N
XP_022009815.1/1-285 ---------------DG--V-DDVTVL-------VNS------SPEKLMG-L-ILPTP-N
XP_022009809.1/1-285 ---------------DG--V-DDVTVL-------VNS------SPEKLMG-L-ILPTP-N
XP_023759500.1/1-286 ---------------DG--N-EDVTIL-------VNT------SPEKLMG-L-NLSFG-N
XP_023751740.1/1-285 ---------------DG--V-DDVTIQ-------VNS------SPEKLMG-L-NLPFA-N
XP_024971803.1/1-285 ---------------DG--M-DDVTIL-------VNS------SPEKLMG-L-NLSFT-N
XP_022033649.1/1-285 ---------------DG--V-DDVTIL-------VNS------SPEKLMG-L-NLPIA-N
XP_024972195.1/1-285 ---------------DG--V-DDVTIL-------VNS------SPEKLMG-F-NPPFA-N
XP_024972196.1/1-289 ---------------DG--V-DDVTIL-------VNS------SPEKLMG-F-NPPFA-N
XP_020248539.1/1-284 ---------------DG--V-DDVTIL-------VNS------SPSKMMS-L-NLGF-LD
XP_020269197.1/1-284 ---------------DG--V-DDVAIL-------VNS------SPSKMMS-L-NLGF-VN
XP_020269196.1/1-284 ---------------DG--V-DDVAIL-------VNS------SPSKMMS-L-NLGF-VN
XP_009399813.1/1-284 ---------------DG--M-EDITVL-------VNS------SCSKLMG-L-NLSF-VN
XP_009399812.1/1-284 ---------------DG--M-EDITVL-------VNS------SCSKLMG-L-NLSF-VN
XP_009413853.1/1-284 ---------------DG--I-DDVTVF-------VNS------STSKMMG-L-NLGI-IN
XP_024985513.1/1-286 ---------------DG--L-DDITVL-------INS------SPDKVMGA--T-PMYAD
XP_023729871.1/1-286 ---------------DG--V-DDVTVL-------VNS------SPDKVMGA--A-PIYAD
XP_022000974.1/1-282 ---------------DG--V-DDVTVL-------VNS------SPDKVMGA--T-PLYAD
XP_022023495.1/1-282 ---------------DG--I-DDVTVL-------VNS------SPDKVMGA--T-PMYAD
XP_010526102.1/1-286 ---------------DG--I-DDVTVL-------VNS------SPGKMMSS--NLPFSNG
XP_019086035.1/1-282 ---------------DG--I-DDVTLL-------VNS------SPTKMMLTS-SLPF-SN
XP_010437837.1/1-288 ---------------DG--I-DDVTLL-------VNS------SPTKMMMTS-SLPF-SN
XP_010437838.1/1-288 ---------------DG--I-DDVTLL-------VNS------SPTKMMMTS-SLPF-SN
XP_022549566.1/1-283 ---------------DG--I-DDVTLL-------VNS------SPTKMMMTS-SLPF-SN
XP_013670651.1/1-283 ---------------DG--I-DDVTLL-------VNS------SPTKMMMTS-SLPF-SN
XP_020874560.1/1-283 ---------------DG--I-DDVTLL-------VNS------SPTKMMMTS-SLPF-AN
XP_020874561.1/1-283 ---------------DG--I-DDVTLL-------VNS------SPTKMMMTS-SLPF-AN
XP_020874562.1/1-283 ---------------DG--I-DDVTLL-------VNS------SPTKMMMTS-SLPF-AN
NP_195014.1/1-283    ---------------DG--I-DDVTLL-------VNS------SPTKMMMTS-SLPF-AN
XP_006412419.1/1-283 ---------------DG--I-DDVTLL-------VNS------SPTKMMMTS-SLPF-SN
XP_006412418.1/1-283 ---------------DG--I-DDVTLL-------VNS------SPTKMMMTS-SLPF-SN
XP_018471819.1/1-283 ---------------DG--I-DDVTLL-------VNS------SPTKMMMTS-SLPF-SN
XP_009125299.1/1-283 ---------------DG--I-DDVTLL-------VNS------SPTKMMMTS-SLPF-SN
XP_010432643.1/1-283 ---------------DG--I-DDVTLL-------VNS------SPTKMMLTS-SLPF-SN
XP_010432644.1/1-283 ---------------DG--I-DDVTLL-------VNS------SPTKMMLTS-SLPF-SN
XP_006283109.1/1-283 ---------------DG--I-DDVTLL-------VNS------SPTKMMMTS-SLPF-AN
XP_010437839.1/1-283 ---------------DG--I-DDVTLL-------VNS------SPTKMMMTS-SLPF-SN
XP_010437840.1/1-283 ---------------DG--I-DDVTLL-------VNS------SPTKMMMTS-SLPF-SN
XP_010447325.1/1-283 ---------------DG--I-DDVTLL-------VNS------SPTKMMMTS-SLPF-SN
XP_010447326.1/1-283 ---------------DG--I-DDVTLL-------VNS------SPTKMMMTS-SLPF-SN
XP_013622315.1/1-283 ---------------DG--I-DDVTLL-------VNS------SPTKMMMTS-SLPF-LN
XP_013656273.1/1-283 ---------------DG--I-DDVTLL-------VNS------SPTKMMMTS-SLPF-SN
XP_009108824.1/1-283 ---------------DG--I-DDVTLL-------VNS------SPTKMMMTS-SLPF-SN
XP_013730326.1/1-283 ---------------DG--I-DDVTLL-------MNS------SPTKMMMTS-SLPF-SN
XP_013730325.1/1-283 ---------------DG--I-DDVTLL-------MNS------SPTKMMMTS-SLPF-SN
XP_018447492.1/1-283 ---------------DG--I-DDVTLL-------VNS------SPTKMMMTS-SLPF-SN
XP_019415259.1/1-283 ---------------DG--V-DGITLH-------VNS------SP-SKMMGV-NLGY-NN
XP_022980940.1/1-286 ---------------DG--I-DDVTLL-------VNM------SNAKTMMGA-NISY-SN
XP_023523539.1/1-286 ---------------DG--I-DDVTLL-------VNM------SNGKTMMGA-NISY-NN
XP_022941184.1/1-286 ---------------DG--I-DDVTLL-------VTM------SNGKTMMGA-NISY-NN
XP_022134366.1/1-286 ---------------DG--I-DDVTLL-------VNL------SHGKTMMGA-NISY-SN
XP_004136224.1/1-286 ---------------DG--V-DDVTLL-------VNM------SHGKTMMGA-NISY-SN
XP_008466070.1/1-286 ---------------DG--V-DDVTLL-------VNM------SHGKTMMGA-NISY-SN
XP_022976315.1/1-286 ---------------DG--I-DDVTLL-------VNM------SHGKTMMGA-NISY-SN
XP_022937345.1/1-286 ---------------DG--I-DDVTLL-------VNM------SHGKTMMGA-NMSY-SN
XP_023534956.1/1-286 ---------------DG--I-DDVTLL-------VNM------SHGKTMMGA-NMSY-SN
XP_023535295.1/1-286 ---------------DG--I-DDVTLL-------VNM------SHGKTMMGA-NMSY-SN
XP_019175908.1/1-285 ---------------DG--I-DDVTIM-------VNS------STAKLMG-A-NLPYA-N
XP_019175907.1/1-285 ---------------DG--I-DDVTIM-------VNS------STAKLMG-A-NLPYA-N
XP_009626659.1/1-285 ---------------DG--I-DDVTLL-------VNS------SPSKLMG-A-NISYA-N
XP_019266312.1/1-285 ---------------DG--I-DDVTLL-------VNS------SPSKLMG-A-NISYA-N
XP_009781184.1/1-285 ---------------DG--I-DDVTLL-------VNS------SPSKLMG-A-NISYA-N
XP_016488110.1/1-285 ---------------DG--I-DDVTLL-------VNS------SPSKLMG-A-NISYA-N
XP_016537761.1/1-285 ---------------DG--I-DDVTIL-------VNS------SPSKLMG-A-NLSYA-N
XP_006359741.1/1-285 ---------------DG--I-DDVTIL-------VNS------SPSKLMG-A-NLSYA-N
XP_010325078.1/1-288 ---------------DG--V-DDVTIL-------VNS------SPSKLMG-A-NISYA-N
XP_004245155.1/1-285 ---------------DG--V-DDVTIL-------VNS------SPSKLMG-A-NISYA-N
XP_015085523.1/1-285 ---------------DG--I-DDVTIL-------VNS------SPSKLMG-A-NISYA-N
XP_022868182.1/1-285 ---------------DG--I-DDVAVL-------VNS------SPGKLMG-P-NLTYA-N
XP_022868181.1/1-285 ---------------DG--I-DDVAVL-------VNS------SPGKLMG-P-NLTYA-N
XP_022854559.1/1-285 ---------------DG--I-DDVGVL-------VNS------SPGKLMG-P-NLTYA-N
XP_011081776.1/1-285 ---------------DG--I-DDVTVH-------VNS------SPGKLMG-T-ILTYA-N
XP_012857948.1/1-282 ---------------DG--I-DDVTVH-------VNT------SPGKLMG-T-IQTYA-N
XP_021847759.1/1-284 ---------------DG--I-DDVTLL-------VNS------SPSKMMG-E-NQFYS-N
XP_010668539.1/1-284 ---------------DG--I-DDVTLL-------VNS------SPSKMMG-E-NQFYS-N
XP_021739137.1/1-284 ---------------DG--I-DDVTLL-------VNS------SPSKMMG-E-NQFYS-N
XP_021737291.1/1-284 ---------------DG--I-DDVTLL-------VNS------SPSKMMG-E-NQFYS-N
XP_010047286.1/1-285 ---------------DG--V-DDVTLL-------INS------SPAKMAG-V-NISYA-S
XP_003603630.2/1-285 ---------------DG--I-DDVTLL-------VNS------SPSKMMG-L-NLGYN-N
XP_004501146.1/1-286 ---------------DG--I-DDITLL-------VNS------SPSKMMG-V-NLGYN-N
XP_012571693.1/1-286 ---------------DG--I-DDITLL-------VNS------SPSKMMG-V-NLGYN-N
XP_012571692.1/1-286 ---------------DG--I-DDITLL-------VNS------SPSKMMG-V-NLGYN-N
XP_019417530.1/1-285 ---------------DG--I-DDVTLL-------VNS------SPSKMMG-V-NLGYN-N
XP_019417531.1/1-285 ---------------DG--I-DDVTLL-------VNS------SPSKMMG-V-NLGYN-N
XP_016180268.1/1-286 ---------------DG--I-DDVTLL-------VNS------SPSKMMG-A-NLGYNGG
XP_025623723.1/1-286 ---------------DG--I-DDVTLL-------VNS------SPSKMMG-A-NLGYNGG
XP_015945672.1/1-286 ---------------DG--I-DDVTLL-------VNS------SPSKMMG-A-NLGYNGG
XP_007137231.1/1-287 ---------------DG--I-DDVTLL-------VNS------SPSKMMG-A-NLGYNNN
XP_022636494.1/1-293 ---------------DG--I-DDVTLL-------VNS------SPSKMMG-A-NLGYNNN
XP_017409217.1/1-287 ---------------DG--I-DDVTLL-------VNS------SPSKMMG-A-NLGYNTN
XP_014501941.1/1-287 ---------------DG--I-DDVTLL-------VNS------SPSKMMG-A-NLGYNNN
XP_003526496.1/1-286 ---------------DG--I-DDVTLL-------VNS------SPSKMMG-V-SLVYNNN
XP_003522716.1/1-286 ---------------DG--I-DDVTLL-------VNS------SPSKMMG-V-NLGYNNN
XP_020217689.1/1-286 ---------------DG--I-DDVTLL-------VNS------SPSKMMG-V-NLGYNNN
XP_016683069.1/1-283 ---------------DG--V-DDVTLL-------VNS------SPGKMMD-I-NFSYS-N
XP_016677926.1/1-283 ---------------DG--I-DDVTLL-------VNS------SPGKMMD-I-NFSYS-N
XP_017643110.1/1-283 ---------------DG--I-DDVTLL-------VNS------SPGKMMD-I-NFSYS-N
XP_017643111.1/1-283 ---------------DG--I-DDVTLL-------VNS------SPGKMMD-I-NFSYS-N
XP_012451775.1/1-283 ---------------DG--I-DDVTLL-------VNS------SPGKMMD-I-NFSYS-N
XP_016744766.1/1-285 ---------------DG--A-DDITLL-------VNS------SPGKIMG-I-NLSYT-N
XP_017643921.1/1-285 ---------------DG--A-DDITLL-------VNS------SPGKMMG-I-NLSYT-N
XP_012436119.1/1-285 ---------------DG--A-DDITLL-------VNS------SPGKMMG-I-NLSYS-N
XP_016681632.1/1-285 ---------------DG--A-DDITLL-------VNS------SPGKMMG-I-NLSYT-N
XP_016689618.1/1-285 ---------------DG--V-DDVTLL-------VNS------SPSKMMG-I-NLSYG-N
XP_016689617.1/1-285 ---------------DG--V-DDVTLL-------VNS------SPSKMMG-I-NLSYG-N
XP_012445947.1/1-285 ---------------DG--V-DDVTLL-------VNS------SPSKMMG-I-NLSYG-N
XP_012445944.1/1-285 ---------------DG--V-DDVTLL-------VNS------SPSKMMG-I-NLSYG-N
XP_012445945.1/1-285 ---------------DG--V-DDVTLL-------VNS------SPSKMMG-I-NLSYG-N
XP_017614652.1/1-285 ---------------DG--V-DDVTLL-------VNS------SPSKMMG-I-NLSYG-N
XP_016732732.1/1-285 ---------------DG--V-DDVTLL-------VNS------SPSKMMG-I-NLSYG-N
XP_016732731.1/1-285 ---------------DG--V-DDVTLL-------VNS------SPSKMMG-I-NLSYG-N
XP_022754596.1/1-285 ---------------DG--V-DDVTLL-------VNS------SPGKMMG-I-NLSYS-N
XP_022750159.1/1-285 ---------------DG--V-DDVTLL-------VNS------SPGKMMG-I-DLAYS-N
XP_022729737.1/1-285 ---------------DG--V-DDVTLL-------VNS------SPGKMMG-I-NLSYS-N
XP_022729738.1/1-285 ---------------DG--V-DDVTLL-------VNS------SPGKMMG-I-NLSYS-N
XP_021276132.1/1-285 ---------------DG--V-DDVTLL-------VNS------SPGKMMG-I-NLSYS-N
XP_017983822.1/1-285 ---------------DG--V-DDVTLL-------VNS------SPGKMMG-I-NLSYS-N
XP_002283717.3/1-285 ---------------DG--I-DDVTLL-------VNS------SPAKMMG-V-NLSYA-S
XP_024446213.1/1-284 ---------------DG--I-DDVTVL-------VNS------SPTKTMG-V-NFSYD-N
XP_024446215.1/1-284 ---------------DG--I-DDVTVL-------VNS------SPTKTMG-V-NFSYD-N
XP_011006428.1/1-284 ---------------DG--I-DDVTVL-------VNS------SPTKTMG-V-NFSYD-N
XP_024459734.1/1-284 ---------------DG--I-DDVTVL-------VNS------SPAKMMG-V-NFSYA-N
XP_011019595.1/1-284 ---------------DG--I-DDVTVL-------VNS------SPAKMMG-V-NFSYA-N
XP_015573174.1/1-284 ---------------DG--I-DDVTVL-------VNS------SPSKMMG-L-NISYA-S
XP_012077376.1/1-284 ---------------DG--I-DDVTVL-------VNS------SPGKMMG-L-NISYA-S
XP_021608403.1/1-284 ---------------DG--I-DDVTVL-------VNS------SPGKILG-L-NISYA-S
XP_021608402.1/1-284 ---------------DG--I-DDVTVL-------VNS------SPGKILG-L-NISYA-S
XP_021597909.1/1-284 ---------------DG--I-DDVTVL-------VNS------SPGKMMG-L-NISYA-N
XP_021642839.1/1-284 ---------------DG--I-DDVTVL-------VNS------SPGKMMG-L-NISYA-N
XP_021689146.1/1-284 ---------------DG--I-DDVTVL-------VNS------SPGKMMG-L-NISYA-S
XP_018805752.1/1-285 ---------------DG--I-DDVTVL-------VNS------SPAKMMG-G-DISYA-N
XP_023918167.1/1-285 ---------------DG--I-DDVTIL-------VNS------SPAKMMG-G-NLSYA-N
XP_018845499.1/1-285 ---------------DG--I-DDVTIL-------VNS------SPAKMMG-G-SLSYA-N
XP_018845500.1/1-285 ---------------DG--I-DDVTIL-------VNS------SPAKMMG-G-SLSYA-N
NP_001280750.1/1-285 ---------------DG--V-DDVTLL-------VNS------SPGKMMS-A-NL-YT-N
NP_001280796.1/1-285 ---------------DG--V-DDVTLL-------VNS------SPGKMMS-A-NL-YT-D
XP_009367440.1/1-285 ---------------DG--V-DDVTLL-------VNS------SPGKMMS-A-NL-YT-D
XP_017182108.1/1-285 ---------------DG--V-DDVTLL-------VNS------SPSKMMS-A-NL-YT-D
XP_004291167.1/1-285 ---------------DG--V-DDVTLL-------VNS------SPGKMMG-A-NL-YA-N
XP_024162655.1/1-285 ---------------DG--V-DDITLL-------VNS------SPGKMMG-A-NL-YA-N
XP_021819449.1/1-284 ---------------DG--V-DDVTLL-------VNS------SPGK-MG-A-NL-YA-N
XP_007225281.1/1-285 ---------------DG--V-DDVTLL-------VNS------SPGKMMG-A-NL-YA-N
XP_008220092.1/1-285 ---------------DG--V-DDVTLL-------VNS------SPGKMMG-A-NL-YA-N
XP_006450706.1/1-285 ---------------DG--I-DDVTVH-------VNS------SPSKMMG-V-QLSYV-N
XP_006476026.1/1-285 ---------------DG--I-DDVTVH-------VNS------SPSKMMG-V-QLSYV-N
XP_024930942.1/1-285 ---------------DG--I-DDVTLL-------VNS------SPSKMMG-V-NLSYP-N
XP_010102406.2/1-285 ---------------DG--I-DDVTLL-------VNS------SPSKMMG-M-NLPYA-N
XP_024540686.1/1-258 ---------------DG--L-DNVAVS-------CNA------TINFSLS-----KFQ-S
XP_024399220.1/1-283 ---------------DG--M-DDVSVM-------LNA------TPKSMEG-----QIA-T
XP_024399219.1/1-283 ---------------DG--M-DDVSVM-------LNA------TPKSMEG-----QIA-T
XP_024399215.1/1-283 ---------------DG--M-DDVSVM-------LNA------TPKSMEG-----QIA-T
XP_024390746.1/1-284 ---------------DG--L-DDVSVM-------LNA------TPKSMEG-----QIA-S
XP_024390752.1/1-284 ---------------DG--L-DDVSVM-------LNA------TPKSMEG-----QIA-S
XP_024373354.1/1-283 ---------------DG--L-DDVSVM-------VNA------TPKSMEG-----QIA-S
XP_024373357.1/1-283 ---------------DG--L-DDVSVM-------VNA------TPKSMEG-----QIA-S
XP_024370817.1/1-284 ---------------DG--L-EDVSVM-------LNA------TPKSMEG-----QIA-S
XP_024370815.1/1-284 ---------------DG--L-EDVSVM-------LNA------TPKSMEG-----QIA-S
XP_024370814.1/1-284 ---------------DG--L-EDVSVM-------LNA------TPKSMEG-----QIA-S
XP_024386785.1/1-285 ---------------DG--L-DDVSVI-------LNA------TSQSMEG-----QIA-S
XP_024386782.1/1-285 ---------------DG--L-DDVSVI-------LNA------TSQSMEG-----QIA-S
XP_024386791.1/1-285 ---------------DG--L-DDVSVI-------LNA------TSQSMEG-----QIA-S
XP_002961227.1/1-277 ---------------ID--GDVTVAIN-------VAP------NASSIGG-----QVM-P
XP_002969458.1/1-277 ---------------ID--GDVTVAIN-------VAP------NASSIGG-----QVM-P
XP_024533017.1/1-265 ---------------DD----VTISIN-------TAL------GAKAAGA-----Q----
XP_011076983.1/1-261 ---------------DA--P-GDIIVS-------VKR------LKNFE-------DNR-N
XP_011076980.1/1-264 ---------------DA--P-GDIIVS-------VKR------LKNFE-------DNR-N
XP_011076981.1/1-263 ---------------DA--P-GDIIVS-------VKR------LKNFE-------DNR-N
XP_011084330.1/1-264 ---------------NA--S-DDIIMS-------IKR------TASFG-------VGA-N
XP_011084333.1/1-264 ---------------NA--S-DDIIMS-------IKR------TASFG-------VGA-N
XP_011084331.1/1-264 ---------------NA--S-DDIIMS-------IKR------TASFG-------VGA-N
XP_012834933.1/1-260 ---------------DA--S-DDVIMS-------IKR------TTIFG-------AYT-T
XP_012834932.1/1-260 ---------------DA--S-DDVIMS-------IKR------TTIFG-------AYT-T
XP_012834931.1/1-260 ---------------DA--S-DDVIMS-------IKR------TTIFG-------AYT-T
XP_012834934.1/1-260 ---------------DA--S-DDVIMS-------IKR------TTIFG-------AYT-T
XP_002274194.1/1-284 ---------------DG--A-EDLIIS-------VNS------AKNLS-------TIS-N
XP_019079956.1/1-284 ---------------DG--A-EDLIIS-------VNS------AKNLS-------TIS-N
XP_023759583.1/1-283 ---------------DG--V-DDVTVF-------VNL------APAKATGAN-QMYAA-H
XP_020087984.1/1-275 ---------------DG--A-DDVTIL-------VKN------Y-----------GFT-N
XP_015693848.1/1-289 ---------------DG--V-DDVCVS-------VNS------SK--VIGSS-NAAFN-N
XP_015624690.1/1-290 ---------------DG--V-DDVCIS-------VNS------SK--VIGC--NATFS-S
XP_008655743.1/1-292 ---------------DG--V-DDVCVS-------VSS------SPSRVVS-C-NATFS-N
XP_020183693.1/1-281 ---------------DG--V-DDVCIS-------VNS------SPSKVMS-C-TATFS-D
XP_020183687.1/1-281 ---------------DG--V-DDVCIS-------VNS------SPSKVMS-C-TATFS-D
XP_003565084.1/1-284 ---------------DG--V-DDVCIS-------VNS------SLNKVIS-C-NATFG-D
XP_002454995.1/1-286 ---------------DG--V-DDVCIS-------VNS------SPSKVIN-C-NATFN-N
XP_012701181.1/1-286 ---------------DG--V-DDVCIS-------VNS------SPSKVIN-C-NATFN-N
XP_012701180.1/1-286 ---------------DG--V-DDVCIS-------VNS------SPSKVIN-C-NATFN-N
XP_012701179.1/1-286 ---------------DG--V-DDVCIS-------VNS------SPSKVIN-C-NATFN-N
XP_025816436.1/1-287 ---------------DG--V-DDVCIS-------VNS------SPSRVIN-C-NAAFN-N
XP_018726848.1/1-227 ------------------GAG-EMRVI-------TMN-SI--DDP--------G-R----
XP_018726849.1/1-227 ------------------GAG-EMRVI-------TMN-SI--DDP--------G-R----
XP_019193052.1/1-216 ------------------GSD-DVRIM-------IRK-SV--DDP--------G-R----
XP_019193050.1/1-216 ------------------GSD-DVRIM-------IRK-SV--DDP--------G-R----
XP_021834047.1/1-194 ------------------GAD-DVRVM-------TRK-SV--DDP--------G------
XP_010522330.1/1-229 ------------------GAD-DVRVM-------TRK-SL--XEC--------V------
XP_009802524.1/1-219 ------------------SEN-DVRIM-------FRK-SI--NDP--------G-R----
XP_009802526.1/1-219 ------------------SEN-DVRIM-------FRK-SI--NDP--------G-R----
XP_009802525.1/1-219 ------------------SEN-DVRIM-------FRK-SI--NDP--------G-R----
XP_009802527.1/1-219 ------------------SEN-DVRIM-------FRK-SI--NDP--------G-R----
XP_019250148.1/1-219 ------------------SEN-DVRIM-------FRK-SI--NDP--------G-R----
XP_019250149.1/1-219 ------------------SEN-DVRIM-------FRK-SI--NDP--------G-R----
XP_016449923.1/1-219 ------------------SEN-DVRIM-------VRK-SI--NDP--------G-R----
XP_009629760.1/1-219 ------------------SEN-DVRIM-------FRK-SI--NDP--------G-R----
XP_009629762.1/1-219 ------------------SEN-DVRIM-------FRK-SI--NDP--------G-R----
XP_010234287.2/1-230 ------------------GEA-DVRVM-------TRK-SV--DDP--------G-R----
XP_021806177.1/1-204 ------------------GAD-DVRVM-------TRK-SV--DDP--------S-R----
XP_016580642.1/1-229 ------------------TDE-NVRIM-------TRK-SV--DDP--------G-R----
XP_008667763.1/1-266 -------------------AD-DVRVM-------TRK-SV--DDP--------G-R----
XP_006352040.1/1-227 ------------------TDE-NVRVM-------TRK-SV--GDP--------G-R----
XP_009611638.1/1-223 ------------------SDD-NVRVM-------TRK-SV--DDP--------G-R----
XP_009611623.1/1-223 ------------------SDD-NVRVM-------TRK-SV--DDP--------G-R----
XP_016510654.1/1-223 ------------------SDD-NVRVM-------TRK-SV--DDP--------G-R----
XP_009766038.1/1-223 ------------------TDD-NVRVM-------TRK-SV--DDP--------G-R----
XP_020210648.1/1-236 ------------------GGE-DLRVM-------TRK-SV--NDP--------G-R----
XP_007143210.1/1-219 ------------------AGE-DVRVM-------TRK-SV--DDP--------G-R----
XP_014491869.1/1-226 ------------------AGE-DVRVM-------TRK-SV--DDP--------G-R----
XP_022633652.1/1-226 ------------------AGE-DVRVM-------TRK-SV--DDP--------G-R----
XP_017412341.1/1-227 ------------------ASE-DVRVM-------TRK-SV--DDP--------G-R----
XP_022011155.1/1-226 ------------------GLD-DVRIM-------TRK-NV--DDP--------G-R----
XP_003555330.2/1-228 ------------------GLE-DVRVM-------TRK-SV--DDP--------G-R----
XP_014628518.1/1-229 ------------------GLE-DVRVM-------TRK-SV--DDP--------G-R----
XP_017647009.1/1-225 ------------------GSD-EIRVM-------TRK-SI--DDP--------G-R----
XP_016711277.1/1-225 ------------------GSD-EIRVM-------TRK-SI--DDP--------G-R----
XP_012457027.1/1-225 ------------------GSD-EIRVM-------TRK-SI--DDP--------G-R----
XP_016723454.1/1-225 ------------------GSD-EIRVM-------TRK-SI--DDP--------G-R----
XP_020978973.1/1-214 ------------------DMD-DVRVM-------TRK-SM--DDP--------G-R----
XP_016202355.1/1-214 ------------------DMD-DVRVM-------TRK-SM--DDP--------G-R----
XP_015964876.1/1-214 ------------------DMD-DVRVM-------TRK-SM--DDP--------G-R----
XP_025699645.1/1-214 ------------------DMD-DVRVM-------TRK-SM--DDP--------G-R----
XP_008441375.1/1-228 ------------------DSD-DVRVM-------ARK-SL--DDP--------G-R----
XP_004154226.2/1-228 ------------------DSD-DVRVM-------ARK-SL--DDP--------G-R----
XP_023519981.1/1-226 ------------------DSD-HVRVM-------ARK-SL--DDP--------G-R----
XP_023001231.1/1-226 ------------------DSD-HVRVM-------ARK-SL--DDP--------G-R----
XP_022933178.1/1-226 ------------------DSD-HVRVM-------ARK-SL--DDP--------G-R----
XP_004511340.1/1-228 ------------------GPE-DIRVM-------TKK-YN--DEP------GSG-R----
XP_002979015.2/1-232 ------------------GED-DVRVM-------TRK-SV--DNP--------G-E----
XP_002988402.1/1-232 ------------------GED-DVRVM-------TRK-SV--DNP--------G-E----
XP_024390340.1/1-238 ------------------GDD-DVRVM-------TRK-SV--DNP--------G-E----
XP_024368074.1/1-235 ------------------GDD-DVRVM-------TRK-SV--DNP--------G-E----
XP_024401280.1/1-235 ------------------GDD-DVRVM-------TRK-SV--DNP--------G-E----
XP_024394840.1/1-235 ------------------GDD-DVRVM-------TRK-SV--DNP--------G-E----
XP_024394838.1/1-235 ------------------GDD-DVRVM-------TRK-SV--DNP--------G-E----
XP_017256650.1/1-227 ------------------MAE-NVRIM-------TRK-SV--DDP--------G-R----
XP_019186546.1/1-227 ------------------GAD-DVRVM-------TRK-SI--DDP--------G-T----
XP_024988375.1/1-226 ------------------GAD-DVRVM-------TRK-TT--DDA--------G-R----
XP_017231769.1/1-226 ------------------GAD-NVRVM-------TRK-SI--DDP--------G-R----
XP_009108529.1/1-235 ------------------GSD-DVRVM-------TRK-SM--DDP--------G-R----
XP_018469534.1/1-236 ------------------GSD-DVRVM-------TRK-SM--DDP--------G-R----
XP_018467466.1/1-236 ------------------GSD-DVRVM-------TRK-SM--DDP--------G-R----
XP_022543253.1/1-234 ------------------GSD-DVRVM-------TRK-SM--DDP--------G-R----
XP_009135181.1/1-234 ------------------GSD-DVRVM-------TRK-SM--DDP--------G-R----
XP_013642975.1/1-234 ------------------GSD-DVRVM-------TRK-SM--DDP--------G-R----
XP_009135189.1/1-234 ------------------GSD-DVRVM-------TRK-SM--DDP--------G-R----
XP_018515487.1/1-234 ------------------GSD-DVRVM-------TRK-SM--DDP--------G-R----
XP_013623861.1/1-234 ------------------GSD-DVRVM-------TRK-SM--DDP--------G-R----
XP_013623905.1/1-234 ------------------GSD-DVRVM-------TRK-SM--DDP--------G-R----
XP_013623953.1/1-236 ------------------GSD-DVRVM-------TRK-SM--DDP--------G-R----
XP_013623587.1/1-236 ------------------GSD-DVRVM-------TRK-SM--DDP--------G-R----
XP_013623995.1/1-236 ------------------GSD-DVRVM-------TRK-SM--DDP--------G-R----
XP_013623820.1/1-236 ------------------GSD-DVRVM-------TRK-SM--DDP--------G-R----
XP_022926381.1/1-227 ------------------GSD-DVRVV-------TRK-ST--DEP--------G-R----
XP_023517485.1/1-227 ------------------GSD-DVRVV-------TRK-ST--DEP--------G-R----
XP_023002930.1/1-227 ------------------GSD-DVRVV-------TRK-ST--DEP--------G-R----
XP_004134971.1/1-229 ------------------AGD-EVRVV-------TRK-ST--DEP--------G-R----
XP_008439968.1/1-229 ------------------AGD-EVRVV-------TRK-ST--DEP--------G-R----
XP_022950176.1/1-227 ------------------AGD-DVRVV-------TRK-ST--DEP--------G-R----
XP_022979090.1/1-227 ------------------AGD-DVRVV-------TRK-ST--DEP--------G-R----
XP_023543388.1/1-249 ------------------AGD-DVRVV-------TRK-ST--DEP--------G-R----
XP_023543387.1/1-227 ------------------AGD-DVRVV-------TRK-ST--DEP--------G-R----
XP_009134323.1/1-228 ------------------GSD-DVRVM-------TRK-SM--DDP--------G-R----
XP_019176002.1/1-227 ------------------GAD-DVRVM-------TRK-SI--DDP--------G-R----
XP_015070655.1/1-221 ------------------GAD-DVRVM-------TRK-SI--DDP--------G-R----
XP_015070652.1/1-221 ------------------GAD-DVRVM-------TRK-SI--DDP--------G-R----
XP_025885589.1/1-221 ------------------GAD-DVRVM-------TRK-SI--DDP--------G-R----
XP_019422236.1/1-227 ------------------GCD-DVRVM-------TRK-SM--DDP--------G-R----
XP_007157166.1/1-226 ------------------GSD-DVRVM-------TRK-ST--DEP--------G-R----
XP_003538765.1/1-227 ------------------GCD-DVRVM-------TRK-ST--DEP--------G-R----
XP_003517589.1/1-227 ------------------GCD-DVRVM-------TRK-ST--DEP--------G-R----
XP_006573890.1/1-227 ------------------GCD-DVRVM-------TRK-ST--DEP--------G-R----
XP_011000687.1/1-226 ------------------GSD-DVRVM-------TRK-SM--DDP--------G-R----
XP_024436817.1/1-226 ------------------GSD-DVRVM-------TRK-SM--DDP--------G-R----
XP_024436820.1/1-226 ------------------GSD-DVRVM-------TRK-SM--DDP--------G-R----
XP_002518520.2/1-226 ------------------GSD-DVRVM-------TRK-SM--DDP--------G-R----
XP_015574304.1/1-226 ------------------GSD-DVRVM-------TRK-SM--DDP--------G-R----
XP_021642058.1/1-227 ------------------GSD-DIRVM-------TRK-SM--DDP--------G-R----
XP_012091395.1/1-226 ------------------GSD-DVRVM-------TRK-SM--DDP--------G-R----
XP_021635824.1/1-226 ------------------GSD-DVRVM-------TRK-SM--DDP--------G-R----
XP_021596887.1/1-226 ------------------GSD-DVRVM-------TRK-SM--DDP--------G-R----
XP_010057969.1/1-228 ------------------GSDNNVRVM-------TRK-SM--DEP--------G-R----
XP_006413738.1/1-245 ------------------GSD-DVRVM-------TRK-SM--DDP--------G-R----
XP_013630219.1/1-235 ------------------GSD-DVRVM-------TRK-SM--DDP--------G-R----
XP_013656136.1/1-235 ------------------GSD-DVRVM-------TRK-SM--DDP--------G-R----
XP_013685757.1/1-235 ------------------RSD-DVRVM-------TRK-SM--DDP--------G-R----
XP_013696499.1/1-240 ------------------GSD-DVRVM-------TRK-SM--DDP--------G-R----
XP_009137213.1/1-240 ------------------GSD-DVRVM-------TRK-SM--DDP--------G-R----
XP_013598423.1/1-241 ------------------GSD-DVRVM-------TRK-SM--DDP--------G-R----
XP_013717184.1/1-241 ------------------GSD-DVRVM-------TRK-SM--DDP--------G-R----
XP_018480642.1/1-241 ------------------GSD-DVRVM-------TRK-SM--DDP--------G-R----
XP_018477464.1/1-241 ------------------GSD-DVRVM-------TRK-SM--DDP--------G-R----
NP_193906.2/1-248    ------------------GSD-DVRVM-------TRK-SM--DDP--------G-R----
XP_020873301.1/1-248 ------------------GSD-DVRVM-------TRK-SM--DDP--------G-R----
XP_006285476.1/1-249 ------------------GSD-DVRVM-------TRK-SM--DDP--------G-R----
XP_010434132.1/1-252 ------------------GSD-DVRVM-------TRK-SM--DDP--------G-R----
XP_010439429.1/1-252 ------------------GSD-DVRVM-------TRK-SM--DDP--------G-R----
XP_010449009.1/1-254 ------------------GSD-DVRVM-------TRK-SM--DDP--------G-R----
XP_010432053.1/1-234 ------------------GSD-DVRVM-------TRK-SM--DDP--------G-R----
XP_020876375.1/1-234 ------------------GSD-DVRVM-------TRK-SM--DDP--------G-R----
XP_023636874.1/1-234 ------------------GSD-DVRVM-------TRK-SM--DDP--------G-R----
XP_010422328.1/1-234 ------------------GSD-DVRVM-------TRK-SM--DDP--------G-R----
XP_010455798.1/1-234 ------------------GSD-DVRVM-------TRK-SM--DDP--------G-R----
NP_567274.1/1-235    ------------------GSD-DVRVM-------TRK-SM--DDP--------G-R----
XP_024009884.1/1-236 ------------------GSD-DVRVM-------TRK-SM--DDP--------G-R----
XP_018452346.1/1-236 ------------------GSD-DVRVM-------TRK-SM--DDP--------G-R----
XP_013611160.1/1-236 ------------------GSD-DVRVM-------TRK-SM--DDP--------G-R----
XP_009114536.1/1-235 ------------------GSD-DVRVM-------TRK-SM--DDP--------G-R----
XP_013662000.1/1-235 ------------------GSD-DVRVM-------TRK-SM--DDP--------G-R----
XP_018472664.1/1-233 ------------------GSD-DVRVM-------TRK-SM--DDP--------G-R----
XP_022555047.1/1-234 ------------------GSD-DVRVM-------TRK-SM--DDP--------G-R----
XP_013735469.1/1-234 ------------------GSD-DVRVM-------TRK-SM--DDP--------G-R----
XP_013629800.1/1-234 ------------------GSD-DVRVM-------TRK-SM--DDP--------G-R----
XP_013735464.1/1-234 ------------------GSD-DVRVM-------TRK-SM--DDP--------G-R----
XP_022571893.1/1-234 ------------------GSD-DVRVM-------TRK-SM--DDP--------G-R----
XP_010538642.1/1-236 ------------------GSD-DVRVM-------TRK-SM--DDP--------G-R----
XP_019057303.1/1-229 ------------------GSD-DVRVM-------TRK-SM--DDP--------G-R----
XP_016545426.1/1-226 ------------------GAD-DVRVM-------TRK-SI--DDP--------G-R----
XP_009793614.1/1-227 ------------------GAD-DVRVM-------TRK-SI--DDP--------G-R----
XP_016508926.1/1-227 ------------------GAD-DVRVM-------TRK-SI--DDP--------G-R----
XP_016560096.1/1-235 ------------------GAD-DVRVM-------TRK-SI--DDP--------G-R----
XP_021993721.1/1-227 ------------------GAD-DVRVM-------TRK-SV--DDP--------G-R----
XP_022036928.1/1-226 ------------------GPD-DVRVM-------TRK-SM--DDP--------G-R----
XP_023742870.1/1-226 ------------------GGD-DVRVM-------TRK-SV--DDP--------G-R----
XP_006352605.1/1-223 ------------------GAD-DVRVM-------TRK-SI--DDP--------G-R----
XP_010327237.1/1-223 ------------------GAD-DVRVM-------TRK-SI--DDP--------G-R----
XP_015055984.1/1-223 ------------------GAD-DVRVM-------TRK-SI--DDP--------G-R----
XP_009609606.1/1-229 ------------------GAD-DVRVM-------TRK-SI--DDP--------G-R----
XP_009609608.1/1-229 ------------------GAD-DVRVM-------TRK-SI--DDP--------G-R----
XP_018628742.1/1-229 ------------------GAD-DVRVM-------TRK-SI--DDP--------G-R----
XP_019237831.1/1-229 ------------------GAD-DVRVM-------TRK-SI--DDP--------G-R----
XP_018849836.1/1-227 ------------------GSD-EVRVM-------TRK-SM--DDP--------G-R----
XP_007214541.1/1-230 ------------------GSD-DVRVM-------TRK-SM--DDP--------G-R----
XP_021821565.1/1-230 ------------------GSD-DVRVM-------TRK-SM--DDP--------G-R----
XP_008224792.1/1-230 ------------------GSD-DVRVM-------TRK-SM--DDP--------G-R----
XP_024198966.1/1-227 ------------------GSD-DVRVM-------TRK-SM--DDP--------G-R----
XP_004293268.1/1-227 ------------------GSD-DVRVM-------TRK-SM--DDP--------G-R----
XP_009364098.1/1-227 ------------------GSD-DVRVM-------TRK-SM--DDP--------G-R----
XP_008371114.1/1-227 ------------------GSD-DVRVM-------TRK-SM--DDP--------G-R----
XP_008384034.1/1-227 ------------------GSD-DVRVM-------TRK-SM--DDP--------G-R----
XP_009340946.1/1-227 ------------------GSD-DVRVM-------TRK-SM--DDP--------G-R----
XP_009357415.1/1-227 ------------------GSD-DVRVM-------TRK-SM--DDP--------G-R----
XP_011037708.1/1-226 ------------------GSD-DVRVM-------TRK-SM--DDP--------G-R----
XP_024454819.1/1-226 ------------------GSD-DVRVM-------TRK-SM--DDP--------G-R----
XP_015884145.1/1-226 ------------------GSD-DVRVM-------TRK-SM--DDP--------G-R----
XP_024024693.1/1-228 ------------------GSD-DVRVM-------TRK-SM--DDP--------G-R----
XP_024024692.1/1-228 ------------------GSD-DVRVM-------TRK-SM--DDP--------G-R----
XP_018820351.1/1-226 ------------------GSD-DVRVM-------TRK-SM--DDP--------G-R----
XP_023904570.1/1-226 ------------------GSD-DVRVM-------TRK-SM--DDP--------G-R----
XP_023904569.1/1-226 ------------------GSD-DVRVM-------TRK-SM--DDP--------G-R----
XP_006449076.1/1-226 ------------------GSD-DVRVM-------TRK-SM--DDP--------G-R----
XP_006468235.1/1-226 ------------------GSD-DVRVM-------TRK-SM--DDP--------G-R----
XP_022721034.1/1-226 ------------------GSD-DVKVM-------TRK-SM--DDP--------G-R----
XP_022721035.1/1-226 ------------------GSD-DVKVM-------TRK-SM--DDP--------G-R----
XP_022724746.1/1-226 ------------------GSD-DVRVM-------TRK-SM--DDP--------G-R----
XP_007026002.1/1-226 ------------------GSD-DVRVM-------TRK-SM--DDP--------G-R----
XP_021294392.1/1-226 ------------------GSD-DVRVM-------TRK-SM--DDP--------G-R----
XP_022777405.1/1-226 ------------------GSD-DVRVM-------TRK-SM--DDP--------G-R----
XP_017615807.1/1-226 ------------------GSD-DVRVM-------TRK-SM--DDP--------G-R----
XP_016753649.1/1-226 ------------------GSD-DVRVM-------TRK-SM--DDP--------G-R----
XP_016683607.1/1-226 ------------------GSD-DVRVM-------TRK-SM--DDP--------G-R----
XP_012451363.1/1-226 ------------------GSD-DVRVM-------TRK-SM--DDP--------G-R----
XP_002266688.1/1-226 ------------------GAD-DVRVM-------TRK-SM--DDP--------G-R----
XP_021853797.1/1-224 ------------------GAD-DVRVM-------TRK-SM--DDP--------G-R----
XP_021719656.1/1-227 ------------------GAD-DVRVM-------TRK-SM--DDP--------G-R----
XP_021766927.1/1-227 ------------------GAD-DVRVM-------TRK-SM--DDP--------G-R----
XP_010665524.1/1-227 ------------------GAD-DVRVM-------TRK-SM--DDP--------G-R----
XP_009605864.1/1-234 ------------------GAD-DVRVM-------TRK-SI--DDP--------G-R----
XP_019223778.1/1-233 ------------------GAD-DVRVM-------TRK-SI--DDP--------G-R----
XP_016440385.1/1-233 ------------------GAD-DVRVM-------TRK-SI--DDP--------G-R----
XP_009790281.1/1-233 ------------------GAD-DVRVM-------TRK-SI--DDP--------G-R----
XP_016570434.1/1-222 ------------------GAD-DVRVM-------TRK-SI--DDP--------G-R----
XP_016486479.1/1-229 ------------------GAD-DVRVM-------TRK-SI--DDP--------G-R----
XP_009597904.1/1-229 ------------------GAD-DVRVM-------TRK-SI--DDP--------G-R----
XP_009799795.1/1-229 ------------------GAD-DVRVM-------TRK-SI--DDP--------G-R----
XP_019249652.1/1-229 ------------------GAD-DVRVM-------TRK-SI--DDP--------G-R----
XP_006343296.1/1-221 ------------------GAD-DVRVM-------TRK-SI--DDP--------G-R----
XP_020217012.1/1-226 ------------------GCD-DVRVM-------TRK-SA--DDP--------G-R----
XP_017406677.1/1-226 ------------------GCD-DVRVM-------TRK-ST--DEP--------G-R----
XP_014519998.1/1-226 ------------------GCD-DVRVM-------TRK-ST--DEP--------G-R----
XP_014519997.1/1-226 ------------------GCD-DVRVM-------TRK-ST--DEP--------G-R----
XP_012841999.1/1-239 ------------------GAD-DVRVM-------TRK-SM--DDP--------G-R----
XP_024984347.1/1-227 ------------------GAD-DVRVM-------TRK-SV--DDP--------G-R----
XP_023751202.1/1-227 ------------------GAD-DVRVM-------TRK-SV--DDP--------G-R----
XP_023751208.1/1-227 ------------------GAD-DVRVM-------TRK-SV--DDP--------G-R----
XP_022041933.1/1-227 ------------------GAD-DVRVM-------TRK-SV--DDP--------G-R----
XP_024988632.1/1-226 ------------------GAD-DVRVM-------TRK-SM--NDP--------G-R----
XP_022018253.1/1-226 ------------------GAD-DVRVM-------TRK-SM--DDP--------G-R----
XP_023771293.1/1-226 ------------------GAD-DVRVM-------TRK-SM--DDP--------G-R----
XP_019170324.1/1-226 ------------------GAD-DVRVM-------TRK-SI--DDP--------G-R----
XP_017230122.1/1-226 ------------------GAD-DVRVM-------TRK-SI--DDP--------G-R----
XP_011096101.1/1-226 ------------------GAD-DVRVM-------TRK-SM--DDP--------G-R----
XP_012848972.1/1-227 ------------------GAD-DVRVM-------TRK-SM--DDP--------G-R----
XP_022876975.1/1-227 ------------------GAD-DVRVM-------TRK-SV--DDP--------G-R----
XP_022863158.1/1-227 ------------------GAD-DVRVM-------TRK-SV--DDP--------G-R----
XP_022877991.1/1-227 ------------------GAD-DVRVM-------TRK-SV--DDP--------G-R----
XP_019223745.1/1-223 ------------------SDD-NVRVM-------TRK-SV--DDP--------G-R----
XP_021800220.1/1-221 ------------------GAD-DVQVM-------TRN-SV--DDP--------G-R----
XP_021684589.1/1-235 ------------------GAD-DVRVT-------TRK-SV--DDP--------G-R----
XP_021684519.1/1-235 ------------------GAD-DVRVT-------TRK-SV--DDP--------G-R----
XP_021684722.1/1-235 ------------------GAD-DVRVT-------TRK-SV--DDP--------G-R----
XP_021633498.1/1-230 ------------------GAD-DVRVM-------TRK-SI--DDL--------G-R----
XP_009420270.1/1-216 ------------------GAE-DVRVM-------TRK-SV--GDP--------G-R----
XP_003573403.1/1-232 ------------------GAE-DVRVM-------TRK-SV--DDP--------G-R----
XP_006659894.1/1-229 ------------------GAE-DVRVM-------TRK-SV--DDP--------G-R----
XP_015650022.1/1-263 ------------------GAE-DVRVM-------TRK-SV--DDP--------G-R----
XP_015650023.1/1-230 ------------------GAE-DVRVM-------TRK-SV--DDP--------G-R----
XP_020407343.1/1-231 ------------------GAE-DVRVM-------TRK-SV--DDP--------G-R----
NP_001105127.2/1-231 ------------------GAE-DVRVM-------TRK-SV--DDP--------G-R----
XP_008661805.1/1-239 ------------------GAE-DVRVM-------TRK-SV--DDP--------G-R----
NP_001145750.1/1-239 ------------------GAE-DVRVM-------TRK-SV--DDP--------G-R----
XP_025821536.1/1-230 ------------------GAE-DVRVM-------TRK-SV--DDP--------G-R----
XP_002443965.1/1-229 ------------------GAE-DVRVM-------TRK-SV--DDP--------G-R----
XP_004972697.1/1-229 ------------------GAE-DVRVM-------TRK-SV--DDP--------G-R----
XP_008667765.1/1-230 ------------------GAD-DVRVM-------TRK-SV--DDP--------G-R----
NP_001347891.1/1-231 ------------------GAD-DVRVM-------TRK-SV--DDP--------G-R----
XP_015636340.1/1-229 ------------------GAE-DVRVM-------TRK-SV--DDP--------G-R----
XP_015636338.1/1-229 ------------------GAE-DVRVM-------TRK-SV--DDP--------G-R----
XP_006652859.1/1-228 ------------------GAE-DVRVM-------TRK-SV--DDP--------G-R----
XP_003580598.1/1-233 ------------------GAE-DVRVM-------TRQ-SV--DDP--------G-R----
XP_020199198.1/1-227 ------------------GAE-DVRVM-------TRK-SV--DDP--------G-R----
XP_020400772.1/1-230 ------------------GAE-DVRVM-------TRK-SV--DDP--------G-R----
NP_001334663.1/1-230 ------------------GAE-DVRVM-------TRK-SV--DDP--------G-R----
XP_008662197.1/1-270 ------------------GAE-DVRVM-------TRK-SV--DDP--------G-R----
XP_008662196.1/1-270 ------------------GAE-DVRVM-------TRK-SV--DDP--------G-R----
XP_025823505.1/1-227 ------------------GAE-DVRVM-------TRK-SV--DDP--------G-R----
XP_004976894.1/1-227 ------------------GAE-DVRVM-------TRK-SV--DDP--------G-R----
XP_002448563.2/1-227 ------------------GAE-DVRVM-------TRK-SV--DDP--------G-R----
XP_019414154.1/1-227 ------------------GSD-DVRVM-------TRK-SV--DDP--------G-R----
XP_006348085.1/1-234 ------------------GAD-DVRVM-------TRK-SI--DDP--------G-R----
XP_004232734.1/1-234 ------------------GAD-DVRVM-------TRK-SI--DDP--------G-R----
XP_015066898.1/1-234 ------------------GAD-DVRVM-------TRK-SI--DDP--------G-R----
XP_003605900.1/1-232 ------------------GAD-DVRVM-------TRK-SV--DDP--------G-R----
XP_022988693.1/1-229 ------------------GAD-DVRVM-------TRK-SI--DDP--------G-R----
XP_022930609.1/1-229 ------------------GAD-DVRVM-------TRK-SI--DDP--------G-R----
XP_023530197.1/1-229 ------------------GAD-DVRVM-------TRK-SI--DDP--------G-R----
XP_016188415.1/1-237 ------------------GVD-DVRVM-------TRK-SV--DDP--------G-R----
XP_011628991.1/1-236 ------------------GAE-DVRVM-------TRK-SI--DDP--------G-R----
XP_006826512.2/1-236 ------------------GAE-DVRVM-------TRK-SI--DDP--------G-R----
XP_020177398.1/1-220 ------------------GAE-DVRVM-------TRK-SV--DDP--------G-R----
XP_015695486.1/1-219 ------------------GAE-DVRVM-------TRK-SV--DDP--------G-R----
XP_015648539.1/1-217 ------------------GAE-DVRVM-------TRK-SV--DDP--------G-R----
XP_020401599.1/1-218 ------------------GAE-DVRVM-------TRK-SV--DDP--------G-R----
XP_020401598.1/1-218 ------------------GAE-DVRVM-------TRK-SV--DDP--------G-R----
XP_020401597.1/1-218 ------------------GAE-DVRVM-------TRK-SV--DDP--------G-R----
XP_021319954.1/1-239 ------------------GAE-DVRVM-------TRK-SV--DDP--------G-R----
XP_002445014.2/1-239 ------------------GAE-DVRVM-------TRK-SV--DDP--------G-R----
XP_025819913.1/1-218 ------------------GPE-DVRVM-------TRK-SV--DDP--------G-R----
XP_004972909.1/1-218 ------------------GPE-DVRVM-------TRK-SV--DDP--------G-R----
XP_020100432.1/1-229 ------------------GAE-DVRVM-------TRK-SI--DDP--------G-R----
XP_009385059.1/1-228 ------------------GAE-DVRVM-------TRK-SV--DDP--------G-R----
XP_018678797.1/1-226 ------------------GAE-DVRVM-------TRK-SV--DDP--------G-R----
XP_009412012.1/1-233 ------------------GAE-DVRVM-------TRK-SV--DDP--------G-R----
XP_018685149.1/1-236 ------------------GAE-DVRVM-------TRK-SV--DDP--------G-R----
XP_020591278.1/1-230 ------------------GAE-DVRVM-------TRK-SV--DDP--------G-R----
XP_020702932.1/1-229 ------------------GAE-DVRVM-------TRK-SV--DDP--------G-R----
XP_020702933.1/1-230 ------------------GAE-DVRVM-------TRK-SV--DDP--------G-R----
XP_020702931.1/1-230 ------------------GAE-DVRVM-------TRK-SV--DDP--------G-R----
XP_020692781.1/1-229 ------------------GAE-DVRVM-------TRK-SV--DDP--------G-R----
XP_020692780.1/1-229 ------------------GAE-DVRVM-------TRK-SV--DDP--------G-R----
XP_020591591.1/1-229 ------------------GAE-DVRVM-------TRK-SL--DDP--------G-R----
XP_020591590.1/1-229 ------------------GAE-DVRVM-------TRK-SL--DDP--------G-R----
XP_020109359.1/1-227 ------------------GAE-DVRVM-------TRK-SV--DDP--------G-R----
XP_020270114.1/1-229 ------------------GAE-DVRVM-------TRK-SI--DDP--------G-R----
XP_010908855.1/1-227 ------------------GAE-DVRVM-------TRK-SV--DDP--------G-R----
XP_008808987.1/1-227 ------------------GAE-DVRVM-------TRK-SV--DDP--------G-R----
XP_008784552.1/1-223 ------------------GAE-DVRVM-------TRK-SI--DDP--------G-R----
XP_010931820.1/1-223 ------------------GAE-DVRVM-------TRK-SV--DDP--------G-R----
XP_003540308.2/1-226 ------------------GAD-DVRVM-------TRK-SV--DDP--------G-R----
XP_006592818.1/1-226 ------------------GAD-DVRVM-------TRK-SV--DDP--------G-R----
XP_006592816.1/1-226 ------------------GAD-DVRVM-------TRK-SV--DDP--------G-R----
XP_003543387.1/1-226 ------------------GAD-DVRVM-------TRK-SV--DDP--------G-R----
XP_020223651.1/1-226 ------------------GAD-DVRVM-------TRK-SV--DDP--------G-R----
XP_020223648.1/1-226 ------------------GAD-DVRVM-------TRK-SV--DDP--------G-R----
XP_007149968.1/1-226 ------------------GAD-DVRVM-------TRK-SV--DDP--------G-R----
XP_017425582.1/1-226 ------------------GAD-DVRVM-------TRK-SV--DDP--------G-R----
XP_014501634.1/1-226 ------------------GAD-DVRVM-------TRK-SV--DDP--------G-R----
XP_022776632.1/1-226 ------------------GAD-DVRVM-------TRK-SV--DDP--------G-R----
XP_022776631.1/1-226 ------------------GAD-DVRVM-------TRK-SV--DDP--------G-R----
XP_022776629.1/1-226 ------------------GAD-DVRVM-------TRK-SV--DDP--------G-R----
XP_022733641.1/1-226 ------------------GAD-DVRVM-------TRK-SV--DDP--------G-R----
XP_022733640.1/1-226 ------------------GAD-DVRVM-------TRK-SV--DDP--------G-R----
XP_022733638.1/1-226 ------------------GAD-DVRVM-------TRK-SV--DDP--------G-R----
XP_007045612.1/1-226 ------------------GAD-DVRVM-------TRK-SV--DDP--------G-R----
XP_007045611.1/1-226 ------------------GAD-DVRVM-------TRK-SV--DDP--------G-R----
XP_017971478.1/1-226 ------------------GAD-DVRVM-------TRK-SV--DDP--------G-R----
XP_021292769.1/1-226 ------------------GAD-DVRVM-------TRK-SV--DDP--------G-R----
XP_021292767.1/1-226 ------------------GAD-DVRVM-------TRK-SV--DDP--------G-R----
XP_021292765.1/1-226 ------------------GAD-DVRVM-------TRK-SV--DDP--------G-R----
XP_010657309.1/1-226 ------------------GAD-DVRVM-------TRK-SV--DDP--------G-R----
XP_010657313.1/1-226 ------------------GAD-DVRVM-------TRK-SV--DDP--------G-R----
XP_010657310.1/1-226 ------------------GAD-DVRVM-------TRK-SV--DDP--------G-R----
XP_010657311.1/1-226 ------------------GAD-DVRVM-------TRK-SV--DDP--------G-R----
XP_012086347.1/1-228 ------------------GAD-DVRVM-------TRK-SV--DDP--------G-R----
XP_012086344.1/1-228 ------------------GAD-DVRVM-------TRK-SV--DDP--------G-R----
XP_024029967.1/1-226 ------------------GAD-DVRVM-------TRK-RV--DDP--------G-R----
XP_010109517.1/1-226 ------------------GAD-DVRVM-------TRK-RV--DDP--------G-R----
XP_006469697.1/1-226 ------------------GAD-DVRVM-------TRK-SV--DDP--------G-R----
XP_024046029.1/1-226 ------------------GAD-DVRVM-------TRK-SV--DDP--------G-R----
XP_020234199.1/1-226 ------------------GAD-DVRVM-------TRK-SV--DDP--------G-R----
XP_020234201.1/1-226 ------------------GAD-DVRVM-------TRK-SV--DDP--------G-R----
XP_021630856.1/1-226 ------------------GAD-DVRVM-------TRK-SV--DDP--------G-R----
XP_021630853.1/1-226 ------------------GAD-DVRVM-------TRK-SV--DDP--------G-R----
XP_021907367.1/1-221 ------------------GAD-DVRVM-------TRK-SV--DDP--------G-R----
XP_010275996.1/1-221 ------------------GAE-DVRVM-------TRK-SI--DDP--------G-R----
XP_020870784.1/1-223 ------------------GAE-DVRVM-------TRK-SV--DDP--------G-R----
NP_172015.1/1-223    ------------------GAE-DVRVM-------TRK-SV--DDP--------G-R----
XP_006418050.1/1-223 ------------------GAE-DVRVM-------TRK-SV--DDR--------G-R----
XP_018447753.1/1-223 ------------------GAE-DVRVM-------TRK-SV--DDP--------G-R----
XP_010457605.1/1-218 ------------------GAE-DVRVM-------TRK-SV--DDP--------G-R----
XP_023632765.1/1-218 ------------------GAE-DVRVM-------TRK-SV--DDP--------G-R----
XP_010484829.1/1-218 ------------------GAE-DVRVM-------TRK-SV--DDP--------G-R----
XP_010475208.1/1-218 ------------------GAE-DVRVM-------TRK-SV--DDP--------G-R----
XP_018487097.1/1-220 ------------------GAE-DVRVM-------TRK-SV--DDP--------G-R----
XP_013664795.1/1-220 ------------------GAE-DVRVM-------TRK-SV--DDP--------G-R----
XP_013716253.1/1-220 ------------------GAE-DVRVM-------TRK-SV--DDP--------G-R----
XP_009118607.1/1-220 ------------------GAE-DVRVM-------TRK-SV--DDP--------G-R----
XP_013605632.1/1-220 ------------------GAE-DVRVM-------TRK-SV--DDP--------G-R----
XP_010475204.1/1-220 ------------------GAE-DVRVM-------TRK-SV--DDP--------G-R----
XP_010457604.1/1-220 ------------------GAE-DVRVM-------TRK-SV--DDP--------G-R----
XP_023632762.1/1-220 ------------------GAE-DVRVM-------TRK-SV--DDP--------G-R----
XP_010484823.1/1-220 ------------------GAE-DVRVM-------TRK-SV--DDP--------G-R----
XP_022558401.1/1-220 ------------------GAE-DVRVM-------TRK-SV--DDP--------G-R----
XP_013586641.1/1-220 ------------------GAE-DVRVM-------TRK-SV--DDP--------G-R----
XP_022558399.1/1-220 ------------------GAE-DVRVM-------TRK-SV--DDP--------G-R----
XP_022558400.1/1-220 ------------------GAE-DVRVM-------TRK-SV--DDP--------G-R----
XP_013586642.1/1-220 ------------------GAE-DVRVM-------TRK-SV--DDP--------G-R----
XP_013696832.1/1-220 ------------------GAE-DVRVM-------TRK-SV--DDP--------G-R----
XP_022548878.1/1-220 ------------------GAE-DVRVM-------TRK-SV--DDP--------G-R----
XP_013666552.1/1-220 ------------------GAE-DVRVM-------TRK-SV--DDP--------G-R----
XP_009119177.1/1-220 ------------------GAE-DVRVM-------TRK-SV--DDP--------G-R----
XP_020964430.1/1-225 ------------------GAD-DVRVM-------TRK-SV--DDP--------G-R----
XP_015936069.1/1-225 ------------------GAD-DVRVM-------TRK-SV--DDP--------G-R----
XP_020964429.1/1-225 ------------------GAD-DVRVM-------TRK-SV--DDP--------G-R----
XP_015936068.1/1-225 ------------------GAD-DVRVM-------TRK-SV--DDP--------G-R----
XP_015577880.1/1-228 ------------------GAD-DVRVM-------TRK-SV--DDP--------G-R----
XP_002524172.2/1-228 ------------------GAD-DVRVM-------TRK-SV--DDP--------G-R----
XP_007132557.1/1-221 ------------------GAD-DVRVM-------TRK-SV--DDP--------G-R----
XP_017433694.1/1-221 ------------------GAD-DVRVM-------TRK-SV--DDP--------G-R----
XP_014494419.1/1-221 ------------------GAD-DVRVM-------TRK-SV--DDP--------G-R----
XP_022634310.1/1-221 ------------------GAD-DVRVM-------TRK-SV--DDP--------G-R----
XP_010267735.1/1-225 ------------------GAE-DVRVM-------TRK-SI--DDP--------G-R----
XP_010267734.1/1-225 ------------------GAE-DVRVM-------TRK-SI--DDP--------G-R----
XP_024185199.1/1-224 ------------------GAD-DVRVM-------TRK-SV--DDP--------G-R----
XP_024185197.1/1-224 ------------------GAD-DVRVM-------TRK-SV--DDP--------G-R----
XP_009342246.1/1-222 ------------------GAD-DVRVM-------TRK-SV--DDP--------G-R----
XP_009342244.1/1-222 ------------------GAD-DVRVM-------TRK-SV--DDP--------G-R----
XP_009342240.1/1-222 ------------------GAD-DVRVM-------TRK-SV--DDP--------G-R----
XP_020416503.1/1-221 ------------------GAD-DVRVM-------TRK-SV--DDP--------G-R----
XP_021822854.1/1-221 ------------------GAD-DVRVM-------TRK-SV--DDP--------G-R----
XP_007217584.1/1-221 ------------------GAD-DVRVM-------TRK-SV--DDP--------G-R----
XP_021822852.1/1-221 ------------------GAD-DVRVM-------TRK-SV--DDP--------G-R----
XP_018730806.1/1-228 ------------------GAD-DVRVM-------TRK-SI--DDP--------G-R----
XP_010051064.1/1-228 ------------------GAD-DVRVM-------TRK-SI--DDP--------G-R----
XP_015881130.2/1-229 ------------------GAE-DVRVM-------TRK-SV--DDP--------G-R----
XP_023534533.1/1-219 ------------------GAD-DVRVM-------TRK-SI--DDP--------G-R----
XP_022947095.1/1-219 ------------------GAD-DVRVM-------TRK-SI--DDP--------G-R----
XP_022947096.1/1-219 ------------------GAD-DVRVM-------TRK-SI--DDP--------G-R----
XP_022970950.1/1-219 ------------------GAD-DVRVM-------TRK-SI--DDP--------G-R----
XP_022970951.1/1-219 ------------------GAD-DVRVM-------TRK-SI--DDP--------G-R----
XP_022140692.1/1-217 ------------------GAD-DVRVM-------TRK-SI--DDP--------G-R----
XP_022140691.1/1-217 ------------------GAD-DVRVM-------TRK-SI--DDP--------G-R----
XP_008455853.1/1-220 ------------------GAD-DVRVM-------TRK-SI--DDP--------G-R----
XP_011650012.1/1-220 ------------------GAD-DVRVM-------TRK-SI--DDP--------G-R----
XP_008455850.1/1-220 ------------------GAD-DVRVM-------TRK-SI--DDP--------G-R----
XP_011650011.1/1-220 ------------------GAD-DVRVM-------TRK-SI--DDP--------G-R----
XP_023513403.1/1-220 ------------------GAD-DVRVM-------TRK-SI--DDP--------G-R----
XP_022944761.1/1-220 ------------------GAD-DVRVM-------TRK-SI--DDP--------G-R----
XP_023513401.1/1-220 ------------------GAD-DVRVM-------TRK-SI--DDP--------G-R----
XP_022944759.1/1-220 ------------------GAD-DVRVM-------TRK-SI--DDP--------G-R----
XP_022986270.1/1-220 ------------------GAD-DVRVM-------TRK-SI--DDP--------G-R----
XP_022986268.1/1-220 ------------------GAD-DVRVM-------TRK-SI--DDP--------G-R----
XP_022136006.1/1-224 ------------------GAD-DVRVM-------TRK-SV--DDP--------G-R----
XP_008455423.1/1-229 ------------------GAD-DVRVM-------TRK-SV--DDP--------G-R----
XP_011658704.1/1-229 ------------------GAD-DVRVM-------TRK-SV--DDP--------G-R----
XP_004144488.1/1-229 ------------------GAD-DVRVM-------TRK-SV--DDP--------G-R----
XP_022968990.1/1-229 ------------------GAD-DVRVM-------TRK-SV--DDP--------G-R----
XP_023554403.1/1-229 ------------------GAD-DVRVM-------TRK-SV--DDP--------G-R----
XP_022952948.1/1-229 ------------------GAD-DVRVM-------TRK-SI--DDP--------G-R----
XP_019447751.1/1-228 ------------------GAD-DVRVM-------TRK-SV--DDP--------G-R----
XP_019447749.1/1-228 ------------------GAD-DVRVM-------TRK-SV--DDP--------G-R----
XP_004515074.1/1-228 ------------------GAD-DVRVM-------TRK-SV--DDP--------G-R----
XP_012463792.1/1-235 ------------------GAD-DVRVM-------TRK-SV--DDP--------G-R----
XP_012463790.1/1-235 ------------------GAD-DVRVM-------TRK-SV--DDP--------G-R----
XP_016678783.1/1-235 ------------------GAD-DVRVM-------TRK-SV--DDP--------G-R----
XP_016678782.1/1-235 ------------------GAD-DVRVM-------TRK-SV--DDP--------G-R----
XP_017631648.1/1-235 ------------------GAD-DVRVM-------TRK-SV--DDP--------G-R----
XP_016732503.1/1-235 ------------------GAD-DVRVM-------TRK-SV--DDP--------G-R----
XP_017631647.1/1-235 ------------------GAD-DVRVM-------TRK-SV--DDP--------G-R----
XP_012438430.1/1-229 ------------------GAD-DVRVM-------TRK-SV--DDP--------G-R----
XP_012438427.1/1-229 ------------------GAD-DVRVM-------TRK-SV--DDP--------G-R----
XP_012438429.1/1-229 ------------------GAD-DVRVM-------TRK-SV--DDP--------G-R----
XP_017616162.1/1-229 ------------------GAD-DVRVM-------TRK-SV--DDP--------G-R----
XP_017616160.1/1-229 ------------------GAD-DVRVM-------TRK-SV--DDP--------G-R----
XP_017616161.1/1-229 ------------------GAD-DVRVM-------TRK-SV--DDP--------G-R----
XP_016726347.1/1-229 ------------------GAD-DVRVM-------TRK-RV--DDP--------G-R----
XP_016726344.1/1-229 ------------------GAD-DVRVM-------TRK-RV--DDP--------G-R----
XP_016726345.1/1-229 ------------------GAD-DVRVM-------TRK-RV--DDP--------G-R----
XP_019448933.1/1-227 ------------------GAD-DVRVM-------TRK-SV--DDP--------G-R----
XP_004487400.1/1-228 ------------------GAD-DVRVM-------TRK-SV--DDP--------G-R----
XP_003596999.2/1-227 ------------------GAD-DVRVM-------TRK-SV--DDP--------G-R----
XP_024631890.1/1-227 ------------------GAD-DVRVM-------TRK-SV--DDP--------G-R----
XP_024631891.1/1-227 ------------------GAD-DVRVM-------TRK-SV--DDP--------G-R----
XP_004287935.1/1-224 ------------------GAD-DVRVM-------TRK-SV--DDP--------G-R----
XP_018808969.1/1-228 ------------------GAD-DVRVM-------TRK-SI--DDP--------G-R----
XP_018808967.1/1-228 ------------------GAD-DVRVM-------TRK-SI--DDP--------G-R----
XP_018817598.1/1-227 ------------------GAD-DVRVM-------TRK-SI--DDP--------G-R----
XP_018817597.1/1-227 ------------------GAD-DVRVM-------TRK-SI--DDP--------G-R----
XP_018817594.1/1-227 ------------------GAD-DVRVM-------TRK-SI--DDP--------G-R----
XP_018817599.1/1-227 ------------------GAD-DVRVM-------TRK-SI--DDP--------G-R----
XP_023915056.1/1-227 ------------------GAD-DVRVM-------TRK-SI--DDP--------G-R----
XP_023915054.1/1-227 ------------------GAD-DVRVM-------TRK-SI--DDP--------G-R----
XP_023915053.1/1-227 ------------------GAD-DVRVM-------TRK-SI--DDP--------G-R----
XP_021685023.1/1-228 ------------------GAD-DVRVT-------TRK-SV--DDP--------G-R----
XP_021684657.1/1-228 ------------------GAD-DVRVT-------TRK-SV--DDP--------G-R----
XP_021685089.1/1-228 ------------------GAD-DVRVT-------TRK-SV--DDP--------G-R----
XP_015383009.1/1-229 ------------------GAD-DVRVM-------TRK-SV--DDP--------G-R----
XP_015383007.1/1-229 ------------------GAD-DVRVM-------TRK-SV--DDP--------G-R----
XP_020539441.1/1-235 ------------------GAD-DVRVM-------TRK-SV--DDP--------G-R----
XP_020539439.1/1-235 ------------------GAD-DVRVM-------TRK-SV--DDP--------G-R----
XP_015577881.1/1-233 ------------------GAD-DVRVM-------TRK-SV--DDP--------G-R----
XP_015577879.1/1-233 ------------------GAD-DVRVM-------TRK-SV--DDP--------G-R----
XP_015577877.1/1-233 ------------------GAD-DVRVM-------TRK-SV--DDP--------G-R----
XP_021630854.1/1-231 ------------------GAD-DVRVM-------TRK-SV--DDP--------G-R----
XP_021630849.1/1-231 ------------------GAD-DVRVM-------TRK-SV--DDP--------G-R----
XP_021652390.1/1-229 ------------------GAD-DVRVM-------TRK-SV--DDP--------G-R----
XP_021652388.1/1-229 ------------------GAD-DVRVM-------TRK-SV--DDP--------G-R----
XP_021652389.1/1-234 ------------------GAD-DVRVM-------TRK-SV--DDP--------G-R----
XP_021652386.1/1-234 ------------------GAD-DVRVM-------TRK-SV--DDP--------G-R----
XP_006592380.1/1-228 ------------------GAD-DVRVM-------TRK-SV--DDP--------G-R----
XP_003539872.2/1-228 ------------------GAD-DVRVM-------TRK-SV--DDP--------G-R----
XP_025984806.1/1-230 ------------------GAD-DVRVM-------TRK-SV--DDP--------G-R----
XP_014632311.1/1-230 ------------------GAD-DVRVM-------TRK-SV--DDP--------G-R----
XP_024450362.1/1-227 ------------------GAD-DVRVM-------TRK-SV--DDP--------G-R----
XP_002302880.2/1-227 ------------------GAD-DVRVM-------TRK-SV--DDP--------G-R----
XP_024450361.1/1-227 ------------------GAD-DVRVM-------TRK-SV--DDP--------G-R----
XP_024450360.1/1-227 ------------------GAD-DVRVM-------TRK-SV--DDP--------G-R----
XP_011011493.1/1-227 ------------------GAD-DVRVM-------TRK-SV--DDP--------G-R----
XP_011011491.1/1-227 ------------------GAD-DVRVM-------TRK-SV--DDP--------G-R----
XP_011011492.1/1-227 ------------------GAD-DVRVM-------TRK-SV--DDP--------G-R----
XP_011011489.1/1-227 ------------------GAD-DVRVM-------TRK-SV--DDP--------G-R----
XP_011011488.1/1-227 ------------------GAD-DVRVM-------TRK-SV--DDP--------G-R----
XP_011005116.1/1-225 ------------------GAD-DVRVM-------TRK-SV--DDP--------G-R----
XP_011005113.1/1-225 ------------------GAD-DVRVM-------TRK-SV--DDP--------G-R----
XP_011005112.1/1-225 ------------------GAD-DVRVM-------TRK-SV--DDP--------G-R----
XP_024440391.1/1-225 ------------------GAD-DVRVM-------TRK-SV--DDP--------G-R----
XP_002320458.2/1-225 ------------------GAD-DVRVM-------TRK-SV--DDP--------G-R----
XP_024440389.1/1-225 ------------------GAD-DVRVM-------TRK-SV--DDP--------G-R----
XP_019453767.1/1-227 ------------------GAD-DVRVM-------TRK-SV--DDP--------G-R----
XP_012478857.1/1-227 ------------------GAD-DVRVM-------TRK-SV--DDP--------G-R----
XP_012478855.1/1-227 ------------------GAD-DVRVM-------TRK-SV--DDP--------G-R----
XP_012478856.1/1-227 ------------------GAD-DVRVM-------TRK-SV--DDP--------G-R----
XP_012478851.1/1-227 ------------------GAD-DVRVM-------TRK-SV--DDP--------G-R----
XP_016724999.1/1-227 ------------------GAD-DVRVM-------TRK-SV--DDP--------G-R----
XP_017638204.1/1-227 ------------------GAD-DVRVM-------TRK-SV--DDP--------G-R----
XP_017638203.1/1-227 ------------------GAD-DVRVM-------TRK-SV--DDP--------G-R----
XP_016667113.1/1-227 ------------------GAD-DVRVM-------TRK-RV--DDP--------G-R----
XP_016667112.1/1-227 ------------------GAD-DVRVM-------TRK-RV--DDP--------G-R----
XP_015881131.2/1-227 ------------------GAE-DVRVM-------TRK-SV--DDP--------G-R----
XP_008348131.1/1-222 ------------------GAD-DVRVM-------TRK-SV--DDP--------G-R----
XP_008348133.1/1-220 ------------------GAD-DVRVM-------TRK-SV--DDP--------G-R----
XP_018502305.1/1-220 ------------------GAD-DVRVM-------TRK-SV--DDP--------G-R----
XP_009342243.1/1-220 ------------------GAD-DVRVM-------TRK-SV--DDP--------G-R----
XP_017179321.1/1-222 ------------------GAD-DVRVM-------TRK-SV--DDP--------G-R----
XP_017179320.1/1-222 ------------------GAD-DVRVM-------TRK-SV--DDP--------G-R----
XP_008341986.1/1-222 ------------------GAD-DVRVM-------TRK-SV--DDP--------G-R----
XP_017179319.1/1-220 ------------------GAD-DVRVM-------TRK-SV--DDP--------G-R----
XP_024185198.1/1-222 ------------------GAD-DVRVM-------TRK-SV--DDP--------G-R----
XP_011463209.1/1-222 ------------------GAD-DVRVM-------TRK-SV--DDP--------G-R----
XP_020416501.1/1-219 ------------------GAD-DVRVM-------TRK-SV--DDP--------G-R----
XP_021822853.1/1-219 ------------------GAD-DVRVM-------TRK-SV--DDP--------G-R----
XP_008230079.1/1-219 ------------------GAD-DVRAM-------TRK-SA--DDP--------G-R----
XP_020870786.1/1-221 ------------------GAE-DVRVM-------TRK-SV--DDP--------G-R----
NP_001117231.1/1-221 ------------------GAE-DVRVM-------TRK-SV--DDP--------G-R----
XP_018817596.1/1-225 ------------------GAD-DVRVM-------TRK-SI--DDP--------G-R----
XP_019079350.1/1-224 ------------------GAD-DVRVM-------TRK-SV--DDP--------G-R----
XP_022733639.1/1-224 ------------------GAD-DVRVM-------TRK-SV--DDP--------G-R----
XP_021292768.1/1-224 ------------------GAD-DVRVM-------TRK-SV--DDP--------G-R----
XP_021292766.1/1-224 ------------------GAD-DVRVM-------TRK-SV--DDP--------G-R----
XP_017971480.1/1-224 ------------------GAD-DVRVM-------TRK-SV--DDP--------G-R----
XP_018721484.1/1-222 ------------------GAD-DVRVM-------TRK-SV--DDP--------G-R----
XP_018721483.1/1-222 ------------------GAD-DVRVM-------TRK-SV--DDP--------G-R----
XP_018721482.1/1-222 ------------------GAD-DVRVM-------TRK-SV--DDP--------G-R----
XP_025687365.1/1-237 ------------------GVD-DVRVM-------TRK-SV--DDP--------G-R----
XP_006851770.2/1-243 ------------------NASDDVRVM-------TRK-SI--DDP--------G-E----
XP_003623826.1/1-230 ------------------NVGEDVRVM-------TRK-SV--DDP--------G-E----
XP_004492678.1/1-230 ------------------NVGVDVRVM-------TRK-SI--DDP--------G-E----
XP_004492677.1/1-230 ------------------NVGVDVRVM-------TRK-SI--DDP--------G-E----
XP_012847897.1/1-236 ------------------NVDDDVQVM-------TRK-SV--DDP--------G-E----
XP_012843673.1/1-229 ------------------NVDDDVQVM-------TRK-SV--DDP--------G-E----
XP_021279608.1/1-239 ------------------NVDEDVRVM-------TRK-SV--DDP--------G-E----
XP_025641241.1/1-241 -----------------NTVDEDVRVM-------TRK-SV--DDP--------G-E----
XP_025641246.1/1-241 -----------------NTVDEDVRVM-------TRK-SV--DDP--------G-E----
XP_020991942.1/1-241 -----------------NTVDEDVRVM-------TRK-SV--DDP--------G-E----
XP_025632115.1/1-241 -----------------NTVDEDVRVM-------TRK-SV--DDP--------G-E----
XP_025632116.1/1-241 -----------------NTVDEDVRVM-------TRK-SV--DDP--------G-E----
XP_017442222.1/1-246 -----------------N-VDEDVRVM-------TRK-SV--DDPXXXXXXXXG-E----
XP_017442225.1/1-246 -----------------N-VDEDVRVM-------TRK-SV--DDPXXXXXXXXG-E----
XP_006576359.1/1-244 -----------------N-VDEDVRVM-------TRK-SV--DDP--------G-E----
XP_003521284.1/1-244 -----------------N-VDEDVRVM-------TRK-SV--DDP--------G-E----
XP_024174849.1/1-237 ------------------NVDEDVRYM-------TRE-SM--DDP--------G-E----
XP_024174854.1/1-237 ------------------NVDEDVRYM-------TRE-SM--DDP--------G-E----
XP_004306832.1/1-241 ------------------NVDEDVRYM-------TRE-SM--DDP--------G-E----
XP_011469062.1/1-241 ------------------NVDEDVRYM-------TRE-SM--DDP--------G-E----
XP_009340632.2/1-243 ------------------NVDEDVRVM-------TRE-SL--DDP--------G-E----
XP_008385703.1/1-243 ------------------NVDEDVRVM-------TRE-SL--DDP--------G-E----
XP_008385704.1/1-243 ------------------NVDEDVRVM-------TRE-SL--DDP--------G-E----
XP_008232741.1/1-243 ------------------NVDEDVRVM-------TRE-SL--DDP--------G-E----
XP_008232742.1/1-243 ------------------NVDEDVRVM-------TRE-SL--DDP--------G-E----
XP_007220256.1/1-243 ------------------NVDEDVRVM-------TRE-SL--DDP--------G-E----
XP_020413144.1/1-243 ------------------NVDEDVRVM-------TRE-SL--DDP--------G-E----
XP_021817103.1/1-243 ------------------NVDEDVRVM-------TRE-SL--DDP--------G-E----
XP_021817105.1/1-243 ------------------NVDEDVRVM-------TRE-SL--DDP--------G-E----
XP_022872559.1/1-230 ------------------NVGEDVRIM-------TRK-SV--DDP--------G-E----
XP_016575742.1/1-238 ----------------------DVRVM-------PRK-TV--DDP--------G-E----
XP_017608938.1/1-232 ------------------NGEEDVRVM-------TRK-SV--DNP--------G-E----
XP_017608947.1/1-232 ------------------NGEEDVRVM-------TRK-SV--DNP--------G-E----
XP_010255501.1/1-245 ------------------NVDEDVRVM-------TRK-SV--DDP--------G-E----
XP_011023407.1/1-238 ------------------NVDEDVRVM-------TRK-RV--NDP--------G-E----
XP_011023404.1/1-238 ------------------NVDEDVRVM-------TRK-RV--NDP--------G-E----
XP_011023405.1/1-238 ------------------NVDEDVRVM-------TRK-RV--NDP--------G-E----
XP_019152252.1/1-243 ------------------NVDEDVRVM-------TRK-SV--DDP--------G-E----
XP_022870029.1/1-235 ------------------NVDEDVRVM-------TRK-SA--DDP--------G-E----
XP_017699285.1/1-225 ------------------NIGEDVRVM-------TRQ-SV--DDP--------G-E----
XP_008795402.1/1-225 ------------------NIGEDVRVM-------TRQ-SV--DDP--------G-E----
XP_010933869.1/1-224 ------------------NIGEDVRVM-------TRQ-SV--DDP--------G-E----
XP_008799528.1/1-222 ------------------SIGEDVRVM-------TRQ-SV--DDP--------G-E----
XP_010919252.1/1-223 ------------------SIGEDVRVM-------TRQ-SV--DDP--------G-E----
XP_010919250.1/1-223 ------------------SIGEDVRVM-------TRQ-SV--DDP--------G-E----
XP_007139955.1/1-229 ------------------NVGEDVRVM-------TRK-SV--DDP--------G-E----
XP_017418814.1/1-230 ------------------NVGEDVRVM-------TRK-SV--DDP--------G-E----
XP_014490274.1/1-230 ------------------NVGEDVRVM-------TRK-SV--DDP--------G-E----
XP_003534596.1/1-229 ------------------NVGEDVRVM-------TRK-SV--DDP--------G-E----
XP_006587871.1/1-229 ------------------NVGEDVRVM-------TRK-SV--DDP--------G-E----
XP_003552359.1/1-228 ------------------NVGEDVRVM-------TRK-SV--DDP--------G-E----
XP_015961616.1/1-228 ------------------NFGEDVRVM-------TRK-SL--DDP--------G-E----
XP_015961617.1/1-228 ------------------NFGEDVRVM-------TRK-SL--DDP--------G-E----
XP_025696060.1/1-228 ------------------NFGEDVRVM-------TRK-SL--DDP--------G-E----
XP_025696061.1/1-228 ------------------NFGEDVRVM-------TRK-SL--DDP--------G-E----
XP_016198931.1/1-228 ------------------NFGEDVRVM-------TRK-SL--DDP--------G-E----
XP_016198932.1/1-228 ------------------NFGEDVRVM-------TRK-SL--DDP--------G-E----
XP_025649261.1/1-228 ------------------NFGEDVRVM-------TRK-SL--DDP--------G-E----
XP_025649262.1/1-228 ------------------NFGEDVRVM-------TRK-SL--DDP--------G-E----
XP_021849370.1/1-228 ------------------NVDEDVRVM-------TRK-SI--DDP--------G-E----
XP_021767325.1/1-228 ------------------NVDEDVRVM-------TRK-SI--DDP--------G-E----
XP_021748881.1/1-228 ------------------NVDEDVRVM-------TRK-SI--DDP--------G-E----
XP_019461451.1/1-231 ------------------NVDEDVRVM-------TRK-SV--DDP--------G-E----
XP_012574211.1/1-233 ------------------NVDEDVRVM-------TRK-XX--XDP--------G-E----
XP_004510857.1/1-233 ------------------NVDEDVRVM-------TRK-XX--XDP--------G-E----
XP_020237185.1/1-234 ------------------NVDEDVRVM-------TRK-SV--XDP--------G-E----
XP_020237261.1/1-234 ------------------NVDEDVRVM-------TRK-SV--XDP--------G-E----
XP_019456357.1/1-238 ------------------NVDEDVRVM-------TRK-SV--DDP--------G-E----
XP_013444922.1/1-235 ------------------NVDEDVRVM-------TRK-SV--DDP--------G-E----
XP_003528880.1/1-238 ------------------NVDEDVRVM-------TRK-SV--DDP--------G-E----
XP_006583340.1/1-238 ------------------NVDEDVRVM-------TRK-SV--DDP--------G-E----
XP_014516480.1/1-238 ------------------NVDEDVRVM-------TRK-SV--DDP--------G-E----
XP_014516483.1/1-238 ------------------NVDEDVRVM-------TRK-SV--DDP--------G-E----
XP_007134961.1/1-238 ------------------NVDEDVRVM-------TRK-SV--DDP--------G-E----
XP_010671720.1/1-229 ------------------NVDEDVQVM-------TRK-SI--DDP--------G-E----
XP_012482732.1/1-232 ------------------NVDEDVRVM-------TRK-SV--DNP--------G-E----
XP_012482742.1/1-232 ------------------NVDEDVRVM-------TRK-SV--DNP--------G-E----
XP_016745638.1/1-232 ------------------NVDEDVRVM-------TRK-SV--DNP--------G-E----
XP_016745639.1/1-232 ------------------NVDEDVRVM-------TRK-SV--DNP--------G-E----
XP_010278578.1/1-242 ------------------NVDEDVRVM-------TRK-SV--DDP--------G-E----
XP_010278577.1/1-242 ------------------NVDEDVRVM-------TRK-SV--DDP--------G-E----
XP_006445143.1/1-238 ------------------NVDEDVRVM-------TRK-SV--DDP--------G-E----
XP_006445141.1/1-238 ------------------NVDEDVRVM-------TRK-SV--DDP--------G-E----
XP_022770361.1/1-238 ------------------NVGEDVRVM-------TRK-SV--DDP--------G-E----
XP_022757219.1/1-237 ------------------NVDEDVRVM-------TRK-SI--DDP--------G-E----
XP_022757220.1/1-237 ------------------NVDEDVRVM-------TRK-SI--DDP--------G-E----
NP_001314420.1/1-237 ------------------NVDEDVRVM-------TRK-SI--DDP--------G-E----
XP_016735217.1/1-237 ------------------NVDEDVRVM-------TRK-SI--DDP--------G-E----
XP_012437660.1/1-237 ------------------NVDEDVRVM-------TRK-SI--DDP--------G-E----
XP_012437661.1/1-237 ------------------NVDEDVRVM-------TRK-SI--DDP--------G-E----
XP_017615505.1/1-237 ------------------NVDEDVRVM-------TRK-SI--DDP--------G-E----
XP_017615506.1/1-237 ------------------NVDEDVRVM-------TRK-SI--DDP--------G-E----
XP_016723220.1/1-237 ------------------NVDEDVRVM-------TRK-SI--DDP--------G-E----
XP_016723229.1/1-237 ------------------NVDEDVRVM-------TRK-SI--DDP--------G-E----
XP_021904309.1/1-244 ------------------NVDEDVRVM-------TRK-SV--DDP--------G-E----
XP_021904311.1/1-244 ------------------NVDEDVRVM-------TRK-SV--DDP--------G-E----
XP_007051912.2/1-241 ------------------DVDEDVRVM-------TRK-SV--DDP--------G-E----
XP_007051913.2/1-241 ------------------DVDEDVRVM-------TRK-SV--DDP--------G-E----
XP_012489878.1/1-239 ------------------NVDEDVRVM-------TRK-SV--DDP--------G-E----
XP_016748231.1/1-239 ------------------NVDEDVRVM-------TRK-SV--DDP--------G-E----
XP_017631289.1/1-239 ------------------NVDEDVRVM-------TRK-SV--DDP--------G-E----
XP_023006686.1/1-236 ------------------SVDEDVRVM-------TRK-SV--DDP--------G-E----
XP_022958954.1/1-236 ------------------SVDEDVRVM-------TRK-SV--DDP--------G-E----
XP_023548045.1/1-236 ------------------SVDEDVRVM-------TRK-SV--DDP--------G-E----
XP_008460172.1/1-241 ------------------SVDEDVRVM-------TRK-SV--DDP--------G-E----
XP_004144982.1/1-241 ------------------SVDEDVRVM-------TRK-SV--DDP--------G-E----
XP_023514722.1/1-242 ------------------SVDEDVRVM-------TRK-SV--DDP--------G-E----
XP_022999814.1/1-242 ------------------SVDEDVRVM-------TRK-SV--DDP--------G-E----
XP_022964504.1/1-242 ------------------SVDEDVRVM-------TRK-SV--DDP--------G-E----
XP_010053664.1/1-245 ------------------NVDEDVRVM-------TRK-SV--DDP--------G-E----
XP_019189515.1/1-235 ------------------NVDDDVRVM-------TRK-SV--DDP--------G-E----
XP_023730775.1/1-234 ------------------NVDKDVRVM-------TRQ-SV--DDP--------G-E----
XP_023730774.1/1-234 ------------------NVDKDVRVM-------TRQ-SV--DDP--------G-E----
XP_022020543.1/1-230 ------------------NVDEDVRVM-------TRQ-SV--DDP--------G-E----
XP_024962572.1/1-236 ------------------NVDEDVRVM-------TRQ-SV--DDP--------G-E----
XP_022017696.1/1-236 ------------------NVDEDVRVM-------TRQ-SV--DDP--------G-E----
XP_024018220.1/1-245 ------------------NVDEDVRVM-------TRK-SV--DDP--------G-E----
XP_011093166.1/1-234 ------------------NVDEDVRVM-------TRK-SV--DDP--------G-E----
XP_011093167.1/1-234 ------------------NVDEDVRVM-------TRK-SV--DDP--------G-E----
XP_006339457.1/1-238 ------------------NVDEDVRVM-------TRK-SV--DDP--------G-E----
XP_015059298.1/1-238 ------------------NVDEDVRVM-------TRK-SV--DDP--------G-E----
NP_001234657.2/1-238 ------------------NVDEDVRVM-------TRK-SV--DDP--------G-E----
XP_009602857.1/1-241 ------------------NVDEDVRVM-------TRK-SV--DDP--------G-E----
XP_009602856.1/1-241 ------------------NVDEDVRVM-------TRK-SV--DDP--------G-E----
XP_009782444.1/1-245 ------------------NVDEDVRVM-------TRK-SV--DDP--------G-E----
XP_009782443.1/1-245 ------------------NVDEDVRVM-------TRK-SV--DDP--------G-E----
XP_019259406.1/1-245 ------------------NVDEDVRVM-------TRK-SV--DDP--------G-E----
XP_015867375.1/1-242 ------------------NVDEDVRVM-------TRK-SV--DDP--------G-E----
XP_015867374.1/1-242 ------------------NVDEDVRVM-------TRK-SV--DDP--------G-E----
XP_015888672.1/1-242 ------------------NVDEDVRVM-------TRK-XX--XDP--------G-E----
XP_018810322.1/1-233 ------------------NVDEDVRVM-------TRK-SV--DDP--------G-E----
XP_018859074.1/1-233 ------------------NVDEDVRVM-------TRK-SV--DDP--------G-E----
XP_017231243.1/1-235 ------------------NVDEDVRVM-------TRN-SV--DDP--------G-E----
XP_017231244.1/1-235 ------------------NVDEDVRVM-------TRN-SV--DDP--------G-E----
XP_017231245.1/1-235 ------------------NVDEDVRVM-------TRN-SV--DDP--------G-E----
XP_017236322.1/1-235 ------------------NVDEDVRVM-------TRK-SV--DDP--------G-E----
XP_002301331.2/1-238 ------------------NVDEDVRVM-------TRK-SV--DDP--------G-E----
XP_024451706.1/1-238 ------------------NVDEDVRVM-------TRK-SV--DDP--------G-E----
XP_002320755.1/1-241 ------------------NVDEDVRVM-------TRK-SV--DDP--------G-E----
XP_011035097.1/1-241 ------------------NVDEDVRVM-------TRK-SV--DDP--------G-E----
XP_018813891.1/1-238 ------------------NVDEDVRVM-------TRK-SV--DDP--------G-E----
XP_012083470.1/1-239 ------------------NVDEDVRVM-------TRK-SV--DDP--------G-E----
XP_021651338.1/1-240 ------------------NVDEDVRVM-------TRK-SV--DDP--------G-E----
XP_021613538.1/1-241 ------------------NVDEDVRVM-------TRK-SV--DDP--------G-E----
XP_021686862.1/1-241 ------------------NVDEDVRVM-------TRK-SV--DDP--------G-E----
XP_021686863.1/1-241 ------------------NVDEDVRVM-------TRK-SV--DDP--------G-E----
XP_021612685.1/1-241 ------------------NVDEDVRVM-------TRK-SV--DDP--------G-E----
XP_023879166.1/1-242 ------------------NVDEDVRVM-------TRQ-SV--DDP--------G-E----
XP_002272264.2/1-234 ------------------NVDEDVRVM-------TRK-SV--DDP--------G-E----
XP_010661562.1/1-234 ------------------NVDEDVRVM-------TRK-SV--DDP--------G-E----
XP_002511801.1/1-237 ------------------NVDEDVRVM-------TRK-SV--DDP--------G-E----
XP_015584500.1/1-237 ------------------NVDEDVRVM-------TRK-SV--DDP--------G-E----
XP_022141257.1/1-238 ------------------NISEDVKVM-------ARK-SI--DDP--------G-E----
XP_023885612.1/1-244 ------------------NVGENVRVM-------TRR-NL--DDP--------G-E----
XP_023885611.1/1-244 ------------------NVGENVRVM-------TRR-NL--DDP--------G-E----
XP_018830842.1/1-239 ------------------NVGEDVRVM-------TRK-NL--DDP--------G-E----
XP_018830843.1/1-239 ------------------NVGEDVRVM-------TRK-NL--DDP--------G-E----
XP_018830840.1/1-239 ------------------NVGEDVRVM-------TRK-NL--DDP--------G-E----
XP_018830841.1/1-239 ------------------NVGEDVRVM-------TRK-NL--DDP--------G-E----
XP_012090293.1/1-240 ------------------NVGENVRIL-------TRK-NV--NDP--------G-E----
XP_012090292.1/1-240 ------------------NVGENVRIL-------TRK-NV--NDP--------G-E----
XP_021614581.1/1-239 ------------------NVGEDMRIL-------TRK-NI--NDP--------G-E----
XP_021614580.1/1-239 ------------------NVGEDMRIL-------TRK-NI--NDP--------G-E----
XP_015581247.1/1-240 ------------------NIGEDVRIL-------TRK-NI--NDP--------G-E----
XP_002510822.1/1-240 ------------------NIGEDVRIL-------TRK-NI--NDP--------G-E----
XP_021648646.1/1-240 ------------------NVGEDMRIL-------TRK-NI--NDP--------G-E----
XP_021634593.1/1-234 ------------------NVGEDVRIL-------TRK-NI--NDP--------G-E----
XP_021634592.1/1-234 ------------------NVGEDVRIL-------TRK-NI--NDP--------G-E----
XP_006490348.1/1-238 ------------------NVGEDVRVL-------TRK-NV--NDP--------G-E----
XP_006490346.1/1-238 ------------------NVGEDVRVL-------TRK-NV--NDP--------G-E----
XP_024958654.1/1-238 ------------------NVGEDVRVL-------TRK-NV--NDP--------G-E----
XP_006490345.1/1-238 ------------------NVGEDVRVL-------TRK-NV--NDP--------G-E----
XP_006421879.2/1-238 ------------------NVGEDVRVL-------TRK-NV--NDP--------G-E----
XP_021892212.1/1-221 ------------------NVSEDVKVM-------TRK-NI--NEA--------G-E----
XP_021892211.1/1-221 ------------------NVSEDVKVM-------TRK-NI--NEA--------G-E----
XP_021892210.1/1-221 ------------------NVSEDVKVM-------TRK-NI--NEA--------G-E----
XP_022765148.1/1-244 ------------------NVGEDVRVM-------TRK-NI--NDP--------G-E----
XP_022765158.1/1-244 ------------------NVGEDVRVM-------TRK-NI--NDP--------G-E----
XP_022765120.1/1-244 ------------------NVGEDVRVM-------TRK-NI--NDP--------G-E----
XP_022765114.1/1-244 ------------------NVGEDVRVM-------TRK-NI--NDP--------G-E----
XP_022736693.1/1-237 ------------------NVGEDVRVM-------TRK-NI--NDP--------G-E----
XP_022736692.1/1-237 ------------------NVGEDVRVM-------TRK-NI--NDP--------G-E----
XP_022736689.1/1-237 ------------------NVGEDVRVM-------TRK-NI--NDP--------G-E----
XP_022734775.1/1-244 ------------------NVGEDVRVM-------TRK-NI--NDP--------G-E----
XP_016743353.1/1-244 ------------------NVGEDVRVM-------TRK-NI--NDP--------G-E----
XP_012474605.1/1-244 ------------------NVGEDVRVM-------TRK-NI--NDP--------G-E----
XP_012474606.1/1-244 ------------------NVGEDVRVM-------TRK-NI--NDP--------G-E----
XP_016743352.1/1-244 ------------------NVGEDVRVM-------TRK-NI--NDP--------G-E----
XP_016743351.1/1-244 ------------------NVGEDVRVM-------TRK-NI--NDP--------G-E----
XP_012474604.1/1-244 ------------------NVGEDVRVM-------TRK-NI--NDP--------G-E----
XP_012474603.1/1-244 ------------------NVGEDVRVM-------TRK-NI--NDP--------G-E----
XP_017646297.1/1-244 ------------------NVGEDVRVM-------TRK-NI--NDP--------G-E----
XP_016736315.1/1-244 ------------------NVGEDVRVM-------TRK-NI--NDP--------G-E----
XP_017646296.1/1-244 ------------------NVGEDVRVM-------TRK-NI--NDP--------G-E----
XP_017646298.1/1-244 ------------------NVGEDVRVM-------TRK-NI--NDP--------G-E----
XP_016736306.1/1-244 ------------------NVGEDVRVM-------TRK-NI--NDP--------G-E----
XP_017646295.1/1-244 ------------------NVGEDVRVM-------TRK-NI--NDP--------G-E----
XP_017972896.1/1-244 ------------------NVGEDVRVM-------TRK-NI--DDP--------G-E----
XP_017972895.1/1-244 ------------------NVGEDVRVM-------TRK-NI--DDP--------G-E----
XP_017972893.1/1-244 ------------------NVGEDVRVM-------TRK-NI--DDP--------G-E----
XP_021274823.1/1-244 ------------------NVGEDVRVM-------TRK-NI--DDP--------G-E----
XP_024536343.1/1-231 -------------------GEDDVRVM-------TRK-SI--DNP--------G-E----
XP_006366174.1/1-244 -----------------NNVEDYVRVL-------TRK-SV--DDP--------G-E----
XP_020219711.1/1-212 -----------------GNVGEDVRVM-------TRK-----------------------
XP_022897056.1/1-222 -----------------GNADEDIQIM-------TRK-SV--DDP--------G-E----
NP_001281015.1/1-243 -----------------GNVDEDVRVM-------TRE-SL--DDP--------G-E----
XP_008383260.1/1-243 -----------------GNVDEDVRVM-------TRE-SL--DDP--------G-E----
XP_022896229.1/1-235 -----------------GNVDEDVRVM-------TRK-SV--DDP--------G-E----
XP_022896230.1/1-235 -----------------GNVDEDVRVM-------TRK-SV--DDP--------G-E----
XP_025816451.1/1-225 -----------------GSIGEDVRVM-------ARK-SV--DEP--------G-E----
XP_004968001.1/1-225 -----------------GSIGEDVRVM-------ARK-SV--DEP--------G-E----
XP_004968002.1/1-225 -----------------GSIGEDVRVM-------ARK-SV--DEP--------G-E----
XP_002457484.1/1-226 -----------------GSIGEDVRVM-------ARK-SV--DEP--------G-E----
XP_009400446.1/1-224 -----------------VDIGEDVRVM-------TRQ-SV--ANP--------G-E----
XP_018676535.1/1-225 -----------------INIGEDVRVM-------TRQ-SV--ADP--------G-V----
XP_009401771.1/1-232 -----------------INIGEDVRVM-------TRQ-SV--ADP--------G-E----
XP_009384534.1/1-228 -----------------VNIGEDVRVM-------TRQ-SV--ADP--------G-E----
XP_020672447.1/1-217 ------------------SIGEDVRVM-------TRQ-SM--DDP--------G-E----
XP_020582116.1/1-218 ------------------SISEEVRVM-------TRQ-SM--DDP--------G-E----
XP_020675647.1/1-231 ------------------SIGEDVRMM-------TRQ-SM--EDP--------G-E----
XP_020675648.1/1-231 ------------------SIGEDVRMM-------TRQ-SM--EDP--------G-E----
XP_020585632.1/1-228 -----------------SSIGEDVRVM-------TRQ-SM--EDP--------G-E----
XP_016176402.1/1-228 -------------------LDDDMRVM-------TRK-NV--DDP--------G-E----
XP_016176403.1/1-228 -------------------LDDDMRVM-------TRK-NV--DDP--------G-E----
XP_016176400.1/1-228 -------------------LDDDMRVM-------TRK-NV--DDP--------G-E----
XP_016176401.1/1-228 -------------------LDDDMRVM-------TRK-NV--DDP--------G-E----
XP_010523042.1/1-245 -------------------VGPDVRVM-------TRK-SV--DDP--------G-E----
XP_010523041.1/1-240 -------------------VGPDVRVM-------TRK-SV--DDP--------G-E----
XP_020878511.1/1-224 -------------------VDPDVRVM-------TRK-SV--DDP--------G-E----
NP_567183.2/1-228    -------------------VDPDVRVM-------TRK-SV--DDP--------G-E----
XP_006287095.1/1-228 -------------------VDPDVRVM-------TRK-SV--DDP--------G-E----
XP_010456250.1/1-228 -------------------VDPDVRVM-------TRK-SV--DDP--------G-E----
XP_010419627.1/1-228 -------------------VDPDVRVM-------TRK-SV--DDP--------G-E----
XP_010427345.1/1-228 -------------------VDPDVRVM-------TRK-SV--DDP--------G-E----
XP_009111469.1/1-229 -------------------VDPDVRIM-------TRK-SV--DDP--------S-E----
XP_018487039.1/1-229 -------------------VDPDVRIM-------TRK-SV--DDP--------S-E----
XP_018453129.1/1-229 -------------------VDPDVRIM-------TRK-SV--DDP--------S-E----
XP_013612299.1/1-229 -------------------VDPDVRIM-------TRK-SV--DDP--------S-E----
XP_006396268.1/1-225 -------------------VDPDVRVM-------TRK-SG--E-----------------
XP_018475317.1/1-221 -------------------VDPDVRVM-------TRK-SV--D-----------------
XP_013629750.1/1-221 -------------------VDPDVRVM-------TRK-SV--D-----------------
XP_009134533.1/1-221 -------------------VDPDVRVM-------TRK-SV--D-----------------
XP_020531377.1/1-244 -------------VGSMTAGGEEVRVM-------TRK-SI--DDP--------G-E----
XP_006857866.1/1-244 -------------VGSMTAGGEEVRVM-------TRK-SI--DDP--------G-E----
XP_018434263.1/1-219 -------------------IDEDVRIM-------TRK-NV--DDS--------G-E----
XP_018434260.1/1-219 -------------------IDEDVRIM-------TRK-NV--DDS--------G-E----
XP_018434262.1/1-219 -------------------IDEDVRIM-------TRK-NV--DDS--------G-E----
XP_013688926.1/1-219 -------------------IDEDVRIM-------TRK-NV--DDS--------G-E----
XP_013637558.1/1-219 -------------------IDEDVRIM-------TRK-NV--DDS--------G-E----
XP_013637556.1/1-219 -------------------IDEDVRIM-------TRK-NV--DDS--------G-E----
XP_013637557.1/1-219 -------------------IDEDVRIM-------TRK-NV--DDS--------G-E----
XP_009143168.1/1-219 -------------------IDEDVRIM-------TRK-NV--NDS--------G-E----
XP_009143167.1/1-219 -------------------IDEDVRIM-------TRK-NV--NDS--------G-E----
XP_009143166.1/1-219 -------------------IDEDVRIM-------TRK-NV--NDS--------G-E----
XP_013692686.1/1-219 -------------------IDEDVRIM-------TRK-NV--NDS--------G-E----
XP_022574820.1/1-219 -------------------IDEDVRIM-------TRK-NV--NDS--------G-E----
XP_013692684.1/1-219 -------------------IDEDVRIM-------TRK-NV--NDS--------G-E----
XP_013748127.1/1-219 -------------------IDEDVRIM-------TRK-NV--NDS--------G-E----
XP_013692685.1/1-219 -------------------IDEDVRIM-------TRK-NV--NDS--------G-E----
XP_013748124.1/1-219 -------------------IDEDVRIM-------TRK-NV--NDS--------G-E----
XP_013748126.1/1-219 -------------------IDEDVRIM-------TRK-NV--NDS--------G-E----
XP_022574821.1/1-219 -------------------IDEDVRIM-------TRK-NV--NDS--------G-E----
XP_006290613.1/1-229 -------------------VDEDVRIM-------TRK-SV--NNP--------G-E----
XP_013592301.1/1-230 -------------------VDEDVRIM-------TRK-SV--NDP--------G-E----
XP_018442581.1/1-230 -------------------VDEDVRIM-------TRK-SV--NDP--------G-E----
XP_013750213.1/1-232 -------------------VDEDVRIM-------TRK-SV--NDP--------G-E----
XP_013648430.1/1-232 -------------------VDEDVRIM-------TRK-SV--DDP--------G-E----
XP_009104292.1/1-232 -------------------VDEDVRIM-------TRK-SV--DDP--------G-E----
XP_019058514.1/1-223 -------------------IDEDVRIM-------TRK-SV--DDP--------G-E----
XP_010523728.1/1-220 -------------------IDEDVRIM-------TRK-SV--DNP--------G-E----
XP_024514927.1/1-242 -------------------GEEDIRVM-------TRK-SV--NNP--------G-E----
XP_002985242.1/1-242 -------------------GEEDIRVM-------TRK-SV--NNP--------G-E----
XP_024530500.1/1-242 -------------------GEEDIRVM-------TRK-SV--NNP--------G-E----
XP_024530499.1/1-242 -------------------GEEDIRVM-------TRK-SV--NNP--------G-E----
XP_024530501.1/1-242 -------------------GEEDIRVM-------TRK-SV--NNP--------G-E----
XP_010107411.1/1-242 ------------------TVGEDVRVM-------TRK-SM--DDP--------G-E----
XP_024028635.1/1-242 ------------------TVGEDVRVM-------TRK-SM--DDP--------G-E----
XP_015877999.1/1-244 ------------------NVGEDVKVM-------TRK-SV--DDP--------G-E----
XP_019427294.1/1-247 ------------------TLGDDMRVM-------TRK-NM--DDP--------G-E----
XP_019427292.1/1-247 ------------------TLGDDMRVM-------TRK-NM--DDP--------G-E----
XP_019452814.1/1-239 ------------------TLGDDMRVM-------TRK-NV--DDP--------G-E----
XP_019452815.1/1-239 ------------------TLGDDMRVM-------TRK-NV--DDP--------G-E----
XP_003592295.2/1-235 ------------------TLSDDMRVM-------TRK-NV--DDP--------G-E----
XP_024625328.1/1-235 ------------------TLSDDMRVM-------TRK-NV--DDP--------G-E----
XP_012570175.1/1-236 ------------------TLSDDMRVM-------TRK-NV--DDP--------G-E----
XP_012570176.1/1-236 ------------------TLSDDMRVM-------TRK-NV--DDP--------G-E----
XP_004496593.1/1-236 ------------------TLSDDMRVM-------TRK-NV--DDP--------G-E----
XP_025620154.1/1-234 ------------------TLDDDMRVM-------TRK-NV--DDP--------G-E----
XP_015941562.1/1-234 ------------------TLDDDMRVM-------TRK-NV--DDP--------G-E----
XP_025620153.1/1-234 ------------------TLDDDMRVM-------TRK-NV--DDP--------G-E----
XP_020239673.1/1-232 ------------------TLGDDMKVM-------TRK-NV--DDP--------G-E----
XP_020239648.1/1-232 ------------------TLGDDMKVM-------TRK-NV--DDP--------G-E----
XP_007143377.1/1-235 ------------------TLGDDMKVM-------TRK-NV--DDP--------G-E----
XP_007143378.1/1-235 ------------------TLGDDMKVM-------TRK-NV--DDP--------G-E----
XP_017415467.1/1-234 ------------------TLGDDMKVM-------TRK-NV--DDP--------G-E----
XP_017415466.1/1-234 ------------------TLGDDMKVM-------TRK-NV--DDP--------G-E----
XP_014514421.1/1-236 ------------------TLGDDMKVM-------TRK-NV--DDP--------G-E----
XP_014514419.1/1-236 ------------------TLGDDMKVM-------TRK-NV--DDP--------G-E----
XP_006606097.1/1-235 ------------------TLGDDMKVM-------TRK-NV--DDP--------G-E----
XP_006606096.1/1-235 ------------------TLGDDMKVM-------TRK-NV--DDP--------G-E----
XP_003536477.1/1-234 ------------------TLGDDMKVM-------TRK-NV--DDP--------G-E----
XP_006589557.1/1-234 ------------------TLGDDMKVM-------TRK-NV--DDP--------G-E----
XP_006402516.1/1-230 ------------------NVDEDVRIM-------TRK-SV--NDP--------G-E----
NP_191674.1/1-237    ------------------NVDEDVRIM-------TRK-SV--NNP--------G-E----
XP_002876596.1/1-239 ------------------NVDEDVRIM-------TRK-SV--NNP--------G-E----
XP_010413451.1/1-238 ------------------NVDEDVRIM-------TRK-SV--NIP--------G-E----
XP_010413452.1/1-238 ------------------NVDEDVRIM-------TRK-SV--NIP--------G-E----
XP_010413453.1/1-238 ------------------NVDEDVRIM-------TRK-SV--NIP--------G-E----
XP_010469036.1/1-242 ------------------NVDEDVRIM-------TRK-SV--NIP--------G-E----
XP_010469037.1/1-242 ------------------NVDEDVRIM-------TRK-SV--NIP--------G-E----
XP_010469038.1/1-242 ------------------NVDEDVRIM-------TRK-SV--NIP--------G-E----
XP_010512448.1/1-238 ------------------NVDEDVRIM-------TRK-SV--NIP--------G-E----
XP_010512449.1/1-238 ------------------NVDEDVRIM-------TRK-SV--NIP--------G-E----
XP_006397757.1/1-219 ------------------NIDEDVRIM-------TRK-NE--DDS--------G-E----
XP_024010837.1/1-220 ------------------NIDEDVRIM-------TRK-NE--DDS--------G-E----
XP_023640300.1/1-220 ------------------DIDEDVRIM-------TRK-NV--DDS--------G-E----
XP_006293718.1/1-220 ------------------DIDEDVRIM-------TRK-NV--DDS--------G-E----
XP_010506553.1/1-219 ------------------NIDEDVRIM-------TGK-NM--DDS--------G-E----
XP_010508114.1/1-219 ------------------NIDEDVRIM-------TGK-NM--DDS--------G-E----
XP_010508115.1/1-219 ------------------NIDEDVRIM-------TGK-NM--DDS--------G-E----
XP_022147276.1/1-219 ------------------SVGEDVRVM-------TRN-SV--DDP--------G-E----
XP_023527464.1/1-222 -----------------NNVGEDVKVM-------TRK-SV--EDP--------G-E----
XP_022980233.1/1-222 -----------------NNVGEDVRVM-------TRK-SV--EDP--------G-E----
XP_022980231.1/1-222 -----------------NNVGEDVRVM-------TRK-SV--EDP--------G-E----
XP_022924366.1/1-223 -----------------NNVGEDVRVM-------TRK-SV--EDP--------G-E----
XP_022924364.1/1-223 -----------------NNVGEDVRVM-------TRK-SV--EDP--------G-E----
XP_023527467.1/1-221 -----------------NNVGEDVKVM-------TRK-SV--EDP--------G-E----
XP_023527463.1/1-221 -----------------NNVGEDVKVM-------TRK-SV--EDP--------G-E----
XP_023527465.1/1-221 -----------------NNVGEDVKVM-------TRK-SV--EDP--------G-E----
XP_022980232.1/1-221 -----------------NNVGEDVRVM-------TRK-SV--EDP--------G-E----
XP_022924365.1/1-222 -----------------NNVGEDVRVM-------TRK-SV--EDP--------G-E----
XP_009412599.1/1-224 ------------------NVGDEVRVM-------TRM-SV--NEP--------G-E----
XP_018677084.1/1-231 -----------------INIGEDVRVK-------TSQ-NV--AEP--------G-E----
XP_020114860.1/1-246 ---------------GCAGVGDEVRVL-------TRQ-SV--SDP--------G-E----
XP_020114857.1/1-246 ---------------GCAGVGDEVRVL-------TRQ-SV--SDP--------G-E----
XP_020114859.1/1-246 ---------------GCAGVGDEVRVL-------TRQ-SV--SDP--------G-E----
XP_020084803.1/1-234 AGRG--------GGGGAMGVEEEVRVM-------TRQ-SV--DDP--------G-E----
XP_015625616.1/1-226 ------------------SIGEDVRVM-------ARK-SV--SEP--------G-E----
XP_006647677.1/1-226 ------------------SIGEDVRVM-------ARK-SV--SEP--------G-E----
NP_001105493.1/1-226 ------------------SIGEDVRVM-------ARK-SV--DEP--------G-E----
XP_020178635.1/1-225 ------------------SIAEDVRVM-------ARK-SV--SEP--------G-E----
XP_003570085.1/1-223 ------------------SIGEDVRVM-------ARK-SV--SEP--------G-E----
XP_020247871.1/1-226 -------------------SDDITRVK-------TQK-NI---EP--------G-Q----
XP_020247870.1/1-226 -------------------SDDITRVK-------TQK-NI---EP--------G-Q----
XP_024004764.1/1-211 -------------------TKDTVRIT-------TRK-V---------------------
XP_018449374.1/1-217 -------------------PKETARIT-------TRK-V---------------------
XP_013602839.1/1-215 -------------------PKETVIIT-------TRK-V---------------------
XP_013655966.1/1-215 -------------------PKETVRIT-------SRK-V---------------------
XP_009108342.1/1-215 -------------------PKETVRIT-------TRK-V---------------------
XP_013655964.1/1-215 -------------------PKETVRIT-------SRK-V---------------------
XP_020872468.1/1-222 -------------------TKDTVRIV-------TRK-V---------------------
NP_193506.2/1-221    -------------------TKDTVKIV-------SRK-V---------------------
XP_023633855.1/1-222 -------------------TKDTLRIL-------TRK-V---------------------
XP_010449572.1/1-221 -------------------TKDTVRIV-------TRK-V---------------------
XP_010439957.1/1-223 -------------------TKDTVRIV-------TRT-V---------------------
XP_010439959.1/1-223 -------------------TKDTVRIV-------TRT-V---------------------
XP_010439958.1/1-223 -------------------TKDTVRIV-------TRT-V---------------------
XP_006854056.1/1-234 -------------------PEDTVRIT-------TRK-NT---EV--------G-Q----
XP_020529072.1/1-234 -------------------PEDTVRIT-------TRK-NT---EV--------G-Q----
NP_199499.3/1-224    -------------------TKDTVRIT-------TRK-MC---EP--------G-Q----
XP_020871346.1/1-224 -------------------TKDTVRIT-------TRK-MC---EP--------G-Q----
XP_006281890.1/1-224 -------------------TKDTVRIT-------TRK-MC---EA--------G-Q----
XP_010481400.1/1-224 -------------------SKDTVRIT-------TRK-MC---EP--------G-Q----
XP_010494885.1/1-224 -------------------SKDTVRIT-------TRK-MC---EP--------G-Q----
XP_018455882.1/1-227 -------------------TKDTVRIT-------TRK-VC---EP--------G-Q----
XP_013713146.1/1-227 -------------------TKDTVRIT-------TRK-VC---EP--------G-Q----
XP_013607209.1/1-227 -------------------TKDTVRIT-------TRK-VC---EP--------G-Q----
XP_009114317.1/1-227 -------------------AKDTVRIT-------TRK-VC---EP--------G-Q----
XP_013661958.1/1-227 -------------------AKDTVRIT-------TRK-VC---EP--------G-Q----
XP_006398384.1/1-227 -------------------SKDTVRIT-------TRK-TC---EP--------G-Q----
XP_024011152.1/1-227 -------------------SKDTVRIT-------TRK-TC---EP--------G-Q----
XP_018482944.1/1-227 -------------------TKDTVRIT-------TRK-AC---EP--------G-Q----
XP_009101472.1/1-227 -------------------TKDTVRIT-------TRK-VC---EP--------G-Q----
XP_013693979.1/1-227 -------------------TKDTVRIT-------TRK-MC---EP--------G-Q----
XP_013615089.1/1-227 -------------------TKDTVRIT-------TRK-VC---EP--------G-Q----
XP_013704013.1/1-227 -------------------TKDTVRIT-------TRK-VC---EP--------G-Q----
XP_009373477.1/1-246 -------------------PDDTVRIT-------TRK-VT---EP--------G-Q----
XP_006662631.2/1-256 -------------------TEDTIRVT-------TRK-NT---EP--------G-Q----
XP_015613461.1/1-259 -------------------TQDTIRVT-------TRK-NT---EP--------G-Q----
NP_001337805.1/1-240 -------------------TEDTIRVT-------TRK-NT---DP--------G-Q----
XP_020163828.1/1-239 -------------------TDDTVRIT-------TRK-NT---EP--------G-Q----
XP_010235052.1/1-238 -------------------TEDTIRVT-------TRK-NT---EP--------G-Q----
XP_025798256.1/1-238 -------------------TEDTIRVT-------TRK-NT---DP--------G-Q----
XP_004983555.1/1-238 -------------------TEDTIRVT-------TRK-NT---DP--------G-Q----
XP_002464880.2/1-238 -------------------TEDTIRVT-------TRK-NT---DP--------G-Q----
XP_020262583.1/1-231 -------------------SDDTIRVT-------TRK-SM---EP--------G-Q----
XP_020677410.1/1-231 -------------------PDDTIRVT-------TRK-NT---LP--------G-Q----
XP_020577604.1/1-229 -------------------PDDTIRVT-------TRK-NT---LP--------G-Q----
XP_020577606.1/1-229 -------------------PDDTIRVT-------TRK-NT---LP--------G-Q----
XP_020577607.1/1-229 -------------------PDDTIRVT-------TRK-NT---LP--------G-Q----
XP_019709148.1/1-245 -------------------SDDTIRVT-------TRK-NT---EP--------G-Q----
XP_008803567.1/1-241 -------------------SDDTIRVT-------TRK-NT---EP--------G-Q----
XP_008803570.2/1-241 -------------------SDDTIRVT-------TRK-NT---EP--------G-Q----
XP_018681950.1/1-231 -------------------SDDTVRVT-------TKK-NT---AP--------G-Q----
XP_018681951.1/1-231 -------------------SDDTVRVT-------TKK-NT---AP--------G-Q----
XP_010919976.1/1-237 -------------------SDDTTRVT-------TRK-NT---EP--------G-Q----
XP_008800084.1/1-237 -------------------SDDTIRVT-------TRK-NT---EP--------G-Q----
XP_018679997.1/1-229 -------------------SDDTIRVT-------TRK-NT---EP--------G-Q----
XP_009397149.1/1-229 -------------------SDDTIRVT-------TRK-NT---EP--------G-Q----
XP_024995523.1/1-235 -------------------MEDTVRIT-------TRK-VT---EP--------G-Q----
XP_023736252.1/1-233 -------------------VEDTVRIT-------TRK-VT---EP--------G-Q----
XP_023736253.1/1-233 -------------------VEDTVRIT-------TRK-VT---EP--------G-Q----
XP_022022041.1/1-231 -------------------VEETVRIT-------TRK-VT---EP--------G-Q----
XP_021969857.1/1-226 -------------------VEDTVRIT-------TRK-VT---EP--------G-Q----
XP_010245213.1/1-236 -------------------VDDTVRIT-------TRK-NT---EA--------G-Q----
XP_010245210.1/1-236 -------------------VDDTVRIT-------TRK-NT---EA--------G-Q----
XP_010276832.1/1-235 -------------------ADDTVRIT-------TRK-NT---EP--------G-Q----
XP_010276837.1/1-235 -------------------ADDTVRIT-------TRK-NT---EP--------G-Q----
XP_019155823.1/1-230 -------------------AEDTVRIT-------TRK-VT---EP--------G-Q----
XP_022881662.1/1-238 -------------------AEDTVRIT-------TRK-VT---EP--------G-Q----
XP_022877113.1/1-234 -------------------AEDTVRIT-------TRK-VT---EP--------G-Q----
XP_016450789.1/1-246 -------------------LEDTVRIT-------TRK-VT---EP--------G-Q----
XP_009597691.1/1-246 -------------------LEDTVRIT-------TRK-VT---EP--------G-Q----
XP_009786380.1/1-246 -------------------LEDTVRIT-------TRK-VT---EP--------G-Q----
XP_016494823.1/1-246 -------------------LEDTVRIT-------TRK-VT---EP--------G-Q----
XP_016494822.1/1-246 -------------------LEDTVRIT-------TRK-VT---EP--------G-Q----
XP_019230103.1/1-246 -------------------LEDTVRIT-------TRK-VT---EP--------G-Q----
XP_016573090.1/1-250 -------------------LEDTVRIT-------TRK-VT---EP--------G-Q----
XP_016573102.1/1-250 -------------------LEDTVRIT-------TRK-VT---EP--------G-Q----
XP_006352911.1/1-250 -------------------PDDTVRIT-------TRK-VT---EP--------G-Q----
XP_015166604.1/1-250 -------------------PDDTVRIT-------TRK-VT---EP--------G-Q----
XP_015085651.1/1-250 -------------------PDDTVRIT-------TRK-VT---EP--------G-Q----
XP_015085654.1/1-250 -------------------PDDTVRIT-------TRK-VT---EP--------G-Q----
XP_004245919.1/1-250 -------------------PDDTVRIT-------TRK-VT---EP--------G-Q----
XP_025888165.1/1-250 -------------------PDDTVRIT-------TRK-VT---EP--------G-Q----
XP_011070327.1/1-236 -------------------ADDTVRIT-------TRK-VT---EP--------G-Q----
XP_011070329.1/1-236 -------------------ADDTVRIT-------TRK-VT---EP--------G-Q----
XP_012843107.1/1-236 -------------------ADDTVRIT-------TRK-VT---QP--------G-Q----
XP_019423575.1/1-234 -------------------PEDTVRIT-------TRK-IT---EP--------G-Q----
XP_019423571.1/1-234 -------------------PEDTVRIT-------TRK-IT---EP--------G-Q----
XP_019435331.1/1-234 -------------------PEDTVRIT-------TRK-IT---DS--------G-Q----
XP_003612811.1/1-239 -------------------PEDTVRIT-------TRK-IT---EP--------G-Q----
XP_020978415.1/1-227 -------------------PEDTVRIT-------TRK-IT---EP--------G-Q----
XP_015963259.1/1-227 -------------------PEDTVRIT-------TRK-IT---EP--------G-Q----
XP_025697880.1/1-227 -------------------PEDTVRIT-------TRK-IT---EP--------G-Q----
XP_025697882.1/1-227 -------------------PEDTVRIT-------TRK-IT---EP--------G-Q----
XP_025697881.1/1-227 -------------------PEDTVRIT-------TRK-IT---EP--------G-Q----
XP_020217931.1/1-233 -------------------PEDTVRIT-------TRK-VT---EP--------G-Q----
XP_007158253.1/1-232 -------------------PEDTVRIT-------TRK-VT---EP--------G-Q----
XP_017427665.1/1-232 -------------------PEDTVRIT-------TRK-VT---EP--------G-Q----
XP_017427666.1/1-232 -------------------PEDTVRIT-------TRK-VT---EP--------G-Q----
XP_014521371.1/1-232 -------------------PEDTVRIT-------TRK-VT---EP--------G-Q----
XP_014521373.1/1-232 -------------------PEDTVRIT-------TRK-VT---EP--------G-Q----
XP_006587604.1/1-242 -------------------PEDTVRIT-------TRK-IT---EP--------G-Q----
NP_001352051.1/1-233 -------------------PEDTVRIT-------TRK-IT---EP--------G-Q----
XP_018840870.1/1-239 -------------------PDDTVRIA-------TRK-IT---EP--------G-Q----
XP_018852246.1/1-240 -------------------PDDTVRIS-------TRK-IT---EP--------G-Q----
XP_010053633.1/1-234 -------------------SDDTIRIT-------TRK-IT---EP--------G-Q----
XP_021675948.1/1-236 -------------------CNDTVRIT-------TRK-IT---EP--------G-Q----
XP_012081064.1/1-228 -------------------SDDTVRIT-------TRK-IT---EP--------G-Q----
XP_011028126.1/1-235 -------------------YDDTVRIT-------TRK-IT---EP--------G-Q----
XP_002303433.2/1-235 -------------------YDDTVRIT-------TRK-IT---EP--------G-Q----
XP_015897558.1/1-247 -------------------PEDTVRIT-------TRK-VT---EP--------G-Q----
XP_002277673.1/1-238 -------------------SDDTVRIT-------TRK-IT---EP--------G-Q----
XP_022996123.1/1-236 -------------------PDDTVRIT-------TRK-IV---EP--------G-Q----
XP_023534245.1/1-236 -------------------PDDTVRIT-------TRK-IV---EP--------G-Q----
XP_022931244.1/1-236 -------------------PDDTVRIT-------TQK-IV---EP--------G-Q----
XP_022931242.1/1-236 -------------------PDDTVRIT-------TQK-IV---EP--------G-Q----
XP_022159691.1/1-235 -------------------PDDTVRIT-------TRK-IV---EP--------G-Q----
XP_011652640.1/1-232 -------------------PEDTVRIT-------TRK-VV---EP--------G-Q----
XP_011652639.1/1-232 -------------------PEDTVRIT-------TRK-VV---EP--------G-Q----
XP_008466007.1/1-232 -------------------PEDTVRIT-------TRK-VV---EP--------G-Q----
XP_023511589.1/1-230 -------------------PDDTVRIT-------TRK-IV---EP--------G-Q----
XP_022963906.1/1-230 -------------------PDDTVRIT-------TRK-IV---EP--------G-Q----
XP_023511591.1/1-230 -------------------PDDTVRIT-------TRK-IV---EP--------G-Q----
XP_022963908.1/1-230 -------------------PDDTVRIT-------TRK-IV---EP--------G-Q----
XP_022967184.1/1-230 -------------------PDDTVRIT-------TRK-II---EP--------G-Q----
XP_022967186.1/1-230 -------------------PDDTVRIT-------TRK-II---EP--------G-Q----
XP_010518669.1/1-225 -------------------AQDTVRIT-------TRK-LS---EP--------G-H----
XP_010677365.1/1-235 -------------------AEDTVRIT-------TRK-IM---EP--------G-Q----
XP_010677364.1/1-235 -------------------AEDTVRIT-------TRK-IM---EP--------G-Q----
XP_021852084.1/1-226 -------------------ADDTVRIT-------TRK-IM---EP--------G-Q----
XP_021852076.1/1-226 -------------------ADDTVRIT-------TRK-IM---EP--------G-Q----
XP_021765591.1/1-233 -------------------ADDTVRIT-------TRK-IM---EP--------G-Q----
XP_021768254.1/1-233 -------------------SDDTVRIT-------TRK-IM---EP--------G-Q----
XP_017232690.1/1-221 -------------------PEDTIRIT-------TTK-AT---EP--------G-H----
XP_017232692.1/1-221 -------------------PEDTIRIT-------TTK-AT---EP--------G-H----
XP_024021697.1/1-248 -------------------PDDTVRIT-------TRK-IT---EP--------G-Q----
XP_010096931.1/1-248 -------------------PDDTVRIT-------TRK-IT---EP--------G-Q----
XP_024021696.1/1-248 -------------------PDDTVRIT-------TRK-IT---EP--------G-Q----
XP_017621310.1/1-230 -------------------SDDTVRIT-------TRK-VS---EA--------G-Q----
XP_016731150.1/1-230 -------------------SDDTVRIT-------TRK-VS---EA--------G-Q----
XP_012472199.1/1-230 -------------------SDDTVRIT-------TRK-VS---EA--------G-Q----
XP_016727305.1/1-230 -------------------SDDTVRIT-------TRK-VS---EA--------G-Q----
XP_016731145.1/1-230 -------------------SDDTVRIT-------TRK-VS---EA--------G-Q----
XP_012472200.1/1-230 -------------------TDDTVRIT-------TRK-VS---EA--------G-Q----
XP_012472201.1/1-230 -------------------TDDTVRIT-------TRK-VS---EA--------G-Q----
NP_001314348.1/1-230 -------------------SDDTVRIT-------TRK-VS---EA--------G-Q----
XP_016727300.1/1-230 -------------------TDDTVRIT-------TRK-VS---EA--------G-Q----
XP_022738572.1/1-235 -------------------SDDTVRIS-------TKK-VT---EP--------G-Q----
XP_021297648.1/1-235 -------------------ADDTVRIT-------TRK-VT---EP--------G-Q----
XP_017973778.1/1-235 -------------------ADDTVRIT-------TSK-VT---EA--------G-Q----
XP_017973780.1/1-235 -------------------ADDTVRIT-------TSK-VT---EA--------G-Q----
XP_022717458.1/1-235 -------------------ADDTVRIT-------TRK-IT---EP--------G-Q----
XP_022717453.1/1-235 -------------------ADDTVRIT-------TRK-IT---EP--------G-Q----
XP_022717437.1/1-235 -------------------ADDTVRIT-------TRK-IT---EP--------G-Q----
XP_022756982.1/1-232 -------------------SDDTVRIT-------TRK-VM---EP--------G-Q----
XP_004300207.1/1-230 -------------------PDDTIRIT-------TRK-VS---EA--------G-Q----
XP_024169578.1/1-230 -------------------PDDTIRIT-------TRK-VS---EA--------G-Q----
XP_008238183.1/1-249 -------------------PDDTVRIT-------TRK-VT---DP--------G-Q----
XP_016651445.1/1-249 -------------------PDDTVRIT-------TRK-VT---DP--------G-Q----
XP_020418924.1/1-249 -------------------PDDTVRIT-------TRK-VT---DP--------G-Q----
XP_021804146.1/1-249 -------------------PDDTVRIT-------TRK-VT---DP--------G-Q----
XP_008238184.1/1-248 -------------------PDDTVRIT-------TRK-VT---DP--------G-Q----
XP_016651446.1/1-248 -------------------PDDTVRIT-------TRK-VT---DP--------G-Q----
XP_007209851.1/1-248 -------------------PDDTVRIT-------TRK-VT---DP--------G-Q----
XP_021804154.1/1-248 -------------------PDDTVRIT-------TRK-VT---DP--------G-Q----
XP_017179076.1/1-239 -------------------PDDTVRIT-------TRK-VT---DP--------G-Q----
XP_008356445.1/1-246 -------------------PGDTVRIT-------TRK-VT---EA--------G-Q----
XP_008356446.1/1-240 -------------------PGDTVRIT-------TRK-VT---EA--------G-Q----
XP_008356447.1/1-239 -------------------PGDTVRIT-------TRK-VT---EA--------G-Q----
XP_009373485.1/1-239 -------------------PDDTVRIT-------TRK-VT---EP--------G-Q----
XP_009373484.1/1-240 -------------------PDDTVRIT-------TRK-VT---EP--------G-Q----
XP_004512486.1/1-245 -------------------PEDTVRIT-------TRK-IT---EP--------G-Q----
XP_023898631.1/1-242 -------------------PDDTVRIN-------TRK-IS---EL--------G-Q----
XP_024465375.1/1-238 -------------------HDGTVRII-------SRE-IT---EP--------G-Q----
XP_011018502.1/1-238 -------------------HDGTVRII-------SRE-IT---EP--------G-Q----
XP_011018503.1/1-238 -------------------HDGTVRII-------SRE-IT---EP--------G-Q----
XP_021893615.1/1-233 -------------------VDDTVRIT-------TRK-IT---EP--------G-Q----
XP_024952893.1/1-247 -------------------CADTVRIT-------TRK-IT---EP--------G-Q----
XP_006471522.1/1-248 -------------------CADTVRIT-------TRK-IT---EP--------G-Q----
XP_006432745.2/1-248 -------------------CADTVRIT-------TRK-IT---EP--------G-Q----
XP_021640807.1/1-241 -------------------CDDIVRIT-------TRK-IT---EP--------G-Q----
XP_002518976.1/1-243 -------------------SDDTVRIT-------TRK-IT---EP--------G-Q----
XP_021598537.1/1-237 -------------------SDDAVRIT-------TRK-IT---EP--------G-Q----
XP_021598535.1/1-239 -------------------SDDAVRIT-------TRK-IT---EP--------G-Q----
XP_006659280.1/1-226 -----------------MLQDVDVTQF-------ANQ-IIG-GAT--------GE-----
XP_025801379.1/1-254 --------------GVERFD-VAVRMV-------TWE-KAG-SSV--------AGE----
XP_023156326.1/1-229 --------------GARRTD-TAVRMV-------TSK-KAG-T-----------------
NP_001349156.1/1-229 --------------GARRTD-TAVRMV-------TSK-KAG-T-----------------
XP_021310588.1/1-246 --------------DARRTDDAAVRMV-------TWK-KPG-S-----------------
XP_020404516.1/1-224 --------------TGEMLE-PAVRMV-------LGN-YFG-AMD--------GQP----
XP_022684239.1/1-229 --------------GAKTFL-ATVRMV-------IWN-IST---M--------GQP----
XP_020404892.1/1-233 --------------GAERVE-AVVRLA-------VWN-CAD-IMP--------GEP----
XP_022684238.1/1-237 --------------AAERVD-AAVRMV-------IWN-CAE-IMP--------GEP----
XP_025825251.1/1-229 --------------GAERVD-AAVRMV-------TWN-CAE-IMP--------GEP----
XP_020183090.1/1-242 ----------------ESFQ-LAMHVL-------TPL-AAPG------------EQ----
XP_020194819.1/1-240 --------------GAERFE-VDVRVV-------TFP-VG--------------------
XP_024314575.1/1-244 --------------GSERFE-LAVHFV-------TL------------------------
XP_024314342.1/1-235 --------------GAEKLH-LAVRFV-------TSL-AAIYNGD--------IQT----
XP_024959674.1/1-192 ------------------GSAEPIYLK-------WDY-F-QY-DL--------G-R----
XP_019196338.1/1-215 ------------------DA-KEIFVR-------LNT--NLD-DL--------G-L----
XP_006646400.1/1-226 ------------------GTEKDVRVA-------YRG-ITEG-S-----------S----
XP_015632244.1/1-226 ------------------GTEKDIRVA-------YRR-TTEG-S-----------S----
XP_024314625.1/1-231 ------------------GTEQDIKVA-------HKR-NDD--------------G----
XP_020164122.1/1-228 ------------------GTEDDIRIT-------YRK-NND--------------D----
XP_025814677.1/1-221 ------------------GTEEDVKIA-------YRR-NDD--------------G----
XP_022682466.1/1-225 ------------------GTEEDVRIV-------YRR-NDD--------------T----
XP_002456467.1/1-226 ------------------GTEQDIKIM-------YKT-QNES-TS--------S-G----
XP_008674944.1/1-228 ------------------GTEQDIKIM-------YKR-QSEG-SS--------S-S----
XP_010523539.1/1-228 -----------------RLLDHNIRVF-------SRE-TQD---R--------G-N----
XP_020873847.1/1-203 ------------------GRTFDVRVF-------TRE-SPDM--I--------R-H----
NP_001190248.1/1-203 -----------------GVSANNIRMF-------TQE-SRGM--S--------G-I----
NP_196343.2/1-203    -----------------GVSANNIRMF-------TQE-SRGM--S--------G-I----
XP_020876233.1/1-195 -----------------GGSVNNIRMF-------TRE-SRDM--S--------G-I----
XP_010423235.1/1-188 -----------------GFSVNNIRMF-------TRE-SRDM--N--------R-I----
XP_010452682.1/1-218 -----------------GFSVHNIRMF-------TRE-SRDM--N--------G-I----
XP_010491330.1/1-188 -----------------GFSVNNIRMF-------TRE-SRDM--S--------G-I----
XP_006413300.2/1-224 --------------NDEEGQEKNVRLM-------SRK-NVCV--R--------G-E----
XP_022008499.1/1-216 -------------------VPGRPRIM-------ARM-YN-N--F--------Q-G----
XP_022008502.1/1-215 -------------------VPGRPRIM-------ARM-YN-D--F--------Q-G----
XP_022007635.1/1-226 -------------------APGIPRIM-------ARK-CITG--S--------G-E----
XP_022008501.1/1-224 -------------------APGRPRIM-------ARN-FISG--F--------S-E----
XP_021986984.1/1-226 -------------------TPKRPRIM-------AHN-YISG--F--------G-E----
XP_021996009.1/1-224 -------------------APRRPRIM-------AHN-YISG--F--------G-E----
XP_021996011.1/1-224 -------------------APKRPRIM-------AHN-YISG--F--------G-E----
XP_022026050.1/1-224 -------------------APRRPRIM-------AHN-YISG--F--------G-E----
XP_022026083.1/1-224 -------------------APRRPRIM-------AHN-YISG--F--------G-E----
XP_021722389.1/1-209 ---------------------GGDDVL-------IKT-LDS------------D-D----
XP_021847315.1/1-219 ---------------------GGDCIL-------IKT-LNS------------D-D----
XP_020224300.1/1-216 -----------------QENSNAIKIT-------LRT-NTAP-----------D-M----
XP_021765248.1/1-224 -----------------ELKSNRVKID-------VVK-NTTS-----------D-L----
XP_004510020.1/1-219 -----------------NISNSDEIQF-------NH--------R--------K-F----
XP_004487953.1/1-231 -----------------ENNNNEPLIF-------IRQ-RTILSQS--------Q-R----
XP_008244376.1/1-218 --------------------GADIMIS-------TKS-STDD--H--------G-K----
XP_008244380.1/1-218 --------------------GADIMIS-------TKS-STDD--H--------G-K----
XP_012569676.1/1-224 -------------------VIGGIRLS-------IRE-TGI------------G-E----
XP_019418393.1/1-229 --------------------NEGVWIS-------LNQ-MNIE--P--------G-H----
XP_019440118.1/1-223 --------------------SEGIWIS-------VK--ENIK--H--------G-H----
XP_014517363.2/1-226 -----------------E--NTDIMVL-------AGK-YTNP--S--------Q-S----
XP_012569675.1/1-231 -----------------DADYNGIRLS-------VRK-NGGL--N--------E-P----
XP_004486570.1/1-223 -----------------DADDSGVRFC-------LRN-NTN-------------------
XP_003594569.3/1-220 -----------------DTDDSGVRFS-------IPK-NRNL--F--------L-S----
XP_024632692.1/1-220 -----------------DTDDSGVRFS-------IRK-NRNL--F--------L-S----
XP_025887127.1/1-229 -------------------TNENPKLM-------MRT--NVS-DP--------S-E----
XP_015078684.1/1-230 -------------------TNENPKLM-------MRT--NVS-DP--------S-E----
XP_025887126.1/1-230 -------------------TNENPKLM-------MRT--NVS-DP--------S-E----
XP_015078401.1/1-220 -------------------KGKDVKLM-------KRN--NIS-DL--------G-E----
XP_015158871.1/1-217 -------------------KGKGVKLM-------KRN--NIS-DL--------G-E----
XP_015078778.1/1-220 -------------------NGKDANLM-------MRR--NIS-DA--------G-E----
XP_010321992.1/1-216 -------------------NGKDANLM-------MRR--NIS-DA--------G-E----
XP_004240735.1/1-216 -------------------NGKDANLM-------MRR--NIS-DA--------G-E----
XP_015169793.1/1-213 -------------------NGVDAKLM-------MRT--SIG-DH--------T-E----
XP_006285631.1/1-212 ------------------NVAQNMRFM-------TRK--SLN-EP--------G-E----
XP_023635531.1/1-198 ------------------NVAQNMRFM-------TRK--SLN-EP--------G-E----
NP_567722.1/1-217    ------------------NVAQNMSFM-------IRK--NVN-EP--------G-E----
XP_020873337.1/1-219 ------------------NVVQNMSFM-------IRK--NVN-EP--------G-E----
XP_020873338.1/1-219 ------------------NVVQNMSFM-------IRK--NVN-EP--------G-E----
XP_023000325.1/1-225 -------------------IGEIIRVA-------SRK--NLN-DP--------R-E----
XP_023000326.1/1-214 -------------------IGEIIRVA-------SRK--NLN-DP--------R-E----
XP_022963997.1/1-214 -------------------MGENIRVA-------SRK--NLN-DP--------R-E----
XP_023513617.1/1-208 -------------------IGENIRVA-------SRK--NLN-DP--------R-E----
XP_021312124.1/1-225 ------------------NGPCEIRVM-------CRK--SRG-EG--------G-E----
XP_012701492.1/1-243 ---------------RGGGQGDGVRMT-------SRR--NIG-DP--------G-E----
XP_025815339.1/1-229 -----------NLGGGGGGAGHGVRVT-------SRR--NVG-DP--------G-E----
XP_015644459.1/1-237 --------------GSGGGGDDDIWLT-------SRE--NAGDDP--------G-E----
XP_024314016.1/1-228 --------------GAG-----DIRVT-------SRR--SAG-----------G-E----
XP_020168333.1/1-225 --------------GAG-----DVRLT-------SRT--NAG-DP--------G-E----
XP_013589242.1/1-206 ------------------SARQDIRVM-------IRK---N-XNV--------G-E----
XP_022548456.1/1-204 ------------------SARQDIRVM-------IRK---NNNNV--------G-E----
XP_024632691.1/1-227 -------------------SIGGVRVS-------IRA--TTDDDA--------S-Q----
XP_017256377.1/1-220 -------------------NGENILVK-------TSF---NVDDP--------G-H----
XP_009632057.1/1-224 -------------------GGGNNFAK-------TSL---NLDAR--------G-T----
XP_016502932.1/1-224 -------------------GGGNNFAK-------TSL---NLDAR--------G-T----
XP_019258997.1/1-221 -------------------GGGDIFVK-------TGL---NLDAR--------G-T----
XP_009792862.1/1-224 -------------------GGGNIFVK-------TSL---NSDAR--------G-T----
XP_016544784.1/1-213 -------------------GGEDIFAK-------TSM---NLDVP--------G-T----
XP_015089689.1/1-213 -------------------GGEDIFVK-------TNM---NLDAP--------G-T----
XP_025883585.1/1-213 -------------------GGEDIFVK-------TNM---NLDAP--------G-T----
XP_011095790.1/1-221 -------------------GAEEILIK-------TSF---NLDDP--------Q-I----
XP_020553953.1/1-221 -------------------GAEEILIK-------TSF---NLDDP--------Q-I----
XP_022857758.1/1-208 -------------------GAQDTLIK-------TSL---NIDKP--------G-V----
XP_004487739.1/1-224 ------------R------GSTDVRVM-------INN--NMNGTT--------N-K----
XP_003596663.1/1-221 ------------P-----AGSADIRVM-------IAN--DMAGST--------N-E----
XP_003596665.1/1-227 ------------P-----AGSEDIRVM-------IGN--NMAGI----------------
XP_020201972.1/1-216 ------------T------GCTDVRVM-------MKN---NKELT--------M-N----
XP_025980734.1/1-220 ------------H------GDSDVKVM-------IKN---NVEDT--------A-M----
XP_007149715.1/1-218 -------------------YSPYVRIM-------VKN---NMDEP--------G-K----
XP_017423636.1/1-218 -------------------HSPYVRIM-------VKN---NMEEA--------G-K----
XP_022631965.1/1-218 -------------------HSPYVRIM-------VKN---NMGEA--------G-K----
XP_014522034.2/1-218 -------------------HSPYVRIM-------VKN---NMGEA--------G-K----
XP_025669173.1/1-223 ------------E-----SDSSDVRVM-------IRN---NIEDP--------G-K----
XP_020963786.1/1-223 ------------E-----SDSSDVRVM-------IRN---NIEDP--------G-K----
XP_025614692.1/1-223 ------------E-----SDSSDVRVM-------IRN---NIEDP--------G-K----
XP_015936351.1/1-223 ------------E-----SDSSDVRVM-------IRN---NIEDP--------G-K----
XP_019451675.1/1-226 ------------P------GSADVKFI-------VKN--N-SDEI--------G-K----
XP_019465428.1/1-219 ------------Y------GSTDVRYI-------VKN--NNNSET--------G-K----
XP_004295798.2/1-220 -------------------GAEDVKVT-------TIT---NLNDI--------G-K----
XP_021807338.1/1-219 -------------------G-ADIMIS-------TKS---STDDH--------G-K----
XP_021807340.1/1-219 -------------------G-ADIMIS-------TKS---STDDH--------G-K----
XP_020417197.1/1-219 -------------------S-ADIMIS-------TKS---STDDH--------G-K----
XP_020417200.1/1-219 -------------------S-ADIMIS-------TKS---STDDH--------G-K----
XP_009333923.1/1-206 -----------PV-----SGAENVRIS-------MKS---STDDP--------G-K----
XP_017184363.1/1-206 -----------XI-----SGAENVRIS-------MKS---STDDP--------G-K----
XP_021892911.1/1-224 -------------------GGEDIKYM-------TRV---FALGP--------A-R----
XP_002278971.2/1-210 -------------------GAENVRAM-------IRS---GEDEE--------G-R----
XP_010644412.1/1-211 -------------------GAENIEAI-------IRF---VKDKE--------G-R----
XP_024450660.1/1-210 -------------------DGEDFRLM-------TKT---IYALN----------G----
XP_002522867.2/1-214 -------------------GAEDFRMM-------TKS---IGDNS----------G----
XP_020534605.1/1-220 -------------------RAEDFRVM-------TKT---ITPSP----------A----
XP_021685850.1/1-217 -------------------GGEDVRVM-------TKS---ITPAT--------S-E----
XP_021594464.1/1-220 -------------------GGEDFRVM-------TKS---ITAAI--------S-G----
XP_018731177.1/1-223 ------------S------GGQDMRVT-------TRT--NRGGEI--------G-R----
XP_024018593.1/1-217 ------------R-----ANNRDTTLM-------MNS-TFNKDDH--------G-K----
XP_022139420.1/1-219 -------------------GAEDIRVM-------TRN---GKDDP--------G-K----
XP_022981426.1/1-220 -------------------GPHDIRVM-------TQK--AKENDP--------G-R----
XP_022981427.1/1-220 -------------------GPHDIRVM-------TQK--AKENDP--------G-R----
XP_022941489.1/1-220 -------------------GPHDIRVM-------TQK--AKENDP--------G-R----
XP_022941488.1/1-220 -------------------GPHDIRVM-------TQK--AKENDP--------G-R----
XP_023525599.1/1-220 -------------------GPHDIRVM-------TQK--AKENDP--------G-R----
XP_023525597.1/1-220 -------------------GPHDIRVM-------TQK--AKENDP--------G-R----
XP_008454681.2/1-220 -------------------GPQDIRVV-------VTP--NLNDDP--------G-R----
XP_011648553.1/1-223 -------------------GPQDIRVV-------VTP--NLNDDP--------G-R----
XP_018822340.1/1-211 ------------P-----GADQDVRIM-------TKF---NVDDP--------G-K----
XP_018822367.1/1-211 ------------P-----GADQDVRIM-------TKF---NVDDP--------G-K----
XP_023876961.1/1-210 -------------------GAEDMRIM-------TKY---SKYEP--------G-I----
XP_023876962.1/1-210 -------------------GAEDMRIM-------TKY---SKYEP--------G-I----
XP_024034242.1/1-215 -------------------GAEDIRAM-------TKG---SIPVP--------G-M----
XP_015381349.2/1-215 -------------------GAEDIRAM-------TKG---SIPVP--------G-M----
XP_022717683.1/1-216 ------------I-----G-GEDIRFR-------FGN---TLAVP--------R-K----
XP_016729588.1/1-216 ------------F-----A-AQDVRLR-------YGN---ILKVP--------G-K----
XP_016729589.1/1-216 ------------F-----A-AQDVRLR-------YGN---ILKVP--------G-K----
XP_016702140.1/1-208 ------------F-----A-AQDVRLR-------YGN---ILKVP--------G-K----
XP_012460158.1/1-216 ------------F-----A-AQDVRLR-------YGN---ILKVP--------G-K----
XP_012460145.1/1-216 ------------F-----A-AQDVRLR-------YGN---ILKVP--------G-K----
XP_017245631.1/1-197 ------------------------------IHTTFRK---C-TDL--------V-Y----
XP_004956045.3/1-247 -------------------GVERPDAP--VRMVTWRK---AGTAS--------G-E----
XP_014660176.1/1-240 -------------------GAERIAAA--VRMVTWKK---AGSMD--------G-E----
XP_017239609.1/1-213 -------------------FGGGVRVM-------STR---VTDHP--------G-L----
XP_023740378.1/1-206 -------------------PARI---M-------TRE---S---M--------S-S----
XP_020095129.1/1-235 -------------------GTEEEAAAKVMSKTINNN---GDQDP--------N-N----
XP_020258373.1/1-212 -------------------GTESVKIA-------SKR---NVGDP--------R-M----
XP_008812148.1/1-175 -------------------GAEDVKIT-------AKR---NPLAT--------C-E----
XP_010930674.1/1-230 -------------------GAEDVKIT-------AKR---NTVAS--------G-S----
XP_010931877.1/1-230 -------------------GAEDVKIT-------AKR---NTVVT--------G-S----
XP_009380061.1/1-225 -------------------N-DDLRVT-------LHR-TD-S-S----------------
XP_010939971.1/1-229 ------------------TD-VGVRLT-------IYR-SS-G-PA--------S-L----
XP_019710626.1/1-218 ------------------TD-VGVRLT-------IYR-SS-G-PA--------S-L----
XP_008787891.1/1-229 ------------------TD-VGVRLT-------IYR-SP-G-PA--------S-H----
XP_008787892.1/1-218 ------------------TD-VGVRLT-------IYR-SP-G-PA--------S-H----
XP_008799024.1/1-230 ------------------TD-VGIRLT-------VYR-SP-G-PS--------D-H----
XP_010936845.1/1-230 ------------------TD-VGIRLT-------VYR-SP-G-PS--------D-Y----
XP_003570542.1/1-223 ------------SSGGGGDD-VGVRVM-------VHR-ST-D-P---------G-Q----
XP_004964922.1/1-229 ------------------TD-VSVRVS-------SHR-SA-D-P---------G-Q----
XP_002438026.1/1-230 ------------------TD-VSVRVS-------THR-ST-D-SG--------G-Q----
XP_020399989.1/1-232 ------------------TD-VSVCVS-------THR-ST-D-S---------G-Q----
XP_008649207.1/1-222 ------------------TD-VSVRVS-------THR-ST-D-S---------G-Q----
XP_020256822.1/1-228 ------------------NE-VRVRFI-------PHK-SA-D-P---------G-Q----
XP_009403550.1/1-228 ------------------ND-VGVRVV-------LQK-TS-D-A---------G-Q----
XP_020582158.1/1-232 ------------------ND-VGVRVT-------VHR-CS-D-P---------G-Q----
XP_020699118.1/1-240 ------------------ND-SGVRVT-------VHK-CS-D-S---------G-QANGV
XP_020600375.1/1-232 ------------------ND-VGIRVT-------VHK-CS-D-P---------G-Q----
XP_020600376.1/1-232 ------------------ND-VGIRVT-------VHK-CS-D-P---------G-Q----
XP_020684394.1/1-232 ------------------ND-VGIRVT-------VHK-CS-D-S---------G-Q----
XP_017257147.1/1-219 ------------------ND-FEVRAT-------LQK-CT-D--P--------G-H----
XP_010244428.1/1-230 ------------------GD-SGVRVI-------AHK-ST-E--P--------G-Q----
XP_010244355.1/1-230 ------------------GD-SGVRVI-------AHK-ST-E--P--------G-Q----
XP_017240757.1/1-236 -------------------DELEVRAS-------IHK-FT-N--P--------G-Q----
XP_017245021.1/1-233 ------------------PNEFEVRAN-------LHR-FA-N--L--------G-Q----
XP_012829269.1/1-233 ------------------AE-YEVRAA-------LHK-ST-D--L--------G-Q----
XP_022889938.1/1-236 ------------------NE-FEVRAA-------LHK-ST-D--P--------G-Q----
XP_022897471.1/1-232 ------------------NE-FEVRAA-------LHK-ST-D--P--------G-Q----
XP_009606250.1/1-235 ------------------DE-FEIRAT-------LQK-SI-D--P--------G-Q----
XP_016468230.1/1-235 ------------------DE-FEIRAT-------LQK-SI-D--P--------G-Q----
XP_019250773.1/1-235 ------------------DE-FEVRAT-------LQK-ST-D--P--------G-Q----
XP_009778533.1/1-235 ------------------DE-FEVRAT-------LQK-ST-D--P--------G-Q----
XP_016451582.1/1-235 ------------------DE-FEVRAT-------LQK-ST-D--P--------G-Q----
XP_016578176.1/1-240 ------------------NE-FEVRAT-------LQK-CT-D--P--------G-Q----
XP_006347370.1/1-231 ------------------DE-FEVRAT-------LQK-CT-D--P--------G-Q----
XP_004241474.1/1-230 ------------------DE-FEVRAT-------LQK-CT-D--P--------G-Q----
XP_015077748.1/1-230 ------------------DE-FEVRAT-------LQK-CT-D--P--------G-Q----
XP_022037776.1/1-241 ------------------NE-FEVRAI-------LYK-ST-D--P--------G-H----
XP_024992062.1/1-237 ------------------NE-FEVRAF-------LHK-SN-D--P--------G-Q----
XP_023772270.1/1-245 ------------------NDQFEVRAT-------LYK-TT-D-PA--------G-H----
XP_012855673.1/1-237 ------------------NE-FEVRAT-------LHR-ST-D--P--------A-Q----
XP_022858328.1/1-236 ------------------NE-FEVRAT-------LHK-ST-D--P--------G-Q----
XP_011072039.1/1-232 ------------------NE-FEVRAT-------VHR-II-D--P--------G-Q----
XP_006306921.1/1-215 ------------------DE-FAIRVT-------SHK-SL-------------H-E----
NP_564041.2/1-215    ------------------DE-FGIRVT-------SHK-SR-------------H-E----
XP_020866823.1/1-216 ------------------DE-FGIRVT-------SHK-SR-------------H-E----
XP_010476956.1/1-215 ------------------DE-FAVRVT-------SRK-SR-------------H-E----
XP_010498167.1/1-217 ------------------DE-FAVRVA-------SHK-SR-------------H-E----
XP_019091528.1/1-217 ------------------DE-FAVRVA-------SHK-SR-------------H-E----
XP_013613976.1/1-227 ------------------DA-FGIRVT-------SYK-SQ-------------H-E----
XP_018432674.1/1-227 ------------------DA-FGIRVT-------SYK-SQ-------------N-E----
XP_018432667.1/1-227 ------------------DA-FGIRVT-------SYK-SQ-------------N-E----
XP_013641731.1/1-225 ------------------DA-FGIRVT-------SYK-SQ-------------H-E----
XP_009149243.1/1-227 ------------------DA-FGIRVT-------SYK-SQ-------------H-E----
XP_006416656.2/1-224 ------------------DE-FGIRVT-------SYK-SQ-------------H-E----
XP_018451633.1/1-224 ------------------NE-FGIRVT-------SYK-SQ-------------H-E----
XP_018451632.1/1-225 ------------------NE-FGIRVT-------SYK-SQ-------------H-E----
XP_018451634.1/1-195 ------------------NE-FGIRVT-------SYK-SQ-------------H-E----
XP_013690958.1/1-228 ------------------NE-FGIRVT-------SYK-SH-------------H-E----
XP_009110376.1/1-227 ------------------NE-FGIRVT-------SYK-SH-------------H-E----
XP_009110377.1/1-227 ------------------NE-FGIRVT-------SYK-SH-------------H-E----
XP_013601964.1/1-227 ------------------NE-FGIRVT-------SYK-SQ-------------H-E----
XP_013653874.1/1-227 ------------------NE-FGIRVT-------SYK-SQ-------------H-E----
XP_018445004.1/1-237 ------------------ND-VGVRVT-------AHK-S--------------P-E----
XP_018445005.1/1-237 ------------------ND-VGVRVT-------AHK-S--------------P-E----
XP_013648946.1/1-228 ------------------ND-VGVRVT-------AHK-S--------------P-E----
XP_009104812.1/1-228 ------------------ND-VGVRVT-------AHK-S--------------P-E----
XP_013588523.1/1-228 ------------------NE-VGVRVT-------AHK-S--------------P-E----
XP_013699248.1/1-228 ------------------NE-VGVRVT-------AHK-S--------------P-E----
XP_018457296.1/1-228 ------------------NE-VGVRVT-------AHK-S--------------P-E----
XP_009105998.1/1-230 ------------------NE-VGVRVT-------AHK-S--------------P-E----
NP_001302850.1/1-231 ------------------NE-VGVRVT-------AHK-S--------------P-E----
XP_013698656.1/1-231 ------------------NE-VGVRVT-------AHK-S--------------P-E----
XP_013649619.1/1-231 ------------------NE-AGVRVT-------AHK-S--------------P-E----
XP_013592873.1/1-231 ------------------NE-VGVRVT-------AHK-S--------------P-E----
XP_006390565.1/1-233 ------------------NE-VGIRVT-------AQK-S--------------P-E----
XP_006301097.1/1-230 ------------------NE-VGIRVT-------AHK-S--------------P-E----
XP_010428260.1/1-231 ------------------NE-VGVRVT-------AHK-S--------------P-E----
XP_010471396.1/1-230 ------------------NE-VGVRVT-------EHK-S--------------P-E----
XP_010416135.1/1-230 ------------------NE-VGVRVT-------AHK-S--------------P-E----
NP_177479.1/1-227    ------------------NE-VGIRVT-------AHK-S--------------P-E----
XP_020890987.1/1-228 ------------------NE-VGIRVT-------AHK-S--------------P-E----
XP_010537520.1/1-232 ------------------NE-VGVRVT-------AHK-S--------------P-E----
XP_010540042.1/1-226 ------------------NE-VGVRVT-------AHK-S--------------P-E----
XP_010540046.1/1-196 ------------------NE-VGVRVT-------AHK-S--------------P-E----
XP_019432660.1/1-235 ------------------DE-VVFRVT-------AHK-ST-N--P--------G-Q----
XP_019420678.1/1-233 ------------------NE-IRFRVT-------YHK-GT-D--P--------K-Q----
XP_013452816.1/1-243 ------------------NE-TGVRVT-------VRK-ST-E--L--------G-Q----
XP_020215886.1/1-221 ------------------NE-IGVRVT-------VQ------------------------
XP_020215888.1/1-221 ------------------NE-IGVRVT-------VQ------------------------
XP_007160572.1/1-230 ------------------NE-ILIRVT-------VHK-SS-N--P--------G-Q----
XP_017424661.1/1-212 ------------------NE-ILIRVT-------VHK-SS-N--P--------G-Q----
XP_017424660.1/1-230 ------------------NE-ILIRVT-------VHK-SS-N--P--------G-Q----
XP_014508329.1/1-230 ------------------NE-ILIRVT-------VHK-SS-N--P--------G-Q----
XP_022632295.1/1-230 ------------------NE-ILIRVT-------VHK-SS-N--P--------G-Q----
XP_019225984.1/1-234 ------------------LNEFEVRAT-------LQK-ST-D--P--------G-Q----
XP_016441514.1/1-233 ------------------LNEFEVRAT-------LQK-ST-D--P--------G-Q----
XP_009587298.1/1-233 ------------------LNEFEVRAT-------LQK-ST-D--P--------G-Q----
XP_016441515.1/1-233 ------------------LNEFEVRAT-------LQK-ST-D--P--------G-Q----
XP_009786509.1/1-233 ------------------LNEFEVRAT-------LQK-ST-D--P--------G-Q----
XP_016563952.1/1-234 ------------------LNEFEVRAT-------LQK-ST-D--P--------G-Q----
XP_004235382.1/1-234 ------------------LNEFEVRAT-------LQK-CT-D--P--------G-Q----
XP_015068940.1/1-234 ------------------LNEFEVRAT-------LQK-CT-D--P--------G-Q----
XP_006364383.1/1-234 ------------------LNEFEVRAT-------LQK-CT-D--P--------G-Q----
XP_019167409.1/1-245 ------------------ANEFEVRAT-------LQK-ST-D--P--------G-Q----
XP_011086973.1/1-238 ------------------LNEFEVRAT-------LHK-ST-D--P--------G-Q----
XP_010268321.1/1-230 ------------------LNDIGVRVT-------VHK-ST-E--A--------G-Q----
XP_010268322.1/1-230 ------------------LNDIGVRVT-------VHK-ST-E--A--------G-Q----
XP_010069492.1/1-236 ------------------SSEVGVRVA-------LHK-ST-D--P--------G-Q----
XP_002527933.1/1-229 ------------------SNEVGVR---------VHK-ST-D--P--------G-Q----
XP_021678368.1/1-229 ------------------MNEVGVR---------VHK-ST-D--P--------G-Q----
XP_021678369.1/1-229 ------------------MNEVGVR---------VHK-ST-D--P--------G-Q----
XP_021602738.1/1-229 ------------------MNEVGVR---------VHK-ST-D--P--------G-Q----
XP_012079215.1/1-230 ------------------SNEVGVR---------VHK-ST-D--P--------G-Q----
XP_021687701.1/1-229 ------------------SNEVGVR---------VHK-ST-D--P--------G-Q----
XP_006374212.1/1-231 ------------------LHDVGVRVT-------LHK-ST-D--P--------G-Q----
XP_011035552.1/1-231 ------------------LHDVGVRVT-------LHK-ST-D--P--------G-Q----
XP_002317722.3/1-231 ------------------LHDVGVRIT-------LHK-NT-D--P--------G-Q----
XP_011000766.1/1-231 ------------------LHDVGVRVT-------LHK-ST-D--P--------G-Q----
XP_021907827.1/1-230 ------------------FNEVGVRVS-------AHK-SS-D--P--------G-Q----
XP_017635401.1/1-230 ------------------SNEIGVRVT-------VHK-SS-D--P--------G-Q----
XP_016686699.1/1-230 ------------------SNEVGVRVT-------VHK-SS-D--P--------G-Q----
XP_012440361.1/1-230 ------------------SNEVGVRVT-------VHK-SS-D--P--------G-Q----
NP_001313868.1/1-230 ------------------SNEVGVRVT-------VHK-SS-D--P--------G-Q----
XP_023919022.1/1-230 ------------------LNEVGVRVT-------VHK-ST-D--P--------G-Q----
XP_006478112.1/1-232 ------------------LNEVGVRVT-------LHK-SM-D--P--------G-Q----
XP_024042646.1/1-232 ------------------LNEVGVRVT-------LHK-SM-D--P--------G-Q----
XP_022715403.1/1-231 ------------------LNEVGVRVT-------VHK-KY-D--P--------G-Q----
XP_022767009.1/1-255 ------------------LNEVGVRVT-------VHK-SS-D--P--------G-Q----
XP_022737050.1/1-231 ------------------LNEVGVRVT-------VHK-SS-D--P--------G-Q----
XP_017973835.1/1-231 ------------------LNEVGVRVT-------VHK-SS-D--P--------G-Q----
XP_015967008.1/1-238 ------------------MNEIGVKVT-------VHK-ST-D--P--------G-Q----
XP_025701434.1/1-239 ------------------MNEIGVKVT-------VHK-ST-D--P--------G-Q----
XP_020980110.1/1-239 ------------------MNEIGVKVT-------VHK-ST-D--P--------G-Q----
XP_019434881.1/1-231 ------------------LDEVGVRVT-------VHK-ST-D--P--------G-Q----
XP_019458275.1/1-234 ------------------LDEVGVRVN-------VHK-ST-D--P--------G-Q----
XP_019458276.1/1-234 ------------------LDEVGVRVN-------VHK-ST-D--P--------G-Q----
XP_006584922.1/1-234 ------------------LNEIVVRVT-------VHK-SS-D--P--------G-Q----
XP_003524332.1/1-231 ------------------LNEIVVRVT-------VHK-SS-D--P--------G-Q----
XP_004515249.1/1-229 ------------------LNEIGVRVT-------VRK-ST-D--P--------G-Q----
XP_019431381.1/1-229 ------------------LNEIGVRVI-------VHN-SS-H--P--------G-Q----
XP_019436771.1/1-231 ------------------LNEIGVRVT-------VHN-SS-D--P--------G-Q----
XP_019430342.1/1-233 ------------------MDEIGVRVT-------VHS-SS-D--P--------G-Q----
XP_006587436.1/1-240 ------------------MNEIGVRVT-------VHK-SS-D--P--------G-Q----
XP_003549189.1/1-236 ------------------MNEVGVRVT-------VHK-SS-D--P--------G-Q----
XP_020221986.1/1-232 ------------------MNEIGVRVT-------VHK-ST-D--P--------G-Q----
XP_007152598.1/1-234 ------------------MNEIGVRVT-------VHK-ST-D--P--------G-Q----
XP_017437542.1/1-234 ------------------MNEIGVRVT-------VHK-ST-D--P--------G-Q----
XP_014505752.1/1-234 ------------------MNEIGVRVT-------VHK-ST-D--P--------G-Q----
XP_015881991.1/1-234 ------------------MNDAGVKVT-------VHK-CT-D--P--------G-Q----
XP_004299483.1/1-232 ------------------MNEVGVRVT-------IHK-ST-D--P--------G-Q----
XP_024174647.1/1-232 ------------------MNEVGVRVT-------IHK-ST-D--P--------G-K----
XP_008375041.1/1-227 ------------------TNEIGVRVT-------IHK-IT-D--P--------G-Q----
XP_017188172.1/1-199 ------------------TNEIGVRVT-------IHK-IT-D--P--------G-Q----
XP_009355964.1/1-227 ------------------TNEIGVRVT-------IHK-IT-D--P--------G-Q----
XP_009355965.1/1-199 ------------------TNEIGVRVT-------IHK-IT-D--P--------G-Q----
XP_023523434.1/1-228 ------------------TDEVGVYVS-------VHK-SM-D--P--------G-Q----
XP_022949138.1/1-227 ------------------TDEVGVCVS-------VHK-SM-D--P--------G-Q----
XP_022998050.1/1-227 ------------------TDEVGVCVS-------VHK-SM-D--P--------G-Q----
XP_002271012.1/1-231 ------------------LNEVGVRVT-------IHK-NT-D--P--------G-Q----
XP_010663934.1/1-231 ------------------LNEVGVRVT-------IHK-NT-D--P--------G-Q----
XP_010099679.1/1-237 ------------------LNEVGVRVT-------VHK-SS-D--P--------G-Q----
XP_018823334.1/1-236 ------------------MNEDGVRVS-------VHR-ST-D--P--------G-Q----
XP_023001145.1/1-228 ------------------MNEVGVRVT-------VHK-SM-D--P--------G-Q----
XP_023519889.1/1-228 ------------------MNEVGVRVT-------VHK-SM-D--P--------G-Q----
XP_022924191.1/1-228 ------------------MNEVGVRVT-------VHK-SM-D--P--------G-Q----
XP_018849090.1/1-235 ------------------MNEVGVRVT-------VHK-ST-D--P--------G-Q----
XP_008240378.1/1-228 ------------------MNEVGVRVT-------IHK-ST-D--P--------G-Q----
XP_007210460.2/1-228 ------------------MNEVGVRVT-------IHK-ST-D--P--------G-Q----
XP_021801797.1/1-228 ------------------MNEVGVRVT-------IHK-ST-D--P--------G-Q----
XP_022136988.1/1-228 ------------------MNEVGVRVT-------VHK-SM-D--P--------G-Q----
XP_016898817.1/1-228 ------------------MNEVGVRVT-------VHK-ST-D--S--------G-Q----
XP_004137568.1/1-228 ------------------MNEVGVRVT-------VHK-ST-D--S--------G-Q----
XP_015642098.1/1-238 --------------------EVSVRVT-------MHR-ST-D--P--------G-Q----
XP_020186741.1/1-220 ------------AGV---DDAAGVRVM-------VHR-ST-D--P--------G-Q----
XP_025828617.1/1-229 --------------------DVGVRVM-------VHR-ST-D--P--------G-Q----
XP_004954072.1/1-226 --------------------DVGVRVM-------VHR-ST-D--P--------G-Q----
XP_002452898.1/1-231 --------------------DVGVRVM-------VHR-ST-D--P--------G-Q----
NP_001142912.2/1-230 --------------------DVGVRVT-------VHR-ST-D--P--------G-Q----
XP_020089796.1/1-231 --------------------DVGVRVT-------VHR-SS-D--P--------G-Q----
XP_020089795.1/1-241 --------------------DVGVRVT-------VHR-SS-D--P--------G-Q----
XP_019708739.1/1-228 --------------------DVGVRVT-------VHK-ST-N--L--------G-Q----
XP_008789232.1/1-228 --------------------DVGVRVT-------VHK-ST-N--L--------G-Q----
XP_008789231.1/1-228 --------------------DVGVRVT-------VHK-ST-N--L--------G-Q----
XP_008789233.1/1-200 --------------------DVGVRVT-------VHK-ST-N--L--------G-Q----
XP_019704584.1/1-228 --------------------DVGVRVT-------VHK-ST-N--P--------G-Q----
XP_010917295.1/1-228 --------------------DVGVRVT-------VHK-ST-N--P--------G-Q----
XP_008806630.1/1-228 --------------------DVGVRVT-------VHK-ST-N--P--------G-Q----
XP_009388746.1/1-226 --------------------DVGVRVT-------IHK-TT-D--A--------G-R----
XP_018675467.1/1-228 --------------------DVGVRVT-------LHK-NT-D--A--------G-Q----
XP_018675466.1/1-228 --------------------DVGVRVT-------LHK-NT-D--A--------G-Q----
XP_009409108.1/1-228 --------------------DVGVRVT-------LHK-ST-D--A--------G-Q----
XP_024625408.1/1-220 --------------------EITV-----------------R--K--------S-Q----
XP_010550261.1/1-219 --------------------NSGVRVS-------VRK-SM-E--A--------G-Q----
XP_018816719.1/1-228 --------------------NSGVRVS-------VHK-SR-E--P--------G-Q----
XP_008365756.2/1-218 --------------------NTGIQVS-------VHT-ST-E--I--------G-Q----
XP_008381522.1/1-218 --------------------NTGIQVS-------VHT-ST-E--I--------G-Q----
XP_018506060.1/1-218 --------------------SSGIRVS-------VHK-SA-E--I--------G-Q----
XP_008362571.1/1-218 --------------------SSGVRVS-------VHK-ST-E--I--------G-Q----
XP_023870350.1/1-229 --------------------NSGVRVS-------VRK-RS-E--P--------G-Q----
XP_016649213.1/1-222 --------------------NSGVRVS-------VRK-NT-E--P--------G-Q----
XP_020415996.1/1-222 --------------------NSGVRVS-------VRK-NT-E--P--------G-Q----
XP_021823813.1/1-222 --------------------NSGVRVS-------VRK-NT-E--P--------G-Q----
XP_009367126.1/1-222 --------------------NSGVRVS-------VRK-SA-E--S--------G-Q----
XP_008348615.1/1-222 --------------------NSGVRVS-------VRK-SA-E--S--------G-Q----
XP_017179578.1/1-222 --------------------NSGVRVS-------VRK-ST-E--T--------G-Q----
XP_009370612.1/1-224 --------------------NSGVRVS-------VRK-ST-E--P--------G-Q----
XP_012845089.1/1-228 --------------------NSGVRVS-------LRK-SD-G--Q--------G-Q----
XP_012845088.1/1-228 --------------------NSGVRVS-------LRK-SD-G--Q--------G-Q----
XP_012845087.1/1-228 --------------------NSGVRVS-------LRK-SD-G--Q--------G-Q----
XP_011085930.1/1-227 --------------------NSGVRVS-------LRK-SN-G--L--------G-Q----
XP_020551334.1/1-222 --------------------NSGVRVS-------LRK-SN-G--L--------G-Q----
XP_021297998.1/1-224 --------------------NSGVRVS-------VRQ-SI-E--P--------G-Q----
XP_017974480.1/1-224 --------------------NSGVRVS-------VRQ-SI-E--P--------G-Q----
XP_024448785.1/1-229 --------------------NSGVRVA-------IRK-NT-E--Q--------G-Q----
XP_024455385.1/1-229 --------------------NSGVRVA-------IRK-NT-E--Q--------G-Q----
XP_011005937.1/1-269 --------------------NSGVRVA-------IRK-NT-E--Q--------G-Q----
XP_011038413.1/1-229 --------------------NSGVRVA-------ICK-NA-E--Q--------G-Q----
XP_018731153.1/1-226 --------------------NSGVRVC-------VRK-SE-E--P--------G-Q----
XP_024190515.1/1-228 --------------------NSGVRVN-------VRK-SI-D--P--------G-E----
XP_012074722.2/1-231 --------------------NSGVRVS-------VRR-SG-G--P--------G-Q----
XP_010089514.2/1-229 --------------------NSGVRVS-------VRE-AT-E--T--------G-Q----
XP_024949330.1/1-235 --------------------NSGVRVS-------VRK-SL-G--H--------G-Q----
XP_006421085.1/1-235 --------------------NSGVRVS-------VRK-SL-G--H--------G-Q----
XP_002268272.1/1-229 --------------------TSGVRVS-------VRK-ST-E--P--------G-Q----
XP_004140733.2/1-228 --------------------NSGVRIS-------VRI-SS-E--L--------G-Q----
XP_008457114.1/1-228 --------------------NSGVRIS-------VRI-SS-E--L--------G-Q----
XP_008457113.1/1-229 --------------------NSGVRIS-------VRI-SS-E--L--------G-Q----
XP_011651208.1/1-229 --------------------NSGVRIS-------VRI-SS-E--L--------G-Q----
XP_022143367.1/1-228 --------------------NSGVRIS-------VRI-ST-E--L--------G-Q----
XP_022935490.1/1-228 --------------------NSGVRIS-------VRI-SS-E--L--------G-Q----
XP_022984101.1/1-228 --------------------NSGVRIS-------VRI-SS-E--L--------G-Q----
XP_022935488.1/1-229 --------------------NSGVRIS-------VRI-SS-E--L--------G-Q----
XP_022984098.1/1-229 --------------------NSGVRIS-------VRI-SS-E--L--------G-Q----
XP_022984102.1/1-229 --------------------NSGVRIS-------VRI-SS-E--L--------G-Q----
XP_015579295.1/1-234 --------------------NSGVRVS-------VRK-ST-E--P--------G-Q----
XP_002526595.2/1-234 --------------------NSGVRVS-------VRK-ST-E--P--------G-Q----
XP_015579294.1/1-234 --------------------NSGVRVS-------VRK-ST-E--P--------G-Q----
XP_021643520.1/1-232 --------------------NSGVRVS-------VRK-SM-E--T--------G-Q----
XP_021605433.1/1-231 --------------------NSGVRIS-------VRK-SM-E--L--------G-Q----
XP_023739098.1/1-228 --------------------DFEIHAN-------LYK-ST-D--C--------V-Q----
XP_024966239.1/1-227 --------------------DFEVHMA-------LYK-SS-D--P--------S-Q----
XP_025813558.1/1-232 --------------------DVSIRVA-------AQR-SA-E--P--------G-Q----
XP_020150479.1/1-234 ------------------GNEVCVRVS-------MNQ-GA-Q--P--------G-H----
XP_003564122.2/1-224 -------------------NDMNVRVS-------LHR-VD-E--P--------G-L----
XP_006585049.2/1-225 -------------------NNGEVTII-------VRK-NN-S-EQ--------D-V----
XP_007143783.1/1-226 --------------------NGGVRVA-------VRV-NM-S-EP--------G-E----
XP_007158859.1/1-226 --------------------NSGVRVA-------VRV-NM-S-DP--------G-E----
XP_020230815.1/1-227 --------------------NNRVTIS-------AQK-NT-N--P--------S-Q----
XP_006586851.1/1-227 --------------------NTGIRIC-------ARK-VT-N--S--------N-Q----
XP_021888058.1/1-234 --------------------GTGIRVC-------IRE-GM-G--P--------G-Q----
XP_020886111.1/1-226 --------------------KNGVRVS-------IRM-NN-E--A--------G-Q----
XP_020886110.1/1-228 --------------------KNGVRVS-------IRM-NN-E--A--------G-Q----
XP_011469296.1/1-224 --------------------NSGVRVN-------IRK-SI-E--P--------G-E----
XP_009367128.2/1-218 --------------------DTGIQVS-------VHT-ST-E--I--------G-Q----
XP_008348673.1/1-198 --------------------NNGIQVS-------VHK-ST-E--I--------G-Q----
XP_012832506.1/1-227 --------------------YSGVCVA-------LRK-SD-G--P--------H-Q----
XP_012832508.1/1-227 --------------------YSGVCVA-------LRK-SD-G--P--------H-Q----
XP_012832516.1/1-227 --------------------NSGVCVA-------LRK-SN-G--P--------H-Q----
XP_012832518.1/1-227 --------------------NSGVCVA-------LRK-SN-G--P--------H-Q----
XP_012835569.1/1-227 --------------------NSGVCVA-------LRK-SN-G--P--------H-Q----
XP_012835571.1/1-226 --------------------NSGVCVA-------LRK-SN-G--P--------H-Q----
XP_019172066.1/1-226 --------------------NSGVRIS-------IRK-SA-GETA--------Q-S----
XP_009631364.2/1-226 --------------------NSGVRVS-------FRK-NE-D--I--------S-Q----
XP_016435660.1/1-229 --------------------NSGVRVS-------FRK-NE-D--I--------S-Q----
XP_009771801.1/1-226 --------------------NSGVRVS-------FRK-NE-D--M--------S-Q----
XP_016493115.1/1-226 --------------------NSGVRVS-------FRK-NE-D--M--------S-Q----
XP_019248352.1/1-226 --------------------NSGVRVS-------FRK-NE-D--I--------S-Q----
XP_025691463.1/1-225 --------------------NNGVRVG-------IRR-SI---EA--------G-V----
XP_020995109.1/1-225 --------------------NNGVRVG-------IRR-SI---EA--------G-V----
XP_016190415.2/1-225 --------------------NNGVRVG-------IRR-SI---EA--------G-V----
XP_004486568.1/1-232 --------------------IGGVRVS-------VRK-TT---ST--------G-Q----
XP_024632687.1/1-226 --------------------IGGIRVS-------FRS-TT-DDDT--------S-Q----
XP_020977993.1/1-241 --------------------NSGVRIS-------ARR-CT---EK--------G-Q----
XP_025647253.1/1-241 --------------------NSGVRIS-------ARR-CT---EK--------G-Q----
XP_020997095.1/1-240 --------------------NSGVRIS-------ARR-CT---EK--------G-Q----
XP_025692389.1/1-228 --------------------NSGVRIS-------ARR-CT---EK--------G-Q----
XP_019418389.1/1-226 --------------------NGGIKVS-------CRK-SI---EP--------G-Q----
XP_007147460.1/1-227 --------------------TSGVRVS-------IRE-SK---MI--------G-Q----
XP_014517364.1/1-227 --------------------TSGVRVS-------IRE-SK---IN--------G-Q----
XP_017435438.1/1-226 --------------------TSGVRVS-------IRE-SK---IN--------G-Q----
XP_017435439.1/1-217 --------------------TSGVRVS-------IRE-SK---IN--------G-Q----
XP_025981664.1/1-227 --------------------NSGVRVS-------IRK-NR-N-HL--------G-Q----
XP_006586853.1/1-227 --------------------NSGLRVS-------IRK-NR-N-HL--------G-Q----
XP_020230831.1/1-226 --------------------NSGVRVS-------IRK-NT---DL--------G-Q----
XP_004489663.1/1-217 --------------------IGGIRVS-------FQD-IT-S--S--------G-Q----
XP_018489833.1/1-227 ---------------------CGVRIS-------IRM-NM-E--A--------G-Q----
XP_013584487.1/1-219 ---------------------CGVRIS-------IRM-NM-E--P--------G-Q----
XP_009130351.1/1-219 ---------------------CGVRIS-------IRM-NK-E--P--------G-Q----
XP_013747906.2/1-219 ---------------------CGVRIS-------IRM-NK-E--P--------G-Q----
XP_013747661.1/1-219 ---------------------CGVRIS-------IRM-NK-E--P--------G-Q----
XP_013619678.1/1-219 ---------------------CGVRIS-------IRM-NM-E--P--------G-Q----
XP_022551084.1/1-219 ---------------------CGVRIS-------IRM-NM-E--P--------G-Q----
XP_009126392.1/1-219 ---------------------GGVRIL-------IRM-NM-K--P--------G-Q----
XP_018437612.1/1-222 ---------------------CGVRIS-------IRM-NN-E--A--------G-Q----
XP_013591191.1/1-225 ---------------------CGVRIS-------IRM-NH-D--S--------G-Q----
XP_022567005.1/1-225 ---------------------CGVRIS-------IRM-NH-D--S--------G-Q----
XP_022547796.1/1-225 ---------------------CGVRIS-------IRM-NH-D--S--------G-Q----
XP_009119205.1/1-225 ---------------------CGVRIS-------IRM-NH-D--S--------G-Q----
XP_009119199.1/1-225 ---------------------CGVRIS-------IRM-NH-D--S--------G-Q----
XP_006408322.1/1-232 ---------------------CGVRIS-------IRM-NM-E--P--------G-Q----
XP_023642295.1/1-229 ---------------------CGVRVS-------IRL-NQ-E--Q--------G-Q----
XP_023642294.1/1-229 ---------------------CGVRVS-------IRL-NQ-E--Q--------G-Q----
NP_186976.2/1-228    ---------------------NGVRVS-------IRM-NI-E--A--------G-Q----
XP_010507998.1/1-229 ---------------------CGVRVS-------MRI-NL-E--A--------G-Q----
XP_010485661.1/1-229 ---------------------CGVRVS-------MRM-NL-E--A--------G-Q----
XP_010450732.1/1-230 ---------------------CGVRVS-------MRM-NL-E--A--------G-Q----
XP_010463776.1/1-230 ---------------------CGVRVS-------MRM-NL-E--A--------G-Q----
XP_010463774.1/1-230 ---------------------CGVRVS-------MRM-NL-E--A--------G-Q----
XP_019092960.1/1-229 ---------------------CGVRVS-------MRM-NL-E--A--------G-Q----
XP_022551077.1/1-228 --------------------SSGVRVS-------VRV-NS-E--F--------G-Q----
XP_013619236.1/1-228 --------------------SSGVRVS-------VRV-NS-E--F--------G-Q----
XP_009126177.1/1-226 --------------------SSGVRVS-------VRV-NS-E--A--------G-Q----
XP_013652421.1/1-226 --------------------SSGVRVS-------VRV-NS-E--A--------G-Q----
XP_013652420.1/1-226 --------------------SSGVRVS-------VRV-NS-E--A--------G-Q----
XP_006400265.1/1-228 --------------------SSGVRVS-------VRV-NN-E--A--------G-Q----
XP_013590801.1/1-226 --------------------SSGVNIS-------VRV-NK-E--A--------G-Q----
XP_013651910.1/1-222 --------------------SSGVNIS-------VRV-NK-E--A--------G-Q----
XP_009103958.1/1-222 --------------------SSGVNIS-------VRV-NK-E--A--------G-Q----
XP_018444485.1/1-226 --------------------SSGIRIA-------VRV-NE-E--V--------G-Q----
XP_009121397.1/1-226 --------------------SSGVRIS-------VRV-NE-E--V--------G-Q----
XP_013659428.1/1-226 --------------------SSGVRIS-------VRV-NE-E--V--------G-Q----
XP_022565333.1/1-226 --------------------SSGVRIS-------VRV-NE-E--V--------G-Q----
XP_013610327.1/1-226 --------------------SSGVRIS-------VRV-NE-E--V--------G-Q----
XP_006286655.1/1-225 --------------------NSGVRIS-------VRT-ND-A--A--------G-E----
XP_010492677.1/1-224 --------------------NSGVTIA-------MRL-NH-E--A--------G-Q----
XP_010420445.1/1-224 --------------------SSGVTIA-------MRL-NN-E--A--------G-Q----
XP_010453918.1/1-224 --------------------NSGVSIA-------VRL-NN-E--A--------G-Q----
XP_006303739.1/1-224 --------------------NSKIRIS-------VRT-NT-Q--A--------G-Q----
XP_020876074.1/1-222 --------------------NSGVRIS-------VRI-NN-E--A--------G-Q----
NP_197234.1/1-222    --------------------NSGIRIG-------VRI-NN-E--A--------G-Q----
NP_174724.1/1-221    --------------------NSGVRIS-------VRT-NT-E--A--------G-Q----
XP_020869876.1/1-222 --------------------NSGVRIS-------VRK-NT-E--A--------G-Q----
XP_019091098.1/1-222 --------------------NSGVRIS-------VRT-NI-E--A--------G-Q----
XP_010478910.1/1-223 --------------------NSGVRIS-------VRT-NI-E--A--------G-Q----
XP_010501781.1/1-221 ---------------------SGVRIS-------VRT-NI-E--A--------G-Q----
XP_015089790.1/1-222 --------------------NNTYRVS-------IRE-NE-E--T--------T-Q----
XP_015089784.1/1-222 --------------------NNTYRVS-------IRE-NE-E--T--------T-Q----
XP_016549855.1/1-224 --------------------NSGDRVS-------IRT-NE-E--I--------TLS----
XP_015057403.1/1-221 --------------------SNGDRIS-------IRK-NE-E--I--------T-Q----
XP_019066399.1/1-221 --------------------SSGNRIS-------IRK-NE-E--I--------T-Q----
XP_015166059.1/1-216 --------------------NRGDRIS-------IRK-NE-E--I--------V-Q----
XP_019071012.2/1-216 --------------------NRGDRIS-------IRI-NE-E--I--------I-Q----
XP_015086709.1/1-216 --------------------NRGDRIS-------IRI-NE-E--I--------I-Q----
XP_015087432.1/1-219 --------------------NRVDRIS-------VRE-SE-E--I--------I-E----
XP_004247342.2/1-219 --------------------NRVDRIS-------VRE-NE-E--I--------I-E----
XP_025888717.1/1-219 --------------------NRVDRIS-------VRE-NE-E--I--------I-E----
XP_015087599.1/1-221 --------------------NRGDRIS-------IRK-NE-E--I--------T-Q----
XP_019071156.1/1-221 --------------------NRGDRIS-------IRK-NE-E--I--------T-Q----
XP_019070762.2/1-220 --------------------NRGDRIS-------IVK-NE-E--T--------I-Q----
XP_015087071.1/1-220 --------------------NRGDRIS-------IRK-NE-E--T--------I-Q----
XP_025888845.1/1-217 --------------------NRGDRIS-------IRK-NE-E--T--------I-Q----
XP_015170367.1/1-232 --------------------NSEDRIS-------IRK-NE-E--F--------T-Q----
XP_025886268.1/1-232 --------------------KSEDRVS-------IRK-NE-D--I--------T-Q----
XP_015072491.1/1-224 --------------------KSEDRVS-------IRK-NE-D--I--------T-Q----
XP_010327374.2/1-219 --------------------NNEDRVS-------IRK-NK-E--T--------T-Q----
XP_015089749.1/1-219 --------------------NNEDRVS-------IRK-NK-E--T--------T-Q----
XP_015170971.1/1-209 --------------------NSGDRVS-------IRK-NI-E--I--------T-Q----
XP_025887726.1/1-223 --------------------NCGERVS-------VRK-NL-E--I--------T-Q----
XP_006586358.1/1-227 --------------------NNGVKLS-------IRV-NH-T--G--------PNE----
XP_014617351.1/1-228 --------------------NTGIRIC-------ARK-VT-N--S--------NQS----
XP_007147461.1/1-227 --------------------NINIRIT-------TRK-YT-N--S--------SLS----
XP_017435408.1/1-226 --------------------NTNIMIH-------ARK-YT-N--S--------SQS----
XP_017215828.1/1-223 -----------------TG-E-DIRIA-------WRK-NL--SDP--------K-E----
XP_012856110.1/1-229 -----------------TG-D-DIRVS-------LRN-NL--NEQ--------G-E----
XP_023738891.1/1-226 -----------------TG-D-DIRVA-------SRK-NL--NDP--------G-E----
XP_022017964.1/1-227 -----------------TG-Y-DIRVA-------SRM-NL--NDP--------G-E----
XP_022154996.1/1-226 -----------------TG-E-NIRVA-------SRK-NL--NDP--------R-E----
XP_008462169.1/1-225 -----------------IG-E-TIRIA-------SRK-NL--KNP--------H-E----
XP_004141841.1/1-226 -----------------IG-E-TIRIA-------SRK-NL--KNP--------H-E----
XP_020098514.1/1-228 -----------------GG-E-DIRVT-------TRK-NI--NEP--------G-E----
XP_020098513.1/1-228 -----------------GG-E-DIRVT-------TRK-NI--NEP--------G-E----
XP_020263950.1/1-229 -----------------SG-D-DISFT-------SRK-NI--SDP--------G-E----
XP_020263948.1/1-255 -----------------SG-D-DISFT-------SRK-NI--SDP--------G-E----
XP_009415234.2/1-232 -----------------GG-D-EIRFT-------SRK-NI--NDP--------G-E----
XP_008789333.1/1-230 -----------------GG-E-DIRFT-------SRK-NV--NDP--------G-E----
XP_010920420.1/1-235 -----------------SG-E-DIRFT-------SRK-NV--NDP--------G-E----
XP_010920411.1/1-235 -----------------SG-E-DIRFT-------SRK-NV--NDP--------G-E----
XP_010920404.1/1-235 -----------------SG-E-DIRFT-------SRK-NV--NDP--------G-E----
XP_010521515.1/1-219 -----------------TG-E-DIRVS-------SRK-NL--NDP--------G-E----
XP_013652056.1/1-227 -----------------TG-Q-DMRVS-------SRK-NL--HDP--------G-E----
XP_009104428.1/1-227 -----------------TG-Q-DMRVS-------SRK-NL--HDP--------G-E----
XP_018445476.1/1-226 -----------------TG-Q-DMRVS-------SRK-NL--HDP--------G-E----
XP_013590784.1/1-226 -----------------TG-Q-DMRVS-------SRK-NL--HDP--------G-E----
XP_013680822.1/1-226 -----------------TG-Q-DMRVS-------SRK-NL--HDP--------G-E----
XP_006389720.2/1-226 -----------------TG-Q-DMRVS-------SRK-NL--HDP--------G-E----
XP_023642973.1/1-226 -----------------TG-Q-DMRVS-------SRK-NL--HDP--------G-E----
XP_020891640.1/1-226 -----------------TG-Q-DMRVS-------SRK-NL--HDP--------S-E----
NP_001320267.1/1-226 -----------------TG-Q-DMRVS-------SRK-NL--HDP--------G-E----
NP_565223.1/1-226    -----------------TG-Q-DMRVS-------SRK-NL--HDP--------G-E----
NP_001185443.1/1-226 -----------------TG-Q-DMRVS-------SRK-NL--HDP--------G-E----
XP_010473021.1/1-226 -----------------TG-Q-DMRVS-------SRK-NL--HDP--------G-E----
XP_010430051.1/1-226 -----------------TG-Q-DMRVS-------SRK-NL--HDP--------G-E----
XP_025617667.1/1-241 -----------------NG-E-DIRIS-------SRK-NL--NDP--------G-Q----
XP_015938429.1/1-242 -----------------NG-E-DIRIS-------SRK-NL--NDP--------G-Q----
XP_016176999.1/1-241 -----------------NG-E-DIRIS-------SRK-NL--NDP--------G-Q----
XP_021862846.1/1-225 -----------------TG-E-DVRVA-------SRK-NL--NDP--------C-E----
XP_022979290.1/1-231 -----------------TG-E-DIRVC-------SRK-NP--SDL--------G-E----
XP_022955725.1/1-233 -----------------TG-E-DIRVC-------SRK-NL--SDL--------G-E----
XP_022998610.1/1-231 -----------------TG-E-DIRVC-------SRK-NL--SDP--------G-E----
XP_022998611.1/1-227 -----------------TG-E-DIRVC-------SRK-NL--SDP--------G-E----
XP_023523626.1/1-229 -----------------TG-E-DIRVC-------SRK-NL--SDP--------G-E----
XP_022948296.1/1-231 -----------------TG-E-DIRVC-------SRK-NL--SDP--------G-E----
XP_023523628.1/1-225 -----------------TG-E-DIRVC-------SRK-NL--SDP--------G-E----
XP_022948295.1/1-227 -----------------TG-E-DIRVC-------SRK-NL--SDP--------G-E----
XP_010249693.1/1-228 -----------------GG-E-DIRVA-------SRK-NL--NDP--------A-E----
XP_010254081.1/1-230 -----------------GG-D-DIRVA-------SRK-NL--NDP--------A-E----
XP_003529763.1/1-227 -----------------TG-E-DIRIS-------SRK-NL--NEP--------G-E----
XP_003533048.1/1-226 -----------------TG-E-DIRIS-------SRK-NL--NDP--------G-E----
XP_014634625.1/1-226 -----------------TG-E-DIRIS-------SRK-NL--NDP--------G-E----
XP_007150635.1/1-226 -----------------NG-E-DIRIS-------SRK-NL--NDP--------G-E----
XP_014498860.1/1-226 -----------------NG-E-DIRIS-------SRK-NL--NDP--------G-E----
XP_017425661.1/1-226 -----------------NG-E-DIRIS-------SRK-NL--NDP--------G-E----
XP_020207725.1/1-226 -----------------TG-E-DIRIS-------SRK-NL--NDP--------G-E----
XP_003546423.2/1-226 -----------------TG-E-DIRIS-------SRK-NL--NDP--------G-E----
XP_003543638.1/1-226 -----------------TG-E-DIRIS-------SRK-NL--NDP--------G-E----
XP_019457980.1/1-226 -----------------TG-E-DIRIS-------FRK-NL--NDP--------G-E----
XP_003597736.1/1-226 -----------------TG-E-DIRIS-------SRK-NL--NDP--------S-E----
XP_004486751.1/1-226 -----------------TG-E-DIRVS-------SRK-NL--NDP--------G-E----
XP_016169615.1/1-226 -----------------TG-E-DIRIS-------SRK-NL--NDP--------G-E----
XP_025611006.1/1-226 -----------------TG-E-DIRIS-------SRK-NL--NDP--------G-E----
XP_020982478.1/1-227 -----------------TG-E-DIRIS-------SRK-NL--NDP--------G-E----
XP_021892835.1/1-225 -----------------TG-E-DVRVS-------SRK-NL--NDP--------G-E----
XP_012442963.1/1-227 -----------------TG-E-DIRVS-------SRK-NL--NDP--------G-E----
NP_001314068.1/1-227 -----------------TG-E-DIRVS-------SRK-NL--NDP--------G-E----
XP_016684379.1/1-227 -----------------TG-E-DIRVS-------SRK-NL--NDP--------G-E----
XP_016730082.1/1-227 -----------------TG-E-DIRVS-------SRK-NL--NDP--------G-E----
NP_001316938.1/1-227 -----------------TG-E-DVRVS-------SRK-NL--NDP--------G-E----
XP_017606636.1/1-227 -----------------TG-E-DIRVS-------SRK-NL--NDP--------G-E----
XP_022714992.1/1-229 -----------------TG-E-DIRVS-------SRK-NL--NDP--------G-E----
XP_022721436.1/1-227 -----------------TG-E-DIRVS-------SRK-NL--SDP--------G-E----
XP_022721435.1/1-227 -----------------TG-E-DIRVS-------SRK-NL--SDP--------G-E----
XP_007024189.1/1-227 -----------------TG-E-DIRVS-------SRK-NL--NDP--------G-E----
XP_021298731.1/1-227 -----------------TG-E-DIRVS-------SRK-NL--NDP--------G-E----
XP_004302894.1/1-228 -----------------TG-D-DIRIA-------SRK-NL--NDP--------G-E----
XP_011466687.1/1-235 -----------------TG-D-DIRIA-------SRK-NL--NDP--------G-E----
XP_024180978.1/1-228 -----------------TG-D-DIRIS-------SRK-NL--NDP--------G-E----
XP_024180977.1/1-235 -----------------TG-D-DIRIS-------SRK-NL--NDP--------G-E----
XP_009353605.1/1-226 -----------------TG-D-DIRIA-------SRK-NL--NDP--------G-E----
XP_008380909.1/1-226 -----------------TG-D-DIRIA-------SRK-NL--NDP--------G-E----
NP_001280927.1/1-227 -----------------TG-D-DIRIA-------SRK-NS--NDP--------G-E----
XP_009338062.1/1-226 -----------------TG-D-DIRIA-------SRK-NL--NDP--------G-E----
XP_009338046.1/1-226 -----------------TG-D-DIRIA-------SRK-NL--NDP--------G-E----
XP_021829136.1/1-226 -----------------TG-D-DIRIA-------SRK-NL--NDP--------G-E----
XP_008228560.1/1-226 -----------------TG-D-DIRIA-------SRK-NL--NDP--------G-E----
XP_007217160.1/1-226 -----------------TG-D-DIRIA-------SRK-NL--NDP--------G-E----
XP_022891611.1/1-228 -----------------TG-D-DIRVA-------SRK-NI--NDP--------G-E----
XP_002304207.2/1-228 -----------------TG-E-DIRIS-------SRK-NL--NEP--------G-E----
XP_011010522.1/1-228 -----------------TG-E-DIRIS-------SRK-NL--NDP--------G-E----
XP_011009828.1/1-227 -----------------TG-E-DIRVS-------SRK-NL--NDP--------G-E----
XP_024462179.1/1-228 -----------------TG-E-DIRVS-------SRK-NL--NDP--------G-E----
XP_018837483.1/1-226 -----------------TG-E-DIRVS-------SRK-NL--NDP--------G-E----
XP_018856342.1/1-226 -----------------TG-E-DIRIS-------SRK-NL--NDP--------G-E----
XP_018856994.1/1-226 -----------------TG-E-DIRIS-------SRK-NL--NDP--------G-E----
XP_018858360.1/1-226 -----------------TG-E-DIRIS-------SRK-NL--NDP--------G-E----
XP_015867693.1/1-226 -----------------TG-E-DIRIA-------SRK-NL--NDP--------G-E----
XP_010062490.1/1-226 -----------------TG-E-DIRVA-------SRK-NL--NDP--------G-E----
XP_010098082.1/1-228 -----------------TG-E-DIRIA-------SRK-NL--NDP--------G-E----
XP_002284502.1/1-228 -----------------TG-D-DIRVA-------SRK-NL--NDP--------G-E----
XP_023875609.1/1-226 -----------------TG-E-DIRIT-------SRK-NL--NDP--------G-E----
XP_002516023.1/1-226 -----------------TG-E-DIRIS-------SRK-NL--NDP--------A-E----
XP_021652755.1/1-228 -----------------TG-D-DIRFS-------SRK-NL--NDP--------S-E----
XP_021654904.1/1-226 -----------------TG-D-DIRLS-------SRK-NL--NDP--------S-E----
XP_021611342.1/1-229 -----------------TG-D-DIRVS-------SRK-NL--NDP--------S-E----
XP_012073376.1/1-226 -----------------TG-E-DIRVS-------SRK-NL--NDP--------G-E----
XP_006465622.1/1-226 -----------------TG-E-DIRVS-------SRK-NL--NDP--------G-E----
XP_006426950.1/1-226 -----------------TG-E-DIRVS-------SRK-NL--NDP--------G-E----
XP_009392419.1/1-229 -----------------SG-D-EIRFT-------SRK-NM--NDP--------G-E----
XP_020518333.1/1-227 -----------------GGPN-DIRAT-------SRK-NV--NDP--------G-E----
XP_018629167.1/1-227 -----------------TG-D-DIRVA-------SRK-NL--TDP--------R-E----
XP_009611250.1/1-227 -----------------TG-D-DIRVA-------SRK-NL--TDP--------R-E----
XP_009611249.1/1-227 -----------------TG-D-DIRVA-------SRK-NL--TDP--------R-E----
XP_009611248.1/1-227 -----------------TG-D-DIRVA-------SRK-NL--TDP--------R-E----
XP_016479156.1/1-227 -----------------TG-D-DIRVA-------SRK-NL--TDP--------R-E----
XP_016479155.1/1-227 -----------------TG-D-DIRVA-------SRK-NL--TDP--------R-E----
XP_016479154.1/1-227 -----------------TG-D-DIRVA-------SRK-NL--TDP--------R-E----
XP_009778213.1/1-229 -----------------TGQE-DIRVA-------SRK-NL--TDT--------G-E----
XP_016501674.1/1-229 -----------------TGQE-DIRVA-------SRK-NL--TDT--------G-E----
XP_019224885.1/1-229 -----------------TGQE-DIRVA-------SRK-NL--TDT--------G-E----
XP_019224886.1/1-229 -----------------TGQE-DIRVA-------SRK-NL--TDT--------G-E----
XP_009592772.1/1-229 -----------------TGQE-DIRVA-------SRK-NL--TDT--------G-E----
XP_016496403.1/1-229 -----------------TGQE-DIRVA-------SRK-NL--TDT--------G-E----
XP_016562690.1/1-228 -----------------TGQE-DIRVA-------SRK-NL--TDP--------G-E----
XP_006343080.1/1-229 -----------------TGQE-DIRMT-------SRR-NL--TDP--------G-E----
XP_004235676.1/1-229 -----------------TGQE-DIRVI-------SRR-NL--TDP--------G-E----
XP_020227921.1/1-228 -----------------TG-E-DIRIS-------SRK-NL--NDP--------G-E----
XP_007135673.1/1-226 -----------------TG-E-DIRIS-------SRK-NL--NDP--------G-E----
XP_014515030.1/1-226 -----------------TG-E-DIRIS-------SRK-NL--NDP--------G-E----
XP_017407449.1/1-226 -----------------TG-E-DIRIS-------SRK-NL--NDP--------G-E----
XP_010691342.1/1-224 -----------------TG-E-DVRVA-------SRK-NL--NDP--------G-E----
XP_021731998.1/1-222 -----------------TG-E-DVRVS-------SRK-NL--NDP--------C-E----
XP_021736870.1/1-224 -----------------TG-E-DVRVS-------SRK-NL--NDP--------C-E----
XP_024969955.1/1-229 -----------------TG-D-DIRVA-------SRK-NL--NDP--------G-E----
XP_024969954.1/1-229 -----------------TG-D-DIRVA-------SRK-NL--NDP--------G-E----
XP_024969953.1/1-229 -----------------TG-D-DIRVA-------SRK-NL--NDP--------G-E----
XP_021984354.1/1-227 -----------------TG-Y-DIRVA-------SRK-NL--NDA--------G-E----
XP_017232370.1/1-228 -----------------NG-E-DIRIA-------SRK-NF--NDP--------G-E----
XP_017216573.1/1-228 -----------------TG-E-DIRIA-------WRK-NL--SDP--------G-E----
XP_017248777.1/1-227 -----------------SG-D-DIRIS-------SRK-NL--NDP--------G-E----
XP_021627148.1/1-226 -----------------TG-D-DIRVS-------SRK-NL--NDP--------S-E----
XP_021627146.1/1-226 -----------------TG-D-DIRVS-------SRK-NL--NDP--------S-E----
XP_021627145.1/1-226 -----------------TG-D-DIRVS-------SRK-NL--NDP--------S-E----
XP_019196965.1/1-228 -----------------TG-D-DIRVA-------SRK-NL--SDP--------G-E----
XP_019196967.1/1-228 -----------------TG-D-DIRVA-------SRK-NL--SDP--------G-E----
XP_022848789.1/1-228 -----------------TG-D-DIRVA-------SRK-NI--NDP--------G-E----
XP_011072373.1/1-228 -----------------TG-E-EIRVA-------SRK-NL--NDT--------G-E----
XP_021907286.1/1-215 ----------------GSGID-DVKIA-------TRS-NV--DEP--------G-R----
XP_020885309.1/1-217 ----------------GVAGE-DIRVM-------TMK-SV--NDL--------G-K----
XP_020885307.1/1-217 ----------------GVAGE-DIRVM-------TMK-SV--NDL--------G-K----
NP_180796.2/1-218    ----------------GVEGE-DIRVM-------TMK-SV--NDP--------G-K----
XP_006296541.1/1-217 ----------------GVAGD-DIRVM-------TMK-NM--NDP--------G-R----
XP_010510028.1/1-215 ----------------GAGGD-DIRVM-------TMK-NI--NDP--------G-R----
XP_010414006.1/1-214 ----------------GVGGD-DIRVM-------TMK-NI--NDP--------G-R----
XP_010469602.1/1-219 ----------------GVGGD-DIRMM-------TMK-NI--NDP--------G-R----
XP_010417818.1/1-218 ----------------GVGGK-DIRVM-------KMK-NL--IEP--------G-R----
XP_010510869.1/1-220 ----------------GVGGK-GIRVM-------KMK-NL--NGP--------G-R----
XP_010510870.1/1-218 ----------------GVGGK-GIRVM-------KMK-NL--NGP--------G-R----
XP_018484769.1/1-219 ----------------GNTGN-PVRVM-------ARK-SF--NDP--------G-R----
XP_018484754.1/1-219 ----------------GNTGN-PVRVM-------ARK-SF--NDP--------G-R----
XP_013634882.1/1-219 ----------------GNAGS-TVRAM-------TRK-SL--NDP--------G-R----
XP_018513778.1/1-219 ----------------GNGGN-TVRAM-------TRK-SL--NDP--------G-R----
XP_009141217.1/1-219 ----------------GNGGN-TVRAM-------TRK-SL--NDP--------G-R----
XP_013747219.1/1-219 ----------------GNGGN-TVRAM-------TRK-SL--NDP--------G-R----
XP_024003849.1/1-217 ----------------GYAGD-SIRLM-------TRT-SV--NDP--------G-R----
XP_018437853.1/1-211 ----------------DYTGE-RVRVM-------TRK-SM--NDP--------G-K----
XP_009143975.1/1-211 ----------------GYTGD-SVKVT-------TRT-SL--NDP--------G-R----
XP_009143974.1/1-211 ----------------GYTGD-SVKVT-------TRT-SL--NDP--------G-R----
XP_022575029.1/1-211 ----------------DYTGD-SVKVT-------TRT-SL--NDP--------G-R----
XP_013748684.1/1-211 ----------------DYTGD-SVKVT-------TRT-SL--NDP--------G-R----
XP_013694194.1/1-211 ----------------DYTGD-SVKVT-------TRT-SL--NDP--------G-R----
XP_013634261.1/1-211 ----------------DYTGD-SVSVM-------TRK-SL--NDP--------G-R----
XP_013741430.1/1-210 ----------------GYTGD-SVKVT-------TRT-SL--NDP--------G-R----
XP_017254373.1/1-221 ----------------ESAHG-TARIM-------TKN-NF--SDP--------G-T----
XP_017254240.1/1-222 ----------------GDGAT-KAKIM-------AKK-NT--DDP--------G-M----
XP_017221582.1/1-224 ----------------GEGAT-DAKIM-------TRK-NT--DDP--------R-T----
XP_017221584.1/1-224 ----------------GDGAT-DAKIM-------TRK-NT--DDP--------R-T----
XP_015941575.1/1-215 ----------------PSTNH-SNIRI-------ITR-KV-EDDP--------G-R----
XP_025620358.1/1-215 ----------------PSTNH-SNIRI-------ITR-KV-EDDP--------G-R----
XP_016176493.1/1-215 ----------------PSTNH-SNIRI-------ITR-KV-EDDP--------G-R----
XP_003610661.2/1-228 ----------------GNGPE-VVMVM-------TKR-YIDESSI--------D-K----
XP_004496853.1/1-220 ----------------SNSND-V-RVM-------IKR-SIREPGT--------N-T----
XP_006353342.1/1-230 ----------------SGTAE-ESKLI-------MRK-GV--GEP--------G-D----
XP_015070591.1/1-229 ----------------LGTAE-ESKFI-------MRK-GV--GEP--------G-D----
XP_019068356.1/1-229 ----------------LGTAE-ESKFI-------MRK-GV--GEP--------G-D----
XP_004234441.1/1-229 ----------------LGTAE-ESKFI-------MRK-GV--GEP--------G-D----
XP_024926606.1/1-217 ----------------LSCAE-GIKVM-------AKN-SM--DDP--------G-K----
XP_024525400.1/1-231 ----------------GSVHD-DIRVM-------TRK-SV--DNP--------G-E----
XP_002965186.2/1-231 ----------------GSVHD-DIRVM-------TRK-SV--DNP--------G-E----
XP_002962272.2/1-231 ----------------GSVHD-DIRVM-------TRK-SV--DNP--------G-E----
XP_019441536.1/1-226 -----------------PPCD-SVRIM-------RRT-SM--NDP--------G-R----
XP_019441537.1/1-226 -----------------PPCD-SVRIM-------RRT-SM--NDP--------G-R----
XP_019441535.1/1-226 -----------------PPCD-SVRIM-------RRT-SM--NDP--------G-R----
XP_019451758.1/1-229 -----------------PHYD-GVRVM-------SAT-NI--NDP--------G-R----
XP_012850835.1/1-238 ----------------GIGND-SVKVI-------TRK-NV--DDP--------T-M----
XP_020552079.1/1-225 ----------------GNGSE-NVRVM-------TRN-NM--DDL--------S-M----
XP_020552078.1/1-225 ----------------GNGSE-NVRVM-------TRN-NM--DDL--------S-M----
XP_020552080.1/1-223 ----------------GNGSE-NVRVM-------TRN-NM--DDL--------S-M----
XP_009408807.2/1-226 ----------------GNVAD-HVRIR-------SRN-ND--GDP--------G-R----
XP_006342404.1/1-225 ----------------GYNTN-DVQVM-------TRQ-SI--NDP--------G-R----
XP_025887998.1/1-218 ----------------GYNIN-DLQVK-------TRR-SI--NDP--------G-R----
XP_015081481.1/1-225 ----------------GYNIN-DLQVM-------TRQ-SI--NDP--------G-R----
XP_022000230.1/1-232 -----------------DSSE-AAKIM-------IRK-SL--NNP--------G-D----
XP_022000229.1/1-232 -----------------DSSE-AAKIM-------IRK-SL--NNP--------G-D----
XP_022037790.1/1-222 -----------------NEPE-AARIL-------IRK-SL--NNL--------G-D----
XP_022037789.1/1-222 -----------------NEPE-AARIL-------IRK-SL--NNL--------G-D----
XP_024991925.1/1-226 -----------------NAAE-AAKIM-------IRK-SL--NNP--------G-D----
XP_023760005.1/1-229 -----------------NGAE-AAKIM-------IRK-SL--NNH--------G-D----
XP_023760001.1/1-229 -----------------NGAE-AAKIM-------IRK-SL--NNH--------G-D----
XP_017251723.1/1-236 -----------------NMGD-DSRFL-------VRK-GI--PDP--------G-E----
XP_017243321.1/1-239 -----------------NVGE-GARLM-------MRK-NV--HSL--------G-E----
XP_017243319.1/1-239 -----------------NVGE-GARLM-------MRK-NV--HSL--------G-E----
XP_017243320.1/1-239 -----------------NVGE-GARLM-------MRK-NV--HSL--------G-E----
XP_017255563.1/1-234 -----------------NMGE-DARLM-------IRK-GV--NGP--------G-E----
XP_017255562.1/1-234 -----------------NMGE-DARLM-------IRK-GV--NGP--------G-E----
XP_012852237.1/1-226 -----------------E----NMRIM-------VRK-SM--GNS--------G-E----
XP_012852238.1/1-226 -----------------E----NMRIM-------VRK-SM--GNS--------G-E----
XP_012852236.1/1-226 -----------------E----NMRIM-------VRK-SM--GNS--------G-E----
XP_012852235.1/1-226 -----------------E----NMRIM-------VRK-SM--GNS--------G-E----
XP_011076208.1/1-227 -----------------D----NTKLM-------MRK-SM--GNP--------G-E----
XP_019200484.1/1-228 -----------------NGDD-AARLN-------MRK-SV--GEP--------G-E----
XP_019200481.1/1-228 -----------------NGDD-AARLN-------MRK-SV--GEP--------G-E----
XP_022879034.1/1-223 -----------------NADN-T-KLM-------MRE-SI--GNP--------G-E----
XP_018623115.1/1-238 -----------------NGED-A-KLM-------MRK-NI--GDP--------G-E----
XP_018623116.1/1-238 -----------------NGED-A-KLM-------MRK-NI--GDP--------G-E----
XP_016449095.1/1-238 -----------------NGED-A-KLM-------MRK-NI--GDP--------G-E----
XP_009771835.1/1-238 -----------------NGED-A-KLM-------MRK-NI--GDP--------G-E----
XP_009771833.1/1-238 -----------------NGED-A-KLM-------MRK-NI--GDP--------G-E----
XP_019248454.1/1-225 -----------------NGED-A-KLM-------MRK-NI--GDP--------G-E----
XP_016564677.1/1-235 -----------------SGEE-T-KLM-------MRK-SV--GEL--------G-E----
XP_016564676.1/1-235 -----------------SGEE-T-KLM-------MRK-SV--GEL--------G-E----
XP_009606534.1/1-237 -----------------TAED-T-KLM-------MRK-SI--GDP--------G-E----
XP_016457239.1/1-237 -----------------TAED-T-KLM-------MRK-SI--GDP--------G-E----
XP_009606533.1/1-237 -----------------TAED-T-KLM-------MRK-SI--GDP--------G-E----
XP_009606532.1/1-237 -----------------TAED-T-KLM-------MRK-SI--GDP--------G-E----
XP_016457238.1/1-237 -----------------TAED-T-KLM-------MRK-SI--GDP--------G-E----
XP_016468064.1/1-237 -----------------TAED-T-KLM-------MRK-SI--GEP--------G-E----
XP_009774404.1/1-237 -----------------TAED-T-KLM-------MRK-SI--GEP--------G-E----
XP_016468062.1/1-237 -----------------TAED-T-KLM-------MRK-SI--GEP--------G-E----
XP_016468061.1/1-237 -----------------TAED-T-KLM-------MRK-SI--GEP--------G-E----
XP_009774402.1/1-237 -----------------TAED-T-KLM-------MRK-SI--GEP--------G-E----
XP_009774401.1/1-237 -----------------TAED-T-KLM-------MRK-SI--GEP--------G-E----
XP_019251817.1/1-237 -----------------TAED-T-KLM-------MRK-SI--GDP--------G-E----
XP_019251815.1/1-237 -----------------TAED-T-KLM-------MRK-SI--GDP--------G-E----
XP_019251816.1/1-237 -----------------TAED-T-KLM-------MRK-SI--GDP--------G-E----
XP_011470395.1/1-233 -----------------NVSD-NVRVM-------VRQ-SL---EV--------G-E----
XP_004309219.2/1-233 -----------------NVSD-NVRVM-------VRQ-SL---EV--------G-E----
XP_024180881.1/1-233 -----------------NVSD-NVRVM-------VRQ-SL---EV--------G-E----
XP_024180911.1/1-233 -----------------NVSD-NVRVM-------VRQ-SL---EV--------G-E----
XP_019450525.1/1-216 -----------------ALDD-NMKVM-------ARK-N----VA--------G-E----
XP_019465255.1/1-230 -----------------VMDP-NIKVM-------GRK-N----VV--------G-E----
XP_014502466.1/1-227 -----------------NLAD-DMKIM-------ARK-IV--DDP--------N-E----
XP_017438850.1/1-222 -----------------SLAD-DMKIM-------ARK-IV--DDP--------N-E----
XP_006599623.1/1-221 -----------------NLAD-DMRIM-------ARK-I---DDP--------T-E----
XP_006599622.1/1-221 -----------------NLAD-DMRIM-------ARK-I---DDP--------T-E----
XP_006599621.1/1-221 -----------------NLAD-DMRIM-------ARK-I---DDP--------T-E----
XP_004515730.1/1-240 -----------------NMGNSDMKIM-------SRK-HQ--DNS--------G-E----
XP_013442370.1/1-239 -----------------NMGDSDLKIM-------YRQ-HP--DA----------------
XP_004252035.1/1-225 ----------------NGTDE-NVRIM-------TRK-TV--GDP--------S-R----
XP_015060726.1/1-225 ----------------NGTDE-NVRIM-------TRK-TV--GDP--------S-R----
XP_006589608.1/1-229 ------------------DLE-NVRVM-------TRK-SV--DDP--------G-R----
XP_006589605.1/1-230 ------------------DLE-NVRVM-------TRK-SV--DDP--------G-R----
XP_006849468.1/1-231 -------------------DD-GVRVT-------THK-S---TDP--------G-Q----
XP_020526173.1/1-231 -------------------DD-GVRVT-------THK-S---TDP--------G-Q----
XP_020109515.1/1-228 -------------------DV-GVRVT-------VHR-S---SDP--------G-Q----
NP_001310555.1/1-224 -------------------DV-GVRVM-------VHR-S---TDP--------G-Q----
XP_010544375.1/1-225 ----------------GHVDR-DVRVM-------TR-----------------G-E----
NP_001314001.1/1-230 ----------------GNVGE-DVRVM-------TRK-NV--NDP--------G-E----
XP_016732454.1/1-230 ----------------GNVGE-DVRVM-------TRK-NV--NDP--------G-E----
XP_016732453.1/1-230 ----------------GNVGE-DVRVM-------TRK-NV--NDP--------G-E----
XP_012484067.1/1-230 ----------------GNVGE-DVRVM-------TRK-NV--NDP--------G-E----
XP_012484066.1/1-230 ----------------GNVGE-DVRVM-------TRK-NV--NDP--------G-E----
XP_017631791.1/1-230 ----------------GNVGE-DVRVM-------TRK-NV--NDP--------G-E----
XP_017631789.1/1-230 ----------------GNVGE-DVRVM-------TRK-NV--NDP--------G-E----
XP_016728129.1/1-230 ----------------GNVGE-DVRVM-------TRK-NV--NDP--------G-E----
XP_016728128.1/1-230 ----------------GNVGE-DVRVM-------TRK-NV--NDP--------G-E----
XP_021671684.1/1-240 ----------------GNVSE-DIRIL-------TRK-NI--NEP--------G-K----
XP_021671683.1/1-240 ----------------GNVSE-DIRIL-------TRK-NI--NEP--------G-K----
XP_021671685.1/1-240 ----------------GNVSE-DIRIL-------TRK-NI--NEP--------G-K----
XP_002318231.3/1-240 ----------------GNVSE-DVRIL-------TRK-SI--NEP--------G-E----
XP_024438602.1/1-240 ----------------GNVSE-DVRIL-------TRK-SI--NEP--------G-E----
XP_024442055.1/1-239 ----------------ESVSE-DVRIL-------TRK-II--NEP--------G-E----
XP_002322460.1/1-239 ----------------ESVSE-DVRIL-------TRK-II--NEP--------G-E----
XP_011048682.1/1-239 ----------------ESVSE-DVRIL-------TRK-II--DEP--------G-E----
XP_011048684.1/1-239 ----------------ESVSE-DVRIL-------TRK-II--DEP--------G-E----
XP_011048683.1/1-239 ----------------ESVSE-DVRIL-------TRK-II--DEP--------G-E----
XP_019081404.1/1-234 ----------------ASLGE-DVKVM-------TRK-SM--NIP--------G-E----
XP_003634081.1/1-234 ----------------ASLGE-DVKVM-------TRK-SM--NIP--------G-E----
XP_008377249.1/1-231 ----------------ENVSE-DTRVM-------ARK-SM--NDP--------G-E----
XP_017189459.1/1-231 ----------------ENVSE-DTRVM-------ARK-SM--NDP--------G-E----
XP_008377244.1/1-231 ----------------ENVSE-DTRVM-------ARK-SM--NDP--------G-E----
XP_009371302.1/1-231 ----------------ENVSE-DVRVM-------ARK-SM--NDP--------G-E----
XP_009371299.1/1-231 ----------------ENVSE-DVRVM-------ARK-SM--NDP--------G-E----
XP_008376545.1/1-231 ----------------ENVSE-DVRVM-------ARK-SM--NDP--------G-E----
XP_008376544.1/1-231 ----------------ENVSE-DVRVM-------ARK-SM--NDP--------G-E----
XP_017188609.1/1-231 ----------------ENVSE-DVRVM-------ARK-SM--NDP--------G-E----
XP_022945426.1/1-238 ----------------GNISE-DVKMM-------ARN-SI--NDP--------G-E----
XP_022945425.1/1-238 ----------------GNISE-DVKMM-------ARN-SI--NDP--------G-E----
XP_023541229.1/1-238 ----------------GNISE-DVKMM-------ARN-SI--NDP--------G-E----
XP_023541228.1/1-238 ----------------GNISE-DVKMM-------ARN-SI--NDP--------G-E----
XP_022968414.1/1-238 ----------------GNISE-DVKMM-------ARN-SI--NDP--------G-E----
XP_022968413.1/1-238 ----------------GNISE-DVKMM-------ARN-SI--NDP--------G-E----
XP_023552381.1/1-232 ----------------GNISE-DVKVM-------ARK-SI--NEP--------G-E----
XP_022922479.1/1-232 ----------------GNISE-DVKVM-------ARK-SI--NEP--------G-E----
XP_023552380.1/1-232 ----------------GNISE-DVKVM-------ARK-SI--NEP--------G-E----
XP_022922478.1/1-232 ----------------GNISE-DVKVM-------ARK-SI--NEP--------G-E----
XP_022984644.1/1-232 ----------------GNISE-DVKVM-------ARK-SI--NEP--------G-E----
XP_022984643.1/1-232 ----------------GNISE-DVKVM-------ARK-SI--NEP--------G-E----
XP_008439185.1/1-242 ----------------GNISE-DVKVM-------ARK-SI--NDP--------G-E----
XP_008439184.1/1-242 ----------------GNISE-DVKVM-------ARK-SI--NDP--------G-E----
XP_011651140.1/1-242 ----------------GNISE-DVKVM-------ARK-SI--NDP--------G-E----
XP_004140784.1/1-242 ----------------GNISE-DVKVM-------ARK-SI--NDP--------G-E----
XP_009391954.1/1-234 ----------------ETIGE-DVRVM-------SRM-SV--NVP--------G-E----
XP_003581542.2/1-227 ----------------DNIGN-DVRII-------ERK-SV--DEP--------G-V----
XP_006652658.1/1-224 ----------------GNIGE-DVHVM-------AHK-SV--DEP--------G-M----
XP_015637147.1/1-225 ----------------GNIGE-DVHVM-------ARK-SV--DEP--------G-T----
XP_025823430.1/1-225 ----------------GSMRK-DVRVM-------VRK-SV--DEP--------G-V----
XP_004978185.1/1-225 ----------------GSMRK-DVRVM-------VRN-SV--DEP--------G-V----
XP_021317935.1/1-228 ----------------GSMGK-DVRVM-------VQN-SV--DEP--------G-V----
NP_001336434.1/1-226 ----------------GSTGK-DVRVM-------VQN-SV--DEP--------G-V----
XP_019709552.1/1-234 ----------------GSFGE-DVRVM-------TRK-SV--NNP--------G-E----
XP_015696802.1/1-257 ---GG--------GGAGDGED-KVRMM-------ARH-SV--GAP--------G-E----
XP_015612621.1/1-258 GGGGG--------GGGGDGED-KVRMM-------ARH-SV--GAP--------G-E----
XP_004957413.1/1-253 G----GAAG---NGAAGEGEK-KVRMM-------ARQ-SV--GAP--------G-E----
XP_025801167.1/1-251 G---GGAAG--NGGGAGEGEE-KVRMM-------ARQ-SV--GAP--------G-E----
NP_001105125.2/1-256 -GEGGDAAGNGGSAAAGEGEE-KVRMM-------ARH-SV--GAP--------G-D----
XP_002462701.1/1-258 EGGGGSSAGNGGGGAAGEGEE-KVRMM-------ARQ-SV--GAP--------G-E----
XP_020262629.1/1-222 --------------GDNNIAD-DVRVM-------TRQ-SL--NEP--------G-E----
XP_020699782.1/1-235 --------------EVG--ED-MVRVM-------TRQ-SV--DDP--------G-E----
XP_020699776.1/1-235 --------------EVG--ED-MVRVM-------TRQ-SV--DDP--------G-E----
XP_020573166.1/1-235 --------------EVG--ED-MVRVM-------TRQ-SV--DDP--------G-E----
XP_020187432.1/1-228 ---------------TGSIGE-DVRIM-------ARR-SV--DEP--------G-V----
XP_025656795.1/1-233 -----------------NVGD-EIKVM-------TRK-NI--DVP--------G-E----
XP_025656792.1/1-233 -----------------NVGD-EIKVM-------TRK-NI--DVP--------G-E----
XP_025656794.1/1-233 -----------------NVGD-EIKVM-------TRK-NI--DVP--------G-E----
XP_020979736.1/1-233 -----------------NVGD-EIKVM-------TRK-NI--DVP--------G-E----
XP_020999151.1/1-233 -----------------NVGD-EIKVM-------TRK-NT--DVP--------G-E----
XP_015967451.1/1-233 -----------------NVGD-EIKVM-------TRK-NT--DVP--------G-E----
XP_020999150.1/1-233 -----------------NVGD-EIKVM-------TRK-NT--DVP--------G-E----
XP_025701970.1/1-233 -----------------NVGD-EIKVM-------TRK-NT--DVP--------G-E----
XP_025701968.1/1-233 -----------------NVGD-EIKVM-------TRK-NT--DVP--------G-E----
XP_025701969.1/1-233 -----------------NVGD-EIKVM-------TRK-NT--DVP--------G-E----
XP_020221720.1/1-222 -----------------NLGD-DMRIM-------ARK-NG--DDP--------N-E----
XP_007152327.1/1-222 -----------------SLAD-DMKIM-------ARK-TV--DDP--------N-E----
XP_006587345.1/1-222 -----------------NLAD-DMRIM-------ARKINM--DDP--------T-E----
XP_006587344.1/1-222 -----------------NLAD-DMRIM-------ARKINM--DDP--------T-E----
XP_010035104.1/1-239 -----------------NMNG-DVRVT-------TRK-NA--GRP--------G-E----
XP_021765162.1/1-234 -----------------GLGA-DVCVM-------SRV-SV--DEP--------G-E----
XP_021765160.1/1-234 -----------------GLGA-DVCVM-------SRV-SV--DEP--------G-E----
XP_021730123.1/1-234 -----------------VLGA-DVCVM-------SRV-SV--DEP--------G-E----
XP_021842103.1/1-232 -----------------GLGA-DVCVM-------SRV-SI--DEP--------G-E----
XP_021842106.1/1-232 -----------------GLGA-DVCVM-------SRV-SI--DEP--------G-E----
XP_021842105.1/1-232 -----------------GLGA-DVCVM-------SRV-SI--DEP--------G-E----
XP_021842104.1/1-232 -----------------GLGA-DVCVM-------SRV-SI--DEP--------G-E----
XP_010693471.1/1-235 -----------------GLGA-DVCVM-------SRV-SI--DEP--------G-E----
XP_019107938.1/1-235 -----------------GLGA-DVCVM-------SRV-SI--DEP--------G-E----
XP_010693469.1/1-235 -----------------GLGA-DVCVM-------SRV-SI--DEP--------G-E----
XP_019107937.1/1-235 -----------------GLGA-DVCVM-------SRV-SI--DEP--------G-E----
XP_020163116.1/1-258 AGGR----------DPASGED-KVRMM-------ARQ-SV--GAP--------G-E----
XP_020163109.1/1-258 AGGR----------DPASGED-KVRMM-------ARQ-SV--GAP--------G-E----
XP_003578475.1/1-267 AMPGGGDQQQ-HQAGGGDGDK-EVRMM-------ARH-SV--GAP--------G-E----
XP_011012369.1/1-242 -----------------NVSE-DVRIL-------TRK-SI--NEP--------G-E----
XP_011012370.1/1-242 -----------------NVSE-DVRIL-------TRK-SI--NEP--------G-E----
XP_008234278.1/1-230 -----------------NVSE-DLRVM-------ARK-SV--DDP--------G-E----
XP_021827117.1/1-230 -----------------NVSE-DLRVM-------ARK-SV--DDP--------G-E----
XP_021827116.1/1-230 -----------------NVSE-DLRVM-------ARK-SV--DDP--------G-E----
XP_020413581.1/1-230 -----------------NVSE-DLRVM-------ARK-SV--DDP--------G-E----
XP_007219555.2/1-230 -----------------NVSE-DLRVM-------ARK-SV--DDP--------G-E----
XP_018456926.1/1-230 -----------------NVGQ-DTRIL-------TRR-SL---------------Q----
XP_013613235.1/1-226 -----------------NVGQ-DTRIL-------TRK-SL---------------Q----
XP_013686466.1/1-227 -----------------NVGQ-ETRIL-------TRK-SL---------------Q----
XP_009106897.1/1-227 -----------------NVGQ-ETRIL-------TRK-SL---------------Q----
XP_006401857.1/1-229 -----------------NVGQ-GTRIL-------TRK-SL---------------E----
XP_020871109.1/1-230 -----------------NVGQ-DMRIL-------TRK-SL---------------E----
NP_001332093.1/1-229 -----------------NVGQ-DTRIL-------TRK-SL---------------E----
NP_200030.1/1-229    -----------------NVGQ-DTRIL-------TRK-SL---------------E----
XP_006282099.1/1-225 -----------------NVGQ-DTRIL-------TRK-SF---------------E----
XP_019095386.1/1-229 -----------------NVGQ-DTRIL-------TRK-SL---------------E----
XP_019095387.1/1-229 -----------------NVGQ-DTRIL-------TRK-SL---------------E----
XP_019091174.1/1-228 -----------------NVGQ-DTRIL-------TRK-SL---------------E----
XP_019091179.1/1-228 -----------------NVGQ-DTRIL-------TRK-SL---------------E----
XP_019087173.1/1-228 -----------------NVGQ-DTRIL-------TRK-SL---------------E----
XP_010555654.1/1-234 -----------------NVGE-DMRIR-------TRK-NL--IDP--------G-E----
XP_010555656.1/1-234 -----------------NVGE-DMRIR-------TRK-NL--IDP--------G-E----
XP_010555655.1/1-234 -----------------NVGE-DMRIR-------TRK-NL--IDP--------G-E----
XP_010537199.1/1-242 -----------------NVGE-DMRIL-------TRK-NV--SDP--------G-E----
XP_010537197.1/1-242 -----------------NVGE-DMRIL-------TRK-NV--SDP--------G-E----
XP_010537198.1/1-241 -----------------NVGE-DMRIL-------TRK-NV--SDP--------G-E----
Con_90%              ......................slpl........hpp..................p....
Con_80%              ......................slplh.......hpp......ps........t.p....
Con_70%              ......................DVplh.......spp......sP........s.p....
Con_60%              ...................st.DVRlh.......s++.sh...sP........G.p....
Con_50%              ...................sppDVRVh.......oRK.sh...-P........G.c....


XP_020582785.1/1-284 MLS--SLGGG-IVCAKASML--------------------------LQNVPPALLIRFLR
XP_020682623.1/1-284 KFS--SLGGS-ILCAKASML--------------------------LQNVPPALLVRFLR
XP_020682624.1/1-284 KFS--SLGGS-ILCAKASML--------------------------LQNVPPALLVRFLR
XP_006846015.1/1-286 AIS--TFGGG-ILCAKASML--------------------------LQNVPPALLVRFLR
NP_001142394.1/1-289 LFS--AMGGG-IMCAKASML--------------------------LQNVPPALLVRFLR
NP_001146215.1/1-288 FFS--AMGGG-IMCAKASML--------------------------LQNVPPALLVRFLR
XP_020405718.1/1-288 FFS--AMGGG-IMCAKASML--------------------------LQNVPPALLVRFLR
XP_002443548.1/1-287 FFS--AMGGG-IMCAKASML--------------------------LQNVPPAILVRFLR
XP_025808876.1/1-287 FFS--AIGGG-IMCAKASML--------------------------LQNVPPALLVRFLR
XP_004963232.1/1-287 FFS--AIGGG-IMCAKASML--------------------------LQNVPPALLVRFLR
XP_020192001.1/1-285 MFT--AIGGG-VLCAKASML--------------------------LQNVPPALLVRFLR
XP_003562005.1/1-285 LFS--AIGGG-ILCAKASML--------------------------LQNVPPALLVRFLR
XP_004982339.1/1-286 LFS--AIGGG-ILCAKASML--------------------------LQNVPPALLVRFLR
XP_025796542.1/1-286 LFS--AIGGG-ILCAKASML--------------------------LQNVPPALLVRFLR
NP_001347456.1/1-286 LFT--AIGGG-ILCAKASML--------------------------LQNVPPALLVRFLR
NP_001352464.1/1-286 LFT--AIGGG-ILCAKASML--------------------------LQNVPPALLVRFLR
XP_002464180.1/1-286 LFT--TIGGG-ILCAKASML--------------------------LQNVPPALLVRFLR
XP_015689987.1/1-286 LFS--AIGGG-ILCAKASML--------------------------LQNVPPALLVRFLR
XP_015632484.1/1-286 LFS--AIGGG-ILCAKASML--------------------------LQNVPPALLVRFLR
XP_015620816.1/1-285 LFS--TVGGG-ILCAKASML--------------------------LQNVPPALLVRFLR
XP_006664738.1/1-285 LFS--TVGGG-ILCAKASML--------------------------LQNVPPALLVRFLR
XP_020190954.1/1-286 LFS--AIGGG-ILCAKASML--------------------------VQDVPPALLVRFLR
XP_020163543.1/1-286 LFS--AIGGG-ILCAKASML--------------------------VQDVPPALLVRFLR
XP_003577453.1/1-286 LFS--AIGGG-ILCAKASML--------------------------LQNVPPALLVRFLR
XP_020575104.1/1-285 MFS--SLGGG-ILCAKASML--------------------------LQNVPPALLVQFLR
XP_020696902.1/1-286 LFS--SLGGG-ILCAKASML--------------------------LQNVPPALLVQFLR
XP_020578108.1/1-284 PSF--AFGGG-ILCAKASML--------------------------LQNVPPALLVRFLR
XP_020698923.1/1-284 PLF--AFGGG-ILCAKASML--------------------------LQNVPSALLVRFLR
XP_020096610.1/1-279 AFF--CAVGG-VLCVKASML--------------------------LQNVPPALLVRFLR
XP_009412508.1/1-287 AFS--GIGCG-ILCAKASML--------------------------LQNVPPALLVCFLR
XP_020271156.1/1-284 IFS--PIGGG-ILCAKASML--------------------------LQNVPPALLVRFLR
XP_020273236.1/1-287 IFS--PIGGG-ILCAKASML--------------------------LQNVPPALLVRFLR
XP_020092733.1/1-287 LFS--ANGGG-VLCAKASML--------------------------LQNVPPALLVRFLR
XP_019701934.1/1-288 MFS--AMGGG-ILCAKASML--------------------------LQNVPPALLVRFLR
XP_010905421.1/1-288 MFS--AMGGG-ILCAKASML--------------------------LQNVPPALLVRFLR
XP_010905420.1/1-289 MFS--AMGGG-ILCAKASML--------------------------LQNVPPALLVRFLR
XP_010923678.1/1-288 MFS--AMGGG-ILCAKASML--------------------------LQNVPPALLVRFLR
XP_008794452.1/1-288 MFS--AMGGG-ILCAKASML--------------------------LQNVPPALLVRFLR
XP_009397391.1/1-288 MFS--TLGVG-VLCAKASML--------------------------LQNVPPAMLVRFLR
XP_009408551.1/1-288 LFS--TMGGG-VLCAKASML--------------------------LQNVPPAILVRFLR
XP_009399045.1/1-288 VFS--TLGGG-VLCAKASML--------------------------LQNVPPAILVQFLR
XP_009388964.1/1-288 LYS--TLGGG-ILCAKASML--------------------------LQNVPPAILVRFLR
XP_009397717.1/1-288 IYS--TLGGG-VLCAKASML--------------------------LQNVPPAILVRFLR
XP_018488876.1/1-280 FLP--SFGSG-VLCAKASML--------------------------LQNVPPLVLIRFLR
XP_013587486.1/1-280 FLP--SFGSG-VLCAKASML--------------------------LQNVPPLVLIRFLR
XP_009115139.1/1-280 FLP--SFGSG-VLCAKASML--------------------------LQNVPPLVLIRFLR
XP_013662459.1/1-280 FLP--SFGSG-VLCAKASML--------------------------LQNVPPLVLIRFLR
XP_013707293.1/1-280 FLP--SFGSG-VLCAKASML--------------------------LQNVPPLVLIRFLR
XP_006415465.2/1-280 FLP--SFGSG-VLCAKASML--------------------------LQNVPPLVLIRFLR
XP_019099288.1/1-280 FLP--SFGSG-VLCAKASML--------------------------LQNVPPLVLIRFLR
NP_174337.1/1-280    FLP--SFGSG-VLCAKASML--------------------------LQNVPPLVLIRFLR
XP_020867051.1/1-280 FLP--SFGSG-VLCAKASML--------------------------LQNVPPLVLIRFLR
XP_020867052.1/1-280 FLP--SFGSG-VLCAKASML--------------------------LQNVPPLVLIRFLR
XP_010460847.1/1-280 FLP--SFGSG-VLCAKASML--------------------------LQNVPPLVLIRFLR
XP_019094482.1/1-280 FLP--SFGSG-VLCAKASML--------------------------LQNVPPLVLIRFLR
XP_006306764.1/1-280 FLP--SFGSG-VLCAKASML--------------------------LQNVPPLVLIRFLR
XP_010538268.1/1-285 LFP--SFGEG-VLCAKASML--------------------------LQNVPPAVLVRFLR
XP_010519070.1/1-284 FLP--SFSGG-VLCAKASML--------------------------LQNVPPAVLVRFVR
XP_018436356.1/1-280 FLP--SFGSG-VLCAKASML--------------------------LENVPPAVLIRFLR
XP_013637594.1/1-286 FLP--SFGSG-VLCAKASML--------------------------LENVPPAVLVRFLR
XP_013692700.1/1-286 FLP--SFGSG-VLCAKASML--------------------------LENVPPAVLVRFLR
XP_009143747.1/1-286 FLP--SFGSG-VLCAKASML--------------------------LENVPSAVLVRFLR
XP_018484893.1/1-285 FLP--SFGSG-VLCAKASML--------------------------LQNVPPAVLVRFLR
XP_013637264.1/1-280 FLP--SFGSG-VLCAKASML--------------------------LQNVPPAVLVRFLR
XP_013637263.1/1-280 FLP--SFGSG-VLCAKASML--------------------------LQNVPPAVLVRFLR
XP_022557131.1/1-280 FLP--SFGSG-VLCAKASML--------------------------LQNVPPAVLVRFLR
XP_013729051.1/1-280 FLP--SFGSG-VLCAKASML--------------------------LQNVPPAVLVRFLR
XP_013744548.1/1-280 FLP--SFGSG-VLCAKASML--------------------------LQNVPPAVLVRFLR
XP_009141396.1/1-280 FLP--SFGSG-VLCAKASML--------------------------LQNVPPAVLVRFLR
XP_006410621.1/1-284 FLP--SFGSG-VLCAKASML--------------------------LQNVPPAVLVRFLR
NP_181018.1/1-284    FLP--SFGSG-VLCAKASML--------------------------LQNVPPAVLVRFLR
XP_020884308.1/1-284 FLP--SFGSG-VLCAKASML--------------------------LQNVPPAVLVRFLR
XP_006293359.1/1-284 FLP--SFGSG-VLCAKASML--------------------------LQNVPPAVLVRFLR
XP_010516626.1/1-284 FLP--SFGSG-VLCAKASML--------------------------LQNVPPAVLIRFLR
XP_010509720.1/1-284 FLP--SFGSG-VLCAKASML--------------------------LQNVPPAVLIRFLR
XP_010504942.1/1-284 FLP--SFGSG-VLCAKASML--------------------------LQNVPPAVLIRFLR
XP_017223336.1/1-283 MLP--TFG-G-VLCAKASML--------------------------IQDVPPPLLVRFLR
XP_016559371.1/1-285 ILP--TFG-G-VLCARASML--------------------------LQDVCPALLVRFLR
XP_016559374.1/1-285 ILP--TFG-G-VLCARASML--------------------------LQDVCPALLVRFLR
XP_012830207.1/1-281 MLP--TFG-G-VLSARASML--------------------------LQNVPPALLVRFLR
XP_012827547.1/1-287 MLS--TYG-G-VLCARASML--------------------------LQNVPPALLVRFLR
XP_022866806.1/1-284 MLP--TFG-G-ILCARASML--------------------------LQDVPPALLIRFLR
XP_022866805.1/1-285 MLP--TFG-G-ILCARASML--------------------------LQDVPPALLIRFLR
XP_023001017.1/1-285 LYP--TFG-G-VLCAKASML--------------------------LQNVPPALLVRFLR
XP_023001016.1/1-285 LYP--TFG-G-VLCAKASML--------------------------LQNVPPALLVRFLR
XP_023519837.1/1-285 LYP--TFG-G-VLCAKASML--------------------------LQNVPPALLVRFLR
XP_023519836.1/1-285 LYP--TFG-G-VLCAKASML--------------------------LQNVPPALLVRFLR
XP_022927139.1/1-285 LYP--TFG-G-VLCAKASML--------------------------LQNVPPALLVRFLR
XP_022927138.1/1-285 LYP--TFG-G-VLCAKASML--------------------------LQNVPPALLVRFLR
XP_022139982.1/1-285 LYP--TFG-G-VLCAKASML--------------------------LQNVPPALLVRFLR
XP_004135550.1/1-285 LYP--TFG-G-VLCAKASML--------------------------LQNVPPALLVRFLR
XP_008445207.1/1-285 LYP--TFG-G-VLCAKASML--------------------------LQNVPPALLVRFLR
XP_017230337.1/1-286 MLP--TFG-G-VLCAKASML--------------------------LQDVPPALLVRFLR
XP_017230338.1/1-286 MLP--TFG-G-VLCAKASML--------------------------LQDVPPALLVRFLR
XP_010316959.1/1-285 ILP--TFG-G-VLCARASML--------------------------LQDVCPALLVRFLR
XP_004233170.1/1-285 ILP--TFG-G-VLCARASML--------------------------LQDVCPALLVRFLR
XP_015066839.1/1-285 ILP--TFG-G-VLCARASML--------------------------LQDVCPALLVRFLR
XP_015066842.1/1-285 ILP--TFG-G-VLCARASML--------------------------LQDVCPALLVRFLR
XP_015066843.1/1-285 ILP--TFG-G-VLCARASML--------------------------LQDVCPALLVRFLR
XP_006353018.1/1-285 ILP--TFG-G-VLCARASML--------------------------LQDVCPALLVRFLR
XP_006353020.1/1-285 ILP--TFG-G-VLCARASML--------------------------LQDVCPALLVRFLR
XP_019178964.1/1-287 MLP--TFG-G-VLCARASML--------------------------LQNVPPALLVRFLR
XP_019178963.1/1-287 MLP--TFG-G-VLCARASML--------------------------LQNVPPALLVRFLR
XP_019228489.1/1-285 ILP--TFG-G-VLCARASML--------------------------LQNVPPALLVRFLR
XP_019228488.1/1-285 ILP--TFG-G-VLCARASML--------------------------LQNVPPALLVRFLR
NP_001289498.1/1-285 ILP--TFG-G-VLCARASML--------------------------LQNVPPALLVRFLR
XP_009777911.1/1-285 ILP--TFG-G-VLCARASML--------------------------LQNVPPALLVRFLR
XP_009612342.1/1-285 ILP--TFG-G-VLCARASML--------------------------LQNVPPALLVRFLR
XP_009612341.1/1-285 ILP--TFG-G-VLCARASML--------------------------LQNVPPALLVRFLR
XP_006363649.1/1-287 ILP--TFG-G-VLCARASML--------------------------LQNVPPALLVRFLR
XP_015066689.1/1-287 ILP--TFG-G-VLCARASML--------------------------LQNVPPALLVRFLR
XP_010315636.1/1-287 ILP--TFG-G-VLCARASML--------------------------LQNVPPALLVRFLR
XP_022879368.1/1-285 ILP--SFG-G-VLCARASML--------------------------VQNVPPALLVRFLR
XP_020554026.1/1-285 MLP--TFG-G-VLCARASML--------------------------LQNVPPALLVRFLR
XP_011096678.1/1-285 MLP--TFG-G-VLCARASML--------------------------LQNVPPALLVRFLR
XP_011085207.1/1-285 MLP--TFG-G-VLCARASML--------------------------LQNVPPALLVRFLR
XP_023003193.1/1-285 MYP--SFG-G-VMCAKASML--------------------------LQNVPPALLIRFLR
XP_022926723.1/1-285 MYP--SFG-G-VMCAKASML--------------------------LQNVPPALLIRFLR
XP_023518237.1/1-285 MYP--SFG-G-VMCAKASML--------------------------LQNVPPALLIRFLR
XP_022146537.1/1-286 MYP--SFGGG-VMCAKASML--------------------------LQNVPPALLVRFLR
XP_011658319.1/1-286 LYP--SFGGG-VMCAKASML--------------------------LQNVPPALLVRFLR
XP_008439554.1/1-286 LYP--SFGGG-VMCAKASML--------------------------LQNVPPALLVRFLR
XP_022978372.1/1-286 MYP--SYGGG-VMCAKASML--------------------------LQDVPPALLVRFLR
XP_023543833.1/1-286 MYP--SFGGG-VMCAKASML--------------------------LQDVPPALLVRFLR
XP_022926042.1/1-286 MYP--SFGGG-VMCAKASML--------------------------LQDVPPALLVRFLR
XP_010247499.1/1-286 VFS--NFGGG-VLCAKASML--------------------------LQNVPPALLVRFLR
XP_016717556.1/1-285 MVP--SFGGG-VLCAKASML--------------------------LQSVPPALLVRFLR
XP_012455108.1/1-285 MVP--SFGGG-VLCAKASML--------------------------LQSVPPALLVRFLR
XP_016717554.1/1-287 MVP--SFGGG-VLCAKASML--------------------------LQSVPPALLVRFLR
XP_012455107.1/1-287 MVP--SFGGG-VLCAKASML--------------------------LQSVPPALLVRFLR
XP_016711412.1/1-287 MVP--SFGGG-VLCAKASML--------------------------LQSVPPALLVRFLR
XP_017649973.1/1-287 MVP--SFGGG-VLCAKASML--------------------------LQSVPPALLVRFLR
XP_022716449.1/1-285 -FP--SFGGG-VLCAKASML--------------------------LQNVPPALLVRFLR
XP_022724664.1/1-287 MFP--SFGGG-VLCAKASML--------------------------LQNVPPALLVRFLR
XP_021298192.1/1-285 MFP--SFGGG-VLCAKASML--------------------------LQNVPPALLVRFLR
XP_021298193.1/1-294 MFP--SFGGG-VLCAKASML--------------------------LQNVPPALLVRFLR
XP_021298191.1/1-294 MFP--SFGGG-VLCAKASML--------------------------LQNVPPALLVRFLR
XP_007022874.2/1-285 MFP--SFGGG-VLCAKASML--------------------------LQNVPPALLVRFLR
XP_017979205.1/1-294 MFP--SFGGG-VLCAKASML--------------------------LQNVPPALLVRFLR
XP_017979204.1/1-294 MFP--SFGGG-VLCAKASML--------------------------LQNVPPALLVRFLR
XP_017236680.1/1-286 MLP--TF-GG-VLCAKASML--------------------------LQDVPPALLVRFLR
XP_010051959.1/1-285 IFP--NFGRG-VLCAKASML--------------------------LQNVPPAVLVRFLR
XP_010051960.1/1-285 IFP--NFGRG-VLCAKASML--------------------------LQNVPPAVLVRFLR
XP_015882975.1/1-284 IFP--TFG-G-VLCAKASML--------------------------LQNVPPALLVRFLR
XP_015882797.1/1-284 IFP--TFG-G-VLCAKASML--------------------------LQNVPPALLVRFLR
XP_010098570.1/1-284 MFP--TFG-G-VLCAKASML--------------------------LQNVPPALLVRFLR
XP_011012084.1/1-284 MSP--TFG-G-VLCAKASML--------------------------LQNVPPALLVRFLR
XP_011012083.1/1-284 MSP--TFG-G-VLCAKASML--------------------------LQNVPPALLVRFLR
XP_006377667.2/1-284 ISP--TFG-G-VLCAKASML--------------------------LQNVPPALLVRFLR
XP_002281868.2/1-285 MFP--TFGGG-VLCAKASML--------------------------LQNVPPALLVRFLR
XP_018847863.1/1-285 MFS--TFGGG-VLCAKASML--------------------------LQNVPPALLVRFLR
XP_018847862.1/1-285 MFS--TFGGG-VLCAKASML--------------------------LQNVPPALLVRFLR
XP_023919942.1/1-285 MFS--TFGGG-VLCAKASML--------------------------LQNVPPALLVRFLR
XP_023919941.1/1-285 MFS--TFGGG-VLCAKASML--------------------------LQNVPPALLVRFLR
XP_018828101.1/1-285 MFS--TFGGG-VLCAKASML--------------------------LQDVPPALLVRFLR
XP_018828100.1/1-285 MFS--TFGGG-VLCAKASML--------------------------LQDVPPALLVRFLR
XP_002298892.1/1-285 MFP--TFGGG-VLCAKASML--------------------------LQNVPPALLVRFLR
XP_011038887.1/1-285 MFP--TFGGG-VLCAKASML--------------------------LQNVPPALLVRFLR
XP_011038886.1/1-285 MFP--TFGGG-VLCAKASML--------------------------LQNVPPALLVRFLR
XP_021657817.1/1-285 VFP--TFGGG-VLCAKASML--------------------------LQNVPPALLVQFLR
XP_021657818.1/1-285 VFP--TFGGG-VLCAKASML--------------------------LQNVPPALLVQFLR
XP_015577248.1/1-260 MFP--TFGGG-VLCAKASML--------------------------LQNVPPALLVRFLR
XP_021616343.1/1-285 IFP--TFGGG-VLCAKASML--------------------------LQNVPPAVLVRFLR
XP_021645103.1/1-285 MFP--TFGGG-VLCAKASML--------------------------LQNVPPALLVRFLR
XP_021592762.1/1-285 MFP--TFGGG-VLCAKASML--------------------------LQNVPPALLVRFLR
XP_012075627.1/1-285 MFP--TFGGG-VLCAKASML--------------------------LQNVPPALLVRFLR
XP_008372224.1/1-285 IFS--PFGGG-VLCAKASML--------------------------LQSVPPALLVRFLR
XP_009362238.1/1-285 IFS--PFGGG-VLCAKASML--------------------------LQSVPPALLVRFLR
XP_009344405.1/1-284 IFP--PFGGG-VLCAKASML--------------------------LQSVPPALLVRFLR
XP_008349150.1/1-284 IFP--PFGGG-VLCAKASML--------------------------LQSVPPALLVRFLR
XP_007214622.1/1-285 IFP--TFGGG-VLCAKASML--------------------------LQSVPPALLVRFLR
XP_021812393.1/1-285 IFP--TFGGG-VLCAKASML--------------------------LQSVPPALLVRFLR
XP_024161164.1/1-285 IFP--SFGGG-VLCAKASML--------------------------LQSVPPALLVRFLR
XP_004293989.1/1-285 IFP--SFGGG-VLCAKASML--------------------------LQSVPPALLVRFLR
XP_019419795.1/1-282 MFP--SFGGG-VLCAKASML--------------------------LQNVPPPLLVRFLR
XP_019426301.1/1-282 MFP--AFGGG-VMCAKASML--------------------------LQNVPPALLVRFLR
XP_003524993.1/1-285 MLP--AFGGG-VMCAKASML--------------------------LQNVPPALLVRFLR
XP_003532788.1/1-285 MLP--AFGGG-VLCAKASML--------------------------LQNVPPALLVRFLR
XP_020215009.1/1-285 MLP--AFGGG-VLCAKASML--------------------------LQNVPPALLVRFLR
XP_017409410.1/1-285 MLP--TFGGG-VLCAKASML--------------------------LQNVPPALLVRFLR
XP_017409418.1/1-285 MLP--TFGGG-VLCAKASML--------------------------LQNVPPALLVRFLR
XP_014509941.1/1-285 MLP--TFGSG-VLCAKASML--------------------------LQNVPPALLVRFLR
XP_007159548.1/1-285 MLP--TLGGG-VLCAKASML--------------------------LQNVPPALLVRFLR
XP_024047818.1/1-288 MLP--AFG-G-VLCAKASML--------------------------LQNVPPALLVRFLR
XP_006448915.2/1-288 MLP--AFG-G-VLCAKASML--------------------------LQNVPPALLVRFLR
XP_006468302.1/1-288 MLP--AFG-G-VLCAKASML--------------------------LQNVPPALLVRFLR
XP_006468303.1/1-287 MLP--AFG-G-VLCAKASML--------------------------LQNVPPALLVRFLR
XP_024047817.1/1-287 MLP--AFG-G-VLCAKASML--------------------------LQNVPPALLVRFLR
XP_004504217.1/1-285 MLP--AFGGG-VMCAKASML--------------------------LKNVPPALLVRFLR
XP_003594520.2/1-281 MFP--TFGGG-VLCAKASML--------------------------LQNVPPALLVRFLR
XP_024632601.1/1-281 MFP--TFGGG-VLCAKASML--------------------------LQNVPPALLVRFLR
XP_004486450.1/1-285 MFP--SFGGG-VLCAKASML--------------------------LQNVPPALLVRFLR
XP_007147487.1/1-285 MFP--SFGGG-VLCAKASML--------------------------LQNVPPALLVRFLR
XP_017437412.1/1-285 MFP--AFGGG-VLCAKASML--------------------------LQNVPPALLVRFLR
XP_014518680.1/1-285 MFP--AFGGG-VLCAKASML--------------------------LQNVPPALLVRFLR
XP_003546255.1/1-285 MFP--AFGGG-VLCAKASML--------------------------LQNVPPALLVRFLR
XP_020233213.1/1-285 MFP--AFGGG-VLCAKASML--------------------------LQNVPPPLLVRFLR
XP_003535078.1/1-285 MFP--AFGGG-VLCAKASML--------------------------LQNVPPALLVRFLR
XP_014617344.1/1-285 MFP--AFGGG-VLCAKASML--------------------------LQNVPPALLVRFLR
XP_015959291.1/1-283 MFP--AFGGG-VLCAKASML--------------------------LQNVPPALLVRFLR
XP_016197724.1/1-283 MFP--AFGGG-VLCAKASML--------------------------LQNVPPALLVRFLR
XP_025694278.1/1-283 MFP--AFGGG-VLCAKASML--------------------------LQNVPPALLVRFLR
XP_006846777.1/1-281 -HV--GMSGG-ILCAKSSML--------------------------LQNVPPALLIRFLR
XP_003574071.1/1-280 FES----PGG-VICAKASML--------------------------LQSVPPAVLVRFLR
XP_020174778.1/1-278 FIA----PGG-VICAKASML--------------------------LQSVPPAVLVRFLR
XP_006661867.1/1-281 FGP--P--GG-VICAKASML--------------------------LQSVPPAVLVRFLR
XP_015614901.1/1-281 FVT--P--GG-VICAKASML--------------------------LQSVPPAVLVRFLR
XP_004982838.1/1-282 FGA--P--GG-IICAKASML--------------------------LQSVPPAVLVRFLR
XP_025796396.1/1-282 FGA--P--GG-IICAKASML--------------------------LQSVPPAVLVRFLR
NP_001316502.1/1-284 FGA--PGPGG-IICAKASML--------------------------LQSVPPAVLVRFLR
XP_021306737.1/1-282 FGA--P--GG-IICAKASML--------------------------LQSVPPAVLVRFLR
XP_021306733.1/1-293 FGA--P--GG-IICAKASML--------------------------LQSVPPAVLVRFLR
XP_020162053.1/1-281 FEA----LGG-TICAKASML--------------------------LQSVPPAILVRFLR
XP_006649248.1/1-282 FGA----PGG-IICAKASML--------------------------LQSVPPAVLVRFLR
XP_015632709.1/1-282 FGA----PGG-IICAKASML--------------------------LQSVPPAVLVRFLR
NP_001296150.1/1-282 FGA----PGG-IICAKASML--------------------------LQSVPPAVLVRFLR
XP_008665074.1/1-283 FGA----PGG-IICAKASML--------------------------LQSVPPAVLVRFLR
NP_001105994.1/1-283 FGA----PGG-IICAKASML--------------------------LQSVPPAVLVRFLR
XP_008660404.1/1-283 FGA----PGG-IICAKASML--------------------------LQSVPPAVLVRFLR
XP_002468669.1/1-283 FGA----PGG-IICAKASML--------------------------LQSVPPAVLVRFLR
XP_021309716.1/1-285 FGA----PGG-IICAKASML--------------------------LQSVPPAVLVRFLR
XP_004986037.1/1-283 FGA----PGG-IICAKASML--------------------------LQSVPPAVLVRFLR
XP_025795001.1/1-283 FGA----PGG-IICAKASML--------------------------LQSVPPAVLVRFLR
XP_020093893.1/1-283 FSV----TGG-VICARASML--------------------------LQSVPPALLVRFLR
XP_020093894.1/1-282 FSV----TGG-VICARASML--------------------------LQSVPPALLVRFLR
XP_020259754.1/1-283 VTM----PDG-VVCAKASML--------------------------LQNVPPALLVRFLR
XP_009401955.1/1-282 FAS----AGG-VICAKASML--------------------------LQSVPPALLVRFLR
XP_008791541.1/1-283 FSV----SGG-VICAKASML--------------------------LQNVPPALLVRFLR
XP_017698637.1/1-283 FSV----SGG-VICAKASML--------------------------LQNVPPALLVRFLR
XP_010920422.1/1-283 FSV----SGG-VICAKASML--------------------------LQNVPPALLVQFLR
XP_010920423.1/1-283 FSV----SGG-VICAKASML--------------------------LQNVPPALLVQFLR
XP_010920419.1/1-283 FSV----SGG-VICAKASML--------------------------LQNVPPALLVQFLR
XP_010920426.1/1-283 FSV----SGG-VICAKASML--------------------------LQNVPPALLVQFLR
XP_019709326.1/1-287 FSV----SGG-VICAKASML--------------------------LQNVPPALLVRFLR
XP_008785281.1/1-283 FSV----SGG-VICAKASML--------------------------LQNVPPALLVRFLR
XP_008462593.1/1-288 SLA--FH-GG-ILCAKASML--------------------------LQNVPPIALIRFLR
XP_022969629.1/1-286 SLA--FH-AG-VLCAKASML--------------------------IQNVPPIALIRFLR
XP_023536970.1/1-286 SLA--FH-AG-VLCAKASML--------------------------IQNVPPIALIRFLR
XP_022937855.1/1-288 SLA--FH-AG-VLCAKASML--------------------------IQNVPPIALIRFLR
XP_022937858.1/1-288 SLA--FH-AG-VLCAKASML--------------------------IQNVPPIALIRFLR
XP_012827869.1/1-285 --P--MVGGG-ILCAKASML--------------------------LQNVPPAVLVRFLR
XP_022874815.1/1-279 --S--FL-GG-ILCVKASML--------------------------LQNVPPAVLIRFLR
XP_022874814.1/1-283 --S--FL-GG-ILCVKASML--------------------------LQNVPPAVLIRFLR
XP_018434000.1/1-280 --S--FL-GG-VLCAKASML--------------------------LQNVSPAVLIRFLR
XP_013642496.1/1-279 --S--FL-GG-VLCAKASML--------------------------LQNVCPAVLVRFLR
XP_009150087.1/1-279 --S--FL-GG-VLCAKASML--------------------------LQNVCPAVLVRFLR
XP_009150088.1/1-279 --S--FL-GG-VLCAKASML--------------------------LQNVCPAVLVRFLR
XP_022563181.1/1-279 --S--FL-GG-VLCAKASML--------------------------LHNVCPAVLIRFLR
XP_013675790.1/1-280 --S--FL-GG-VLCAKASML--------------------------LHNVCPAVLIRFLR
XP_013623090.1/1-279 --S--FL-GG-VLCAKASML--------------------------LHNVCPAVLIRFLR
XP_013623089.1/1-280 --S--FL-GG-VLCAKASML--------------------------LHNVCPAVLIRFLR
XP_020885782.1/1-285 --S--FL-GG-VLCAKASML--------------------------LQNVPPAVLIRFLR
XP_006280004.1/1-285 --S--FL-GG-VLCAKASML--------------------------LQNVPPAVLIRFLR
XP_019088563.1/1-285 --S--FL-GG-VLCAKASML--------------------------LQNVPPAVLIRFLR
XP_010483719.1/1-285 --S--FL-GG-VLCAKASML--------------------------LQNVPPAVLIRFLR
XP_010456679.1/1-285 --S--FL-GG-VLCAKASML--------------------------LQNVPPAVLIRFLR
XP_006400821.1/1-284 --S--FL-GG-VLCAKASML--------------------------LQNVPPAVLIRFLR
NP_200877.1/1-284    --S--FL-GG-VLCAKASML--------------------------LQNVPPAVLIRFLR
XP_013611543.1/1-282 --S--FL-GG-VLCAKASML--------------------------LQNVPPAVLIRFLR
XP_009120605.1/1-284 --S--FL-GG-VLCAKASML--------------------------LQNVPPAVLIRFLR
XP_013667152.1/1-284 --S--FL-GG-VLCAKASML--------------------------LQNVPPAVLIRFLR
XP_018446535.1/1-284 --S--FL-GG-VLCAKASML--------------------------LQNVPPAVLIRFLR
XP_018454374.1/1-284 --S--FL-GG-VLCAKASML--------------------------LQNVPPAVLIQFLR
XP_009126588.1/1-285 --S--FL-GG-VLCAKATML--------------------------LQNVPPAVLIQFLR
XP_013680521.1/1-285 --S--FL-GG-VLCAKASML--------------------------LQNVPPAVLIQFLR
XP_013612427.1/1-285 --S--FL-GG-VLCGKASML--------------------------LQNVPPAVLIQFLR
XP_010535608.1/1-283 --S--FL-GG-VLCAKASML--------------------------LQNVPPAVLVRFLR
XP_010520372.1/1-276 --T--FL-GG-VLCAKASML--------------------------LQDVPPAVLVRFLR
XP_004487103.1/1-286 SLT--LL-GG-IVCAKASML--------------------------LQNITPAVLVRFLR
XP_003597252.1/1-283 SLS--LL-GG-IVCAKASML--------------------------LQNTTPAVLVRFLR
XP_024455683.1/1-286 SLS--FL-GG-ILCAKASML--------------------------LQNVHPAVLVCFLR
XP_022026352.1/1-288 SLS--YL-GG-ILCAKASML--------------------------FQNVPPAVLVRFLR
XP_022000486.1/1-288 SPP--YL-GG-ILCAKASML--------------------------FENVPPAVLVRFLR
XP_016717477.1/1-281 GLS--FL-GG-VLCAKASML--------------------------LQNVPPAVLVRFLR
XP_017643014.1/1-281 GLS--FL-GG-VLCAKASML--------------------------LQNVPPAVLVRFLR
XP_016724213.1/1-281 GLS--FL-GG-VLCAKASML--------------------------LQNVPPAVLVRFLR
XP_012462938.1/1-281 GLS--FL-GG-VLCAKASML--------------------------LQDVPPAVLVRFLR
XP_012471509.1/1-292 ALP--FV-GG-VLCAKASML--------------------------LQNVPPAVLVRFLR
XP_017642742.1/1-292 ALP--FV-GG-VLCAKASML--------------------------LQNVPPAVLVRFLR
XP_016755876.1/1-292 ALP--FV-GG-VLCAKASML--------------------------LQNVPPAVLVRFLR
XP_012471510.1/1-286 ALP--FV-GG-VLCAKASML--------------------------LQNVPPAVLVRFLR
XP_016753711.1/1-286 ALP--FV-GG-VLCAKASML--------------------------LQNVPPAVLVRFLR
XP_017642744.1/1-286 ALP--FV-GG-VLCAKASML--------------------------LQNVPPAVLVRFLR
XP_016755877.1/1-286 ALP--FV-GG-VLCAKASML--------------------------LQNVPPAVLVRFLR
XP_016750468.1/1-286 ALS--FL-GG-VLCAKASML--------------------------LQNIAPAVLVRFLR
XP_016688657.1/1-286 ALS--FL-GG-VLCAKASML--------------------------LQNIAAAVLVRFLR
XP_012443272.1/1-286 ALS--FL-GG-VLCAKASML--------------------------LQNIAPAVLVRFLR
XP_017603001.1/1-286 ALS--FL-GG-VLCAKASML--------------------------LQNIAPAVLVRFLR
XP_022755632.1/1-285 ALS--FL-GG-VLCAKASML--------------------------LQNVPPAVLVRFLR
XP_022755633.1/1-285 ALS--FL-GG-VLCAKASML--------------------------LQNVPPAVLVRFLR
XP_022729815.1/1-286 ALS--FL-GG-VLCAKASML--------------------------LQNVPPAVLVRFLR
XP_022729816.1/1-286 ALS--FL-GG-VLCAKASML--------------------------LQNVPPAVLVRFLR
XP_022752857.1/1-286 AFS--FL-GG-VLCAKASML--------------------------LQNVPPAVLVRFLR
XP_022752856.1/1-286 AFS--FL-GG-VLCAKASML--------------------------LQNVPPAVLVRFLR
XP_021277981.1/1-286 ALS--FL-GG-VLCAKASML--------------------------LQNVPPAVLVRFLR
XP_017983554.1/1-286 ALS--FL-GG-VLCAKASML--------------------------LQNVPPAVLVRFLR
XP_011089912.1/1-283 ALS--LL-GG-VLCAKASML--------------------------LQNVPPAVLVRFLR
XP_007208090.1/1-286 LAL--LG-GG-VLCAKASML--------------------------LQNVPPAVLVRFLR
XP_008225704.1/1-286 LAL--LG-GG-VLCAKASML--------------------------LQNVPPAVLVRFLR
XP_021830853.1/1-286 LAL--LG-GG-VLCAKASML--------------------------LQNVPPAVLVRFLR
XP_008349754.1/1-286 SLA--LL-GG-VLCAKASML--------------------------LQNVPPAVLVRFLR
NP_001315783.1/1-288 SLA--LL-GG-VLCAKASML--------------------------LQNVPPAVLVRFLR
XP_009367069.1/1-286 SLA--LL-GG-VLCAKASML--------------------------LQNVPPAVLVRFLR
XP_008344872.1/1-286 SLA--LL-GG-XLCAKASML--------------------------LQNVPPAVLXRFLR
XP_015952399.1/1-286 SLT--FL-GG-ILCAKASML--------------------------LQNVPPAVLVRFLR
XP_020982118.1/1-286 SLT--FL-GG-ILCAKASML--------------------------LQNVPPAVLVRFLR
XP_025685402.1/1-286 SLT--FL-GG-ILCAKASML--------------------------LQNVPPAVLVRFLR
XP_016187430.1/1-286 SLT--FL-GG-ILCAKASML--------------------------LQNVPPAVLVRFLR
XP_020214107.1/1-284 SLT--FL-GG-ILCAKASML--------------------------LQNVPPAVLVRFLR
XP_006592258.1/1-284 SLT--FL-GG-ILCAKASML--------------------------LQNVPPAVLVRFLR
XP_007132262.1/1-285 SLT--FL-GG-ILCAKASML--------------------------LQNVPPAVLVRFLR
XP_003538150.1/1-285 SLT--FL-GG-ILCAKASML--------------------------LQNVPPAVLVRFLR
XP_017433493.1/1-285 SLT--FL-GG-ILCAKASML--------------------------LQNVPPALLVRFLR
XP_014494018.1/1-285 SLT--FL-GG-ILCAKASML--------------------------LQNVPPALLVRFLR
XP_010089983.2/1-286 SLA--FL-GG-VLCAKASML--------------------------LQNVPPAVLVRFLR
XP_006378974.2/1-285 SLS--FL-GG-ILCAKASML--------------------------LQNVPPAVLVRFLR
XP_011010051.1/1-285 SLS--FL-GG-ILCAKASML--------------------------LQNVPPAVLVRFLR
XP_011010050.1/1-285 SLS--FL-GG-ILCAKASML--------------------------LQNVPPAVLVRFLR
XP_021905657.1/1-285 SLT--YL-GG-VLCAKASML--------------------------LQNVPPAVLVRFLR
XP_021610391.1/1-286 SLS--FL-GG-ILCAKASML--------------------------LQNVPPAVLVRFLR
XP_002529946.1/1-286 SFA--FL-GG-ILCAKASML--------------------------LQNVPPAVLVRFLR
XP_021658728.1/1-286 SFS--FL-GG-ILCAKASML--------------------------LQNVPPAVLVCFLR
XP_012064816.1/1-286 SLS--FL-GG-ILCAKASML--------------------------LQNVPPAVLVRFLR
XP_021628343.1/1-286 SLS--FL-GG-VLCAKASML--------------------------LQNVPPAVLVRFLR
XP_021657859.1/1-286 ALS--FL-GG-ILCAKASML--------------------------LQNVPPAVLVRFLR
XP_015874442.1/1-285 TLA--LL-GG-VLCAKASML--------------------------LQNVPPAVLVRFLR
XP_023926843.1/1-286 SLS--LV-GG-ILCAKASML--------------------------LQNVPPAVLVRFLR
XP_018819529.1/1-286 SLS--LV-GG-ILCAKASML--------------------------LQNVPPAVLVRFLR
XP_010651164.1/1-286 SLS--LP-GG-VLCAKASML--------------------------LQNVPPAVLVRFLR
XP_024034672.1/1-286 SLA--FL-GG-ILCAKASML--------------------------LQNVPPALLVRFLR
XP_006488084.1/1-286 SLA--FL-GG-ILCAKASML--------------------------LQNVPPALLVRFLR
XP_024034673.1/1-286 SLA--FL-GG-ILCAKASML--------------------------LQNVPPALLVRFLR
XP_010264677.1/1-287 TLT--LP-GG-ILCVKASML--------------------------LQNVPPALLVRFLR
XP_010253680.1/1-288 TLA--LS-GG-ILCAKASML--------------------------LQNVPPALLVRFLR
XP_022005829.1/1-282 PLS--FL-GG-VLCAKASML--------------------------FHNVPPAVLVRFLR
XP_023751537.1/1-278 SLS--FL-GG-VLCAKASML--------------------------FQNVPPAVLVRFLR
XP_024971452.1/1-283 SLS--FL-GG-VLCAKASML--------------------------FQNVPAAVLVRFLR
XP_024971444.1/1-287 SLS--FL-GG-VLCAKASML--------------------------FQNVPAAVLVRFLR
XP_024985647.1/1-287 SLS--FL-GG-ILCAKASML--------------------------FQNVPPAVLVRFLR
XP_023749448.1/1-288 SLS--FL-GG-ILCAKASML--------------------------FQNVPPAVLVRFLR
XP_010689538.1/1-281 SLP--MV-GG-VLCAKASML--------------------------LQNVPPAVLVRFLR
XP_021858740.1/1-280 SLP--MV-GG-VLCAKASML--------------------------LQNVPPAVLVRFLR
XP_021773317.1/1-281 SLP--MV-GG-VLCAKASML--------------------------LQNVPPAVLVRFLR
XP_021745941.1/1-281 SLP--MV-GG-VLCAKASML--------------------------LQNVPPAVLVRFLR
XP_016729039.1/1-286 ALP--LL-GG-VLCAKASML--------------------------IQNVSPAVLIRFLR
XP_017623828.1/1-286 ALP--LL-GG-VLCAKASML--------------------------IQNVSPAVLIRFLR
XP_012476406.1/1-286 ALP--LL-GG-VLCAKASML--------------------------IQNVSPAVLVRFLR
XP_016754776.1/1-286 ALP--LL-GG-VLCAKASML--------------------------IQNVSPAVLVRFLR
XP_022152944.1/1-286 SLA--FH-GG-ILCAKASML--------------------------LQNVPPIALIRFLR
XP_011040884.1/1-286 SLS--FL-GG-ILCAKASML--------------------------LQNVHPAVLVCFLR
XP_010043753.1/1-286 PLS--FV-GG-ILCAKASML--------------------------LQNVPPAVLVRFLR
XP_024158223.1/1-285 SFA--FL-GG-ILCAKASML--------------------------LQNVPPAVLVRFLR
XP_004291573.1/1-285 SFA--FL-GG-ILCAKASML--------------------------LQNVPPAVLVRFLR
XP_018808765.1/1-285 FLA--FG-GG-ILCAKASML--------------------------LQNVPPAMLVRFLR
XP_023512455.1/1-283 SLT--YP-GG-VLCAKASML--------------------------LQNVSPAVLVRFLR
XP_023512456.1/1-283 SLT--YP-GG-VLCAKASML--------------------------LQNVSPAVLVRFLR
XP_022944186.1/1-283 SLT--YP-GG-VLCAKASML--------------------------LQNVSPAVLVRFLR
XP_022944185.1/1-283 SLT--YP-GG-VLCAKASML--------------------------LQNVSPAVLVRFLR
XP_022985614.1/1-283 SLT--YP-GG-VLCAKASML--------------------------LQNVSPAVLVRFLR
XP_022985615.1/1-283 SLT--YP-GG-VLCAKASML--------------------------LQNVSPAVLVRFLR
XP_011656996.1/1-283 SLT--YP-GG-VLCAKASML--------------------------LQNVPPAVLVRFLR
XP_011656997.1/1-283 SLT--YP-GG-VLCAKASML--------------------------LQNVPPAVLVRFLR
XP_008463945.1/1-283 SLT--FP-GG-VLCAKASML--------------------------LQNIPPAVLVRFLR
XP_008463946.1/1-283 SLT--FP-GG-VLCAKASML--------------------------LQNIPPAVLVRFLR
XP_022140363.1/1-286 SLT--YP-GG-VLCAKASML--------------------------LQNVPPAVLVRFLR
XP_022995706.1/1-286 SLT--YP-GG-VLCAKASML--------------------------LQNVPPAVLVRFLR
XP_023534373.1/1-286 SLT--YP-GG-VLCAKASML--------------------------LQNVPPAVLVRFLR
XP_022958713.1/1-286 SLT--YP-GG-VLCAKASML--------------------------LQNVPPAVLVRFLR
XP_025614062.1/1-285 SLT--FL-GG-ILCAKASML--------------------------LQNIPPAVLVRFLR
XP_025614063.1/1-285 SLT--FL-GG-ILCAKASML--------------------------LQNIPPAVLVRFLR
XP_016170485.1/1-285 SLT--FL-GG-ILCAKASML--------------------------LQNIPPAVLVRFLR
XP_016170486.1/1-285 SLT--FL-GG-ILCAKASML--------------------------LQNIPPAVLVRFLR
XP_015935757.1/1-285 SLT--FL-GG-ILCAKASML--------------------------LQNIPPAVLVRFLR
XP_015935758.1/1-285 SLT--FL-GG-ILCAKASML--------------------------LQNIPPAVLVRFLR
XP_004505999.1/1-286 SLT--FL-GG-ILCAKASML--------------------------LQNVPPAVLVRFLR
XP_013456006.1/1-286 SLT--LL-GG-ILCAKASML--------------------------LQNVPPAVLVRFLR
XP_019453966.1/1-285 SVT--FL-GG-VLCAKASML--------------------------IQNVPPAVLVRFLR
XP_019448740.1/1-279 SSA--FL-GG-VLCAKASML--------------------------LQNVSPAVLIRFLR
XP_019448741.1/1-279 SSA--FL-GG-VLCAKASML--------------------------LQNVSPAVLIRFLR
XP_019458095.1/1-286 SLT--FF-GG-ILCAKASML--------------------------LQNVPPSVLIRFLR
XP_019447870.1/1-286 PLT--SL-GG-ILCAKASML--------------------------LQNVPPAALIRFLR
XP_012838629.1/1-280 -NS--LL-GG-VLCAKASML--------------------------LQNVPPAVLVRFLR
XP_012838630.1/1-280 -NS--LL-GG-VLCAKASML--------------------------LQNVPPAVLVRFLR
XP_012838627.1/1-280 -NS--LL-GG-VLCAKASML--------------------------LQNVPPAVLVRFLR
XP_012838628.1/1-280 -NS--LL-GG-VLCAKASML--------------------------LQNVPPAVLVRFLR
XP_017214964.1/1-286 SIS--MV-GG-VLCAKASML--------------------------LQNVPPAVLVRFLR
XP_017234817.1/1-286 SLS--LV-GG-VLCAKASML--------------------------LQNVPPAVLVRFLR
XP_019178153.1/1-282 TLP--LL-GG-ILCAKASML--------------------------LQNVPPAVLVRFLR
XP_019166562.1/1-283 TLQ--ML-GG-ILCAKASML--------------------------LQNVPPAVLVRFLR
XP_011089294.1/1-282 TLS--VL-GG-VLCAKASML--------------------------IQNVPPAVLVRFLR
XP_022892122.1/1-274 SLS--FI-GG-VLCAKASML--------------------------LQNVPPAVLVRFLR
XP_022883393.1/1-279 NLS--FI-GG-VLCAKASML--------------------------LQNVPPAVLVRVLR
XP_022883398.1/1-279 NLS--FI-GG-VLCAKASML--------------------------LQNVPPAVLVRVLR
XP_022883384.1/1-283 NLS--FI-GG-VLCAKASML--------------------------LQNVPPAVLVRVLR
XP_022883389.1/1-283 NLS--FI-GG-VLCAKASML--------------------------LQNVPPAVLVRVLR
XP_009776589.1/1-283 PLS--PL-GG-ILCAKASML--------------------------LQNVPPVVLVRFLR
NP_001312504.1/1-283 PLS--PL-GG-ILCAKASML--------------------------LQNVPPVVLVRFLR
XP_016472209.1/1-283 PLS--PL-GG-ILCAKASML--------------------------LQNVPPVVLVRFLR
XP_019255429.1/1-283 PLS--PL-GG-ILCAKASML--------------------------LQNVPPAVLVRFLR
XP_009624234.1/1-283 PLS--PL-GG-ILCAKASML--------------------------LQNVPPAVLVRFLR
XP_016504967.1/1-283 PLS--PL-GG-ILCAKASML--------------------------LQNVPPAVLVRFLR
XP_016547131.1/1-283 PLS--PP-GG-ILCAKASML--------------------------LQNVPPAVLVRFLR
XP_004251138.1/1-283 PLS--PL-GG-VLCAKASML--------------------------LQNVPPAVLVRFLR
XP_015058512.1/1-283 PLS--PL-GG-VLCAKASML--------------------------LQNVPPAVLVRFLR
XP_006340193.1/1-283 PLS--SL-GG-ILCAKASML--------------------------LQNVPPAVLVRFLR
XP_004235650.1/1-283 GFS--PMSNA-VMCAKASML--------------------------LQNVPPAILLRFLR
XP_006343047.1/1-283 GFS--PMSNA-VMCAKASML--------------------------LQNVPPAILLRFLR
XP_009774805.1/1-285 GFS--SLSNA-VMCAKASML--------------------------LQNVPPAILLRFLR
XP_016498616.1/1-285 GFS--SLSNA-VMCAKASML--------------------------LQNVPPAILLRFLR
XP_016434103.1/1-285 GFS--SLSNA-VMCAKASML--------------------------LQNVPPAILLRFLR
XP_009593967.1/1-285 GFS--SLSNA-VMCAKASML--------------------------LQNVPPAILLRFLR
XP_004510297.1/1-285 GFP--SVSNA-VLCAKASML--------------------------LQNVPPAILLRFLR
XP_004510296.1/1-285 GFP--SVSNA-VLCAKASML--------------------------LQNVPPAILLRFLR
XP_003627005.1/1-284 GFP--SVSNA-VLCAKASML--------------------------LQNVPPAILLRFLR
XP_003531653.1/1-284 GFP--SVSNA-VLCAKASML--------------------------LQNVHPAILLRFLR
XP_006585576.1/1-288 GFP--SVSNA-VLCAKASML--------------------------LQNVHPAILLRFLR
XP_014634618.1/1-288 GFP--SVSNA-VLCAKASML--------------------------LQNVHPAILLRFLR
XP_014634619.1/1-288 GFP--SVSNA-VLCAKASML--------------------------LQNVHPAILLRFLR
XP_003531652.1/1-285 GFP--SVSNA-VLCAKASML--------------------------LQNVPPAILLRFLR
XP_003530109.1/1-284 GFP--SVSNA-VLCAKASML--------------------------LQNVPPAILLRFLR
XP_007135643.1/1-285 GFP--SVSNA-VLCAKASML--------------------------LQNVPPAILLRFLR
XP_003530112.1/1-285 GFP--SVSNA-VLCAKASML--------------------------LQNVPPAILLRFLR
XP_020226142.1/1-285 GFP--SVSNA-VLCAKASML--------------------------LQNVPPAILLRFLR
XP_014516466.1/1-285 GFP--SVSNA-VLCAKASML--------------------------LQNVPPAILLRFLR
XP_017407594.1/1-285 GFP--SVSNA-VLCAKASML--------------------------LQNVPPAILLRFLR
XP_019448178.1/1-285 GFP--VVSNA-VLCAKASML--------------------------LQNVPPVVLLRFLR
XP_019429328.1/1-285 GFP--SVSNS-VLCAKASML--------------------------LQNVPPAILLRFLR
XP_004486776.1/1-285 GFP--SVSNA-VLCAKASML--------------------------LQNVPPAILLRFLR
XP_003597690.1/1-286 GFP--SVSNA-VLCAKASML--------------------------LQNVPPAILLRFLR
XP_015932679.1/1-285 GFP--SVSNA-VLCAKASML--------------------------LQNVPPAILLRFLR
XP_025674177.1/1-285 GFP--SVSNA-VLCAKASML--------------------------LQNVPPAILLRFLR
XP_025610204.1/1-285 GFP--SVSNA-VLCAKASML--------------------------LQNVPPAILLRFLR
XP_020226055.1/1-285 GFP--AVSNA-VLCAKASML--------------------------LQNVPPAILLRFLR
XP_007150614.1/1-285 GFP--SISNA-VLCAKASML--------------------------LQNVPPAILLRFLR
XP_014501306.1/1-285 GFP--SISNA-VLCAKASML--------------------------LQNVPPAILLRFLR
XP_017425270.1/1-285 GFP--SISNA-VLCAKASML--------------------------LQNVPPAILLRFLR
XP_021836126.1/1-285 GFP--AVSNT-VLCAKASML--------------------------LQNVPPAVLLRFLR
XP_010691423.1/1-284 GFP--AVSNT-VLCAKASML--------------------------LQNVPPAVLLRFLR
XP_010691424.1/1-284 GFP--AVSNT-VLCAKASML--------------------------LQNVPPAVLLRFLR
XP_021765330.1/1-284 GFP--AVSNT-VLCAKASML--------------------------LQNVPPAVLLRFLR
XP_021749885.1/1-284 GFP--AVSNT-VLCAKASML--------------------------LQNVPPAVLLRFLR
XP_010267183.1/1-285 GFP--AMNNS-VLCAKASML--------------------------LQNVPPAILLRFLR
XP_017258627.1/1-285 GFT--PVSNG-VLCAKASML--------------------------LQNVPPAILLRFLR
XP_023000315.1/1-284 GFS--SISNA-VLCARASML--------------------------LQNVPPAILLRFLR
XP_023000313.1/1-284 GFS--SISNA-VLCARASML--------------------------LQNVPPAILLRFLR
XP_023514554.1/1-284 GFS--SISNA-VLCARASML--------------------------LQNVPPAILLRFLR
XP_023514552.1/1-284 GFS--SISNA-VLCARASML--------------------------LQNVPPAILLRFLR
XP_022964458.1/1-284 GFS--SISNA-VLCARASML--------------------------LQNVPPAILLRFLR
XP_022964454.1/1-284 GFS--SISNA-VLCARASML--------------------------LQNVPPAILLRFLR
XP_022147627.1/1-284 GFS--AISNA-VLCARASML--------------------------LQNVPPAILLRFLR
XP_022147619.1/1-284 GFS--AISNA-VLCARASML--------------------------LQNVPPAILLRFLR
XP_008462437.1/1-284 GFS--AISNA-VLCARASML--------------------------LQNVPPAILLRFLR
XP_004141659.1/1-284 GFS--AISNA-VLCARASML--------------------------LQNVPPAILLRFLR
XP_008462436.1/1-284 GFS--AISNA-VLCARASML--------------------------LQNVPPAILLRFLR
XP_011659682.1/1-284 GFS--AISNA-VLCARASML--------------------------LQNVPPAILLRFLR
XP_022992111.1/1-284 GFS--AISNA-VLCARASML--------------------------LQNVPPAILLRFLR
XP_023548534.1/1-284 GFS--AISNA-VLCARASML--------------------------LQNVPPAILLRFLR
XP_022953371.1/1-284 GFS--AISNA-VLCARASML--------------------------LQNVPPAILLRFLR
XP_002304217.1/1-285 GFP--AVSSA-VLCAKASML--------------------------LQNVPPAILLRFLR
XP_011013041.1/1-285 GFP--AVSSA-VLCAKASML--------------------------LQNVPPAILLRFLR
XP_011013042.1/1-285 GFP--AVSSA-VLCAKASML--------------------------LQNVPPAILLRFLR
XP_011046759.1/1-284 GFP--AVSSA-VLCAKASML--------------------------LQNVPPAILLRFLR
XP_024464085.1/1-284 GFP--AVSSA-VLCAKASML--------------------------LQNVPPAILLRFLR
XP_024183498.1/1-286 GYP--AVSNA-VLCAKASML--------------------------LQNVPPAILLRFLR
XP_004302866.1/1-286 GFP--AVSNA-VLCAKASML--------------------------LQNVPPAILLRFLR
NP_001280898.1/1-285 GFP--AVSNS-VLCAKASML--------------------------LQNVPPAILLRFLR
XP_007214960.1/1-285 GFP--AVSNS-VLCAKASML--------------------------LQNVPPAILLRFLR
XP_008228437.1/1-285 GFP--AVSNS-VLCAKASML--------------------------LQNVPPAILLRFLR
XP_021818763.1/1-285 GFP--AVSNS-VLCAKASML--------------------------LQNVPPAILLRFLR
XP_022723515.1/1-285 GFP--SVNNA-VLCAKASML--------------------------LQNVPPAILLRFLR
XP_016688508.1/1-285 GFP--SVSNA-VLCAKASML--------------------------LQNVPPAILLRFLR
XP_012443274.1/1-285 GFP--SVSNA-VLCAKASML--------------------------LQNVPPAILLRFLR
XP_022721457.1/1-285 GFP--SVISA-VLCAKASML--------------------------LQNVPPAILLRFLR
XP_022715018.1/1-285 GFP--SVSNA-VLCAKASML--------------------------LQNVPPAILLRFLR
XP_017979082.1/1-285 GFP--SVSNA-VLCAKASML--------------------------LQNVPPAILLRFLR
XP_021293768.1/1-285 GFP--SVSNA-VLCAKASML--------------------------LQNVPPAILLRFLR
XP_010062518.1/1-285 GFP--AVSNA-VLCARASML--------------------------LQNVPPAVLLRFLR
XP_006426893.1/1-285 GFP--AVSNA-VLCAKASML--------------------------LQNVPPAILLRFLR
XP_021610318.1/1-285 GFS--AVSNA-VLCAKASML--------------------------LQNVPPAILLRFLR
XP_010098130.1/1-285 EFP--AVSNA-VLCAKASML--------------------------LQNVPPAILLRFLR
XP_021635282.1/1-296 GFP--AVSNA-VLCAKASML--------------------------LQNVPPAILLRFLR
XP_002284003.2/1-285 GFP--AVSNA-VLCAKASML--------------------------LQNVPPAILLRFLR
XP_002515977.1/1-285 GFP--AVSNA-VLCAKASML--------------------------LQNVPPAILLRFLR
XP_021643260.1/1-285 GFP--AVSNA-VLCAKASML--------------------------LQNVPPAILLRFLR
XP_021628194.1/1-285 GFP--AVSNA-VLCAKASML--------------------------LQNVPPAILLRFLR
XP_015894591.1/1-285 GFP--AVSNA-VLCAKASML--------------------------LQNVPPAILLRFLR
XP_012069098.1/1-285 GFP--SVSNA-VLCAKASML--------------------------LQNVPPAILLRFLR
XP_018847857.1/1-285 GFP--AVSNA-VLCAKASML--------------------------LQNVPPAILLRFLR
XP_023898511.1/1-285 GFP--AVSNA-VLCAKASML--------------------------LQNVPPAILLRFLR
XP_010545673.1/1-285 NGF-PHVSNA-VLCAKASML--------------------------LQNVSPAILLRFLR
XP_010545670.1/1-286 NGF-PHVSNA-VLCAKASML--------------------------LQNVSPAILLRFLR
XP_010535179.1/1-285 GFP--LVSNT-VLCAKASML--------------------------LQNVPPAILLRFLR
XP_018436929.1/1-283 GFA-PPVSNV-VLCAKASML--------------------------LQNVPPGILLRFLR
XP_013592012.1/1-283 GFT-P-VSSV-VLCAKASML--------------------------LQNVPPAILLRFLR
XP_013640375.1/1-284 GFA-P-VSNV-VLCAKASML--------------------------LQNVPPGILLRFLR
XP_013640376.1/1-283 GFA-P-VSNV-VLCAKASML--------------------------LQNVPPGILLRFLR
XP_009147709.1/1-283 GFA-P-VSNV-VLCAKASML--------------------------LQNVPPGILLRFLR
XP_006306297.1/1-281 GFA-P-VSSV-VLCAKASML--------------------------LQNVPPAILLRFLR
XP_010462051.1/1-281 GFA-P-VSSV-VLCAKASML--------------------------LQNVPPAILLRFLR
XP_010479714.1/1-281 GFA-P-VSSV-VLCAKASML--------------------------LQNVPPAILLRFLR
XP_010462052.1/1-281 GFA-P-VSSV-VLCAKASML--------------------------LQNVPPAILLRFLR
XP_010500807.1/1-281 GFA-P-VSSV-VLCAKASML--------------------------LQNVPPAILLRFLR
NP_849795.1/1-282    GFA-P-VSNV-VLCAKASML--------------------------LQNVPPAILLRFLR
NP_001077702.1/1-281 GFA-P-VSNV-VLCAKASML--------------------------LQNVPPAILLRFLR
NP_175627.1/1-281    GFA-P-VSNV-VLCAKASML--------------------------LQNVPPAILLRFLR
XP_020868586.1/1-281 GFA-P-VSNV-VLCAKASML--------------------------LQNVPPAILLRFLR
XP_006392953.1/1-282 GFA-P-VNSV-VLCAKASML--------------------------LQNVPPAILLRFLR
XP_018460807.1/1-282 GFA-P-VSNV-VLCAKASML--------------------------LQNVPPAILLRFLR
XP_013621760.1/1-283 GFA-P-VSNV-VLCAKASML--------------------------LQNVPPAILLRFLR
XP_018451524.1/1-282 GFA-P-VSNV-VLCAKASML--------------------------LQNVPPAILLRFLR
XP_009107148.1/1-282 GFA-P-VSNV-VLCAKASML--------------------------LQNVPPAILLRFLR
XP_009107147.1/1-283 GFA-P-VSNV-VLCAKASML--------------------------LQNVPPAILLRFLR
XP_009600774.1/1-284 EFT--SLSNA-VLCAKASML--------------------------LQSVTPAVLLRFLR
XP_016459079.1/1-284 EFT--SLSNA-VLCAKASML--------------------------LQSVTPAVLLRFLR
XP_019236029.1/1-285 EFT--SLSNA-VLCAKASML--------------------------LQSVTPAVLLRFLR
XP_009771098.1/1-285 EFT--SLSNA-VLCAKASML--------------------------LQSVTPAVLLRFLR
XP_016550962.1/1-285 GFT--SISNG-VLCAKASML--------------------------LQSVTPAVLLRFLR
XP_016550960.1/1-285 GFT--SISNG-VLCAKASML--------------------------LQSVTPAVLLRFLR
XP_016550961.1/1-285 GFT--SISNG-VLCAKASML--------------------------LQSVTPAVLLRFLR
XP_006359469.1/1-285 GFT--SLSNA-ILCAKASML--------------------------LQSVTPAILLRFLR
XP_004252354.1/1-285 GFT--SLSNA-VLCAKASML--------------------------LQSVTPAILLRFLR
XP_015061298.1/1-285 GFT--SLSNA-VLCAKASML--------------------------LQSVTPAILLRFLR
XP_019198775.1/1-285 GFT--SISNA-VLCAKASML--------------------------LQNVPPAMLLRFLR
XP_019194412.1/1-285 GFT--PISNA-VLCAKASML--------------------------LQNVPPAVLLRFLR
XP_016562656.1/1-283 GFS--SMSNA-VMCAKASML--------------------------LQNVPPAILLRFLR
XP_019267274.1/1-285 GFS--SLSNA-VMCAKASML--------------------------LQNVPPAILLRFLR
XP_019267275.1/1-282 GFS--SLSNA-VMCAKASML--------------------------LQNVPPAILLRFLR
XP_022889649.1/1-285 GYT--SVSNA-VLCAKASML--------------------------LQNVPPAILLGFLR
XP_022889648.1/1-285 GYT--SVSNA-VLCAKASML--------------------------LQNVPPAILLGFLR
XP_012852689.1/1-281 GYT--SISNS-VLCAKASML--------------------------LQNVPPAILLRFLR
XP_012856468.1/1-279 GYN--SISNS-VLCAKASML--------------------------LQNVPPAILLRFLR
XP_011080516.1/1-285 GYA--SVCNA-VLCAKASML--------------------------LQNVPPAILVRFLR
XP_011072422.1/1-285 GYN--SICNS-ILCAKASML--------------------------LQNVPPAILLRFLR
XP_022851148.1/1-285 GYP--SVCNA-VLCAKASML--------------------------LQNVPPAILLRFLR
XP_022844668.1/1-285 GYP--SVSNA-VLCAKASML--------------------------LQNVPPAILLRFLR
XP_006853643.2/1-288 GFP--TVCGA-VLCAKASML--------------------------LQNVPPALLIRFLR
XP_010249545.1/1-285 GFP--VMNNA-ILCAKASML--------------------------LQNVPPAILLRFLR
XP_010249539.1/1-285 GFP--VMNNA-ILCAKASML--------------------------LQNVPPAILLRFLR
XP_010915409.1/1-284 GFP--SLSNA-VLCAKASML--------------------------LQNVSPAMLLRFVR
XP_008791633.1/1-284 GFP--SLSNA-VLCAKASML--------------------------LQNVSPAMLLRFLR
XP_017235966.1/1-285 GLP--SIGHV-VLCAKASML--------------------------LQNVPPAILVRFLR
XP_023761873.1/1-288 GYP--SVNTA-ILCAKASMLL--------------------------QNVPPALLLRFLR
XP_021997125.1/1-286 GYP--SVNTA-VLCAKASMLL-------------------------PPNVPPALLLRFLR
XP_021990052.1/1-285 GYP--SVNTA-VLCAKASMLL--------------------------QNVPPAILLRFLR
XP_024979339.1/1-285 GYP--SVNTS-VMCAKASMLL--------------------------QNVPPALLLRFLR
XP_024979341.1/1-290 GYP--SVNTS-VMCAKASMLL--------------------------QNVPPALLLRFLR
XP_024979340.1/1-291 GYP--SVNTS-VMCAKASMLL--------------------------QNVPPALLLRFLR
XP_024979342.1/1-291 GYP--SVNTS-VMCAKASMLL--------------------------QNVPPALLLRFLR
XP_022016208.1/1-285 GYT--TVSNA-VMCAKASML--------------------------LQNVPPALLLRFLR
XP_022016207.1/1-311 GYT--TVSNA-VMCAKASMLLQVRLLSPRQLLHGCNFLIFFFFFFFLQNVPPALLLRFLR
XP_022009819.1/1-285 GYS--SVTNV-VLCAKASML--------------------------LQNVPPALLLRFLR
XP_022009815.1/1-285 GYS--SVTNV-VLCAKASML--------------------------LQNVPPALLLRFLR
XP_022009809.1/1-285 GYS--SVTNV-VLCAKASML--------------------------LQNVPPALLLRFLR
XP_023759500.1/1-286 GFT--PVSNA-VMCAKASML--------------------------LQNVPPALLLRFLR
XP_023751740.1/1-285 RYP--PVTNS-VLCAKASML--------------------------LQNVPPALLLRFLR
XP_024971803.1/1-285 GYT--PVSNA-VLCAKASML--------------------------LQNVPPALLLRFLR
XP_022033649.1/1-285 GYP--SVSNA-VLCAKASML--------------------------LQNVPPALLLRFLR
XP_024972195.1/1-285 GYQ--TVSNS-VLCAKASML--------------------------LQNVPPALLLRFLR
XP_024972196.1/1-289 GYQ--TVSNS-VLCAKASML--------------------------LQNVPPALLLRFLR
XP_020248539.1/1-284 GYS--SVTNS-VLCAKASML--------------------------LQNVHPAILLRFLR
XP_020269197.1/1-284 GFP--SVTNS-VLCAKASML--------------------------LQNVSPALLLRFLR
XP_020269196.1/1-284 GFP--SVTNS-VLCAKASML--------------------------LQNVSPALLLRFLR
XP_009399813.1/1-284 GCP--STTSS-VLCAKATML--------------------------LQNISPPMLLRFLR
XP_009399812.1/1-284 GCP--STTSS-VLCAKATML--------------------------LQNISPPMLLRFLR
XP_009413853.1/1-284 GFA--STSSS-VLCAKASML--------------------------LQNISPPMLLKFLR
XP_024985513.1/1-286 GFP--SISNG-VLCAKASML--------------------------LQNVPPAILTRFLR
XP_023729871.1/1-286 GFP--SISNA-ILCAKASML--------------------------LQNVPPAILTRFLR
XP_022000974.1/1-282 GFP--SISNA-VLCAKASML--------------------------LQNVPPAILTRFLR
XP_022023495.1/1-282 GFP--SIGHA-VLCAKASML--------------------------LQNVPPAILTRFLR
XP_010526102.1/1-286 GFQ--SMPNA-VLCAKASML--------------------------LQNVPPAILLRFLR
XP_019086035.1/1-282 GFT--SMPSA-VLCAKASML--------------------------LQNVPPSILLRFLR
XP_010437837.1/1-288 GFT--SMPSA-VLCAKASML--------------------------LQNVPPSILLRFLR
XP_010437838.1/1-288 GFT--SMPSA-VLCAKASML--------------------------LQNVPPSILLRFLR
XP_022549566.1/1-283 GFT--SMPSA-VLCAKASML--------------------------LQNVPPSILLRFLR
XP_013670651.1/1-283 GFT--SMPSA-VLCAKASML--------------------------LQNVPPSILLRFLR
XP_020874560.1/1-283 GFT--SMPSA-VLCAKASML--------------------------LQNVPPSILLRFLR
XP_020874561.1/1-283 GFT--SMPSA-VLCAKASML--------------------------LQNVPPSILLRFLR
XP_020874562.1/1-283 GFT--SMPSA-VLCAKASML--------------------------LQNVPPSILLRFLR
NP_195014.1/1-283    GYT--SMPSA-VLCAKASML--------------------------LQNVPPSILLRFLR
XP_006412419.1/1-283 GFT--SMPSA-VLCAKASML--------------------------LQNVPPSILLRFLR
XP_006412418.1/1-283 GFT--SMPSA-VLCAKASML--------------------------LQNVPPSILLRFLR
XP_018471819.1/1-283 GFT--SMPSA-VLCAKASML--------------------------LQNVPPSILLRFLR
XP_009125299.1/1-283 GFT--SMPSA-VLCAKASML--------------------------LQNVPPSILLRFLR
XP_010432643.1/1-283 GFT--SMPSA-VLCAKASML--------------------------LQNVPPSILLRFLR
XP_010432644.1/1-283 GFT--SMPSA-VLCAKASML--------------------------LQNVPPSILLRFLR
XP_006283109.1/1-283 GFT--SMPSA-VLCAKASML--------------------------LQNVPPSILLRFLR
XP_010437839.1/1-283 GFT--SMPSA-VLCAKASML--------------------------LQNVPPSILLRFLR
XP_010437840.1/1-283 GFT--SMPSA-VLCAKASML--------------------------LQNVPPSILLRFLR
XP_010447325.1/1-283 GFT--SMPSA-VLCAKASML--------------------------LQNVPPSILLRFLR
XP_010447326.1/1-283 GFT--SMPSA-VLCAKASML--------------------------LQNVPPSILLRFLR
XP_013622315.1/1-283 GFT--SMPSA-VLCAKASML--------------------------LQNVPPSILLRFLR
XP_013656273.1/1-283 GFT--SMPSA-VLCAKASML--------------------------LQNVPPSILLRFLR
XP_009108824.1/1-283 GFT--SMPSA-VLCAKASML--------------------------LQNVPPSILLRFLR
XP_013730326.1/1-283 GFT--SMPSA-VLCAKASML--------------------------LQNVPPSILLRFLR
XP_013730325.1/1-283 GFT--SMPSA-VLCAKASML--------------------------LQNVPPSILLRFLR
XP_018447492.1/1-283 GFT--SMPSA-VLCAKASML--------------------------LQNVPPSILLRFLR
XP_019415259.1/1-283 GFP--SVTSS-ILCAKASIL--------------------------LQNVPPVILLRFLR
XP_022980940.1/1-286 GFP--SMSNA-VLCAKASML--------------------------LQNVTPAMLIRFLR
XP_023523539.1/1-286 GFP--SMSNA-VLCAKASML--------------------------LQNVTPAMLIRFLR
XP_022941184.1/1-286 GFP--SMSNA-VLCAKASML--------------------------LQNVTPAMLIRFLR
XP_022134366.1/1-286 GFP--SMSNA-VLCAKASML--------------------------LQNVTPAMLIRFLR
XP_004136224.1/1-286 GFP--SMSNA-VLCAKASML--------------------------LQNVTPAMLIRFLR
XP_008466070.1/1-286 GFP--SMSNA-VLCAKASML--------------------------LQNVTPAMLIRFLR
XP_022976315.1/1-286 GFP--SMSNA-VLCAKASML--------------------------LQNVTPAMLIRFLR
XP_022937345.1/1-286 GFP--SMSNA-VLCAKASML--------------------------LQNVTPAMLIRFLR
XP_023534956.1/1-286 GFP--SMSNA-VLCAKASML--------------------------LQNVTPAMLIRFLR
XP_023535295.1/1-286 GFP--SMSNA-VLCAKASML--------------------------LQNVTPAMLIRFLR
XP_019175908.1/1-285 GLP--PMSGA-VLCAKASML--------------------------LQNVPPAILLRFLR
XP_019175907.1/1-285 GLP--PMSGA-VLCAKASML--------------------------LQNVPPAILLRFLR
XP_009626659.1/1-285 GFP--SMSSA-VLCAKASML--------------------------LQNVPPVILLRFLR
XP_019266312.1/1-285 GFP--SMSSA-VLCAKASML--------------------------LQNVPPAILLRFLR
XP_009781184.1/1-285 GFP--SMSSA-VLCAKASML--------------------------LQNVPPAILLRFLR
XP_016488110.1/1-285 GFP--SMSSA-VLCAKASML--------------------------LQNVPPAILLRFLR
XP_016537761.1/1-285 GFP--SMSSA-VLCAKASML--------------------------LQNVPPSILLRFLR
XP_006359741.1/1-285 GFP--SMSSA-VLCAKASML--------------------------LQNVPPAILLRFLR
XP_010325078.1/1-288 GFP--SMSSA-VLCAKASML--------------------------LQNVPPPILLRFLR
XP_004245155.1/1-285 GFP--SMSSA-VLCAKASML--------------------------LQNVPPPILLRFLR
XP_015085523.1/1-285 GFP--SMSSA-VLCAKASML--------------------------LQNVPPPILLRFLR
XP_022868182.1/1-285 GLP--SMSTS-VLCAKASML--------------------------LQNVPPAILLRFLR
XP_022868181.1/1-285 GLP--SMSTS-VLCAKASML--------------------------LQNVPPAILLRFLR
XP_022854559.1/1-285 GFP--SMITS-VLCAKASML--------------------------LQNVPPAILLRFLR
XP_011081776.1/1-285 GFP--SMSNA-VLCAKASML--------------------------LQNVPPAILLRFLR
XP_012857948.1/1-282 GFP--SMSNA-VLCAKASML--------------------------LKNVPPAILLRFLR
XP_021847759.1/1-284 GFP--SAGTA-VLCARASML--------------------------LQNVPPAVLLRFLR
XP_010668539.1/1-284 GFP--SVGTA-VLCARASML--------------------------LQNVPPAVLLRFLR
XP_021739137.1/1-284 GFP--SVGTA-VLCARASML--------------------------LQNVPPAVLLRFLR
XP_021737291.1/1-284 GFP--SVGTA-VLCARASML--------------------------LQNVPPAVLLRFLR
XP_010047286.1/1-285 GFP--SMTSA-VLCAKASML--------------------------LQNVPPAILLRFLR
XP_003603630.2/1-285 GFP--SVTSS-VLCAKASML--------------------------LQNVPPAILLRFLR
XP_004501146.1/1-286 GFP--SMTSS-VLCAKASML--------------------------LQNVPPAILLRFLR
XP_012571693.1/1-286 GFP--SMTSS-VLCAKASML--------------------------LQNVPPAILLRFLR
XP_012571692.1/1-286 GFP--SMTSS-VLCAKASML--------------------------LQNVPPAILLRFLR
XP_019417530.1/1-285 GFP--SVTSS-ILCAKASML--------------------------LQNVPPALLVRFLR
XP_019417531.1/1-285 GFP--SVTSS-ILCAKASML--------------------------LQNVPPALLVRFLR
XP_016180268.1/1-286 GFA--SVTSS-VLCAKASML--------------------------LQNVPPAILLRFLR
XP_025623723.1/1-286 GFA--SVTSS-VLCAKASML--------------------------LQNVPPAILLRFLR
XP_015945672.1/1-286 GFA--SVTSS-VLCAKASML--------------------------LQNVPPAILLRFLR
XP_007137231.1/1-287 GFP--SVSSS-VLCAKASML--------------------------LQNVPPAILLRFLR
XP_022636494.1/1-293 GFP--SMSSS-VLCAKASML--------------------------LQNVPPAILLRFLR
XP_017409217.1/1-287 GFP--SMSSS-VLCAKASML--------------------------LQNVPPAILLRFLR
XP_014501941.1/1-287 GFP--SMSSS-VLCAKASML--------------------------LQNVPPAILLRFLR
XP_003526496.1/1-286 GFP--SVSSS-VLCAKASML--------------------------LQNVPPAILLRFLR
XP_003522716.1/1-286 GFP--SVSSS-LLCAKASML--------------------------LQNVPPAILLRFLR
XP_020217689.1/1-286 GFP--SVSSS-VLCAKASML--------------------------LQNVPPAILLRFLR
XP_016683069.1/1-283 GFP--SMGNA-VLCAKASML--------------------------LQNVPPAILLRFLR
XP_016677926.1/1-283 GFP--SMGNA-VLCAKASML--------------------------LQNVPPAILLRFLR
XP_017643110.1/1-283 GFP--SMGNA-VLCAKASML--------------------------LQNVPPAILLRFLR
XP_017643111.1/1-283 GFP--SMGNA-VLCAKASML--------------------------LQNVPPAILLRFLR
XP_012451775.1/1-283 GFP--SMGNA-VLCAKASML--------------------------LQNVPPAILLRFLR
XP_016744766.1/1-285 GFP--SVGTA-VLCAKASML--------------------------LQNVPPAILLRFLR
XP_017643921.1/1-285 GFP--SVGTA-VLCAKASML--------------------------LQNVPPAILLRFLR
XP_012436119.1/1-285 GFP--SVGTA-VLCAKASML--------------------------LQNVPPAILLRFLR
XP_016681632.1/1-285 GFP--SVGTA-VLCAKASML--------------------------LQNVPPAILLRFLR
XP_016689618.1/1-285 GFP--SMGNA-ILCAKASML--------------------------LQNVPPAILLRFLR
XP_016689617.1/1-285 GFP--SMGNA-ILCAKASML--------------------------LQNVPPAILLRFLR
XP_012445947.1/1-285 GFP--SMGNA-ILCAKASML--------------------------LQNVPPAILLRFLR
XP_012445944.1/1-285 GFP--SMGNA-ILCAKASML--------------------------LQNVPPAILLRFLR
XP_012445945.1/1-285 GFP--SMGNA-ILCAKASML--------------------------LQNVPPAILLRFLR
XP_017614652.1/1-285 GFP--SMGNA-ILCAKASML--------------------------LQNVPPAILLRFLR
XP_016732732.1/1-285 GFP--SMGNA-ILCAKASML--------------------------LQNVPPAILLRFLR
XP_016732731.1/1-285 GFP--SMGNA-ILCAKASML--------------------------LQNVPPAILLRFLR
XP_022754596.1/1-285 GFP--SMGNA-VLCAKASML--------------------------LQNVPPAILLRFLR
XP_022750159.1/1-285 GFP--SMGNA-VLCAKASML--------------------------LQNVPPAILLRFLR
XP_022729737.1/1-285 GFP--SMGSA-VLCAKASML--------------------------LQNVPPAILLRFLR
XP_022729738.1/1-285 GFP--SMGSA-VLCAKASML--------------------------LQNVPPAILLRFLR
XP_021276132.1/1-285 GFP--SMGNA-VLCAKASML--------------------------LQNVPPAILLRFLR
XP_017983822.1/1-285 GFP--SMGNA-VLCAKASML--------------------------LQNVPPAILLRFLR
XP_002283717.3/1-285 GFP--SMSNA-VLCAKASML--------------------------LQNVPPAILLRFLR
XP_024446213.1/1-284 GFP--SMNNA-VLCAKASML--------------------------LQNVPPAILLRFLR
XP_024446215.1/1-284 GFP--SMNNA-VLCAKASML--------------------------LQNVPPAILLRFLR
XP_011006428.1/1-284 GFP--SMNNA-VLCAKASML--------------------------LQNVPPAILLRFLR
XP_024459734.1/1-284 GFP--SMSNA-VLCAKASML--------------------------LQNVPPAILLRFLR
XP_011019595.1/1-284 GFP--SMSNA-VLCAKASML--------------------------LQNVPPAILLRFLR
XP_015573174.1/1-284 GFP--AMSNG-VLCAKASML--------------------------LQNVPPAILLRFLR
XP_012077376.1/1-284 GFP--SMSNG-VLCAKASML--------------------------LQNVPPAILLRFLR
XP_021608403.1/1-284 GFP--SMSNG-VLCAKASML--------------------------LQNVPPAILLRFLR
XP_021608402.1/1-284 GFP--SMSNG-VLCAKASML--------------------------LQNVPPAILLRFLR
XP_021597909.1/1-284 GFP--SMSNG-VLCAKASML--------------------------LQNVPPAILLRFLR
XP_021642839.1/1-284 GFP--SMSNG-VLCAKASML--------------------------LQNVPPAILLRFLR
XP_021689146.1/1-284 GFP--SMSNG-VLCAKASML--------------------------LQNVPPAILLRFLR
XP_018805752.1/1-285 GFP--SMSNA-VLCAKASML--------------------------LQNVPPAVLLRFLR
XP_023918167.1/1-285 GFQ--SMSNA-VLCAKASML--------------------------LQNVPPAILLRFLR
XP_018845499.1/1-285 GFP--SVSNA-VLCAKASML--------------------------VQNVPPAVLLRFLR
XP_018845500.1/1-285 GFP--SVSNA-VLCAKASML--------------------------VQNVPPAVLLRFLR
NP_001280750.1/1-285 GVP--SMSTA-VLCAKASML--------------------------LQNVPPAILLRFLR
NP_001280796.1/1-285 GVP--SMSTA-VLCAKASML--------------------------LQNVPPAILLRFLR
XP_009367440.1/1-285 GVP--SMSTA-VLCAKASML--------------------------LQNVPPAILLRFLR
XP_017182108.1/1-285 GVP--SMSTA-XLCAKASML--------------------------LQNVPPAILLRFLR
XP_004291167.1/1-285 GVP--SMSNA-VLCAKASML--------------------------LQNVPPAILLRFLR
XP_024162655.1/1-285 GVP--SMSNA-VLCAKASML--------------------------LQNVPPAILLRFLR
XP_021819449.1/1-284 GVP--SMSNA-VLCAKASML--------------------------LQNVPPAILLRFLR
XP_007225281.1/1-285 GVP--SMSNA-VLCAKASML--------------------------LQNVPPAILLRFLR
XP_008220092.1/1-285 GVP--SMSNA-VLCAKASML--------------------------LQNVPPAILLRFLR
XP_006450706.1/1-285 GFP--SMSNA-VLCAKASML--------------------------LQDVPPAILLRFLR
XP_006476026.1/1-285 GFP--SMSNA-VLCAKASML--------------------------LQDVPPAILLRFLR
XP_024930942.1/1-285 GFP--SVSNA-VLCAKASML--------------------------LQNVPPAILLRFLR
XP_010102406.2/1-285 GFP--SASNS-VLCAKASML--------------------------LQNVPPAILLRFLR
XP_024540686.1/1-258 --DRLSSLAG-VLCAKASML--------------------------LQNVHPSYLIRFLR
XP_024399220.1/1-283 DKLLFSLGGG-ILCAKASML--------------------------LQNVPPALLIRFLR
XP_024399219.1/1-283 DKLLFSLGGG-ILCAKASML--------------------------LQNVPPALLIRFLR
XP_024399215.1/1-283 DKLLFSLGGG-ILCAKASML--------------------------LQNVPPALLIRFLR
XP_024390746.1/1-284 DKLLYSLGGG-ILCAKASML--------------------------LQNVPSSLLIRFLR
XP_024390752.1/1-284 DKLLYSLGGG-ILCAKASML--------------------------LQNVPSSLLIRFLR
XP_024373354.1/1-283 DKLLYSLGGG-ILCAKASML--------------------------LQNVPPSLLIRFLR
XP_024373357.1/1-283 DKLLYSLGGG-ILCAKASML--------------------------LQNVPPSLLIRFLR
XP_024370817.1/1-284 DKLLYSLGGG-ILCAKASML--------------------------LQNVSPSLLIGFLR
XP_024370815.1/1-284 DKLLYSLGGG-ILCAKASML--------------------------LQNVSPSLLIGFLR
XP_024370814.1/1-284 DKLLYSLGGG-ILCAKASML--------------------------LQNVSPSLLIGFLR
XP_024386785.1/1-285 DKLLYSLGGG-ILCAKASML--------------------------LQNVPPSILIKFLR
XP_024386782.1/1-285 DKLLYSLGGG-ILCAKASML--------------------------LQNVPPSILIKFLR
XP_024386791.1/1-285 DKLLYSLGGG-ILCAKASML--------------------------LQNVPPSILIKFLR
XP_002961227.1/1-277 PDRLYAVGGG-VLCAKASML--------------------------LQNVPPATLIRFLR
XP_002969458.1/1-277 PDRLYAVGGG-VLCAKASML--------------------------LQNVPPATLIRFLR
XP_024533017.1/1-265 GGDRFCAFGG-ILCAKSSML--------------------------LQNVPAATLIRFLR
XP_011076983.1/1-261 -------YDS-VICMKASLL--------------------------LQKVLPAKLMMLLK
XP_011076980.1/1-264 -------YDS-VICMKASLL--------------------------LQKVLPAKLMMLLK
XP_011076981.1/1-263 -------YDS-VICMKASLL--------------------------LQ-VLPAKLMMLLK
XP_011084330.1/1-264 -------YDS-IICVKASLL--------------------------LQNVIPASLVRLLK
XP_011084333.1/1-264 -------YDS-IICVKASLL--------------------------LQNVIPASLVRLLK
XP_011084331.1/1-264 -------YDS-IICVKASLL--------------------------LQNVIPASLVRLLK
XP_012834933.1/1-260 -------SDT-VICVKASCL--------------------------LHNIVPASMVQLLK
XP_012834932.1/1-260 -------SDT-VICVKASCL--------------------------LHNIVPASMVQLLK
XP_012834931.1/1-260 -------SDT-VICVKASCL--------------------------LHNIVPASMVQLLK
XP_012834934.1/1-260 -------SDT-VICVKASCL--------------------------LHNIVPASMVQLLK
XP_002274194.1/1-284 STAALSLPGG-ILCVKAAML--------------------------LQNVSPSLMVRFLR
XP_019079956.1/1-284 STAALSLPGG-ILCVKAAML--------------------------LQNVSPSLMVRFLR
XP_023759583.1/1-283 GIPV--VSNA-VLCAKASML--------------------------LQDVAPAILMRFLR
XP_020087984.1/1-275 EFS---MCNA-VLCAKASML--------------------------LQNVSPAMLLRFLR
XP_015693848.1/1-289 GLPM--VSTG-VLCAKASML--------------------------LQDVSPPALLQFLR
XP_015624690.1/1-290 GLPI--VSTG-VLCAKASML--------------------------LQDVSPPSLLQFLR
XP_008655743.1/1-292 GPVVGTGSSS-VLCAKASML--------------------------LQDVSPPELLRFMR
XP_020183693.1/1-281 GLPM--VSTG-VLCAKASML--------------------------LQDVSPPSLLRFLR
XP_020183687.1/1-281 GLPM--VSTG-VLCAKASML--------------------------LQDVSPPSLLRFLR
XP_003565084.1/1-284 GLPI--VSAG-VLCAKASML--------------------------LQDVSPPSLLQFLH
XP_002454995.1/1-286 GLPV--VSSS-VLCAKASML--------------------------LQDVSPPALLRFMR
XP_012701181.1/1-286 GLPI--VSSS-VLCAKASML--------------------------LQDVSPPALLRFMR
XP_012701180.1/1-286 GLPI--VSSS-VLCAKASML--------------------------LQDVSPPALLRFMR
XP_012701179.1/1-286 GLPI--VSSS-VLCAKASML--------------------------LQDVSPPALLRFMR
XP_025816436.1/1-287 GLPI--VSSS-VLCAKASML--------------------------LQDVSPPALLRFMR
XP_018726848.1/1-227 --P----PGL-VLSAATSFW--------------------------LPV-LPNRVFDFLC
XP_018726849.1/1-227 --P----PGL-VLSAATSFW--------------------------LPV-LPNRVFDFLC
XP_019193052.1/1-216 --P----SGL-VLSASTSFW--------------------------LPT-QPKRVFDFLR
XP_019193050.1/1-216 --P----SGL-VLSASTSFW--------------------------LPT-QPKRVFDFLR
XP_021834047.1/1-194 -----------SL-----------------------------------------------
XP_010522330.1/1-229 -----------VGRVCMYIC--------------------------IPV-PPKRVFDFLR
XP_009802524.1/1-219 --P----PGI-VMCAATSFW--------------------------LPI-SAKTVFDFLR
XP_009802526.1/1-219 --P----PGI-VMCAATSFW--------------------------LPI-SAKTVFDFLR
XP_009802525.1/1-219 --P----PGI-VMCAATSFW--------------------------LPI-SAKTVFDFLR
XP_009802527.1/1-219 --P----PGI-VMCAATSFW--------------------------LPI-SAKTVFDFLR
XP_019250148.1/1-219 --P----PGI-VMCAATTFW--------------------------LPI-SPKIVFDFLR
XP_019250149.1/1-219 --P----PGI-VMCAATTFW--------------------------LPI-SPKIVFDFLR
XP_016449923.1/1-219 --P----PGI-VMCAATSFW--------------------------LPI-SPKTVFDFLR
XP_009629760.1/1-219 --P----PGI-VMCAATSFW--------------------------LPI-SPKTAFDFLR
XP_009629762.1/1-219 --P----PGI-VMCAATSFW--------------------------LPI-SPKTAFDFLR
XP_010234287.2/1-230 --P----PGI-ILNAATSFW--------------------------LPV-SPAEVFAFLR
XP_021806177.1/1-204 --P----PGI-VLSAATSFW--------------------------LPV-PPKRVFEFLR
XP_016580642.1/1-229 --P----PGV-VLSAATSFW--------------------------LPV-PPKMVFDYLR
XP_008667763.1/1-266 --P----PGI-VLNAATSFW--------------------------LPI-TPKRVFDFLR
XP_006352040.1/1-227 --P----HGV-VISAATSFW--------------------------LPI-SPKTVFDYLR
XP_009611638.1/1-223 --P----PGI-VLSAATSFW--------------------------LPI-SPKRVFDFLR
XP_009611623.1/1-223 --P----PGI-VLSAATSFW--------------------------LPI-SPKRVFDFLR
XP_016510654.1/1-223 --P----PGI-VLSAATSFW--------------------------LPI-SPKRVFDFLR
XP_009766038.1/1-223 --P----PGI-VLSAATSFW--------------------------LPI-SPKRVFDFLR
XP_020210648.1/1-236 --P----PGI-VLSAATSLW--------------------------LPV-PPTRVFHFLR
XP_007143210.1/1-219 --P----SGI-VLSAATSLW--------------------------LPL-PSTTVFSFLR
XP_014491869.1/1-226 --P----SGI-VLSASTSFW--------------------------LPI-PSRTVFDFLR
XP_022633652.1/1-226 --P----SGI-VLSASTSFW--------------------------LPI-PSRTVFDFLR
XP_017412341.1/1-227 --P----SGI-VLSASTSLW--------------------------LPI-PSRTVFDFLR
XP_022011155.1/1-226 --P----TGL-VLSAATSFW--------------------------LPV-PPRQSFNYLH
XP_003555330.2/1-228 --P----PGI-VLSAATSLW--------------------------LPV-PARRVFEFLR
XP_014628518.1/1-229 --P----PGI-VLSAATSLW--------------------------LPV-PARRVFEFLR
XP_017647009.1/1-225 --P----PGI-VLSAATSFW--------------------------VAV-PPRKAFNILR
XP_016711277.1/1-225 --P----PGI-VLSAATSFW--------------------------VAV-PPRKAFNILR
XP_012457027.1/1-225 --P----PGI-VLSAATSFW--------------------------VAV-PPRKAFNILR
XP_016723454.1/1-225 --P----PGI-VLSAATSFW--------------------------VAV-PPRKAFNILR
XP_020978973.1/1-214 --P----PGI-VLCAATSFS--------------------------LPV-PPNTLFHFLR
XP_016202355.1/1-214 --P----PGI-VLCAATSFS--------------------------LPV-PPNTLFHFLR
XP_015964876.1/1-214 --P----PGI-VLCAATSFS--------------------------LPV-PPNTLFHFLR
XP_025699645.1/1-214 --P----PGI-VLCAATSFS--------------------------LPV-PPNTLFHFLR
XP_008441375.1/1-228 --P----PGI-VLNAATSFW--------------------------IPI-PPNRVFNFLR
XP_004154226.2/1-228 --P----PGI-VLNAATSFW--------------------------IPI-PPNRVFNFLR
XP_023519981.1/1-226 --P----PGI-VLNAATSFW--------------------------IPI-PPNRVFHFLR
XP_023001231.1/1-226 --P----PGI-VLNAATSFW--------------------------IPI-PPNRVFHFLR
XP_022933178.1/1-226 --P----PGI-VLNAATSFW--------------------------IPI-PPNRVFHFLR
XP_004511340.1/1-228 --P----TGV-VLSTVTSFW--------------------------LPV-PPKRLFDFLR
XP_002979015.2/1-232 --P----HGI-VLSAATSLW--------------------------LPV-PPKRVFEFLR
XP_002988402.1/1-232 --P----HGI-VLSAATSLW--------------------------LPV-PPKRVFEFLR
XP_024390340.1/1-238 --P----QGI-VLSAATSMW--------------------------LAV-SAARVFEFLR
XP_024368074.1/1-235 --P----HGI-VLSAATSMW--------------------------LPV-SPARVFQFLR
XP_024401280.1/1-235 --P----HGI-VLSAATSMW--------------------------LPV-SPARVFQFLR
XP_024394840.1/1-235 --P----HGI-VLSAATSMW--------------------------LPV-SPARVFQFLR
XP_024394838.1/1-235 --P----HGI-VLSAATSMW--------------------------LPV-SPARVFQFLR
XP_017256650.1/1-227 --P----PGI-VLSAATSFW--------------------------LPV-PPKAVFDFLR
XP_019186546.1/1-227 --P----PGI-VLSAATSFF--------------------------LPL-PPKTVFDFLR
XP_024988375.1/1-226 --P----AGI-VLCAATSFW--------------------------LPV-PPKQVFNFLR
XP_017231769.1/1-226 --P----AGI-VLSAATSFW--------------------------IPV-APKRVFDFLR
XP_009108529.1/1-235 --P----PGI-VLSAATSFW--------------------------IPM-GLKRVFDFLR
XP_018469534.1/1-236 --S----PGI-VLSAATSFW--------------------------IPV-APKRVFNFLR
XP_018467466.1/1-236 --P----PGI-VLSAATSFW--------------------------IPV-APKRVFDFLR
XP_022543253.1/1-234 --P----PGI-VLSAATSFW--------------------------IPV-APKRVFDFLR
XP_009135181.1/1-234 --P----PGI-VLSAATSFW--------------------------IPV-APKRVFDFLR
XP_013642975.1/1-234 --P----PGI-VLSAATSFW--------------------------IPV-APKRVFDFLR
XP_009135189.1/1-234 --P----PGI-VLSAATSFW--------------------------IPV-APKRVFDFLR
XP_018515487.1/1-234 --P----PGI-VLSAATSFW--------------------------IPV-APKRVFDFLR
XP_013623861.1/1-234 --P----PGI-VLSAATSFW--------------------------IPV-APKRVFDFLK
XP_013623905.1/1-234 --P----PGI-VLSAATSFW--------------------------IPV-APKRVFDFLK
XP_013623953.1/1-236 --P----PGI-VLSAATSFW--------------------------IPV-APKRVFDFLK
XP_013623587.1/1-236 --P----PGI-VLSAATSFW--------------------------IPV-APKRVFDFLK
XP_013623995.1/1-236 --P----PGI-VLSAATSFW--------------------------IPV-APKRVFDFLK
XP_013623820.1/1-236 --P----PGI-VLSAATSFW--------------------------IPV-APKRVFDFLK
XP_022926381.1/1-227 --P----PGV-VLSAATSFW--------------------------IPV-SPKVVFDFLR
XP_023517485.1/1-227 --P----PGV-VLSAATSFW--------------------------IPV-SPKVVFDFLR
XP_023002930.1/1-227 --P----PGV-VLSAATSFW--------------------------IPV-SPKVVFDFLR
XP_004134971.1/1-229 --P----PGV-VLSAATSFW--------------------------IPV-SPKVVFDFLR
XP_008439968.1/1-229 --P----PGV-VLSAATSFW--------------------------IPV-SPKVVFDFLR
XP_022950176.1/1-227 --P----PGV-VLSAATSFW--------------------------IPV-PPKVVFDFLR
XP_022979090.1/1-227 --P----PGV-VLSAATSFW--------------------------IPV-PPKVVFDFLR
XP_023543388.1/1-249 --P----PGV-VLSAATSFW--------------------------IPV-PPKAVFDFLR
XP_023543387.1/1-227 --P----PGV-VLSAATSFW--------------------------IPV-PPKAVFDFLR
XP_009134323.1/1-228 --P----PGI-VLSAATSFW--------------------------IPV-APKRVFDFLR
XP_019176002.1/1-227 --P----PGI-VLSAATSFW--------------------------LPV-PPKRVFDFLR
XP_015070655.1/1-221 --P----PGI-VLSAATSFW--------------------------LPV-PPKRVFDFLR
XP_015070652.1/1-221 --P----PGI-VLSAATSFW--------------------------LPV-PPKRVFDFLR
XP_025885589.1/1-221 --P----PGI-VLSAATSFW--------------------------LPV-PPKRVFDFLR
XP_019422236.1/1-227 --P----PGI-VLSAATSFW--------------------------LSV-PPKTVFDFLR
XP_007157166.1/1-226 --P----PGI-VLSAATSFW--------------------------LPV-PPKKVFDFLR
XP_003538765.1/1-227 --P----PGI-VLSAATSFW--------------------------LPV-PPNRVFDFLR
XP_003517589.1/1-227 --P----PGI-VLSAATSFW--------------------------LPV-PPKRVFHFLR
XP_006573890.1/1-227 --P----PGI-VLSAATSFW--------------------------LPV-PPKRVFHFLR
XP_011000687.1/1-226 --P----PGI-VLSAATSFW--------------------------IPV-PSKKVFDFLK
XP_024436817.1/1-226 --P----PGI-VLSAATSFW--------------------------IPV-PSKKVFDFLK
XP_024436820.1/1-226 --P----PGI-VLSAATSFW--------------------------IPV-PSKKVFDFLK
XP_002518520.2/1-226 --P----PGI-VLCAATSFW--------------------------LPV-PPKRVFQFLS
XP_015574304.1/1-226 --P----PGI-VLCAATSFW--------------------------LPV-PPKRVFQFLS
XP_021642058.1/1-227 --P----PGI-VLSAATSFW--------------------------IPV-PPKRVFEFLS
XP_012091395.1/1-226 --P----PGI-VLSAATSFW--------------------------IPV-PPKKVFEFLS
XP_021635824.1/1-226 --P----PGI-VLSAATSFW--------------------------IPV-PPKRVFAFLS
XP_021596887.1/1-226 --P----PGI-VLSAATSFW--------------------------IPV-PPKRVFAFLS
XP_010057969.1/1-228 --P----PGI-VLSAATSFW--------------------------LPV-PPKRVFDFLR
XP_006413738.1/1-245 --P----PGI-VLSAATSFW--------------------------LPV-SPKRVFDFLR
XP_013630219.1/1-235 --P----PGI-VLSAATSFW--------------------------IPV-GPKRVFDFLR
XP_013656136.1/1-235 --P----PGI-VLSAATSFW--------------------------IPM-GPKRVFDFLR
XP_013685757.1/1-235 --P----PGI-VLSAATSFW--------------------------IPV-GPKRVFDFLR
XP_013696499.1/1-240 --P----PGI-VLSAATSFW--------------------------IPV-APKRVFDFLR
XP_009137213.1/1-240 --P----PGI-VLSAATSFW--------------------------IPV-APKRVFDFLR
XP_013598423.1/1-241 --P----PGI-VLSAATSFW--------------------------IPV-APKRVFDFLR
XP_013717184.1/1-241 --P----PGI-VLSAATSFW--------------------------IPV-APKRVFDFLR
XP_018480642.1/1-241 --P----PGI-VLSAATSFW--------------------------IPV-APKRVFDFLR
XP_018477464.1/1-241 --P----PGI-VLSAATSFW--------------------------IPV-APKRVFDFLR
NP_193906.2/1-248    --P----PGI-VLSAATSFW--------------------------IPV-APKRVFDFLR
XP_020873301.1/1-248 --P----PGI-VLSAATSFW--------------------------IPV-APKRVFDFLR
XP_006285476.1/1-249 --P----PGI-VLSAATSFW--------------------------IPV-APKRVFDFLR
XP_010434132.1/1-252 --P----PGI-VLSAATSFW--------------------------IPV-APKRVFDFLR
XP_010439429.1/1-252 --P----PGI-VLSAATSFW--------------------------IPV-APKRVFDFLR
XP_010449009.1/1-254 --P----PGI-VLSAATSFW--------------------------IPV-APKRVFDFLR
XP_010432053.1/1-234 --P----PGI-VLSAATSFW--------------------------IPV-APKRVFDFLR
XP_020876375.1/1-234 --P----PGI-VLSAATSFW--------------------------IPV-APKRVFDFLR
XP_023636874.1/1-234 --P----PGI-VLSAATSFW--------------------------IPV-APKRVFDFLR
XP_010422328.1/1-234 --P----PGI-VLSAATSFW--------------------------IPV-APKRVFDFLR
XP_010455798.1/1-234 --P----PGI-VLSAATSFW--------------------------IPV-APKRVFDFLR
NP_567274.1/1-235    --P----PGI-VLSAATSFW--------------------------IPV-APKRVFDFLR
XP_024009884.1/1-236 --P----PGI-VLSAATSFW--------------------------IPV-APKRVFDFLR
XP_018452346.1/1-236 --P----PGI-VLSAATSFW--------------------------IPV-APKRVFDFLR
XP_013611160.1/1-236 --P----PGI-VLSAATSFW--------------------------IPV-APKRVFDFLR
XP_009114536.1/1-235 --P----PGI-VLSAATSFW--------------------------IPV-APKRVFDFLR
XP_013662000.1/1-235 --P----PGI-VLSAATSFW--------------------------IPV-APKRVFDFLR
XP_018472664.1/1-233 --P----PGI-VLSAATSFW--------------------------IPV-APKRVFDFLR
XP_022555047.1/1-234 --P----PGI-VLSAATSFW--------------------------IPV-APKRVFDFLR
XP_013735469.1/1-234 --P----PGI-VLSAATSFW--------------------------IPV-APKRVFDFLR
XP_013629800.1/1-234 --P----PGI-VLSAATSFW--------------------------IPV-APKRVFDFLR
XP_013735464.1/1-234 --P----PGI-VLSAATSFW--------------------------IPV-APKRGFDFLR
XP_022571893.1/1-234 --P----PGI-VLSAATSFW--------------------------IPV-APKRGFDFLR
XP_010538642.1/1-236 --P----PGI-VLSAATSFW--------------------------IPV-PPKRVFDFLR
XP_019057303.1/1-229 --P----PGI-VLSAATSFW--------------------------IPV-SPKRIFDFLR
XP_016545426.1/1-226 --P----PGI-VLSAATSFW--------------------------LPV-PTKRIFDFLR
XP_009793614.1/1-227 --P----PGI-VLSAATSFW--------------------------LPV-PTKRVFDFLR
XP_016508926.1/1-227 --P----PGI-VLSAATSFW--------------------------LPV-PTKRVFDFLR
XP_016560096.1/1-235 --P----PGI-VLSAATSFW--------------------------IPV-SPKRVFDFLR
XP_021993721.1/1-227 --P----PGI-VLSAATSFW--------------------------IPV-PPKRVFEFLR
XP_022036928.1/1-226 --P----PGI-VLSAATSFW--------------------------LPI-PPKQVFEFLR
XP_023742870.1/1-226 --P----SGI-VLSAATSFW--------------------------LPV-HPKQVFNFLR
XP_006352605.1/1-223 --P----PGI-VLSAATSFW--------------------------MPV-PTKRVFDFLR
XP_010327237.1/1-223 --P----PGI-VLSAATSFW--------------------------MPV-PTKRVFDFLR
XP_015055984.1/1-223 --P----PGI-VLSAATSFW--------------------------MPV-PTKRVFDFLR
XP_009609606.1/1-229 --P----PGI-VLSAATSFW--------------------------LPV-PTKRVFDFLR
XP_009609608.1/1-229 --P----PGI-VLSAATSFW--------------------------LPV-PTKRVFDFLR
XP_018628742.1/1-229 --P----PGI-VLSAATSFW--------------------------LPV-PTKRVFDFLR
XP_019237831.1/1-229 --P----PGI-VLSAATSFW--------------------------LPV-PTKRVFDFLR
XP_018849836.1/1-227 --P----PGI-VLSAATSFW--------------------------IPV-PTKRVFDFLR
XP_007214541.1/1-230 --P----PGI-VLSAATSFW--------------------------IPV-PPKRVFDFLR
XP_021821565.1/1-230 --P----PGI-VLSAATSFW--------------------------IPV-PPKRVFDFLR
XP_008224792.1/1-230 --P----PGI-VLSAATSFW--------------------------IPV-PPKRVFDFLR
XP_024198966.1/1-227 --P----PGI-VLSAATSFW--------------------------LPV-PPKRVFDFLR
XP_004293268.1/1-227 --P----PGI-VLSAATSFW--------------------------LPV-PPKRVFDFLR
XP_009364098.1/1-227 --P----PGI-VLSAATSFW--------------------------IPV-PPKRVFDFLR
XP_008371114.1/1-227 --P----PGI-VXSAATSFW--------------------------IPV-PPKRVFDFLR
XP_008384034.1/1-227 --P----PGI-VLSAATSFW--------------------------IPV-PPKRVFDFLR
XP_009340946.1/1-227 --P----PGI-VLSAATSFW--------------------------IPV-PPKRVFNFLR
XP_009357415.1/1-227 --P----PGI-VLSAATSFW--------------------------IPV-PPKRVFNFLR
XP_011037708.1/1-226 --P----PGI-VLSAATSFW--------------------------IPV-QSKRMFDFLR
XP_024454819.1/1-226 --P----PGI-VLSAATSFW--------------------------IPV-QSKRMFDFLR
XP_015884145.1/1-226 --P----PGI-VLSAATSFW--------------------------LPV-QPKRVFDLLR
XP_024024693.1/1-228 --P----PGI-VLSAATSFW--------------------------LPV-PPKRLFDFLR
XP_024024692.1/1-228 --P----PGI-VLSAATSFW--------------------------LPV-PPKRLFDFLR
XP_018820351.1/1-226 --P----PGI-VLSAATSFW--------------------------IPV-PAKRIFDFLR
XP_023904570.1/1-226 --P----PGI-VLSAATSFW--------------------------IPV-PPKRVFDFLR
XP_023904569.1/1-226 --P----PGI-VLSAATSFW--------------------------IPV-PPKRVFDFLR
XP_006449076.1/1-226 --P----PGI-VLSAATSFW--------------------------IPV-PPRRIFDFLR
XP_006468235.1/1-226 --P----PGI-VLSAATSFW--------------------------IPV-PPRRIFDFLR
XP_022721034.1/1-226 --P----PGI-VLSAATSFW--------------------------IPV-PPKRVFDFLR
XP_022721035.1/1-226 --P----PGI-VLSAATSFW--------------------------IPV-PPKRVFDFLR
XP_022724746.1/1-226 --P----PGI-VLSAATSFW--------------------------IPV-PPKRVFDFLR
XP_007026002.1/1-226 --P----PGI-VLSAATSFW--------------------------IPV-PPKRVFDFLR
XP_021294392.1/1-226 --P----PGI-VLSAATSFW--------------------------IPV-PPKRVFDFLR
XP_022777405.1/1-226 --P----PGI-VLSAATSFW--------------------------IPV-PLKRVFDFLR
XP_017615807.1/1-226 --P----PGI-VLSAATSFW--------------------------IPV-PPKRVFDFLR
XP_016753649.1/1-226 --P----PGI-VLSAATSFW--------------------------IPV-PPKRVFDFLR
XP_016683607.1/1-226 --P----PGI-VLSAATSFW--------------------------IQV-PPKRVFDFLR
XP_012451363.1/1-226 --P----PGI-VLSAATSFW--------------------------IQV-PPKRVFDFLR
XP_002266688.1/1-226 --P----PGI-VLSAATSFW--------------------------IPV-PPKRVFDFLR
XP_021853797.1/1-224 --P----PGI-VLSAATSFW--------------------------MPV-SPKRVFDFLR
XP_021719656.1/1-227 --P----PGI-VLSAATSFW--------------------------MPV-SPKRVFDFLR
XP_021766927.1/1-227 --P----PGI-VLSAATSFW--------------------------MPV-SPKRVFDFLR
XP_010665524.1/1-227 --P----PGI-VLSAATSFW--------------------------MPV-SPKRVFDFLR
XP_009605864.1/1-234 --P----PGI-VLSAATSFW--------------------------LPV-SPKRVFDFLR
XP_019223778.1/1-233 --P----PGI-VLSAATSFW--------------------------LPV-SPKRVFDFLR
XP_016440385.1/1-233 --P----PGI-VLSAATSFW--------------------------LPV-SPKRVFDFLR
XP_009790281.1/1-233 --P----PGI-VLSAATSFW--------------------------LPV-SPKRVFDFLR
XP_016570434.1/1-222 --P----PGI-VLSAATSFW--------------------------LPV-SPKRVFDFLR
XP_016486479.1/1-229 --P----PGI-VLSAATSFW--------------------------LPV-PPKRVFDFLR
XP_009597904.1/1-229 --P----PGI-VLSAATSFW--------------------------LPV-PPKRVFDFLR
XP_009799795.1/1-229 --P----PGI-VLSAATSFW--------------------------LPV-PPKRVFDFLR
XP_019249652.1/1-229 --P----PGI-VLSAATSFW--------------------------LPV-PPKRVFDFLR
XP_006343296.1/1-221 --P----PGI-VLSAATSFW--------------------------LPV-PPKRVFDFLR
XP_020217012.1/1-226 --P----PGI-VLSAATSFW--------------------------LPV-PPKRLFDFLR
XP_017406677.1/1-226 --P----PGI-VLSAATSFW--------------------------LPV-PPKRVFDFLR
XP_014519998.1/1-226 --P----PGI-VLSAATSFW--------------------------LPV-PPKRVFDFLR
XP_014519997.1/1-226 --P----PGI-VLSAATSFW--------------------------LPV-PPKRVFDFLR
XP_012841999.1/1-239 --P----PGI-VLSAATSFW--------------------------LPV-PPKTVFDFLR
XP_024984347.1/1-227 --P----PGI-VLSAATSFW--------------------------IPV-PPKRVFDFLR
XP_023751202.1/1-227 --P----PGI-VLSAATSFW--------------------------IPV-PPKRVFDFLR
XP_023751208.1/1-227 --P----PGI-VLSAATSFW--------------------------IPV-PPKRVFDFLR
XP_022041933.1/1-227 --P----PGI-VLSAATSFW--------------------------LPV-PPKQVFDFLR
XP_024988632.1/1-226 --P----PGI-VLSAATSFW--------------------------IPV-QPKRVFDFLR
XP_022018253.1/1-226 --P----PGI-VLSAATSFW--------------------------IPV-QPKRVFEFLR
XP_023771293.1/1-226 --P----PGI-VLSAATSFW--------------------------IPV-QPKRVFDFLR
XP_019170324.1/1-226 --P----PGI-VLSAATSFW--------------------------LPV-PPKRVFDFLR
XP_017230122.1/1-226 --P----PGI-VLSAATSFW--------------------------LPV-PPKRVFDFLR
XP_011096101.1/1-226 --P----PGI-VLSAATSFW--------------------------LPV-PPKRVFDFLR
XP_012848972.1/1-227 --P----PGI-VLSAATSFW--------------------------LPV-PPKRVFDFLR
XP_022876975.1/1-227 --P----PGI-VLSAATSFW--------------------------LPV-PPKRVFDFLR
XP_022863158.1/1-227 --P----PGI-VLSAATSFW--------------------------LPV-PPKRVFDFLR
XP_022877991.1/1-227 --P----PGI-VLSAATSFW--------------------------LPV-QPKRVFDFLR
XP_019223745.1/1-223 --P----PGI-VLSAATSFW--------------------------LPI-SPKRVFDFLR
XP_021800220.1/1-221 --P----PGI-VLSVATSFW--------------------------LPV-PPKRVFEFLR
XP_021684589.1/1-235 --P----PGI-VLSAATSFW--------------------------LPV-PPKRVFDFLR
XP_021684519.1/1-235 --P----PGI-VLSAATSFW--------------------------LPV-PPKRVFDFLR
XP_021684722.1/1-235 --P----PGI-VLSAATSFW--------------------------LPV-PPKRVFDFLR
XP_021633498.1/1-230 --P----PGI-VLSAATSFW--------------------------LPI-PPKRVFDFLR
XP_009420270.1/1-216 --P----PGI-VLNAAKSFW--------------------------LPV-PPKRVFDFLR
XP_003573403.1/1-232 --P----PGI-VLNAATSFW--------------------------LPV-SPNTVFDFLR
XP_006659894.1/1-229 --P----PGI-VLNAATSFW--------------------------LPV-PPAAVFDFLR
XP_015650022.1/1-263 --P----PGI-VLNAATSFW--------------------------LPV-PPAAVFDFLR
XP_015650023.1/1-230 --P----PGI-VLNAATSFW--------------------------LPV-PPAAVFDFLR
XP_020407343.1/1-231 --P----PGI-VLNAATSFW--------------------------LPV-PPKRVFDFLR
NP_001105127.2/1-231 --P----PGI-VLNAATSFW--------------------------LPV-PPKRVFDFLR
XP_008661805.1/1-239 --P----PGI-VLNAATSFW--------------------------LPV-PPKRVFDFLR
NP_001145750.1/1-239 --P----PGI-VLNAATSFW--------------------------LPV-PPKRVFDFLR
XP_025821536.1/1-230 --P----PGI-VLNAATSFW--------------------------LPV-PPKRVFDFLR
XP_002443965.1/1-229 --P----PGI-VLNAATSFW--------------------------LPV-PPKRVFDFLR
XP_004972697.1/1-229 --P----PGI-VLNAATSFW--------------------------LPV-PPKRVFDFLR
XP_008667765.1/1-230 --P----PGI-VLNAATSFW--------------------------LPI-TPKRVFDFLR
NP_001347891.1/1-231 --P----PGI-VLNAATSFW--------------------------LPI-TPKRVFDFLR
XP_015636340.1/1-229 --P----PGI-VLNAATSFW--------------------------LPV-PPKRVFDFLR
XP_015636338.1/1-229 --P----PGI-VLNAATSFW--------------------------LPV-PPKRVFDFLR
XP_006652859.1/1-228 --P----PGI-VLNAATSFW--------------------------LPV-PPKRVFDFLR
XP_003580598.1/1-233 --P----PGI-VLNAATSFW--------------------------LPV-PPKRVFDFLR
XP_020199198.1/1-227 --P----PGI-VLNAATSFW--------------------------LPV-PPKRVFDFLR
XP_020400772.1/1-230 --P----PGI-VLNAATSFW--------------------------LPV-PPKRVFDFLR
NP_001334663.1/1-230 --P----PGI-VLNAATSFW--------------------------LPV-PPKRVFDFLR
XP_008662197.1/1-270 --P----PGI-VLNAATSFW--------------------------LPV-PPKRVFDFLR
XP_008662196.1/1-270 --P----PGI-VLNAATSFW--------------------------LPV-PPKRVFDFLR
XP_025823505.1/1-227 --P----PGI-VLNAATSFW--------------------------LPV-PPKRVFDFLR
XP_004976894.1/1-227 --P----PGI-VLNAATSFW--------------------------LPV-PPKRVFDFLR
XP_002448563.2/1-227 --P----PGI-VLNAATSFW--------------------------LPV-PPKRVFDFLR
XP_019414154.1/1-227 --P----PGI-VLSAATSFW--------------------------LQV-PPKRVFDFLR
XP_006348085.1/1-234 --P----PGI-VLSAATSFW--------------------------LPV-SPKRVFDFLR
XP_004232734.1/1-234 --P----PGI-VLSAATSFW--------------------------LPV-SPKRVFDFLR
XP_015066898.1/1-234 --P----PGI-VLSAATSFW--------------------------LPV-SPKRVFDFLR
XP_003605900.1/1-232 --P----PGI-VLSAATSFW--------------------------LPV-PPTQVFEFLR
XP_022988693.1/1-229 --P----PGI-VLSAATSFW--------------------------LPV-PPKRVFDFLR
XP_022930609.1/1-229 --P----PGI-VLSAATSFW--------------------------LPV-PPKRVFDFLR
XP_023530197.1/1-229 --P----PGI-VLSAATSFW--------------------------LPV-PPKRVFDFLR
XP_016188415.1/1-237 --P----PGI-VLSAATSFW--------------------------LPV-PPKTAFDFLR
XP_011628991.1/1-236 --P----PGI-VLSAATSFW--------------------------LPV-PPKRVFDFLR
XP_006826512.2/1-236 --P----PGI-VLSAATSFW--------------------------LPV-PPKRVFDFLR
XP_020177398.1/1-220 --P----PGI-ILNAATSFW--------------------------LPV-PPSRVFGFLR
XP_015695486.1/1-219 --P----PGI-ILNAATSFW--------------------------LPV-PPSRVFDFLR
XP_015648539.1/1-217 --P----PGI-ILNAATSFW--------------------------LPV-PPSRVFDFLR
XP_020401599.1/1-218 --P----PGI-ILNAATSFW--------------------------LPV-APARVFGFLR
XP_020401598.1/1-218 --P----PGI-ILNAATSFW--------------------------LPV-APARVFGFLR
XP_020401597.1/1-218 --P----PGI-ILNAATSFW--------------------------LPV-APARVFGFLR
XP_021319954.1/1-239 --P----PGI-ILNAATSFW--------------------------LPV-PPARVFGFLR
XP_002445014.2/1-239 --P----PGI-ILNAATSFW--------------------------LPV-PPARVFGFLR
XP_025819913.1/1-218 --P----PGI-ILNAATSFW--------------------------LPV-PPARVFSFLR
XP_004972909.1/1-218 --P----PGI-ILNAATSFW--------------------------LPV-PPARVFGFLR
XP_020100432.1/1-229 --P----PGI-VLNAATSFW--------------------------LPV-SPKRVFDFLR
XP_009385059.1/1-228 --P----PGI-VLNAATSFW--------------------------LPV-PPKRVFDFLR
XP_018678797.1/1-226 --P----PGI-VLNAATSFW--------------------------LPV-PPKRVFDFLR
XP_009412012.1/1-233 --P----PGI-VLNAATSFW--------------------------LPV-PPKRVFDFLR
XP_018685149.1/1-236 --P----PGI-VLNAATSFW--------------------------LPV-PPKRVFDFLR
XP_020591278.1/1-230 --P----PGI-VLNAATSFW--------------------------LPV-SPKRVFDFLR
XP_020702932.1/1-229 --P----PGI-VLNAATSFW--------------------------LPV-SPKRVFDFLR
XP_020702933.1/1-230 --P----PGI-VLNAATSFW--------------------------LPV-SPKRVFDFLR
XP_020702931.1/1-230 --P----PGI-VLNAATSFW--------------------------LPV-SPKRVFDFLR
XP_020692781.1/1-229 --P----PGI-VLNAATSFW--------------------------LPV-APKRVFDFLR
XP_020692780.1/1-229 --P----PGI-VLNAATSFW--------------------------LPV-APKRVFDFLR
XP_020591591.1/1-229 --P----PGI-VLNAATSFW--------------------------LPV-APKRVFDFLR
XP_020591590.1/1-229 --P----PGI-VLNAATSFW--------------------------LPV-APKRVFDFLR
XP_020109359.1/1-227 --P----PGI-VLNAATSFW--------------------------LPV-SPKRVFDFLR
XP_020270114.1/1-229 --P----PGI-VLNAATSFW--------------------------LPV-PPKRVFDFLR
XP_010908855.1/1-227 --P----PGI-VLNAATSFW--------------------------LPV-PPKRVFDFLR
XP_008808987.1/1-227 --P----PGI-VLNAATSFW--------------------------LPV-PPKRVFDFLR
XP_008784552.1/1-223 --P----PGI-VLNAATSFW--------------------------LPV-PPKRVFDFLR
XP_010931820.1/1-223 --P----PGI-VLNAATSFW--------------------------LPV-PPKRVFDFLR
XP_003540308.2/1-226 --P----PGI-VLSAATSFW--------------------------LPV-SPKRVFEFLR
XP_006592818.1/1-226 --P----PGI-VLSAATSFW--------------------------LPV-SPKRVFEFLR
XP_006592816.1/1-226 --P----PGI-VLSAATSFW--------------------------LPV-SPKRVFEFLR
XP_003543387.1/1-226 --P----PGI-VLSAATSFW--------------------------LPV-SPKRVFEFLR
XP_020223651.1/1-226 --P----PGI-VLSAATSFW--------------------------LPV-SPKRVFEFLR
XP_020223648.1/1-226 --P----PGI-VLSAATSFW--------------------------LPV-SPKRVFEFLR
XP_007149968.1/1-226 --P----PGI-VLSAATSFW--------------------------LPV-SPKRVFEFLR
XP_017425582.1/1-226 --P----PGI-VLSAATSFW--------------------------LPV-SPKRVFEFLR
XP_014501634.1/1-226 --P----PGI-VLSAATSFW--------------------------LPV-SPKRVFEFLR
XP_022776632.1/1-226 --P----PGI-VLSAATSFW--------------------------LPV-SPKRVFDFLR
XP_022776631.1/1-226 --P----PGI-VLSAATSFW--------------------------LPV-SPKRVFDFLR
XP_022776629.1/1-226 --P----PGI-VLSAATSFW--------------------------LPV-SPKRVFDFLR
XP_022733641.1/1-226 --P----PGI-VLSAATSFW--------------------------LPV-SPKRVFDFLR
XP_022733640.1/1-226 --P----PGI-VLSAATSFW--------------------------LPV-SPKRVFDFLR
XP_022733638.1/1-226 --P----PGI-VLSAATSFW--------------------------LPV-SPKRVFDFLR
XP_007045612.1/1-226 --P----PGI-VLSAATSFW--------------------------LPV-SPKRVFDFLR
XP_007045611.1/1-226 --P----PGI-VLSAATSFW--------------------------LPV-SPKRVFDFLR
XP_017971478.1/1-226 --P----PGI-VLSAATSFW--------------------------LPV-SPKRVFDFLR
XP_021292769.1/1-226 --P----PGI-VLSAATSFW--------------------------LPV-SPKRVFDFLR
XP_021292767.1/1-226 --P----PGI-VLSAATSFW--------------------------LPV-SPKRVFDFLR
XP_021292765.1/1-226 --P----PGI-VLSAATSFW--------------------------LPV-SPKRVFDFLR
XP_010657309.1/1-226 --P----PGI-VLSAATSFW--------------------------LPV-PPKRVFDFLR
XP_010657313.1/1-226 --P----PGI-VLSAATSFW--------------------------LPV-PPKRVFDFLR
XP_010657310.1/1-226 --P----PGI-VLSAATSFW--------------------------LPV-PPKRVFDFLR
XP_010657311.1/1-226 --P----PGI-VLSAATSFW--------------------------LPV-PPKRVFDFLR
XP_012086347.1/1-228 --P----PGI-VLSAATSFW--------------------------LPV-PPKRVFDFLR
XP_012086344.1/1-228 --P----PGI-VLSAATSFW--------------------------LPV-PPKRVFDFLR
XP_024029967.1/1-226 --P----PGI-VLSAATSFW--------------------------LPV-PPKRVFDFLR
XP_010109517.1/1-226 --P----PGI-VLSAATSFW--------------------------LPV-PPKRVFDFLR
XP_006469697.1/1-226 --P----PGI-VLSAATSFW--------------------------LPV-PPKRVFDFLR
XP_024046029.1/1-226 --P----PGI-VLSAATSFW--------------------------LPV-PPKRVFDFLR
XP_020234199.1/1-226 --P----PGI-VLSAATSFW--------------------------LPV-PPKRVFDFLR
XP_020234201.1/1-226 --P----PGI-VLSAATSFW--------------------------LPV-PPKRVFDFLR
XP_021630856.1/1-226 --P----PGI-VLSAATSFW--------------------------LPV-PPKRVFDFLR
XP_021630853.1/1-226 --P----PGI-VLSAATSFW--------------------------LPV-PPKRVFDFLR
XP_021907367.1/1-221 --P----PGI-VLSAATSFW--------------------------LPV-HPKVVFDFLR
XP_010275996.1/1-221 --P----PGI-VLSAATSFW--------------------------LPV-PPKKVFDFLR
XP_020870784.1/1-223 --P----PGI-VLSAATSFW--------------------------IPV-PPKRVFDFLR
NP_172015.1/1-223    --P----PGI-VLSAATSFW--------------------------IPV-PPKRVFDFLR
XP_006418050.1/1-223 --P----PGI-VLSAATSFW--------------------------IPV-PPKRVFDFLR
XP_018447753.1/1-223 --P----PGI-VLSAATSFW--------------------------IPV-PPKRVFDFLR
XP_010457605.1/1-218 --P----PGI-VLSAATSFW--------------------------IPV-PPKRVFDFLR
XP_023632765.1/1-218 --P----PGI-VLSAATSFW--------------------------IPV-PPKRVFDFLR
XP_010484829.1/1-218 --P----PGI-VLSAATSFW--------------------------IPV-PPKRVFDFLR
XP_010475208.1/1-218 --P----PGI-VLSAATSFW--------------------------IPV-PPKRVFDFLR
XP_018487097.1/1-220 --P----PGI-VLSAATSFG--------------------------IPV-PPKRVFDFLR
XP_013664795.1/1-220 --P----PGI-VLSAATSFG--------------------------IPV-PPKRVFDFLR
XP_013716253.1/1-220 --P----PGI-VLSAATSFG--------------------------IPV-PPKRVFDFLR
XP_009118607.1/1-220 --P----PGI-VLSAATSFG--------------------------IPV-PPKRVFDFLR
XP_013605632.1/1-220 --P----PGI-VLSAATSFG--------------------------IPV-PPKRVFDFLR
XP_010475204.1/1-220 --P----PGI-VLSAATSFW--------------------------IPV-PPKRVFDFLR
XP_010457604.1/1-220 --P----PGI-VLSAATSFW--------------------------IPV-PPKRVFDFLR
XP_023632762.1/1-220 --P----PGI-VLSAATSFW--------------------------IPV-PPKRVFDFLR
XP_010484823.1/1-220 --P----PGI-VLSAATSFW--------------------------IPV-PPKRVFDFLR
XP_022558401.1/1-220 --P----PGI-VLSAATSFW--------------------------IPV-PPKRVFDFLR
XP_013586641.1/1-220 --P----PGI-VLSAATSFW--------------------------IPV-PPKRVFDFLR
XP_022558399.1/1-220 --P----PGI-VLSAATSFW--------------------------IPV-PPKRVFDFLR
XP_022558400.1/1-220 --P----PGI-VLSAATSFW--------------------------IPV-PPKRVFDFLR
XP_013586642.1/1-220 --P----PGI-VLSAATSFW--------------------------IPV-PPKRVFDFLR
XP_013696832.1/1-220 --P----PGI-VLSAATSFW--------------------------IPV-PPKRVFDFLR
XP_022548878.1/1-220 --P----PGI-VLSAATSFW--------------------------IPV-PPKRVFDFLR
XP_013666552.1/1-220 --P----PGI-VLSAATSFW--------------------------IPV-PPKRVFDFLR
XP_009119177.1/1-220 --P----PGI-VLSAATSFW--------------------------IPV-PPKRVFDFLR
XP_020964430.1/1-225 --P----PGI-VLSAATSFW--------------------------LPV-PPKRVFEFLR
XP_015936069.1/1-225 --P----PGI-VLSAATSFW--------------------------LPV-PPKRVFEFLR
XP_020964429.1/1-225 --P----PGI-VLSAATSFW--------------------------LPV-PPKRVFEFLR
XP_015936068.1/1-225 --P----PGI-VLSAATSFW--------------------------LPV-PPKRVFEFLR
XP_015577880.1/1-228 --P----PGI-VLSAATSFW--------------------------LPV-PPKRVFDFLR
XP_002524172.2/1-228 --P----PGI-VLSAATSFW--------------------------LPV-PPKRVFDFLR
XP_007132557.1/1-221 --P----PGI-VLSAATSFW--------------------------LPV-PPKRVFDFLR
XP_017433694.1/1-221 --P----PGI-VLSAATSFW--------------------------LPV-PPKRVFDFLR
XP_014494419.1/1-221 --P----PGI-VLSAATSFW--------------------------LPV-PPKRVFDFLR
XP_022634310.1/1-221 --P----PGI-VLSAATSFW--------------------------LPV-PPKRVFDFLR
XP_010267735.1/1-225 --P----PGI-VLSAATSFW--------------------------LPV-PPKRVFDFLR
XP_010267734.1/1-225 --P----PGI-VLSAATSFW--------------------------LPV-PPKRVFDFLR
XP_024185199.1/1-224 --P----PGI-VLSAATSFW--------------------------LPV-PPKRVFEFLR
XP_024185197.1/1-224 --P----PGI-VLSAATSFW--------------------------LPV-PPKRVFEFLR
XP_009342246.1/1-222 --P----PGI-VLSAATSFW--------------------------LPV-PPKRVFEFLR
XP_009342244.1/1-222 --P----PGI-VLSAATSFW--------------------------LPV-PPKRVFEFLR
XP_009342240.1/1-222 --P----PGI-VLSAATSFW--------------------------LPV-PPKRVFEFLR
XP_020416503.1/1-221 --P----PGI-VLSAATSFW--------------------------LPV-PPKRVFEFLR
XP_021822854.1/1-221 --P----PGI-VLSAATSFW--------------------------LPV-PPKRVFEFLR
XP_007217584.1/1-221 --P----PGI-VLSAATSFW--------------------------LPV-PPKRVFEFLR
XP_021822852.1/1-221 --P----PGI-VLSAATSFW--------------------------LPV-PPKRVFEFLR
XP_018730806.1/1-228 --P----PGI-VLSAATSFW--------------------------LPV-QPNRVFDFLR
XP_010051064.1/1-228 --P----PGI-VLSAATSFW--------------------------LPV-QPNRVFDFLR
XP_015881130.2/1-229 --P----PGI-VLSAATSFW--------------------------LPV-PPKRVFDFLR
XP_023534533.1/1-219 --P----PGI-VLSAATSFW--------------------------LPV-PPKRIFDFLR
XP_022947095.1/1-219 --P----PGI-VLSAATSFW--------------------------LPV-PPKRIFDFLR
XP_022947096.1/1-219 --P----PGI-VLSAATSFW--------------------------LPV-PPKRIFDFLR
XP_022970950.1/1-219 --P----PGI-VLSAATSFW--------------------------LPI-PPKRIFDFLR
XP_022970951.1/1-219 --P----PGI-VLSAATSFW--------------------------LPI-PPKRIFDFLR
XP_022140692.1/1-217 --P----PGI-VLSAATSFW--------------------------LPV-PPKRIFDFLR
XP_022140691.1/1-217 --P----PGI-VLSAATSFW--------------------------LPV-PPKRIFDFLR
XP_008455853.1/1-220 --P----HGI-VLSAATSFW--------------------------LPV-PPKRIFDFLR
XP_011650012.1/1-220 --P----HGI-VLSAATSFW--------------------------LPV-PPKRIFDFLR
XP_008455850.1/1-220 --P----HGI-VLSAATSFW--------------------------LPV-PPKRIFDFLR
XP_011650011.1/1-220 --P----HGI-VLSAATSFW--------------------------LPV-PPKRIFDFLR
XP_023513403.1/1-220 --P----PGI-VLSAATSFW--------------------------LPV-PPKTIFDFLR
XP_022944761.1/1-220 --P----PGI-VLSAATSFW--------------------------LPV-PPKTIFDFLR
XP_023513401.1/1-220 --P----PGI-VLSAATSFW--------------------------LPV-PPKTIFDFLR
XP_022944759.1/1-220 --P----PGI-VLSAATSFW--------------------------LPV-PPKTIFDFLR
XP_022986270.1/1-220 --P----PGI-VLSAATSFW--------------------------LPV-PPKTIFDFLR
XP_022986268.1/1-220 --P----PGI-VLSAATSFW--------------------------LPV-PPKTIFDFLR
XP_022136006.1/1-224 --P----PGI-VLSAATSFW--------------------------LPI-PPNRVFHFLR
XP_008455423.1/1-229 --P----SGI-VLSAATSFW--------------------------LPL-PPNRVFHFLR
XP_011658704.1/1-229 --P----SGI-VLSAATSFW--------------------------LPL-PPNRVFHFLR
XP_004144488.1/1-229 --P----SGI-VLSAATSFW--------------------------LPL-PPNRVFHFLR
XP_022968990.1/1-229 --P----PGI-VLSAATSFW--------------------------LPV-PPKRVFDFLR
XP_023554403.1/1-229 --P----PGI-VLSAATSFW--------------------------LPV-PPKRVFDFLR
XP_022952948.1/1-229 --P----PGI-VLSAATSFW--------------------------LPV-PPKRVFDFLR
XP_019447751.1/1-228 --P----PGI-VLSAATSFW--------------------------LPV-SPKSVFEFLR
XP_019447749.1/1-228 --P----PGI-VLSAATSFW--------------------------LPV-SPKSVFEFLR
XP_004515074.1/1-228 --P----PGI-VLSAATSFW--------------------------LPV-PPKRVFDFLR
XP_012463792.1/1-235 --P----PGI-VLSAATSFW--------------------------LPV-SPKRVFDFLR
XP_012463790.1/1-235 --P----PGI-VLSAATSFW--------------------------LPV-SPKRVFDFLR
XP_016678783.1/1-235 --P----PGI-VLSAATSFW--------------------------LPV-SPKRVFDFLR
XP_016678782.1/1-235 --P----PGI-VLSAATSFW--------------------------LPV-SPKRVFDFLR
XP_017631648.1/1-235 --P----PGI-VLSAATSFW--------------------------LPV-SPKRVFDFLR
XP_016732503.1/1-235 --P----PGI-VLSAATSFW--------------------------LPV-SPKRVFDFLR
XP_017631647.1/1-235 --P----PGI-VLSAATSFW--------------------------LPV-SPKRVFDFLR
XP_012438430.1/1-229 --P----PGI-VLSAATSFW--------------------------LPV-SPKRVFDFLR
XP_012438427.1/1-229 --P----PGI-VLSAATSFW--------------------------LPV-SPKRVFDFLR
XP_012438429.1/1-229 --P----PGI-VLSAATSFW--------------------------LPV-SPKRVFDFLR
XP_017616162.1/1-229 --P----PGI-VLSAATSFW--------------------------LPV-SPKRVFDFLR
XP_017616160.1/1-229 --P----PGI-VLSAATSFW--------------------------LPV-SPKRVFDFLR
XP_017616161.1/1-229 --P----PGI-VLSAATSFW--------------------------LPV-SPKRVFDFLR
XP_016726347.1/1-229 --P----PGL-VLSAATSFW--------------------------LPV-SPKRVFDFLR
XP_016726344.1/1-229 --P----PGL-VLSAATSFW--------------------------LPV-SPKRVFDFLR
XP_016726345.1/1-229 --P----PGL-VLSAATSFW--------------------------LPV-SPKRVFDFLR
XP_019448933.1/1-227 --P----PGI-VLSAATSFW--------------------------LPV-PMKRVFDFLR
XP_004487400.1/1-228 --P----AGI-VLSAATSFW--------------------------LPV-PPKRVFEFLR
XP_003596999.2/1-227 --P----AGI-VLSAATSFW--------------------------LPV-PPKRVFEFLR
XP_024631890.1/1-227 --P----AGI-VLSAATSFW--------------------------LPV-PPKRVFEFLR
XP_024631891.1/1-227 --P----AGI-VLSAATSFW--------------------------LPV-PPKRVFEFLR
XP_004287935.1/1-224 --P----PGI-VLSAATSFW--------------------------LPV-PPKKVFEFLR
XP_018808969.1/1-228 --P----PGI-VLSAATSFW--------------------------LPV-PPKRVFDFLR
XP_018808967.1/1-228 --P----PGI-VLSAATSFW--------------------------LPV-PPKRVFDFLR
XP_018817598.1/1-227 --P----PGI-VLSAATSFW--------------------------LPV-SPKRVFDFLR
XP_018817597.1/1-227 --P----PGI-VLSAATSFW--------------------------LPV-SPKRVFDFLR
XP_018817594.1/1-227 --P----PGI-VLSAATSFW--------------------------LPV-SPKRVFDFLR
XP_018817599.1/1-227 --P----PGI-VLSAATSFW--------------------------LPV-SPKRVFDFLR
XP_023915056.1/1-227 --P----PGI-VLSAATSFW--------------------------LPV-PPKRVFDFLR
XP_023915054.1/1-227 --P----PGI-VLSAATSFW--------------------------LPV-PPKRVFDFLR
XP_023915053.1/1-227 --P----PGI-VLSAATSFW--------------------------LPV-PPKRVFDFLR
XP_021685023.1/1-228 --P----PGI-VLSAATSFW--------------------------LPV-PPKRVFDFLR
XP_021684657.1/1-228 --P----PGI-VLSAATSFW--------------------------LPV-PPKRVFDFLR
XP_021685089.1/1-228 --P----PGI-VLSAATSFW--------------------------LPV-PPKRVFDFLR
XP_015383009.1/1-229 --P----PGI-VLSAATSFW--------------------------LPV-PPKRVFDFLR
XP_015383007.1/1-229 --P----PGI-VLSAATSFW--------------------------LPV-PPKRVFDFLR
XP_020539441.1/1-235 --P----PGI-VLSAATSFW--------------------------LPV-PPKRVFDFLR
XP_020539439.1/1-235 --P----PGI-VLSAATSFW--------------------------LPV-PPKRVFDFLR
XP_015577881.1/1-233 --P----PGI-VLSAATSFW--------------------------LPV-PPKRVFDFLR
XP_015577879.1/1-233 --P----PGI-VLSAATSFW--------------------------LPV-PPKRVFDFLR
XP_015577877.1/1-233 --P----PGI-VLSAATSFW--------------------------LPV-PPKRVFDFLR
XP_021630854.1/1-231 --P----PGI-VLSAATSFW--------------------------LPV-PPKRVFDFLR
XP_021630849.1/1-231 --P----PGI-VLSAATSFW--------------------------LPV-PPKRVFDFLR
XP_021652390.1/1-229 --P----PGI-VLSAATSFW--------------------------LPV-PPKRVFDFLR
XP_021652388.1/1-229 --P----PGI-VLSAATSFW--------------------------LPV-PPKRVFDFLR
XP_021652389.1/1-234 --P----PGI-VLSAATSFW--------------------------LPV-PPKRVFDFLR
XP_021652386.1/1-234 --P----PGI-VLSAATSFW--------------------------LPV-PPKRVFDFLR
XP_006592380.1/1-228 --P----PGI-VLSAATSFW--------------------------LPV-PPKRVFDFLR
XP_003539872.2/1-228 --P----PGI-VLSAATSFW--------------------------LPV-PPKRVFDFLR
XP_025984806.1/1-230 --P----PGI-VLSAATSFW--------------------------LPV-PPKRVFDFLR
XP_014632311.1/1-230 --P----PGI-VLSAATSFW--------------------------LPV-PPKRVFDFLR
XP_024450362.1/1-227 --P----PGI-VLSAATSFW--------------------------LPV-PPKRVFDFLR
XP_002302880.2/1-227 --P----PGI-VLSAATSFW--------------------------LPV-PPKRVFDFLR
XP_024450361.1/1-227 --P----PGI-VLSAATSFW--------------------------LPV-PPKRVFDFLR
XP_024450360.1/1-227 --P----PGI-VLSAATSFW--------------------------LPV-PPKRVFDFLR
XP_011011493.1/1-227 --P----PGI-VLSAATSFW--------------------------LPV-PPKRVFDFLR
XP_011011491.1/1-227 --P----PGI-VLSAATSFW--------------------------LPV-PPKRVFDFLR
XP_011011492.1/1-227 --P----PGI-VLSAATSFW--------------------------LPV-PPKRVFDFLR
XP_011011489.1/1-227 --P----PGI-VLSAATSFW--------------------------LPV-PPKRVFDFLR
XP_011011488.1/1-227 --P----PGI-VLSAATSFW--------------------------LPV-PPKRVFDFLR
XP_011005116.1/1-225 --P----PGI-VLSAATSFW--------------------------LPV-PPKRVFDFLR
XP_011005113.1/1-225 --P----PGI-VLSAATSFW--------------------------LPV-PPKRVFDFLR
XP_011005112.1/1-225 --P----PGI-VLSAATSFW--------------------------LPV-PPKRVFDFLR
XP_024440391.1/1-225 --P----PGI-VLSAATSFW--------------------------LPV-PPKRVFDFLR
XP_002320458.2/1-225 --P----PGI-VLSAATSFW--------------------------LPV-PPKRVFDFLR
XP_024440389.1/1-225 --P----PGI-VLSAATSFW--------------------------LPV-PPKRVFDFLR
XP_019453767.1/1-227 --P----PGI-VLSAATSFW--------------------------LPV-PPRRVFDFLR
XP_012478857.1/1-227 --P----PGI-VLSAATSFW--------------------------LPV-SPKRVFDFLR
XP_012478855.1/1-227 --P----PGI-VLSAATSFW--------------------------LPV-SPKRVFDFLR
XP_012478856.1/1-227 --P----PGI-VLSAATSFW--------------------------LPV-SPKRVFDFLR
XP_012478851.1/1-227 --P----PGI-VLSAATSFW--------------------------LPV-SPKRVFDFLR
XP_016724999.1/1-227 --P----PGI-VLSAATSFW--------------------------LPV-SPKRVFDFLR
XP_017638204.1/1-227 --P----PGI-VLSAATSFW--------------------------LPV-SPKRVFDFLR
XP_017638203.1/1-227 --P----PGI-VLSAATSFW--------------------------LPV-SPKRVFDFLR
XP_016667113.1/1-227 --P----PGI-VLSAATSFW--------------------------LPV-SPKRVFDFLR
XP_016667112.1/1-227 --P----PGI-VLSAATSFW--------------------------LPV-SPKRVFDFLR
XP_015881131.2/1-227 --P----PGI-VLSAATSFW--------------------------LPV-PPKRVFDFLR
XP_008348131.1/1-222 --P----PGI-VLSAATSFW--------------------------LPV-PPKRVFEFLR
XP_008348133.1/1-220 --P----PGI-VLSAATSFW--------------------------LPV-PPKRVFEFLR
XP_018502305.1/1-220 --P----PGI-VLSAATSFW--------------------------LPV-PPKRVFEFLR
XP_009342243.1/1-220 --P----PGI-VLSAATSFW--------------------------LPV-PPKRVFEFLR
XP_017179321.1/1-222 --P----PGI-VLSAATSFW--------------------------LPV-PPKRVFEFLR
XP_017179320.1/1-222 --P----PGI-VLSAATSFW--------------------------LPV-PPKRVFEFLR
XP_008341986.1/1-222 --P----PGI-VLSAATSFW--------------------------LPV-PPKRVFEFLR
XP_017179319.1/1-220 --P----PGI-VLSAATSFW--------------------------LPV-PPKRVFEFLR
XP_024185198.1/1-222 --P----PGI-VLSAATSFW--------------------------LPV-PPKRVFEFLR
XP_011463209.1/1-222 --P----PGI-VLSAATSFW--------------------------LPV-PPKKVFEFLR
XP_020416501.1/1-219 --P----PGI-VLSAATSFW--------------------------LPV-PPKRVFEFLR
XP_021822853.1/1-219 --P----PGI-VLSAATSFW--------------------------LPV-PPKRVFEFLR
XP_008230079.1/1-219 --X----PGI-VLSAATSFW--------------------------LPV-PPKRVFEFLR
XP_020870786.1/1-221 --P----PGI-VLSAATSFW--------------------------IPV-PPKRVFDFLR
NP_001117231.1/1-221 --P----PGI-VLSAATSFW--------------------------IPV-PPKRVFDFLR
XP_018817596.1/1-225 --P----PGI-VLSAATSFW--------------------------LPV-SPKRVFDFLR
XP_019079350.1/1-224 --P----PGI-VLSAATSFW--------------------------LPV-PPKRVFDFLR
XP_022733639.1/1-224 --P----PGI-VLSAATSFW--------------------------LPV-SPKRVFDFLR
XP_021292768.1/1-224 --P----PGI-VLSAATSFW--------------------------LPV-SPKRVFDFLR
XP_021292766.1/1-224 --P----PGI-VLSAATSFW--------------------------LPV-SPKRVFDFLR
XP_017971480.1/1-224 --P----PGI-VLSAATSFW--------------------------LPV-SPKRVFDFLR
XP_018721484.1/1-222 --P----PGI-VLCAATSFW--------------------------LPV-SPKQVFDFLR
XP_018721483.1/1-222 --P----PGI-VLCAATSFW--------------------------LPV-SPKQVFDFLR
XP_018721482.1/1-222 --P----PGI-VLCAATSFW--------------------------LPV-SPKQVFDFLR
XP_025687365.1/1-237 --P----PGI-VLSAATSFW--------------------------LPV-PPKTAFDFLR
XP_006851770.2/1-243 --P----PGV-VLSAATSVW--------------------------LPI-SPLRLFNFLR
XP_003623826.1/1-230 --P----PGI-VLSAATSVW--------------------------LPA-SPQKVFDFLR
XP_004492678.1/1-230 --P----PGI-VLSAGTSVW--------------------------LPA-SPQRVFDFLR
XP_004492677.1/1-230 --P----PGI-VLSAGTSVW--------------------------LPA-SPQRVFDFLR
XP_012847897.1/1-236 --P----PGI-VLSAATSVW--------------------------LPV-TPQRVFDFLR
XP_012843673.1/1-229 --P----PGI-VLSAATSVW--------------------------LPV-TPQRVFDFLR
XP_021279608.1/1-239 --P----PGI-VLSAATSVW--------------------------LPV-SPQRLFDFLR
XP_025641241.1/1-241 --P----PGI-VLSAATSVW--------------------------LPV-SPQRLFDFLR
XP_025641246.1/1-241 --P----PGI-VLSAATSVW--------------------------LPV-SPQRLFDFLR
XP_020991942.1/1-241 --P----PGI-VLSAATSVW--------------------------LPV-SPQRLFDFLR
XP_025632115.1/1-241 --P----PGI-VLSAATSVW--------------------------LPV-SPQRLFDFLR
XP_025632116.1/1-241 --P----PGI-VLSAATSVW--------------------------LPV-SPQRLFDFLR
XP_017442222.1/1-246 --P----PGI-VLSAATSVW--------------------------LPV-SPQRLFDFLR
XP_017442225.1/1-246 --P----PGI-VLSAATSVW--------------------------LPV-SPQRLFDFLR
XP_006576359.1/1-244 --P----PGI-VLSAATSVW--------------------------LPV-SPHRLFDFLR
XP_003521284.1/1-244 --P----PGI-VLSAATSVW--------------------------LPV-SPHRLFDFLR
XP_024174849.1/1-237 --P----PGI-VLSAATSVW--------------------------LPV-SPQRLFNFLR
XP_024174854.1/1-237 --P----PGI-VLSAATSVW--------------------------LPV-SPQRLFNFLR
XP_004306832.1/1-241 --P----PGI-VLSAATSVW--------------------------LPV-SPQRLFNFLR
XP_011469062.1/1-241 --P----PGI-VLSAATSVW--------------------------LPV-SPQRLFNFLR
XP_009340632.2/1-243 --P----PGV-VLSAATSVW--------------------------LPV-SPQRLFDFLR
XP_008385703.1/1-243 --P----PGV-VLSAATSVW--------------------------LPV-SPQRLFDFLR
XP_008385704.1/1-243 --P----PGV-VLSAATSVW--------------------------LPV-SPQRLFDFLR
XP_008232741.1/1-243 --P----PGI-VLSAATSVW--------------------------LPV-SPQRLFDFLR
XP_008232742.1/1-243 --P----PGI-VLSAATSVW--------------------------LPV-SPQRLFDFLR
XP_007220256.1/1-243 --P----PGI-VLSAATSVW--------------------------LPV-SPQRLFDFLR
XP_020413144.1/1-243 --P----PGI-VLSAATSVW--------------------------LPV-SPQRLFDFLR
XP_021817103.1/1-243 --P----PGI-VLSAATSVW--------------------------LPV-SPQRLFDFLR
XP_021817105.1/1-243 --P----PGI-VLSAATSVW--------------------------LPV-SPQRLFDFLR
XP_022872559.1/1-230 --P----PGI-VLSAATSVW--------------------------LPV-SPQRLFDFLR
XP_016575742.1/1-238 --P----PGI-VLSAATSVW--------------------------LPV-SPQRLFDFLR
XP_017608938.1/1-232 --P----PGI-VLSAATSVW--------------------------LPV-SPQRLFDFLR
XP_017608947.1/1-232 --P----PGI-VLSAATSVW--------------------------LPV-SPQRLFDFLR
XP_010255501.1/1-245 --P----PGV-VLSAATSVW--------------------------LPV-SPQRLFDFLR
XP_011023407.1/1-238 --P----PGI-VLSAATSVW--------------------------LPV-SPQRLFDFLR
XP_011023404.1/1-238 --P----PGI-VLSAATSVW--------------------------LPV-SPQRLFDFLR
XP_011023405.1/1-238 --P----PGI-VLSAATSVW--------------------------LPV-SPQRLFDFLR
XP_019152252.1/1-243 --P----SGI-VLSAATSVW--------------------------LPV-SPQRLFDFLR
XP_022870029.1/1-235 --P----PGI-VLSASTSVW--------------------------LPV-SPHRLFDFLR
XP_017699285.1/1-225 --P----PGV-VLSAATSVW--------------------------LPV-SPLRLFDFLR
XP_008795402.1/1-225 --P----PGV-VLSAATSVW--------------------------LPV-SPLRLFDFLR
XP_010933869.1/1-224 --P----PGV-VLSAATSVW--------------------------LPV-SPMRLFDFLR
XP_008799528.1/1-222 --P----PGV-VLSAATSVW--------------------------LPV-SPQRLFDFLR
XP_010919252.1/1-223 --P----PGV-VLSAATSVW--------------------------LPV-SPQRLFDFLR
XP_010919250.1/1-223 --P----PGV-VLSAATSVW--------------------------LPV-SPQRLFDFLR
XP_007139955.1/1-229 --P----PGI-VLSAATSVW--------------------------LPV-SAQRLFDFLR
XP_017418814.1/1-230 --P----PGI-VLSAATSVW--------------------------LPV-SPQRLFDFLR
XP_014490274.1/1-230 --P----PGI-VLSAATSVW--------------------------LPV-SPQRLFDFLR
XP_003534596.1/1-229 --P----PGI-VLSAATSVW--------------------------LPV-SPQRLFDFLR
XP_006587871.1/1-229 --P----PGI-VLSAATSVW--------------------------LPV-SPQRLFDFLR
XP_003552359.1/1-228 --P----PGI-VLSAATSVW--------------------------LPV-SSQRLFDFLR
XP_015961616.1/1-228 --P----PGI-VLSAATSVW--------------------------LPV-SPQRLFDFLR
XP_015961617.1/1-228 --P----PGI-VLSAATSVW--------------------------LPV-SPQRLFDFLR
XP_025696060.1/1-228 --P----PGI-VLSAATSVW--------------------------LPV-SPQRLFDFLR
XP_025696061.1/1-228 --P----PGI-VLSAATSVW--------------------------LPV-SPQRLFDFLR
XP_016198931.1/1-228 --P----PGI-VLSAATSVW--------------------------LPV-SPQRLFDFLR
XP_016198932.1/1-228 --P----PGI-VLSAATSVW--------------------------LPV-SPQRLFDFLR
XP_025649261.1/1-228 --P----PGI-VLSAATSVW--------------------------LPV-SPQRLFDFLR
XP_025649262.1/1-228 --P----PGI-VLSAATSVW--------------------------LPV-SPQRLFDFLR
XP_021849370.1/1-228 --P----SGI-VLSAATSVW--------------------------LPV-SQQRLFDFLR
XP_021767325.1/1-228 --P----PGI-VLSAATSVW--------------------------LPV-SPQRLFDFLR
XP_021748881.1/1-228 --P----PGI-VLSAATSVW--------------------------LPV-SPQRLFDFLR
XP_019461451.1/1-231 --P----PGV-VLSAATSVW--------------------------LPV-SSQRLFDFLR
XP_012574211.1/1-233 --P----PGI-VLSAATSVW--------------------------LPV-SPQRLFDFLR
XP_004510857.1/1-233 --P----PGI-VLSAATSVW--------------------------LPV-SPQRLFDFLR
XP_020237185.1/1-234 --P----PGV-VLSAATSVW--------------------------LPV-SPHRLFHFLR
XP_020237261.1/1-234 --P----PGV-VLSAATSVW--------------------------LPV-SPHRLFHFLR
XP_019456357.1/1-238 --P----PGV-VLSAATSVW--------------------------LPV-SPKRVFDFLR
XP_013444922.1/1-235 --P----PGI-VLSAATSVW--------------------------LPV-SPQRLFDFLR
XP_003528880.1/1-238 --P----PGI-VLSAATSVW--------------------------LPV-SPHRLFDFLR
XP_006583340.1/1-238 --P----PGI-VLSAATSVW--------------------------LPV-SPHRLFDFLR
XP_014516480.1/1-238 --P----PGI-VLSAATSVW--------------------------LPV-SPQRLFDFLR
XP_014516483.1/1-238 --P----PGI-VLSAATSVW--------------------------LPV-SPQRLFDFLR
XP_007134961.1/1-238 --P----PGI-VLSAATSVW--------------------------LPV-SPQRLFDFLR
XP_010671720.1/1-229 --P----SGI-VLSAATAVW--------------------------LPV-SPQRLFDFLR
XP_012482732.1/1-232 --P----PGI-VLSAATSVW--------------------------LPV-SPQRLFDFLR
XP_012482742.1/1-232 --P----PGI-VLSAATSVW--------------------------LPV-SPQRLFDFLR
XP_016745638.1/1-232 --P----PGI-VLSAATSVW--------------------------LPV-SPQRLFDFLR
XP_016745639.1/1-232 --P----PGI-VLSAATSVW--------------------------LPV-SPQRLFDFLR
XP_010278578.1/1-242 --P----PGV-VLSAATSVW--------------------------LPV-SPQRLFDFLR
XP_010278577.1/1-242 --P----PGV-VLSAATSVW--------------------------LPV-SPQRLFDFLR
XP_006445143.1/1-238 --P----PGI-VLSAATSVW--------------------------LPV-SPQRLFNFLR
XP_006445141.1/1-238 --P----PGI-VLSAATSVW--------------------------LPV-SPQRLFNFLR
XP_022770361.1/1-238 --P----PGI-VLSAATSVW--------------------------LPV-SPQRLFDFLR
XP_022757219.1/1-237 --P----PGI-VLSAATSVW--------------------------LPV-SPQRLFDFLR
XP_022757220.1/1-237 --P----PGI-VLSAATSVW--------------------------LPV-SPQRLFDFLR
NP_001314420.1/1-237 --P----PGI-VLSAATSVW--------------------------LPV-SPQRLFDFLR
XP_016735217.1/1-237 --P----PGI-VLSAATSVW--------------------------LPV-SPQRLFDFLR
XP_012437660.1/1-237 --P----PGI-VLSAATSVW--------------------------LPV-SPQRLFDFLR
XP_012437661.1/1-237 --P----PGI-VLSAATSVW--------------------------LPV-SPQRLFDFLR
XP_017615505.1/1-237 --P----PGI-VLSAATSVW--------------------------LPV-SPQRLFDFLR
XP_017615506.1/1-237 --P----PGI-VLSAATSVW--------------------------LPV-SPQRLFDFLR
XP_016723220.1/1-237 --P----PGI-VLSAATSVW--------------------------LPV-SPQRLFDFLR
XP_016723229.1/1-237 --P----PGI-VLSAATSVW--------------------------LPV-SPQRLFDFLR
XP_021904309.1/1-244 --P----PGI-VLSAATSVW--------------------------LPV-SPQRLFDFLR
XP_021904311.1/1-244 --P----PGI-VLSAATSVW--------------------------LPV-SPQRLFDFLR
XP_007051912.2/1-241 --P----PGI-VLSAATSVW--------------------------LPV-SPQRLFDFLR
XP_007051913.2/1-241 --P----PGI-VLSAATSVW--------------------------LPV-SPQRLFDFLR
XP_012489878.1/1-239 --P----PGI-VLSAATSVW--------------------------LPV-SPQRLFDFLR
XP_016748231.1/1-239 --P----PGI-VLSAATSVW--------------------------LPV-SPQRLFDFLR
XP_017631289.1/1-239 --P----PGI-VLSAATSVW--------------------------LPV-SPQRLFDFLR
XP_023006686.1/1-236 --P----PGI-VLSAATSVW--------------------------LPV-SPQRLFDFLR
XP_022958954.1/1-236 --P----PGI-VLSAATSVW--------------------------LPV-SPQRLFDFLR
XP_023548045.1/1-236 --P----PGI-VLSAATSVW--------------------------LPV-SPQRLFDFLR
XP_008460172.1/1-241 --P----PGI-VLSAATSVW--------------------------LPV-SPQRLFDFLR
XP_004144982.1/1-241 --P----PGI-VLSAATSVW--------------------------LPV-SPQRLFDFLR
XP_023514722.1/1-242 --P----PGI-VLSAATSVW--------------------------LPL-SPQRLFDFLR
XP_022999814.1/1-242 --P----PGI-VLSAATSVW--------------------------LPL-SPQRLFDFLR
XP_022964504.1/1-242 --P----PGI-VLSAATSVW--------------------------LPL-SPQRLFDFLR
XP_010053664.1/1-245 --P----PGI-VLSAATSVW--------------------------LPV-SPQRLFDFLR
XP_019189515.1/1-235 --P----PGI-VLSAATSVW--------------------------LPI-SPGRLFDFLR
XP_023730775.1/1-234 --P----PGI-VLSAATSVW--------------------------LPV-SPQRLFDFLR
XP_023730774.1/1-234 --P----PGI-VLSAATSVW--------------------------LPV-SPQRLFDFLR
XP_022020543.1/1-230 --P----PGI-VLSAATSVW--------------------------LPV-SPQRLFDFLR
XP_024962572.1/1-236 --P----PGI-VLSAATSVW--------------------------LPV-SPQRLFDFLR
XP_022017696.1/1-236 --P----PGI-VLSAATSVW--------------------------LPV-SPQRLFDFLR
XP_024018220.1/1-245 --P----PGI-VLSAATSVW--------------------------LPV-SPNRLFDFLR
XP_011093166.1/1-234 --P----PGI-VLSAATSVW--------------------------LPV-TPQRLFDFLR
XP_011093167.1/1-234 --P----PGI-VLSAATSVW--------------------------LPV-TPQRLFDFLR
XP_006339457.1/1-238 --P----AGI-VLSAATSVW--------------------------LPV-SPQRLFDFLR
XP_015059298.1/1-238 --P----AGI-VLSAATSVW--------------------------LPV-SPQRLFDFLR
NP_001234657.2/1-238 --P----AGI-VLSAATSVW--------------------------LPV-SPQRLFDFLR
XP_009602857.1/1-241 --P----PGI-VLSAATSVW--------------------------LPI-SPQRLFDFLR
XP_009602856.1/1-241 --P----PGI-VLSAATSVW--------------------------LPI-SPQRLFDFLR
XP_009782444.1/1-245 --P----PGI-VLSAATSVW--------------------------LPI-SPQRLFDFLR
XP_009782443.1/1-245 --P----PGI-VLSAATSVW--------------------------LPI-SPQRLFDFLR
XP_019259406.1/1-245 --P----PGI-VLSAATSVW--------------------------LPV-SPQRLFDFLR
XP_015867375.1/1-242 --P----PGI-VLSAATSVW--------------------------LPV-SPQRLFDFLR
XP_015867374.1/1-242 --P----PGI-VLSAATSVW--------------------------LPV-SPQRLFDFLR
XP_015888672.1/1-242 --P----PGI-VLSAATSVW--------------------------LPV-SPQRLFDFLR
XP_018810322.1/1-233 --P----PGV-VLSAATSVW--------------------------LPV-SPQRLFDFLR
XP_018859074.1/1-233 --P----PGV-VLSAATSVW--------------------------LPV-SPQRLFDFLR
XP_017231243.1/1-235 --P----PGI-VLSAATSVW--------------------------LPV-SPQRLFNFLR
XP_017231244.1/1-235 --P----PGI-VLSAATSVW--------------------------LPV-SPQRLFNFLR
XP_017231245.1/1-235 --P----PGI-VLSAATSVW--------------------------LPV-SPQRLFNFLR
XP_017236322.1/1-235 --P----PGI-VLSAATSVW--------------------------LPI-SPQRLFDFLR
XP_002301331.2/1-238 --P----PGI-VLSAATSVW--------------------------LPV-SPQRLFDFLR
XP_024451706.1/1-238 --P----PGI-VLSAATSVW--------------------------LPV-SPQRLFDFLR
XP_002320755.1/1-241 --P----PGI-VLSAATSVW--------------------------LPV-SPQRLFDFLR
XP_011035097.1/1-241 --P----PGI-VLSAATSVW--------------------------LPV-SPQRLFDFLR
XP_018813891.1/1-238 --P----PGI-VLSAATSVW--------------------------LPV-SPQKLFDFLR
XP_012083470.1/1-239 --P----PGI-VLSAATSVW--------------------------LPV-SPQRLFDFLR
XP_021651338.1/1-240 --P----PGI-VLSAATSVW--------------------------LPV-SPQRLFDFLR
XP_021613538.1/1-241 --P----PGI-VLSAATSVW--------------------------LPV-SPQRLFDFLR
XP_021686862.1/1-241 --P----PGI-VLSAATSVW--------------------------LPV-SPHRLFDFLR
XP_021686863.1/1-241 --P----PGI-VLSAATSVW--------------------------LPV-SPHRLFDFLR
XP_021612685.1/1-241 --P----PGI-VLSAATSVW--------------------------LPV-SPQRLFDFLR
XP_023879166.1/1-242 --P----PGI-VLSAATSVW--------------------------LPV-SPQRLFDFLR
XP_002272264.2/1-234 --P----PGI-VLSAATSVW--------------------------LPV-SPQRLFDFLR
XP_010661562.1/1-234 --P----PGI-VLSAATSVW--------------------------LPV-SPQRLFDFLR
XP_002511801.1/1-237 --P----PGI-VLSAATSVW--------------------------LPV-SPQRLFDFLR
XP_015584500.1/1-237 --P----PGI-VLSAATSVW--------------------------LPV-SPQRLFDFLR
XP_022141257.1/1-238 --P----PGI-VLSASTSVW--------------------------MPV-AQQRLFAFLQ
XP_023885612.1/1-244 --P----HGI-VVSAATSVW--------------------------MPV-SQQGLFDFLR
XP_023885611.1/1-244 --P----HGI-VVSAATSVW--------------------------MPV-SQQGLFDFLR
XP_018830842.1/1-239 --P----PGI-VLSAATSVW--------------------------IPV-AQPRLFDFLR
XP_018830843.1/1-239 --P----PGI-VLSAATSVW--------------------------IPV-AQPRLFDFLR
XP_018830840.1/1-239 --P----PGI-VLSAATSVW--------------------------IPV-AQPRLFDFLR
XP_018830841.1/1-239 --P----PGI-VLSAATSVW--------------------------IPV-AQPRLFDFLR
XP_012090293.1/1-240 --P----PGV-VLSAATSVW--------------------------LPV-MRQRLFDFLR
XP_012090292.1/1-240 --P----PGV-VLSAATSVW--------------------------LPV-MRQRLFDFLR
XP_021614581.1/1-239 --P----PGI-VLSAATSVW--------------------------LPV-MRDRLFDFLR
XP_021614580.1/1-239 --P----PGI-VLSAATSVW--------------------------LPV-MRDRLFDFLR
XP_015581247.1/1-240 --P----PGI-VLSAATSVW--------------------------LPV-MRQRLFDFLR
XP_002510822.1/1-240 --P----PGI-VLSAATSVW--------------------------LPV-MRQRLFDFLR
XP_021648646.1/1-240 --P----PGI-VLSAATSVW--------------------------LPV-MRQRLFDFLR
XP_021634593.1/1-234 --P----PGV-VLSATTSVW--------------------------LPI-TRQRLFDFLR
XP_021634592.1/1-234 --P----PGV-VLSATTSVW--------------------------LPI-TRQRLFDFLR
XP_006490348.1/1-238 --P----PGV-VLCAATTVW--------------------------LPM-TRQRLFDFMR
XP_006490346.1/1-238 --P----PGV-VLCAATTVW--------------------------LPM-TRQRLFDFMR
XP_024958654.1/1-238 --P----PGV-VLCAATTVW--------------------------LPM-TRQRLFDFMR
XP_006490345.1/1-238 --P----PGV-VLCAATTVW--------------------------LPM-TRQRLFDFMR
XP_006421879.2/1-238 --P----PGV-VLCAATTVW--------------------------LPM-TRQRLFDFMR
XP_021892212.1/1-221 --P----PGV-VLSAATSVW--------------------------MPV-TQERLFNFLR
XP_021892211.1/1-221 --P----PGV-VLSAATSVW--------------------------MPV-TQERLFNFLR
XP_021892210.1/1-221 --P----PGV-VLSAATSVW--------------------------MPV-TQERLFNFLR
XP_022765148.1/1-244 --P----RGV-VLSASTSVW--------------------------MLI-NQKSLFDFLR
XP_022765158.1/1-244 --P----RGV-VLSASTSVW--------------------------MLI-NQKSLFDFLR
XP_022765120.1/1-244 --P----RGV-VLSASTSVW--------------------------MLI-NQKSLFDFLR
XP_022765114.1/1-244 --P----RGV-VLSASTSVW--------------------------MLI-NQKSLFDFLR
XP_022736693.1/1-237 --P----HGV-VLSASTSVW--------------------------MPI-TQQRLFDFLR
XP_022736692.1/1-237 --P----HGV-VLSASTSVW--------------------------MPI-TQQRLFDFLR
XP_022736689.1/1-237 --P----HGV-VLSASTSVW--------------------------MPI-TQQRLFDFLR
XP_022734775.1/1-244 --P----HGV-VLSAATSVW--------------------------MSI-TQQRLFDFLR
XP_016743353.1/1-244 --P----HGV-VLSAATSVW--------------------------MPV-TQERLFDFLR
XP_012474605.1/1-244 --P----HGV-VLSAATSVW--------------------------MPV-TQERLFDFLR
XP_012474606.1/1-244 --P----HGV-VLSAATSVW--------------------------MPV-TQERLFDFLR
XP_016743352.1/1-244 --P----HGV-VLSAATSVW--------------------------MPV-TQERLFDFLR
XP_016743351.1/1-244 --P----HGV-VLSAATSVW--------------------------MPV-TQERLFDFLR
XP_012474604.1/1-244 --P----HGV-VLSAATSVW--------------------------MPV-TQERLFDFLR
XP_012474603.1/1-244 --P----HGV-VLSAATSVW--------------------------MPV-TQERLFDFLR
XP_017646297.1/1-244 --P----HGV-VLSAATSVW--------------------------MPV-TQERLFDFLR
XP_016736315.1/1-244 --P----HGV-VLSAATSVW--------------------------MPV-TQERLFDFLR
XP_017646296.1/1-244 --P----HGV-VLSAATSVW--------------------------MPV-TQERLFDFLR
XP_017646298.1/1-244 --P----HGV-VLSAATSVW--------------------------MPV-TQERLFDFLR
XP_016736306.1/1-244 --P----HGV-VLSAATSVW--------------------------MPV-TQERLFDFLR
XP_017646295.1/1-244 --P----HGV-VLSAATSVW--------------------------MPV-TQERLFDFLR
XP_017972896.1/1-244 --P----AGV-VLSAATSVW--------------------------MPI-TQQRLFDFLR
XP_017972895.1/1-244 --P----AGV-VLSAATSVW--------------------------MPI-TQQRLFDFLR
XP_017972893.1/1-244 --P----AGV-VLSAATSVW--------------------------MPI-TQQRLFDFLR
XP_021274823.1/1-244 --P----AGV-VLSAATSVW--------------------------MPI-TQQRLFDFLR
XP_024536343.1/1-231 --P----PGI-VLSAATSLW--------------------------MPV-SPQRVFEFLR
XP_006366174.1/1-244 --P----PGI-VVNAATSVW--------------------------LPV-SPQRLFEFLR
XP_020219711.1/1-212 ------------------IW--------------------------LPV-SPQRLFDFLR
XP_022897056.1/1-222 --P----PGI-VLSAATSVW--------------------------LPV-SPQRLFDFLH
NP_001281015.1/1-243 --P----PGV-VLSAATSVW--------------------------LPV-SPQRLFDFLR
XP_008383260.1/1-243 --P----PGV-VLSAATSVW--------------------------LPV-SPQRLFDFLR
XP_022896229.1/1-235 --P----PGI-VLSASTSVW--------------------------LPV-SPQRLFDFLR
XP_022896230.1/1-235 --P----PGI-VLSASTSVW--------------------------LPV-SPQRLFDFLR
XP_025816451.1/1-225 --P----PGV-VLSAATSVW--------------------------VPV-APGKLFNFLR
XP_004968001.1/1-225 --P----PGV-VLSAATSVW--------------------------VPV-APEKLFNFLR
XP_004968002.1/1-225 --P----PGV-VLSAATSVW--------------------------VPV-APEKLFNFLR
XP_002457484.1/1-226 --P----PGV-VLSAATSVW--------------------------VPV-APEKLFNFLR
XP_009400446.1/1-224 --P----PGV-VLSAATSVW--------------------------LPV-APQRLFDFLR
XP_018676535.1/1-225 --P----PGV-VLSAATSVW--------------------------LPA-SPQRLFDFLR
XP_009401771.1/1-232 --P----PGV-VLSAATSVW--------------------------LPV-SPQRLFDFLR
XP_009384534.1/1-228 --P----PGV-VLSAATSVW--------------------------LPL-SPQRLFDFLR
XP_020672447.1/1-217 --P----PGV-VLSAATSVW--------------------------LPI-SPQRLFDFLR
XP_020582116.1/1-218 --P----PGV-VLSAATSVW--------------------------LPI-SPQRLFDFLR
XP_020675647.1/1-231 --P----PGV-VLSAATSVW--------------------------LPV-SPHRLFDFLR
XP_020675648.1/1-231 --P----PGV-VLSAATSVW--------------------------LPV-SPHRLFDFLR
XP_020585632.1/1-228 --P----PGV-VLSAATSVW--------------------------LPV-SPNRLFDFLR
XP_016176402.1/1-228 --P----PGI-VLSAATSVW--------------------------VPV-SRQRLFEFLR
XP_016176403.1/1-228 --P----PGI-VLSAATSVW--------------------------VPV-SRQRLFEFLR
XP_016176400.1/1-228 --P----PGI-VLSAATSVW--------------------------VPV-SRQRLFEFLR
XP_016176401.1/1-228 --P----PGI-VLSAATSVW--------------------------VPV-SRQRLFEFLR
XP_010523042.1/1-245 --P----PGI-VLSAATSVW--------------------------VPG-SPQRLFDFLR
XP_010523041.1/1-240 --P----PGI-VLSAATSVW--------------------------VPG-SPQRLFDFLR
XP_020878511.1/1-224 --P----PGI-VLSAATSVW--------------------------LPA-SPQRLYDFLR
NP_567183.2/1-228    --P----PGI-VLSAATSVW--------------------------LPA-APQRLYDFLR
XP_006287095.1/1-228 --P----PGI-VLSAATSVW--------------------------LPA-SPQRLFDFLR
XP_010456250.1/1-228 --P----PGI-VLSAATSVW--------------------------LPA-SPQRLFDFVR
XP_010419627.1/1-228 --P----PGI-VLSAATSVW--------------------------LPA-SPQRLFDFLR
XP_010427345.1/1-228 --P----PGI-VLSAATSVW--------------------------LPA-SPQRLFDFLR
XP_009111469.1/1-229 --A----PGI-VLSAATSVW--------------------------LPA-SPQRLFYFLR
XP_018487039.1/1-229 --A----PGI-VLSAATSVW--------------------------LPA-SPQRLFDFLR
XP_018453129.1/1-229 --A----PGI-VLSAATSVW--------------------------LPA-SPQRLFDFLR
XP_013612299.1/1-229 --A----PGI-VLSAATSVW--------------------------LPA-SPQRLFDFLR
XP_006396268.1/1-225 --D----SGI-ILSAATSVW--------------------------LPA-SPQRLFDFLR
XP_018475317.1/1-221 --D----SGI-VLSAATSVW--------------------------LPA-SPQRLFDFLR
XP_013629750.1/1-221 --D----SGI-VLSAATSVW--------------------------LPA-SPQRLFDFLR
XP_009134533.1/1-221 --D----SGI-VLSAATSVW--------------------------LPA-SPQRLFDFLR
XP_020531377.1/1-244 --P----PGV-VLSAATSVW--------------------------LPV-APQRLFEFLR
XP_006857866.1/1-244 --P----PGV-VLSAATSVW--------------------------LPV-APQRLFEFLR
XP_018434263.1/1-219 --P----PGI-LLSASTSVW--------------------------VPV-SPRRLFDFLR
XP_018434260.1/1-219 --P----PGI-LLSASTSVW--------------------------VPV-SPRRLFDFLR
XP_018434262.1/1-219 --P----PGI-LLSASTSVW--------------------------VPV-SPRRLFDFLR
XP_013688926.1/1-219 --P----PGI-LLSAATSVW--------------------------VPV-TPRRLFDFLR
XP_013637558.1/1-219 --P----PGI-LLSAATSVW--------------------------VPV-TPRRLFDFLR
XP_013637556.1/1-219 --P----PGI-LLSAATSVW--------------------------VPV-TPRRLFDFLR
XP_013637557.1/1-219 --P----PGI-LLSAATSVW--------------------------VPV-TPRRLFDFLR
XP_009143168.1/1-219 --P----PGI-LLSAATSVW--------------------------VPV-TPRRLFDFLR
XP_009143167.1/1-219 --P----PGI-LLSAATSVW--------------------------VPV-TPRRLFDFLR
XP_009143166.1/1-219 --P----PGI-LLSAATSVW--------------------------VPV-TPRRLFDFLR
XP_013692686.1/1-219 --P----PGI-LLSAATSVW--------------------------VPV-TPRRLFDFLR
XP_022574820.1/1-219 --P----PGI-LLSAATSVW--------------------------VPV-TPRRLFDFLR
XP_013692684.1/1-219 --P----PGI-LLSAATSVW--------------------------VPV-TPRRLFDFLR
XP_013748127.1/1-219 --P----PGI-LLSAATSVW--------------------------VPV-TPRRLFDFLR
XP_013692685.1/1-219 --P----PGI-LLSAATSVW--------------------------VPV-TPRRLFDFLR
XP_013748124.1/1-219 --P----PGI-LLSAATSVW--------------------------VPV-TPRRLFDFLR
XP_013748126.1/1-219 --P----PGI-LLSAATSVW--------------------------VPV-TPRRLFDFLR
XP_022574821.1/1-219 --P----PGI-LLSAATSVW--------------------------VPV-TPRRLFDFLR
XP_006290613.1/1-229 --P----PGI-VLNAATSVW--------------------------MPV-SPRRLFDFLG
XP_013592301.1/1-230 --P----PGI-VLNAATSVW--------------------------MPV-SPKRLFDFLG
XP_018442581.1/1-230 --P----PGI-VLNAATSVW--------------------------MPV-SPKRLFDFLG
XP_013750213.1/1-232 --P----PGI-VLNAATSVW--------------------------MPV-SPKRLFDFLG
XP_013648430.1/1-232 --P----PGI-VLNAATSVW--------------------------MPV-SPKRLFDFLG
XP_009104292.1/1-232 --P----PGI-VLNAATSVW--------------------------MPV-SPKRLFDFLG
XP_019058514.1/1-223 --P----PGI-VLSAATSVW--------------------------LPETAPRRLFDFLR
XP_010523728.1/1-220 --P----PGI-VLSAATSVW--------------------------LRGEGARRLFDFLR
XP_024514927.1/1-242 --P----PGI-VLSAATSLW--------------------------VPV-NSQRLFEFLR
XP_002985242.1/1-242 --P----PGI-VLSAATSLW--------------------------VPV-NSQRLFEFLR
XP_024530500.1/1-242 --P----PGI-VLSAATSLW--------------------------VPV-NSQRLFEFLR
XP_024530499.1/1-242 --P----PGI-VLSAATSLW--------------------------VPV-NSQRLFEFLR
XP_024530501.1/1-242 --P----PGI-VLSAATSLW--------------------------VPV-NSQRLFEFLR
XP_010107411.1/1-242 --P----PGI-ILSAATSVW--------------------------IPV-SQKRLFEFLR
XP_024028635.1/1-242 --P----PGI-ILSAATSVW--------------------------IPV-SQKRLFEFLR
XP_015877999.1/1-244 --P----PGI-VLSAATSVW--------------------------IPV-PQQRLFDFLR
XP_019427294.1/1-247 --P----PGI-VLSAATSVW--------------------------MPV-SRQRLFDFLR
XP_019427292.1/1-247 --P----PGI-VLSAATSVW--------------------------MPV-SRQRLFDFLR
XP_019452814.1/1-239 --P----PGI-VLSAATSVW--------------------------MPV-SQQRLFNFLR
XP_019452815.1/1-239 --P----PGI-VLSAATSVW--------------------------MPV-SQQRLFNFLR
XP_003592295.2/1-235 --P----PGI-VLSAATSVW--------------------------MPV-SRQRLFDFLR
XP_024625328.1/1-235 --P----PGI-VLSAATSVW--------------------------MPV-SRQRLFDFLR
XP_012570175.1/1-236 --P----PGI-VLSAATSVW--------------------------MPV-SRQRLFDFLR
XP_012570176.1/1-236 --P----PGI-VLSAATSVW--------------------------MPV-SRQRLFDFLR
XP_004496593.1/1-236 --P----PGI-VLSAATSVW--------------------------MPV-SRQRLFDFLR
XP_025620154.1/1-234 --P----PGI-VLSAATSVW--------------------------VPV-SRQRLFEFLR
XP_015941562.1/1-234 --P----PGI-VLSAATSVW--------------------------VPV-SRQRLFEFLR
XP_025620153.1/1-234 --P----PGI-VLSAATSVW--------------------------VPV-SRQRLFEFLR
XP_020239673.1/1-232 --P----PGI-VLSAATSVW--------------------------VPV-TRQRLFDFLR
XP_020239648.1/1-232 --P----PGI-VLSAATSVW--------------------------VPV-TRQRLFDFLR
XP_007143377.1/1-235 --P----PGI-VLSAATSVW--------------------------MPV-SRQRVFDFLR
XP_007143378.1/1-235 --P----PGI-VLSAATSVW--------------------------MPV-SRQRVFDFLR
XP_017415467.1/1-234 --P----PGI-VLSAATSVW--------------------------MPV-SRQRVFDFLR
XP_017415466.1/1-234 --P----PGI-VLSAATSVW--------------------------MPV-SRQRVFDFLR
XP_014514421.1/1-236 --P----PGI-VLSAATSVW--------------------------MPV-SRQRVFDFLR
XP_014514419.1/1-236 --P----PGI-VLSAATSVW--------------------------MPV-SRQRVFDFLR
XP_006606097.1/1-235 --P----PGI-VLSAATSVW--------------------------MPV-SRQRLFDFLR
XP_006606096.1/1-235 --P----PGI-VLSAATSVW--------------------------MPV-SRQRLFDFLR
XP_003536477.1/1-234 --P----PGI-VLSAATSVW--------------------------VPV-SRQRLFDFLR
XP_006589557.1/1-234 --P----PGI-VLSAATSVW--------------------------VPV-SRQRLFDFLR
XP_006402516.1/1-230 --P----PGI-ILNAATSVW--------------------------MPV-SPRRLFDFLG
NP_191674.1/1-237    --P----PGI-ILNAATSVW--------------------------MPV-SPRRLFDFLG
XP_002876596.1/1-239 --P----PGI-ILNAATSVW--------------------------MPI-SPRRLFDFLG
XP_010413451.1/1-238 --P----PGI-VLNAATSVW--------------------------MPV-SPRRLFDFLG
XP_010413452.1/1-238 --P----PGI-VLNAATSVW--------------------------MPV-SPRRLFDFLG
XP_010413453.1/1-238 --P----PGI-VLNAATSVW--------------------------MPV-SPRRLFDFLG
XP_010469036.1/1-242 --P----PGI-VLNAATSVW--------------------------MPV-SPRRLFDFLG
XP_010469037.1/1-242 --P----PGI-VLNAATSVW--------------------------MPV-SPRRLFDFLG
XP_010469038.1/1-242 --P----PGI-VLNAATSVW--------------------------MPV-SPRRLFDFLG
XP_010512448.1/1-238 --P----PGI-VLNAATSVW--------------------------MPV-SPRRLFDFLG
XP_010512449.1/1-238 --P----PGI-VLNAATSVW--------------------------MPV-SPRRLFDFLG
XP_006397757.1/1-219 --P----PGI-LLSAATSVW--------------------------VPV-SPRRLFDFLR
XP_024010837.1/1-220 --P----PGI-LLSAATSVW--------------------------VPV-SPRRLFDFLR
XP_023640300.1/1-220 --P----PGI-LLSAATSVW--------------------------VPV-PPRQLFDFLR
XP_006293718.1/1-220 --P----PGI-LLSAATSVW--------------------------VPV-PPRQLFDFLR
XP_010506553.1/1-219 --P----PGI-LLSAATSVW--------------------------IPV-PPRRLFDFLR
XP_010508114.1/1-219 --P----PGI-LLSAATSVW--------------------------IPV-PPRRLFDFLR
XP_010508115.1/1-219 --P----PGI-LLSAATSVW--------------------------IPV-PPRRLFDFLR
XP_022147276.1/1-219 --P----PGT-VLSAATSVW--------------------------LPAASPERVFEFLR
XP_023527464.1/1-222 --P----PGT-VLSAATSVW--------------------------VAA-TAERVFEFLR
XP_022980233.1/1-222 --P----PGT-VLSAATSVW--------------------------VAA-TAERVFEFLR
XP_022980231.1/1-222 --P----PGT-VLSAATSVW--------------------------VAA-TAERVFEFLR
XP_022924366.1/1-223 --P----PGT-VLSAATSVW--------------------------VAA-TAERVFEFLR
XP_022924364.1/1-223 --P----PGT-VLSAATSVW--------------------------VAA-TAERVFEFLR
XP_023527467.1/1-221 --P----PGT-VLSAATSVW--------------------------VAA-TAERVFEFLR
XP_023527463.1/1-221 --P----PGT-VLSAATSVW--------------------------VAA-TAERVFEFLR
XP_023527465.1/1-221 --P----PGT-VLSAATSVW--------------------------VAA-TAERVFEFLR
XP_022980232.1/1-221 --P----PGT-VLSAATSVW--------------------------VAA-TAERVFEFLR
XP_022924365.1/1-222 --P----PGT-VLSAATSVW--------------------------VAA-TAERVFEFLR
XP_009412599.1/1-224 --P----AGV-VLSAATSVW--------------------------IPES-PKRLFDFLR
XP_018677084.1/1-231 --P----PGV-VLSAATSVW--------------------------LPIA-PQRLFDFLR
XP_020114860.1/1-246 --P----PGV-VLSAATSVW--------------------------LPV-PPHRLFDFLR
XP_020114857.1/1-246 --P----PGV-VLSAATSVW--------------------------LPV-PPHRLFDFLR
XP_020114859.1/1-246 --P----PGV-VLSAATSVW--------------------------LPV-PPHRLFDFLR
XP_020084803.1/1-234 --P----PGV-VLSAATSVW--------------------------VPAVPPHRLFDFLR
XP_015625616.1/1-226 --P----PGV-VLSAATSVW--------------------------VPVA-PEKLFNFLR
XP_006647677.1/1-226 --P----PGV-VLSAATSVW--------------------------VPVA-PEKLFNFLR
NP_001105493.1/1-226 --P----PGV-VLSARTSVW--------------------------VPVA-PEKLFNFLR
XP_020178635.1/1-225 --P----PGV-VLSAATSVW--------------------------VPIA-PEKLFDFLR
XP_003570085.1/1-223 --P----PGV-VLSAATSVW--------------------------VPVA-PEKLFDFLR
XP_020247871.1/1-226 --P----TGV-ILTAVSTAW--------------------------LPFSH-QQVFDLLA
XP_020247870.1/1-226 --P----TGV-ILTAVSTAW--------------------------LPFSH-QQVFDLLA
XP_024004764.1/1-211 --S----GGV-VLCAVSTTF--------------------------LPYSH-HQVFDLLC
XP_018449374.1/1-217 --C----GGV-VLCAVSTTL--------------------------LPYSH-HQVFDLLR
XP_013602839.1/1-215 --C----GGV-VLCGVSTTL--------------------------LPYSH-HQVFDLLR
XP_013655966.1/1-215 --C----GGV-VLCGVSTTL--------------------------LPYSH-HQVFDFLR
XP_009108342.1/1-215 --C----GGV-VLCGVSTTL--------------------------LPYSH-HQVFDFLR
XP_013655964.1/1-215 --C----GGV-VLCGVSTTL--------------------------LPYSH-HQVFDFLR
XP_020872468.1/1-222 --C----GGL-VPCAVSVTL--------------------------LPYSH-HQVFDLLR
NP_193506.2/1-221    --C----GGL-VPCAVSVTL--------------------------LPYSH-QQVFDLLR
XP_023633855.1/1-222 --C----GGL-VPCAVSVTY--------------------------LPYSH-HKVFDLLR
XP_010449572.1/1-221 --S----DGF-VPCAVSVTY--------------------------LPYSH-HKVFDLLR
XP_010439957.1/1-223 --S----DGF-VPCAVSVTY--------------------------LPYSH-HKVFDLLR
XP_010439959.1/1-223 --S----DGF-VPCAVSVTY--------------------------LPYSH-HKVFDLLR
XP_010439958.1/1-223 --S----DGF-VPCAVSVTY--------------------------LPYSH-HKVFDLLR
XP_006854056.1/1-234 --P----IGL-ILCAITTTW--------------------------LPVPP-HQVFDLLR
XP_020529072.1/1-234 --P----IGL-ILCAITTTW--------------------------LPVPP-HQVFDLLR
NP_199499.3/1-224    --P----TGV-VLCAVSTTW--------------------------LPFSH-HQVFDLIR
XP_020871346.1/1-224 --P----TGV-VLCAVSTTW--------------------------LPFSH-HQVFDLIR
XP_006281890.1/1-224 --P----TGV-VLCAVSTTW--------------------------LPFSH-HQVFDLIR
XP_010481400.1/1-224 --P----TGV-VLSAVSTTW--------------------------LPFTH-HQVFDLIR
XP_010494885.1/1-224 --P----TGV-LLSAVSTTW--------------------------LPFTH-HQVFDLIR
XP_018455882.1/1-227 --P----TGV-VLVAVSTTW--------------------------LPFSH-FKVFDLLR
XP_013713146.1/1-227 --P----TGV-VLVAVSTTW--------------------------LPFSH-FKVFDLLR
XP_013607209.1/1-227 --P----TGV-VLVAVSTTW--------------------------LPFSH-FKVFDLLR
XP_009114317.1/1-227 --P----TGV-VLVAVSTTW--------------------------LPFSH-FKVFDLLR
XP_013661958.1/1-227 --P----TGV-VLVAVSTTW--------------------------LPFSH-FKVFDLLR
XP_006398384.1/1-227 --P----TGV-VLAAVSTTW--------------------------LPFSH-LKVFDLLR
XP_024011152.1/1-227 --P----TGV-VLAAVSTTW--------------------------LPFSH-LKVFDLLR
XP_018482944.1/1-227 --P----TGV-VLAAVSTTW--------------------------LPFSH-VKVFDLLR
XP_009101472.1/1-227 --P----TGV-VLAAVSTIW--------------------------LPFSH-AKVFDLLR
XP_013693979.1/1-227 --P----TGV-VLAAVSTTW--------------------------LPFSH-AKVFDLLR
XP_013615089.1/1-227 --P----TGV-VLAAVSTTC--------------------------LPFSH-AKVFDLLR
XP_013704013.1/1-227 --P----TGV-VLAAVSTTC--------------------------LPFSH-AKVFDLLR
XP_009373477.1/1-246 --P----IGV-ILTAVSTTW--------------------------LPYSH-YRVFELLR
XP_006662631.2/1-256 --P----SGV-ILTAVSTSW--------------------------LPFTH-HQVFELLA
XP_015613461.1/1-259 --P----SGV-ILTAVSTSW--------------------------LPFTH-QQVFELLA
NP_001337805.1/1-240 --P----SGV-ILTAVSTSW--------------------------LPFNH-QKVFELLA
XP_020163828.1/1-239 --P----SGV-ILTAVSTSW--------------------------LPFSH-QQVFELLA
XP_010235052.1/1-238 --P----SGV-ILTAVSTSW--------------------------LPFSH-QQVFELLA
XP_025798256.1/1-238 --P----SGV-ILTAVSTSW--------------------------LPFSH-QRVFELLA
XP_004983555.1/1-238 --P----SGV-ILTAVSTSW--------------------------LSFSH-QQVFELLA
XP_002464880.2/1-238 --P----SGV-ILTAVSTSW--------------------------LPFSH-QQVFELLA
XP_020262583.1/1-231 --P----NGV-ILTAVSTTW--------------------------LPFSQ-DQVFDLLT
XP_020677410.1/1-231 --P----NGV-ILSAVSTVW--------------------------LPFSY-HQVFDLLT
XP_020577604.1/1-229 --P----NGV-ILSAVSTAW--------------------------LPFSY-HQVFDLLT
XP_020577606.1/1-229 --P----NGV-ILSAVSTAW--------------------------LPFSY-HQVFDLLT
XP_020577607.1/1-229 --P----NGV-ILSAVSTAW--------------------------LPFSY-HQVFDLLT
XP_019709148.1/1-245 --P----NGV-ILSAVSTTW--------------------------LPFPH-QQVFELLT
XP_008803567.1/1-241 --P----NGV-ILSAASTTW--------------------------LPFPH-QQVFDLLA
XP_008803570.2/1-241 --P----NGV-ILSAASTTW--------------------------LPFPH-QQVFDLLA
XP_018681950.1/1-231 --P----NGV-ILTAVSTTW--------------------------LPSSH-HQVFELLT
XP_018681951.1/1-231 --P----NGV-ILTAVSTTW--------------------------LPSSH-HQVFELLT
XP_010919976.1/1-237 --P----NGV-ILTAVSTTW--------------------------LPFSH-QQVFDLLT
XP_008800084.1/1-237 --P----NGV-ILTAVSTTW--------------------------LPFSH-QQVFDLLT
XP_018679997.1/1-229 --P----NGV-ILTAVSTTW--------------------------LPFSH-QQVFELLT
XP_009397149.1/1-229 --P----NGV-ILTAVSTTW--------------------------LPFSH-QQVFELLT
XP_024995523.1/1-235 --P----HGL-ILTAVSTTW--------------------------LPHPH-YQVFDLLR
XP_023736252.1/1-233 --P----NGL-ILTAVSTTW--------------------------LPHPH-YQVFDLLR
XP_023736253.1/1-233 --P----NGL-ILTAVSTTW--------------------------LPHPH-YQVFDLLR
XP_022022041.1/1-231 --P----NGL-ILTAVSTTW--------------------------LPYPH-YQVFDLLR
XP_021969857.1/1-226 --P----NGL-ILTAVSTIW--------------------------LPHPH-YQVFDLLR
XP_010245213.1/1-236 --P----NGM-ILCAVSTTW--------------------------LPFPH-HQVFDLLR
XP_010245210.1/1-236 --P----NGM-ILCAVSTTW--------------------------LPFPH-HQVFDLLR
XP_010276832.1/1-235 --P----NGT-ILGAVSTSW--------------------------LPFPC-HQVFDLLR
XP_010276837.1/1-235 --P----NGT-ILGAVSTSW--------------------------LPFPC-HQVFDLLR
XP_019155823.1/1-230 --P----AGL-ILSAVSTTW--------------------------LPYHH-FQVFDLLR
XP_022881662.1/1-238 --P----NGL-ILSAVSTIW--------------------------LPFPH-KQVFDFLR
XP_022877113.1/1-234 --P----NGL-ILGAVSTIW--------------------------LPFPH-NKVFDFLR
XP_016450789.1/1-246 --P----NGL-ILCAVSTTW--------------------------IPYNH-FQVFDLLR
XP_009597691.1/1-246 --P----NGL-ILCAVSTTW--------------------------IPYNH-FQVFDLLR
XP_009786380.1/1-246 --P----NGL-ILCAVSTTW--------------------------IPYNH-FQVFDLLR
XP_016494823.1/1-246 --P----NGL-ILCAVSTTW--------------------------IPYNH-FQVFDLLR
XP_016494822.1/1-246 --P----NGL-ILCAVSTTW--------------------------IPYNH-FQVFDLLR
XP_019230103.1/1-246 --P----NGL-ILCAVSTTW--------------------------IPYNH-FQVFDLLR
XP_016573090.1/1-250 --P----NGL-ILSAVSTSW--------------------------LPYNH-FQVFDLLR
XP_016573102.1/1-250 --P----NGL-ILSAVSTSW--------------------------LPYNH-FQVFDLLR
XP_006352911.1/1-250 --P----NGL-ILSAVSTSW--------------------------LPYNH-FQVFDLLR
XP_015166604.1/1-250 --P----NGL-ILSAVSTSW--------------------------LPYNH-FQVFDLLR
XP_015085651.1/1-250 --P----NGL-ILSAVSTSW--------------------------LPYNH-FQVFDLLR
XP_015085654.1/1-250 --P----NGL-ILSAVSTSW--------------------------LPYNH-FQVFDLLR
XP_004245919.1/1-250 --P----NGL-ILSAVSTSW--------------------------LPYNH-FQVFDLLR
XP_025888165.1/1-250 --P----NGL-ILSAVSTSW--------------------------LPYNH-FQVFDLLR
XP_011070327.1/1-236 --P----NGL-ILSAVSTTW--------------------------LPYQH-NQVFDFLR
XP_011070329.1/1-236 --P----NGL-ILSAVSTTW--------------------------LPYQH-NQVFDFLR
XP_012843107.1/1-236 --P----NGL-ILSAVSTTW--------------------------LPYSH-KQVFDFLR
XP_019423575.1/1-234 --P----NGV-ILGAVSTIW--------------------------LPFSH-TKVFDLLR
XP_019423571.1/1-234 --P----NGV-ILGAVSTIW--------------------------LPFSH-TKVFDLLR
XP_019435331.1/1-234 --P----NGV-ILGAASTIW--------------------------LPYPH-TKVFDFLR
XP_003612811.1/1-239 --P----SGV-ILAAVSTTW--------------------------LPYTH-TKVFDLLR
XP_020978415.1/1-227 --P----NGV-ILGAVSTTW--------------------------LPYSH-TKVFDLLR
XP_015963259.1/1-227 --P----NGV-ILGAVSTTW--------------------------LPYSH-TKVFDLLR
XP_025697880.1/1-227 --P----NGV-ILGAVSTTW--------------------------LPYSH-TKVFDLLR
XP_025697882.1/1-227 --P----NGV-ILGAVSTTW--------------------------LPYSH-TKVFDLLR
XP_025697881.1/1-227 --P----NGV-ILGAVSTTW--------------------------LPYSH-TKVFDLLR
XP_020217931.1/1-233 --P----NGV-ILSAVSTTW--------------------------LPYSH-TRVFDLLR
XP_007158253.1/1-232 --P----NGV-ILSAVSTTW--------------------------LPYSH-TKVFDLLR
XP_017427665.1/1-232 --P----NGV-ILSAVSTTW--------------------------LPYSH-TKVFDLLR
XP_017427666.1/1-232 --P----NGV-ILSAVSTTW--------------------------LPYSH-TKVFDLLR
XP_014521371.1/1-232 --P----NGV-ILSAVSTTW--------------------------LPYSH-TKVFDLLR
XP_014521373.1/1-232 --P----NGV-ILSAVSTTW--------------------------LPYSH-TKVFDLLR
XP_006587604.1/1-242 --P----NGV-ILSAVSTTW--------------------------LPYSH-TKVFDLLR
NP_001352051.1/1-233 --P----NGV-ILSAVSTTW--------------------------LPYSH-TKVFDLLR
XP_018840870.1/1-239 --P----NGL-ILCAVSSTW--------------------------LPYPH-NHVFGLLR
XP_018852246.1/1-240 --P----NGL-VLCAVSSTW--------------------------LPYTH-DLVFDLLR
XP_010053633.1/1-234 --P----NGV-VLAAVSTTW--------------------------LPYPH-HHVFELLK
XP_021675948.1/1-236 --P----NGV-ILSAVSTTW--------------------------LPFPH-YHVFELLR
XP_012081064.1/1-228 --P----NGV-ILSAVSTTW--------------------------LPYPH-FQVFDLLR
XP_011028126.1/1-235 --P----NGV-ILSAVSTTW--------------------------LPYPH-YQVFDLLR
XP_002303433.2/1-235 --P----NGV-ILSAVSTTW--------------------------LPYPH-YQVFDLLR
XP_015897558.1/1-247 --P----NGV-IPSAVSTTW--------------------------LPYSH-YQVFDLLR
XP_002277673.1/1-238 --P----NGV-ILSAVSTTW--------------------------LPHPH-YHVFDLLR
XP_022996123.1/1-236 --P----NGV-ILSAVSTTW--------------------------LPYPH-YRVFDLLR
XP_023534245.1/1-236 --P----NGV-ILSAVSTTW--------------------------LPYPH-YGVFDLLR
XP_022931244.1/1-236 --P----NGV-ILSAVSTTW--------------------------LPYPH-YRVFDLLR
XP_022931242.1/1-236 --P----NGV-ILSAVSTTW--------------------------LPYPH-YRVFDLLR
XP_022159691.1/1-235 --P----NGV-ILSAVSTTW--------------------------LPYPP-YRVFDLLR
XP_011652640.1/1-232 --P----NGV-ILSAVSTTW--------------------------LPYPH-YRVFDLLR
XP_011652639.1/1-232 --P----NGV-ILSAVSTTW--------------------------LPYPH-YRVFDLLR
XP_008466007.1/1-232 --P----NGV-ILSAVSTTW--------------------------LPYPH-YRVFDLLR
XP_023511589.1/1-230 --P----NGV-ILSAVSTTW--------------------------LPYPH-YRVFDLLR
XP_022963906.1/1-230 --P----NGV-ILSAVSTTW--------------------------LPYPH-YRVFDLLR
XP_023511591.1/1-230 --P----NGV-ILSAVSTTW--------------------------LPYPH-YRVFDLLR
XP_022963908.1/1-230 --P----NGV-ILSAVSTTW--------------------------LPYPH-YRVFDLLR
XP_022967184.1/1-230 --P----NGV-ILSAVSTTW--------------------------IPYPH-YRVFDLLR
XP_022967186.1/1-230 --P----NGV-ILSAVSTTW--------------------------IPYPH-YRVFDLLR
XP_010518669.1/1-225 --P----TGV-VLCAVSTTW--------------------------LPFSH-HQVFDLLR
XP_010677365.1/1-235 --P----NGL-ILCAVSTTW--------------------------LPYSH-DQVFDLLK
XP_010677364.1/1-235 --P----NGL-ILCAVSTTW--------------------------LPYSH-DQVFDLLK
XP_021852084.1/1-226 --P----NGL-ILCAVTTTW--------------------------LPYSH-DQVFELLK
XP_021852076.1/1-226 --P----NGL-ILCAVTTTW--------------------------LPYSH-DQVFELLK
XP_021765591.1/1-233 --P----NGL-ILCAVSTTW--------------------------LPYSH-DRVFDLLK
XP_021768254.1/1-233 --P----NGL-ILCAVSTTW--------------------------LPYSH-DRVFDLLK
XP_017232690.1/1-221 --P----NGL-ILSAVSTTW--------------------------LPYSH-HQVFDLLR
XP_017232692.1/1-221 --P----NGL-ILSAVSTTW--------------------------LPYSH-HQVFDLLR
XP_024021697.1/1-248 --P----NGL-ILSAVSTTW--------------------------LPYPH-NQVFELLR
XP_010096931.1/1-248 --P----NGL-ILSAVSTTW--------------------------LPYPH-NQVFELLR
XP_024021696.1/1-248 --P----NGL-ILSAVSTTW--------------------------LPYPH-NQVFELLR
XP_017621310.1/1-230 --P----NGL-ILCAVSTTW--------------------------LPYPH-HHVFDLLR
XP_016731150.1/1-230 --P----NGL-ILCAVSTTW--------------------------LPYPH-HHVFDLLR
XP_012472199.1/1-230 --P----NGL-ILCAVSTTW--------------------------LPYPH-HHVFDLLR
XP_016727305.1/1-230 --P----NGL-ILCAVSTTW--------------------------LPYPH-HHVFDLLR
XP_016731145.1/1-230 --P----NGL-ILCAVSTTW--------------------------LPYPH-HHVFDLLR
XP_012472200.1/1-230 --P----NGL-ILCAVSTTW--------------------------LPYPH-HDVFDLLR
XP_012472201.1/1-230 --P----NGL-ILCAVSTTW--------------------------LPYPH-HDVFDLLR
NP_001314348.1/1-230 --P----NGL-ILCAVSTTW--------------------------LPYPH-HHVFDLLR
XP_016727300.1/1-230 --P----NGL-ILCAVSTTW--------------------------LPYPH-HHVFDLLR
XP_022738572.1/1-235 --P----NGL-ILSAVSTTW--------------------------LPYPH-YEVFDLLR
XP_021297648.1/1-235 --P----NGL-ILCAVSTTW--------------------------LPYPH-DQVFDLLR
XP_017973778.1/1-235 --P----NGL-ILCAVSTTW--------------------------LPYPH-DQVFDLLR
XP_017973780.1/1-235 --P----NGL-ILCAVSTTW--------------------------LPYPH-DQVFDLLR
XP_022717458.1/1-235 --P----NGL-ILCAVSTTW--------------------------LPYPH-YQVFDLLR
XP_022717453.1/1-235 --P----NGL-ILCAVSTTW--------------------------LPYPH-YQVFDLLR
XP_022717437.1/1-235 --P----NGL-ILCAVSTTW--------------------------LPYPH-YQVFDLLR
XP_022756982.1/1-232 --P----NGL-ILCAVSTTW--------------------------LPYPH-YQVFDLLK
XP_004300207.1/1-230 --P----NGV-ILSAVSTTW--------------------------LPYSH-YRVFELLR
XP_024169578.1/1-230 --P----NGV-ILSAVSTTW--------------------------LPYSH-YRVFELLR
XP_008238183.1/1-249 --P----IGV-ILSAVSTTW--------------------------LPYSH-FRVFELLR
XP_016651445.1/1-249 --P----IGV-ILSAVSTTW--------------------------LPYSH-FRVFELLR
XP_020418924.1/1-249 --P----IGV-ILSAVSTTW--------------------------LPYSH-YRVFELLR
XP_021804146.1/1-249 --P----IGV-ILSAVSTTW--------------------------LPYSH-YRVFELLR
XP_008238184.1/1-248 --P----IGV-ILSAVSTTW--------------------------LPYSH-FRVFELLR
XP_016651446.1/1-248 --P----IGV-ILSAVSTTW--------------------------LPYSH-FRVFELLR
XP_007209851.1/1-248 --P----IGV-ILSAVSTTW--------------------------LPYSH-YRVFELLR
XP_021804154.1/1-248 --P----IGV-ILSAVSTTW--------------------------LPYSH-YRVFELLR
XP_017179076.1/1-239 --P----IGV-ILTAVSTTW--------------------------LPYSH-YCVFELLR
XP_008356445.1/1-246 --P----IGV-ILTAVSTTW--------------------------LPYSH-YRVFELLR
XP_008356446.1/1-240 --P----IGV-ILTAVSTTW--------------------------LPYSH-YRVFELLR
XP_008356447.1/1-239 --P----IGV-ILTAVSTTW--------------------------LPYSH-YRVFELLR
XP_009373485.1/1-239 --P----IGV-ILTAVSTTW--------------------------LPYSH-YRVFELLR
XP_009373484.1/1-240 --P----IGV-ILTAVSTTW--------------------------LPYSH-YRVFELLR
XP_004512486.1/1-245 --P----NGV-ILAAVSTTW--------------------------LPYSH-TKIFDFLR
XP_023898631.1/1-242 --P----NGL-ILCAVSTTW--------------------------LPYPH-GHVFDLLR
XP_024465375.1/1-238 --P----NGV-ILSAVSTTW--------------------------LPYPH-FLVFDLLR
XP_011018502.1/1-238 --P----NGV-ILSAVSTTW--------------------------LPYHH-FLVFDLLR
XP_011018503.1/1-238 --P----NGV-ILSAVSTTW--------------------------LPYHH-FLVFDLLR
XP_021893615.1/1-233 --P----NGV-ILCAVSTTW--------------------------LPYPH-YHVFDLLR
XP_024952893.1/1-247 --P----NGL-ILCAVSTTW--------------------------LPYPH-YQVFDLLR
XP_006471522.1/1-248 --P----NGL-ILCAVSTTW--------------------------LPYPH-YQVFDLLR
XP_006432745.2/1-248 --P----NGL-ILCAVSTTW--------------------------LPYPH-YQVFDLLR
XP_021640807.1/1-241 --P----NGI-ILSAVSTTW--------------------------MPFPH-YQVFDLIR
XP_002518976.1/1-243 --P----NGV-ILSAVSTTW--------------------------LPYPH-YQVFDILR
XP_021598537.1/1-237 --P----NGV-ILSAVSTTW--------------------------LPFAH-YQVFELLR
XP_021598535.1/1-239 --P----NGV-ILSAVSTTW--------------------------LPFAH-YQVFELLR
XP_006659280.1/1-226 --P----VGQ-LLSATTTIW--------------------------LPGVKPRHVYDYLH
XP_025801379.1/1-254 --P----ASW-FLSATTTLW--------------------------LPGTPPERVFDYLR
XP_023156326.1/1-229 --V----ADL-VLSASTTVW--------------------------LPNTPPQLVFRYLR
NP_001349156.1/1-229 --V----ADL-VLSASTTVW--------------------------LPNTPPQLVFRYLR
XP_021310588.1/1-246 --V----ADL-VLSASTTVW--------------------------LPNTPPQLVFQYLC
XP_020404516.1/1-224 --S----PLQ-VLSATTTVW--------------------------LPGTPPESVFNYLC
XP_022684239.1/1-229 --P-----AL-VLCATTTVW--------------------------LPGVPPHRVHEYLC
XP_020404892.1/1-233 --A-----VT-VLSATTTVW--------------------------LPGTPPMRVREYLF
XP_022684238.1/1-237 --A-----IT-VLSATTTVR--------------------------LPGTPPVRVFEYLC
XP_025825251.1/1-229 --A-----IT-VLSATATVR--------------------------LPGTPPLRVFEYIC
XP_020183090.1/1-242 --A----AGP-VLCATTTVW--------------------------LPEIPPQRVFDYIC
XP_020194819.1/1-240 --T----GAT-VLRATTTVW--------------------------LPGTPAQQVFNYLC
XP_024314575.1/1-244 --D----HQG-VLSATTTVW--------------------------LPGVPAGRVFHYLC
XP_024314342.1/1-235 --G----TQL-ILSAMTTVW--------------------------LPGIPAERVFNYLY
XP_024959674.1/1-192 --P----DGH-VIMMASSVR--------------------------SSF-TVDHIYDALR
XP_019196338.1/1-215 --P----RGV-SITIYTSVL--------------------------LPI-PQITLFEFLK
XP_006646400.1/1-226 --S----TNA-VLSVSASLR--------------------------LPL-PMRKIFDLLR
XP_015632244.1/1-226 --S----YNA-ILSVTASLR--------------------------LPL-PMRKTFDLLR
XP_024314625.1/1-231 --S----NTS-VVSVSASFH--------------------------LPI-PLRVTFDLLR
XP_020164122.1/1-228 --S----NNA-VVSVCASFH--------------------------LPV-PLKVTFDLLR
XP_025814677.1/1-221 --S----NTA-IVCTSATFL--------------------------LPL-PMRMVFDLLK
XP_022682466.1/1-225 --S----NTA-VVCASATFL--------------------------LEL-PMRRVFDLLK
XP_002456467.1/1-226 --S----PTA-VVCASASFL--------------------------VPL-HMGKAFELLK
XP_008674944.1/1-228 --S----NTA-VVCACASFL--------------------------LPL-RMRKAFDLLK
XP_010523539.1/1-228 --P-----CV-LLSASGLVR--------------------------MQA-KPDVIFGLLC
XP_020873847.1/1-203 --P-----CG-IISASGLAK--------------------------IHA-KPEMLFPFIY
NP_001190248.1/1-203 --P-----CV-LVSATGLAR--------------------------MHT-KPEVMFGLIN
NP_196343.2/1-203    --P-----CV-LVSATGLAR--------------------------MHT-KPEVMFGLIN
XP_020876233.1/1-195 --P-----CV-LVTATGLAR--------------------------MQA-RPEVIFGLIK
XP_010423235.1/1-188 --P-----WG-FVSATGMAR--------------------------MQV-TQEILFRLIN
XP_010452682.1/1-218 --P-----WG-FVSATGMAR--------------------------MQV-TQEILFRLIN
XP_010491330.1/1-188 --P-----WG-FVSATGMAR--------------------------MQV-TQEILFRLIN
XP_006413300.2/1-224 --H----TGL-VLNVATSVW--------------------------FPV-NQQTMFDFLT
XP_022008499.1/1-216 --V----SGV-VMSATHSVW--------------------------IAA-NHRHLFEMML
XP_022008502.1/1-215 --V----SGV-VMSATHSVW--------------------------IAA-NHRHLFEMML
XP_022007635.1/1-226 --S----MGI-VTSATYSIW--------------------------IPT-NHQHLFNMLL
XP_022008501.1/1-224 --P----MGI-VTSATYSAW--------------------------MPA-NHQHLFNMLI
XP_021986984.1/1-226 --P----MGI-VTSATYLVW--------------------------IPT-NHQHLFDMLK
XP_021996009.1/1-224 --P----MGI-VTSATYSVW--------------------------IPT-NHQHLFDMLK
XP_021996011.1/1-224 --P----MGI-VTSATYSVW--------------------------IPT-NHQHLFDMLK
XP_022026050.1/1-224 --P----MGI-VTSATYSVW--------------------------IPT-SHQYLFDMLK
XP_022026083.1/1-224 --P----MGI-VTSATYSVW--------------------------IPT-NHRHLFDMLK
XP_021722389.1/1-209 --Q----SPG-LLTVTTSSW--------------------------FPF-SAKKVFSFLG
XP_021847315.1/1-219 --Q----SPA-LLTVTTSSW--------------------------FPF-SANKVFSFLG
XP_020224300.1/1-216 --P----EGI-IATAVTSIR--------------------------LPV-PPHQIVSLLT
XP_021765248.1/1-224 --P----TGL-VISVARSFW--------------------------VPA-SPNKVVEFLS
XP_004510020.1/1-219 --K----DGI-LFTATGSVR--------------------------LHV-APETVFYFLS
XP_004487953.1/1-231 --R----NLM-VLTCVAYAW--------------------------ISV-SPHTLFYFLN
XP_008244376.1/1-218 --I-----LG-TTVFATSVH--------------------------LPA-PSKQVFSLLR
XP_008244380.1/1-218 --I-----LG-TTVFATSVH--------------------------LPA-PSKQVFSLLR
XP_012569676.1/1-224 --P----NGM-VIVAASTIW--------------------------LPQ-PSDKVIEFLT
XP_019418393.1/1-229 --G----NRR-SLVAAASFW--------------------------LPN-PLNDVFHFFI
XP_019440118.1/1-223 --E----NAK-SLVVATSFW--------------------------VPN-PSNDVVNFFI
XP_014517363.2/1-226 --N----AMI-IFIATASFR--------------------------LPL-PSEYVFDFFR
XP_012569675.1/1-231 --S----NVS-IVVGASSAP--------------------------FPL-PSKILFDFLK
XP_004486570.1/1-223 --------SS-IVIVASSIS--------------------------LPI-PSHIVFNFLK
XP_003594569.3/1-220 --N----DPF-IVIVASSVS--------------------------LPL-PSHTVFDFLR
XP_024632692.1/1-220 --N----DPF-IVIVASSVS--------------------------LPL-PSHTVFDFLR
XP_025887127.1/1-229 --Y----VGV-ILSATKTIW--------------------------LPTQQ-QTLFKFFN
XP_015078684.1/1-230 --Y----VGV-ILSATKTIW--------------------------LPTQQ-QTLFKFFN
XP_025887126.1/1-230 --Y----VGV-ILSATKTIW--------------------------LPTQQ-QTLFKFFN
XP_015078401.1/1-220 --P----NGI-VLSATKTIW--------------------------LPVKH-QRLFEFLI
XP_015158871.1/1-217 --P----SGT-VLSATKTIW--------------------------LPVKH-QRLFEFLM
XP_015078778.1/1-220 --P----IGV-ILSATKTIQ--------------------------LPIKS-QCLFQFFT
XP_010321992.1/1-216 --P----IGV-ILSATKTIQ--------------------------LPIKS-QCLFQFFT
XP_004240735.1/1-216 --P----IGV-ILSATKTIQ--------------------------LPIKS-QCLFQFFT
XP_015169793.1/1-213 --P----IGI-VLCAIKTIR--------------------------LPVKQ-QHLFEFFI
XP_006285631.1/1-212 --Y----TGI-VLSAAISVW--------------------------FPVNQ-RTLFAFLN
XP_023635531.1/1-198 --Y----TGI-VLSAAISVW--------------------------FPVNQ-RTLFAFLN
NP_567722.1/1-217    --L----TGI-VLSASTSVW--------------------------LPVNQ-HTLFAFIS
XP_020873337.1/1-219 --P----TGI-LLSAATSVW--------------------------FPVKQ-KALFAFLS
XP_020873338.1/1-219 --P----TGI-LLSAATSVW--------------------------FPVKQ-KALFAFLS
XP_023000325.1/1-225 --P----LGL-ILCAVASVW--------------------------LPISP-KLLFEFLI
XP_023000326.1/1-214 --P----LGL-ILCAVASVW--------------------------LPISP-KLLFEFLI
XP_022963997.1/1-214 --P----LGL-ILCAVASVW--------------------------LPISP-KLLFEFLI
XP_023513617.1/1-208 --P----LGL-ILCAVASVW--------------------------LPISP-KLLFEFLI
XP_021312124.1/1-225 --P----QGL-VACAVLSQW--------------------------LPVNP-AAIFGYLL
XP_012701492.1/1-243 --P----RGL-IACAVLSAW--------------------------LPVNP-AALFDFLR
XP_025815339.1/1-229 --P----QGL-IACAVLSAW--------------------------LPVNP-AALFDFLR
XP_015644459.1/1-237 --P----QGL-IACAAASTW--------------------------LPVNP-TALLDLLR
XP_024314016.1/1-228 --P----QGL-IVCAVLSTW--------------------------LPVSP-TALLDFVR
XP_020168333.1/1-225 --P----QGL-IACAVLSTW--------------------------LPVSP-TALLDFLR
XP_013589242.1/1-206 --H----TRI-VLSAATSLW--------------------------LPVSK-KTTFDFLI
XP_022548456.1/1-204 --H----TRI-VLSAATSLW--------------------------LPVSK-KTAFDFLI
XP_024632691.1/1-227 --P----NGT-VVTAATTLW--------------------------LPLPA-QKVFEFLK
XP_017256377.1/1-220 --P----HGV-VITVATSLR--------------------------LNIPP-NDVFEFLC
XP_009632057.1/1-224 --P----RGV-AVTISTSVW--------------------------LPIPQ-KDVFEFLR
XP_016502932.1/1-224 --P----RGV-AVTISTSVW--------------------------LPIPQ-KDVFEFLR
XP_019258997.1/1-221 --P----RGV-AVTISTSVW--------------------------LPIPQ-KDVFEFLR
XP_009792862.1/1-224 --P----RGV-AVTISTSVW--------------------------LPIPQ-KDVFEFLR
XP_016544784.1/1-213 --P----RGV-SVMISTSVW--------------------------LPIPQ-NDLFKFFR
XP_015089689.1/1-213 --P----RGV-VVMISTSVW--------------------------LPIPQ-NNVFKFLR
XP_025883585.1/1-213 --P----RGV-VVMISTSVW--------------------------LPIPQ-NNVFKFLR
XP_011095790.1/1-221 --P----RGV-TVTVATSVW--------------------------LPVKQ-NDVFSFLH
XP_020553953.1/1-221 --P----RGV-TVTVATSVW--------------------------LPVKQ-NDVFSFLH
XP_022857758.1/1-208 --P----CGV-SVTIATSLH--------------------------LQVRQ-SDVFHFFR
XP_004487739.1/1-224 --P----LGT-TVVFCTTIW--------------------------LNVSP-NRLFNFLR
XP_003596663.1/1-221 --P----IGT-SLFFCTTLW--------------------------LNVSP-NRLFNFLR
XP_003596665.1/1-227 ---------A-SLVFCTTLW--------------------------LNVSP-NRLFNFLR
XP_020201972.1/1-216 --L----HAT-TVVFTTSLW--------------------------LDVSP-NQLFDFLR
XP_025980734.1/1-220 --P----PGT-SAVFTTSVW--------------------------LEVSP-NRLFNFLR
XP_007149715.1/1-218 --P----PGT-SVVFTTSVW--------------------------LDVSP-NRLFNFLR
XP_017423636.1/1-218 --P----PGT-SVVFTTSVW--------------------------LDVSR-NRLFNFLR
XP_022631965.1/1-218 --P----PGT-SVVFTTSVW--------------------------LDVSR-TRLFNFLR
XP_014522034.2/1-218 --P----PGT-SVVFTTSVW--------------------------LDVSR-TRLFNFLR
XP_025669173.1/1-223 --P----IAT-TVVFSTCLW--------------------------LNVSP-SRLFNFLR
XP_020963786.1/1-223 --P----IAT-TVVFSTCLW--------------------------LNVSP-SRLFNFLR
XP_025614692.1/1-223 --P----IAT-TVVFSTCLW--------------------------LKVSP-SRLFNFLR
XP_015936351.1/1-223 --P----IAT-TVVFSTCLW--------------------------LKVSP-SRLFNFLR
XP_019451675.1/1-226 --P----IGT-SVVFTTSLW--------------------------VNASP-NQLFNFLR
XP_019465428.1/1-219 --P----PGT-SVIFTTSVW--------------------------IHASP-NRLFNFLR
XP_004295798.2/1-220 --P----PGT-TLSFATSVH--------------------------LPVPR-TQIYNLLR
XP_021807338.1/1-219 --I----LGT-TTVFATSVH--------------------------LPAPS-KQVFSLLR
XP_021807340.1/1-219 --I----LGT-TTVFATSVH--------------------------LPAPS-KQVFSLLR
XP_020417197.1/1-219 --I----LGT-TTVFATSVH--------------------------LPVPS-KQVFSLLR
XP_020417200.1/1-219 --I----LGT-TTVFATSVH--------------------------LPVPS-KQVFSLLR
XP_009333923.1/1-206 --P----PGS-TVVFTTSIN--------------------------LPFPR-KQLFSFLR
XP_017184363.1/1-206 --P----PGS-TVVFTTSIN--------------------------LPFPR-KQVFSFLR
XP_021892911.1/1-224 --E----LSY-TVAFTTSVY--------------------------LPCAP-KQTFDFLR
XP_002278971.2/1-210 --P----PGT-AIVIATSVW--------------------------VPASP-RRVFDFLH
XP_010644412.1/1-211 --R----PGP-ALVMATTIR--------------------------IPASS-RRIFDFLQ
XP_024450660.1/1-210 --S----PSS-TLVFTTSLW--------------------------VPPPP-KRVFDFLR
XP_002522867.2/1-214 --W----SIT-TIAFTYSLW--------------------------LPAPP-SRVFDFLR
XP_020534605.1/1-220 --S----PTT-SIAFASSLW--------------------------LPAPP-RSIFNFLR
XP_021685850.1/1-217 --C----PST-TIAFTSSIW--------------------------LPFPP-RRVYDFLR
XP_021594464.1/1-220 --C----ATA-TIAFTSSLW--------------------------LPLPP-RRVFDFLR
XP_018731177.1/1-223 --P----YGS-SIAFTTSVR--------------------------LPFPV-KKLFNFLS
XP_024018593.1/1-217 --P----LVA-TVVFATSVE--------------------------LPATP-KKVFDFLS
XP_022139420.1/1-219 --P----PCT-SVIFSTSVH--------------------------IPTTP-RQVFHFLR
XP_022981426.1/1-220 --P----PCT-SVIFATSAQ--------------------------VPNNP-NEVFQFLR
XP_022981427.1/1-220 --P----PCT-SVIFATSAQ--------------------------VPNNP-NEVFQFLR
XP_022941489.1/1-220 --P----PCT-SVIFATSAQ--------------------------VPNNP-NEVFQFLR
XP_022941488.1/1-220 --P----PCT-SVIFATSAQ--------------------------VPNNP-NEVFQFLR
XP_023525599.1/1-220 --P----PCT-SVIFTTSAQ--------------------------VPNNP-NEVFQFLR
XP_023525597.1/1-220 --P----PCT-SVIFTTSAQ--------------------------VPNNP-NEVFQFLR
XP_008454681.2/1-220 --P----PCT-SVVFATSVH--------------------------VPTNP-KHLFHYLR
XP_011648553.1/1-223 --P----PCT-SVVFSTSVH--------------------------VPTNP-KHLFHYLR
XP_018822340.1/1-211 --P----PGA-SVAFATSIW--------------------------LPGSP-KFLFNFLR
XP_018822367.1/1-211 --P----PGA-SVAFATSIW--------------------------LPGSP-KFLFNFLR
XP_023876961.1/1-210 --P----PGP-SVVFATSVQ--------------------------IPASR-KRICNFLQ
XP_023876962.1/1-210 --P----PGP-SVVFATSVQ--------------------------IPASR-KRICNFLQ
XP_024034242.1/1-215 --P----PSS-KIVFTTSIR--------------------------LAVPP-KRLFDFLR
XP_015381349.2/1-215 --P----PSS-KIVFTTSIR--------------------------LAVPP-KRLFDFLR
XP_022717683.1/1-216 --P----PSS-TIIFTTSLR--------------------------LPIPT-KILFDFLR
XP_016729588.1/1-216 --P----SGN-IVIFTTSIQ--------------------------IPVPM-EVLFDFLR
XP_016729589.1/1-216 --P----SGN-IVIFTTSIQ--------------------------IPVPM-EVLFDFLR
XP_016702140.1/1-208 --P----SGN-IVIFTTSIQ--------------------------IPVPM-EVLFDFLR
XP_012460158.1/1-216 --P----SGN-IVIFTTSIQ--------------------------IPVPM-EVLFDFLR
XP_012460145.1/1-216 --P----SGN-IVIFTTSIQ--------------------------IPVPM-EVLFDFLR
XP_017245631.1/1-197 --P----DSV-VLSAAATTW--------------------------FPSAP-LSIFNFLI
XP_004956045.3/1-247 --P----ADL-VLSATTTLW--------------------------LPSTPPERVFDYLR
XP_014660176.1/1-240 --P----AGL-VLSSSTTVW--------------------------LPNTPPQLVFEYLC
XP_017239609.1/1-213 --P----NAT-VLSVSTSFV--------------------------VPFAP-KMVFDSLR
XP_023740378.1/1-206 --P----PGT-ILSATISVW--------------------------IPITH-RRLFDLLL
XP_020095129.1/1-235 --P----TGV-ALSACISFW--------------------------LAVPM-RRVFEFLR
XP_020258373.1/1-212 --P----SGV-IVNASVSFW--------------------------VPAMP-KRVFDFLC
XP_008812148.1/1-175 --P----PGI-LVNACMSLW--------------------------LPLPP-GR------
XP_010930674.1/1-230 --P----PGI-TVNASTSLW--------------------------LPMPP-GRIFHFLR
XP_010931877.1/1-230 --P----PGI-TINASTSLW--------------------------LPLPP-GRIFHFLR
XP_009380061.1/1-225 -LP----HGV-VLSAATSIW--------------------------LPL-PRDRVFSFLK
XP_010939971.1/1-229 --P----TGL-ILGAATSIW--------------------------LPV-PSQRVFSFLK
XP_019710626.1/1-218 --P----TGL-ILGAATSIW--------------------------LPV-PSQRVFSFLK
XP_008787891.1/1-229 --P----SGL-ILSAATSIW--------------------------LPV-PSQRVFSFLK
XP_008787892.1/1-218 --P----SGL-ILSAATSIW--------------------------LPV-PSQRVFSFLK
XP_008799024.1/1-230 --P----NGL-ILSATTSIW--------------------------LPL-PSERVFSFIK
XP_010936845.1/1-230 --P----SGL-ILSATTSIW--------------------------LPL-PSQRVFSYIK
XP_003570542.1/1-223 --P----NGV-VLSAATSIW--------------------------LPV-PCDRVYAFVR
XP_004964922.1/1-229 --P----NGV-VLSAATSIW--------------------------LPV-PGDHVFAFVR
XP_002438026.1/1-230 --P----NGV-VLSAATSIW--------------------------LPV-PGDHVFAFVR
XP_020399989.1/1-232 --P----NGV-VLSAATSIW--------------------------LPV-PGDHVFAFVR
XP_008649207.1/1-222 --P----NGV-VLSAATSIW--------------------------LPV-PGDHVFAFVR
XP_020256822.1/1-228 --P----NGV-VLSAATSTW--------------------------LPL-SCQRVFSFFK
XP_009403550.1/1-228 --P----SGV-VLCAATSIW--------------------------LPV-SAERVFSLFK
XP_020582158.1/1-232 --P----NGV-FLCAATSLW--------------------------LPY-SPHSVFSFLR
XP_020699118.1/1-240 FQP----NGV-FLSAATSIW--------------------------LPY-SPESVFSFFR
XP_020600375.1/1-232 --P----NGV-FLSAATSIW--------------------------MPF-SPQAVFSFFR
XP_020600376.1/1-232 --P----NGV-FLSAATSIW--------------------------MPF-SPQAVFSFFR
XP_020684394.1/1-232 --P----NGV-FLTAATSIW--------------------------LPF-SPQAVFSFFR
XP_017257147.1/1-219 --P----NSM-VLSAATSIW--------------------------LPF-SPQKVFDFLR
XP_010244428.1/1-230 --P----TGV-ILSAATSIW--------------------------VPV-PWQNVFNFIR
XP_010244355.1/1-230 --P----TGV-ILSAATSIW--------------------------VPV-PWQNVFNFIR
XP_017240757.1/1-236 --S----HGM-VLSAATTIW--------------------------LPV-PSQIVFNFFK
XP_017245021.1/1-233 --P----HGM-VLSAAATIW--------------------------LPL-PPQIVFNFFR
XP_012829269.1/1-233 --P----NGV-ILSAAATVW--------------------------LPI-APQIVFNFFR
XP_022889938.1/1-236 --P----NAV-VLSAATTIW--------------------------LPI-SAQHIFNFFR
XP_022897471.1/1-232 --P----NAV-VLSAATTIW--------------------------LPV-SAQNIFNFFR
XP_009606250.1/1-235 --P----NGV-IISAASTIW--------------------------LPV-PSQHIFNFLR
XP_016468230.1/1-235 --P----NGV-IISAASTIW--------------------------LPV-PSQHIFNFLR
XP_019250773.1/1-235 --P----NGV-IISAASTIW--------------------------LPL-PSQHVFNFLR
XP_009778533.1/1-235 --P----NGI-IISAASTIW--------------------------LPV-PSQHVFNFLR
XP_016451582.1/1-235 --P----NGI-IISAASTIW--------------------------LPV-PSQHVFNFLR
XP_016578176.1/1-240 --P----NGV-VISAASTIW--------------------------LPV-PPQHVFNFLR
XP_006347370.1/1-231 --P----NGV-VISAASTIW--------------------------LPV-PPQHVFNFLR
XP_004241474.1/1-230 --P----NGV-VISAASTIW--------------------------LPV-PPQHVFNFLR
XP_015077748.1/1-230 --P----NGV-VISAASTIW--------------------------LPV-PPQHVFNFLR
XP_022037776.1/1-241 --P----HGL-ILSASTTIW--------------------------LPI-PPQSVFDFLR
XP_024992062.1/1-237 --P----NSM-VLSASTTIW--------------------------LPV-PPQNVFDFFR
XP_023772270.1/1-245 --P----NGM-VLSASTTIW--------------------------LPV-PPQNVFDFFR
XP_012855673.1/1-237 --P----NAV-VLSAAATIW--------------------------LPI-PPQSVFNFFR
XP_022858328.1/1-236 --P----NAM-VLSAASTIW--------------------------LPI-PPENAFSFFR
XP_011072039.1/1-232 --P----NGV-VLSAAATIW--------------------------LPV-PPQNVFNFFK
XP_006306921.1/1-215 --P----NGM-VLCAATSFW--------------------------LPN-SPQNVFNFLK
NP_564041.2/1-215    --P----NGM-VLCAATSFW--------------------------LPI-SPQNVFNFLK
XP_020866823.1/1-216 --P----NGM-VLCAATSFW--------------------------LPI-SPQNVFNFLK
XP_010476956.1/1-215 --P----NGM-VLCAATSFW--------------------------LPN-SPQNVFNFLK
XP_010498167.1/1-217 --P----NGM-VLCAATSFW--------------------------LPN-SPQNVFNFLK
XP_019091528.1/1-217 --P----NGM-VLCAATSFW--------------------------LPN-SPQNVFNFLK
XP_013613976.1/1-227 --P----NGM-VLCAATSFW--------------------------LPV-SPQNVFNYLK
XP_018432674.1/1-227 --P----NGM-VLCAATSFW--------------------------LPV-SPQRVFNFLK
XP_018432667.1/1-227 --P----NGM-VLCAATSFW--------------------------LPV-SPQRVFNFLK
XP_013641731.1/1-225 --P----NGM-VLCAATSFW--------------------------LPV-SPQNVFNFLK
XP_009149243.1/1-227 --P----NGM-VLCAATSFW--------------------------LPV-SPQNVFNFLK
XP_006416656.2/1-224 --P----NGM-VLCAATSFW--------------------------LPI-SPQNVFNFLK
XP_018451633.1/1-224 --P----NGT-VLCAATSFW--------------------------LPV-SPLIVFNFLK
XP_018451632.1/1-225 --P----NGT-VLCAATSFW--------------------------LPV-SPLIVFNFLK
XP_018451634.1/1-195 --P----NGT-VLCAATSFW--------------------------LPV-SPLIVFNFLK
XP_013690958.1/1-228 --P----NGM-VLCAATSFW--------------------------LPV-SPLIVFNFLK
XP_009110376.1/1-227 --P----NGM-VLCAATSFW--------------------------LPV-SPLIVFNFLK
XP_009110377.1/1-227 --P----NGM-VLCAATSFW--------------------------LPV-SPLIVFNFLK
XP_013601964.1/1-227 --P----NGM-VLCAATSFW--------------------------LPV-SPLIVFNFLK
XP_013653874.1/1-227 --P----NGM-VLCAATSFW--------------------------LPV-SPLIVFNFLK
XP_018445004.1/1-237 --P----NGT-VLSAATTFW--------------------------LPN-SPQNVFNFLK
XP_018445005.1/1-237 --P----NGT-VLSAATTFW--------------------------LPN-SPQNVFNFLK
XP_013648946.1/1-228 --P----NGT-ILSAATTFW--------------------------LPN-SPQNVFNFLK
XP_009104812.1/1-228 --P----NGT-ILSAATTFW--------------------------LPN-SPQNVFNFLK
XP_013588523.1/1-228 --P----NGT-ILSAATTFW--------------------------LPN-SPQNVFNFLK
XP_013699248.1/1-228 --P----NGT-ILSAATTFW--------------------------LPN-SPQNVFNFLK
XP_018457296.1/1-228 --P----NGT-ILSAATTFW--------------------------LPN-SPQNVFNFLK
XP_009105998.1/1-230 --P----NGT-ILCAATTFW--------------------------LPN-SPQNVFNFLK
NP_001302850.1/1-231 --P----NGT-ILCAATTVW--------------------------LPN-SPQNVFNFLK
XP_013698656.1/1-231 --P----NGT-ILCAATTFW--------------------------LPN-SPQSVFNFLK
XP_013649619.1/1-231 --P----NGT-ILCAATTFW--------------------------LPN-SPQSVFNFLK
XP_013592873.1/1-231 --P----NGT-ILCAATTFW--------------------------LPN-SPQSVFNFLK
XP_006390565.1/1-233 --P----NGT-ILCAATTFW--------------------------LPS-SPQNVFNFLK
XP_006301097.1/1-230 --P----NGT-VLCAATTFW--------------------------LSN-SPQNVFNFLK
XP_010428260.1/1-231 --P----NGT-VLCAATTFW--------------------------LPN-SPQNVFNFLK
XP_010471396.1/1-230 --P----NGT-VLCAATTFW--------------------------LPN-SPQNVFNFLK
XP_010416135.1/1-230 --P----NGT-VLCAATTFW--------------------------LPN-SPQNVFNFLK
NP_177479.1/1-227    --P----NGT-VLCAATTFW--------------------------LPN-SPQNVFNFLK
XP_020890987.1/1-228 --P----NGT-ILCAATTFW--------------------------LPN-SPQNVFNFLK
XP_010537520.1/1-232 --P----NGM-ILCAATTFW--------------------------LPI-SPQNIFNFLK
XP_010540042.1/1-226 --P----NST-VLCAATTFW--------------------------LPV-SPQNIFNFLK
XP_010540046.1/1-196 --P----NST-VLCAATTFW--------------------------LPV-SPQNIFNFLK
XP_019432660.1/1-235 --S----NGA-VLSSATTIR--------------------------LPI-PPNIVFSFLK
XP_019420678.1/1-233 --T----NGV-VLSAATTIW--------------------------LPI-PLHIVFNFFK
XP_013452816.1/1-243 --P----SGV-VLCAATTIW--------------------------LPL-PPQTVFNFFK
XP_020215886.1/1-221 -------NGV-VLSAATTIW--------------------------LPT-PPHTVFNFFK
XP_020215888.1/1-221 -------NGV-VLSAATTIW--------------------------LPT-PPHTVFNFFK
XP_007160572.1/1-230 --P----NSV-VLSAATTIW--------------------------LPT-PPHTVFNFFK
XP_017424661.1/1-212 --P----NGV-VLSAATTIW--------------------------LPT-PPHTVFNFFK
XP_017424660.1/1-230 --P----NGV-VLSAATTIW--------------------------LPT-PPHTVFNFFK
XP_014508329.1/1-230 --P----NGV-VLSAATTIW--------------------------LPT-PPHTVFNFFK
XP_022632295.1/1-230 --P----NGV-VLSAATTIW--------------------------LPT-PPHTVFNFFK
XP_019225984.1/1-234 --P----NGV-IISAATTIW--------------------------LPV-PPQNVFNFFR
XP_016441514.1/1-233 --P----NGV-IISAATTIW--------------------------LPV-PPQNVFNFFR
XP_009587298.1/1-233 --P----NGV-IISAATTIW--------------------------LPV-PPQNVFNFFR
XP_016441515.1/1-233 --P----NGV-IISAATTIW--------------------------LPV-PPQNVFNFFR
XP_009786509.1/1-233 --P----NGV-IISAATTIW--------------------------LPV-PPQNVFNFFR
XP_016563952.1/1-234 --P----NGV-VISAATTIW--------------------------LPI-PPQHVFNFFR
XP_004235382.1/1-234 --P----NGV-VISAAATIW--------------------------LPI-PPQQVFNFFR
XP_015068940.1/1-234 --P----NGV-VISAAATIW--------------------------LPI-PPQQVFNFFR
XP_006364383.1/1-234 --P----NGV-VISAAATIW--------------------------LPI-PPQHVFNFFR
XP_019167409.1/1-245 --P----NGV-VISAATTIW--------------------------LPI-SPQLVFNFFR
XP_011086973.1/1-238 --P----NGV-VLSAAATIW--------------------------LPI-PPQIVFNFFR
XP_010268321.1/1-230 --P----NGV-VLSATTSIW--------------------------LPV-SPQNVFNFFR
XP_010268322.1/1-230 --P----NGV-VLSATTSIW--------------------------LPV-SPQNVFNFFR
XP_010069492.1/1-236 --P----NGV-VLSAATTIW--------------------------LPL-SPQNVFNFFK
XP_002527933.1/1-229 --P----NGV-VLNAATTFW--------------------------LPV-SPQNVFNFFK
XP_021678368.1/1-229 --P----NGV-VLNAATTFW--------------------------LPV-SPQNVFNFFK
XP_021678369.1/1-229 --P----NGV-VLNAATTFW--------------------------LPV-SPQNVFNFFK
XP_021602738.1/1-229 --P----NGV-VLNAATTFR--------------------------LPV-SPQNVFNFFK
XP_012079215.1/1-230 --P----NGV-VLNAATTFW--------------------------LPV-SPQNVFNFFK
XP_021687701.1/1-229 --P----NGV-VLNAATTFW--------------------------LPV-SPQNVFNFFK
XP_006374212.1/1-231 --P----NGV-VLSAATTFS--------------------------LPV-SPQNVFSFFK
XP_011035552.1/1-231 --P----NGV-VLSAATTFS--------------------------LPV-SPQNVFSFFK
XP_002317722.3/1-231 --P----NGV-VLSAATTFS--------------------------LPV-SPQNVFNFFK
XP_011000766.1/1-231 --P----NGV-VLSAATTFS--------------------------LPV-SPQIVFNFFK
XP_021907827.1/1-230 --P----SGV-VFSAATTFW--------------------------LPV-SPQNVFNFFK
XP_017635401.1/1-230 --P----NGI-VLSAATTFW--------------------------LPV-SPQNVFSFFK
XP_016686699.1/1-230 --P----NGI-VLSAATTFW--------------------------LPV-SPQNVFNFFK
XP_012440361.1/1-230 --P----NGI-VLSAATTFW--------------------------LPV-SPQNVFNFFK
NP_001313868.1/1-230 --P----NGI-VLSAATTFW--------------------------LPV-SPQNVFNFFK
XP_023919022.1/1-230 --P----NGV-VLSAATTIW--------------------------LPI-SPQNVFNFFK
XP_006478112.1/1-232 --P----NGV-VLNAATTFW--------------------------LPV-PPQNVFNFFK
XP_024042646.1/1-232 --P----NGV-VLNAATTFW--------------------------LPI-PPQNVFNFFK
XP_022715403.1/1-231 --P----NGV-VLSAATSFW--------------------------LPV-TPQNVFNFFK
XP_022767009.1/1-255 --P----NGV-VLSAATTFW--------------------------LPV-TPQNVFIFFK
XP_022737050.1/1-231 --P----NGV-VLSAATTFW--------------------------LPV-TPQNVFNFFK
XP_017973835.1/1-231 --P----NGV-VLSAATTFW--------------------------LPV-TPQNVFNFFK
XP_015967008.1/1-238 --P----SGV-VLSAATTIW--------------------------LPL-PPQTVFNFFK
XP_025701434.1/1-239 --P----SGV-VLSAATTIW--------------------------LPL-PPQTVFNFFK
XP_020980110.1/1-239 --P----SGV-VLSAATTIW--------------------------LPL-PPQTVFNFFK
XP_019434881.1/1-231 --P----NGV-VLSAATTIW--------------------------LPI-SPHIVFNFFK
XP_019458275.1/1-234 --P----NGV-VLSAATTIW--------------------------LPI-PPHIVFNFFK
XP_019458276.1/1-234 --P----NGV-VLSAATTIW--------------------------LPI-PPHIVFNFFK
XP_006584922.1/1-234 --P----NGV-VLSAATTIW--------------------------LPT-PPHAVFNFFK
XP_003524332.1/1-231 --P----NGV-VLSAATTIW--------------------------LPT-PPHTVFNFFK
XP_004515249.1/1-229 --P----NGV-VLSAATTIW--------------------------LPL-PPQTVFNFFK
XP_019431381.1/1-229 --P----NGV-VLSAATTIW--------------------------LPI-PPQIVFNFFK
XP_019436771.1/1-231 --P----NGV-VLSAATTIW--------------------------LPI-PPQTVFNFFK
XP_019430342.1/1-233 --P----NGV-VLSAATTIW--------------------------LPI-PPQIVFNFFK
XP_006587436.1/1-240 --P----NGV-VLSAATTIW--------------------------LPI-PPQTVFNFFK
XP_003549189.1/1-236 --P----NGV-VLSAATTIW--------------------------LPI-PPQTVFNFFK
XP_020221986.1/1-232 --P----NGV-VLSAATTIW--------------------------LPI-PPQTVFNFFK
XP_007152598.1/1-234 --P----NGV-VLSAATTIW--------------------------LPI-PPQTVFNFFK
XP_017437542.1/1-234 --P----NGV-VLSAATTIW--------------------------LPI-PPQTVFNFFK
XP_014505752.1/1-234 --P----NGV-VLSAATTIW--------------------------LPI-PPQTVFNFFK
XP_015881991.1/1-234 --P----NGM-ILSAATTIW--------------------------LPI-PPQDVFNFFK
XP_004299483.1/1-232 --P----NGV-VLSAATTIW--------------------------LPL-SPQTVFNFFK
XP_024174647.1/1-232 --P----NGV-VLSAATTIW--------------------------LPL-SPQTVFNFFK
XP_008375041.1/1-227 --P----NGV-VLSAATTIW--------------------------LPL-SPQNVFNFFK
XP_017188172.1/1-199 --P----NGV-VLSAATTIW--------------------------LPL-SPQNVFNFFK
XP_009355964.1/1-227 --P----NGV-VLSAATTIW--------------------------LPL-SPQNVFNFFK
XP_009355965.1/1-199 --P----NGV-VLSAATTIW--------------------------LPL-SPQNVFNFFK
XP_023523434.1/1-228 --R----NGV-ALSAATTIW--------------------------LPV-PPQTIFNFFK
XP_022949138.1/1-227 --R----NGV-VLSAATTIW--------------------------LPV-PPQTIFNFFK
XP_022998050.1/1-227 --R----NGV-VLSAATTIW--------------------------LPV-PPQTIFNFFK
XP_002271012.1/1-231 --P----NGV-VLSAATTIW--------------------------LPV-SPQNVFNFFR
XP_010663934.1/1-231 --P----NGV-VLSAATTIW--------------------------LPV-SPQNVFNFFR
XP_010099679.1/1-237 --P----NGV-VLSAATTIW--------------------------LPI-SPQDVFNFFK
XP_018823334.1/1-236 --P----NGV-ILSAATTIW--------------------------LPV-SPQNVFNFFK
XP_023001145.1/1-228 --P----NGV-VLSAATTIW--------------------------LSV-SPQTIFNFFK
XP_023519889.1/1-228 --P----NGV-VLSAATTIW--------------------------LSV-SPQTIFNFFK
XP_022924191.1/1-228 --P----NGV-VLSAATTIW--------------------------LSV-SPQTIFNFFK
XP_018849090.1/1-235 --P----NGV-VLSAATTIW--------------------------LPI-SPQNVFNFFK
XP_008240378.1/1-228 --P----NGV-VLSAATTIW--------------------------LPV-SPQNVFNFFK
XP_007210460.2/1-228 --P----NGV-VLSAATTIW--------------------------LPV-SPQNVFNFFK
XP_021801797.1/1-228 --P----NGV-VLSAATTIW--------------------------LPV-SPQNVFNFFK
XP_022136988.1/1-228 --S----NGV-VLSAATTIW--------------------------LPV-SPQTIFNFFK
XP_016898817.1/1-228 --P----NGV-VLSAATTIW--------------------------LPV-SPQTIFNFFK
XP_004137568.1/1-228 --P----NGV-VLSAATTIW--------------------------LPV-SPQTIFNFFK
XP_015642098.1/1-238 --P----NGV-VLSAATSIW--------------------------LPV-PCDHVFAFVR
XP_020186741.1/1-220 --P----SGV-VLSAATSIW--------------------------LPV-SCDRVFAFVS
XP_025828617.1/1-229 --P----SGV-VLSAATSIW--------------------------LPV-PCDRVFAFIC
XP_004954072.1/1-226 --P----SGV-VLSAATSIW--------------------------LPV-PCDRVFAFVR
XP_002452898.1/1-231 --P----SGV-VLSAATSIW--------------------------LPV-PCDRVFAFVR
NP_001142912.2/1-230 --P----SGV-VLSAATSIW--------------------------LPV-PCDRVFAFVR
XP_020089796.1/1-231 --P----SGV-VLSAATSLW--------------------------LPM-SCERVFSFFR
XP_020089795.1/1-241 --P----SGV-VLSAATSLW--------------------------LPM-SCERVFSFFR
XP_019708739.1/1-228 --P----NGV-VLSAATSIW--------------------------LPM-SSERVFSFFK
XP_008789232.1/1-228 --P----NGV-VLSAATSIW--------------------------LPM-SSERVFSFFK
XP_008789231.1/1-228 --P----NGV-VLSAATSIW--------------------------LPM-SSERVFSFFK
XP_008789233.1/1-200 --P----NGV-VLSAATSIW--------------------------LPM-SSERVFSFFK
XP_019704584.1/1-228 --P----NGV-VLSAATSIW--------------------------LPI-SSERVFSFLK
XP_010917295.1/1-228 --P----NGV-VLSAATSIW--------------------------LPI-SSERVFSFLK
XP_008806630.1/1-228 --P----NGV-VLSAATSIW--------------------------LPI-SSERVFSFFK
XP_009388746.1/1-226 --P----DGI-VLNAATSMW--------------------------LPI-SSEKVFGFFK
XP_018675467.1/1-228 --P----NGV-VLSAATSIW--------------------------LPI-SAERIFSFFK
XP_018675466.1/1-228 --P----NGV-VLSAATSIW--------------------------LPI-SAERIFSFFK
XP_009409108.1/1-228 --P----NGV-VLSAATSIW--------------------------LPI-STERVFSFFK
XP_024625408.1/1-220 --P----NGE-VLSASTIIW--------------------------LPL-PPQTVFNFLN
XP_010550261.1/1-219 --P----SGV-IVSAASSLW--------------------------LPL-PPSKVFNFFR
XP_018816719.1/1-228 --P----GGI-IVSAATSLW--------------------------LPL-PPQNVFDFIR
XP_008365756.2/1-218 --P----HGT-IXSVATSXW--------------------------LPL-STQMIFNFFK
XP_008381522.1/1-218 --P----HGT-IXSVATSXW--------------------------LPL-STQMIFNFFK
XP_018506060.1/1-218 --P----HGT-IVSIATSLW--------------------------LPL-SSQTIFNFFR
XP_008362571.1/1-218 --P----HGT-IVSVATSLW--------------------------LPL-SSQTIFNFLR
XP_023870350.1/1-229 --P----CGM-IVSAATSLW--------------------------LPL-PPHHVFSFFR
XP_016649213.1/1-222 --P----NGM-VVSVATSLW--------------------------LPL-PSHNVFNFFR
XP_020415996.1/1-222 --P----NGM-VVSVATSLW--------------------------LPL-PSQNVFNFFR
XP_021823813.1/1-222 --P----NGM-VVSVATSLW--------------------------LPL-PSQNVFNFFR
XP_009367126.1/1-222 --P----HGM-IVSVATSLW--------------------------LPL-PSETVFNFFR
XP_008348615.1/1-222 --P----HGM-IVSVATSLW--------------------------LPL-PSETVXNFFR
XP_017179578.1/1-222 --P----HGM-IVSIATSLW--------------------------LPL-PSQTVFSFFR
XP_009370612.1/1-224 --P----HGM-IVSIATSLW--------------------------LPL-PSQTIFSFFR
XP_012845089.1/1-228 --P----DGL-IVSAATSLW--------------------------LPF-SIENLFKFFR
XP_012845088.1/1-228 --P----DGL-IVSAATSLW--------------------------LPF-SIENLFKFFR
XP_012845087.1/1-228 --P----DGL-IVSAATSLW--------------------------LPF-SIENLFKFFR
XP_011085930.1/1-227 --P----DGF-IVSAATSLW--------------------------LPV-SNENLFNFFK
XP_020551334.1/1-222 --P----DGF-IVSAATSLW--------------------------LPV-SNENLFNFFK
XP_021297998.1/1-224 --P----RGM-IVSAATSLW--------------------------LPL-PCQSVFNLLN
XP_017974480.1/1-224 --P----RGM-IVSAATSLW--------------------------LPL-PCQSVFNLLN
XP_024448785.1/1-229 --P----IGM-VVSAATSLW--------------------------LPL-SPQNVFNFFK
XP_024455385.1/1-229 --P----IGM-VVSAATSLW--------------------------LPL-SPQNVFNFFK
XP_011005937.1/1-269 --P----VGM-VVSAATSLW--------------------------LPL-SPQIVFNFFK
XP_011038413.1/1-229 --P----IGT-VVSAATSFW--------------------------LPL-SPQNVFNFFK
XP_018731153.1/1-226 --P----SGT-IVSAATSLW--------------------------LPV-APQAVFNFFR
XP_024190515.1/1-228 --P----SGM-VVVAATSLW--------------------------LPL-HSQDVFNFFT
XP_012074722.2/1-231 --H----FGM-IISAATSLR--------------------------LPL-PPETVFNFFK
XP_010089514.2/1-229 --P----SGI-IVGAATSLW--------------------------LPL-PPQAVFDFFR
XP_024949330.1/1-235 --P----SGI-VVSAATSLW--------------------------LPL-PSHNVFNFFK
XP_006421085.1/1-235 --P----SGI-VVSAATSLW--------------------------LPL-PSHNVFNFFK
XP_002268272.1/1-229 --P----GGL-IVSAATSLW--------------------------LPM-QCQTIFDFIR
XP_004140733.2/1-228 --P----SGT-VVSAATSLW--------------------------LPL-QPETIFNFFR
XP_008457114.1/1-228 --P----SGT-VVSAATSLW--------------------------LPL-QPETIFNFFR
XP_008457113.1/1-229 --P----SGT-VVSAATSLW--------------------------LPL-QPETIFNFFR
XP_011651208.1/1-229 --P----SGT-VVSAATSLW--------------------------LPL-QPETIFNFFR
XP_022143367.1/1-228 --P----SGT-IVSAATSLW--------------------------LPL-QPESIFNFFR
XP_022935490.1/1-228 --P----SGT-IVSAATSLW--------------------------LPL-QPESIFNFFK
XP_022984101.1/1-228 --P----SGT-IVSAATSLW--------------------------LPL-QPESIFNFFK
XP_022935488.1/1-229 --P----SGT-IVSAATSLW--------------------------LPL-QPESIFNFFK
XP_022984098.1/1-229 --P----SGT-IVSAATSLW--------------------------LPL-QPESIFNFFK
XP_022984102.1/1-229 --P----SGT-IVSAATSLW--------------------------LPL-QPESIFNFFK
XP_015579295.1/1-234 --P----GGL-IVSAATSLW--------------------------LPL-PPQNVFSFFK
XP_002526595.2/1-234 --P----GGL-IVSAATSLW--------------------------LPL-PPQNVFSFFK
XP_015579294.1/1-234 --P----GGL-IVSAATSLW--------------------------LPL-PPQNVFSFFK
XP_021643520.1/1-232 --P----GGM-IVSAATSLW--------------------------LPL-LPQNVFNFFK
XP_021605433.1/1-231 --P----SGM-IVSAATSLW--------------------------LPL-LPQDVFSFFR
XP_023739098.1/1-228 --P----NTT-VLSAAATVW--------------------------LPH-SPQFLFDFLR
XP_024966239.1/1-227 --A----NTM-VLAVATTVW--------------------------MPF-SPQFVFDFLR
XP_025813558.1/1-232 --P----NGV-VLTAATSIW--------------------------LPV-PANHAFAFLR
XP_020150479.1/1-234 --P----NGV-VLGAATSVW--------------------------LPV-PADHVFGFLR
XP_003564122.2/1-224 --P----NGV-VLSAATSVW--------------------------LPV-PCDHVFVFLR
XP_006585049.2/1-225 --P----QGL-ILSAATSFL--------------------------LPH-SPENVFDFLI
XP_007143783.1/1-226 --P----KGM-ILSAATSFW--------------------------LPL-SPQHVFDYLI
XP_007158859.1/1-226 --P----KGM-ILSAATSFW--------------------------LPL-SPQHVFDYLI
XP_020230815.1/1-227 --P----NSMITITAATSFW--------------------------LPL-PSQNLYDFFR
XP_006586851.1/1-227 --S----NPNIIITATTSFR--------------------------LPL-PSQNVFDFFR
XP_021888058.1/1-234 --P----SGT-IVNAASSLW--------------------------LPL-PPLHVFNFLK
XP_020886111.1/1-226 --P----PGI-VVSASSSLT--------------------------IPL-TPLQVFVFLQ
XP_020886110.1/1-228 --P----PGI-VVSASSSLT--------------------------IPL-TPLQVFVFLQ
XP_011469296.1/1-224 --P----SGM-VVVAATSLW--------------------------LPL-HSQDVFNFFT
XP_009367128.2/1-218 --P----HGT-IVSVATSLW--------------------------LPL-STQTILNFFK
XP_008348673.1/1-198 --P----HGT-IVSVATSPW--------------------------LPL-STHTIFNFFK
XP_012832506.1/1-227 --P----DGF-IVSAATSLW--------------------------LPF-SKEHLFNFFQ
XP_012832508.1/1-227 --P----DGF-IVSAATSLW--------------------------LPF-SKEHLFNFFQ
XP_012832516.1/1-227 --P----DGF-IVSAATSLW--------------------------LPF-SKEHLFNFFH
XP_012832518.1/1-227 --P----DGF-IVSAATSLW--------------------------LPF-SKEHLFNFFH
XP_012835569.1/1-227 --P----DGF-IVSVATSLW--------------------------LPF-SKEHLFNFFH
XP_012835571.1/1-226 --P----DGF-IVSVATSLW--------------------------LPF-SKEHLFNFFH
XP_019172066.1/1-226 --A----DAL-IVCAASSLW--------------------------LPL-PLDHLFNFFR
XP_009631364.2/1-226 --P----KGL-IVTAATSLS--------------------------LPL-SFDTVFNFFK
XP_016435660.1/1-229 --P----KGL-IVTAATSLW--------------------------LPL-SFDTVFNFFK
XP_009771801.1/1-226 --P----KGL-IVTAATSLW--------------------------LPL-SFDTVFNFFK
XP_016493115.1/1-226 --P----KGL-IVTAATSLW--------------------------LPL-SFDTVFNFFK
XP_019248352.1/1-226 --P----KGL-IVTAATSIW--------------------------LPL-SFDTVFNFFK
XP_025691463.1/1-225 --P----KGL-IVTASTSLW--------------------------LPL-LPQTLFNFFK
XP_020995109.1/1-225 --P----KGL-IVTASTSLW--------------------------LPL-LPQTLFNFFK
XP_016190415.2/1-225 --P----KGL-IVTASISLW--------------------------LPL-LPQALFNFFK
XP_004486568.1/1-232 --P----NGM-VVAAATTLW--------------------------LPL-SADKVFEFLR
XP_024632687.1/1-226 --P----NGT-IVTAATTLW--------------------------LPL-PALKVFEFLK
XP_020977993.1/1-241 --P----NGT-IVIAATSLW--------------------------LPL-HYHTVFEFLT
XP_025647253.1/1-241 --P----NGT-IVIAATSLW--------------------------LPL-HYHTVFEFLT
XP_020997095.1/1-240 --P----NGT-IVIAATSLW--------------------------LPL-HYHTVFEFLT
XP_025692389.1/1-228 --P----NGT-IVIAATSLW--------------------------LPL-HYHTVFEFLT
XP_019418389.1/1-226 --P----NGI-IVTAISSLW--------------------------LPL-PYQQVYNFFT
XP_007147460.1/1-227 --P----SGM-IVAAATSIW--------------------------LPL-HYMKIFEFFS
XP_014517364.1/1-227 --P----SGM-IVAAATSIW--------------------------LPI-HYMKVFEFFL
XP_017435438.1/1-226 --P----SGM-IVAAATSIW--------------------------LPL-HYMKVFEFFL
XP_017435439.1/1-217 --P----SGM-IVAAATSIW--------------------------LPL-HYMKVFEFFL
XP_025981664.1/1-227 --P----KGV-IVVAATSIW--------------------------LPL-HYMKVFEFLT
XP_006586853.1/1-227 --P----KGM-IVVAATSIW--------------------------LPL-HYMKVFEFFT
XP_020230831.1/1-226 --P----NGM-IVVAATSLW--------------------------LPL-HYMKVFEFFT
XP_004489663.1/1-217 --P----NGK-GVVAIATLW--------------------------TPL-PSDKVFEFLI
XP_018489833.1/1-227 --P----PGL-VASAASCLS--------------------------ISR-TPLQVFNCLR
XP_013584487.1/1-219 --L----PGL-VVSAASCLS--------------------------IPL-TPLQVFNCLR
XP_009130351.1/1-219 --L----PGL-VVTAASCLS--------------------------IPL-TPLQVFNFLR
XP_013747906.2/1-219 --L----PGL-VVTAASCLS--------------------------IPL-TPLQVFNCLR
XP_013747661.1/1-219 --L----PGL-VVTAASCLS--------------------------IPL-TPLQVFNCLR
XP_013619678.1/1-219 --L----PGL-VVSAASCLS--------------------------IPL-TPLQVFNSLR
XP_022551084.1/1-219 --L----PGL-VVSAASCLS--------------------------IPL-TPLQVFNSLR
XP_009126392.1/1-219 --I----PGL-VASAASCLS--------------------------IPL-TPLQVFNSLR
XP_018437612.1/1-222 --P----SGL-VVSAASSFS--------------------------VPV-TPLQVYNTLR
XP_013591191.1/1-225 --P----SGL-VVSAASSFS--------------------------VPI-TPLQVFDSLL
XP_022567005.1/1-225 --P----SGL-VVSAASSFS--------------------------VPI-TPLQVFDSLL
XP_022547796.1/1-225 --P----SGL-VVSAASSFS--------------------------VPI-TPLQVFDSLL
XP_009119205.1/1-225 --P----SGL-VVSAASSFS--------------------------VPI-TPLQVFDSLL
XP_009119199.1/1-225 --P----SGL-VVSAASSFS--------------------------VPI-TPLQVFDSLL
XP_006408322.1/1-232 --P----TGL-VVSAASCLS--------------------------IPL-SPLQVFNCLR
XP_023642295.1/1-229 --P----PGL-IVSAASSLS--------------------------IPL-TPSQVFDFLL
XP_023642294.1/1-229 --P----PGL-IVSAASSLS--------------------------IPL-TPSQVFDFLL
NP_186976.2/1-228    --P----PGI-VVSASSSLA--------------------------IPL-TPLQVFAFLQ
XP_010507998.1/1-229 --P----RGL-IVSAASSFP--------------------------IPL-PPVQVFDNLR
XP_010485661.1/1-229 --P----PGL-IVSAASSLA--------------------------ISL-PPVQVFDLLR
XP_010450732.1/1-230 --P----PGL-IVSAASSLR--------------------------IPL-PPVQVFDILR
XP_010463776.1/1-230 --P----PGL-IVSAASSLR--------------------------IPL-PPVQVFDILR
XP_010463774.1/1-230 --P----PGL-IVSAASSLR--------------------------IPL-PPVQVFDILR
XP_019092960.1/1-229 --P----PGL-IVSAASSLR--------------------------IPL-PPVQVFDILR
XP_022551077.1/1-228 --P----TGL-IVCAASSLC--------------------------LPL-SPLEVYNFLT
XP_013619236.1/1-228 --P----TGL-IVCAASSLC--------------------------LPL-SPLEVYNFLT
XP_009126177.1/1-226 --P----TGL-IVCAASSIC--------------------------LPL-SPLEVYNFLT
XP_013652421.1/1-226 --P----TGL-IVCAASSIC--------------------------LPL-SPLEVYNFLT
XP_013652420.1/1-226 --P----TGL-IVCAASSIC--------------------------LPL-SPLEVYNFLT
XP_006400265.1/1-228 --P----PGL-IVCAGSSLC--------------------------LPL-SPVQVYNFLK
XP_013590801.1/1-226 --P----PGL-IVCACSSLC--------------------------LPL-APLQVYNFLK
XP_013651910.1/1-222 --P----VGL-IVCACSSLC--------------------------LPL-APLQVYNFLK
XP_009103958.1/1-222 --P----VGL-IVCACSSLC--------------------------LPL-APLQVYNFLK
XP_018444485.1/1-226 --P----AGL-IVCAGLSLS--------------------------LPL-APLQVYNFLK
XP_009121397.1/1-226 --P----AGL-IVCAGLSLC--------------------------LPL-APLQVYSFLK
XP_013659428.1/1-226 --P----AGL-IVCAGLSLC--------------------------LPL-APLQVYSFLK
XP_022565333.1/1-226 --P----AGL-IVCAGLSLC--------------------------LPL-APLQVYNFLK
XP_013610327.1/1-226 --P----AGL-IVCAGLSLC--------------------------LPL-APLQVYNFLK
XP_006286655.1/1-225 --P----PGL-IVCAGSSLS--------------------------LPL-PPLQVYDFLR
XP_010492677.1/1-224 --P----PGL-ILCAGSSLC--------------------------LPL-PPPQVYDFLR
XP_010420445.1/1-224 --P----PGL-ILCAGSSLC--------------------------LPL-PPPQVYDFLR
XP_010453918.1/1-224 --P----PGL-ILCAGSSLC--------------------------LPL-PPPQVYDFLR
XP_006303739.1/1-224 --P----VGL-IVCASSSLS--------------------------LPV-PPVQVYNLLK
XP_020876074.1/1-222 --P----SGL-IVCAGSSLS--------------------------LPL-PPIHVYDFLK
NP_197234.1/1-222    --P----PGL-IVCAGSSLS--------------------------LPL-PPVQVYDFLK
NP_174724.1/1-221    --P----PGL-IVCAGSSLS--------------------------LPL-PPLQVYDFLR
XP_020869876.1/1-222 --P----TGL-IVCAGSSLS--------------------------LPL-PPLQVYDFLR
XP_019091098.1/1-222 --P----TGL-IVCASSSLY--------------------------LPL-TPLRVYDFLS
XP_010478910.1/1-223 --P----TGL-IVCASSSLY--------------------------LPL-TPLRVYDFLS
XP_010501781.1/1-221 --P----TGL-IVCASSSLY--------------------------LPL-TPLRVYDFLS
XP_015089790.1/1-222 --S----KGF-IATAATSLW--------------------------FPF-AFKTIFNFLK
XP_015089784.1/1-222 --S----KGF-IATAATSLW--------------------------FPF-AFKTIFNFLK
XP_016549855.1/1-224 --P----KGW-IVTAATSLC--------------------------LPL-SFQTVFDFFK
XP_015057403.1/1-221 --T----KGF-IATAASSLW--------------------------IPL-SFQDVFNFFK
XP_019066399.1/1-221 --T----KGF-IATASSSLW--------------------------IPL-SFQDVFNFFK
XP_015166059.1/1-216 --P----KGF-IATAATSLW--------------------------LPL-SFQDVFNFFK
XP_019071012.2/1-216 --P----KGF-IATAATSLW--------------------------LPL-SFQDVFNFFK
XP_015086709.1/1-216 --P----KGF-IATAATSLW--------------------------LPL-SFQDVFNFFK
XP_015087432.1/1-219 --P----KGF-IAIATASLW--------------------------LPL-SFQDVFNFFK
XP_004247342.2/1-219 --P----KGF-IAIATASLW--------------------------LPL-SFQDVFDFFK
XP_025888717.1/1-219 --P----KGF-IAIATASLW--------------------------LPL-SFQDVFDFFK
XP_015087599.1/1-221 --P----EGF-IAIATTSLW--------------------------LPR-SLQDVFNFFE
XP_019071156.1/1-221 --P----KGF-IAIATTSLW--------------------------LPR-SFQDVFNFFE
XP_019070762.2/1-220 --P----KGF-IAIATTSLW--------------------------LPL-SFQDVFSFFN
XP_015087071.1/1-220 --P----KGV-IAIATTSLW--------------------------LPL-SFQDVFSFFN
XP_025888845.1/1-217 --P----KGF-IAIATTSVW--------------------------LPL-SFQDVFSFFN
XP_015170367.1/1-232 --K----KGF-IVTASTSIW--------------------------LPH-SFQTVFNFLK
XP_025886268.1/1-232 --Q----KGF-IITAATSLW--------------------------LPL-SCRNVFNFLK
XP_015072491.1/1-224 --Q----KGF-IITAATSLW--------------------------LPL-SCRNVFNFLK
XP_010327374.2/1-219 --S----KGF-IVTAATSLW--------------------------VPL-SFETIFDLLN
XP_015089749.1/1-219 --S----KGF-IATAATSLW--------------------------VPL-SFETIFDLLN
XP_015170971.1/1-209 --P----QGF-IASAATSLW--------------------------LPL-SFETIFNFLK
XP_025887726.1/1-223 --Q----QGF-IATAATSLW--------------------------LPL-SFETIFNFLK
XP_006586358.1/1-227 --P----KGT-IIGAAICFR--------------------------VPL-SPQIVFDNLI
XP_014617351.1/1-228 --N----PNI-IITATTSFR--------------------------LPL-PSQNVFDFFR
XP_007147461.1/1-227 --N----GMI-IFIATTSFR--------------------------LPL-PSENVFDFFR
XP_017435408.1/1-226 --N----AMI-IFIATTSFR--------------------------LPV-SSEYVFDFFR
XP_017215828.1/1-223 --P----LGV-VLCAVLSVW--------------------------LPV-SRQNLFNFLR
XP_012856110.1/1-229 --P----IGT-ILSAVSSVW--------------------------LPL-SHHALFDFLR
XP_023738891.1/1-226 --P----LGV-ILCAVSSIW--------------------------LPV-SHTVLFDFLR
XP_022017964.1/1-227 --P----LGV-ILCAVSSIW--------------------------LPV-SHAVLFDFLR
XP_022154996.1/1-226 --P----LGL-ILCAVASVW--------------------------LPV-SPKVLFEFLI
XP_008462169.1/1-225 --P----TGL-ILCAVASVW--------------------------LPV-SPKILFEFLI
XP_004141841.1/1-226 --P----TGL-ILCAVASIW--------------------------LPV-SPKLLFEFLI
XP_020098514.1/1-228 --P----QGL-VACVVLSTW--------------------------LPL-SSIALFDFLR
XP_020098513.1/1-228 --P----QGL-VACVVLSTW--------------------------LPL-SSIALFDFLR
XP_020263950.1/1-229 --P----LGL-IVSAVSSTW--------------------------LPV-ASPVLFEFLR
XP_020263948.1/1-255 --P----LGL-IVSAVSSTW--------------------------LPV-ASPVLFEFLR
XP_009415234.2/1-232 --P----LGL-IICSVLSTW--------------------------LPV-PAMSLFNFLR
XP_008789333.1/1-230 --P----TGV-IVCAVSSTW--------------------------LPV-PSTALFDYLR
XP_010920420.1/1-235 --P----TGV-IVCAVSSTW--------------------------LPV-PSMALFDYLR
XP_010920411.1/1-235 --P----TGV-IVCAVSSTW--------------------------LPV-PSMALFDYLR
XP_010920404.1/1-235 --P----TGV-IVCAVSSTW--------------------------LPV-PSMALFDYLR
XP_010521515.1/1-219 --P----LGV-IVCAASSLW--------------------------LPV-PPTVLFDFLR
XP_013652056.1/1-227 --P----TGV-IVCASSSLW--------------------------LPV-SPTLLFDFFR
XP_009104428.1/1-227 --P----TGV-IVCASSSLW--------------------------LPV-SPTLLFDFFR
XP_018445476.1/1-226 --P----TGV-IVCASSSLW--------------------------LPV-SPTLLFDFFR
XP_013590784.1/1-226 --P----TGV-IVCASSSLW--------------------------LPV-SPTLLFDFFR
XP_013680822.1/1-226 --P----TGV-IVCASSSLW--------------------------LPV-SPTLLFDFFR
XP_006389720.2/1-226 --P----TGV-IVCASSSLW--------------------------LPV-SPTLLFDFFR
XP_023642973.1/1-226 --P----TGV-IVCASSSLW--------------------------LPV-SPTLLFDYFR
XP_020891640.1/1-226 --P----TGV-IVCASSSLW--------------------------LPV-SPTLLFDFFR
NP_001320267.1/1-226 --P----TGV-IVCASSSLW--------------------------LPV-SPALLFDFFR
NP_565223.1/1-226    --P----TGV-IVCASSSLW--------------------------LPV-SPALLFDFFR
NP_001185443.1/1-226 --P----TGV-IVCASSSLW--------------------------LPV-SPALLFDFFR
XP_010473021.1/1-226 --P----TGV-IVCASSSLW--------------------------LPV-SPTLLFDFFR
XP_010430051.1/1-226 --P----TGV-IVCASSSLW--------------------------LPV-SPTLLFDFFR
XP_025617667.1/1-241 --P----LGV-ILSAVSSVW--------------------------LPL-PPNVLFHFLR
XP_015938429.1/1-242 --P----LGV-ILSAVSSVW--------------------------LPL-PPNVLFHFLR
XP_016176999.1/1-241 --P----LGV-ILSAVSSVW--------------------------LPL-PPNVLFHFLR
XP_021862846.1/1-225 --P----IGV-ILCAVSSVW--------------------------LPV-SPTVLFDFLR
XP_022979290.1/1-231 --P----IGA-ILCSVSSLW--------------------------LPV-SPHLLFHFLR
XP_022955725.1/1-233 --P----IGA-ILCAVSSLW--------------------------LPV-SPDLLFHFLL
XP_022998610.1/1-231 --P----IGV-ILCAVFSLW--------------------------LPV-PPHTLFHFLR
XP_022998611.1/1-227 --P----IGV-ILCAVFSLW--------------------------LPV-PPHTLFHFLR
XP_023523626.1/1-229 --P----IGV-ILCAVFSLW--------------------------LPV-PPHTLFHFLR
XP_022948296.1/1-231 --P----IGV-ILCAVFSLW--------------------------LPV-PPHTLFHFLR
XP_023523628.1/1-225 --P----IGV-ILCAVFSLW--------------------------LPV-PPHTLFHFLR
XP_022948295.1/1-227 --P----IGV-ILCAVFSLW--------------------------LPV-PPHTLFHFLR
XP_010249693.1/1-228 --P----LGV-ILCAVSSVC--------------------------LPV-SPHILFDFLR
XP_010254081.1/1-230 --P----LGV-ILCAVSSVF--------------------------LPV-SPHVLFDFLR
XP_003529763.1/1-227 --P----LGV-ILCAVSSVW--------------------------LPV-SPNVLFDFLR
XP_003533048.1/1-226 --P----LGV-ILSAVSSVW--------------------------LPV-STNVLFDFLR
XP_014634625.1/1-226 --P----LGV-ILSAVSSVW--------------------------LPV-STNVLFDFLR
XP_007150635.1/1-226 --P----LGL-ILCAVSSVW--------------------------LPV-SQNVLFDFLR
XP_014498860.1/1-226 --P----LGL-ILCAVCSVW--------------------------LPV-SQNVLFDFLR
XP_017425661.1/1-226 --P----LGL-ILCAVCSVW--------------------------LPV-SQNVLFDFLR
XP_020207725.1/1-226 --P----LGL-ILCAVCSVW--------------------------LPV-APNVLFDFLR
XP_003546423.2/1-226 --P----LGL-ILCAVCSVW--------------------------LPV-SPNVLFDFLR
XP_003543638.1/1-226 --P----LGL-ILCAVCSVW--------------------------LPV-SPNVLFDFLR
XP_019457980.1/1-226 --P----VGV-ILCAVSSVW--------------------------LSV-SPNVLFDFLR
XP_003597736.1/1-226 --P----LGL-IVCAVSSIW--------------------------LPI-SPNVLFDFLR
XP_004486751.1/1-226 --P----LGL-ILCAVSSVW--------------------------LPI-SPNVLFDFLR
XP_016169615.1/1-226 --P----LGL-ILCAVSSVW--------------------------LPV-SPNVLFDFLR
XP_025611006.1/1-226 --P----LGL-ILCAVSSVW--------------------------LPV-SPNVLFDFLR
XP_020982478.1/1-227 --P----LGL-ILCAVSSVW--------------------------LPV-SPNVLFDFLR
XP_021892835.1/1-225 --P----VGV-IVCAVSSVW--------------------------LPV-CPHLLFDFLR
XP_012442963.1/1-227 --P----HGV-IVCAVSSVW--------------------------LPV-SPTLLFDFLR
NP_001314068.1/1-227 --P----HGV-IVCAVSSVW--------------------------LPV-SPTLLFDFLR
XP_016684379.1/1-227 --P----HGV-IVCAVSSVW--------------------------LPV-SPTLLFDFLR
XP_016730082.1/1-227 --P----HGV-IVCAVSSVW--------------------------LPV-SPTLLFDFLR
NP_001316938.1/1-227 --P----HGV-IVCAVSSVC--------------------------LPV-SPTLLFDFLR
XP_017606636.1/1-227 --P----HGV-IVCAVSSVC--------------------------LPV-SPTLLFDFLR
XP_022714992.1/1-229 --P----LGV-IVCAVSSVW--------------------------LPV-SPNLLFDFLR
XP_022721436.1/1-227 --P----LGV-IVCAVSSVW--------------------------LPV-SPNLLFDFLR
XP_022721435.1/1-227 --P----LGV-IVCAVSSVW--------------------------LPV-SPNLLFDFLR
XP_007024189.1/1-227 --P----LGV-IVCAVSSVW--------------------------LPV-SPNALFDFLR
XP_021298731.1/1-227 --P----LGV-IVCAVSSVW--------------------------LPV-SPNVLFDFLR
XP_004302894.1/1-228 --P----IGV-ILCAVSSVW--------------------------LPV-SPFTLFDFLR
XP_011466687.1/1-235 --P----IGV-ILCAVSSVW--------------------------LPV-SPFTLFDFLR
XP_024180978.1/1-228 --P----LGV-ILCAVSSVW--------------------------LPV-SPYALFDFLR
XP_024180977.1/1-235 --P----LGV-ILCAVSSVW--------------------------LPV-SPYALFDFLR
XP_009353605.1/1-226 --P----LGV-ILCAVSSVW--------------------------LPV-SPHVLFDFLR
XP_008380909.1/1-226 --P----LGV-ILCAVSSVW--------------------------LPI-SPHVLFDFLR
NP_001280927.1/1-227 --P----LGV-ILCAVSSVW--------------------------LPV-SPYLLFDFLR
XP_009338062.1/1-226 --P----LGV-ILCAVSSVW--------------------------LPV-SPYLLFDFLR
XP_009338046.1/1-226 --P----LGV-ILCAVSSVW--------------------------LPV-SPYLLFDFLR
XP_021829136.1/1-226 --P----LGV-ILCAVSSVW--------------------------LPV-CPYVLFDFLR
XP_008228560.1/1-226 --P----LGV-ILCAVSSVW--------------------------LPV-CPYVLFDFLR
XP_007217160.1/1-226 --P----LGV-ILCAVSSVW--------------------------LPV-CPYVLFDFLR
XP_022891611.1/1-228 --P----PGV-ILCAVSSVW--------------------------LPV-SHHILFDFLR
XP_002304207.2/1-228 --P----VGL-ILCAVSSVW--------------------------LPV-PPHILFDFLR
XP_011010522.1/1-228 --P----VGL-ILCAVSSVW--------------------------LPV-PPHILFDFLR
XP_011009828.1/1-227 --P----IGV-ILCAVSSVW--------------------------LPV-VPHILFDFLR
XP_024462179.1/1-228 --P----VGV-ILCAVSSVW--------------------------LPV-VPHILFDFLR
XP_018837483.1/1-226 --P----HGV-ILCAVSSVW--------------------------LPV-SPQVLFDFLR
XP_018856342.1/1-226 --P----LGL-ILCAVSSIW--------------------------LPV-SPHVLFDFLR
XP_018856994.1/1-226 --P----LGL-ILCAVSSIW--------------------------LPV-SPHVLFDFLR
XP_018858360.1/1-226 --P----LGL-ILCAVSSIW--------------------------LPV-SPHVLFDFLR
XP_015867693.1/1-226 --P----LGL-ILCAVSSVW--------------------------LPI-SPHVLFDFLR
XP_010062490.1/1-226 --P----LGV-ILCAVSSVL--------------------------LPV-SPHVLFDFLR
XP_010098082.1/1-228 --P----LGV-ILCAVSSVW--------------------------LPV-SPHALFDFLR
XP_002284502.1/1-228 --P----QGV-ILCAVSSVW--------------------------LPV-APHVIFDFLR
XP_023875609.1/1-226 --P----LGV-ILCAVSSVW--------------------------LPV-SPHVLFDFLR
XP_002516023.1/1-226 --P----LGV-ILCAVSSVW--------------------------LPV-SPHVLFDYLR
XP_021652755.1/1-228 --P----IGV-ILCVVSSAW--------------------------LPV-SPPVLFDFLR
XP_021654904.1/1-226 --P----TGV-ILCAVSSAW--------------------------LPV-SPHVLFDFLR
XP_021611342.1/1-229 --P----TGV-ILCAVSSVW--------------------------LPV-SPHVLFDFLR
XP_012073376.1/1-226 --P----LGV-ILCAVSSVW--------------------------LPV-SPHVLFDFLR
XP_006465622.1/1-226 --P----LGV-ILCAVSSVW--------------------------LPV-SPNVLFDFLR
XP_006426950.1/1-226 --P----LGV-ILCAVSSVW--------------------------LPV-SPNVLFDFLR
XP_009392419.1/1-229 --P----PGL-IICAVLSTW--------------------------LPV-PTMTLFDFLR
XP_020518333.1/1-227 --P----QGL-ILSAVASVW--------------------------LPV-SPTVLFDFLR
XP_018629167.1/1-227 --P----FGL-ILCATSSVW--------------------------LPF-SRNVLFDFLR
XP_009611250.1/1-227 --P----FGL-ILCATSSVW--------------------------LPF-SRNVLFDFLR
XP_009611249.1/1-227 --P----FGL-ILCATSSVW--------------------------LPF-SRNVLFDFLR
XP_009611248.1/1-227 --P----FGL-ILCATSSVW--------------------------LPF-SRNVLFDFLR
XP_016479156.1/1-227 --P----FGL-ILCATSSVW--------------------------LPV-SRNVLFDFLR
XP_016479155.1/1-227 --P----FGL-ILCATSSVW--------------------------LPV-SRNVLFDFLR
XP_016479154.1/1-227 --P----FGL-ILCATSSVW--------------------------LPV-SRNVLFDFLR
XP_009778213.1/1-229 --P----QGV-ILCVVSSIW--------------------------LPV-SRNVLFDFLK
XP_016501674.1/1-229 --P----QGV-ILCVVSSIW--------------------------LPV-SRNVLFDFLK
XP_019224885.1/1-229 --P----HGV-ILCAVSSIW--------------------------LPV-SRNVLFDFLK
XP_019224886.1/1-229 --P----HGV-ILCAVSSIW--------------------------LPV-SRNVLFDFLK
XP_009592772.1/1-229 --P----QGV-ILCAVSSIW--------------------------LPV-SRNLLFDFLK
XP_016496403.1/1-229 --P----QGV-ILCAVSSIW--------------------------LPV-SRNLLFDFLK
XP_016562690.1/1-228 --P----LGL-ILCAVSSIW--------------------------LPV-SRNVLFDFLK
XP_006343080.1/1-229 --P----QGL-ILCAVSSIW--------------------------LPV-SRNVLFDFLK
XP_004235676.1/1-229 --P----QGL-ILCAASSIW--------------------------LPV-SRNVLFDFLK
XP_020227921.1/1-228 --P----LGV-ILSAVTSVW--------------------------LPV-SPNVLFDFLR
XP_007135673.1/1-226 --P----LGV-ILSAVSSLW--------------------------LPV-SPNVLFDFLR
XP_014515030.1/1-226 --P----LGV-ILSAVSSVW--------------------------LPV-SPNALFDFLR
XP_017407449.1/1-226 --P----LGV-ILSAVSSVW--------------------------LPV-SPNALFDFLR
XP_010691342.1/1-224 --P----IGV-ILCAVSSVW--------------------------LPV-SPSVVFDFLR
XP_021731998.1/1-222 --P----IGV-ILCAVSSVW--------------------------LPV-SPSLLFDFLR
XP_021736870.1/1-224 --P----IGV-ILCAVSSVW--------------------------LPV-SPSVLFDFLR
XP_024969955.1/1-229 --P----LGV-ILCAVSSIW--------------------------LPV-SHTVLFDFLR
XP_024969954.1/1-229 --P----LGV-ILCAVSSIW--------------------------LPV-SHTVLFDFLR
XP_024969953.1/1-229 --P----LGV-ILCAVSSIW--------------------------LPV-SHTVLFDFLR
XP_021984354.1/1-227 --P----LGV-ILCAVSSIW--------------------------LPV-SHTLLFDFLR
XP_017232370.1/1-228 --P----LGA-VLCAILSVW--------------------------LPV-PHQSLFDFLR
XP_017216573.1/1-228 --P----LGV-VLCAVLSVW--------------------------LPV-SHQNLFDFLR
XP_017248777.1/1-227 --P----LGV-ILCAVTSVW--------------------------LPV-SHLVLFDFLR
XP_021627148.1/1-226 --P----IGV-ILCAVSSIW--------------------------LPV-SPSVLFDFLR
XP_021627146.1/1-226 --P----IGV-ILCAVSSIW--------------------------LPV-SPSVLFDFLR
XP_021627145.1/1-226 --P----IGV-ILCAVSSIW--------------------------LPV-SPSVLFDFLR
XP_019196965.1/1-228 --P----LGV-ILSAVSSVW--------------------------LPV-SHHALFEFLR
XP_019196967.1/1-228 --P----LGV-ILSAVSSVW--------------------------LPV-SHHALFEFLR
XP_022848789.1/1-228 --P----LGV-ILCAVSSVW--------------------------LPI-SHHVLFDFLR
XP_011072373.1/1-228 --P----LGT-ILSAVSCVW--------------------------LPV-SHHVLFDFLR
XP_021907286.1/1-215 --P----LGV-LLCATHCLW--------------------------LPI-ESRKLFDFFR
XP_020885309.1/1-217 --P----PGI-ILCAATSFW--------------------------VPV-PPQTVFDYLR
XP_020885307.1/1-217 --P----PGI-ILCAATSFW--------------------------VPV-PPQTVFDYLR
NP_180796.2/1-218    --P----PGV-IICAATSFW--------------------------LPA-PPNTVFDFLR
XP_006296541.1/1-217 --P----PGS-VLAAASSFW--------------------------VPV-PPKTVFDFLR
XP_010510028.1/1-215 --P----PGV-VISAATSFW--------------------------VPA-PPNIVFDSLR
XP_010414006.1/1-214 --P----PGV-VFSAATSFW--------------------------VPA-PPKIVFDFLR
XP_010469602.1/1-219 --P----PGV-VFSAATSFW--------------------------VPA-PPKIVFDFLR
XP_010417818.1/1-218 --P----PGV-VLSASTSFW--------------------------VPV-PPTIVFDFLR
XP_010510869.1/1-220 --P----PGV-VISASTSFW--------------------------VPC-PPTIVFDFLR
XP_010510870.1/1-218 --P----PGV-VISASTSFW--------------------------VPC-PPTIVFDFLR
XP_018484769.1/1-219 --P----VGV-ILYASTSFW--------------------------LPV-SPNTVFDFLR
XP_018484754.1/1-219 --P----VGV-ILYASTSFW--------------------------LPV-SPNTVFDFLR
XP_013634882.1/1-219 --P----VGV-ILYASTSFW--------------------------LPV-PPKTVFDFLR
XP_018513778.1/1-219 --P----VGV-ILYASTSFW--------------------------LPV-PPKTVFDFLR
XP_009141217.1/1-219 --P----VGV-ILYASTSFW--------------------------LPV-PPKTVFDFLR
XP_013747219.1/1-219 --P----VGV-ILYASTSFW--------------------------LPV-PPKTVFDFLR
XP_024003849.1/1-217 --P----PGV-ILCAATSFW--------------------------VPV-PPSTVFDFLR
XP_018437853.1/1-211 --P----EGL-ILCAAHSFW--------------------------VPV-TPITVFDYLR
XP_009143975.1/1-211 --P----EGL-ILCAAHSFW--------------------------VPA-PPTTVFDYLR
XP_009143974.1/1-211 --P----EGL-ILCAAHSFW--------------------------VPA-PPTTVFDYLR
XP_022575029.1/1-211 --P----EGL-ILCAAHSFW--------------------------VPA-PPTTVFDYLR
XP_013748684.1/1-211 --P----EGL-ILCAAHSFW--------------------------VPA-PPTTVFDYLR
XP_013694194.1/1-211 --P----EGL-ILCAAHSFW--------------------------VPA-PPTTVFDYLR
XP_013634261.1/1-211 --P----EGL-ILCAAHSFW--------------------------VPV-PPITVFDYLR
XP_013741430.1/1-210 --P----EGL-ILCAAHSFW--------------------------VPV-PPITVFDYLR
XP_017254373.1/1-221 --P----PGI-VISGATSFW--------------------------IPV-PPKRVFDFLR
XP_017254240.1/1-222 --P----YGT-IVSVASSFW--------------------------IQF-SPEKVFDFLN
XP_017221582.1/1-224 --P----AGT-VISAASSFW--------------------------IPV-PPKRVFDFLI
XP_017221584.1/1-224 --P----DGT-VISAASSFW--------------------------IPV-PPKRVFDFLI
XP_015941575.1/1-215 --P----SGI-VIGAATCFH--------------------------LAV-PPNSFFDRVR
XP_025620358.1/1-215 --P----SGI-VIGAATCFH--------------------------LAV-PPNSFFDRVR
XP_016176493.1/1-215 --P----SGI-VIGAATCFH--------------------------LAV-PPNSFFDRVR
XP_003610661.2/1-228 --P----VSV-VLSAATSFW--------------------------LPV-PPRRVFDFLR
XP_004496853.1/1-220 --G----SGL-ILSAGTSLW--------------------------LPV-LPKRLFDFLR
XP_006353342.1/1-230 --P----NGM-VLSASRSLW--------------------------LPV-THQRLFDFLR
XP_015070591.1/1-229 --L----NGM-VLSASRTLW--------------------------LPI-THQRLFDFLR
XP_019068356.1/1-229 --L----NGM-VLSASRTLW--------------------------LPI-THERLSDFLR
XP_004234441.1/1-229 --L----NGM-VLSASRTLW--------------------------LPI-THERLSDFLR
XP_024926606.1/1-217 --P----PGT-TLVFATSLG--------------------------LSA-PPKKLFNFLR
XP_024525400.1/1-231 --P----HGI-VLSAATTLW--------------------------LPL-APARVFDYLR
XP_002965186.2/1-231 --P----HGI-VLSAATTLW--------------------------LPL-APARVFDYLR
XP_002962272.2/1-231 --P----HGI-VLSAATTLW--------------------------LPL-APARVFDYLR
XP_019441536.1/1-226 --P----SGL-VLNIATSFR--------------------------LQI-PPIRVFTFLR
XP_019441537.1/1-226 --P----SGL-VLNIATSFR--------------------------LQI-PPIRVFTFLR
XP_019441535.1/1-226 --P----SGL-VLNIATSFR--------------------------LQI-PPIRVFTFLR
XP_019451758.1/1-229 --P----IGL-ILNVSTSFL--------------------------LPV-SPIKIFNFLR
XP_012850835.1/1-238 --P----FGI-QLCVATSFW--------------------------LPI-PPKRVFNFLR
XP_020552079.1/1-225 --P----VGL-HLTIATSFW--------------------------LPI-PPKKVFDFLR
XP_020552078.1/1-225 --P----VGL-HLTIATSFW--------------------------LPI-PPKKVFDFLR
XP_020552080.1/1-223 --P----VGL-HLTIATSFW--------------------------LPI-PPKKVFDFLR
XP_009408807.2/1-226 --P----PGV-VLNASTSIW--------------------------LPI-TPKRVFELLR
XP_006342404.1/1-225 --P----RGL-VLCASTSIW--------------------------LPV-LPKLVFDFLG
XP_025887998.1/1-218 --P----RGL-VLCASTSIW--------------------------LPV-LPKLLFDFLR
XP_015081481.1/1-225 --P----RGL-VLCASTSIW--------------------------LPV-LPKLLFDFLR
XP_022000230.1/1-232 --P----SGG-VLSANMSVW--------------------------MPM-PHQRLFALML
XP_022000229.1/1-232 --P----SGG-VLSANMSVW--------------------------MPM-PHQRLFALML
XP_022037790.1/1-222 --P----SGA-VLSASMSVW--------------------------MPI-QHQRLFALML
XP_022037789.1/1-222 --P----SGA-VLSASMSVW--------------------------MPI-QHQRLFALML
XP_024991925.1/1-226 --P----SGA-VLSATMSVW--------------------------MPM-PHQSLFALML
XP_023760005.1/1-229 --P----S-A-VLSATMSVW--------------------------MPM-PHQRLFALML
XP_023760001.1/1-229 --P----S-A-VLSATMSVW--------------------------MPM-PHQRLFALML
XP_017251723.1/1-236 --P----SEV-VLSATMSVR--------------------------MPV-QPHQLFGMLK
XP_017243321.1/1-239 --P----SGI-VLSATLSVW--------------------------MPV-KQQQLFDLLQ
XP_017243319.1/1-239 --P----SGI-VLSATLSVW--------------------------MPV-KQQQLFDLLQ
XP_017243320.1/1-239 --P----SGI-VLSATLSVW--------------------------MPV-KQQQLFDLLQ
XP_017255563.1/1-234 --P----SGI-ILSATLSVW--------------------------IPV-QQQQLFELLQ
XP_017255562.1/1-234 --P----SGI-ILSATLSVW--------------------------IPV-QQQQLFELLQ
XP_012852237.1/1-226 --P----SGI-ILSATTTVW--------------------------MPV-SPHHLLEFLQ
XP_012852238.1/1-226 --P----SGI-ILSATTTVW--------------------------MPV-SPHHLLEFLQ
XP_012852236.1/1-226 --P----SGI-ILSATTTVW--------------------------MPV-SPHHLLEFLQ
XP_012852235.1/1-226 --P----SGI-ILSATTTVW--------------------------MPV-SPHHLLEFLQ
XP_011076208.1/1-227 --P----PGV-IMSATTTVW--------------------------MPV-LPHHLLEFLQ
XP_019200484.1/1-228 --P----PGV-VLSATKTIW--------------------------LPI-SRQHLFDFLR
XP_019200481.1/1-228 --P----PGV-VLSATKTIW--------------------------LPI-SRQHLFDFLR
XP_022879034.1/1-223 --P----LGT-VLSAVTTVW--------------------------LPI-SCHYLFDFLR
XP_018623115.1/1-238 --P----IGV-MLSATKTIW--------------------------LPV-KQQRLLDFLL
XP_018623116.1/1-238 --P----IGV-MLSATKTIW--------------------------LPV-KQQRLLDFLL
XP_016449095.1/1-238 --P----IGV-MLSATKTIW--------------------------LPV-KQQRLLDFLL
XP_009771835.1/1-238 --P----IGV-VLSATKTIW--------------------------LPV-KQQRLLDFLL
XP_009771833.1/1-238 --P----IGV-VLSATKTIW--------------------------LPV-KQQRLLDFLL
XP_019248454.1/1-225 --P----IGV-VLSATKTIW--------------------------LPV-KQQRLLDFLL
XP_016564677.1/1-235 --P----SGV-MLSATRTIW--------------------------LPI-THQRLFDFLR
XP_016564676.1/1-235 --P----SGV-MLSATRTIW--------------------------LPI-THQRLFDFLR
XP_009606534.1/1-237 --P----PGI-VLSATRTIW--------------------------LPV-THQRLFDFLR
XP_016457239.1/1-237 --P----PGI-VLSATRTIW--------------------------LPV-THQRLFDFLR
XP_009606533.1/1-237 --P----PGI-VLSATRTIW--------------------------LPV-THQRLFDFLR
XP_009606532.1/1-237 --P----PGI-VLSATRTIW--------------------------LPV-THQRLFDFLR
XP_016457238.1/1-237 --P----PGI-VLSATRTIW--------------------------LPV-THQRLFDFLR
XP_016468064.1/1-237 --P----PGI-VLSATRTIW--------------------------LPV-THQRLFDFLR
XP_009774404.1/1-237 --P----PGI-VLSATRTIW--------------------------LPV-THQRLFDFLR
XP_016468062.1/1-237 --P----PGI-VLSATRTIW--------------------------LPV-THQRLFDFLR
XP_016468061.1/1-237 --P----PGI-VLSATRTIW--------------------------LPV-THQRLFDFLR
XP_009774402.1/1-237 --P----PGI-VLSATRTIW--------------------------LPV-THQRLFDFLR
XP_009774401.1/1-237 --P----PGI-VLSATRTIW--------------------------LPV-THQRLFDFLR
XP_019251817.1/1-237 --P----PGI-VLSATRTIW--------------------------LPV-THQRLFDFLR
XP_019251815.1/1-237 --P----PGI-VLSATRTIW--------------------------LPV-THQRLFDFLR
XP_019251816.1/1-237 --P----PGI-VLSATRTIW--------------------------LPV-THQRLFDFLR
XP_011470395.1/1-233 --P----SGF-VLSAATSVW--------------------------MPV-SRSRLFDFLR
XP_004309219.2/1-233 --P----SGF-VLSAATSVW--------------------------MPV-SRSRLFDFLR
XP_024180881.1/1-233 --P----SGF-VLSAATSVW--------------------------MPV-SRSRLFDFLR
XP_024180911.1/1-233 --P----SGF-VLSAATSVW--------------------------MPV-SRSRLFDFLR
XP_019450525.1/1-216 --P----IGI-VLSAATSVW--------------------------MPV-SQQRLFDFLR
XP_019465255.1/1-230 --P----SGI-VLSAATSVW--------------------------MPV-SQQRLFDFLH
XP_014502466.1/1-227 --A----PGY-VLSASTCVW--------------------------LPV-PRQRVFDFLR
XP_017438850.1/1-222 --V----PGY-VLSASTCVW--------------------------LPV-PRQRVFDFLR
XP_006599623.1/1-221 --A----PGI-VLSASTSVW--------------------------MPV-SRKRVFDFLR
XP_006599622.1/1-221 --A----PGI-VLSASTSVW--------------------------MPV-SRKRVFDFLR
XP_006599621.1/1-221 --A----PGI-VLSASTSVW--------------------------MPV-SRKRVFDFLR
XP_004515730.1/1-240 --S----TSI-VLSAATSVW--------------------------LPV-SRQRVFDFLR
XP_013442370.1/1-239 --N----SSI-VLSAATSVW--------------------------IPL-PRQRVFDFLR
XP_004252035.1/1-225 --P----PGI-VLSAATSFW--------------------------LPI-APKTVFDYLR
XP_015060726.1/1-225 --P----PGI-VLSAATSFW--------------------------LPI-APKTVFDYLR
XP_006589608.1/1-229 --P----SGI-VLSAATSLW--------------------------LPV-PARRVFDFLR
XP_006589605.1/1-230 --P----SGI-VLSAATSLW--------------------------LPV-PARRVFDFLR
XP_006849468.1/1-231 --P----NGV-VLSAATSLW--------------------------LPV-SPQNIFNFFR
XP_020526173.1/1-231 --P----NGV-VLSAATSLW--------------------------LPV-SPQNIFNFFR
XP_020109515.1/1-228 --P----NGV-VLSAATSLW--------------------------LPV-SCERVFSFFR
NP_001310555.1/1-224 --P----SGV-VLSAATSIW--------------------------LPV-PCDRAFAFVR
XP_010544375.1/1-225 --P----TGI-IRSAATSVW--------------------------LPV-HSQRLFDFLR
NP_001314001.1/1-230 --P----LGI-VLSAATSVW--------------------------MPI-TQQTLFGFLR
XP_016732454.1/1-230 --P----LGI-VLSAATSVW--------------------------MPI-TQQTLFGFLR
XP_016732453.1/1-230 --P----LGI-VLSAATSVW--------------------------MPI-TQQTLFGFLR
XP_012484067.1/1-230 --P----LGI-VLSAATSVW--------------------------MPI-TQQTLFGFLR
XP_012484066.1/1-230 --P----LGI-VLSAATSVW--------------------------MPI-TQQTLFGFLR
XP_017631791.1/1-230 --P----LGI-VLSAATSVW--------------------------MPI-TQQTLFNLLR
XP_017631789.1/1-230 --P----LGI-VLSAATSVW--------------------------MPI-TQQTLFNLLR
XP_016728129.1/1-230 --P----LGI-VLSAATSVW--------------------------MPI-TQQTLFNLLR
XP_016728128.1/1-230 --P----LGI-VLSAATSVW--------------------------MPI-TQQTLFNLLR
XP_021671684.1/1-240 --P----PGI-VLSAATSVW--------------------------LPV-MRERLFDFLR
XP_021671683.1/1-240 --P----PGI-VLSAATSVW--------------------------LPV-MRERLFDFLR
XP_021671685.1/1-240 --P----PGI-VLSAATSVW--------------------------LPV-MRERLFDFLR
XP_002318231.3/1-240 --P----DGI-VLSAATSVW--------------------------LPV-SRQRLFDFLR
XP_024438602.1/1-240 --P----DGI-VLSAATSVW--------------------------LPV-SRQRLFDFLR
XP_024442055.1/1-239 --P----DGI-VLSVSTSVW--------------------------LPV-SQQRLFDFLR
XP_002322460.1/1-239 --P----DGI-VLSVSTSVW--------------------------LPV-SQQRLFDFLR
XP_011048682.1/1-239 --P----DGI-VLSVSTSVW--------------------------LPV-SQQRLFDFLR
XP_011048684.1/1-239 --P----DGI-VLSVSTSVW--------------------------LPV-SQQRLFDFLR
XP_011048683.1/1-239 --P----DGI-VLSVSTSVW--------------------------LPV-SQQRLFDFLR
XP_019081404.1/1-234 --P----PGV-ILSAATSVW--------------------------MPI-MHQQLFNFLR
XP_003634081.1/1-234 --P----PGV-ILSAATSVW--------------------------MPI-MHQQLFNFLR
XP_008377249.1/1-231 --P----PGI-VLSVATTVW--------------------------VPV-SRHRVFEFLR
XP_017189459.1/1-231 --P----PGI-VLSVATTVW--------------------------VPV-SRHRVFEFLR
XP_008377244.1/1-231 --P----PGI-VLSVATTVW--------------------------VPV-SRHRVFEFLR
XP_009371302.1/1-231 --P----PGI-VLSVATTVW--------------------------VPV-SRHRVFEFLR
XP_009371299.1/1-231 --P----PGI-VLSVATTVW--------------------------VPV-SRHRVFEFLR
XP_008376545.1/1-231 --P----PGI-VLSVTTTVW--------------------------VPV-SRHRVFEFLR
XP_008376544.1/1-231 --P----PGI-VLSVTTTVW--------------------------VPV-SRHRVFEFLR
XP_017188609.1/1-231 --P----PGI-VLSVTTTVW--------------------------VPV-SRHRVFEFLR
XP_022945426.1/1-238 --P----PGI-VLSAATSVW--------------------------MPV-TQQRLFAFLQ
XP_022945425.1/1-238 --P----PGI-VLSAATSVW--------------------------MPV-TQQRLFAFLQ
XP_023541229.1/1-238 --P----PGI-VLSAATSVW--------------------------MPV-TQQRLFAFLQ
XP_023541228.1/1-238 --P----PGI-VLSAATSVW--------------------------MPV-TQQRLFAFLQ
XP_022968414.1/1-238 --P----PGI-VLSASTSVW--------------------------MPV-TQQRLFAFLQ
XP_022968413.1/1-238 --P----PGI-VLSASTSVW--------------------------MPV-TQQRLFAFLQ
XP_023552381.1/1-232 --P----PGI-VLSAATSVW--------------------------MPV-TQQRLFAFLQ
XP_022922479.1/1-232 --P----PGI-VLSAATSVW--------------------------MPV-TQQRLFAFLQ
XP_023552380.1/1-232 --P----PGI-VLSAATSVW--------------------------MPV-TQQRLFAFLQ
XP_022922478.1/1-232 --P----PGI-VLSAATSVW--------------------------MPV-TQQRLFAFLQ
XP_022984644.1/1-232 --P----PGI-VLSAATSVW--------------------------MPV-TQQRLFAFLQ
XP_022984643.1/1-232 --P----PGI-VLSAATSVW--------------------------MPV-TQQRLFAFLQ
XP_008439185.1/1-242 --P----PGI-VLSAATSVW--------------------------MPV-TQQRLFEFLQ
XP_008439184.1/1-242 --P----PGI-VLSAATSVW--------------------------MPV-TQQRLFEFLQ
XP_011651140.1/1-242 --P----PGI-VLSAATSVW--------------------------MPV-TQQRLFAFLQ
XP_004140784.1/1-242 --P----PGI-VLSAATSVW--------------------------MPV-TQQRLFAFLQ
XP_009391954.1/1-234 --P----AGL-VLSVATSVW--------------------------IPA-PPKRLFDFLC
XP_003581542.2/1-227 --P----PGV-VLCAATSVW--------------------------MLV-TPERLFQFLC
XP_006652658.1/1-224 --P----PGV-VLSAATSVW--------------------------MPV-MPHRLFNFLR
XP_015637147.1/1-225 --P----PGV-VLSAATSVW--------------------------MPV-MPERLFNFLH
XP_025823430.1/1-225 --P----PGV-VLSAATAVW--------------------------MPV-TPERLFNFLR
XP_004978185.1/1-225 --P----PGV-VLSAATAVW--------------------------MPV-TPERLFNFLR
XP_021317935.1/1-228 --P----PGV-VLSVATAVW--------------------------LPV-TPERLFNFLR
NP_001336434.1/1-226 --P----PGV-VLSVATAVW--------------------------LPV-TPERLFNFLR
XP_019709552.1/1-234 --P----QGV-VLSAATSVL--------------------------VPV-PPQRLFDFLR
XP_015696802.1/1-257 --P----PGV-VLSATTSVR--------------------------LPGTLPQRVFDYLR
XP_015612621.1/1-258 --P----PGV-VLSATTSVR--------------------------LPGTLPQRVFDYLR
XP_004957413.1/1-253 --P----PGV-VLSATTSVR--------------------------LPSTPPQRVFDYLR
XP_025801167.1/1-251 --P----PGV-VLSATTSVR--------------------------LPATPPQRVFDYLR
NP_001105125.2/1-256 --P----PGV-VLSATTSVR--------------------------LPATSPQRVFDYLR
XP_002462701.1/1-258 --P----PGV-VLSATTSVR--------------------------LPVTSPQRVFDYLR
XP_020262629.1/1-222 --P----PGV-VLSAATSVW--------------------------VPVP----------
XP_020699782.1/1-235 --P----PGV-VLSAATSVW--------------------------LPLVSAQRLFEFLR
XP_020699776.1/1-235 --P----PGV-VLSAATSVW--------------------------LPLVSAQRLFEFLR
XP_020573166.1/1-235 --P----PGV-VLSAATSVW--------------------------LPMVSAQRLFEFLR
XP_020187432.1/1-228 --P----AGV-VLCAATSVW--------------------------MLVT-PKRLFNFLC
XP_025656795.1/1-233 --P----PSI-VLCAATSVW--------------------------MPVS-RQRVFDFLR
XP_025656792.1/1-233 --P----PSI-VLCAATSVW--------------------------MPVS-RQRVFDFLR
XP_025656794.1/1-233 --P----PSI-VLCAATSVW--------------------------MPVS-RQRVFDFLR
XP_020979736.1/1-233 --P----PSI-VLCAATSVW--------------------------MPVS-RQRVFDFLR
XP_020999151.1/1-233 --P----PSI-VLCAATSVW--------------------------MPVS-RQRVFDFLR
XP_015967451.1/1-233 --P----PSI-VLCAATSVW--------------------------MPVS-RQRVFDFLR
XP_020999150.1/1-233 --P----PSI-VLCAATSVW--------------------------MPVS-RQRVFDFLR
XP_025701970.1/1-233 --P----PSI-VLCAATSVW--------------------------MPVS-RQRVFDFLR
XP_025701968.1/1-233 --P----PSI-VLCAATSVW--------------------------MPVS-RQRVFDFLR
XP_025701969.1/1-233 --P----PSI-VLCAATSVW--------------------------MPVS-RQRVFDFLR
XP_020221720.1/1-222 --A----PGI-VLSASTSVW--------------------------MPVS-RQKVFDFLR
XP_007152327.1/1-222 --A----PGI-VLSASTSVW--------------------------MPVS-RQRVFDFLR
XP_006587345.1/1-222 --A----PGI-VLSASTSVW--------------------------MPVS-RQRVFDFLR
XP_006587344.1/1-222 --A----PGI-VLSASTSVW--------------------------MPVS-RQRVFDFLR
XP_010035104.1/1-239 --P----PGI-VLSAATSIW--------------------------MPIA-HETVFNFVR
XP_021765162.1/1-234 --P----SGV-VLSASTSVW--------------------------IPVP-RQHIFEFLR
XP_021765160.1/1-234 --P----SGV-VLSASTSVW--------------------------IPVP-RQHIFEFLR
XP_021730123.1/1-234 --P----SGV-VLSASTSVW--------------------------IPVP-RQHIFEFLR
XP_021842103.1/1-232 --P----SGV-VLSASTSVW--------------------------IPVP-RQHIFDFLR
XP_021842106.1/1-232 --P----SGV-VLSASTSVW--------------------------IPVP-RQHIFDFLR
XP_021842105.1/1-232 --P----SGV-VLSASTSVW--------------------------IPVP-RQHIFDFLR
XP_021842104.1/1-232 --P----SGV-VLSASTSVW--------------------------IPVP-RQHIFDFLR
XP_010693471.1/1-235 --P----SGV-VLSASTSVW--------------------------LPVT-RQHLFDFLR
XP_019107938.1/1-235 --P----SGV-VLSASTSVW--------------------------LPVT-RQHLFDFLR
XP_010693469.1/1-235 --P----SGV-VLSASTSVW--------------------------LPVT-RQHLFDFLR
XP_019107937.1/1-235 --P----SGV-VLSASTSVW--------------------------LPVT-RQHLFDFLR
XP_020163116.1/1-258 --P----PGV-VLSATTSVR--------------------------LPGTPPQRVFDYLR
XP_020163109.1/1-258 --P----PGV-VLSATTSVR--------------------------LPGTPPQRVFDYLR
XP_003578475.1/1-267 --P----PGV-VLSATTSVR--------------------------LPGTPPQRVFDYLR
XP_011012369.1/1-242 --P----DGI-VLSAATSVW--------------------------LPVS-RQRLFDFLR
XP_011012370.1/1-242 --P----DGI-VLSAATSVW--------------------------LPVS-RQRLFDFLR
XP_008234278.1/1-230 --P----SGI-VLSGSTSVW--------------------------LPVS-RHRLFDFLR
XP_021827117.1/1-230 --P----AGI-VLSGSTSVW--------------------------LPVS-RHRLFDFLR
XP_021827116.1/1-230 --P----AGI-VLSGSTSVW--------------------------LPVS-RHRLFDFLR
XP_020413581.1/1-230 --P----AGI-VLSGSTSVW--------------------------LPVS-RHRLFDFLR
XP_007219555.2/1-230 --P----AGI-VLSGSTSVW--------------------------LPVS-RHRLFDFLR
XP_018456926.1/1-230 --P----SGV-VLSAATSMW--------------------------LPVT-QQRLFEFLC
XP_013613235.1/1-226 --P----SGV-VLSAATSMW--------------------------LPVT-QQRLFEFLC
XP_013686466.1/1-227 --P----SGV-VLSAATSMW--------------------------LPVT-QQRLFEFLC
XP_009106897.1/1-227 --P----SGV-VLSAATSMW--------------------------LPVT-QQRLFEFLC
XP_006401857.1/1-229 --P----SGV-VLSAATSMW--------------------------LPVT-QQRLFEFLC
XP_020871109.1/1-230 --P----SGI-VLSAATSLW--------------------------LPVT-QQRLFEFLC
NP_001332093.1/1-229 --P----SGI-VLSAATSLW--------------------------LPVT-QQRLFEFLC
NP_200030.1/1-229    --P----SGI-VLSAATSLW--------------------------LPVT-QQRLFEFLC
XP_006282099.1/1-225 --P----SGI-VLSAATSLW--------------------------LPVT-QQRLFEFLC
XP_019095386.1/1-229 --P----SGI-VLSAATSLW--------------------------LPVT-QQRLFEFLC
XP_019095387.1/1-229 --P----SGI-VLSAATSLW--------------------------LPVT-QQRLFEFLC
XP_019091174.1/1-228 --P----SGI-VLSAATSLW--------------------------LPVT-QQRLFEFLC
XP_019091179.1/1-228 --P----SGI-VLSAATSLW--------------------------LPVT-QQRLFEFLC
XP_019087173.1/1-228 --P----SGI-VLSAATSLW--------------------------LPVT-QQRLFEFLC
XP_010555654.1/1-234 --P----SGI-VLSAATSVW--------------------------LPLD-PWRLFDFLR
XP_010555656.1/1-234 --P----SGI-VLSAATSVW--------------------------LPLD-PWRLFDFLR
XP_010555655.1/1-234 --P----SGI-VLSAATSVW--------------------------LPLD-PWRLFDFLR
XP_010537199.1/1-242 --P----SGV-ILSAATSVW--------------------------IPVA-QHRLFDFLR
XP_010537197.1/1-242 --P----SGV-ILSAATSVW--------------------------IPVA-QHRLFDFLR
XP_010537198.1/1-241 --P----SGV-ILSAATSVW--------------------------IPVA-QHRLFDFLR
Con_90%              ..s.....sh.llsAhsohh..........................l.....t.lhphl+
Con_80%              ..P....sGh.lLsAtsShh..........................l...s.thlhcFLR
Con_70%              ..P....sGh.lLsAssShh..........................Lss.sPthlacFLR
Con_60%              ..P....sGl.VLsAsoShW..........................LPs.sPpplFcFLR
Con_50%              ..P....sGl.VLoAuTShW..........................LPl.sPppLF-FLR


XP_020582785.1/1-284 EH-------RS----------------------------------E-------WADSLID
XP_020682623.1/1-284 EH-------RS----------------------------------E-------WADSIVD
XP_020682624.1/1-284 EH-------RS----------------------------------E-------WADSIVD
XP_006846015.1/1-286 EH-------RS----------------------------------E-------WADSGVD
NP_001142394.1/1-289 EH-------RS----------------------------------E-------WADPGID
NP_001146215.1/1-288 EH-------RS----------------------------------E-------WADPGVD
XP_020405718.1/1-288 EH-------RS----------------------------------E-------WADPGVD
XP_002443548.1/1-287 EH-------RS----------------------------------E-------WADPGVD
XP_025808876.1/1-287 EH-------RS----------------------------------E-------WADPGVD
XP_004963232.1/1-287 EH-------RS----------------------------------E-------WADPGVD
XP_020192001.1/1-285 EH-------RS----------------------------------E-------WADPGVD
XP_003562005.1/1-285 EH-------RS----------------------------------E-------WADPGVD
XP_004982339.1/1-286 EH-------RS----------------------------------E-------WADPGVD
XP_025796542.1/1-286 EH-------RS----------------------------------E-------WADPGVD
NP_001347456.1/1-286 EH-------RS----------------------------------E-------WADPGVD
NP_001352464.1/1-286 EH-------RS----------------------------------E-------WADPGVD
XP_002464180.1/1-286 EH-------RS----------------------------------E-------WADPGVD
XP_015689987.1/1-286 EH-------RS----------------------------------E-------WADPGVD
XP_015632484.1/1-286 EH-------RS----------------------------------E-------WADPGVD
XP_015620816.1/1-285 EH-------RS----------------------------------E-------WADPGVD
XP_006664738.1/1-285 EH-------RS----------------------------------E-------WADPGVD
XP_020190954.1/1-286 EH-------RS----------------------------------E-------WADPGVD
XP_020163543.1/1-286 EH-------RS----------------------------------E-------WADPGVD
XP_003577453.1/1-286 EH-------RS----------------------------------E-------WADPGVD
XP_020575104.1/1-285 EH-------RS----------------------------------E-------WADSSVD
XP_020696902.1/1-286 EH-------RS----------------------------------E-------WADSGVD
XP_020578108.1/1-284 EH-------RS----------------------------------E-------WADFGAD
XP_020698923.1/1-284 EH-------RS----------------------------------E-------WADYGAD
XP_020096610.1/1-279 EH-------RS----------------------------------E-------WADCGVD
XP_009412508.1/1-287 EH-------RS----------------------------------E-------WADFGVD
XP_020271156.1/1-284 EH-------RS----------------------------------E-------WADCGVD
XP_020273236.1/1-287 EH-------RS----------------------------------E-------WADCGVD
XP_020092733.1/1-287 EH-------RS----------------------------------E-------WADCGID
XP_019701934.1/1-288 EH-------RS----------------------------------E-------WADCGVD
XP_010905421.1/1-288 EH-------RS----------------------------------E-------WADCGVD
XP_010905420.1/1-289 EH-------RS----------------------------------E-------WADCGVD
XP_010923678.1/1-288 EH-------RS----------------------------------E-------WADCGVD
XP_008794452.1/1-288 EH-------RS----------------------------------E-------WADCAVD
XP_009397391.1/1-288 EH-------RS----------------------------------E-------WADCGVD
XP_009408551.1/1-288 EH-------RS----------------------------------E-------WADCGVD
XP_009399045.1/1-288 EH-------RS----------------------------------E-------WADCGVD
XP_009388964.1/1-288 EH-------RS----------------------------------E-------WADCGVD
XP_009397717.1/1-288 EH-------RS----------------------------------E-------WADCGVD
XP_018488876.1/1-280 EH-------RA----------------------------------E-------WADYGVD
XP_013587486.1/1-280 EH-------RA----------------------------------E-------WADYGVD
XP_009115139.1/1-280 EH-------RA----------------------------------E-------WADYGVD
XP_013662459.1/1-280 EH-------RA----------------------------------E-------WADYGVD
XP_013707293.1/1-280 EH-------RA----------------------------------E-------WADYGVD
XP_006415465.2/1-280 EH-------RA----------------------------------E-------WADYGVD
XP_019099288.1/1-280 EH-------RA----------------------------------E-------WADYGVD
NP_174337.1/1-280    EH-------RA----------------------------------E-------WADYGVD
XP_020867051.1/1-280 EH-------RA----------------------------------E-------WADYGVD
XP_020867052.1/1-280 EH-------RA----------------------------------E-------WADYGVD
XP_010460847.1/1-280 EH-------RA----------------------------------E-------WADYGVD
XP_019094482.1/1-280 EH-------RA----------------------------------E-------WADYGVD
XP_006306764.1/1-280 EH-------RA----------------------------------E-------WADYGVD
XP_010538268.1/1-285 EH-------RS----------------------------------E-------WADHGVD
XP_010519070.1/1-284 EH-------RS----------------------------------E-------WADYGVD
XP_018436356.1/1-280 EH-------RS----------------------------------E-------WADYGVD
XP_013637594.1/1-286 EH-------RS----------------------------------E-------WADYGVD
XP_013692700.1/1-286 EH-------RS----------------------------------E-------WADYGVD
XP_009143747.1/1-286 EH-------RS----------------------------------E-------WADYGVD
XP_018484893.1/1-285 EH-------RS----------------------------------E-------WADYGVD
XP_013637264.1/1-280 EH-------RS----------------------------------E-------WADYGVD
XP_013637263.1/1-280 EH-------RS----------------------------------E-------WADYGVD
XP_022557131.1/1-280 EH-------RS----------------------------------E-------WADYGVD
XP_013729051.1/1-280 EH-------RS----------------------------------E-------WADYGVD
XP_013744548.1/1-280 EH-------RS----------------------------------E-------WADYGMD
XP_009141396.1/1-280 EH-------RS----------------------------------E-------WADYGVD
XP_006410621.1/1-284 EH-------RS----------------------------------E-------WADYGVD
NP_181018.1/1-284    EH-------RS----------------------------------E-------WADYGVD
XP_020884308.1/1-284 EH-------RS----------------------------------E-------WADYGVD
XP_006293359.1/1-284 EH-------RS----------------------------------E-------WADYGVD
XP_010516626.1/1-284 EH-------RS----------------------------------E-------WADYGVD
XP_010509720.1/1-284 EH-------RS----------------------------------E-------WADYGVD
XP_010504942.1/1-284 EH-------RS----------------------------------E-------WADYGVD
XP_017223336.1/1-283 EH-------RS----------------------------------E-------WADSMID
XP_016559371.1/1-285 EH-------RS----------------------------------E-------WADYGVD
XP_016559374.1/1-285 EH-------RS----------------------------------E-------WADYGVD
XP_012830207.1/1-281 EH-------RS----------------------------------E-------WADYAVD
XP_012827547.1/1-287 EH-------RS----------------------------------E-------WADYGVD
XP_022866806.1/1-284 EH-------RS----------------------------------E-------WADYGVD
XP_022866805.1/1-285 EH-------RS----------------------------------E-------WADYGVD
XP_023001017.1/1-285 EH-------RS----------------------------------E-------WADYGVD
XP_023001016.1/1-285 EH-------RS----------------------------------E-------WADYGVD
XP_023519837.1/1-285 EH-------RS----------------------------------E-------WADYGVD
XP_023519836.1/1-285 EH-------RS----------------------------------E-------WADYGVD
XP_022927139.1/1-285 EH-------RS----------------------------------E-------WADYGVD
XP_022927138.1/1-285 EH-------RS----------------------------------E-------WADYGVD
XP_022139982.1/1-285 EH-------RS----------------------------------E-------WADYGVD
XP_004135550.1/1-285 EH-------RS----------------------------------E-------WADYGVD
XP_008445207.1/1-285 EH-------RS----------------------------------E-------WADYGVD
XP_017230337.1/1-286 EH-------RS----------------------------------E-------WADYAID
XP_017230338.1/1-286 EH-------RS----------------------------------E-------WADYAID
XP_010316959.1/1-285 EH-------RS----------------------------------E-------WADYGVD
XP_004233170.1/1-285 EH-------RS----------------------------------E-------WADYGVD
XP_015066839.1/1-285 EH-------RS----------------------------------E-------WADYGVD
XP_015066842.1/1-285 EH-------RS----------------------------------E-------WADYGVD
XP_015066843.1/1-285 EH-------RS----------------------------------E-------WADYGVD
XP_006353018.1/1-285 EH-------RS----------------------------------E-------WADYGVD
XP_006353020.1/1-285 EH-------RS----------------------------------E-------WADYGVD
XP_019178964.1/1-287 EH-------RS----------------------------------E-------WADYGVD
XP_019178963.1/1-287 EH-------RS----------------------------------E-------WADYGVD
XP_019228489.1/1-285 EH-------RS----------------------------------E-------WADYGVD
XP_019228488.1/1-285 EH-------RS----------------------------------E-------WADYGVD
NP_001289498.1/1-285 EH-------RS----------------------------------E-------WADYGVD
XP_009777911.1/1-285 EH-------RS----------------------------------E-------WADYGVD
XP_009612342.1/1-285 EH-------RS----------------------------------E-------WADYGVD
XP_009612341.1/1-285 EH-------RS----------------------------------E-------WADYGVD
XP_006363649.1/1-287 EH-------RS----------------------------------E-------WADYGVD
XP_015066689.1/1-287 EH-------RS----------------------------------E-------WADYGVD
XP_010315636.1/1-287 EH-------RS----------------------------------E-------WADYGVD
XP_022879368.1/1-285 EH-------RS----------------------------------E-------WADYEID
XP_020554026.1/1-285 EH-------RS----------------------------------E-------WADYGVD
XP_011096678.1/1-285 EH-------RS----------------------------------E-------WADYGVD
XP_011085207.1/1-285 EH-------RS----------------------------------E-------WADYGVD
XP_023003193.1/1-285 EH-------RS----------------------------------E-------WADYGVD
XP_022926723.1/1-285 EH-------RS----------------------------------E-------WADYGVD
XP_023518237.1/1-285 EH-------RS----------------------------------E-------WADYGVD
XP_022146537.1/1-286 EH-------RS----------------------------------E-------WADYGVD
XP_011658319.1/1-286 EH-------RS----------------------------------E-------WADYGVD
XP_008439554.1/1-286 EH-------RS----------------------------------E-------WADYGVD
XP_022978372.1/1-286 EH-------RS----------------------------------E-------WADYGVD
XP_023543833.1/1-286 EH-------RS----------------------------------E-------WADYGVD
XP_022926042.1/1-286 EH-------RS----------------------------------E-------WADYGVD
XP_010247499.1/1-286 EH-------RS----------------------------------E-------WADCGVD
XP_016717556.1/1-285 EH-------RS----------------------------------E-------WADYGID
XP_012455108.1/1-285 EH-------RS----------------------------------E-------WADYGID
XP_016717554.1/1-287 EH-------RS----------------------------------E-------WADYGID
XP_012455107.1/1-287 EH-------RS----------------------------------E-------WADYGID
XP_016711412.1/1-287 EH-------RS----------------------------------E-------WADYGID
XP_017649973.1/1-287 EH-------RS----------------------------------E-------WADYGID
XP_022716449.1/1-285 EH-------RS----------------------------------E-------WADYGVD
XP_022724664.1/1-287 EH-------RS----------------------------------E-------WADHGVD
XP_021298192.1/1-285 EH-------RS----------------------------------E-------WADYGVD
XP_021298193.1/1-294 EH-------RS----------------------------------E-------WADYGVD
XP_021298191.1/1-294 EH-------RS----------------------------------E-------WADYGVD
XP_007022874.2/1-285 EH-------RS----------------------------------E-------WADYGVD
XP_017979205.1/1-294 EH-------RS----------------------------------E-------WADYGVD
XP_017979204.1/1-294 EH-------RS----------------------------------E-------WADYGVD
XP_017236680.1/1-286 EH-------RS----------------------------------E-------WADYGID
XP_010051959.1/1-285 EH-------RS----------------------------------E-------WADHGID
XP_010051960.1/1-285 EH-------RS----------------------------------E-------WADHGID
XP_015882975.1/1-284 EH-------RS----------------------------------E-------WADYGVD
XP_015882797.1/1-284 EH-------RS----------------------------------E-------WADYGVD
XP_010098570.1/1-284 EH-------RS----------------------------------E-------WADYGVD
XP_011012084.1/1-284 EH-------RS----------------------------------E-------WADYGVD
XP_011012083.1/1-284 EH-------RS----------------------------------E-------WADYGVD
XP_006377667.2/1-284 EH-------RS----------------------------------E-------WADYGVD
XP_002281868.2/1-285 EH-------RS----------------------------------E-------WADYGVD
XP_018847863.1/1-285 EH-------RS----------------------------------E-------WADYGVD
XP_018847862.1/1-285 EH-------RS----------------------------------E-------WADYGVD
XP_023919942.1/1-285 EH-------RS----------------------------------E-------WADYGVD
XP_023919941.1/1-285 EH-------RS----------------------------------E-------WADYGVD
XP_018828101.1/1-285 EH-------RS----------------------------------E-------WADYGVD
XP_018828100.1/1-285 EH-------RS----------------------------------E-------WADYGVD
XP_002298892.1/1-285 EH-------RS----------------------------------E-------WADYGVD
XP_011038887.1/1-285 EH-------RS----------------------------------E-------WADYGVD
XP_011038886.1/1-285 EH-------RS----------------------------------E-------WADYGVD
XP_021657817.1/1-285 EH-------RS----------------------------------E-------WADYGVD
XP_021657818.1/1-285 EH-------RS----------------------------------E-------WADYGVD
XP_015577248.1/1-260 EH-------RS----------------------------------E-------WADYGVD
XP_021616343.1/1-285 EH-------RS----------------------------------E-------WADYGVD
XP_021645103.1/1-285 EH-------RS----------------------------------E-------WADYGVD
XP_021592762.1/1-285 EH-------RS----------------------------------E-------WADYGVD
XP_012075627.1/1-285 EH-------RS----------------------------------E-------WADYGVD
XP_008372224.1/1-285 EH-------RS----------------------------------E-------WADYGVD
XP_009362238.1/1-285 EH-------RS----------------------------------E-------WADYGVD
XP_009344405.1/1-284 EH-------RS----------------------------------E-------WADYGVD
XP_008349150.1/1-284 EH-------RA----------------------------------E-------WADYGVD
XP_007214622.1/1-285 EH-------RS----------------------------------E-------WADYGVD
XP_021812393.1/1-285 EH-------RS----------------------------------E-------WADYGVD
XP_024161164.1/1-285 EH-------RS----------------------------------E-------WADYGVD
XP_004293989.1/1-285 EH-------RS----------------------------------E-------WADYGVD
XP_019419795.1/1-282 EH-------RS----------------------------------E-------WADYGVD
XP_019426301.1/1-282 EH-------RS----------------------------------E-------WADYGVD
XP_003524993.1/1-285 EH-------RS----------------------------------E-------WANYEVD
XP_003532788.1/1-285 EH-------RS----------------------------------E-------WANYGVD
XP_020215009.1/1-285 EH-------RS----------------------------------E-------WADYGVD
XP_017409410.1/1-285 EH-------RS----------------------------------E-------WADYGVD
XP_017409418.1/1-285 EH-------RS----------------------------------E-------WADYGVD
XP_014509941.1/1-285 EH-------RS----------------------------------E-------WADYGVD
XP_007159548.1/1-285 EH-------RS----------------------------------E-------WADYGVD
XP_024047818.1/1-288 EH-------RS----------------------------------E-------WADYGVD
XP_006448915.2/1-288 EH-------RS----------------------------------E-------WADYGVD
XP_006468302.1/1-288 EH-------RS----------------------------------E-------WADYGVD
XP_006468303.1/1-287 EH-------RS----------------------------------E-------WADYGVD
XP_024047817.1/1-287 EH-------RS----------------------------------E-------WADYGVD
XP_004504217.1/1-285 EH-------RS----------------------------------E-------WADYGVD
XP_003594520.2/1-281 EH-------RS----------------------------------E-------WADYGVD
XP_024632601.1/1-281 EH-------RS----------------------------------E-------WADYGVD
XP_004486450.1/1-285 EH-------RS----------------------------------E-------WADYGVD
XP_007147487.1/1-285 EH-------RS----------------------------------E-------WADYGVD
XP_017437412.1/1-285 EH-------RS----------------------------------E-------WADYGVD
XP_014518680.1/1-285 EH-------RS----------------------------------E-------WADYGVD
XP_003546255.1/1-285 EH-------RS----------------------------------E-------WADYGVD
XP_020233213.1/1-285 EH-------RS----------------------------------E-------WADYGVD
XP_003535078.1/1-285 EH-------RS----------------------------------E-------WADYGVD
XP_014617344.1/1-285 EH-------RS----------------------------------E-------WADYGVD
XP_015959291.1/1-283 EH-------RS----------------------------------E-------WADYGVD
XP_016197724.1/1-283 EH-------RS----------------------------------E-------WADYGVD
XP_025694278.1/1-283 EH-------RS----------------------------------E-------WADYGVD
XP_006846777.1/1-281 EH-------RS----------------------------------E-------WADSNID
XP_003574071.1/1-280 EH-------RS----------------------------------E-------WADYNFD
XP_020174778.1/1-278 EH-------RS----------------------------------E-------WADYNFD
XP_006661867.1/1-281 EH-------RS----------------------------------E-------WADYNFD
XP_015614901.1/1-281 EH-------RS----------------------------------E-------WADYNFD
XP_004982838.1/1-282 EH-------RS----------------------------------E-------WADYNFD
XP_025796396.1/1-282 EH-------RS----------------------------------E-------WADYNFD
NP_001316502.1/1-284 EH-------RS----------------------------------E-------WADYNFD
XP_021306737.1/1-282 EH-------RS----------------------------------E-------WADYNFD
XP_021306733.1/1-293 EH-------RS----------------------------------E-------WADYNFD
XP_020162053.1/1-281 EH-------RS----------------------------------E-------WADYSMD
XP_006649248.1/1-282 EH-------RS----------------------------------E-------WADYNID
XP_015632709.1/1-282 EH-------RS----------------------------------E-------WADYNID
NP_001296150.1/1-282 EH-------RS----------------------------------E-------WADYNID
XP_008665074.1/1-283 EH-------RS----------------------------------E-------WADYNID
NP_001105994.1/1-283 EH-------RS----------------------------------E-------WADYNID
XP_008660404.1/1-283 EH-------RS----------------------------------E-------WADYNID
XP_002468669.1/1-283 EH-------RS----------------------------------E-------WADYNMD
XP_021309716.1/1-285 EH-------RS----------------------------------E-------WADYNMD
XP_004986037.1/1-283 EH-------RS----------------------------------E-------WADYNID
XP_025795001.1/1-283 EH-------RS----------------------------------E-------WADYNID
XP_020093893.1/1-283 EH-------RS----------------------------------E-------WADYNMD
XP_020093894.1/1-282 EH-------RS----------------------------------E-------WADYNMD
XP_020259754.1/1-283 EH-------RS----------------------------------E-------WADYNID
XP_009401955.1/1-282 EH-------RS----------------------------------E-------WADYNVD
XP_008791541.1/1-283 EH-------RS----------------------------------E-------WADYNID
XP_017698637.1/1-283 EH-------RS----------------------------------E-------WADYNID
XP_010920422.1/1-283 EH-------RS----------------------------------E-------WADYNVD
XP_010920423.1/1-283 EH-------RS----------------------------------E-------WADYNVD
XP_010920419.1/1-283 EH-------RS----------------------------------E-------WADYNVD
XP_010920426.1/1-283 EH-------RS----------------------------------E-------WADYNVD
XP_019709326.1/1-287 EH-------RS----------------------------------E-------WADYKID
XP_008785281.1/1-283 EH-------RS----------------------------------E-------WADYRID
XP_008462593.1/1-288 EH-------RS----------------------------------E-------WADFNVD
XP_022969629.1/1-286 EH-------RS----------------------------------E-------WADFNVD
XP_023536970.1/1-286 EH-------RS----------------------------------E-------WADFNVD
XP_022937855.1/1-288 EH-------RS----------------------------------E-------WADFNVD
XP_022937858.1/1-288 EH-------RS----------------------------------E-------WADFNVD
XP_012827869.1/1-285 EH-------RS----------------------------------E-------WADFNVD
XP_022874815.1/1-279 EH-------RS----------------------------------E-------WADFNVD
XP_022874814.1/1-283 EH-------RS----------------------------------E-------WADFNVD
XP_018434000.1/1-280 EH-------RS----------------------------------E-------WADFNVD
XP_013642496.1/1-279 EH-------RS----------------------------------E-------WADFNVD
XP_009150087.1/1-279 EH-------RS----------------------------------E-------WADFNVD
XP_009150088.1/1-279 EH-------RS----------------------------------E-------WADFNVD
XP_022563181.1/1-279 EH-------RS----------------------------------E-------WADFNVD
XP_013675790.1/1-280 EH-------RS----------------------------------E-------WADFNVD
XP_013623090.1/1-279 EH-------RS----------------------------------E-------WADFNVD
XP_013623089.1/1-280 EH-------RS----------------------------------E-------WADFNVD
XP_020885782.1/1-285 EH-------RS----------------------------------E-------WADFNVD
XP_006280004.1/1-285 EH-------RS----------------------------------E-------WADFNVD
XP_019088563.1/1-285 EH-------RS----------------------------------E-------WADFNVD
XP_010483719.1/1-285 EH-------RS----------------------------------E-------WADFNVD
XP_010456679.1/1-285 EH-------RS----------------------------------E-------WADFNVD
XP_006400821.1/1-284 EH-------RS----------------------------------E-------WADFNVD
NP_200877.1/1-284    EH-------RS----------------------------------E-------WADFNVD
XP_013611543.1/1-282 EH-------RS----------------------------------E-------WADFNVD
XP_009120605.1/1-284 EH-------RS----------------------------------E-------WADFNVD
XP_013667152.1/1-284 EH-------RS----------------------------------E-------WADFNVD
XP_018446535.1/1-284 EH-------RS----------------------------------E-------WADFNVD
XP_018454374.1/1-284 EH-------RS----------------------------------E-------WADFHVD
XP_009126588.1/1-285 EH-------RS----------------------------------E-------WADFHVD
XP_013680521.1/1-285 EH-------RS----------------------------------E-------WADFHVD
XP_013612427.1/1-285 EH-------RS----------------------------------E-------WADFHVD
XP_010535608.1/1-283 EH-------RS----------------------------------E-------WADFNVD
XP_010520372.1/1-276 EH-------RS----------------------------------E-------WADFSVD
XP_004487103.1/1-286 EH-------RS----------------------------------E-------WADFSVD
XP_003597252.1/1-283 EH-------RS----------------------------------E-------WADFSVD
XP_024455683.1/1-286 EH-------HA----------------------------------E-------WADFSVD
XP_022026352.1/1-288 EH-------RS----------------------------------E-------WADFNVD
XP_022000486.1/1-288 EH-------RS----------------------------------E-------WADFNVD
XP_016717477.1/1-281 EH-------RL----------------------------------E-------WADFNVD
XP_017643014.1/1-281 EH-------RL----------------------------------E-------WADFNVD
XP_016724213.1/1-281 EH-------RL----------------------------------E-------WADFNVD
XP_012462938.1/1-281 EH-------RL----------------------------------E-------WADFNVD
XP_012471509.1/1-292 EH-------RS----------------------------------E-------WADFNVD
XP_017642742.1/1-292 EH-------RS----------------------------------E-------WADFNVD
XP_016755876.1/1-292 EH-------RS----------------------------------E-------WADFNVD
XP_012471510.1/1-286 EH-------RS----------------------------------E-------WADFNVD
XP_016753711.1/1-286 EH-------RS----------------------------------E-------WADFNVD
XP_017642744.1/1-286 EH-------RS----------------------------------E-------WADFNVD
XP_016755877.1/1-286 EH-------RS----------------------------------E-------WADFNVD
XP_016750468.1/1-286 EH-------RS----------------------------------E-------WADFNVD
XP_016688657.1/1-286 EH-------RS----------------------------------E-------WADFNVD
XP_012443272.1/1-286 EH-------RS----------------------------------E-------WADFNVD
XP_017603001.1/1-286 EH-------RS----------------------------------E-------WADFNVD
XP_022755632.1/1-285 EH-------RS----------------------------------E-------WADFNVD
XP_022755633.1/1-285 EH-------RS----------------------------------E-------WADFNVD
XP_022729815.1/1-286 EH-------RS----------------------------------E-------WADFNVD
XP_022729816.1/1-286 EH-------RS----------------------------------E-------WADFNVD
XP_022752857.1/1-286 EH-------RS----------------------------------E-------WADFNVD
XP_022752856.1/1-286 EH-------RS----------------------------------E-------WADFNVD
XP_021277981.1/1-286 EH-------RS----------------------------------E-------WADFNVD
XP_017983554.1/1-286 EH-------RS----------------------------------E-------WADFNVD
XP_011089912.1/1-283 EH-------RS----------------------------------E-------WADFNVD
XP_007208090.1/1-286 EH-------RS----------------------------------E-------WADFNVD
XP_008225704.1/1-286 EH-------RS----------------------------------E-------WADFNVD
XP_021830853.1/1-286 EH-------RS----------------------------------E-------WADFNVD
XP_008349754.1/1-286 EH-------RS----------------------------------E-------WADFNVD
NP_001315783.1/1-288 EH-------RS----------------------------------E-------WADFNVD
XP_009367069.1/1-286 EH-------RS----------------------------------E-------WADFNVD
XP_008344872.1/1-286 EH-------RS----------------------------------E-------WADFNVD
XP_015952399.1/1-286 EH-------RS----------------------------------E-------WADFNVD
XP_020982118.1/1-286 EH-------RS----------------------------------E-------WADFNVD
XP_025685402.1/1-286 EH-------RS----------------------------------E-------WADFNVD
XP_016187430.1/1-286 EH-------RS----------------------------------E-------WADFNVD
XP_020214107.1/1-284 EH-------RS----------------------------------E-------WADFNVD
XP_006592258.1/1-284 EH-------RS----------------------------------E-------WADFSVD
XP_007132262.1/1-285 EH-------RS----------------------------------E-------WADFNVD
XP_003538150.1/1-285 EH-------RS----------------------------------E-------WADFNVD
XP_017433493.1/1-285 EH-------RS----------------------------------E-------WADFNVD
XP_014494018.1/1-285 EH-------RS----------------------------------E-------WADFNVD
XP_010089983.2/1-286 EH-------RS----------------------------------E-------WADFNVD
XP_006378974.2/1-285 EH-------RS----------------------------------E-------WADFSVD
XP_011010051.1/1-285 EH-------RS----------------------------------E-------WADFSVD
XP_011010050.1/1-285 EH-------RS----------------------------------E-------WADFSVD
XP_021905657.1/1-285 EH-------RS----------------------------------E-------WADFNVD
XP_021610391.1/1-286 EH-------RS----------------------------------E-------WADFNVD
XP_002529946.1/1-286 EH-------RS----------------------------------E-------WADFNVD
XP_021658728.1/1-286 EH-------RS----------------------------------E-------WADFNVD
XP_012064816.1/1-286 EH-------RS----------------------------------E-------WADFNVD
XP_021628343.1/1-286 EH-------RS----------------------------------E-------WADFNVD
XP_021657859.1/1-286 EH-------RS----------------------------------E-------WADFNVD
XP_015874442.1/1-285 EH-------RS----------------------------------E-------WADFNVD
XP_023926843.1/1-286 EH-------RS----------------------------------E-------WADFNVD
XP_018819529.1/1-286 EH-------RS----------------------------------E-------WADFNVD
XP_010651164.1/1-286 EH-------RS----------------------------------E-------WADFSVD
XP_024034672.1/1-286 EH-------RS----------------------------------E-------WADFNVD
XP_006488084.1/1-286 EH-------RS----------------------------------E-------WADFNVD
XP_024034673.1/1-286 EH-------RS----------------------------------E-------WADFNVD
XP_010264677.1/1-287 EH-------RS----------------------------------E-------WADFNVD
XP_010253680.1/1-288 EH-------RS----------------------------------E-------WADFSID
XP_022005829.1/1-282 EH-------RS----------------------------------E-------WADFNVD
XP_023751537.1/1-278 EH-------RS----------------------------------E-------WADFNVD
XP_024971452.1/1-283 EH-------RS----------------------------------E-------WADFNVD
XP_024971444.1/1-287 EH-------RS----------------------------------E-------WADFNVD
XP_024985647.1/1-287 EH-------RS----------------------------------E-------WADFNVD
XP_023749448.1/1-288 EH-------RS----------------------------------E-------WADFNVD
XP_010689538.1/1-281 EH-------RS----------------------------------E-------WADFSVD
XP_021858740.1/1-280 EH-------RS----------------------------------E-------WADFSVD
XP_021773317.1/1-281 EH-------RS----------------------------------E-------WADFSVD
XP_021745941.1/1-281 EH-------RS----------------------------------E-------WADFSVD
XP_016729039.1/1-286 EH-------RS----------------------------------E-------WADFNVD
XP_017623828.1/1-286 EH-------RS----------------------------------E-------WADFNVD
XP_012476406.1/1-286 EH-------RS----------------------------------E-------WADFNVD
XP_016754776.1/1-286 EH-------RS----------------------------------E-------WADFNVD
XP_022152944.1/1-286 EH-------RS----------------------------------E-------WADFNAD
XP_011040884.1/1-286 EH-------HA----------------------------------E-------WADFSVD
XP_010043753.1/1-286 EH-------RS----------------------------------E-------WADFNVD
XP_024158223.1/1-285 EH-------RS----------------------------------E-------WADFNVD
XP_004291573.1/1-285 EH-------RS----------------------------------E-------WADFNVD
XP_018808765.1/1-285 EH-------RS----------------------------------E-------WADFNVD
XP_023512455.1/1-283 EH-------RS----------------------------------E-------WADFNID
XP_023512456.1/1-283 EH-------RS----------------------------------E-------WADFNID
XP_022944186.1/1-283 EH-------RS----------------------------------E-------WADFNID
XP_022944185.1/1-283 EH-------RS----------------------------------E-------WADFNID
XP_022985614.1/1-283 EH-------RS----------------------------------E-------WADFNID
XP_022985615.1/1-283 EH-------RS----------------------------------E-------WADFNID
XP_011656996.1/1-283 EH-------RS----------------------------------E-------WADFNID
XP_011656997.1/1-283 EH-------RS----------------------------------E-------WADFNID
XP_008463945.1/1-283 EH-------RS----------------------------------E-------WADFNID
XP_008463946.1/1-283 EH-------RS----------------------------------E-------WADFNID
XP_022140363.1/1-286 EH-------RS----------------------------------E-------WADFNID
XP_022995706.1/1-286 EH-------RS----------------------------------E-------WADFNID
XP_023534373.1/1-286 EH-------RS----------------------------------E-------WADFNID
XP_022958713.1/1-286 EH-------RS----------------------------------E-------WADFNID
XP_025614062.1/1-285 EH-------RS----------------------------------E-------WADFNVD
XP_025614063.1/1-285 EH-------RS----------------------------------E-------WADFNVD
XP_016170485.1/1-285 EH-------RS----------------------------------E-------WADFNVD
XP_016170486.1/1-285 EH-------RS----------------------------------E-------WADFNVD
XP_015935757.1/1-285 EH-------RS----------------------------------E-------WADFNVD
XP_015935758.1/1-285 EH-------RS----------------------------------E-------WADFNVD
XP_004505999.1/1-286 EH-------RS----------------------------------E-------WADFNVD
XP_013456006.1/1-286 EH-------RS----------------------------------E-------WADFNVD
XP_019453966.1/1-285 EH-------RS----------------------------------E-------WADFNVD
XP_019448740.1/1-279 EH-------RS----------------------------------E-------WADFNVD
XP_019448741.1/1-279 EH-------RS----------------------------------E-------WADFNVD
XP_019458095.1/1-286 EH-------RS----------------------------------E-------WADFNVD
XP_019447870.1/1-286 EH-------RS----------------------------------E-------WADFNID
XP_012838629.1/1-280 EH-------RS----------------------------------E-------WADFNID
XP_012838630.1/1-280 EH-------RS----------------------------------E-------WADFNID
XP_012838627.1/1-280 EH-------RS----------------------------------E-------WADFNID
XP_012838628.1/1-280 EH-------RS----------------------------------E-------WADFNID
XP_017214964.1/1-286 EH-------RS----------------------------------E-------WADFSID
XP_017234817.1/1-286 EH-------RS----------------------------------E-------WADFNID
XP_019178153.1/1-282 EH-------RS----------------------------------E-------WADFNVD
XP_019166562.1/1-283 EH-------RS----------------------------------E-------WADFNVD
XP_011089294.1/1-282 EH-------RS----------------------------------E-------WADFNVD
XP_022892122.1/1-274 EH-------RS----------------------------------E-------WADFNVD
XP_022883393.1/1-279 EH-------RS----------------------------------E-------WADFTVD
XP_022883398.1/1-279 EH-------RS----------------------------------E-------WADFTVD
XP_022883384.1/1-283 EH-------RS----------------------------------E-------WADFTVD
XP_022883389.1/1-283 EH-------RS----------------------------------E-------WADFTVD
XP_009776589.1/1-283 EH-------RS----------------------------------E-------WADFNVD
NP_001312504.1/1-283 EH-------RS----------------------------------E-------WADFNVD
XP_016472209.1/1-283 EH-------RS----------------------------------E-------WADFNVD
XP_019255429.1/1-283 EH-------RS----------------------------------E-------WADFNVD
XP_009624234.1/1-283 EH-------RS----------------------------------E-------WADFNVD
XP_016504967.1/1-283 EH-------RS----------------------------------E-------WADFNVD
XP_016547131.1/1-283 EH-------RS----------------------------------E-------WADFNVD
XP_004251138.1/1-283 EH-------RS----------------------------------E-------WADFNVD
XP_015058512.1/1-283 EH-------RS----------------------------------E-------WADFNVD
XP_006340193.1/1-283 EH-------RS----------------------------------E-------WADFNVD
XP_004235650.1/1-283 EH-------RS----------------------------------E-------WADNNID
XP_006343047.1/1-283 EH-------RS----------------------------------E-------WADNNID
XP_009774805.1/1-285 EH-------RS----------------------------------E-------WADNNID
XP_016498616.1/1-285 EH-------RS----------------------------------E-------WADNNID
XP_016434103.1/1-285 EH-------RS----------------------------------E-------WADNNID
XP_009593967.1/1-285 EH-------RS----------------------------------E-------WADNNID
XP_004510297.1/1-285 EH-------RS----------------------------------E-------WADTNMD
XP_004510296.1/1-285 EH-------RS----------------------------------E-------WADTNMD
XP_003627005.1/1-284 EH-------RS----------------------------------E-------WADHNMD
XP_003531653.1/1-284 EH-------RS----------------------------------E-------WADNNMD
XP_006585576.1/1-288 EH-------RS----------------------------------E-------WADNNMD
XP_014634618.1/1-288 EH-------RS----------------------------------E-------WADNNMD
XP_014634619.1/1-288 EH-------RS----------------------------------E-------WADNNMD
XP_003531652.1/1-285 EH-------RS----------------------------------E-------WADNNMD
XP_003530109.1/1-284 EH-------RS----------------------------------E-------WADNNMD
XP_007135643.1/1-285 EH-------RS----------------------------------E-------WADNNMD
XP_003530112.1/1-285 EH-------RS----------------------------------E-------WADNNMD
XP_020226142.1/1-285 EH-------RS----------------------------------E-------WADNNMD
XP_014516466.1/1-285 EH-------RS----------------------------------E-------WADNNMD
XP_017407594.1/1-285 EH-------RS----------------------------------E-------WADNNMD
XP_019448178.1/1-285 EH-------RS----------------------------------E-------WADNNMD
XP_019429328.1/1-285 EH-------RS----------------------------------E-------WADNNMD
XP_004486776.1/1-285 EH-------RS----------------------------------E-------WADNNMD
XP_003597690.1/1-286 EH-------RS----------------------------------E-------WADNNMD
XP_015932679.1/1-285 EH-------RS----------------------------------E-------WADNNMD
XP_025674177.1/1-285 EH-------RS----------------------------------E-------WADNNMD
XP_025610204.1/1-285 EH-------RS----------------------------------E-------WADNNMD
XP_020226055.1/1-285 EH-------RS----------------------------------E-------WADNNMD
XP_007150614.1/1-285 EH-------RS----------------------------------E-------WADNSMD
XP_014501306.1/1-285 EH-------RS----------------------------------E-------WADNNMD
XP_017425270.1/1-285 EH-------RS----------------------------------E-------WADNNMD
XP_021836126.1/1-285 EH-------RS----------------------------------E-------WADNNID
XP_010691423.1/1-284 EH-------RS----------------------------------E-------WADNNID
XP_010691424.1/1-284 EH-------RS----------------------------------E-------WADNNID
XP_021765330.1/1-284 EH-------RS----------------------------------E-------WADNNID
XP_021749885.1/1-284 EH-------RS----------------------------------E-------WADNNID
XP_010267183.1/1-285 EH-------RS----------------------------------E-------WADSNID
XP_017258627.1/1-285 EH-------RS----------------------------------E-------WADTNID
XP_023000315.1/1-284 EH-------RS----------------------------------E-------WADNNID
XP_023000313.1/1-284 EH-------RS----------------------------------E-------WADNNID
XP_023514554.1/1-284 EH-------RS----------------------------------E-------WADNNID
XP_023514552.1/1-284 EH-------RS----------------------------------E-------WADNNID
XP_022964458.1/1-284 EH-------RS----------------------------------E-------WADNNID
XP_022964454.1/1-284 EH-------RS----------------------------------E-------WADNNID
XP_022147627.1/1-284 EH-------RS----------------------------------E-------WADNNID
XP_022147619.1/1-284 EH-------RS----------------------------------E-------WADNNID
XP_008462437.1/1-284 EH-------RS----------------------------------E-------WADNNID
XP_004141659.1/1-284 EH-------RS----------------------------------E-------WADNNID
XP_008462436.1/1-284 EH-------RS----------------------------------E-------WADNNID
XP_011659682.1/1-284 EH-------RS----------------------------------E-------WADNNID
XP_022992111.1/1-284 EH-------RS----------------------------------E-------WADNNID
XP_023548534.1/1-284 EH-------RS----------------------------------E-------WADNNID
XP_022953371.1/1-284 EH-------RS----------------------------------E-------WADNNID
XP_002304217.1/1-285 EH-------RS----------------------------------E-------WADNNID
XP_011013041.1/1-285 EH-------RS----------------------------------E-------WADNNID
XP_011013042.1/1-285 EH-------RS----------------------------------E-------WADNNID
XP_011046759.1/1-284 EH-------RS----------------------------------E-------WADNNID
XP_024464085.1/1-284 EH-------RS----------------------------------E-------WADNNID
XP_024183498.1/1-286 EH-------RS----------------------------------E-------WADNNID
XP_004302866.1/1-286 EH-------RS----------------------------------E-------WADNNID
NP_001280898.1/1-285 EH-------RS----------------------------------E-------WADNNID
XP_007214960.1/1-285 EH-------RS----------------------------------E-------WADNNID
XP_008228437.1/1-285 EH-------RS----------------------------------E-------WADNNID
XP_021818763.1/1-285 EH-------RS----------------------------------E-------WADNNID
XP_022723515.1/1-285 EH-------RS----------------------------------E-------WADSSID
XP_016688508.1/1-285 EH-------RS----------------------------------E-------WADSSID
XP_012443274.1/1-285 EH-------RS----------------------------------E-------WADSSID
XP_022721457.1/1-285 EH-------RS----------------------------------E-------WADSSID
XP_022715018.1/1-285 EH-------RS----------------------------------E-------WADSSID
XP_017979082.1/1-285 EH-------RS----------------------------------E-------WADSSID
XP_021293768.1/1-285 EH-------RS----------------------------------E-------WADSSID
XP_010062518.1/1-285 EH-------RS----------------------------------E-------WADNSID
XP_006426893.1/1-285 EH-------RS----------------------------------E-------WADNNID
XP_021610318.1/1-285 EH-------RS----------------------------------E-------WADNNID
XP_010098130.1/1-285 EH-------RS----------------------------------E-------WADNNID
XP_021635282.1/1-296 EH-------RS----------------------------------E-------WADNNID
XP_002284003.2/1-285 EH-------RS----------------------------------E-------WADNNID
XP_002515977.1/1-285 EH-------RS----------------------------------E-------WADNNID
XP_021643260.1/1-285 EH-------RS----------------------------------E-------WADNNID
XP_021628194.1/1-285 EH-------RS----------------------------------E-------WADNNID
XP_015894591.1/1-285 EH-------RS----------------------------------E-------WADNNID
XP_012069098.1/1-285 EH-------RS----------------------------------E-------WADNNID
XP_018847857.1/1-285 EH-------RS----------------------------------E-------WADNNID
XP_023898511.1/1-285 EH-------RS----------------------------------E-------WADNNID
XP_010545673.1/1-285 EH-------RS----------------------------------E-------WADNSID
XP_010545670.1/1-286 EH-------RS----------------------------------E-------WADNSID
XP_010535179.1/1-285 EH-------RS----------------------------------E-------WVDNSID
XP_018436929.1/1-283 EH-------RS----------------------------------E-------WADNNID
XP_013592012.1/1-283 EH-------RS----------------------------------E-------WADNNID
XP_013640375.1/1-284 EH-------RS----------------------------------E-------WADNNID
XP_013640376.1/1-283 EH-------RS----------------------------------E-------WADNNID
XP_009147709.1/1-283 EH-------RS----------------------------------E-------WADNNID
XP_006306297.1/1-281 EH-------RS----------------------------------E-------WADNNID
XP_010462051.1/1-281 EH-------RS----------------------------------E-------WADNNID
XP_010479714.1/1-281 EH-------RS----------------------------------E-------WADNNID
XP_010462052.1/1-281 EH-------RS----------------------------------E-------WADNNID
XP_010500807.1/1-281 EH-------RS----------------------------------E-------WADNNID
NP_849795.1/1-282    EH-------RS----------------------------------E-------WADNNID
NP_001077702.1/1-281 EH-------RS----------------------------------E-------WADNNID
NP_175627.1/1-281    EH-------RS----------------------------------E-------WADNNID
XP_020868586.1/1-281 EH-------RS----------------------------------E-------WADNNID
XP_006392953.1/1-282 EH-------RS----------------------------------E-------WADNNID
XP_018460807.1/1-282 EH-------RS----------------------------------E-------WADNNID
XP_013621760.1/1-283 EH-------RS----------------------------------E-------WADNNIN
XP_018451524.1/1-282 EH-------RS----------------------------------E-------WADNNID
XP_009107148.1/1-282 EH-------RS----------------------------------E-------WADNNID
XP_009107147.1/1-283 EH-------RS----------------------------------E-------WADNNID
XP_009600774.1/1-284 EH-------RS----------------------------------E-------WVDSDID
XP_016459079.1/1-284 EH-------RS----------------------------------E-------WVDSDID
XP_019236029.1/1-285 EH-------RS----------------------------------E-------WVDNDID
XP_009771098.1/1-285 EH-------RS----------------------------------E-------WVDNDID
XP_016550962.1/1-285 EH-------RS----------------------------------E-------WVDNNID
XP_016550960.1/1-285 EH-------RS----------------------------------E-------WVDNNID
XP_016550961.1/1-285 EH-------RS----------------------------------E-------WVDNNID
XP_006359469.1/1-285 EH-------RS----------------------------------E-------WVDNNID
XP_004252354.1/1-285 EH-------RS----------------------------------E-------WVDNNID
XP_015061298.1/1-285 EH-------RS----------------------------------E-------WVDNNID
XP_019198775.1/1-285 EH-------RS----------------------------------E-------WAENNID
XP_019194412.1/1-285 EH-------RS----------------------------------E-------WADNNIE
XP_016562656.1/1-283 EH-------RS----------------------------------E-------WADNNID
XP_019267274.1/1-285 EH-------RS----------------------------------E-------WADNNID
XP_019267275.1/1-282 EH-------RS----------------------------------E-------WADNNID
XP_022889649.1/1-285 EH-------RS----------------------------------E-------WADNNID
XP_022889648.1/1-285 EH-------RS----------------------------------E-------WADNNID
XP_012852689.1/1-281 EH-------RS----------------------------------E-------WADN-FD
XP_012856468.1/1-279 EH-------RS----------------------------------E-------WADTSID
XP_011080516.1/1-285 EH-------RS----------------------------------E-------WADSSID
XP_011072422.1/1-285 EH-------RS----------------------------------E-------WADNNID
XP_022851148.1/1-285 EH-------RS----------------------------------E-------WADNNVD
XP_022844668.1/1-285 EH-------RS----------------------------------E-------WADNNVD
XP_006853643.2/1-288 EH-------RS----------------------------------E-------WADSNID
XP_010249545.1/1-285 EH-------RS----------------------------------E-------WADSNID
XP_010249539.1/1-285 EH-------RS----------------------------------E-------WADSNID
XP_010915409.1/1-284 EH-------RS----------------------------------E-------WADSNID
XP_008791633.1/1-284 EH-------RS----------------------------------E-------WADSNID
XP_017235966.1/1-285 EH-------RS----------------------------------E-------WADCNVD
XP_023761873.1/1-288 EH-------RS----------------------------------E-------WADNNVD
XP_021997125.1/1-286 EH-------RS----------------------------------E-------WADNNVD
XP_021990052.1/1-285 EH-------RS----------------------------------E-------WADNNVD
XP_024979339.1/1-285 EH-------RS----------------------------------E-------WADNNVD
XP_024979341.1/1-290 EH-------RS----------------------------------E-------WADNNVD
XP_024979340.1/1-291 EH-------RS----------------------------------E-------WADNNVD
XP_024979342.1/1-291 EH-------RS----------------------------------E-------WADNNVD
XP_022016208.1/1-285 EH-------RS----------------------------------E-------WADSSID
XP_022016207.1/1-311 EH-------RS----------------------------------E-------WADSSID
XP_022009819.1/1-285 EH-------RS----------------------------------E-------WADYNID
XP_022009815.1/1-285 EH-------RS----------------------------------E-------WADYNID
XP_022009809.1/1-285 EH-------RS----------------------------------E-------WADYNID
XP_023759500.1/1-286 EH-------RS----------------------------------E-------WADNNID
XP_023751740.1/1-285 EH-------RS----------------------------------E-------WADNNFD
XP_024971803.1/1-285 EH-------RS----------------------------------E-------WADNNID
XP_022033649.1/1-285 EH-------RS----------------------------------E-------WADNNMD
XP_024972195.1/1-285 EH-------RS----------------------------------E-------WADNSID
XP_024972196.1/1-289 EH-------RS----------------------------------E-------WADNSID
XP_020248539.1/1-284 EH-------RS----------------------------------E-------WADSSID
XP_020269197.1/1-284 EH-------RS----------------------------------E-------WADSSID
XP_020269196.1/1-284 EH-------RS----------------------------------E-------WADSSID
XP_009399813.1/1-284 EH-------RS----------------------------------E-------WADSNID
XP_009399812.1/1-284 EH-------RS----------------------------------E-------WADSNID
XP_009413853.1/1-284 EH-------RS----------------------------------E-------WADRNID
XP_024985513.1/1-286 EH-------RS----------------------------------E-------WADSSID
XP_023729871.1/1-286 EH-------RS----------------------------------E-------WADSSID
XP_022000974.1/1-282 EH-------RS----------------------------------E-------WADSSID
XP_022023495.1/1-282 EH-------RS----------------------------------E-------WADSSID
XP_010526102.1/1-286 EH-------RQ----------------------------------E-------WADNSID
XP_019086035.1/1-282 EH-------RQ----------------------------------E-------WADNSID
XP_010437837.1/1-288 EH-------RQ----------------------------------E-------WADNSID
XP_010437838.1/1-288 EH-------RQ----------------------------------E-------WADNSID
XP_022549566.1/1-283 EH-------RQ----------------------------------E-------WADNSID
XP_013670651.1/1-283 EH-------RQ----------------------------------E-------WADNSID
XP_020874560.1/1-283 EH-------RQ----------------------------------E-------WADNSID
XP_020874561.1/1-283 EH-------RQ----------------------------------E-------WADNSID
XP_020874562.1/1-283 EH-------RQ----------------------------------E-------WADNSID
NP_195014.1/1-283    EH-------RQ----------------------------------E-------WADNSID
XP_006412419.1/1-283 EH-------RQ----------------------------------E-------WADNSID
XP_006412418.1/1-283 EH-------RQ----------------------------------E-------WADNSID
XP_018471819.1/1-283 EH-------RQ----------------------------------E-------WADNSID
XP_009125299.1/1-283 EH-------RQ----------------------------------E-------WADNSID
XP_010432643.1/1-283 EH-------RQ----------------------------------E-------WADNSID
XP_010432644.1/1-283 EH-------RQ----------------------------------E-------WADNSID
XP_006283109.1/1-283 EH-------RQ----------------------------------E-------WADNSID
XP_010437839.1/1-283 EH-------RQ----------------------------------E-------WADNSID
XP_010437840.1/1-283 EH-------RQ----------------------------------E-------WADNSID
XP_010447325.1/1-283 EH-------RQ----------------------------------E-------WADNSID
XP_010447326.1/1-283 EH-------RQ----------------------------------E-------WADNSID
XP_013622315.1/1-283 EH-------RQ----------------------------------E-------WADNSID
XP_013656273.1/1-283 EH-------RQ----------------------------------E-------WADNSID
XP_009108824.1/1-283 EH-------RQ----------------------------------E-------WADNSID
XP_013730326.1/1-283 EH-------RQ----------------------------------E-------WADNSID
XP_013730325.1/1-283 EH-------RQ----------------------------------E-------WADNSID
XP_018447492.1/1-283 EH-------RQ----------------------------------E-------WADNSID
XP_019415259.1/1-283 EH-------RS----------------------------------E-------WADSRID
XP_022980940.1/1-286 EH-------RS----------------------------------E-------WADTSID
XP_023523539.1/1-286 EH-------RS----------------------------------E-------WADTSID
XP_022941184.1/1-286 EH-------RS----------------------------------E-------WADTSID
XP_022134366.1/1-286 EH-------RS----------------------------------E-------WADSSID
XP_004136224.1/1-286 EH-------RS----------------------------------E-------WADSSID
XP_008466070.1/1-286 EH-------RS----------------------------------E-------WADSSID
XP_022976315.1/1-286 EH-------RS----------------------------------E-------WADSSID
XP_022937345.1/1-286 EH-------RS----------------------------------E-------WADSSID
XP_023534956.1/1-286 EH-------RS----------------------------------E-------WADSSID
XP_023535295.1/1-286 EH-------RS----------------------------------E-------WADSSID
XP_019175908.1/1-285 EH-------RS----------------------------------E-------WADSSID
XP_019175907.1/1-285 EH-------RS----------------------------------E-------WADSSID
XP_009626659.1/1-285 EH-------RS----------------------------------E-------WADSGID
XP_019266312.1/1-285 EH-------RS----------------------------------E-------WADSGID
XP_009781184.1/1-285 EH-------RS----------------------------------E-------WADSGID
XP_016488110.1/1-285 EH-------RS----------------------------------E-------WADSGID
XP_016537761.1/1-285 EH-------RS----------------------------------E-------WADSGID
XP_006359741.1/1-285 EH-------RS----------------------------------E-------WADSGID
XP_010325078.1/1-288 EH-------RS----------------------------------E-------WADSGID
XP_004245155.1/1-285 EH-------RS----------------------------------E-------WADSGID
XP_015085523.1/1-285 EH-------RS----------------------------------E-------WADSGID
XP_022868182.1/1-285 EH-------RS----------------------------------E-------WADNGID
XP_022868181.1/1-285 EH-------RS----------------------------------E-------WADNGID
XP_022854559.1/1-285 EH-------RS----------------------------------E-------WADNGID
XP_011081776.1/1-285 EH-------RS----------------------------------E-------WADSGID
XP_012857948.1/1-282 EH-------RS----------------------------------E-------WADSGVD
XP_021847759.1/1-284 EH-------RS----------------------------------E-------WADSGID
XP_010668539.1/1-284 EH-------RS----------------------------------E-------WADSGID
XP_021739137.1/1-284 EH-------RS----------------------------------E-------WADSGID
XP_021737291.1/1-284 EH-------RS----------------------------------E-------WADSGID
XP_010047286.1/1-285 EH-------RS----------------------------------E-------WADSSID
XP_003603630.2/1-285 EH-------RS----------------------------------E-------WADTSID
XP_004501146.1/1-286 EH-------RS----------------------------------E-------WADTSID
XP_012571693.1/1-286 EH-------RS----------------------------------E-------WADTSID
XP_012571692.1/1-286 EH-------RS----------------------------------E-------WADTSID
XP_019417530.1/1-285 EH-------RS----------------------------------E-------WADSSID
XP_019417531.1/1-285 EH-------RS----------------------------------E-------WADSSID
XP_016180268.1/1-286 EH-------RS----------------------------------E-------WADSSID
XP_025623723.1/1-286 EH-------RS----------------------------------E-------WADSSID
XP_015945672.1/1-286 EH-------RS----------------------------------E-------WADSSID
XP_007137231.1/1-287 EH-------RS----------------------------------E-------WADSSID
XP_022636494.1/1-293 EH-------RS----------------------------------E-------WADSSID
XP_017409217.1/1-287 EH-------RS----------------------------------E-------WADSSID
XP_014501941.1/1-287 EH-------RS----------------------------------E-------WADSSID
XP_003526496.1/1-286 EH-------RS----------------------------------E-------WADSSID
XP_003522716.1/1-286 EH-------RS----------------------------------E-------WADSSID
XP_020217689.1/1-286 EH-------RS----------------------------------E-------WADSSID
XP_016683069.1/1-283 EH-------RS----------------------------------E-------WADSGID
XP_016677926.1/1-283 EH-------RS----------------------------------E-------WADSGID
XP_017643110.1/1-283 EH-------RS----------------------------------E-------WADSGID
XP_017643111.1/1-283 EH-------RS----------------------------------E-------WADSGID
XP_012451775.1/1-283 EH-------RS----------------------------------E-------WADSGID
XP_016744766.1/1-285 EH-------RS----------------------------------E-------WADSGID
XP_017643921.1/1-285 EH-------RS----------------------------------E-------WADSGID
XP_012436119.1/1-285 EH-------RS----------------------------------E-------WADSGID
XP_016681632.1/1-285 EH-------RS----------------------------------E-------WADSGID
XP_016689618.1/1-285 EH-------RA----------------------------------E-------WADSGID
XP_016689617.1/1-285 EH-------RA----------------------------------E-------WADSGID
XP_012445947.1/1-285 EH-------RS----------------------------------E-------WADSGID
XP_012445944.1/1-285 EH-------RS----------------------------------E-------WADSGID
XP_012445945.1/1-285 EH-------RS----------------------------------E-------WADSGID
XP_017614652.1/1-285 EH-------RS----------------------------------E-------WADSGID
XP_016732732.1/1-285 EH-------RS----------------------------------E-------WADSGID
XP_016732731.1/1-285 EH-------RS----------------------------------E-------WADSGID
XP_022754596.1/1-285 EH-------RS----------------------------------E-------WADSGID
XP_022750159.1/1-285 EH-------RS----------------------------------E-------WADSGID
XP_022729737.1/1-285 EH-------RS----------------------------------E-------WADSGID
XP_022729738.1/1-285 EH-------RS----------------------------------E-------WADSGID
XP_021276132.1/1-285 EH-------RS----------------------------------E-------WADSGID
XP_017983822.1/1-285 EH-------RS----------------------------------E-------WADSGID
XP_002283717.3/1-285 EH-------RS----------------------------------E-------WADSSID
XP_024446213.1/1-284 EH-------RS----------------------------------E-------WADNGID
XP_024446215.1/1-284 EH-------RS----------------------------------E-------WADNGID
XP_011006428.1/1-284 EH-------RS----------------------------------E-------WADNGID
XP_024459734.1/1-284 EH-------RS----------------------------------E-------WADSGID
XP_011019595.1/1-284 EH-------RS----------------------------------E-------WADSGID
XP_015573174.1/1-284 EH-------RS----------------------------------E-------WADTGID
XP_012077376.1/1-284 EH-------RS----------------------------------E-------WADSGID
XP_021608403.1/1-284 EH-------RS----------------------------------E-------WADSGID
XP_021608402.1/1-284 EH-------RS----------------------------------E-------WADSGID
XP_021597909.1/1-284 EH-------RS----------------------------------E-------WADSGID
XP_021642839.1/1-284 EH-------RS----------------------------------E-------WADSGID
XP_021689146.1/1-284 EH-------RS----------------------------------E-------WADSGID
XP_018805752.1/1-285 EH-------RS----------------------------------E-------WADSSID
XP_023918167.1/1-285 EH-------RS----------------------------------E-------WADSSID
XP_018845499.1/1-285 EH-------RS----------------------------------E-------WADSSID
XP_018845500.1/1-285 EH-------RS----------------------------------E-------WADSSID
NP_001280750.1/1-285 EH-------RS----------------------------------E-------WADRSID
NP_001280796.1/1-285 EH-------RS----------------------------------E-------WADRSID
XP_009367440.1/1-285 EH-------RS----------------------------------E-------WADRSID
XP_017182108.1/1-285 EH-------RS----------------------------------E-------WADRSID
XP_004291167.1/1-285 EH-------RS----------------------------------E-------WADRSID
XP_024162655.1/1-285 EH-------RS----------------------------------E-------WADRSID
XP_021819449.1/1-284 EH-------RS----------------------------------E-------WADRSID
XP_007225281.1/1-285 EH-------RS----------------------------------E-------WADRSID
XP_008220092.1/1-285 EH-------RS----------------------------------E-------WADRSID
XP_006450706.1/1-285 EH-------RS----------------------------------E-------WADSSID
XP_006476026.1/1-285 EH-------RS----------------------------------E-------WADSSID
XP_024930942.1/1-285 EH-------RS----------------------------------E-------WADSSID
XP_010102406.2/1-285 EH-------RS----------------------------------E-------WADSNID
XP_024540686.1/1-258 DH-------RS----------------------------------E-------WGCN-MD
XP_024399220.1/1-283 EH-------RS----------------------------------E-------WADHEID
XP_024399219.1/1-283 EH-------RS----------------------------------E-------WADHEID
XP_024399215.1/1-283 EH-------RS----------------------------------E-------WADHEID
XP_024390746.1/1-284 EH-------RS----------------------------------E-------WADYDID
XP_024390752.1/1-284 EH-------RS----------------------------------E-------WADYDID
XP_024373354.1/1-283 EH-------RS----------------------------------E-------WADYDMD
XP_024373357.1/1-283 EH-------RS----------------------------------E-------WADYDMD
XP_024370817.1/1-284 EH-------RS----------------------------------E-------WADFDID
XP_024370815.1/1-284 EH-------RS----------------------------------E-------WADFDID
XP_024370814.1/1-284 EH-------RS----------------------------------E-------WADFDID
XP_024386785.1/1-285 EH-------RS----------------------------------E-------WADYDMD
XP_024386782.1/1-285 EH-------RS----------------------------------E-------WADYDMD
XP_024386791.1/1-285 EH-------RS----------------------------------E-------WADYDMD
XP_002961227.1/1-277 EH-------RS----------------------------------E-------WADCNVV
XP_002969458.1/1-277 EH-------RS----------------------------------E-------WADCNVV
XP_024533017.1/1-265 EH-------RS----------------------------------E-------WAGVNIA
XP_011076983.1/1-261 ER-------RS----------------------------------C-------WMNINLA
XP_011076980.1/1-264 ER-------RS----------------------------------C-------WMNINLA
XP_011076981.1/1-263 ER-------RS----------------------------------C-------WMNINLA
XP_011084330.1/1-264 EH-------RS----------------------------------A-------WMDFNFA
XP_011084333.1/1-264 EH-------RS----------------------------------A-------WMDFNFA
XP_011084331.1/1-264 EH-------RS----------------------------------A-------WMDFNFA
XP_012834933.1/1-260 ER-------RS----------------------------------A-------WMDFDFS
XP_012834932.1/1-260 ER-------RS----------------------------------A-------WMDFDFS
XP_012834931.1/1-260 ER-------RS----------------------------------A-------WMDFDFS
XP_012834934.1/1-260 ER-------RS----------------------------------A-------WMDFDFS
XP_002274194.1/1-284 EH-------RS----------------------------------E-------WADFSVD
XP_019079956.1/1-284 EH-------RS----------------------------------E-------WADFSVD
XP_023759583.1/1-283 EY-------RS----------------------------------E-------WADSSMD
XP_020087984.1/1-275 EH-------RS----------------------------------E-------WGDSNMD
XP_015693848.1/1-289 EH-------RS----------------------------------Q-------WADSNLD
XP_015624690.1/1-290 EH-------RS----------------------------------Q-------WADSNLD
XP_008655743.1/1-292 EQ-------RS----------------------------------Q-------WADSNLD
XP_020183693.1/1-281 EH-------RS----------------------------------Q-------WADSSLD
XP_020183687.1/1-281 EH-------RS----------------------------------Q-------WADSSLD
XP_003565084.1/1-284 EH-------RS----------------------------------Q-------WADSTLD
XP_002454995.1/1-286 EQ-------RS----------------------------------Q-------WADSNLD
XP_012701181.1/1-286 EQ-------RS----------------------------------Q-------WADSNLD
XP_012701180.1/1-286 EQ-------RS----------------------------------Q-------WADSNLD
XP_012701179.1/1-286 EQ-------RS----------------------------------Q-------WADSNLD
XP_025816436.1/1-287 EQ-------RS----------------------------------Q-------WADSNLD
XP_018726848.1/1-227 DMNS-----RS-----------------------------------------EWDILSN-
XP_018726849.1/1-227 DMNS-----RS-----------------------------------------EWDILSN-
XP_019193052.1/1-216 SEHS-----RS-----------------------------------------EWDILSN-
XP_019193050.1/1-216 SEHS-----RS-----------------------------------------EWDILSN-
XP_021834047.1/1-194 EITC-----TF-----------------------------------------QWDILSN-
XP_010522330.1/1-229 DENS-----RN-----------------------------------------EWDILSN-
XP_009802524.1/1-219 DVNT-----RS-----------------------------------------EWDILSN-
XP_009802526.1/1-219 DVNT-----RS-----------------------------------------EWDILSN-
XP_009802525.1/1-219 DVNT-----RS-----------------------------------------EWDILSN-
XP_009802527.1/1-219 DVNT-----RS-----------------------------------------EWDILSN-
XP_019250148.1/1-219 DENT-----RT-----------------------------------------EWDILSN-
XP_019250149.1/1-219 DENT-----RT-----------------------------------------EWDILSN-
XP_016449923.1/1-219 DENT-----RS-----------------------------------------EWDILSN-
XP_009629760.1/1-219 DENT-----RS-----------------------------------------EWDILSN-
XP_009629762.1/1-219 DENT-----RS-----------------------------------------EWDILSN-
XP_010234287.2/1-230 DDST-----RS-----------------------------------------EWDILSN-
XP_021806177.1/1-204 DENS-----RN-----------------------------------------EWDILSN-
XP_016580642.1/1-229 DESR-----RT-----------------------------------------EWDILSN-
XP_008667763.1/1-266 DESS-----RSEARTRAPTHTQFFFNREHVLPS------YEPPFLFFFFLFLQWDILSN-
XP_006352040.1/1-227 DENT-----RT-----------------------------------------EWDILSN-
XP_009611638.1/1-223 DENT-----RS-----------------------------------------EWDILSN-
XP_009611623.1/1-223 DENT-----RS-----------------------------------------EWDILSN-
XP_016510654.1/1-223 DENT-----RS-----------------------------------------EWDILSN-
XP_009766038.1/1-223 DENT-----RS-----------------------------------------EWDILSN-
XP_020210648.1/1-236 DEKA-----RN-----------------------------------------RWDILSN-
XP_007143210.1/1-219 SENS-----RN-----------------------------------------QWDILSN-
XP_014491869.1/1-226 SENS-----RN-----------------------------------------QWDILSN-
XP_022633652.1/1-226 SENS-----RN-----------------------------------------QWDILSN-
XP_017412341.1/1-227 SENS-----RN-----------------------------------------QWDILSN-
XP_022011155.1/1-226 DENT-----RG-----------------------------------------EWDPLLN-
XP_003555330.2/1-228 SENT-----RN-----------------------------------------QWDILST-
XP_014628518.1/1-229 SENT-----RNQ----------------------------------------VWDILST-
XP_017647009.1/1-225 SEKF-----RS-----------------------------------------EWDILSN-
XP_016711277.1/1-225 SEKF-----RS-----------------------------------------EWDILSN-
XP_012457027.1/1-225 SEKF-----RS-----------------------------------------EWDILSN-
XP_016723454.1/1-225 SEKF-----RS-----------------------------------------EWDILSN-
XP_020978973.1/1-214 DQNS-----RS-----------------------------------------QWDILSN-
XP_016202355.1/1-214 DQNS-----RS-----------------------------------------QWDILSN-
XP_015964876.1/1-214 DQNS-----RS-----------------------------------------QWDILSN-
XP_025699645.1/1-214 DQNS-----RS-----------------------------------------QWDILSN-
XP_008441375.1/1-228 DQNT-----RN-----------------------------------------QWDILSN-
XP_004154226.2/1-228 DQNT-----RN-----------------------------------------QWDILSN-
XP_023519981.1/1-226 DQNT-----RS-----------------------------------------QWDVLSN-
XP_023001231.1/1-226 DQNT-----RS-----------------------------------------QWDVLSN-
XP_022933178.1/1-226 DQNT-----RS-----------------------------------------QWDVLSN-
XP_004511340.1/1-228 NQNS-----RS-----------------------------------------QWDILSS-
XP_002979015.2/1-232 DERL-----RN-----------------------------------------EWDILSN-
XP_002988402.1/1-232 DERL-----RN-----------------------------------------EWDILSN-
XP_024390340.1/1-238 DERL-----RS-----------------------------------------EWDILSN-
XP_024368074.1/1-235 DERL-----RS-----------------------------------------EWDILSN-
XP_024401280.1/1-235 DERL-----RS-----------------------------------------EWDILSN-
XP_024394840.1/1-235 DERL-----RS-----------------------------------------EWDILSN-
XP_024394838.1/1-235 DERL-----RS-----------------------------------------EWDILSN-
XP_017256650.1/1-227 DENS-----RN-----------------------------------------QWDILSS-
XP_019186546.1/1-227 DQNS-----RR-----------------------------------------EWDILSN-
XP_024988375.1/1-226 DENS-----RI-----------------------------------------EWDILSN-
XP_017231769.1/1-226 DENT-----RN-----------------------------------------EWDILSN-
XP_009108529.1/1-235 DENS-----RS-----------------------------------------QWDILSN-
XP_018469534.1/1-236 DENS-----RS-----------------------------------------EWDILSN-
XP_018467466.1/1-236 DENS-----RS-----------------------------------------EWDILSN-
XP_022543253.1/1-234 DENS-----RS-----------------------------------------EWDILSN-
XP_009135181.1/1-234 DENS-----RS-----------------------------------------EWDILSN-
XP_013642975.1/1-234 DENS-----RS-----------------------------------------EWDILSN-
XP_009135189.1/1-234 DENS-----RS-----------------------------------------EWDILSN-
XP_018515487.1/1-234 DENS-----RS-----------------------------------------EWDILSN-
XP_013623861.1/1-234 DENS-----RS-----------------------------------------EWDILSN-
XP_013623905.1/1-234 DENS-----RS-----------------------------------------EWDILSN-
XP_013623953.1/1-236 DENS-----RS-----------------------------------------EWDILSN-
XP_013623587.1/1-236 DENS-----RS-----------------------------------------EWDILSN-
XP_013623995.1/1-236 DENS-----RS-----------------------------------------EWDILSN-
XP_013623820.1/1-236 DENS-----RS-----------------------------------------EWDILSN-
XP_022926381.1/1-227 KEKS-----RS-----------------------------------------EWDILSN-
XP_023517485.1/1-227 KEKS-----RS-----------------------------------------EWDILSN-
XP_023002930.1/1-227 KEKS-----RS-----------------------------------------EWDILSN-
XP_004134971.1/1-229 KEKS-----RS-----------------------------------------EWDILSN-
XP_008439968.1/1-229 KEKS-----RS-----------------------------------------EWDILSN-
XP_022950176.1/1-227 KEKS-----RS-----------------------------------------EWDILSN-
XP_022979090.1/1-227 KEKS-----RS-----------------------------------------EWDILSN-
XP_023543388.1/1-249 KEKS-----RS-----------------------------------------EWDILSN-
XP_023543387.1/1-227 KEKS-----RS-----------------------------------------EWDILSN-
XP_009134323.1/1-228 DENS-----RS-----------------------------------------EWDILSN-
XP_019176002.1/1-227 DENS-----RN-----------------------------------------EWDILSN-
XP_015070655.1/1-221 DENS-----RS-----------------------------------------EWDILSN-
XP_015070652.1/1-221 DENS-----RS-----------------------------------------EWDILSN-
XP_025885589.1/1-221 DENS-----RS-----------------------------------------EWDILSN-
XP_019422236.1/1-227 DENS-----RR-----------------------------------------EWDILSN-
XP_007157166.1/1-226 AENS-----RN-----------------------------------------EWDILSN-
XP_003538765.1/1-227 DENS-----RN-----------------------------------------EWDILSN-
XP_003517589.1/1-227 DQNS-----RN-----------------------------------------EWDILSN-
XP_006573890.1/1-227 DQNS-----RN-----------------------------------------EWDILSN-
XP_011000687.1/1-226 DENH-----RS-----------------------------------------EWDILSN-
XP_024436817.1/1-226 DENH-----RS-----------------------------------------EWDILSN-
XP_024436820.1/1-226 DENH-----RS-----------------------------------------EWDILSN-
XP_002518520.2/1-226 DENH-----RS-----------------------------------------EWDILSN-
XP_015574304.1/1-226 DENH-----RS-----------------------------------------EWDILSN-
XP_021642058.1/1-227 DENH-----RR-----------------------------------------EWDILSN-
XP_012091395.1/1-226 DENH-----RS-----------------------------------------EWDILSN-
XP_021635824.1/1-226 DENH-----RS-----------------------------------------EWDILSN-
XP_021596887.1/1-226 DENH-----RS-----------------------------------------EWDILSN-
XP_010057969.1/1-228 DENS-----RN-----------------------------------------EWDILSN-
XP_006413738.1/1-245 DENS-----RS-----------------------------------------EWDILSN-
XP_013630219.1/1-235 DENS-----RS-----------------------------------------QWDILSN-
XP_013656136.1/1-235 DENS-----RS-----------------------------------------QWDILSN-
XP_013685757.1/1-235 DENS-----RS-----------------------------------------QWDILSN-
XP_013696499.1/1-240 DENS-----RS-----------------------------------------EWDILSN-
XP_009137213.1/1-240 DENS-----RS-----------------------------------------EWDILSN-
XP_013598423.1/1-241 DENS-----RS-----------------------------------------EWDILSN-
XP_013717184.1/1-241 DENS-----RS-----------------------------------------EWDILSN-
XP_018480642.1/1-241 DENS-----RS-----------------------------------------EWDILSN-
XP_018477464.1/1-241 DENS-----RS-----------------------------------------EWDILSN-
NP_193906.2/1-248    DENS-----RS-----------------------------------------EWDILSN-
XP_020873301.1/1-248 DENS-----RS-----------------------------------------EWDILSN-
XP_006285476.1/1-249 DENS-----RS-----------------------------------------EWDILSN-
XP_010434132.1/1-252 DENS-----RS-----------------------------------------EWDILSN-
XP_010439429.1/1-252 DENS-----RS-----------------------------------------EWDILSN-
XP_010449009.1/1-254 DENS-----RS-----------------------------------------EWDILSN-
XP_010432053.1/1-234 DENS-----RK-----------------------------------------EWDILSN-
XP_020876375.1/1-234 DENS-----RK-----------------------------------------EWDILSN-
XP_023636874.1/1-234 DENS-----RK-----------------------------------------EWDILSN-
XP_010422328.1/1-234 DENS-----RK-----------------------------------------EWDILSN-
XP_010455798.1/1-234 DENS-----RK-----------------------------------------EWDILSN-
NP_567274.1/1-235    DENS-----RK-----------------------------------------EWDILSN-
XP_024009884.1/1-236 DENS-----RS-----------------------------------------EWDILSN-
XP_018452346.1/1-236 DENS-----RS-----------------------------------------EWDILSN-
XP_013611160.1/1-236 DENS-----RS-----------------------------------------EWDILSN-
XP_009114536.1/1-235 DENS-----RS-----------------------------------------EWDILSN-
XP_013662000.1/1-235 DENS-----RS-----------------------------------------EWDILSN-
XP_018472664.1/1-233 DENS-----RS-----------------------------------------EWDILSN-
XP_022555047.1/1-234 DENS-----RS-----------------------------------------EWDILSN-
XP_013735469.1/1-234 DENS-----RS-----------------------------------------EWDILSN-
XP_013629800.1/1-234 DENS-----RS-----------------------------------------EWDILSN-
XP_013735464.1/1-234 DENS-----RS-----------------------------------------EWDILSN-
XP_022571893.1/1-234 DENS-----RS-----------------------------------------EWDILSN-
XP_010538642.1/1-236 DENS-----RS-----------------------------------------EWDILSN-
XP_019057303.1/1-229 DENS-----RS-----------------------------------------EWDILSN-
XP_016545426.1/1-226 DENS-----RN-----------------------------------------EWDILSN-
XP_009793614.1/1-227 NENS-----RN-----------------------------------------EWDILSN-
XP_016508926.1/1-227 NENS-----RN-----------------------------------------EWDILSN-
XP_016560096.1/1-235 DENS-----RS-----------------------------------------EWDILSN-
XP_021993721.1/1-227 DDNS-----RS-----------------------------------------EWDILSN-
XP_022036928.1/1-226 DENS-----RS-----------------------------------------EWDILSN-
XP_023742870.1/1-226 DENS-----RS-----------------------------------------EWDILSN-
XP_006352605.1/1-223 DENS-----RN-----------------------------------------EWDILSN-
XP_010327237.1/1-223 DENS-----RN-----------------------------------------EWDILSN-
XP_015055984.1/1-223 DENS-----RN-----------------------------------------EWDILSN-
XP_009609606.1/1-229 NENS-----RN-----------------------------------------EWDILSN-
XP_009609608.1/1-229 NENS-----RN-----------------------------------------EWDILSN-
XP_018628742.1/1-229 NENS-----RN-----------------------------------------EWDILSN-
XP_019237831.1/1-229 NENS-----RN-----------------------------------------EWDILSN-
XP_018849836.1/1-227 YENS-----RT-----------------------------------------QWDILSN-
XP_007214541.1/1-230 DENS-----RT-----------------------------------------EWDILSN-
XP_021821565.1/1-230 DENS-----RT-----------------------------------------EWDILSN-
XP_008224792.1/1-230 DENS-----RT-----------------------------------------EWDILSN-
XP_024198966.1/1-227 DENS-----RS-----------------------------------------EWDILSN-
XP_004293268.1/1-227 DENS-----RS-----------------------------------------EWDILSN-
XP_009364098.1/1-227 DENS-----RS-----------------------------------------EWDILSN-
XP_008371114.1/1-227 DENS-----RS-----------------------------------------EWDILSN-
XP_008384034.1/1-227 DENS-----RS-----------------------------------------EWDILSN-
XP_009340946.1/1-227 DENS-----RS-----------------------------------------EWDILSN-
XP_009357415.1/1-227 DENS-----RS-----------------------------------------EWDILSN-
XP_011037708.1/1-226 DENH-----RS-----------------------------------------EWDILSN-
XP_024454819.1/1-226 DENH-----RS-----------------------------------------EWDILSN-
XP_015884145.1/1-226 DENS-----RN-----------------------------------------EWDILSN-
XP_024024693.1/1-228 DENS-----RS-----------------------------------------EWDILSN-
XP_024024692.1/1-228 DENS-----RS-----------------------------------------EWDILSN-
XP_018820351.1/1-226 DENS-----RR-----------------------------------------EWDILSN-
XP_023904570.1/1-226 DENS-----RS-----------------------------------------EWDILSN-
XP_023904569.1/1-226 DENS-----RS-----------------------------------------EWDILSN-
XP_006449076.1/1-226 DENS-----RS-----------------------------------------EWDILSN-
XP_006468235.1/1-226 DENS-----RS-----------------------------------------EWDILSN-
XP_022721034.1/1-226 DENS-----RS-----------------------------------------EWDILSN-
XP_022721035.1/1-226 DENS-----RS-----------------------------------------EWDILSN-
XP_022724746.1/1-226 DENS-----RS-----------------------------------------EWDILSN-
XP_007026002.1/1-226 DENS-----RS-----------------------------------------EWDILSN-
XP_021294392.1/1-226 DENS-----RS-----------------------------------------EWDILSN-
XP_022777405.1/1-226 DENS-----RS-----------------------------------------EWDILSN-
XP_017615807.1/1-226 DENS-----RS-----------------------------------------EWDILSN-
XP_016753649.1/1-226 DENS-----RS-----------------------------------------EWDILSN-
XP_016683607.1/1-226 DENS-----RS-----------------------------------------EWDILSN-
XP_012451363.1/1-226 DENS-----RS-----------------------------------------EWDILSN-
XP_002266688.1/1-226 AENS-----RS-----------------------------------------EWDILSN-
XP_021853797.1/1-224 DENS-----RN-----------------------------------------EWDILSN-
XP_021719656.1/1-227 DENS-----RS-----------------------------------------EWDILSN-
XP_021766927.1/1-227 DENS-----RS-----------------------------------------EWDILSN-
XP_010665524.1/1-227 DENS-----RS-----------------------------------------EWDILSN-
XP_009605864.1/1-234 DENS-----RS-----------------------------------------EWDILSN-
XP_019223778.1/1-233 DENS-----RS-----------------------------------------EWDILSN-
XP_016440385.1/1-233 DENS-----RS-----------------------------------------EWDILSN-
XP_009790281.1/1-233 DENS-----RS-----------------------------------------EWDILSN-
XP_016570434.1/1-222 DENS-----RN-----------------------------------------EWDILSN-
XP_016486479.1/1-229 DENS-----RS-----------------------------------------EWDILSN-
XP_009597904.1/1-229 DENS-----RS-----------------------------------------EWDILSN-
XP_009799795.1/1-229 DENS-----RS-----------------------------------------EWDILSN-
XP_019249652.1/1-229 DENS-----RS-----------------------------------------EWDILSN-
XP_006343296.1/1-221 DENS-----RS-----------------------------------------EWDILSN-
XP_020217012.1/1-226 DENS-----RN-----------------------------------------EWDILSN-
XP_017406677.1/1-226 DENS-----RN-----------------------------------------EWDILSN-
XP_014519998.1/1-226 DENS-----RN-----------------------------------------EWDILSN-
XP_014519997.1/1-226 DENS-----RN-----------------------------------------EWDILSN-
XP_012841999.1/1-239 DENS-----RS-----------------------------------------EWDILSN-
XP_024984347.1/1-227 DENS-----RS-----------------------------------------EWDILSN-
XP_023751202.1/1-227 DENS-----RS-----------------------------------------EWDILSN-
XP_023751208.1/1-227 DENS-----RS-----------------------------------------EWDILSN-
XP_022041933.1/1-227 DENS-----RS-----------------------------------------EWDILSN-
XP_024988632.1/1-226 DENS-----RS-----------------------------------------EWDILSN-
XP_022018253.1/1-226 DENS-----RS-----------------------------------------EWDILSN-
XP_023771293.1/1-226 DENS-----RS-----------------------------------------EWDILSN-
XP_019170324.1/1-226 DENS-----RS-----------------------------------------EWDILSN-
XP_017230122.1/1-226 DENS-----RS-----------------------------------------EWDILSN-
XP_011096101.1/1-226 DENS-----RS-----------------------------------------EWDILSN-
XP_012848972.1/1-227 DENS-----RS-----------------------------------------EWDILSN-
XP_022876975.1/1-227 DENL-----RN-----------------------------------------EWDILSN-
XP_022863158.1/1-227 DENS-----RN-----------------------------------------EWDILSN-
XP_022877991.1/1-227 DENS-----RN-----------------------------------------EWDILSN-
XP_019223745.1/1-223 DENT-----RS-----------------------------------------EWDILSN-
XP_021800220.1/1-221 DENS-----RN-----------------------------------------EWDIFSN-
XP_021684589.1/1-235 DENS-----RNEP----------------------------------HYSCLQWDILSN-
XP_021684519.1/1-235 DENS-----RNEP----------------------------------HYSCLQWDILSN-
XP_021684722.1/1-235 DENS-----RNEP----------------------------------HYSCLQWDILSN-
XP_021633498.1/1-230 DENS-----R-----------------------------------------SQWDILSN-
XP_009420270.1/1-216 DERS-----RN-----------------------------------------EWDILSN-
XP_003573403.1/1-232 DETS-----RS-----------------------------------------EWDILSN-
XP_006659894.1/1-229 DETS-----RS-----------------------------------------EWDILSN-
XP_015650022.1/1-263 DETS-----RSEVPNLPPIPREFIQFADQILYVI--------KFIPDEFGFPQWDILSN-
XP_015650023.1/1-230 DETS-----RS-----------------------------------------EWDILSN-
XP_020407343.1/1-231 DETS-----RS-----------------------------------------EWDILSN-
NP_001105127.2/1-231 DETS-----RS-----------------------------------------EWDILSN-
XP_008661805.1/1-239 DETS-----RS-----------------------------------------EWDILSN-
NP_001145750.1/1-239 DETS-----RS-----------------------------------------EWDILSN-
XP_025821536.1/1-230 DETS-----RS-----------------------------------------EWDILSN-
XP_002443965.1/1-229 DETS-----RS-----------------------------------------EWDILSN-
XP_004972697.1/1-229 DETS-----RS-----------------------------------------EWDILSN-
XP_008667765.1/1-230 DESS-----RS-----------------------------------------EWDILSN-
NP_001347891.1/1-231 DESS-----RS-----------------------------------------EWDILSN-
XP_015636340.1/1-229 DESS-----RS-----------------------------------------EWDILSN-
XP_015636338.1/1-229 DESS-----RS-----------------------------------------EWDILSN-
XP_006652859.1/1-228 DESS-----RS-----------------------------------------EWDILSN-
XP_003580598.1/1-233 DETS-----RS-----------------------------------------QWDILSN-
XP_020199198.1/1-227 DETS-----RS-----------------------------------------EWDILSN-
XP_020400772.1/1-230 DESS-----RS-----------------------------------------EWDILSN-
NP_001334663.1/1-230 DESS-----RS-----------------------------------------EWDILSN-
XP_008662197.1/1-270 DESS-----RSEARA-HSIPNNFYLFNHERTRSRMHPVREPHFCSVLFFSFVQWDILSN-
XP_008662196.1/1-270 DESS-----RSEARA-HSIPNNFYLFNHERTRSRMHPVREPHFCSVLFFSFVQWDILSN-
XP_025823505.1/1-227 DESS-----RS-----------------------------------------EWDILSN-
XP_004976894.1/1-227 DESS-----RS-----------------------------------------EWDILSN-
XP_002448563.2/1-227 DESS-----RS-----------------------------------------EWDILSN-
XP_019414154.1/1-227 DENS-----RN-----------------------------------------EWDILSN-
XP_006348085.1/1-234 DENS-----RN-----------------------------------------EWDILSN-
XP_004232734.1/1-234 DENS-----RN-----------------------------------------EWDILSN-
XP_015066898.1/1-234 DENS-----RN-----------------------------------------EWDILSN-
XP_003605900.1/1-232 NENS-----RT-----------------------------------------EWDILSN-
XP_022988693.1/1-229 DENS-----RN-----------------------------------------EWDILSN-
XP_022930609.1/1-229 DENS-----RN-----------------------------------------EWDILSN-
XP_023530197.1/1-229 DENS-----RN-----------------------------------------EWDILSN-
XP_016188415.1/1-237 DENS-----RS-----------------------------------------EWDILSN-
XP_011628991.1/1-236 DENS-----RS-----------------------------------------QWDILSN-
XP_006826512.2/1-236 DENS-----RS-----------------------------------------QWDILSN-
XP_020177398.1/1-220 DDST-----RS-----------------------------------------EWDILSN-
XP_015695486.1/1-219 DDST-----RS-----------------------------------------EWDILSN-
XP_015648539.1/1-217 DDST-----RS-----------------------------------------EWDILSN-
XP_020401599.1/1-218 DDAT-----RS-----------------------------------------EWDMLSN-
XP_020401598.1/1-218 DDAT-----RS-----------------------------------------EWDMLSN-
XP_020401597.1/1-218 DDAT-----RS-----------------------------------------EWDMLSN-
XP_021319954.1/1-239 DDAT-----RS-----------------------------------------EWDILSN-
XP_002445014.2/1-239 DDAT-----RS-----------------------------------------EWDILSN-
XP_025819913.1/1-218 DDAT-----RS-----------------------------------------EWDILSN-
XP_004972909.1/1-218 DDAT-----RS-----------------------------------------EWDILSN-
XP_020100432.1/1-229 DESS-----RS-----------------------------------------EWDILSN-
XP_009385059.1/1-228 DESS-----RS-----------------------------------------EWDILSN-
XP_018678797.1/1-226 DESS-----RS-----------------------------------------EWDILSN-
XP_009412012.1/1-233 DESS-----RS-----------------------------------------EWDILSN-
XP_018685149.1/1-236 DESS-----RS-----------------------------------------EWDILSN-
XP_020591278.1/1-230 DESS-----RS-----------------------------------------EWDILSN-
XP_020702932.1/1-229 DESS-----RS-----------------------------------------EWDILSN-
XP_020702933.1/1-230 DESS-----RS-----------------------------------------EWDILSN-
XP_020702931.1/1-230 DESS-----RS-----------------------------------------EWDILSN-
XP_020692781.1/1-229 DESS-----RS-----------------------------------------EWDILSN-
XP_020692780.1/1-229 DESS-----RS-----------------------------------------EWDILSN-
XP_020591591.1/1-229 DESS-----RS-----------------------------------------EWDILSN-
XP_020591590.1/1-229 DESS-----RS-----------------------------------------EWDILSN-
XP_020109359.1/1-227 DESS-----RS-----------------------------------------VWDILSN-
XP_020270114.1/1-229 DESS-----RS-----------------------------------------EWDILSN-
XP_010908855.1/1-227 NESS-----RS-----------------------------------------EWDILSN-
XP_008808987.1/1-227 NESS-----RS-----------------------------------------EWDILSN-
XP_008784552.1/1-223 DESS-----RS-----------------------------------------EWDILSN-
XP_010931820.1/1-223 DESS-----RS-----------------------------------------EWDILSN-
XP_003540308.2/1-226 DENS-----RS-----------------------------------------EWDILSN-
XP_006592818.1/1-226 DENS-----RS-----------------------------------------EWDILSN-
XP_006592816.1/1-226 DENS-----RS-----------------------------------------EWDILSN-
XP_003543387.1/1-226 DENS-----RS-----------------------------------------EWDILSN-
XP_020223651.1/1-226 DENS-----RS-----------------------------------------EWDILSN-
XP_020223648.1/1-226 DENS-----RS-----------------------------------------EWDILSN-
XP_007149968.1/1-226 DENS-----RS-----------------------------------------EWDILSN-
XP_017425582.1/1-226 DENS-----RS-----------------------------------------EWDILSN-
XP_014501634.1/1-226 DENS-----RS-----------------------------------------EWDILSN-
XP_022776632.1/1-226 DENS-----RN-----------------------------------------EWDILSN-
XP_022776631.1/1-226 DENS-----RN-----------------------------------------EWDILSN-
XP_022776629.1/1-226 DENS-----RN-----------------------------------------EWDILSN-
XP_022733641.1/1-226 DENS-----RS-----------------------------------------EWDILSN-
XP_022733640.1/1-226 DENS-----RS-----------------------------------------EWDILSN-
XP_022733638.1/1-226 DENS-----RS-----------------------------------------EWDILSN-
XP_007045612.1/1-226 DENS-----RS-----------------------------------------EWDILSN-
XP_007045611.1/1-226 DENS-----RS-----------------------------------------EWDILSN-
XP_017971478.1/1-226 DENS-----RS-----------------------------------------EWDILSN-
XP_021292769.1/1-226 DENS-----RS-----------------------------------------EWDILSN-
XP_021292767.1/1-226 DENS-----RS-----------------------------------------EWDILSN-
XP_021292765.1/1-226 DENS-----RS-----------------------------------------EWDILSN-
XP_010657309.1/1-226 DENS-----RS-----------------------------------------EWDILSN-
XP_010657313.1/1-226 DENS-----RS-----------------------------------------EWDILSN-
XP_010657310.1/1-226 DENS-----RS-----------------------------------------EWDILSN-
XP_010657311.1/1-226 DENS-----RS-----------------------------------------EWDILSN-
XP_012086347.1/1-228 DENS-----RS-----------------------------------------EWDILSN-
XP_012086344.1/1-228 DENS-----RS-----------------------------------------EWDILSN-
XP_024029967.1/1-226 DENS-----RS-----------------------------------------EWDILSN-
XP_010109517.1/1-226 DENS-----RS-----------------------------------------EWDILSN-
XP_006469697.1/1-226 DENT-----RS-----------------------------------------EWDILSN-
XP_024046029.1/1-226 DENT-----RS-----------------------------------------EWDILSN-
XP_020234199.1/1-226 DENS-----RN-----------------------------------------EWDILSN-
XP_020234201.1/1-226 DENS-----RN-----------------------------------------EWDILSN-
XP_021630856.1/1-226 DENS-----RS-----------------------------------------EWDILSN-
XP_021630853.1/1-226 DENS-----RS-----------------------------------------EWDILSN-
XP_021907367.1/1-221 DDNS-----RN-----------------------------------------EWDILSN-
XP_010275996.1/1-221 DENS-----RS-----------------------------------------EWDILSN-
XP_020870784.1/1-223 DENS-----RN-----------------------------------------EWDILSN-
NP_172015.1/1-223    DENS-----RN-----------------------------------------EWDILSN-
XP_006418050.1/1-223 DENS-----RN-----------------------------------------EWDILSN-
XP_018447753.1/1-223 DENS-----RN-----------------------------------------EWDILSN-
XP_010457605.1/1-218 DENS-----RN-----------------------------------------EWDILSN-
XP_023632765.1/1-218 DENS-----RN-----------------------------------------EWDILSN-
XP_010484829.1/1-218 DENS-----RN-----------------------------------------EWDILSN-
XP_010475208.1/1-218 DENS-----RN-----------------------------------------EWDILSN-
XP_018487097.1/1-220 DENS-----RN-----------------------------------------EWDILSN-
XP_013664795.1/1-220 DENS-----RN-----------------------------------------EWDILSN-
XP_013716253.1/1-220 DENS-----RN-----------------------------------------EWDILSN-
XP_009118607.1/1-220 DENS-----RN-----------------------------------------EWDILSN-
XP_013605632.1/1-220 DENS-----RN-----------------------------------------EWDILSN-
XP_010475204.1/1-220 DENS-----RN-----------------------------------------EWDILSN-
XP_010457604.1/1-220 DENS-----RN-----------------------------------------EWDILSN-
XP_023632762.1/1-220 DENS-----RN-----------------------------------------EWDILSN-
XP_010484823.1/1-220 DENS-----RN-----------------------------------------EWDILSN-
XP_022558401.1/1-220 DENS-----RN-----------------------------------------EWDILSN-
XP_013586641.1/1-220 DENS-----RN-----------------------------------------EWDILSN-
XP_022558399.1/1-220 DENS-----RN-----------------------------------------EWDILSN-
XP_022558400.1/1-220 DENS-----RN-----------------------------------------EWDILSN-
XP_013586642.1/1-220 DENS-----RN-----------------------------------------EWDILSN-
XP_013696832.1/1-220 DENS-----RN-----------------------------------------EWDILSN-
XP_022548878.1/1-220 DENS-----RN-----------------------------------------EWDILSN-
XP_013666552.1/1-220 DENS-----RN-----------------------------------------EWDILSN-
XP_009119177.1/1-220 DENS-----RN-----------------------------------------EWDILSN-
XP_020964430.1/1-225 DENS-----RS-----------------------------------------EWDILSN-
XP_015936069.1/1-225 DENS-----RS-----------------------------------------EWDILSN-
XP_020964429.1/1-225 DENS-----RS-----------------------------------------EWDILSN-
XP_015936068.1/1-225 DENS-----RS-----------------------------------------EWDILSN-
XP_015577880.1/1-228 DENS-----RN-----------------------------------------QWDILSN-
XP_002524172.2/1-228 DENS-----RN-----------------------------------------QWDILSN-
XP_007132557.1/1-221 DENS-----RN-----------------------------------------EWDILSN-
XP_017433694.1/1-221 DENS-----RN-----------------------------------------EWDILSN-
XP_014494419.1/1-221 DENS-----RN-----------------------------------------EWDILSN-
XP_022634310.1/1-221 DENS-----RN-----------------------------------------EWDILSN-
XP_010267735.1/1-225 DENS-----RS-----------------------------------------EWDILSN-
XP_010267734.1/1-225 DENS-----RS-----------------------------------------EWDILSN-
XP_024185199.1/1-224 DENS-----RN-----------------------------------------EWDILSN-
XP_024185197.1/1-224 DENS-----RN-----------------------------------------EWDILSN-
XP_009342246.1/1-222 DENS-----RS-----------------------------------------EWDILSN-
XP_009342244.1/1-222 DENS-----RS-----------------------------------------EWDILSN-
XP_009342240.1/1-222 DENS-----RS-----------------------------------------EWDILSN-
XP_020416503.1/1-221 DENS-----RN-----------------------------------------EWDILSN-
XP_021822854.1/1-221 DENS-----RN-----------------------------------------EWDILSN-
XP_007217584.1/1-221 DENS-----RN-----------------------------------------EWDILSN-
XP_021822852.1/1-221 DENS-----RN-----------------------------------------EWDILSN-
XP_018730806.1/1-228 DENS-----RS-----------------------------------------EWDILSN-
XP_010051064.1/1-228 DENS-----RS-----------------------------------------EWDILSN-
XP_015881130.2/1-229 DENS-----RS-----------------------------------------EWDILSN-
XP_023534533.1/1-219 DENS-----RS-----------------------------------------EWDILSN-
XP_022947095.1/1-219 DENS-----RS-----------------------------------------EWDILSN-
XP_022947096.1/1-219 DENS-----RS-----------------------------------------EWDILSN-
XP_022970950.1/1-219 DENS-----RS-----------------------------------------EWDILSN-
XP_022970951.1/1-219 DENS-----RS-----------------------------------------EWDILSN-
XP_022140692.1/1-217 DENS-----RS-----------------------------------------EWDILSN-
XP_022140691.1/1-217 DENS-----RS-----------------------------------------EWDILSN-
XP_008455853.1/1-220 DENS-----RS-----------------------------------------EWDILSN-
XP_011650012.1/1-220 DENS-----RS-----------------------------------------EWDILSN-
XP_008455850.1/1-220 DENS-----RS-----------------------------------------EWDILSN-
XP_011650011.1/1-220 DENS-----RS-----------------------------------------EWDILSN-
XP_023513403.1/1-220 DENS-----RS-----------------------------------------EWDILSN-
XP_022944761.1/1-220 DENS-----RS-----------------------------------------EWDILSN-
XP_023513401.1/1-220 DENS-----RS-----------------------------------------EWDILSN-
XP_022944759.1/1-220 DENS-----RS-----------------------------------------EWDILSN-
XP_022986270.1/1-220 DENS-----RN-----------------------------------------EWDILSN-
XP_022986268.1/1-220 DENS-----RN-----------------------------------------EWDILSN-
XP_022136006.1/1-224 DENS-----RN-----------------------------------------EWDILSN-
XP_008455423.1/1-229 DENS-----RN-----------------------------------------EWDILSN-
XP_011658704.1/1-229 DENS-----RN-----------------------------------------EWDILSN-
XP_004144488.1/1-229 DENS-----RN-----------------------------------------EWDILSN-
XP_022968990.1/1-229 DENS-----RN-----------------------------------------EWDILSN-
XP_023554403.1/1-229 DENS-----RN-----------------------------------------EWDILSN-
XP_022952948.1/1-229 DENS-----RN-----------------------------------------EWDILSN-
XP_019447751.1/1-228 DENS-----RS-----------------------------------------EWDILSN-
XP_019447749.1/1-228 DENS-----RS-----------------------------------------EWDILSN-
XP_004515074.1/1-228 DENS-----RN-----------------------------------------EWDILSN-
XP_012463792.1/1-235 DENS-----RS-----------------------------------------EWDILSN-
XP_012463790.1/1-235 DENS-----RS-----------------------------------------EWDILSN-
XP_016678783.1/1-235 DENS-----RS-----------------------------------------EWDILSN-
XP_016678782.1/1-235 DENS-----RS-----------------------------------------EWDILSN-
XP_017631648.1/1-235 DENS-----RS-----------------------------------------EWDILSN-
XP_016732503.1/1-235 DENS-----RS-----------------------------------------EWDILSN-
XP_017631647.1/1-235 DENS-----RS-----------------------------------------EWDILSN-
XP_012438430.1/1-229 DENS-----RN-----------------------------------------EWDILSN-
XP_012438427.1/1-229 DENS-----RN-----------------------------------------EWDILSN-
XP_012438429.1/1-229 DENS-----RN-----------------------------------------EWDILSN-
XP_017616162.1/1-229 DENS-----RN-----------------------------------------EWDILSN-
XP_017616160.1/1-229 DENS-----RN-----------------------------------------EWDILSN-
XP_017616161.1/1-229 DENS-----RN-----------------------------------------EWDILSN-
XP_016726347.1/1-229 DENS-----RN-----------------------------------------EWDILSN-
XP_016726344.1/1-229 DENS-----RN-----------------------------------------EWDILSN-
XP_016726345.1/1-229 DENS-----RN-----------------------------------------EWDILSN-
XP_019448933.1/1-227 DENS-----RS-----------------------------------------EWDILSN-
XP_004487400.1/1-228 DENS-----RS-----------------------------------------EWDILSN-
XP_003596999.2/1-227 DENS-----RS-----------------------------------------EWDILSN-
XP_024631890.1/1-227 DENS-----RS-----------------------------------------EWDILSN-
XP_024631891.1/1-227 DENS-----RS-----------------------------------------EWDILSN-
XP_004287935.1/1-224 DENS-----RN-----------------------------------------EWDILSN-
XP_018808969.1/1-228 DENS-----RS-----------------------------------------EWDILSN-
XP_018808967.1/1-228 DENS-----RS-----------------------------------------EWDILSN-
XP_018817598.1/1-227 DENY-----RN-----------------------------------------EWDILSN-
XP_018817597.1/1-227 DENY-----RN-----------------------------------------EWDILSN-
XP_018817594.1/1-227 DENY-----RN-----------------------------------------EWDILSN-
XP_018817599.1/1-227 DENY-----RN-----------------------------------------EWDILSN-
XP_023915056.1/1-227 DENS-----RS-----------------------------------------EWDILSN-
XP_023915054.1/1-227 DENS-----RS-----------------------------------------EWDILSN-
XP_023915053.1/1-227 DENS-----RS-----------------------------------------EWDILSN-
XP_021685023.1/1-228 DENS-----RN-----------------------------------------EWDILSN-
XP_021684657.1/1-228 DENS-----RN-----------------------------------------EWDILSN-
XP_021685089.1/1-228 DENS-----RN-----------------------------------------EWDILSN-
XP_015383009.1/1-229 DENT-----RS-----------------------------------------EWDILSN-
XP_015383007.1/1-229 DENT-----RS-----------------------------------------EWDILSN-
XP_020539441.1/1-235 DENS-----RSELY----------------------------------YSYLQWDILSN-
XP_020539439.1/1-235 DENS-----RSELY----------------------------------YSYLQWDILSN-
XP_015577881.1/1-233 DENS-----RN-----------------------------------------QWDILSN-
XP_015577879.1/1-233 DENS-----RN-----------------------------------------QWDILSN-
XP_015577877.1/1-233 DENS-----RN-----------------------------------------QWDILSN-
XP_021630854.1/1-231 DENS-----RS-----------------------------------------EWDILSN-
XP_021630849.1/1-231 DENS-----RS-----------------------------------------EWDILSN-
XP_021652390.1/1-229 DENS-----RS-----------------------------------------EWDILSN-
XP_021652388.1/1-229 DENS-----RS-----------------------------------------EWDILSN-
XP_021652389.1/1-234 DENS-----RS-----------------------------------------EWDILSN-
XP_021652386.1/1-234 DENS-----RS-----------------------------------------EWDILSN-
XP_006592380.1/1-228 DENS-----RN-----------------------------------------EWDILSN-
XP_003539872.2/1-228 DENS-----RN-----------------------------------------EWDILSN-
XP_025984806.1/1-230 DENS-----RN-----------------------------------------EWDILSN-
XP_014632311.1/1-230 DENS-----RN-----------------------------------------EWDILSN-
XP_024450362.1/1-227 DENT-----RN-----------------------------------------EWDILSN-
XP_002302880.2/1-227 DENT-----RN-----------------------------------------EWDILSN-
XP_024450361.1/1-227 DENT-----RN-----------------------------------------EWDILSN-
XP_024450360.1/1-227 DENT-----RN-----------------------------------------EWDILSN-
XP_011011493.1/1-227 DENT-----RN-----------------------------------------EWDILSN-
XP_011011491.1/1-227 DENT-----RN-----------------------------------------EWDILSN-
XP_011011492.1/1-227 DENT-----RN-----------------------------------------EWDILSN-
XP_011011489.1/1-227 DENT-----RN-----------------------------------------EWDILSN-
XP_011011488.1/1-227 DENT-----RN-----------------------------------------EWDILSN-
XP_011005116.1/1-225 DEST-----RN-----------------------------------------EWDILSN-
XP_011005113.1/1-225 DEST-----RN-----------------------------------------EWDILSN-
XP_011005112.1/1-225 DEST-----RN-----------------------------------------EWDILSN-
XP_024440391.1/1-225 DEST-----RN-----------------------------------------EWDILSN-
XP_002320458.2/1-225 DEST-----RN-----------------------------------------EWDILSN-
XP_024440389.1/1-225 DEST-----RN-----------------------------------------EWDILSN-
XP_019453767.1/1-227 DENS-----RS-----------------------------------------EWDILSN-
XP_012478857.1/1-227 DEHS-----RS-----------------------------------------EWDILSN-
XP_012478855.1/1-227 DEHS-----RS-----------------------------------------EWDILSN-
XP_012478856.1/1-227 DEHS-----RS-----------------------------------------EWDILSN-
XP_012478851.1/1-227 DEHS-----RS-----------------------------------------EWDILSN-
XP_016724999.1/1-227 DEHS-----RS-----------------------------------------EWDILSN-
XP_017638204.1/1-227 DEHS-----RS-----------------------------------------EWDILSN-
XP_017638203.1/1-227 DEHS-----RS-----------------------------------------EWDILSN-
XP_016667113.1/1-227 DEHS-----RS-----------------------------------------EWDILSN-
XP_016667112.1/1-227 DEHS-----RS-----------------------------------------EWDILSN-
XP_015881131.2/1-227 DENS-----RS-----------------------------------------EWDILSN-
XP_008348131.1/1-222 DENS-----RS-----------------------------------------EWDILSN-
XP_008348133.1/1-220 DENS-----RS-----------------------------------------EWDILSN-
XP_018502305.1/1-220 DENS-----RS-----------------------------------------EWDILSN-
XP_009342243.1/1-220 DENS-----RS-----------------------------------------EWDILSN-
XP_017179321.1/1-222 DENS-----RS-----------------------------------------EWDILSN-
XP_017179320.1/1-222 DENS-----RS-----------------------------------------EWDILSN-
XP_008341986.1/1-222 DENS-----RS-----------------------------------------EWDILSN-
XP_017179319.1/1-220 DENS-----RS-----------------------------------------EWDILSN-
XP_024185198.1/1-222 DENS-----RN-----------------------------------------EWDILSN-
XP_011463209.1/1-222 DENS-----RN-----------------------------------------EWDILSN-
XP_020416501.1/1-219 DENS-----RN-----------------------------------------EWDILSN-
XP_021822853.1/1-219 DENS-----RN-----------------------------------------EWDILSN-
XP_008230079.1/1-219 DENS-----RN-----------------------------------------EWDILSN-
XP_020870786.1/1-221 DENS-----RN-----------------------------------------EWDILSN-
NP_001117231.1/1-221 DENS-----RN-----------------------------------------EWDILSN-
XP_018817596.1/1-225 DENY-----RN-----------------------------------------EWDILSN-
XP_019079350.1/1-224 DENS-----RS-----------------------------------------EWDILSN-
XP_022733639.1/1-224 DENS-----RS-----------------------------------------EWDILSN-
XP_021292768.1/1-224 DENS-----RS-----------------------------------------EWDILSN-
XP_021292766.1/1-224 DENS-----RS-----------------------------------------EWDILSN-
XP_017971480.1/1-224 DENS-----RS-----------------------------------------EWDILSN-
XP_018721484.1/1-222 DENS-----RS-----------------------------------------EWDILSN-
XP_018721483.1/1-222 DENS-----RS-----------------------------------------EWDILSN-
XP_018721482.1/1-222 DENS-----RS-----------------------------------------EWDILSN-
XP_025687365.1/1-237 DENS-----RS-----------------------------------------EWDILSN-
XP_006851770.2/1-243 DEHL-----RS-----------------------------------------QWDILSN-
XP_003623826.1/1-230 NEKL-----RS-----------------------------------------EWDILSN-
XP_004492678.1/1-230 DERL-----RS-----------------------------------------EWDILSN-
XP_004492677.1/1-230 DERL-----RS-----------------------------------------EWDILSN-
XP_012847897.1/1-236 DERL-----RS-----------------------------------------EWDILSN-
XP_012843673.1/1-229 DERL-----RS-----------------------------------------EWDILSN-
XP_021279608.1/1-239 DERL-----RS-----------------------------------------EWDILSN-
XP_025641241.1/1-241 DERL-----RS-----------------------------------------EWDILSN-
XP_025641246.1/1-241 DERL-----RS-----------------------------------------EWDILSN-
XP_020991942.1/1-241 DERL-----RS-----------------------------------------EWDILSN-
XP_025632115.1/1-241 DERL-----RS-----------------------------------------EWDILSN-
XP_025632116.1/1-241 DERL-----RS-----------------------------------------EWDILSN-
XP_017442222.1/1-246 DERL-----RS-----------------------------------------EWDILSN-
XP_017442225.1/1-246 DERL-----RS-----------------------------------------EWDILSN-
XP_006576359.1/1-244 DERL-----RS-----------------------------------------EWDILSN-
XP_003521284.1/1-244 DERL-----RS-----------------------------------------EWDILSN-
XP_024174849.1/1-237 DERL-----RS-----------------------------------------EWDILSN-
XP_024174854.1/1-237 DERL-----RS-----------------------------------------EWDILSN-
XP_004306832.1/1-241 DERL-----RS-----------------------------------------EWDILSN-
XP_011469062.1/1-241 DERL-----RS-----------------------------------------EWDILSN-
XP_009340632.2/1-243 DERL-----RS-----------------------------------------EWDILSN-
XP_008385703.1/1-243 DERL-----RS-----------------------------------------EWDILSN-
XP_008385704.1/1-243 DERL-----RS-----------------------------------------EWDILSN-
XP_008232741.1/1-243 DERL-----RS-----------------------------------------EWDILSN-
XP_008232742.1/1-243 DERL-----RS-----------------------------------------EWDILSN-
XP_007220256.1/1-243 DERL-----RS-----------------------------------------EWDILSN-
XP_020413144.1/1-243 DERL-----RS-----------------------------------------EWDILSN-
XP_021817103.1/1-243 DERL-----RS-----------------------------------------EWDILSN-
XP_021817105.1/1-243 DERL-----RS-----------------------------------------EWDILSN-
XP_022872559.1/1-230 DEHL-----RS-----------------------------------------EWDILSN-
XP_016575742.1/1-238 DERL-----RS-----------------------------------------EWDILSN-
XP_017608938.1/1-232 DERL-----RS-----------------------------------------EWDILSN-
XP_017608947.1/1-232 DERL-----RS-----------------------------------------EWDILSN-
XP_010255501.1/1-245 DERL-----RS-----------------------------------------EWDILSN-
XP_011023407.1/1-238 NERL-----RS-----------------------------------------EWDILSN-
XP_011023404.1/1-238 NERL-----RS-----------------------------------------EWDILSN-
XP_011023405.1/1-238 NERL-----RS-----------------------------------------EWDILSN-
XP_019152252.1/1-243 DERL-----RS-----------------------------------------EWDILSN-
XP_022870029.1/1-235 DERL-----RS-----------------------------------------EWDILSN-
XP_017699285.1/1-225 DERL-----RS-----------------------------------------EWDILSN-
XP_008795402.1/1-225 DERL-----RS-----------------------------------------EWDILSN-
XP_010933869.1/1-224 DERL-----RS-----------------------------------------EWDILSN-
XP_008799528.1/1-222 DERL-----RS-----------------------------------------EWDILSN-
XP_010919252.1/1-223 DERL-----RS-----------------------------------------EWDILSN-
XP_010919250.1/1-223 DERL-----RS-----------------------------------------EWDILSN-
XP_007139955.1/1-229 DERL-----RS-----------------------------------------EWDILSN-
XP_017418814.1/1-230 DERL-----RS-----------------------------------------EWDILSN-
XP_014490274.1/1-230 DERL-----RS-----------------------------------------EWDILSN-
XP_003534596.1/1-229 DERL-----RS-----------------------------------------EWDILSN-
XP_006587871.1/1-229 DERL-----RS-----------------------------------------EWDILSN-
XP_003552359.1/1-228 DERL-----RS-----------------------------------------EWDILSN-
XP_015961616.1/1-228 DERL-----RS-----------------------------------------EWDILSN-
XP_015961617.1/1-228 DERL-----RS-----------------------------------------EWDILSN-
XP_025696060.1/1-228 DERL-----RS-----------------------------------------EWDILSN-
XP_025696061.1/1-228 DERL-----RS-----------------------------------------EWDILSN-
XP_016198931.1/1-228 DERL-----RS-----------------------------------------EWDILSN-
XP_016198932.1/1-228 DERL-----RS-----------------------------------------EWDILSN-
XP_025649261.1/1-228 DERL-----RS-----------------------------------------EWDILSN-
XP_025649262.1/1-228 DERL-----RS-----------------------------------------EWDILSN-
XP_021849370.1/1-228 DERL-----RS-----------------------------------------EWDILSN-
XP_021767325.1/1-228 DERL-----RS-----------------------------------------EWDILSN-
XP_021748881.1/1-228 DERL-----RS-----------------------------------------EWDILSN-
XP_019461451.1/1-231 DERL-----RS-----------------------------------------EWDILSN-
XP_012574211.1/1-233 DERL-----RS-----------------------------------------EWDILSN-
XP_004510857.1/1-233 DERL-----RS-----------------------------------------EWDILSN-
XP_020237185.1/1-234 DERL-----RS-----------------------------------------EWDILSN-
XP_020237261.1/1-234 DERL-----RS-----------------------------------------EWDILSN-
XP_019456357.1/1-238 DERL-----RS-----------------------------------------EWDILSN-
XP_013444922.1/1-235 DERL-----RS-----------------------------------------EWDILSN-
XP_003528880.1/1-238 DERL-----RS-----------------------------------------EWDILSN-
XP_006583340.1/1-238 DERL-----RS-----------------------------------------EWDILSN-
XP_014516480.1/1-238 DERL-----RS-----------------------------------------EWDILSN-
XP_014516483.1/1-238 DERL-----RS-----------------------------------------EWDILSN-
XP_007134961.1/1-238 DERL-----RS-----------------------------------------EWDILSN-
XP_010671720.1/1-229 DERL-----RS-----------------------------------------EWDILSN-
XP_012482732.1/1-232 DERL-----RS-----------------------------------------EWDILSN-
XP_012482742.1/1-232 DERL-----RS-----------------------------------------EWDILSN-
XP_016745638.1/1-232 DERL-----RS-----------------------------------------EWDILSN-
XP_016745639.1/1-232 DERL-----RS-----------------------------------------EWDILSN-
XP_010278578.1/1-242 DERL-----RS-----------------------------------------EWDILSN-
XP_010278577.1/1-242 DERL-----RS-----------------------------------------EWDILSN-
XP_006445143.1/1-238 DERL-----RS-----------------------------------------EWDILSN-
XP_006445141.1/1-238 DERL-----RS-----------------------------------------EWDILSN-
XP_022770361.1/1-238 DERL-----RS-----------------------------------------EWDILSN-
XP_022757219.1/1-237 DERL-----RS-----------------------------------------EWDILSN-
XP_022757220.1/1-237 DERL-----RS-----------------------------------------EWDILSN-
NP_001314420.1/1-237 NERL-----RS-----------------------------------------EWDILSN-
XP_016735217.1/1-237 DERL-----RS-----------------------------------------EWDILSN-
XP_012437660.1/1-237 DERL-----RS-----------------------------------------EWDILSN-
XP_012437661.1/1-237 DERL-----RS-----------------------------------------EWDILSN-
XP_017615505.1/1-237 DERL-----RS-----------------------------------------EWDILSN-
XP_017615506.1/1-237 DERL-----RS-----------------------------------------EWDILSN-
XP_016723220.1/1-237 DERL-----RS-----------------------------------------EWDILSN-
XP_016723229.1/1-237 DERL-----RS-----------------------------------------EWDILSN-
XP_021904309.1/1-244 DERL-----RS-----------------------------------------EWDILSN-
XP_021904311.1/1-244 DERL-----RS-----------------------------------------EWDILSN-
XP_007051912.2/1-241 DERL-----RS-----------------------------------------EWDILSN-
XP_007051913.2/1-241 DERL-----RS-----------------------------------------EWDILSN-
XP_012489878.1/1-239 DERL-----RS-----------------------------------------EWDILSN-
XP_016748231.1/1-239 DERL-----RS-----------------------------------------EWDILSN-
XP_017631289.1/1-239 DERL-----RS-----------------------------------------EWDILSN-
XP_023006686.1/1-236 DERL-----RS-----------------------------------------EWDILSN-
XP_022958954.1/1-236 DERL-----RS-----------------------------------------EWDILSN-
XP_023548045.1/1-236 DERL-----RS-----------------------------------------EWDILSN-
XP_008460172.1/1-241 DERL-----RS-----------------------------------------EWDILSN-
XP_004144982.1/1-241 DERL-----RS-----------------------------------------EWDILSN-
XP_023514722.1/1-242 DERL-----RS-----------------------------------------EWDILSN-
XP_022999814.1/1-242 DERL-----RS-----------------------------------------EWDILSN-
XP_022964504.1/1-242 DERL-----RS-----------------------------------------EWDILSN-
XP_010053664.1/1-245 DEQL-----RS-----------------------------------------EWDILSN-
XP_019189515.1/1-235 DERL-----RS-----------------------------------------EWDILSN-
XP_023730775.1/1-234 DERL-----RS-----------------------------------------EWDILSN-
XP_023730774.1/1-234 DERL-----RS-----------------------------------------EWDILSN-
XP_022020543.1/1-230 DERL-----RS-----------------------------------------EWDILSN-
XP_024962572.1/1-236 DERL-----RS-----------------------------------------EWDILSN-
XP_022017696.1/1-236 DEQL-----RS-----------------------------------------EWDILSN-
XP_024018220.1/1-245 DERL-----RS-----------------------------------------EWDILSN-
XP_011093166.1/1-234 NEHL-----RS-----------------------------------------EWDILSN-
XP_011093167.1/1-234 NEHL-----RS-----------------------------------------EWDILSN-
XP_006339457.1/1-238 DERL-----RS-----------------------------------------EWDILSN-
XP_015059298.1/1-238 DERL-----RS-----------------------------------------EWDILSN-
NP_001234657.2/1-238 DERL-----RS-----------------------------------------EWDILSN-
XP_009602857.1/1-241 DERL-----RS-----------------------------------------EWDILSN-
XP_009602856.1/1-241 DERL-----RS-----------------------------------------EWDILSN-
XP_009782444.1/1-245 DERL-----RS-----------------------------------------EWDILSN-
XP_009782443.1/1-245 DERL-----RS-----------------------------------------EWDILSN-
XP_019259406.1/1-245 DERL-----RS-----------------------------------------EWDILSN-
XP_015867375.1/1-242 DERL-----RS-----------------------------------------EWDILSN-
XP_015867374.1/1-242 DERL-----RS-----------------------------------------EWDILSN-
XP_015888672.1/1-242 DERL-----RS-----------------------------------------EWDILSN-
XP_018810322.1/1-233 NERL-----RS-----------------------------------------EWDILSN-
XP_018859074.1/1-233 NERL-----RS-----------------------------------------EWDILSN-
XP_017231243.1/1-235 DERL-----RS-----------------------------------------EWDILSN-
XP_017231244.1/1-235 DERL-----RS-----------------------------------------EWDILSN-
XP_017231245.1/1-235 DERL-----RS-----------------------------------------EWDILSN-
XP_017236322.1/1-235 DERL-----RS-----------------------------------------EWDILSN-
XP_002301331.2/1-238 NERL-----RS-----------------------------------------EWDILSN-
XP_024451706.1/1-238 NERL-----RS-----------------------------------------EWDILSN-
XP_002320755.1/1-241 DERL-----RS-----------------------------------------EWDILSN-
XP_011035097.1/1-241 DERL-----RS-----------------------------------------EWDILSN-
XP_018813891.1/1-238 DERL-----RS-----------------------------------------EWDILSN-
XP_012083470.1/1-239 DERL-----RS-----------------------------------------EWDILSN-
XP_021651338.1/1-240 DERL-----RS-----------------------------------------EWDILSN-
XP_021613538.1/1-241 DERL-----RS-----------------------------------------EWDILSN-
XP_021686862.1/1-241 DERL-----RS-----------------------------------------EWDILSN-
XP_021686863.1/1-241 DERL-----RS-----------------------------------------EWDILSN-
XP_021612685.1/1-241 DERL-----RS-----------------------------------------EWDILSN-
XP_023879166.1/1-242 DERL-----RS-----------------------------------------EWDILSN-
XP_002272264.2/1-234 DERL-----RS-----------------------------------------EWDILSN-
XP_010661562.1/1-234 DERL-----RS-----------------------------------------EWDILSN-
XP_002511801.1/1-237 DERL-----RS-----------------------------------------EWDILSN-
XP_015584500.1/1-237 DERL-----RS-----------------------------------------EWDILSN-
XP_022141257.1/1-238 NEHL-----RS-----------------------------------------EWDILSN-
XP_023885612.1/1-244 DDRS-----RS-----------------------------------------QWDILCV-
XP_023885611.1/1-244 DDRS-----RS-----------------------------------------QWDILCV-
XP_018830842.1/1-239 DDRL-----RS-----------------------------------------EWDILST-
XP_018830843.1/1-239 DDRL-----RS-----------------------------------------EWDILST-
XP_018830840.1/1-239 DDRL-----RS-----------------------------------------EWDILST-
XP_018830841.1/1-239 DDRL-----RS-----------------------------------------EWDILST-
XP_012090293.1/1-240 DERL-----RC-----------------------------------------EWDILSH-
XP_012090292.1/1-240 DERL-----RC-----------------------------------------EWDILSH-
XP_021614581.1/1-239 DERS-----RS-----------------------------------------EWDILSH-
XP_021614580.1/1-239 DERS-----RS-----------------------------------------EWDILSH-
XP_015581247.1/1-240 DERS-----RC-----------------------------------------EWDILSH-
XP_002510822.1/1-240 DERS-----RC-----------------------------------------EWDILSH-
XP_021648646.1/1-240 DEQL-----RS-----------------------------------------EWDILSH-
XP_021634593.1/1-234 DERL-----RS-----------------------------------------EWDILSH-
XP_021634592.1/1-234 DERL-----RS-----------------------------------------EWDILSH-
XP_006490348.1/1-238 DERM-----RS-----------------------------------------EWDILSN-
XP_006490346.1/1-238 DERM-----RS-----------------------------------------EWDILSN-
XP_024958654.1/1-238 DERM-----RS-----------------------------------------EWDILSN-
XP_006490345.1/1-238 DERM-----RS-----------------------------------------EWDILSN-
XP_006421879.2/1-238 DERM-----RS-----------------------------------------EWDILSN-
XP_021892212.1/1-221 DERL-----RS-----------------------------------------EWDILSN-
XP_021892211.1/1-221 DERL-----RS-----------------------------------------EWDILSN-
XP_021892210.1/1-221 DERL-----RS-----------------------------------------EWDILSN-
XP_022765148.1/1-244 DEQM-----RS-----------------------------------------QWDILSN-
XP_022765158.1/1-244 DEQM-----RS-----------------------------------------QWDILSN-
XP_022765120.1/1-244 DEQM-----RS-----------------------------------------QWDILSN-
XP_022765114.1/1-244 DEQM-----RS-----------------------------------------QWDILSN-
XP_022736693.1/1-237 DERM-----RS-----------------------------------------HWDILSN-
XP_022736692.1/1-237 DERM-----RS-----------------------------------------HWDILSN-
XP_022736689.1/1-237 DERM-----RS-----------------------------------------HWDILSN-
XP_022734775.1/1-244 DEQM-----RS-----------------------------------------QWDILSN-
XP_016743353.1/1-244 DERM-----RS-----------------------------------------EWDILSN-
XP_012474605.1/1-244 DERM-----RS-----------------------------------------EWDILSN-
XP_012474606.1/1-244 DERM-----RS-----------------------------------------EWDILSN-
XP_016743352.1/1-244 DERM-----RS-----------------------------------------EWDILSN-
XP_016743351.1/1-244 DERM-----RS-----------------------------------------EWDILSN-
XP_012474604.1/1-244 DERM-----RS-----------------------------------------EWDILSN-
XP_012474603.1/1-244 DERM-----RS-----------------------------------------EWDILSN-
XP_017646297.1/1-244 DERM-----RS-----------------------------------------EWDILSH-
XP_016736315.1/1-244 DERM-----RS-----------------------------------------EWDILSH-
XP_017646296.1/1-244 DERM-----RS-----------------------------------------EWDILSH-
XP_017646298.1/1-244 DERM-----RS-----------------------------------------EWDILSH-
XP_016736306.1/1-244 DERM-----RS-----------------------------------------EWDILSH-
XP_017646295.1/1-244 DERM-----RS-----------------------------------------EWDILSH-
XP_017972896.1/1-244 DERM-----RS-----------------------------------------QWDILSN-
XP_017972895.1/1-244 DERM-----RS-----------------------------------------QWDILSN-
XP_017972893.1/1-244 DERM-----RS-----------------------------------------QWDILSN-
XP_021274823.1/1-244 DERM-----RS-----------------------------------------QWDILSN-
XP_024536343.1/1-231 DDRL-----RS-----------------------------------------EWDILSN-
XP_006366174.1/1-244 DEQL-----RS-----------------------------------------EWDILSN-
XP_020219711.1/1-212 DERL-----RS-----------------------------------------EWDILSN-
XP_022897056.1/1-222 DEHL-----RS-----------------------------------------EWDILSN-
NP_001281015.1/1-243 DERL-----RS-----------------------------------------EWDILSN-
XP_008383260.1/1-243 DERL-----RS-----------------------------------------EWDILSN-
XP_022896229.1/1-235 NERL-----RS-----------------------------------------EWDILSN-
XP_022896230.1/1-235 NERL-----RS-----------------------------------------EWDILSN-
XP_025816451.1/1-225 DEQL-----RA-----------------------------------------EWDILSN-
XP_004968001.1/1-225 DEQL-----RA-----------------------------------------EWDILSN-
XP_004968002.1/1-225 DEQL-----RA-----------------------------------------EWDILSN-
XP_002457484.1/1-226 DEQL-----RA-----------------------------------------EWDILSN-
XP_009400446.1/1-224 NERL-----RS-----------------------------------------QWDILSN-
XP_018676535.1/1-225 NEQL-----RS-----------------------------------------QWDILSN-
XP_009401771.1/1-232 NEQL-----RS-----------------------------------------QWDILSN-
XP_009384534.1/1-228 NEQL-----RS-----------------------------------------QWDILSN-
XP_020672447.1/1-217 DETL-----RC-----------------------------------------QWDILSN-
XP_020582116.1/1-218 DENL-----RC-----------------------------------------QWDILSN-
XP_020675647.1/1-231 NETL-----RS-----------------------------------------QWDILSN-
XP_020675648.1/1-231 NETL-----RS-----------------------------------------QWDILSN-
XP_020585632.1/1-228 NESL-----RS-----------------------------------------QWDILSN-
XP_016176402.1/1-228 DERL-----RS-----------------------------------------EWDILSN-
XP_016176403.1/1-228 DERL-----RS-----------------------------------------EWDILSN-
XP_016176400.1/1-228 DERL-----RS-----------------------------------------EWDILSN-
XP_016176401.1/1-228 DERL-----RS-----------------------------------------EWDILSN-
XP_010523042.1/1-245 DERL-----RC-----------------------------------------EWDILSN-
XP_010523041.1/1-240 DERL-----RC-----------------------------------------EWDILSN-
XP_020878511.1/1-224 NERM-----RC-----------------------------------------EWDILSN-
NP_567183.2/1-228    NERM-----RC-----------------------------------------EWDILSN-
XP_006287095.1/1-228 NERM-----RC-----------------------------------------EWDILSN-
XP_010456250.1/1-228 NERM-----RC-----------------------------------------EWDILSN-
XP_010419627.1/1-228 NERM-----RC-----------------------------------------EWDILSN-
XP_010427345.1/1-228 NERM-----RC-----------------------------------------EWDILSN-
XP_009111469.1/1-229 NERM-----RC-----------------------------------------EWDILSN-
XP_018487039.1/1-229 NERM-----RC-----------------------------------------EWDILSN-
XP_018453129.1/1-229 NERM-----RC-----------------------------------------EWDILSN-
XP_013612299.1/1-229 NERM-----RC-----------------------------------------EWDILSN-
XP_006396268.1/1-225 NERM-----RC-----------------------------------------EWDILSN-
XP_018475317.1/1-221 NERM-----RC-----------------------------------------EWDILSN-
XP_013629750.1/1-221 NERM-----RC-----------------------------------------EWDILSN-
XP_009134533.1/1-221 NERM-----RC-----------------------------------------EWDILSN-
XP_020531377.1/1-244 DERL-----RS-----------------------------------------EWDILSN-
XP_006857866.1/1-244 DERL-----RS-----------------------------------------EWDILSN-
XP_018434263.1/1-219 DELM-----RS-----------------------------------------EWDILSN-
XP_018434260.1/1-219 DELM-----RS-----------------------------------------EWDILSN-
XP_018434262.1/1-219 DELM-----RS-----------------------------------------EWDILSN-
XP_013688926.1/1-219 DELL-----RS-----------------------------------------EWDILSN-
XP_013637558.1/1-219 DELL-----RS-----------------------------------------EWDILSN-
XP_013637556.1/1-219 DELL-----RS-----------------------------------------EWDILSN-
XP_013637557.1/1-219 DELL-----RS-----------------------------------------EWDILSN-
XP_009143168.1/1-219 DELL-----RS-----------------------------------------EWDILSN-
XP_009143167.1/1-219 DELL-----RS-----------------------------------------EWDILSN-
XP_009143166.1/1-219 DELL-----RS-----------------------------------------EWDILSN-
XP_013692686.1/1-219 DELL-----RS-----------------------------------------EWDILSN-
XP_022574820.1/1-219 DELL-----RS-----------------------------------------EWDILSN-
XP_013692684.1/1-219 DELL-----RS-----------------------------------------EWDILSN-
XP_013748127.1/1-219 DELL-----RS-----------------------------------------EWDILSN-
XP_013692685.1/1-219 DELL-----RS-----------------------------------------EWDILSN-
XP_013748124.1/1-219 DELL-----RS-----------------------------------------EWDILSN-
XP_013748126.1/1-219 DELL-----RS-----------------------------------------EWDILSN-
XP_022574821.1/1-219 DELL-----RS-----------------------------------------EWDILSN-
XP_006290613.1/1-229 NERL-----RS-----------------------------------------EWDILSN-
XP_013592301.1/1-230 NERL-----RS-----------------------------------------EWDILSN-
XP_018442581.1/1-230 NERL-----RS-----------------------------------------EWDILSN-
XP_013750213.1/1-232 NERL-----RS-----------------------------------------EWDILSN-
XP_013648430.1/1-232 NERL-----RS-----------------------------------------EWDILSN-
XP_009104292.1/1-232 NERL-----RS-----------------------------------------EWDILSN-
XP_019058514.1/1-223 DERL-----RS-----------------------------------------EWDILSN-
XP_010523728.1/1-220 DERL-----RS-----------------------------------------EWDILSN-
XP_024514927.1/1-242 DERL-----RS-----------------------------------------EWDILSN-
XP_002985242.1/1-242 DERL-----RS-----------------------------------------EWDILSN-
XP_024530500.1/1-242 DERL-----RS-----------------------------------------EWDILSN-
XP_024530499.1/1-242 DERL-----RS-----------------------------------------EWDILSN-
XP_024530501.1/1-242 DERL-----RS-----------------------------------------EWDILSN-
XP_010107411.1/1-242 DERF-----RS-----------------------------------------DWDILSN-
XP_024028635.1/1-242 DERF-----RS-----------------------------------------DWDILSN-
XP_015877999.1/1-244 NERL-----RS-----------------------------------------EWDILSN-
XP_019427294.1/1-247 NEGL-----RS-----------------------------------------EWDILSN-
XP_019427292.1/1-247 NEGL-----RS-----------------------------------------EWDILSN-
XP_019452814.1/1-239 NEQL-----RS-----------------------------------------EWDILSN-
XP_019452815.1/1-239 NEQL-----RS-----------------------------------------EWDILSN-
XP_003592295.2/1-235 DERL-----RS-----------------------------------------EWDILSN-
XP_024625328.1/1-235 DERL-----RS-----------------------------------------EWDILSN-
XP_012570175.1/1-236 DERL-----RS-----------------------------------------EWDILSN-
XP_012570176.1/1-236 DERL-----RS-----------------------------------------EWDILSN-
XP_004496593.1/1-236 DERL-----RS-----------------------------------------EWDILSN-
XP_025620154.1/1-234 DERL-----RS-----------------------------------------EWDILSN-
XP_015941562.1/1-234 DERL-----RS-----------------------------------------EWDILSN-
XP_025620153.1/1-234 DERL-----RS-----------------------------------------EWDILSN-
XP_020239673.1/1-232 DERL-----RS-----------------------------------------EWDILSN-
XP_020239648.1/1-232 DERL-----RS-----------------------------------------EWDILSN-
XP_007143377.1/1-235 DERL-----RS-----------------------------------------EWDILSN-
XP_007143378.1/1-235 DERL-----RS-----------------------------------------EWDILSN-
XP_017415467.1/1-234 DERL-----RS-----------------------------------------EWDILSN-
XP_017415466.1/1-234 DERL-----RS-----------------------------------------EWDILSN-
XP_014514421.1/1-236 DERL-----RS-----------------------------------------EWDILSN-
XP_014514419.1/1-236 DERL-----RS-----------------------------------------EWDILSN-
XP_006606097.1/1-235 DERL-----RS-----------------------------------------EWDILSN-
XP_006606096.1/1-235 DERL-----RS-----------------------------------------EWDILSN-
XP_003536477.1/1-234 DERL-----RS-----------------------------------------EWDILSN-
XP_006589557.1/1-234 DERL-----RS-----------------------------------------EWDILSN-
XP_006402516.1/1-230 NERL-----RS-----------------------------------------EWDILSN-
NP_191674.1/1-237    NERL-----RS-----------------------------------------EWDILSN-
XP_002876596.1/1-239 NERL-----RS-----------------------------------------EWDILSN-
XP_010413451.1/1-238 NEVL-----RS-----------------------------------------EWDILSN-
XP_010413452.1/1-238 NEVL-----RS-----------------------------------------EWDILSN-
XP_010413453.1/1-238 NEVL-----RS-----------------------------------------EWDILSN-
XP_010469036.1/1-242 NEGL-----RS-----------------------------------------EWDILSN-
XP_010469037.1/1-242 NEGL-----RS-----------------------------------------EWDILSN-
XP_010469038.1/1-242 NEGL-----RS-----------------------------------------EWDILSN-
XP_010512448.1/1-238 NEGL-----RS-----------------------------------------EWDILSN-
XP_010512449.1/1-238 NEGL-----RS-----------------------------------------EWDILSN-
XP_006397757.1/1-219 DELL-----RS-----------------------------------------DWDILSN-
XP_024010837.1/1-220 DELL-----RS-----------------------------------------DWDILSN-
XP_023640300.1/1-220 DELL-----RS-----------------------------------------EWDILSN-
XP_006293718.1/1-220 DELL-----RS-----------------------------------------EWDILSN-
XP_010506553.1/1-219 DVLL-----RS-----------------------------------------EWDILSN-
XP_010508114.1/1-219 DVLL-----RS-----------------------------------------EWDILSN-
XP_010508115.1/1-219 DVLL-----RS-----------------------------------------EWDILSN-
XP_022147276.1/1-219 DERL-----RS-----------------------------------------EWDILSN-
XP_023527464.1/1-222 DERL-----RS-----------------------------------------EWDILSN-
XP_022980233.1/1-222 DERL-----RS-----------------------------------------EWDILSN-
XP_022980231.1/1-222 DERL-----RS-----------------------------------------EWDILSN-
XP_022924366.1/1-223 DERL-----RS-----------------------------------------EWDILSN-
XP_022924364.1/1-223 DERL-----RS-----------------------------------------EWDILSN-
XP_023527467.1/1-221 DERL-----RS-----------------------------------------EWDILSN-
XP_023527463.1/1-221 DERL-----RS-----------------------------------------EWDILSN-
XP_023527465.1/1-221 DERL-----RS-----------------------------------------EWDILSN-
XP_022980232.1/1-221 DERL-----RS-----------------------------------------EWDILSN-
XP_022924365.1/1-222 DERL-----RS-----------------------------------------EWDILSN-
XP_009412599.1/1-224 EASF-----RS-----------------------------------------KWDILSN-
XP_018677084.1/1-231 NQQL-----RS-----------------------------------------QWDILSN-
XP_020114860.1/1-246 DERL-----RS-----------------------------------------QWDILSN-
XP_020114857.1/1-246 DERL-----RS-----------------------------------------QWDILSN-
XP_020114859.1/1-246 DERL-----RS-----------------------------------------QWDILSN-
XP_020084803.1/1-234 DERL-----RS-----------------------------------------QWDILSN-
XP_015625616.1/1-226 DEQL-----RA-----------------------------------------EWDILSN-
XP_006647677.1/1-226 DEQL-----RA-----------------------------------------EWDILSN-
NP_001105493.1/1-226 DEQL-----RA-----------------------------------------EWDILSN-
XP_020178635.1/1-225 DEQL-----RA-----------------------------------------EWDILSN-
XP_003570085.1/1-223 DEQL-----RA-----------------------------------------EWDILSN-
XP_020247871.1/1-226 NEQR-----RS----------------------------------Q-------LDVLSS-
XP_020247870.1/1-226 NEQR-----RS----------------------------------Q-------LDVLSS-
XP_024004764.1/1-211 DDYH-----RS----------------------------------Q-------LEILFN-
XP_018449374.1/1-217 NDHR-----RS----------------------------------Q-------MEMLFN-
XP_013602839.1/1-215 HDHG-----RS----------------------------------Q-------MEMLFS-
XP_013655966.1/1-215 HDHG-----RS----------------------------------Q-------MEEMFN-
XP_009108342.1/1-215 HDHG-----RS----------------------------------Q-------MEEMFN-
XP_013655964.1/1-215 HDHG-----RS----------------------------------Q-------MEEMFN-
XP_020872468.1/1-222 DNQR-----LS----------------------------------Q-------LEILFN-
NP_193506.2/1-221    DNQR-----LS----------------------------------Q-------LEILFM-
XP_023633855.1/1-222 NNQR-----LS----------------------------------Q-------LEILFN-
XP_010449572.1/1-221 DNQR-----LS----------------------------------Q-------LEILFN-
XP_010439957.1/1-223 DNQR-----LS----------------------------------Q-------LEILFN-
XP_010439959.1/1-223 DNQR-----LS----------------------------------Q-------LEILFN-
XP_010439958.1/1-223 DNQR-----LS----------------------------------Q-------LEILFN-
XP_006854056.1/1-234 DDRR-----RT----------------------------------E-------LDVLSS-
XP_020529072.1/1-234 DDRR-----RT----------------------------------E-------LDVLSS-
NP_199499.3/1-224    DQHH-----QS----------------------------------L-------LEVLFN-
XP_020871346.1/1-224 DQHH-----QS----------------------------------L-------LEVLFN-
XP_006281890.1/1-224 DQHH-----QS----------------------------------L-------LEVLFN-
XP_010481400.1/1-224 DQHH-----QS----------------------------------L-------LEVLFN-
XP_010494885.1/1-224 DQHH-----QS----------------------------------L-------LEVLFN-
XP_018455882.1/1-227 DQHH-----HS----------------------------------L-------LEVLFS-
XP_013713146.1/1-227 DQHH-----HS----------------------------------L-------LEVLFS-
XP_013607209.1/1-227 DQHH-----HS----------------------------------L-------LEVLFS-
XP_009114317.1/1-227 DQHH-----HS----------------------------------L-------LEVLFS-
XP_013661958.1/1-227 DQHH-----HS----------------------------------L-------LEVLFS-
XP_006398384.1/1-227 DQHH-----HS----------------------------------L-------LEVLFN-
XP_024011152.1/1-227 DQHH-----HS----------------------------------L-------LEVLFN-
XP_018482944.1/1-227 HQHH-----HS----------------------------------L-------LEVLFN-
XP_009101472.1/1-227 HQHH-----HS----------------------------------L-------LEVLFS-
XP_013693979.1/1-227 HQHH-----HS----------------------------------L-------LEVLFS-
XP_013615089.1/1-227 HQHH-----HS----------------------------------L-------LEVLFS-
XP_013704013.1/1-227 HQHH-----HS----------------------------------L-------LEVLFS-
XP_009373477.1/1-246 DERR-----RV----------------------------------QPPALCQQLDVLTN-
XP_006662631.2/1-256 DEQQ-----RC----------------------------------Q-------LEILSN-
XP_015613461.1/1-259 DEQQ-----RC----------------------------------Q-------LEILSN-
NP_001337805.1/1-240 DEQQ-----RC----------------------------------Q-------LEIMSS-
XP_020163828.1/1-239 DEQQ-----RC----------------------------------Q-------LEILSN-
XP_010235052.1/1-238 DEQQ-----RC----------------------------------Q-------LEIMSN-
XP_025798256.1/1-238 DEQQ-----RC----------------------------------Q-------LEILSN-
XP_004983555.1/1-238 DEQQ-----RC----------------------------------Q-------LEILSN-
XP_002464880.2/1-238 DEQQ-----RC----------------------------------Q-------LEILSN-
XP_020262583.1/1-231 DEQR-----RS----------------------------------Q-------LDVLSN-
XP_020677410.1/1-231 DEQR-----RS----------------------------------Q-------MDALSS-
XP_020577604.1/1-229 DQQR-----RS----------------------------------Q-------LDALSS-
XP_020577606.1/1-229 DQQR-----RS----------------------------------Q-------LDALSS-
XP_020577607.1/1-229 DQQR-----RS----------------------------------Q-------LDALSS-
XP_019709148.1/1-245 DERC-----RC----------------------------------Q-------LEALSN-
XP_008803567.1/1-241 DEQR-----RS----------------------------------Q-------L-ALSS-
XP_008803570.2/1-241 DEQR-----RS----------------------------------Q-------L-ALSS-
XP_018681950.1/1-231 DEQR-----RS----------------------------------QL-----QLDVLSS-
XP_018681951.1/1-231 DEQR-----RS----------------------------------QL-----QLDVLSS-
XP_010919976.1/1-237 DEQR-----RS----------------------------------Q-------LDVLSN-
XP_008800084.1/1-237 DERR-----RS----------------------------------Q-------LDVLSN-
XP_018679997.1/1-229 DEQR-----RS----------------------------------Q-------LDVLSS-
XP_009397149.1/1-229 DEQR-----RS----------------------------------Q-------LDVLSS-
XP_024995523.1/1-235 DERR-----RS----------------------------------Q-------LDVLSN-
XP_023736252.1/1-233 DERR-----RS----------------------------------Q-------LDVLSN-
XP_023736253.1/1-233 DERR-----RS----------------------------------Q-------LDVLSN-
XP_022022041.1/1-231 DERR-----RS----------------------------------Q-------LDILSN-
XP_021969857.1/1-226 DERR-----RP----------------------------------Q-------LNIISN-
XP_010245213.1/1-236 DERR-----RS----------------------------------Q-------LDVLSG-
XP_010245210.1/1-236 DERR-----RS----------------------------------Q-------LDVLSG-
XP_010276832.1/1-235 DERR-----RS----------------------------------Q-------LDVLSS-
XP_010276837.1/1-235 DERR-----RS----------------------------------Q-------LDVLSS-
XP_019155823.1/1-230 DERH-----RA----------------------------------Q-------LDALSN-
XP_022881662.1/1-238 DERC-----RA----------------------------------Q-------LDVLSN-
XP_022877113.1/1-234 DEQR-----RI----------------------------------Q-------LDVLSN-
XP_016450789.1/1-246 DERR-----RA----------------------------------Q-------LDVLSN-
XP_009597691.1/1-246 DERR-----RA----------------------------------Q-------LDVLSN-
XP_009786380.1/1-246 DERR-----RA----------------------------------Q-------LDVLSN-
XP_016494823.1/1-246 DERR-----RA----------------------------------Q-------LDVLSN-
XP_016494822.1/1-246 DERR-----RA----------------------------------Q-------LDVLSN-
XP_019230103.1/1-246 DERR-----RA----------------------------------Q-------LDVLSN-
XP_016573090.1/1-250 DERR-----RA----------------------------------Q-------LDVLSN-
XP_016573102.1/1-250 DERR-----RA----------------------------------Q-------LDVLSN-
XP_006352911.1/1-250 DERR-----RA----------------------------------Q-------LDVLSN-
XP_015166604.1/1-250 DERR-----RA----------------------------------Q-------LDVLSN-
XP_015085651.1/1-250 DERR-----RA----------------------------------Q-------LDVLSN-
XP_015085654.1/1-250 DERR-----RA----------------------------------Q-------LDVLSN-
XP_004245919.1/1-250 DERR-----RA----------------------------------Q-------LDVLSN-
XP_025888165.1/1-250 DERR-----RA----------------------------------Q-------LDVLSN-
XP_011070327.1/1-236 DERR-----RA----------------------------------Q-------LEVLSN-
XP_011070329.1/1-236 DERR-----RA----------------------------------Q-------LEVLSN-
XP_012843107.1/1-236 DERS-----RA----------------------------------Q-------LEVLSN-
XP_019423575.1/1-234 DERH-----RS----------------------------------Q-------MDALSN-
XP_019423571.1/1-234 DERH-----RS----------------------------------Q-------MDALSN-
XP_019435331.1/1-234 DEHQ-----RS----------------------------------Q-------MDALSN-
XP_003612811.1/1-239 DERH-----RS----------------------------------Q-------MDALSN-
XP_020978415.1/1-227 DERH-----RS----------------------------------Q-------MDALSN-
XP_015963259.1/1-227 DERH-----RS----------------------------------Q-------MDALSN-
XP_025697880.1/1-227 DERH-----RS----------------------------------Q-------MDALSN-
XP_025697882.1/1-227 DERH-----RS----------------------------------Q-------MDALSN-
XP_025697881.1/1-227 DERH-----RS----------------------------------Q-------MDALSN-
XP_020217931.1/1-233 DERH-----RS----------------------------------Q-------MDALSN-
XP_007158253.1/1-232 DERH-----RS----------------------------------Q-------MDALSN-
XP_017427665.1/1-232 DERH-----RS----------------------------------Q-------MDALSN-
XP_017427666.1/1-232 DERH-----RS----------------------------------Q-------MDALSN-
XP_014521371.1/1-232 DERH-----RS----------------------------------Q-------MDALSN-
XP_014521373.1/1-232 DERH-----RS----------------------------------Q-------MDALSN-
XP_006587604.1/1-242 DERR-----RS----------------------------------Q-------MDALSN-
NP_001352051.1/1-233 DERH-----RS----------------------------------Q-------MDTLSN-
XP_018840870.1/1-239 DERR-----RS----------------------------------Q-------LDVISN-
XP_018852246.1/1-240 DERR-----RS----------------------------------Q-------LDVLSN-
XP_010053633.1/1-234 DERR-----RS----------------------------------Q-------LDVLSN-
XP_021675948.1/1-236 DEHR-----RS----------------------------------Q-------LDVLSN-
XP_012081064.1/1-228 DEQR-----RS----------------------------------Q-------LDVLSN-
XP_011028126.1/1-235 DEHR-----RS----------------------------------Q-------LEVLSN-
XP_002303433.2/1-235 DEHR-----RS----------------------------------Q-------LELLSN-
XP_015897558.1/1-247 DERR-----RS----------------------------------Q-------LDVLSN-
XP_002277673.1/1-238 DERR-----RA----------------------------------Q-------LDVLSN-
XP_022996123.1/1-236 DERQ-----RS----------------------------------Q-------LEVLSN-
XP_023534245.1/1-236 DERQ-----RS----------------------------------Q-------LEVLSN-
XP_022931244.1/1-236 DERQ-----RF----------------------------------Q-------LEVLSN-
XP_022931242.1/1-236 DERQ-----RS----------------------------------Q-------LEVLSN-
XP_022159691.1/1-235 DERR-----RS----------------------------------Q-------LEVLSN-
XP_011652640.1/1-232 DERR-----RS----------------------------------Q-------LEVLSN-
XP_011652639.1/1-232 DERR-----RS----------------------------------Q-------LEVLSN-
XP_008466007.1/1-232 DERR-----RS----------------------------------Q-------LEVLSN-
XP_023511589.1/1-230 DERR-----RS----------------------------------Q-------LEVLSN-
XP_022963906.1/1-230 DERR-----RS----------------------------------Q-------LEVLSN-
XP_023511591.1/1-230 DERR-----RS----------------------------------Q-------LEVLSN-
XP_022963908.1/1-230 DERR-----RS----------------------------------Q-------LEVLSN-
XP_022967184.1/1-230 DERR-----RS----------------------------------Q-------LEVLSN-
XP_022967186.1/1-230 DERR-----RS----------------------------------Q-------LEVLSN-
XP_010518669.1/1-225 DERC-----RS----------------------------------Q-------LEVMFN-
XP_010677365.1/1-235 NEYR-----RS----------------------------------Q-------LEALSN-
XP_010677364.1/1-235 NEYR-----RS----------------------------------Q-------LEALSN-
XP_021852084.1/1-226 NEYR-----RS----------------------------------Q-------LEALSN-
XP_021852076.1/1-226 NEYR-----RS----------------------------------Q-------LEALSN-
XP_021765591.1/1-233 NEYR-----RS----------------------------------Q-------LEALSN-
XP_021768254.1/1-233 NEYR-----RS----------------------------------Q-------LEALSN-
XP_017232690.1/1-221 DERS-----RA----------------------------------Q-------MEVLSN-
XP_017232692.1/1-221 DERS-----RA----------------------------------Q-------MEVLSN-
XP_024021697.1/1-248 DEHR-----RC----------------------------------Q-------LEVLSN-
XP_010096931.1/1-248 DEHR-----RC----------------------------------Q-------LEVLSN-
XP_024021696.1/1-248 DEHR-----RC----------------------------------Q-------LEVLSN-
XP_017621310.1/1-230 DERR-----RA----------------------------------Q-------LEVLSN-
XP_016731150.1/1-230 DERR-----RA----------------------------------Q-------LEVLSN-
XP_012472199.1/1-230 DERR-----RA----------------------------------Q-------LEVLSN-
XP_016727305.1/1-230 DERR-----RA----------------------------------Q-------LEVLSN-
XP_016731145.1/1-230 DERR-----RA----------------------------------Q-------LEVLSN-
XP_012472200.1/1-230 DERR-----RA----------------------------------Q-------LEVLSN-
XP_012472201.1/1-230 DERR-----RA----------------------------------Q-------LEVLSN-
NP_001314348.1/1-230 DERR-----RA----------------------------------Q-------LEVLSN-
XP_016727300.1/1-230 DERR-----RA----------------------------------Q-------LEVLSN-
XP_022738572.1/1-235 DESR-----RA----------------------------------Q-------LEVFSN-
XP_021297648.1/1-235 DERR-----RS----------------------------------Q-------LEVLSN-
XP_017973778.1/1-235 DERS-----RS----------------------------------Q-------LEVLSN-
XP_017973780.1/1-235 DERS-----RS----------------------------------Q-------LEVLSN-
XP_022717458.1/1-235 DERR-----RA----------------------------------Q-------LEVLSN-
XP_022717453.1/1-235 DERR-----RA----------------------------------Q-------LEVLSN-
XP_022717437.1/1-235 DERR-----RA----------------------------------Q-------LEVLSN-
XP_022756982.1/1-232 DERR-----RA----------------------------------Q-------LEVLSN-
XP_004300207.1/1-230 DERR-----RA----------------------------------Q-------LDVFSN-
XP_024169578.1/1-230 DERR-----RA----------------------------------Q-------LDVLSN-
XP_008238183.1/1-249 DEHR-----RA----------------------------------Q------QLDVLSN-
XP_016651445.1/1-249 DEHR-----RA----------------------------------Q------QLDVLSN-
XP_020418924.1/1-249 DEHR-----RA----------------------------------Q------QLDVLSN-
XP_021804146.1/1-249 DEHR-----RA----------------------------------Q------QLDVLSN-
XP_008238184.1/1-248 DEHR-----RA-----------------------------------------QLDVLSN-
XP_016651446.1/1-248 DEHR-----RA-----------------------------------------QLDVLSN-
XP_007209851.1/1-248 DEHR-----RA-----------------------------------------QLDVLSN-
XP_021804154.1/1-248 DEHR-----RA-----------------------------------------QLDVLSN-
XP_017179076.1/1-239 EERR-----RA----------------------------------QP-------DVLTN-
XP_008356445.1/1-246 DERR-----RA----------------------------------QPPALCQQLDVLTN-
XP_008356446.1/1-240 DERR-----RA----------------------------------------QQLDVLTN-
XP_008356447.1/1-239 DERR-----RA-----------------------------------------QLDVLTN-
XP_009373485.1/1-239 DERR-----RV-----------------------------------------QLDVLTN-
XP_009373484.1/1-240 DERR-----RV----------------------------------------QQLDVLTN-
XP_004512486.1/1-245 DERH-----RS----------------------------------Q-------MDALSN-
XP_023898631.1/1-242 DERR-----RS----------------------------------Q-------LEVLSN-
XP_024465375.1/1-238 DEHR-----RS----------------------------------Q-------LEVLSN-
XP_011018502.1/1-238 DEHR-----RS----------------------------------Q-------LEVLSN-
XP_011018503.1/1-238 DEHR-----RS----------------------------------Q-------LEVLSN-
XP_021893615.1/1-233 DEQR-----RS----------------------------------Q-------LEVLSN-
XP_024952893.1/1-247 DERR-----RS-----------------------------------------QLDVLSN-
XP_006471522.1/1-248 DERR-----RS----------------------------------Q------QLDVLSN-
XP_006432745.2/1-248 DERR-----RS----------------------------------Q------QLDVLSN-
XP_021640807.1/1-241 DEQR-----IS----------------------------------Q-------LDVLSN-
XP_002518976.1/1-243 DERR-----RS----------------------------------Q-------LDVLSN-
XP_021598537.1/1-237 DERR-----RS----------------------------------Q-------LDVLSN-
XP_021598535.1/1-239 DERR-----RS----------------------------------QV-----QLDVLSN-
XP_006659280.1/1-226 DEQC-----LV----------------------------------E-------WRYLFGD
XP_025801379.1/1-254 NEQR-----RG----------------------------------E-------WDFVHT-
XP_023156326.1/1-229 DDQR-----RG----------------------------------E-------WDAFFA-
NP_001349156.1/1-229 DDQR-----RG----------------------------------E-------WDAFFA-
XP_021310588.1/1-246 DGQR-----RG----------------------------------E-------WDVFA--
XP_020404516.1/1-224 NGQR-----RG----------------------------------E-------WDAFVC-
XP_022684239.1/1-229 NGQR-----RG----------------------------------E-------WDKFGY-
XP_020404892.1/1-233 DLQR-----RG----------------------------------E-------WDAHAD-
XP_022684238.1/1-237 NLQR-----RG----------------------------------E-------WDSFVN-
XP_025825251.1/1-229 NSQR-----RG----------------------------------E-------WDNFVN-
XP_020183090.1/1-242 DGER-----RG----------------------------------E-------WDVLAE-
XP_020194819.1/1-240 DGDR-----RT----------------------------------E-------WDIGAN-
XP_024314575.1/1-244 DGSR-----RG----------------------------------E-------WDSLAS-
XP_024314342.1/1-235 DGNR-----RW----------------------------------E-------WDSLVA-
XP_024959674.1/1-192 CAEC-----RP----------------------------------K-------WDMAVL-
XP_019196338.1/1-215 NGSN-----RS----------------------------------K-------WDLLSF-
XP_006646400.1/1-226 NLTL-----RS----------------------------------K-------WDVLVH-
XP_015632244.1/1-226 NLTH-----RC----------------------------------K-------WDVLVH-
XP_024314625.1/1-231 NNVL-----RA----------------------------------K-------WDVLAS-
XP_020164122.1/1-228 NNLL-----RP----------------------------------K-------WDVLVN-
XP_025814677.1/1-221 SNLL-----RV----------------------------------K-------WDVLVN-
XP_022682466.1/1-225 NNLL-----RI----------------------------------K-------WDVLVN-
XP_002456467.1/1-226 NNML-----RA----------------------------------K-------WDVLVN-
XP_008674944.1/1-228 NNLL-----RA----------------------------------K-------WDVLVN-
XP_010523539.1/1-228 DARN-----MD----------------------------------L-------WDHMRH-
XP_020873847.1/1-203 GVKK-----RE----------------------------------I-------FNHLRL-
NP_001190248.1/1-203 GAEK-----QE----------------------------------I-------WSYLES-
NP_196343.2/1-203    GAEK-----QE----------------------------------I-------WSYLES-
XP_020876233.1/1-195 RAEK-----QE----------------------------------I-------WDYLES-
XP_010423235.1/1-188 RAKK-----QY----------------------------------I-------WEHLAS-
XP_010452682.1/1-218 RAKK-----QD----------------------------------I-------WENLAS-
XP_010491330.1/1-188 RAKK-----QD----------------------------------I-------WENLAS-
XP_006413300.2/1-224 NPNL-----RD----------------------------------K-------WDLLAT-
XP_022008499.1/1-216 IKDL-----RS----------------------------------V-------WDVLCH-
XP_022008502.1/1-215 IKDL-----RS----------------------------------V-------WDVLCH-
XP_022007635.1/1-226 TKD------RC----------------------------------I-------WDVICH-
XP_022008501.1/1-224 TKD------RC----------------------------------I-------WDVIYH-
XP_021986984.1/1-226 TKD------RC----------------------------------I-------WDVICH-
XP_021996009.1/1-224 TKD------RC----------------------------------I-------WDVICH-
XP_021996011.1/1-224 TKD------RC----------------------------------I-------WDVICH-
XP_022026050.1/1-224 TKD------RC----------------------------------I-------WDVICH-
XP_022026083.1/1-224 TKD------RC----------------------------------I-------WDVICH-
XP_021722389.1/1-209 NEKN-----RT----------------------------------K-------WDALAL-
XP_021847315.1/1-219 NDRN-----RT----------------------------------K-------WDALAL-
XP_020224300.1/1-216 EARK-----RS----------------------------------K-------WDVLSC-
XP_021765248.1/1-224 DYTT-----RS----------------------------------Q-------WDVLCN-
XP_004510020.1/1-219 DETR-----RH----------------------------------E-------WDIVVE-
XP_004487953.1/1-231 DDNT-----RH----------------------------------R-------WDPIAS-
XP_008244376.1/1-218 DVKF-----RR----------------------------------Q-------WDIYGR-
XP_008244380.1/1-218 DVKF-----RR----------------------------------Q-------WDIYGR-
XP_012569676.1/1-224 DPIK-----RP----------------------------------Q-------WDVLSD-
XP_019418393.1/1-229 DANK-----RA----------------------------------Q-------WDLYWR-
XP_019440118.1/1-223 GAEK-----RA----------------------------------K-------WDFHST-
XP_014517363.2/1-226 DPTK-----RA----------------------------------K-------WDVMCC-
XP_012569675.1/1-231 DPSR-----RF----------------------------------E-------WDVICH-
XP_004486570.1/1-223 DPAR-----RF----------------------------------Q-------WDVICN-
XP_003594569.3/1-220 DPAR-----RF----------------------------------E-------WDKFCD-
XP_024632692.1/1-220 DPAR-----RF----------------------------------E-------WDKFCD-
XP_025887127.1/1-229 NEQT-----RS----------------------------------Q-------WDVLYN-
XP_015078684.1/1-230 NEQT-----RS----------------------------------Q-------WDVLYN-
XP_025887126.1/1-230 NEQT-----RS----------------------------------Q-------WDVLYN-
XP_015078401.1/1-220 NERT-----RS----------------------------------Q-------WDVFSN-
XP_015158871.1/1-217 NEQT-----RS----------------------------------Q-------WDVWT--
XP_015078778.1/1-220 NTNL-----RR----------------------------------Q-------WDILSC-
XP_010321992.1/1-216 NKNL-----RR----------------------------------Q-------WDILSC-
XP_004240735.1/1-216 NKNL-----RR----------------------------------Q-------WDILSC-
XP_015169793.1/1-213 NNKT-----RS----------------------------------Q-------WDVLSC-
XP_006285631.1/1-212 HLGS-----RH----------------------------------E-------WDTLT--
XP_023635531.1/1-198 HLGS-----RH----------------------------------E-------WDTLT--
NP_567722.1/1-217    HLSF-----RH----------------------------------E-------WDILT--
XP_020873337.1/1-219 NPSF-----RH----------------------------------V-------WDTLT--
XP_020873338.1/1-219 NPSF-----RH----------------------------------V-------WDTLT--
XP_023000325.1/1-225 NGSR-----RI----------------------------------E-------WDGML--
XP_023000326.1/1-214 NGSR-----RI----------------------------------E-------WDGML--
XP_022963997.1/1-214 NGSR-----RI----------------------------------E-------WDVML--
XP_023513617.1/1-208 NGSR-----RI----------------------------------E-------WDVML--
XP_021312124.1/1-225 DKSR-----RH----------------------------------E-------WDLMM--
XP_012701492.1/1-243 DESR-----RH----------------------------------E-------WDVMLP-
XP_025815339.1/1-229 DESR-----RH----------------------------------E-------WDVMLL-
XP_015644459.1/1-237 DESR-----RP----------------------------------E-------WDVML--
XP_024314016.1/1-228 DESR-----RP----------------------------------E-------WDATL--
XP_020168333.1/1-225 DESR-----RP----------------------------------E-------WDVTL--
XP_013589242.1/1-206 NPSF-----RN----------------------------------Q-------WDILA--
XP_022548456.1/1-204 NPSF-----RN----------------------------------Q-------WDILA--
XP_024632691.1/1-227 DPTK-----RS----------------------------------Q-------WNGLS--
XP_017256377.1/1-220 SGSN-----RS----------------------------------K-------WDLLS--
XP_009632057.1/1-224 AGEN-----RC----------------------------------K-------WDLLS--
XP_016502932.1/1-224 AGEN-----RC----------------------------------K-------WDLLS--
XP_019258997.1/1-221 AGDN-----RC----------------------------------K-------WDLLS--
XP_009792862.1/1-224 AGDN-----RC----------------------------------K-------WDLLS--
XP_016544784.1/1-213 AGEN-----RW----------------------------------K-------WDLLS--
XP_015089689.1/1-213 AGGN-----RW----------------------------------K-------WDLLS--
XP_025883585.1/1-213 AGGN-----RW----------------------------------K-------WDLLS--
XP_011095790.1/1-221 SGYN-----RN----------------------------------K-------WDVLS--
XP_020553953.1/1-221 SGYN-----RN----------------------------------K-------WDVLS--
XP_022857758.1/1-208 CEQN-----RN----------------------------------K-------WDILS--
XP_004487739.1/1-224 HEKS-----RN----------------------------------K-------WDTLS--
XP_003596663.1/1-221 HEKS-----RS----------------------------------K-------WDKHS--
XP_003596665.1/1-227 HEKS-----RS----------------------------------K-------WDKLS--
XP_020201972.1/1-216 HEDS-----RT----------------------------------K-------WDVLS--
XP_025980734.1/1-220 HENS-----RT----------------------------------K-------WDMLS--
XP_007149715.1/1-218 HENS-----RT----------------------------------K-------WDSLS--
XP_017423636.1/1-218 HENS-----RT----------------------------------K-------WDLLS--
XP_022631965.1/1-218 HGNS-----RT----------------------------------K-------WDLLS--
XP_014522034.2/1-218 HGNS-----RT----------------------------------K-------WDLLS--
XP_025669173.1/1-223 HEDS-----RN----------------------------------K-------WDLLS--
XP_020963786.1/1-223 HEDS-----RN----------------------------------K-------WDLLS--
XP_025614692.1/1-223 HEDS-----RN----------------------------------K-------WDILS--
XP_015936351.1/1-223 HEDS-----RN----------------------------------K-------WDILS--
XP_019451675.1/1-226 HGSS-----RK----------------------------------K-------WDLLS--
XP_019465428.1/1-219 HESS-----RK----------------------------------K-------WDLLS--
XP_004295798.2/1-220 DGES-----RY----------------------------------M-------WDMMS--
XP_021807338.1/1-219 DVKF-----RR----------------------------------Q-------WDIYG--
XP_021807340.1/1-219 DVKF-----RR----------------------------------Q-------WDIYG--
XP_020417197.1/1-219 DVKF-----RR----------------------------------Q-------WDIYG--
XP_020417200.1/1-219 DVKF-----RR----------------------------------Q-------WDIYG--
XP_009333923.1/1-206 DEKC-----RN----------------------------------E-------WDMLA--
XP_017184363.1/1-206 DEKX-----RN----------------------------------E-------WDMLA--
XP_021892911.1/1-224 DEKT-----RP----------------------------------M-------WDMIS--
XP_002278971.2/1-210 DVHT-----RN----------------------------------R-------WDILT--
XP_010644412.1/1-211 DENS-----RN----------------------------------K-------WDILT--
XP_024450660.1/1-210 HGDS-----RN----------------------------------K-------LDLLA--
XP_002522867.2/1-214 HEDC-----RN----------------------------------K-------WDLLS--
XP_020534605.1/1-220 RSDS-----RN----------------------------------K-------WDLLS--
XP_021685850.1/1-217 HEDS-----RN----------------------------------K-------WDLLS--
XP_021594464.1/1-220 REDS-----RN----------------------------------K-------WDLLS--
XP_018731177.1/1-223 DATL-----RD----------------------------------K-------WDALA--
XP_024018593.1/1-217 DEES-----RT----------------------------------R-------WDILS--
XP_022139420.1/1-219 HEKS-----RN----------------------------------K-------WDILS--
XP_022981426.1/1-220 HEKS-----RN----------------------------------K-------WDILS--
XP_022981427.1/1-220 HEKS-----RN----------------------------------K-------WDILS--
XP_022941489.1/1-220 HEKS-----RN----------------------------------K-------WDILS--
XP_022941488.1/1-220 HEKS-----RN----------------------------------K-------WDILS--
XP_023525599.1/1-220 HEKS-----RN----------------------------------K-------WDILS--
XP_023525597.1/1-220 HEKS-----RN----------------------------------K-------WDILS--
XP_008454681.2/1-220 HEKS-----RN----------------------------------K-------WDILS--
XP_011648553.1/1-223 HEKS-----RN----------------------------------K-------WDILS--
XP_018822340.1/1-211 HEST-----RS----------------------------------K-------WDILS--
XP_018822367.1/1-211 HEST-----RS----------------------------------K-------WDILS--
XP_023876961.1/1-210 HANS-----RN----------------------------------K-------WDILS--
XP_023876962.1/1-210 HANS-----RN----------------------------------K-------WDILS--
XP_024034242.1/1-215 DEKS-----RT----------------------------------K-------WDTRL--
XP_015381349.2/1-215 DENS-----RT----------------------------------K-------WDTRL--
XP_022717683.1/1-216 HEHS-----RN----------------------------------K-------WDLLS--
XP_016729588.1/1-216 HERT-----RN----------------------------------R-------WDLLS--
XP_016729589.1/1-216 HERT-----RN----------------------------------R-------WDLLS--
XP_016702140.1/1-208 HERT-----RN----------------------------------R-------WDLLS--
XP_012460158.1/1-216 HERT-----RN----------------------------------R-------WDLLS--
XP_012460145.1/1-216 HERT-----RN----------------------------------R-------WDLLS--
XP_017245631.1/1-197 DEKC-----RP----------------------------------Q-------WDVIT--
XP_004956045.3/1-247 NEKL-----RG----------------------------------E-------WDMLT--
XP_014660176.1/1-240 NGQC-----RG----------------------------------E-------WDTFA--
XP_017239609.1/1-213 DHKF-----RK----------------------------------E-------TNIFA--
XP_023740378.1/1-206 NKEL-----RC----------------------------------M-------WDVLC--
XP_020095129.1/1-235 SETS-----RH----------------------------------E-------WDAFY--
XP_020258373.1/1-212 DESS-----RD----------------------------------K-------WDSPS--
XP_008812148.1/1-175 ------------------------------------------------------------
XP_010930674.1/1-230 TSRNEVFRAPS----------------------------------I-------WDVLA--
XP_010931877.1/1-230 TSRNLVFRAPS----------------------------------I-------WDVLA--
XP_009380061.1/1-225 DEQN-----RP----------------------------------Q-------WDVFSI-
XP_010939971.1/1-229 DEKT-----RA----------------------------------E-------WDVLSN-
XP_019710626.1/1-218 DEKT-----RA----------------------------------E--------------
XP_008787891.1/1-229 DEKT-----RA----------------------------------E-------WDVLSN-
XP_008787892.1/1-218 DEKT-----RA----------------------------------E--------------
XP_008799024.1/1-230 DEKT-----RA----------------------------------E-------WDVLSN-
XP_010936845.1/1-230 DAKT-----RA----------------------------------E-------WDVLSN-
XP_003570542.1/1-223 DENT-----RS----------------------------------Q-------WDVLSH-
XP_004964922.1/1-229 DETV-----RS----------------------------------Q-------WDVLSH-
XP_002438026.1/1-230 DENA-----RS----------------------------------Q-------WDVLSH-
XP_020399989.1/1-232 DENA-----RS----------------------------------Q-------WDVLSH-
XP_008649207.1/1-222 DENA-----RS----------------------------------Q-------WDVLSH-
XP_020256822.1/1-228 DERT-----RA----------------------------------Q-------WDVLSN-
XP_009403550.1/1-228 DERT-----RT----------------------------------Q-------WHILSN-
XP_020582158.1/1-232 DEQS-----RV----------------------------------Q-------WDVLSR-
XP_020699118.1/1-240 DERS-----RV----------------------------------Q-------WDVLSH-
XP_020600375.1/1-232 DERS-----RV----------------------------------Q-------WDVLSN-
XP_020600376.1/1-232 DERS-----RV----------------------------------Q-------WDVLSN-
XP_020684394.1/1-232 DERS-----RV----------------------------------Q-------WDVLSN-
XP_017257147.1/1-219 DERS-----RP----------------------------------Q-------WDVLSN-
XP_010244428.1/1-230 DERN-----RA----------------------------------Q-------WDLLSH-
XP_010244355.1/1-230 DERN-----RA----------------------------------Q-------WDLLSH-
XP_017240757.1/1-236 DERT-----RA----------------------------------Q-------WDVLSD-
XP_017245021.1/1-233 DERT-----RP----------------------------------Q-------WDVLSD-
XP_012829269.1/1-233 DERN-----RP----------------------------------Q-------WDVMSN-
XP_022889938.1/1-236 DERT-----RP----------------------------------Q-------WDVLSN-
XP_022897471.1/1-232 DERT-----RP----------------------------------Q-------WDVLSN-
XP_009606250.1/1-235 DEMT-----RP----------------------------------Q-------WDVLSN-
XP_016468230.1/1-235 DEMT-----RP----------------------------------Q-------WDVLSN-
XP_019250773.1/1-235 DERT-----RP----------------------------------Q-------WDVLSN-
XP_009778533.1/1-235 DERT-----RP----------------------------------Q-------WDVLSN-
XP_016451582.1/1-235 DERT-----RP----------------------------------Q-------WDVLSN-
XP_016578176.1/1-240 DERT-----RS----------------------------------Q-------WDVLSN-
XP_006347370.1/1-231 DERT-----RP----------------------------------Q-------WDVLSN-
XP_004241474.1/1-230 DERT-----RP----------------------------------Q-------WDVLSN-
XP_015077748.1/1-230 DERT-----RP----------------------------------Q-------WDVLSN-
XP_022037776.1/1-241 DGRT-----RP----------------------------------Q-------WDVLSN-
XP_024992062.1/1-237 DGRT-----RP----------------------------------Q-------WDVLSN-
XP_023772270.1/1-245 DGRT-----RP----------------------------------Q-------WDVLSN-
XP_012855673.1/1-237 DERT-----RP----------------------------------Q-------WDVLSN-
XP_022858328.1/1-236 DERT-----RP----------------------------------R-------WDVLSN-
XP_011072039.1/1-232 DERS-----RP----------------------------------Q-------WDVLSN-
XP_006306921.1/1-215 DERT-----RP----------------------------------Q-------WDVLSN-
NP_564041.2/1-215    DERT-----RP----------------------------------Q-------WDVLSN-
XP_020866823.1/1-216 DERT-----RP----------------------------------Q-------WDVLSN-
XP_010476956.1/1-215 DERT-----RP----------------------------------Q-------WDVLSN-
XP_010498167.1/1-217 DERT-----RP----------------------------------Q-------WDVLSN-
XP_019091528.1/1-217 DERT-----RP----------------------------------Q-------WDVLSN-
XP_013613976.1/1-227 DERT-----RP----------------------------------Q-------WDVLLN-
XP_018432674.1/1-227 DERT-----RP----------------------------------Q-------WDVLLD-
XP_018432667.1/1-227 DERT-----RP----------------------------------Q-------WDVLLD-
XP_013641731.1/1-225 DERT-----RP----------------------------------Q-------WDVLLN-
XP_009149243.1/1-227 DERT-----RP----------------------------------Q-------WDVLLN-
XP_006416656.2/1-224 DERT-----RP----------------------------------Q-------WDVLSN-
XP_018451633.1/1-224 DERT-----RP----------------------------------Q-------WDVLLN-
XP_018451632.1/1-225 DERT-----RP----------------------------------Q-------WDVLLN-
XP_018451634.1/1-195 DERT-----RP----------------------------------Q-------WDVLLN-
XP_013690958.1/1-228 DERT-----RP----------------------------------Q-------WDVLLN-
XP_009110376.1/1-227 DERT-----RP----------------------------------Q-------WDVLLN-
XP_009110377.1/1-227 DERT-----RP----------------------------------Q-------WDVLLN-
XP_013601964.1/1-227 DERT-----RP----------------------------------Q-------WDVLLN-
XP_013653874.1/1-227 DERT-----RP----------------------------------Q-------WDVLLN-
XP_018445004.1/1-237 DERT-----RP----------------------------------Q-------WDVLSN-
XP_018445005.1/1-237 DERT-----RP----------------------------------Q-------WDVLSN-
XP_013648946.1/1-228 DERT-----RP----------------------------------Q-------WDVLSN-
XP_009104812.1/1-228 DERT-----RP----------------------------------Q-------WDVLSN-
XP_013588523.1/1-228 DERT-----RP----------------------------------Q-------WDVLSN-
XP_013699248.1/1-228 DERT-----RP----------------------------------Q-------WDVLSN-
XP_018457296.1/1-228 DERT-----RP----------------------------------Q-------WDVLSN-
XP_009105998.1/1-230 DERT-----RP----------------------------------Q-------WDVLSN-
NP_001302850.1/1-231 DERI-----RP----------------------------------Q-------WDVLSN-
XP_013698656.1/1-231 DERT-----RP----------------------------------Q-------WDVLSN-
XP_013649619.1/1-231 DERT-----RP----------------------------------Q-------WDVLSN-
XP_013592873.1/1-231 DERT-----RP----------------------------------Q-------WDVLSN-
XP_006390565.1/1-233 DERT-----RP----------------------------------Q-------WDVLSN-
XP_006301097.1/1-230 DERT-----RP----------------------------------Q-------WDVLSN-
XP_010428260.1/1-231 DERT-----RP----------------------------------Q-------WDVLSN-
XP_010471396.1/1-230 DERT-----RP----------------------------------Q-------WDVLSN-
XP_010416135.1/1-230 DERT-----RP----------------------------------Q-------WDVLSN-
NP_177479.1/1-227    DERT-----RP----------------------------------Q-------WDVLSN-
XP_020890987.1/1-228 DERT-----RP----------------------------------Q-------WDVLSN-
XP_010537520.1/1-232 DERT-----RP----------------------------------Q-------WDVLSN-
XP_010540042.1/1-226 DERN-----RP----------------------------------Q-------WDVLSN-
XP_010540046.1/1-196 DERN-----RP----------------------------------Q--------------
XP_019432660.1/1-235 DGRN-----RP----------------------------------K-------WDVLAN-
XP_019420678.1/1-233 DEKR-----RP----------------------------------Q-------WDVLSN-
XP_013452816.1/1-243 DERK-----RS----------------------------------Q-------WDVLSN-
XP_020215886.1/1-221 DERK-----RP----------------------------------Q-------WDVLSN-
XP_020215888.1/1-221 DERK-----RP----------------------------------Q-------WDVLSN-
XP_007160572.1/1-230 DETK-----RP----------------------------------Q-------WDVLSN-
XP_017424661.1/1-212 DETK-----RP----------------------------------Q-------WDVLSN-
XP_017424660.1/1-230 DETK-----RP----------------------------------Q-------WDVLSN-
XP_014508329.1/1-230 DETK-----RP----------------------------------Q-------WDVLSN-
XP_022632295.1/1-230 DETK-----RP----------------------------------Q-------WDVLSN-
XP_019225984.1/1-234 DERT-----RP----------------------------------Q-------WDVLSN-
XP_016441514.1/1-233 DERN-----RP----------------------------------Q-------WDVLSN-
XP_009587298.1/1-233 DERN-----RP----------------------------------Q-------WDVLSN-
XP_016441515.1/1-233 DERN-----RP----------------------------------Q-------WDVLSN-
XP_009786509.1/1-233 DERT-----RP----------------------------------Q-------WDVLSN-
XP_016563952.1/1-234 DERT-----RP----------------------------------Q-------WDVLSN-
XP_004235382.1/1-234 DERT-----RP----------------------------------Q-------WDVLSN-
XP_015068940.1/1-234 DERT-----RP----------------------------------Q-------WDVLSN-
XP_006364383.1/1-234 DERT-----RP----------------------------------Q-------WDVLSN-
XP_019167409.1/1-245 DERT-----RP----------------------------------Q-------WDVLSN-
XP_011086973.1/1-238 DERT-----RP----------------------------------Q-------WDVLSN-
XP_010268321.1/1-230 DERT-----RA----------------------------------Q-------WDVLSN-
XP_010268322.1/1-230 DERT-----RA----------------------------------Q-------WDVLSN-
XP_010069492.1/1-236 DERT-----RA----------------------------------Q-------WDVLSN-
XP_002527933.1/1-229 DERT-----RA----------------------------------Q-------WDVLSS-
XP_021678368.1/1-229 DERT-----RA----------------------------------Q-------WDVLSS-
XP_021678369.1/1-229 DERT-----RA----------------------------------Q-------WDVLSS-
XP_021602738.1/1-229 DERT-----RS----------------------------------Q-------WDVLSS-
XP_012079215.1/1-230 DERT-----RA----------------------------------Q-------WDVLSS-
XP_021687701.1/1-229 DERT-----RP----------------------------------Q-------WDVLSS-
XP_006374212.1/1-231 DERT-----RP----------------------------------Q-------WDVLSS-
XP_011035552.1/1-231 DERT-----RP----------------------------------Q-------WDVLSS-
XP_002317722.3/1-231 DEKT-----RP----------------------------------Q-------WDVLSS-
XP_011000766.1/1-231 DEKT-----RP----------------------------------Q-------WDVLSS-
XP_021907827.1/1-230 DERT-----RP----------------------------------Q-------WDVLSN-
XP_017635401.1/1-230 DERT-----RP----------------------------------Q-------WDVLSN-
XP_016686699.1/1-230 DERT-----RP----------------------------------Q-------WDVLSN-
XP_012440361.1/1-230 DERT-----RP----------------------------------Q-------WDVLSN-
NP_001313868.1/1-230 DERT-----RP----------------------------------Q-------WDVLSS-
XP_023919022.1/1-230 DERT-----RP----------------------------------Q-------WDVLSN-
XP_006478112.1/1-232 DERT-----RP----------------------------------Q-------WDVLTN-
XP_024042646.1/1-232 DERT-----RP----------------------------------Q-------WDVLTN-
XP_022715403.1/1-231 DERT-----RP----------------------------------Q-------WDVLSN-
XP_022767009.1/1-255 DERT-----RP----------------------------------Q-------WDVLSN-
XP_022737050.1/1-231 DERT-----RP----------------------------------Q-------WDVLSN-
XP_017973835.1/1-231 DERT-----RP----------------------------------Q-------WDVLSN-
XP_015967008.1/1-238 DERK-----RP----------------------------------Q-------WDVLSN-
XP_025701434.1/1-239 DERK-----RP----------------------------------Q-------WDVLSN-
XP_020980110.1/1-239 DERK-----RP----------------------------------Q-------WDVLSN-
XP_019434881.1/1-231 DDRK-----RP----------------------------------Q-------WDVLSN-
XP_019458275.1/1-234 DERK-----RP----------------------------------Q-------WDVLSN-
XP_019458276.1/1-234 DERK-----RP----------------------------------Q-------WDVLSN-
XP_006584922.1/1-234 DENK-----RP----------------------------------Q-------WDVLSN-
XP_003524332.1/1-231 DENK-----RP----------------------------------Q-------WDVLSN-
XP_004515249.1/1-229 DERK-----RS----------------------------------Q-------WDVLSN-
XP_019431381.1/1-229 DERK-----RP----------------------------------Q-------WDVLSN-
XP_019436771.1/1-231 DERK-----RP----------------------------------Q-------WDVLSN-
XP_019430342.1/1-233 DERK-----RP----------------------------------Q-------WDVLSN-
XP_006587436.1/1-240 DEKK-----RP----------------------------------Q-------WDVLSN-
XP_003549189.1/1-236 DEKK-----RP----------------------------------Q-------WDVLSN-
XP_020221986.1/1-232 DEKK-----RP----------------------------------Q-------WDVLSN-
XP_007152598.1/1-234 DEKK-----RP----------------------------------Q-------WDVLSN-
XP_017437542.1/1-234 DEKK-----RP----------------------------------Q-------WDVLSN-
XP_014505752.1/1-234 DEKK-----RP----------------------------------Q-------WDVLSN-
XP_015881991.1/1-234 DERT-----RS----------------------------------Q-------WDVLSN-
XP_004299483.1/1-232 DERT-----RP----------------------------------Q-------WDVLSN-
XP_024174647.1/1-232 DERT-----RP----------------------------------Q-------WDVLSN-
XP_008375041.1/1-227 DERT-----RP----------------------------------Q-------WDVLSN-
XP_017188172.1/1-199 DERT-----RP----------------------------------Q-------WDVLSN-
XP_009355964.1/1-227 DERT-----RP----------------------------------Q-------WDVLSN-
XP_009355965.1/1-199 DERT-----RP----------------------------------Q-------WDVLSN-
XP_023523434.1/1-228 NDRT-----RS----------------------------------Q-------WDVLSD-
XP_022949138.1/1-227 NDRT-----RS----------------------------------Q-------WDVLSD-
XP_022998050.1/1-227 NDRT-----RS----------------------------------Q-------WDVLSD-
XP_002271012.1/1-231 DERT-----RP----------------------------------Q-------WDVLSN-
XP_010663934.1/1-231 DERT-----RP----------------------------------Q-------WDVLSN-
XP_010099679.1/1-237 DERT-----RS----------------------------------Q-------WDVLSN-
XP_018823334.1/1-236 DERT-----RA----------------------------------Q-------WDVLSN-
XP_023001145.1/1-228 NDRT-----RS----------------------------------Q-------WDVLSD-
XP_023519889.1/1-228 NDRT-----RS----------------------------------Q-------WDVLSD-
XP_022924191.1/1-228 NDRT-----RS----------------------------------Q-------WDVLSD-
XP_018849090.1/1-235 DERT-----RG----------------------------------Q-------WDVLSN-
XP_008240378.1/1-228 DERT-----RP----------------------------------Q-------WDVLSN-
XP_007210460.2/1-228 DERT-----RP----------------------------------Q-------WDVLSN-
XP_021801797.1/1-228 DERT-----RP----------------------------------Q-------WDVLSN-
XP_022136988.1/1-228 NDRT-----RS----------------------------------Q-------WDVLSD-
XP_016898817.1/1-228 NDRT-----RS----------------------------------Q-------WDVLSD-
XP_004137568.1/1-228 NDRT-----RS----------------------------------Q-------WDVLSE-
XP_015642098.1/1-238 DENT-----RS----------------------------------Q-------WDVLSH-
XP_020186741.1/1-220 DENT-----RS----------------------------------Q-------WDVLSH-
XP_025828617.1/1-229 DENT-----RS----------------------------------Q-------WDVLSH-
XP_004954072.1/1-226 DENT-----RS----------------------------------Q-------WDVLSH-
XP_002452898.1/1-231 DEHT-----RS----------------------------------Q-------WDVLSH-
NP_001142912.2/1-230 DEHT-----RS----------------------------------Q-------WDVLSH-
XP_020089796.1/1-231 DENM-----RP----------------------------------Q-------WDVLSS-
XP_020089795.1/1-241 DENM-----RP----------------------------------Q-------WDVLSS-
XP_019708739.1/1-228 DEHT-----RA----------------------------------Q-------WDVLSS-
XP_008789232.1/1-228 DEHT-----RA----------------------------------Q-------WDVLSS-
XP_008789231.1/1-228 DEHT-----RA----------------------------------Q-------WDVLSS-
XP_008789233.1/1-200 DEHT-----RA----------------------------------Q-------WDVLSS-
XP_019704584.1/1-228 DERT-----RA----------------------------------Q-------WDVLSS-
XP_010917295.1/1-228 DERT-----RA----------------------------------Q-------WDVLSS-
XP_008806630.1/1-228 DERT-----RA----------------------------------Q-------WDVLSS-
XP_009388746.1/1-226 DERT-----RS----------------------------------Q-------WDVLSN-
XP_018675467.1/1-228 DERT-----RT----------------------------------Q-------WDVLSN-
XP_018675466.1/1-228 DERT-----RT----------------------------------Q-------WDVLSN-
XP_009409108.1/1-228 DEQT-----RT----------------------------------Q-------WDVLAN-
XP_024625408.1/1-220 DERK-----RS----------------------------------Q-------SNVLSN-
XP_010550261.1/1-219 DVHL-----RH----------------------------------Q-------WDVLCH-
XP_018816719.1/1-228 EEKM-----RV----------------------------------Q-------WDVLSD-
XP_008365756.2/1-218 DEKT-----RV----------------------------------Q-------WDVLSN-
XP_008381522.1/1-218 DEKT-----RV----------------------------------Q-------WDVLSN-
XP_018506060.1/1-218 DEKT-----RI----------------------------------Q-------WDVLSH-
XP_008362571.1/1-218 DEKT-----RI----------------------------------Q-------WDVLSH-
XP_023870350.1/1-229 EEKT-----RV----------------------------------Q-------WDVLSS-
XP_016649213.1/1-222 DEET-----RL----------------------------------Q-------WDVLCN-
XP_020415996.1/1-222 DEET-----RL----------------------------------Q-------WDVLCN-
XP_021823813.1/1-222 DEET-----RL----------------------------------Q-------WDVLCN-
XP_009367126.1/1-222 DEKN-----RV----------------------------------Q-------WDVLSN-
XP_008348615.1/1-222 DEKN-----RV----------------------------------Q-------WDVLSN-
XP_017179578.1/1-222 DERN-----RV----------------------------------Q-------WDVLSN-
XP_009370612.1/1-224 DERN-----RV----------------------------------Q-------WDVLSN-
XP_012845089.1/1-228 DEKK-----RA----------------------------------Q-------WDILSS-
XP_012845088.1/1-228 DEKK-----RA----------------------------------Q-------WDILSS-
XP_012845087.1/1-228 DEKK-----RA----------------------------------Q-------WDILSS-
XP_011085930.1/1-227 DEKK-----RV----------------------------------Q-------WDVLSS-
XP_020551334.1/1-222 DEKK-----RV----------------------------------Q-------WDVLSS-
XP_021297998.1/1-224 DEKV-----RF----------------------------------Q-------WDVLCH-
XP_017974480.1/1-224 DEKV-----RF----------------------------------Q-------WDVLCH-
XP_024448785.1/1-229 DEKS-----RI----------------------------------Q-------WDILSN-
XP_024455385.1/1-229 DEKS-----RI----------------------------------Q-------WDILSN-
XP_011005937.1/1-269 DERS-----RIQVYE------------------------------K-------WDVLSN-
XP_011038413.1/1-229 AEKS-----RT----------------------------------Q-------WDILSN-
XP_018731153.1/1-226 NENT-----RA----------------------------------Q-------WDVLCN-
XP_024190515.1/1-228 NEKT-----RV----------------------------------Q-------WDVLSN-
XP_012074722.2/1-231 DEKT-----RV----------------------------------Q-------WDILST-
XP_010089514.2/1-229 DDKT-----RF----------------------------------Q-------WDVLSN-
XP_024949330.1/1-235 DEKM-----RA----------------------------------Q-------WDVLSN-
XP_006421085.1/1-235 DEKM-----RV----------------------------------Q-------WDVLSN-
XP_002268272.1/1-229 DEKM-----RP----------------------------------Q-------WDVLSS-
XP_004140733.2/1-228 DEKA-----RV----------------------------------Q-------WDVLSY-
XP_008457114.1/1-228 DEKA-----RV----------------------------------Q-------WDVLSY-
XP_008457113.1/1-229 DEKA-----RV----------------------------------Q-------WDVLSY-
XP_011651208.1/1-229 DEKA-----RV----------------------------------Q-------WDVLSY-
XP_022143367.1/1-228 DEKA-----RV----------------------------------Q-------WDVLSN-
XP_022935490.1/1-228 DEKA-----RV----------------------------------Q-------WDVLSN-
XP_022984101.1/1-228 DEKA-----RV----------------------------------Q-------WDVLSN-
XP_022935488.1/1-229 DEKA-----RV----------------------------------Q-------WDVLSN-
XP_022984098.1/1-229 DEKA-----RV----------------------------------Q-------WDVLSN-
XP_022984102.1/1-229 DEKA-----RV----------------------------------Q-------WDVLSN-
XP_015579295.1/1-234 DEKT-----RV----------------------------------Q-------WDILSN-
XP_002526595.2/1-234 DEKT-----RV----------------------------------Q-------WDILSN-
XP_015579294.1/1-234 DEKT-----RV----------------------------------Q-------WDILSN-
XP_021643520.1/1-232 DEKT-----RV----------------------------------Q-------WDILSN-
XP_021605433.1/1-231 DEKS-----RP----------------------------------Q-------WDILSS-
XP_023739098.1/1-228 DEKN-----RP----------------------------------K-------WDVLTN-
XP_024966239.1/1-227 NESN-----RH----------------------------------Q-------WDVLTN-
XP_025813558.1/1-232 DESA-----RS----------------------------------Q-------WDILAH-
XP_020150479.1/1-234 DENT-----RT----------------------------------Q-------WDVLCK-
XP_003564122.2/1-224 DVNT-----RN----------------------------------Q-------WDVLTH-
XP_006585049.2/1-225 DNKK-----RA----------------------------------K-------WEPFWY-
XP_007143783.1/1-226 DDRK-----RA----------------------------------K-------WDVLCY-
XP_007158859.1/1-226 DDRK-----RA----------------------------------K-------WDVLCS-
XP_020230815.1/1-227 DPIR-----RA----------------------------------K-------WDVMCY-
XP_006586851.1/1-227 DPIR-----RV----------------------------------K-------WDAMCY-
XP_021888058.1/1-234 DEKM-----RG----------------------------------K-------WDIMCY-
XP_020886111.1/1-226 NLDT-----RQ----------------------------------Q-------WDILSY-
XP_020886110.1/1-228 NLDT-----RQ----------------------------------Q-------WDILSY-
XP_011469296.1/1-224 NEQT-----RE----------------------------------Q-------WDVLSN-
XP_009367128.2/1-218 DEKT-----RV----------------------------------Q-------WDVLSN-
XP_008348673.1/1-198 DEKT-----RV----------------------------------Q-------WDVLSN-
XP_012832506.1/1-227 DEKR-----RA----------------------------------Q-------WDVLSN-
XP_012832508.1/1-227 DEKR-----RA----------------------------------Q-------WDVLSN-
XP_012832516.1/1-227 DEKR-----RA----------------------------------Q-------WDALSN-
XP_012832518.1/1-227 DEKR-----RA----------------------------------Q-------WDALSN-
XP_012835569.1/1-227 DEKR-----RA----------------------------------L-------WDVLSN-
XP_012835571.1/1-226 DEKR-----RA----------------------------------L-------WDVLSN-
XP_019172066.1/1-226 DDQL-----RA----------------------------------H-------WDVLSN-
XP_009631364.2/1-226 DEKT-----RT----------------------------------Q-------WDVLTN-
XP_016435660.1/1-229 DDKT-----RP----------------------------------Q-------WDVLTN-
XP_009771801.1/1-226 DDKT-----RT----------------------------------Q-------WDVLTN-
XP_016493115.1/1-226 DDKT-----RT----------------------------------Q-------WDVLTN-
XP_019248352.1/1-226 DDKT-----RT----------------------------------Q-------WDVLTN-
XP_025691463.1/1-225 DPST-----RH----------------------------------Q-------WDVLCH-
XP_020995109.1/1-225 DPST-----RH----------------------------------Q-------WDVLCH-
XP_016190415.2/1-225 DPST-----RQ----------------------------------Q-------WDVLCH-
XP_004486568.1/1-232 DPTK-----RS----------------------------------Q-------WDVLSC-
XP_024632687.1/1-226 DPTK-----RS----------------------------------Q-------WDGLSC-
XP_020977993.1/1-241 DINK-----RS----------------------------------Q-------WDVLFC-
XP_025647253.1/1-241 DINK-----RS----------------------------------Q-------WDVLFC-
XP_020997095.1/1-240 DINK-----RS----------------------------------Q-------WDVLFC-
XP_025692389.1/1-228 DINK-----RS----------------------------------Q-------WDVLFC-
XP_019418389.1/1-226 DDTK-----RF----------------------------------Q-------WDVLSY-
XP_007147460.1/1-227 DDKN-----RA----------------------------------Q-------WDVLTC-
XP_014517364.1/1-227 DDKN-----RA----------------------------------Q-------WDVLTC-
XP_017435438.1/1-226 DDKN-----RA----------------------------------Q-------WDVLTC-
XP_017435439.1/1-217 DDKN-----RA-------------------------------------------------
XP_025981664.1/1-227 DDRR-----RA----------------------------------Q-------WDVLCC-
XP_006586853.1/1-227 DDRR-----RA----------------------------------Q-------WDVLCF-
XP_020230831.1/1-226 DDKR-----RA----------------------------------Q-------WDVLCC-
XP_004489663.1/1-217 DPTK-----RS----------------------------------K-------WDVLAY-
XP_018489833.1/1-227 SNSI-----RH----------------------------------K-------WDALCH-
XP_013584487.1/1-219 SNDT-----RH----------------------------------Q-------WDVLCR-
XP_009130351.1/1-219 SNDT-----RH----------------------------------Q-------WDVLCR-
XP_013747906.2/1-219 SKDT-----RH----------------------------------Q-------WDVLCR-
XP_013747661.1/1-219 SKDT-----RH----------------------------------Q-------WDVLCR-
XP_013619678.1/1-219 SNDT-----RH----------------------------------Q-------WDVLCH-
XP_022551084.1/1-219 SNDT-----RH----------------------------------Q-------WDVLCH-
XP_009126392.1/1-219 SNDT-----RH----------------------------------Q-------WDVLCH-
XP_018437612.1/1-222 NNET-----RH----------------------------------Q-------WDVLCY-
XP_013591191.1/1-225 NNET-----RH----------------------------------Q-------WDVLCY-
XP_022567005.1/1-225 NNET-----RH----------------------------------Q-------WDVLCY-
XP_022547796.1/1-225 NNET-----RH----------------------------------Q-------WDVLCY-
XP_009119205.1/1-225 NNET-----RH----------------------------------Q-------WDVLCY-
XP_009119199.1/1-225 NNET-----RH----------------------------------Q-------WDVLCY-
XP_006408322.1/1-232 DNNT-----RH----------------------------------Q-------WDVLCY-
XP_023642295.1/1-229 KLDN-----RH----------------------------------Q-------WDVLSY-
XP_023642294.1/1-229 KLDN-----RH----------------------------------Q-------WDVLSY-
NP_186976.2/1-228    NLDT-----RQ----------------------------------Q-------WDILSY-
XP_010507998.1/1-229 KLDP-----RQ----------------------------------Q-------WDVLAY-
XP_010485661.1/1-229 KLDT-----RE----------------------------------Q-------WDVLAY-
XP_010450732.1/1-230 KLDT-----RQ----------------------------------Q-------WDVLAY-
XP_010463776.1/1-230 KLDT-----RQ----------------------------------Q-------WDVLAY-
XP_010463774.1/1-230 KLDT-----RQ----------------------------------Q-------WDVLAY-
XP_019092960.1/1-229 KLDT-----RQ----------------------------------Q-------WDVLAY-
XP_022551077.1/1-228 NLEI-----RH----------------------------------Q-------WDVLCH-
XP_013619236.1/1-228 NLEI-----RH----------------------------------Q-------WDVLCH-
XP_009126177.1/1-226 NLEI-----RH----------------------------------Q-------WDVLCH-
XP_013652421.1/1-226 NLEI-----RH----------------------------------Q-------WDVLCH-
XP_013652420.1/1-226 NLEI-----RH----------------------------------Q-------WDVLCH-
XP_006400265.1/1-228 NLDV-----RH----------------------------------Q-------WDVLCQ-
XP_013590801.1/1-226 NLEV-----RH----------------------------------Q-------WDVLCH-
XP_013651910.1/1-222 NLEV-----RH----------------------------------Q-------WDVLCH-
XP_009103958.1/1-222 NLEV-----RH----------------------------------Q-------WDVLCH-
XP_018444485.1/1-226 SLEV-----RH----------------------------------Q-------WDALCQ-
XP_009121397.1/1-226 NLEV-----RH----------------------------------Q-------WDVLCQ-
XP_013659428.1/1-226 NLEV-----RH----------------------------------Q-------WDVLCQ-
XP_022565333.1/1-226 NLEV-----RH----------------------------------Q-------WDVLCQ-
XP_013610327.1/1-226 NLEV-----RH----------------------------------Q-------WDVLCQ-
XP_006286655.1/1-225 NLEV-----RH----------------------------------Q-------WDVLCQ-
XP_010492677.1/1-224 NLDI-----RH----------------------------------Q-------WDVLCN-
XP_010420445.1/1-224 NLNI-----RH----------------------------------Q-------WDVLCN-
XP_010453918.1/1-224 NLNI-----RH----------------------------------Q-------WDVLCN-
XP_006303739.1/1-224 NLEV-----RN----------------------------------Q-------WDVLCH-
XP_020876074.1/1-222 NLEV-----RH----------------------------------Q-------WDVLCH-
NP_197234.1/1-222    NLEV-----RH----------------------------------Q-------WDVLCH-
NP_174724.1/1-221    NLEV-----RH----------------------------------Q-------WDVHCQ-
XP_020869876.1/1-222 NLEV-----RH----------------------------------Q-------WDVLCH-
XP_019091098.1/1-222 NLEV-----RH----------------------------------Q-------WDVLCH-
XP_010478910.1/1-223 NLEV-----RH----------------------------------Q-------WDVLCH-
XP_010501781.1/1-221 NLEV-----RH----------------------------------Q-------WDVLCH-
XP_015089790.1/1-222 DDKT-----RY----------------------------------Q-------WDVFAE-
XP_015089784.1/1-222 DDKT-----RY----------------------------------Q-------WDVFAE-
XP_016549855.1/1-224 DDKT-----RH----------------------------------E-------WDLLTS-
XP_015057403.1/1-221 DDKT-----RS----------------------------------K-------WDILTG-
XP_019066399.1/1-221 DNKT-----RS----------------------------------K-------WDILTG-
XP_015166059.1/1-216 DDKT-----RS----------------------------------Q-------WDILTG-
XP_019071012.2/1-216 DDKT-----RS----------------------------------Q-------WDILTG-
XP_015086709.1/1-216 DDKT-----RS----------------------------------Q-------WDILTG-
XP_015087432.1/1-219 DDKT-----RN----------------------------------Q-------WDVLTG-
XP_004247342.2/1-219 DDKT-----RN----------------------------------Q-------WDILIG-
XP_025888717.1/1-219 DDKT-----RN----------------------------------Q-------WDILIG-
XP_015087599.1/1-221 DDKT-----RN----------------------------------Q-------WDILTG-
XP_019071156.1/1-221 DDKT-----RN----------------------------------Q-------WDILTG-
XP_019070762.2/1-220 DYKT-----RN----------------------------------Q-------WDILTG-
XP_015087071.1/1-220 DDKT-----RN----------------------------------Q-------WDILTG-
XP_025888845.1/1-217 DDKT-----RN----------------------------------Q-------WDILTG-
XP_015170367.1/1-232 DDNT-----RC----------------------------------Q-------WDVLTV-
XP_025886268.1/1-232 DNNS-----RC----------------------------------QVCFIILIWDVLAG-
XP_015072491.1/1-224 DNNS-----RC----------------------------------Q-------WDVLAG-
XP_010327374.2/1-219 DINT-----RY----------------------------------Q-------WDAFSG-
XP_015089749.1/1-219 DINT-----RY----------------------------------Q-------WDAFSG-
XP_015170971.1/1-209 DDNT-----RC----------------------------------Q-------WDVLCG-
XP_025887726.1/1-223 DDNT-----RC----------------------------------Q-------WDVLYG-
XP_006586358.1/1-227 DNNK-----RA----------------------------------K-------WDTLCD-
XP_014617351.1/1-228 DPIR-----RV----------------------------------K-------WDAMCY-
XP_007147461.1/1-227 DPIK-----RA----------------------------------K-------WDVMCY-
XP_017435408.1/1-226 DPTK-----RA----------------------------------K-------WDVMCY-
XP_017215828.1/1-223 DETR-----RN----------------------------------E-------WQTIVN-
XP_012856110.1/1-229 DENR-----RN----------------------------------E-------WDIMSN-
XP_023738891.1/1-226 DETR-----RN----------------------------------E-------WDIMSN-
XP_022017964.1/1-227 DDNQ-----RN----------------------------------E-------WDIMSS-
XP_022154996.1/1-226 DEAR-----RL----------------------------------E-------WDVMSG-
XP_008462169.1/1-225 DEAR-----RP----------------------------------E-------WDVMLS-
XP_004141841.1/1-226 DEAR-----RP----------------------------------E-------WDVMLS-
XP_020098514.1/1-228 DESR-----RT----------------------------------E-------WDITLS-
XP_020098513.1/1-228 DESR-----RT----------------------------------E-------WDITLS-
XP_020263950.1/1-229 DESR-----RT----------------------------------E-------WDIILS-
XP_020263948.1/1-255 DESR-----RT----------------------------------E-------WDIILS-
XP_009415234.2/1-232 DDSR-----RT----------------------------------E-------WDIMLT-
XP_008789333.1/1-230 DESR-----RT----------------------------------E-------WDIMLT-
XP_010920420.1/1-235 DESR-----RN----------------------------------E-------WDIMLA-
XP_010920411.1/1-235 DESR-----RN----------------------------------E-------WDIMLA-
XP_010920404.1/1-235 DESR-----RN----------------------------------E-------WDIMLA-
XP_010521515.1/1-219 DETR-----RH----------------------------------E-------WDILTN-
XP_013652056.1/1-227 DETR-----RH----------------------------------E-------WDALSN-
XP_009104428.1/1-227 DETR-----RH----------------------------------E-------WDALSN-
XP_018445476.1/1-226 DESR-----RH----------------------------------E-------WDVLSN-
XP_013590784.1/1-226 DETR-----RH----------------------------------E-------WDALSN-
XP_013680822.1/1-226 DETR-----RH----------------------------------E-------WDALSN-
XP_006389720.2/1-226 DEAR-----RH----------------------------------E-------WDTLSS-
XP_023642973.1/1-226 DEAR-----RH----------------------------------E-------WDALSN-
XP_020891640.1/1-226 DEAR-----RH----------------------------------E-------WDGLSN-
NP_001320267.1/1-226 DEAR-----RH----------------------------------E-------WDALSN-
NP_565223.1/1-226    DEAR-----RH----------------------------------E-------WDALSN-
NP_001185443.1/1-226 DEAR-----RH----------------------------------E-------WDALSN-
XP_010473021.1/1-226 DEAR-----RH----------------------------------E-------WDALSN-
XP_010430051.1/1-226 DEAR-----RH----------------------------------E-------WDALSN-
XP_025617667.1/1-241 DENH-----RS----------------------------------Q-------WDIMVT-
XP_015938429.1/1-242 DENH-----RS----------------------------------Q-------WDIMVT-
XP_016176999.1/1-241 DENH-----RS----------------------------------Q-------WDIMVT-
XP_021862846.1/1-225 DDAH-----RH----------------------------------E-------WDVMAK-
XP_022979290.1/1-231 DQAC-----RH----------------------------------Q-------WDVMFG-
XP_022955725.1/1-233 DQAR-----AI----------------------------------R-------WDAMFG-
XP_022998610.1/1-231 DDAR-----RN----------------------------------E-------WDAMFG-
XP_022998611.1/1-227 DDAR-----RN----------------------------------E-------WDAMFG-
XP_023523626.1/1-229 DEAR-----RN----------------------------------E-------WDAMFG-
XP_022948296.1/1-231 DEAR-----RN----------------------------------E-------WDAMFG-
XP_023523628.1/1-225 DEAR-----RN----------------------------------E-------WDAMFG-
XP_022948295.1/1-227 DEAR-----RN----------------------------------E-------WDAMFG-
XP_010249693.1/1-228 DDSR-----RH----------------------------------E-------WDIMLT-
XP_010254081.1/1-230 DEAQ-----RT----------------------------------E-------WDIMST-
XP_003529763.1/1-227 DEAR-----RN----------------------------------E-------WDIMSS-
XP_003533048.1/1-226 DEAR-----RS----------------------------------E-------WDIMSS-
XP_014634625.1/1-226 DEAR-----RS----------------------------------E-------WDIMSS-
XP_007150635.1/1-226 DETR-----RS----------------------------------E-------WDIMSS-
XP_014498860.1/1-226 DETR-----RS----------------------------------E-------WDIMSS-
XP_017425661.1/1-226 DETR-----RS----------------------------------E-------WDIMSS-
XP_020207725.1/1-226 DEAR-----RT----------------------------------E-------WDIMSS-
XP_003546423.2/1-226 DETR-----RT----------------------------------E-------WDIMSS-
XP_003543638.1/1-226 DENR-----RT----------------------------------E-------WDIMSS-
XP_019457980.1/1-226 DEAR-----RT----------------------------------E-------WDIMSS-
XP_003597736.1/1-226 DETR-----RT----------------------------------E-------WDIMSN-
XP_004486751.1/1-226 DEAR-----RT----------------------------------E-------WDIMSS-
XP_016169615.1/1-226 DETR-----RT----------------------------------E-------WDIMSS-
XP_025611006.1/1-226 DETR-----RT----------------------------------E-------WDIMSS-
XP_020982478.1/1-227 DETR-----RT----------------------------------E-------WDIMSS-
XP_021892835.1/1-225 DEAR-----HS----------------------------------E-------WDMMSN-
XP_012442963.1/1-227 DESR-----RS----------------------------------E-------WDIMSN-
NP_001314068.1/1-227 DESR-----RS----------------------------------E-------WDIMSN-
XP_016684379.1/1-227 DESR-----RS----------------------------------E-------WDIMSN-
XP_016730082.1/1-227 DESR-----RS----------------------------------E-------WDIMSN-
NP_001316938.1/1-227 DESR-----RS----------------------------------E-------WDIMSN-
XP_017606636.1/1-227 DESR-----RS----------------------------------E-------WDIMSN-
XP_022714992.1/1-229 DEAR-----RN----------------------------------E-------WDIMSN-
XP_022721436.1/1-227 DEAR-----RN----------------------------------E-------WDIISN-
XP_022721435.1/1-227 DEAR-----RN----------------------------------E-------WDIISN-
XP_007024189.1/1-227 DEAH-----RN----------------------------------E-------WDIMSN-
XP_021298731.1/1-227 DEAR-----RN----------------------------------E-------WDIMSN-
XP_004302894.1/1-228 DETR-----RN----------------------------------E-------WDIMLN-
XP_011466687.1/1-235 DETR-----RN----------------------------------E-------WDIMLN-
XP_024180978.1/1-228 DENR-----RN----------------------------------E-------WDIMLN-
XP_024180977.1/1-235 DENR-----RN----------------------------------E-------WDIMLN-
XP_009353605.1/1-226 DETR-----RN----------------------------------E-------WDIMLN-
XP_008380909.1/1-226 DETR-----RN----------------------------------E-------WDIMLN-
NP_001280927.1/1-227 DETR-----RN----------------------------------E-------WDIMLN-
XP_009338062.1/1-226 DETR-----RN----------------------------------E-------WDIMLN-
XP_009338046.1/1-226 DETR-----RN----------------------------------E-------WDIMLN-
XP_021829136.1/1-226 DETR-----RN----------------------------------E-------WDIMLN-
XP_008228560.1/1-226 DETR-----RN----------------------------------E-------WDIMLN-
XP_007217160.1/1-226 DETR-----RN----------------------------------E-------WDIMIN-
XP_022891611.1/1-228 DENH-----RN----------------------------------E-------WDIMSN-
XP_002304207.2/1-228 DEAR-----RN----------------------------------E-------WDIMSN-
XP_011010522.1/1-228 DESR-----RN----------------------------------E-------WDIMSN-
XP_011009828.1/1-227 DEAR-----RN----------------------------------E-------WDIMSN-
XP_024462179.1/1-228 DEAR-----RN----------------------------------E-------WDIMSN-
XP_018837483.1/1-226 DEAR-----RN----------------------------------E-------WDIISN-
XP_018856342.1/1-226 DEAR-----RN----------------------------------E-------WDIMFK-
XP_018856994.1/1-226 DEAR-----RN----------------------------------E-------WDIMFK-
XP_018858360.1/1-226 DEAR-----RN----------------------------------E-------WDIMFK-
XP_015867693.1/1-226 DESR-----RN----------------------------------E-------WDIMSN-
XP_010062490.1/1-226 DESR-----RS----------------------------------E-------WDIMAS-
XP_010098082.1/1-228 DESH-----RS----------------------------------E-------WDIISR-
XP_002284502.1/1-228 DEAR-----RS----------------------------------E-------WDIMLS-
XP_023875609.1/1-226 DEAH-----RS----------------------------------E-------WDIMSS-
XP_002516023.1/1-226 DDTH-----RN----------------------------------E-------WDIMSN-
XP_021652755.1/1-228 DETH-----RN----------------------------------E-------WDIISN-
XP_021654904.1/1-226 DDDH-----RN----------------------------------E-------WDIMSN-
XP_021611342.1/1-229 DDAH-----RN----------------------------------E-------WDIMSN-
XP_012073376.1/1-226 DDAR-----RN----------------------------------E-------WDIMSN-
XP_006465622.1/1-226 DEAH-----RN----------------------------------E-------WDIMSN-
XP_006426950.1/1-226 DEAH-----RN----------------------------------E-------WDIMSN-
XP_009392419.1/1-229 DESR-----RA----------------------------------E-------WDIMLT-
XP_020518333.1/1-227 DDAR-----RK----------------------------------E-------WDIMSS-
XP_018629167.1/1-227 DETR-----QH----------------------------------E-------CNIMSN-
XP_009611250.1/1-227 DETR-----QH----------------------------------E-------CNIMSN-
XP_009611249.1/1-227 DETR-----QH----------------------------------E-------CNIMSN-
XP_009611248.1/1-227 DETR-----QH----------------------------------E-------CNIMSN-
XP_016479156.1/1-227 DETR-----QH----------------------------------E-------CNIMSN-
XP_016479155.1/1-227 DETR-----QH----------------------------------E-------CNIMSN-
XP_016479154.1/1-227 DETR-----QH----------------------------------E-------CNIMSN-
XP_009778213.1/1-229 DETR-----RH----------------------------------E-------WDVMSN-
XP_016501674.1/1-229 DETR-----RH----------------------------------E-------WDVMSN-
XP_019224885.1/1-229 DETR-----RH----------------------------------E-------WDVMSN-
XP_019224886.1/1-229 DETR-----RH----------------------------------E-------WDVMSN-
XP_009592772.1/1-229 DETR-----RH----------------------------------E-------WDVMSN-
XP_016496403.1/1-229 DETR-----RH----------------------------------E-------WDVMSN-
XP_016562690.1/1-228 DENR-----RH----------------------------------E-------WDVMSN-
XP_006343080.1/1-229 DENH-----RH----------------------------------E-------WDVMSN-
XP_004235676.1/1-229 DENH-----RH----------------------------------E-------WDVMSN-
XP_020227921.1/1-228 DEAH-----RS----------------------------------K-------WDSKSS-
XP_007135673.1/1-226 DEAR-----RS----------------------------------E-------WDMMST-
XP_014515030.1/1-226 DEAH-----RS----------------------------------E-------WDMMSS-
XP_017407449.1/1-226 DEAR-----RS----------------------------------E-------WDMMSC-
XP_010691342.1/1-224 DDTH-----RQ----------------------------------E-------WDVMAN-
XP_021731998.1/1-222 DDSR-----RH----------------------------------E-------WDVMAN-
XP_021736870.1/1-224 DDSR-----RH----------------------------------E-------WDVMAN-
XP_024969955.1/1-229 DETQ-----RN----------------------------------E-------WDIMSN-
XP_024969954.1/1-229 DETQ-----RN----------------------------------E-------WDIMSN-
XP_024969953.1/1-229 DETQ-----RN----------------------------------E-------WDIMSN-
XP_021984354.1/1-227 DETR-----RN----------------------------------E-------WDIMSN-
XP_017232370.1/1-228 DETR-----RN----------------------------------E-------WEIMAN-
XP_017216573.1/1-228 DETR-----RN----------------------------------E-------WEISVN-
XP_017248777.1/1-227 DETR-----RN----------------------------------E-------WDIMSN-
XP_021627148.1/1-226 DETH-----KT----------------------------------E-------WDIMSN-
XP_021627146.1/1-226 DETH-----KT----------------------------------E-------WDIMSN-
XP_021627145.1/1-226 DETH-----KT----------------------------------E-------WDIMSN-
XP_019196965.1/1-228 DETH-----RH----------------------------------E-------WDIMSN-
XP_019196967.1/1-228 DETH-----RH----------------------------------E-------WDIMSN-
XP_022848789.1/1-228 DENH-----RN----------------------------------E-------WDIMSN-
XP_011072373.1/1-228 DGNR-----RN----------------------------------E-------WDIMFN-
XP_021907286.1/1-215 DENN-----RN----------------------------------N-------WDVLCK-
XP_020885309.1/1-217 DETN-----RA----------------------------------Q-------WDVLCK-
XP_020885307.1/1-217 DETN-----RA----------------------------------Q-------WDVLCK-
NP_180796.2/1-218    EATH-----RH----------------------------------N-------WDVLCN-
XP_006296541.1/1-217 NEQN-----RP----------------------------------Q-------WDVLFT-
XP_010510028.1/1-215 DVDH-----RD----------------------------------S-------WDVLCG-
XP_010414006.1/1-214 DVEH-----RA----------------------------------S-------WDVLCA-
XP_010469602.1/1-219 DVDH-----RA----------------------------------S-------WDVLCA-
XP_010417818.1/1-218 NEDH-----RA----------------------------------N-------WDVLCN-
XP_010510869.1/1-220 NENH-----RA----------------------------------N-------WDALCN-
XP_010510870.1/1-218 NENH-----RA----------------------------------N-------WDALCN-
XP_018484769.1/1-219 DANN-----RT----------------------------------Y-------WDVITT-
XP_018484754.1/1-219 DANN-----RT----------------------------------Y-------WDVITT-
XP_013634882.1/1-219 DAKN-----RT----------------------------------N-------WDVITT-
XP_018513778.1/1-219 DANN-----RT----------------------------------N-------WDVITT-
XP_009141217.1/1-219 DANN-----RT----------------------------------N-------WDVITT-
XP_013747219.1/1-219 DANN-----RT----------------------------------N-------WDVITT-
XP_024003849.1/1-217 DENT-----RI----------------------------------H-------WDVLSS-
XP_018437853.1/1-211 DENN-----RS----------------------------------N-------WDVLFL-
XP_009143975.1/1-211 DENN-----RV----------------------------------N-------WDVLFL-
XP_009143974.1/1-211 DENN-----RV----------------------------------N-------WDVLFL-
XP_022575029.1/1-211 DENN-----RV----------------------------------N-------WDVLFL-
XP_013748684.1/1-211 DENN-----RV----------------------------------N-------WDVLFL-
XP_013694194.1/1-211 DENN-----RV----------------------------------N-------WDVLFL-
XP_013634261.1/1-211 DEKN-----RT----------------------------------N-------WDVLFL-
XP_013741430.1/1-210 DEKN-----RT----------------------------------N-------WDVLFL-
XP_017254373.1/1-221 DPQL-----RS----------------------------------K-------WDIMSN-
XP_017254240.1/1-222 DETS-----RN----------------------------------K-------WDMIAS-
XP_017221582.1/1-224 DVRS-----RS----------------------------------K-------WDLLTK-
XP_017221584.1/1-224 DVRS-----RS----------------------------------K-------WDLLTK-
XP_015941575.1/1-215 TANA-----RA----------------------------------E-------WDVLCT-
XP_025620358.1/1-215 TANA-----RA----------------------------------E-------WDVLCT-
XP_016176493.1/1-215 TANA-----RA----------------------------------E-------WDVLCT-
XP_003610661.2/1-228 DQNT-----RK----------------------------------H-------WDILSA-
XP_004496853.1/1-220 SQNS-----RS----------------------------------E-------WDIISI-
XP_006353342.1/1-230 NEQT-----RS----------------------------------Q-------WDVLSQ-
XP_015070591.1/1-229 NEQM-----RG----------------------------------Q-------WDVLSQ-
XP_019068356.1/1-229 NEQT-----RG----------------------------------Q-------WDVLSQ-
XP_004234441.1/1-229 NEQT-----RG----------------------------------Q-------WDVLSQ-
XP_024926606.1/1-217 QGDS-----RP----------------------------------K-------WDILSH-
XP_024525400.1/1-231 SEHL-----RS----------------------------------E-------WD---N-
XP_002965186.2/1-231 SEHL-----RS----------------------------------E-------WD---N-
XP_002962272.2/1-231 SEHL-----RS----------------------------------E-------WD---N-
XP_019441536.1/1-226 DVKF-----RS----------------------------------Q-------WDVLCT-
XP_019441537.1/1-226 DVKF-----RS----------------------------------Q-------WDVLCT-
XP_019441535.1/1-226 DVKF-----RS----------------------------------Q-------WDVLCT-
XP_019451758.1/1-229 EAKF-----RS----------------------------------Q-------WDVLCR-
XP_012850835.1/1-238 NLKR-----RK----------------------------------E-------WDMLSS-
XP_020552079.1/1-225 DPHT-----RN----------------------------------K-------WDMLSN-
XP_020552078.1/1-225 DPHT-----RN----------------------------------K-------WDMLSN-
XP_020552080.1/1-223 DPHT-----RN----------------------------------K-------WDMLSN-
XP_009408807.2/1-226 SESL-----RN----------------------------------E-------WDILLK-
XP_006342404.1/1-225 NENT-----RG----------------------------------K-------WDILSN-
XP_025887998.1/1-218 NENT-----RG----------------------------------K-------WDILIN-
XP_015081481.1/1-225 NENT-----RG----------------------------------K-------WDILTN-
XP_022000230.1/1-232 NEEL-----RS----------------------------------Q-------WDVLSP-
XP_022000229.1/1-232 NEEL-----RS----------------------------------Q-------WDVLSP-
XP_022037790.1/1-222 NEEL-----RS----------------------------------K-------WDVLSP-
XP_022037789.1/1-222 NEEL-----RS----------------------------------K-------WDVLSP-
XP_024991925.1/1-226 NEEL-----RS----------------------------------Q-------WDVLSH-
XP_023760005.1/1-229 NEEL-----RS----------------------------------Q-------WDVLSH-
XP_023760001.1/1-229 NEEL-----RS----------------------------------Q-------WDVLSH-
XP_017251723.1/1-236 DEEH-----RS----------------------------------R-------WDILSH-
XP_017243321.1/1-239 NEDM-----RS----------------------------------R-------WDLLSH-
XP_017243319.1/1-239 NEDM-----RS----------------------------------R-------WDLLSH-
XP_017243320.1/1-239 NEDM-----RS----------------------------------R-------WDLLSH-
XP_017255563.1/1-234 NEEL-----RS----------------------------------R-------WDLLSH-
XP_017255562.1/1-234 NEEL-----RS----------------------------------R-------WDLLSH-
XP_012852237.1/1-226 NVNN-----RA----------------------------------H-------WDGLSR-
XP_012852238.1/1-226 NVNN-----RA----------------------------------H-------WDGLSR-
XP_012852236.1/1-226 NVNN-----RA----------------------------------H-------WDGLSR-
XP_012852235.1/1-226 NVNN-----RA----------------------------------H-------WDGLSR-
XP_011076208.1/1-227 NEKM-----RS----------------------------------H-------WDVLSQ-
XP_019200484.1/1-228 SEET-----RN----------------------------------Q-------WDVLSN-
XP_019200481.1/1-228 SEET-----RN----------------------------------Q-------WDVLSN-
XP_022879034.1/1-223 NEQT-----RG----------------------------------H-------WDVLSQ-
XP_018623115.1/1-238 NEQT-----RS----------------------------------Q-------WDILFN-
XP_018623116.1/1-238 NEQT-----RS----------------------------------Q-------WDILFN-
XP_016449095.1/1-238 NEQT-----RS----------------------------------Q-------WDILFN-
XP_009771835.1/1-238 NEQT-----RS----------------------------------Q-------WDILFN-
XP_009771833.1/1-238 NEQT-----RS----------------------------------Q-------WDILFN-
XP_019248454.1/1-225 NEQT-----RT----------------------------------Q-------WDILFK-
XP_016564677.1/1-235 NAQT-----RR----------------------------------Q-------WDVLFH-
XP_016564676.1/1-235 NAQT-----RR----------------------------------Q-------WDVLFH-
XP_009606534.1/1-237 NEQT-----RS----------------------------------Q-------WDVLSH-
XP_016457239.1/1-237 NEQT-----RS----------------------------------Q-------WDVLSH-
XP_009606533.1/1-237 NEQT-----RS----------------------------------Q-------WDVLSH-
XP_009606532.1/1-237 NEQT-----RS----------------------------------Q-------WDVLSH-
XP_016457238.1/1-237 NEQT-----RS----------------------------------Q-------WDVLSH-
XP_016468064.1/1-237 NEQT-----RS----------------------------------Q-------WDVLSH-
XP_009774404.1/1-237 NEQT-----RS----------------------------------Q-------WDVLSH-
XP_016468062.1/1-237 NEQT-----RS----------------------------------Q-------WDVLSH-
XP_016468061.1/1-237 NEQT-----RS----------------------------------Q-------WDVLSH-
XP_009774402.1/1-237 NEQT-----RS----------------------------------Q-------WDVLSH-
XP_009774401.1/1-237 NEQT-----RS----------------------------------Q-------WDVLSH-
XP_019251817.1/1-237 NEQT-----RS----------------------------------Q-------WDVLSH-
XP_019251815.1/1-237 NEQT-----RS----------------------------------Q-------WDVLSH-
XP_019251816.1/1-237 NEQT-----RS----------------------------------Q-------WDVLSH-
XP_011470395.1/1-233 NWQL-----RG----------------------------------Q-------WDILVG-
XP_004309219.2/1-233 NWQL-----RG----------------------------------Q-------WDILVG-
XP_024180881.1/1-233 DWQL-----RG----------------------------------Q-------WDILVS-
XP_024180911.1/1-233 DWQL-----RG----------------------------------Q-------WDILVS-
XP_019450525.1/1-216 DERL-----RG----------------------------------E-------WDVLAN-
XP_019465255.1/1-230 DERL-----RG----------------------------------E-------WDVLSN-
XP_014502466.1/1-227 NEQL-----RG----------------------------------E-------WDMLSK-
XP_017438850.1/1-222 NEQL-----RG----------------------------------E-------WDMLSK-
XP_006599623.1/1-221 DENL-----RG----------------------------------E-------WDLLSK-
XP_006599622.1/1-221 DENL-----RG----------------------------------E-------WDLLSK-
XP_006599621.1/1-221 DENL-----RG----------------------------------E-------WDLLSK-
XP_004515730.1/1-240 EARL-----RG----------------------------------E-------WDVLTG-
XP_013442370.1/1-239 EAQM-----RG----------------------------------G-------WDALSN-
XP_004252035.1/1-225 NENT-----RI----------------------------------E-------WDILSN-
XP_015060726.1/1-225 NENT-----RI----------------------------------E-------WDILSN-
XP_006589608.1/1-229 SENT-----RN----------------------------------Q-------WDILSS-
XP_006589605.1/1-230 SENT-----RN---Q------------------------------V-------WDILSS-
XP_006849468.1/1-231 DERT-----RP----------------------------------Q-------WDVLSS-
XP_020526173.1/1-231 DERT-----RP----------------------------------Q-------WDVLSS-
XP_020109515.1/1-228 DETM-----RT----------------------------------Q-------WDVLSS-
NP_001310555.1/1-224 DEHT-----RS----------------------------------Q-------WDVLSH-
XP_010544375.1/1-225 DGRM-----RC----------------------------------E-------WDILSN-
NP_001314001.1/1-230 NERM-----RN----------------------------------Q-------WDILSS-
XP_016732454.1/1-230 NERM-----RN----------------------------------Q-------WDILSS-
XP_016732453.1/1-230 NERM-----RN----------------------------------Q-------WDILSS-
XP_012484067.1/1-230 NERM-----RN----------------------------------Q-------WDILSS-
XP_012484066.1/1-230 NERM-----RN----------------------------------Q-------WDILSS-
XP_017631791.1/1-230 NERM-----RN----------------------------------Q-------WDILSS-
XP_017631789.1/1-230 NERM-----RN----------------------------------Q-------WDILSS-
XP_016728129.1/1-230 NERM-----RN----------------------------------Q-------WDILSS-
XP_016728128.1/1-230 NERM-----RN----------------------------------Q-------WDILSS-
XP_021671684.1/1-240 DERS-----RS----------------------------------E-------WDILSH-
XP_021671683.1/1-240 DERS-----RS----------------------------------E-------WDILSH-
XP_021671685.1/1-240 DERS-----RS----------------------------------E-------WDILSH-
XP_002318231.3/1-240 DEQS-----RS----------------------------------H-------WDILSN-
XP_024438602.1/1-240 DEQS-----RS----------------------------------H-------WDILSN-
XP_024442055.1/1-239 DEQS-----RS----------------------------------Q-------WDILSN-
XP_002322460.1/1-239 DEQS-----RS----------------------------------Q-------WDILSN-
XP_011048682.1/1-239 DEQS-----RS----------------------------------Q-------WDILSN-
XP_011048684.1/1-239 DEQS-----RS----------------------------------Q-------WDILSN-
XP_011048683.1/1-239 DEQS-----RS----------------------------------Q-------WDILSN-
XP_019081404.1/1-234 DERQ-----RS----------------------------------K-------WDILSN-
XP_003634081.1/1-234 DERQ-----RS----------------------------------K-------WDILSN-
XP_008377249.1/1-231 DEQF-----RK----------------------------------E-------WDILSS-
XP_017189459.1/1-231 DEQF-----RK----------------------------------E-------WDILSS-
XP_008377244.1/1-231 DEQF-----RK----------------------------------E-------WDILSS-
XP_009371302.1/1-231 DEQF-----RK----------------------------------D-------WDILSS-
XP_009371299.1/1-231 DEQF-----RK----------------------------------D-------WDILSS-
XP_008376545.1/1-231 DEQF-----RK----------------------------------D-------WDILSS-
XP_008376544.1/1-231 DEQF-----RK----------------------------------D-------WDILSS-
XP_017188609.1/1-231 DEQF-----RK----------------------------------D-------WDILSS-
XP_022945426.1/1-238 DECL-----RS----------------------------------E-------WDILSN-
XP_022945425.1/1-238 DECL-----RS----------------------------------E-------WDILSN-
XP_023541229.1/1-238 DECL-----RS----------------------------------E-------WDILSN-
XP_023541228.1/1-238 DECL-----RS----------------------------------E-------WDILSN-
XP_022968414.1/1-238 DECL-----RS----------------------------------E-------WDILSN-
XP_022968413.1/1-238 DECL-----RS----------------------------------E-------WDILSN-
XP_023552381.1/1-232 DDRL-----RS----------------------------------E-------WDILSN-
XP_022922479.1/1-232 DDRL-----RS----------------------------------E-------WDILSN-
XP_023552380.1/1-232 DDRL-----RS----------------------------------E-------WDILSN-
XP_022922478.1/1-232 DDRL-----RS----------------------------------E-------WDILSN-
XP_022984644.1/1-232 DDHF-----RS----------------------------------E-------WDILSN-
XP_022984643.1/1-232 DDHF-----RS----------------------------------E-------WDILSN-
XP_008439185.1/1-242 DECL-----RS----------------------------------E-------WDILSN-
XP_008439184.1/1-242 DECL-----RS----------------------------------E-------WDILSN-
XP_011651140.1/1-242 DECL-----RS----------------------------------E-------WDILSN-
XP_004140784.1/1-242 DECL-----RS----------------------------------E-------WDILSN-
XP_009391954.1/1-234 ETRF-----RS----------------------------------K-------WDILSN-
XP_003581542.2/1-227 DEGT-----RA----------------------------------E-------WDILST-
XP_006652658.1/1-224 NEGL-----RA----------------------------------E-------WDILSN-
XP_015637147.1/1-225 NKGL-----RA----------------------------------E-------WDILSN-
XP_025823430.1/1-225 NEGM-----RA----------------------------------E-------WDILSN-
XP_004978185.1/1-225 NEGL-----RA----------------------------------E-------WDILSN-
XP_021317935.1/1-228 DEEL-----RA----------------------------------E-------WDILSN-
NP_001336434.1/1-226 DEEL-----RA----------------------------------E-------WDILSN-
XP_019709552.1/1-234 DERR-----RG----------------------------------E-------WDILSN-
XP_015696802.1/1-257 DEQR-----RG----------------------------------D-------WDILAN-
XP_015612621.1/1-258 DEQR-----RG----------------------------------D-------WDILAN-
XP_004957413.1/1-253 DEQR-----RG----------------------------------E-------WDILAN-
XP_025801167.1/1-251 DEQR-----RG----------------------------------E-------WDILAN-
NP_001105125.2/1-256 DEQR-----RG----------------------------------E-------WDILAN-
XP_002462701.1/1-258 DEQR-----RG----------------------------------E-------WDILAN-
XP_020262629.1/1-222 -ERL-----RS----------------------------------Q-------WDILSN-
XP_020699782.1/1-235 DEHL-----RR----------------------------------E-------WDILSN-
XP_020699776.1/1-235 DEHL-----RR----------------------------------E-------WDILSN-
XP_020573166.1/1-235 DEQL-----RR----------------------------------E-------WDILSN-
XP_020187432.1/1-228 NEKT-----RA----------------------------------E-------WDILSK-
XP_025656795.1/1-233 DERM-----RG----------------------------------E-------WVILSA-
XP_025656792.1/1-233 DERM-----RG----------------------------------E-------WVILSA-
XP_025656794.1/1-233 DERM-----RG----------------------------------E-------WVILSA-
XP_020979736.1/1-233 DERM-----RG----------------------------------E-------WVILSA-
XP_020999151.1/1-233 DERM-----RG----------------------------------E-------WVILSG-
XP_015967451.1/1-233 DERM-----RG----------------------------------E-------WVILSG-
XP_020999150.1/1-233 DERM-----RG----------------------------------E-------WVILSG-
XP_025701970.1/1-233 DERM-----RG----------------------------------E-------WVILSG-
XP_025701968.1/1-233 DERM-----RG----------------------------------E-------WVILSG-
XP_025701969.1/1-233 DERM-----RG----------------------------------E-------WVILSG-
XP_020221720.1/1-222 SGQF-----RG----------------------------------E-------WDMLSN-
XP_007152327.1/1-222 NEQL-----RG----------------------------------E-------WDMLSK-
XP_006587345.1/1-222 DENL-----RG----------------------------------E-------WDMLSK-
XP_006587344.1/1-222 DENL-----RG----------------------------------E-------WDMLSK-
XP_010035104.1/1-239 NEQL-----RS----------------------------------E-------WDILSH-
XP_021765162.1/1-234 DEKL-----RG----------------------------------K-------WDILAN-
XP_021765160.1/1-234 DEKL-----RG----------------------------------K-------WDILAN-
XP_021730123.1/1-234 DEKL-----RG----------------------------------K-------WDILAN-
XP_021842103.1/1-232 DEKL-----RG----------------------------------K-------WDILAN-
XP_021842106.1/1-232 DEKL-----RG----------------------------------K-------WDILAN-
XP_021842105.1/1-232 DEKL-----RG----------------------------------K-------WDILAN-
XP_021842104.1/1-232 DEKL-----RG----------------------------------K-------WDILAN-
XP_010693471.1/1-235 DEKL-----RG----------------------------------K-------WDILAN-
XP_019107938.1/1-235 DEKL-----RG----------------------------------K-------WDILAN-
XP_010693469.1/1-235 DEKL-----RG----------------------------------K-------WDILAN-
XP_019107937.1/1-235 DEKL-----RG----------------------------------K-------WDILAN-
XP_020163116.1/1-258 DEQR-----RG----------------------------------E-------WDILAN-
XP_020163109.1/1-258 DEQR-----RG----------------------------------E-------WDILAN-
XP_003578475.1/1-267 DEQR-----RG----------------------------------E-------WDILAN-
XP_011012369.1/1-242 DEKS-----RS----------------------------------H-------WDILSN-
XP_011012370.1/1-242 DEKS-----RS----------------------------------H-------WDILSN-
XP_008234278.1/1-230 DEQL-----RD----------------------------------Q-------WDVLSK-
XP_021827117.1/1-230 DEQL-----RD----------------------------------Q-------WDVLSK-
XP_021827116.1/1-230 DEQL-----RD----------------------------------Q-------WDVLSK-
XP_020413581.1/1-230 DEQL-----RD----------------------------------Q-------WDVLSK-
XP_007219555.2/1-230 DEQL-----RD----------------------------------Q-------WDVLSK-
XP_018456926.1/1-230 DSKC-----RN----------------------------------Q-------WDILCN-
XP_013613235.1/1-226 DGKC-----RN----------------------------------Q-------WDILCN-
XP_013686466.1/1-227 DSKC-----RN----------------------------------Q-------WDILCN-
XP_009106897.1/1-227 DSKC-----RN----------------------------------Q-------WDILCN-
XP_006401857.1/1-229 DGKC-----RN----------------------------------Q-------WDILSN-
XP_020871109.1/1-230 DGKC-----RN----------------------------------Q-------WDILSN-
NP_001332093.1/1-229 DGKC-----RN----------------------------------Q-------WDILSN-
NP_200030.1/1-229    DGKC-----RN----------------------------------Q-------WDILSN-
XP_006282099.1/1-225 DGKC-----RN----------------------------------Q-------WDILSN-
XP_019095386.1/1-229 DGKC-----RN----------------------------------Q-------WDILSN-
XP_019095387.1/1-229 DGKC-----RN----------------------------------Q-------WDILSN-
XP_019091174.1/1-228 DGKC-----RN----------------------------------Q-------WDILSN-
XP_019091179.1/1-228 DGKC-----RN----------------------------------Q-------WDILSN-
XP_019087173.1/1-228 DGKC-----RN----------------------------------Q-------WDILSN-
XP_010555654.1/1-234 DPKC-----RN----------------------------------Q-------WDILSN-
XP_010555656.1/1-234 DPKC-----RN----------------------------------Q-------WDILSN-
XP_010555655.1/1-234 DPKC-----RN----------------------------------Q-------WDILSN-
XP_010537199.1/1-242 DQNC-----RN----------------------------------Q-------WDILSN-
XP_010537197.1/1-242 DQNC-----RN----------------------------------Q-------WDILSN-
XP_010537198.1/1-241 DQNC-----RN----------------------------------Q-------WDILSN-
Con_90%              pp.......Rs..........................................Ws..s..
Con_80%              -c.......Rs..........................................Ws.hs..
Con_70%              -ct......Rs..........................................W-hhus.
Con_60%              --p......RS..................................p.......WDlLSs.
Con_50%              DEpp.....RS..................................p.......WDlLSN.


XP_020582785.1/1-284 AYSTASLRGSTY-EVPGFSDNSGF--LANHVIIPLAYTV-E--NE----E--FLEVI-R-
XP_020682623.1/1-284 AYSTASLRGCPN-AFPGFFDNSSF--AANHVIIPLAHTV-E--NE----E--FLEVI-R-
XP_020682624.1/1-284 AYSTASLRGCPN-AFPGFFDNSSF--AANHVIIPLAHTV-E--NE----E--FLEVI-R-
XP_006846015.1/1-286 AYAASSMRTGQF-VVPGSRT-GSF--PGSQVILPLAQTV-E--HE----E--FLEVI-R-
NP_001142394.1/1-289 AYSVASLRANPY-TVPGLRA-GGF--MGNQVILPLARTL-E--HE----E--SLEVI-R-
NP_001146215.1/1-288 AYSAASLRANPY-NVPGLRA-GGF--MGNQVILPLARTV-E--HE----E--CLEVI-R-
XP_020405718.1/1-288 AYSAASLRANPY-NVPGLRA-GGF--MGNQVILPLARTV-E--HE----E--CLEVI-R-
XP_002443548.1/1-287 AYSAASLRANPY-AVPGLRA-GGF--MGNQVILPLARTL-E--HE----E--CLEVI-R-
XP_025808876.1/1-287 AYSAASLRANPY-AVPGLRA-GGF--MGNQVILPLARTL-E--HE----E--SLEVI-R-
XP_004963232.1/1-287 AYSAASLRANPY-AVPGLRA-GGF--MGNQVILPLARTL-E--HD----E--SLEVI-R-
XP_020192001.1/1-285 AYSAAALRASPY-AVPGLRA-SGF--MGSQVILPLAHTL-E--HE----E--FLEVI-R-
XP_003562005.1/1-285 AYSAAALRASPY-AVPGLRA-SGF--MGSQVILPLAHTL-E--HE----E--FLEVI-R-
XP_004982339.1/1-286 AYSAAALRASPY-AVPGLRA-SGF--MGSQVILPLAHTL-E--HE----E--FLEVI-R-
XP_025796542.1/1-286 AYSAAALRASPY-AVPGLRA-SGF--MGSQVILPLAHTL-E--HE----E--FLEVI-R-
NP_001347456.1/1-286 AYSAAALRASPY-AVPGLRA-SGF--TGSQVILPLAHTL-E--HE----E--FLEVI-R-
NP_001352464.1/1-286 AYSAAALRASPY-AVPGLRA-SGF--MGSQVILPLAHTL-E--HE----E--FLEVI-R-
XP_002464180.1/1-286 AYSAAALRASPY-AVPGLRA-SGF--MGSQVILPLAHTL-E--HE----E--FLEVI-R-
XP_015689987.1/1-286 AYSAAALRASPY-AVPGLRA-GGF--MGSQVILPLAHTL-E--HE----E--FLEVI-R-
XP_015632484.1/1-286 AYSAAALRASPY-AVPGLRA-GGF--MGSQVILPLAHTL-E--HE----E--FLEVI-R-
XP_015620816.1/1-285 AYSAASLRASPY-AVPGLRT-SGF--MGSQVILPLAHTL-E--HE----E--FLEVI-R-
XP_006664738.1/1-285 AYSAASLRASPY-TVPGLRT-SGF--MGSQVILPLAHTL-E--HE----E--FLEVI-R-
XP_020190954.1/1-286 AYSAASLRASPY-AVPGLRA-GGF--MGNQVILPLAHTL-E--HE----E--FLEVL-R-
XP_020163543.1/1-286 AYSAASLRASPY-AVPGLRA-GGF--MGNQVILPLAHTL-E--HE----E--FLEVL-R-
XP_003577453.1/1-286 AYSAASLRASPY-AVPGLRA-GGF--MGSQVILPLAHTL-E--HE----E--FLEVL-R-
XP_020575104.1/1-285 VYSAASLRASPY-AVPCLRVGSAF--LGSHVIIPLAHTV-E--NE----E--VLEVV-R-
XP_020696902.1/1-286 AYSASSLRASPY-AVPGLRVSNGF--LGSHVIIPLAHTV-E--NE----E--VLEVV-R-
XP_020578108.1/1-284 AYSTATLRASSY-PILGVRAGGSF--HGGQVILPLAQTV-E--NE----E--FLEVI-R-
XP_020698923.1/1-284 AYSTASLRASSY-PILGVRASGSF--HGGQVILPLAQTV-E--NE----E--FLEVI-R-
XP_020096610.1/1-279 AYSAASLRASPF-AVPGLRAYNGF--MGSQVILPLAHTL-E--HE----E--FLEVI-R-
XP_009412508.1/1-287 AYSASSLRASPY-AVPGVRSGSNF--VGNQIILPLAHTT-E--HE----E--TLEVV-R-
XP_020271156.1/1-284 AYSAASLRASPY-AVPGLRASSGL--FGGQVILPLAHTV-E--HE----E--FLEVI-R-
XP_020273236.1/1-287 AYSAASLRASPY-AVPGLRASNGF--MGGQVILPLAHTV-E--HE----E--FLEVI-R-
XP_020092733.1/1-287 AYSAASLRASPY-ALPGLRSSSGF--LGSQVILPLGHTV-E--HE----E--FLEVI-R-
XP_019701934.1/1-288 AYSAASLRASPY-AVPGVRASSGF--LGGQVILPLAHTI-E--HE----E--FLEVI-R-
XP_010905421.1/1-288 AYSAASLRASPY-AVPGVRASSGF--LGGQVILPLAHTI-E--HE----E--FLEVI-R-
XP_010905420.1/1-289 AYSAASLRASPY-AVPGVRASSGF--LGGQVILPLAHTI-E--HE----E--FLEVI-R-
XP_010923678.1/1-288 AYSAASLRACPY-AVPGVRASSGF--LGGQVILPLAHTI-E--HE----E--FLEVI-R-
XP_008794452.1/1-288 AYSAASLRACPY-AVPGVRASSGF--LGGQVILPLAHTI-E--HE----E--FLEVI-R-
XP_009397391.1/1-288 AYSAASMRASPY-AVPGVRASSGF--LGSQMILPLAHTV-E--HE----E--FLEVI-R-
XP_009408551.1/1-288 AYSAASLRTSPY-AVPGVRTSSGL--SGSQVILPLAHTL-E--LE----E--FLEVI-K-
XP_009399045.1/1-288 AYSAASLTANPY-AVPAVRASGGF--LGSQVILPLAHTV-E--HE----E--FLEVI-R-
XP_009388964.1/1-288 AYSAASLRANPY-AVPGVRASTGF--SGSQVILPLAHTA-E--HE----E--FLEVI-R-
XP_009397717.1/1-288 AYSAASLRTGSY-AVPGVRPNSGF--LGSQVILPLAHTA-E--NE----E--FLEVI-R-
XP_018488876.1/1-280 AYCAASLRATPF-SVPCVRT-GGF--PSNQVILPLAQTL-E--HE----E--FLEVV-R-
XP_013587486.1/1-280 AYCAASLRATPF-AVPCVRT-GGF--PSNQVILPLAQTL-E--HE----E--FLEVV-R-
XP_009115139.1/1-280 AYCAASLRATPF-AVPCVRT-GGF--PSNQVILPLAQTL-E--HE----E--FLEVV-R-
XP_013662459.1/1-280 AYCAASLRATPF-AVPCVRT-GGF--PSNQVILPLAQTL-E--HE----E--FLEVV-R-
XP_013707293.1/1-280 AYCAASLRATPF-AVPCVRT-GGF--PSNQVILPLAQTL-E--HE----E--FLEVV-R-
XP_006415465.2/1-280 AYSAASLRATPF-AVPCVRT-GGF--PSNQVILPLAQTL-E--HE----E--FLEVV-R-
XP_019099288.1/1-280 AYSAASLRANPF-AVPCVRT-GGF--PSNQVILPLAQTL-E--HE----E--FLEVV-R-
NP_174337.1/1-280    AYSAASLRATPY-AVPCVRT-GGF--PSNQVILPLAQTL-E--HE----E--FLEVV-R-
XP_020867051.1/1-280 AYSAASLRATPF-TVPCVRT-GGF--PSNQVILPLAQTL-E--HE----E--FLEVV-R-
XP_020867052.1/1-280 AYSAASLRATPF-TVPCVRT-GGF--PSNQVILPLAQTL-E--HE----E--FLEVV-R-
XP_010460847.1/1-280 AYSAASLRATPF-AVPCVRT-GGF--PSNQVILPLAQTL-E--HE----E--FIEVV-R-
XP_019094482.1/1-280 AYSAASLRATPF-AVPCVRT-GGF--PSNQVILPLAQTL-E--HE----E--FLEVV-R-
XP_006306764.1/1-280 AYSAASLRATPF-AVPCVRT-GGF--PSNQVILPLAQTL-E--HE----E--FLEVV-R-
XP_010538268.1/1-285 AYSAACLRTNPF-SVPCARA-CGF--PSSQVILPLAQTI-E--HE----E--FLEVV-R-
XP_010519070.1/1-284 AYSSACLRTNPF-AVPCARA-GGF--PSSQVILPLAQTI-E--HE----E--FLEVV-R-
XP_018436356.1/1-280 AYAAASLRASPF-TVPCARA-GGF--PSNQVILPLAQTV-E--HE----E--FLEVV-R-
XP_013637594.1/1-286 AYAAASLRASPF-AVPCARA-GGF--PSNQVILPLAQTV-E--HE----E--FLEVV-R-
XP_013692700.1/1-286 AYAAASLRASPF-AVPCARA-GGF--PSNQVILPLAQTV-E--HE----E--FLEVV-R-
XP_009143747.1/1-286 AYAAASLRASPF-AVPCARA-GGF--PSNQVILPLAQTV-E--HE----E--FLEVV-R-
XP_018484893.1/1-285 AYAAASLRASPF-AVPCARA-GGF--PSNQVILPLAQTV-E--HE----E--FLEVV-R-
XP_013637264.1/1-280 AYAAASLRASPF-AVPCARA-GGF--PSNQVILPLAQTV-E--HE----E--FLEVV-R-
XP_013637263.1/1-280 AYAAASLRASPF-AVPCARA-GGF--PSNQVILPLAQTV-E--HE----E--FLEVV-R-
XP_022557131.1/1-280 AYAAASLRASPF-AVPCARA-GGF--PSNQVILPLAQTV-E--HE----E--FLEVV-R-
XP_013729051.1/1-280 AYAAASLRASPF-AVPCARA-GGF--PSNQVILPLAQTV-E--HE----E--FLEVV-R-
XP_013744548.1/1-280 AYAAASLRASPF-AVPCARA-GGF--PSNQVILPLAQTV-E--HE----E--FLEVV-R-
XP_009141396.1/1-280 AYAAASLRASPF-AVPCARA-GGL--PSNQVILPLAQTV-E--HE----E--FLEVV-R-
XP_006410621.1/1-284 AYAAASLRASPF-AVPCARA-GGF--PSNQVILPLAQTV-E--HE----E--FLEVV-R-
NP_181018.1/1-284    AYAAASLRASPF-AVPCARA-GGF--PSNQVILPLAQTV-E--HE----E--SLEVV-R-
XP_020884308.1/1-284 AYAAASLRASPF-AVPCARA-GGF--PSNQVILPLAQTV-E--HE----E--SLEVV-R-
XP_006293359.1/1-284 AYAAASLRASPF-AVPCARA-GGF--PSNQVILPLAQTV-E--HE----E--SLEVV-R-
XP_010516626.1/1-284 AYAAASLRASPF-AVPCARA-GGF--PSNQVILPLAQTV-E--HE----E--SLEVV-R-
XP_010509720.1/1-284 AYAAASLRASPF-AVPCARA-GGF--PSNQVILPLAQTV-E--HE----E--SLEVV-R-
XP_010504942.1/1-284 AYAAASLRASPF-AVPCARA-GGF--PSNQVILPLAQTV-E--HE----E--SLEVV-R-
XP_017223336.1/1-283 AYNAASLKASPY-AVPCARP-GAP--PSTQVILPLAHSM-E--NE----E--LLEVV-R-
XP_016559371.1/1-285 AYSSASLKASPY-AVPCARP-GVF--PSSQVILPLAQTV-E--HE----E--FLEVV-R-
XP_016559374.1/1-285 AYSSASLKASPY-AVPCARP-GVF--PSSQVILPLAQTV-E--HE----E--FLEVV-R-
XP_012830207.1/1-281 FYSAASLKSSPY-AIPFAKP-GGF--PTSQVILPLAHTV-E--HE----E--VLEVV-R-
XP_012827547.1/1-287 VYCADSLKASPY-AVPCAKT-GGF--PSSQVILPLAQTV-E--NE----E--SLEVV-R-
XP_022866806.1/1-284 AYCAASLKASPY-AVPCAKL-GGF--PSSQVILPLAQTV-E--HE----E--FLEVV-R-
XP_022866805.1/1-285 AYCAASLKASPY-AVPCAKL-GGF--PSSQVILPLAQTV-E--HE----E--FLEVV-R-
XP_023001017.1/1-285 AYSAACLKSSAY-AVPCARP-GGF--PSGQVILPLAHTV-E--NE----E--FLEVV-R-
XP_023001016.1/1-285 AYSAACLKSSAY-AVPCARP-GGF--PSGQVILPLAHTV-E--NE----E--FLEVV-R-
XP_023519837.1/1-285 AYSAACLKSSAY-AVPCARP-GGF--PSGQVILPLAHTV-E--NE----E--FLEVV-R-
XP_023519836.1/1-285 AYSAACLKSSAY-AVPCARP-GGF--PSGQVILPLAHTV-E--NE----E--FLEVV-R-
XP_022927139.1/1-285 AYSAACLKSSAY-AVPCARP-GGF--PSGQVILPLAHTV-E--NE----E--FLEVV-R-
XP_022927138.1/1-285 AYSAACLKSSAY-AVPCARP-GGF--PSGQVILPLAHTV-E--NE----E--FLEVV-R-
XP_022139982.1/1-285 AYSATCLKASAY-AVPCARP-GGF--SSGQVILPLAHTV-E--NE----E--FLEVV-R-
XP_004135550.1/1-285 AYSAACLKASAY-AVPCARP-GGF--PGGQVILPLAHTV-E--NE----E--FLEVV-R-
XP_008445207.1/1-285 AYSAACLKASAY-AVPCARP-GGF--PGGQVILPLAHTV-E--NE----E--FLEVV-R-
XP_017230337.1/1-286 AYSAASLKSSPY-AIPYARP-GAF--PGTQVILPLAHTV-E--HE----E--FLEVV-R-
XP_017230338.1/1-286 AYSAASLKSSPY-AIPYARP-GAF--PGTQVILPLAHTV-E--HE----E--FLEVV-R-
XP_010316959.1/1-285 AYSSASLKASPY-AVPCARP-GVF--PSSQVILPLAQTV-E--HE----E--FLEVV-R-
XP_004233170.1/1-285 AYSSASLKASPY-AVPCARP-GVF--PSSQVILPLAQTV-E--HE----E--FLEVV-R-
XP_015066839.1/1-285 AYSSASLKASPY-AVPCARP-GVF--PSSQVILPLAQTV-E--HE----E--FLEVV-R-
XP_015066842.1/1-285 AYSSASLKASPY-AVPCARP-GVF--PSSQVILPLAQTV-E--HE----E--FLEVV-R-
XP_015066843.1/1-285 AYSSASLKASPY-AVPCARP-GVF--PSSQVILPLAQTV-E--HE----E--FLEVV-R-
XP_006353018.1/1-285 AYSSASLKASPY-AVPCARP-GVF--PSSQVILPLAQTV-E--HE----E--FLEVV-R-
XP_006353020.1/1-285 AYSSASLKASPY-AVPCARP-GVF--PSSQVILPLAQTV-E--HE----E--FLEVV-R-
XP_019178964.1/1-287 AYSAASLKASPY-AVPCARP-GGF--PSSQVILPLAQTV-E--HE----E--FLEVV-R-
XP_019178963.1/1-287 AYSAASLKASPY-AVPCARP-GGF--PSSQVILPLAQTV-E--HE----E--FLEVV-R-
XP_019228489.1/1-285 AYSAASLKSSPY-AVPCARP-GGF--PSSQVILPLAQTV-E--HE----E--FLEVV-R-
XP_019228488.1/1-285 AYSAASLKSSPY-AVPCARP-GGF--PSSQVILPLAQTV-E--HE----E--FLEVV-R-
NP_001289498.1/1-285 AYSAASLKASPY-AVPCARP-GGF--PSSQVILPLAQTV-E--HE----E--FLEVV-R-
XP_009777911.1/1-285 AYSAASLKASPY-AVPCARP-GGF--PSSQVILPLAQTV-E--HE----E--FLEVV-R-
XP_009612342.1/1-285 AYSAASLKASPY-AVPCARP-GGF--PSSQVILPLAQTV-E--HE----E--FLEVV-R-
XP_009612341.1/1-285 AYSAASLKASPY-AVPCARP-GGF--PSSQVILPLAQTV-E--HE----E--FLEVV-R-
XP_006363649.1/1-287 AYSSASLKASPY-AVPCARP-GGF--PSSQVILPLAQTV-E--HE----E--FLEVV-R-
XP_015066689.1/1-287 AYSSASLKASPY-AVPCARP-GGF--PSSQVILPLAQTV-E--HE----E--FLEVV-R-
XP_010315636.1/1-287 AYSSASLKASPY-AVPCARP-GGF--PSSQVILPLAQTV-E--HE----E--FLEVV-R-
XP_022879368.1/1-285 AYSAASLKASPY-AVPCARL-SGF--PGSQVILPLAQTV-E--HE----E--LLEVV-R-
XP_020554026.1/1-285 VYSAASLKASPY-AVPCAKL-GGF--PSSQVILPLAQTV-E--HE----E--FLEVV-R-
XP_011096678.1/1-285 VYSAASLKASPY-AVPCAKL-GGF--PSSQVILPLAQTV-E--HE----E--FLEVV-R-
XP_011085207.1/1-285 VYSAASLKASPY-AVPCAKP-GAI--PSSHVTLPLAQTV-E--HE----E--FLEVV-R-
XP_023003193.1/1-285 TYSAASLKASPY-AVPCARP-GGF--PSNQVILPLAHTV-E--HV----E--FLEVV-R-
XP_022926723.1/1-285 AYSAASLKASPY-AVPCARP-GGF--PSSQVILPLAHTV-E--HE----E--FLEVV-R-
XP_023518237.1/1-285 AYSAASLKASPY-AVPCARP-GGF--PSSQVILPLAHTV-E--HE----E--FLEVV-R-
XP_022146537.1/1-286 AYSAACLKASPY-AVPCARP-GGF--PSSQVILPLAHTV-E--HE----E--FLEVV-R-
XP_011658319.1/1-286 AYSAASLKASPY-AVPCARP-GGF--PSSQVILPLATTV-E--HE----E--FLEVV-R-
XP_008439554.1/1-286 AYSAASLKASPY-AVPCARP-GGF--PSSQVILPLATTV-E--HE----E--FLEVV-R-
XP_022978372.1/1-286 AYCAASLKASPY-AVPCARP-GGF--PSSQVILPLASTV-E--HE----E--FLEVV-R-
XP_023543833.1/1-286 AYCAASLKASPY-AVPCARP-GGF--PSSQVILPLASTV-E--HE----E--FLEVV-R-
XP_022926042.1/1-286 AYCAASLKASPY-AVPCARP-GGF--PSSQVILPLASTV-E--RE----E--FLEVV-R-
XP_010247499.1/1-286 AYSAASLKASPC-AVLGARP-SGF--PSSQVILPLAHTV-E--HE----E--FLEVI-R-
XP_016717556.1/1-285 TYSAACLKPNPY-TVPCARP-SGF--PSSQVILPLAHTV-E--HE----E--FLEVV-R-
XP_012455108.1/1-285 TYSAACLKPNPY-TVPCARP-SGF--PSSQVILPLAHTV-E--HE----E--FLEVV-R-
XP_016717554.1/1-287 TYSAACLKPNPY-TVPCARP-SGF--PSSQVILPLAHTV-E--HE----E--FLEVV-R-
XP_012455107.1/1-287 TYSAACLKPNPY-TVPCARP-SGF--PSSQVILPLAHTV-E--HE----E--FLEVV-R-
XP_016711412.1/1-287 TYSASCLKPNPY-TVPCARP-SGF--PSSQVILPLAHTV-E--HE----E--FLEVV-R-
XP_017649973.1/1-287 TYSASCLKPNPY-TVPCARP-SGF--PSSQVILPLAHTV-E--HE----E--FLEVV-R-
XP_022716449.1/1-285 TYSAACLKASPY-AVPCTRP-GGF--PCSQVILPLAHTV-E--HE----E--LLEVV-R-
XP_022724664.1/1-287 TYSVACLKASPY-AVPCARP-GGF--PSSQVILPLAHTV-E--HE----E--FLEVV-R-
XP_021298192.1/1-285 TYSAACLKASPY-AVPCARP-GGF--PSSQVILPLAHTV-E--HE----E--FLEVV-R-
XP_021298193.1/1-294 TYSAACLKASPY-AVPCARP-GGF--PSSQVILPLAHTV-E--HE----E--FLEVV-R-
XP_021298191.1/1-294 TYSAACLKASPY-AVPCARP-GGF--PSSQVILPLAHTV-E--HE----E--FLEVV-R-
XP_007022874.2/1-285 TYSAACLKASPY-AVPCARP-GGF--PSSQVILPLAHTV-E--HE----E--FLEVV-R-
XP_017979205.1/1-294 TYSAACLKASPY-AVPCARP-GGF--PSSQVILPLAHTV-E--HE----E--FLEVV-R-
XP_017979204.1/1-294 TYSAACLKASPY-AVPCARP-GGF--PSSQVILPLAHTV-E--HE----E--FLEVV-R-
XP_017236680.1/1-286 AYSAASLKASPY-AVPCARP-GGF--SGTQVILPLAHTV-E--HE----E--FLEVV-R-
XP_010051959.1/1-285 AYSAASLKTSSY-AIPCVRP-GGF--PSSHVILPLAHTV-E--QE----E--FLEVV-R-
XP_010051960.1/1-285 AYSAASLKTSSY-AIPCVRP-GGF--PSSHVILPLAHTV-E--QE----E--FLEVV-R-
XP_015882975.1/1-284 AYSAACLKASPY-AVPCARP-GGF--PGSQVILPLAHTV-E--HE----E--FMEVV-R-
XP_015882797.1/1-284 AYSAACLKASPY-AVPCARP-GGF--PGSQVILPLAHTV-E--HE----E--FMEVV-R-
XP_010098570.1/1-284 AYSASCLKASPY-AIPCARP-GGF--PSSQVILPLAHTV-E--HE----E--FLEVV-R-
XP_011012084.1/1-284 VYSAACLKASPY-AVPCARP-GGF--PSSQVILPLAHTV-E--HE----E--FLEVV-R-
XP_011012083.1/1-284 VYSAACLKASPY-AVPCARP-GGF--PSSQVILPLAHTV-E--HE----E--FLEVV-R-
XP_006377667.2/1-284 VYSAACLKASPY-AVPCARP-GGF--PSSQVILPLAHTV-E--HE----E--FLEVV-R-
XP_002281868.2/1-285 AYSAACLKASPY-EVPCARP-GGF--PSSQVILPLAHTV-E--HE----E--FLEVV-R-
XP_018847863.1/1-285 AYSAACLRASPF-AVPCARP-GGF--PGSQVILPLAHTV-E--HE----E--FLEVV-R-
XP_018847862.1/1-285 AYSAACLRASPF-AVPCARP-GGF--PGSQVILPLAHTV-E--HE----E--FLEVV-R-
XP_023919942.1/1-285 AYSAACLKASPY-AVPCARP-GGF--PGSQVILPLAHTV-E--HE----E--FLEVV-R-
XP_023919941.1/1-285 AYSAACLKASPY-AVPCARP-GGF--PGSQVILPLAHTV-E--HE----E--FLEVV-R-
XP_018828101.1/1-285 AYSAACLKASPY-AVPCARP-GGF--PGSQVILPLAHTV-E--HE----E--FLEVV-R-
XP_018828100.1/1-285 AYSAACLKASPY-AVPCARP-GGF--PGSQVILPLAHTV-E--HE----E--FLEVV-R-
XP_002298892.1/1-285 AYSAACLKASPY-AVPCARP-GGF--PSSQVILPLAHTM-E--HE----E--FLEVV-R-
XP_011038887.1/1-285 AYSAACLKASPY-AVPCARA-GGF--PSSQVILPLAHTM-E--HE----E--FLEVV-R-
XP_011038886.1/1-285 AYSAACLKASPY-AVPCARA-GGF--PSSQVILPLAHTM-E--HE----E--FLEVV-R-
XP_021657817.1/1-285 TYSSACLKASPY-AIPCARP-GGF--PSGQVILPLAHTV-E--HE----E--FLEVV-R-
XP_021657818.1/1-285 TYSSACLKASPY-AIPCARP-GGF--PSGQVILPLAHTV-E--HE----E--FLEVV-R-
XP_015577248.1/1-260 TYSAACLKASPY-AVPCARP-GGF--PSSQVILPLAHTV-E--HE----E--FLEVV-R-
XP_021616343.1/1-285 TYSAHCLKASPY-AIPCARP-GGF--PSSQVILPLAHTV-E--HE----E--FLEVV-R-
XP_021645103.1/1-285 TYSAACLKASPY-AVPCARP-GGF--PCSQVILPLAYTV-E--HE----E--FLEVV-R-
XP_021592762.1/1-285 TYSAACLKASPY-AVPCARP-GGF--PSSQVILPLAHTV-E--HE----E--FLEVV-R-
XP_012075627.1/1-285 TYSAACLKASPY-AVPCARP-GGF--PSSQVILPLAHTV-E--HE----E--FLEVV-R-
XP_008372224.1/1-285 AYSAACLKASPY-AVPLARG-GGF--PTTQNILPLAQTV-E--HE----E--FLEVV-R-
XP_009362238.1/1-285 AYSAACLKASPY-AVPLARG-GGF--PTTQNILPLAQTV-E--HE----E--FLEVV-R-
XP_009344405.1/1-284 AYSAACLKASPY-AVPLARG-GGF--PSTQNILPLAQTV-E--HE----E--FLEVV-R-
XP_008349150.1/1-284 AYSAACLKASPY-AVPLARG-GGF--PSTQNILPLAQTV-E--HE----E--FLEVV-R-
XP_007214622.1/1-285 AYSAACLKASPY-AVPCARP-GGF--PSSQVILPLAQTV-E--NE----E--FLEVV-R-
XP_021812393.1/1-285 AYSAACLKASPY-AVPCARP-GGF--PSSQVILPLAQTV-E--NE----E--FLEVV-R-
XP_024161164.1/1-285 AYSAACLKASPY-AVPCARP-GGF--PSSQVILPLAPTV-E--NE----E--FLEVV-R-
XP_004293989.1/1-285 AYSAACLKASPY-AVPCARP-GGF--PSSQVILPLAPTV-E--NE----E--FLEVV-R-
XP_019419795.1/1-282 AYSAACHKASPY-AVPSARP-ADF--PTSQIILPLAHTI-E--HE----E--FLEVV-R-
XP_019426301.1/1-282 AYAAACHRASPY-AVPCARP-ADF--PTSQIILPLAHTI-E--HE----E--FLEVV-R-
XP_003524993.1/1-285 AYSSACLKASPY-AVPCARP-SGF--PSSHVIIPLAHTI-E--HE----E--FLEVV-R-
XP_003532788.1/1-285 AYSSACLKASPY-AVPCARP-SGF--PSSHVIIPLAHTI-E--HE----E--FLEVV-R-
XP_020215009.1/1-285 AYSSACLKASPY-AVPCARP-SGF--PSSQVILPLAHTI-E--HE----E--FLEVV-R-
XP_017409410.1/1-285 AYSAACLKASPY-AVPCGRP-SGF--PSSQVILPLAHTI-E--HE----E--FLEVV-R-
XP_017409418.1/1-285 AYSAACLKASPY-AVPCGRP-SGF--PSSQVILPLAHTI-E--HE----E--FLEVV-R-
XP_014509941.1/1-285 AYSAACLKASPY-AVPCARP-SGF--PSSQVILPLAHTI-E--HE----E--FLEVV-R-
XP_007159548.1/1-285 AYSAACLKASPY-AVPCARP-SGF--PSSQVILPLAHTI-E--HE----E--FLEVV-R-
XP_024047818.1/1-288 AYSAACLKASPY-AVPCARP-GGF--PSSHVILPLAHTV-E--HE----E--FLEVV-R-
XP_006448915.2/1-288 AYSAACLKASPY-AVPCARP-GGF--PSSHVILPLAHTV-E--HE----E--FLEVV-R-
XP_006468302.1/1-288 AYSAACLKASPY-AVPCARP-GGF--PSSHVILPLAHTV-E--HE----E--FLEVV-R-
XP_006468303.1/1-287 AYSAACLKASPY-AVPCARP-GGF--PSSHVILPLAHTV-E--HE----E--FLEVV-R-
XP_024047817.1/1-287 AYSAACLKASPY-AVPCARP-GGF--PSSHVILPLAHTV-E--HE----E--FLEVV-R-
XP_004504217.1/1-285 AYSATCLKASPF-AVPCARP-GGF--PSSQVILPLAHTL-E--HE----E--FLEVV-R-
XP_003594520.2/1-281 AYSATCLKSSPY-AVPCPRP-GGF--PSSQVILPLAPTI-E--HE----E--FLEVV-R-
XP_024632601.1/1-281 AYSATCLKSSPY-AVPCPRP-GGF--PSSQVILPLAPTI-E--HE----E--FLEVV-R-
XP_004486450.1/1-285 AYSSACLKTSPY-AVPCPRP-GGF--PSSQVILPLAHTI-E--HE----E--FLEVV-R-
XP_007147487.1/1-285 AYSAACLKASSY-AVPCARP-GGF--PSSQVILPLAHTI-E--HE----E--FLEVV-R-
XP_017437412.1/1-285 AYSAACLKASPY-AVPCARP-GGF--PSSQVILPLAHTI-E--HE----E--FLEVV-R-
XP_014518680.1/1-285 AYSAACLKASPY-AVPCARP-GGF--PSSQVILPLAHTI-E--HE----E--FLEVV-R-
XP_003546255.1/1-285 AYSAACLKASPY-AVPCARP-GGF--PSTQVILPLAHTI-E--HE----E--FLEVV-R-
XP_020233213.1/1-285 AYSAACLKASPY-AVPCARP-GGF--PSSQVILPLAHTI-E--HE----E--FLEVV-R-
XP_003535078.1/1-285 AYSAACLKASPY-AVPCARP-GGF--PSTQVILPLAHTI-E--HE----E--FLEVV-R-
XP_014617344.1/1-285 AYSAACLKASPY-AVPCARP-GGF--PSTQVILPLAHTI-E--HE----E--FLEVV-R-
XP_015959291.1/1-283 AYSAACLKATPY-AVPCARP-GGF--PSTQVILPLAHTI-E--HE----E--FLEVV-R-
XP_016197724.1/1-283 AYSAACLKATPY-AVPCARP-GGF--PSTQVILPLAHTI-E--HE----E--FLEVV-R-
XP_025694278.1/1-283 AYSAACLKATPY-AVPCARP-GGF--PSTQVILPLAHTI-E--HE----E--FLEVV-R-
XP_006846777.1/1-281 AYCAASLKANPF-AIPGSGP-SRF--TGSQIILPLAHTV-E--NE----E--FVEVI-R-
XP_003574071.1/1-280 AYSASALKTSSC-SLPGLRP-MRF--SGSQIIMPLAHTV-E--NE----E--ILEVV-R-
XP_020174778.1/1-278 AYSASALKSSSC-SLPGLRP-MRF--SGSQIIMPLAHTV-E--NE----E--ILEVV-R-
XP_006661867.1/1-281 AYSASSLKTSSC-SLPGLRP-MRF--SGSQIIMPLAHTV-E--NE----E--ILEVV-R-
XP_015614901.1/1-281 AYSASSLKTSSC-SLPGLRP-MRF--SGSQIIMPLAHTV-E--NE----E--ILEVV-R-
XP_004982838.1/1-282 AYSASALKTSPC-SLPGLRS-MRF--SGSQIIMPLAHTV-E--NE----E--ILEVV-R-
XP_025796396.1/1-282 AYSASALKTSPC-SLPGLRP-MRF--SGSQIIMPLAHTV-E--NE----E--ILEVV-R-
NP_001316502.1/1-284 AYSASALKTSPC-SLPGLRP-MRF--SGSQIIMPLAHTV-E--NE----E--ILEVV-R-
XP_021306737.1/1-282 AYSASALKTSPC-SLPGLRP-MRF--SGSQIIMPLAHTV-E--NE----E--ILEVV-R-
XP_021306733.1/1-293 AYSASALKTSPC-SLPGLRP-MRF--SGSQIIMPLAHTV-E--NE----E--ILEVV-R-
XP_020162053.1/1-281 AYLASAVKTSTC-SFPGLRP-MRF--SGSQIIMPLAHTV-E--NE----E--ILEVV-R-
XP_006649248.1/1-282 AYLASTLKTSAC-SLPGLRP-MRF--SGSQIIIPLAHTV-E--NE----E--ILEVV-R-
XP_015632709.1/1-282 AYLASTLKTSAC-SLTGLRP-MRF--SGSQIIIPLAHTV-E--NE----E--ILEVV-R-
NP_001296150.1/1-282 AYLASSLKTSAC-SLPGLRP-MRF--SEGQMIMPLAHTV-E--NE----E--ILEVV-R-
XP_008665074.1/1-283 AYLASSLKTSAC-SLPGLRP-MRF--SGGQMIMPLAHTV-E--NE----E--ILEVV-R-
NP_001105994.1/1-283 AYLASSLKTSAC-SLPGLRP-MRF--SGGQMIMPLAHTV-E--NE----E--ILEVV-R-
XP_008660404.1/1-283 AYLASSLKTSAC-SLPGLRP-MRF--SGGQMIMPLAHTV-E--NE----E--ILEVV-R-
XP_002468669.1/1-283 AYLASSLKTSAC-SLPGLRP-MRF--SGGQMIMPLAHTV-E--NE----E--ILEVV-R-
XP_021309716.1/1-285 AYLASSLKTSAC-SLPGLRP-MRF--SGGQMIMPLAHTV-E--NE----E--ILEVV-R-
XP_004986037.1/1-283 AYLASSLKASAC-SLPGLRP-MRF--SGGQMIMPLAHTV-E--NE----E--ILEVV-R-
XP_025795001.1/1-283 AYLASSLKASAC-SLPGLRP-MRF--SGGQMIMPLAHTV-E--NE----E--ILEVV-R-
XP_020093893.1/1-283 AYCAATLKASSC-SFPALRP-TRF--PGSQIIMSLGHTV-E--NE----E--ILEVV-R-
XP_020093894.1/1-282 AYCAATLKASSC-SFPALRP-TRF--PGSQIIMSLGHTV-E--NE----E--ILEVV-R-
XP_020259754.1/1-283 AYSASSLKAGSA-FFPGLRS-TRF--SGSQTIMHLAHTV-E--NE----E--LLEVV-R-
XP_009401955.1/1-282 AYSAASLKAGVS-SFSGSRP-NRF--SGSQIIMPLAHTV-E--NE----E--FLEVV-R-
XP_008791541.1/1-283 AYSAASLKTGSC-SFPGLRP-LRF--SGSQIIMPLAHTV-E--NE----E--FLEVV-R-
XP_017698637.1/1-283 AYSAASLKTGSC-SFPGLRP-LRF--SGSQIIMPLAHTV-E--NE----E--FLEVV-R-
XP_010920422.1/1-283 AYSAASLKAGSC-SFPGLRP-LRF--SGSQIIMALAHTV-E--NE----E--FLEVV-R-
XP_010920423.1/1-283 AYSAASLKAGSC-SFPGLRP-LRF--SGSQIIMALAHTV-E--NE----E--FLEVV-R-
XP_010920419.1/1-283 AYSAASLKAGSC-SFPGLRP-LRF--SGSQIIMALAHTV-E--NE----E--FLEVV-R-
XP_010920426.1/1-283 AYSAASLKAGSC-SFPGLRP-LRF--SGSQIIMALAHTV-E--NE----E--FLEVV-R-
XP_019709326.1/1-287 AYSATSLKAGSC-SFPGLRH-CRF--SGSQIIMPLAHTV-E--NE----E--FLEVV-R-
XP_008785281.1/1-283 AYSATSLKAGSG-SFPGLGP-CRF--SGSQIIMPLAHTV-E--NE----E--FLEVV-R-
XP_008462593.1/1-288 AYSAASLKTGSY-AYPGMRP-TRF--TGSQIIMPLGHTI-E--QE----E--LLEVV-R-
XP_022969629.1/1-286 AYSAASLKTGSY-VYPGMRP-TRF--TGSQIIMPLGHTI-E--QE----E--LLEVV-R-
XP_023536970.1/1-286 AYSAASLKTGSY-VYPGMRP-TRF--TGSQIIMPLGHTI-E--QE----E--LLEVV-R-
XP_022937855.1/1-288 AYSAASLKTGSY-VYPGMRP-TRF--TGSQIIMPLGHTI-E--QE----E--LLEVV-R-
XP_022937858.1/1-288 AYSAASLKTGSY-VYPGMRP-TRF--TGSQIIMPLGHTI-E--QE----E--LLEVV-R-
XP_012827869.1/1-285 AYAAASLK-STY-AYPGMRDHTRF--TGSQIIMPLGQTI-E--YE----E--MLEVI-R-
XP_022874815.1/1-279 AYSAAALKASSY-SYPGMRP-TRF--TGSQIIMPLGHTI-E--HF----Q--MLEMI-R-
XP_022874814.1/1-283 AYSAAALKASSY-SYPGMRP-TRF--TGSQIIMPLGHTI-E--HF----Q--MLEMI-R-
XP_018434000.1/1-280 AYSAATLKAGAF-AYSGIRS-TAF--TGSQIIMPLGNTI-E--KE----E--MLEVV-R-
XP_013642496.1/1-279 AYSAATLKAGAF-AYSGMRP-TTF--TGSQIIMPLGNTI-E--KE----E--MLEVV-R-
XP_009150087.1/1-279 AYSAATLKAGAF-AYSGMRP-TTF--TGSQIIMPLGNTI-E--KE----E--MLEVV-R-
XP_009150088.1/1-279 AYSAATLKAGAF-AYSGMRP-TTF--TGSQIIMPLGNTI-E--KE----E--MLEVV-R-
XP_022563181.1/1-279 AYSAATLKAGAF-AYSRMRP-TTF--TGSQIIMPLGNTI-E--KE----E--MLEVV-R-
XP_013675790.1/1-280 AYSAATLKAGAF-AYSRMRP-TTF--TGSQIIMPLGNTI-E--KE----E--MLEVV-R-
XP_013623090.1/1-279 AYSAATLKAGAF-AYSRMRP-TTF--TGSQIIMPLGNTI-E--KE----E--MLEVV-R-
XP_013623089.1/1-280 AYSAATLKAGAF-AYSRMRP-TTF--TGSQIIMPLGNTI-E--KE----E--MLEVV-R-
XP_020885782.1/1-285 AYSAATLKAGTF-AYPGMRP-TRF--TGSQIIMPLGHTI-E--HE----E--MLEVV-R-
XP_006280004.1/1-285 AYSAATLKAGTF-AYPGMRP-TRF--TGSQIIMPLGHTI-E--HE----E--MLEVV-R-
XP_019088563.1/1-285 AYSAATLKAGTF-AYPGMRP-TRF--TGSQIIMPLGHTI-E--HE----E--MLEVV-R-
XP_010483719.1/1-285 AYSAATLKAGTF-AYPGMRP-TRF--TGSQIIMPLGHTI-E--HE----E--MLEVV-R-
XP_010456679.1/1-285 AYSAATLKAGTF-AYPGMRP-TRF--TGSQIIMPLGHTI-E--HE----E--MLEVV-R-
XP_006400821.1/1-284 AYSAATLKAGTF-AYPGMRP-TRF--TGSQIIMPLGHTI-E--HE----E--MLEVV-R-
NP_200877.1/1-284    AYSAATLKAGSF-AYPGMRP-TRF--TGSQIIMPLGHTI-E--HE----E--MLEVV-R-
XP_013611543.1/1-282 AYSAATLKAGSF-AYPGMRP-TRF--TGSQIIMPLGHTI-E--HE----E--MLEVV-R-
XP_009120605.1/1-284 AYSAATLKAGSF-AYPGMRP-TRF--TGSQIIMPLGHTI-E--HE----E--MLEVV-R-
XP_013667152.1/1-284 AYSAATLKAGSF-AYPGMRP-TRF--TGSQIIMPLGHTI-E--HE----E--MLEVV-R-
XP_018446535.1/1-284 AYSAATLKAGSF-AYPGMRP-TRF--TGSQIIMPLGHTI-E--HE----E--MLEVV-R-
XP_018454374.1/1-284 AYSAATLKADTF-AYPVVRP-TRF--TGSQVIMPLGHTI-E--HE----E--MLEVV-R-
XP_009126588.1/1-285 AYSAATLKAGAF-AYPGVRP-TRF--TGSQIIMPLGHTI-E--HE----E--MLEVV-R-
XP_013680521.1/1-285 AYSAATLKAGAF-AYHGVRP-TRF--TGSQIIMPLGHTI-E--HE----E--TLEVV-R-
XP_013612427.1/1-285 AYSAATLKTGAF-AYPGVRP-TRF--TGTQIIMPLGHTI-E--HE----E--TLEVV-R-
XP_010535608.1/1-283 AYSAATLKAGTY-GYPGMRP-TRF--TGSQIIMPLGHTI-E--HE----E--MLEVV-R-
XP_010520372.1/1-276 AYSAATLKAGTY-SYPGMRP-TRF--TGSQIIMPLGHTI-E--HE----E--MLEVV-R-
XP_004487103.1/1-286 AFSAASLKAGSY-AYPGLRP-TRF--TGNQAIMPLGHSI-E--HE----E--MLEVI-R-
XP_003597252.1/1-283 AFSAASLKAGSY-GYPGMRS-TKF--TGNQAIMPLGHTI-E--HE----E--MLEII-R-
XP_024455683.1/1-286 AYSAALWKAGSY-AYPGMRP-MRF--TGSQITMPLGHTI-E--QE----D--LLEVI-R-
XP_022026352.1/1-288 AYSAASVKANSYNTYPGMRP-TRF--TGSQIIMPLGHTI-E--HE----E--MLEVV-R-
XP_022000486.1/1-288 AYSAASVKPNSYNTHPGMRP-TRF--TGSQIIMPLGHTI-E--HE----E--MLEVV-R-
XP_016717477.1/1-281 AYSAASLKAGTY-TYPGMRP-TSF--TGSQIIMPLGQTV-E--HE----E--LLEVI-R-
XP_017643014.1/1-281 AYSAASLKAGTY-AYPGMRP-TSF--TGSQIIMPLGQTV-E--HE----E--LLEVI-R-
XP_016724213.1/1-281 AYSAASLKAGTY-AYPGMRP-TSF--TGSQIIMPLGQTA-E--HE----E--LLEVI-R-
XP_012462938.1/1-281 AYSAASLKAGTY-AYPGMRP-TSF--TGSQIIMPLGQTV-E--HE----E--LLEVI-R-
XP_012471509.1/1-292 AYTAASLKAGTY-AYPGMRP-TRF--SGSQIIMPLGHTI-E--HE----E--ILEVI-R-
XP_017642742.1/1-292 AYTAASLKAGTY-AYPGMRP-TRF--TGSQIIMPLGHTI-E--HE----E--ILEVI-R-
XP_016755876.1/1-292 AYTAASLKAGTY-AYPGMRP-TRF--TGSQIIMPLGHTI-E--HE----E--ILEVI-R-
XP_012471510.1/1-286 AYTAASLKAGTY-AYPGMRP-TRF--SGSQIIMPLGHTI-E--HE----E--ILEVI-R-
XP_016753711.1/1-286 AYTAASLKAGTY-AYPGMRP-TRF--TGSQIIMPLGHTI-E--HE----E--ILEVI-R-
XP_017642744.1/1-286 AYTAASLKAGTY-AYPGMRP-TRF--TGSQIIMPLGHTI-E--HE----E--ILEVI-R-
XP_016755877.1/1-286 AYTAASLKAGTY-AYPGMRP-TRF--TGSQIIMPLGHTI-E--HE----E--ILEVI-R-
XP_016750468.1/1-286 AYCATSLKAGTN-AYPGMRH-TRF--TGSQIIMPLGHTI-E--HE----E--LLEVI-R-
XP_016688657.1/1-286 AYCAASLKAGTN-AYPGMRP-TRF--TGSQIIMPLGHTI-E--HE----E--LLEVI-R-
XP_012443272.1/1-286 AYCAASLKAGTN-AYPGMRP-TRF--TGSQIIMPLGHTI-E--HE----E--LLEVI-R-
XP_017603001.1/1-286 AYCAASLKAGTN-AYPGMRP-TRF--TGSQIIMPLGHTI-E--HE----E--LLEVI-R-
XP_022755632.1/1-285 AYCAASLKAGTY-AYPGMRP-TRF--TGSQIIMPLGHTI-E--NE----E--LLEVI-R-
XP_022755633.1/1-285 AYCAASLKAGTY-AYPGMRP-TRF--TGSQIIMPLGHTI-E--NE----E--LLEVI-R-
XP_022729815.1/1-286 AYSAASLKAGTY-AYPGMRP-TRF--TGSQIIMPLGHTI-E--NE----E--LLEVI-R-
XP_022729816.1/1-286 AYSAASLKAGTY-AYPGMRP-TRF--TGSQIIMPLGHTI-E--NE----E--LLEVI-R-
XP_022752857.1/1-286 AYSAASLKAGTY-AYPGMRP-TRF--TGSQTIMPLGHTI-E--HE----E--LLEVI-R-
XP_022752856.1/1-286 AYSAASLKAGTY-AYPGMRP-TRF--TGSQTIMPLGHTI-E--HE----E--LLEVI-R-
XP_021277981.1/1-286 AYSAASLKAGTY-SYPGMRP-TRF--TGSQIIMPLGHTI-E--HE----E--LLEVI-R-
XP_017983554.1/1-286 AYSALSLKAGTY-SYPGMRP-TRF--TGSQIIMPLGHTI-E--HE----E--LLEVI-R-
XP_011089912.1/1-283 AYAAVSLKASTY-AYPGMRP-MRF--TGSQIIMPLGHTI-E--HE----E--MLEVI-R-
XP_007208090.1/1-286 AYSAASLKAGSY-AYPGMRP-TRF--TGGQIIMPLGHTI-E--HE----E--LLEVV-R-
XP_008225704.1/1-286 AYSAASLKAGSY-AYPGMRP-TRF--TGGQIIMPLGHTI-E--HE----E--LLEVV-R-
XP_021830853.1/1-286 AYAAASLKAGSY-AYPGMRP-TRF--TGGQIIMPLGHTI-E--HE----E--LLEVV-R-
XP_008349754.1/1-286 AYSATSMKAGXY-AYPGMRP-TRF--TGGQIIMPLGHTI-E--QE----E--LLEVV-R-
NP_001315783.1/1-288 AYSATSMKAGAY-AYPGMRP-TRF--TGGQIIMPLGHTI-E--QE----E--LLEVV-R-
XP_009367069.1/1-286 AYSAASLKAGSY-AYPGMRP-TRF--TGGQIIMPLGHTI-E--QE----E--LLEVV-R-
XP_008344872.1/1-286 AYSAASLKAGSY-AYPGMRP-TRF--TGGQIIMPLGHTI-E--QE----E--LLEVV-R-
XP_015952399.1/1-286 AYAAASPKAGSY-AYPGMRP-TRF--TGSQIIMPLGHTI-E--HE----E--MLEVI-R-
XP_020982118.1/1-286 AYAAASLKAGSY-AYPGMRP-TRF--TGSQIIMPLGHTI-E--HE----E--MLEVI-R-
XP_025685402.1/1-286 AYAAASLKAGSY-AYPGMRP-TRF--TGSQIIMPLGHTI-E--HE----E--MLEVI-R-
XP_016187430.1/1-286 AYAAASLKAGSY-AYPGMRP-TRF--TGSQIIMPLGHTI-E--HE----E--MLEVI-R-
XP_020214107.1/1-284 AYAAASLKAGTY-AYPGMRP-TRF--TGSQIIMPLGHTI-E--HE----E--MLEVI-R-
XP_006592258.1/1-284 AYSAASLKAGTY-AYPGMRP-TRF--TGSQIIMPLGHTI-E--HE----E--MLEVI-R-
XP_007132262.1/1-285 AYSAASLKAGTY-AYPGMRP-TRF--TGSQIIMPLGHTI-E--HE----E--MLEVI-R-
XP_003538150.1/1-285 AYSAASLKAGTY-AYPGMRP-TRF--TGSQIIMPLGHTI-E--HE----E--MLEVI-R-
XP_017433493.1/1-285 AYSAASLKAGTY-AYPGMRP-TRF--TGSQIIMPLGHTI-E--HE----E--MLEVI-R-
XP_014494018.1/1-285 AYSAASLKAGTY-AYPGMRP-TRF--TGSQIIMPLGHTI-E--HE----E--MLEVI-R-
XP_010089983.2/1-286 GYTASALKAGSY-AFPGMRL-TRF--TGSQIIMPLGHTI-E--HE----E--LLEVV-R-
XP_006378974.2/1-285 AYSAASLKAGSY-AYPGMRS-MRF--TGSQIIMPLGHTI-E--QE----E--LLEVI-R-
XP_011010051.1/1-285 AYSAASLKAGSY-AYPGMRS-MRF--TGSQIIMPLGHTI-E--QE----E--LLEVI-R-
XP_011010050.1/1-285 AYSAASLKAGSY-AYPGMRS-MRF--TGSQIIMPLGHTI-E--QE----E--LLEVI-R-
XP_021905657.1/1-285 AYSAASLKAGSF-AYPGMRP-TRF--TGSQIIMPLGHTI-E--HE----E--LLEVI-R-
XP_021610391.1/1-286 AYSAVSLKAGSY-VFPGMRA-TRF--TGSQIIMPLGHTI-E--HE----E--LLEVV-R-
XP_002529946.1/1-286 AYSAASLKAGSY-AFPGMRP-TRF--TGSQIIMPLGHTI-E--HE----E--LLEVI-R-
XP_021658728.1/1-286 AYSAASLKAGSY-AFPGMRP-TRF--TGSQVIMPLGHTI-E--HE----E--LLEVI-R-
XP_012064816.1/1-286 AYSAASLKAGSY-AFPGMRP-TRF--TGSQIIMPLGHTI-E--HE----E--LLEVI-R-
XP_021628343.1/1-286 AYSAASLKAGSY-AFPGMRP-ARF--TGSQIIMPLGHTI-E--HE----E--LLEVI-R-
XP_021657859.1/1-286 AYSAASLKAGSY-AFPGMRP-ARF--TGSQIIMPLGHTI-E--HE----E--LLEVI-R-
XP_015874442.1/1-285 AYSAAALKAGSY-AYPGMRP-TRF--TGGQIIMPLGPTI-E--HE----E--LLEVV-R-
XP_023926843.1/1-286 AYSAASLKAGSY-AYPGMRP-TRF--TGSQIIMPLGHTI-E--HE----E--LLEVI-R-
XP_018819529.1/1-286 AYSAASLKAGPY-THPGMRP-TRF--TGSQIIMPLGHTI-E--HE----E--VLEVI-R-
XP_010651164.1/1-286 AYSAASLKASPY-SYPGMRP-TRF--TGSQIIMPLGHTI-E--HE----E--LLEVI-R-
XP_024034672.1/1-286 AYSAASLKAGSY-AYPGMRP-TRF--TGSQIIMPLGHTI-E--HE----E--LLEVI-R-
XP_006488084.1/1-286 AYSAASLKAGSY-AYPGMRP-TRF--TGSQIIMPLGHTI-E--HE----E--LLEVI-R-
XP_024034673.1/1-286 AYSAASLKAGSY-AYPGMRP-TRF--TGSQIIMPLGHTI-E--HE----E--LLEVI-R-
XP_010264677.1/1-287 AYAAASMKAGSY-AFQGLRP-TRF--SGSQVIMPLGHTV-E--QE----E--LLEVI-R-
XP_010253680.1/1-288 AYSAGSLKSGSY-AFQGLRS-TRF--SGSQVVMPLGHTV-E--HE----E--LLEVI-R-
XP_022005829.1/1-282 AYSAASVKASPY-GYQGTRP-TRF--TGSQIIMPLGQTI-E--HE----E--MLEVI-R-
XP_023751537.1/1-278 AYSAASVKASPY-GYPGMRP-TRF--TGSQIIMPLGQTI-E--HE----E--MLEVI-R-
XP_024971452.1/1-283 AYSAASVKASPY-GNPGMRP-TRF--TGSQIIMPLGQTI-E--HE----E--MLEVI-R-
XP_024971444.1/1-287 AYSAASVKASPY-GNPGMRP-TRF--TGSQIIMPLGQTI-E--HE----E--MLEVI-R-
XP_024985647.1/1-287 AYSAASVKANPY-AYPGMRP-TRF--TGSQIIMPLGHTI-E--HE----E--MLEVV-R-
XP_023749448.1/1-288 AYSAASVKPNPY-SYPGMRP-TRF--TGSQIIMPLGHTI-E--HE----E--MLEVV-R-
XP_010689538.1/1-281 AYSAAALKANSY-AFPGMRA-TRF--TGSQIIMPLGHTI-E--NE----E--MLEVI-R-
XP_021858740.1/1-280 AYSAAALKSNAY-AFPGMRA-TRF--TGSQIIMPLGHTI-E--NE----E--MLEVI-R-
XP_021773317.1/1-281 AYSAAALKSNAY-AFPGMRA-TRF--TGSQIIMPLGHTI-E--NE----E--MLEVI-R-
XP_021745941.1/1-281 AYSAAALKSNAY-AFPGMRA-TRF--TGSQIIMPLGHTI-E--NE----E--MLEVI-R-
XP_016729039.1/1-286 AYSAASLKAGSY-AYPGMRP-TRF--TGSQIIMPLGHTI-E--HE----E--LLEVV-R-
XP_017623828.1/1-286 AYSAASLKAGSY-AYPGMRP-TRF--TGSQIIMQLGHTI-E--HE----E--LLEVV-R-
XP_012476406.1/1-286 AYSAVSLKAGSY-AYPGMRP-TRF--TGSQIIMPLGHTI-E--HE----E--LLEVV-R-
XP_016754776.1/1-286 AYSAVSLKAGSY-AYPGMRP-TRF--TGSQIIMPLGHTI-E--HE----E--LLEVV-R-
XP_022152944.1/1-286 AYSAASLKTGSY-AYPGMRP-TRF--TGSQIIMPLGHTI-E--QE----E--LLEVV-R-
XP_011040884.1/1-286 AYSAALWKAGSY-AYPGMRP-MRF--TGSQITMPLGHTI-E--QE----E--LLEVI-R-
XP_010043753.1/1-286 AYSAASLKASPF-GYPGMRP-TRF--TGSQIIMPLGHTI-E--HE----E--MLEVI-R-
XP_024158223.1/1-285 AYSAASLKSGSY-AYPGMRP-TRF--TGGQIIMPLGHTI-E--HE----E--LLEVV-R-
XP_004291573.1/1-285 AYSAASLKSGSY-AYPGMRP-TRF--TGGQIIMPLGHTI-E--QE----E--LLEVV-R-
XP_018808765.1/1-285 AYSAASLKAGSY-TFPGMRP-TRF--TGSQIIMPLGHTI-E--HE----E--VLEII-R-
XP_023512455.1/1-283 AYSAATLKANSY-AYPGMRP-TRF--TGSQIIMPLGHTI-E--NE----E--LLEVI-R-
XP_023512456.1/1-283 AYSAATLKANSY-AYPGMRP-TRF--TGSQIIMPLGHTI-E--NE----E--LLEVI-R-
XP_022944186.1/1-283 AYSAATLKANSY-AYPGMRP-TRF--TGSQIIMPLGHTI-E--NE----E--LLEVI-R-
XP_022944185.1/1-283 AYSAATLKANSY-AYPGMRP-TRF--TGSQIIMPLGHTI-E--NE----E--LLEVI-R-
XP_022985614.1/1-283 AYSAATLKANSY-AYPGMRP-TRF--TGSQIIMPLGHTI-E--NE----E--LLEVI-R-
XP_022985615.1/1-283 AYSAATLKANSY-AYPGMRP-TRF--TGSQIIMPLGHTI-E--NE----E--LLEVI-R-
XP_011656996.1/1-283 AYSAATLKANSY-TYPGMRP-TRF--TGSQIIMPLGHTI-E--HE----E--LLEVI-R-
XP_011656997.1/1-283 AYSAATLKANSY-TYPGMRP-TRF--TGSQIIMPLGHTI-E--HE----E--LLEVI-R-
XP_008463945.1/1-283 AYSAATLKANSY-TYPGMRP-TRF--TGSQIIMPLGHTI-E--HE----E--LLEVI-R-
XP_008463946.1/1-283 AYSAATLKANSY-TYPGMRP-TRF--TGSQIIMPLGHTI-E--HE----E--LLEVI-R-
XP_022140363.1/1-286 AYSAATLKANSY-AYPGMRP-TRF--TGSQIIMPLGHTI-E--HE----E--LLEVI-R-
XP_022995706.1/1-286 AYSAATLKANSY-AYPGMRP-TRF--TGSQIIMPLGHTI-E--HE----E--LLEVI-R-
XP_023534373.1/1-286 AYSAATLKANSY-AYPGMRP-TRF--TGSQIIMPLGHTI-E--HE----E--LLEVI-R-
XP_022958713.1/1-286 AYSAATLKANSY-AYPGMRP-TRF--TGSQIIMPLGHTI-E--HE----E--LLEVI-R-
XP_025614062.1/1-285 AYSAASLKSGSY-AYPGMRP-TRF--TGNQTIMSLGHTI-E--HQ----E--MLEVI-R-
XP_025614063.1/1-285 AYSAASLKSGSY-AYPGMRP-TRF--TGNQTIMSLGHTI-E--HQ----E--MLEVI-R-
XP_016170485.1/1-285 AYSAASLKSGSY-AYPGMRP-TRF--TGNQTIMSLGHTI-E--HQ----E--MLEVI-R-
XP_016170486.1/1-285 AYSAASLKSGSY-AYPGMRP-TRF--TGNQTIMSLGHTI-E--HQ----E--MLEVI-R-
XP_015935757.1/1-285 AYSAASLKSGSY-AYPGMRP-TRF--TGNQTIMSLGHTI-E--HQ----E--MLEVI-R-
XP_015935758.1/1-285 AYSAASLKSGSY-AYPGMRP-TRF--TGNQTIMSLGHTI-E--HQ----E--MLEVI-R-
XP_004505999.1/1-286 AYSAASLKAGNY-AYPGMRP-TRF--TGNQIIMPLGHTI-E--HE----E--MLEVI-R-
XP_013456006.1/1-286 AYSAASLKAGTY-AYPGMRP-TRF--TGNQIIMPLGNTI-E--HE----E--MLEVI-R-
XP_019453966.1/1-285 AYAAASLKSGTY-AHPGMRP-TRF--TGSQIIMPLGHTI-E--HE----E--MLEVI-R-
XP_019448740.1/1-279 AYAAASLKADTY-AYPGMRP-TRF--TGNQIIMPLGHTI-E--HE----E--MLEVI-R-
XP_019448741.1/1-279 AYAAASLKADTY-AYPGMRP-TRF--TGNQIIMPLGHTI-E--HE----E--MLEVI-R-
XP_019458095.1/1-286 AYSAAALKADSY-AYPGMRP-IKF--TGNQIIMPLGHTI-E--HE----E--MLEVI-R-
XP_019447870.1/1-286 AYSAASLKSGSY-TYPGTRP-TSF--TGNQIIMPLGHTI-E--HE----E--MLEVV-R-
XP_012838629.1/1-280 AYAAASLKASTY-GCTGIRA-TRF--TGSQIIMPLGHTI-E--HE----E--MLEVI-R-
XP_012838630.1/1-280 AYAAASLKASTY-GCTGIRA-TRF--TGSQIIMPLGHTI-E--HE----E--MLEVI-R-
XP_012838627.1/1-280 AYAAASLKASTY-GCTGIRA-TRF--TGSQIIMPLGHTI-E--HE----E--MLEVI-R-
XP_012838628.1/1-280 AYAAASLKASTY-GCTGIRA-TRF--TGSQIIMPLGHTI-E--HE----E--MLEVI-R-
XP_017214964.1/1-286 AYSAASLKGSSY-AYPEMRP-TRF--TGSQIIMPLGQTI-E--HE----E--MLEVI-R-
XP_017234817.1/1-286 AYSAASLKGSSY-AYPEMRP-TRF--TGNQIIMPLGQTI-E--HE----E--MLEVI-R-
XP_019178153.1/1-282 AYSATALKTNSC-AYPGMRP-TRF--TGSQIIMPLGHTI-E--HE----E--MLEVI-R-
XP_019166562.1/1-283 AYFAAALKSSSY-SYPGMRP-TRF--TGSQIIMPLGQTI-E--HE----E--MLEVI-R-
XP_011089294.1/1-282 AYAAAALK-NTY-AYPGMRP-TRF--TGSQIIMPLGHTI-E--HE----E--MLEVI-R-
XP_022892122.1/1-274 AFSATSLKASSY-AYPGMRP-TRF--TGGQIIMPLGHTI-E--HE----E--MLEVI-R-
XP_022883393.1/1-279 AFSATSLKARLY-AYPGMRP-TGF--TGSQIIMPLGNTI-E--HE----E--MLEVI-R-
XP_022883398.1/1-279 AFSATSLKARLY-AYPGMRP-TGF--TGSQIIMPLGNTI-E--HE----E--MLEVI-R-
XP_022883384.1/1-283 AFSATSLKARLY-AYPGMRP-TGF--TGSQIIMPLGNTI-E--HE----E--MLEVI-R-
XP_022883389.1/1-283 AFSATSLKARLY-AYPGMRP-TGF--TGSQIIMPLGNTI-E--HE----E--MLEVI-R-
XP_009776589.1/1-283 AYVASSMKSCSY-AYPGMRP-TRF--TGSQIIMPLGHTI-E--HE----E--MLEVI-R-
NP_001312504.1/1-283 AYVASSMKSCSY-AYPGMRP-TRF--TGSQIIMPLGHTI-E--HE----E--MLEVI-R-
XP_016472209.1/1-283 AYVASSMKSCSY-AYPGMRP-TRF--TGSQIIMPLGHTI-E--HE----E--MLEVI-R-
XP_019255429.1/1-283 AYVASSMKSCSY-AYPGMRP-TRF--TGSQIIMPLGHTI-E--HE----E--MLEVI-R-
XP_009624234.1/1-283 AYVASSMKSCSY-AYPGVRP-TRF--TGSQIIMPLGHTI-E--HE----E--MLEVI-R-
XP_016504967.1/1-283 AYVASSMKSCSY-AYPGVRP-TRF--TGSQIIMPLGHTI-E--HE----E--MLEVI-R-
XP_016547131.1/1-283 AYVASSLKSCPY-SYPGMRP-TRF--TGSQIIMPLGHTI-E--HE----E--MLEVI-R-
XP_004251138.1/1-283 AFVASALKSCPY-TYPGMRP-TRF--TGSQIIMPLGHTI-E--HE----E--MLEVI-R-
XP_015058512.1/1-283 AFVASALKSCPY-TYPGMRP-TRF--TGSQIIMPLGHTI-E--HE----E--MLEVI-R-
XP_006340193.1/1-283 AFVASALKSCPY-TYPGMRP-TRF--TGSQIIMPLGHTI-E--HE----E--MLEVI-R-
XP_004235650.1/1-283 AYAAAAIKVGPC-SLPGA-R-VGN--FGGQVILPLAHTV-E--HE----E--LLEVI-K-
XP_006343047.1/1-283 AYAAAAIKVGPC-SLPGA-R-VGN--FGGQVILPLAHTV-E--HE----E--LLEVI-K-
XP_009774805.1/1-285 AYSAAAIKVGPC-CLPGT-R-VGN--FGGQVILPLAHTV-E--HE----E--LLEVI-K-
XP_016498616.1/1-285 AYSAAAIKVGPC-CLPGT-R-VGN--FGGQVILPLAHTV-E--HE----E--LLEVI-K-
XP_016434103.1/1-285 AYSAAAIKVGPC-CLPGA-Q-VGN--FGGQVILPLAHTV-E--HE----E--LLEVI-K-
XP_009593967.1/1-285 AYSAAAIKVGPC-CLPGA-Q-VGN--FGGQVILPLAHTV-E--HE----E--LLEVI-K-
XP_004510297.1/1-285 AYTAAAIKVGPC-SLTGS-R-IGN--YGGQVILPLAHTI-E--HE----E--FLEVI-K-
XP_004510296.1/1-285 AYTAAAIKVGPC-SLTGS-R-IGN--YGGQVILPLAHTI-E--HE----E--FLEVI-K-
XP_003627005.1/1-284 AYTAAAIKVGPC-SLTGS-R-VGN--YGGQVILPLAHTI-E--HE----E--FLEVI-K-
XP_003531653.1/1-284 AYTAAAIKVGPC-SFSGS-R-VGN--YGGQVILPLAHTI-E--HE----E--FLEVI-K-
XP_006585576.1/1-288 AYTAAAIKVGPC-SFSGS-R-VGN--YGGQVILPLAHTI-E--HE----E--FLEVI-K-
XP_014634618.1/1-288 AYTAAAIKVGPC-SFSGS-R-VGN--YGGQVILPLAHTI-E--HE----E--FLEVI-K-
XP_014634619.1/1-288 AYTAAAIKVGPC-SFSGS-R-VGN--YGGQVILPLAHTI-E--HE----E--FLEVI-K-
XP_003531652.1/1-285 AYTAAAIKVGPC-SLSGS-C-VGN--FGGQVILPLAHTI-E--HE----E--FLEVI-K-
XP_003530109.1/1-284 AYTAAAIKVGPC-SLSGS-R-VGN--YGGQVILPLAHTI-E--HE----E--FLEVI-K-
XP_007135643.1/1-285 AYTAAAIKVGPC-SLSGS-R-VGN--YGGQVILPLAHTI-E--HE----E--CLEVI-K-
XP_003530112.1/1-285 AYTAAAIKVGPC-SLSGS-R-VGN--YGGQVILPLAHTI-E--HE----E--FLEVI-K-
XP_020226142.1/1-285 AYTAAAIKVGPC-SLSGS-R-VGN--YGGQVILPLAHTI-E--HE----E--FLEVI-K-
XP_014516466.1/1-285 AYTAAAIKVGPC-SLSGS-R-VGN--YGGQVILPLAHTI-E--HE----E--FLEVI-K-
XP_017407594.1/1-285 AYTAAAIKVGPC-SVSGS-R-VGN--YGGQVILPLAHTI-E--HE----E--FLEVI-K-
XP_019448178.1/1-285 AYSAAAIKVGPS-SFSGS-R-VGN--YGGQVILPLAHTI-E--HE----E--FLEVI-K-
XP_019429328.1/1-285 AYSAAAVKVGPC-SFSGS-R-VAN--YGGQVILPLAHTV-E--HE----E--FLEVI-K-
XP_004486776.1/1-285 AYSAAAIKVGPC-SLSGS-R-VGN--YGGQVILPLAHTI-E--HE----E--FLEVI-K-
XP_003597690.1/1-286 AYSAAAIKAGPC-SFSGS-R-VGN--YGGQVILPLAHTI-E--HE----E--FLEVI-K-
XP_015932679.1/1-285 AYSAAAIKVGPC-SLSGS-R-VGN--YGGQVILPLAHTI-E--HE----E--FLEVI-K-
XP_025674177.1/1-285 AYSAAAIKVGPC-SLSGS-R-VGN--YGGQVILPLAHTI-E--HE----E--FLEVI-K-
XP_025610204.1/1-285 AYSAAAIKVGPC-SLSGS-R-VGN--YGGQVILPLAHTI-E--HE----E--FLEVI-K-
XP_020226055.1/1-285 AYSAAAIKVGPC-GLPGS-R-VGN--YGGQVILPLAHTI-E--HE----E--FLEVI-K-
XP_007150614.1/1-285 AYSAAAIKVGPC-GLPGS-R-VGN--YGGQVILPLAHTI-E--HE----E--FLEVI-K-
XP_014501306.1/1-285 AYSAAAIKVGPC-GLPGS-R-VGN--YGGQVILPLAHTI-E--HE----E--FLEVI-K-
XP_017425270.1/1-285 AYSAAAVKVGPC-GLPGT-R-VGN--YGGQVILPLAHTI-E--HE----E--FLEVI-K-
XP_021836126.1/1-285 AYLAAAAKISPC-SLPGA-R-IGG--FGNQVILPLAHTI-E--HE----E--LLEVI-K-
XP_010691423.1/1-284 AYLAAAAKISPC-SLPGA-R-IGG--FGNQVILPLAHTI-E--HE----E--LLEVI-K-
XP_010691424.1/1-284 AYLAAAAKISPC-SLPGA-R-IGG--FGNQVILPLAHTI-E--HE----E--LLEVI-K-
XP_021765330.1/1-284 AYLAAAAKISPC-SLPGA-R-IGG--FGNQVILPLAHTI-E--HE----E--LLEVI-K-
XP_021749885.1/1-284 AYLAAAAKISPC-SLPGS-R-IGG--FGNQVILPLAHTI-E--HE----E--LLEVI-K-
XP_010267183.1/1-285 AYSAASLKAGPC-SLPGS-R-LGS--FGGQVILPLAHTI-E--HE----E--FLEVI-K-
XP_017258627.1/1-285 AYSAAAVKVGPC-SLPGS-R-VGN--YGGQVILPLAHTI-E--HE----E--LLEVI-K-
XP_023000315.1/1-284 AFSAAAVKVGPW-SLPGS-R-VGS--FANQVILPLAHTI-E--HE----E--LLEVI-K-
XP_023000313.1/1-284 AFSAAAVKVGPW-SLPGS-R-VGS--FANQVILPLAHTI-E--HE----E--LLEVI-K-
XP_023514554.1/1-284 AYSAAAVKVGPW-SLPGS-R-VGS--FANQVILPLAHTI-E--HE----E--LLEVI-K-
XP_023514552.1/1-284 AYSAAAVKVGPW-SLPGS-R-VGS--FANQVILPLAHTI-E--HE----E--LLEVI-K-
XP_022964458.1/1-284 AYSAAAVKVGPW-SLPGS-R-VGS--FANQVILPLAHTI-E--HE----E--LLEVI-K-
XP_022964454.1/1-284 AYSAAAVKVGPW-SLPGS-R-VGS--FANQVILPLAHTI-E--HE----E--LLEVI-K-
XP_022147627.1/1-284 AYSAAAVKVGPW-SLPGS-R-VGS--FGNQVILPLAHTI-E--HE----E--FLEVI-K-
XP_022147619.1/1-284 AYSAAAVKVGPW-SLPGS-R-VGS--FGNQVILPLAHTI-E--HE----E--FLEVI-K-
XP_008462437.1/1-284 AYSAAAVKVGPW-SLPGS-R-VGS--FGNQVILPLAHTI-E--HE----E--FLEVI-K-
XP_004141659.1/1-284 AYSAAAVKVGPW-SLPGS-R-VGS--FGNQVILPLAHTI-E--HE----E--FLEVI-K-
XP_008462436.1/1-284 AYSAAAVKVGPW-SLPGS-R-VGS--FGNQVILPLAHTI-E--HE----E--FLEVI-K-
XP_011659682.1/1-284 AYSAAAVKVGPW-SLPGS-R-VGS--FGNQVILPLAHTI-E--HE----E--FLEVI-K-
XP_022992111.1/1-284 AYSAAAVKVGPW-SLPGS-R-VGS--FGNQVILPLAHTI-E--HE----E--FLEVI-K-
XP_023548534.1/1-284 AYSAAAVKVGPW-SLPGS-R-VGS--FGNQVILPLAHTI-E--HE----E--FLEVI-K-
XP_022953371.1/1-284 AYSAAAVKVGPW-SLPGS-R-VGS--FGNQVILPLAHTI-E--HE----E--FLEVI-K-
XP_002304217.1/1-285 AYAAAAVKVGPF-SLQGS-R-VGS--FGGQVILPLAHTI-E--HE----E--FLEVI-K-
XP_011013041.1/1-285 AYAAAAVKVGPF-SLQGS-R-VGS--FGGQVILPLAHTI-E--HE----E--FLEVI-K-
XP_011013042.1/1-285 AYAAAAVKVGPF-SLQGS-R-VGS--FGGQVILPLAHTI-E--HE----E--FLEVI-K-
XP_011046759.1/1-284 AYAAAAVKVGPC-SLQGS-R-IGN--FGGQVILPLAHTI-E--HE----E--FLEVI-K-
XP_024464085.1/1-284 AYAAAAVKVGPC-SLQGS-R-VGN--FGGQVILPLAHTV-E--HE----E--FLEVI-K-
XP_024183498.1/1-286 AYSAAAVKVGPC-SLAGS-R-VGS--FGGQVILPLAHTI-E--HE----E--FLEVI-K-
XP_004302866.1/1-286 AYSAAAVKVGPC-SLAGS-R-VGS--FGGQVILPLAHTI-E--HE----E--FLEVI-K-
NP_001280898.1/1-285 AYSAAAVKVGPC-SLAGS-R-VGS--FGGQVILPLAHTL-E--HE----E--FLEVI-K-
XP_007214960.1/1-285 AYSAAAVKVGPC-SLAGS-R-VGS--FGGQVILPLAHTI-E--HE----E--FLEVI-K-
XP_008228437.1/1-285 AYSAAAVKVGPC-SLAGS-R-VGS--FGGQVILPLAHTI-E--HE----E--FLEVI-K-
XP_021818763.1/1-285 AYSAAAVKVGPC-SLAGS-R-VGS--FGGQVILPLAHTI-E--HE----E--FLEVI-K-
XP_022723515.1/1-285 AYSAAAVKVGPC-NSQGS-R-VGG--FSGQVILPLAHTI-E--HE----E--FLEVI-K-
XP_016688508.1/1-285 AYSAAVVKVGPC-SLRGS-R-VGG--FGSQVILPLAHTV-E--HE----E--FLEVI-K-
XP_012443274.1/1-285 AYSAAVVKVGPC-SLRGS-R-VGG--FGSQVILPLAHTV-E--HE----E--FLEVI-K-
XP_022721457.1/1-285 AYSAAAIKVGPC-SLPGS-R-VGG--FAGQVILPLAHTV-E--HE----E--FLEVI-K-
XP_022715018.1/1-285 AYSAAAVKVGPC-SLPGS-R-VGG--FGGQVILPLAHTI-E--HE----E--FLEVI-K-
XP_017979082.1/1-285 AYSAAAVKVGPC-SLPGS-R-VGG--FGGQVILPLAHTI-E--HE----E--FLEVI-K-
XP_021293768.1/1-285 AYSAAAVKVGPC-SLPGS-R-VGG--FGGQVILPLAHTI-E--HE----E--FLEVI-K-
XP_010062518.1/1-285 AYSAAAVKVGSC-ALPGS-R-IGS--FGGQVILPLAHTI-E--HE----E--FLEVI-K-
XP_006426893.1/1-285 VYSAAAIKVGPC-SLPGS-R-VGT--FGSQVILPLAHTI-E--HE----E--FMEVI-K-
XP_021610318.1/1-285 AYSAAAIKVGPC-SLLGS-R-VGS--YGGQVILPLAHTI-E--HE----E--FLEVI-K-
XP_010098130.1/1-285 AYSAAAVKVGPC-SLAGS-R-VGS--FGGQVILPLAQTI-E--HD----E--FLEVI-K-
XP_021635282.1/1-296 AYSAASIKVSPC-SLPGS-R-VGS--FGGQVILPLAHTI-E--HE----E--FLEVI-K-
XP_002284003.2/1-285 AYSAAAVKVGPC-SLPGS-R-VGS--FGSQVILPLAHTI-E--HE----E--FLEVI-K-
XP_002515977.1/1-285 AYSAAAIKVGPC-TLPGT-R-IGS--FGGQVILPLAHTI-E--HE----E--FLEVI-K-
XP_021643260.1/1-285 AYSAAAIKVGPC-SLPGS-R-VGS--FGGQVILPLAHTI-E--HE----E--FLEVI-K-
XP_021628194.1/1-285 AYSAAAIKVGPS-GLPGS-R-VGS--FGGQVILPLAHTI-E--HE----E--FLEVI-K-
XP_015894591.1/1-285 AYSAAAIKVGPC-SLAGS-R-VGS--FGGQVILPLAHTI-E--HE----E--FLEVI-K-
XP_012069098.1/1-285 AYSAAAIKIGPC-SLPGA-R-VGS--FGGQVILPLAHTI-E--HE----E--FLEVI-K-
XP_018847857.1/1-285 AYSAAAVKIGPC-SLPGS-R-VGS--FGGQVILPLAHTM-E--HE----E--FLEVI-K-
XP_023898511.1/1-285 AYSAAAVKIGPC-SLPGS-R-VGS--FGGQVILPLAHTM-E--HE----E--FLEVI-K-
XP_010545673.1/1-285 AYSAASIKLGPC-SLPGA-W-VGG--CGGQVILPLAHTI-E--HE----E--FLEVI-K-
XP_010545670.1/1-286 AYSAASIKLGPC-SLPGA-W-VGG--CGGQVILPLAHTI-E--HE----E--FLEVI-K-
XP_010535179.1/1-285 AYAAAAIKVGPC-SFSGA-R-VGG--FGGQVILPLAHTV-E--HE----E--FLEVV-K-
XP_018436929.1/1-283 AYLAAAVKVGPC-S---A-R-VGG--FGGQVILPLAHTI-E--HE----E--SMEVI-K-
XP_013592012.1/1-283 AYLAAAVKVGPC-S---A-R-VGG--FGGQVILPLAHTI-E--HE----E--SMEVI-K-
XP_013640375.1/1-284 AYLAAAVKVGPC-S---A-R-VGG--FGGQVILPLAHTI-E--HE----E--SMEVI-K-
XP_013640376.1/1-283 AYLAAAVKVGPC-S---A-R-VGG--FGGQVILPLAHTI-E--HE----E--SMEVI-K-
XP_009147709.1/1-283 AYLAAAVKVGPC-S---A-R-VGG--FGGQVILPLAHTI-E--HE----E--SMEVI-K-
XP_006306297.1/1-281 AYLAAAVKVGPC-S---A-R-VGG--FGGQVILPLAHTI-E--HE----E--FMEVI-K-
XP_010462051.1/1-281 AYLAAAVKVGPC-S---A-R-VGG--FGGQVILPLAHTI-E--HE----E--FMEVI-K-
XP_010479714.1/1-281 AYLAAAVKVGPC-S---A-R-VGG--FGGQVILPLAHTI-E--HE----E--FMEVI-K-
XP_010462052.1/1-281 AYLAAAVKVGPC-S---A-R-VGG--FGGQVILPLAHTI-E--HE----E--FMEVI-K-
XP_010500807.1/1-281 AYLAAAVKVGPC-S---A-R-VGG--FGGQVILPLAHTI-E--HE----E--FMEVI-K-
NP_849795.1/1-282    AYLAAAVKVGPC-S---A-R-VGG--FGGQVILPLAHTI-E--HE----E--FMEVI-K-
NP_001077702.1/1-281 AYLAAAVKVGPC-S---A-R-VGG--FGGQVILPLAHTI-E--HE----E--FMEVI-K-
NP_175627.1/1-281    AYLAAAVKVGPC-S---A-R-VGG--FGGQVILPLAHTI-E--HE----E--FMEVI-K-
XP_020868586.1/1-281 AYLAAAVKVGPC-S---A-R-VGG--FGGQVILPLAHTI-E--HE----E--FMEVI-K-
XP_006392953.1/1-282 AYLAAAVKVGPC-S---A-R-VGG--FGGQVILPLAHTI-E--HE----E--FMEVI-K-
XP_018460807.1/1-282 AYLAAAVKVGPC-S---A-R-VGG--FGGQVILPLAHTV-E--HE----E--FMEVI-K-
XP_013621760.1/1-283 AYLAAAVKVGPC-S---A-R-VGG--FGGQVILPLAHTI-E--HE----E--FMEVI-K-
XP_018451524.1/1-282 AYLAAAVKVGPC-S---A-R-VGG--FGGQVILPLAHTI-E--HE----E--FMEVI-K-
XP_009107148.1/1-282 AYLAAAVKVGPC-S---A-R-VGG--FGGQVILPLAHTI-E--HE----E--FMEVI-K-
XP_009107147.1/1-283 AYLAAAVKVGPC-S---A-R-VGG--FGGQVILPLAHTI-E--HE----E--FMEVI-K-
XP_009600774.1/1-284 AYAAAAVKVGPC-SLPGA-R-VCN--FGGQVILPLAHTA-E--HE----E--LLEVI-K-
XP_016459079.1/1-284 AYAAAAVKVGPC-SLPGA-R-VCN--FGGQVILPLAHTA-E--HE----E--LLEVI-K-
XP_019236029.1/1-285 AYAAAAVKVGPC-SLPGA-R-VCN--FGGQVILPLAHTA-E--HE----E--LLEVI-K-
XP_009771098.1/1-285 AYAAAAVKVGPC-SLPGA-R-VCN--FGGQVILPLAHTA-E--HE----E--LLEVI-K-
XP_016550962.1/1-285 AYSAAAVKVGPC-SLPGA-R-VAN--FGGQVILPLAHTV-E--HE----E--LLEVI-K-
XP_016550960.1/1-285 AYSAAAVKVGPC-SLPGA-R-VAN--FGGQVILPLAHTV-E--HE----E--LLEVI-K-
XP_016550961.1/1-285 AYSAAAVKVGPC-SLPGA-R-VAN--FGGQVILPLAHTV-E--HE----E--LLEVI-K-
XP_006359469.1/1-285 AYSAAAVKVGPC-SLPGV-R-VSN--FGGQVILPLAHTV-E--HE----E--LLEVI-K-
XP_004252354.1/1-285 AYSAAAVKVGPC-SLPGV-R-VSN--FGGQVILPLAHTV-E--HE----E--LLEVI-K-
XP_015061298.1/1-285 AYSAAAVKVGPC-SLPGV-R-VSN--FGGQVILPLAHTV-E--HE----E--LLEVI-K-
XP_019198775.1/1-285 AYSAAAIKVGPC-ILPEA-R-IFN--FGNQVILPLAHAV-E--HE----E--LLEVI-K-
XP_019194412.1/1-285 AYSAAAVKVGPC-SLPGA-R-ICN--FGGQVTLPLAHTI-E--HE----E--LLEVI-K-
XP_016562656.1/1-283 AYAAAAIKVGPC-SLPGA-R-VGN--FGGQVILPLAHTV-E--HE----E--LLEVI-K-
XP_019267274.1/1-285 AYSAAAIKVGPC-CLPGA-R-VGN--FGGQVILPLAHTV-E--HE----E--LLEVI-K-
XP_019267275.1/1-282 AYSAAAIKVGPC-CLPGA-R-VGN--FGGQVILPLAHTV-E--HE----E--LLEVI-K-
XP_022889649.1/1-285 AYSAAAIKVGPC-NFIGS-R-VGN--FGGQVILPLAHTI-E--HE----E--FLEVI-K-
XP_022889648.1/1-285 AYSAAAIKVGPC-NFIGS-R-VGN--FGGQVILPLAHTI-E--HE----E--FLEVI-K-
XP_012852689.1/1-281 TYSAAAVKIGPS-NFLGT-R-VAN--FGGQVVLPLAQTI-E--HE----E--LLEVI-K-
XP_012856468.1/1-279 AYSAAAIKIG-------P-R-VGN--FGGQVILPLAQTF-E--HE----E--LLEVI-K-
XP_011080516.1/1-285 AYSAAAVKVGPN-TLLGA-R-VGN--FGGQVILPLAQTI-E--QE----E--LLEVI-K-
XP_011072422.1/1-285 AYSAAAVKFGPC-NFLGP-R-VGN--FGGQVILPLAQTV-E--HE----E--LLEVI-K-
XP_022851148.1/1-285 AYSAAAVKVGPC-TFLGS-Q-VGS--FGGQVILPLAQTI-E--HG----E--LLEVI-K-
XP_022844668.1/1-285 AYSATAVKVGPC-TFLGS-Q-IGS--FGGQVILPLAQTI-E--HG----E--LLEVI-K-
XP_006853643.2/1-288 AYSAAALKSNPG-TLPTS-R-MGG--FGGQVILPLAHTV-E--HE----E--FLEVI-K-
XP_010249545.1/1-285 AYSAAALKVGSC-SMPVF-R-VAS--FGGQVILPLAHTI-E--HE----E--FLEII-K-
XP_010249539.1/1-285 AYSAAALKVGSC-SMPVF-R-VAS--FGGQVILPLAHTI-E--HE----E--FLEII-K-
XP_010915409.1/1-284 AYSAAAVRASPC-TLPMS-R-IGC--FGGQVILPLAHTL-E--HE----E--FLEVL-K-
XP_008791633.1/1-284 AYSAAAVRASPC-TLPMS-R-IGC--VGGQVILPLAHTL-E--HE----E--FLEVL-K-
XP_017235966.1/1-285 AYSAAAVKIGPC-SFPGS-R-MGN--TGSQVTLPLAHTI-E--HE----E--LLEVV-K-
XP_023761873.1/1-288 AYAASAVKLGPC-SLPGSTR-LGS--FGGQVILPLAHTI-E--HE----E--LLEVI-K-
XP_021997125.1/1-286 AYSAAAVKLGPC-NLLGT-R-AGN--FGGQVILPLAQTI-E--HE----E--LLEVI-K-
XP_021990052.1/1-285 AYLAAAIKLGPC-SIPGA-R-AGN--FGGQVILPLAHTI-E--HE----E--LLEVI-K-
XP_024979339.1/1-285 AYSAAAIKLGPC-SLPGS-R-AGN--FGGQIILPLAHTI-E--HE----E--LLEVI-K-
XP_024979341.1/1-290 AYSAAAIKLGPC-SLPGS-R-AGN--FGGQIILPLAHTI-E--HE----E--LLEVI-K-
XP_024979340.1/1-291 AYSAAAIKLGPC-SLPGS-R-AGN--FGGQIILPLAHTI-E--HE----E--LLEVI-K-
XP_024979342.1/1-291 AYSAAAIKLGPC-SLPGS-R-AGN--FGGQIILPLAHTI-E--HE----E--LLEVI-K-
XP_022016208.1/1-285 AYAAAAVKLGPC-GVPGS-R-IRD--YGSQVVLPLAQTI-E--HE----E--LLEVI-K-
XP_022016207.1/1-311 AYAAAAVKLGPC-GVPGS-R-IRD--YGSQVVLPLAQTI-E--HE----E--LLEVI-K-
XP_022009819.1/1-285 AYTAAAVKLGPC-SLPGG-Q-IGN--FGGQVILPLAHTI-E--HE----E--LLEVI-K-
XP_022009815.1/1-285 AYTAAAVKLGPC-SLPGG-Q-IGN--FGGQVILPLAHTI-E--HE----E--LLEVI-K-
XP_022009809.1/1-285 AYTAAAVKLGPC-SLPGG-Q-IGN--FGGQVILPLAHTI-E--HE----E--LLEVI-K-
XP_023759500.1/1-286 AYSAAAIKLGPC-SVPGG-R-IGN--YGGQVILPLAHTI-E--HE----E--LLEVI-K-
XP_023751740.1/1-285 AYSAASVKLGPC-SFAGS-R-IGN--FGGQVILPLAHTI-E--HE----E--VLEVI-K-
XP_024971803.1/1-285 AYSAAAVKLGPC-SLPGS-R-IGN--FGGQVILPLAHTI-E--HE----E--LLEVI-K-
XP_022033649.1/1-285 AYSAAAVKLGPC-SLPGA-R-IGS--FGGQVILPLAHMV-E--HE----E--LLEVI-K-
XP_024972195.1/1-285 AYSAAAVKLGPC-SLPGS-R-VGN--FGGQVILPLAHTI-E--HE----E--LLEVI-K-
XP_024972196.1/1-289 AYSAAAVKLGPC-SLPGS-R-VGN--FGGQVILPLAHTI-E--HE----E--LLEVI-K-
XP_020248539.1/1-284 AYSAAAVKTAPC-TLPGSHI--AG--FGGQVILPLAHTL-E--HE----E--FLEVL-K-
XP_020269197.1/1-284 AYSAVAVKATPC-TLPASQF--AG--FGGQVILPLAHTL-E--NE----E--FLEVL-K-
XP_020269196.1/1-284 AYSAVAVKATPC-TLPASQF--AG--FGGQVILPLAHTL-E--NE----E--FLEVL-K-
XP_009399813.1/1-284 GYSAASVKAIPH-TLPISRT--GC--SGGQVILPLAHTL-E--HE----E--FLEVI-K-
XP_009399812.1/1-284 GYSAASVKAIPH-TLPISRT--GC--SGGQVILPLAHTL-E--HE----E--FLEVI-K-
XP_009413853.1/1-284 AYSAAAVKATPW-ALPVSRA--GC--YGGQVILPLAHTL-D--HE----D--FLEVI-K-
XP_024985513.1/1-286 GYLAASVKAGPC-GLPLARS--GN--FGGQVILPLAHTI-E--HE----E--FMEVI-K-
XP_023729871.1/1-286 GYSAASVKSGPC-GLPMARN--GN--FGGQVILPLAHTI-E--HE----E--FMEVI-K-
XP_022000974.1/1-282 GYSAASVKGGPC-GLPLARS--GG--FGGQVILPLAHTI-E--HE----E--FMEVI-K-
XP_022023495.1/1-282 GYSAASVKGGSC-GIPLARS--GG--FGGQVILPLAHTI-E--HE----E--FMEVI-K-
XP_010526102.1/1-286 VYSAAAIRAGPC-SIPIPRP--GS--FGGQVILPLAQTI-E--NE----E--FMEVI-K-
XP_019086035.1/1-282 AYSAAAIKAGPC-SLPIPRP--GS--FGGQVILPLAHTI-E--NE----E--FMEVI-K-
XP_010437837.1/1-288 AYSAAAIKAGPC-SLPIPRP--GS--FGGQVILPLAHTI-E--NE----E--FMEVI-K-
XP_010437838.1/1-288 AYSAAAIKAGPC-SLPIPRP--GS--FGGQVILPLAHTI-E--NE----E--FMEVI-K-
XP_022549566.1/1-283 AYSASAIKAGPC-SLPIPRP--GS--FGGQVILPLAHTI-E--NE----E--FMEVI-K-
XP_013670651.1/1-283 AYSASAIKAGPC-SLPIPRP--GS--FGGQVILPLAHTI-E--NE----E--FMEVI-K-
XP_020874560.1/1-283 AYSAAAIKAGPC-SLPIPRP--GS--FGGQVILPLAHTI-E--NE----E--FMEVI-K-
XP_020874561.1/1-283 AYSAAAIKAGPC-SLPIPRP--GS--FGGQVILPLAHTI-E--NE----E--FMEVI-K-
XP_020874562.1/1-283 AYSAAAIKAGPC-SLPIPRP--GS--FGGQVILPLAHTI-E--NE----E--FMEVI-K-
NP_195014.1/1-283    AYSAAAIKAGPC-SLPIPRP--GS--FGGQVILPLAHTI-E--NE----E--FMEVI-K-
XP_006412419.1/1-283 AYSAAAIKAGPC-SVPIPRP--GS--FGGQVILPLAHTI-E--NE----E--FMEVI-K-
XP_006412418.1/1-283 AYSAAAIKAGPC-SVPIPRP--GS--FGGQVILPLAHTI-E--NE----E--FMEVI-K-
XP_018471819.1/1-283 AYSAAAIKAGPC-SLPIPRP--GS--FGGQVILPLAHTI-E--NE----E--FMEVI-K-
XP_009125299.1/1-283 AYSAAAIKAGPC-SLPIPRP--GS--FGGQVILPLAHTI-E--NE----E--FMEVI-K-
XP_010432643.1/1-283 AYSAAAIKAGPC-SLPIPRP--GS--FGGQVILPLAHTI-E--NE----E--FMEVI-K-
XP_010432644.1/1-283 AYSAAAIKAGPC-SLPIPRP--GS--FGGQVILPLAHTI-E--NE----E--FMEVI-K-
XP_006283109.1/1-283 AYSAAAIKAGPC-SLPIPRP--GS--FGGQVILPLAHTI-E--NE----E--FMEVI-K-
XP_010437839.1/1-283 AYSAAAIKAGPC-SLPIPRP--GS--FGGQVILPLAHTI-E--NE----E--FMEVI-K-
XP_010437840.1/1-283 AYSAAAIKAGPC-SLPIPRP--GS--FGGQVILPLAHTI-E--NE----E--FMEVI-K-
XP_010447325.1/1-283 AYSAAAIKAGPC-SLPIPRP--GS--FGGQVILPLAHTI-E--NE----E--FMEVI-K-
XP_010447326.1/1-283 AYSAAAIKAGPC-SLPIPRP--GS--FGGQVILPLAHTI-E--NE----E--FMEVI-K-
XP_013622315.1/1-283 AYSAAAIKAGPC-SLPIPRP--GS--FGGQVILPLAHTI-E--NE----E--FMEVI-K-
XP_013656273.1/1-283 AYSAAAIKAGPC-SLPVPRP--GS--FGGQVILPLAHTI-E--NE----E--FMEVI-K-
XP_009108824.1/1-283 AYSAAAIKAGPC-SLPIHRP--GS--FGGQVILPLAHTI-E--NE----E--FMEVI-K-
XP_013730326.1/1-283 AYSAAAIKAGPC-SLPIPRP--GS--FGGQVILPLAHTI-E--NE----E--FMEVI-K-
XP_013730325.1/1-283 AYSAAAIKAGPC-SLPIPRP--GS--FGGQVILPLAHTI-E--NE----E--FMEVI-K-
XP_018447492.1/1-283 AYSAAAIKAGPC-SLPIPRP--GS--FGGQVILPLAHTI-E--NE----E--FMEVI-K-
XP_019415259.1/1-283 AYSAAAIKAGPC-TLPGPQA--GG--FGGQVILPLSHTI-E--HE----E--FMEVI-K-
XP_022980940.1/1-286 AYSAAAIKTGQC-GLPGSHA--GT--FGGQVTLPLAQTV-E--HE----E--FMEVL-K-
XP_023523539.1/1-286 AYSAAAIKTGQC-GLPGSHA--GT--FGGQVTLPLAQTV-E--HE----E--FMEVL-K-
XP_022941184.1/1-286 AYSAAAIKTGQC-GLPGSHA--GT--FGGQVTLPLAQTV-E--HE----E--FMEVL-K-
XP_022134366.1/1-286 AYSAAAIKTGQC-GLPGSHA--GT--FGGQVILPLAQTV-E--HE----E--FMEVI-K-
XP_004136224.1/1-286 AYSAAAIKTGQC-GLPGSHA--GT--FGGQVILPLAQTV-E--HE----E--FMEVV-K-
XP_008466070.1/1-286 AYSAAAIRTGQC-GLPGSHA--GT--FGGQVILPLAQTV-E--HE----E--FMEVV-K-
XP_022976315.1/1-286 AYSAAAIKTGQC-GLPGSHA--GT--FGGQIILPLAQTV-E--HE----E--FMEVV-K-
XP_022937345.1/1-286 AYSAAAIKTGQC-GLPGSHA--GT--FGGQIILPLAQTV-E--HE----E--FMEVV-K-
XP_023534956.1/1-286 AYSAAAIKTGQC-GLPGSHA--GT--FGGQIILPLAQTV-E--HE----E--FMEVV-K-
XP_023535295.1/1-286 AYSAAAIKTGQC-GLPGSHA--GT--FGGQIILPLAQTV-E--HE----E--FMEVV-K-
XP_019175908.1/1-285 AYSAAAVKAGPC-SLPVAR--TGN--FGGQVILPLAQTI-E--HE----E--FMEVV-R-
XP_019175907.1/1-285 AYSAAAVKAGPC-SLPVAR--TGN--FGGQVILPLAQTI-E--HE----E--FMEVV-R-
XP_009626659.1/1-285 AYSAAAVKAGPC-SIPVTR--TGS--FGGQIILPLSHTI-E--HE----E--FMEVI-R-
XP_019266312.1/1-285 AYSAAAVKAGPC-SIPVTR--TGS--FGGQIILPLAHTI-E--HE----E--FMEVI-R-
XP_009781184.1/1-285 AYSAAAVKAGPC-SIPVTR--TGC--FGGQIILPLAHTI-E--HE----E--FMEVI-R-
XP_016488110.1/1-285 AYSAAAVKAGPC-SIPVTR--TGC--FGGQIILPLAHTI-E--HE----E--FMEVI-R-
XP_016537761.1/1-285 AYSAAAVKAGPC-SIPVTR--TGS--FGGQIILPLAHTI-E--HE----E--FMEVI-R-
XP_006359741.1/1-285 AYSAAAVKAGPC-SIPVTR--TGS--FGGQIILPLAHTI-E--HE----E--FMEVI-R-
XP_010325078.1/1-288 AYSAAAVKAGPC-SIPVTR--TGS--FGGQIILPLAHTI-E--HE----E--FMEVI-R-
XP_004245155.1/1-285 AYSAAAVKAGPC-SIPVTR--TGS--FGGQIILPLAHTI-E--HE----E--FMEVI-R-
XP_015085523.1/1-285 AYSAAAVKAGPC-SIPVTR--IGS--FGGQIILPLAHTI-E--HE----E--FMEVI-R-
XP_022868182.1/1-285 AYLSAAFTAGPC-SLPVAR--AGY--FGGQAILPLAHTI-E--HE----E--FMEVV-R-
XP_022868181.1/1-285 AYLSAAFTAGPC-SLPVAR--AGY--FGGQAILPLAHTI-E--HE----E--FMEVV-R-
XP_022854559.1/1-285 AYLSAAFTAGPC-SLPVAR--AGY--FGGQAILPLAHTI-E--QE----E--FMEVV-R-
XP_011081776.1/1-285 AYSAAAVKAGPC-SLPVSR--VGC--FGGQVILPLAHTI-E--NE----E--FMEVI-R-
XP_012857948.1/1-282 AYAAAAVKAGPS-SLPVSR--GGC--FGGQVILPLSQTI-E--NE----E--FMEVI-R-
XP_021847759.1/1-284 AYSAAAVKAAPC-TIPVAR--TGC--VGGQVILPLAHTI-E--NE----E--FMEVV-R-
XP_010668539.1/1-284 AYAAAAVKAAPC-TIPVSR--TGC--VGGQVILPLAHTI-E--NE----E--FMEVV-R-
XP_021739137.1/1-284 AYAAAAVKAAPC-TIPVAR--TGC--VGGQVILPLAHTI-E--NE----E--FMEVV-R-
XP_021737291.1/1-284 AYAAAAVKAAPC-TIPVAR--TGC--VGGQVILPLAHTI-E--NE----E--FMEVV-R-
XP_010047286.1/1-285 AYSAAAIKASPC-NMPGTR--IGG--FGSQVILPLAHTI-E--HE----E--FMEVV-K-
XP_003603630.2/1-285 AYSAAAIKAGPC-SLPGAR--TGA--FGGQVILPLAHTI-E--HE----E--FMEVI-K-
XP_004501146.1/1-286 AYSAAAIKAGPC-TLPGAR--AGG--FGGQVILPLAQTI-E--HE----E--FMEVI-K-
XP_012571693.1/1-286 AYSAAAIKAGPC-TLPGAR--AGG--FGGQVILPLAQTI-E--HE----E--FMEVI-K-
XP_012571692.1/1-286 AYSAAAIKAGPC-TLPGAR--AGG--FGGQVILPLAQTI-E--HE----E--FMEVI-K-
XP_019417530.1/1-285 AYSAAAIKAGPC-SLPVPQ--EGV--FGAQVILPLAHTI-E--HE----E--FMEVI-K-
XP_019417531.1/1-285 AYSAAAIKAGPC-SLPVPQ--EGV--FGAQVILPLAHTI-E--HE----E--FMEVI-K-
XP_016180268.1/1-286 AYSAAAIKAGPC-NLPGVRA--GG--FGGQVILPLAHTI-E--HE----E--FMEVI-K-
XP_025623723.1/1-286 AYSAAAIKAGPC-NLPGVRA--GG--FGGQVILPLAHTI-E--HE----E--FMEVI-K-
XP_015945672.1/1-286 AYSAAAIKAGPC-NLPGVRA--GG--FGGQVILPLAHTI-E--HE----E--FMEVI-K-
XP_007137231.1/1-287 AYSAAAIKAGPC-SLPGARP-GGG--FGGQVILPLAHTI-E--HE----E--FMEVI-K-
XP_022636494.1/1-293 AYSAAAIKAGPC-SLPGARP-GGG--FGGQVILPLAHTI-E--HE----E--FMEVI-K-
XP_017409217.1/1-287 AYSAAAIKAGPC-SLPGARP-GGG--FGGQVILPLAHTI-E--HE----E--FMEVI-K-
XP_014501941.1/1-287 AYSAAAIKAGPC-SLPGARP-GGG--FGGQVILPLAHTI-E--HE----E--FMEVI-K-
XP_003526496.1/1-286 AYSAAAIKAGPC-SLPGARS--GG--FGGQVILPLAHTI-E--HE----E--FMEVI-K-
XP_003522716.1/1-286 AYSAAAIKAGPC-SLPGARP--GG--FGGQVILPLAHTI-E--HE----E--FMEVI-K-
XP_020217689.1/1-286 AYSAAAIKAGPC-SLPGARP--GG--FGGQVILPLAHTI-E--HE----E--FMEVI-K-
XP_016683069.1/1-283 AYSAAAVKAGPC-SLPVPQ--GGS--FGGQVILPLAHTI-E--HE----E--FMEVV-K-
XP_016677926.1/1-283 AYSAAAVKAGPC-SLPVPR--GGS--FGGQVILPLAHTI-E--HE----E--FMEVV-K-
XP_017643110.1/1-283 AYSAAAVKAGPC-SLPVPR--GGS--FGGQVILPLAHTI-E--HE----E--FMEVV-K-
XP_017643111.1/1-283 AYSAAAVKAGPC-SLPVPR--GGS--FGGQVILPLAHTI-E--HE----E--FMEVV-K-
XP_012451775.1/1-283 AYSAAAVKAGPC-SLPVPQ--GGS--FGGQVILPLAHTI-E--HE----E--FMEVV-K-
XP_016744766.1/1-285 AYSAAAIKAGPC-SLPMSR--GGS--FGGQVILPLAHTI-E--HE----E--FMEVI-R-
XP_017643921.1/1-285 AYSAAAIKAGPC-SLPMSR--GGS--FGGQVILPLAHTI-E--HE----E--FMEVI-K-
XP_012436119.1/1-285 AYSAAAIKAGPC-SLPMSR--GGG--FGGQVILPLAHTI-E--HE----E--FMEVI-K-
XP_016681632.1/1-285 AYSAAAIKAGPC-SLPMSR--GGS--FGGQVILPLAHTI-E--HE----E--FMEVI-K-
XP_016689618.1/1-285 AYSAAAVKAGRC-GLPVAR--GGR--FGGQVILPLAHTI-E--HE----E--FMEVI-K-
XP_016689617.1/1-285 AYSAAAVKAGRC-GLPVAR--GGR--FGGQVILPLAHTI-E--HE----E--FMEVI-K-
XP_012445947.1/1-285 AYSAAAVKAGPC-GLPVAR--GGS--FGGQVILPLAHTI-E--HE----E--FMEVI-K-
XP_012445944.1/1-285 AYSAAAVKAGPC-GLPVAR--GGS--FGGQVILPLAHTI-E--HE----E--FMEVI-K-
XP_012445945.1/1-285 AYSAAAVKAGPC-GLPVAR--GGS--FGGQVILPLAHTI-E--HE----E--FMEVI-K-
XP_017614652.1/1-285 AYSAAAVKAGPC-GLPVAR--GGS--FGGQVILPLAHTI-E--HE----E--FMEVI-K-
XP_016732732.1/1-285 AYSAAAVKAGPC-GLPVAR--GGS--FGGQVILPLAHTI-E--HE----E--FMEVI-K-
XP_016732731.1/1-285 AYSAAAVKAGPC-GLPVAR--GGS--FGGQVILPLAHTI-E--HE----E--FMEVI-K-
XP_022754596.1/1-285 AYSAAAVKAGPC-SLPVSR--GGS--FGGQVILPLAHTI-E--HE----E--FMEVI-K-
XP_022750159.1/1-285 AYSAAAVKAGPR-NLPVSR--GGS--FGGQVILPLAHTI-E--HE----E--FMEVI-K-
XP_022729737.1/1-285 AYSAAAVKAGPC-SLPVAR--GGS--FGGQLILPLAHTI-E--HE----E--FMEVI-K-
XP_022729738.1/1-285 AYSAAAVKAGPC-SLPVAR--GGS--FGGQLILPLAHTI-E--HE----E--FMEVI-K-
XP_021276132.1/1-285 AYSAAAVKAGPC-SLPVSR--GGS--FGGQVILPLAHTI-E--HE----E--FMEVI-K-
XP_017983822.1/1-285 AYSAAAVKAGPC-SLPVSR--GGS--FGGQVILPLAHTI-E--HE----E--FMEVI-K-
XP_002283717.3/1-285 AYSAAAVKAGPC-TLPVSR--AGG--YGGQVILPLAHTI-E--HE----E--FMEVI-K-
XP_024446213.1/1-284 AYAASAIKAGPC-SLPVSR--AGN--FGGQVILPLAHTI-E--NE----E--FIEVI-K-
XP_024446215.1/1-284 AYAASAIKAGPC-SLPVSR--AGN--FGGQVILPLAHTI-E--NE----E--FIEVI-K-
XP_011006428.1/1-284 AYTASAIKAGPC-SLPVSR--AGN--FGGQVILPLAHTI-E--NE----E--FIEVI-K-
XP_024459734.1/1-284 AYAAAAVKAGPC-SLPMSR--AGN--FGGQVILPLAHTI-E--HE----E--FMEVI-K-
XP_011019595.1/1-284 AYAAAAVKAGPC-SLPMSR--AGN--FGGQVILPLAHTI-E--HE----E--FMEVI-K-
XP_015573174.1/1-284 AYAAAAVKAGPC-TLPVAR--AGN--FGGQVILPLAHTI-E--HE----E--FMEVI-K-
XP_012077376.1/1-284 AYAAAAVKAGPC-TLPVSR--AGN--FGGQVILPLAHTI-E--HE----E--FMEVI-K-
XP_021608403.1/1-284 AYAAAAVKAGPC-TLPVPR--VGN--FGGQVILPLAHTI-E--HE----E--FMEVI-K-
XP_021608402.1/1-284 AYAAAAVKAGPC-TLPVPR--VGN--FGGQVILPLAHTI-E--HE----E--FMEVI-K-
XP_021597909.1/1-284 AYAAAAVKAGPC-TLPVSR--VGN--FGGQVILPLAHTI-E--HE----E--FMEVI-K-
XP_021642839.1/1-284 AYAAAAVKAGPC-TLPVSR--AGN--FGGQVILPLAHTI-E--HE----E--FMEVI-K-
XP_021689146.1/1-284 AYAAAAVKAGPC-TLPVSR--VGN--FGGQVILPLAHTI-E--HE----E--FMEVI-K-
XP_018805752.1/1-285 AYSAAAIKAGPS-SLIGVR--TAS--FGSQVILPLAHTI-E--HE----E--FMEVI-K-
XP_023918167.1/1-285 AYSAAAIKAGPC-SLPGVR--AGS--FGGQVILPLAHTI-E--HE----E--FMEVI-K-
XP_018845499.1/1-285 AYSAAAVKAGPC-SLPGVR--AGS--FGGQVILPLAHTI-E--NE----E--FMEVI-K-
XP_018845500.1/1-285 AYSAAAVKAGPC-SLPGVR--AGS--FGGQVILPLAHTI-E--NE----E--FMEVI-K-
NP_001280750.1/1-285 AYSAAAIKAGPC-NMLGPR--AGS--FGDQVILPLAHTI-E--HE----E--FMEVI-K-
NP_001280796.1/1-285 AYSAAAVKAGPC-NMLGPR--VGS--FGDNVILPLAHTI-E--HE----E--FMEVI-K-
XP_009367440.1/1-285 AYSAAAVKAGPC-NMLGPR--VGS--FGDQVILPLAHTI-E--HE----E--FMEVI-K-
XP_017182108.1/1-285 AYSAAAVKAGPC-NMLGPR--VGS--FGDQVILPLAHTI-E--HE----E--FMEVI-K-
XP_004291167.1/1-285 AYSAAAIKAGPC-GMIGSR--AGS--FGDQVILPLAHTI-E--HE----E--FMEVI-K-
XP_024162655.1/1-285 AYSAAAIKAGPC-GMLGSR--AGS--FGDQVILPLAHTI-E--HE----E--FMEVI-K-
XP_021819449.1/1-284 AYSAAAIKPGPC-GLLGSR--AGS--FGDQVILPLAHTI-E--HE----E--FMEVI-K-
XP_007225281.1/1-285 AYSAAAIKPGPC-GLLGSR--AGG--FGDQVIHPLAHTI-E--HE----E--FMEVI-K-
XP_008220092.1/1-285 AYSAAAIKPGPC-GLLGSR--AGN--FGDQVIHPLAHTI-E--HE----E--FMEVI-K-
XP_006450706.1/1-285 AYSAAAVKAGPC-SLPVPR--AGN--FGGQVILPLAHTI-E--HE----E--FLEVI-K-
XP_006476026.1/1-285 AYSAAAVKAGPC-SLPVPR--AGN--FGGQVILPLAHTI-E--HE----E--FLEVI-K-
XP_024930942.1/1-285 AYSAAAIKAGPC-SLPVSR--AGS--FGGQVILPLAHTI-E--HE----E--FMEVI-K-
XP_010102406.2/1-285 AYSAAAIKAVPC-SLPVPR--AGS--FGGQVILPLAYTI-E--HE----E--FMEVI-K-
XP_024540686.1/1-258 FFQ-----QDA----AS-RS-HG----KRQAHVPLFH-T-A--KE----D--FLEAV-I-
XP_024399220.1/1-283 ANAATAFRGASN-GHVS-R--GRM--SHVQLPLPLAQFG-E--QG----E--FLEVV-K-
XP_024399219.1/1-283 ANAATAFRGASN-GHVS-R--GRM--SHVQLPLPLAQFG-E--QG----E--FLEVV-K-
XP_024399215.1/1-283 ANAATAFRGASN-GHVS-R--GRM--SHVQLPLPLAQFG-E--QG----E--FLEVV-K-
XP_024390746.1/1-284 ANVA-SFRSNGN-GYVP-RC-GGV--SHVQLPLPLAYSG-E--SG----E--ILEVV-K-
XP_024390752.1/1-284 ANVA-SFRSNGN-GYVP-RC-GGV--SHVQLPLPLAYSG-E--SG----E--ILEVV-K-
XP_024373354.1/1-283 D-MA-SFRSNGN-GYVP-RG-GGV--SHVQLPLPLAQSG-E--CG----E--ILEVV-K-
XP_024373357.1/1-283 D-MA-SFRSNGN-GYVP-RG-GGV--SHVQLPLPLAQSG-E--CG----E--ILEVV-K-
XP_024370817.1/1-284 ANVATSFRSNGN-SYA--RG-GGV--SHVQLPLPLAHSG-E--HG----E--ILEVV-K-
XP_024370815.1/1-284 ANVATSFRSNGN-SYA--RG-GGV--SHVQLPLPLAHSG-E--HG----E--ILEVV-K-
XP_024370814.1/1-284 ANVATSFRSNGN-SYA--RG-GGV--SHVQLPLPLAHSG-E--HG----E--ILEVV-K-
XP_024386785.1/1-285 ANLATSFRSNGN-GYAP-QG-GGV--SHVQLPLPLPNSG-E--HG----E--ILEVV-K-
XP_024386782.1/1-285 ANLATSFRSNGN-GYAP-QG-GGV--SHVQLPLPLPNSG-E--HG----E--ILEVV-K-
XP_024386791.1/1-285 ANLATSFRSNGN-GYAP-QG-GGV--SHVQLPLPLPNSG-E--HG----E--ILEVV-K-
XP_002961227.1/1-277 LDTAS-MRASAC-GFS-----RGN--VIGQCPVPLVQSA-E--EE----E--FLEVV-K-
XP_002969458.1/1-277 LDTAS-MRASAC-GFS-----RGN--VIGQCPVPLVQSA-E--EE----E--FLEVV-K-
XP_024533017.1/1-265 SDSVSTLKIGGY-GTS-----KGN--ADEECLFP---------------E--LLEVI-K-
XP_011076983.1/1-261 DHSAVFLGPACF-AFPGSRT-YNL--SGTG--VLLGHTN-Y--ED----E--ILEVL-R-
XP_011076980.1/1-264 DHSAVFLGPACF-AFPGSRT-YNL--SGTG--VLLGHTN-Y--ED----E--ILEVL-R-
XP_011076981.1/1-263 DHSAVFLGPACF-AFPGSRT-YNL--SGTG--VLLGHTN-Y--ED----E--ILEVL-R-
XP_011084330.1/1-264 DHSVAFSKAACY-AYPGPTI-HNL--SGTP--VMLGHTN-H--DD----E--ELEVI-R-
XP_011084333.1/1-264 DHSVAFSKAACY-AYPGPTI-HNL--SGTP--VMLGHTN-H--DD----E--ELEVI-R-
XP_011084331.1/1-264 DHSVAFSKAACY-AYPGPTI-HNL--SGTP--VMLGHTN-H--DD----E--ELEVI-R-
XP_012834933.1/1-260 DHSVAFTKAACF-SYPGHNT-HNL--SGAP--VLLGHNN-H--ED----E--ALEVI-R-
XP_012834932.1/1-260 DHSVAFTKAACF-SYPGHNT-HNL--SGAP--VLLGHNN-H--ED----E--ALEVI-R-
XP_012834931.1/1-260 DHSVAFTKAACF-SYPGHNT-HNL--SGAP--VLLGHNN-H--ED----E--ALEVI-R-
XP_012834934.1/1-260 DHSVAFTKAACF-SYPGHNT-HNL--SGAP--VLLGHNN-H--ED----E--ALEVI-R-
XP_002274194.1/1-284 AYAAASLRGDSF-ALPGLSP-SQF--SGNQTTMSLGITA-E---N----E--ILEII-Q-
XP_019079956.1/1-284 AYAAASLRGDSF-ALPGLSP-SQF--SGNQTTMSLGITA-E---N----E--ILEII-Q-
XP_023759583.1/1-283 AYSAASVKAGPT-SLPM-AR-NGS--FGSQVILPLAHTL-E--HE----E--FMEVV-K-
XP_020087984.1/1-275 AFSAAALRANSC-NFSV-PH-IGP--FGGQVILPLAHSL-D--NE----E--LLEVL-K-
XP_015693848.1/1-289 AFFASAMKPNFC-NLPM-NR-LGG--FSGQVILPLAHTF-E--PE----E--FLEVI-K-
XP_015624690.1/1-290 AFFASTMKPNFC-NLPM-SR-LGG--FSGQVILPLAHTF-E--PE----E--FLEVI-K-
XP_008655743.1/1-292 AFFASAMKPDFC-SLPIPPR-LGG--FGGQVILPLAHTF-D--PE----E--FLEVI-K-
XP_020183693.1/1-281 AFFASALKPNFS-NLPM-SR-LGG--FSGQVILPLAHTS-D--PE----E--FLEVI-K-
XP_020183687.1/1-281 AFFASALKPNFS-NLPM-SR-LGG--FSGQVILPLAHTS-D--PE----E--FLEVI-K-
XP_003565084.1/1-284 AFFASALKPNFC-NLPM-SR-LGG--FSGQVILPLAHTF-D--PE----E--FLEVI-K-
XP_002454995.1/1-286 AFFASAMKPNFC-NLPM-SR-LGG--FSGQVILPLAHTF-D--PE----E--FLEVI-K-
XP_012701181.1/1-286 AFFASAMKPNFC-NLPM-SR-LGG--FGGQVILPLAHTF-D--PE----E--FLEVI-K-
XP_012701180.1/1-286 AFFASAMKPNFC-NLPM-SR-LGG--FGGQVILPLAHTF-D--PE----E--FLEVI-K-
XP_012701179.1/1-286 AFFASAMKPNFC-NLPM-SR-LGG--FGGQVILPLAHTF-D--PE----E--FLEVI-K-
XP_025816436.1/1-287 AFFASAMKPNFC-NLPM-SR-LGG--FSGQVILPLAHTF-D--PE----E--FLEVI-K-
XP_018726848.1/1-227 ----------------------RG--V-V-EMVHIP-NG-RD--T--G-N--CVSLL-Q-
XP_018726849.1/1-227 ----------------------RG--V-V-EMVHIP-NG-RD--T--G-N--CVSLL-Q-
XP_019193052.1/1-216 ----------------------SG--T-VTEMAHIA-NG-RE--T--G-N--CVSLL-R-
XP_019193050.1/1-216 ----------------------SG--T-VTEMAHIA-NG-RE--T--G-N--CVSLL-R-
XP_021834047.1/1-194 ----------------------GG--I-VQEMAHIA-NG-RD--T--V-N--CVSLL-R-
XP_010522330.1/1-229 ----------------------GG--V-VQEMAHIA-NG-RE--T--G-N--CVSLL-R-
XP_009802524.1/1-219 ----------------------GS--V-IQRLVYIA-NG-RE--T--G-S--SVSIL-R-
XP_009802526.1/1-219 ----------------------GS--V-IQRLVYIA-NG-RE--T--G-S--SVSIL-R-
XP_009802525.1/1-219 ----------------------GS--V-IQRLVYIA-NG-RE--T--G-S--SVSIL-R-
XP_009802527.1/1-219 ----------------------GS--V-IQRLVYIA-NG-RE--T--G-S--SVSIL-R-
XP_019250148.1/1-219 ----------------------GG--V-TQRLVHIA-NG-RE--K--G-N--SVSIL-R-
XP_019250149.1/1-219 ----------------------GG--V-TQRLVHIA-NG-RE--K--G-N--SVSIL-R-
XP_016449923.1/1-219 ----------------------GG--V-IQRLVHIA-NG-RE--T--G-N--SVSIL-R-
XP_009629760.1/1-219 ----------------------GG--V-IQRLVHIA-NG-RE--T--G-N--SVSIL-R-
XP_009629762.1/1-219 ----------------------GG--V-IQRLVHIA-NG-RE--T--G-N--SVSIL-R-
XP_010234287.2/1-230 ----------------------GG--V-VHEMAHIA-NG-QN--H--G-N--AVSLL-R-
XP_021806177.1/1-204 ----------------------GV--I-VQEMVHIT-NG-RD--T--G-N--CVSS----
XP_016580642.1/1-229 ----------------------GG--T-IQEMAHIA-NG-RE--T--G-N--CVSLL-K-
XP_008667763.1/1-266 ----------------------GG--V-VQEMAHIA-NG-RD--H--G-N--CVSLL-R-
XP_006352040.1/1-227 ----------------------GG--L-LQEVAQIT-NG-TE--T--G-N--CVSLL-R-
XP_009611638.1/1-223 ----------------------GG--V-VQEMAHIA-NG-RE--T--G-N--CVSLL-R-
XP_009611623.1/1-223 ----------------------GG--V-VQEMAHIA-NG-RE--T--G-N--CVSLL-R-
XP_016510654.1/1-223 ----------------------GG--V-VQEMAHIA-NG-RE--T--G-N--CVSLL-R-
XP_009766038.1/1-223 ----------------------GG--I-VQEMAHIA-NG-RE--T--G-N--CVSLL-R-
XP_020210648.1/1-236 ----------------------GA--I-VEEVAHIG-NG-RD--P--G-N--SVSLL-R-
XP_007143210.1/1-219 ----------------------GG--Q-VVELAHIA-NG-HD--T--G-N--CVSLL-R-
XP_014491869.1/1-226 ----------------------GG--Q-VEELAHIA-NG-HD--S--G-N--CVSLL-R-
XP_022633652.1/1-226 ----------------------GG--Q-VEELAHIA-NG-HD--S--G-N--CVSLL-R-
XP_017412341.1/1-227 ----------------------GG--Q-VEELAHIA-NG-HD--T--G-N--CVSLL-R-
XP_022011155.1/1-226 ----------------------SV--L-VQEIVHIP-SG-RD--P--G-N--CVSLL-R-
XP_003555330.2/1-228 ----------------------GA--Q-VNELAHIA-NG-RD--H--G-N--CVSLL-R-
XP_014628518.1/1-229 ----------------------GA--Q-VNELAHIA-NG-RD--H--G-N--CVSLL-R-
XP_017647009.1/1-225 ----------------------GG--V-VDEMAHIA-NG-RD--P--G-N--CVSLL-R-
XP_016711277.1/1-225 ----------------------GG--V-VDEMAHIA-NG-RD--P--G-N--CVSLL-R-
XP_012457027.1/1-225 ----------------------GG--V-VDEMAHIA-NG-RD--P--G-N--CVSLL-R-
XP_016723454.1/1-225 ----------------------GG--V-VDEMAHIA-NG-RD--P--G-N--CVSLL-R-
XP_020978973.1/1-214 ----------------------RG--L-VQEITHIA-S------S--S-N--ALSLL-R-
XP_016202355.1/1-214 ----------------------RG--L-VQEITHIA-S------S--S-N--ALSLL-R-
XP_015964876.1/1-214 ----------------------RG--L-VQEITHIA-S------S--A-N--VLSLL-R-
XP_025699645.1/1-214 ----------------------RG--L-VQEITHIA-S------S--A-N--VLSLL-R-
XP_008441375.1/1-228 ----------------------GG--L-VQEMARIG-ND-RN--S--G-N--CVSLL-R-
XP_004154226.2/1-228 ----------------------GG--L-VQEMARIG-ND-RN--S--G-N--CVSLL-R-
XP_023519981.1/1-226 ----------------------GG--L-VQEMARIG-ND-RN--P--G-N--CVSLL-R-
XP_023001231.1/1-226 ----------------------GG--L-VQEMARIG-ND-RN--P--G-N--CVSLL-R-
XP_022933178.1/1-226 ----------------------GG--L-VQEMARIG-ND-RN--P--G-N--CVSLL-R-
XP_004511340.1/1-228 ----------------------GG--I-VQEMAHIA-NG-HD--P--G-N--CVSLL-R-
XP_002979015.2/1-232 ----------------------GG--M-VQEMAHIA-KG-QD--P--G-N--SVSLL-R-
XP_002988402.1/1-232 ----------------------GG--M-VQEMAHIA-KG-QD--P--G-N--SVSLL-R-
XP_024390340.1/1-238 ----------------------GG--M-VTEMAHIA-KG-RD--P--G-N--SVSLL-K-
XP_024368074.1/1-235 ----------------------GG--M-VTEMAHIA-KG-QD--P--G-N--SVSLL-K-
XP_024401280.1/1-235 ----------------------GG--M-VTEMAHIA-KG-QD--P--G-N--SVSLL-R-
XP_024394840.1/1-235 ----------------------GG--I-VTEMAHIA-KG-QD--P--G-N--SVSLL-R-
XP_024394838.1/1-235 ----------------------GG--I-VTEMAHIA-KG-QD--P--G-N--SVSLL-R-
XP_017256650.1/1-227 ----------------------GG--A-IQEMAHFA-NG-RE--T--G-N--CVSLL-R-
XP_019186546.1/1-227 ----------------------GR--L-VQEIAHIA-NG-RD--P--G-N--NVSLL-R-
XP_024988375.1/1-226 ----------------------CG--L-VQKIVHIA-NG-RD--P--G-N--YVSLL-R-
XP_017231769.1/1-226 ----------------------GG--L-VQEMAHTA-NG-RD--P--G-N--CVSLL-R-
XP_009108529.1/1-235 ----------------------GG--L-VEEMAHIA-NG-RD--P--G-N--SVSLL-R-
XP_018469534.1/1-236 ----------------------GG--L-VQEMAHIA-NG-RD--P--G-N--SVSLL-R-
XP_018467466.1/1-236 ----------------------GG--L-VQEMAHIA-NG-RD--P--G-N--SVSLL-R-
XP_022543253.1/1-234 ----------------------GG--L-VQKMAHIA-NG-RD--P--G-N--SISLL-R-
XP_009135181.1/1-234 ----------------------GG--L-VQKMAHIA-NG-RD--P--G-N--SISLL-R-
XP_013642975.1/1-234 ----------------------GG--L-VQKMAHIA-NG-RD--P--G-N--SISLL-R-
XP_009135189.1/1-234 ----------------------GG--L-VQKMAHIA-NG-RD--P--G-N--SISLL-R-
XP_018515487.1/1-234 ----------------------GG--L-VQKMAHIA-NG-RD--P--G-N--SISLL-R-
XP_013623861.1/1-234 ----------------------GG--L-VQEMAHIA-NG-RD--P--G-N--CVSLL-R-
XP_013623905.1/1-234 ----------------------GG--L-VQEMAHIA-NG-RD--P--G-N--CVSLL-R-
XP_013623953.1/1-236 ----------------------GG--L-VQEMAHIA-NG-RD--P--G-N--CVSLL-R-
XP_013623587.1/1-236 ----------------------GG--L-VQEMAHIA-NG-RD--P--G-N--CVSLL-R-
XP_013623995.1/1-236 ----------------------GG--L-VQEMAHIA-NG-RD--P--G-N--CVSLL-R-
XP_013623820.1/1-236 ----------------------GG--L-VQEMAHIA-NG-RD--P--G-N--CVSLL-R-
XP_022926381.1/1-227 ----------------------RG--L-VQEMANIA-NG-RH--S--G-N--CVSLL-R-
XP_023517485.1/1-227 ----------------------RG--L-VQEMANIA-NG-RH--S--G-N--CVSLL-R-
XP_023002930.1/1-227 ----------------------RG--L-VQEMANIA-NG-RH--S--G-N--CVSLL-R-
XP_004134971.1/1-229 ----------------------GG--L-VQEMAHIA-NG-RH--S--G-N--CVSLL-R-
XP_008439968.1/1-229 ----------------------GG--L-VQEMAHIA-NG-RH--S--G-N--CVSLL-R-
XP_022950176.1/1-227 ----------------------GG--L-VQEMAHIA-NG-RH--S--G-N--CVSLL-R-
XP_022979090.1/1-227 ----------------------GG--L-VQEMAHIA-NG-RH--S--G-N--CVSLL-R-
XP_023543388.1/1-249 ----------------------GG--L-VQEMAHIA-NG-RH--T--G-N--CVSLL-R-
XP_023543387.1/1-227 ----------------------GG--L-VQEMAHIA-NG-RH--T--G-N--CVSLL-R-
XP_009134323.1/1-228 ----------------------GG--M-VQEMAHIA-NG-RE--P--G-N--CVSLL-R-
XP_019176002.1/1-227 ----------------------GG--L-VQEMAHIA-NG-RD--P--G-N--CVSLL-R-
XP_015070655.1/1-221 ----------------------GG--L-VQEMAHIA-NG-RD--P--G-N--CVSLL-R-
XP_015070652.1/1-221 ----------------------GG--L-VQEMAHIA-NG-RD--P--G-N--CVSLL-R-
XP_025885589.1/1-221 ----------------------GG--L-VQEMAHIA-NG-RD--P--G-N--CVSLL-R-
XP_019422236.1/1-227 ----------------------GG--Q-VQEMAHIA-NG-HD--P--G-N--CVSLL-R-
XP_007157166.1/1-226 ----------------------GG--L-VQELAHIA-NG-RE--P--G-N--CVSLL-R-
XP_003538765.1/1-227 ----------------------GG--L-VQELAHIA-NG-RD--P--G-N--CVSLL-R-
XP_003517589.1/1-227 ----------------------GG--L-VQELAHIA-NG-RD--P--G-N--CVSLL-R-
XP_006573890.1/1-227 ----------------------GG--L-VQELAHIA-NG-RD--P--G-N--CVSLL-R-
XP_011000687.1/1-226 ----------------------GG--Q-VQEMAHIA-NG-RN--P--G-N--CVSLL-R-
XP_024436817.1/1-226 ----------------------GG--Q-VQEMAHIA-NG-RD--P--G-N--CVSLL-R-
XP_024436820.1/1-226 ----------------------GG--Q-VQEMAHIA-NG-RD--P--G-N--CVSLL-R-
XP_002518520.2/1-226 ----------------------GG--Q-VEEMAHIA-NG-RD--P--G-N--CVSLL-R-
XP_015574304.1/1-226 ----------------------GG--Q-VEEMAHIA-NG-RD--P--G-N--CVSLL-R-
XP_021642058.1/1-227 ----------------------GG--Q-VEEMAHIA-NG-RD--P--G-N--SVSLL-R-
XP_012091395.1/1-226 ----------------------GG--Q-VQEMAHIA-NG-RD--P--G-N--YVSLL-R-
XP_021635824.1/1-226 ----------------------GG--Q-IQEMAHIA-NG-RD--P--G-N--CVSLL-R-
XP_021596887.1/1-226 ----------------------GG--Q-VQEMAHIA-NG-RD--P--G-N--CVSLL-R-
XP_010057969.1/1-228 ----------------------GG--Q-VQEMAHIA-NG-RD--P--G-N--SVSLL-R-
XP_006413738.1/1-245 ----------------------GG--L-VQEMAHIA-NG-RD--P--G-N--CVSLL-R-
XP_013630219.1/1-235 ----------------------GG--L-VEEMAHIA-NG-RD--P--G-N--SVSLL-R-
XP_013656136.1/1-235 ----------------------GG--L-VKEMAHIA-NG-RD--P--G-N--SVSLL-R-
XP_013685757.1/1-235 ----------------------GG--L-VKEMAHIA-NG-RD--P--G-N--SVSLL-R-
XP_013696499.1/1-240 ----------------------GG--L-VQEMAHIA-NG-RD--P--G-N--SVSLF-R-
XP_009137213.1/1-240 ----------------------GG--L-VQEMAHIA-NG-RD--P--G-N--SVSLF-R-
XP_013598423.1/1-241 ----------------------GG--L-VQEMAHIA-NG-RD--P--G-N--SVSLF-R-
XP_013717184.1/1-241 ----------------------GG--L-VQEMAHIA-NG-RD--P--G-N--SVSLF-R-
XP_018480642.1/1-241 ----------------------GG--L-VQEMAHIA-NG-RD--P--G-N--SVSLF-R-
XP_018477464.1/1-241 ----------------------GG--L-VQEMAHIA-NG-RD--P--G-N--SVSLF-R-
NP_193906.2/1-248    ----------------------GG--L-VQEMAHIA-NG-RD--P--G-N--SVSLL-R-
XP_020873301.1/1-248 ----------------------GG--L-VQEMAHIA-NG-RD--P--G-N--SVSLL-R-
XP_006285476.1/1-249 ----------------------GG--L-VQEMAHIA-NG-RD--P--G-N--SVSLL-R-
XP_010434132.1/1-252 ----------------------GG--L-VQEMAHIA-NG-RD--P--G-N--SVSLL-R-
XP_010439429.1/1-252 ----------------------GG--L-VQEMAHIA-NG-RD--P--G-N--SVSLL-R-
XP_010449009.1/1-254 ----------------------GG--L-VQEMAHIA-NG-RD--P--G-N--SVSLL-R-
XP_010432053.1/1-234 ----------------------GG--M-VQEMAHIA-NG-HE--P--G-N--CVSLL-R-
XP_020876375.1/1-234 ----------------------GG--M-VQEMAHIA-NG-HE--P--G-N--CVSLL-R-
XP_023636874.1/1-234 ----------------------GG--M-VQEMAHIA-NG-RE--P--G-N--CVSLL-R-
XP_010422328.1/1-234 ----------------------GG--M-VQEMAHIA-NG-HE--P--G-N--CVSLL-R-
XP_010455798.1/1-234 ----------------------GG--M-VQEMAHIA-NG-HE--P--G-N--CVSLL-R-
NP_567274.1/1-235    ----------------------GG--M-VQEMAHIA-NG-HE--P--G-N--CVSLL-R-
XP_024009884.1/1-236 ----------------------GG--M-VQEMAHIA-NG-RE--P--G-N--CVSLL-R-
XP_018452346.1/1-236 ----------------------GG--M-VQEMAHIA-NG-RE--P--G-N--CVSLL-R-
XP_013611160.1/1-236 ----------------------GG--M-VQEMAHIA-NG-RE--P--G-N--CVSLL-R-
XP_009114536.1/1-235 ----------------------GG--M-VQEMAHIA-NG-RE--P--G-N--CVSLL-R-
XP_013662000.1/1-235 ----------------------GG--M-VQEMAHIA-NG-RE--P--G-N--CVSLL-R-
XP_018472664.1/1-233 ----------------------GG--M-VQEMAHIA-NG-RE--P--G-N--CVSLL-R-
XP_022555047.1/1-234 ----------------------GG--M-VQEMAHIA-NG-RE--P--G-N--CVSLL-R-
XP_013735469.1/1-234 ----------------------GG--M-VQEMAHIA-NG-RE--P--G-N--CVSLL-R-
XP_013629800.1/1-234 ----------------------GG--M-VQEMAHIA-NG-RE--P--G-N--CVSLL-R-
XP_013735464.1/1-234 ----------------------GG--M-VQEMAHIA-NG-RE--P--G-N--CVSLL-R-
XP_022571893.1/1-234 ----------------------GG--M-VQEMAHIA-NG-RE--P--G-N--CVSLL-R-
XP_010538642.1/1-236 ----------------------GG--L-IQEMAHIA-NG-RD--P--G-N--CVSLL-R-
XP_019057303.1/1-229 ----------------------GG--L-VQEMAHIA-NG-RD--P--G-N--CVSLL-R-
XP_016545426.1/1-226 ----------------------GG--L-VQEMAHIA-NG-RD--S--G-N--SVSLL-R-
XP_009793614.1/1-227 ----------------------GG--L-AQEMAHIA-NG-RN--S--G-N--SVSLL-R-
XP_016508926.1/1-227 ----------------------GG--L-AQEMAHIA-NG-RN--S--G-N--SVSLL-R-
XP_016560096.1/1-235 ----------------------GG--V-IQEMAHIA-NG-RD--P--G-N--CVSLL-R-
XP_021993721.1/1-227 ----------------------GG--L-VQEMAHIA-NG-RD--P--G-N--CVSLL-R-
XP_022036928.1/1-226 ----------------------CG--L-VQEMAHIA-NG-RD--P--G-N--CVSLL-R-
XP_023742870.1/1-226 ----------------------CG--H-VEEIAHIA-NG-RD--P--G-N--CVSLL-R-
XP_006352605.1/1-223 ----------------------GG--L-VQEMAHIA-NG-RD--S--G-N--SVSLL-R-
XP_010327237.1/1-223 ----------------------GG--L-VQEMAHIA-NG-RD--S--G-N--SVSLL-R-
XP_015055984.1/1-223 ----------------------GG--L-VQEMAHIA-NG-RD--S--G-N--SVSLL-R-
XP_009609606.1/1-229 ----------------------GG--L-VQEMAHIA-NG-RD--S--G-N--SVSLL-R-
XP_009609608.1/1-229 ----------------------GG--L-VQEMAHIA-NG-RD--S--G-N--SVSLL-R-
XP_018628742.1/1-229 ----------------------GG--L-VQEMAHIA-NG-RD--S--G-N--SVSLL-R-
XP_019237831.1/1-229 ----------------------GG--L-VQEMAHIA-NG-RD--S--G-N--SVSLL-R-
XP_018849836.1/1-227 ----------------------GG--L-VQEMAHIA-NG-RD--Q--G-N--CVSLL-R-
XP_007214541.1/1-230 ----------------------GG--L-VQEMAHIA-NG-RD--P--G-N--CVSLL-R-
XP_021821565.1/1-230 ----------------------GG--L-VQEMAHIA-NG-RD--P--G-N--CVSLL-R-
XP_008224792.1/1-230 ----------------------GG--L-VQEMAHIA-NG-RD--P--G-N--SVCLL-R-
XP_024198966.1/1-227 ----------------------GG--L-VQEMAHIA-NG-RD--P--G-N--CVSLL-R-
XP_004293268.1/1-227 ----------------------GG--L-VQEMAHIA-NG-RD--P--G-N--CVSLL-R-
XP_009364098.1/1-227 ----------------------GG--L-VQEMAHIA-NG-RD--P--G-N--CVSLL-R-
XP_008371114.1/1-227 ----------------------GG--L-VQEMAHIA-NG-RD--P--G-N--CVSLL-R-
XP_008384034.1/1-227 ----------------------GG--L-VQEMAHIA-NG-RD--P--G-N--CVSLL-R-
XP_009340946.1/1-227 ----------------------GG--L-VQEMAHIA-NG-RD--P--G-N--CVSLL-R-
XP_009357415.1/1-227 ----------------------GG--L-VQEMAHIA-NG-RD--P--G-N--CVSLL-R-
XP_011037708.1/1-226 ----------------------GG--E-VQEMAHIA-NG-RD--P--G-N--CVSLL-R-
XP_024454819.1/1-226 ----------------------GG--E-VQEMAHIA-NG-RD--P--G-N--CVSLL-R-
XP_015884145.1/1-226 ----------------------GG--L-VQEMAHIA-NG-RD--P--G-N--CVSLL-R-
XP_024024693.1/1-228 ----------------------GG--L-VQEMAHIA-NG-RD--P--G-N--CVSLL-R-
XP_024024692.1/1-228 ----------------------GG--L-VQEMAHIA-NG-RD--P--G-N--CVSLL-R-
XP_018820351.1/1-226 ----------------------GG--L-VQEMAHIA-NG-RD--P--G-N--CVSLL-R-
XP_023904570.1/1-226 ----------------------GG--L-VQEMAHIA-NG-RD--P--G-N--CVSLL-R-
XP_023904569.1/1-226 ----------------------GG--L-VQEMAHIA-NG-RD--P--G-N--CVSLL-R-
XP_006449076.1/1-226 ----------------------GG--L-VQEMAHIA-NG-RD--P--G-N--CVSLL-R-
XP_006468235.1/1-226 ----------------------GG--L-VQEMAHIA-NG-RD--P--G-N--CVSLL-R-
XP_022721034.1/1-226 ----------------------GG--L-VQEMAHIA-NG-RD--P--G-N--CVSLL-R-
XP_022721035.1/1-226 ----------------------GG--L-VQEMAHIA-NG-RD--P--G-N--CVSLL-R-
XP_022724746.1/1-226 ----------------------GG--L-VQEMAHIA-NG-RD--P--G-N--CVSLL-R-
XP_007026002.1/1-226 ----------------------GG--L-VQEMAHIA-NG-RD--P--G-N--CVSLL-R-
XP_021294392.1/1-226 ----------------------GG--L-VQEMAHIA-NG-RD--P--G-N--CVSLL-R-
XP_022777405.1/1-226 ----------------------GG--L-VQEMAHIA-NG-RD--P--G-N--CVSLL-R-
XP_017615807.1/1-226 ----------------------GG--L-VQEMAHIA-NG-RD--P--G-N--CVSLL-R-
XP_016753649.1/1-226 ----------------------GG--L-VQEMAHIA-NG-RD--P--G-N--CVSLL-R-
XP_016683607.1/1-226 ----------------------GG--L-VQEMAHIA-NG-RD--P--G-N--CVSLL-R-
XP_012451363.1/1-226 ----------------------GG--L-VQEMAHIA-NG-RD--P--G-N--CVSLL-R-
XP_002266688.1/1-226 ----------------------GG--L-VQEMAHIA-NG-RD--P--G-N--CVSLL-R-
XP_021853797.1/1-224 ----------------------GG--L-VQEMAHIA-NG-RD--P--G-N--CVSLL-R-
XP_021719656.1/1-227 ----------------------GG--L-VQEMAHIA-NG-RD--P--G-N--CVSLL-R-
XP_021766927.1/1-227 ----------------------GG--L-VQEMAHIA-NG-RD--P--G-N--CVSLL-R-
XP_010665524.1/1-227 ----------------------GG--L-VQEMAHIA-NG-RD--P--G-N--CVSLL-R-
XP_009605864.1/1-234 ----------------------GG--L-VQEMAHIA-NG-RD--P--G-N--CVSLL-R-
XP_019223778.1/1-233 ----------------------GG--L-VQEMAHIA-NG-RD--P--G-N--CVSLL-R-
XP_016440385.1/1-233 ----------------------GG--L-VQEMAHIA-NG-RD--P--G-N--CVSLL-R-
XP_009790281.1/1-233 ----------------------GG--L-VQEMAHIA-NG-RD--P--G-N--CVSLL-R-
XP_016570434.1/1-222 ----------------------GG--L-VQEMAHIA-NG-RD--P--G-N--CVSLL-R-
XP_016486479.1/1-229 ----------------------GG--L-VQEMAHIA-NG-RD--P--G-N--CVSLL-R-
XP_009597904.1/1-229 ----------------------GG--L-VQEMAHIA-NG-RD--P--G-N--CVSLL-R-
XP_009799795.1/1-229 ----------------------GG--L-VQEMAHIA-NG-RD--P--G-N--CVSLL-R-
XP_019249652.1/1-229 ----------------------GG--L-VQEMAHIA-NG-RD--P--G-N--CVSLL-R-
XP_006343296.1/1-221 ----------------------GG--L-VQEMAHIA-NG-RD--P--G-N--CVSLL-R-
XP_020217012.1/1-226 ----------------------GG--L-VQELAHIA-NG-RD--P--G-N--CVSLL-R-
XP_017406677.1/1-226 ----------------------GG--L-VQELAHIA-NG-RD--P--G-N--CVSLL-R-
XP_014519998.1/1-226 ----------------------GG--L-VQELAHIA-NG-RD--P--G-N--CVSLL-R-
XP_014519997.1/1-226 ----------------------GG--L-VQELAHIA-NG-RD--P--G-N--CVSLL-R-
XP_012841999.1/1-239 ----------------------GG--L-VQEMAHIA-NG-RD--P--G-N--SVSLL-R-
XP_024984347.1/1-227 ----------------------GG--L-VQEMAHIA-NG-RD--P--G-N--CVSLL-R-
XP_023751202.1/1-227 ----------------------GG--L-VQEMAHIA-NG-RD--P--G-N--CVSLL-R-
XP_023751208.1/1-227 ----------------------GG--L-VQEMAHIA-NG-RD--P--G-N--CVSLL-R-
XP_022041933.1/1-227 ----------------------GG--L-VQEMAHIA-NG-RD--P--G-N--CVSLL-R-
XP_024988632.1/1-226 ----------------------GG--L-VQEMAHIA-NG-RD--Q--G-N--CVSLL-R-
XP_022018253.1/1-226 ----------------------GG--L-VQEMAHIA-NG-PD--P--G-N--CVSLL-R-
XP_023771293.1/1-226 ----------------------GG--L-VQEMAHIA-NG-RD--P--G-N--SVSLL-R-
XP_019170324.1/1-226 ----------------------GG--L-VQEMAHIA-NG-RD--P--G-N--SVSLL-R-
XP_017230122.1/1-226 ----------------------GG--L-VQEMAHIA-NG-RD--P--G-N--CVSLL-R-
XP_011096101.1/1-226 ----------------------GG--L-VQEMAHIA-NG-RD--P--G-N--SVSLL-R-
XP_012848972.1/1-227 ----------------------GG--L-VQEMAHIA-NG-RD--P--G-N--SVSLL-R-
XP_022876975.1/1-227 ----------------------EG--L-VQEMAHIA-NG-RD--P--G-N--SVSLL-R-
XP_022863158.1/1-227 ----------------------GG--L-VQEMAHIA-NG-RD--P--G-N--SVSLL-R-
XP_022877991.1/1-227 ----------------------GG--L-VQEMAHIA-NG-RD--P--G-N--SVSLL-R-
XP_019223745.1/1-223 ----------------------GG--V-VQEMAHIA-NG-RE--T--G-N--CVSLL-R-
XP_021800220.1/1-221 ----------------------GG--I-VQEMAHIA-NG-SD--T--G-N--CVSLL-R-
XP_021684589.1/1-235 ----------------------GG--V-VQEMAHIA-NG-RD--T--G-N--CVSLL-R-
XP_021684519.1/1-235 ----------------------GG--V-VQEMAHIA-NG-RD--T--G-N--CVSLL-R-
XP_021684722.1/1-235 ----------------------GG--V-VQEMAHIA-NG-RD--T--G-N--CVSLL-R-
XP_021633498.1/1-230 ----------------------GG--V-VQEMAHIA-NG-RD--T--G-N--CVSLL-R-
XP_009420270.1/1-216 ----------------------GG--G-VQEMAHIA-NG-RD--H--G-N--CVSLL-R-
XP_003573403.1/1-232 ----------------------GG--I-VQEMAHIA-NG-RD--H--G-N--CVSLL-R-
XP_006659894.1/1-229 ----------------------GG--A-VQEMAHIA-NG-RD--H--G-N--SVSLL-R-
XP_015650022.1/1-263 ----------------------GG--A-VQEMAHIA-NG-RD--H--G-N--SVSLL-R-
XP_015650023.1/1-230 ----------------------GG--A-VQEMAHIA-NG-RD--H--G-N--SVSLL-R-
XP_020407343.1/1-231 ----------------------GG--A-VQEMAHIA-NG-RD--H--G-N--CVSLL-R-
NP_001105127.2/1-231 ----------------------GG--A-VQEMAHIA-NG-RD--H--G-N--CVSLL-R-
XP_008661805.1/1-239 ----------------------GG--A-VQEMAHIA-NG-RD--H--G-N--CVSLL-R-
NP_001145750.1/1-239 ----------------------GG--A-VQEMAHIA-NG-RD--H--G-N--CVSLL-R-
XP_025821536.1/1-230 ----------------------GG--A-VQEMAHIA-NG-RD--H--G-N--CVSLL-R-
XP_002443965.1/1-229 ----------------------GG--A-VQEMAHIA-NG-RD--H--G-N--CVSLL-R-
XP_004972697.1/1-229 ----------------------GG--A-VQEMAHIA-NG-RD--H--G-N--CVSLL-R-
XP_008667765.1/1-230 ----------------------GG--V-VQEMAHIA-NG-RD--H--G-N--CVSLL-R-
NP_001347891.1/1-231 ----------------------GG--V-VQEMAHIA-NG-RD--H--G-N--CVSLL-R-
XP_015636340.1/1-229 ----------------------GG--I-VQEMAHIA-NG-RD--Q--G-N--CVSLL-R-
XP_015636338.1/1-229 ----------------------GG--I-VQEMAHIA-NG-RD--Q--G-N--CVSLL-R-
XP_006652859.1/1-228 ----------------------GG--I-VQEMAHIA-NG-RD--Q--G-N--CVSLL-R-
XP_003580598.1/1-233 ----------------------GG--V-VQEMAHIA-NG-RD--H--G-N--CVSLL-R-
XP_020199198.1/1-227 ----------------------GG--I-VQEMAHIA-NG-RD--H--G-N--CVSLL-R-
XP_020400772.1/1-230 ----------------------GG--V-VQEMAHIA-NG-RD--H--G-N--CVSLL-R-
NP_001334663.1/1-230 ----------------------GG--V-VQEMAHIA-NG-RD--H--G-N--CVSLL-R-
XP_008662197.1/1-270 ----------------------GG--V-VQEMAHIA-NG-RD--H--G-N--CVSLL-R-
XP_008662196.1/1-270 ----------------------GG--V-VQEMAHIA-NG-RD--H--G-N--CVSLL-R-
XP_025823505.1/1-227 ----------------------GG--I-VQEMAHIA-NG-RD--H--G-N--CVSLL-R-
XP_004976894.1/1-227 ----------------------GG--V-VQEMAHIA-NG-RD--H--G-N--CVSLL-R-
XP_002448563.2/1-227 ----------------------GG--V-VQEMAHIA-NG-RD--H--G-N--CVSLL-R-
XP_019414154.1/1-227 ----------------------GG--V-VQEMAHIA-NG-RE--T--G-N--CVSLL-R-
XP_006348085.1/1-234 ----------------------GG--I-VQEMAHIA-NG-RD--P--G-N--CVSLL-R-
XP_004232734.1/1-234 ----------------------GG--I-VQEMAHIA-NG-RD--P--G-N--CVSLL-R-
XP_015066898.1/1-234 ----------------------GG--I-VQEMAHIA-NG-RD--P--G-N--CVSLL-R-
XP_003605900.1/1-232 ----------------------GG--V-VQEMAHIA-NG-RD--T--G-N--CVSLL-R-
XP_022988693.1/1-229 ----------------------GG--V-VQEMAHIA-NG-RD--T--G-N--SVSLL-R-
XP_022930609.1/1-229 ----------------------GG--V-VQEMAHIA-NG-RD--T--G-N--SVSLL-R-
XP_023530197.1/1-229 ----------------------GG--V-VQEMAHIA-NG-RD--T--G-N--SVSLL-R-
XP_016188415.1/1-237 ----------------------GG--A-VQEMAHIA-NG-RD--T--G-N--CVSLL-R-
XP_011628991.1/1-236 ----------------------GG--V-VQEMAHIA-NG-PD--P--G-N--CVSLL-R-
XP_006826512.2/1-236 ----------------------GG--V-VQEMAHIA-NG-PD--P--G-N--CVSLL-R-
XP_020177398.1/1-220 ----------------------GG--V-VQEMAHIA-NG-SH--H--G-N--AVSLL-R-
XP_015695486.1/1-219 ----------------------GG--V-VQEMAHIA-NG-RD--H--G-N--AVSLL-R-
XP_015648539.1/1-217 ----------------------GG--V-VQEMAHIA-NG-RD--H--G-N--AVSLL-R-
XP_020401599.1/1-218 ----------------------GG--D-VQEMAHIA-NG-RD--H--G-N--AVSLL-R-
XP_020401598.1/1-218 ----------------------GG--D-VQEMAHIA-NG-RD--H--G-N--AVSLL-R-
XP_020401597.1/1-218 ----------------------GG--D-VQEMAHIA-NG-RD--H--G-N--AVSLL-R-
XP_021319954.1/1-239 ----------------------GG--D-VQEMAHIA-NG-RD--H--G-N--AVSLL-R-
XP_002445014.2/1-239 ----------------------GG--D-VQEMAHIA-NG-RD--H--G-N--AVSLL-R-
XP_025819913.1/1-218 ----------------------GG--D-VQEMAHIA-NG-RD--H--G-N--AVSLL-R-
XP_004972909.1/1-218 ----------------------GG--D-VQEMAHIA-NG-RD--H--G-N--AVSLL-R-
XP_020100432.1/1-229 ----------------------GG--I-VQEMAHIA-NG-RD--H--G-N--CVSLL-R-
XP_009385059.1/1-228 ----------------------GG--G-VQEMAHIA-NG-RD--H--G-N--CVSLL-R-
XP_018678797.1/1-226 ----------------------GG--G-VQEMAHIA-NG-QD--H--G-N--CVSLL-R-
XP_009412012.1/1-233 ----------------------GG--V-VQEMAHIA-NG-QD--H--G-N--CVSLL-R-
XP_018685149.1/1-236 ----------------------GG--V-VQEMAHIA-NG-QD--H--G-N--CVSLL-R-
XP_020591278.1/1-230 ----------------------GG--V-VQEMAHIA-NG-RD--H--G-N--CVSLL-R-
XP_020702932.1/1-229 ----------------------GG--V-VQEMAHIA-NG-RD--H--G-N--CVSLL-R-
XP_020702933.1/1-230 ----------------------GG--V-VQEMAHIA-NG-RD--H--G-N--CVSLL-R-
XP_020702931.1/1-230 ----------------------GG--V-VQEMAHIA-NG-RD--H--G-N--CVSLL-R-
XP_020692781.1/1-229 ----------------------GG--V-VQEMAHIA-NG-RD--H--G-N--SVSLL-R-
XP_020692780.1/1-229 ----------------------GG--V-VQEMAHIA-NG-RD--H--G-N--SVSLL-R-
XP_020591591.1/1-229 ----------------------GG--V-VQEMAHIA-NG-RE--H--G-N--CVSLL-R-
XP_020591590.1/1-229 ----------------------GG--V-VQEMAHIA-NG-RE--H--G-N--CVSLL-R-
XP_020109359.1/1-227 ----------------------GG--V-VQEMAHIA-NG-RD--H--G-N--CVSLL-R-
XP_020270114.1/1-229 ----------------------GG--V-VQEMAHIA-NG-RD--H--G-N--CVSLL-R-
XP_010908855.1/1-227 ----------------------GG--V-VQEMAHIA-NG-RD--H--G-N--CVSLL-R-
XP_008808987.1/1-227 ----------------------GG--V-VQEMAHIA-NG-RD--H--G-N--CVSLL-R-
XP_008784552.1/1-223 ----------------------GG--V-VQEMAHIA-NG-RD--H--G-N--CVSLL-R-
XP_010931820.1/1-223 ----------------------GG--V-VQEMAHIA-NG-RD--H--G-N--CVSLL-R-
XP_003540308.2/1-226 ----------------------GG--V-VQEMAHIA-NG-RD--T--G-N--CVSLL-R-
XP_006592818.1/1-226 ----------------------GG--V-VQEMAHIA-NG-RD--T--G-N--CVSLL-R-
XP_006592816.1/1-226 ----------------------GG--V-VQEMAHIA-NG-RD--T--G-N--CVSLL-R-
XP_003543387.1/1-226 ----------------------GG--V-VQEMAHIA-NG-RD--T--G-N--CVSLL-R-
XP_020223651.1/1-226 ----------------------GG--V-VQEMAHIA-NG-RD--T--G-N--CVSLL-R-
XP_020223648.1/1-226 ----------------------GG--V-VQEMAHIA-NG-RD--T--G-N--CVSLL-R-
XP_007149968.1/1-226 ----------------------GG--V-VQEMAHIA-NG-RE--T--G-N--CVSLL-R-
XP_017425582.1/1-226 ----------------------GG--V-VQEMAHIA-NG-RD--T--G-N--CVSLL-R-
XP_014501634.1/1-226 ----------------------GG--V-VQEMAHIA-NG-RD--T--G-N--CVSLL-R-
XP_022776632.1/1-226 ----------------------GG--V-VQEMAHIA-NG-RD--T--G-N--CVSLL-R-
XP_022776631.1/1-226 ----------------------GG--V-VQEMAHIA-NG-RD--T--G-N--CVSLL-R-
XP_022776629.1/1-226 ----------------------GG--V-VQEMAHIA-NG-RD--T--G-N--CVSLL-R-
XP_022733641.1/1-226 ----------------------GG--V-VQEMAHIA-NG-RD--T--G-N--CVSLL-R-
XP_022733640.1/1-226 ----------------------GG--V-VQEMAHIA-NG-RD--T--G-N--CVSLL-R-
XP_022733638.1/1-226 ----------------------GG--V-VQEMAHIA-NG-RD--T--G-N--CVSLL-R-
XP_007045612.1/1-226 ----------------------GG--V-VQEMAHIA-NG-RD--T--G-N--CVSLL-R-
XP_007045611.1/1-226 ----------------------GG--V-VQEMAHIA-NG-RD--T--G-N--CVSLL-R-
XP_017971478.1/1-226 ----------------------GG--V-VQEMAHIA-NG-RD--T--G-N--CVSLL-R-
XP_021292769.1/1-226 ----------------------GG--V-VQEMAHIA-NG-RD--T--G-N--CVSLL-R-
XP_021292767.1/1-226 ----------------------GG--V-VQEMAHIA-NG-RD--T--G-N--CVSLL-R-
XP_021292765.1/1-226 ----------------------GG--V-VQEMAHIA-NG-RD--T--G-N--CVSLL-R-
XP_010657309.1/1-226 ----------------------GG--V-VQEMAHIA-NG-QD--T--G-N--CVSLL-R-
XP_010657313.1/1-226 ----------------------GG--V-VQEMAHIA-NG-QD--T--G-N--CVSLL-R-
XP_010657310.1/1-226 ----------------------GG--V-VQEMAHIA-NG-QD--T--G-N--CVSLL-R-
XP_010657311.1/1-226 ----------------------GG--V-VQEMAHIA-NG-QD--T--G-N--CVSLL-R-
XP_012086347.1/1-228 ----------------------GG--V-VQEMAHIA-NG-RD--T--G-N--CVSLL-R-
XP_012086344.1/1-228 ----------------------GG--V-VQEMAHIA-NG-RD--T--G-N--CVSLL-R-
XP_024029967.1/1-226 ----------------------GG--V-VQEMAHIA-NG-RD--T--G-N--CVSLL-R-
XP_010109517.1/1-226 ----------------------GG--V-VQEMAHIA-NG-RD--T--G-N--CVSLL-R-
XP_006469697.1/1-226 ----------------------GG--V-VQEMAHIA-NG-RD--T--G-N--CVSLL-R-
XP_024046029.1/1-226 ----------------------GG--V-VQEMAHIA-NG-RD--T--G-N--CVSLL-R-
XP_020234199.1/1-226 ----------------------GG--V-VQEMAHIA-NG-RD--T--G-N--CVSLL-R-
XP_020234201.1/1-226 ----------------------GG--V-VQEMAHIA-NG-RD--T--G-N--CVSLL-R-
XP_021630856.1/1-226 ----------------------GG--V-VQEMAHIA-NG-RD--T--G-N--CVSLL-R-
XP_021630853.1/1-226 ----------------------GG--V-VQEMAHIA-NG-RD--T--G-N--CVSLL-R-
XP_021907367.1/1-221 ----------------------GG--V-VSEMAHIA-NG-RD--T--G-N--SVSLL-R-
XP_010275996.1/1-221 ----------------------GG--V-VQEMAHIA-NG-RD--P--G-N--CVSLL-R-
XP_020870784.1/1-223 ----------------------GG--V-VQEMAHIA-NG-RD--T--G-N--CVSLL-R-
NP_172015.1/1-223    ----------------------GG--V-VQEMAHIA-NG-RD--T--G-N--CVSLL-R-
XP_006418050.1/1-223 ----------------------GG--V-VQEMAHIA-NG-RD--T--G-N--CVSLL-R-
XP_018447753.1/1-223 ----------------------GG--V-VQEMAHIA-NG-RD--T--G-N--CVSLL-R-
XP_010457605.1/1-218 ----------------------GG--V-VQEMAHIA-NG-RD--T--G-N--CVSLL-R-
XP_023632765.1/1-218 ----------------------GG--V-VQEMAHIA-NG-RD--T--G-N--CVSLL-R-
XP_010484829.1/1-218 ----------------------GG--V-VQEMAHIA-NG-RD--T--G-N--CVSLL-R-
XP_010475208.1/1-218 ----------------------GG--V-VQEMAHIA-NG-RD--T--G-N--CVSLL-R-
XP_018487097.1/1-220 ----------------------GG--V-VQEMAHIA-NG-RD--T--G-N--CVSLL-R-
XP_013664795.1/1-220 ----------------------GG--V-VQEMAHIA-NG-RE--T--G-N--CVSLL-R-
XP_013716253.1/1-220 ----------------------GG--V-VQEMAHIA-NG-RE--T--G-N--CVSLL-R-
XP_009118607.1/1-220 ----------------------GG--V-VQEMAHIA-NG-RE--T--G-N--CVSLL-R-
XP_013605632.1/1-220 ----------------------GG--V-VQEMAHIA-NG-RD--T--G-N--CVSLL-R-
XP_010475204.1/1-220 ----------------------GG--V-VQEMAHIA-NG-RD--T--G-N--CVSLL-R-
XP_010457604.1/1-220 ----------------------GG--V-VQEMAHIA-NG-RD--T--G-N--CVSLL-R-
XP_023632762.1/1-220 ----------------------GG--V-VQEMAHIA-NG-RD--T--G-N--CVSLL-R-
XP_010484823.1/1-220 ----------------------GG--V-VQEMAHIA-NG-RD--T--G-N--CVSLL-R-
XP_022558401.1/1-220 ----------------------GG--V-VQEMAHIA-NG-RD--T--G-N--CVSLL-R-
XP_013586641.1/1-220 ----------------------GG--V-VQEMAHIA-NG-RD--T--G-N--CVSLL-R-
XP_022558399.1/1-220 ----------------------GG--V-VQEMAHIA-NG-RD--T--G-N--CVSLL-R-
XP_022558400.1/1-220 ----------------------GG--V-VQEMAHIA-NG-RD--T--G-N--CVSLL-R-
XP_013586642.1/1-220 ----------------------GG--V-VQEMAHIA-NG-RD--T--G-N--CVSLL-R-
XP_013696832.1/1-220 ----------------------GG--V-VQEMAHIA-NG-RD--T--G-N--CVSLL-R-
XP_022548878.1/1-220 ----------------------GG--V-VQEMAHIA-NG-RD--T--G-N--CVSLL-R-
XP_013666552.1/1-220 ----------------------GG--V-VQEMAHIA-NG-RD--T--G-N--CVSLL-R-
XP_009119177.1/1-220 ----------------------GG--V-VQEMAHIA-NG-RD--T--G-N--CVSLL-R-
XP_020964430.1/1-225 ----------------------GG--V-VQEMAHIA-NG-RD--T--G-N--CVSLL-R-
XP_015936069.1/1-225 ----------------------GG--V-VQEMAHIA-NG-RD--T--G-N--CVSLL-R-
XP_020964429.1/1-225 ----------------------GG--V-VQEMAHIA-NG-RD--T--G-N--CVSLL-R-
XP_015936068.1/1-225 ----------------------GG--V-VQEMAHIA-NG-RD--T--G-N--CVSLL-R-
XP_015577880.1/1-228 ----------------------GG--V-VQEMAHIA-NG-RD--T--G-N--CVSLL-R-
XP_002524172.2/1-228 ----------------------GG--V-VQEMAHIA-NG-RD--T--G-N--CVSLL-R-
XP_007132557.1/1-221 ----------------------GG--V-VQEMAHIA-NG-RD--T--G-N--CVSLL-R-
XP_017433694.1/1-221 ----------------------GG--V-VQEMAHIA-NG-RD--T--G-N--CVSLL-R-
XP_014494419.1/1-221 ----------------------GG--V-VQEMAHIA-NG-RD--T--G-N--CVSLL-R-
XP_022634310.1/1-221 ----------------------GG--V-VQEMAHIA-NG-RD--T--G-N--CVSLL-R-
XP_010267735.1/1-225 ----------------------GG--V-VQEMAHIA-NG-RD--P--G-N--CVSLL-R-
XP_010267734.1/1-225 ----------------------GG--V-VQEMAHIA-NG-RD--P--G-N--CVSLL-R-
XP_024185199.1/1-224 ----------------------GG--V-VQEMAHIA-NG-RD--T--G-N--CVSLL-R-
XP_024185197.1/1-224 ----------------------GG--V-VQEMAHIA-NG-RD--T--G-N--CVSLL-R-
XP_009342246.1/1-222 ----------------------GG--I-VQEMAHIA-NG-RD--T--G-N--CVSLL-R-
XP_009342244.1/1-222 ----------------------GG--I-VQEMAHIA-NG-RD--T--G-N--CVSLL-R-
XP_009342240.1/1-222 ----------------------GG--I-VQEMAHIA-NG-RD--T--G-N--CVSLL-R-
XP_020416503.1/1-221 ----------------------GG--I-VQEMAHIA-NG-RD--T--G-N--CVSLL-R-
XP_021822854.1/1-221 ----------------------GG--I-VQEMAHIA-NG-RD--T--G-N--CVSLL-R-
XP_007217584.1/1-221 ----------------------GG--I-VQEMAHIA-NG-RD--T--G-N--CVSLL-R-
XP_021822852.1/1-221 ----------------------GG--I-VQEMAHIA-NG-RD--T--G-N--CVSLL-R-
XP_018730806.1/1-228 ----------------------GG--V-VQEMAHIA-NG-RD--T--G-N--CVSLL-R-
XP_010051064.1/1-228 ----------------------GG--V-VQEMAHIA-NG-RD--T--G-N--CVSLL-R-
XP_015881130.2/1-229 ----------------------GG--A-VQEMAHIA-NG-RD--T--G-N--CVSLL-R-
XP_023534533.1/1-219 ----------------------GG--V-VQEMAHIA-NG-RD--T--G-N--CVSLL-R-
XP_022947095.1/1-219 ----------------------GG--V-VQEMAHIA-NG-RD--T--G-N--CVSLL-R-
XP_022947096.1/1-219 ----------------------GG--V-VQEMAHIA-NG-RD--T--G-N--CVSLL-R-
XP_022970950.1/1-219 ----------------------GG--V-VQEMAHIA-NG-RD--T--G-N--CVSLL-R-
XP_022970951.1/1-219 ----------------------GG--V-VQEMAHIA-NG-RD--T--G-N--CVSLL-R-
XP_022140692.1/1-217 ----------------------GG--V-VQEMAHIA-NG-RD--T--G-N--CVSLL-R-
XP_022140691.1/1-217 ----------------------GG--V-VQEMAHIA-NG-RD--T--G-N--CVSLL-R-
XP_008455853.1/1-220 ----------------------GG--V-VQEMAHIA-NG-RD--T--G-N--CVSLL-R-
XP_011650012.1/1-220 ----------------------GG--V-VQEMAHIA-NG-RD--T--G-N--CVSLL-R-
XP_008455850.1/1-220 ----------------------GG--V-VQEMAHIA-NG-RD--T--G-N--CVSLL-R-
XP_011650011.1/1-220 ----------------------GG--V-VQEMAHIA-NG-RD--T--G-N--CVSLL-R-
XP_023513403.1/1-220 ----------------------GG--V-VQEMAHIA-NG-RD--T--G-N--CVSLL-R-
XP_022944761.1/1-220 ----------------------GG--V-VQEMAHIA-NG-RD--T--G-N--CVSLL-R-
XP_023513401.1/1-220 ----------------------GG--V-VQEMAHIA-NG-RD--T--G-N--CVSLL-R-
XP_022944759.1/1-220 ----------------------GG--V-VQEMAHIA-NG-RD--T--G-N--CVSLL-R-
XP_022986270.1/1-220 ----------------------GG--V-VQEMAHIA-NG-HD--T--G-N--CVSLL-R-
XP_022986268.1/1-220 ----------------------GG--V-VQEMAHIA-NG-HD--T--G-N--CVSLL-R-
XP_022136006.1/1-224 ----------------------GG--V-VQEMAHIA-NG-RD--T--G-N--CVSLL-R-
XP_008455423.1/1-229 ----------------------GG--V-VQEMAHIA-NG-RD--T--G-N--CVSLL-R-
XP_011658704.1/1-229 ----------------------GG--V-VQEMAHIA-NG-RD--T--G-N--CVSLL-R-
XP_004144488.1/1-229 ----------------------GG--V-VQEMAHIA-NG-RD--T--G-N--CVSLL-R-
XP_022968990.1/1-229 ----------------------GG--I-VQEMAHIA-NG-RD--T--G-N--CVSLL-R-
XP_023554403.1/1-229 ----------------------GG--I-VQEMAHIA-NG-RD--T--G-N--CVSLL-R-
XP_022952948.1/1-229 ----------------------GG--I-VQEMAHIA-NG-RD--T--G-N--CVSLL-R-
XP_019447751.1/1-228 ----------------------GG--V-VQEMAHIA-NG-RD--T--G-N--SVSLL-R-
XP_019447749.1/1-228 ----------------------GG--V-VQEMAHIA-NG-RD--T--G-N--SVSLL-R-
XP_004515074.1/1-228 ----------------------GG--V-VQEMAHIA-NG-RD--T--G-N--CVSLL-R-
XP_012463792.1/1-235 ----------------------GG--V-VQEMAHIA-NG-RD--T--G-N--CVSLL-R-
XP_012463790.1/1-235 ----------------------GG--V-VQEMAHIA-NG-RD--T--G-N--CVSLL-R-
XP_016678783.1/1-235 ----------------------GG--V-VQEMAHIA-NG-RD--T--G-N--CVSLL-R-
XP_016678782.1/1-235 ----------------------GG--V-VQEMAHIA-NG-RD--T--G-N--CVSLL-R-
XP_017631648.1/1-235 ----------------------GG--V-VQEMAHIA-NG-RD--T--G-N--CVSLL-R-
XP_016732503.1/1-235 ----------------------GG--V-VQEMAHIA-NG-RD--T--G-N--CVSLL-R-
XP_017631647.1/1-235 ----------------------GG--V-VQEMAHIA-NG-RD--T--G-N--CVSLL-R-
XP_012438430.1/1-229 ----------------------GG--V-AQEMAHIA-NG-RD--T--G-N--CVSLL-R-
XP_012438427.1/1-229 ----------------------GG--V-AQEMAHIA-NG-RD--T--G-N--CVSLL-R-
XP_012438429.1/1-229 ----------------------GG--V-AQEMAHIA-NG-RD--T--G-N--CVSLL-R-
XP_017616162.1/1-229 ----------------------GG--V-VQEMAHIA-NG-RD--T--G-N--CVSLL-R-
XP_017616160.1/1-229 ----------------------GG--V-VQEMAHIA-NG-RD--T--G-N--CVSLL-R-
XP_017616161.1/1-229 ----------------------GG--V-VQEMAHIA-NG-RD--T--G-N--CVSLL-R-
XP_016726347.1/1-229 ----------------------GG--V-VQEMAHIA-NG-RD--T--G-N--CVSLL-R-
XP_016726344.1/1-229 ----------------------GG--V-VQEMAHIA-NG-RD--T--G-N--CVSLL-R-
XP_016726345.1/1-229 ----------------------GG--V-VQEMAHIA-NG-RD--T--G-N--CVSLL-R-
XP_019448933.1/1-227 ----------------------GG--A-VQEMAHIA-NG-RE--T--G-N--CVSLL-R-
XP_004487400.1/1-228 ----------------------GG--V-VQEMAHIA-NG-RD--T--G-N--CVSLL-R-
XP_003596999.2/1-227 ----------------------GG--V-VQEMAHIA-NG-RD--T--G-N--CVSLL-R-
XP_024631890.1/1-227 ----------------------GG--V-VQEMAHIA-NG-RD--T--G-N--CVSLL-R-
XP_024631891.1/1-227 ----------------------GG--V-VQEMAHIA-NG-RD--T--G-N--CVSLL-R-
XP_004287935.1/1-224 ----------------------GG--V-VQEMAHIA-NG-RD--T--G-N--CVSLL-R-
XP_018808969.1/1-228 ----------------------GG--V-VQEMAHIA-NG-RD--T--G-N--SVSLL-R-
XP_018808967.1/1-228 ----------------------GG--V-VQEMAHIA-NG-RD--T--G-N--SVSLL-R-
XP_018817598.1/1-227 ----------------------GG--V-VQEMAHIA-NG-RD--T--G-N--CVSLL-R-
XP_018817597.1/1-227 ----------------------GG--V-VQEMAHIA-NG-RD--T--G-N--CVSLL-R-
XP_018817594.1/1-227 ----------------------GG--V-VQEMAHIA-NG-RD--T--G-N--CVSLL-R-
XP_018817599.1/1-227 ----------------------GG--V-VQEMAHIA-NG-RD--T--G-N--CVSLL-R-
XP_023915056.1/1-227 ----------------------GG--V-VQEMAHIA-NG-RE--T--G-N--CVSLL-R-
XP_023915054.1/1-227 ----------------------GG--V-VQEMAHIA-NG-RE--T--G-N--CVSLL-R-
XP_023915053.1/1-227 ----------------------GG--V-VQEMAHIA-NG-RE--T--G-N--CVSLL-R-
XP_021685023.1/1-228 ----------------------GG--V-VQEMAHIA-NG-RD--T--G-N--CVSLL-R-
XP_021684657.1/1-228 ----------------------GG--V-VQEMAHIA-NG-RD--T--G-N--CVSLL-R-
XP_021685089.1/1-228 ----------------------GG--V-VQEMAHIA-NG-RD--T--G-N--CVSLL-R-
XP_015383009.1/1-229 ----------------------GG--V-VQEMAHIA-NG-RD--T--G-N--CVSLL-R-
XP_015383007.1/1-229 ----------------------GG--V-VQEMAHIA-NG-RD--T--G-N--CVSLL-R-
XP_020539441.1/1-235 ----------------------GG--V-VQEMAHIA-NG-RD--T--G-N--CVSLL-R-
XP_020539439.1/1-235 ----------------------GG--V-VQEMAHIA-NG-RD--T--G-N--CVSLL-R-
XP_015577881.1/1-233 ----------------------GG--V-VQEMAHIA-NG-RD--T--G-N--CVSLL-R-
XP_015577879.1/1-233 ----------------------GG--V-VQEMAHIA-NG-RD--T--G-N--CVSLL-R-
XP_015577877.1/1-233 ----------------------GG--V-VQEMAHIA-NG-RD--T--G-N--CVSLL-R-
XP_021630854.1/1-231 ----------------------GG--V-VQEMAHIA-NG-RD--T--G-N--CVSLL-R-
XP_021630849.1/1-231 ----------------------GG--V-VQEMAHIA-NG-RD--T--G-N--CVSLL-R-
XP_021652390.1/1-229 ----------------------GG--V-VQEMAHIA-NG-RD--T--G-N--CVSLL-R-
XP_021652388.1/1-229 ----------------------GG--V-VQEMAHIA-NG-RD--T--G-N--CVSLL-R-
XP_021652389.1/1-234 ----------------------GG--V-VQEMAHIA-NG-RD--T--G-N--CVSLL-R-
XP_021652386.1/1-234 ----------------------GG--V-VQEMAHIA-NG-RD--T--G-N--CVSLL-R-
XP_006592380.1/1-228 ----------------------GG--V-VQEMAHIA-NG-RD--T--G-N--CVSLL-R-
XP_003539872.2/1-228 ----------------------GG--V-VQEMAHIA-NG-RD--T--G-N--CVSLL-R-
XP_025984806.1/1-230 ----------------------GG--V-VQEMAHIA-NG-RD--T--G-N--CVSLL-R-
XP_014632311.1/1-230 ----------------------GG--V-VQEMAHIA-NG-RD--T--G-N--CVSLL-R-
XP_024450362.1/1-227 ----------------------GG--V-VQEMAHIA-NG-RD--T--G-N--CVSLI-R-
XP_002302880.2/1-227 ----------------------GG--V-VQEMAHIA-NG-RD--T--G-N--CVSLI-R-
XP_024450361.1/1-227 ----------------------GG--V-VQEMAHIA-NG-RD--T--G-N--CVSLI-R-
XP_024450360.1/1-227 ----------------------GG--V-VQEMAHIA-NG-RD--T--G-N--CVSLI-R-
XP_011011493.1/1-227 ----------------------GG--V-VQEMAHIA-NG-RD--T--G-N--SVSLI-R-
XP_011011491.1/1-227 ----------------------GG--V-VQEMAHIA-NG-RD--T--G-N--SVSLI-R-
XP_011011492.1/1-227 ----------------------GG--V-VQEMAHIA-NG-RD--T--G-N--SVSLI-R-
XP_011011489.1/1-227 ----------------------GG--V-VQEMAHIA-NG-RD--T--G-N--SVSLI-R-
XP_011011488.1/1-227 ----------------------GG--V-VQEMAHIA-NG-RD--T--G-N--SVSLI-R-
XP_011005116.1/1-225 ----------------------GG--V-VQEMAHIA-NG-RD--T--G-N--CVSLL-R-
XP_011005113.1/1-225 ----------------------GG--V-VQEMAHIA-NG-RD--T--G-N--CVSLL-R-
XP_011005112.1/1-225 ----------------------GG--V-VQEMAHIA-NG-RD--T--G-N--CVSLL-R-
XP_024440391.1/1-225 ----------------------GG--V-VQEMAHIA-NG-RD--T--G-N--CVSLL-R-
XP_002320458.2/1-225 ----------------------GG--V-VQEMAHIA-NG-RD--T--G-N--CVSLL-R-
XP_024440389.1/1-225 ----------------------GG--V-VQEMAHIA-NG-RD--T--G-N--CVSLL-R-
XP_019453767.1/1-227 ----------------------GG--A-VQEMAHIA-NG-QE--T--G-N--CVSLL-R-
XP_012478857.1/1-227 ----------------------GG--A-VQEMAHIA-NG-RD--P--G-N--CVSLL-R-
XP_012478855.1/1-227 ----------------------GG--A-VQEMAHIA-NG-RD--P--G-N--CVSLL-R-
XP_012478856.1/1-227 ----------------------GG--A-VQEMAHIA-NG-RD--P--G-N--CVSLL-R-
XP_012478851.1/1-227 ----------------------GG--A-VQEMAHIA-NG-RD--P--G-N--CVSLL-R-
XP_016724999.1/1-227 ----------------------GG--A-VQEMAHIA-NG-RD--P--G-N--CVSLL-R-
XP_017638204.1/1-227 ----------------------GG--A-VQEMAHIA-NG-RD--P--G-N--CVSLL-R-
XP_017638203.1/1-227 ----------------------GG--A-VQEMAHIA-NG-RD--P--G-N--CVSLL-R-
XP_016667113.1/1-227 ----------------------GG--A-VQEMAHIA-NG-RD--P--G-N--CVSLL-R-
XP_016667112.1/1-227 ----------------------GG--A-VQEMAHIA-NG-RD--P--G-N--CVSLL-R-
XP_015881131.2/1-227 ----------------------GG--A-VQEMAHIA-NG-RD--T--G-N--CVSLL-R-
XP_008348131.1/1-222 ----------------------GG--I-VQEMAHIA-NG-RD--T--G-N--CVSLL-R-
XP_008348133.1/1-220 ----------------------GG--I-VQEMAHIA-NG-RD--T--G-N--CVSLL-R-
XP_018502305.1/1-220 ----------------------GG--I-VQEMAHIA-NG-RD--T--G-N--CVSLL-R-
XP_009342243.1/1-220 ----------------------GG--I-VQEMAHIA-NG-RD--T--G-N--CVSLL-R-
XP_017179321.1/1-222 ----------------------GG--I-VQEMAHIA-NG-HD--T--G-N--CVSLL-R-
XP_017179320.1/1-222 ----------------------GG--I-VQEMAHIA-NG-HD--T--G-N--CVSLL-R-
XP_008341986.1/1-222 ----------------------GG--I-VQEMAHIA-NG-HD--T--G-N--CVSLL-R-
XP_017179319.1/1-220 ----------------------GG--I-VQEMAHIA-NG-HD--T--G-N--CVSLL-R-
XP_024185198.1/1-222 ----------------------GG--V-VQEMAHIA-NG-RD--T--G-N--CVSLL-R-
XP_011463209.1/1-222 ----------------------GG--V-VQEMAHIA-NG-RD--T--G-N--CVSLL-R-
XP_020416501.1/1-219 ----------------------GG--I-VQEMAHIA-NG-RD--T--G-N--CVSLL-R-
XP_021822853.1/1-219 ----------------------GG--I-VQEMAHIA-NG-RD--T--G-N--CVSLL-R-
XP_008230079.1/1-219 ----------------------GG--I-VQEMAHIA-NG-RD--T--G-N--CVSLL-R-
XP_020870786.1/1-221 ----------------------GG--V-VQEMAHIA-NG-RD--T--G-N--CVSLL-R-
NP_001117231.1/1-221 ----------------------GG--V-VQEMAHIA-NG-RD--T--G-N--CVSLL-R-
XP_018817596.1/1-225 ----------------------GG--V-VQEMAHIA-NG-RD--T--G-N--CVSLL-R-
XP_019079350.1/1-224 ----------------------GG--V-VQEMAHIA-NG-QD--T--G-N--CVSLL-R-
XP_022733639.1/1-224 ----------------------GG--V-VQEMAHIA-NG-RD--T--G-N--CVSLL-R-
XP_021292768.1/1-224 ----------------------GG--V-VQEMAHIA-NG-RD--T--G-N--CVSLL-R-
XP_021292766.1/1-224 ----------------------GG--V-VQEMAHIA-NG-RD--T--G-N--CVSLL-R-
XP_017971480.1/1-224 ----------------------GG--V-VQEMAHIA-NG-RD--T--G-N--CVSLL-R-
XP_018721484.1/1-222 ----------------------GG--I-VQEMGHIA-NG-RD--T--G-N--CVSLL-R-
XP_018721483.1/1-222 ----------------------GG--I-VQEMGHIA-NG-RD--T--G-N--CVSLL-R-
XP_018721482.1/1-222 ----------------------GG--I-VQEMGHIA-NG-RD--T--G-N--CVSLL-R-
XP_025687365.1/1-237 ----------------------GG--A-VQEMAHIA-NG-RD--T--G-N--CVSLL-R-
XP_006851770.2/1-243 ----------------------GG--P-MQEMAHIA-KG-QD--H--G-N--CVSLL-R-
XP_003623826.1/1-230 ----------------------GG--P-MQEMAHIA-KG-HD--H--G-N--CVSLL-R-
XP_004492678.1/1-230 ----------------------GG--P-MQEMAHIA-KG-HN--H--G-N--SVSLL-R-
XP_004492677.1/1-230 ----------------------GG--P-MQEMAHIA-KG-HN--H--G-N--SVSLL-R-
XP_012847897.1/1-236 ----------------------GG--P-MQEMAHIA-KG-QD--H--G-N--CVSLL-R-
XP_012843673.1/1-229 ----------------------GG--P-MQEMAHIA-KG-QD--H--G-N--CVSLL-R-
XP_021279608.1/1-239 ----------------------GG--P-MQEMAHIA-KG-QD--H--G-N--CVSLL-R-
XP_025641241.1/1-241 ----------------------GG--P-MQEMAHIA-KG-QD--H--G-N--CVSLL-R-
XP_025641246.1/1-241 ----------------------GG--P-MQEMAHIA-KG-QD--H--G-N--CVSLL-R-
XP_020991942.1/1-241 ----------------------GG--P-MQEMAHIA-KG-QD--H--G-N--CVSLL-R-
XP_025632115.1/1-241 ----------------------GG--P-MQEMAHIA-KG-QD--H--G-N--CVSLL-R-
XP_025632116.1/1-241 ----------------------GG--P-MQEMAHIA-KG-QD--H--G-N--CVSLL-R-
XP_017442222.1/1-246 ----------------------GG--P-MQEMAHIA-KG-QD--H--G-N--CVSLL-R-
XP_017442225.1/1-246 ----------------------GG--P-MQEMAHIA-KG-QD--H--G-N--CVSLL-R-
XP_006576359.1/1-244 ----------------------GG--P-MQEMAHIA-KG-QD--H--G-N--AVSLL-R-
XP_003521284.1/1-244 ----------------------GG--P-MQEMAHIA-KG-QD--H--G-N--AVSLL-R-
XP_024174849.1/1-237 ----------------------GG--P-MQEMAHIA-KG-QD--Q--G-N--CVSLL-R-
XP_024174854.1/1-237 ----------------------GG--P-MQEMAHIA-KG-QD--Q--G-N--CVSLL-R-
XP_004306832.1/1-241 ----------------------GG--P-MQEMAHIA-KG-QD--Q--G-N--CVSLL-R-
XP_011469062.1/1-241 ----------------------GG--P-MQEMAHIA-KG-QD--Q--G-N--CVSLL-R-
XP_009340632.2/1-243 ----------------------GG--P-MQEMAHIA-KG-QD--P--G-N--CVSLL-R-
XP_008385703.1/1-243 ----------------------GG--P-MQEMAHIA-KG-QD--P--G-N--CVSLL-R-
XP_008385704.1/1-243 ----------------------GG--P-MQEMAHIA-KG-QD--P--G-N--CVSLL-R-
XP_008232741.1/1-243 ----------------------GG--P-MQEMAHIA-KG-QD--P--G-N--CVSLL-R-
XP_008232742.1/1-243 ----------------------GG--P-MQEMAHIA-KG-QD--P--G-N--CVSLL-R-
XP_007220256.1/1-243 ----------------------GG--P-MQEMAHIA-KG-QD--P--G-N--CVSLL-R-
XP_020413144.1/1-243 ----------------------GG--P-MQEMAHIA-KG-QD--P--G-N--CVSLL-R-
XP_021817103.1/1-243 ----------------------GG--P-MQEMAHIA-KG-QD--P--G-N--CVSLL-R-
XP_021817105.1/1-243 ----------------------GG--P-MQEMAHIA-KG-QD--P--G-N--CVSLL-R-
XP_022872559.1/1-230 ----------------------GG--P-MQEMAHIA-KG-QV--H--G-N--CVSLL-R-
XP_016575742.1/1-238 ----------------------GG--P-MQEMAHIA-KG-QD--H--G-N--CVSLL-R-
XP_017608938.1/1-232 ----------------------GG--P-MQEIAHIA-KG-QD--H--G-N--CVSLL-R-
XP_017608947.1/1-232 ----------------------GG--P-MQEIAHIA-KG-QD--H--G-N--CVSLL-R-
XP_010255501.1/1-245 ----------------------GG--P-MQEMAHIA-KG-QD--P--G-N--CVSLL-R-
XP_011023407.1/1-238 ----------------------GG--P-MQEMAHIA-KG-QD--H--G-N--CVSLL-R-
XP_011023404.1/1-238 ----------------------GG--P-MQEMAHIA-KG-QD--H--G-N--CVSLL-R-
XP_011023405.1/1-238 ----------------------GG--P-MQEMAHIA-KG-QD--H--G-N--CVSLL-R-
XP_019152252.1/1-243 ----------------------GG--P-MHEMAHIA-KG-QD--H--G-N--CVSLL-R-
XP_022870029.1/1-235 ----------------------GG--P-MQEMAHIA-KG-QD--H--G-N--CVSLL-R-
XP_017699285.1/1-225 ----------------------GG--P-MQEMAHIA-KG-QD--H--G-N--AVSLL-R-
XP_008795402.1/1-225 ----------------------GG--P-MQEMAHIA-KG-QD--H--G-N--AVSLL-R-
XP_010933869.1/1-224 ----------------------GG--P-MQEMAHIA-KG-QD--H--G-N--AVSLL-R-
XP_008799528.1/1-222 ----------------------GG--P-MQEMAHIA-KG-QD--H--G-N--AVSLL-R-
XP_010919252.1/1-223 ----------------------GG--P-MQEMAHIA-KG-QD--H--G-N--AVSLL-R-
XP_010919250.1/1-223 ----------------------GG--P-MQEMAHIA-KG-QD--H--G-N--AVSLL-R-
XP_007139955.1/1-229 ----------------------GG--P-MQEMAHIA-KG-QD--H--A-N--CVSLL-R-
XP_017418814.1/1-230 ----------------------GG--P-MQEMAHIA-KG-QD--H--A-N--CVSLL-R-
XP_014490274.1/1-230 ----------------------GG--P-MQEMAHIA-KG-QD--H--A-N--CVSLL-R-
XP_003534596.1/1-229 ----------------------GG--P-MQEMAHIA-KG-QD--H--A-N--CVSLL-R-
XP_006587871.1/1-229 ----------------------GG--P-MQEMAHIA-KG-QD--H--A-N--CVSLL-R-
XP_003552359.1/1-228 ----------------------GG--P-MQEMAHIA-KG-QD--H--A-N--CVSLL-R-
XP_015961616.1/1-228 ----------------------GG--P-MQEMAHIA-KG-QD--H--G-N--CVSLL-R-
XP_015961617.1/1-228 ----------------------GG--P-MQEMAHIA-KG-QD--H--G-N--CVSLL-R-
XP_025696060.1/1-228 ----------------------GG--P-MQEMAHIA-KG-QD--H--G-N--CVSLL-R-
XP_025696061.1/1-228 ----------------------GG--P-MQEMAHIA-KG-QD--H--G-N--CVSLL-R-
XP_016198931.1/1-228 ----------------------GG--P-MQEMAHIA-KG-QD--H--G-N--CVSLL-R-
XP_016198932.1/1-228 ----------------------GG--P-MQEMAHIA-KG-QD--H--G-N--CVSLL-R-
XP_025649261.1/1-228 ----------------------GG--P-MQEMAHIA-KG-QD--H--G-N--CVSLL-R-
XP_025649262.1/1-228 ----------------------GG--P-MQEMAHIA-KG-QD--H--G-N--CVSLL-R-
XP_021849370.1/1-228 ----------------------GG--P-MQEMAHIA-KG-QD--Q--G-N--CVSLL-R-
XP_021767325.1/1-228 ----------------------GG--P-MQEMAHIA-KG-QD--Q--G-N--CVSLL-R-
XP_021748881.1/1-228 ----------------------GG--P-MQEMAHIA-KG-QD--Q--G-N--CVSLL-R-
XP_019461451.1/1-231 ----------------------GG--P-MQEMAHIA-KG-QD--H--G-N--CVSLL-R-
XP_012574211.1/1-233 ----------------------GG--P-MQEMAHIA-KG-QD--H--G-N--CVSLL-R-
XP_004510857.1/1-233 ----------------------GG--P-MQEMAHIA-KG-QD--H--G-N--CVSLL-R-
XP_020237185.1/1-234 ----------------------GG--P-MQEMAHIA-KG-QD--H--G-N--CVSLL-R-
XP_020237261.1/1-234 ----------------------GG--P-MQEMAHIA-KG-QD--H--G-N--CVSLL-R-
XP_019456357.1/1-238 ----------------------GG--P-MQEMAHIA-KG-QD--H--G-N--CVSLL-R-
XP_013444922.1/1-235 ----------------------GG--P-MQEMAHIA-KG-QD--H--G-N--CVSLL-R-
XP_003528880.1/1-238 ----------------------GG--P-MQEMAHIA-KG-QD--H--G-N--AVSLL-R-
XP_006583340.1/1-238 ----------------------GG--P-MQEMAHIA-KG-QD--H--G-N--AVSLL-R-
XP_014516480.1/1-238 ----------------------GG--P-MQEMAHIA-KG-QD--H--G-N--CVSLL-R-
XP_014516483.1/1-238 ----------------------GG--P-MQEMAHIA-KG-QD--H--G-N--CVSLL-R-
XP_007134961.1/1-238 ----------------------GG--P-MQEMAHIA-KG-QD--H--G-N--CVSLL-R-
XP_010671720.1/1-229 ----------------------GG--P-MQEMAHIA-KG-QD--Q--G-N--CVSLL-R-
XP_012482732.1/1-232 ----------------------GG--P-MQEIAHIA-KG-QD--H--G-N--CVSLL-R-
XP_012482742.1/1-232 ----------------------GG--P-MQEIAHIA-KG-QD--H--G-N--CVSLL-R-
XP_016745638.1/1-232 ----------------------GG--P-MQEIAHIA-KG-QD--H--G-N--CVSLL-R-
XP_016745639.1/1-232 ----------------------GG--P-MQEIAHIA-KG-QD--H--G-N--CVSLL-R-
XP_010278578.1/1-242 ----------------------GG--P-MQEMAHIA-KG-QD--H--G-N--CVSLL-R-
XP_010278577.1/1-242 ----------------------GG--P-MQEMAHIA-KG-QD--H--G-N--CVSLL-R-
XP_006445143.1/1-238 ----------------------GG--P-MQEMAHIA-KG-QD--H--G-N--CVSLL-R-
XP_006445141.1/1-238 ----------------------GG--P-MQEMAHIA-KG-QD--H--G-N--CVSLL-R-
XP_022770361.1/1-238 ----------------------GG--P-MQEMAHIA-KG-QD--H--G-N--CVSLL-R-
XP_022757219.1/1-237 ----------------------GG--P-MQEMAHIA-KG-QD--H--G-N--CVSLL-R-
XP_022757220.1/1-237 ----------------------GG--P-MQEMAHIA-KG-QD--H--G-N--CVSLL-R-
NP_001314420.1/1-237 ----------------------GG--P-MQEMAHIA-KG-QD--H--G-N--CVSLL-R-
XP_016735217.1/1-237 ----------------------GG--P-MQEMAHIA-KG-QD--H--G-N--CVSLL-R-
XP_012437660.1/1-237 ----------------------GG--P-MQEMAHIA-KG-QD--H--G-N--CVSLL-R-
XP_012437661.1/1-237 ----------------------GG--P-MQEMAHIA-KG-QD--H--G-N--CVSLL-R-
XP_017615505.1/1-237 ----------------------GG--P-MQEMAHIA-KG-QD--H--G-N--CVSLL-R-
XP_017615506.1/1-237 ----------------------GG--P-MQEMAHIA-KG-QD--H--G-N--CVSLL-R-
XP_016723220.1/1-237 ----------------------GG--P-MQEMAHIA-KG-QD--H--G-N--CVSLL-R-
XP_016723229.1/1-237 ----------------------GG--P-MQEMAHIA-KG-QD--H--G-N--CVSLL-R-
XP_021904309.1/1-244 ----------------------GG--P-MQEMAHIA-KG-QD--H--G-N--CVSLL-R-
XP_021904311.1/1-244 ----------------------GG--P-MQEMAHIA-KG-QD--H--G-N--CVSLL-R-
XP_007051912.2/1-241 ----------------------GG--P-MQEMAHIA-KG-QD--H--G-N--CVSLL-R-
XP_007051913.2/1-241 ----------------------GG--P-MQEMAHIA-KG-QD--H--G-N--CVSLL-R-
XP_012489878.1/1-239 ----------------------GG--P-MQEMAHIA-KG-QD--H--G-N--CVSLL-R-
XP_016748231.1/1-239 ----------------------GG--P-MQEMAHIA-KG-QE--H--G-N--CVSLL-R-
XP_017631289.1/1-239 ----------------------GG--P-MQEMAHIA-KG-QD--H--G-N--CVSLL-R-
XP_023006686.1/1-236 ----------------------GG--P-MQEMAHIA-KG-QD--H--G-N--CVSLL-R-
XP_022958954.1/1-236 ----------------------GG--P-MQEMAHIA-KG-QD--H--G-N--CVSLL-R-
XP_023548045.1/1-236 ----------------------GG--P-MQEMAHIA-KG-QD--H--G-N--CVSLL-R-
XP_008460172.1/1-241 ----------------------GG--P-MQEMAHIA-KG-QD--H--G-N--CVSLL-R-
XP_004144982.1/1-241 ----------------------GG--P-MQEMAHIA-KG-QD--H--G-N--CVSLL-R-
XP_023514722.1/1-242 ----------------------GG--P-MQEMAHIA-KG-QD--H--G-N--CVSLL-R-
XP_022999814.1/1-242 ----------------------GG--P-MQEMAHIA-KG-QD--H--G-N--CVSLL-R-
XP_022964504.1/1-242 ----------------------GG--P-MQEMAHIA-KG-QD--H--G-N--CVSLL-R-
XP_010053664.1/1-245 ----------------------GG--P-MQEMAHIA-KG-QD--H--G-N--CVSLL-R-
XP_019189515.1/1-235 ----------------------GG--P-MQEMAHIA-KG-QD--H--G-N--CVSLL-R-
XP_023730775.1/1-234 ----------------------GG--P-MQEMAHIA-KG-QD--H--G-N--CVSLL-R-
XP_023730774.1/1-234 ----------------------GG--P-MQEMAHIA-KG-QD--H--G-N--CVSLL-R-
XP_022020543.1/1-230 ----------------------GG--P-MQEMAHIA-KG-QD--H--G-N--CVSLL-R-
XP_024962572.1/1-236 ----------------------GG--P-MQEMAHIA-KG-QD--H--G-N--CVSLL-R-
XP_022017696.1/1-236 ----------------------GG--P-MQEMAHIA-KG-QD--H--G-N--CVSLL-R-
XP_024018220.1/1-245 ----------------------GG--P-MQEMAHIA-KG-QD--H--G-N--CVSLL-R-
XP_011093166.1/1-234 ----------------------GG--P-MQEMAHIA-KG-QD--H--G-N--CVSLL-R-
XP_011093167.1/1-234 ----------------------GG--P-MQEMAHIA-KG-QD--H--G-N--CVSLL-R-
XP_006339457.1/1-238 ----------------------GG--P-MQEMAHIA-KG-QD--H--G-N--CVSLL-R-
XP_015059298.1/1-238 ----------------------GG--P-MQEMAHIA-KG-QD--H--G-N--CVSLL-R-
NP_001234657.2/1-238 ----------------------GG--P-MQEMAHIA-KG-QD--H--G-N--CVSLL-R-
XP_009602857.1/1-241 ----------------------GG--P-MQEMAHIA-KG-QD--H--G-N--CVSLL-R-
XP_009602856.1/1-241 ----------------------GG--P-MQEMAHIA-KG-QD--H--G-N--CVSLL-R-
XP_009782444.1/1-245 ----------------------GG--P-MQEMAHIA-KG-QD--H--G-N--CVSLL-R-
XP_009782443.1/1-245 ----------------------GG--P-MQEMAHIA-KG-QD--H--G-N--CVSLL-R-
XP_019259406.1/1-245 ----------------------GG--P-MQEMAHIA-KG-QD--H--G-N--CVSLL-R-
XP_015867375.1/1-242 ----------------------GG--P-MQEMAHIA-KG-QD--P--G-N--CVSLL-R-
XP_015867374.1/1-242 ----------------------GG--P-MQEMAHIA-KG-QD--P--G-N--CVSLL-R-
XP_015888672.1/1-242 ----------------------GG--P-MQEMAHIA-KG-QD--P--G-N--CVSLL-R-
XP_018810322.1/1-233 ----------------------GG--P-MQEMAHIA-KG-QD--H--G-N--CVSLL-R-
XP_018859074.1/1-233 ----------------------GG--P-MQEMAHIA-KG-QD--H--G-N--CVSLL-R-
XP_017231243.1/1-235 ----------------------GG--P-MQEMAHVA-KG-QD--H--G-N--CVSLL-R-
XP_017231244.1/1-235 ----------------------GG--P-MQEMAHVA-KG-QD--H--G-N--CVSLL-R-
XP_017231245.1/1-235 ----------------------GG--P-MQEMAHVA-KG-QD--H--G-N--CVSLL-R-
XP_017236322.1/1-235 ----------------------GG--P-MQEMAHIA-KG-QD--H--G-N--CVSLL-R-
XP_002301331.2/1-238 ----------------------GG--P-MQEMAHIA-KG-QD--H--G-N--CVSLL-R-
XP_024451706.1/1-238 ----------------------GG--P-MQEMAHIA-KG-QD--H--G-N--CVSLL-R-
XP_002320755.1/1-241 ----------------------GG--P-MQEMAHIA-KG-QD--H--G-N--CVSLL-R-
XP_011035097.1/1-241 ----------------------GG--P-MQEMAHIA-KG-QD--H--G-N--CVSLL-R-
XP_018813891.1/1-238 ----------------------GG--P-MQEMAHIA-KG-QD--H--G-N--CVSLL-R-
XP_012083470.1/1-239 ----------------------GG--P-MQEMAHIA-KG-QD--H--G-N--CVSLL-R-
XP_021651338.1/1-240 ----------------------GG--P-MQEMAHIA-KG-QD--H--G-N--CVSLL-R-
XP_021613538.1/1-241 ----------------------GG--P-MQEMAHIA-KG-QD--Q--G-N--CVSLL-R-
XP_021686862.1/1-241 ----------------------GG--P-MQEMAHIA-KG-QD--H--G-N--CVSLL-R-
XP_021686863.1/1-241 ----------------------GG--P-MQEMAHIA-KG-QD--H--G-N--CVSLL-R-
XP_021612685.1/1-241 ----------------------GG--P-MQEMAHIA-KG-QD--H--G-N--CVSLL-R-
XP_023879166.1/1-242 ----------------------GG--P-MQEMAHIA-KG-QD--H--G-N--CVSLL-R-
XP_002272264.2/1-234 ----------------------GG--P-MQEMAHIA-KG-QD--H--G-N--CVSLL-R-
XP_010661562.1/1-234 ----------------------GG--P-MQEMAHIA-KG-QD--H--G-N--CVSLL-R-
XP_002511801.1/1-237 ----------------------GG--P-MQEMAHIA-KG-QD--H--G-N--CVSLL-R-
XP_015584500.1/1-237 ----------------------GG--P-MQEMAHIA-KG-QD--H--G-N--CVSLL-R-
XP_022141257.1/1-238 ----------------------GR--P-MLEMLHIS-KG-QG--L--D-N--RVSLL-R-
XP_023885612.1/1-244 ----------------------GR--P-IQEMIHIA-KG-QK--Q--G-N--CVSLL-R-
XP_023885611.1/1-244 ----------------------GR--P-IQEMIHIA-KG-QK--Q--G-N--CVSLL-R-
XP_018830842.1/1-239 ----------------------GR--P-IQEMVHIA-KG-HK--Q--G-N--CVSLL-R-
XP_018830843.1/1-239 ----------------------GR--P-IQEMVHIA-KG-HK--Q--G-N--CVSLL-R-
XP_018830840.1/1-239 ----------------------GR--P-IQEMVHIA-KG-HK--Q--G-N--CVSLL-R-
XP_018830841.1/1-239 ----------------------GR--P-IQEMVHIA-KG-HK--Q--G-N--CVSLL-R-
XP_012090293.1/1-240 ----------------------GG--L-MQEMVHIT-KG-NS--S--G-N--CVSLL-R-
XP_012090292.1/1-240 ----------------------GG--L-MQEMVHIT-KG-NS--S--G-N--CVSLL-R-
XP_021614581.1/1-239 ----------------------GG--M-MQEMVHIS-KG-HN--R--G-N--CVSLL-R-
XP_021614580.1/1-239 ----------------------GG--M-MQEMVHIS-KG-HN--R--G-N--CVSLL-R-
XP_015581247.1/1-240 ----------------------GG--M-LQEMVHIS-KS-HS--R--A-N--CVSLL-R-
XP_002510822.1/1-240 ----------------------GG--M-LQEMVHIS-KS-HS--R--A-N--CVSLL-R-
XP_021648646.1/1-240 ----------------------GG--M-MQEMVHIS-KG-HS--G--G-N--CVSLL-R-
XP_021634593.1/1-234 ----------------------GG--M-MQEMVHIS-KG-HS--R--G-N--CVSLL-R-
XP_021634592.1/1-234 ----------------------GG--M-MQEMVHIS-KG-HS--R--G-N--CVSLL-R-
XP_006490348.1/1-238 ----------------------GG--P-LQEMVHIA-KG-QA--P--G-N--CASLL-R-
XP_006490346.1/1-238 ----------------------GG--P-LQEMVHIA-KG-QA--P--G-N--CASLL-R-
XP_024958654.1/1-238 ----------------------GG--P-LQEMVHIA-KG-QA--P--G-N--CASLL-R-
XP_006490345.1/1-238 ----------------------GG--P-LQEMVHIA-KG-QA--P--G-N--CASLL-R-
XP_006421879.2/1-238 ----------------------GG--P-LQEMVHIA-KG-QA--P--G-N--CASLL-R-
XP_021892212.1/1-221 ----------------------GG--P-MQEMLRIA-KG-EE--R--G-N--CVSLM-R-
XP_021892211.1/1-221 ----------------------GG--P-MQEMLRIA-KG-EE--R--G-N--CVSLM-R-
XP_021892210.1/1-221 ----------------------GG--P-MQEMLRIA-KG-EE--R--G-N--CVSLM-R-
XP_022765148.1/1-244 ----------------------GG--P-MQEMVKVA-KG-RG--H--G-N--YVSLL-R-
XP_022765158.1/1-244 ----------------------GG--P-MQEMVKVA-KG-RG--H--G-N--YVSLL-R-
XP_022765120.1/1-244 ----------------------GG--P-MQEMVKVA-KG-RG--H--G-N--YVSLL-R-
XP_022765114.1/1-244 ----------------------GG--P-MQEMVKVA-KG-RG--H--G-N--YVSLL-R-
XP_022736693.1/1-237 ----------------------GG--P-MQEVVNVA-KG-PQ--Q--G-N--CVSLV-R-
XP_022736692.1/1-237 ----------------------GG--P-MQEVVNVA-KG-PQ--Q--G-N--CVSLV-R-
XP_022736689.1/1-237 ----------------------GG--P-MQEVVNVA-KG-PQ--Q--G-N--CVSLV-R-
XP_022734775.1/1-244 ----------------------GG--P-MQEMVNVA-KG-QG--H--G-N--CVSLL-H-
XP_016743353.1/1-244 ----------------------GG--P-MQEMVHVA-KG-MG--H--G-N--CVSLL-R-
XP_012474605.1/1-244 ----------------------GG--P-MQEMVHVA-KG-MG--H--G-N--CVSLL-R-
XP_012474606.1/1-244 ----------------------GG--P-MQEMVHVA-KG-MG--H--G-N--CVSLL-R-
XP_016743352.1/1-244 ----------------------GG--P-MQEMVHVA-KG-MG--H--G-N--CVSLL-R-
XP_016743351.1/1-244 ----------------------GG--P-MQEMVHVA-KG-MG--H--G-N--CVSLL-R-
XP_012474604.1/1-244 ----------------------GG--P-MQEMVHVA-KG-MG--H--G-N--CVSLL-R-
XP_012474603.1/1-244 ----------------------GG--P-MQEMVHVA-KG-MG--H--G-N--CVSLL-R-
XP_017646297.1/1-244 ----------------------GG--P-MQEMVHVA-KG-MG--H--G-N--CVSLL-R-
XP_016736315.1/1-244 ----------------------GG--P-MQEMVHVA-KG-MG--H--G-N--CVSLL-R-
XP_017646296.1/1-244 ----------------------GG--P-MQEMVHVA-KG-MG--H--G-N--CVSLL-R-
XP_017646298.1/1-244 ----------------------GG--P-MQEMVHVA-KG-MG--H--G-N--CVSLL-R-
XP_016736306.1/1-244 ----------------------GG--P-MQEMVHVA-KG-MG--H--G-N--CVSLL-R-
XP_017646295.1/1-244 ----------------------GG--P-MQEMVHVA-KG-MG--H--G-N--CVSLL-R-
XP_017972896.1/1-244 ----------------------GG--P-MQGMVKIA-KG-PG--H--G-N--CVSLL-R-
XP_017972895.1/1-244 ----------------------GG--P-MQGMVKIA-KG-PG--H--G-N--CVSLL-R-
XP_017972893.1/1-244 ----------------------GG--P-MQGMVKIA-KG-PG--H--G-N--CVSLL-R-
XP_021274823.1/1-244 ----------------------GG--P-MQEMVKIA-KG-PG--H--G-N--CVSLL-R-
XP_024536343.1/1-231 ----------------------GG--S-VQEMAHIA-KG-HD--P--G-N--VISLL-R-
XP_006366174.1/1-244 ----------------------GG--P-MQEMAHIA-KG-QD--H--G-N--CVSLL-R-
XP_020219711.1/1-212 ----------------------GG--P-MQEMAHIA-KG-QD--H--A-N--CVSLL-R-
XP_022897056.1/1-222 ----------------------GG--P-MQEMAHIA-KG-QD--H--G-N--CVSLL-R-
NP_001281015.1/1-243 ----------------------GG--P-MQEMAHIA-KG-QD--P--G-N--CVSLL-R-
XP_008383260.1/1-243 ----------------------GG--P-MQEMAHIA-KG-QD--P--G-N--CVSLL-R-
XP_022896229.1/1-235 ----------------------GG--P-MQEMAHIA-KG-QD--H--G-N--CVSLL-R-
XP_022896230.1/1-235 ----------------------GG--P-MQEMAHIA-KG-QD--H--G-N--CVSLL-R-
XP_025816451.1/1-225 ----------------------GG--P-MQEMANIA-KGQ-E--H--G-N--SVSLL-R-
XP_004968001.1/1-225 ----------------------GG--P-MQEMANIA-KGQ-E--H--G-N--SVSLL-R-
XP_004968002.1/1-225 ----------------------GG--P-MQEMANIA-KGQ-E--H--G-N--SVSLL-R-
XP_002457484.1/1-226 ----------------------GG--P-MQEMANIA-KGQ-E--H--G-N--SVSLL-R-
XP_009400446.1/1-224 ----------------------GG--P-MQEMAHIA-KGQDT--T--G-N--AVTLL-R-
XP_018676535.1/1-225 ----------------------GG--P-MQEIAHIA-KGQN---T--G-N--AVSLL-R-
XP_009401771.1/1-232 ----------------------GG--P-MQEMAHIA-KGQN---T--G-N--AVSLL-R-
XP_009384534.1/1-228 ----------------------GG--P-MQEMAHIA-KGQN---T--G-N--AVSLL-R-
XP_020672447.1/1-217 ----------------------GG--P-MREMAHIA-KG-QD--Q--G-N--AVSLL-R-
XP_020582116.1/1-218 ----------------------GG--P-MREMAHIA-KGLQD--Q--G-N--AVSLL-C-
XP_020675647.1/1-231 ----------------------GG--P-MQEMAHIA-KG-QD--E--G-N--AVSLL-R-
XP_020675648.1/1-231 ----------------------GG--P-MQEMAHIA-KG-QD--E--G-N--AVSLL-R-
XP_020585632.1/1-228 ----------------------GG--P-MQEMAHIA-KG-QD--Q--G-N--AVSLL-R-
XP_016176402.1/1-228 ----------------------GG--P-MQEMVHIA-KG-QG--H--G-N--CVSLL-R-
XP_016176403.1/1-228 ----------------------GG--P-MQEMVHIA-KG-QG--H--G-N--CVSLL-R-
XP_016176400.1/1-228 ----------------------GG--P-MQEMVHIA-KG-QG--H--G-N--CVSLL-R-
XP_016176401.1/1-228 ----------------------GG--P-MQEMVHIA-KG-QG--H--G-N--CVSLL-R-
XP_010523042.1/1-245 ----------------------GG--P-MQEMAHIA-KG-QD--E--G-N--SVCLL-R-
XP_010523041.1/1-240 ----------------------GG--P-MQEMAHIA-KG-QD--E--G-N--SVCLL-R-
XP_020878511.1/1-224 ----------------------GG--P-MQEMAHIA-KG-QD--Q--G-----VCLL-R-
NP_567183.2/1-228    ----------------------GG--P-MQEMAHIT-KG-QD--Q--G-----VSLL-R-
XP_006287095.1/1-228 ----------------------GG--P-MQEMAHIA-KG-QD--Q--G-----VSLL-R-
XP_010456250.1/1-228 ----------------------GG--P-MQEMAHIA-KG-QD--Q--G-----VSLL-R-
XP_010419627.1/1-228 ----------------------GG--P-MQEMAHIA-KG-QD--Q--G-----VSLL-R-
XP_010427345.1/1-228 ----------------------GG--P-MQEMAHIA-KG-QD--Q--G-----VSLL-R-
XP_009111469.1/1-229 ----------------------GG--P-MQEMAHIA-KG-QD--Q--G-N--SVSLL-R-
XP_018487039.1/1-229 ----------------------GG--P-MQEMAHIA-KG-QD--Q--G-N--SVSLL-R-
XP_018453129.1/1-229 ----------------------GG--P-MQEMAHIA-KG-QD--Q--G-N--SVSLL-R-
XP_013612299.1/1-229 ----------------------GG--P-MQEMAHIA-KG-QD--Q--G-N--SVSLL-R-
XP_006396268.1/1-225 ----------------------GG--P-MQEMVHIA-KG-QD--Q--G-N--SVSLL-R-
XP_018475317.1/1-221 ----------------------GG--P-MQEMAHIA-KG-QD--Q--G-N--SVSLL-R-
XP_013629750.1/1-221 ----------------------GG--P-MQEMAHIA-KG-QD--Q--G-N--SVSLL-R-
XP_009134533.1/1-221 ----------------------GG--P-MQEMAHIA-KG-QD--Q--G-N--SVSLL-R-
XP_020531377.1/1-244 ----------------------GG--P-MQEMAHIA-KG-QD--H--G-N--CVSLL-R-
XP_006857866.1/1-244 ----------------------GG--P-MQEMAHIA-KG-QD--H--G-N--CVSLL-R-
XP_018434263.1/1-219 ----------------------GG--P-MQEIANIF-KG-QD--H--S-N--SVSLL-R-
XP_018434260.1/1-219 ----------------------GG--P-MQEIANIF-KG-QD--H--S-N--SVSLL-R-
XP_018434262.1/1-219 ----------------------GG--P-MQEIANIF-KG-QD--H--S-N--SVSLL-R-
XP_013688926.1/1-219 ----------------------GG--P-MQEIANIF-KG-QD--H--S-N--SVSLL-R-
XP_013637558.1/1-219 ----------------------GG--P-MQEIANIF-KG-QD--H--S-N--SVSLL-R-
XP_013637556.1/1-219 ----------------------GG--P-MQEIANIF-KG-QD--H--S-N--SVSLL-R-
XP_013637557.1/1-219 ----------------------GG--P-MQEIANIF-KG-QD--H--S-N--SVSLL-R-
XP_009143168.1/1-219 ----------------------GG--P-MQEIANIF-KG-QD--H--S-N--SVSLL-R-
XP_009143167.1/1-219 ----------------------GG--P-MQEIANIF-KG-QD--H--S-N--SVSLL-R-
XP_009143166.1/1-219 ----------------------GG--P-MQEIANIF-KG-QD--H--S-N--SVSLL-R-
XP_013692686.1/1-219 ----------------------GG--P-MQEIANIF-KG-QD--Y--S-N--SVSLL-R-
XP_022574820.1/1-219 ----------------------GG--P-MQEIANIF-KG-QD--Y--S-N--SVSLL-R-
XP_013692684.1/1-219 ----------------------GG--P-MQEIANIF-KG-QD--Y--S-N--SVSLL-R-
XP_013748127.1/1-219 ----------------------GG--P-MQEIANIF-KG-QD--Y--S-N--SVSLL-R-
XP_013692685.1/1-219 ----------------------GG--P-MQEIANIF-KG-QD--Y--S-N--SVSLL-R-
XP_013748124.1/1-219 ----------------------GG--P-MQEIANIF-KG-QD--Y--S-N--SVSLL-R-
XP_013748126.1/1-219 ----------------------GG--P-MQEIANIF-KG-QD--Y--S-N--SVSLL-R-
XP_022574821.1/1-219 ----------------------GG--P-MQEIANIF-KG-QD--Y--S-N--SVSLL-R-
XP_006290613.1/1-229 ----------------------GG--P-MKEMAHIA-KG-HD--H--S-N--SVSLL-R-
XP_013592301.1/1-230 ----------------------GG--P-MQEMAHIA-KG-HD--H--S-N--SISLL-R-
XP_018442581.1/1-230 ----------------------GG--P-MQEMAHIA-KG-HD--H--S-N--SVSLL-R-
XP_013750213.1/1-232 ----------------------GG--P-MQEMAHIA-KG-HD--H--S-N--SVSLL-R-
XP_013648430.1/1-232 ----------------------GG--P-MQEMAHIA-KG-HD--H--S-N--SVSLL-R-
XP_009104292.1/1-232 ----------------------GG--P-MQEMAHIA-KG-HD--H--S-N--SVSLL-R-
XP_019058514.1/1-223 ----------------------GG--P-MQEMAHIA-KG-QD--H--S-N--CVSLL-R-
XP_010523728.1/1-220 ----------------------GG--P-MQEMAHVA-KG-QD--L--S-N--CVSLL-R-
XP_024514927.1/1-242 ----------------------GG--A-VQEIARVA-TG-QD--P--G-C--VVSLL-R-
XP_002985242.1/1-242 ----------------------GG--A-VQEIARVA-TG-QD--P--G-C--VVSLL-R-
XP_024530500.1/1-242 ----------------------GG--A-VQEIARVA-TG-QD--P--G-C--VVSLL-R-
XP_024530499.1/1-242 ----------------------GG--A-VQEIARVA-TG-QD--P--G-C--VVSLL-R-
XP_024530501.1/1-242 ----------------------GG--A-VQEIARVA-TG-QD--P--G-C--VVSLL-R-
XP_010107411.1/1-242 ----------------------GG--P-MQEMVHIT-KC-QR--H--G-N--HVSLL-R-
XP_024028635.1/1-242 ----------------------GG--P-MQEMVHIT-KC-QR--H--G-N--HVSLL-R-
XP_015877999.1/1-244 ----------------------GG--P-MQEMIHIA-KG-QG--H--G-N--CVSLL-R-
XP_019427294.1/1-247 ----------------------GG--P-MQEMVHIA-KG-QG--H--G-N--CVSLL-R-
XP_019427292.1/1-247 ----------------------GG--P-MQEMVHIA-KG-QG--H--G-N--CVSLL-R-
XP_019452814.1/1-239 ----------------------GG--P-MQEMVHIA-KG-QG--H--G-N--CVSLL-R-
XP_019452815.1/1-239 ----------------------GG--P-MQEMVHIA-KG-QG--H--G-N--CVSLL-R-
XP_003592295.2/1-235 ----------------------GG--P-MQEMVHIA-KG-QG--Q--G-N--CVSLL-R-
XP_024625328.1/1-235 ----------------------GG--P-MQEMVHIA-KG-QG--Q--G-N--CVSLL-R-
XP_012570175.1/1-236 ----------------------GG--P-MQEMVHIA-KG-QG--Q--G-N--CVSLL-R-
XP_012570176.1/1-236 ----------------------GG--P-MQEMVHIA-KG-QG--Q--G-N--CVSLL-R-
XP_004496593.1/1-236 ----------------------GG--P-MQEMVHIA-KG-QG--Q--G-N--CVSLL-R-
XP_025620154.1/1-234 ----------------------GG--P-MQEMVHIA-KG-QG--H--G-N--CVSLL-R-
XP_015941562.1/1-234 ----------------------GG--P-MQEMVHIA-KG-QG--H--G-N--CVSLL-R-
XP_025620153.1/1-234 ----------------------GG--P-MQEMVHIA-KG-QG--H--G-N--CVSLL-R-
XP_020239673.1/1-232 ----------------------GG--P-MQEMVHIA-KG-QG--H--G-N--CVSLL-R-
XP_020239648.1/1-232 ----------------------GG--P-MQEMVHIA-KG-QG--H--G-N--CVSLL-R-
XP_007143377.1/1-235 ----------------------GG--P-MQEMVHIA-KG-QG--H--G-N--CVSLL-R-
XP_007143378.1/1-235 ----------------------GG--P-MQEMVHIA-KG-QG--H--G-N--CVSLL-R-
XP_017415467.1/1-234 ----------------------GG--P-MQEMVHIA-KG-QG--H--G-N--CVSLL-R-
XP_017415466.1/1-234 ----------------------GG--P-MQEMVHIA-KG-QG--H--G-N--CVSLL-R-
XP_014514421.1/1-236 ----------------------GG--P-MQEMVHIA-KG-QG--H--G-N--CVSLL-R-
XP_014514419.1/1-236 ----------------------GG--P-MQEMVHIA-KG-QG--H--G-N--CVSLL-R-
XP_006606097.1/1-235 ----------------------GG--P-MQEMVHIA-KG-QG--H--G-N--CVSLL-R-
XP_006606096.1/1-235 ----------------------GG--P-MQEMVHIA-KG-QG--H--G-N--CVSLL-R-
XP_003536477.1/1-234 ----------------------GG--P-MQEMVHIA-KG-QG--H--G-N--CVSLL-R-
XP_006589557.1/1-234 ----------------------GG--P-MQEMVHIA-KG-QG--H--G-N--CVSLL-R-
XP_006402516.1/1-230 ----------------------GG--P-MQEMAHIA-KG-HD--H--S-N--SVSLL-R-
NP_191674.1/1-237    ----------------------GG--P-MKEMAHIA-KG-HD--R--S-N--SVSLL-R-
XP_002876596.1/1-239 ----------------------GG--P-MKEMAHIA-KG-HD--H--S-N--SVSLL-R-
XP_010413451.1/1-238 ----------------------GG--P-MKEMAHIA-KG-HD--H--S-N--SVSLL-R-
XP_010413452.1/1-238 ----------------------GG--P-MKEMAHIA-KG-HD--H--S-N--SVSLL-R-
XP_010413453.1/1-238 ----------------------GG--P-MKEMAHIA-KG-HD--H--S-N--SVSLL-R-
XP_010469036.1/1-242 ----------------------GG--P-MKEMAHIA-KG-HD--H--S-N--SVSLL-R-
XP_010469037.1/1-242 ----------------------GG--P-MKEMAHIA-KG-HD--H--S-N--SVSLL-R-
XP_010469038.1/1-242 ----------------------GG--P-MKEMAHIA-KG-HD--H--S-N--SVSLL-R-
XP_010512448.1/1-238 ----------------------GG--P-MKEMAHIA-KG-HD--H--S-N--SVSLL-R-
XP_010512449.1/1-238 ----------------------GG--P-MKEMAHIA-KG-HD--H--S-N--SVSLL-R-
XP_006397757.1/1-219 ----------------------GG--P-MQEIANIF-KG-QD--H--S-N--SVSLL-R-
XP_024010837.1/1-220 ----------------------GG--P-MQEIANIF-KG-QD--H--S-N--SVSLL-R-
XP_023640300.1/1-220 ----------------------GG--P-MQEIANIF-KG-QD--Q--S-N--SVSLL-R-
XP_006293718.1/1-220 ----------------------GG--P-MQEIANIF-KG-QD--Q--S-N--SVSLL-R-
XP_010506553.1/1-219 ----------------------GG--P-MQEIANIF-KG-QD--Q--S-N--SVSLL-R-
XP_010508114.1/1-219 ----------------------GG--P-MQEIANIF-KG-QD--Q--S-N--SVSLL-R-
XP_010508115.1/1-219 ----------------------GG--P-MQEIANIF-KG-QD--Q--S-N--SVSLL-R-
XP_022147276.1/1-219 ----------------------GG--P-MQEMLHIP-KA-QH--H--A-N--SVSLL-R-
XP_023527464.1/1-222 ----------------------GG--P-MQEMLHIP-KA-HHHHH--A-N--AVSLL-R-
XP_022980233.1/1-222 ----------------------GG--P-MQEMLHIP-KA-HHHHH--A-N--AVSLL-R-
XP_022980231.1/1-222 ----------------------GG--P-MQEMLHIP-KA-HHHHH--A-N--AVSLL-R-
XP_022924366.1/1-223 ----------------------GG--P-MQEMLHIP-KA-HHHHH--A-N--AVSLL-R-
XP_022924364.1/1-223 ----------------------GG--P-MQEMLHIP-KA-HHHHH--A-N--AVSLL-R-
XP_023527467.1/1-221 ----------------------GG--P-MQEMLHIP-KA-HHHHH--A-N--AVSLL-R-
XP_023527463.1/1-221 ----------------------GG--P-MQEMLHIP-KA-HHHHH--A-N--AVSLL-R-
XP_023527465.1/1-221 ----------------------GG--P-MQEMLHIP-KA-HHHHH--A-N--AVSLL-R-
XP_022980232.1/1-221 ----------------------GG--P-MQEMLHIP-KA-HHHHH--A-N--AVSLL-R-
XP_022924365.1/1-222 ----------------------GG--P-MQEMLHIP-KA-HHHHH--A-N--AVSLL-R-
XP_009412599.1/1-224 ----------------------GA--P-MYEMAHIA-KG-QD--A--G-N--AVSLL-R-
XP_018677084.1/1-231 ----------------------GG--P-MEEMAHIA-NG-KE--T--G-N--TVSLL-R-
XP_020114860.1/1-246 ----------------------GG--P-MQEMAHIA-KG-QH--H--G-N--AVSLL-R-
XP_020114857.1/1-246 ----------------------GG--P-MQEMAHIA-KG-QH--H--G-N--AVSLL-R-
XP_020114859.1/1-246 ----------------------GG--P-MQEMAHIA-KG-QH--H--G-N--AVSLL-R-
XP_020084803.1/1-234 ----------------------GG--P-MHQMAHIP-KG-HH--H--G-N--AVSLL-R-
XP_015625616.1/1-226 ----------------------GG--P-MQEMTQIA-KG-QR--D--G-N--SVSLL-R-
XP_006647677.1/1-226 ----------------------GG--P-MQEMTQIA-KG-QR--D--G-N--SVSLL-R-
NP_001105493.1/1-226 ----------------------GG--P-MQEMANIA-KG-QE--H--G-N--SVSLL-R-
XP_020178635.1/1-225 ----------------------GG--P-MQEMARIA-KG-HQ--N--G-N--SVSLL-R-
XP_003570085.1/1-223 ----------------------GG--P-MQEMTRIA-KG-HQ--N--G-N--SVSLL-R-
XP_020247871.1/1-226 ----------------------GS--S-YHEVAHIA-NG-S--YQ--K-N--CVSLL-R-
XP_020247870.1/1-226 ----------------------GS--S-YHEVAHIA-NG-S--YQ--K-N--CVSLL-R-
XP_024004764.1/1-211 ----------------------GN--T-LQEVSHIA-NG-S--HL--R-N--CISLL-RN
XP_018449374.1/1-217 ----------------------EN--P-FQEVAHIA-NG-S--HP--G-N--CISLL-H-
XP_013602839.1/1-215 ----------------------EN--P-FQEVAHIA-NG-S--HP--G-N--CISLL-H-
XP_013655966.1/1-215 ----------------------EN--P-FQEVAHIA-NG-S--HP--G-N--CISLL-H-
XP_009108342.1/1-215 ----------------------EN--P-FQEVAHIE-NG-S--HP--G-N--CISLL-H-
XP_013655964.1/1-215 ----------------------EN--P-FQEVAHIA-NG-S--HP--G-N--CISLL-H-
XP_020872468.1/1-222 ----------------------RS--S-FQEVAHIA-NG-S--HP--G-N--CISLL-Q-
NP_193506.2/1-221    ----------------------GS--S-FQEVAHIA-NG-S--HL--G-N--SISLL-R-
XP_023633855.1/1-222 ----------------------GS--S-FQEVAHIA-NG-S--HP--G-N--CISLL-R-
XP_010449572.1/1-221 ----------------------GS--S-FQEVAHIA-NG-S--HL--G-N--CISLL-R-
XP_010439957.1/1-223 ----------------------GS--S-FQEVAHIA-NG-S--HP--G-N--NTSLL-R-
XP_010439959.1/1-223 ----------------------GS--S-FQEVAHIA-NG-S--HP--G-N--NTSLL-R-
XP_010439958.1/1-223 ----------------------GS--S-FQEVAHIA-NG-S--HP--G-N--NTSLL-R-
XP_006854056.1/1-234 ----------------------GS--A-SNEVAHIA-NG-T--HT--G-N--CISLY-R-
XP_020529072.1/1-234 ----------------------GS--A-SNEVAHIA-NG-T--HT--G-N--CISLY-R-
NP_199499.3/1-224    ----------------------GN--S-PHEVAHIA-NG-S--HP--G-N--CISLL-R-
XP_020871346.1/1-224 ----------------------GN--S-PHEVAHIA-NG-S--HP--G-N--CISLL-R-
XP_006281890.1/1-224 ----------------------GN--S-PHEVAHIA-NG-S--HP--G-N--CISLL-R-
XP_010481400.1/1-224 ----------------------GN--S-PHEVAHIA-NG-S--HP--G-N--CISLL-R-
XP_010494885.1/1-224 ----------------------GN--S-PHEVAHIA-NG-S--HP--G-N--CISLL-R-
XP_018455882.1/1-227 ----------------------GN--S-PHEVAHIA-NG-S--HP--G-N--CISLL-R-
XP_013713146.1/1-227 ----------------------GN--S-PHEVAHIA-NG-S--HP--G-N--CISLL-R-
XP_013607209.1/1-227 ----------------------GN--S-PHEVAHIA-NG-S--HP--G-N--CISLL-R-
XP_009114317.1/1-227 ----------------------GN--S-SHEVAHIA-NG-S--HP--G-N--CISLL-R-
XP_013661958.1/1-227 ----------------------GN--S-SHEVAHIA-NG-S--HP--G-N--CISLL-R-
XP_006398384.1/1-227 ----------------------GN--S-PHEIAHIA-NG-S--HP--G-N--CISLL-R-
XP_024011152.1/1-227 ----------------------GN--S-PHEIAHIA-NG-S--HP--G-N--CISLL-R-
XP_018482944.1/1-227 ----------------------GS--S-PHEVAHIA-NG-S--QP--G-N--CISLL-R-
XP_009101472.1/1-227 ----------------------GS--S-PHEVAHIA-NG-S--HP--G-N--CISLL-R-
XP_013693979.1/1-227 ----------------------GS--S-PHEVAHIA-NG-S--HP--G-N--CISLL-R-
XP_013615089.1/1-227 ----------------------GS--S-PHEVAHIA-NG-S--HP--G-N--CISLL-R-
XP_013704013.1/1-227 ----------------------GS--S-PHEVAHIA-NG-S--HP--G-N--CISLL-R-
XP_009373477.1/1-246 ----------------------GN--S-LHEVAHIA-NG-S--HP--G-N--CISLL-R-
XP_006662631.2/1-256 ----------------------GG--S-LHEVAHIA-NG-S--HP--R-N--CISLL-R-
XP_015613461.1/1-259 ----------------------GG--S-LHEVAHIA-NG-S--HP--R-N--CISLL-R-
NP_001337805.1/1-240 ----------------------GG--S-LHEVAHIA-NG-S--HP--K-N--CISLL-R-
XP_020163828.1/1-239 ----------------------GG--S-LHEVAHIA-NG-S--HP--R-N--CISLL-R-
XP_010235052.1/1-238 ----------------------GS--S-LQEVAHIA-NG-S--HP--R-N--CISLL-R-
XP_025798256.1/1-238 ----------------------GG--S-LHEVAHIA-NG-S--HP--R-N--CISLL-R-
XP_004983555.1/1-238 ----------------------GG--S-LHEVAHIA-NG-S--HP--R-N--CISLL-R-
XP_002464880.2/1-238 ----------------------GG--S-LHEVAHIA-NG-S--HP--R-N--CISLL-R-
XP_020262583.1/1-231 ----------------------GN--S-LHEVAHIA-NG-S--HR--R-N--CVSLL-R-
XP_020677410.1/1-231 ----------------------GN--S-LHEVAHIA-NG-S--HP--R-N--CVSLL-R-
XP_020577604.1/1-229 ----------------------GN--S-LHEIDHIA-NG-S--HP--K-N--CVSLL-R-
XP_020577606.1/1-229 ----------------------GN--S-LHEIDHIA-NG-S--HP--K-N--CVSLL-R-
XP_020577607.1/1-229 ----------------------GN--S-LHEIDHIA-NG-S--HP--K-N--CVSLL-R-
XP_019709148.1/1-245 ----------------------GN--S-FHEVAHIA-NG-S--HP--R-N--CVSLL-R-
XP_008803567.1/1-241 ----------------------GN--S-FHEVAHIA-NG-L--HP--R-N--CVTLL-R-
XP_008803570.2/1-241 ----------------------GN--S-FHEVAHIA-NG-L--HP--R-N--CVTLL-R-
XP_018681950.1/1-231 ----------------------GN--S-LHEVAHIA-NG-S--HP--R-N--CISLL-R-
XP_018681951.1/1-231 ----------------------GN--S-LHEVAHIA-NG-S--HP--R-N--CISLL-R-
XP_010919976.1/1-237 ----------------------GN--S-YHEVAHIA-NG-S--HP--R-N--CVSLL-R-
XP_008800084.1/1-237 ----------------------GN--S-YHEVAHIA-NG-S--HP--R-N--CVSLL-R-
XP_018679997.1/1-229 ----------------------GN--S-LHEVAHIA-NG-S--HP--R-N--CVSLL-R-
XP_009397149.1/1-229 ----------------------GN--S-LHEVAHIA-NG-S--HP--R-N--CVSLL-R-
XP_024995523.1/1-235 ----------------------GN--P-LQEVAHIA-NG-S--HP--G-N--CISLL-R-
XP_023736252.1/1-233 ----------------------GI--P-LQEVAHIA-NG-S--HP--G-N--SISLL-R-
XP_023736253.1/1-233 ----------------------GI--P-LQEVAHIA-NG-S--HP--G-N--SISLL-R-
XP_022022041.1/1-231 ----------------------GN--P-LEEVAHIA-NG-S--HP--G-N--CISLL-R-
XP_021969857.1/1-226 ----------------------GS--P-LEEVAHIA-NG-S--HP--G-N--CISLL-R-
XP_010245213.1/1-236 ----------------------GN--S-LHEVAHIA-NG-S--HP--G-N--CISLL-R-
XP_010245210.1/1-236 ----------------------GN--S-LHEVAHIA-NG-S--HP--G-N--CISLL-R-
XP_010276832.1/1-235 ----------------------GN--S-LHEVAHIA-NG-S--HP--G-N--CISLL-R-
XP_010276837.1/1-235 ----------------------GN--S-LHEVAHIA-NG-S--HP--G-N--CISLL-R-
XP_019155823.1/1-230 ----------------------GN--S-LHEVAHIA-NG-S--HP--G-N--CISLL-R-
XP_022881662.1/1-238 ----------------------GN--P-LHEVAHIA-NG-S--HP--G-N--CISLF-R-
XP_022877113.1/1-234 ----------------------GN--S-LHEVAHIA-NG-S--HP--G-N--SVSLL-R-
XP_016450789.1/1-246 ----------------------GN--S-LHEVAHIA-NG-S--HP--G-N--CISLL-R-
XP_009597691.1/1-246 ----------------------GN--S-LHEVAHIA-NG-S--HP--G-N--CISLL-R-
XP_009786380.1/1-246 ----------------------GN--S-LHEVAHIA-NG-S--HP--G-N--CISLL-R-
XP_016494823.1/1-246 ----------------------GN--S-LHEVAHIA-NG-S--HP--G-N--CISLL-R-
XP_016494822.1/1-246 ----------------------GN--S-LHEVAHIA-NG-S--HP--G-N--CISLL-R-
XP_019230103.1/1-246 ----------------------GN--S-LHEVAHIA-NG-S--HP--G-N--CISLL-R-
XP_016573090.1/1-250 ----------------------GN--S-LHEVAHIA-NG-S--HP--G-N--CISLL-R-
XP_016573102.1/1-250 ----------------------GN--S-LHEVAHIA-NG-S--HP--G-N--CISLL-R-
XP_006352911.1/1-250 ----------------------GN--S-LHEVAHIA-NG-S--HP--G-N--CISLL-R-
XP_015166604.1/1-250 ----------------------GN--S-LHEVAHIA-NG-S--HP--G-N--CISLL-R-
XP_015085651.1/1-250 ----------------------GN--S-LHEVAHIA-NG-S--HP--G-N--CISLL-R-
XP_015085654.1/1-250 ----------------------GN--S-LHEVAHIA-NG-S--HP--G-N--CISLL-R-
XP_004245919.1/1-250 ----------------------GN--S-LHEVAHIA-NG-S--HP--G-N--CISLL-R-
XP_025888165.1/1-250 ----------------------GN--S-LHEVAHIA-NG-S--HP--G-N--CISLL-R-
XP_011070327.1/1-236 ----------------------GN--S-LHEVAHIA-NG-S--HP--G-N--CISLL-R-
XP_011070329.1/1-236 ----------------------GN--S-LHEVAHIA-NG-S--HP--G-N--CISLL-R-
XP_012843107.1/1-236 ----------------------GN--S-LHEVAHIA-NG-S--NP--G-N--CISLL-R-
XP_019423575.1/1-234 ----------------------GN--S-LNEVAHIA-NG-S--HP--G-N--CISLL-R-
XP_019423571.1/1-234 ----------------------GN--S-LNEVAHIA-NG-S--HP--G-N--CISLL-R-
XP_019435331.1/1-234 ----------------------GN--S-LNEVAHIA-NG-S--HP--G-N--CISLL-R-
XP_003612811.1/1-239 ----------------------GN--S-LNEVAHIA-NG-S--HP--G-N--CISLL-R-
XP_020978415.1/1-227 ----------------------GN--S-LNEVAHIA-NG-S--HP--G-N--CISLL-R-
XP_015963259.1/1-227 ----------------------GN--S-LNEVAHIA-NG-S--HP--G-N--CISLL-R-
XP_025697880.1/1-227 ----------------------GN--S-LNEVAHIA-NG-S--HP--G-N--CISLL-R-
XP_025697882.1/1-227 ----------------------GN--S-LNEVAHIA-NG-S--HP--G-N--CISLL-R-
XP_025697881.1/1-227 ----------------------GN--S-LNEVAHIA-NG-S--HP--G-N--CISLL-R-
XP_020217931.1/1-233 ----------------------GN--S-LNEVAHIA-NG-S--HP--G-N--CISLL-R-
XP_007158253.1/1-232 ----------------------GN--S-LNEVAHIA-NG-S--HP--G-N--CISLL-R-
XP_017427665.1/1-232 ----------------------GN--S-LNEVAHIA-NG-S--HP--G-N--CISLL-R-
XP_017427666.1/1-232 ----------------------GN--S-LNEVAHIA-NG-S--HP--G-N--CISLL-R-
XP_014521371.1/1-232 ----------------------GN--S-LNEVAHIA-NG-S--HP--G-N--CISLL-R-
XP_014521373.1/1-232 ----------------------GN--S-LNEVAHIA-NG-S--HP--G-N--CISLL-R-
XP_006587604.1/1-242 ----------------------GN--S-LNEVAHIA-NG-S--HP--G-N--CISLL-R-
NP_001352051.1/1-233 ----------------------GN--S-LNEVAHIA-NG-S--HP--G-N--CISLL-R-
XP_018840870.1/1-239 ----------------------GN--S-LHEVAHIA-NG-S--HP--G-N--CVSLL-R-
XP_018852246.1/1-240 ----------------------GN--S-LHEVAHIA-NG-S--HQ--G-N--CISLL-R-
XP_010053633.1/1-234 ----------------------GN--S-LQEVAHIA-NG-S--HP--G-N--CISLL-R-
XP_021675948.1/1-236 ----------------------GN--A-LHEVAHIA-NG-S--HP--G-N--CISLL-R-
XP_012081064.1/1-228 ----------------------GN--A-LHEVAHIA-NG-S--HP--G-N--CISLL-R-
XP_011028126.1/1-235 ----------------------GN--A-LHEVAHIA-NG-S--HP--G-N--CISLL-R-
XP_002303433.2/1-235 ----------------------GN--A-LHEVAHIA-NG-S--HP--G-N--CISLL-R-
XP_015897558.1/1-247 ----------------------GN--S-LHEVAHIA-NG-S--HP--G-N--CISLL-R-
XP_002277673.1/1-238 ----------------------GN--S-LHEVAHIA-NG-S--HP--G-N--CISLL-R-
XP_022996123.1/1-236 ----------------------GN--S-LHEVAHIA-NG-S--HP--G-N--CISLL-R-
XP_023534245.1/1-236 ----------------------GN--S-LHEVAHIA-NG-S--HP--G-N--CISLL-R-
XP_022931244.1/1-236 ----------------------GN--S-LHEVAHIA-NG-S--HP--G-N--CISLL-R-
XP_022931242.1/1-236 ----------------------GN--S-LHEVAHIA-NG-S--HP--G-N--CISLL-R-
XP_022159691.1/1-235 ----------------------GN--S-LHEVAHIA-NG-S--HP--G-N--CISLL-R-
XP_011652640.1/1-232 ----------------------GN--S-LHEVAHIA-NG-S--HP--G-N--CISLL-R-
XP_011652639.1/1-232 ----------------------GN--S-LHEVAHIA-NG-S--HP--G-N--CISLL-R-
XP_008466007.1/1-232 ----------------------GN--S-LHEVAHIA-NG-S--HP--G-N--CISLL-R-
XP_023511589.1/1-230 ----------------------GN--S-LHEVAHIA-NG-S--HP--G-N--CISLL-R-
XP_022963906.1/1-230 ----------------------GN--S-LHEVAHIA-NG-S--HP--G-N--CISLL-R-
XP_023511591.1/1-230 ----------------------GN--S-LHEVAHIA-NG-S--HP--G-N--CISLL-R-
XP_022963908.1/1-230 ----------------------GN--S-LHEVAHIA-NG-S--HP--G-N--CISLL-R-
XP_022967184.1/1-230 ----------------------GN--S-LHEVAHIA-NG-S--HP--G-N--CISLL-R-
XP_022967186.1/1-230 ----------------------GN--S-LHEVAHIA-NG-S--HP--G-N--CISLL-R-
XP_010518669.1/1-225 ----------------------GN--S-LHEVAHIA-NG-S--HP--G-N--CISLL-R-
XP_010677365.1/1-235 ----------------------GS--S-LHEVAHIA-NG-S--HP--G-N--CISLF-R-
XP_010677364.1/1-235 ----------------------GS--S-LHEVAHIA-NG-S--HP--G-N--CISLF-R-
XP_021852084.1/1-226 ----------------------GS--S-LHEVAHIA-NG-S--HP--G-N--SISLF-R-
XP_021852076.1/1-226 ----------------------GS--S-LHEVAHIA-NG-S--HP--G-N--SISLF-R-
XP_021765591.1/1-233 ----------------------GS--S-LHEVAHIA-NG-S--HP--G-N--CISLF-R-
XP_021768254.1/1-233 ----------------------GS--S-SHEVAHIA-NG-S--HP--G-N--CISLF-R-
XP_017232690.1/1-221 ----------------------GY--S-LNEVAHIA-NG-S--HP--G-N--CISLL-R-
XP_017232692.1/1-221 ----------------------GY--S-LNEVAHIA-NG-S--HP--G-N--CISLL-R-
XP_024021697.1/1-248 ----------------------GN--S-LHEVAHIA-NG-S--HP--G-N--CISLL-R-
XP_010096931.1/1-248 ----------------------GN--S-LHEVAHIA-NG-S--HP--G-N--CISLL-R-
XP_024021696.1/1-248 ----------------------GN--S-LHEVAHIA-NG-S--HP--G-N--CISLL-R-
XP_017621310.1/1-230 ----------------------GN--A-LHEVAHIA-NG-S--HP--G-N--CISLL-R-
XP_016731150.1/1-230 ----------------------GN--A-LHEVAHIA-NG-S--HP--G-N--CISLL-R-
XP_012472199.1/1-230 ----------------------GN--A-LHEVAHIA-NG-S--HP--G-N--CISLL-R-
XP_016727305.1/1-230 ----------------------GN--A-LHEVAHIA-NG-S--HP--G-N--CISLL-R-
XP_016731145.1/1-230 ----------------------GN--A-LHEVAHIA-NG-S--HP--G-N--CISLL-R-
XP_012472200.1/1-230 ----------------------GN--A-LHEVAHIA-NG-S--HP--G-N--CISLL-R-
XP_012472201.1/1-230 ----------------------GN--A-LHEVAHIA-NG-S--HP--G-N--CISLL-R-
NP_001314348.1/1-230 ----------------------GN--A-LHEVAHIA-NG-S--HP--G-N--CISLL-R-
XP_016727300.1/1-230 ----------------------GN--A-LHEVAHIA-NG-S--HP--G-N--CISLL-R-
XP_022738572.1/1-235 ----------------------GN--A-LHKVAHIA-NG-A--HP--G-N--CISLL-R-
XP_021297648.1/1-235 ----------------------GN--A-LHEVAHIA-NG-A--HP--G-N--CISLL-R-
XP_017973778.1/1-235 ----------------------GN--A-LHEVAHIA-NG-A--HP--G-N--CISLL-R-
XP_017973780.1/1-235 ----------------------GN--A-LHEVAHIA-NG-A--HP--G-N--CISLL-R-
XP_022717458.1/1-235 ----------------------GN--A-LHEVAHIA-NG-A--HP--G-N--CISLL-R-
XP_022717453.1/1-235 ----------------------GN--A-LHEVAHIA-NG-A--HP--G-N--CISLL-R-
XP_022717437.1/1-235 ----------------------GN--A-LHEVAHIA-NG-A--HP--G-N--CISLL-R-
XP_022756982.1/1-232 ----------------------GN--A-LHEVAHIA-NG-A--HP--G-N--CISLL-R-
XP_004300207.1/1-230 ----------------------GT--S-LQEVAHIA-NG-S--HP--G-N--CISVL-R-
XP_024169578.1/1-230 ----------------------GT--S-LQEVAHIA-NG-S--HP--G-N--CISVL-R-
XP_008238183.1/1-249 ----------------------GN--S-LHEVAHIA-NG-S--HP--G-N--CISLL-R-
XP_016651445.1/1-249 ----------------------GN--S-LHEVAHIA-NG-S--HP--G-N--CISLL-R-
XP_020418924.1/1-249 ----------------------GN--S-LHEVAHIA-NG-S--HP--G-N--CISLL-R-
XP_021804146.1/1-249 ----------------------GN--S-LHEVAHIA-NG-S--HP--G-N--CISLL-R-
XP_008238184.1/1-248 ----------------------GN--S-LHEVAHIA-NG-S--HP--G-N--CISLL-R-
XP_016651446.1/1-248 ----------------------GN--S-LHEVAHIA-NG-S--HP--G-N--CISLL-R-
XP_007209851.1/1-248 ----------------------GN--S-LHEVAHIA-NG-S--HP--G-N--CISLL-R-
XP_021804154.1/1-248 ----------------------GN--S-LHEVAHIA-NG-S--HP--G-N--CISLL-R-
XP_017179076.1/1-239 ----------------------GN--S-LHEVARIA-NG-X--HP--G-N--CISLF-R-
XP_008356445.1/1-246 ----------------------GN--S-LHEVAHIA-NG-S--HP--G-N--CISLL-R-
XP_008356446.1/1-240 ----------------------GN--S-LHEVAHIA-NG-S--HP--G-N--CISLL-R-
XP_008356447.1/1-239 ----------------------GN--S-LHEVAHIA-NG-S--HP--G-N--CISLL-R-
XP_009373485.1/1-239 ----------------------GN--S-LHEVAHIA-NG-S--HP--G-N--CISLL-R-
XP_009373484.1/1-240 ----------------------GN--S-LHEVAHIA-NG-S--HP--G-N--CISLL-R-
XP_004512486.1/1-245 ----------------------GN--S-LNEVAHIA-NG-S--HP--G-N--CISLL-R-
XP_023898631.1/1-242 ----------------------GN--S-LHEVAHIA-NG-S--HP--G-N--CISLL-R-
XP_024465375.1/1-238 ----------------------GN--A-LHEVAHIA-NG-S--HP--G-N--CISLL-R-
XP_011018502.1/1-238 ----------------------GN--A-LHEVAHIA-NG-S--HP--G-N--CISLL-R-
XP_011018503.1/1-238 ----------------------GN--A-LHEVAHIA-NG-S--HP--G-N--CISLL-R-
XP_021893615.1/1-233 ----------------------GS--A-LHEVAHIA-NG-S--HQ--G-N--CISLL-R-
XP_024952893.1/1-247 ----------------------GN--A-LHEVAHIA-NG-S--HP--G-N--CISLL-R-
XP_006471522.1/1-248 ----------------------GN--A-LHEVAHIA-NG-S--HP--G-N--CISLL-R-
XP_006432745.2/1-248 ----------------------GN--A-LHEVAHIA-NG-S--HP--G-N--CISLL-R-
XP_021640807.1/1-241 ----------------------GN--T-LHEVAHIA-NG-S--HP--G-N--CISLL-R-
XP_002518976.1/1-243 ----------------------GN--A-LHEVAHIA-NG-S--HP--G-N--CISLL-R-
XP_021598537.1/1-237 ----------------------GN--S-LHEVAHIA-NG-S--HP--G-N--CISLL-R-
XP_021598535.1/1-239 ----------------------GN--S-LHEVAHIA-NG-S--HP--G-N--CISLL-R-
XP_006659280.1/1-226 QLLQG--NALPY---GAPRN--GE--A-VQKFYRMV-NG-L--HE--G-H--AISLI-N-
XP_025801379.1/1-254 -------------------N--GA--V-VEEMSSVS-MG-Y--LA--G-N--VTSIL-S-
XP_023156326.1/1-229 -------------------S--SA--A-VTELCSVP-TG-H--LN--G-N--AVSVL-Y-
NP_001349156.1/1-229 -------------------S--SA--A-VTELCSVP-TG-H--LN--G-N--AVSVL-Y-
XP_021310588.1/1-246 -------------------N--GT--A-VAELCSVA-TG-P--LH--G-N--AVSVL-Y-
XP_020404516.1/1-224 -------------------A--G---A-VQELSSVATCP-H--LH--G-N--AVSVL-C-
XP_022684239.1/1-229 -------------------G--G---A-VQELSSIVTCR-Q--LR--G-N--VVSVL-Q-
XP_020404892.1/1-233 -------------------G--G---E-VQELGSVATSS-R--LH--G-N--AVSVL-Q-
XP_022684238.1/1-237 -------------------G--G---Q-VKELGYVSTSA-R--LH--G-N--AVSVL-R-
XP_025825251.1/1-229 -------------------G--G---P-VEEVV---------------------------
XP_020183090.1/1-242 -------------------G---A--R-VQEEASLA-TA-Q--FP--L-S--GVSVL-R-
XP_020194819.1/1-240 -------------------R--TS--T-IRQEGCFG-TG-Q--LD--G-N--SVSLL-R-
XP_024314575.1/1-244 -------------------S---G--P-MKEVACVA-TG-Q--LY--G-N--SVSVL-R-
XP_024314342.1/1-235 -------------------N--GE--P-MQEEVRIT-TG-Q--FY--G-N--HVSIF-R-
XP_024959674.1/1-192 ----------------------RG--F-ARKDLHVP-IG-N----DPS-T--GVSVL-H-
XP_019196338.1/1-215 ----------------------GS--N-IKEVMRVS-SV-R----DAT-S--SISQL-M-
XP_006646400.1/1-226 ----------------------GS--T-VKEEVSIA-RG-V----GSD-D--AVTVL-H-
XP_015632244.1/1-226 ----------------------GS--V-VKEEVTIA-RG-V----GND-D--TVTVL-H-
XP_024314625.1/1-231 ----------------------GG--A-VREENLVC-KG-T----GSN-D--NVSIL-H-
XP_020164122.1/1-228 ----------------------GN--S-VREEVAVC-KG-V--AAGID-D--VVSIL-H-
XP_025814677.1/1-221 ----------------------GG--C-VKEVVRVA-NG-V----GSE-D--AVSIL-H-
XP_022682466.1/1-225 ----------------------GG--C-VKEEVRVA-NG-V----GSE-D--AVSIL-H-
XP_002456467.1/1-226 ----------------------GG--T-VKEEVRVA-DG-V----GSG-D--AVSIL-H-
XP_008674944.1/1-228 ----------------------GG--S-VKEEVRVA-NG-V----GSD-D--AVSIL-H-
XP_010523539.1/1-228 -------------------S--G---E-IEERVRVS-KG-P--NP--G-N--HVSIF-R-
XP_020873847.1/1-203 -------------------S--GN--G-LKQVLRIT-RDDT--TP--R-N--DVSLF-S-
NP_001190248.1/1-203 ----------------------AK--D-MKELIRIG-RH-P--NS--W-N--EVSVF-S-
NP_196343.2/1-203    ----------------------AK--D-MKELIRIG-RH-P--NS--W-N--EVSVF-S-
XP_020876233.1/1-195 ----------------------AR--D-MKELIRIG-RY-P--NT--W-N--EVSVF-S-
XP_010423235.1/1-188 ----------------------VT--D-MKELICIK-RC-P--NP--G-N--EVSVF-C-
XP_010452682.1/1-218 ----------------------VK--D-MKELIRIK-RC-P--NP--G-N--EVSVF-C-
XP_010491330.1/1-188 ----------------------VK--D-MKELIRIK-RC-P--NP--G-N--EVSVF-C-
XP_006413300.2/1-224 ----------------------ET--Y-FEEKIKIQ-KS-R--SR--E-H--YVSLL-Q-
XP_022008499.1/1-216 ----------------------TI--A-TRDMYSFP-LS-Q--DEANF-N--CVSIL-D-
XP_022008502.1/1-215 ----------------------TI--A-TRDMYSFP-LS-Q--DEANF-N--CVSIL-D-
XP_022007635.1/1-226 ----------------------RI--A-ARNMIHLP-LG-Q--DETSP-N--CISIL-N-
XP_022008501.1/1-224 ----------------------RV--A-ARNVIRLP-LD-Q--DETSP-N--CISIL-N-
XP_021986984.1/1-226 ----------------------RI--A-ARNMIHLP-LG-Q--DETSP-N--CISIL-Y-
XP_021996009.1/1-224 ----------------------RI--A-ARNMIHLP-LG-Q--DETSP-N--CISIL-Y-
XP_021996011.1/1-224 ----------------------RI--A-ARNMIHLP-LG-Q--DETSP-N--CISIL-Y-
XP_022026050.1/1-224 ----------------------RI--A-VRNMIHLP-LG-Q--DETSP-N--CISIL-Y-
XP_022026083.1/1-224 ----------------------RI--A-VRNMIHLP-LG-Q--DETSP-N--CISIL-Y-
XP_021722389.1/1-209 ----------------------KR--A-SEEVFHVH-SG-Q--GP--E-D--IVSIT-K-
XP_021847315.1/1-219 ----------------------DR--S-PEEVFHIH-SG-Q--GQ--E-D--IVSIT-K-
XP_020224300.1/1-216 ----------------------GL--P-VNELRHFT-IG-G--SR--I------SIL-K-
XP_021765248.1/1-224 ----------------------GN--P-VHEMIRIP-TG-I--QS--I-N--AISIL-Q-
XP_004510020.1/1-219 ----------------------DS--R-MHEIAHIS-SS-Q--NF-SY-N--RVSIH-E-
XP_004487953.1/1-231 ----------------------ST--P-GHNIAHIS-IG-A--ND-AC-N--RVSII-Q-
XP_008244376.1/1-218 ----------------------NH--R-FDEHAYIS-NG-D--SP--E-N--GVSIL-R-
XP_008244380.1/1-218 ----------------------NH--R-FDEHAYIS-NG-D--SP--E-N--GVSIL-R-
XP_012569676.1/1-224 ----------------------GD--P-VNEIVHIS-NG-L--YP--E-N--FTSII-R-
XP_019418393.1/1-229 -------------------E--SN--P-PIELSRIC-NR-Q--HS--A-N--FISVF-Q-
XP_019440118.1/1-223 -------------------E--NN--P-LLELCRIS-NR-P--HS--A-N--FISVF-Q-
XP_014517363.2/1-226 ----------------------ES--P-VHEVARVS-VG-T--NP--S-N--CISII-Q-
XP_012569675.1/1-231 ----------------------GD--P-WHKVAHIS-TG-N--HF--S-N--YISII-Q-
XP_004486570.1/1-223 ----------------------GK--P-WQEVAHIS-TG-T--HP--N-N--YVSII-E-
XP_003594569.3/1-220 ----------------------GN--P-WHEIAHIS-TG-T--HP--N-H--YVSII-Q-
XP_024632692.1/1-220 ----------------------GN--P-WHEIAHIS-TG-T--HP--N-H--YVSII-Q-
XP_025887127.1/1-229 -------------------N--ST----MERMIQFS-KG-Q--NI--D-S--NISIY-F-
XP_015078684.1/1-230 -------------------N--ST----MERMIQFP-KG-Q--NI--D-S--NISIY-F-
XP_025887126.1/1-230 -------------------N--ST----MERMIQFS-KG-Q--NI--D-S--NISIY-F-
XP_015078401.1/1-220 -------------------I--GD--F-MKLVVYIP-KD-H--NI--D-N--NISVF-Q-
XP_015158871.1/1-217 -------------------I--SY--F-MKLMVYIP-KD-H--NI--D-N--NISLF-H-
XP_015078778.1/1-220 -------------------S--GA----MENIIHIT-KG-E--NL--E-S--SVSLL-C-
XP_010321992.1/1-216 -------------------S--GA----MENIIHIN-KD-E--NL--E-S--SVSLL-C-
XP_004240735.1/1-216 -------------------S--GA----MENIIHIN-KD-E--NL--E-S--SVSLL-C-
XP_015169793.1/1-213 -------------------N--GP----IQELVRVS-KD-Q--NL--E-S--SIYLL-R-
XP_006285631.1/1-212 -------------------L---T--S-MEEIFRIQ-KA-N--RH--E-N--TISLL-R-
XP_023635531.1/1-198 -------------------L---T--S-MEEIFRIQ-KA-N--RH--E-N--TISLL-R-
NP_567722.1/1-217    -------------------N--DT--T-MEETIRIQ-KA-K--RH--G-N--IISLL-K-
XP_020873337.1/1-219 -------------------H--NA--T-MEETIRIQ-KA-K--RH--G-N--IISLL-K-
XP_020873338.1/1-219 -------------------H--NA--T-MEETIRIQ-KA-K--RH--G-N--IISLL-K-
XP_023000325.1/1-225 ----------------------SG--P-VETLAVFA-KG-Q--NR--G-N--AVTIQ-A-
XP_023000326.1/1-214 ----------------------SG--P-VETLAVFA-KG-Q--NR--G-N--AVTIQ-A-
XP_022963997.1/1-214 ----------------------SG--P-VETLAVFA-KG-Q--NR--G-N--AVTIQ-A-
XP_023513617.1/1-208 ----------------------SG--P-VETLAVFA-KG-Q--NR--G-N--AVTIQ-A-
XP_021312124.1/1-225 -------------------H--GQ--S-VQSYVTVR-KG-D---D--G-N--CVTAY-A-
XP_012701492.1/1-243 -------------------P--GR--P-VQSCASVA-KG-K--DR--R-N--CVTAY-A-
XP_025815339.1/1-229 -------------------P--GR--P-VRSCVSVA-KG-K--DR--G-N--CVTAY-A-
XP_015644459.1/1-237 -------------------P--GK--S-VQSRVNLA-KG-K--DR--T-N--CVTAY-A-
XP_024314016.1/1-228 -------------------S--GG--T-VQRRVNLA-KG-K--DR--G-N--CVTAY-A-
XP_020168333.1/1-225 -------------------A--GR--A-VQCRVNLT-KG-K--DR--C-N--CVTAY-V-
XP_013589242.1/1-206 -------------------I--DVTTS-IEEKIKIQ-KS-R--FH--R-N--EVSLL-Q-
XP_022548456.1/1-204 -------------------I--DVTTS-IEEKIKIQ-KS-R--FH--R-N--EVSLL-Q-
XP_024632691.1/1-227 -------------------C--GN--P-MHEIAHIS-NG-P--YH--G-N--CISVI-K-
XP_017256377.1/1-220 -------------------H--DC--A-TQDIAYFA-TG-R--EP--S-S--RVSLV-V-
XP_009632057.1/1-224 -------------------I--GC--M-TRDELHIS-SA-R--DP--A-N--SVSLV-M-
XP_016502932.1/1-224 -------------------I--GC--M-TRDELHIS-SA-R--DP--A-N--SVSLV-M-
XP_019258997.1/1-221 -------------------I--GC--M-TRDELHIS-SA-R--DP--A-N--SVSLV-M-
XP_009792862.1/1-224 -------------------I--GC--M-TRDELHIS-SA-R--DP--A-N--FVSLV-M-
XP_016544784.1/1-213 -------------------Y--CC--M-TRDVLHIP-SA-R--DP--V-N--AVSLV-L-
XP_015089689.1/1-213 -------------------Y--GC--M-TRDALHIP-SA-R--DP--A-N--TVSLV-I-
XP_025883585.1/1-213 -------------------Y--GC--M-TRDALHIP-SA-R--DP--A-N--TVSLV-I-
XP_011095790.1/1-221 -------------------H--GL--E-IQDVIRMS-SA-R--NS--T-D--CISVI-S-
XP_020553953.1/1-221 -------------------H--GL--E-IQDVIRMS-SA-R--NS--T-D--CISVI-S-
XP_022857758.1/1-208 -------------------Y--GL--T-IRDITCIS-SA-R--NS--T-D--CISVA-L-
XP_004487739.1/1-224 -------------------R--KL--S-IREVAYMI-TG-E--HL--E-N--RVSLL-Q-
XP_003596663.1/1-221 -------------------Q--NL--S-IREFACIL-TG-K--HP--E-N--RVSLL-R-
XP_003596665.1/1-227 -------------------E--NL--A-IQEFACML-TG-K--HP--E-N--RVSLL-S-
XP_020201972.1/1-216 -------------------H--RL--T-IREFASMI-KG-E--NL--G-N--RVSLM-R-
XP_025980734.1/1-220 -------------------H--RL--V-IRKVASIP-KG-E--NP--G-N--CVSLL-R-
XP_007149715.1/1-218 -------------------R--RH--T-IREFASML-KG-Q--NP--G-N--RVSLM-R-
XP_017423636.1/1-218 -------------------H--RH--T-IREFASML-KG-E--NP--G-N--RVSLM-R-
XP_022631965.1/1-218 -------------------R--RH--N-IREFASML-KG-E--NP--G-N--RVSLM-S-
XP_014522034.2/1-218 -------------------R--RH--N-IREFASML-KG-E--NP--G-N--RVSLM-S-
XP_025669173.1/1-223 -------------------R--NL--T-IREFAYMN-KG-T--NP--G-N--RVSLM-R-
XP_020963786.1/1-223 -------------------R--NL--T-IREFAYMN-KG-T--NP--G-N--RVSLM-R-
XP_025614692.1/1-223 -------------------R--NL--T-IREFAYMN-KG-T--NP--G-N--RVSLM-R-
XP_015936351.1/1-223 -------------------R--NL--T-IREFAYMN-KG-T--NP--G-N--RVSLM-R-
XP_019451675.1/1-226 -------------------H--HL--S-VREFAYMN-NG-K--NL--K-N--RVSLM-R-
XP_019465428.1/1-219 -------------------R--QL--L-IREFAYIN-IG-R--NP--K-N--RVSLM-R-
XP_004295798.2/1-220 -------------------H--EK--D-TKEIAYIA-TG-D--HP--G-N--RVSII-E-
XP_021807338.1/1-219 -------------------R--NH--R-FDEHAYIS-NG-D--NP--E-N--GVSIL-R-
XP_021807340.1/1-219 -------------------R--NH--R-FDEHAYIS-NG-D--NP--E-N--GVSIL-R-
XP_020417197.1/1-219 -------------------R--NH--R-FDEHAYIS-NG-D--SP--E-N--GVSIL-R-
XP_020417200.1/1-219 -------------------R--NH--R-FDEHAYIS-NG-D--SP--E-N--GVSIL-R-
XP_009333923.1/1-206 -------------------S--NH--G-FDEYAYFA-NG-K--TP--G-N--RVSIL-R-
XP_017184363.1/1-206 -------------------S--NH--G-FDEYXYFA-NG-K--TP--G-N--RVSIL-R-
XP_021892911.1/1-224 -------------------Y--GQ--T-VEEVAYV--MG-T--NP--F-N--RVSIL-Q-
XP_002278971.2/1-210 -------------------C--GH--V-VKETGHID-NG-C--DH--G-N--RVSIL-E-
XP_010644412.1/1-211 -------------------H--GH--I-VQQTRSIS-NG-C--VP--G-N--RVSVL-E-
XP_024450660.1/1-210 -------------------R--GY--A-VQEIMHII-KG-E--SP--E-N--RVSIM-Q-
XP_002522867.2/1-214 -------------------H--EL--E-VQELTHII-KG-E--NQ--E-N--RISVL-R-
XP_020534605.1/1-220 -------------------R--EL--V-IRELTYII-KG-D--NP--E-N--RISIL-Q-
XP_021685850.1/1-217 -------------------R--EL--V-IQELTYIN-KG-D--NP--D-N--RISIL-Q-
XP_021594464.1/1-220 -------------------Q--EL--E-IQELTHII-KG-D--NP--D-N--RISVL-Q-
XP_018731177.1/1-223 -------------------Q--ER--V-IREATCIC-TG-A--DP--A-I--RVSIM-Q-
XP_024018593.1/1-217 -------------------Q--GH--S-IKELARIS-TG-E--DP--G-N--HLSLI-Q-
XP_022139420.1/1-219 -------------------Y--GL--C-ITELDYII-NG-D--DP--R-N--RVSVI-Q-
XP_022981426.1/1-220 -------------------F--GY--V-ITELACII-NG-N--DS--R-N--RVSII-Q-
XP_022981427.1/1-220 -------------------F--GY--V-ITELACII-NG-N--DS--R-N--RVSII-Q-
XP_022941489.1/1-220 -------------------F--GY--V-ITELACII-NG-N--DS--R-N--RVSII-Q-
XP_022941488.1/1-220 -------------------F--GY--V-ITELACII-NG-N--DS--R-N--RVSII-Q-
XP_023525599.1/1-220 -------------------F--GY--V-ITELACII-NG-N--DS--R-N--RVSII-Q-
XP_023525597.1/1-220 -------------------F--GY--V-ITELACII-NG-N--DS--R-N--RVSII-Q-
XP_008454681.2/1-220 -------------------Y--GH--V-ITELSCII-NG-T--DS--R-N--RVSII-Q-
XP_011648553.1/1-223 -------------------Y--GH--V-ITELSCII-NG-T--DS--R-N--RVSII-Q-
XP_018822340.1/1-211 -------------------H--GH--T-IREFAYVV-NG-E--NP--G-N--RTSIM-Q-
XP_018822367.1/1-211 -------------------H--GH--T-IREFAYVV-NG-E--NP--G-N--RTSIM-Q-
XP_023876961.1/1-210 -------------------Y--GY--S-IKELAYII-NG-E--DP--G-N--RISII-Q-
XP_023876962.1/1-210 -------------------Y--GY--S-IKELAYII-NG-E--DP--G-N--RISII-Q-
XP_024034242.1/1-215 -------------------F--GH--I-IRELAYVI-NG-N--NP--E-N--RVSIL-Q-
XP_015381349.2/1-215 -------------------F--GH--I-IRELAYVI-NG-N--NP--E-N--RVSIL-Q-
XP_022717683.1/1-216 -------------------N--ER--F-VRELAYVI-NG-E--NP--E-N--RVSVM-Q-
XP_016729588.1/1-216 -------------------N--QR--H-VRELVYVS-NG-E--NP--K-N--RVSIM-Q-
XP_016729589.1/1-216 -------------------N--QR--H-VRELVYVS-NG-E--NP--K-N--RVSIM-Q-
XP_016702140.1/1-208 -------------------N--QR--H-VRELVYVS-NG-E--NP--K-N--RVSIM-Q-
XP_012460158.1/1-216 -------------------N--QR--H-VRELVYVS-NG-E--NP--G-N--RVSIM-Q-
XP_012460145.1/1-216 -------------------N--QR--H-VRELVYVS-NG-E--NP--G-N--QVSIM-Q-
XP_017245631.1/1-197 -------------------D--IG--W-VREAGRIG----F--HS--G-N--CISLL-E-
XP_004956045.3/1-247 -------------------I--DT--A-VKELGYIA-TG-H---P--G-N--VVSVL-C-
XP_014660176.1/1-240 -------------------N--GA--A-VEELGSVA-TG-H--LD--G-N--AVSVL-C-
XP_017239609.1/1-213 -------------------N--AN--D-YEPVCCIS-SG-E--DT--K-N--CVSVH-K-
XP_023740378.1/1-206 -------------------N--AT--G-NVAHFPMS-HD-T--NN--P-N--SISLL-S-
XP_020095129.1/1-235 -------------------D--GS--E-IQ-VAEVA-NC-Q--GR--Q-N--CVSIL-R-
XP_020258373.1/1-212 -------------------E--RE--K-LVPVVLIP-TG-I--ES--G-N--YISIL-K-
XP_008812148.1/1-175 ------------------------------------------------------------
XP_010930674.1/1-230 -------------------N--GA--T-IEEACQIA-NG-R--EE--G-N--CISIL-S-
XP_010931877.1/1-230 -------------------N--GA--T-IEEACQIA-NG-R--EE--G-N--CISIL-N-
XP_009380061.1/1-225 ----------------------GN--N-LQKVAHIT-NG-S--DQ--G-N--AISIL-R-
XP_010939971.1/1-229 ----------------------GN--S-VQEVVRIT-NG-S--HP--G-N--CISLL-R-
XP_019710626.1/1-218 ------------------------------EVVRIT-NG-S--HP--G-N--CISLL-R-
XP_008787891.1/1-229 ----------------------GN--S-VQEVARIT-NG-S--HP--G-N--CISLL-R-
XP_008787892.1/1-218 ------------------------------EVARIT-NG-S--HP--G-N--CISLL-R-
XP_008799024.1/1-230 ----------------------GN--S-VQEVARIT-NG-S--HQ--G-N--GISLL-R-
XP_010936845.1/1-230 ----------------------GN--S-VQEVARIT-NG-S--HQ--G-N--CISML-R-
XP_003570542.1/1-223 ----------------------GN--P-VQEVSRIP-NG-S--HP--AAS--SISLL-R-
XP_004964922.1/1-229 ----------------------GN--Q-VQEVSRIP-NG-S--NP--G-N--CISLL-R-
XP_002438026.1/1-230 ----------------------GN--Q-VQEVSRIP-NG-S--NP--G-N--CISLL-R-
XP_020399989.1/1-232 ----------------------GN--Q-VQEVSRIP-NG-S--NP--G-N--CISLL-R-
XP_008649207.1/1-222 ----------------------GN--Q-VQEVSRIP-NG-S--NP--G-N--CISLL-R-
XP_020256822.1/1-228 ----------------------GN--M-VQEVTNIT-TG-L--NP--G-N--SISLL-R-
XP_009403550.1/1-228 ----------------------GN--T-LQEVAHIT-NG-S--HP--G-N--CISLL-R-
XP_020582158.1/1-232 ----------------------GK--A-VQEVTRIP-NG-Q--HP--G-N--CISLL-R-
XP_020699118.1/1-240 ----------------------GK--A-VQEVTRIS-ND-Q--HP--G-N--CISLL-R-
XP_020600375.1/1-232 ----------------------GK--A-VQEVTRIQ-NG-Q--HP--G-N--CISLL-R-
XP_020600376.1/1-232 ----------------------GK--A-VQEVTRIQ-NG-Q--HP--G-N--CISLL-R-
XP_020684394.1/1-232 ----------------------GK--A-VQEVTRIA-NG-Q--HP--G-N--CISLL-R-
XP_017257147.1/1-219 ----------------------HN--P-VQEVGHIA-CG-S--HP--G-N--CISVL-R-
XP_010244428.1/1-230 ----------------------GN--S-VQEVAHIA-NG-S--HP--G-N--CISVL-R-
XP_010244355.1/1-230 ----------------------GN--S-VQEVAHIA-NG-S--HP--G-N--CISVL-R-
XP_017240757.1/1-236 ----------------------QN--P-VHEVAHIA-NG-S--HP--G-N--CISVL-R-
XP_017245021.1/1-233 ----------------------HN--P-VQEVAHIA-NG-S--HS--G-N--CISVL-R-
XP_012829269.1/1-233 ----------------------QN--P-VQEIAHIA-NG-S--HP--G-N--CISVL-R-
XP_022889938.1/1-236 ----------------------QN--P-VQEIAHIA-NG-A--HP--G-N--CISVL-R-
XP_022897471.1/1-232 ----------------------QN--P-VQEVAHIS-NG-A--HP--G-N--CISVL-Q-
XP_009606250.1/1-235 ----------------------QN--P-VQEVAHIA-NG-S--HS--G-N--CISVL-R-
XP_016468230.1/1-235 ----------------------QN--P-VQEVAHIA-NG-S--HS--G-N--CISVL-R-
XP_019250773.1/1-235 ----------------------QN--P-VQEVAHIA-NG-S--HP--G-N--CISVL-R-
XP_009778533.1/1-235 ----------------------QN--P-VQEVAHIA-NG-S--HP--G-N--CISVL-R-
XP_016451582.1/1-235 ----------------------QN--P-VQEVAHIA-NG-S--HP--G-N--CISVL-R-
XP_016578176.1/1-240 ----------------------QN--P-VQEVAHIA-NG-S--HP--G-N--SISVL-R-
XP_006347370.1/1-231 ----------------------QN--P-VQEVAHIA-NG-S--HP--G-N--SISVL-R-
XP_004241474.1/1-230 ----------------------QN--P-VQEVAHIA-NG-S--HP--G-N--SISVL-R-
XP_015077748.1/1-230 ----------------------QN--P-VQEVAHIA-NG-S--HP--G-N--SISVL-R-
XP_022037776.1/1-241 ----------------------QN--P-VQEVANIA-NG-T--HP--G-N--CIAVL-K-
XP_024992062.1/1-237 ----------------------QN--P-VQEVAHIA-NG-T--HP--G-N--CISVL-R-
XP_023772270.1/1-245 ----------------------QN--P-VQEVAHIA-NG-T--HP--G-N--CISVL-R-
XP_012855673.1/1-237 ----------------------QN--P-VQEVAHIS-NG-S--HP--G-N--CISIL-R-
XP_022858328.1/1-236 ----------------------QN--P-VEEVAHIA-NG-S--HP--G-N--CISVI-R-
XP_011072039.1/1-232 ----------------------QN--P-VQEVAHIA-NG-S--HP--G-N--CISVL-R-
XP_006306921.1/1-215 ----------------------GN--P-VQEVANIA-NG-S--NP--G-N--CISVL-R-
NP_564041.2/1-215    ----------------------GN--S-VQEVAHIT-NG-S--NP--G-N--CISVL-R-
XP_020866823.1/1-216 ----------------------GN--S-VQEVAHIT-NG-S--NP--G-N--CISVL-R-
XP_010476956.1/1-215 ----------------------GN--S-VQEVANIT-NG-S--NP--G-N--CISVL-R-
XP_010498167.1/1-217 ----------------------GN--S-VQEVANIA-NG-S--NP--G-N--CISVL-R-
XP_019091528.1/1-217 ----------------------GN--S-VQEVANIA-NG-S--NP--G-N--CISVL-R-
XP_013613976.1/1-227 ----------------------GN--S-VQEVAHIA-NG-S--HP--G-N--CISVL-R-
XP_018432674.1/1-227 ----------------------GN--S-VQEVAHIA-NG-S--HP--G-N--CISVL-R-
XP_018432667.1/1-227 ----------------------GN--S-VQEVSHIA-NG-S--HP--G-N--CISVL-R-
XP_013641731.1/1-225 ----------------------GN--S-VQEVAHIA-NG-S--HP--G-N--CISVL-R-
XP_009149243.1/1-227 ----------------------GS--A-VQEVAHIA-NG-S--HP--G-N--CISVL-R-
XP_006416656.2/1-224 ----------------------GN--S-VQEVAHIA-NG-S--HP--G-N--CISVL-R-
XP_018451633.1/1-224 ----------------------GS--S-VQEVAHIA-NG-S--HP--G-N--CISVL-R-
XP_018451632.1/1-225 ----------------------GS--S-VQEVAHIA-NG-S--HP--G-N--CISVL-R-
XP_018451634.1/1-195 ----------------------GS--S-VQEVAHIA-NG-S--HP--G-N--CISVL-R-
XP_013690958.1/1-228 ----------------------GS--S-VQEVAHIA-NG-S--HP--G-N--CISVL-R-
XP_009110376.1/1-227 ----------------------GS--S-VQEVAHIA-NG-S--HP--G-N--CISVL-R-
XP_009110377.1/1-227 ----------------------GS--S-VQEVAHIA-NG-S--HP--G-N--CISVL-R-
XP_013601964.1/1-227 ----------------------GS--S-VQEVAHIA-NG-S--HP--G-N--CISIL-R-
XP_013653874.1/1-227 ----------------------GS--S-VQEVAHIA-NG-S--HP--G-N--CISIL-R-
XP_018445004.1/1-237 ----------------------SN--A-VQEVAHIA-NG-S--HP--G-C--CISVL-R-
XP_018445005.1/1-237 ----------------------SN--A-VQEVAHIA-NG-S--HP--G-C--CISVL-R-
XP_013648946.1/1-228 ----------------------SN--A-VQEVAHIA-NG-S--HP--G-C--CISVL-R-
XP_009104812.1/1-228 ----------------------SN--A-VQEVAHIA-NG-S--HP--G-C--CISVL-R-
XP_013588523.1/1-228 ----------------------SN--A-VQEVAHIA-NG-S--HP--G-C--CISVL-R-
XP_013699248.1/1-228 ----------------------SN--A-VQEVAHIA-NG-S--HP--G-C--CISVL-R-
XP_018457296.1/1-228 ----------------------RN--A-VQEVAHIA-NG-S--HP--G-C--CVSVL-R-
XP_009105998.1/1-230 ----------------------RN--A-VQEVAHIA-NG-S--HP--G-C--CISVL-R-
NP_001302850.1/1-231 ----------------------RN--A-VQEVAHIA-NG-S--HP--G-C--CISVL-R-
XP_013698656.1/1-231 ----------------------RN--A-VQEVAHIA-NG-S--HP--G-C--CISVL-R-
XP_013649619.1/1-231 ----------------------RN--A-VQEVAHIA-NG-S--HP--G-C--CISVL-R-
XP_013592873.1/1-231 ----------------------RN--A-VQEVAHIA-NV-S--HP--G-C--CISVL-R-
XP_006390565.1/1-233 ----------------------GN--A-VQEVAHIA-NG-S--HP--G-C--CVSVL-R-
XP_006301097.1/1-230 ----------------------GN--S-VQEVAHIS-SG-S--HP--G-N--CISVL-R-
XP_010428260.1/1-231 ----------------------GN--S-VQEVAHIS-NG-S--HP--G-N--CISVL-R-
XP_010471396.1/1-230 ----------------------GN--S-VQEVAHIS-NG-S--HP--G-N--CISVL-R-
XP_010416135.1/1-230 ----------------------GN--S-VHEVAHIS-NG-S--HP--G-N--CISVL-R-
NP_177479.1/1-227    ----------------------GN--A-VQEVAHIS-NG-S--HP--G-N--CISVL-R-
XP_020890987.1/1-228 ----------------------GN--A-VQEVAHIS-NG-S--HP--G-N--CISVL-R-
XP_010537520.1/1-232 ----------------------GN--A-VQEVAHIA-NG-S--HP--G-N--CISVL-R-
XP_010540042.1/1-226 ----------------------GN--A-VQEVAHIE-NG-S--HP--G-N--CISVL-R-
XP_010540046.1/1-196 ------------------------------------------------------------
XP_019432660.1/1-235 ----------------------GI--P-MQEVAHIP-CG-S--HP--S-N--CISVL-R-
XP_019420678.1/1-233 ----------------------GN--H-MQEVANIA-NG-S--HP--G-N--CISIL-Q-
XP_013452816.1/1-243 ----------------------GN--A-VQEVAHIA-NG-S--HP--G-N--SISVL-R-
XP_020215886.1/1-221 ----------------------GD--V-VQEVARIA-NG-S--HP--G-N--SISVL-R-
XP_020215888.1/1-221 ----------------------GD--V-VQEVARIA-NG-S--HP--G-N--SISVL-R-
XP_007160572.1/1-230 ----------------------GN--A-VQEVAQIA-NG-P--HP--G-N--SISVL-R-
XP_017424661.1/1-212 ----------------------GN--A-VQEVAQIT-NG-Q--NA--G-N--SISVL-R-
XP_017424660.1/1-230 ----------------------GN--A-VQEVAQIT-NG-Q--NA--G-N--SISVL-R-
XP_014508329.1/1-230 ----------------------GN--A-VQEVAQIT-NG-Q--NA--G-N--SISVL-R-
XP_022632295.1/1-230 ----------------------GN--A-VQEVAQIT-NG-Q--NA--G-N--SISVL-R-
XP_019225984.1/1-234 ----------------------QN--P-VQEVAHIA-NG-S--HP--G-N--CISVL-R-
XP_016441514.1/1-233 ----------------------QN--P-VQEVAHIA-NG-S--HP--G-N--CISVL-R-
XP_009587298.1/1-233 ----------------------QN--P-VQEVAHIA-NG-S--HP--G-N--CISVL-R-
XP_016441515.1/1-233 ----------------------QN--P-VQEVAHIA-NG-S--HP--G-N--CISVL-R-
XP_009786509.1/1-233 ----------------------QN--P-VQEVAHIA-NG-S--HP--G-N--CISVL-R-
XP_016563952.1/1-234 ----------------------QN--P-VQEVAHIA-NG-S--HS--G-N--CISVL-R-
XP_004235382.1/1-234 ----------------------QN--P-VQEVAHIA-NG-S--HS--G-N--CISVL-R-
XP_015068940.1/1-234 ----------------------QN--P-VQEVAHIA-NG-S--HS--G-N--CISVL-R-
XP_006364383.1/1-234 ----------------------QN--P-VQEVAHIA-NG-S--HS--G-N--CISVL-R-
XP_019167409.1/1-245 ----------------------QN--P-VQEVAHIA-NG-S--HP--G-N--CISVL-R-
XP_011086973.1/1-238 ----------------------QN--P-VQEVAHIA-NG-S--HP--G-N--CISVL-R-
XP_010268321.1/1-230 ----------------------GN--S-VQEVAHIA-NG-S--HP--G-N--CISVL-R-
XP_010268322.1/1-230 ----------------------GN--S-VQEVAHIA-NG-S--HP--G-N--CISVL-R-
XP_010069492.1/1-236 ----------------------GN--A-VQEVAHLA-NG-S--HP--G-N--CISVL-R-
XP_002527933.1/1-229 ----------------------GN--A-VQEVAHIA-NG-S--HP--G-N--CISVL-R-
XP_021678368.1/1-229 ----------------------GN--A-VQEVAHIA-NG-S--HP--G-N--CISVL-R-
XP_021678369.1/1-229 ----------------------GN--A-VQEVAHIA-NG-S--HP--G-N--CISVL-R-
XP_021602738.1/1-229 ----------------------GN--A-VQEVAHIA-NG-S--HP--G-N--CISVL-R-
XP_012079215.1/1-230 ----------------------GN--A-VQEVAHIA-NG-S--HP--G-N--CISVL-R-
XP_021687701.1/1-229 ----------------------GN--A-VQEVAHIA-NG-S--HP--G-N--CISVL-R-
XP_006374212.1/1-231 ----------------------GN--A-VQEVAHIT-NG-S--HP--G-N--CISVL-R-
XP_011035552.1/1-231 ----------------------GN--A-VQEVAHIT-NG-S--HP--G-N--CISVL-R-
XP_002317722.3/1-231 ----------------------GN--A-VQEVAHIA-NG-S--HP--G-N--CISVL-R-
XP_011000766.1/1-231 ----------------------GN--A-VQEVAHIA-NG-S--HP--G-N--CISVL-R-
XP_021907827.1/1-230 ----------------------GN--A-VQEVAHIA-NG-S--HP--G-N--CISVL-R-
XP_017635401.1/1-230 ----------------------GN--A-VQEVAHIA-NG-S--HP--G-N--CISVL-R-
XP_016686699.1/1-230 ----------------------GN--A-VQEVAHIA-NG-S--HP--G-N--CISVL-R-
XP_012440361.1/1-230 ----------------------GN--A-VQEVAHIA-NG-S--HP--G-N--CISVL-R-
NP_001313868.1/1-230 ----------------------GN--A-VQEVAHIA-NG-S--HP--G-N--CISVL-R-
XP_023919022.1/1-230 ----------------------GN--A-VQEVAHIA-NG-S--HP--G-N--CISVL-R-
XP_006478112.1/1-232 ----------------------GN--A-VQEVAHIA-NG-S--NP--G-N--CISVL-R-
XP_024042646.1/1-232 ----------------------GN--A-VQEVAHIA-NG-S--NP--G-N--CISVL-R-
XP_022715403.1/1-231 ----------------------GN--A-VQEVAHIA-NG-S--HP--G-N--CISVL-R-
XP_022767009.1/1-255 ----------------------GN--A-VQEVAHIA-NG-S--HP--G-N--CISVL-R-
XP_022737050.1/1-231 ----------------------GN--A-VQEVAHIA-NG-S--HP--G-N--CISVL-R-
XP_017973835.1/1-231 ----------------------SN--A-VQEVAHIA-NG-S--HP--G-N--CISVL-R-
XP_015967008.1/1-238 ----------------------GN----VQEVAHIA-NG-S--HP--G-N--CISVL-R-
XP_025701434.1/1-239 ----------------------GN----VQEVAHIA-NG-S--HP--G-N--CISVL-R-
XP_020980110.1/1-239 ----------------------GN----VQEVAHIA-NG-S--HP--G-N--CISVL-R-
XP_019434881.1/1-231 ----------------------GN--P-VQEVAHIA-NG-S--HP--G-N--RISIL-R-
XP_019458275.1/1-234 ----------------------GN--P-VQEVAHIA-NG-S--HP--G-N--CISVL-R-
XP_019458276.1/1-234 ----------------------GN--P-VQEVAHIA-NG-S--HP--G-N--CISVL-R-
XP_006584922.1/1-234 ----------------------GN--A-VQEVANIA-NG-L--HP--G-N--SISVL-R-
XP_003524332.1/1-231 ----------------------GN--A-VQEVANIA-NG-L--HP--G-N--CISVL-R-
XP_004515249.1/1-229 ----------------------GN--A-VQEVAHIA-NG-S--HP--G-N--CISVL-R-
XP_019431381.1/1-229 ----------------------GN--A-VQEVAHIA-NG-S--NP--G-N--CISVL-R-
XP_019436771.1/1-231 ----------------------GN--A-VQEVAHIA-NG-S--HP--G-N--CVSVL-R-
XP_019430342.1/1-233 ----------------------GN--D-VQEVAHIA-NG-S--HP--G-N--CISVL-R-
XP_006587436.1/1-240 ----------------------GN--A-VQEVAHIA-NG-P--HP--G-N--CISVL-R-
XP_003549189.1/1-236 ----------------------GN--A-VQEVAHIA-NG-S--HP--G-N--CISVL-R-
XP_020221986.1/1-232 ----------------------GN--A-MQEVAHIA-NG-P--HP--G-N--CISVL-R-
XP_007152598.1/1-234 ----------------------GN--A-VQEVAHIA-NG-A--HP--G-N--CISVL-R-
XP_017437542.1/1-234 ----------------------GN--A-VQEVAHIA-NG-A--HP--G-N--CISVL-R-
XP_014505752.1/1-234 ----------------------GN--A-VQEVAHIA-NG-A--HP--G-N--CISVL-R-
XP_015881991.1/1-234 ----------------------GN--A-VQEVAHIA-NG-S--HP--G-N--CISVL-R-
XP_004299483.1/1-232 ----------------------GN--A-VQEVAHIA-NG-S--HP--G-N--CISVL-R-
XP_024174647.1/1-232 ----------------------GN--A-VQEVAHIA-NG-S--HP--G-N--CISVL-R-
XP_008375041.1/1-227 ----------------------GN--A-VQDVAHIA-NG-S--HP--G-N--CISVL-R-
XP_017188172.1/1-199 ----------------------GN--A-VQDVAHIA-NG-S--HP--G-N--CISVL-R-
XP_009355964.1/1-227 ----------------------GN--A-VQDVAHIA-NG-S--HP--G-N--CISVL-R-
XP_009355965.1/1-199 ----------------------GN--A-VQDVAHIA-NG-S--HP--G-N--CISVL-R-
XP_023523434.1/1-228 ----------------------GN--P-VQEVAHIT-NG-S--HP--G-N--CISVL-R-
XP_022949138.1/1-227 ----------------------GN--P-VQEVAHIT-NG-S--HP--G-N--CISVL-R-
XP_022998050.1/1-227 ----------------------GN--P-VQEVAHIT-NG-S--HP--G-N--CISVL-R-
XP_002271012.1/1-231 ----------------------GN--A-VQEVAHIA-NG-P--HP--G-N--CISVL-R-
XP_010663934.1/1-231 ----------------------GN--A-VQEVAHIA-NG-P--HP--G-N--CISVL-R-
XP_010099679.1/1-237 ----------------------GN--A-VQEVAHIA-NG-S--HP--G-N--CISVL-R-
XP_018823334.1/1-236 ----------------------GN--G-VQEVAHIA-NG-S--HP--G-N--CISVL-R-
XP_023001145.1/1-228 ----------------------GN--P-VQEVAHIT-NG-S--HP--G-N--AISVL-R-
XP_023519889.1/1-228 ----------------------GN--P-VQEVAHIT-NG-S--HP--G-N--AISVL-R-
XP_022924191.1/1-228 ----------------------GN--P-VQEVAHIT-NG-S--HP--G-N--AISVL-R-
XP_018849090.1/1-235 ----------------------GN--G-VQEVAHIA-NG-S--HP--G-N--CISVL-R-
XP_008240378.1/1-228 ----------------------NN--A-VQEVAHIA-NG-S--HP--G-N--CISVL-R-
XP_007210460.2/1-228 ----------------------NN--A-VQEVAHIA-NG-S--HP--G-N--CISVL-R-
XP_021801797.1/1-228 ----------------------NN--A-VQEVAHIA-NG-S--HP--G-N--CISVL-R-
XP_022136988.1/1-228 ----------------------GN--P-VQEVAHIA-NG-S--HP--G-N--CISVL-R-
XP_016898817.1/1-228 ----------------------GN--P-VQEVAHIS-NG-S--HP--G-N--CISVL-R-
XP_004137568.1/1-228 ----------------------GN--P-VQEVAHIS-NG-S--HP--G-N--CISVL-R-
XP_015642098.1/1-238 ----------------------GN--Q-VQEVSRIP-NG-S--NP--G-N--CISLL-R-
XP_020186741.1/1-220 ----------------------GN--P-VQEVSRIP-NG-S--HP--G-N--SISLL-R-
XP_025828617.1/1-229 ----------------------GN--P-VQEVSRIP-NG-S--HP--G-N--CISLL-R-
XP_004954072.1/1-226 ----------------------GN--Q-VQEVSRIP-NG-S--HP--G-N--CISLL-R-
XP_002452898.1/1-231 ----------------------GN--P-VQEVSRIP-NG-S--HP--G-N--CISLL-R-
NP_001142912.2/1-230 ----------------------GN--P-VQEVSRIP-NG-S--HP--G-N--CISLL-R-
XP_020089796.1/1-231 ----------------------GN--P-VQEVARIS-NG-S--DP--G-N--CISLL-R-
XP_020089795.1/1-241 ----------------------GN--P-VQEVARIS-NG-S--DP--G-N--CISLL-R-
XP_019708739.1/1-228 ----------------------GN--S-VQEVARIV-NG-S--HP--G-N--CISLL-R-
XP_008789232.1/1-228 ----------------------GN--S-VQEVARII-NG-S--HP--G-N--CISLL-R-
XP_008789231.1/1-228 ----------------------GN--S-VQEVARII-NG-S--HP--G-N--CISLL-R-
XP_008789233.1/1-200 ----------------------GN--S-VQEVARII-NG-S--HP--G-N--CISLL-R-
XP_019704584.1/1-228 ----------------------GN--S-VQEVACVI-NG-S--HP--G-N--CISLL-R-
XP_010917295.1/1-228 ----------------------GN--S-VQEVACVI-NG-S--HP--G-N--CISLL-R-
XP_008806630.1/1-228 ----------------------GN--S-VQEVACVI-NG-S--HP--G-N--CISLL-R-
XP_009388746.1/1-226 ----------------------GN--T-LQEVAHIT-NG-S--HP--G-N--CISLL-R-
XP_018675467.1/1-228 ----------------------GN--T-VQEVAHIT-NG-S--HP--G-N--CISLL-R-
XP_018675466.1/1-228 ----------------------GN--T-VQEVAHIT-NG-S--HP--G-N--CISLL-R-
XP_009409108.1/1-228 ----------------------GN--T-VQEVAHIT-NG-S--HP--G-N--CISLL-R-
XP_024625408.1/1-220 ----------------------GN--D-VQEVVHIA-NV-S--RP--G-N--CISVF-N-
XP_010550261.1/1-219 ----------------------GN--A-VAEVGRIL-CG-P--DE--N-N--CVTVL---
XP_018816719.1/1-228 ----------------------GK--P-VHEISRIS-IG-A--HL--G-N--SISII-Q-
XP_008365756.2/1-218 ----------------------GN--P-VHXIAYIS-TG-T--HP--E-N--RISII---
XP_008381522.1/1-218 ----------------------GN--P-VHXIAYIS-TG-T--HP--E-N--RISII---
XP_018506060.1/1-218 ----------------------GI--P-VHEIAYIS-TG-T--HP--E-N--HISII---
XP_008362571.1/1-218 ----------------------GN--P-VQEIAYIS-TG-T--HP--E-N--HISII---
XP_023870350.1/1-229 ----------------------GN--P-VHEIARIA-SG-A--HP--G-K--CVSVI---
XP_016649213.1/1-222 ----------------------GN--P-VHEVTRIS-TG-T--HP--G-N--CISII---
XP_020415996.1/1-222 ----------------------GN--P-VHEVTRIS-TG-T--HP--G-N--CISII---
XP_021823813.1/1-222 ----------------------GN--P-VHEVARIS-TG-T--HP--G-N--CISII---
XP_009367126.1/1-222 ----------------------GN--P-VHEIANIS-TG-T--HP--G-N--CVSVI---
XP_008348615.1/1-222 ----------------------GN--P-VHEIANIS-TG-T--HP--G-N--CISVI---
XP_017179578.1/1-222 ----------------------GN--P-VHEIANIP-TG-T--HP--G-N--CISVI---
XP_009370612.1/1-224 ----------------------GN--P-VHEIANIP-TG-T--HP--G-N--CISVI---
XP_012845089.1/1-228 ----------------------GN--P-VNAIARIS-TG-A--DP--G-N--CISVI-Q-
XP_012845088.1/1-228 ----------------------GN--P-VNAIARIS-TG-A--DP--G-N--CISVI-Q-
XP_012845087.1/1-228 ----------------------GN--P-VNAIARIS-TG-A--DP--G-N--CISVI-Q-
XP_011085930.1/1-227 ----------------------GN--P-VNAVAHIS-TG-T--HP--G-N--CISII-Q-
XP_020551334.1/1-222 ----------------------GN--P-VNAVAHIS-TG-T--HP--G-N--CISII-Q-
XP_021297998.1/1-224 ----------------------GN--P-VKEIVNIS-TG-N--TP--G-N--CISII-T-
XP_017974480.1/1-224 ----------------------GN--R-VKEMANIS-TG-N--TP--G-N--CISII-T-
XP_024448785.1/1-229 ----------------------SN--P-VHVISHIS-NG-T--NP--G-N--CISIT---
XP_024455385.1/1-229 ----------------------SN--P-VHVISHIS-NG-T--NP--G-N--CISIT---
XP_011005937.1/1-269 ----------------------SN--P-VHVISHIS-NG-T--NP--G-N--CISIT-R-
XP_011038413.1/1-229 ----------------------GN--P-VREISHIS-NG-A--DP--G-N--CISII---
XP_018731153.1/1-226 ----------------------GN--P-VQEIARIP-SG-T--HH--G-N--SISII-Q-
XP_024190515.1/1-228 ----------------------GN--P-MNVIAHIS-TG-A--HP--G-N--CISII-Q-
XP_012074722.2/1-231 ----------------------AN--P-VHEIAHIP-IG-T--HP--G-N--CISII-R-
XP_010089514.2/1-229 ----------------------GY--P-VHEIEHIS-TG-A--HP--G-N--CISII-R-
XP_024949330.1/1-235 ----------------------GS--P-VQEIVHIS-NG-S--HL--G-N--FTSII-W-
XP_006421085.1/1-235 ----------------------GS--P-VQEIVHIS-NG-S--HL--G-N--CTSII-W-
XP_002268272.1/1-229 ----------------------GN--P-VHEIAHIA-NG-V--NP--G-N--CTSII-R-
XP_004140733.2/1-228 ----------------------GN--P-VHEIAHIL-TG-V--HP--G-N--LISII-R-
XP_008457114.1/1-228 ----------------------GN--P-VHEIAHIL-TG-V--HP--G-N--LISII-R-
XP_008457113.1/1-229 ----------------------GN--P-VHEIAHIL-TG-V--HP--G-N--LISII-R-
XP_011651208.1/1-229 ----------------------GN--P-VHEIAHIL-TG-V--HP--G-N--LISII-R-
XP_022143367.1/1-228 ----------------------GN--P-VHEIAHIL-TG-V--HP--G-N--SISII---
XP_022935490.1/1-228 ----------------------GS--P-VHEIAHIL-TG-V--HP--G-N--SILII---
XP_022984101.1/1-228 ----------------------GS--P-VHEIAHIL-TG-V--HP--G-N--SILII---
XP_022935488.1/1-229 ----------------------GS--P-VHEIAHIL-TG-V--HP--G-N--SILII-R-
XP_022984098.1/1-229 ----------------------GS--P-VHEIAHIL-TG-V--HP--G-N--SILII-R-
XP_022984102.1/1-229 ----------------------GS--P-VHEIAHIL-TG-V--HP--G-N--SILII-R-
XP_015579295.1/1-234 ----------------------GK--P-VHEIAHIS-NG-T--HP--G-N--CIAII-R-
XP_002526595.2/1-234 ----------------------GK--P-VHEIAHIS-NG-T--HP--G-N--CIAII-R-
XP_015579294.1/1-234 ----------------------GK--P-VHEIAHIS-NG-T--HP--G-N--CIAII-R-
XP_021643520.1/1-232 ----------------------GN--P-VHEIAHIS-GG-A--HP--G-N--CISII-R-
XP_021605433.1/1-231 ----------------------GN--P-VHELTQIS-NG-V--HP--G-N--CISII-R-
XP_023739098.1/1-228 ----------------------NK--P-VQEITRIT-NR-A--HP--G-N--CISVL-Q-
XP_024966239.1/1-227 ----------------------HN--P-VQEVTHIA-NG-A--HP--G-N--CISLL-R-
XP_025813558.1/1-232 ----------------------AN--Q-VQEVLRIP-NG-S--NP--D-N--CVSVL-R-
XP_020150479.1/1-234 ----------------------EN--S-VQPVSRIP-NG-P--NP--G-N--CITLL-R-
XP_003564122.2/1-224 ----------------------GN--T-VQEACRIP-NG-S--DP--A-N--CVTLL-R-
XP_006585049.2/1-225 ----------------------GK--P-GHEIQRIS-TG-N--NP--G-N--FISIT-K-
XP_007143783.1/1-226 ----------------------GN--A-GHEIQRIT-TG-S--NP--G-N--CISIM-R-
XP_007158859.1/1-226 ----------------------GN--S-GHEIQRIT-TG-S--NP--G-N--CISIM-R-
XP_020230815.1/1-227 ----------------------KN--S-VQEIARIS-SG-T--HP--S-N--YISII-Q-
XP_006586851.1/1-227 ----------------------KR--P-LHEIARIS-TG-T--HP--N-N--YISII-Q-
XP_021888058.1/1-234 ----------------------GN--P-VNVINQIT-NS-N--NS--G-N--CISII-K-
XP_020886111.1/1-226 ----------------------GT--L-VNEIARIA-TG-S--SE--T-N--CLTIL-R-
XP_020886110.1/1-228 ----------------------GT--L-VNEIARIA-TG-S--SE--T-N--CLTIL-R-
XP_011469296.1/1-224 ----------------------DN--P-MGEIAHIP-TG-A--HP--G-N--CISII-Q-
XP_009367128.2/1-218 ----------------------GN--P-VHEIAYIS-TG-T--HP--E-N--RISII-R-
XP_008348673.1/1-198 ----------------------GN--P-VHEIAYIS-TG-T--HP--E-N--RVSII-R-
XP_012832506.1/1-227 ----------------------GS--P-VIPVAHIS-TG-T--RP--G-N--RISII-Q-
XP_012832508.1/1-227 ----------------------GS--P-VIPVAHIS-TG-T--RP--G-N--RISII-Q-
XP_012832516.1/1-227 ----------------------GN--P-VTTVAHIS-TG-T--HP--G-N--RISII-Q-
XP_012832518.1/1-227 ----------------------GN--P-VTTVAHIS-TG-T--HP--G-N--RISII-Q-
XP_012835569.1/1-227 ----------------------GN--P-VTTVAHIS-TG-K--HP--G-N--RISII-Q-
XP_012835571.1/1-226 ----------------------GN--P-VTTVAHIS-TG-K--HP--G-N--RISII-Q-
XP_019172066.1/1-226 ----------------------GY--P-VTEVARIP-TG-N--HP--G-N--CVSII-Q-
XP_009631364.2/1-226 ----------------------GN--A-VGELARIP-TG-F--FP--G-N--CISII-Q-
XP_016435660.1/1-229 ----------------------GN--A-VGELAQIP-TG-F--FP--G-N--CISII-Q-
XP_009771801.1/1-226 ----------------------GN--A-VGELARIP-TG-Y--FP--G-N--CISII-Q-
XP_016493115.1/1-226 ----------------------GN--A-VGELARIP-TG-Y--FP--G-N--CISII-Q-
XP_019248352.1/1-226 ----------------------GN--A-VGELARIP-TG-F--FP--G-N--CISII-Q-
XP_025691463.1/1-225 ----------------------GN--P-VTQIQRIS-NG-T--FS--D-N--FTTIL-R-
XP_020995109.1/1-225 ----------------------GN--P-VTQIQRIS-NG-T--FS--D-N--FTTIL-R-
XP_016190415.2/1-225 ----------------------GN--P-VTQIQRIS-NG-T--FS--D-N--FTTIL-R-
XP_004486568.1/1-232 ----------------------GN--P-VHEIAHIS-NG-L--YP--G-N--CISII-Q-
XP_024632687.1/1-226 ----------------------GN--P-MHEIAHIS-NG-P--YH--G-N--CISII-K-
XP_020977993.1/1-241 ----------------------TT--P-VHYIAHIS-NG-T--HP--A-N--NTSII-Q-
XP_025647253.1/1-241 ----------------------TT--P-VHYIAHIS-NG-T--HP--A-N--NTSII-Q-
XP_020997095.1/1-240 ----------------------TT--P-VHYIAHIS-NG-T--HP--A-N--NTSII-Q-
XP_025692389.1/1-228 ----------------------TT--P-VHYIAHIS-NG-T--HP--A-N--NTSII-Q-
XP_019418389.1/1-226 ----------------------GS--M-IHKIAQIS-TR-N--QP--E-N--SISII-Q-
XP_007147460.1/1-227 ----------------------GN--Q-SHKVAHIS-NG-I--HP--G-N--CVSII-R-
XP_014517364.1/1-227 ----------------------GN--Q-AHKVANIS-NG-I--HP--G-N--CVSVI-R-
XP_017435438.1/1-226 ----------------------GN--Q-AHKVAHIS-NG-I--HP--G-N--CVSVI-R-
XP_017435439.1/1-217 --------------------------Q-AHKVAHIS-NG-I--HP--G-N--CVSVI-R-
XP_025981664.1/1-227 ----------------------GN--N-ANKVAHIS-NG-I--HP--G-N--CISIS-R-
XP_006586853.1/1-227 ----------------------GN--D-ANKVAHIS-NE-I--HP--G-N--CISIY-R-
XP_020230831.1/1-226 ----------------------GN--P-THRVAHIS-NG-I--HP--H-N--CISIL-Q-
XP_004489663.1/1-217 ----------------------GN--R-VHEIAHIS-NG-Y--HH--G-N--FISLL-K-
XP_018489833.1/1-227 ----------------------EN--V-ITETAHIA-TG-S--SE--T-N--YINLL-Q-
XP_013584487.1/1-219 ----------------------GN--A-ITETARIF-TG-S--SG--T-N--CINLL-Q-
XP_009130351.1/1-219 ----------------------GN--A-ITESARIF-TG-S--SG--T-N--CITLL-Q-
XP_013747906.2/1-219 ----------------------GN--T-ITETARIF-TG-S--SG--T-N--CITLL-Q-
XP_013747661.1/1-219 ----------------------GN--T-ITETARIF-TG-S--SG--T-N--CITLL-Q-
XP_013619678.1/1-219 ----------------------GN--A-ITETARIS-TG-S--SG--T-N--YINLL-Q-
XP_022551084.1/1-219 ----------------------GN--A-ITETARIS-TG-S--SG--T-N--YINLL-Q-
XP_009126392.1/1-219 ----------------------GN--A-ITETARIF-TG-S--SG--T-N--YINLL-Q-
XP_018437612.1/1-222 ----------------------RN--A-VSEIARVF-TG-S--NE--N-N--YITIL-Q-
XP_013591191.1/1-225 ----------------------RS--A-VSVTARIL-TG-S--NE--N-N--YITIL-L-
XP_022567005.1/1-225 ----------------------RS--A-VSVTARIL-TG-S--NE--N-N--YITIL-L-
XP_022547796.1/1-225 ----------------------RS--A-VSVTARIL-TG-Y--NE--N-N--YITIL-Q-
XP_009119205.1/1-225 ----------------------RS--A-VSVTARIL-TG-Y--NE--N-N--YITIL-Q-
XP_009119199.1/1-225 ----------------------RS--A-VSVTARIL-TG-Y--NE--N-N--YITIL-Q-
XP_006408322.1/1-232 ----------------------GK----VSEIARVF-TG-S--SE--T-N--YLNIL-R-
XP_023642295.1/1-229 ----------------------GT--A-VSEIARIA-TG-S--NE--S-N--CLTIL-R-
XP_023642294.1/1-229 ----------------------GT--A-VSEIARIA-TG-S--NE--S-N--CLTIL-R-
NP_186976.2/1-228    ----------------------GT--V-VNEIARIV-TG-S--SE--T-N--CVTIL-R-
XP_010507998.1/1-229 ----------------------GT--V-VTEIARVA-TG-S--SE--T-N--CLSIL-R-
XP_010485661.1/1-229 ----------------------GT--V-VTEIARIA-TG-S--SE--T-N--CLSIL-R-
XP_010450732.1/1-230 ----------------------GT--V-VTEIARIA-TG-S--SE--T-N--CLSIL-R-
XP_010463776.1/1-230 ----------------------GT--V-VTEIARIA-TG-S--SE--T-N--CLSIL-R-
XP_010463774.1/1-230 ----------------------GT--V-VTEIARIA-TG-S--SE--T-N--CLSIL-R-
XP_019092960.1/1-229 ----------------------GT--V-VTEIARIA-TG-S--SE--T-N--CLSIL-R-
XP_022551077.1/1-228 ----------------------GY--P-AAEVAGFA-TG-T--DD--K-N--RVNIL-Q-
XP_013619236.1/1-228 ----------------------GY--P-AAEVAGFA-TG-T--DD--K-N--RVNIL-Q-
XP_009126177.1/1-226 ----------------------GY--P-AAEVAGFA-TG-T--DD--K-N--RVNIL-Q-
XP_013652421.1/1-226 ----------------------GY--P-AAEVAGFA-TG-T--DD--K-N--RVNIL-Q-
XP_013652420.1/1-226 ----------------------GY--P-AAEVAGFA-TG-T--DD--K-N--RVNIL-Q-
XP_006400265.1/1-228 ----------------------GK--P-VAEVARFV-TG-L--DN--K-C--SVTIL-Q-
XP_013590801.1/1-226 ----------------------AS--P-VTEVARFV-TG-A--NN--K-N--CVNIL-Q-
XP_013651910.1/1-222 ----------------------AS--P-VTEVARFV-TG-A--NN--K-N--CVNIL-Q-
XP_009103958.1/1-222 ----------------------AS--P-VTEVARFV-TG-A--NN--K-N--CVNIL-Q-
XP_018444485.1/1-226 ----------------------AS--P-VTEVARFV-TG-T--DN--K-N--CVNIL-K-
XP_009121397.1/1-226 ----------------------AS--P-VTEVARFV-TG-T--DN--K-N--CVNIL-K-
XP_013659428.1/1-226 ----------------------AS--P-VTEVARFV-TG-T--DN--K-N--CVNIL-K-
XP_022565333.1/1-226 ----------------------AS--P-VTEVARFV-TG-A--DN--K-N--CVNIL-K-
XP_013610327.1/1-226 ----------------------AS--P-VTEVARFV-TG-A--DN--K-N--CVNIL-K-
XP_006286655.1/1-225 ----------------------GN--P-VTEVARFI-TG-T--DT--K-N--NVNFL-E-
XP_010492677.1/1-224 ----------------------GN--S-VTEAARFV-TG-T--DT--N-N--NVNFI-Q-
XP_010420445.1/1-224 ----------------------GN--P-VTEVARFV-TG-T--DK--K-N--NVNFI-E-
XP_010453918.1/1-224 ----------------------GN--P-VTEPARFV-TG-T--DT--K-N--NVNFI-Q-
XP_006303739.1/1-224 ----------------------GN--P-VIEATRFV-TG-L--KK--K-N--NVTFL-Q-
XP_020876074.1/1-222 ----------------------GN--P-ATEAARFV-TG-S--DQ--K-N--TVTFL-Q-
NP_197234.1/1-222    ----------------------GN--P-ATEAARFV-TG-S--NP--R-N--TVSFL-E-
NP_174724.1/1-221    ----------------------GN--P-VTEAARFV-TG-P--DQ--K-N--NVTFL-Q-
XP_020869876.1/1-222 ----------------------GN--P-VTEAARFV-TG-T--DQ--K-N--NVTFL-Q-
XP_019091098.1/1-222 ----------------------GN--P-ATEAARFV-TG-S--NQ--K-N--NVTFL-K-
XP_010478910.1/1-223 ----------------------GN--P-ATEAARFV-TG-S--DQ--K-N--NVTFL-K-
XP_010501781.1/1-221 ----------------------GN--P-ATEAARFV-TG-S--DQ--K-N--NVTFL-K-
XP_015089790.1/1-222 ----------------------GN--N-VTELVRVL-TG-T--LP--G-N--DITII-Q-
XP_015089784.1/1-222 ----------------------GN--N-VTELVRVL-TG-T--LP--G-N--DITII-Q-
XP_016549855.1/1-224 ----------------------GN--N-VTELTRVP-TG-A--FP--G-N--SVTII-Q-
XP_015057403.1/1-221 ----------------------GL--K-MTELARVP-TG-T--FS--E-N--CITII-Q-
XP_019066399.1/1-221 ----------------------GL--K-MTELARVS-TG-T--FP--E-N--CITII-Q-
XP_015166059.1/1-216 ----------------------GN--N-VIELAQVL-TG-V--HP--G-N--SITII-Q-
XP_019071012.2/1-216 ----------------------GN--N-VIELARVL-TG-T--LP--E-N--SITII-Q-
XP_015086709.1/1-216 ----------------------GN--N-VIELARVL-TG-T--LP--E-N--SITII-Q-
XP_015087432.1/1-219 ----------------------EN--N-VFELDRVP-TG-T--FS--G-N--NITII-Q-
XP_004247342.2/1-219 ----------------------EN--N-VIELDRVP-TG-T--FL--G-N--NITII-Q-
XP_025888717.1/1-219 ----------------------EN--N-VIELDRVP-TG-T--FL--G-N--NITII-Q-
XP_015087599.1/1-221 ----------------------GN--N-VIELDRVL-TG-T--FP--A-N--NITII-Q-
XP_019071156.1/1-221 ----------------------EN--N-VIELDRVL-TG-T--FP--A-N--NITII-Q-
XP_019070762.2/1-220 ----------------------GN--N-VIELDRVL-TG-T--FP--G-N--NITVI-Q-
XP_015087071.1/1-220 ----------------------GN--N-VIELDRVP-TG-T--FP--G-N--NITII-Q-
XP_025888845.1/1-217 ----------------------GN--N-VIELDRVL-TG-T--FP--G-N--NITII-Q-
XP_015170367.1/1-232 ----------------------GN--N-VTELARIN-TG-S--IP--G-N--NITII-Q-
XP_025886268.1/1-232 ----------------------EN--T-VTELARIE-TG-C--VP--G-N--NITII-Q-
XP_015072491.1/1-224 ----------------------EN--T-VTELARIE-TG-C--VP--G-N--NITII-Q-
XP_010327374.2/1-219 ----------------------GN--Y-TAEMDRLS-TG-T--ST--K-N--CITFI-E-
XP_015089749.1/1-219 ----------------------GN--Y-TAEMDRVS-TG-T--ST--K-N--CITFI-Q-
XP_015170971.1/1-209 ----------------------EN--N-VIEKDKVQ-TG-T--SL--E-N--NITII-Q-
XP_025887726.1/1-223 ----------------------AN--N-VFEKDRVK-TG-A--SL--E-N--NIKII-Q-
XP_006586358.1/1-227 ----------------------GS--A-GHEIQRIS-TG-S--NP--G-N--CISIM-R-
XP_014617351.1/1-228 ----------------------KR--P-LHEIARIS-TG-T--HP--N-N--YISIIQQ-
XP_007147461.1/1-227 ----------------------ES--P-VHEVARIS-IG-T--QP--S-N--YISII-Q-
XP_017435408.1/1-226 ----------------------ES--P-VHEVARVS-VG-T--NP--S-N--CISII-Q-
XP_017215828.1/1-223 ----------------------KG--P-VRNIANLV-KG-Q--DQ--R-N--AVTIQ-A-
XP_012856110.1/1-229 ----------------------GS--T-VHSTVNLA-KG-Q--DR--G-N--AVTVM-D-
XP_023738891.1/1-226 ----------------------GG--P-VQSIANLA-KG-Q--DR--G-N--SVSIH-T-
XP_022017964.1/1-227 ----------------------GG--P-VQSIANLA-KG-Q--DR--V-N--TVSIH-K-
XP_022154996.1/1-226 ----------------------GG--S-AETITNFA-KG-Q--NR--G-N--AVTIQ-A-
XP_008462169.1/1-225 ----------------------GG--Q-VEMLANFA-KG-Q--NR--G-N--AVTIQ-A-
XP_004141841.1/1-226 ----------------------SG--Q-AEMLANFA-KG-Q--NR--G-N--AVTIQ-A-
XP_020098514.1/1-228 ----------------------GS--P-TQTIVNLA-KG-Q--HR--G-N--SVTVH---
XP_020098513.1/1-228 ----------------------GS--P-TQTIVNLA-KG-Q--HR--G-N--SVTVH---
XP_020263950.1/1-229 ----------------------RG--P-AETIVNLV-KG-K--DR--G-N--SITVH---
XP_020263948.1/1-255 ----------------------RG--P-AETIVNLV-KG-K--DR--G-N--SITVH-V-
XP_009415234.2/1-232 ----------------------PS--P-TQTMVNLV-KG-Q--DR--G-N--SVTIY-S-
XP_008789333.1/1-230 ----------------------GG--P-TATIANLA-KG-Q--DR--G-N--SVLVH---
XP_010920420.1/1-235 ----------------------GG--P-TETIANLA-KG-Q--DR--G-N--SVLVH---
XP_010920411.1/1-235 ----------------------GG--P-TETIANLA-KG-Q--DR--G-N--SVLVH---
XP_010920404.1/1-235 ----------------------GG--P-TETIANLA-KG-Q--DR--G-N--SVLVH---
XP_010521515.1/1-219 ----------------------GA--H-VQSIASLS-KG-Q--DR--G-N--SVTVH-A-
XP_013652056.1/1-227 ----------------------GA--H-VQSIVSLS-KG-Q--DR--G-N--SVSIQ-T-
XP_009104428.1/1-227 ----------------------GA--H-VQSIVSLS-KG-Q--DR--G-N--SVSIQ-T-
XP_018445476.1/1-226 ----------------------GA--H-VQSIVSLS-KG-Q--DR--G-N--SVSIQ-T-
XP_013590784.1/1-226 ----------------------GA--H-VQSIVSLS-KG-Q--DR--G-N--SVSIQ-T-
XP_013680822.1/1-226 ----------------------GA--H-VQSIVSLS-KG-Q--DR--G-N--SVSIQ-T-
XP_006389720.2/1-226 ----------------------GA--H-VQSIASLS-KG-Q--DR--G-N--SVSIQ-T-
XP_023642973.1/1-226 ----------------------GA--H-VQSIASLS-KG-Q--DR--G-N--SVAIQ-T-
XP_020891640.1/1-226 ----------------------GA--H-VQSIASLS-KG-Q--DR--G-N--SVAIQ-T-
NP_001320267.1/1-226 ----------------------GA--H-VQSIANLS-KG-Q--DR--G-N--SVAIQ-T-
NP_565223.1/1-226    ----------------------GA--H-VQSIANLS-KG-Q--DR--G-N--SVAIQ-T-
NP_001185443.1/1-226 ----------------------GA--H-VQSIANLS-KG-Q--DR--G-N--SVAIQ-T-
XP_010473021.1/1-226 ----------------------GA--H-VQSIASLS-KG-Q--DR--G-N--SVAIQ-T-
XP_010430051.1/1-226 ----------------------GA--H-VQSIASLS-KG-Q--DR--G-N--SVAIQ-T-
XP_025617667.1/1-241 -------------------G--GS--S-VQSIATLA-KG-Q--DR--G-N--AVNIH-E-
XP_015938429.1/1-242 -------------------G--GS--S-VQSIATLA-KG-Q--DR--G-N--AVNIH-E-
XP_016176999.1/1-241 -------------------G--GS--S-VQSIATLA-KG-Q--DR--G-N--AVNIH-E-
XP_021862846.1/1-225 ----------------------GG--Q-VQTIVNLA-KG-Q--DR--G-N--SVSIQ-T-
XP_022979290.1/1-231 ----------------------GD--E-AKSIANLA-KG-Q--DR--G-N--SVTIQ-T-
XP_022955725.1/1-233 ----------------------GD--E-AKSIANLA-KG-Q--DR--G-N--SVTIQ-T-
XP_022998610.1/1-231 ----------------------GD--K-VDSIANLA-KG-Q--DR--G-N--SVSIQ-T-
XP_022998611.1/1-227 ----------------------GD--K-VDSIANLA-KG-Q--DR--G-N--S-----T-
XP_023523626.1/1-229 ----------------------GD--K-VESIANLA-KG-Q--DR--G-N--SVSIQ-T-
XP_022948296.1/1-231 ----------------------GD--K-VESIANLA-KG-Q--DR--G-N--SVSIQ-T-
XP_023523628.1/1-225 ----------------------GD--K-VESIANLA-KG-Q--DR--G-N--S-----T-
XP_022948295.1/1-227 ----------------------GD--K-VESIANLA-KG-Q--DR--G-N--S-----T-
XP_010249693.1/1-228 ----------------------GG--P-VQSIANLA-KG-Q--DR--G-N--AVTI----
XP_010254081.1/1-230 ----------------------GG--P-VQSIANIA-KG-K--DR--G-N--AVTV----
XP_003529763.1/1-227 ----------------------GG--S-VQSIANLA-KG-K--DR--G-N--VVNIQ---
XP_003533048.1/1-226 ----------------------GG--S-VQSVANLA-KG-K--DR--G-N--VVNIQ---
XP_014634625.1/1-226 ----------------------GG--S-VQSVANLA-KG-K--DR--G-N--VVNIQ---
XP_007150635.1/1-226 ----------------------GG--T-VQSIANLS-KG-Q--DR--G-N--AVTIQ---
XP_014498860.1/1-226 ----------------------GG--T-VQSIANLS-KG-Q--DR--G-N--VVTIQ---
XP_017425661.1/1-226 ----------------------GG--T-VLSIANLS-KG-Q--DR--G-N--AVTIQ---
XP_020207725.1/1-226 ----------------------GG--T-VQSIANLA-KG-Q--DR--G-N--AVSIQ---
XP_003546423.2/1-226 ----------------------GG--T-VQSIANLA-KG-Q--DR--G-N--AVAIQ---
XP_003543638.1/1-226 ----------------------GG--T-VQSIANLA-KG-Q--DR--G-N--AVAIQ---
XP_019457980.1/1-226 ----------------------GG--S-VQSIANLA-KG-Q--DR--G-N--AVTIQ---
XP_003597736.1/1-226 ----------------------GG--T-VQSIANLA-KG-Q--DR--G-N--AVTIQ---
XP_004486751.1/1-226 ----------------------GG--S-VQSIANLA-KG-Q--DR--G-N--AVTIQ---
XP_016169615.1/1-226 ----------------------GG--S-VQSIANLA-KG-Q--DR--G-N--AVTIQ---
XP_025611006.1/1-226 ----------------------GG--S-VQSIANLA-KG-Q--DR--G-N--AVTIQ---
XP_020982478.1/1-227 ----------------------GG--S-VQSIANLA-KG-Q--DR--G-N--AVTIQ---
XP_021892835.1/1-225 ----------------------GG--P-VQSIANLA-KG-Q--DR--G-N--AVTI----
XP_012442963.1/1-227 ----------------------GG--P-VQSIANLA-KG-K--DR--G-N--AVTI----
NP_001314068.1/1-227 ----------------------GG--P-VQSIANLA-KG-K--DR--G-N--AVTI----
XP_016684379.1/1-227 ----------------------GG--P-VQSIANLA-KG-K--DR--G-N--AVTI----
XP_016730082.1/1-227 ----------------------GG--P-VQSIANLA-KG-K--DR--G-N--AVTI----
NP_001316938.1/1-227 ----------------------GG--P-VQSIANLA-KG-K--DR--G-N--AVTI----
XP_017606636.1/1-227 ----------------------GG--P-VQSIANLA-KG-K--DR--G-N--AVTI----
XP_022714992.1/1-229 ----------------------GG--P-VQSIANLA-KG-Q--HR--G-N--AVTI----
XP_022721436.1/1-227 ----------------------GG--P-VQSVANLA-KG-Q--DR--G-N--AVTI----
XP_022721435.1/1-227 ----------------------GG--P-VQSVANLA-KG-Q--DR--G-N--AVTI----
XP_007024189.1/1-227 ----------------------GG--P-VQSIANLA-KG-Q--DR--G-N--AVTI----
XP_021298731.1/1-227 ----------------------GG--P-VQSIANLA-KG-Q--DR--G-N--AVAI----
XP_004302894.1/1-228 ----------------------GG--P-AQTIANLS-KG-Q--DR--G-N--AVTI----
XP_011466687.1/1-235 ----------------------GG--P-AQTIANLS-KG-Q--DR--G-N--AVTIQ-P-
XP_024180978.1/1-228 ----------------------GG--P-AQTIANLS-KG-Q--DR--G-N--AVTI----
XP_024180977.1/1-235 ----------------------GG--P-AQTIANLS-KG-Q--DR--G-N--AVTIQ-P-
XP_009353605.1/1-226 ----------------------GG--P-AQTIANLS-KG-Q--DR--G-N--AVTI----
XP_008380909.1/1-226 ----------------------GG--P-AQTIANLS-KG-Q--DR--G-N--AVTI----
NP_001280927.1/1-227 ----------------------GG--P-AQTIANLS-KG-Q--DR--G-N--AVTI----
XP_009338062.1/1-226 ----------------------GG--P-AQTIANLS-KG-Q--DR--G-N--AVTI----
XP_009338046.1/1-226 ----------------------GG--P-AQTIANLS-KG-Q--DR--G-N--AVTI----
XP_021829136.1/1-226 ----------------------GG--P-AQTIANLS-KG-Q--DR--G-N--AVTI----
XP_008228560.1/1-226 ----------------------GG--P-AQTIANLS-KG-Q--DR--G-N--AVTI----
XP_007217160.1/1-226 ----------------------GG--P-AQTIANLS-KG-Q--DR--G-N--AVTI----
XP_022891611.1/1-228 ----------------------GS--P-VQCIVNLA-KG-Q--DR--G-N--AVTI----
XP_002304207.2/1-228 ----------------------GG--P-VQATANLA-KG-Q--DR--G-N--AVTI----
XP_011010522.1/1-228 ----------------------GG--P-VQATANLA-KG-Q--DR--G-N--AVTI----
XP_011009828.1/1-227 ----------------------GG--P-VQTIANLI-KG-Q--DR--G-N--AAAI----
XP_024462179.1/1-228 ----------------------GG--P-VQTIANLI-KG-Q--DR--G-N--AAAI----
XP_018837483.1/1-226 ----------------------GG--P-AQSIANLA-KG-Q--DR--G-N--AVTI----
XP_018856342.1/1-226 ----------------------GD--Q-TQSIANLS-KG-Q--DR--G-N--AVTI----
XP_018856994.1/1-226 ----------------------GD--Q-TQSIANLS-KG-Q--DR--G-N--AVTI----
XP_018858360.1/1-226 ----------------------GD--Q-TQSIANLS-KG-Q--DR--G-N--AVTI----
XP_015867693.1/1-226 ----------------------GN--Q-VQSMANLS-KG-Q--DR--G-N--AVTI----
XP_010062490.1/1-226 ----------------------GS--P-VQSIANLA-KG-Q--DR--G-N--AVNI----
XP_010098082.1/1-228 ----------------------TG--S-VESMANLA-KG-Q--DR--G-N--AVSI----
XP_002284502.1/1-228 ----------------------GG--P-VQSIANLA-KG-Q--DR--G-N--AVTI----
XP_023875609.1/1-226 ----------------------GG--P-AQSIANLA-KG-Q--DR--G-N--SVAI----
XP_002516023.1/1-226 ----------------------GG--Q-VQSIANLA-KG-Q--DR--G-N--AVTI----
XP_021652755.1/1-228 ----------------------GS--P-VQSIANLA-KG-Q--DR--G-N--AVTI----
XP_021654904.1/1-226 ----------------------GG--P-VQSIANLA-KG-Q--DR--G-N--AVTI----
XP_021611342.1/1-229 ----------------------GD--P-VQSIANLA-KG-K--DR--G-N--AVTI----
XP_012073376.1/1-226 ----------------------GG--T-VQSIANLA-KG-Q--DR--G-N--AVAI----
XP_006465622.1/1-226 ----------------------GG--P-VQTIANLA-KG-Q--DR--G-N--AVNI----
XP_006426950.1/1-226 ----------------------GG--P-VQTIANLA-KG-Q--DR--G-N--AVNI----
XP_009392419.1/1-229 ----------------------PG--P-TQTTVNLA-KG-Q--DR--G-N--SVTM----
XP_020518333.1/1-227 -----------------------S--P-IQPIANIA-KG-Q--DR--G-N--SVAL----
XP_018629167.1/1-227 ----------------------GG--P-VESIANLA-TG-Q--DR--G-N--AVTI----
XP_009611250.1/1-227 ----------------------GG--P-VESIANLA-TG-Q--DR--G-N--AVTI----
XP_009611249.1/1-227 ----------------------GG--P-VESIANLA-TG-Q--DR--G-N--AVTI----
XP_009611248.1/1-227 ----------------------GG--P-VESIANLA-TG-Q--DR--G-N--AVTI----
XP_016479156.1/1-227 ----------------------GG--P-VESIANLA-TG-Q--DR--G-N--AVTI----
XP_016479155.1/1-227 ----------------------GG--P-VESIANLA-TG-Q--DR--G-N--AVTI----
XP_016479154.1/1-227 ----------------------GG--P-VESIANLA-TG-Q--DR--G-N--AVTI----
XP_009778213.1/1-229 ----------------------GG--P-AQSVANLA-KG-Q--DK--G-N--AVSI----
XP_016501674.1/1-229 ----------------------GG--P-AQSVANLA-KG-Q--DK--G-N--AVSI----
XP_019224885.1/1-229 ----------------------GG--P-AQSVANLA-KG-Q--DK--G-N--AVSI----
XP_019224886.1/1-229 ----------------------GG--P-AQSVANLA-KG-Q--DK--G-N--AVSI----
XP_009592772.1/1-229 ----------------------GG--P-AQSVANLA-KG-Q--DK--G-N--AVSI----
XP_016496403.1/1-229 ----------------------GG--P-AQSVANLA-KG-Q--DK--G-N--AVSI----
XP_016562690.1/1-228 ----------------------GG--P-VQSVANLA-KG-Q--DK--G-N--AVSI----
XP_006343080.1/1-229 ----------------------GG--P-VQSVANLA-KG-Q--DK--G-N--AVSI----
XP_004235676.1/1-229 ----------------------GG--P-VQSVANLA-KG-Q--DK--G-N--AVSI----
XP_020227921.1/1-228 ----------------------GG--S-VKSIANLA-KG-K--DR--G-N--VVNI----
XP_007135673.1/1-226 ----------------------GG--S-VQLIANLA-KG-K--DR--G-N--AVNI----
XP_014515030.1/1-226 ----------------------GG--S-VQSIANLA-KG-K--GR--G-N--AVNI----
XP_017407449.1/1-226 ----------------------GG--S-VQSIANLA-KG-K--GR--G-N--AVNI----
XP_010691342.1/1-224 ----------------------GG--H-VQLIANLA-KG-Q--DR--G-N--SVSI----
XP_021731998.1/1-222 ----------------------GG--Q-VQLIANLA-KG-Q--DR--G-N--SVSI----
XP_021736870.1/1-224 ----------------------GG--Q-VQSIANLA-KG-Q--DR--G-N--SVSI----
XP_024969955.1/1-229 ----------------------GG--P-VQSIANLA-KG-K--DR--G-N--TVSI----
XP_024969954.1/1-229 ----------------------GG--P-VQSIANLA-KG-K--DR--G-N--TVSI----
XP_024969953.1/1-229 ----------------------GG--P-VQSIANLA-KG-K--DR--G-N--TVSI----
XP_021984354.1/1-227 ----------------------GG--P-VQSIANLA-KG-Q--DQ--G-N--TVSI----
XP_017232370.1/1-228 ----------------------EG--P-VQNIASMA-KG-Q--DR--G-N--AVTI----
XP_017216573.1/1-228 ----------------------EG--P-AQNIANLV-KG-Q--DH--R-N--AVTI----
XP_017248777.1/1-227 ----------------------GG--P-VQTIVNLA-KG-Q--DR--G-N--AVTI----
XP_021627148.1/1-226 ----------------------GG--L-LQTIANLA-KG-Q--DR--G-N--AVTI----
XP_021627146.1/1-226 ----------------------GG--L-LQTIANLA-KG-Q--DR--G-N--AVTI----
XP_021627145.1/1-226 ----------------------GG--L-LQTIANLA-KG-Q--DR--G-N--AVTI----
XP_019196965.1/1-228 ----------------------RG--P-VESIANLA-KG-Q--DR--G-N--AVTI----
XP_019196967.1/1-228 ----------------------RG--P-VESIANLA-KG-Q--DR--G-N--AVTI----
XP_022848789.1/1-228 ----------------------GS--P-VQCIGNLA-KG-Q--DR--G-N--AVTI----
XP_011072373.1/1-228 ----------------------GS--R-VQSIAKLA-KG-Q--DR--G-N--TVSA----
XP_021907286.1/1-215 ----------------------TG--F-VQKIAHIE-NG-R--DS--E-N--SVYLL-R-
XP_020885309.1/1-217 ----------------------GE--M-MQKIAEVT-NG-R--DN--R-N--CASLL-R-
XP_020885307.1/1-217 ----------------------GE--M-MQKIAEVT-NG-R--DN--R-N--CASLL-R-
NP_180796.2/1-218    ----------------------GE--M-MHKIAEIT-NG-I--DK--R-N--CASLL-R-
XP_006296541.1/1-217 ----------------------GG--N-AQKIVEIA-NG-R--DS--R-N--CASLL-R-
XP_010510028.1/1-215 ----------------------GG--VVVHKISEIA-NG-R--DS--R-N--CATLL-R-
XP_010414006.1/1-214 ----------------------GG--V-VHKISEIA-NG-R--DS--R-N--CATLL-R-
XP_010469602.1/1-219 ----------------------GG--V-VHKISEIA-NG-R--DS--R-N--CATLL-R-
XP_010417818.1/1-218 ----------------------GW--I-LQKISEIA-NG-R--DS--R-N--CATLL-R-
XP_010510869.1/1-220 ----------------------GW--I-VQKISEIA-NG-R--DS--R-N--CATLL-K-
XP_010510870.1/1-218 ----------------------GW--I-VQKISEIA-NG-R--DS--R-N--CATLL-K-
XP_018484769.1/1-219 ----------------------GG--EAVQLMSQIS-NG-R--DI--R-N--CVSLL-R-
XP_018484754.1/1-219 ----------------------GG--EAVQLMSQIS-NG-R--DI--R-N--CVSLL-R-
XP_013634882.1/1-219 ----------------------GG--EDLQLMSQIG-NG-R--DS--R-N--CVSLL-R-
XP_018513778.1/1-219 ----------------------GG--EGLQLMSQIG-NG-R--DS--R-N--CVSLL-R-
XP_009141217.1/1-219 ----------------------GG--EGLQLMSQIG-NG-R--DS--R-N--CVSLL-R-
XP_013747219.1/1-219 ----------------------GG--EGLQLMSQIG-NG-R--DS--R-N--CVSLL-R-
XP_024003849.1/1-217 ----------------------AG--N-VQKLSQIA-NG-R--DS--R-N--CVSIL-Q-
XP_018437853.1/1-211 ----------------------AG--T-PQKLTHIF-NG-R--DT--R-N--CVSLL-R-
XP_009143975.1/1-211 ----------------------GG--N-PQKLTHIF-NG-R--DN--R-N--CVSLL-R-
XP_009143974.1/1-211 ----------------------GG--N-PQKLTHIF-NG-R--DN--R-N--CVSLL-R-
XP_022575029.1/1-211 ----------------------GG--N-PQKLTHIF-NG-R--DN--R-N--CVSLL-R-
XP_013748684.1/1-211 ----------------------GG--N-PQKLTHIF-NG-R--DN--R-N--CVSLL-R-
XP_013694194.1/1-211 ----------------------GG--N-PQKLTHIF-NG-R--DN--R-N--CVSLL-R-
XP_013634261.1/1-211 ----------------------GG--N-PQKLTHIF-NG-R--DD--R-N--CVSLL-R-
XP_013741430.1/1-210 ----------------------GG--N-PQKLTHIF-NG-R--DD--R-N--CVSLL-R-
XP_017254373.1/1-221 ----------------------GG--L-VEEKLHIA-TG-H--GP--A-N--AVSLL-Q-
XP_017254240.1/1-222 ----------------------TG--Q-VYEIMRIS-NG-C--DP--S-S--CISLL-N-
XP_017221582.1/1-224 ----------------------VG--Q-VQPIVHIA-NG-R--DP--S-S--RISLL-C-
XP_017221584.1/1-224 ----------------------VG--Q-VQPIVHIA-NG-R--DP--C-S--RISLL-C-
XP_015941575.1/1-215 ----------------------GG--V-AEEFAHIK-NG-R--DP--G-N--CITLF-R-
XP_025620358.1/1-215 ----------------------GG--V-AEEFAHIK-NG-R--DP--G-N--CITLF-R-
XP_016176493.1/1-215 ----------------------GG--V-AEEFAHIK-NG-R--DP--G-N--CITLF-R-
XP_003610661.2/1-228 ----------------------GG--I-VHELAHIS-NG-R--DS--G-N--YVSLF-R-
XP_004496853.1/1-220 ----------------------GG--I-IQEMAHIP-NG-Q--DP--A-N--CVSLI-R-
XP_006353342.1/1-230 ----------------------GG--S-VHPIVHIG-KG-Q--DL--G-N--SITLF-R-
XP_015070591.1/1-229 ----------------------GG--S-VHRIVHIA-KG-Q--DP--G-N--SITLF-R-
XP_019068356.1/1-229 ----------------------GG--S-VHRIVHIA-KG-Q--DP--G-N--SITLF-R-
XP_004234441.1/1-229 ----------------------GG--S-VHRIVHIA-KG-Q--DP--G-N--SITLF-R-
XP_024926606.1/1-217 ----------------------GL--S-VNEVAYIH-NG-K--DP--L-N--RVSIM-Q-
XP_024525400.1/1-231 ----------------------SG--M-VQEVARIA-KG-Q--AT--G-N--DVSLF-R-
XP_002965186.2/1-231 ----------------------SG--M-VQEVARIA-KG-Q--AT--G-N--DVSLF-R-
XP_002962272.2/1-231 ----------------------SG--M-VQEVARIA-KG-Q--AT--G-N--DVSLF-R-
XP_019441536.1/1-226 ----------------------SA--L-VQEKEHIA-TG-R--DS--G-N--CISLI-Q-
XP_019441537.1/1-226 ----------------------SA--L-VQEKEHIA-TG-R--DS--G-N--CISLI-Q-
XP_019441535.1/1-226 ----------------------SA--L-VQEKEHIA-TG-R--DS--G-N--CISLI-Q-
XP_019451758.1/1-229 ----------------------TG--Q-VQENAHIS-NG-G--EQ--G-N--CITLI-Q-
XP_012850835.1/1-238 ----------------------GA--E-FREMAHIV-SG-D--EI--G-N--RVALY-Q-
XP_020552079.1/1-225 ----------------------GG--D-VEEMMQIV-SG-K--EI--G-N--SVAIY-Q-
XP_020552078.1/1-225 ----------------------GG--D-VEEMMQIV-SG-K--EI--G-N--SVAIY-Q-
XP_020552080.1/1-223 ----------------------GG--D-VEEMMQIV-SG-K--EI--G-N--SVAIY-Q-
XP_009408807.2/1-226 ----------------------GG--M-IHEAACIA-NG-Q--QG--G-N--CVSIY-T-
XP_006342404.1/1-225 ----------------------VG--T-IQQVTHIA-NG-T--EI--G-N--SISIL-R-
XP_025887998.1/1-218 ----------------------GG--T-IQEVTHIA-NG-M--EI--G-N--SISIL-R-
XP_015081481.1/1-225 ----------------------GG--T-IQEVTHIA-NG-M--EI--G-N--SISIS-R-
XP_022000230.1/1-232 ----------------------GS--A-MQNMIQFS-KS-Q--DP--G-NLNTISLL-R-
XP_022000229.1/1-232 ----------------------GS--A-MQNMIQFS-KS-Q--DP--G-NLNTISLL-R-
XP_022037790.1/1-222 ----------------------GS--A-MQNMICLP-KT-Q--DL--G-NLNTISL--R-
XP_022037789.1/1-222 ----------------------GS--A-MQNMICLP-KT-Q--DL--G-NLNTISL--R-
XP_024991925.1/1-226 ----------------------GS--A-MQNMIRFS-KG-Q--DL--G-NPNTISLL-R-
XP_023760005.1/1-229 ----------------------GT--A-MQNMIRFS-KS-Q--DH--G-NLNTISLL-R-
XP_023760001.1/1-229 ----------------------GT--A-MQNMIRFS-KS-Q--DH--G-NLNTISLL-R-
XP_017251723.1/1-236 ----------------------GG--S-MEKIICIS-KG-Q-ADY--G-K--SISVL-R-
XP_017243321.1/1-239 ----------------------GG--P-MQQMACIA-KG-Q--DF--G-N--KISLL-S-
XP_017243319.1/1-239 ----------------------GG--P-MQQMACIA-KG-Q--DF--G-N--KISLL-S-
XP_017243320.1/1-239 ----------------------GG--P-MQQMACIA-KG-Q--DF--G-N--KISLL-S-
XP_017255563.1/1-234 ----------------------GG--Q-MQQMIRIP-KG-Q--DS--G-N--RISLS-C-
XP_017255562.1/1-234 ----------------------GG--Q-MQQMIRIP-KG-Q--DS--G-N--RISLS-C-
XP_012852237.1/1-226 ----------------------DG--P-MQPMLHIP-KS-Q--DF--G-N--TISLL-R-
XP_012852238.1/1-226 ----------------------DG--P-MQPMLHIP-KS-Q--DF--G-N--TISLL-R-
XP_012852236.1/1-226 ----------------------DG--P-MQPMLHIP-KS-Q--DF--G-N--TISLL-R-
XP_012852235.1/1-226 ----------------------DG--P-MQPMLHIP-KS-Q--DF--G-N--TISLL-R-
XP_011076208.1/1-227 ----------------------DG--P-MQQMLQIP-KG-Q--DP--G-N--NICLL-S-
XP_019200484.1/1-228 -----------------------G--T-LQQMIHIS-KG-Q--DP--A-N--QISLY-R-
XP_019200481.1/1-228 -----------------------G--T-LQQMIHIS-KG-Q--DP--A-N--QISLY-R-
XP_022879034.1/1-223 ----------------------DG--C-VQQMLHIP-KS-Q--DL--V-N--SISVL-R-
XP_018623115.1/1-238 ----------------------SG--P-MQQMVHIA-KG-Q--NI--D-N--SISLF-R-
XP_018623116.1/1-238 ----------------------SG--P-MQQMVHIA-KG-Q--NI--D-N--SISLF-R-
XP_016449095.1/1-238 ----------------------SG--P-MQQMVHIA-KG-Q--NI--D-N--SISLF-R-
XP_009771835.1/1-238 ----------------------SG--P-MQQLVHIA-KG-Q--NI--D-N--SISLF-R-
XP_009771833.1/1-238 ----------------------SG--P-MQQLVHIA-KG-Q--NI--D-N--SISLF-R-
XP_019248454.1/1-225 ----------------------SG--P-MQQMVHIA-KG-Q--NI--D-N--SISLF-R-
XP_016564677.1/1-235 ----------------------GD--A-MHEIVHIA-KG-Q--DL--G-N--SISLY-R-
XP_016564676.1/1-235 ----------------------GD--A-MHEIVHIA-KG-Q--DL--G-N--SISLY-R-
XP_009606534.1/1-237 ----------------------GG--P-MHQIVHIA-KG-Q--DL--G-N--SISLF-R-
XP_016457239.1/1-237 ----------------------GG--P-MHQIVHIA-KG-Q--DL--G-N--SISLF-R-
XP_009606533.1/1-237 ----------------------GG--P-MHQIVHIA-KG-Q--DL--G-N--SISLF-R-
XP_009606532.1/1-237 ----------------------GG--P-MHQIVHIA-KG-Q--DL--G-N--SISLF-R-
XP_016457238.1/1-237 ----------------------GG--P-MHQIVHIA-KG-Q--DL--G-N--SISLF-R-
XP_016468064.1/1-237 ----------------------GG--P-MHQIVHIA-KG-Q--DL--G-N--SISLF-R-
XP_009774404.1/1-237 ----------------------GG--P-MHQIVHIA-KG-Q--DL--G-N--SISLF-R-
XP_016468062.1/1-237 ----------------------GG--P-MHQIVHIA-KG-Q--DL--G-N--SISLF-R-
XP_016468061.1/1-237 ----------------------GG--P-MHQIVHIA-KG-Q--DL--G-N--SISLF-R-
XP_009774402.1/1-237 ----------------------GG--P-MHQIVHIA-KG-Q--DL--G-N--SISLF-R-
XP_009774401.1/1-237 ----------------------GG--P-MHQIVHIA-KG-Q--DL--G-N--SISLF-R-
XP_019251817.1/1-237 ----------------------GG--P-MHQIVHIA-KG-Q--DL--G-N--SISLF-R-
XP_019251815.1/1-237 ----------------------GG--P-MHQIVHIA-KG-Q--DL--G-N--SISLF-R-
XP_019251816.1/1-237 ----------------------GG--P-MHQIVHIA-KG-Q--DL--G-N--SISLF-R-
XP_011470395.1/1-233 ----------------------CR--P-LQAMVQID-KD-H--EG--G-N--SVSLF-N-
XP_004309219.2/1-233 ----------------------CR--P-LQAMVQID-KD-H--EG--G-N--SVSLF-N-
XP_024180881.1/1-233 ----------------------CR--P-LQAMVQVD-KD-H--GG--G-N--SVSLF-N-
XP_024180911.1/1-233 ----------------------CR--P-LQAMVQVD-KD-H--GG--G-N--SVSLF-N-
XP_019450525.1/1-216 ----------------------NG--P-MQQLFRIS-KG-Q--GN--G-N--CVSVL-R-
XP_019465255.1/1-230 ----------------------NV--P-MQELLRIS-KG-Q--VN--G-N--CVSVL-S-
XP_014502466.1/1-227 ----------------------GG--P-MKEMLHVP-KG-Q--EH--G-N--CVSIL-H-
XP_017438850.1/1-222 ----------------------GG--P-MKEMLHVP-KG-Q--DH--G-N--CVSIL-H-
XP_006599623.1/1-221 ----------------------DG--P-MKEMLHIA-KG-Q--DR--G-N--CVSIL-H-
XP_006599622.1/1-221 ----------------------DG--P-MKEMLHIA-KG-Q--DR--G-N--CVSIL-H-
XP_006599621.1/1-221 ----------------------DG--P-MKEMLHIA-KG-Q--DR--G-N--CVSIL-H-
XP_004515730.1/1-240 ----------------------GG--P-MQEMVHIP-KG-Q--SL--G-N--SVCIL-R-
XP_013442370.1/1-239 ----------------------GG--P-MQEVVHIA-KG-Q--AL--G-N--SISIL-S-
XP_004252035.1/1-225 ----------------------GS--P-LQEIVQIA-NG-T--QT--G-N--CVSLL-R-
XP_015060726.1/1-225 ----------------------GS--P-LQEIAQIA-NG-T--QT--G-N--CVSLL-R-
XP_006589608.1/1-229 ----------------------GA--Q-VNELAHIA-KG-R--DH--G-N--SVSLL-R-
XP_006589605.1/1-230 ----------------------GA--Q-VNELAHIA-KG-R--DH--G-N--SVSLL-R-
XP_006849468.1/1-231 ----------------------GN--A-VQEVAHIA-NG-S--HP--G-N--CISLL-R-
XP_020526173.1/1-231 ----------------------GN--A-VQEVAHIA-NG-S--HP--G-N--CISLL-R-
XP_020109515.1/1-228 ----------------------GN--S-VQEVARIT-SG-S--HP--G-N--CISLL-R-
NP_001310555.1/1-224 ----------------------GN--P-VQEVSRIP-NG-S--HP--G-N--CISLL-R-
XP_010544375.1/1-225 ----------------------GG--P-VQEMTHFS-IG-R--DR--C-N--SVSLL-R-
NP_001314001.1/1-230 ----------------------GR--P-MQAMFSVA-KG-P--GQ--G-N--CVSIL-R-
XP_016732454.1/1-230 ----------------------GR--P-MQAMFSVA-KG-P--GQ--G-N--CVSIL-R-
XP_016732453.1/1-230 ----------------------GR--P-MQAMFSVA-KG-P--GQ--G-N--CVSIL-R-
XP_012484067.1/1-230 ----------------------GR--P-MQAMFSVA-KG-P--GQ--G-N--CVSIL-R-
XP_012484066.1/1-230 ----------------------GR--P-MQAMFSVA-KG-P--GQ--G-N--CVSIL-R-
XP_017631791.1/1-230 ----------------------GR--P-MQAMYSVA-KG-P--GQ--G-N--CVSIL-R-
XP_017631789.1/1-230 ----------------------GR--P-MQAMYSVA-KG-P--GQ--G-N--CVSIL-R-
XP_016728129.1/1-230 ----------------------GR--P-MQAMYSVA-KG-P--GQ--G-N--CVSIL-R-
XP_016728128.1/1-230 ----------------------GR--P-MQAMYSVA-KG-P--GQ--G-N--CVSIL-R-
XP_021671684.1/1-240 ----------------------GG--M-MQEMVHIS-KG-H--CR--G-N--CVSLL-R-
XP_021671683.1/1-240 ----------------------GG--M-MQEMVHIS-KG-H--CR--G-N--CVSLL-R-
XP_021671685.1/1-240 ----------------------GG--M-MQEMVHIS-KG-H--CR--G-N--CVSLL-R-
XP_002318231.3/1-240 ----------------------GG--M-LQEIIQIP-KG-Q--GQ--C-N--RVSLL-R-
XP_024438602.1/1-240 ----------------------GG--M-LQEIIQIP-KG-Q--GQ--C-N--RVSLL-R-
XP_024442055.1/1-239 ----------------------GG--I-LQEMVQIP-KG-Q--GH--W-N--TVSVL-R-
XP_002322460.1/1-239 ----------------------GG--I-LQEMVQIP-KG-Q--GH--W-N--TVSVL-R-
XP_011048682.1/1-239 ----------------------GG--I-LQEMVQIP-KG-Q--GH--W-N--TVSVL-R-
XP_011048684.1/1-239 ----------------------GG--I-LQEMVQIP-KG-Q--GH--W-N--TVSVL-R-
XP_011048683.1/1-239 ----------------------GG--I-LQEMVQIP-KG-Q--GH--W-N--TVSVL-R-
XP_019081404.1/1-234 ----------------------GG--P-MQEMIHIP-KG-Q--TS--S-N--CVSLL-R-
XP_003634081.1/1-234 ----------------------GG--P-MQEMIHIP-KG-Q--TS--S-N--CVSLL-R-
XP_008377249.1/1-231 ----------------------NV--P-MQRIVQIA-KG-Q--GG--A-N--SVSLL-R-
XP_017189459.1/1-231 ----------------------NV--P-MQRIVQIA-KG-Q--GG--A-N--SVSLL-R-
XP_008377244.1/1-231 ----------------------NV--P-MQRIVQIA-KG-Q--GG--A-N--SVSLL-R-
XP_009371302.1/1-231 ----------------------GV--P-MQRIIQIG-KG-Q--EG--A-N--GVSLL-R-
XP_009371299.1/1-231 ----------------------GV--P-MQRIIQIG-KG-Q--EG--A-N--GVSLL-R-
XP_008376545.1/1-231 ----------------------GV--P-MQRIVQIG-KG-Q--GG--A-N--SVSLL-R-
XP_008376544.1/1-231 ----------------------GV--P-MQRIVQIG-KG-Q--GG--A-N--SVSLL-R-
XP_017188609.1/1-231 ----------------------GV--P-MQRIVQIG-KG-Q--GG--A-N--SVSLL-R-
XP_022945426.1/1-238 ----------------------GR--P-MIEMLRIS-KG-Q--GL--D-N--RVSLL-R-
XP_022945425.1/1-238 ----------------------GR--P-MIEMLRIS-KG-Q--GL--D-N--RVSLL-R-
XP_023541229.1/1-238 ----------------------GR--P-MIEMLRIS-KG-Q--GL--D-N--RVSLL-R-
XP_023541228.1/1-238 ----------------------GR--P-MIEMLRIS-KG-Q--GL--D-N--RVSLL-R-
XP_022968414.1/1-238 ----------------------GR--P-MIEMLCIS-KG-Q--GL--D-N--RVSLL-R-
XP_022968413.1/1-238 ----------------------GR--P-MIEMLCIS-KG-Q--GL--D-N--RVSLL-R-
XP_023552381.1/1-232 ----------------------SR--P-MLEMVRIS-KG-Q--DA--D-N--RVSLL-R-
XP_022922479.1/1-232 ----------------------SR--P-MLEMVRIS-KG-Q--DA--D-N--RVSLL-R-
XP_023552380.1/1-232 ----------------------SR--P-MLEMVRIS-KG-Q--DA--D-N--RVSLL-R-
XP_022922478.1/1-232 ----------------------SR--P-MLEMVRIS-KG-Q--DA--D-N--RVSLL-R-
XP_022984644.1/1-232 ----------------------SR--P-MLEMVRIS-KG-Q--DA--D-N--RVSLL-R-
XP_022984643.1/1-232 ----------------------SR--P-MLEMVRIS-KG-Q--DA--D-N--RVSLL-R-
XP_008439185.1/1-242 ----------------------SR--P-MLEMLRIS-KG-Q--GP--D-N--RVSLL-C-
XP_008439184.1/1-242 ----------------------SR--P-MLEMLRIS-KG-Q--GP--D-N--RVSLL-C-
XP_011651140.1/1-242 ----------------------SR--P-MLEMLRIS-KS-Q--GP--D-N--RVSLL-C-
XP_004140784.1/1-242 ----------------------SR--P-MLEMLRIS-KS-Q--GP--D-N--RVSLL-C-
XP_009391954.1/1-234 ----------------------GG--P-MCEMAHIA-KG-Q--EA--A-N--RVSLI-W-
XP_003581542.2/1-227 ----------------------GG--P-MQEVTNIA-KG-Q--QD--G-N--TVSLL-R-
XP_006652658.1/1-224 ----------------------GG--P-MQEVTSIA-KG-Q--QD--G-N--SVCLL-K-
XP_015637147.1/1-225 ----------------------GG--P-MQEVTSIA-KG-Q--QN--G-N--TVCLL-K-
XP_025823430.1/1-225 ----------------------GG--P-MQQMLRIA-KG-Q--LD--G-N--SVTLL-R-
XP_004978185.1/1-225 ----------------------GG--P-MQQIVRIA-KG-Q--LD--G-N--SVTLL-R-
XP_021317935.1/1-228 ----------------------GG--P-MQQMLRIT-KG-Q--LD--G-N--SVTLL-R-
NP_001336434.1/1-226 ----------------------GG--P-MQQVLRIS-KG-Q--LD--G-N--SVALL-R-
XP_019709552.1/1-234 ----------------------GG--P-MQEVLRVA-KG-H--HA--A-I--AVSLL-R-
XP_015696802.1/1-257 ----------------------GE--A-MQEMDHIA-KG-Q--HH--G-N--AVSLL-R-
XP_015612621.1/1-258 ----------------------GE--A-MQEMDHIA-KG-Q--HH--G-N--AVSLL-R-
XP_004957413.1/1-253 ----------------------GE--A-MQEMGHIA-KG-Q--HH--G-N--AVSLL-R-
XP_025801167.1/1-251 ----------------------GE--A-MQEMDHIA-KG-Q--HH--G-N--AVSLL-R-
NP_001105125.2/1-256 ----------------------GE--A-MQEMDHIA-KG-Q--HH--G-N--AVSLL-R-
XP_002462701.1/1-258 ----------------------GE--A-MQEMDHIA-KG-Q--HH--G-N--AVSLL-R-
XP_020262629.1/1-222 ----------------------GG--P-MQEMAHIA-KG-Q--HQ--G-N--AVSLL-R-
XP_020699782.1/1-235 ----------------------GG--P-MQEVAHIA-KG-Q--DH--G-N--AVSLL-R-
XP_020699776.1/1-235 ----------------------GG--P-MQEVAHIA-KG-Q--DH--G-N--AVSLL-R-
XP_020573166.1/1-235 ----------------------GG--P-MQEVAHIA-KG-H--DR--G-N--SVSLL-R-
XP_020187432.1/1-228 ----------------------GG--P-MQEVTKIY-KG-Q--QN--G-N--AVSLL-K-
XP_025656795.1/1-233 ----------------------IG--Q-MKEMLRIS-KG-Q--VD--G-N--SLSVL-R-
XP_025656792.1/1-233 ----------------------IG--Q-MKEMLRIS-KG-Q--VD--G-N--SLSVL-R-
XP_025656794.1/1-233 ----------------------IG--Q-MKEMLRIS-KG-Q--VD--G-N--SLSVL-R-
XP_020979736.1/1-233 ----------------------IG--Q-MKEMLRIS-KG-Q--VD--G-N--SLSVL-R-
XP_020999151.1/1-233 ----------------------IG--Q-MKEMLRIS-KG-Q--VD--G-N--SLSVL-R-
XP_015967451.1/1-233 ----------------------IG--Q-MKEMLRIS-KG-Q--VD--G-N--SLSVL-R-
XP_020999150.1/1-233 ----------------------IG--Q-MKEMLRIS-KG-Q--VD--G-N--SLSVL-R-
XP_025701970.1/1-233 ----------------------IG--Q-MKEMLRIS-KG-Q--VD--G-N--SLSVL-R-
XP_025701968.1/1-233 ----------------------IG--Q-MKEMLRIS-KG-Q--VD--G-N--SLSVL-R-
XP_025701969.1/1-233 ----------------------IG--Q-MKEMLRIS-KG-Q--VD--G-N--SLSVL-R-
XP_020221720.1/1-222 ----------------------GG--P-MQEMIRIP-KG-Q--ED--G-N--CVSIL-T-
XP_007152327.1/1-222 ----------------------GG--P-MKEMLHVP-KG-Q--EH--G-N--CVSIL-H-
XP_006587345.1/1-222 ----------------------DG--P-MKEMLHIA-KG-Q--DR--G-N--CVSIL-H-
XP_006587344.1/1-222 ----------------------DG--P-MKEMLHIA-KG-Q--DR--G-N--CVSIL-H-
XP_010035104.1/1-239 ----------------------GG--P-MQEIVHVA-KG-P--RR--D-N--CVSLL-R-
XP_021765162.1/1-234 ----------------------GG--P-MEVVARLS-KS-H--DV--N-N--CVSLL-C-
XP_021765160.1/1-234 ----------------------GG--P-MEVVARLS-KS-H--DV--N-N--CVSLL-C-
XP_021730123.1/1-234 ----------------------GG--P-MEVVARLS-KS-H--DV--N-N--CVSLL-C-
XP_021842103.1/1-232 ----------------------GG--P-MEVIARLA-KS-H--DG--N-N--CVSLL-C-
XP_021842106.1/1-232 ----------------------GG--P-MEVIARLA-KS-H--DG--N-N--CVSLL-C-
XP_021842105.1/1-232 ----------------------GG--P-MEVIARLA-KS-H--DG--N-N--CVSLL-C-
XP_021842104.1/1-232 ----------------------GG--P-MEVIARLA-KS-H--DG--N-N--CVSLL-C-
XP_010693471.1/1-235 ----------------------GG--P-MEVIARLS-KS-H--DG--N-N--CVALL-C-
XP_019107938.1/1-235 ----------------------GG--P-MEVIARLS-KS-H--DG--N-N--CVALL-C-
XP_010693469.1/1-235 ----------------------GG--P-MEVIARLS-KS-H--DG--N-N--CVALL-C-
XP_019107937.1/1-235 ----------------------GG--P-MEVIARLS-KS-H--DG--N-N--CVALL-C-
XP_020163116.1/1-258 ----------------------GE--A-MQEMDHIA-KG-Q--HH--G-N--AVSLL-R-
XP_020163109.1/1-258 ----------------------GE--A-MQEMDHIA-KG-Q--HH--G-N--AVSLL-R-
XP_003578475.1/1-267 ----------------------GE--A-MQEMDHIA-KG-Q--LH--G-N--AVSLL-R-
XP_011012369.1/1-242 ----------------------GG--I-LQEIIQIP-KG-QGQGQ--W-N--RVSLL-R-
XP_011012370.1/1-242 ----------------------GG--I-LQEIIQIP-KG-QGQGQ--W-N--RVSLL-R-
XP_008234278.1/1-230 ----------------------TH--K-MQLMLRIA-KS-Q-GG---G-N--CVSLL-R-
XP_021827117.1/1-230 ----------------------TH--K-MQLMLRIA-KS-Q-GG---G-N--CVSLL-R-
XP_021827116.1/1-230 ----------------------TH--K-MQLMLRIA-KS-Q-GG---G-N--CVSLL-R-
XP_020413581.1/1-230 ----------------------TH--K-MQLMLRIA-KS-Q-GG---G-N--CVSLL-R-
XP_007219555.2/1-230 ----------------------TH--K-MQLMLRIA-KS-Q-GG---G-N--CVSLL-R-
XP_018456926.1/1-230 ----------------------GA--S-VENMLLIP-KR-Q-RE---G-R--CVSLL-Q-
XP_013613235.1/1-226 ----------------------GA--S-MENMLLIP-QR-Q-SE---G-R--CISLL-Q-
XP_013686466.1/1-227 ----------------------GA--S-MENMLLIP-QR-Q-SE---G-R--CVSLL-Q-
XP_009106897.1/1-227 ----------------------GA--S-MENMLLIP-QR-Q-SE---G-R--CVSLL-Q-
XP_006401857.1/1-229 ----------------------GA--S-METMLLVP-KG-H-QE---G-R--CVSLL-R-
XP_020871109.1/1-230 ----------------------GA--P-MENTLLVP-KG-Q-RE---G-S--CVSLL-R-
NP_001332093.1/1-229 ----------------------GA--S-MENTLLVP-KG-Q-QE---G-S--CVSLL-R-
NP_200030.1/1-229    ----------------------GA--S-MENTLLVP-KG-Q-QE---G-S--CVSLL-R-
XP_006282099.1/1-225 ----------------------GA--S-MEITLLVP-KG-Q-QE---G-S--CVSLL-R-
XP_019095386.1/1-229 ----------------------GA--S-METTLLVP-KG-Q-HE---G-S--CVSLL-R-
XP_019095387.1/1-229 ----------------------GA--S-METTLLVP-KG-Q-HE---G-S--CVSLL-R-
XP_019091174.1/1-228 ----------------------GA--S-METTLLVP-KG-Q-RE---G-S--CVSLL-R-
XP_019091179.1/1-228 ----------------------GA--S-METTLLVP-KG-Q-RE---G-S--CVSLL-R-
XP_019087173.1/1-228 ----------------------GA--S-METTLLVP-KG-Q-RE---G-S--CVSLL-R-
XP_010555654.1/1-234 ----------------------GS--S-MQEMIRLP-KG-Q-HQ---G-N--CVSVL-R-
XP_010555656.1/1-234 ----------------------GS--S-MQEMIRLP-KG-Q-HQ---G-N--CVSVL-R-
XP_010555655.1/1-234 ----------------------GS--S-MQEMIRLP-KG-Q-HQ---G-N--CVSVL-R-
XP_010537199.1/1-242 ----------------------GG--T-MNEMVRIP-KG-RQHD---G-N--SVSLL-R-
XP_010537197.1/1-242 ----------------------GG--T-MNEMVRIP-KG-RQHD---G-N--SVSLL-R-
XP_010537198.1/1-241 ----------------------GG--T-MNEMVRIP-KG-RQHD---G-N--SVSLL-R-
Con_90%              ......................t......p.hh.ls.ph.p........p...lplh.p.
Con_80%              ......................ut....hp.hh.lu.ps.p........p..hlpll.+.
Con_70%              ......................Gs..s.hQthhtlA.su.p......t.s..tlsll.R.
Con_60%              ......................Gs..s.sQclsplA.sG.p..pp..u.N..slSll.R.
Con_50%              ......................Gu..s.sQElA+IA.sG.c..cp..G.N..slSlL.R.


XP_020582785.1/1-284 VES-----------------H---------------AFSQDN------V-L------S-R
XP_020682623.1/1-284 LES-----------------Q---------------TLNQDD------V-L------S-R
XP_020682624.1/1-284 LES-----------------Q---------------TLNQDD------V-L------S-R
XP_006846015.1/1-286 LEG-----------------H---------------GFSQDD------AVL------P-R
NP_001142394.1/1-289 LEG-----------------H---------------GFSHEE------VLM------S-R
NP_001146215.1/1-288 LQG-----------------H---------------GFSHDE------VLM------S-P
XP_020405718.1/1-288 LQG-----------------H---------------GFSHDE------VLM------S-P
XP_002443548.1/1-287 LEG-----------------H---------------GFSHEE------VLM------S-R
XP_025808876.1/1-287 LEG-----------------H---------------GFSHDE------VLM------S-R
XP_004963232.1/1-287 LEG-----------------H---------------GFSHDE------VLM------S-R
XP_020192001.1/1-285 LEG-----------------H---------------SLCHDE------VVL------S-R
XP_003562005.1/1-285 LEG-----------------H---------------SLCHDE------VVL------S-R
XP_004982339.1/1-286 LEG-----------------H---------------SLCHDE------VVL------S-R
XP_025796542.1/1-286 LEG-----------------H---------------SLCHDE------VVL------S-R
NP_001347456.1/1-286 LEG-----------------H---------------SLCHDE------VVL------S-R
NP_001352464.1/1-286 LEG-----------------H---------------SLCHDE------VVL------S-R
XP_002464180.1/1-286 LEG-----------------H---------------SLCHDE------VVL------S-R
XP_015689987.1/1-286 LEG-----------------H---------------SLCHDE------VVL------S-R
XP_015632484.1/1-286 LEG-----------------H---------------SLCHDE------VVL------S-R
XP_015620816.1/1-285 LEG-----------------H---------------GFSHDE------VLL------S-R
XP_006664738.1/1-285 LEG-----------------H---------------GFSHDE------VLL------S-R
XP_020190954.1/1-286 LEG-----------------H---------------GFSHEE------VLL------A-R
XP_020163543.1/1-286 LEG-----------------H---------------GFSHEE------VLL------A-R
XP_003577453.1/1-286 LEG-----------------H---------------GFSHDE------VLL------A-R
XP_020575104.1/1-285 LES-----------------P---------------GFTQED------V-L------S-R
XP_020696902.1/1-286 LEC-----------------P---------------GFNQED------V-L------S-R
XP_020578108.1/1-284 LEG-----------------H---------------GFSQDD------V--------A-R
XP_020698923.1/1-284 LEG-----------------H---------------GFSQDD------V--------A-R
XP_020096610.1/1-279 LEG-----------------P---------------GFNQDE------I-M------P-R
XP_009412508.1/1-287 IVD-----------------H---------------GFNQDD------V-L------S-R
XP_020271156.1/1-284 LEG-----------------H---------------GLNQDD------ILM------S-R
XP_020273236.1/1-287 LEG-----------------H---------------GFTQDD------VIM------S-R
XP_020092733.1/1-287 LEG-----------------H---------------GFHQDD------IVL------S-R
XP_019701934.1/1-288 LEG-----------------H---------------GFNQEE------VVL------S-R
XP_010905421.1/1-288 LEG-----------------H---------------GFNQEE------VVL------S-R
XP_010905420.1/1-289 LEG-----------------H---------------GFNQEE------VVL------S-R
XP_010923678.1/1-288 LEG-----------------H---------------GFNQEE------AVL------S-R
XP_008794452.1/1-288 LEG-----------------H---------------GFNQDE------VVL------S-R
XP_009397391.1/1-288 LEG-----------------H---------------GFNQDD------VIL------P-R
XP_009408551.1/1-288 LEG-----------------H---------------VFNQDD------VIL------S-R
XP_009399045.1/1-288 LEG-----------------H---------------GFNQDD------VIL------S-R
XP_009388964.1/1-288 LEG-----------------H---------------GFNQDD------VIL------S-R
XP_009397717.1/1-288 LEG-----------------N---------------GFNQDD------VIL------S-R
XP_018488876.1/1-280 LGG-----------------H---------------AYSPED------MGL------S-R
XP_013587486.1/1-280 LGG-----------------H---------------AYSPED------MGL------S-R
XP_009115139.1/1-280 LGG-----------------H---------------AYSPED------MGL------S-R
XP_013662459.1/1-280 LGG-----------------H---------------AYSPED------MGL------S-R
XP_013707293.1/1-280 LGG-----------------H---------------AYSPED------MGL------S-R
XP_006415465.2/1-280 LGG-----------------H---------------AYSPED------MGL------S-R
XP_019099288.1/1-280 LGG-----------------H---------------AYSPED------MGL------S-R
NP_174337.1/1-280    LGG-----------------H---------------AYSPED------MGL------S-R
XP_020867051.1/1-280 LGG-----------------H---------------AYSPED------MGL------S-R
XP_020867052.1/1-280 LGG-----------------H---------------AYSPED------MGL------S-R
XP_010460847.1/1-280 LGG-----------------H---------------AYSPED------MGL------S-R
XP_019094482.1/1-280 LGG-----------------H---------------AYSPED------MGL------S-R
XP_006306764.1/1-280 LGG-----------------H---------------AYSPED------MGL------S-R
XP_010538268.1/1-285 LEG-----------------H---------------AFSPEN------MGL------A-Q
XP_010519070.1/1-284 LEG-----------------H---------------AFSPED------MGL------A-R
XP_018436356.1/1-280 LEG-----------------H---------------AYSPED------MGL------A-R
XP_013637594.1/1-286 LEG-----------------H---------------AYSPED------MGL------A-R
XP_013692700.1/1-286 LEG-----------------H---------------AYSPED------MGL------A-R
XP_009143747.1/1-286 LEG-----------------H---------------AYSPED------MGL------A-R
XP_018484893.1/1-285 LEG-----------------H---------------AYSPED------MGL------A-R
XP_013637264.1/1-280 LEG-----------------H---------------AYSPED------MGL------A-R
XP_013637263.1/1-280 LEG-----------------H---------------AYSPED------MGL------A-R
XP_022557131.1/1-280 LEG-----------------H---------------AYSPED------MGL------A-R
XP_013729051.1/1-280 LEG-----------------H---------------AYSPED------MGL------A-R
XP_013744548.1/1-280 LEG-----------------H---------------AYSPED------VGL------A-R
XP_009141396.1/1-280 LEG-----------------H---------------AYSPED------VGL------A-R
XP_006410621.1/1-284 LEG-----------------H---------------AYSPED------MGL------A-R
NP_181018.1/1-284    LEG-----------------H---------------AYSPED------MGL------A-R
XP_020884308.1/1-284 LEG-----------------H---------------AYSPED------MGL------A-R
XP_006293359.1/1-284 LEG-----------------H---------------AYSPED------MGL------A-R
XP_010516626.1/1-284 LEG-----------------H---------------AYSPED------MGL------A-R
XP_010509720.1/1-284 LEG-----------------H---------------AYSPED------MGL------A-R
XP_010504942.1/1-284 LEG-----------------H---------------AYSPED------MGL------A-R
XP_017223336.1/1-283 LEN-----------------R-----------------APED------LTL------T-S
XP_016559371.1/1-285 LEG-----------------H---------------AFSPEN------IAL------S-R
XP_016559374.1/1-285 LEG-----------------H---------------AFSPEN------IAL------S-R
XP_012830207.1/1-281 LEG-----------------H---------------AFSPED------IAL------S-R
XP_012827547.1/1-287 LEG-----------------H---------------AFSPED------IAL------S-R
XP_022866806.1/1-284 LEG-----------------H---------------AFSPED------LAL------S-R
XP_022866805.1/1-285 LEG-----------------H---------------AFSPED------LAL------S-R
XP_023001017.1/1-285 LEG-----------------H---------------AMFPEE------AAL------GGR
XP_023001016.1/1-285 LEG-----------------H---------------AMFPEE------AAL------GGR
XP_023519837.1/1-285 LEG-----------------H---------------AMFPEE------AAL------GGR
XP_023519836.1/1-285 LEG-----------------H---------------AMFPEE------AAL------GGR
XP_022927139.1/1-285 LEG-----------------H---------------AMFPEE------AAL------GGR
XP_022927138.1/1-285 LEG-----------------H---------------AMFPEE------AAL------GGR
XP_022139982.1/1-285 LEG-----------------H---------------AMFPEE------AAL------AGR
XP_004135550.1/1-285 LEG-----------------H---------------AMFPEE------AAL------GGR
XP_008445207.1/1-285 LEG-----------------H---------------AMFPEE------AAL------GGR
XP_017230337.1/1-286 LEG-----------------H---------------AFSPED------IAL-------SR
XP_017230338.1/1-286 LEG-----------------H---------------AFSPED------IAL-------SR
XP_010316959.1/1-285 LEG-----------------H---------------AFSPED------IAL-------SR
XP_004233170.1/1-285 LEG-----------------H---------------AFSPED------IAL-------SR
XP_015066839.1/1-285 LEG-----------------H---------------AFSPED------IAL-------SR
XP_015066842.1/1-285 LEG-----------------H---------------AFSPED------IAL-------SR
XP_015066843.1/1-285 LEG-----------------H---------------AFSPED------IAL-------SR
XP_006353018.1/1-285 LEG-----------------H---------------AFSPED------IAL-------SR
XP_006353020.1/1-285 LEG-----------------H---------------AFSPED------IAL-------SR
XP_019178964.1/1-287 LEG-----------------H---------------AFSPED------IAL-------SR
XP_019178963.1/1-287 LEG-----------------H---------------AFSPED------IAL-------SR
XP_019228489.1/1-285 LEG-----------------P---------------AFSPED------IAL-------SR
XP_019228488.1/1-285 LEG-----------------P---------------AFSPED------IAL-------SR
NP_001289498.1/1-285 LEG-----------------P---------------AFSPED------IAL-------SR
XP_009777911.1/1-285 LEG-----------------P---------------AFSPED------IAL-------SR
XP_009612342.1/1-285 LEG-----------------P---------------AFSPED------IAL-------SR
XP_009612341.1/1-285 LEG-----------------P---------------AFSPED------IAL-------SR
XP_006363649.1/1-287 LEG-----------------P---------------AFSPED------IAL-------SR
XP_015066689.1/1-287 LEG-----------------P---------------AFSPED------IAL-------SR
XP_010315636.1/1-287 LEG-----------------P---------------AFSPED------IAL-------SR
XP_022879368.1/1-285 LEG-----------------H---------------AFSPEE------IAL-------SR
XP_020554026.1/1-285 LEG-----------------H---------------AFSPED------IAL-------SR
XP_011096678.1/1-285 LEG-----------------H---------------AFSPED------IAL-------SR
XP_011085207.1/1-285 LEG-----------------H---------------AFSPED------IAL-------SR
XP_023003193.1/1-285 LEG-----------------L---------------AFSPED------VAL------AGR
XP_022926723.1/1-285 LEG-----------------L---------------AFSPED------VAL------AGR
XP_023518237.1/1-285 LEG-----------------L---------------AFSPED------VAL------AGR
XP_022146537.1/1-286 LEG-----------------L---------------AFSPED------VAL------AGR
XP_011658319.1/1-286 LEG-----------------L---------------AFSPED------VAL------AGR
XP_008439554.1/1-286 LEG-----------------L---------------AFSPED------VAL------AGR
XP_022978372.1/1-286 LEG-----------------L---------------AFSPDD------VAL------AGR
XP_023543833.1/1-286 LEG-----------------L---------------AFSPDD------VAL------AGR
XP_022926042.1/1-286 LEG-----------------L---------------AFSPDD------VAL------AGR
XP_010247499.1/1-286 LEG-----------------H---------------GFPQED------AVI------P-R
XP_016717556.1/1-285 LEG-----------------Q---------------AFTPED------VAL------A-R
XP_012455108.1/1-285 LEG-----------------Q---------------AFTPED------VAL------A-R
XP_016717554.1/1-287 LEG-----------------Q---------------AFTPED------VAL------A-R
XP_012455107.1/1-287 LEG-----------------Q---------------AFTPED------VAL------A-R
XP_016711412.1/1-287 LEG-----------------Q---------------AFTPED------VAL------A-R
XP_017649973.1/1-287 LEG-----------------Q---------------AFTPED------VAL------A-R
XP_022716449.1/1-285 LEG-----------------H---------------AFTPED------VAL------A-R
XP_022724664.1/1-287 LEG-----------------H---------------AFTPED------VAL------A-R
XP_021298192.1/1-285 LEG-----------------H---------------AFTPED------VSL------A-R
XP_021298193.1/1-294 LEG-----------------H---------------AFTPED------VSL------A-R
XP_021298191.1/1-294 LEG-----------------H---------------AFTPED------VSL------A-R
XP_007022874.2/1-285 LEG-----------------H---------------AFTPED------VAL------A-R
XP_017979205.1/1-294 LEG-----------------H---------------AFTPED------VAL------A-R
XP_017979204.1/1-294 LEG-----------------H---------------AFTPED------VAL------A-R
XP_017236680.1/1-286 LEG-----------------H---------------AFSPED------MAL------S-R
XP_010051959.1/1-285 LEG-----------------H---------------AFSPED------IPL------A-H
XP_010051960.1/1-285 LEG-----------------H---------------AFSPED------IPL------A-H
XP_015882975.1/1-284 LEG-----------------H---------------TFSPED------VAL------A-R
XP_015882797.1/1-284 LEG-----------------H---------------TFSPED------VAL------A-R
XP_010098570.1/1-284 LEG-----------------H---------------AFSPEE------VAL------A-R
XP_011012084.1/1-284 LEG-----------------H---------------AFSPED------VAL------A-Q
XP_011012083.1/1-284 LEG-----------------H---------------AFSPED------VAL------A-Q
XP_006377667.2/1-284 LEG-----------------H---------------AFSPED------VAL------A-Q
XP_002281868.2/1-285 LEG-----------------H---------------AFSPED------VAL------T-R
XP_018847863.1/1-285 LEG-----------------H---------------AFSPED------VAL------A-R
XP_018847862.1/1-285 LEG-----------------H---------------AFSPED------VAL------A-R
XP_023919942.1/1-285 LEG-----------------H---------------AFSPED------VAL------A-R
XP_023919941.1/1-285 LEG-----------------H---------------AFSPED------VAL------A-R
XP_018828101.1/1-285 LEG-----------------H---------------AFSPED------VAL------A-R
XP_018828100.1/1-285 LEG-----------------H---------------AFSPED------VAL------A-R
XP_002298892.1/1-285 LEG-----------------H---------------AFSPED------VAL------A-R
XP_011038887.1/1-285 LEG-----------------H---------------AFSPED------VAL------A-R
XP_011038886.1/1-285 LEG-----------------H---------------AFSPED------VAL------A-R
XP_021657817.1/1-285 LEG-----------------H---------------AFSPED------VAL------A-R
XP_021657818.1/1-285 LEG-----------------H---------------AFSPED------VAL------A-R
XP_015577248.1/1-260 LEG-----------------H---------------AFSPED------VAL------A-R
XP_021616343.1/1-285 LEG-----------------H---------------AFSPED------VAL------A-Q
XP_021645103.1/1-285 LEG-----------------H---------------AFSPED------VAL------A-R
XP_021592762.1/1-285 LEG-----------------H---------------AFSPED------VAL------A-R
XP_012075627.1/1-285 LEG-----------------H---------------AFSPED------VAL------A-R
XP_008372224.1/1-285 LEG-----------------P---------------AFSPED------VAL------A-Q
XP_009362238.1/1-285 LEG-----------------P---------------AFSPED------VAL------A-Q
XP_009344405.1/1-284 LEG-----------------P---------------AFSPED------VAL------T-R
XP_008349150.1/1-284 LEG-----------------P---------------AFSPED------VAL------T-R
XP_007214622.1/1-285 LEG-----------------H---------------AFSPED------VAL------A-R
XP_021812393.1/1-285 LEG-----------------H---------------AFSPED------VAL------A-R
XP_024161164.1/1-285 LEG-----------------H---------------AYSPED------VAL------A-R
XP_004293989.1/1-285 LEG-----------------H---------------AYSPED------VAL------A-R
XP_019419795.1/1-282 IEG-----------------H---------------AFPPDD------VSL------A-R
XP_019426301.1/1-282 IEG-----------------H---------------AFPPED------MSL------A-R
XP_003524993.1/1-285 IEG-----------------N---------------AFPPDD------VAW------A-C
XP_003532788.1/1-285 IEG-----------------N---------------AFPPDD------VAL------A-C
XP_020215009.1/1-285 IEG-----------------H---------------AFPPDD------VAL------A-C
XP_017409410.1/1-285 IEG-----------------N---------------AFPPDD------VAL------A-C
XP_017409418.1/1-285 IEG-----------------N---------------AFPPDD------VAL------A-C
XP_014509941.1/1-285 IEG-----------------N---------------AFPPDD------VAL------A-C
XP_007159548.1/1-285 IEG-----------------N---------------AFPPDD------VAL------A-C
XP_024047818.1/1-288 LEG-----------------H---------------AFSPED------VAL------A-R
XP_006448915.2/1-288 LEG-----------------H---------------AFSPED------VAL------A-R
XP_006468302.1/1-288 LEG-----------------H---------------AFSPED------VAL------A-R
XP_006468303.1/1-287 LEG-----------------H---------------AFSPED------VAL------A-R
XP_024047817.1/1-287 LEG-----------------H---------------AFSPED------VAL------A-R
XP_004504217.1/1-285 IEG-----------------H---------------AFSPED------VAL------A-R
XP_003594520.2/1-281 IEG-----------------H---------------AFSPED------VAL------A-R
XP_024632601.1/1-281 IEG-----------------H---------------AFSPED------VAL------A-R
XP_004486450.1/1-285 IEG-----------------H---------------AFSPED------VAL------A-R
XP_007147487.1/1-285 IEG-----------------H---------------AFSPED------VTL------A-R
XP_017437412.1/1-285 IEG-----------------H---------------AFSPED------VTL------A-R
XP_014518680.1/1-285 IEG-----------------H---------------AFSPED------VTL------A-R
XP_003546255.1/1-285 IEG-----------------H---------------AFSPED------VAM------A-R
XP_020233213.1/1-285 IEG-----------------H---------------AFSPED------VAL------A-R
XP_003535078.1/1-285 IEG-----------------H---------------AFSPED------VAL------A-R
XP_014617344.1/1-285 IEG-----------------H---------------AFSPED------VAL------A-R
XP_015959291.1/1-283 IEG-----------------H---------------AFSPED------VAL------A-R
XP_016197724.1/1-283 IEG-----------------H---------------AFSPED------VAL------A-R
XP_025694278.1/1-283 IEG-----------------H---------------AFSPED------VAL------A-R
XP_006846777.1/1-281 LEG-----------------H---------------GFPQEE------AVV-------CR
XP_003574071.1/1-280 LEG-----------------Q---------------AL--DE------GLL-------SR
XP_020174778.1/1-278 LEG-----------------Q---------------AL--DE------GLL-------SR
XP_006661867.1/1-281 LEG-----------------Q---------------ALTHDD------GLL-------SR
XP_015614901.1/1-281 LEG-----------------Q---------------ALTHDD------GLM-------SR
XP_004982838.1/1-282 LEG-----------------Q---------------ALTHDD------GLL-------SR
XP_025796396.1/1-282 LEG-----------------Q---------------ALTHDE------GVL-------SR
NP_001316502.1/1-284 LEG-----------------Q---------------TLTHDE------GLL-------SR
XP_021306737.1/1-282 LEG-----------------Q---------------TLTHDE------GLL-------SR
XP_021306733.1/1-293 LEG-----------------Q---------------TLTHDE------GLL-------SR
XP_020162053.1/1-281 LEG-----------------Q---------------PLSHDE------VLL-------SR
XP_006649248.1/1-282 LEG-----------------Q---------------PLTHDE------ALL-------SR
XP_015632709.1/1-282 LEG-----------------Q---------------PLTHDE------ALL-------SR
NP_001296150.1/1-282 LEG-----------------Q---------------PLTHDE------ALL-------SR
XP_008665074.1/1-283 LEG-----------------Q---------------PLTHDE------ALL-------SR
NP_001105994.1/1-283 LEG-----------------Q---------------PLTHDE------ALL-------SR
XP_008660404.1/1-283 LEG-----------------Q---------------PLTHDE------ALL-------SR
XP_002468669.1/1-283 LEG-----------------Q---------------PLTHDE------ALL-------SR
XP_021309716.1/1-285 LEG-----------------Q---------------PLTHDE------ALL-------SR
XP_004986037.1/1-283 LEG-----------------Q---------------SLTHDE------ALL-------SR
XP_025795001.1/1-283 LEG-----------------Q---------------SLTHDE------ALL-------SR
XP_020093893.1/1-283 LED-----------------Q---------------AITQDE------AAS-------PR
XP_020093894.1/1-282 LED-----------------Q---------------AITQDE------AAS-------PR
XP_020259754.1/1-283 LEG-----------------Q---------------ALTQDE------AIL-------SR
XP_009401955.1/1-282 LEG-----------------Q---------------ALIQDG------AIL-------SR
XP_008791541.1/1-283 LEG-----------------Q---------------AVAQDE------AIL-------SR
XP_017698637.1/1-283 LEG-----------------Q---------------AVAQDE------AIL-------SR
XP_010920422.1/1-283 LEG-----------------Q---------------AVTQDE------AMV-------SR
XP_010920423.1/1-283 LEG-----------------Q---------------AVTQDE------AMV-------SR
XP_010920419.1/1-283 LEG-----------------Q---------------AVTQDE------AMV-------SR
XP_010920426.1/1-283 LEG-----------------Q---------------AVTQDE------AMV-------SR
XP_019709326.1/1-287 LEG-----------------Q---------------AITQDE--APGQAVL-------SK
XP_008785281.1/1-283 LEG-----------------Q---------------AITQDE------AIL-------SR
XP_008462593.1/1-288 LES-----------------H---------------PLAQED------AFM-------SR
XP_022969629.1/1-286 LES-----------------H---------------PLAQED------TFM-------SR
XP_023536970.1/1-286 LES-----------------H---------------PLAQED------TFM-------SR
XP_022937855.1/1-288 LES-----------------H---------------PLAQED------TFM-------SR
XP_022937858.1/1-288 LES-----------------H---------------PLAQED------TFM-------SR
XP_012827869.1/1-285 LEG-----------------H---------------SLSHED------AFM-------PR
XP_022874815.1/1-279 LEG-----------------D---------------PLSHED------AYI-------SR
XP_022874814.1/1-283 LEG-----------------D---------------PLSHED------AYI-------SR
XP_018434000.1/1-280 LEG-----------------H---------------SLVQEY------TSL-------SR
XP_013642496.1/1-279 LEG-----------------H---------------SLVPED------SFL-------SR
XP_009150087.1/1-279 LEG-----------------H---------------SLVPED------SFL-------SR
XP_009150088.1/1-279 LEG-----------------H---------------SLVPED------SFL-------SR
XP_022563181.1/1-279 LEG-----------------H---------------SLVPED------SFL-------TR
XP_013675790.1/1-280 LEG-----------------H---------------SLVPED------SFL-------TR
XP_013623090.1/1-279 LEG-----------------H---------------SLVPED------SFL-------TR
XP_013623089.1/1-280 LEG-----------------H---------------SLVPED------SFL-------TR
XP_020885782.1/1-285 LEG-----------------H---------------SLAQED------AFM-------SR
XP_006280004.1/1-285 LEG-----------------H---------------SLAQED------AFM-------SR
XP_019088563.1/1-285 LEG-----------------H---------------SLAQED------AYM-------SR
XP_010483719.1/1-285 LEG-----------------H---------------SLAQED------AFM-------SR
XP_010456679.1/1-285 LEG-----------------H---------------SLAQED------AFM-------SR
XP_006400821.1/1-284 LEG-----------------H---------------SLAQED------AFM-------SR
NP_200877.1/1-284    LEG-----------------H---------------SLAQED------AFM-------SR
XP_013611543.1/1-282 LEG-----------------H---------------SLAQED------AFM-------SR
XP_009120605.1/1-284 LEG-----------------H---------------SLAQED------AFM-------SR
XP_013667152.1/1-284 LEG-----------------H---------------SLAQED------AFM-------SR
XP_018446535.1/1-284 LEG-----------------H---------------SLAQED------AFM-------SR
XP_018454374.1/1-284 LEG-----------------H---------------SLAQED------GFM-------SR
XP_009126588.1/1-285 LEG-----------------H---------------SLAQED------AFM-------SR
XP_013680521.1/1-285 LEG-----------------H---------------SLAQED------AFM-------SR
XP_013612427.1/1-285 LEG-----------------H---------------SLAQED------AFM-------SR
XP_010535608.1/1-283 LEG-----------------H---------------SLVQDD------AFV-------SR
XP_010520372.1/1-276 LEG-----------------H---------------SLVQED------AFV-------SR
XP_004487103.1/1-286 LEG-----------------H---------------SLAQED------AFV-------SR
XP_003597252.1/1-283 LEG----------------------------------LAQDD------SFV-------SR
XP_024455683.1/1-286 LEG-----------------H---------------SFAQED------AFV-------SQ
XP_022026352.1/1-288 LEG-----------------H---------------ALGQED------PFT-------SR
XP_022000486.1/1-288 LEG-----------------Q---------------PLGNED------PFT-------SR
XP_016717477.1/1-281 LEG-----------------Q---------------SLTQED------AFL-------SR
XP_017643014.1/1-281 LEG-----------------Q---------------SLTQED------AFL-------SR
XP_016724213.1/1-281 LEG-----------------Q---------------SLTQED------AFL-------SR
XP_012462938.1/1-281 LEG-----------------Q---------------SLTQED------AFL-------SR
XP_012471509.1/1-292 LEG-----------------H---------------SLVQED------AFV-------SR
XP_017642742.1/1-292 LEG-----------------H---------------SLVQED------AFV-------SR
XP_016755876.1/1-292 LEG-----------------H---------------SLVQED------AFV-------SR
XP_012471510.1/1-286 LEG-----------------H---------------SLVQED------AFV-------SR
XP_016753711.1/1-286 LEG-----------------H---------------SLVQED------AFV-------SR
XP_017642744.1/1-286 LEG-----------------H---------------SLVQED------AFV-------SR
XP_016755877.1/1-286 LEG-----------------H---------------SLVQED------AFV-------SR
XP_016750468.1/1-286 LEG-----------------H---------------SFVQED------AFV-------SR
XP_016688657.1/1-286 LEG-----------------H---------------SFVQED------AFV-------SR
XP_012443272.1/1-286 LEG-----------------H---------------SFVQED------AFV-------SR
XP_017603001.1/1-286 LEG-----------------H---------------SFVQED------AFV-------SR
XP_022755632.1/1-285 LEG-----------------H---------------SSALED------AFL-------SG
XP_022755633.1/1-285 LEG-----------------H---------------SSALED------AFL-------SG
XP_022729815.1/1-286 LEG-----------------H---------------SLVQED------AFL-------SR
XP_022729816.1/1-286 LEG-----------------H---------------SLVQED------AFL-------SR
XP_022752857.1/1-286 LEG-----------------H---------------SLAQED------AFL-------SR
XP_022752856.1/1-286 LEG-----------------H---------------SLAQED------AFL-------SR
XP_021277981.1/1-286 LEG-----------------H---------------SLAQED------AFL-------SR
XP_017983554.1/1-286 LEG-----------------H---------------SLAQED------AFL-------SR
XP_011089912.1/1-283 LEG-----------------H---------------SLSHED------AFG-------SR
XP_007208090.1/1-286 LEG-----------------H---------------SLTQED------AFA-------SR
XP_008225704.1/1-286 LEG-----------------H---------------SLTQED------AFA-------SR
XP_021830853.1/1-286 LEG-----------------H---------------SLTQED------AFA-------SR
XP_008349754.1/1-286 LEG-----------------H---------------SLTQED------AFA-------SR
NP_001315783.1/1-288 LEG-----------------H---------------SLTQED------AFA-------SR
XP_009367069.1/1-286 LEG-----------------H---------------SLTQED------AFA-------SR
XP_008344872.1/1-286 LEG-----------------H---------------SLTQED------AFA-------SR
XP_015952399.1/1-286 LEG-----------------H---------------SLAQED------AFA-------SR
XP_020982118.1/1-286 LEG-----------------H---------------SLAQED------AFA-------SR
XP_025685402.1/1-286 LEG-----------------H---------------SLAQED------AFA-------SR
XP_016187430.1/1-286 LEG-----------------H---------------SLAQED------AFA-------SR
XP_020214107.1/1-284 LEG-----------------H---------------SLAQED------AFV-------SR
XP_006592258.1/1-284 LEG-----------------H---------------SLAQED------AFV-------SR
XP_007132262.1/1-285 LEG-----------------H---------------SLAQED------AFV-------SR
XP_003538150.1/1-285 LEG-----------------H---------------SLAQED------AFV-------SR
XP_017433493.1/1-285 LEG-----------------H---------------SLAQED------AFV-------SR
XP_014494018.1/1-285 LEG-----------------H---------------SLAQED------AFV-------SR
XP_010089983.2/1-286 LEG-----------------H---------------SLAQED------AFL-------SR
XP_006378974.2/1-285 LEG-----------------H---------------SFAQED------AFV-------SR
XP_011010051.1/1-285 LEG-----------------H---------------SFAQED------AFV-------SR
XP_011010050.1/1-285 LEG-----------------H---------------SFAQED------AFV-------SR
XP_021905657.1/1-285 LEG-----------------H---------------SLSQED------AFV-------SR
XP_021610391.1/1-286 LEG-----------------H---------------SLAQED------AFI-------SR
XP_002529946.1/1-286 LEG-----------------H---------------SLVQED------AFV-------SR
XP_021658728.1/1-286 LEG-----------------H---------------SLAQED------AFI-------SR
XP_012064816.1/1-286 LEG-----------------H---------------SLAQED------AFV-------SR
XP_021628343.1/1-286 LEG-----------------H---------------SLVQED------AFV-------SR
XP_021657859.1/1-286 LEG-----------------H---------------SLLQED------AFV-------SR
XP_015874442.1/1-285 LEG-----------------H---------------SLAQED------AFV-------SR
XP_023926843.1/1-286 LEG-----------------H---------------SLPQED------AFA-------SR
XP_018819529.1/1-286 LEG-----------------H---------------LLPQED------AFA-------SR
XP_010651164.1/1-286 LEG-----------------H---------------SLAHED------AFM-------SR
XP_024034672.1/1-286 LEG-----------------H---------------SLAQED------AFV-------SR
XP_006488084.1/1-286 LEG-----------------H---------------SLAQED------AFV-------SR
XP_024034673.1/1-286 LEG-----------------H---------------SLAQED------AFV-------SR
XP_010264677.1/1-287 LEG-----------------H---------------ALTQEE------AVL-------SR
XP_010253680.1/1-288 LEG-----------------H---------------ALTQEE------AIF-------SR
XP_022005829.1/1-282 LEG-----------------H---------------AVGQED------PFV-------SR
XP_023751537.1/1-278 LEG-----------------H---------------AVGQED------PFV-------SR
XP_024971452.1/1-283 LEG-----------------H---------------ALGQED------PFV-------SR
XP_024971444.1/1-287 LEG-----------------H---------------ALGQED------PFV-------SR
XP_024985647.1/1-287 LEG-----------------H---------------ALGQED------PFM-------SR
XP_023749448.1/1-288 LEG-----------------H---------------ALGQED------SFM-------SR
XP_010689538.1/1-281 LEG-----------------H---------------SLTPED------AYI-------SR
XP_021858740.1/1-280 LEG-----------------H---------------SLSPED------AYI-------SR
XP_021773317.1/1-281 LEG-----------------H---------------SLTPED------AYI-------SR
XP_021745941.1/1-281 LEG-----------------H---------------SLTPED------AYI-------SR
XP_016729039.1/1-286 LEG-----------------H---------------SFAEDN------AFL-------SR
XP_017623828.1/1-286 LEG-----------------H---------------SFAEDN------AFL-------SR
XP_012476406.1/1-286 LEG-----------------H---------------SFAEDN------AFL-------SR
XP_016754776.1/1-286 LEG-----------------H---------------SFAEDN------AFL-------SR
XP_022152944.1/1-286 LES-----------------H---------------PLAQED------AFM-------SG
XP_011040884.1/1-286 LEG-----------------H---------------SFAQED------AFV-------SQ
XP_010043753.1/1-286 LEG-----------------H---------------SLAQED------AFV-------SR
XP_024158223.1/1-285 LEG-----------------H---------------SFSQEE------AFA-------SR
XP_004291573.1/1-285 LEG-----------------H---------------SFSQEE------AFA-------SR
XP_018808765.1/1-285 LES-----------------H---------------CLSQED------AFV-------SR
XP_023512455.1/1-283 LEG-----------------H---------------PMVQED------AFV-------SR
XP_023512456.1/1-283 LEG-----------------H---------------PMVQED------AFV-------SR
XP_022944186.1/1-283 LEG-----------------H---------------PMVQED------AFV-------SR
XP_022944185.1/1-283 LEG-----------------H---------------PMVQED------AFV-------SR
XP_022985614.1/1-283 LEG-----------------H---------------PMVQED------AFV-------SR
XP_022985615.1/1-283 LEG-----------------H---------------PMVQED------AFV-------SR
XP_011656996.1/1-283 LEG-----------------H---------------PMVQED------AFV-------SR
XP_011656997.1/1-283 LEG-----------------H---------------PMVQED------AFV-------SR
XP_008463945.1/1-283 LEG-----------------H---------------PMVQED------AFV-------SR
XP_008463946.1/1-283 LEG-----------------H---------------PMVQED------AFV-------SR
XP_022140363.1/1-286 LEG-----------------H---------------PMVQED------AFV-------SR
XP_022995706.1/1-286 LEG-----------------H---------------PMVQED------AFV-------SR
XP_023534373.1/1-286 LEG-----------------H---------------PMVQED------AFV-------SR
XP_022958713.1/1-286 LEG-----------------H---------------PMVQED------AFV-------SR
XP_025614062.1/1-285 LEG-----------------H---------------SLAQED------AFA-------SR
XP_025614063.1/1-285 LEG-----------------H---------------SLAQED------AFA-------SR
XP_016170485.1/1-285 LEG-----------------H---------------SLAQED------AFA-------SR
XP_016170486.1/1-285 LEG-----------------H---------------SLAQED------AFA-------SR
XP_015935757.1/1-285 LEG-----------------H---------------SLAQED------AFA-------SR
XP_015935758.1/1-285 LEG-----------------H---------------SLAQED------AFA-------SR
XP_004505999.1/1-286 LEG-----------------H---------------SLAQED------AFV-------SR
XP_013456006.1/1-286 LEG-----------------H---------------SLAQED------AFV-------SR
XP_019453966.1/1-285 LEG-----------------H---------------SLAQED------TFV-------SR
XP_019448740.1/1-279 LEG-----------------H---------------SLAEED------TFV-------SR
XP_019448741.1/1-279 LEG-----------------H---------------SLAEED------TFV-------SR
XP_019458095.1/1-286 LEG-----------------H---------------SLAQED------AFV-------SR
XP_019447870.1/1-286 LEG-----------------H---------------SLAQED------AFV-------SR
XP_012838629.1/1-280 LEG-----------------Q---------------PLNHED------AFA-------SR
XP_012838630.1/1-280 LEG-----------------Q---------------PLNHED------AFA-------SR
XP_012838627.1/1-280 LEG-----------------Q---------------PLNHED------AFA-------SR
XP_012838628.1/1-280 LEG-----------------Q---------------PLNHED------AFA-------SR
XP_017214964.1/1-286 LEG-----------------Y---------------PLSQED------AFM-------SR
XP_017234817.1/1-286 LEG-----------------H---------------PLGQED------SFM-------SR
XP_019178153.1/1-282 LEG-----------------H---------------SLGHEN------AFI-------SG
XP_019166562.1/1-283 LEG-----------------H---------------PLDNED------AFL-------SR
XP_011089294.1/1-282 LEG-----------------H---------------SLSHED------AFM-------SR
XP_022892122.1/1-274 LEG-----------------H---------------SLGQED------AFI-------SR
XP_022883393.1/1-279 LEG-----------------H---------------SLGEED------AFI-------SR
XP_022883398.1/1-279 LEG-----------------H---------------SLGEED------AFI-------SR
XP_022883384.1/1-283 LEG-----------------H---------------SLGEED------AFI-------SR
XP_022883389.1/1-283 LEG-----------------H---------------SLGEED------AFI-------SR
XP_009776589.1/1-283 LEG-----------------H---------------SIGQED------AFM-------PR
NP_001312504.1/1-283 LEG-----------------H---------------SIGQED------TFM-------PR
XP_016472209.1/1-283 LEG-----------------H---------------SIGQED------TFM-------PR
XP_019255429.1/1-283 LEG-----------------H---------------SIGQED------AFM-------PR
XP_009624234.1/1-283 LEG-----------------H---------------STGQED------AFM-------PR
XP_016504967.1/1-283 LEG-----------------H---------------SIGQED------AFM-------PR
XP_016547131.1/1-283 LEG-----------------H---------------SIGQED------AFM-------PR
XP_004251138.1/1-283 LEG-----------------H---------------SIGQED------AFM-------PR
XP_015058512.1/1-283 LEG-----------------H---------------SIGQED------AFM-------PR
XP_006340193.1/1-283 LEG-----------------H---------------SIGQED------AFM-------PR
XP_004235650.1/1-283 LE----------------------------------GHSPED------AIM-------PR
XP_006343047.1/1-283 LE----------------------------------GHSPED------AIM-------PR
XP_009774805.1/1-285 LEG-----------------V---------------GHSPED------AIM-------PR
XP_016498616.1/1-285 LEG-----------------V---------------GHSPED------AIM-------PR
XP_016434103.1/1-285 LEG-----------------V---------------GHSPED------TIM-------PR
XP_009593967.1/1-285 LEG-----------------V---------------GHSPED------TIM-------PR
XP_004510297.1/1-285 LDG-----------------V---------------SHSPED------AML-------PR
XP_004510296.1/1-285 LDG-----------------V---------------SHSPED------AML-------PR
XP_003627005.1/1-284 LDG-----------------V---------------AHSPEE-------MM-------AR
XP_003531653.1/1-284 LEG-----------------V---------------AHSPDD------TIM-------PR
XP_006585576.1/1-288 LEG-----------------V---------------AHSPDD------TIM-------PR
XP_014634618.1/1-288 LEG-----------------V---------------AHSPDD------TIM-------PR
XP_014634619.1/1-288 LEG-----------------V---------------AHSPDD------TIM-------PR
XP_003531652.1/1-285 LEG-----------------I---------------AHSPED------TIM-------PR
XP_003530109.1/1-284 LEG-----------------I---------------AHSPED------TIM-------PR
XP_007135643.1/1-285 LEG-----------------I---------------AHSPED------TIM-------PR
XP_003530112.1/1-285 LEG-----------------I---------------AHSPED------TIM-------PR
XP_020226142.1/1-285 LEG-----------------V---------------AHSPED------TIM-------PR
XP_014516466.1/1-285 LEG-----------------V---------------VHSPED------AIM-------PR
XP_017407594.1/1-285 LEG-----------------V---------------VHSPED------TIM-------PR
XP_019448178.1/1-285 LEG-----------------V---------------AHSPED------AIM-------PR
XP_019429328.1/1-285 LEG-----------------I---------------AHSPEE------AMM-------PR
XP_004486776.1/1-285 LEG-----------------I---------------AHSPED------TIM-------PR
XP_003597690.1/1-286 LEG-----------------I---------------AHSPED------AIM-------PR
XP_015932679.1/1-285 LEG-----------------I---------------AHSPEE------AMM-------PR
XP_025674177.1/1-285 LEG-----------------I---------------AHSPEE------AMM-------PR
XP_025610204.1/1-285 LEG-----------------I---------------AHSPEE------AMM-------PR
XP_020226055.1/1-285 LEG-----------------I---------------ASSPEE------TIM-------PR
XP_007150614.1/1-285 LEG-----------------I---------------AHSPED------AIM-------PR
XP_014501306.1/1-285 LEG-----------------I---------------AHSPED------AIM-------PR
XP_017425270.1/1-285 LEG-----------------I---------------AHSPED------AIM-------PR
XP_021836126.1/1-285 LEG-----------------V---------------VSCPED------AMM-------PR
XP_010691423.1/1-284 LEG-----------------V---------------VSCPED------AMM-------PR
XP_010691424.1/1-284 LEG-----------------V---------------VSCPED------AMM-------PR
XP_021765330.1/1-284 LEG-----------------V---------------VSCPED------AMM-------PR
XP_021749885.1/1-284 LEG-----------------V---------------VSCPED------AMM-------PR
XP_010267183.1/1-285 LES-----------------I---------------GHCQED------PMM-------PR
XP_017258627.1/1-285 LEG-----------------A---------------GHCPEE------AIM-------GR
XP_023000315.1/1-284 LEG-----------------V---------------GHTPED------AMI-------PR
XP_023000313.1/1-284 LEG-----------------V---------------GHTPED------AMI-------PR
XP_023514554.1/1-284 LEG-----------------V---------------GHTPED------AMI-------PR
XP_023514552.1/1-284 LEG-----------------V---------------GHTPED------AMI-------PR
XP_022964458.1/1-284 LEG-----------------V---------------GHTPED------AMI-------PR
XP_022964454.1/1-284 LEG-----------------V---------------GHTPED------AMI-------PR
XP_022147627.1/1-284 LEG-----------------V---------------GHTPED------AMM-------PR
XP_022147619.1/1-284 LEG-----------------V---------------GHTPED------AMM-------PR
XP_008462437.1/1-284 LEG-----------------I---------------GHTPED------AMM-------PR
XP_004141659.1/1-284 LEG-----------------I---------------GHTPED------AMM-------PR
XP_008462436.1/1-284 LEG-----------------I---------------GHTPED------AMM-------PR
XP_011659682.1/1-284 LEG-----------------I---------------GHTPED------AMM-------PR
XP_022992111.1/1-284 LEG-----------------I---------------GHTPED------AMM-------PR
XP_023548534.1/1-284 LEG-----------------I---------------GHTPEE------AMM-------PR
XP_022953371.1/1-284 LEG-----------------I---------------GHTPEE------AMM-------PR
XP_002304217.1/1-285 LEG-----------------V---------------GHSPED------PIM-------PR
XP_011013041.1/1-285 LEG-----------------V---------------GHSPED------PIM-------PR
XP_011013042.1/1-285 LEG-----------------V---------------GHSPED------PIM-------PR
XP_011046759.1/1-284 LEG-----------------V---------------CHSPED------AIM-------PR
XP_024464085.1/1-284 LEG-----------------V---------------CHSPED------AIM-------PR
XP_024183498.1/1-286 LEG-----------------I---------------GHSPED------PMM------QTR
XP_004302866.1/1-286 LEG-----------------I---------------GHSPDD------PMM------QTR
NP_001280898.1/1-285 LEG-----------------V---------------GHSPED------AMM-------PR
XP_007214960.1/1-285 LEG-----------------V---------------GHSPED------AMM-------PR
XP_008228437.1/1-285 LEG-----------------V---------------GHSPED------AMM-------PR
XP_021818763.1/1-285 LEG-----------------V---------------GHSPED------AMM-------PR
XP_022723515.1/1-285 LEG-----------------I---------------VHSPED------TIM-------PR
XP_016688508.1/1-285 LEG-----------------I---------------AQSPED------ALM-------PR
XP_012443274.1/1-285 LEG-----------------I---------------AQSPED------ALM-------PR
XP_022721457.1/1-285 LEG-----------------M---------------VHSPED------AIM-------PR
XP_022715018.1/1-285 LEG-----------------I---------------ANSPED------AIM-------PR
XP_017979082.1/1-285 LEG-----------------V---------------AHSPED------AIM-------PR
XP_021293768.1/1-285 LEG-----------------V---------------AHSPED------AIM-------PR
XP_010062518.1/1-285 LEG-----------------M---------------GHSPED------ALM-------PR
XP_006426893.1/1-285 LEG-----------------V---------------GHSPED------AIM-------PR
XP_021610318.1/1-285 LEG-----------------M---------------GHSPED------AIM-------PR
XP_010098130.1/1-285 LEG-----------------V---------------GHSPED------AMM-------PR
XP_021635282.1/1-296 LEG-----------------V---------------GHSPED------PVM-------SR
XP_002284003.2/1-285 LEG-----------------V---------------GHCPED------AMM-------PR
XP_002515977.1/1-285 LEG-----------------A---------------GHSPED------PIM-------PR
XP_021643260.1/1-285 LEG-----------------V---------------GHSPED------PMM-------PR
XP_021628194.1/1-285 LEG-----------------V---------------SHSPED------PMM-------PR
XP_015894591.1/1-285 LEG-----------------V---------------GHSPED------AIM-------PR
XP_012069098.1/1-285 LEG-----------------V---------------GHSPED------PIM-------PR
XP_018847857.1/1-285 LEG-----------------V---------------GPTPED------TIM-------PR
XP_023898511.1/1-285 LEG-----------------V---------------GHSPED------AIM-------PR
XP_010545673.1/1-285 LEG-----------------L---------------GHSPED------AIV-------PR
XP_010545670.1/1-286 LEG-----------------L---------------GHSPED------AIV-------PR
XP_010535179.1/1-285 LEG-----------------L---------------GHSPED------AMI-------PR
XP_018436929.1/1-283 LEG-----------------L---------------GHSPED------AII-------PR
XP_013592012.1/1-283 LEG-----------------L---------------GHSPED------AIV-------PR
XP_013640375.1/1-284 LEG-----------------L---------------GHSPED------AIV-------PR
XP_013640376.1/1-283 LEG-----------------L---------------GHSPED------AIV-------PR
XP_009147709.1/1-283 LEG-----------------L---------------GHSPED------AIV-------PR
XP_006306297.1/1-281 LEG-----------------L---------------GHSPED------AIV-------PR
XP_010462051.1/1-281 LEG-----------------L---------------GHSPED------AIV-------PR
XP_010479714.1/1-281 LEG-----------------L---------------GHSPED------AIV-------PR
XP_010462052.1/1-281 LEG-----------------L---------------GHSPED------AIV-------PR
XP_010500807.1/1-281 LEG-----------------L---------------GHSPED------AIV-------PR
NP_849795.1/1-282    LEG-----------------L---------------GHSPED------AIV-------PR
NP_001077702.1/1-281 LEG-----------------L---------------GHSPED------AIV-------PR
NP_175627.1/1-281    LEG-----------------L---------------GHSPED------AIV-------PR
XP_020868586.1/1-281 LEG-----------------L---------------GHSPED------AIV-------PR
XP_006392953.1/1-282 LEG-----------------L---------------GHSPED------AIV-------PR
XP_018460807.1/1-282 LEG-----------------L---------------GHSPED------AIV-------PK
XP_013621760.1/1-283 LEG-----------------L---------------GHSPED------AIV-------PR
XP_018451524.1/1-282 LEG-----------------L---------------GHSPED------AIV-------PR
XP_009107148.1/1-282 LEG-----------------L---------------GQSPED------AIV-------PR
XP_009107147.1/1-283 LEG-----------------L---------------GQSPED------AIV-------PR
XP_009600774.1/1-284 LEG-----------------V---------------CHSPED------VIM-------PR
XP_016459079.1/1-284 LEG-----------------V---------------CHSPED------VIM-------PR
XP_019236029.1/1-285 LEG-----------------V---------------CHSPED------VIM-------QR
XP_009771098.1/1-285 LEG-----------------V---------------CHSPED------VIM-------PR
XP_016550962.1/1-285 LEG-----------------V---------------CHSPED------VIM-------PR
XP_016550960.1/1-285 LEG-----------------V---------------CHSPED------VIM-------PR
XP_016550961.1/1-285 LEG-----------------V---------------CHSPED------VIM-------PR
XP_006359469.1/1-285 LEG-----------------V---------------CHSPED------VIM-------PR
XP_004252354.1/1-285 LEG-----------------V---------------CHSPED------VIM-------PR
XP_015061298.1/1-285 LEG-----------------V---------------FHSPED------VIM-------PR
XP_019198775.1/1-285 FEG-----------------N---------------GHSPDD------AIM-------PR
XP_019194412.1/1-285 FEG-----------------I---------------GHSPEE------AMM-------PR
XP_016562656.1/1-283 LE----------------------------------GHSPED------AIM-------PR
XP_019267274.1/1-285 LEG-----------------V---------------GHSPED------AIM-------PR
XP_019267275.1/1-282 LEG-----------------V---------------GHSPED------AIM-------PR
XP_022889649.1/1-285 LEV-----------------G---------------GHSPED------AIM-------PR
XP_022889648.1/1-285 LEV-----------------G---------------GHSPED------AIM-------PR
XP_012852689.1/1-281 LEG-----------------V---------------GQSPED------SMM-------PR
XP_012856468.1/1-279 LEG-----------------V---------------VQSPED------AMM-------PR
XP_011080516.1/1-285 LEG-----------------V---------------AHSPED------AMM-------PR
XP_011072422.1/1-285 LEG-----------------V---------------AHSPED------AMM-------PR
XP_022851148.1/1-285 LEG-----------------V---------------GHSPED------AML-------PR
XP_022844668.1/1-285 LEG-----------------V---------------GHSPED------AML-------PR
XP_006853643.2/1-288 LEN-----------------H---------------GLVQDD------SII-------PR
XP_010249545.1/1-285 LES-----------------V---------------GPCQDN------SVM-------PN
XP_010249539.1/1-285 LES-----------------V---------------GPCQDN------SVM-------PN
XP_010915409.1/1-284 LEN-----------------T---------------GR-SQD------ALM-------AR
XP_008791633.1/1-284 LEN-----------------I---------------GR-SQD------ALM-------PR
XP_017235966.1/1-285 LEG-----------------M---------------VPCPED------SIM-------PR
XP_023761873.1/1-288 LEG-----------------V---------------SNCPED------ALM-------SR
XP_021997125.1/1-286 LEG-----------------V---------------GLCPED------ALM-------GR
XP_021990052.1/1-285 PEG-----------------V---------------GLCPED------ALM-------GR
XP_024979339.1/1-285 LEG-----------------V---------------SHCPED------ALM-------AR
XP_024979341.1/1-290 LEG-----------------V---------------SHCPED------ALM-------AR
XP_024979340.1/1-291 LEG-----------------V---------------SHCPED------ALM-------AR
XP_024979342.1/1-291 LEG-----------------V---------------SHCPED------ALM-------AR
XP_022016208.1/1-285 MES-----------------V---------------EHCPED------ALI-------PR
XP_022016207.1/1-311 MES-----------------V---------------EHCPED------ALI-------PR
XP_022009819.1/1-285 MEG-----------------V---------------GHCAED------ALV-------SG
XP_022009815.1/1-285 MEG-----------------V---------------GHCAED------ALV-------SG
XP_022009809.1/1-285 MEG-----------------V---------------GHCAED------ALV-------SG
XP_023759500.1/1-286 LEG-----------------V---------------DHSPED------ALM-------PR
XP_023751740.1/1-285 MEG-----------------I---------------GHCPED------ALM-------PR
XP_024971803.1/1-285 LEG-----------------V---------------EHCPED------SLM-------PR
XP_022033649.1/1-285 MEG-----------------V---------------GHCPEE------SLI-------PR
XP_024972195.1/1-285 MEG-----------------V---------------GHCPED------GLM-------PR
XP_024972196.1/1-289 MEG-----------------V---------------GHCPED------GLM-------PR
XP_020248539.1/1-284 LEN-----------------I---------------GHIQ--------DTL------MPR
XP_020269197.1/1-284 LEN-----------------I---------------GHIH--------DAP------MPR
XP_020269196.1/1-284 LEN-----------------I---------------GHIH--------DAP------MPR
XP_009399813.1/1-284 FES-----------------I---------------GNNR--------EMP------MPR
XP_009399812.1/1-284 FES-----------------I---------------GNNR--------EMP------MPR
XP_009413853.1/1-284 LEN-----------------F---------------GHNQ--------ETL------MPQ
XP_024985513.1/1-286 LEN-----------------M---------------SNYRAE------DML------VPG
XP_023729871.1/1-286 LEN-----------------M---------------SHYRAE------DML------MPA
XP_022000974.1/1-282 LEN-----------------I---------------SHYRAE------DML------MPG
XP_022023495.1/1-282 LEN-----------------T---------------SHYRAD------DML------MPG
XP_010526102.1/1-286 LEG-----------------L---------------GHYQ-D------DMI------MPA
XP_019086035.1/1-282 LES-----------------L---------------GHYQ-E------DMM------MPA
XP_010437837.1/1-288 LES-----------------L---------------GHYQ-E------DMM------MPA
XP_010437838.1/1-288 LES-----------------L---------------GHYQ-E------DMM------MPA
XP_022549566.1/1-283 LES-----------------L---------------GHYQ-E------DMM------MPA
XP_013670651.1/1-283 LES-----------------L---------------GHYQ-E------DMM------MPA
XP_020874560.1/1-283 LES-----------------L---------------GHYQ-E------DMM------MPA
XP_020874561.1/1-283 LES-----------------L---------------GHYQ-E------DMM------MPA
XP_020874562.1/1-283 LES-----------------L---------------GHYQ-E------DMM------MPA
NP_195014.1/1-283    LES-----------------L---------------GHYQ-E------DMM------MPA
XP_006412419.1/1-283 LES-----------------L---------------GHYQ-E------DMM------MPA
XP_006412418.1/1-283 LES-----------------L---------------GHYQ-E------DMM------MPA
XP_018471819.1/1-283 LES-----------------L---------------GHYQ-E------DMM------MPA
XP_009125299.1/1-283 LES-----------------L---------------GHYQ-E------DMM------MPA
XP_010432643.1/1-283 LES-----------------L---------------GHYQ-E------DMM------MPA
XP_010432644.1/1-283 LES-----------------L---------------GHYQ-E------DMM------MPA
XP_006283109.1/1-283 LES-----------------L---------------GHYQ-E------DMM------MPA
XP_010437839.1/1-283 LES-----------------L---------------GHYQ-E------DMM------MPA
XP_010437840.1/1-283 LES-----------------L---------------GHYQ-E------DMM------MPA
XP_010447325.1/1-283 LES-----------------L---------------GHYQ-E------DMM------MPA
XP_010447326.1/1-283 LES-----------------L---------------GHYQ-E------DMM------MPA
XP_013622315.1/1-283 LES-----------------L---------------GHYQ-E------DMM------MPA
XP_013656273.1/1-283 LES-----------------L---------------GHYQ-E------DMM------MPA
XP_009108824.1/1-283 LES-----------------L---------------GHYQ-E------DMM------MPA
XP_013730326.1/1-283 LES-----------------L---------------GHYQ-E------DMM------MPA
XP_013730325.1/1-283 LES-----------------L---------------GHYQ-E------DMM------MPA
XP_018447492.1/1-283 LKS-----------------L---------------GHYQ-E------DMM------MPA
XP_019415259.1/1-283 LEN-----------------M---------------GYYR-D------DMT------IPG
XP_022980940.1/1-286 FEN-----------------V---------------GHYR-D------DML------MSG
XP_023523539.1/1-286 FEN-----------------V---------------GHYR-D------DML------MSG
XP_022941184.1/1-286 FEN-----------------V---------------GHYR-D------DML------MSG
XP_022134366.1/1-286 FEN-----------------V---------------GHFR-D------DML------MPG
XP_004136224.1/1-286 FEN-----------------V---------------GHYR-D------DML------MPG
XP_008466070.1/1-286 FEN-----------------V---------------GHYR-D------DML------MPG
XP_022976315.1/1-286 FEN-----------------V---------------SHYR-D------DKL------MPG
XP_022937345.1/1-286 FEN-----------------V---------------SHYS-D------DKL------MPG
XP_023534956.1/1-286 FEN-----------------M---------------SHYR-D------DKL------MPG
XP_023535295.1/1-286 FEN-----------------V---------------SHYR-D------DKL------MPG
XP_019175908.1/1-285 LEN-----------------M---------------GHFR-E------DML------TAG
XP_019175907.1/1-285 LEN-----------------M---------------GHFR-E------DML------TAG
XP_009626659.1/1-285 LES-----------------I---------------GHYQ-D------DMI------MPG
XP_019266312.1/1-285 LES-----------------I---------------GHYQ-D------DMI------MPG
XP_009781184.1/1-285 LES-----------------I---------------GHYQ-D------DMI------MPG
XP_016488110.1/1-285 LES-----------------I---------------GHYQ-D------DMI------MPG
XP_016537761.1/1-285 LES-----------------I---------------GHYQ-D------DMI------MPG
XP_006359741.1/1-285 LES-----------------I---------------GHYQ-D------DMI------MPS
XP_010325078.1/1-288 LES-----------------I---------------GHYQ-D------DMI------MPS
XP_004245155.1/1-285 LES-----------------I---------------GHYQ-D------DMI------MPS
XP_015085523.1/1-285 LES-----------------I---------------GHYQ-D------DMI------MPS
XP_022868182.1/1-285 LEN-----------------V---------------SHFR-E------DTI------MPS
XP_022868181.1/1-285 LEN-----------------V---------------SHFR-E------DTI------MPS
XP_022854559.1/1-285 LEN-----------------V---------------SHFQ-E------DTI------IPS
XP_011081776.1/1-285 LEN-----------------M---------------AQYR-D------DMI------MPS
XP_012857948.1/1-282 LEN-----------------M---------------AQYR-D------DVI------MPS
XP_021847759.1/1-284 LEN-----------------L---------------GHYR-E------DMF------MPG
XP_010668539.1/1-284 LEN-----------------L---------------GHYR-E------DMF------MPG
XP_021739137.1/1-284 LEN-----------------L---------------GHYR-E------DMF------MPG
XP_021737291.1/1-284 LEN-----------------L---------------GHYR-E------DMF------MPG
XP_010047286.1/1-285 LEN-----------------M---------------GHYR-D------DMI------MPS
XP_003603630.2/1-285 LEN-----------------M---------------GYYR-D------DMT------MPG
XP_004501146.1/1-286 LEN-----------------M---------------GYYR-D------DMT------MPG
XP_012571693.1/1-286 LEN-----------------M---------------GYYR-D------DMT------MPG
XP_012571692.1/1-286 LEN-----------------M---------------GYYR-D------DMT------MPG
XP_019417530.1/1-285 LEN-----------------M---------------GYYR-D------DMT------MPG
XP_019417531.1/1-285 LEN-----------------M---------------GYYR-D------DMT------MPG
XP_016180268.1/1-286 LEN-----------------M---------------GYYR-D------DMT------IPG
XP_025623723.1/1-286 LEN-----------------M---------------GYYR-D------DMT------IPG
XP_015945672.1/1-286 LEN-----------------M---------------GYYR-D------DMT------IPG
XP_007137231.1/1-287 LEN-----------------M---------------GYYR-D------DMS------IPG
XP_022636494.1/1-293 LEN-----------------M---------------GYYR-D------DMT------IPG
XP_017409217.1/1-287 LEN-----------------M---------------GYYR-D------DMT------IPG
XP_014501941.1/1-287 LEN-----------------M---------------GYYR-D------DMT------IPG
XP_003526496.1/1-286 LEN-----------------M---------------GYYR-D------DMS------IPG
XP_003522716.1/1-286 LEN-----------------M---------------GYYR-D------DMN------IPG
XP_020217689.1/1-286 LEN-----------------M---------------GYYR-D------DMT------IPG
XP_016683069.1/1-283 LEN-----------------V---------------GHYQ--------DMI------MPS
XP_016677926.1/1-283 LEN-----------------V---------------GHYQ--------DMI------MPS
XP_017643110.1/1-283 LEN-----------------V---------------GHYQ--------DMI------MPS
XP_017643111.1/1-283 LEN-----------------V---------------GHYQ--------DMI------MPS
XP_012451775.1/1-283 LEN-----------------V---------------GHYQ--------DMI------MPS
XP_016744766.1/1-285 LEN-----------------M---------------GHYR-D------DMI------MPG
XP_017643921.1/1-285 LEN-----------------M---------------GHYR-D------DMI------MPG
XP_012436119.1/1-285 LEN-----------------M---------------GHYR-D------DMI------MPG
XP_016681632.1/1-285 LEN-----------------M---------------GHYR-D------DMI------MPG
XP_016689618.1/1-285 LEN-----------------M---------------GHYR-E------DMI------MPG
XP_016689617.1/1-285 LEN-----------------M---------------GHYR-E------DMI------MPG
XP_012445947.1/1-285 LEN-----------------M---------------GHYR-E------DMI------MPG
XP_012445944.1/1-285 LEN-----------------M---------------GHYR-E------DMI------MPG
XP_012445945.1/1-285 LEN-----------------M---------------GHYR-E------DMI------MPG
XP_017614652.1/1-285 LEN-----------------M---------------GHYR-E------DMI------MPG
XP_016732732.1/1-285 LEN-----------------M---------------GHYR-E------DMI------MPG
XP_016732731.1/1-285 LEN-----------------M---------------GHYR-E------DMI------MPG
XP_022754596.1/1-285 LEN-----------------M---------------CHYQ-E------DMI------MPG
XP_022750159.1/1-285 LEN-----------------M---------------SHYR-E------DMI------MPG
XP_022729737.1/1-285 LES-----------------M---------------GHYR-E------DMI------MPG
XP_022729738.1/1-285 LES-----------------M---------------GHYR-E------DMI------MPG
XP_021276132.1/1-285 LEN-----------------M---------------GHYR-D------DMI------MPG
XP_017983822.1/1-285 LEN-----------------M---------------GHYR-D------DMI------MPG
XP_002283717.3/1-285 LEN-----------------V---------------DHYR-E------DLM------MSG
XP_024446213.1/1-284 LEN-----------------M----------------GYR-E------DML------MPG
XP_024446215.1/1-284 LEN-----------------M----------------GYR-E------DML------MPG
XP_011006428.1/1-284 LEN-----------------M----------------GYR-E------DML------MPG
XP_024459734.1/1-284 LEN-----------------M----------------GYR-E------DML------MPG
XP_011019595.1/1-284 LEN-----------------M----------------GYR-E------DML------MPS
XP_015573174.1/1-284 LEN-----------------M----------------GYR-E------DMI------MPG
XP_012077376.1/1-284 LEN-----------------M----------------GYR-E------DMI------MPG
XP_021608403.1/1-284 LEN-----------------M----------------GYR-E------DMI------MPG
XP_021608402.1/1-284 LEN-----------------M----------------GYR-E------DMI------MPG
XP_021597909.1/1-284 LEN-----------------M----------------GYR-E------DML------MPG
XP_021642839.1/1-284 LEN-----------------M----------------GYR-E------DMM------MPG
XP_021689146.1/1-284 LEN-----------------M----------------GYQ-E------DMI------MPG
XP_018805752.1/1-285 LES-----------------M---------------GHYR-E------DMI------MAG
XP_023918167.1/1-285 LES-----------------M---------------GHYR-E------DMM------MAG
XP_018845499.1/1-285 LES-----------------M---------------GHYR-E------DMI------MAG
XP_018845500.1/1-285 LES-----------------M---------------GHYR-E------DMI------MAG
NP_001280750.1/1-285 IEN-----------------M---------------GHYR-E------DMM------MPA
NP_001280796.1/1-285 IEN-----------------L---------------GHYR-E------DMI------MPA
XP_009367440.1/1-285 IEN-----------------M---------------GHYR-E------DMI------MPA
XP_017182108.1/1-285 IEN-----------------M---------------GHYR-E------DMI------MPA
XP_004291167.1/1-285 IEN-----------------M---------------GHYR-D------DMM------MPA
XP_024162655.1/1-285 IEN-----------------M---------------GHYR-D------DMM------MPA
XP_021819449.1/1-284 IEN-----------------M---------------GHYR-E------DMI------MPA
XP_007225281.1/1-285 IEN-----------------M---------------GHYR-E------DMI------MPA
XP_008220092.1/1-285 IEN-----------------M---------------GHYR-E------DMI------MPA
XP_006450706.1/1-285 LEN-----------------M---------------AHYR-E------DMI------MPS
XP_006476026.1/1-285 LEN-----------------M---------------AHYR-E------DMI------MQS
XP_024930942.1/1-285 LEN-----------------M---------------GHYR-D------DMI------MPV
XP_010102406.2/1-285 LEN-----------------M---------------GHYQ-D------DMI------MPV
XP_024540686.1/1-258 LEG-----------------H---------------YSAE-D------GTI------LSR
XP_024399220.1/1-283 LEG-----------------H---------------SAVQ--------HSV------LSR
XP_024399219.1/1-283 LEG-----------------H---------------SAVQ--------HSV------LSR
XP_024399215.1/1-283 LEG-----------------H---------------SAVQ--------HSV------LSR
XP_024390746.1/1-284 VEG-----------------H---------------SSVQ--------HMV------LSR
XP_024390752.1/1-284 VEG-----------------H---------------SSVQ--------HMV------LSR
XP_024373354.1/1-283 LEG-----------------H---------------SSVQ--------HMV------LSR
XP_024373357.1/1-283 LEG-----------------H---------------SSVQ--------HMV------LSR
XP_024370817.1/1-284 LEG-----------------H---------------SSVQ--------HMV------LSR
XP_024370815.1/1-284 LEG-----------------H---------------SSVQ--------HMV------LSR
XP_024370814.1/1-284 LEG-----------------H---------------SSVQ--------HMV------LSR
XP_024386785.1/1-285 LED-----------------H---------------SSVQ--------HMV------ISR
XP_024386782.1/1-285 LED-----------------H---------------SSVQ--------HMV------ISR
XP_024386791.1/1-285 LED-----------------H---------------SSVQ--------HMV------ISR
XP_002961227.1/1-277 LEG-----------------H---------------ASGQ-N------GVV------IPR
XP_002969458.1/1-277 LEG-----------------H---------------ASGQ-N------GVV------IPR
XP_024533017.1/1-265 MEG-----------------Y---------------G-----------NGM------LPK
XP_011076983.1/1-261 FDR-----------------I---------------AHPQH--------NV------SSG
XP_011076980.1/1-264 FDR-----------------I---------------AHPQH--------NV------SSG
XP_011076981.1/1-263 FDR-----------------I---------------AHPQH--------NV------SSG
XP_011084330.1/1-264 FER-----------------S---------------ANQ-Q--------HV------SSG
XP_011084333.1/1-264 FER-----------------S---------------ANQ-Q--------HV------SSG
XP_011084331.1/1-264 FER-----------------S---------------ANQ-Q--------HV------SSG
XP_012834933.1/1-260 FDR-----------------S---------------ADLLL--------TV------SSG
XP_012834932.1/1-260 FDR-----------------S---------------ADLLL--------TV------SSG
XP_012834931.1/1-260 FDR-----------------S---------------ADLLL--------TV------SSG
XP_012834934.1/1-260 FDR-----------------S---------------ADLLL--------TV------SSG
XP_002274194.1/1-284 LEG-----------------H---------------ALSQEE------ASV------MWR
XP_019079956.1/1-284 LEG-----------------H---------------ALSQEE------ASV------MWR
XP_023759583.1/1-283 LEN-----------------M---------------GHYQDN------MMM------SPG
XP_020087984.1/1-275 LEN-----------------I---------------GYYRDA------LV--------PR
XP_015693848.1/1-289 LGN-----------------A---------------SNYQDT------LV--------HR
XP_015624690.1/1-290 LGN-----------------A---------------SNYQDT------LV--------HR
XP_008655743.1/1-292 VGN-----------------A---------------GNYQDA------LL--------HR
XP_020183693.1/1-281 IGN-----------------A---------------SNYQDT------LM--------HR
XP_020183687.1/1-281 IGN-----------------A---------------SNYQDT------LM--------HR
XP_003565084.1/1-284 IGN-----------------A---------------SNYQDT------LM--------HR
XP_002454995.1/1-286 LGN-----------------A---------------SNYQDA------LL--------HR
XP_012701181.1/1-286 LGN-----------------A---------------STYQDA------LM--------HR
XP_012701180.1/1-286 LGN-----------------A---------------STYQDA------LM--------HR
XP_012701179.1/1-286 LGN-----------------A---------------STYQDA------LM--------HR
XP_025816436.1/1-287 LGN-----------------A---------------STYQDT------LM--------HR
XP_018726848.1/1-227 AN--------------------------------------S----------AK----SSQ
XP_018726849.1/1-227 AN--------------------------------------S----------AK----SSQ
XP_019193052.1/1-216 VN--------------------------------------S----------AD----WNQ
XP_019193050.1/1-216 VN--------------------------------------S----------AD----WNQ
XP_021834047.1/1-194 ----------------------------------------S----------AN----SSQ
XP_010522330.1/1-229 ----------------------------------------S----------AN----SSQ
XP_009802524.1/1-219 VN--------------------------------------S---------SAI----SSQ
XP_009802526.1/1-219 VN--------------------------------------S---------SAI----SSQ
XP_009802525.1/1-219 VN--------------------------------------S---------SAI----SSQ
XP_009802527.1/1-219 VN--------------------------------------S---------SAI----SSQ
XP_019250148.1/1-219 VN--------------------------------------S---------SAN----STQ
XP_019250149.1/1-219 VN--------------------------------------S---------SAN----STQ
XP_016449923.1/1-219 VN--------------------------------------S---------SAN----LRQ
XP_009629760.1/1-219 VN--------------------------------------S---------SAN----LRQ
XP_009629762.1/1-219 VN--------------------------------------S---------SAN----LRQ
XP_010234287.2/1-230 VN--------------------------------------N----------AN----ANQ
XP_021806177.1/1-204 ---------------------------------------------------AN----SSQ
XP_016580642.1/1-229 VH--------------------------------------G----------QT----ESQ
XP_008667763.1/1-266 VN--------------------------------------Q---------STN----STQ
XP_006352040.1/1-227 VN--------------------------------------S----------DN----VNH
XP_009611638.1/1-223 VN--------------------------------------S----------SN----SSQ
XP_009611623.1/1-223 VN--------------------------------------S----------SN----SSQ
XP_016510654.1/1-223 VN--------------------------------------S----------SN----SSQ
XP_009766038.1/1-223 VN--------------------------------------S----------SN----SSQ
XP_020210648.1/1-236 VN------------------------------------------------SSN----TTQ
XP_007143210.1/1-219 VN------------------------------------------------TQD----SSQ
XP_014491869.1/1-226 VN------------------------------------------------TPN----FGQ
XP_022633652.1/1-226 VN------------------------------------------------TPN----FGQ
XP_017412341.1/1-227 VN------------------------------------------------TPN----FGQ
XP_022011155.1/1-226 VN------------------------------------------------SAN----SIH
XP_003555330.2/1-228 VN------------------------------------------------TQN----VGQ
XP_014628518.1/1-229 VN------------------------------------------------TQN----VGQ
XP_017647009.1/1-225 VN------------------------------------------------GAN----ASQ
XP_016711277.1/1-225 VK------------------------------------------------GAN----ASQ
XP_012457027.1/1-225 VN------------------------------------------------SAN----ASQ
XP_016723454.1/1-225 VN------------------------------------------------SAN----ASQ
XP_020978973.1/1-214 VN------------------------------------------------SVN----SSQ
XP_016202355.1/1-214 VN------------------------------------------------SVN----SSQ
XP_015964876.1/1-214 VN------------------------------------------------SVN----SSQ
XP_025699645.1/1-214 VN------------------------------------------------SVN----SSQ
XP_008441375.1/1-228 VN------------------------------------------------SAN----SSQ
XP_004154226.2/1-228 VN------------------------------------------------SAN----SSQ
XP_023519981.1/1-226 VN------------------------------------------------STN----SSQ
XP_023001231.1/1-226 VN------------------------------------------------STN----SSQ
XP_022933178.1/1-226 VN------------------------------------------------STN----SSQ
XP_004511340.1/1-228 VN------------------------------------------------SPN----IGQ
XP_002979015.2/1-232 VN------------------------------------------------TLN----STH
XP_002988402.1/1-232 VN------------------------------------------------TLN----STH
XP_024390340.1/1-238 VN------------------------------------------------AMN----ANN
XP_024368074.1/1-235 VN------------------------------------------------AMN----TNN
XP_024401280.1/1-235 VN------------------------------------------------AMN----SSQ
XP_024394840.1/1-235 VN------------------------------------------------AMN----SNQ
XP_024394838.1/1-235 VN------------------------------------------------AMN----SNQ
XP_017256650.1/1-227 VH------------------------------------------------NAN----SSQ
XP_019186546.1/1-227 VN------------------------------------------------SAN----SSQ
XP_024988375.1/1-226 IN------------------------------------------------SAN----SSQ
XP_017231769.1/1-226 VT------------------------------------------------GTN----SSQ
XP_009108529.1/1-235 VN------------------------------------------------SSN----SGE
XP_018469534.1/1-236 VN------------------------------------------------GTN----SGQ
XP_018467466.1/1-236 VN------------------------------------------------GTN----SGQ
XP_022543253.1/1-234 VN------------------------------------------------DTN----SGP
XP_009135181.1/1-234 VN------------------------------------------------DTN----SGP
XP_013642975.1/1-234 VN------------------------------------------------DTN----SGP
XP_009135189.1/1-234 VN------------------------------------------------DTN----SGP
XP_018515487.1/1-234 VN------------------------------------------------DTN----SGP
XP_013623861.1/1-234 VN------------------------------------------------GTN----SGQ
XP_013623905.1/1-234 VN------------------------------------------------GTN----SGQ
XP_013623953.1/1-236 VNK-------------------------------------Q---------GTN----SGQ
XP_013623587.1/1-236 VNK-------------------------------------Q---------GTN----SGQ
XP_013623995.1/1-236 VNK-------------------------------------Q---------GTN----SGQ
XP_013623820.1/1-236 VNK-------------------------------------Q---------GTN----SGQ
XP_022926381.1/1-227 VN------------------------------------------------SAN----SSQ
XP_023517485.1/1-227 VN------------------------------------------------SAN----SSQ
XP_023002930.1/1-227 VN------------------------------------------------SAN----SSQ
XP_004134971.1/1-229 VN------------------------------------------------SAN----SSQ
XP_008439968.1/1-229 VN------------------------------------------------SAN----SSQ
XP_022950176.1/1-227 VN------------------------------------------------SAN----SSQ
XP_022979090.1/1-227 VN------------------------------------------------SAN----SSQ
XP_023543388.1/1-249 VN------------------------------------------------SAN----SSQ
XP_023543387.1/1-227 VN------------------------------------------------SAN----SSQ
XP_009134323.1/1-228 VN------------------------------------------------SGN----SSQ
XP_019176002.1/1-227 VNS------------------------------------------------AN----SSQ
XP_015070655.1/1-221 VNS------------------------------------------------GN----SSQ
XP_015070652.1/1-221 VNS------------------------------------------------GN----SSQ
XP_025885589.1/1-221 VNS------------------------------------------------GN----SSQ
XP_019422236.1/1-227 VNS------------------------------------------------VN----SNQ
XP_007157166.1/1-226 VNS------------------------------------------------AN----SSQ
XP_003538765.1/1-227 VNS------------------------------------------------AN----SSQ
XP_003517589.1/1-227 VNS------------------------------------------------AN----SSQ
XP_006573890.1/1-227 VNS------------------------------------------------AN----SSQ
XP_011000687.1/1-226 VNS------------------------------------------------TN----SSQ
XP_024436817.1/1-226 VNS------------------------------------------------TN----SSQ
XP_024436820.1/1-226 VNS------------------------------------------------TN----SSQ
XP_002518520.2/1-226 VIS------------------------------------------------AN----SSQ
XP_015574304.1/1-226 VIS------------------------------------------------AN----SSQ
XP_021642058.1/1-227 VNS------------------------------------------------AN----SNQ
XP_012091395.1/1-226 VNS------------------------------------------------AN----SSQ
XP_021635824.1/1-226 VNS------------------------------------------------AN----SNQ
XP_021596887.1/1-226 VNS------------------------------------------------AN----SSQ
XP_010057969.1/1-228 VNN------------------------------------------------AN----SSQ
XP_006413738.1/1-245 VNS------------------------------------------------VN----SGQ
XP_013630219.1/1-235 VNS------------------------------------------------SN----SGE
XP_013656136.1/1-235 VNS------------------------------------------------SN----SGE
XP_013685757.1/1-235 VNS------------------------------------------------SN----SGE
XP_013696499.1/1-240 VNS------------------------------------------------AN----SGQ
XP_009137213.1/1-240 VNS------------------------------------------------AN----SGQ
XP_013598423.1/1-241 VNS------------------------------------------------AN----SGQ
XP_013717184.1/1-241 VNS------------------------------------------------AN----SGQ
XP_018480642.1/1-241 VNS------------------------------------------------AN----SGQ
XP_018477464.1/1-241 VNS------------------------------------------------AN----SGQ
NP_193906.2/1-248    VNS------------------------------------------------GN----SGQ
XP_020873301.1/1-248 VNS------------------------------------------------GN----SGQ
XP_006285476.1/1-249 VNS------------------------------------------------GN----SGQ
XP_010434132.1/1-252 VNS------------------------------------------------GN----SGQ
XP_010439429.1/1-252 VNS------------------------------------------------GN----SGQ
XP_010449009.1/1-254 VNS------------------------------------------------GN----SGT
XP_010432053.1/1-234 VNS------------------------------------------------GN----ASQ
XP_020876375.1/1-234 VNS------------------------------------------------GN----SSQ
XP_023636874.1/1-234 VNS------------------------------------------------GN----SSQ
XP_010422328.1/1-234 VNS------------------------------------------------GN----SSQ
XP_010455798.1/1-234 VNS------------------------------------------------GN----SSQ
NP_567274.1/1-235    VNS------------------------------------------------GN----SSQ
XP_024009884.1/1-236 VNS------------------------------------------------GN----SSQ
XP_018452346.1/1-236 VNS------------------------------------------------GN----SSQ
XP_013611160.1/1-236 VNS------------------------------------------------GN----SSQ
XP_009114536.1/1-235 VNS------------------------------------------------GN----SSQ
XP_013662000.1/1-235 VNS------------------------------------------------GN----SSQ
XP_018472664.1/1-233 VNS------------------------------------------------GN----SSQ
XP_022555047.1/1-234 VNS------------------------------------------------GN----SSQ
XP_013735469.1/1-234 VNS------------------------------------------------GN----SSQ
XP_013629800.1/1-234 VNS------------------------------------------------GN----SSQ
XP_013735464.1/1-234 VNS------------------------------------------------GN----SSQ
XP_022571893.1/1-234 VNS------------------------------------------------GN----SSQ
XP_010538642.1/1-236 VNS------------------------------------------------GN----SSQ
XP_019057303.1/1-229 VNS------------------------------------------------GN----SSQ
XP_016545426.1/1-226 VNS------------------------------------------------GN----STQ
XP_009793614.1/1-227 VNS------------------------------------------------GN----SSQ
XP_016508926.1/1-227 VNS------------------------------------------------GN----SSQ
XP_016560096.1/1-235 VNS------------------------------------------------GN----SHQ
XP_021993721.1/1-227 VNS------------------------------------------------AN----SSQ
XP_022036928.1/1-226 VNS------------------------------------------------AN----SSQ
XP_023742870.1/1-226 VNS------------------------------------------------AN----SSQ
XP_006352605.1/1-223 VNS------------------------------------------------GN----SSQ
XP_010327237.1/1-223 VNS------------------------------------------------GN----SSQ
XP_015055984.1/1-223 VNS------------------------------------------------GN----SSQ
XP_009609606.1/1-229 VNS------------------------------------------------GN----SSQ
XP_009609608.1/1-229 VNS------------------------------------------------GN----SSQ
XP_018628742.1/1-229 VNS------------------------------------------------GN----SSQ
XP_019237831.1/1-229 VNS------------------------------------------------GN----SSQ
XP_018849836.1/1-227 VNS------------------------------------------------AN----SSQ
XP_007214541.1/1-230 VNS------------------------------------------------AN----SSQ
XP_021821565.1/1-230 VNS------------------------------------------------AN----SSQ
XP_008224792.1/1-230 VNS------------------------------------------------AN----SSQ
XP_024198966.1/1-227 VNS------------------------------------------------AN----SSQ
XP_004293268.1/1-227 VNS------------------------------------------------AN----SSQ
XP_009364098.1/1-227 VNS------------------------------------------------AN----SSQ
XP_008371114.1/1-227 VNS------------------------------------------------AN----SSQ
XP_008384034.1/1-227 VNS------------------------------------------------AN----SSQ
XP_009340946.1/1-227 VNS------------------------------------------------AN----SSQ
XP_009357415.1/1-227 VNS------------------------------------------------AN----SSQ
XP_011037708.1/1-226 VNS------------------------------------------------AN----SSQ
XP_024454819.1/1-226 VNS------------------------------------------------AN----SSQ
XP_015884145.1/1-226 VNS------------------------------------------------VN----SSQ
XP_024024693.1/1-228 VNS------------------------------------------------AN----SSQ
XP_024024692.1/1-228 VNS------------------------------------------------AN----SSQ
XP_018820351.1/1-226 VNS------------------------------------------------AN----SSQ
XP_023904570.1/1-226 VNS------------------------------------------------AN----SSQ
XP_023904569.1/1-226 VNS------------------------------------------------AN----SSQ
XP_006449076.1/1-226 VNS------------------------------------------------AN----SSQ
XP_006468235.1/1-226 VNS------------------------------------------------AN----SSQ
XP_022721034.1/1-226 VNS------------------------------------------------AN----SSQ
XP_022721035.1/1-226 VNS------------------------------------------------AN----SSQ
XP_022724746.1/1-226 VNS------------------------------------------------AN----SSQ
XP_007026002.1/1-226 VNS------------------------------------------------AN----SSQ
XP_021294392.1/1-226 VNS------------------------------------------------AN----SSQ
XP_022777405.1/1-226 VNS------------------------------------------------AN----SSQ
XP_017615807.1/1-226 VNS------------------------------------------------AN----SSQ
XP_016753649.1/1-226 VNS------------------------------------------------AN----SSQ
XP_016683607.1/1-226 VNS------------------------------------------------AN----SSQ
XP_012451363.1/1-226 VNS------------------------------------------------AN----SSQ
XP_002266688.1/1-226 VNS------------------------------------------------AN----SSQ
XP_021853797.1/1-224 VNS------------------------------------------------AN----SSQ
XP_021719656.1/1-227 VNS------------------------------------------------AN----SSQ
XP_021766927.1/1-227 VNS------------------------------------------------AN----SSQ
XP_010665524.1/1-227 VNS------------------------------------------------AN----SSQ
XP_009605864.1/1-234 VNS------------------------------------------------GN----SNQ
XP_019223778.1/1-233 VNS------------------------------------------------GN----SNQ
XP_016440385.1/1-233 VNS------------------------------------------------GN----SNQ
XP_009790281.1/1-233 VNS------------------------------------------------GN----SNQ
XP_016570434.1/1-222 VNS------------------------------------------------GN----SSQ
XP_016486479.1/1-229 VNS------------------------------------------------GN----SSQ
XP_009597904.1/1-229 VNS------------------------------------------------GN----SSQ
XP_009799795.1/1-229 VNS------------------------------------------------GN----SSQ
XP_019249652.1/1-229 VNS------------------------------------------------GN----SSQ
XP_006343296.1/1-221 VNS------------------------------------------------GN----SSQ
XP_020217012.1/1-226 VNS------------------------------------------------AN----SSQ
XP_017406677.1/1-226 VNS------------------------------------------------AN----SSQ
XP_014519998.1/1-226 VNS------------------------------------------------SN----SSQ
XP_014519997.1/1-226 VNS------------------------------------------------SN----SSQ
XP_012841999.1/1-239 VNS------------------------------------------------AN----SSQ
XP_024984347.1/1-227 VNS------------------------------------------------AN----SSQ
XP_023751202.1/1-227 VNS------------------------------------------------AN----SSQ
XP_023751208.1/1-227 VNS------------------------------------------------AN----SSQ
XP_022041933.1/1-227 VNS------------------------------------------------AN----SSQ
XP_024988632.1/1-226 VNS------------------------------------------------AN----SSQ
XP_022018253.1/1-226 VNS------------------------------------------------AN----SSQ
XP_023771293.1/1-226 VNS------------------------------------------------AN----SSQ
XP_019170324.1/1-226 VNS------------------------------------------------AN----SSQ
XP_017230122.1/1-226 VNS------------------------------------------------AN----SSQ
XP_011096101.1/1-226 VNS------------------------------------------------AN----SSQ
XP_012848972.1/1-227 VNS------------------------------------------------AN----SSQ
XP_022876975.1/1-227 VNS------------------------------------------------AN----SSQ
XP_022863158.1/1-227 VNS------------------------------------------------AN----SSQ
XP_022877991.1/1-227 VNS------------------------------------------------AN----SSQ
XP_019223745.1/1-223 VNS------------------------------------------------SN----SSQ
XP_021800220.1/1-221 VNS------------------------------------------------AN----SSQ
XP_021684589.1/1-235 VNS------------------------------------------------AN----SSQ
XP_021684519.1/1-235 VNS------------------------------------------------AN----SSQ
XP_021684722.1/1-235 VNS------------------------------------------------AN----SSQ
XP_021633498.1/1-230 VNS------------------------------------------------AN----SSQ
XP_009420270.1/1-216 VNS------------------------------------------------VK----SSQ
XP_003573403.1/1-232 VNS------------------------------------------------AN----SNQ
XP_006659894.1/1-229 VNS------------------------------------------------AN----SNQ
XP_015650022.1/1-263 VNS------------------------------------------------AN----SNQ
XP_015650023.1/1-230 VNS------------------------------------------------AN----SNQ
XP_020407343.1/1-231 VNS------------------------------------------------AN----SNQ
NP_001105127.2/1-231 VNS------------------------------------------------AN----SNQ
XP_008661805.1/1-239 VNS------------------------------------------------AN----SNQ
NP_001145750.1/1-239 VNS------------------------------------------------AN----SNQ
XP_025821536.1/1-230 VNS------------------------------------------------AN----SNQ
XP_002443965.1/1-229 VNS------------------------------------------------AN----SNQ
XP_004972697.1/1-229 VNS------------------------------------------------AN----SNQ
XP_008667765.1/1-230 VNS------------------------------------------------TN----STQ
NP_001347891.1/1-231 VNQ-----------------------------------------------STN----STQ
XP_015636340.1/1-229 VNS------------------------------------------------SN----SNQ
XP_015636338.1/1-229 VNS------------------------------------------------SN----SNQ
XP_006652859.1/1-228 VNS------------------------------------------------SN----SNQ
XP_003580598.1/1-233 VNS------------------------------------------------TN----SNQ
XP_020199198.1/1-227 VNS------------------------------------------------TN----SNQ
XP_020400772.1/1-230 VNS------------------------------------------------TN----SNQ
NP_001334663.1/1-230 VNS------------------------------------------------TN----SNQ
XP_008662197.1/1-270 VNS------------------------------------------------TN----SNQ
XP_008662196.1/1-270 VNS------------------------------------------------TN----SNQ
XP_025823505.1/1-227 VNS------------------------------------------------TN----SNQ
XP_004976894.1/1-227 VNS------------------------------------------------TN----SNQ
XP_002448563.2/1-227 VNS------------------------------------------------TN----SNQ
XP_019414154.1/1-227 VN------------------------------------------------SAN----SSQ
XP_006348085.1/1-234 VNT-----------------------------------------------GTN----SNQ
XP_004232734.1/1-234 VNT-----------------------------------------------GTN----SNQ
XP_015066898.1/1-234 VNT-----------------------------------------------GTN----SNQ
XP_003605900.1/1-232 VN------------------------------------------------SPN----SSQ
XP_022988693.1/1-229 VN------------------------------------------------SPN----SSQ
XP_022930609.1/1-229 VN------------------------------------------------SPN----SSQ
XP_023530197.1/1-229 VN------------------------------------------------SPN----SSQ
XP_016188415.1/1-237 VN------------------------------------------------SAN----SSQ
XP_011628991.1/1-236 VN------------------------------------------------SAN----SNQ
XP_006826512.2/1-236 VN------------------------------------------------SAN----SNQ
XP_020177398.1/1-220 VN------------------------------------------------NAN----SNQ
XP_015695486.1/1-219 VN------------------------------------------------NAN----SNQ
XP_015648539.1/1-217 VN------------------------------------------------NAN----SNQ
XP_020401599.1/1-218 VD------------------------------------------------NAN----SNQ
XP_020401598.1/1-218 VD------------------------------------------------NAN----SNQ
XP_020401597.1/1-218 VD------------------------------------------------NAN----SNQ
XP_021319954.1/1-239 VN------------------------------------------------NAN----SNQ
XP_002445014.2/1-239 VN------------------------------------------------NAN----SNQ
XP_025819913.1/1-218 VN------------------------------------------------NAN----SNQ
XP_004972909.1/1-218 VN------------------------------------------------NAN----SNQ
XP_020100432.1/1-229 VN------------------------------------------------SAN----SNQ
XP_009385059.1/1-228 VN------------------------------------------------STN----SSQ
XP_018678797.1/1-226 VN------------------------------------------------STN----SNQ
XP_009412012.1/1-233 VN------------------------------------------------STN----SNQ
XP_018685149.1/1-236 VNV-------------------------------------R------N--STN----SNQ
XP_020591278.1/1-230 VNS------------------------------------------------TN----SNQ
XP_020702932.1/1-229 VNS------------------------------------------------TN----SNQ
XP_020702933.1/1-230 VNQ-----------------------------------------------STN----SNQ
XP_020702931.1/1-230 VNQ-----------------------------------------------STN----SNQ
XP_020692781.1/1-229 VNS------------------------------------------------TN----SNQ
XP_020692780.1/1-229 VNS------------------------------------------------TN----SNQ
XP_020591591.1/1-229 VNS------------------------------------------------TN----SSQ
XP_020591590.1/1-229 VNS------------------------------------------------TN----SSQ
XP_020109359.1/1-227 VNS------------------------------------------------VN----SNQ
XP_020270114.1/1-229 VNS------------------------------------------------PN----SNQ
XP_010908855.1/1-227 VNS------------------------------------------------AN----SNQ
XP_008808987.1/1-227 VNS------------------------------------------------AN----SNQ
XP_008784552.1/1-223 VNS------------------------------------------------AN----SNQ
XP_010931820.1/1-223 VNS------------------------------------------------AN----SNQ
XP_003540308.2/1-226 VN------------------------------------------------SAN----SSQ
XP_006592818.1/1-226 VN------------------------------------------------SAN----SSQ
XP_006592816.1/1-226 VN------------------------------------------------SAN----SSQ
XP_003543387.1/1-226 VN------------------------------------------------SAN----SSQ
XP_020223651.1/1-226 VN------------------------------------------------SAN----SSQ
XP_020223648.1/1-226 VN------------------------------------------------SAN----SSQ
XP_007149968.1/1-226 VN------------------------------------------------SAN----SSQ
XP_017425582.1/1-226 VN------------------------------------------------SAN----SSQ
XP_014501634.1/1-226 VN------------------------------------------------SAN----SSQ
XP_022776632.1/1-226 VN------------------------------------------------SAN----SSQ
XP_022776631.1/1-226 VN------------------------------------------------SAN----SSQ
XP_022776629.1/1-226 VN------------------------------------------------SAN----SSQ
XP_022733641.1/1-226 VN------------------------------------------------SAN----SSQ
XP_022733640.1/1-226 VN------------------------------------------------SAN----SSQ
XP_022733638.1/1-226 VN------------------------------------------------SAN----SSQ
XP_007045612.1/1-226 VN------------------------------------------------SAN----SSQ
XP_007045611.1/1-226 VN------------------------------------------------SAN----SSQ
XP_017971478.1/1-226 VN------------------------------------------------SAN----SSQ
XP_021292769.1/1-226 VN------------------------------------------------SAN----SSQ
XP_021292767.1/1-226 VN------------------------------------------------SAN----SSQ
XP_021292765.1/1-226 VN------------------------------------------------SAN----SSQ
XP_010657309.1/1-226 VN------------------------------------------------SAN----SSQ
XP_010657313.1/1-226 VN------------------------------------------------SAN----SSQ
XP_010657310.1/1-226 VN------------------------------------------------SAN----SSQ
XP_010657311.1/1-226 VN------------------------------------------------SAN----SSQ
XP_012086347.1/1-228 VN------------------------------------------------SAN----SSQ
XP_012086344.1/1-228 VN------------------------------------------------SAN----SSQ
XP_024029967.1/1-226 VN------------------------------------------------SAN----SSQ
XP_010109517.1/1-226 VN------------------------------------------------SAN----SSQ
XP_006469697.1/1-226 VN------------------------------------------------SAN----SSQ
XP_024046029.1/1-226 VN------------------------------------------------SAN----SSQ
XP_020234199.1/1-226 VN------------------------------------------------SAN----SSQ
XP_020234201.1/1-226 VN------------------------------------------------SAN----SSQ
XP_021630856.1/1-226 VN------------------------------------------------SAN----SSQ
XP_021630853.1/1-226 VN------------------------------------------------SAN----SSQ
XP_021907367.1/1-221 VN------------------------------------------------SVN----SSQ
XP_010275996.1/1-221 VN------------------------------------------------SAN----SSQ
XP_020870784.1/1-223 VN------------------------------------------------SAN----SSQ
NP_172015.1/1-223    VN------------------------------------------------SAN----SSQ
XP_006418050.1/1-223 VN------------------------------------------------SAN----SSQ
XP_018447753.1/1-223 VN------------------------------------------------SAN----SSQ
XP_010457605.1/1-218 --------------------------------------------------SAN----SSQ
XP_023632765.1/1-218 --------------------------------------------------SAN----SSQ
XP_010484829.1/1-218 --------------------------------------------------SAN----SSQ
XP_010475208.1/1-218 --------------------------------------------------SAN----SSQ
XP_018487097.1/1-220 VN------------------------------------------------SAN----SSQ
XP_013664795.1/1-220 VN------------------------------------------------SAN----SSQ
XP_013716253.1/1-220 VN------------------------------------------------SAN----SSQ
XP_009118607.1/1-220 VN------------------------------------------------SAN----SSQ
XP_013605632.1/1-220 VN------------------------------------------------SAN----SSQ
XP_010475204.1/1-220 VN------------------------------------------------SAN----SSQ
XP_010457604.1/1-220 VN------------------------------------------------SAN----SSQ
XP_023632762.1/1-220 VN------------------------------------------------SAN----SSQ
XP_010484823.1/1-220 VN------------------------------------------------SAN----SSQ
XP_022558401.1/1-220 VN------------------------------------------------SAN----SSQ
XP_013586641.1/1-220 VN------------------------------------------------SAN----SSQ
XP_022558399.1/1-220 VN------------------------------------------------SAN----SSQ
XP_022558400.1/1-220 VN------------------------------------------------SAN----SSQ
XP_013586642.1/1-220 VN------------------------------------------------SAN----SSQ
XP_013696832.1/1-220 VN------------------------------------------------SAN----SSQ
XP_022548878.1/1-220 VN------------------------------------------------SAN----SSQ
XP_013666552.1/1-220 VN------------------------------------------------SAN----SSQ
XP_009119177.1/1-220 VN------------------------------------------------SAN----SSQ
XP_020964430.1/1-225 VN------------------------------------------------SAN----SSQ
XP_015936069.1/1-225 VN------------------------------------------------SAN----SSQ
XP_020964429.1/1-225 VN------------------------------------------------SAN----SSQ
XP_015936068.1/1-225 VN------------------------------------------------SAN----SSQ
XP_015577880.1/1-228 VN------------------------------------------------SAN----SSQ
XP_002524172.2/1-228 VN------------------------------------------------SAN----SSQ
XP_007132557.1/1-221 VN------------------------------------------------SAN----SSQ
XP_017433694.1/1-221 VN------------------------------------------------SAN----SSQ
XP_014494419.1/1-221 VN------------------------------------------------SAN----SSQ
XP_022634310.1/1-221 VN------------------------------------------------SAN----SSQ
XP_010267735.1/1-225 VN------------------------------------------------SAN----SSQ
XP_010267734.1/1-225 VN------------------------------------------------SAN----SSQ
XP_024185199.1/1-224 VN------------------------------------------------SAN----SSQ
XP_024185197.1/1-224 VN------------------------------------------------SAN----SSQ
XP_009342246.1/1-222 VN------------------------------------------------SAN----SSQ
XP_009342244.1/1-222 VN------------------------------------------------SAN----SSQ
XP_009342240.1/1-222 VN------------------------------------------------SAN----SSQ
XP_020416503.1/1-221 VN------------------------------------------------SAN----SSQ
XP_021822854.1/1-221 VN------------------------------------------------SAN----SSQ
XP_007217584.1/1-221 VN------------------------------------------------SAN----SSQ
XP_021822852.1/1-221 VN------------------------------------------------SAN----SSQ
XP_018730806.1/1-228 VN------------------------------------------------SAN----SSQ
XP_010051064.1/1-228 VN------------------------------------------------SAN----SSQ
XP_015881130.2/1-229 VN------------------------------------------------SAN----SSQ
XP_023534533.1/1-219 VN------------------------------------------------SAN----SSQ
XP_022947095.1/1-219 VN------------------------------------------------SAN----SSQ
XP_022947096.1/1-219 VN------------------------------------------------SAN----SSQ
XP_022970950.1/1-219 VN------------------------------------------------SAN----SSQ
XP_022970951.1/1-219 VN------------------------------------------------SAN----SSQ
XP_022140692.1/1-217 VN------------------------------------------------SAN----SSQ
XP_022140691.1/1-217 VN------------------------------------------------SAN----SSQ
XP_008455853.1/1-220 VN------------------------------------------------SAN----SSQ
XP_011650012.1/1-220 VN------------------------------------------------SAN----SSQ
XP_008455850.1/1-220 VN------------------------------------------------SAN----SSQ
XP_011650011.1/1-220 VN------------------------------------------------SAN----SSQ
XP_023513403.1/1-220 VN------------------------------------------------STN----SSQ
XP_022944761.1/1-220 VN------------------------------------------------STN----SSQ
XP_023513401.1/1-220 VN------------------------------------------------STN----SSQ
XP_022944759.1/1-220 VN------------------------------------------------STN----SSQ
XP_022986270.1/1-220 VN------------------------------------------------STN----SSQ
XP_022986268.1/1-220 VN------------------------------------------------STN----SSQ
XP_022136006.1/1-224 VN------------------------------------------------SAN----SSQ
XP_008455423.1/1-229 VN------------------------------------------------SAN----SSQ
XP_011658704.1/1-229 VN------------------------------------------------SAN----SSQ
XP_004144488.1/1-229 VN------------------------------------------------SAN----SSQ
XP_022968990.1/1-229 VN------------------------------------------------SAN----SSQ
XP_023554403.1/1-229 VN------------------------------------------------SAN----SSQ
XP_022952948.1/1-229 VN------------------------------------------------SAN----SSQ
XP_019447751.1/1-228 VN------------------------------------------------SAN----SSQ
XP_019447749.1/1-228 VN------------------------------------------------SAN----SSQ
XP_004515074.1/1-228 VN------------------------------------------------SAN----SSQ
XP_012463792.1/1-235 VN------------------------------------------------SAN----SSQ
XP_012463790.1/1-235 VN------------------------------------------------SAN----SSQ
XP_016678783.1/1-235 VN------------------------------------------------SAN----SSQ
XP_016678782.1/1-235 VN------------------------------------------------SAN----SSQ
XP_017631648.1/1-235 VN------------------------------------------------SAN----SSQ
XP_016732503.1/1-235 VN------------------------------------------------SAN----SSQ
XP_017631647.1/1-235 VN------------------------------------------------SAN----SSQ
XP_012438430.1/1-229 VN------------------------------------------------SAN----SSQ
XP_012438427.1/1-229 VN------------------------------------------------SAN----SSQ
XP_012438429.1/1-229 VN------------------------------------------------SAN----SSQ
XP_017616162.1/1-229 VN------------------------------------------------SAN----SSQ
XP_017616160.1/1-229 VN------------------------------------------------SAN----SSQ
XP_017616161.1/1-229 VN------------------------------------------------SAN----SSQ
XP_016726347.1/1-229 VN------------------------------------------------SAN----SSQ
XP_016726344.1/1-229 VN------------------------------------------------SAN----SSQ
XP_016726345.1/1-229 VN------------------------------------------------SAN----SSQ
XP_019448933.1/1-227 VN------------------------------------------------SAN----SSQ
XP_004487400.1/1-228 VN------------------------------------------------SAN----SSQ
XP_003596999.2/1-227 VN------------------------------------------------SAN----SSQ
XP_024631890.1/1-227 VN------------------------------------------------SAN----SSQ
XP_024631891.1/1-227 VN------------------------------------------------SAN----SSQ
XP_004287935.1/1-224 VN------------------------------------------------SAN----SSQ
XP_018808969.1/1-228 VN------------------------------------------------SAN----SSQ
XP_018808967.1/1-228 VN------------------------------------------------SAN----SSQ
XP_018817598.1/1-227 VN------------------------------------------------SAN----SSQ
XP_018817597.1/1-227 VN------------------------------------------------SAN----SSQ
XP_018817594.1/1-227 VN------------------------------------------------SAN----SSQ
XP_018817599.1/1-227 VN------------------------------------------------SAN----SSQ
XP_023915056.1/1-227 VN------------------------------------------------SAN----SSQ
XP_023915054.1/1-227 VN------------------------------------------------SAN----SSQ
XP_023915053.1/1-227 VN------------------------------------------------SAN----SSQ
XP_021685023.1/1-228 VN------------------------------------------------SAN----SSQ
XP_021684657.1/1-228 VN------------------------------------------------SAN----SSQ
XP_021685089.1/1-228 VN------------------------------------------------SAN----SSQ
XP_015383009.1/1-229 VNC-------------------------------------L--------QSAN----SSQ
XP_015383007.1/1-229 VNC-------------------------------------L--------QSAN----SSQ
XP_020539441.1/1-235 VN------------------------------------------------SAN----SSQ
XP_020539439.1/1-235 VN------------------------------------------------SAN----SSQ
XP_015577881.1/1-233 VNV-------------------------------------S------TTQSAN----SSQ
XP_015577879.1/1-233 VNV-------------------------------------S------TTQSAN----SSQ
XP_015577877.1/1-233 VNV-------------------------------------S------TTQSAN----SSQ
XP_021630854.1/1-231 VNV-------------------------------------S------DTQSAN----SSQ
XP_021630849.1/1-231 VNV-------------------------------------S------DTQSAN----SSQ
XP_021652390.1/1-229 VN------------------------------------------------SAN----SSQ
XP_021652388.1/1-229 VN------------------------------------------------SAN----SSQ
XP_021652389.1/1-234 VNV-------------------------------------S------DAQSAN----SSQ
XP_021652386.1/1-234 VNV-------------------------------------S------DAQSAN----SSQ
XP_006592380.1/1-228 VN------------------------------------------------SAN----SSQ
XP_003539872.2/1-228 VN------------------------------------------------SAN----SSQ
XP_025984806.1/1-230 VN------------------------------------------------SAN----SSQ
XP_014632311.1/1-230 VN------------------------------------------------SAN----SSQ
XP_024450362.1/1-227 VN------------------------------------------------SAN----SSQ
XP_002302880.2/1-227 VN------------------------------------------------SAN----SSQ
XP_024450361.1/1-227 VN------------------------------------------------SAN----SSQ
XP_024450360.1/1-227 VN------------------------------------------------SAN----SSQ
XP_011011493.1/1-227 VN------------------------------------------------SAN----SSQ
XP_011011491.1/1-227 VN------------------------------------------------SAN----SSQ
XP_011011492.1/1-227 VN------------------------------------------------SAN----SSQ
XP_011011489.1/1-227 VN------------------------------------------------SAN----SSQ
XP_011011488.1/1-227 VN------------------------------------------------SAN----SSQ
XP_011005116.1/1-225 VN------------------------------------------------SAN----SSQ
XP_011005113.1/1-225 VN------------------------------------------------SAN----SSQ
XP_011005112.1/1-225 VN------------------------------------------------SAN----SSQ
XP_024440391.1/1-225 VN------------------------------------------------SAN----SSQ
XP_002320458.2/1-225 VN------------------------------------------------SAN----SSQ
XP_024440389.1/1-225 VN------------------------------------------------SAN----SSQ
XP_019453767.1/1-227 VN------------------------------------------------SAN----SSQ
XP_012478857.1/1-227 VN------------------------------------------------SAN----SSQ
XP_012478855.1/1-227 VN------------------------------------------------SAN----SSQ
XP_012478856.1/1-227 VN------------------------------------------------SAN----SSQ
XP_012478851.1/1-227 VN------------------------------------------------SAN----SSQ
XP_016724999.1/1-227 VN------------------------------------------------SAN----SSQ
XP_017638204.1/1-227 VN------------------------------------------------SAN----SSQ
XP_017638203.1/1-227 VN------------------------------------------------SAN----SSQ
XP_016667113.1/1-227 VN------------------------------------------------SAN----SSQ
XP_016667112.1/1-227 VN------------------------------------------------SAN----SSQ
XP_015881131.2/1-227 --------------------------------------------------SAN----SSQ
XP_008348131.1/1-222 VN------------------------------------------------SAN----SSQ
XP_008348133.1/1-220 --------------------------------------------------SAN----SSQ
XP_018502305.1/1-220 --------------------------------------------------SAN----SSQ
XP_009342243.1/1-220 --------------------------------------------------SAN----SSQ
XP_017179321.1/1-222 VN------------------------------------------------SAN----SSQ
XP_017179320.1/1-222 VN------------------------------------------------SAN----SSQ
XP_008341986.1/1-222 VN------------------------------------------------SAN----SSQ
XP_017179319.1/1-220 --------------------------------------------------SAN----SSQ
XP_024185198.1/1-222 --------------------------------------------------SAN----SSQ
XP_011463209.1/1-222 --------------------------------------------------SAN----SSQ
XP_020416501.1/1-219 --------------------------------------------------SAN----SSQ
XP_021822853.1/1-219 --------------------------------------------------SAN----SSQ
XP_008230079.1/1-219 --------------------------------------------------SAN----SSQ
XP_020870786.1/1-221 --------------------------------------------------SAN----SSQ
NP_001117231.1/1-221 --------------------------------------------------SAN----SSQ
XP_018817596.1/1-225 --------------------------------------------------SAN----SSQ
XP_019079350.1/1-224 --------------------------------------------------SAN----SSQ
XP_022733639.1/1-224 --------------------------------------------------SAN----SSQ
XP_021292768.1/1-224 --------------------------------------------------SAN----SSQ
XP_021292766.1/1-224 --------------------------------------------------SAN----SSQ
XP_017971480.1/1-224 --------------------------------------------------SAN----SSQ
XP_018721484.1/1-222 VN------------------------------------------------SAN----SSQ
XP_018721483.1/1-222 VN------------------------------------------------SAN----SSQ
XP_018721482.1/1-222 VN------------------------------------------------SAN----SSQ
XP_025687365.1/1-237 VN------------------------------------------------SAN----SSQ
XP_006851770.2/1-243 AN--------------------------------------A--------T--N----SSQ
XP_003623826.1/1-230 AS--------------------------------------A--------I--N----SSQ
XP_004492678.1/1-230 AS--------------------------------------A--------N--N----SSQ
XP_004492677.1/1-230 AS--------------------------------------A--------N--N----SSQ
XP_012847897.1/1-236 AG--------------------------------------A--------V--N----SSQ
XP_012843673.1/1-229 AG--------------------------------------A--------V--N----SSQ
XP_021279608.1/1-239 AS--------------------------------------A--------M--N----ASQ
XP_025641241.1/1-241 AG--------------------------------------A--------I--N----SNQ
XP_025641246.1/1-241 AG--------------------------------------A--------I--N----SNQ
XP_020991942.1/1-241 AG--------------------------------------A--------I--N----SNQ
XP_025632115.1/1-241 AG--------------------------------------A--------I--N----SNQ
XP_025632116.1/1-241 AG--------------------------------------A--------I--N----SNQ
XP_017442222.1/1-246 AS--------------------------------------A--------I--N----SSQ
XP_017442225.1/1-246 AS--------------------------------------A--------I--N----SSQ
XP_006576359.1/1-244 AS--------------------------------------A--------I--N----SNQ
XP_003521284.1/1-244 AS--------------------------------------A--------I--N----SNQ
XP_024174849.1/1-237 AR--------------------------------------A--------M--N----ANQ
XP_024174854.1/1-237 AR--------------------------------------A--------M--N----ANQ
XP_004306832.1/1-241 AR--------------------------------------A--------M--N----ANQ
XP_011469062.1/1-241 AR--------------------------------------A--------M--N----ANQ
XP_009340632.2/1-243 AR--------------------------------------A-----------N----ANQ
XP_008385703.1/1-243 AR--------------------------------------A-----------N----ANQ
XP_008385704.1/1-243 AR--------------------------------------A-----------N----ANQ
XP_008232741.1/1-243 AR--------------------------------------A--------M--N----ANQ
XP_008232742.1/1-243 AR--------------------------------------A--------M--N----ANQ
XP_007220256.1/1-243 AR--------------------------------------A--------M--N----ANQ
XP_020413144.1/1-243 AR--------------------------------------A--------M--N----ANQ
XP_021817103.1/1-243 AR--------------------------------------A--------M--N----ANQ
XP_021817105.1/1-243 AR--------------------------------------A--------M--N----ANQ
XP_022872559.1/1-230 AS--------------------------------------A--------T--N----ENQ
XP_016575742.1/1-238 AS--------------------------------------A--------M--N----ANQ
XP_017608938.1/1-232 SS--------------------------------------A--------M--N----TNQ
XP_017608947.1/1-232 SS--------------------------------------A--------M--N----TNQ
XP_010255501.1/1-245 AS--------------------------------------A--------M--N----ANQ
XP_011023407.1/1-238 AS--------------------------------------A--------M--N----ANQ
XP_011023404.1/1-238 AS--------------------------------------A--------M--N----ANQ
XP_011023405.1/1-238 AS--------------------------------------A--------M--N----ANQ
XP_019152252.1/1-243 AS--------------------------------------A--------M--N----SNQ
XP_022870029.1/1-235 AS--------------------------------------A--------M--N----SNQ
XP_017699285.1/1-225 AS--------------------------------------A--------V--S----ANQ
XP_008795402.1/1-225 AS--------------------------------------A--------V--S----ANQ
XP_010933869.1/1-224 AS--------------------------------------A--------V--S----ANQ
XP_008799528.1/1-222 AS--------------------------------------A--------V--N----ANQ
XP_010919252.1/1-223 AS--------------------------------------A--------V--S----ANQ
XP_010919250.1/1-223 AS--------------------------------------A--------V--S----ANQ
XP_007139955.1/1-229 AS--------------------------------------A--------M--N----ANQ
XP_017418814.1/1-230 AS--------------------------------------A--------M--N----ANQ
XP_014490274.1/1-230 AS--------------------------------------A--------M--N----ANQ
XP_003534596.1/1-229 AS--------------------------------------A--------I--N----ANQ
XP_006587871.1/1-229 AS--------------------------------------A--------I--N----ANQ
XP_003552359.1/1-228 AS--------------------------------------A--------I--N----ANQ
XP_015961616.1/1-228 AS--------------------------------------A--------M--N----SNQ
XP_015961617.1/1-228 AS--------------------------------------A--------M--N----SNQ
XP_025696060.1/1-228 AS--------------------------------------A--------M--N----SNQ
XP_025696061.1/1-228 AS--------------------------------------A--------M--N----SNQ
XP_016198931.1/1-228 AS--------------------------------------A--------M--N----SNQ
XP_016198932.1/1-228 AS--------------------------------------A--------M--N----SNQ
XP_025649261.1/1-228 AS--------------------------------------A--------M--N----SNQ
XP_025649262.1/1-228 AS--------------------------------------A--------M--N----SNQ
XP_021849370.1/1-228 AG--------------------------------------A--------M--N----PNQ
XP_021767325.1/1-228 AG--------------------------------------A--------M--N----PNQ
XP_021748881.1/1-228 AG--------------------------------------A--------M--N----PNQ
XP_019461451.1/1-231 AS--------------------------------------A--------M--N----SNQ
XP_012574211.1/1-233 AS--------------------------------------A--------M--N----SNQ
XP_004510857.1/1-233 AS--------------------------------------A--------M--N----SNQ
XP_020237185.1/1-234 AS--------------------------------------A--------I--N----SNQ
XP_020237261.1/1-234 AS--------------------------------------A--------I--N----SNQ
XP_019456357.1/1-238 AS--------------------------------------A--------M--N----SNQ
XP_013444922.1/1-235 AS--------------------------------------A--------M--N----SNQ
XP_003528880.1/1-238 AS--------------------------------------A--------I--N----SNQ
XP_006583340.1/1-238 AS--------------------------------------A--------I--N----SNQ
XP_014516480.1/1-238 AS--------------------------------------A--------I--N----SSQ
XP_014516483.1/1-238 AS--------------------------------------A--------I--N----SSQ
XP_007134961.1/1-238 AS--------------------------------------A--------I--N----SNQ
XP_010671720.1/1-229 AG--------------------------------------A--------M--N----PNQ
XP_012482732.1/1-232 SS--------------------------------------A--------M--N----ANQ
XP_012482742.1/1-232 SS--------------------------------------A--------M--N----ANQ
XP_016745638.1/1-232 SS--------------------------------------A--------M--N----ANQ
XP_016745639.1/1-232 SS--------------------------------------A--------M--N----ANQ
XP_010278578.1/1-242 AS--------------------------------------A--------M--N----ANQ
XP_010278577.1/1-242 AS--------------------------------------A--------M--N----ANQ
XP_006445143.1/1-238 AS--------------------------------------A--------I--N----ANQ
XP_006445141.1/1-238 AS--------------------------------------A--------I--N----ANQ
XP_022770361.1/1-238 AS--------------------------------------A--------M--N----ANQ
XP_022757219.1/1-237 AS--------------------------------------A--------M--N----SNQ
XP_022757220.1/1-237 AS--------------------------------------A--------M--N----SNQ
NP_001314420.1/1-237 AS--------------------------------------A--------M--N----ANQ
XP_016735217.1/1-237 AS--------------------------------------A--------M--N----ANQ
XP_012437660.1/1-237 AS--------------------------------------A--------M--N----ANQ
XP_012437661.1/1-237 AS--------------------------------------A--------M--N----ANQ
XP_017615505.1/1-237 AS--------------------------------------A--------M--N----ANQ
XP_017615506.1/1-237 AS--------------------------------------A--------M--N----ANQ
XP_016723220.1/1-237 AS--------------------------------------A--------M--N----ANQ
XP_016723229.1/1-237 AS--------------------------------------A--------M--N----ANQ
XP_021904309.1/1-244 AS--------------------------------------A--------M--N----ANQ
XP_021904311.1/1-244 AS--------------------------------------A--------M--N----ANQ
XP_007051912.2/1-241 AS--------------------------------------A--------M--N----ANQ
XP_007051913.2/1-241 AS--------------------------------------A--------M--N----ANQ
XP_012489878.1/1-239 AS--------------------------------------A--------M--N----ANQ
XP_016748231.1/1-239 AS--------------------------------------A--------M--N----ANQ
XP_017631289.1/1-239 AS--------------------------------------A--------M--N----ANQ
XP_023006686.1/1-236 AS--------------------------------------A--------M--N----ANQ
XP_022958954.1/1-236 AS--------------------------------------A--------M--N----ANQ
XP_023548045.1/1-236 AS--------------------------------------A--------M--N----ANQ
XP_008460172.1/1-241 AS--------------------------------------A--------M--N----ANQ
XP_004144982.1/1-241 AS--------------------------------------A--------M--N----ANQ
XP_023514722.1/1-242 AS--------------------------------------A--------M--N----ANQ
XP_022999814.1/1-242 AS--------------------------------------A--------M--N----ANQ
XP_022964504.1/1-242 AS--------------------------------------A--------M--N----ANQ
XP_010053664.1/1-245 AS--------------------------------------A--------M--N----ANQ
XP_019189515.1/1-235 AS--------------------------------------A--------I--N----ANQ
XP_023730775.1/1-234 AS--------------------------------------A--------M--N----ANQ
XP_023730774.1/1-234 AS--------------------------------------A--------M--N----ANQ
XP_022020543.1/1-230 AS--------------------------------------A--------M--N----ANQ
XP_024962572.1/1-236 AS--------------------------------------A--------M--N----ANQ
XP_022017696.1/1-236 AS--------------------------------------A--------M--N----ANQ
XP_024018220.1/1-245 AS--------------------------------------A--------M--N----TNQ
XP_011093166.1/1-234 AS--------------------------------------A--------M--N----ANQ
XP_011093167.1/1-234 AS--------------------------------------A--------M--N----ANQ
XP_006339457.1/1-238 AS--------------------------------------A--------M--N----ANQ
XP_015059298.1/1-238 AS--------------------------------------A--------M--N----ANQ
NP_001234657.2/1-238 AS--------------------------------------A--------M--N----ANQ
XP_009602857.1/1-241 AS--------------------------------------A--------I--N----ANQ
XP_009602856.1/1-241 AS--------------------------------------A--------I--N----ANQ
XP_009782444.1/1-245 AS--------------------------------------A--------I--N----ANQ
XP_009782443.1/1-245 AS--------------------------------------A--------I--N----ANQ
XP_019259406.1/1-245 AS--------------------------------------A--------I--N----ANQ
XP_015867375.1/1-242 AS--------------------------------------A--------M--N----ANQ
XP_015867374.1/1-242 AS--------------------------------------A--------M--N----ANQ
XP_015888672.1/1-242 AS--------------------------------------A--------M--N----ANQ
XP_018810322.1/1-233 AA--------------------------------------A--------M--N----ANQ
XP_018859074.1/1-233 AA--------------------------------------A--------M--N----ANQ
XP_017231243.1/1-235 AS--------------------------------------A--------M--N----SNQ
XP_017231244.1/1-235 AS--------------------------------------A--------M--N----SNQ
XP_017231245.1/1-235 AS--------------------------------------A--------M--N----SNQ
XP_017236322.1/1-235 AS--------------------------------------A--------M--N----PNQ
XP_002301331.2/1-238 AS--------------------------------------A--------M--N----ANQ
XP_024451706.1/1-238 AS--------------------------------------A--------M--N----ANQ
XP_002320755.1/1-241 AS--------------------------------------A--------M--N----ANQ
XP_011035097.1/1-241 AS--------------------------------------A--------M--N----SNQ
XP_018813891.1/1-238 AG--------------------------------------A--------M--N----ANQ
XP_012083470.1/1-239 AS--------------------------------------A--------M--N----ANQ
XP_021651338.1/1-240 AS--------------------------------------A--------M--N----ANQ
XP_021613538.1/1-241 AS--------------------------------------A--------M--N----ANQ
XP_021686862.1/1-241 AS--------------------------------------A--------M--N----ANQ
XP_021686863.1/1-241 AS--------------------------------------A--------M--N----ANQ
XP_021612685.1/1-241 AS--------------------------------------A--------M--N----ANQ
XP_023879166.1/1-242 AS--------------------------------------A--------M--N----PNQ
XP_002272264.2/1-234 AS--------------------------------------A--------M--N----ANQ
XP_010661562.1/1-234 AS--------------------------------------A--------M--N----ANQ
XP_002511801.1/1-237 AS--------------------------------------A--------M--N----ANQ
XP_015584500.1/1-237 AS--------------------------------------A--------M--N----ANQ
XP_022141257.1/1-238 AN--------------------------------------P--------M--N----ANE
XP_023885612.1/1-244 GN--------------------------------------S--------T--N----ESE
XP_023885611.1/1-244 GN--------------------------------------S--------T--N----ESE
XP_018830842.1/1-239 GS--------------------------------------P--------T--N----ANE
XP_018830843.1/1-239 GS--------------------------------------P--------T--N----ANE
XP_018830840.1/1-239 GS--------------------------------------P--------T--N----ANE
XP_018830841.1/1-239 GS--------------------------------------P--------T--N----ANE
XP_012090293.1/1-240 SS--------------------------------------A--------VSPN----TNE
XP_012090292.1/1-240 SS--------------------------------------A--------VSPN----TNE
XP_021614581.1/1-239 ST--------------------------------------A--------VSPN----ANE
XP_021614580.1/1-239 ST--------------------------------------A--------VSPN----ANE
XP_015581247.1/1-240 ST--------------------------------------A--------VSPN----AND
XP_002510822.1/1-240 ST--------------------------------------A--------VSPN----AND
XP_021648646.1/1-240 SS--------------------------------------A--------VSPN----ANE
XP_021634593.1/1-234 AS--------------------------------------A--------VSAN----ANE
XP_021634592.1/1-234 AS--------------------------------------A--------VSAN----ANE
XP_006490348.1/1-238 AG--------------------------------------A--------M--N----AND
XP_006490346.1/1-238 AG--------------------------------------A--------M--N----AND
XP_024958654.1/1-238 AG--------------------------------------A--------M--N----AND
XP_006490345.1/1-238 AG--------------------------------------A--------M--N----AND
XP_006421879.2/1-238 AG--------------------------------------A--------M--N----AND
XP_021892212.1/1-221 AS--------------------------------------A--------N--D----GNE
XP_021892211.1/1-221 AS--------------------------------------A--------N--D----GNE
XP_021892210.1/1-221 AS--------------------------------------A--------N--D----GNE
XP_022765148.1/1-244 GS--------------------------------------A--------I--N----ANE
XP_022765158.1/1-244 GS--------------------------------------A--------I--N----ANE
XP_022765120.1/1-244 GS--------------------------------------A--------I--N----ANE
XP_022765114.1/1-244 GS--------------------------------------A--------I--N----ANE
XP_022736693.1/1-237 GT--------------------------------------A--------I--N----ANE
XP_022736692.1/1-237 GT--------------------------------------A--------I--N----ANE
XP_022736689.1/1-237 GT--------------------------------------A--------I--N----ANE
XP_022734775.1/1-244 GS--------------------------------------A--------I--N----ANE
XP_016743353.1/1-244 GS--------------------------------------A--------I--N----ANE
XP_012474605.1/1-244 GS--------------------------------------A--------I--N----ANE
XP_012474606.1/1-244 GS--------------------------------------A--------I--N----ANE
XP_016743352.1/1-244 GS--------------------------------------A--------I--N----ANE
XP_016743351.1/1-244 GS--------------------------------------A--------I--N----ANE
XP_012474604.1/1-244 GS--------------------------------------A--------I--N----ANE
XP_012474603.1/1-244 GS--------------------------------------A--------I--N----ANE
XP_017646297.1/1-244 GS--------------------------------------A--------I--N----ANE
XP_016736315.1/1-244 GS--------------------------------------A--------I--N----ANE
XP_017646296.1/1-244 GS--------------------------------------A--------I--N----ANE
XP_017646298.1/1-244 GS--------------------------------------A--------I--N----ANE
XP_016736306.1/1-244 GS--------------------------------------A--------I--N----ANE
XP_017646295.1/1-244 GS--------------------------------------A--------I--N----ANE
XP_017972896.1/1-244 GS--------------------------------------A--------I--N----ANE
XP_017972895.1/1-244 GS--------------------------------------A--------I--N----ANE
XP_017972893.1/1-244 GS--------------------------------------A--------I--N----ANE
XP_021274823.1/1-244 GS--------------------------------------A--------I--N----ANE
XP_024536343.1/1-231 VN--------------------------------------A--------L--N----TSQ
XP_006366174.1/1-244 AS--------------------------------------V--------M--N----ASQ
XP_020219711.1/1-212 AS--------------------------------------A--------I--N----ANQ
XP_022897056.1/1-222 AS----------------------------------------------------------
NP_001281015.1/1-243 AR--------------------------------------A-----------N----ANQ
XP_008383260.1/1-243 AR--------------------------------------A-----------N----ANQ
XP_022896229.1/1-235 AS--------------------------------------A--------M--N----ASQ
XP_022896230.1/1-235 AS--------------------------------------A--------M--N----ASQ
XP_025816451.1/1-225 AS--------------------------------------A--------M--S----ANQ
XP_004968001.1/1-225 AS--------------------------------------A--------M--S----ANQ
XP_004968002.1/1-225 AS--------------------------------------A--------M--S----ANQ
XP_002457484.1/1-226 AS--------------------------------------A--------M--S----ANQ
XP_009400446.1/1-224 AS--------------------------------------A--------V--S----ADQ
XP_018676535.1/1-225 AS--------------------------------------A--------A--N----ATQ
XP_009401771.1/1-232 AS--------------------------------------A--------M--N----ANQ
XP_009384534.1/1-228 AS--------------------------------------A--------V--S----ASQ
XP_020672447.1/1-217 AN--------------------------------------A--------V--N----TNN
XP_020582116.1/1-218 AN--------------------------------------A--------L--N----TDQ
XP_020675647.1/1-231 AS--------------------------------------A--------V--S----ANQ
XP_020675648.1/1-231 AS--------------------------------------A--------V--S----ANQ
XP_020585632.1/1-228 VS--------------------------------------A--------V--S----ANQ
XP_016176402.1/1-228 AN--------------------------------------G--------V--N----AND
XP_016176403.1/1-228 AN--------------------------------------G--------V--N----AND
XP_016176400.1/1-228 AN--------------------------------------G--------V--N----AND
XP_016176401.1/1-228 AN--------------------------------------G--------V--N----AND
XP_010523042.1/1-245 SN--------------------------------------A--------M--N----ASQ
XP_010523041.1/1-240 SN--------------------------------------A--------M--N----ASQ
XP_020878511.1/1-224 SN--------------------------------------A--------M--N----ANQ
NP_567183.2/1-228    SN--------------------------------------A--------M--N----ANQ
XP_006287095.1/1-228 SN--------------------------------------A--------M--N----ANQ
XP_010456250.1/1-228 SN--------------------------------------A--------M--N----ANQ
XP_010419627.1/1-228 SN--------------------------------------A--------M--N----ANQ
XP_010427345.1/1-228 SN--------------------------------------A--------M--N----ANQ
XP_009111469.1/1-229 SN--------------------------------------P--------M--N----ANQ
XP_018487039.1/1-229 SN--------------------------------------P--------M--N----ANQ
XP_018453129.1/1-229 SN--------------------------------------P--------L--N----ANQ
XP_013612299.1/1-229 SN--------------------------------------P--------M--N----ANQ
XP_006396268.1/1-225 SN--------------------------------------P--------M--N----ANQ
XP_018475317.1/1-221 SN--------------------------------------P--------M--N----TNQ
XP_013629750.1/1-221 SN--------------------------------------P--------M--N----ANQ
XP_009134533.1/1-221 SN--------------------------------------P--------M--N----ANQ
XP_020531377.1/1-244 AS--------------------------------------A--------M--N----PTQ
XP_006857866.1/1-244 AS--------------------------------------A--------M--N----PTQ
XP_018434263.1/1-219 ST--------------------------------------A--------M--N----ASQ
XP_018434260.1/1-219 ST--------------------------------------A--------M--N----ASQ
XP_018434262.1/1-219 ST--------------------------------------A--------M--N----ASQ
XP_013688926.1/1-219 ST--------------------------------------A--------M--N----ASQ
XP_013637558.1/1-219 ST--------------------------------------A--------M--N----ASQ
XP_013637556.1/1-219 ST--------------------------------------A--------M--N----ASQ
XP_013637557.1/1-219 ST--------------------------------------A--------M--N----ASQ
XP_009143168.1/1-219 ST--------------------------------------A--------M--N----ASQ
XP_009143167.1/1-219 ST--------------------------------------A--------M--N----ASQ
XP_009143166.1/1-219 ST--------------------------------------A--------M--N----ASQ
XP_013692686.1/1-219 ST--------------------------------------A--------M--N----ASQ
XP_022574820.1/1-219 ST--------------------------------------A--------M--N----ASQ
XP_013692684.1/1-219 ST--------------------------------------A--------M--N----ASQ
XP_013748127.1/1-219 ST--------------------------------------A--------M--N----ASQ
XP_013692685.1/1-219 ST--------------------------------------A--------M--N----ASQ
XP_013748124.1/1-219 ST--------------------------------------A--------M--N----ASQ
XP_013748126.1/1-219 ST--------------------------------------A--------M--N----ASQ
XP_022574821.1/1-219 ST--------------------------------------A--------M--N----ASQ
XP_006290613.1/1-229 AS--------------------------------------A--------V--N----ANQ
XP_013592301.1/1-230 AT--------------------------------------A--------I--N----ANQ
XP_018442581.1/1-230 AT--------------------------------------A--------I--N----ANQ
XP_013750213.1/1-232 AT--------------------------------------A--------I--N----ANQ
XP_013648430.1/1-232 AT--------------------------------------A--------I--N----ANQ
XP_009104292.1/1-232 AT--------------------------------------A--------I--N----ANQ
XP_019058514.1/1-223 VS--------------------------------------A--------M--N----ANE
XP_010523728.1/1-220 AS--------------------------------------A--------M--N----ANQ
XP_024514927.1/1-242 VG--------------------------------------A--------L--N----TSQ
XP_002985242.1/1-242 VG--------------------------------------A--------L--N----TSQ
XP_024530500.1/1-242 VG--------------------------------------A--------L--N----TSQ
XP_024530499.1/1-242 VG--------------------------------------A--------L--N----TSQ
XP_024530501.1/1-242 VG--------------------------------------A--------L--N----TSQ
XP_010107411.1/1-242 AS--------------------------------------T--------I--G----TND
XP_024028635.1/1-242 AS--------------------------------------T--------I--G----TND
XP_015877999.1/1-244 AS--------------------------------------A--------I--N----ASE
XP_019427294.1/1-247 AG--------------------------------------S--------V--N----AND
XP_019427292.1/1-247 AG--------------------------------------S--------V--N----AND
XP_019452814.1/1-239 AS--------------------------------------A--------V--N----AND
XP_019452815.1/1-239 AS--------------------------------------A--------V--N----AND
XP_003592295.2/1-235 AN--------------------------------------A--------V--N----AND
XP_024625328.1/1-235 AN--------------------------------------A--------V--N----AND
XP_012570175.1/1-236 AN--------------------------------------A--------V--N----AND
XP_012570176.1/1-236 AN--------------------------------------A--------V--N----AND
XP_004496593.1/1-236 AN--------------------------------------A--------V--N----AND
XP_025620154.1/1-234 AN--------------------------------------G--------V--N----AND
XP_015941562.1/1-234 AN--------------------------------------G--------V--N----AND
XP_025620153.1/1-234 AN--------------------------------------G--------V--N----AND
XP_020239673.1/1-232 AN--------------------------------------A--------V--N----AND
XP_020239648.1/1-232 AN--------------------------------------A--------V--N----AND
XP_007143377.1/1-235 AN--------------------------------------A--------V--N----AND
XP_007143378.1/1-235 AN--------------------------------------A--------V--N----AND
XP_017415467.1/1-234 AN--------------------------------------A--------V--N----AND
XP_017415466.1/1-234 AN--------------------------------------A--------V--N----AND
XP_014514421.1/1-236 AN--------------------------------------A--------V--N----AND
XP_014514419.1/1-236 AN--------------------------------------A--------V--N----AND
XP_006606097.1/1-235 AN--------------------------------------A--------V--N----AND
XP_006606096.1/1-235 AN--------------------------------------A--------V--N----AND
XP_003536477.1/1-234 AN--------------------------------------A--------V--N----AND
XP_006589557.1/1-234 AN--------------------------------------A--------V--N----AND
XP_006402516.1/1-230 AT--------------------------------------A--------I--N----ANQ
NP_191674.1/1-237    AS--------------------------------------A--------I--N----ANQ
XP_002876596.1/1-239 AS--------------------------------------A--------I--N----ANQ
XP_010413451.1/1-238 AS--------------------------------------A--------V--N----ANQ
XP_010413452.1/1-238 AS--------------------------------------A--------V--N----ANQ
XP_010413453.1/1-238 AS--------------------------------------A--------V--N----ANQ
XP_010469036.1/1-242 AS--------------------------------------A--------V--N----ANQ
XP_010469037.1/1-242 AS--------------------------------------A--------V--N----ANQ
XP_010469038.1/1-242 AS--------------------------------------A--------V--N----ANQ
XP_010512448.1/1-238 AS--------------------------------------A--------V--S----ANQ
XP_010512449.1/1-238 AS--------------------------------------A--------V--S----ANQ
XP_006397757.1/1-219 ST--------------------------------------A--------M--N----ANQ
XP_024010837.1/1-220 STQ-------------------------------------A--------M--N----ANQ
XP_023640300.1/1-220 ST--------------------------------------A--------M--N----ANQ
XP_006293718.1/1-220 ST--------------------------------------A--------M--N----ANQ
XP_010506553.1/1-219 ST--------------------------------------A--------M--N----ANQ
XP_010508114.1/1-219 ST--------------------------------------A--------M--N----ANQ
XP_010508115.1/1-219 ST--------------------------------------A--------M--N----ANQ
XP_022147276.1/1-219 AAQ-------------------------------------S--------L--N----PSQ
XP_023527464.1/1-222 ATQ-------------------------------------S--------L--N----PNQ
XP_022980233.1/1-222 ATQ-------------------------------------S--------L--N----PNQ
XP_022980231.1/1-222 ATQ-------------------------------------S--------L--N----PNQ
XP_022924366.1/1-223 ATQ-------------------------------------S--------L--N----PNQ
XP_022924364.1/1-223 ATQ-------------------------------------S--------L--N----PNQ
XP_023527467.1/1-221 AT--------------------------------------S--------L--N----PNQ
XP_023527463.1/1-221 AT--------------------------------------S--------L--N----PNQ
XP_023527465.1/1-221 AT--------------------------------------S--------L--N----PNQ
XP_022980232.1/1-221 AT--------------------------------------S--------L--N----PNQ
XP_022924365.1/1-222 AT--------------------------------------S--------L--N----PNQ
XP_009412599.1/1-224 AS--------------------------------------A--------A--S----SNQ
XP_018677084.1/1-231 AS--------------------------------------A--------V--S----ADH
XP_020114860.1/1-246 AG--------------------------------------A--------V--N----SNQ
XP_020114857.1/1-246 AG--------------------------------------A--------V--N----SNQ
XP_020114859.1/1-246 AG--------------------------------------A--------V--N----SNQ
XP_020084803.1/1-234 AA--------------------------------------A--------A--S----ANQ
XP_015625616.1/1-226 AS--------------------------------------A--------V--S----ANQ
XP_006647677.1/1-226 AS--------------------------------------A--------V--S----ANQ
NP_001105493.1/1-226 AS--------------------------------------A--------M--S----ANQ
XP_020178635.1/1-225 AS--------------------------------------A--------M--S----ANQ
XP_003570085.1/1-223 AS--------------------------------------A--------M--S----ANQ
XP_020247871.1/1-226 INA-------------------------------------P----------SN-----SS
XP_020247870.1/1-226 INA-------------------------------------P----------SN-----SS
XP_024004764.1/1-211 INV-------------------------------------A----------TK----SSN
XP_018449374.1/1-217 FHG-------------------------------------P----------SS----SSN
XP_013602839.1/1-215 FHG-------------------------------------A----------SS-----SN
XP_013655966.1/1-215 FHG-------------------------------------A----------SS-----SN
XP_009108342.1/1-215 FHG-------------------------------------A----------SS-----SN
XP_013655964.1/1-215 FHG-------------------------------------A----------SS-----SN
XP_020872468.1/1-222 INV-------------------------------------E----------NN----SSQ
NP_193506.2/1-221    INV-------------------------------------E----------SN----SSH
XP_023633855.1/1-222 INV-------------------------------------E----------SN----SSH
XP_010449572.1/1-221 INM-------------------------------------E----------SN----TWH
XP_010439957.1/1-223 INM-------------------------------------E----------SN----TWH
XP_010439959.1/1-223 INM-------------------------------------E----------SN----TWH
XP_010439958.1/1-223 INM-------------------------------------E----------SN----TWH
XP_006854056.1/1-234 VNT-------------------------------------G----------GN----TPQ
XP_020529072.1/1-234 VNT-------------------------------------G----------GN----TPQ
NP_199499.3/1-224    INV-------------------------------------A----------SN----SWH
XP_020871346.1/1-224 INV-------------------------------------A----------SN----SWH
XP_006281890.1/1-224 INV-------------------------------------A----------SN----SWH
XP_010481400.1/1-224 INV-------------------------------------A----------SN----SWH
XP_010494885.1/1-224 INV-------------------------------------A----------SN----SWH
XP_018455882.1/1-227 INV-------------------------------------A----------SN----SWH
XP_013713146.1/1-227 INV-------------------------------------A----------SN----SWH
XP_013607209.1/1-227 INV-------------------------------------A----------SN----SWH
XP_009114317.1/1-227 VNV-------------------------------------A----------SN----SWH
XP_013661958.1/1-227 INV-------------------------------------A----------SN----SWH
XP_006398384.1/1-227 INV-------------------------------------A----------SN----SWH
XP_024011152.1/1-227 INV-------------------------------------A----------SN----SWH
XP_018482944.1/1-227 ISV-------------------------------------S----------SN----SWH
XP_009101472.1/1-227 INV-------------------------------------S----------SN----SWH
XP_013693979.1/1-227 INV-------------------------------------S----------SN----SWH
XP_013615089.1/1-227 INV-------------------------------------S----------SN----SWH
XP_013704013.1/1-227 INV-------------------------------------S----------SN----SWH
XP_009373477.1/1-246 INV-------------------------------------S----------SN----SSQ
XP_006662631.2/1-256 INA-------------------------------------A----------SN----SSQ
XP_015613461.1/1-259 INA-------------------------------------A----------SN----SSQ
NP_001337805.1/1-240 INA-------------------------------------A----------SN----SSQ
XP_020163828.1/1-239 INS-------------------------------------A----------SN----SSQ
XP_010235052.1/1-238 INA-------------------------------------A----------SN----SSQ
XP_025798256.1/1-238 INA-------------------------------------A----------SN----SSQ
XP_004983555.1/1-238 INA-------------------------------------A----------SN----SSQ
XP_002464880.2/1-238 INA-------------------------------------A----------SN----SSQ
XP_020262583.1/1-231 INS------------------------------------------------NN----SSQ
XP_020677410.1/1-231 INA-------------------------------------A----------SN----SSQ
XP_020577604.1/1-229 VNA-------------------------------------A----------SN----SSQ
XP_020577606.1/1-229 VNA-------------------------------------A----------SN----SSQ
XP_020577607.1/1-229 VNA-------------------------------------A----------SN----SSQ
XP_019709148.1/1-245 INA-------------------------------------A----------SN----SSQ
XP_008803567.1/1-241 IDA-------------------------------------A----------SN----SSQ
XP_008803570.2/1-241 IDA-------------------------------------A----------SN----SSQ
XP_018681950.1/1-231 VNA-------------------------------------A----------SN----SSH
XP_018681951.1/1-231 VNA-------------------------------------A----------SN----SSH
XP_010919976.1/1-237 INA-------------------------------------A----------SN----SSQ
XP_008800084.1/1-237 INA-------------------------------------A----------SN----SSQ
XP_018679997.1/1-229 VNA-------------------------------------A----------SN----SSH
XP_009397149.1/1-229 VNA-------------------------------------A----------SN----SSH
XP_024995523.1/1-235 VNV-------------------------------------A----------SN----SSQ
XP_023736252.1/1-233 LNV-------------------------------------A----------SN----SSQ
XP_023736253.1/1-233 LNV-------------------------------------A----------SN----SSQ
XP_022022041.1/1-231 VNV-------------------------------------A----------SN----SSQ
XP_021969857.1/1-226 VNV-------------------------------------A----------CN----SSQ
XP_010245213.1/1-236 INA-------------------------------------S----------SN----SSQ
XP_010245210.1/1-236 INA-------------------------------------S----------SN----SSQ
XP_010276832.1/1-235 INA-------------------------------------S----------SN----SSQ
XP_010276837.1/1-235 INA-------------------------------------S----------SN----SSQ
XP_019155823.1/1-230 INV-------------------------------------A----------SN----SSQ
XP_022881662.1/1-238 INV-------------------------------------A----------SN----SSQ
XP_022877113.1/1-234 INV-------------------------------------A----------SN----SSQ
XP_016450789.1/1-246 INV-------------------------------------A----------SN----SSQ
XP_009597691.1/1-246 INV-------------------------------------A----------SN----SSQ
XP_009786380.1/1-246 INV-------------------------------------A----------SN----SSQ
XP_016494823.1/1-246 INV-------------------------------------A----------SN----SSQ
XP_016494822.1/1-246 INV-------------------------------------A----------SN----SSQ
XP_019230103.1/1-246 INV-------------------------------------A----------SN----SSQ
XP_016573090.1/1-250 INV-------------------------------------A----------SN----SSQ
XP_016573102.1/1-250 INV-------------------------------------A----------SN----SSQ
XP_006352911.1/1-250 INV-------------------------------------A----------SN----SSQ
XP_015166604.1/1-250 INV-------------------------------------A----------SN----SSQ
XP_015085651.1/1-250 INV-------------------------------------A----------SN----SSQ
XP_015085654.1/1-250 INV-------------------------------------A----------SN----SSQ
XP_004245919.1/1-250 INV-------------------------------------A----------SN----SSQ
XP_025888165.1/1-250 INV-------------------------------------A----------SN----SSQ
XP_011070327.1/1-236 INV-------------------------------------A----------SN----SSQ
XP_011070329.1/1-236 INV-------------------------------------A----------SN----SSQ
XP_012843107.1/1-236 INV-------------------------------------A----------SN----SSQ
XP_019423575.1/1-234 INV-------------------------------------A----------SN----SSQ
XP_019423571.1/1-234 INV-------------------------------------A----------SN----SSQ
XP_019435331.1/1-234 INV-------------------------------------A----------SN----SSQ
XP_003612811.1/1-239 INV-------------------------------------A----------SN----SSQ
XP_020978415.1/1-227 INV-------------------------------------A----------SN----SSQ
XP_015963259.1/1-227 INV-------------------------------------A----------SN----SSQ
XP_025697880.1/1-227 INV-------------------------------------A----------SN----SSQ
XP_025697882.1/1-227 INV-------------------------------------A----------SN----SSQ
XP_025697881.1/1-227 INV-------------------------------------A----------SN----SSQ
XP_020217931.1/1-233 INV-------------------------------------A----------SN----SSQ
XP_007158253.1/1-232 INV-------------------------------------A----------SN----SSQ
XP_017427665.1/1-232 INV-------------------------------------A----------SN----SSQ
XP_017427666.1/1-232 INV-------------------------------------A----------SN----SSQ
XP_014521371.1/1-232 INV-------------------------------------A----------SN----SSQ
XP_014521373.1/1-232 INV-------------------------------------A----------SN----SSQ
XP_006587604.1/1-242 INV-------------------------------------A----------SN----SSQ
NP_001352051.1/1-233 INV-------------------------------------A----------SN----SSQ
XP_018840870.1/1-239 INV-------------------------------------A----------SN----SSQ
XP_018852246.1/1-240 INV-------------------------------------A----------SN----SSQ
XP_010053633.1/1-234 INV-------------------------------------A----------SN----SSQ
XP_021675948.1/1-236 INV-------------------------------------A----------SN----SSQ
XP_012081064.1/1-228 INV-------------------------------------A----------SN----SSQ
XP_011028126.1/1-235 INV-------------------------------------A----------SN----SSQ
XP_002303433.2/1-235 INV-------------------------------------A----------SN----SSQ
XP_015897558.1/1-247 INV-------------------------------------A----------SN----SSQ
XP_002277673.1/1-238 INV-------------------------------------A----------SN----SSQ
XP_022996123.1/1-236 INV-------------------------------------A----------SN----FSQ
XP_023534245.1/1-236 INV-------------------------------------A----------SN----FSQ
XP_022931244.1/1-236 INV-------------------------------------A----------SN----FSQ
XP_022931242.1/1-236 INV-------------------------------------A----------SN----FSQ
XP_022159691.1/1-235 INV-------------------------------------A----------SN----SSQ
XP_011652640.1/1-232 INV-------------------------------------A----------SN----SSQ
XP_011652639.1/1-232 INV-------------------------------------A----------SN----SSQ
XP_008466007.1/1-232 INV-------------------------------------A----------SN----SSQ
XP_023511589.1/1-230 INV-------------------------------------A----------SN----SSQ
XP_022963906.1/1-230 INV-------------------------------------A----------SN----SSQ
XP_023511591.1/1-230 INV-------------------------------------A----------SN----SSQ
XP_022963908.1/1-230 INV-------------------------------------A----------SN----SSQ
XP_022967184.1/1-230 INV-------------------------------------A----------SN----SSQ
XP_022967186.1/1-230 INV-------------------------------------A----------SN----SSQ
XP_010518669.1/1-225 VNV-------------------------------------A----------SN----SSH
XP_010677365.1/1-235 INV-------------------------------------A----------SN----SSQ
XP_010677364.1/1-235 INV-------------------------------------A----------SN----SSQ
XP_021852084.1/1-226 INV-------------------------------------A----------SN----SSQ
XP_021852076.1/1-226 INV-------------------------------------A----------SN----SSQ
XP_021765591.1/1-233 INV-------------------------------------A----------SN----SSQ
XP_021768254.1/1-233 INV-------------------------------------A----------SN----SSQ
XP_017232690.1/1-221 INV-------------------------------------A----------SN----SSQ
XP_017232692.1/1-221 INV-------------------------------------A----------SN----SSQ
XP_024021697.1/1-248 INV-------------------------------------A----------SN----SSQ
XP_010096931.1/1-248 INV-------------------------------------A----------SN----SSQ
XP_024021696.1/1-248 INV-------------------------------------A----------SN----SSQ
XP_017621310.1/1-230 INV-------------------------------------S----------SN----SSQ
XP_016731150.1/1-230 INV-------------------------------------A----------SN----SSQ
XP_012472199.1/1-230 INV-------------------------------------A----------SN----SSQ
XP_016727305.1/1-230 INV-------------------------------------A----------SN----SSQ
XP_016731145.1/1-230 INV-------------------------------------A----------SN----SSQ
XP_012472200.1/1-230 INV-------------------------------------S----------SN----SSQ
XP_012472201.1/1-230 INV-------------------------------------S----------SN----SSQ
NP_001314348.1/1-230 INV-------------------------------------S----------SN----SSQ
XP_016727300.1/1-230 INV-------------------------------------S----------SN----SSQ
XP_022738572.1/1-235 INV-------------------------------------A----------SN----STQ
XP_021297648.1/1-235 INV-------------------------------------A----------SN----SSQ
XP_017973778.1/1-235 INV-------------------------------------A----------SN----SSQ
XP_017973780.1/1-235 INV-------------------------------------A----------SN----SSQ
XP_022717458.1/1-235 INV-------------------------------------A----------SN----SSQ
XP_022717453.1/1-235 INV-------------------------------------A----------SN----SSQ
XP_022717437.1/1-235 INV-------------------------------------A----------SN----SSQ
XP_022756982.1/1-232 INV-------------------------------------A----------SN----SSQ
XP_004300207.1/1-230 VNV-------------------------------------A----------SN----STQ
XP_024169578.1/1-230 VNV-------------------------------------A----------SN----STQ
XP_008238183.1/1-249 INV-------------------------------------A----------SN----SAQ
XP_016651445.1/1-249 INV-------------------------------------A----------SN----SAQ
XP_020418924.1/1-249 INV-------------------------------------A----------SN----SAQ
XP_021804146.1/1-249 INV-------------------------------------A----------SN----SAQ
XP_008238184.1/1-248 INV-------------------------------------A----------SN----SAQ
XP_016651446.1/1-248 INV-------------------------------------A----------SN----SAQ
XP_007209851.1/1-248 INV-------------------------------------A----------SN----SAQ
XP_021804154.1/1-248 INV-------------------------------------A----------SN----SAQ
XP_017179076.1/1-239 INV-------------------------------------S----------SN----SSQ
XP_008356445.1/1-246 INV-------------------------------------S----------SN----SSQ
XP_008356446.1/1-240 INV-------------------------------------S----------SN----SSQ
XP_008356447.1/1-239 INV-------------------------------------S----------SN----SSQ
XP_009373485.1/1-239 INV-------------------------------------S----------SN----SSQ
XP_009373484.1/1-240 INV-------------------------------------S----------SN----SSQ
XP_004512486.1/1-245 INV-------------------------------------A----------SN----SSQ
XP_023898631.1/1-242 INV-------------------------------------A----------SN----SSQ
XP_024465375.1/1-238 INV-------------------------------------A----------SN----SSQ
XP_011018502.1/1-238 INV-------------------------------------A----------SN----SSQ
XP_011018503.1/1-238 INV-------------------------------------A----------SN----SSQ
XP_021893615.1/1-233 INV-------------------------------------A----------SN----SSQ
XP_024952893.1/1-247 INV-------------------------------------A----------SN----SSQ
XP_006471522.1/1-248 INV-------------------------------------A----------SN----SSQ
XP_006432745.2/1-248 INV-------------------------------------A----------SN----SSQ
XP_021640807.1/1-241 INV-------------------------------------A----------SN----SSQ
XP_002518976.1/1-243 INV-------------------------------------A----------SN----SSQ
XP_021598537.1/1-237 INV-------------------------------------A----------SN----SSQ
XP_021598535.1/1-239 INV-------------------------------------A----------SN----SSQ
XP_006659280.1/1-226 PRE-------------------------------------I----------D-----GSI
XP_025801379.1/1-254 SNK-------------------------------------T----------D-G---T-N
XP_023156326.1/1-229 SNV-------------------------------------T-----------DG---TDR
NP_001349156.1/1-229 SNV-------------------------------------T-----------DG---TDR
XP_021310588.1/1-246 SNV-------------------------------------T----------TDG---TDS
XP_020404516.1/1-224 PNV-------------------------------------T----------N-----AAN
XP_022684239.1/1-229 PSD-------------------------------------V----------TVR----AN
XP_020404892.1/1-233 STS-------------------------------------S----------VDTD--GTD
XP_022684238.1/1-237 PTI-------------------------------------V----------AQGSRGM-N
XP_025825251.1/1-229 ---------------------------------------------------ADGSHGTNS
XP_020183090.1/1-242 PTA-------------------------------------M----------ARGRGRSSC
XP_020194819.1/1-240 TIA-------------------------------------S-------------NGA---
XP_024314575.1/1-244 PSV-------------------------------------Y-------------NGTTNK
XP_024314342.1/1-235 AIA-------------------------------------S-------------DGT-NN
XP_024959674.1/1-192 IEI-------------------------------------P----------G-------M
XP_019196338.1/1-215 VE-------------------------------------------------SPA---TNK
XP_006646400.1/1-226 VKR------------------------------------------------APG---ENK
XP_015632244.1/1-226 CKR------------------------------------------------AGR---EDR
XP_024314625.1/1-231 VKA-------------------------------------A------------T---GDK
XP_020164122.1/1-228 LKD-------------------------------------P----------PTG---ENR
XP_025814677.1/1-221 VKH------------------------------------------------GSG---ANK
XP_022682466.1/1-225 VKH------------------------------------------------GSG---TNK
XP_002456467.1/1-226 VKH------------------------------------------------GHG---ANR
XP_008674944.1/1-228 VKH------------------------------------------------GHG---ANR
XP_010523539.1/1-228 VRG------------------------------------------------NS----SGA
XP_020873847.1/1-203 FRL------------------------------------------------N------NS
NP_001190248.1/1-203 IEW------------------------------------------------K------GS
NP_196343.2/1-203    IEW------------------------------------------------K------GS
XP_020876233.1/1-195 IEW------------------------------------------------N------GS
XP_010423235.1/1-188 IEC------------------------------------------------N------GP
XP_010452682.1/1-218 IEC------------------------------------------------N------GP
XP_010491330.1/1-188 IEC------------------------------------------------N------GP
XP_006413300.2/1-224 ---------------------------------------------------FAE---GVE
XP_022008499.1/1-216 SNT------------------------------------------------LQA---GVN
XP_022008502.1/1-215 SNT------------------------------------------------LQA---GVY
XP_022007635.1/1-226 SNI------------------------------------------------ATP---TED
XP_022008501.1/1-224 SNI------------------------------------------------EMP---TED
XP_021986984.1/1-226 SNM------------------------------------------------ERT---TED
XP_021996009.1/1-224 SNM------------------------------------------------ERT---NED
XP_021996011.1/1-224 SNM------------------------------------------------ERT---TED
XP_022026050.1/1-224 SNI------------------------------------------------ERT---TED
XP_022026083.1/1-224 SNI------------------------------------------------ERT---TED
XP_021722389.1/1-209 MVD------------------------------------------------G-----KGR
XP_021847315.1/1-219 MVD------------------------------------------------E-----KGR
XP_020224300.1/1-216 IYN------------------------------------------------R------IE
XP_021765248.1/1-224 PMN------------------------------------------------P------NE
XP_004510020.1/1-219 TYN------------------------------------------------F-----DNE
XP_004487953.1/1-231 TLN------------------------------------------------K-----R-E
XP_008244376.1/1-218 AIN------------------------------------------------DE----TKK
XP_008244380.1/1-218 AIN------------------------------------------------DE----TKK
XP_012569676.1/1-224 PFD------------------------------------------------S------NE
XP_019418393.1/1-229 EKN------------------------------------------------PSA---NPS
XP_019440118.1/1-223 ETK------------------------------------------------PA----VPR
XP_014517363.2/1-226 PLH------------------------------------------------R-----TA-
XP_012569675.1/1-231 PME------------------------------------------------A-----PPP
XP_004486570.1/1-223 PME------------------------------------------------P-----RCE
XP_003594569.3/1-220 PLV------------------------------------------------S-----PPK
XP_024632692.1/1-220 PLV------------------------------------------------S-----PPK
XP_025887127.1/1-229 AHG-------------------------------------D----------ESC---A--
XP_015078684.1/1-230 AHG-------------------------------------D----------ESC---A--
XP_025887126.1/1-230 AHG-------------------------------------D----------ESC---A--
XP_015078401.1/1-220 ANA-------------------------------------H----------GTS---TNQ
XP_015158871.1/1-217 VNG-------------------------------------H----------DTS---TNQ
XP_015078778.1/1-220 ANG-------------------------------------G----------------ANE
XP_010321992.1/1-216 ANG-------------------------------------G----------------ANE
XP_004240735.1/1-216 ANG-------------------------------------G----------------ANE
XP_015169793.1/1-213 ADG-------------------------------------D----------STS---AKQ
XP_006285631.1/1-212 VIG-------------------------------------------------------DE
XP_023635531.1/1-198 VIG-------------------------------------------------------DE
NP_567722.1/1-217    IVN---------------------------------------------------------
XP_020873337.1/1-219 FAG---------------------------------------------------------
XP_020873338.1/1-219 FAG---------------------------------------------------------
XP_023000325.1/1-225 IKS-------------------------------------------------------DE
XP_023000326.1/1-214 IKS-------------------------------------------------------DE
XP_022963997.1/1-214 IKS-------------------------------------------------------DE
XP_023513617.1/1-208 IKS-------------------------------------------------------DE
XP_021312124.1/1-225 MKS-------------------------------------S-----------VE----EL
XP_012701492.1/1-243 ARS-------------------------------------P-----------AG----EQ
XP_025815339.1/1-229 GTS-------------------------------------P-----------AG----DQ
XP_015644459.1/1-237 ARP-------------------------------------E-----------EE---EER
XP_024314016.1/1-228 ASS-------------------------------------S-----------AG---AQH
XP_020168333.1/1-225 SSR-------------------------------------A-----------D-----GQ
XP_013589242.1/1-206 IEP-------------------------------------G-------------------
XP_022548456.1/1-204 IEP-------------------------------------G-------------------
XP_024632691.1/1-227 SFI-------------------------------------P-----------------TQ
XP_017256377.1/1-220 VEV-------------------------------------A----------NAR-HLLNK
XP_009632057.1/1-224 VEP-------------------------------------P----------NGR-E--DK
XP_016502932.1/1-224 VEP-------------------------------------P----------NGR-E--DK
XP_019258997.1/1-221 VEP-------------------------------------P----------NGR-E--DK
XP_009792862.1/1-224 VEP-------------------------------------P----------NGR-E--DK
XP_016544784.1/1-213 VEA-------------------------------------P----------NGR-T--DK
XP_015089689.1/1-213 VEP-------------------------------------P----------NGR-T--NK
XP_025883585.1/1-213 VEP-------------------------------------P----------NGR-T--NK
XP_011095790.1/1-221 VEG-------------------------------------S----------S------NR
XP_020553953.1/1-221 VEG-------------------------------------S----------S------NR
XP_022857758.1/1-208 VEP-------------------------------------T----------E------NR
XP_004487739.1/1-224 AST-------------------------------------S----------------KGK
XP_003596663.1/1-221 A--------------------------------------------------------RDK
XP_003596665.1/1-227 AST-------------------------------------S----------------EDK
XP_020201972.1/1-216 ATT-------------------------------------S----------------QGK
XP_025980734.1/1-220 ANT-------------------------------------S----------------QGK
XP_007149715.1/1-218 ANN-------------------------------------S----------------EGK
XP_017423636.1/1-218 AHN-------------------------------------S----------------QGK
XP_022631965.1/1-218 AHN-------------------------------------S----------------QGK
XP_014522034.2/1-218 AHN-------------------------------------S----------------QGK
XP_025669173.1/1-223 AMK-------------------------------------S----------------EDK
XP_020963786.1/1-223 AMK-------------------------------------S----------------EDK
XP_025614692.1/1-223 AMK-------------------------------------S----------------EDK
XP_015936351.1/1-223 AMK-------------------------------------S----------------EDK
XP_019451675.1/1-226 AFT-------------------------------------S----------------EDK
XP_019465428.1/1-219 AIN-------------------------------------S----------------EDK
XP_004295798.2/1-220 VEN-------------------------------------G-----------A----TKE
XP_021807338.1/1-219 AIN-------------------------------------D-----------E----TKK
XP_021807340.1/1-219 AIN-------------------------------------D-----------E----TKK
XP_020417197.1/1-219 AIN-------------------------------------D-----------E----TKK
XP_020417200.1/1-219 AIN-------------------------------------D-----------E----TKK
XP_009333923.1/1-206 AFK-------------------------------------S-----------N------E
XP_017184363.1/1-206 AFK-------------------------------------S-----------N------E
XP_021892911.1/1-224 SYT-------------------------------------S----------------TKR
XP_002278971.2/1-210 VKS-------------------------------------P----------ED------Q
XP_010644412.1/1-211 VKS-------------------------------------A----------ED----PDR
XP_024450660.1/1-210 VNS-------------------------------------A----------------PNQ
XP_002522867.2/1-214 TMS-------------------------------------G-----------Y----SDC
XP_020534605.1/1-220 ANS-------------------------------------D----------------PNR
XP_021685850.1/1-217 ANS-------------------------------------D----------------SSE
XP_021594464.1/1-220 ANS-------------------------------------D----------------SSL
XP_018731177.1/1-223 VEP-------------------------------------G----------------PNS
XP_024018593.1/1-217 VNS-------------------------------------T----------------LDK
XP_022139420.1/1-219 VNS-------------------------------------A----------------PRR
XP_022981426.1/1-220 VNS-------------------------------------S----------------PRK
XP_022981427.1/1-220 VNS-------------------------------------S----------------PRK
XP_022941489.1/1-220 VNS-------------------------------------S----------------PRK
XP_022941488.1/1-220 VNS-------------------------------------S----------------PRK
XP_023525599.1/1-220 VNS-------------------------------------S----------------PRK
XP_023525597.1/1-220 VNS-------------------------------------S----------------PRK
XP_008454681.2/1-220 VNS-------------------------------------A----------------PRR
XP_011648553.1/1-223 VNS-------------------------------------A----------------PRR
XP_018822340.1/1-211 VIS-------------------------------------S----------------P--
XP_018822367.1/1-211 VIS-------------------------------------S----------------P--
XP_023876961.1/1-210 VID-------------------------------------T----------------P--
XP_023876962.1/1-210 VID-------------------------------------T----------------P--
XP_024034242.1/1-215 VNA-------------------------------------T----------------PEE
XP_015381349.2/1-215 VNA-------------------------------------T----------------PEE
XP_022717683.1/1-216 VNS-------------------------------------S----------------PNK
XP_016729588.1/1-216 VNS-------------------------------------S----------------PNK
XP_016729589.1/1-216 VNS-------------------------------------S----------------PNK
XP_016702140.1/1-208 VNS-------------------------------------S----------------PNK
XP_012460158.1/1-216 VNS-------------------------------------S----------------PNK
XP_012460145.1/1-216 VNL-------------------------------------S----------------PNK
XP_017245631.1/1-197 AF-------------------------------------------------------STG
XP_004956045.3/1-247 SNI-------------------------------------T-----------D----GTK
XP_014660176.1/1-240 PNV-------------------------------------T-----------D----GIN
XP_017239609.1/1-213 VPM-------------------------------------T-----------------TS
XP_023740378.1/1-206 ----------------------------------------------------------SE
XP_020095129.1/1-235 TGG-------------------------------------S----------AS----GDE
XP_020258373.1/1-212 QAN-------------------------------------D----------KT-----KH
XP_008812148.1/1-175 --G-------------------------------------P-----------E-----NP
XP_010930674.1/1-230 VRG-------------------------------------P-----------E-----SP
XP_010931877.1/1-230 VRG-------------------------------------P-----------E-----SP
XP_009380061.1/1-225 ------------------------------------GLNY-------------------T
XP_010939971.1/1-229 ------------------------------------ALNP------------------AQ
XP_019710626.1/1-218 ------------------------------------ALNP------------------AQ
XP_008787891.1/1-229 ------------------------------------ALNS------------------AQ
XP_008787892.1/1-218 ------------------------------------ALNS------------------AQ
XP_008799024.1/1-230 ------------------------------------ALNS------------------SP
XP_010936845.1/1-230 ------------------------------------TLNS------------------SQ
XP_003570542.1/1-223 ------------------------------------GLNA------------------SQ
XP_004964922.1/1-229 ------------------------------------GLNA------------------NQ
XP_002438026.1/1-230 ------------------------------------GLNA------------------NQ
XP_020399989.1/1-232 ------------------------------------GLNA------------------NQ
XP_008649207.1/1-222 ------------------------------------GLNA------------------NQ
XP_020256822.1/1-228 ------------------------------------ALTP------------------DQ
XP_009403550.1/1-228 ------------------------------------GLSS------------------SQ
XP_020582158.1/1-232 ------------------------------------ATMNP----------PN----MND
XP_020699118.1/1-240 ------------------------------------ATMNP----------AN----LND
XP_020600375.1/1-232 ------------------------------------AAMNP----------AN----PNE
XP_020600376.1/1-232 ------------------------------------AAMNP----------AN----PNE
XP_020684394.1/1-232 ------------------------------------AAMNP----------SN----PNE
XP_017257147.1/1-219 ------------------------------------AFNTS------------------Q
XP_010244428.1/1-230 ------------------------------------TLNAS------------------Q
XP_010244355.1/1-230 ------------------------------------TLNAS------------------Q
XP_017240757.1/1-236 ------------------------------------AFNTS------------------Q
XP_017245021.1/1-233 ------------------------------------AFNTS------------------Q
XP_012829269.1/1-233 ------------------------------------GFNSS------------------Q
XP_022889938.1/1-236 ------------------------------------ALHNN------------------Q
XP_022897471.1/1-232 ------------------------------------ALNAN------------------Q
XP_009606250.1/1-235 ------------------------------------AYNTS------------------Q
XP_016468230.1/1-235 ------------------------------------AYNTS------------------Q
XP_019250773.1/1-235 ------------------------------------AYNTS------------------Q
XP_009778533.1/1-235 ------------------------------------AYNTS------------------Q
XP_016451582.1/1-235 ------------------------------------AYNTS------------------Q
XP_016578176.1/1-240 ------------------------------------AYNTS------------------Q
XP_006347370.1/1-231 ------------------------------------AYNTS------------------Q
XP_004241474.1/1-230 ------------------------------------AYNTS------------------Q
XP_015077748.1/1-230 ------------------------------------AYNTS------------------Q
XP_022037776.1/1-241 ------------------------------------AYTPS------------------Q
XP_024992062.1/1-237 ------------------------------------AYSPS------------------Q
XP_023772270.1/1-245 ------------------------------------AYTPS------------------Q
XP_012855673.1/1-237 ------------------------------------AYNTS------------------Q
XP_022858328.1/1-236 ------------------------------------ALNTS------------------Q
XP_011072039.1/1-232 ------------------------------------ALNTS------------------Q
XP_006306921.1/1-215 ------------------------------------GFNVS----------S------SQ
NP_564041.2/1-215    ------------------------------------GFNAS----------S------SQ
XP_020866823.1/1-216 ------------------------------------GFNAS----------S------SQ
XP_010476956.1/1-215 ------------------------------------GFNAS----------S-----SAQ
XP_010498167.1/1-217 ------------------------------------GFNAS----------------SAQ
XP_019091528.1/1-217 ------------------------------------GFNTS----------------SAQ
XP_013613976.1/1-227 ------------------------------------GFNAS----------S------SQ
XP_018432674.1/1-227 ------------------------------------GFNAS----------S------SQ
XP_018432667.1/1-227 ------------------------------------GFNAS----------S------SQ
XP_013641731.1/1-225 ------------------------------------GFDAS----------S------SQ
XP_009149243.1/1-227 ------------------------------------GFNAS----------S------SQ
XP_006416656.2/1-224 ------------------------------------GFNAS----------S------SQ
XP_018451633.1/1-224 ------------------------------------GFNAS----------S------PQ
XP_018451632.1/1-225 ------------------------------------GFNAS----------S------SQ
XP_018451634.1/1-195 ------------------------------------GFNAS----------S------SQ
XP_013690958.1/1-228 ------------------------------------GFNAS----------S------SQ
XP_009110376.1/1-227 ------------------------------------GFNAS----------S------SQ
XP_009110377.1/1-227 ------------------------------------GFNAS----------S------SQ
XP_013601964.1/1-227 ------------------------------------GFNAS----------S------SQ
XP_013653874.1/1-227 ------------------------------------GFNAS----------S------SQ
XP_018445004.1/1-237 ------------------------------------ASSAS-----------------QS
XP_018445005.1/1-237 ------------------------------------ASSAS-----------------QS
XP_013648946.1/1-228 ------------------------------------ASSAS-----------------QS
XP_009104812.1/1-228 ------------------------------------ASSAS-----------------QS
XP_013588523.1/1-228 ------------------------------------ASSAS-----------------QS
XP_013699248.1/1-228 ------------------------------------ASSAS-----------------QS
XP_018457296.1/1-228 ------------------------------------ASNAS-----------------QS
XP_009105998.1/1-230 ------------------------------------ASNAS----------Q-----SQS
NP_001302850.1/1-231 ------------------------------------ASNAS----------Q-----SQS
XP_013698656.1/1-231 ------------------------------------ASNAS----------Q-----SQS
XP_013649619.1/1-231 ------------------------------------ASNAS----------Q-----SQS
XP_013592873.1/1-231 ------------------------------------ASNAS----------Q-----SQS
XP_006390565.1/1-233 ------------------------------------ASNAS-----------------QS
XP_006301097.1/1-230 ------------------------------------ASNAP-----------------HS
XP_010428260.1/1-231 ------------------------------------ASNAS-----------------QS
XP_010471396.1/1-230 ------------------------------------ASNAS-----------------QS
XP_010416135.1/1-230 ------------------------------------ASNAS-----------------QS
NP_177479.1/1-227    ------------------------------------GSNAT-----------------HS
XP_020890987.1/1-228 ------------------------------------GSNAT-----------------HS
XP_010537520.1/1-232 ------------------------------------AFNAS------------------Q
XP_010540042.1/1-226 ------------------------------------AFNSS------------------Q
XP_010540046.1/1-196 ------------------------------------AFNSS------------------Q
XP_019432660.1/1-235 ------------------------------------PFNST-----------------MN
XP_019420678.1/1-233 ------------------------------------DFNTT-----------------K-
XP_013452816.1/1-243 ------------------------------------AFNTS-----------------Q-
XP_020215886.1/1-221 ------------------------------------GLQSS-----------------E-
XP_020215888.1/1-221 ------------------------------------GLQSS-----------------E-
XP_007160572.1/1-230 ------------------------------------AFNTS-----------------Q-
XP_017424661.1/1-212 ------------------------------------AFNTS-----------------Q-
XP_017424660.1/1-230 ------------------------------------AFNTS-----------------Q-
XP_014508329.1/1-230 ------------------------------------AFNTS-----------------Q-
XP_022632295.1/1-230 ------------------------------------AFNTS-----------------Q-
XP_019225984.1/1-234 ------------------------------------AYNTS------------------Q
XP_016441514.1/1-233 ------------------------------------AYNTS------------------Q
XP_009587298.1/1-233 ------------------------------------AYNTS------------------Q
XP_016441515.1/1-233 ------------------------------------AYNTS------------------Q
XP_009786509.1/1-233 ------------------------------------AYNTS------------------Q
XP_016563952.1/1-234 ------------------------------------AYNTS------------------Q
XP_004235382.1/1-234 ------------------------------------AYNTS------------------Q
XP_015068940.1/1-234 ------------------------------------AYNTS------------------Q
XP_006364383.1/1-234 ------------------------------------AYNTS------------------Q
XP_019167409.1/1-245 ------------------------------------AYNTS------------------Q
XP_011086973.1/1-238 ------------------------------------AFNTS------------------Q
XP_010268321.1/1-230 ------------------------------------AFNAS------------------Q
XP_010268322.1/1-230 ------------------------------------AFNAS------------------Q
XP_010069492.1/1-236 ------------------------------------AFNTS------------------Q
XP_002527933.1/1-229 ------------------------------------AFNSG------------------Q
XP_021678368.1/1-229 ------------------------------------AFNSG------------------Q
XP_021678369.1/1-229 ------------------------------------AFNSG------------------Q
XP_021602738.1/1-229 ------------------------------------AFNSG------------------Q
XP_012079215.1/1-230 ------------------------------------AFNTG------------------Q
XP_021687701.1/1-229 ------------------------------------AFNTG------------------Q
XP_006374212.1/1-231 ------------------------------------AYNTS------------------Q
XP_011035552.1/1-231 ------------------------------------AYNTS------------------Q
XP_002317722.3/1-231 ------------------------------------AYNTS------------------Q
XP_011000766.1/1-231 ------------------------------------AYNTS------------------Q
XP_021907827.1/1-230 ------------------------------------AFNTS------------------Q
XP_017635401.1/1-230 ------------------------------------AFNTS------------------H
XP_016686699.1/1-230 ------------------------------------AFNTS------------------H
XP_012440361.1/1-230 ------------------------------------AFNTS------------------H
NP_001313868.1/1-230 ------------------------------------AFNTS------------------H
XP_023919022.1/1-230 ------------------------------------ALNNG------------------Q
XP_006478112.1/1-232 ------------------------------------AINTS------------------Q
XP_024042646.1/1-232 ------------------------------------AINTS------------------Q
XP_022715403.1/1-231 ------------------------------------AYNTS------------------Q
XP_022767009.1/1-255 VR--------LLKLLKHFPLFYFNALSNC----FLNAFNTS------------------Q
XP_022737050.1/1-231 ------------------------------------AFNTS------------------Q
XP_017973835.1/1-231 ------------------------------------AFNTS------------------Q
XP_015967008.1/1-238 ------------------------------------AYNTS------------------Q
XP_025701434.1/1-239 ------------------------------------AYNTS------------------Q
XP_020980110.1/1-239 ------------------------------------AYNTS------------------Q
XP_019434881.1/1-231 ------------------------------------AFNTT------------------Q
XP_019458275.1/1-234 ------------------------------------AFSNT------------------Q
XP_019458276.1/1-234 ------------------------------------AFSNT------------------Q
XP_006584922.1/1-234 ------------------------------------AFNNS------------------T
XP_003524332.1/1-231 ------------------------------------AFNNS-------------------
XP_004515249.1/1-229 ------------------------------------AFNTS------------------Q
XP_019431381.1/1-229 ------------------------------------AFNTS------------------Q
XP_019436771.1/1-231 ------------------------------------AFNTS------------------Q
XP_019430342.1/1-233 ------------------------------------AFNTS------------------Q
XP_006587436.1/1-240 ------------------------------------AFNSS------------------Q
XP_003549189.1/1-236 ------------------------------------AFNSS------------------Q
XP_020221986.1/1-232 ------------------------------------AFNTS------------------Q
XP_007152598.1/1-234 ------------------------------------AFNTS------------------Q
XP_017437542.1/1-234 ------------------------------------AFNTS------------------Q
XP_014505752.1/1-234 ------------------------------------AFNTS------------------Q
XP_015881991.1/1-234 ------------------------------------AFNTS------------------Q
XP_004299483.1/1-232 ------------------------------------AFNTS------------------Q
XP_024174647.1/1-232 ------------------------------------AFNTS------------------Q
XP_008375041.1/1-227 ------------------------------------AFNTS------------------Q
XP_017188172.1/1-199 ------------------------------------AFNTS------------------Q
XP_009355964.1/1-227 ------------------------------------AFNTS------------------Q
XP_009355965.1/1-199 ------------------------------------AFNTS------------------Q
XP_023523434.1/1-228 ------------------------------------AMNST------------------Q
XP_022949138.1/1-227 ------------------------------------ALNST------------------Q
XP_022998050.1/1-227 ------------------------------------ALNST------------------Q
XP_002271012.1/1-231 ------------------------------------AFNTS------------------Q
XP_010663934.1/1-231 ------------------------------------AFNTS------------------Q
XP_010099679.1/1-237 ------------------------------------AFNTS------------------Q
XP_018823334.1/1-236 ------------------------------------AFNTS------------------Q
XP_023001145.1/1-228 ------------------------------------AFNTS------------------Q
XP_023519889.1/1-228 ------------------------------------AFNTS------------------Q
XP_022924191.1/1-228 ------------------------------------AFNTS------------------Q
XP_018849090.1/1-235 ------------------------------------AFNTS------------------Q
XP_008240378.1/1-228 ------------------------------------AFNTS------------------Q
XP_007210460.2/1-228 ------------------------------------AFNTS------------------Q
XP_021801797.1/1-228 ------------------------------------AFNTS------------------Q
XP_022136988.1/1-228 ------------------------------------AFNTS------------------Q
XP_016898817.1/1-228 ------------------------------------AFNTS------------------Q
XP_004137568.1/1-228 ------------------------------------GFNTS------------------Q
XP_015642098.1/1-238 ------------------------------------GLNAS------------------Q
XP_020186741.1/1-220 ------------------------------------GLNAS------------------Q
XP_025828617.1/1-229 ------------------------------------GLNAS------------------Q
XP_004954072.1/1-226 ------------------------------------GLNAS------------------Q
XP_002452898.1/1-231 ------------------------------------GLNAS------------------Q
NP_001142912.2/1-230 ------------------------------------GLNAS------------------Q
XP_020089796.1/1-231 ------------------------------------GLNSS------------------Q
XP_020089795.1/1-241 NF----------------TLF------GN----QTQGLNSS------------------Q
XP_019708739.1/1-228 ------------------------------------ALNAS------------------Q
XP_008789232.1/1-228 ------------------------------------ALNAS------------------Q
XP_008789231.1/1-228 ------------------------------------ALNAS------------------Q
XP_008789233.1/1-200 ------------------------------------ALNAS------------------Q
XP_019704584.1/1-228 ------------------------------------ALNAS------------------Q
XP_010917295.1/1-228 ------------------------------------ALNAS------------------Q
XP_008806630.1/1-228 ------------------------------------ALNAS------------------Q
XP_009388746.1/1-226 ------------------------------------GLNSG------------------Q
XP_018675467.1/1-228 ------------------------------------GLNSG------------------Q
XP_018675466.1/1-228 ------------------------------------GLNSG------------------Q
XP_009409108.1/1-228 ------------------------------------GLNSG------------------Q
XP_024625408.1/1-220 ------------------------------------GFSTG------------------N
XP_010550261.1/1-219 -----------------------------------RPFIPT------------------E
XP_018816719.1/1-228 -----------------------------------QPFIPA------------------E
XP_008365756.2/1-218 -----------------------------------RPFTPT------------------E
XP_008381522.1/1-218 -----------------------------------RPFTPT------------------E
XP_018506060.1/1-218 -----------------------------------RPFIPT------------------E
XP_008362571.1/1-218 -----------------------------------RPFIPX------------------E
XP_023870350.1/1-229 -----------------------------------RPYIPT------------------E
XP_016649213.1/1-222 -----------------------------------RPFIPT------------------E
XP_020415996.1/1-222 -----------------------------------RPFIPT------------------E
XP_021823813.1/1-222 -----------------------------------RPFIPT------------------E
XP_009367126.1/1-222 -----------------------------------RPFIPT------------------E
XP_008348615.1/1-222 -----------------------------------RPFIPT------------------E
XP_017179578.1/1-222 -----------------------------------RPFTPT------------------E
XP_009370612.1/1-224 -----------------------------------RPFTPT------------------E
XP_012845089.1/1-228 ------------------------------------PFVPK------------------E
XP_012845088.1/1-228 ------------------------------------PFVPK------------------E
XP_012845087.1/1-228 ------------------------------------PFVPK------------------E
XP_011085930.1/1-227 ------------------------------------PFVPK------------------E
XP_020551334.1/1-222 ------------------------------------PFVPK------------------E
XP_021297998.1/1-224 ------------------------------------PSVPS------------------G
XP_017974480.1/1-224 ------------------------------------PSVPS------------------G
XP_024448785.1/1-229 -----------------------------------HPFIPT------------------E
XP_024455385.1/1-229 -----------------------------------HPFIPT------------------E
XP_011005937.1/1-269 CSINYSQKAYSNSVLIHFQQVYLCNSLIVFPFKLKQPFIPT------------------E
XP_011038413.1/1-229 -----------------------------------RPFIPA------------------E
XP_018731153.1/1-226 ------------------------------------VISLH------------------A
XP_024190515.1/1-228 ------------------------------------PFVPT------------------E
XP_012074722.2/1-231 ------------------------------------PFASN------------------D
XP_010089514.2/1-229 ------------------------------------PFIPT------------------E
XP_024949330.1/1-235 ------------------------------------PFIPT------------------E
XP_006421085.1/1-235 ------------------------------------PFIPT------------------E
XP_002268272.1/1-229 ------------------------------------PFVPT------------------E
XP_004140733.2/1-228 ------------------------------------PFVPT------------------E
XP_008457114.1/1-228 ------------------------------------PFIPT------------------E
XP_008457113.1/1-229 -----------------------------------QPFIPT------------------E
XP_011651208.1/1-229 -----------------------------------QPFVPT------------------E
XP_022143367.1/1-228 -----------------------------------RPFVPT------------------E
XP_022935490.1/1-228 -----------------------------------RPFVPT------------------E
XP_022984101.1/1-228 -----------------------------------RPFVPT------------------E
XP_022935488.1/1-229 -----------------------------------QPFVPT------------------E
XP_022984098.1/1-229 -----------------------------------QPFVPT------------------E
XP_022984102.1/1-229 -----------------------------------QPFVPT------------------E
XP_015579295.1/1-234 ------------------------------------PFVPS------------------E
XP_002526595.2/1-234 ------------------------------------PFVPS------------------E
XP_015579294.1/1-234 ------------------------------------PFVPS------------------E
XP_021643520.1/1-232 ------------------------------------PFIPS------------------E
XP_021605433.1/1-231 ------------------------------------PFAPS------------------E
XP_023739098.1/1-228 ------------------------------------VTNTR------------------D
XP_024966239.1/1-227 ------------------------------------TQNTS------------------Q
XP_025813558.1/1-232 ------------------------------------GTMTD----------------ANQ
XP_020150479.1/1-234 ------------------------------------ALSPSQ--------APQ----GGQ
XP_003564122.2/1-224 ------------------------------------GVNAS------------------H
XP_006585049.2/1-225 ------------------------------------ALGP------------------SD
XP_007143783.1/1-226 ------------------------------------PFIP------------------KV
XP_007158859.1/1-226 ------------------------------------PFIP------------------KE
XP_020230815.1/1-227 ------------------------------------PVHP------------------TM
XP_006586851.1/1-227 ------------------------------------PIHP------------------TA
XP_021888058.1/1-234 ------------------------------------PLNS------------------DE
XP_020886111.1/1-226 --------------------------------------PTQEE-NNDKMVAQD----SSK
XP_020886110.1/1-228 ------------------------------------VHPTQEE-NNDKMVAQD----SSK
XP_011469296.1/1-224 ------------------------------------PFIP------------------TE
XP_009367128.2/1-218 ------------------------------------PFTP------------------TE
XP_008348673.1/1-198 ------------------------------------PFTP------------------TE
XP_012832506.1/1-227 ------------------------------------PFVP------------------KE
XP_012832508.1/1-227 ------------------------------------PFVP------------------KE
XP_012832516.1/1-227 ------------------------------------PFLP------------------KE
XP_012832518.1/1-227 ------------------------------------PFLP------------------KE
XP_012835569.1/1-227 ------------------------------------PFVP------------------KE
XP_012835571.1/1-226 ------------------------------------PFLP------------------KE
XP_019172066.1/1-226 ------------------------------------PYVS------------------KE
XP_009631364.2/1-226 ------------------------------------PYVP------------------KE
XP_016435660.1/1-229 ------------------------------------PYVP------------------KE
XP_009771801.1/1-226 ------------------------------------PYVS------------------KE
XP_016493115.1/1-226 ------------------------------------PYVS------------------KE
XP_019248352.1/1-226 ------------------------------------PYVP------------------KE
XP_025691463.1/1-225 ------------------------------------PYIP------------------TE
XP_020995109.1/1-225 ------------------------------------PYIP------------------TE
XP_016190415.2/1-225 ------------------------------------PYIP------------------TE
XP_004486568.1/1-232 ------------------------------------PFIP------------------SE
XP_024632687.1/1-226 ------------------------------------PFIP------------------TQ
XP_020977993.1/1-241 ------------------------------------PFMS------------------SE
XP_025647253.1/1-241 ------------------------------------PFMS------------------SE
XP_020997095.1/1-240 ------------------------------------PFMS------------------SE
XP_025692389.1/1-228 ------------------------------------PFMS------------------SE
XP_019418389.1/1-226 ------------------------------------PFVP------------------TE
XP_007147460.1/1-227 ------------------------------------PFIP------------------SE
XP_014517364.1/1-227 ------------------------------------PFIP------------------SE
XP_017435438.1/1-226 ------------------------------------PFIP------------------SE
XP_017435439.1/1-217 ------------------------------------PFIP------------------SE
XP_025981664.1/1-227 ------------------------------------PFIP------------------SE
XP_006586853.1/1-227 ------------------------------------PFIP------------------NE
XP_020230831.1/1-226 ------------------------------------PFIP------------------SE
XP_004489663.1/1-217 ------------------------------------PFIPSE------------------
XP_018489833.1/1-227 ------------------------------------HTLAGDI---GKNME-Q----EPD
XP_013584487.1/1-219 ------------------------------------PTPPWDI---GQNMV-Q----EPH
XP_009130351.1/1-219 ------------------------------------PTPLWDI---GQNMV-Q----EPH
XP_013747906.2/1-219 ------------------------------------PTPLWDI---GQNMV-Q----EPQ
XP_013747661.1/1-219 ------------------------------------PTPLWDI---GQNMV-Q----EPQ
XP_013619678.1/1-219 ------------------------------------PTPPWDI---GQNMV-Q----EPH
XP_022551084.1/1-219 ------------------------------------PTPPWDI---GQNMV-Q----EPH
XP_009126392.1/1-219 ------------------------------------PTPPWDI---GQNMV-Q----EPH
XP_018437612.1/1-222 ------------------------------------PTQRHED---V--M-AQ----GPK
XP_013591191.1/1-225 ------------------------------------PTQREDD---VISM-SQ----GPK
XP_022567005.1/1-225 ------------------------------------PTQREDD---VISM-SQ----GPK
XP_022547796.1/1-225 ------------------------------------PTQREDD---VISM-SQ----GPT
XP_009119205.1/1-225 ------------------------------------PTQREDD---VISM-SQ----GPT
XP_009119199.1/1-225 ------------------------------------PTQREDD---VISM-SQ----GPT
XP_006408322.1/1-232 ------------------------------------PPRRE---DIAISMAQD----SDN
XP_023642295.1/1-229 ------------------------------------VHPMQEESDDKSMMERD----PEE
XP_023642294.1/1-229 ------------------------------------VHPMQEESDDKSMMERD----PEE
NP_186976.2/1-228    ------------------------------------VHPTHEEN-NDKMVVQD----SCK
XP_010507998.1/1-229 ------------------------------------VHPTQEE-NNGKLVAED----SDK
XP_010485661.1/1-229 ------------------------------------VHPTQEE-NNGKMMAQD----SDK
XP_010450732.1/1-230 ------------------------------------VHPTQEENNNGKMIAQE----SDK
XP_010463776.1/1-230 ------------------------------------VHPTQEENNNGKMIAQE----SDK
XP_010463774.1/1-230 ------------------------------------VHPTQEENNNGKMIAQE----SDK
XP_019092960.1/1-229 ------------------------------------VHPTQEEN-NGKLMAQD----SDK
XP_022551077.1/1-228 ------------------------------------TTSTTED-----------------
XP_013619236.1/1-228 ------------------------------------TTSTTED-----------------
XP_009126177.1/1-226 ------------------------------------TASTAED-----------------
XP_013652421.1/1-226 ------------------------------------TASTAED-----------------
XP_013652420.1/1-226 ------------------------------------TASTAED-----------------
XP_006400265.1/1-228 ------------------------------------PTTATEN-----------------
XP_013590801.1/1-226 ------------------------------------PSSAADS-----------------
XP_013651910.1/1-222 ------------------------------------PSSAAEG-----------------
XP_009103958.1/1-222 ------------------------------------PSSAAEG-----------------
XP_018444485.1/1-226 ------------------------------------PSSETQN-----------------
XP_009121397.1/1-226 ------------------------------------PSSATEN-----------------
XP_013659428.1/1-226 ------------------------------------PSSATEN-----------------
XP_022565333.1/1-226 ------------------------------------PSSATEN-----------------
XP_013610327.1/1-226 ------------------------------------PSSATEN-----------------
XP_006286655.1/1-225 ------------------------------------PSSGGEK----------------K
XP_010492677.1/1-224 ------------------------------------ASSGGDN----------------N
XP_010420445.1/1-224 ------------------------------------TSSGGDN----------------N
XP_010453918.1/1-224 ------------------------------------ASSGGDN----------------N
XP_006303739.1/1-224 ------------------------------------TSSEGDN-----------------
XP_020876074.1/1-222 ------------------------------------PSIVGDS-----------------
NP_197234.1/1-222    ------------------------------------PSIRDIN-----------------
NP_174724.1/1-221    ------------------------------------PSSVGE------------------
XP_020869876.1/1-222 ------------------------------------PSSEGDN-----------------
XP_019091098.1/1-222 ------------------------------------PSSGGEN-----------------
XP_010478910.1/1-223 ------------------------------------PSSGGDN-----------------
XP_010501781.1/1-221 ------------------------------------PSSGGDN-----------------
XP_015089790.1/1-222 ------------------------------------SHMLKE------------------
XP_015089784.1/1-222 ------------------------------------SYMLKE------------------
XP_016549855.1/1-224 ------------------------------------PYVPTE------------------
XP_015057403.1/1-221 ------------------------------------SY-L-Q------------------
XP_019066399.1/1-221 ------------------------------------SY-L-Q------------------
XP_015166059.1/1-216 ------------------------------------PC-M-Q------------------
XP_019071012.2/1-216 ------------------------------------PC-M-Q------------------
XP_015086709.1/1-216 ------------------------------------PC-M-Q------------------
XP_015087432.1/1-219 ------------------------------------PYNM-H------------------
XP_004247342.2/1-219 ------------------------------------PYNM-H------------------
XP_025888717.1/1-219 ------------------------------------PYNM-H------------------
XP_015087599.1/1-221 ------------------------------------PYNM-Y------------------
XP_019071156.1/1-221 ------------------------------------PYNM-Y------------------
XP_019070762.2/1-220 ------------------------------------PYNM-H------------------
XP_015087071.1/1-220 ------------------------------------PYNM-H------------------
XP_025888845.1/1-217 ------------------------------------PYNM-H------------------
XP_015170367.1/1-232 ------------------------------------PYVSKE------------------
XP_025886268.1/1-232 ------------------------------------PYVPEE------------------
XP_015072491.1/1-224 ------------------------------------PYVPEE------------------
XP_010327374.2/1-219 ------------------------------------LNTTRE------------------
XP_015089749.1/1-219 ------------------------------------LNMTTE------------------
XP_015170971.1/1-209 ------------------------------------PYMSIE------------------
XP_025887726.1/1-223 ------------------------------------CYMPTE------------------
XP_006586358.1/1-227 ------------------------------------PFIPKE------------------
XP_014617351.1/1-228 ------------------------------------PIHPTA------------------
XP_007147461.1/1-227 ------------------------------------PVHRNA------------------
XP_017435408.1/1-226 ------------------------------------PLHRTA------------------
XP_017215828.1/1-223 -----------------------------------------------MKQN--------R
XP_012856110.1/1-229 -----------------------------------------------MKGE--------E
XP_023738891.1/1-226 -----------------------------------------------MKSK--------E
XP_022017964.1/1-227 -----------------------------------------------MKSK--------D
XP_022154996.1/1-226 -----------------------------------------------IKSD--------E
XP_008462169.1/1-225 -----------------------------------------------IKSD--------E
XP_004141841.1/1-226 -----------------------------------------------VKSD--------E
XP_020098514.1/1-228 -------------------------------------------------KMKS----AEK
XP_020098513.1/1-228 -------------------------------------------------KMKS----AEK
XP_020263950.1/1-229 -------------------------------------------------ATKS----TER
XP_020263948.1/1-255 GCD-----------------------INIFNHKLPDTTT-KAYYDVNMQATKS----TER
XP_009415234.2/1-232 -----------------------------------------------LQTTTS----SER
XP_008789333.1/1-230 -------------------------------------------------ATKS----ADK
XP_010920420.1/1-235 -------------------------------------------------ATKS----ADR
XP_010920411.1/1-235 -------------------------------------------------ATKS----ADR
XP_010920404.1/1-235 -------------------------------------------------ATKS----ADR
XP_010521515.1/1-219 -----------------------------------------------VKTRE--------
XP_013652056.1/1-227 -----------------------------------------------VKSRE--------
XP_009104428.1/1-227 -----------------------------------------------VKSRE--------
XP_018445476.1/1-226 -----------------------------------------------VKSRE--------
XP_013590784.1/1-226 -----------------------------------------------VKSRE--------
XP_013680822.1/1-226 -----------------------------------------------VKSRE--------
XP_006389720.2/1-226 -----------------------------------------------VKSRE--------
XP_023642973.1/1-226 -----------------------------------------------VKSRE--------
XP_020891640.1/1-226 -----------------------------------------------VKSRE--------
NP_001320267.1/1-226 -----------------------------------------------VKSRE--------
NP_565223.1/1-226    -----------------------------------------------VKSRE--------
NP_001185443.1/1-226 -----------------------------------------------VKSRE--------
XP_010473021.1/1-226 -----------------------------------------------VKTRE--------
XP_010430051.1/1-226 -----------------------------------------------VKSRE--------
XP_025617667.1/1-241 -----------------------------------------------ITSKEN-------
XP_015938429.1/1-242 -----------------------------------------------ITSKEN-------
XP_016176999.1/1-241 -----------------------------------------------ITSKEN-------
XP_021862846.1/1-225 -----------------------------------------------MKSKD--------
XP_022979290.1/1-231 -----------------------------------------------IGSKES------S
XP_022955725.1/1-233 -----------------------------------------------IGSKES----NSS
XP_022998610.1/1-231 -----------------------------------------------RGSKES----S-S
XP_022998611.1/1-227 -----------------------------------------------RGSKES----S-S
XP_023523626.1/1-229 -----------------------------------------------RGSKES-------
XP_022948296.1/1-231 -----------------------------------------------RGSKES-------
XP_023523628.1/1-225 -----------------------------------------------RGSKES-------
XP_022948295.1/1-227 -----------------------------------------------RGSKES-------
XP_010249693.1/1-228 ------------------------------------------------QAMKS----K-E
XP_010254081.1/1-230 ------------------------------------------------QAMKS----K-E
XP_003529763.1/1-227 ------------------------------------------------KIIQS----K-D
XP_003533048.1/1-226 ------------------------------------------------KI-QS----K-D
XP_014634625.1/1-226 ------------------------------------------------KI-QS----K-D
XP_007150635.1/1-226 ------------------------------------------------T-IKL----K-E
XP_014498860.1/1-226 ------------------------------------------------T-IKL----K-E
XP_017425661.1/1-226 ------------------------------------------------T-IKL----K-E
XP_020207725.1/1-226 ------------------------------------------------T-IKS----N-E
XP_003546423.2/1-226 ------------------------------------------------T-IKS----K-E
XP_003543638.1/1-226 ------------------------------------------------T-IKL----K-E
XP_019457980.1/1-226 ------------------------------------------------T-VKS----K-E
XP_003597736.1/1-226 ------------------------------------------------T-IKS----K-E
XP_004486751.1/1-226 ------------------------------------------------T-VKS----N-E
XP_016169615.1/1-226 ------------------------------------------------T-IKS----K-E
XP_025611006.1/1-226 ------------------------------------------------T-IKS----K-E
XP_020982478.1/1-227 ------------------------------------------------T-IKS----K-E
XP_021892835.1/1-225 ------------------------------------------------QNIKS----K-E
XP_012442963.1/1-227 ------------------------------------------------QAMKS----K-E
NP_001314068.1/1-227 ------------------------------------------------QAMKS----K-E
XP_016684379.1/1-227 ------------------------------------------------QAMKS----K-E
XP_016730082.1/1-227 ------------------------------------------------QAMKS----K-E
NP_001316938.1/1-227 ------------------------------------------------QAMKS----K-E
XP_017606636.1/1-227 ------------------------------------------------QAMKS----K-E
XP_022714992.1/1-229 ------------------------------------------------QTMKS----K-E
XP_022721436.1/1-227 ------------------------------------------------QTMKS----K-E
XP_022721435.1/1-227 ------------------------------------------------QTMKS----K-E
XP_007024189.1/1-227 ------------------------------------------------QAMKS----K-E
XP_021298731.1/1-227 ------------------------------------------------QAMKS----K-E
XP_004302894.1/1-228 ------------------------------------------------QTMKS----K-E
XP_011466687.1/1-235 CIS-----------------------I--------------------FQTMKS----K-E
XP_024180978.1/1-228 ------------------------------------------------QTMKS----K-E
XP_024180977.1/1-235 CIS-----------------------I--------------------FQTMKS----K-E
XP_009353605.1/1-226 ------------------------------------------------QSMKS----K-E
XP_008380909.1/1-226 ------------------------------------------------QSMKS----K-E
NP_001280927.1/1-227 ------------------------------------------------QSMKS----K-E
XP_009338062.1/1-226 ------------------------------------------------QSMKS----K-E
XP_009338046.1/1-226 ------------------------------------------------QSMKS----K-E
XP_021829136.1/1-226 ------------------------------------------------QTMKS----K-E
XP_008228560.1/1-226 ------------------------------------------------QTMKS----K-E
XP_007217160.1/1-226 ------------------------------------------------QTMKS----K-E
XP_022891611.1/1-228 ------------------------------------------------QAMKT----K-D
XP_002304207.2/1-228 ------------------------------------------------LKMKS----K-E
XP_011010522.1/1-228 ------------------------------------------------LKMKS----K-E
XP_011009828.1/1-227 ------------------------------------------------LKMKS----K-E
XP_024462179.1/1-228 ------------------------------------------------LKMKS----K-E
XP_018837483.1/1-226 ------------------------------------------------QAMKS----K-E
XP_018856342.1/1-226 ------------------------------------------------QTMKS----K-E
XP_018856994.1/1-226 ------------------------------------------------QTMKS----K-E
XP_018858360.1/1-226 ------------------------------------------------QTMKS----K-E
XP_015867693.1/1-226 ------------------------------------------------QTTKS----N-E
XP_010062490.1/1-226 ------------------------------------------------QTMKN----K-D
XP_010098082.1/1-228 ------------------------------------------------QTMKS----K-E
XP_002284502.1/1-228 ------------------------------------------------QTMKS----K-D
XP_023875609.1/1-226 ------------------------------------------------QTMKS----K-E
XP_002516023.1/1-226 ------------------------------------------------QTMKS----N-E
XP_021652755.1/1-228 ------------------------------------------------QTMKS----K-E
XP_021654904.1/1-226 ------------------------------------------------QAMKS----K-G
XP_021611342.1/1-229 ------------------------------------------------QTMKP----K-E
XP_012073376.1/1-226 ------------------------------------------------QTMKS----K-E
XP_006465622.1/1-226 ------------------------------------------------QTMKS----K-E
XP_006426950.1/1-226 ------------------------------------------------QTMKS----K-E
XP_009392419.1/1-229 ------------------------------------------------HTTTS----SER
XP_020518333.1/1-227 ------------------------------------------------HATQT----K-E
XP_018629167.1/1-227 ------------------------------------------------QAMKL----K-E
XP_009611250.1/1-227 ------------------------------------------------QAMKL----K-E
XP_009611249.1/1-227 ------------------------------------------------QAMKL----K-E
XP_009611248.1/1-227 ------------------------------------------------QAMKL----K-E
XP_016479156.1/1-227 ------------------------------------------------QAMKL----K-E
XP_016479155.1/1-227 ------------------------------------------------QAMKL----K-E
XP_016479154.1/1-227 ------------------------------------------------QAMKL----K-E
XP_009778213.1/1-229 ------------------------------------------------QAVKL----R-E
XP_016501674.1/1-229 ------------------------------------------------QAVKL----R-E
XP_019224885.1/1-229 ------------------------------------------------QAVKL----R-E
XP_019224886.1/1-229 ------------------------------------------------QAVKL----R-E
XP_009592772.1/1-229 ------------------------------------------------QAVKL----R-E
XP_016496403.1/1-229 ------------------------------------------------QAVKL----R-E
XP_016562690.1/1-228 ------------------------------------------------QAVKL----R-E
XP_006343080.1/1-229 ------------------------------------------------QAVKL----R-E
XP_004235676.1/1-229 ------------------------------------------------QAVKL----R-E
XP_020227921.1/1-228 ------------------------------------------------QKIQS----K-G
XP_007135673.1/1-226 ------------------------------------------------QKIES----K-E
XP_014515030.1/1-226 ------------------------------------------------QKIES----K-E
XP_017407449.1/1-226 ------------------------------------------------HKIES----T-E
XP_010691342.1/1-224 ------------------------------------------------QTMKS----K-D
XP_021731998.1/1-222 ------------------------------------------------QTMKS----K-E
XP_021736870.1/1-224 ------------------------------------------------QTMKS----K-E
XP_024969955.1/1-229 ------------------------------------------------HTMKS----K-E
XP_024969954.1/1-229 ------------------------------------------------HTMKS----K-E
XP_024969953.1/1-229 ------------------------------------------------HTMKS----K-E
XP_021984354.1/1-227 ------------------------------------------------HTMKW----K-E
XP_017232370.1/1-228 ------------------------------------------------QAMKH----K-E
XP_017216573.1/1-228 ------------------------------------------------QAIKQ----K-Q
XP_017248777.1/1-227 ------------------------------------------------QEIKP----K-E
XP_021627148.1/1-226 ------------------------------------------------QTMNS----K-D
XP_021627146.1/1-226 ------------------------------------------------QTMNS----K-D
XP_021627145.1/1-226 ------------------------------------------------QTMNS----K-D
XP_019196965.1/1-228 ------------------------------------------------LATKS----K-E
XP_019196967.1/1-228 ------------------------------------------------LATKS----K-E
XP_022848789.1/1-228 ------------------------------------------------QAMKT----K-D
XP_011072373.1/1-228 ------------------------------------------------LAMTT----D-E
XP_021907286.1/1-215 VNA-----------------------G-------------------------D----GSL
XP_020885309.1/1-217 --K-----------------------V-------------------------N----TCQ
XP_020885307.1/1-217 --K-----------------------V-------------------------N----TCQ
NP_180796.2/1-218    --H-----------------------G-------------------------H----TSK
XP_006296541.1/1-217 --N-----------------------A-------------------------N----TFQ
XP_010510028.1/1-215 --N-----------------------A-------------------------I----PCE
XP_010414006.1/1-214 --N-----------------------E-------------------------I----PCE
XP_010469602.1/1-219 --N-----------------------E-------------------------I----PFE
XP_010417818.1/1-218 --N-----------------------K-------------------------S----TWQ
XP_010510869.1/1-220 LQN-----------------------K-------------------------S----TCQ
XP_010510870.1/1-218 --N-----------------------K-------------------------S----TCQ
XP_018484769.1/1-219 --T-----------------------Q-------------------------N----TSQ
XP_018484754.1/1-219 --T-----------------------Q-------------------------N----TSQ
XP_013634882.1/1-219 --T-----------------------P-------------------------N----TSQ
XP_018513778.1/1-219 --T-----------------------P-------------------------N----TSQ
XP_009141217.1/1-219 --T-----------------------P-------------------------N----TSQ
XP_013747219.1/1-219 --T-----------------------P-------------------------S----TSQ
XP_024003849.1/1-217 --G-----------------------P-------------------------K----TIE
XP_018437853.1/1-211 --S-----------------------P-------------------------N----TRQ
XP_009143975.1/1-211 --S-----------------------P-------------------------N----TSQ
XP_009143974.1/1-211 --S-----------------------P-------------------------N----TSQ
XP_022575029.1/1-211 --S-----------------------P-------------------------N----TSQ
XP_013748684.1/1-211 --S-----------------------P-------------------------N----TSQ
XP_013694194.1/1-211 --S-----------------------P-------------------------N----TSQ
XP_013634261.1/1-211 --S-----------------------P-------------------------N----TSQ
XP_013741430.1/1-210 --S-----------------------P-------------------------N----TSQ
XP_017254373.1/1-221 VYD-----------------------T-------------------------E----CH-
XP_017254240.1/1-222 LHN-------------------------------------------------S----RE-
XP_017221582.1/1-224 LHN-------------------------------------------------P----GK-
XP_017221584.1/1-224 LHN-------------------------------------------------P----GK-
XP_015941575.1/1-215 IKS-----------------------P-------------------------D----AKN
XP_025620358.1/1-215 IKS-----------------------P-------------------------D----AKN
XP_016176493.1/1-215 IKS-----------------------S-------------------------D----AKN
XP_003610661.2/1-228 ITS-----------------------E-------------------------N----SEQ
XP_004496853.1/1-220 VKS-----------------------A-------------------------D----CSQ
XP_006353342.1/1-230 TSV-----------------------A-------------NS----------D----GSQ
XP_015070591.1/1-229 TTV-----------------------A-------------NS----------D----GSQ
XP_019068356.1/1-229 TTV-----------------------A-------------NS----------D----GSQ
XP_004234441.1/1-229 TTV-----------------------A-------------NS----------D----GSQ
XP_024926606.1/1-217 VNA-----------------------S------------------------------QNR
XP_024525400.1/1-231 IDA-----------------------L-------------NQ--------TLN----ANQ
XP_002965186.2/1-231 IDA-----------------------L-------------NQ--------TLN----ANQ
XP_002962272.2/1-231 IDA-----------------------L-------------NQ--------TLN----ANQ
XP_019441536.1/1-226 VNQ-----------------------L-------------------------Q----NEN
XP_019441537.1/1-226 VNQ-----------------------L-------------------------Q----NEN
XP_019441535.1/1-226 VNQ-----------------------L-------------------------Q----NEN
XP_019451758.1/1-229 VNQ-----------------------S-------------------------Q----NAN
XP_012850835.1/1-238 DMS-----------------------S-------------------------R------E
XP_020552079.1/1-225 AKS-----------------------A-------------------------T------S
XP_020552078.1/1-225 AKS-----------------------A-------------------------T------S
XP_020552080.1/1-223 S-------------------------A-------------------------T------S
XP_009408807.2/1-226 VGL-----------------------P-------------------------E----EET
XP_006342404.1/1-225 VNS-----------------------P-------------------------N----PAQ
XP_025887998.1/1-218 VNC-----------------------P-------------------------N----QAP
XP_015081481.1/1-225 VNC-----------------------P-------------------------N----QAQ
XP_022000230.1/1-232 ANG-----------------------A-------------GG---------NN----PGQ
XP_022000229.1/1-232 ANG-----------------------A-------------GG---------NN----PGQ
XP_022037790.1/1-222 ANA-----------------------T-------------GP----------N----ANQ
XP_022037789.1/1-222 ANA-----------------------T-------------GP----------N----ANQ
XP_024991925.1/1-226 ANT-----------------------T-------------GA----------N----ANQ
XP_023760005.1/1-229 ANT-----------------------S-------------GG----------N----ANQ
XP_023760001.1/1-229 ANT-----------------------S-------------GG----------N----ANQ
XP_017251723.1/1-236 PTG-----------------------I-------------TD----------D----MNQ
XP_017243321.1/1-239 AKA-----------------------M-------------GD----------N----SNQ
XP_017243319.1/1-239 AKA-----------------------M-------------GD----------N----SNQ
XP_017243320.1/1-239 AKA-----------------------M-------------GD----------N----SNQ
XP_017255563.1/1-234 ANA-----------------------S-------------GG----------N----SNQ
XP_017255562.1/1-234 ANA-----------------------S-------------GG----------N----SNQ
XP_012852237.1/1-226 STT-----------------------M------------------------------PAG
XP_012852238.1/1-226 STT-----------------------M------------------------------PAG
XP_012852236.1/1-226 STT-----------------------M------------------------------PAG
XP_012852235.1/1-226 STT-----------------------M------------------------------PAG
XP_011076208.1/1-227 TSA-----------------------P-------------AS----------S----ANQ
XP_019200484.1/1-228 NTA-------------------------------------SA----------S----VNQ
XP_019200481.1/1-228 NTA-------------------------------------SA----------S----VNQ
XP_022879034.1/1-223 NSA-------------------------------------AA----------S----ANQ
XP_018623115.1/1-238 ANG-----------------------N-------------AI----------S----DSE
XP_018623116.1/1-238 ANG-----------------------N-------------AI----------S----DSE
XP_016449095.1/1-238 ANG-----------------------N-------------AI----------S----DSE
XP_009771835.1/1-238 ANV-----------------------D-------------AI----------S----DSE
XP_009771833.1/1-238 ANV-----------------------D-------------AI----------S----DSE
XP_019248454.1/1-225 ANG-----------------------D-------------AI----------S----DSE
XP_016564677.1/1-235 TNV-----------------------T-------------GS----------D----GNQ
XP_016564676.1/1-235 TNV-----------------------T-------------GS----------D----GNQ
XP_009606534.1/1-237 ANA-----------------------A-------------SR----------D----ANQ
XP_016457239.1/1-237 ANA-----------------------A-------------SR----------D----ANQ
XP_009606533.1/1-237 ANA-----------------------A-------------SR----------D----ANQ
XP_009606532.1/1-237 ANA-----------------------A-------------SR----------D----ANQ
XP_016457238.1/1-237 ANA-----------------------A-------------SR----------D----ANQ
XP_016468064.1/1-237 ANA-----------------------A-------------AS----------D----ANQ
XP_009774404.1/1-237 ANA-----------------------A-------------AS----------D----ANQ
XP_016468062.1/1-237 ANA-----------------------A-------------AS----------D----ANQ
XP_016468061.1/1-237 ANA-----------------------A-------------AS----------D----ANQ
XP_009774402.1/1-237 ANA-----------------------A-------------AS----------D----ANQ
XP_009774401.1/1-237 ANA-----------------------A-------------AS----------D----ANQ
XP_019251817.1/1-237 ANA-----------------------A-------------AS----------D----ANQ
XP_019251815.1/1-237 ANA-----------------------A-------------AS----------D----ANQ
XP_019251816.1/1-237 ANA-----------------------A-------------AS----------D----ANQ
XP_011470395.1/1-233 AKG-----------------------L------------------------DA----SNN
XP_004309219.2/1-233 AKG-----------------------L------------------------DA----SNN
XP_024180881.1/1-233 AKG-----------------------L------------------------DA----NNN
XP_024180911.1/1-233 AKG-----------------------L------------------------DA----NNN
XP_019450525.1/1-216 SG-------------------------------------------------AN----ASE
XP_019465255.1/1-230 NG-------------------------------------------------PK----GLE
XP_014502466.1/1-227 VSA-----------------------F-------------NK---------YA----NNE
XP_017438850.1/1-222 ---------------------------------------------------YA----NNE
XP_006599623.1/1-221 ---------------------------------------------------SA----NSE
XP_006599622.1/1-221 ---------------------------------------------------SA----NSE
XP_006599621.1/1-221 ---------------------------------------------------SA----NSE
XP_004515730.1/1-240 GNN-----------------------A------------------------SN----GNE
XP_013442370.1/1-239 LNN-----------------------A-------------VN---------PN----GNE
XP_004252035.1/1-225 MIG-----------------------E-------------ND----------------NQ
XP_015060726.1/1-225 MIG-----------------------E-------------ND----------------NQ
XP_006589608.1/1-229 VNT-----------------------Q-------------NV----------------AQ
XP_006589605.1/1-230 VNT-----------------------Q-------------NV----------------AQ
XP_006849468.1/1-231 AFN-----------------------S-------------------------------SQ
XP_020526173.1/1-231 AFN-----------------------S-------------------------------SQ
XP_020109515.1/1-228 GLN-----------------------S-------------------------------SQ
NP_001310555.1/1-224 GLN-----------------------A-------------------------------SQ
XP_010544375.1/1-225 SNA-----------------------M-------------------------N----ASQ
NP_001314001.1/1-230 GAA-----------------------V-------------------------N----GSD
XP_016732454.1/1-230 GAA-----------------------V-------------------------N----GSD
XP_016732453.1/1-230 GAA-----------------------V-------------------------N----GSD
XP_012484067.1/1-230 GAA-----------------------V-------------------------N----GSD
XP_012484066.1/1-230 GAA-----------------------V-------------------------N----GSD
XP_017631791.1/1-230 GAA-----------------------V-------------------------N----GSD
XP_017631789.1/1-230 GAA-----------------------V-------------------------N----GSD
XP_016728129.1/1-230 GAA-----------------------V-------------------------N----GSD
XP_016728128.1/1-230 GAA-----------------------V-------------------------N----GSD
XP_021671684.1/1-240 SSA-----------------------V-------------CP----------N----ANE
XP_021671683.1/1-240 SSA-----------------------V-------------CP----------N----ANE
XP_021671685.1/1-240 SSA-----------------------V-------------CP----------N----ANE
XP_002318231.3/1-240 STA-----------------------V-------------DA----------D----AGE
XP_024438602.1/1-240 STA-----------------------V-------------DA----------D----AGE
XP_024442055.1/1-239 STA-----------------------V-------------DA----------N----AS-
XP_002322460.1/1-239 STA-----------------------V-------------DA----------N----AS-
XP_011048682.1/1-239 STA-----------------------V-------------DA----------N----AS-
XP_011048684.1/1-239 STA-----------------------V-------------DA----------N----AS-
XP_011048683.1/1-239 STA-----------------------V-------------DA----------N----AS-
XP_019081404.1/1-234 PNA-----------------------R-------------------------N----QND
XP_003634081.1/1-234 PNA-----------------------R-------------------------N----QND
XP_008377249.1/1-231 ANE-----------------------T-------------------------N----AND
XP_017189459.1/1-231 ANE-----------------------T-------------------------N----AND
XP_008377244.1/1-231 ANE-----------------------T-------------------------N----AND
XP_009371302.1/1-231 ANA-----------------------P-------------------------N----ANN
XP_009371299.1/1-231 ANA-----------------------P-------------------------N----ANN
XP_008376545.1/1-231 ANA-----------------------P-------------------------D----ANN
XP_008376544.1/1-231 ANA-----------------------P-------------------------D----ANN
XP_017188609.1/1-231 ANA-----------------------P-------------------------D----ANN
XP_022945426.1/1-238 ANP-----------------------M-------------------------N----ANE
XP_022945425.1/1-238 ANP-----------------------M-------------------------N----ANE
XP_023541229.1/1-238 ANP-----------------------M-------------------------N----ANE
XP_023541228.1/1-238 ANP-----------------------M-------------------------N----ANE
XP_022968414.1/1-238 ANP-----------------------M-------------------------N----ANE
XP_022968413.1/1-238 ANP-----------------------M-------------------------N----ANE
XP_023552381.1/1-232 ANP-----------------------M-------------------------N----ANE
XP_022922479.1/1-232 ANP-----------------------M-------------------------N----ANE
XP_023552380.1/1-232 ANP-----------------------M-------------------------N----ANE
XP_022922478.1/1-232 ANP-----------------------M-------------------------N----ANE
XP_022984644.1/1-232 ANP-----------------------M-------------------------K----ANE
XP_022984643.1/1-232 ANP-----------------------M-------------------------K----ANE
XP_008439185.1/1-242 ANP-----------------------M-------------------------N----ANE
XP_008439184.1/1-242 ANP-----------------------M-------------------------N----ANE
XP_011651140.1/1-242 ANP-----------------------M-------------------------N----ANE
XP_004140784.1/1-242 ANP-----------------------M-------------------------N----ANE
XP_009391954.1/1-234 ATA-----------------------T-------------NA----------------SQ
XP_003581542.2/1-227 TNT-----------------------T-------------NT----------------QQ
XP_006652658.1/1-224 ASP-----------------------T-------------NA----------------NQ
XP_015637147.1/1-225 ASP-----------------------T-------------KD----------------KQ
XP_025823430.1/1-225 ADP-----------------------T-------------NT----------------HL
XP_004978185.1/1-225 ADH-----------------------T-------------NT----------------HL
XP_021317935.1/1-228 ADH-----------------------T-------------NS----------------HL
NP_001336434.1/1-226 ADH-----------------------T-------------DS----------------HL
XP_019709552.1/1-234 PSV-----------------------V-------------GA----------------SE
XP_015696802.1/1-257 PNA-----------------------T-------------SG----------------NQ
XP_015612621.1/1-258 PNA-----------------------T-------------SG----------------NQ
XP_004957413.1/1-253 PNA-----------------------T-------------SG----------------NQ
XP_025801167.1/1-251 PNA-----------------------T-------------SG----------------NQ
NP_001105125.2/1-256 PNA-----------------------T-------------SG----------------NQ
XP_002462701.1/1-258 PNA-----------------------T-------------SG----------------NQ
XP_020262629.1/1-222 ASA-----------------------A-------------TG----------G----TQG
XP_020699782.1/1-235 ATV-----------------------P-------------ATSSSMDANGNQK----HED
XP_020699776.1/1-235 ATV-----------------------P-------------ATSSSMDANGNQK----HED
XP_020573166.1/1-235 ATV-----------------------P-------------AAASAMDANGNQK----QEE
XP_020187432.1/1-228 ATA-------------------------------------PN----------T----HQD
XP_025656795.1/1-233 ANA-----------------------N-------------NG----------G----ESS
XP_025656792.1/1-233 ANA-----------------------N-------------NG----------G----ESS
XP_025656794.1/1-233 ANA-----------------------N-------------NG----------G----ESS
XP_020979736.1/1-233 ANA-----------------------N-------------NG----------G----ESS
XP_020999151.1/1-233 ANA-----------------------N-------------NG----------G----ESS
XP_015967451.1/1-233 ANA-----------------------N-------------NG----------G----ESS
XP_020999150.1/1-233 ANA-----------------------N-------------NG----------G----ESS
XP_025701970.1/1-233 ANA-----------------------N-------------NG----------G----ESS
XP_025701968.1/1-233 ANA-----------------------N-------------NG----------G----ESS
XP_025701969.1/1-233 ANA-----------------------N-------------NG----------G----ESS
XP_020221720.1/1-222 ANS-------------------------------------------------------TD
XP_007152327.1/1-222 YAN-------------------------------------------------------NE
XP_006587345.1/1-222 SAN-------------------------------------------------------SE
XP_006587344.1/1-222 SAN-------------------------------------------------------SE
XP_010035104.1/1-239 AN--------------------------------------AL----------S----ASD
XP_021765162.1/1-234 PP--------------------------------------AA----------P----ARE
XP_021765160.1/1-234 PP--------------------------------------AA----------P----ARE
XP_021730123.1/1-234 PP--------------------------------------AA----------P----ARE
XP_021842103.1/1-232 PP--------------------------------------AA----------P----ARE
XP_021842106.1/1-232 PP--------------------------------------AA----------P----ARE
XP_021842105.1/1-232 PP--------------------------------------AA----------P----ARE
XP_021842104.1/1-232 PP--------------------------------------AA----------P----ARE
XP_010693471.1/1-235 PP--------------------------------------AA----------P----ARE
XP_019107938.1/1-235 PP--------------------------------------AA----------P----ARE
XP_010693469.1/1-235 PP--------------------------------------AA----------P----ARE
XP_019107937.1/1-235 PP--------------------------------------AA----------P----ARE
XP_020163116.1/1-258 PN--------------------------------------AT----------S----GNQ
XP_020163109.1/1-258 PN--------------------------------------AT----------S----GNQ
XP_003578475.1/1-267 PN--------------------------------------AT----------S----GNQ
XP_011012369.1/1-242 STA-----------------------V-------------DA----------D----AVE
XP_011012370.1/1-242 STA-----------------------V-------------DA----------D----AVE
XP_008234278.1/1-230 -A------------------------N-------------VV----------D----ANE
XP_021827117.1/1-230 -A------------------------N-------------VI----------D----ANE
XP_021827116.1/1-230 -A------------------------N-------------VI----------D----ANE
XP_020413581.1/1-230 -A------------------------N-------------VI----------D----ANE
XP_007219555.2/1-230 -A------------------------N-------------VI----------D----ANE
XP_018456926.1/1-230 -P------------------------A-------------GK----------H----QSG
XP_013613235.1/1-226 -P------------------------A-------------GK----------H----QNE
XP_013686466.1/1-227 -H------------------------A-------------GK----------H----QNE
XP_009106897.1/1-227 -H------------------------A-------------GK----------H----QNE
XP_006401857.1/1-229 -P------------------------A-------------GK----------D----QNE
XP_020871109.1/1-230 -A------------------------A-------------GK----------D----QNE
NP_001332093.1/1-229 -A------------------------A-------------GN----------D----QNE
NP_200030.1/1-229    -A------------------------A-------------GN----------D----QNE
XP_006282099.1/1-225 -A------------------------A-------------GK----------D----QNE
XP_019095386.1/1-229 -A------------------------A-------------GK----------D----QNE
XP_019095387.1/1-229 -A------------------------A-------------GK----------D----QNE
XP_019091174.1/1-228 -A------------------------A-------------GK----------D----QNE
XP_019091179.1/1-228 -A------------------------A-------------GK----------D----QNE
XP_019087173.1/1-228 -A------------------------A-------------GK----------D----QNE
XP_010555654.1/1-234 -T------------------------S-------------GI----------D----QAE
XP_010555656.1/1-234 -T------------------------S-------------GI----------D----QAE
XP_010555655.1/1-234 -T------------------------S-------------GI----------D----QAE
XP_010537199.1/1-242 AS------------------------Q-------------GI----------D----QTE
XP_010537197.1/1-242 AS------------------------Q-------------GI----------D----QTE
XP_010537198.1/1-241 A-------------------------S-------------GI----------D----QTE
Con_90%              ...........................................................t
Con_80%              ...........................................................p
Con_70%              hp........................................................sp
Con_60%              hp......................................t.................sp
Con_50%              lps.....................................t...........s....ssp


XP_020582785.1/1-284 -DMY-LL------QLCEGIDENSV-GACAQLIFSPADQSFV---------------D---
XP_020682623.1/1-284 -DIY-LL------QLCEGIDENAV-GACAQLIFSPTDPSFA---------------D---
XP_020682624.1/1-284 -DIY-LL------QLCEGIDENAV-GACAQLIFSPTDPSFA---------------D---
XP_006846015.1/1-286 -DMF-LL------QLCSGVDENAV-GACAQLVFAPIDESFA---------------D---
NP_001142394.1/1-289 -DMF-LL------QLCSGVDEDAP-GACAQLVFAPIDESFA---------------D---
NP_001146215.1/1-288 -DMF-LL------QLCSGIDEDAP-GACAQLVFAPIDESFA---------------D---
XP_020405718.1/1-288 -DMF-LL------QLCSGIDEDAP-GACAQLVFAPIDESFA---------------D---
XP_002443548.1/1-287 -DMF-LL------QLCSGVDENAP-GACAQLVFAPIDESFA---------------D---
XP_025808876.1/1-287 -DMF-LL------QLCSGVDESAP-GACAQLVFAPIDESFA---------------D---
XP_004963232.1/1-287 -DMF-LL------QLCTGVDESAP-GACAQLVFAPIDESFA---------------D---
XP_020192001.1/1-285 -DMY-LL------QLCSGVDENAA-GACAQLVFAPIDESFA---------------D---
XP_003562005.1/1-285 -DMY-LL------QLCSGVDENAA-GACAQLVFAPIDESFA---------------D---
XP_004982339.1/1-286 -DMY-LL------QLCSGVDENAA-GACAQLVFAPIDESFA---------------D---
XP_025796542.1/1-286 -DMY-LL------QLCSGVDENAA-GACAQLVFAPIDESFA---------------D---
NP_001347456.1/1-286 -DMY-LL------QLCSGVDENAA-GACAQLVFAPIDESFA---------------D---
NP_001352464.1/1-286 -DMY-LL------QLCSGVDENAA-GACAQLVFAPIDESFA---------------D---
XP_002464180.1/1-286 -DMY-LL------QLCSGVDENAA-GACAQLVFAPIDESFA---------------D---
XP_015689987.1/1-286 -DMY-LL------QLCSGVDENAA-GACAQLVFAPIDESFA---------------D---
XP_015632484.1/1-286 -DMY-LL------QLCSGVDENAA-GACAQLVFAPIDESFA---------------D---
XP_015620816.1/1-285 -DMY-LL------QLCSGVDENAT-SASAQLVFAPIDESFA---------------D---
XP_006664738.1/1-285 -DMY-LL------QLCSGVDENAT-SASAQLVFAPIDESFA---------------D---
XP_020190954.1/1-286 -DMY-LL------QLCSGVDENAS-GACAQLVFAPIDESFA---------------D---
XP_020163543.1/1-286 -DMY-LL------QLCSGVDENAS-GACAQLVFAPIDESFA---------------D---
XP_003577453.1/1-286 -DMY-LL------QLCSGVDENAA-GACAQLVFAPIDESFA---------------D---
XP_020575104.1/1-285 -DMY-LL------QLCSGIDENAV-GACAQLVFAPIDESFA---------------D---
XP_020696902.1/1-286 -DMY-LL------QLCSGIDENAV-GACAQLVFAPIDESFA---------------D---
XP_020578108.1/1-284 -DMY-LL------QLCSGVDENAV-GACSQLVFAPIDESFA---------------D---
XP_020698923.1/1-284 -DMY-LL------QLCSGVDENAV-GACSQLVFAPIDESFA---------------D---
XP_020096610.1/1-279 -DMY-LL------QLCSGVDE--I-GACAQLVFAPIDESFA---------------D---
XP_009412508.1/1-287 -NIH-LL------QLCRGVDENAA-GACAQLVFAPIDESFA---------------D---
XP_020271156.1/1-284 -DMF-LL------QLCSGVDDNSV-GACAQLVFAPIDESFA---------------D---
XP_020273236.1/1-287 -DMF-LL------QLCSGVDENSV-GACAQLVFAPIDESFA---------------D---
XP_020092733.1/1-287 -DMY-LL------QVCSGVDENAV-GACAQLVFAPIDESFA---------------D---
XP_019701934.1/1-288 -DMY-LL------QLCSGVDENAV-GACAQLVFAPIDESFA---------------D---
XP_010905421.1/1-288 -DMY-LL------QLCSGVDENAV-GACAQLVFAPIDESFA---------------D---
XP_010905420.1/1-289 -DMY-LL------QLCSGVDENAV-GACAQLVFAPIDESFA---------------D---
XP_010923678.1/1-288 -DMY-LL------QLCSGVDENAV-GACAQLVFAPIDESFA---------------D---
XP_008794452.1/1-288 -DMY-LL------QLCSGVDENAV-GACAQLVFAPIDESFA---------------D---
XP_009397391.1/1-288 -DMY-LL------QLCSGVDENAI-GSCAQLVFAPIDESFA---------------D---
XP_009408551.1/1-288 -DVY-LL------QLCSGVDEDAV-GACAQLVFAPIDESFA---------------D---
XP_009399045.1/1-288 -DMY-FL------QLCSGVDENAV-GACAQLVFAPIDESFA---------------D---
XP_009388964.1/1-288 -DMY-ML------QLCSGVDESAV-GACAQLVFAPIDESFD---------------D---
XP_009397717.1/1-288 -DMY-LL------QLCSGVEENAV-GACAQLVFAPIDESFD---------------D---
XP_018488876.1/1-280 -DMY-LL------QLCSGVDENVV-GGCAQLVFAPIDESFA---------------D---
XP_013587486.1/1-280 -DMY-LL------QLCSGVDENVV-GGCAQLVFAPIDESFA---------------D---
XP_009115139.1/1-280 -DMY-LL------QLCSGVDENVV-GGCAQLVFAPIDESFA---------------D---
XP_013662459.1/1-280 -DMY-LL------QLCSGVDENVV-GGCAQLVFAPIDESFA---------------D---
XP_013707293.1/1-280 -DMY-LL------QLCSGVDENVV-GGCAQLVFAPIDESFA---------------D---
XP_006415465.2/1-280 -DMY-LL------QLCSGVDENVV-GGCAQLVFAPIDESFA---------------D---
XP_019099288.1/1-280 -DMY-LL------QLCSGVDENVV-GGCAQLVFAPIDESFA---------------D---
NP_174337.1/1-280    -DMY-LL------QLCSGVDENVV-GGCAQLVFAPIDESFA---------------D---
XP_020867051.1/1-280 -DMY-LL------QLCSGVDENVV-GGCAQLVFAPIDESFA---------------D---
XP_020867052.1/1-280 -DMY-LL------QLCSGVDENVV-GGCAQLVFAPIDESFA---------------D---
XP_010460847.1/1-280 -DMY-LL------QLCSGVDENVV-GGCAQLVFAPIDESFA---------------D---
XP_019094482.1/1-280 -DMY-LL------QLCSGVDENVV-GGCAQLVFAPIDESFA---------------D---
XP_006306764.1/1-280 -DMY-LL------QLCSGVDENVV-GGCAQLVFAPIDESFA---------------D---
XP_010538268.1/1-285 -DMY-LL------QLCSGTDETAV-GGCAQLIFAPIDESFA---------------D---
XP_010519070.1/1-284 -DMY-LL------QLCSGVEETAV-GGCAQLVFAPIDESFA---------------D---
XP_018436356.1/1-280 -DMY-LL------QLCSGVDENVV-GGCAQLVFAPIDESFA---------------D---
XP_013637594.1/1-286 -DMY-LL------QLCSGVDENVV-GGCAQLVFAPIDESFA---------------D---
XP_013692700.1/1-286 -DMY-LL------QLCSGVDENVV-GGCAQLVFAPIDESFA---------------D---
XP_009143747.1/1-286 -DMY-LL------QLCSGVDENVV-GGCAQLVFAPIDESFA---------------D---
XP_018484893.1/1-285 -DMY-LL------QLCSGVDEDVV-GGCAQLVFAPIDESFA---------------D---
XP_013637264.1/1-280 -DMY-LL------QLCSGVDEDVV-GGCAQLVFAPIDESFA---------------D---
XP_013637263.1/1-280 -DMY-LL------QLCSGVDEDVV-GGCAQLVFAPIDESFA---------------D---
XP_022557131.1/1-280 -DMY-LL------QLCSGVDEDVV-GGCAQLVFAPIDESFA---------------D---
XP_013729051.1/1-280 -DMY-LL------QLCSGVDEDVV-GGCAQLVFAPIDESFA---------------D---
XP_013744548.1/1-280 -DMY-LL------QLCSGVDEDVV-GGCAQLVFAPIDESFA---------------D---
XP_009141396.1/1-280 -DMY-LL------QLCSGVDEDVV-GGCAQLVFAPIDESFA---------------D---
XP_006410621.1/1-284 -DMY-LL------QLCSGVDENVV-GGCAQLVFAPIDESFA---------------D---
NP_181018.1/1-284    -DMY-LL------QLCSGVDENVV-GGCAQLVFAPIDESFA---------------D---
XP_020884308.1/1-284 -DMY-LL------QLCSGVDENVV-GGCAQLVFAPIDESFA---------------D---
XP_006293359.1/1-284 -DMY-LL------QLCSGVDENVV-GGCAQLVFAPIDESFA---------------D---
XP_010516626.1/1-284 -DMY-LL------QLCSGVDENVV-GGCAQLVFAPIDESFA---------------D---
XP_010509720.1/1-284 -DMY-LL------QLCSGVDENVV-GGCAQLVFAPIDESFA---------------D---
XP_010504942.1/1-284 -DMY-LL------QLCSGVDENVV-GGCAQLVFAPIDESFA---------------D---
XP_017223336.1/1-283 -NMY-LL------QLCSGIDEKTA-GGCAQLVFAPIDESFA---------------D---
XP_016559371.1/1-285 -DMY-LL------QLCSGIEENAT-GACAQLVFAHIDESFG---------------D---
XP_016559374.1/1-285 -DMY-LL------QLCSGIEENAT-GACAQLVFAHIDESFG---------------D---
XP_012830207.1/1-281 -DMY-LL------QLCSGIDEANT-GECAQLIFAPIDESFG---------------D---
XP_012827547.1/1-287 -DMY-LL------QLCSGIDETTSGATCAQLVFAPIDESFG---------------D---
XP_022866806.1/1-284 -DMY-LL------QLCSGVDETS--GSCSQLVFAPIDESFG---------------D---
XP_022866805.1/1-285 -DMY-LL------QLCSGVDETS--GSCSQLVFAPIDESFG---------------D---
XP_023001017.1/1-285 -DMY-LL------QLCSGVDESTV-GSCAQLVFAPIDESFA---------------D---
XP_023001016.1/1-285 -DMY-LL------QLCSGVDESTV-GSCAQLVFAPIDESFA---------------D---
XP_023519837.1/1-285 -DMY-LL------QLCSGVDESTV-GSCAQLVFAPIDESFA---------------D---
XP_023519836.1/1-285 -DMY-LL------QLCSGVDESTV-GSCAQLVFAPIDESFA---------------D---
XP_022927139.1/1-285 -DMY-LL------QLCSGVDESTV-GSCAQLVFAPIDESFA---------------D---
XP_022927138.1/1-285 -DMY-LL------QLCSGVDESTV-GSCAQLVFAPIDESFA---------------D---
XP_022139982.1/1-285 -DMY-LL------QLCSGIDENAV-GSCAQLVFAPIDESFA---------------D---
XP_004135550.1/1-285 -DMY-LL------QLCSGVDENTV-GACAQLVFAPIDESFA---------------D---
XP_008445207.1/1-285 -DMY-LL------QLCSGVEENTV-GACAQLVFAPIDESFA---------------D---
XP_017230337.1/1-286 -DMY-LL------QLCSGLDENAA-GGCAQLVFAPIDETCA---------------D---
XP_017230338.1/1-286 -DMY-LL------QLCSGLDENAA-GGCAQLVFAPIDETCA---------------D---
XP_010316959.1/1-285 -DMY-LL------QLCSGVEENAT-GACAQLVFAHIDESFG---------------D---
XP_004233170.1/1-285 -DMY-LL------QLCSGVEENAT-GACAQLVFAHIDESFG---------------D---
XP_015066839.1/1-285 -DMY-LL------QLCSGVEENAT-GACAQLVFAHIDESFG---------------D---
XP_015066842.1/1-285 -DMY-LL------QLCSGVEENAT-GACAQLVFAHIDESFG---------------D---
XP_015066843.1/1-285 -DMY-LL------QLCSGVEENAT-GACAQLVFAHIDESFG---------------D---
XP_006353018.1/1-285 -DMY-LL------QLCSGVEENAT-GACAQLVFAHIDESFG---------------D---
XP_006353020.1/1-285 -DMY-LL------QLCSGVEENAT-GACAQLVFAHIDESFG---------------D---
XP_019178964.1/1-287 -DMY-LL------QLCSGIDENAA-GACAQLVFAPIDESFG---------------D---
XP_019178963.1/1-287 -DMY-LL------QLCSGIDENAA-GACAQLVFAPIDESFG---------------D---
XP_019228489.1/1-285 -DMY-LL------QLCSGVDENAS-GACAQLVFAPIDESFG---------------D---
XP_019228488.1/1-285 -DMY-LL------QLCSGVDENAS-GACAQLVFAPIDESFG---------------D---
NP_001289498.1/1-285 -DMY-LL------QLCSGVDENAS-GACAQLVFAPIDESFG---------------D---
XP_009777911.1/1-285 -DMY-LL------QLCSGVDENAS-GACAQLVFAPIDESFG---------------D---
XP_009612342.1/1-285 -DMY-LL------QLCSGVDENAS-GACAQLVFAPIDESFG---------------D---
XP_009612341.1/1-285 -DMY-LL------QLCSGVDENAS-GACAQLVFAPIDESFG---------------D---
XP_006363649.1/1-287 -DMY-LL------QLCSGVDENAA-GACAQLVFAPIDESFG---------------D---
XP_015066689.1/1-287 -DMY-LL------QLCSGVDENAA-GACAQLVFAPIDESFG---------------D---
XP_010315636.1/1-287 -DMY-LL------QLCSGVDENAA-GACAQLVFAPIDESFG---------------D---
XP_022879368.1/1-285 -DMY-LL------QLCSGVDESTS-GACAQLVFAPIDESFG---------------D---
XP_020554026.1/1-285 -DMY-LL------QLCSGIDETTS-GACAQLVFAPIDESFG---------------D---
XP_011096678.1/1-285 -DMY-LL------QLCSGIDETTS-GACAQLVFAPIDESFG---------------D---
XP_011085207.1/1-285 -DMY-LL------QLCSGVDETNT-GACAQLVFAPIDESFG---------------D---
XP_023003193.1/1-285 -DMY-LL------QLCNGVDENAV-GACAQLVFAPIDESFA---------------D---
XP_022926723.1/1-285 -DMY-LL------QLCNGVDENAV-GACAQLVFAPIDESFA---------------D---
XP_023518237.1/1-285 -DMY-LL------QLCNGVDENAV-GACAQLVFAPIDESFA---------------D---
XP_022146537.1/1-286 -DMY-LL------QLCSGVDENAV-GACAQLVFAPIDESFA---------------D---
XP_011658319.1/1-286 -DMY-LL------QLCSGVDENAV-GACAQLVFAPIDESFA---------------D---
XP_008439554.1/1-286 -DMY-LL------QLCSGVDENAV-GACAQLVFAPIDESFA---------------D---
XP_022978372.1/1-286 -DMY-LL------QLCSGVDENAV-GACAQLVFAPIDESFA---------------D---
XP_023543833.1/1-286 -DMY-LL------QLCSGVDENAV-GACAQLVFAPIDESFA---------------D---
XP_022926042.1/1-286 -DMY-LL------QLCSGVDENAV-GACAQLVFAPIDESFA---------------D---
XP_010247499.1/1-286 -DMY-LL------QLCSGVDENAA-GACAQLVFAPIDESFA---------------D---
XP_016717556.1/1-285 -DMY-LL------QLCSGIDENAV-GACAQLVFAPIDESFA---------------D---
XP_012455108.1/1-285 -DMY-LL------QLCSGIDENAV-GACAQLVFAPIDESFA---------------D---
XP_016717554.1/1-287 -DMY-LL------QLCSGIDENAV-GACAQLVFAPIDESFA---------------D---
XP_012455107.1/1-287 -DMY-LL------QLCSGIDENAV-GACAQLVFAPIDESFA---------------D---
XP_016711412.1/1-287 -DMY-LL------QLCSGIDENEV-GACAQLVFAPIDESFA---------------D---
XP_017649973.1/1-287 -DMY-LL------QLCSGIDENEV-GACAQLVFAPIDESFA---------------D---
XP_022716449.1/1-285 -DMY-LL------QLCSGIDENAV-DACAQLVFAPIDESFA---------------D---
XP_022724664.1/1-287 -DMY-LL------QLCSGIDENAV-GACAQLVFAPIDESFA---------------D---
XP_021298192.1/1-285 -DMY-LL------QLCSGIDENAV-GTCAQLVFAPIDESFA---------------D---
XP_021298193.1/1-294 -DMY-LL------QLCSGIDENAV-GTCAQLVFAPIDESFA---------------D---
XP_021298191.1/1-294 -DMY-LL------QLCSGIDENAV-GTCAQLVFAPIDESFA---------------D---
XP_007022874.2/1-285 -DMY-LL------QLCSGIDENAV-GACAQLVFAPIDESFA---------------D---
XP_017979205.1/1-294 -DMY-LL------QLCSGIDENAV-GACAQLVFAPIDESFA---------------D---
XP_017979204.1/1-294 -DMY-LL------QLCSGIDENAV-GACAQLVFAPIDESFA---------------D---
XP_017236680.1/1-286 -DMY-LL------QLCSGLDENAA-GACAQLVFAPIDESCA---------------D---
XP_010051959.1/1-285 -DMY-LL------QLCSGVDENAV-GACAQLVFAPIDESFA---------------D---
XP_010051960.1/1-285 -DMY-LL------QLCSGVDENAV-GACAQLVFAPIDESFA---------------D---
XP_015882975.1/1-284 -DMY-IL------QLCSGIDENAV-GACAQLVFAPIDESFA---------------D---
XP_015882797.1/1-284 -DMY-IL------QLCSGIDENAV-GACAQLVFAPIDESFA---------------D---
XP_010098570.1/1-284 -DMY-LL------QLCSGVDESAV-GACAQLVFAPIDESFA---------------D---
XP_011012084.1/1-284 -DMY-LL------QLCSGVDENAL-GACAQLIFAPIDESFA---------------D---
XP_011012083.1/1-284 -DMY-LL------QLCSGVDENAL-GACAQLIFAPIDESFA---------------D---
XP_006377667.2/1-284 -DMY-LL------QLCSGVDENAL-GACAQLVFAPIDESFA---------------D---
XP_002281868.2/1-285 -DMY-LL------QLCSGVDENAA-GACAQLVFAPIDESFA---------------D---
XP_018847863.1/1-285 -DMY-LL------QLCSGVDENAV-GASAQLVFAPIDESFA---------------D---
XP_018847862.1/1-285 -DMY-LL------QLCSGVDENAV-GASAQLVFAPIDESFA---------------D---
XP_023919942.1/1-285 -DMY-LL------QLCSGVDENAV-GACAQLVFAPIDESFA---------------D---
XP_023919941.1/1-285 -DMY-LL------QLCSGVDENAV-GACAQLVFAPIDESFA---------------D---
XP_018828101.1/1-285 -DMY-LL------QLCSGVDENAV-GACAQLVFAPIDESFA---------------D---
XP_018828100.1/1-285 -DMY-LL------QLCSGVDENAV-GACAQLVFAPIDESFA---------------D---
XP_002298892.1/1-285 -DMY-LL------QLCSGVDENAV-GACAQLVFAPIDESFA---------------D---
XP_011038887.1/1-285 -DMY-LL------QLCSGVDENAV-GACAQLVFAPIDESFA---------------D---
XP_011038886.1/1-285 -DMY-LL------QLCSGVDENAV-GACAQLVFAPIDESFA---------------D---
XP_021657817.1/1-285 -DMY-LL------QLCGGVDEKAV-GACAQLVFAPIDESFA---------------D---
XP_021657818.1/1-285 -DMY-LL------QLCGGVDEKAV-GACAQLVFAPIDESFA---------------D---
XP_015577248.1/1-260 -DMY-LL------QLCSGVDENAV-GACAQLVFAPIDESFA---------------D---
XP_021616343.1/1-285 -DMY-LL------QLCSGVDETAI-GACAQLVFAPIDESFA---------------D---
XP_021645103.1/1-285 -DMY-LL------QLCSGVDENAV-GACAQLVFAPIDESFA---------------D---
XP_021592762.1/1-285 -DMY-LL------QLCSGVDENAI-GACAQLVFAPIDESFA---------------D---
XP_012075627.1/1-285 -DMY-LL------QLCSGVDENAV-GACAQLVFAPIDESFA---------------D---
XP_008372224.1/1-285 -DMY-LL------QLCSGVDENAV-GACAQLVFAPIDESFA---------------D---
XP_009362238.1/1-285 -DMY-LL------ELCSGVDENAV-GACAQLVFAPIDESFA---------------D---
XP_009344405.1/1-284 -DMY-LL------QLCSGVDENAV-GACAQLVFAPIDESFA---------------D---
XP_008349150.1/1-284 -DMY-LL------QLCSGVDENAV-GACAQLVFAPIDESFA---------------D---
XP_007214622.1/1-285 -DMY-LL------QLCSGVDENAV-GSCAQLVFAPIDESFA---------------D---
XP_021812393.1/1-285 -DMY-LL------QLCSGVDENAV-GSCAQLVFAPIDESFA---------------D---
XP_024161164.1/1-285 -DMF-LL------QLCSGVDENAV-GACAQLVFAPIDESFA---------------D---
XP_004293989.1/1-285 -DMF-LL------QMCSGVDENAV-GSCAQLVFAPIDESFA---------------D---
XP_019419795.1/1-282 -DMY-LL------QLCSGIDENSI-GACAQLVFAPIDESFA---------------D---
XP_019426301.1/1-282 -DMY-LL------QLCSGVDENSI-GSCAQLVFAPIDESFA---------------D---
XP_003524993.1/1-285 -DMY-LM------QLCSGIDENAI-GACAQLVFAPIDESFA---------------D---
XP_003532788.1/1-285 -DMY-LM------QLCSGIDENAI-GACAQLVFAPIDESFA---------------D---
XP_020215009.1/1-285 -DMY-LL------QLCSGVDENAI-GACAQLVFAPIDESFA---------------D---
XP_017409410.1/1-285 -DMY-LL------QLCSGVDENAI-GACAQLVFAPIDESFA---------------D---
XP_017409418.1/1-285 -DMY-LL------QLCSGVDENAI-GACAQLVFAPIDESFA---------------D---
XP_014509941.1/1-285 -DMY-LL------QLCSGVDENAI-GACAQLVFAPIDESFA---------------D---
XP_007159548.1/1-285 -DMY-LL------QLCSGVDENAI-GACAQLVFAPIDESFA---------------D---
XP_024047818.1/1-288 -DMY-LL------QLCSGIDENTV-GACAQLVFAPIDESFA---------------D---
XP_006448915.2/1-288 -DMY-LL------QLCSGIDENTV-GACAQLVFAPIDESFA---------------D---
XP_006468302.1/1-288 -DMY-LL------QLCSGIDENTV-GACAQLVFAPIDESFA---------------D---
XP_006468303.1/1-287 -DMY-LL------QLCSGIDENTV-GACAQLVFAPIDESFA---------------D---
XP_024047817.1/1-287 -DMY-LL------QLCSGIDENTV-GACAQLVFAPIDESFA---------------D---
XP_004504217.1/1-285 -DMY-LL------QLCSGVDENSV-GACAQLVFAPIDESFA---------------D---
XP_003594520.2/1-281 -DMY-LL------QLCSGIDENSV-GACAQLVFAPIDESFA---------------D---
XP_024632601.1/1-281 -DMY-LL------QLCSGIDENSV-GACAQLVFAPIDESFA---------------D---
XP_004486450.1/1-285 -DMY-LL------QLCSGIDENSI-GACAQLVFAPIDESFA---------------D---
XP_007147487.1/1-285 -DMY-LL------QLCSGVDENAI-GACAQLVFAPIDESFA---------------D---
XP_017437412.1/1-285 -DMY-LL------QLCSGVDENAI-GACAQLVFAPIDESFA---------------D---
XP_014518680.1/1-285 -DMY-LL------QLCSGVDENAI-GACAQLVFAPIDESFA---------------D---
XP_003546255.1/1-285 -DMY-LL------QLCSGVDESAV-GACAQLVFAPIDESFA---------------D---
XP_020233213.1/1-285 -DMY-LL------QLCSGVDENAI-GACAQLVFAPIDESFA---------------D---
XP_003535078.1/1-285 -DMY-LL------QLCSGVDENAI-GACAQLVFAPIDESFA---------------D---
XP_014617344.1/1-285 -DMY-LL------QLCSGVDENAI-GACAQLVFAPIDESFA---------------D---
XP_015959291.1/1-283 -DMY-LL------QLCSGVDENAI-GACAQLVFAPIDESFA---------------D---
XP_016197724.1/1-283 -DMY-LL------QLCSGVDENAI-GACAQLVFAPIDESFA---------------D---
XP_025694278.1/1-283 -DMY-LL------QLCSGVDENAI-GACAQLVFAPIDESFA---------------D---
XP_006846777.1/1-281 -DLY-LL------QLCSGVDENAV-GAISQLVFAPIDDSFA---------------D---
XP_003574071.1/1-280 -DIH-LL------QFCTGIDEKSM-GSCFQLVFAPIDELFP---------------D---
XP_020174778.1/1-278 -DIH-LL------QFCTGLDEKSM-GSCFQLVFAPIDELFP---------------D---
XP_006661867.1/1-281 -DIH-LL------QLCTGIDEKSM-GSCFQLVFAPIDELFP---------------D---
XP_015614901.1/1-281 -DIH-LL------QLCTGIDEKSM-GSCFQLVSAPIDELFP---------------D---
XP_004982838.1/1-282 -DIH-LL------QLCTGIEEKSM-GSCFQLVFAPIDELFP---------------D---
XP_025796396.1/1-282 -DIH-LL------QLCTGIDEKSM-GSCFQLVFAPIDELFP---------------D---
NP_001316502.1/1-284 -DIH-LL------QLCTGMDEKSM-GSCFQLVFAPIDELFP---------------D---
XP_021306737.1/1-282 -DIH-LL------QLCTGIDEKSM-GSCFQLVFAPIDELFP---------------D---
XP_021306733.1/1-293 -DIH-LL------QLCTGIDEKSM-GSCFQLVFAPIDELFP---------------D---
XP_020162053.1/1-281 -DID-ML------QLCTGIDEKSV-GSSFQLVFAPID-DFP---------------D---
XP_006649248.1/1-282 -DIH-LL------QLCTGIDEKSV-GSSFQLVFAPID-DFP---------------D---
XP_015632709.1/1-282 -DIH-LL------QLCTGIDEKSV-GSSFQLVFAPID-DFP---------------D---
NP_001296150.1/1-282 -DIH-LL------QLCTGIDEKSV-GSSFQLVFAPIDEHFP---------------D---
XP_008665074.1/1-283 -DIH-LL------QLCTGIDEKSV-GSSFQLVFAPIDEHFP---------------D---
NP_001105994.1/1-283 -DIH-LL------QLCTGIDEKSV-GSSFQLVFAPIDEHFP---------------D---
XP_008660404.1/1-283 -DIH-LL------QLCTGIDEKSV-GSSFQLVFAPIDEHFP---------------D---
XP_002468669.1/1-283 -DIH-LL------QLCTGIDEKSV-GSSFQLVFAPIDEHFP---------------D---
XP_021309716.1/1-285 -DIH-LL----QFQLCTGIDEKSV-GSSFQLVFAPIDEHFP---------------D---
XP_004986037.1/1-283 -DIH-LL------QLCTGIDEKSV-GSSFQLVFAPIDEHFP---------------D---
XP_025795001.1/1-283 -DIH-LL------QLCTGIDEKSV-GSSFQLVFAPIDEHFP---------------D---
XP_020093893.1/1-283 -DVH-LL------QLCCGIDENAV-GSCFQLVFAPIDEHFP---------------D---
XP_020093894.1/1-282 -DVH-LL------QLCCGIDENAV-GSCFQLVFAPIDEHFP---------------D---
XP_020259754.1/1-283 -DIH-LL------QLCSGIDENAA-GSCVQLVFAPIDELFP---------------D---
XP_009401955.1/1-282 -DIH-LL------QLCCGIDENSL-GACFQLVFAPIDELFP---------------D---
XP_008791541.1/1-283 -DVH-LL------QLCSGIDENAV-GACFQLVFALIDELFP---------------D---
XP_017698637.1/1-283 -DVH-LL------QLCSGIDENAV-GACFQLVFALIDELFP---------------D---
XP_010920422.1/1-283 -DVH-LL------QLCSGIDENAV-GACFQLVFAPIDELFP---------------D---
XP_010920423.1/1-283 -DVH-LL------QLCSGIDENAV-GACFQLVFAPIDELFP---------------D---
XP_010920419.1/1-283 -DVH-LL------QLCSGIDENAV-GACFQLVFAPIDELFP---------------D---
XP_010920426.1/1-283 -DVH-LL------QLCSGIDENAV-GACFQLVFAPIDELFP---------------D---
XP_019709326.1/1-287 -DIH-LL------QLCSGIDENAV-GACFELVFAPIDELFL---------------D---
XP_008785281.1/1-283 -DIH-LL------QLCSGINENAV-GACFELVFAPIDEHFL---------------D---
XP_008462593.1/1-288 -DIH-LL------QMCTGIDENAV-GACSELVFAPIDEMFP---------------D---
XP_022969629.1/1-286 -DVH-LL------QMCTGIDENAV-GACSELVFAPINEMFP---------------D---
XP_023536970.1/1-286 -DVH-LL------QMCTGIDENAV-GACSELVFAPINEMFP---------------D---
XP_022937855.1/1-288 -DVH-LL------QMCTGIDENAV-GACSELVFAPINEMFP---------------D---
XP_022937858.1/1-288 -DVH-LL------QMCTGIDENAV-GACSELVFAPINEMFP---------------D---
XP_012827869.1/1-285 -DIH-LL------QLCTGIDESAV-GACSELIFAPIDEMFP---------------D---
XP_022874815.1/1-279 -DIH-LL------QMCSGVDENTV-GACSELVFAPIDEMFP---------------D---
XP_022874814.1/1-283 -DIH-LL------QMCSGVDENTV-GACSELVFAPIDEMFP---------------D---
XP_018434000.1/1-280 -DVH-LL------QICTGVDEDAV-GACSELIFAPINEMFP---------------D---
XP_013642496.1/1-279 -DVH-LL------QICTGVDEDAV-GACSELVFAPVNEMFP---------------D---
XP_009150087.1/1-279 -DVH-LL------QICTGVDEDAV-GACSELVFAPVNEMFP---------------D---
XP_009150088.1/1-279 -DVH-LL------QICTGVDEDAV-GACSELVFAPVNEMFP---------------D---
XP_022563181.1/1-279 -DVH-LL------QICTGIDEDAV-GACSELVFAPVNEMFP---------------D---
XP_013675790.1/1-280 -DVH-LL------QICTGIDEDAV-GACSELVFAPVNEMFP---------------D---
XP_013623090.1/1-279 -DVH-LL------QICTGIDEDAL-GACSELVFAPVNEMFP---------------D---
XP_013623089.1/1-280 -DVH-LL------QICTGIDEDAL-GACSELVFAPVNEMFP---------------D---
XP_020885782.1/1-285 -DVH-LL------QICTGIDENAV-GACSELIFAPINEMFP---------------D---
XP_006280004.1/1-285 -DVH-LL------QICTGIDENAV-GACSELIFAPINEMFP---------------D---
XP_019088563.1/1-285 -DVH-LL------QICTGIDENAV-GACSELIFAPINEMFP---------------D---
XP_010483719.1/1-285 -DVH-LL------QICTGIDENAV-GACSELIFAPINEMFP---------------D---
XP_010456679.1/1-285 -DVH-LL------QICTGIDENAV-GACSELIFAPINEMFP---------------D---
XP_006400821.1/1-284 -DVH-LL------QICTGIDENAV-GACSELIFAPINEMFP---------------D---
NP_200877.1/1-284    -DVH-LL------QICTGIDENAV-GACSELIFAPINEMFP---------------D---
XP_013611543.1/1-282 -DVH-LL------QICTGIDENAV-GACSELIFAPINEMFP---------------D---
XP_009120605.1/1-284 -DVH-LL------QICTGIDENAV-GACSELIFAPINEMFP---------------D---
XP_013667152.1/1-284 -DVH-LL------QICTGIDENAV-GACSELIFAPINEMFP---------------D---
XP_018446535.1/1-284 -DVH-LL------QICTGIDENAV-GACSELIFAPINEMFP---------------D---
XP_018454374.1/1-284 -DVH-LL------QICAGIDENAV-GACSELIFAPINEMFP---------------D---
XP_009126588.1/1-285 -DVH-LL------QICTGIDENAV-GACSELIFAPINEIFP---------------D---
XP_013680521.1/1-285 -DVH-LL------QICSGIDENAV-GACSELIFAPINEMFP---------------D---
XP_013612427.1/1-285 -DVH-LL------QICSGIDENAV-GACSELIFAPINEMFP---------------D---
XP_010535608.1/1-283 -DVH-LL------QICTGIDENAV-GACSELVFAPINEMFP---------------D---
XP_010520372.1/1-276 -DVH-LL------QICTGIDENVI-GACSELVFAPINEMFP---------------D---
XP_004487103.1/1-286 -DVH-LL------QLCSGIDENAV-GACSELIFAPIDDMFP---------------D---
XP_003597252.1/1-283 -DVH-LL------QLCTGIDENAV-GACSELIFAPIDDMFP---------------E---
XP_024455683.1/1-286 -DIH-LL------QICSGIDENAV-GACSELVFAPIDETFP---------------D---
XP_022026352.1/1-288 -DIH-LL------QLCNGIDENAV-GACSELVFAPIDEMFP---------------D---
XP_022000486.1/1-288 -DIH-LL------QLCNGIDENAV-GACSELIFAPIDEMFP---------------D---
XP_016717477.1/1-281 -DIH-LL------QICSGIDDNAV-GACSELVFAPIDEMFP---------------D---
XP_017643014.1/1-281 -DIH-LL------QICSGIDDNAV-GACSELVFAPIDEMFP---------------D---
XP_016724213.1/1-281 -DIH-LL------QICSGIDDNAV-GACSELVFAPIDEMFP---------------D---
XP_012462938.1/1-281 -DIH-LL------QICSGIDDNAV-GACSELVFAPIDEMFP---------------D---
XP_012471509.1/1-292 -DIH-LL------QICSGIDENAV-GACSELVFAPIDEMFP---------------D---
XP_017642742.1/1-292 -DIH-LL------QICSGIDENAV-GACSELVFAPIDEMFP---------------D---
XP_016755876.1/1-292 -DIH-LL------QICSGIDENAV-GACSELVFAPIDEMFP---------------D---
XP_012471510.1/1-286 -DIH-LL------QICSGIDENAV-GACSELVFAPIDEMFP---------------D---
XP_016753711.1/1-286 -DIH-LL------QICSGIDENAV-GACSELVFAPIDEMFP---------------D---
XP_017642744.1/1-286 -DIH-LL------QICSGIDENAV-GACSELVFAPIDEMFP---------------D---
XP_016755877.1/1-286 -DIH-LL------QICSGIDENAV-GACSELVFAPIDEMFP---------------D---
XP_016750468.1/1-286 -DIH-LL------QICSGIDENAV-GACSELVFAPIDEMFP---------------D---
XP_016688657.1/1-286 -DIH-LL------QICSGIDENAV-GACSELVFAPIDEMFP---------------D---
XP_012443272.1/1-286 -DIH-LL------QICSGIDENAV-GACSELVFAPIDEMFP---------------D---
XP_017603001.1/1-286 -DIH-LL------QICSGIDENAV-GACSELVFAPIDEMFP---------------D---
XP_022755632.1/1-285 -DIH-LL------QICSGIDENAV-GACSELVFAPIDEMFP---------------D---
XP_022755633.1/1-285 -DIH-LL------QICSGIDENAV-GACSELVFAPIDEMFP---------------D---
XP_022729815.1/1-286 -DVH-LL------QICCGIDENAV-GACSELVFAPIDEMFP---------------D---
XP_022729816.1/1-286 -DVH-LL------QICCGIDENAV-GACSELVFAPIDEMFP---------------D---
XP_022752857.1/1-286 -DIH-LL------QICSGIDENAV-GACSELVFAPIDEMFP---------------D---
XP_022752856.1/1-286 -DIH-LL------QICSGIDENAV-GACSELVFAPIDEMFP---------------D---
XP_021277981.1/1-286 -DIH-LL------QICSGIDENAV-GACSELVFAPIDEMFP---------------D---
XP_017983554.1/1-286 -DIH-LL------QICSGIDENAV-GACSELVFAPIDEMFP---------------D---
XP_011089912.1/1-283 -DIH-LL------QMCSGVDENAV-GACSELVFAPIDEMFP---------------D---
XP_007208090.1/1-286 -DIH-LL------QICSGVDENAV-GACSELVFAPIDEMFP---------------D---
XP_008225704.1/1-286 -DIH-LL------QICSGVDENAV-GACSELVFAPIDEMFP---------------D---
XP_021830853.1/1-286 -DIH-LL------QICSGVDENAV-GACSELVFAPIDEMFP---------------D---
XP_008349754.1/1-286 -DIH-LL------QICSGVDENAV-GACSELVFAPIDEMFP---------------D---
NP_001315783.1/1-288 -DIH-LL------QICSGVDENAV-GACSELVFAPIDEMFP---------------D---
XP_009367069.1/1-286 -DIH-LL------QICSGVDENAA-GACSELVFAPIDEMFP---------------D---
XP_008344872.1/1-286 -DIH-LL------QICSGVDENAA-GACSELVFAPIDEMFP---------------D---
XP_015952399.1/1-286 -DIH-LL------QICSGIDENAV-GACSELIFAPIDEMFP---------------D---
XP_020982118.1/1-286 -DIH-LL------QICSGIDENAV-GACSELIFAPIDEMFP---------------D---
XP_025685402.1/1-286 -DIH-LL------QICSGIDENAV-GACSELIFAPIDEMFP---------------D---
XP_016187430.1/1-286 -DIH-LL------QICSGIDENAV-GACSELIFAPIDEMFP---------------D---
XP_020214107.1/1-284 -DIH-LL------QICSGIDEHAV-GACSELIFAPIDEMFP---------------D---
XP_006592258.1/1-284 -DIH-LL------QICSGIDENAV-GACSELVFAPIDEMFP---------------D---
XP_007132262.1/1-285 -DIH-LL------QICSGIDENAV-GACSELVFAPIDEMFP---------------D---
XP_003538150.1/1-285 -DIH-LL------QICSGIDENAV-GACSELVFAPIDEMFP---------------D---
XP_017433493.1/1-285 -DIH-LL------QICSGIDENAV-GACSELVFAPIDEMFP---------------D---
XP_014494018.1/1-285 -DIH-LL------QICSGIDENAV-GACSELVFAPIDEMFP---------------D---
XP_010089983.2/1-286 -DIH-LL------QICSGVDDNAV-GACSELVFAPIDEMFP---------------D---
XP_006378974.2/1-285 -DIH-LL------QICSGIDENAV-GACSELVFAPIDEMFP---------------D---
XP_011010051.1/1-285 -DIH-LL------QICSGIDENAV-GACSELVFAPIDEMFP---------------D---
XP_011010050.1/1-285 -DIH-LL------QICSGIDENAV-GACSELVFAPIDEMFP---------------D---
XP_021905657.1/1-285 -DIH-LL------QICSGIDENAV-GACSELVFAPIDEMFP---------------D---
XP_021610391.1/1-286 -DIH-LL------QICSGIDENAV-GACAELVFAPIDEMFP---------------D---
XP_002529946.1/1-286 -DIH-LL------QICSGIDENAV-GACSELVFAPIDEMFP---------------D---
XP_021658728.1/1-286 -DIH-LL------QICSGIDENAV-GACSELVFAPIDEMFP---------------D---
XP_012064816.1/1-286 -DIH-LL------QICSGIDENAV-GACSELVFAPIDEMFP---------------D---
XP_021628343.1/1-286 -DIH-LL------QICSGIDENAV-GACSELVFAPIDEMFP---------------D---
XP_021657859.1/1-286 -DIH-LL------QICCGIDENAV-GACSELVFAPIDEMFP---------------D---
XP_015874442.1/1-285 -DIH-LL------QICSGVDENAV-GACSELVFAPIDEMFP---------------D---
XP_023926843.1/1-286 -DIH-LL------QICSGVDENAV-GACSELVFAPIDEMFP---------------D---
XP_018819529.1/1-286 -DIH-LL------QICSGVDEHAV-GACSELVFAPIDEMFP---------------D---
XP_010651164.1/1-286 -DIH-LL------QICSGVDENAV-GACSELVFAPIDEMFP---------------D---
XP_024034672.1/1-286 -DIH-LL------QICSGVDENAV-GACSELVFAPIDEMFP---------------D---
XP_006488084.1/1-286 -DIH-LL------QICSGVDENAV-GACSELVFAPIDEMFP---------------D---
XP_024034673.1/1-286 -DIH-LL------QICSGVDENAV-GACSELVFAPIDEMFP---------------D---
XP_010264677.1/1-287 -DIH-LL------QICSGVDENAV-GACSELVFAPIDEMFP---------------D---
XP_010253680.1/1-288 -DIH-LL------QICCGLDENAV-GSCSELVFAPIDEMFP---------------D---
XP_022005829.1/1-282 -DIH-LL------QLCSGIDENAV-GACSELVFAPIDEMFP---------------D---
XP_023751537.1/1-278 -DIH-LL------QLCSGVDENAV-GACSELIFAPIDEMFP---------------D---
XP_024971452.1/1-283 -DIH-LL------QLCSGIDENAV-GACSELIFAPIDEMFP---------------D---
XP_024971444.1/1-287 -DIH-LL------QLCSGIDENAV-GACSELIFAPIDEMFP---------------D---
XP_024985647.1/1-287 -DIH-LL------QLCSGIDENAV-GACSELVFAPIDEMFP---------------D---
XP_023749448.1/1-288 -DIH-LL------QLCSGIDENAV-GACSELIFAPIDEMFP---------------D---
XP_010689538.1/1-281 -DIH-LL------QICIGIDENAV-GACSELVFAPIDEMFP---------------D---
XP_021858740.1/1-280 -DIH-LL------QICSGIDENAV-GACSELVFAPIDEMFP---------------D---
XP_021773317.1/1-281 -DIH-LL------QICSGIDENAV-GACSELVFAPIDEMFP---------------D---
XP_021745941.1/1-281 -DIH-LL------QICSGIDENAV-GACSELVFAPIDEMFP---------------D---
XP_016729039.1/1-286 -DIH-LL------QLCSGIDENAV-GGCSELLFAPINEMFP---------------D---
XP_017623828.1/1-286 -DIH-LL------QLCSGIDENAV-GGCSELLFAPINEMFP---------------D---
XP_012476406.1/1-286 -DIH-LL------QLCSGIDENAV-GGCSELLFAPINEMFP---------------D---
XP_016754776.1/1-286 -DIH-LL------QLCSGIDENAV-GGCSELLFAPINEMFP---------------D---
XP_022152944.1/1-286 -DIH-LL------QMCTGIDENAV-GACSELVFAPIDEMFP---------------D---
XP_011040884.1/1-286 -DIH-LL------QICSGIDENAV-GACSELVFAPIDEMFP---------------D---
XP_010043753.1/1-286 -DIH-LL------QICSGIDENAV-GVCSELIFAPIDEMFP---------------D---
XP_024158223.1/1-285 -DIH-LL------QICSGVDENAV-GACSELVFAPIDEMFP---------------D---
XP_004291573.1/1-285 -DIH-LL------QICSGVDENAV-GACSELFFAPIDEMFP---------------D---
XP_018808765.1/1-285 -DIH-LL------QICNGVDEHAV-GACSELIFATIDEMFP---------------D---
XP_023512455.1/1-283 -DIH-LL------QICSGIDENAV-GACSELIFAPIDEMFP---------------D---
XP_023512456.1/1-283 -DIH-LL------QICSGIDENAV-GACSELIFAPIDEMFP---------------D---
XP_022944186.1/1-283 -DIH-LL------QICSGIDENAV-GACSELIFAPIDEMFP---------------D---
XP_022944185.1/1-283 -DIH-LL------QICSGIDENAV-GACSELIFAPIDEMFP---------------D---
XP_022985614.1/1-283 -DIH-LL------QICSGIDENAV-GACSELIFAPIDEMFP---------------D---
XP_022985615.1/1-283 -DIH-LL------QICSGIDENAV-GACSELIFAPIDEMFP---------------D---
XP_011656996.1/1-283 -DIH-LL------QICSGIDENAV-GACSELIFAPIDEMFP---------------D---
XP_011656997.1/1-283 -DIH-LL------QICSGIDENAV-GACSELIFAPIDEMFP---------------D---
XP_008463945.1/1-283 -DIH-LL------QICSGIDENAV-GACSELIFAPIDEMFP---------------D---
XP_008463946.1/1-283 -DIH-LL------QICSGIDENAV-GACSELIFAPIDEMFP---------------D---
XP_022140363.1/1-286 -DIH-LL------QICSGIDENAV-GACSELIFAPIDEMFP---------------D---
XP_022995706.1/1-286 -DIH-LL------QICSGINENAV-GACSELIFAPIDEMFP---------------D---
XP_023534373.1/1-286 -DIH-LL------QICSGINENAV-GACSELIFAPIDEMFP---------------D---
XP_022958713.1/1-286 -DIH-LL------QICSGINENAV-GACSELIFAPIDEMFP---------------D---
XP_025614062.1/1-285 -DVH-LL------QLCSGIDENAV-GACSELIFAPIDEMFP---------------D---
XP_025614063.1/1-285 -DVH-LL------QLCSGIDENAV-GACSELIFAPIDEMFP---------------D---
XP_016170485.1/1-285 -DVH-LL------QLCSGIDENAV-GACSELIFAPIDEMFP---------------D---
XP_016170486.1/1-285 -DVH-LL------QLCSGIDENAV-GACSELIFAPIDEMFP---------------D---
XP_015935757.1/1-285 -DVH-LL------QLCSGIDENAV-GACSELIFAPIDEMFP---------------D---
XP_015935758.1/1-285 -DVH-LL------QLCSGIDENAV-GACSELIFAPIDEMFP---------------D---
XP_004505999.1/1-286 -DIH-LL------QICSGTDENAV-GTCSELIFAPIDEMFP---------------D---
XP_013456006.1/1-286 -DIH-LL------QICSGTDENAV-GTCSELIFAPIDEMFP---------------D---
XP_019453966.1/1-285 -DIH-LL------QICSGIDENDT-GACSELIFAPIDEMFP---------------D---
XP_019448740.1/1-279 -DIH-LL------QICSGVDENAI-GACSELIFAPIDEMFL---------------D---
XP_019448741.1/1-279 -DIH-LL------QICSGVDENAI-GACSELIFAPIDEMFL---------------D---
XP_019458095.1/1-286 -DIH-LL------QICSGIDENAV-GLCSELIFAPINEMFP---------------D---
XP_019447870.1/1-286 -DIH-LL------QICSGIDENSV-GPCSELIFAPIDEMFP---------------D---
XP_012838629.1/1-280 -DIH-LL------QMCSGVDENAV-GACSELIFAPIDEMFP---------------D---
XP_012838630.1/1-280 -DIH-LL------QMCSGVDENAV-GACSELIFAPIDEMFP---------------D---
XP_012838627.1/1-280 -DIH-LL------QMCSGVDENAV-GACSELIFAPIDEMFP---------------D---
XP_012838628.1/1-280 -DIH-LL------QMCSGVDENAV-GACSELIFAPIDEMFP---------------D---
XP_017214964.1/1-286 -DIH-LL------QLCSGVDESAV-GACSELVFAPIDEMFP---------------D---
XP_017234817.1/1-286 -DIH-LL------QLCCGVDENAV-GACSELVFAPIDEMFP---------------D---
XP_019178153.1/1-282 -DIH-LL------QMCRGIDESAV-GACAELVFAPIDEMFP---------------D---
XP_019166562.1/1-283 -DIH-LL------QMCSGIDENAV-GACSELVFAPIDEMFP---------------D---
XP_011089294.1/1-282 -DIH-LL------QMCSGIDENAV-GACSELIFAPIDEMFP---------------D---
XP_022892122.1/1-274 -DIH-LL------QMCSGVDENAV-GACSELVFAPIDEMFP---------------D---
XP_022883393.1/1-279 -DIH-LL------QMCSGVDENAV-GACSELVFAPIDEIFP---------------D---
XP_022883398.1/1-279 -DIH-LL------QMCSGVDENAV-GACSELVFAPIDEIFP---------------D---
XP_022883384.1/1-283 -DIH-LL------QMCSGVDENAV-GACSELVFAPIDEIFP---------------D---
XP_022883389.1/1-283 -DIH-LL------QMCSGVDENAV-GACSELVFAPIDEIFP---------------D---
XP_009776589.1/1-283 -DVH-LL------QMCSGTDENAV-GACSELVFAAIDEMFP---------------D---
NP_001312504.1/1-283 -DVH-LL------QMCSGTDENAV-GACSELVFAAIDGMFP---------------D---
XP_016472209.1/1-283 -DVH-LL------QMCSGTDENAV-GACSELVFAAIDEMFP---------------D---
XP_019255429.1/1-283 -DIH-LL------QMCSGTDENAV-GACSELVFAAIDEMFP---------------D---
XP_009624234.1/1-283 -DIH-LL------QMCSGTDENAV-GACSELVFAAIDEMFP---------------D---
XP_016504967.1/1-283 -DIH-LL------QMCSGTDENAV-GACSELVFAAIDEMFP---------------D---
XP_016547131.1/1-283 -DIH-LL------QMCSGTDENAV-GACSELVFAPIDEMFP---------------D---
XP_004251138.1/1-283 -DIH-LL------QMCSGTDENAV-GACSELVFAPIDEMFP---------------D---
XP_015058512.1/1-283 -DIH-LL------QMCSGTDENAV-GACSELVFAPIDEMFP---------------D---
XP_006340193.1/1-283 -DIH-LL------QMCSGTDENAV-GACSELVFAPIDEMFP---------------D---
XP_004235650.1/1-283 -DMF-LL------QLCSGMDENAV-GTCAELVFAPIDASFA---------------D---
XP_006343047.1/1-283 -DMF-LL------QLCSGMDENAV-GTCAELVFAPIDASFA---------------D---
XP_009774805.1/1-285 -DMF-LL------QLCSGMDENAV-GTSAELIFAPIDASFA---------------D---
XP_016498616.1/1-285 -DMF-LL------QLCSGMDENAV-GTSAELIFAPIDASFA---------------D---
XP_016434103.1/1-285 -DMF-LL------QLCSGMDENAV-GTSAELIFAPIDASFA---------------D---
XP_009593967.1/1-285 -DMF-LL------QLCSGMDENAV-GTSAELIFAPIDASFA---------------D---
XP_004510297.1/1-285 -ELF-LL------QLCSGMDENAI-GTCAELIFAPIDASFA---------------D---
XP_004510296.1/1-285 -ELF-LL------QLCSGMDENAI-GTCAELIFAPIDASFA---------------D---
XP_003627005.1/1-284 -ELF-LL------QLCSGMDENAI-GTCAELIFAPIDASFA---------------D---
XP_003531653.1/1-284 -EMF-LL------QLCSGMDENAV-GTCAELISAPIDASFA---------------D---
XP_006585576.1/1-288 -EMF-LL------QLCSGMDENAV-GTCAELISAPIDASFA---------------D---
XP_014634618.1/1-288 -EMF-LL------QLCSGMDENAV-GTCAELISAPIDASFA---------------D---
XP_014634619.1/1-288 -EMF-LL------QLCSGMDENAV-GTCAELISAPIDASFA---------------D---
XP_003531652.1/1-285 -EMF-LL------QLCSGMDENAV-GTCAELISAPIDASFA---------------D---
XP_003530109.1/1-284 -EMF-LL------QLCSGMDENAV-GTCAELISAPIDASFA---------------D---
XP_007135643.1/1-285 -EMF-LL------QLCSGMDENAV-GTCAELISAPIDASFA---------------D---
XP_003530112.1/1-285 -EMF-LL------QLCSGMDENAV-GTCAELISAPIDASFA---------------D---
XP_020226142.1/1-285 -EMF-LL------QLCSGMDENAV-GTCAELISAPIDASFA---------------D---
XP_014516466.1/1-285 -EMF-LL------QLCSGMDENAV-GTCAELISAPIDASFA---------------D---
XP_017407594.1/1-285 -EMF-LL------QLCSGMDENAV-GTCAELISAPIDASFA---------------D---
XP_019448178.1/1-285 -EMF-LL------QLCTGMDENAV-GTSAELIFAPIDVSFA---------------D---
XP_019429328.1/1-285 -EVF-LL------QLCSGMDENAV-GTCAELIFAPIDASFA---------------D---
XP_004486776.1/1-285 -EVF-LL------QLCSGMDENAV-GTCAELIFAPIDASFA---------------D---
XP_003597690.1/1-286 -EVF-LL------QLCSGMDENAV-GTCAELIFAPIDASFA---------------D---
XP_015932679.1/1-285 -EVF-LL------QLCSGMDENAV-GTCAELIFAPIDASFA---------------D---
XP_025674177.1/1-285 -EVF-LL------QLCSGMDENAV-GTCAELIFAPIDASFA---------------D---
XP_025610204.1/1-285 -EVF-LL------QLCSGMDENAV-GTCAELIFAPIDASFA---------------D---
XP_020226055.1/1-285 -EVF-LL------QLCSGMDENAV-GTCAELIFAPIDASFA---------------D---
XP_007150614.1/1-285 -EVF-LL------QLCSGMDENAV-GTCAELIFAPIDASFA---------------D---
XP_014501306.1/1-285 -EVF-LL------QLCSGMDENAV-GTCAELIFAPIDASFA---------------D---
XP_017425270.1/1-285 -EVF-LL------QLCSGMDENAV-GTCAELIFAPIDASFA---------------D---
XP_021836126.1/1-285 -DMF-LL------QLCSGMDENAI-GTCAELIFAPIDASFA---------------D---
XP_010691423.1/1-284 -DMF-LL------QLCSGMDENAV-GTCAELIFAPIDASFA---------------D---
XP_010691424.1/1-284 -DMF-LL------QLCSGMDENAV-GTCAELIFAPIDASFA---------------D---
XP_021765330.1/1-284 -DMF-LL------QLCSGMDENAV-GTCAELIFAPIDASFA---------------D---
XP_021749885.1/1-284 -DMF-LL------QLCSGMDENAV-GTCAELIFAPIDASFA---------------D---
XP_010267183.1/1-285 -DMF-LL------QLCSGVDENAV-GTCAELIFAPIDASFA---------------D---
XP_017258627.1/1-285 -DMF-LL------QLCSGMDENAV-GICAELIFAPIDASFA---------------D---
XP_023000315.1/1-284 -EMF-LL------QLCSGMDENAI-GTCAELIFAPIDASFA---------------D---
XP_023000313.1/1-284 -EMF-LL------QLCSGMDENAI-GTCAELIFAPIDASFA---------------D---
XP_023514554.1/1-284 -EMF-LL------QLCSGMDENAI-GTCAELIFAPIDASFA---------------D---
XP_023514552.1/1-284 -EMF-LL------QLCSGMDENAI-GTCAELIFAPIDASFA---------------D---
XP_022964458.1/1-284 -EMF-LL------QLCSGMDENAI-GTCAELIFAPIDASFA---------------D---
XP_022964454.1/1-284 -EMF-LL------QLCSGMDENAI-GTCAELIFAPIDASFA---------------D---
XP_022147627.1/1-284 -EMF-LL------QLCSGMDENAI-GTCAELIFAPIDASFA---------------D---
XP_022147619.1/1-284 -EMF-LL------QLCSGMDENAI-GTCAELIFAPIDASFA---------------D---
XP_008462437.1/1-284 -EMF-LL------QLCSGMDENAI-GTCAELIFAPIDASFA---------------D---
XP_004141659.1/1-284 -EMF-LL------QLCSGMDENAI-GTCAELIFAPIDASFA---------------D---
XP_008462436.1/1-284 -EMF-LL------QLCSGMDENAI-GTCAELIFAPIDASFA---------------D---
XP_011659682.1/1-284 -EMF-LL------QLCSGMDENAI-GTCAELIFAPIDASFA---------------D---
XP_022992111.1/1-284 -EMF-LL------QLCSGMDENAI-GTCAELIFAPIDASFA---------------D---
XP_023548534.1/1-284 -EMF-LL------QLCSGMDENAI-GTCAELIFAPIDASFA---------------D---
XP_022953371.1/1-284 -EMF-LL------QLCSGMDENAI-GTCAELIFAPIDASFA---------------D---
XP_002304217.1/1-285 -DVF-LL------QLCCGMDENAV-GTCAELIFAPIDATFA---------------D---
XP_011013041.1/1-285 -DVF-LL------QLCCGMDENAV-GACAELIFAPIDATFA---------------D---
XP_011013042.1/1-285 -DVF-LL------QLCCGMDENAV-GACAELIFAPIDATFA---------------D---
XP_011046759.1/1-284 -DVF-LL------QLCCGMDENAV-GTCAELIFAPIDATFA---------------D---
XP_024464085.1/1-284 -DVF-LL------QLCCGMDENAV-GTCAELIFAPIDATFA---------------D---
XP_024183498.1/1-286 -EMF-LL------QLCSGMDENAV-GSCAELIFAPIDASFA---------------D---
XP_004302866.1/1-286 -EMF-LL------QLCSGMDETAV-GSCAELIFAPIDASFA---------------D---
NP_001280898.1/1-285 -EMF-LL------QLCSGMDENAV-GSCAELIFAPIDASFA---------------D---
XP_007214960.1/1-285 -EMF-LL------QLCSGMDENAV-GSCAELIFAPIDASFA---------------D---
XP_008228437.1/1-285 -EMF-LL------QLCSGMDENAV-GSCAELIFAPIDASFA---------------D---
XP_021818763.1/1-285 -EMF-LL------QLCSGMDENAV-GSCAELIFAPIDASFA---------------D---
XP_022723515.1/1-285 -DIF-LL------QLCSGMDENAV-GACAELIFAPIDASFA---------------D---
XP_016688508.1/1-285 -DVF-LL------QLCSGMDENAV-GTCAELIFAPIDASFA---------------D---
XP_012443274.1/1-285 -DVF-LL------QLCSGMDENAV-GTCAELIFAPIDASFA---------------D---
XP_022721457.1/1-285 -DVF-LL------QLCSGMDENAV-GTCAELIFAPIDASFA---------------D---
XP_022715018.1/1-285 -DVF-LL------QLCSGMDENAV-GTCAELIFAPIDASFA---------------D---
XP_017979082.1/1-285 -DVF-LL------QLCSGMDENAV-GTCAELIFAPIDASFA---------------D---
XP_021293768.1/1-285 -DVF-LL------QLCSGMDENAV-GTCAELIFAPIDASFA---------------D---
XP_010062518.1/1-285 -DIF-FL------QMCSGVDENAV-GTFAELIFAPIDASFA---------------D---
XP_006426893.1/1-285 -DMF-LL------QLCSGMDENAV-GTCAELIFAPIDASFA---------------D---
XP_021610318.1/1-285 -DMF-LL------QLCSGMDENAI-GTCAELIFAPIDASFA---------------D---
XP_010098130.1/1-285 -EMF-LL------QLCSGMDENAV-GSCAELIFAPIDASFA---------------D---
XP_021635282.1/1-296 -DMF-LL------QLCSGMDENAV-GTCAELIFAPIDASFA---------------D---
XP_002284003.2/1-285 -DMF-LL------QLCSGMDENAV-GTCAELIFAPIDASFA---------------D---
XP_002515977.1/1-285 -DMF-LL------QLCSGMDENAV-GTCAELIFAPIDASFA---------------D---
XP_021643260.1/1-285 -DMF-LL------QLCSGMDENAI-GTCAELIFAPIDASFA---------------D---
XP_021628194.1/1-285 -DMF-LL------QLCSGMDENAI-GTCAELIFAPIDASFA---------------D---
XP_015894591.1/1-285 -EMF-LL------QLCSGMDENAV-GSCAELIFAPIDASFA---------------D---
XP_012069098.1/1-285 -DMF-LL------QLCSGMDENAI-GTCAELIFAPIDASFA---------------D---
XP_018847857.1/1-285 -EMF-LL------QLCSGMDENAV-GTCAELIFAPIDASFA---------------D---
XP_023898511.1/1-285 -EMF-LL------QLCSGMDENAV-GTCAELIFAPIDASFA---------------D---
XP_010545673.1/1-285 -DIF-LL------QLCSGIDESAV-GTCGELIFAPIDASFA---------------D---
XP_010545670.1/1-286 -DIF-LL------QLCSGIDESAV-GTCGELIFAPIDASFA---------------D---
XP_010535179.1/1-285 -DIF-LL------QLCSGMDENAV-GTCAELIFAPIDASFA---------------D---
XP_018436929.1/1-283 -DIF-LL------QLCSGMDENAV-GTCAELIFAPIDASFA---------------D---
XP_013592012.1/1-283 -DIF-LL------QLCSGMDENAV-GTCAELIFAPIDASFA---------------D---
XP_013640375.1/1-284 -DIF-LL------QLCSGMDENAV-GTCAELIFAPIDASFA---------------D---
XP_013640376.1/1-283 -DIF-LL------QLCSGMDENAV-GTCAELIFAPIDASFA---------------D---
XP_009147709.1/1-283 -DIF-LL------QLCSGMDENAV-GTCAELIFAPIDASFA---------------D---
XP_006306297.1/1-281 -DIF-LL------QLCSGMDESAV-GTCAELIFAPIDASFA---------------D---
XP_010462051.1/1-281 -DIF-LL------QLCSGMDESAV-GTCAELIFAPIDASFA---------------D---
XP_010479714.1/1-281 -DIF-LL------QLCSGMDESAV-GTCAELIFAPIDASFA---------------D---
XP_010462052.1/1-281 -DIF-LL------QLCSGMDESAV-GTCAELIFAPIDASFA---------------D---
XP_010500807.1/1-281 -DIF-LL------QLCSGMDESAV-GTCAELIFAPIDASFA---------------D---
NP_849795.1/1-282    -DIF-LL------QLCSGMDENAV-GTCAELIFAPIDASFA---------------D---
NP_001077702.1/1-281 -DIF-LL------QLCSGMDENAV-GTCAELIFAPIDASFA---------------D---
NP_175627.1/1-281    -DIF-LL------QLCSGMDENAV-GTCAELIFAPIDASFA---------------D---
XP_020868586.1/1-281 -DIF-LL------QLCSGMDENAV-GTCAELIFAPIDASFA---------------D---
XP_006392953.1/1-282 -DIF-LL------QLCSGMDENAV-GTCAELIFAPIDASFA---------------D---
XP_018460807.1/1-282 -DIF-LL------QLCSGMDENAV-GTCAELIFAPIDASFA---------------D---
XP_013621760.1/1-283 -DIF-LL------QLCSGMDENAV-GTCAELIFAPIDASFA---------------D---
XP_018451524.1/1-282 -DIF-LL------QLCSGMDENAV-GTCAELIFAPIDASFA---------------D---
XP_009107148.1/1-282 -DIF-LL------QLCSGMDENAL-GTCAELIFAPIDASFA---------------D---
XP_009107147.1/1-283 -DIF-LL------QLCSGMDENAL-GTCAELIFAPIDASFA---------------D---
XP_009600774.1/1-284 -DMF-LL------QMCSGMDENAV-GTCAELIFAPIDASFA---------------D---
XP_016459079.1/1-284 -DMF-LL------QMCSGMDENAV-GTCAELIFAPIDASFA---------------D---
XP_019236029.1/1-285 -DMF-LL------QMCSGMDENAV-GTCAELIFAPIDASFA---------------D---
XP_009771098.1/1-285 -DMF-LL------QMCSGMDENAV-GTCAELIFAPIDASFA---------------D---
XP_016550962.1/1-285 -DMF-LL------QLCSGMDENAV-GTCAELVFAPIDASFA---------------D---
XP_016550960.1/1-285 -DMF-LL------QLCSGMDENAV-GTCAELVFAPIDASFA---------------D---
XP_016550961.1/1-285 -DMF-LL------QLCSGMDENAV-GTCAELVFAPIDASFA---------------D---
XP_006359469.1/1-285 -DMF-LL------QLCSGMDENAV-GTCAELIFAPIDASFA---------------D---
XP_004252354.1/1-285 -DMF-LL------QLCSGMDENAV-GTCAELVFAPIDASFA---------------D---
XP_015061298.1/1-285 -DMF-LL------QLCSGMDENAV-GTCAELVFAPIDASFA---------------D---
XP_019198775.1/1-285 -DMF-LL------QLCSGMDENAV-GTCAELVFAPIDASFA---------------D---
XP_019194412.1/1-285 -DMF-LL------QLCSGMDENAV-GTCAELIFAPIDASFA---------------D---
XP_016562656.1/1-283 -DMF-LL------QLCSGMDENAV-GTCAELVFAPIDASFA---------------D---
XP_019267274.1/1-285 -DMF-LL------QLCSGMDENAV-GTSAELIFAPIDASFA---------------D---
XP_019267275.1/1-282 -DMF-LL------QLCSGMDENAV-GTSAELIFAPIDASFA---------------D---
XP_022889649.1/1-285 -DLL-LL------QLCSGMDENAV-GTCAELIFAPIDASLA---------------D---
XP_022889648.1/1-285 -DLL-LL------QLCSGMDENAV-GTCAELIFAPIDASLA---------------D---
XP_012852689.1/1-281 -DLF-LL------QLCSGMDENAV-GTCGELIFAPIDASFV---------------E---
XP_012856468.1/1-279 -DVL-LL------QLCSGMDENTV-GTCAELIFAPIDASFT---------------D---
XP_011080516.1/1-285 -DIF-LL------QLCGGMDETAV-GTCAELIFAPIDASFA---------------D---
XP_011072422.1/1-285 -DVL-LL------QLCSGMDEHAV-GTCAELIFAPIDASFT---------------D---
XP_022851148.1/1-285 -DVF-LL------QLCSGIDENAV-GTCAELIFAPIDASFA---------------D---
XP_022844668.1/1-285 -DVF-LL------QLCSGIDENAV-GTCAELIFAPIDASFA---------------D---
XP_006853643.2/1-288 -DMF-LL------QLCSGVDENAV-GACAELVFAPIDPSFA---------------D---
XP_010249545.1/1-285 -DVF-LL------QHYSGVDENAV-GMCSELIFAPIDAAFS---------------D---
XP_010249539.1/1-285 -DVF-LL------QHYSGVDENAV-GMCSELIFAPIDAAFS---------------D---
XP_010915409.1/1-284 -DLF-LL------QLCSGVDENAV-GTCSELVFAPIDASFA---------------D---
XP_008791633.1/1-284 -DLF-LL------QLCNGVDENAV-GTCSELVFAPIDASFA---------------D---
XP_017235966.1/1-285 -DMF-LL------QLCGGMDENAF-GTCAELIFAPIDPSFA---------------D---
XP_023761873.1/1-288 -DMF-LL------QLCSGMDENAV-GMCSELVFAPIDASFA---------------D---
XP_021997125.1/1-286 -DMF-FL------QLCSGIDESAV-GMCSELIFAPIDASFA---------------D---
XP_021990052.1/1-285 -DMF-LL------QLCSGMDEKAV-GMCSELIFAPIDASFA---------------D---
XP_024979339.1/1-285 -DMF-LL------QLCSGMDENAV-GMCSELVFAPIDASFA---------------D---
XP_024979341.1/1-290 -DMF-LL------QLCSGMDENAV-GMCSELVFAPIDASFA---------------D---
XP_024979340.1/1-291 -DMF-LL------QLCSGMDENAV-GMCSELVFAPIDASFA---------------D---
XP_024979342.1/1-291 -DMF-LL------QLCSGMDENAV-GMCSELVFAPIDASFA---------------D---
XP_022016208.1/1-285 -DMF-LL------QLCSGMDENAV-GMCAELIFAPIDSSFA---------------D---
XP_022016207.1/1-311 -DMF-LL------QLCSGMDENAV-GMCAELIFAPIDSSFA---------------D---
XP_022009819.1/1-285 -DMF-LL------QLCNGMDENAV-GMCAELIFAPIDASFA---------------D---
XP_022009815.1/1-285 -DMF-LL------QLCNGMDENAV-GMCAELIFAPIDASFA---------------D---
XP_022009809.1/1-285 -DMF-LL------QLCNGMDENAV-GMCAELIFAPIDASFA---------------D---
XP_023759500.1/1-286 -DMF-LL------QLCNGMDENAV-GMCAELIFAPIDSSFA---------------D---
XP_023751740.1/1-285 -DMF-LL------QLCNGMDENAV-GMSAELIFAPIDASFA---------------D---
XP_024971803.1/1-285 -DMF-LL------QLCSGMDENAV-GMCAELIFAPIDSSFA---------------D---
XP_022033649.1/1-285 -DMF-LL------QLCNGMDENAV-GMCAELIFAPIDASFA---------------D---
XP_024972195.1/1-285 -DMF-LL------QLCNGMDENAV-GMCAELIFAPIDASFA---------------D---
XP_024972196.1/1-289 -DMF-LL------QLCNGMDENAV-GMCAELIFAPIDASFA---------------D---
XP_020248539.1/1-284 -DTF-LM------QLCSGVDEKAI-GTCSELVFAPIDASFA---------------D---
XP_020269197.1/1-284 -DLF-LL------QVCSGVDENAI-GNCSELVFAPIDASFA---------------D---
XP_020269196.1/1-284 -DLF-LL------QVCSGVDENAI-GNCSELVFAPIDASFA---------------D---
XP_009399813.1/1-284 -DLY-LL------QLCNGVDENAV-GTCSELIFAPIDASFA---------------D---
XP_009399812.1/1-284 -DLY-LL------QLCNGVDENAV-GTCSELIFAPIDASFA---------------D---
XP_009413853.1/1-284 -DLY-LL------QLCNGVDGNAF-GTCSELIFAPIDASFA---------------D---
XP_024985513.1/1-286 -DIF-FL------QLCSGVDEKAI-GTSAELIFAPIDASFT---------------D---
XP_023729871.1/1-286 -DIF-FL------QLCSGVDENSI-GTSAELIFAPIDASFT---------------D---
XP_022000974.1/1-282 -DIF-FL------QLCSGVDENAI-GTSAELIFAPIDASFT---------------D---
XP_022023495.1/1-282 -DIF-FL------QLCNGVDENAI-GTSAELIFAPIDASFT---------------D---
XP_010526102.1/1-286 -DIF-LL------QMCNGVDENAI-GTCAELIFAPIDASFS---------------D---
XP_019086035.1/1-282 -DIF-LL------QMCSGVDENAV-ESCAELIFAPIDASFS---------------D---
XP_010437837.1/1-288 -DIF-LL------QMCSGVDENAV-ESCAELIFAPIDASFS---------------D---
XP_010437838.1/1-288 -DIF-LL------QMCSGVDENAV-ESCAELIFAPIDASFS---------------D---
XP_022549566.1/1-283 -DIF-LL------QMCGGVDENAV-ESCSELIFAPIDASFS---------------D---
XP_013670651.1/1-283 -DIF-LL------QMCGGVDENAV-ESCSELIFAPIDASFS---------------D---
XP_020874560.1/1-283 -DIF-LL------QMCSGVDENAV-ESCAELIFAPIDASFS---------------D---
XP_020874561.1/1-283 -DIF-LL------QMCSGVDENAV-ESCAELIFAPIDASFS---------------D---
XP_020874562.1/1-283 -DIF-LL------QMCSGVDENAV-ESCAELIFAPIDASFS---------------D---
NP_195014.1/1-283    -DIF-LL------QMCSGVDENAV-ESCAELIFAPIDASFS---------------D---
XP_006412419.1/1-283 -DIF-LL------QMCSGVDENAV-ESCAELIFAPIDASFS---------------D---
XP_006412418.1/1-283 -DIF-LL------QMCSGVDENAV-ESCAELIFAPIDASFS---------------D---
XP_018471819.1/1-283 -DIF-LL------QMCSGVDENAV-ESCAELIFAPIDASFS---------------D---
XP_009125299.1/1-283 -DIF-LL------QMCSGVDENAV-ESCAELIFAPIDASFS---------------D---
XP_010432643.1/1-283 -DIF-LL------QMCSGVDENAV-ESCAELIFAPIDASFS---------------D---
XP_010432644.1/1-283 -DIF-LL------QMCSGVDENAV-ESCAELIFAPIDASFS---------------D---
XP_006283109.1/1-283 -DIF-LL------QMCSGVDENAV-ESCAELIFAPIDASFS---------------D---
XP_010437839.1/1-283 -DIF-LL------QMCSGVDENAV-ESCAELIFAPIDASFS---------------D---
XP_010437840.1/1-283 -DIF-LL------QMCSGVDENAV-ESCAELIFAPIDASFS---------------D---
XP_010447325.1/1-283 -DIF-LL------QMCSGVDENAV-ESCAELIFAPIDASFS---------------D---
XP_010447326.1/1-283 -DIF-LL------QMCSGVDENAV-ESCAELIFAPIDASFS---------------D---
XP_013622315.1/1-283 -DVF-LL------QMCSGVDENAV-ESCAELIFAPIDASFS---------------D---
XP_013656273.1/1-283 -DVF-LL------QMCSGVDENAV-ESCAELIFAPIDASFS---------------D---
XP_009108824.1/1-283 -DVF-LL------QMCSGVDENAV-ESCAELIFAPIDASFS---------------D---
XP_013730326.1/1-283 -DVF-LL------QMCSGVDENAV-ESCAELIFAPIDASFS---------------D---
XP_013730325.1/1-283 -DVF-LL------QMCSGVDENAV-ESCAELIFAPIDASFS---------------D---
XP_018447492.1/1-283 -DVF-LL------QMCSGVDENAV-ESCSELIFAPIDASFS---------------D---
XP_019415259.1/1-283 -DVF-LL------QLCSGVDEHAA-GTSAELIFAPIDASLS---------------D---
XP_022980940.1/1-286 -DIF-LL------QLCNGVEENAV-GTSAELIFAPIDASFS---------------D---
XP_023523539.1/1-286 -DIF-LL------QLCNGVEENAV-GTSAELIFAPIDASFS---------------D---
XP_022941184.1/1-286 -DIF-LL------QLCNGVEENAV-GTSAELIFAPIDASFS---------------D---
XP_022134366.1/1-286 -DIF-LL------QLCNGVDENAV-GTSAELIFAPIDASFS---------------D---
XP_004136224.1/1-286 -DIF-LL------QLCNGVDENTV-GTSAELIFAPIDASFS---------------D---
XP_008466070.1/1-286 -DIF-LL------QLCNGVDENAV-GTSAELIFAPIDASFS---------------D---
XP_022976315.1/1-286 -DIF-LL------QLCNGVDENTV-GTSAELIFAPIDASFS---------------D---
XP_022937345.1/1-286 -DIF-LL------QLCNGVDENTV-GTSAELIFAPIDASFS---------------D---
XP_023534956.1/1-286 -DIF-LL------QLCNGVDENTV-GTSAELIFAPIDASFS---------------D---
XP_023535295.1/1-286 -DIF-LL------QLCNGVDENTV-GTSAELIFAPIDASFS---------------D---
XP_019175908.1/1-285 -DIF-LL------QLCSGVDENAV-GTCAELISAPIDASFA---------------D---
XP_019175907.1/1-285 -DIF-LL------QLCSGVDENAV-GTCAELISAPIDASFA---------------D---
XP_009626659.1/1-285 -DIF-LL------QLCSGVDENAV-GTCAELVFAPIDASFA---------------D---
XP_019266312.1/1-285 -DIF-LL------QLCSGVDENAV-GTCAELVFAPIDASFA---------------D---
XP_009781184.1/1-285 -DIF-LL------QLCSGVDENAV-GTCAELVFAPIDASFA---------------D---
XP_016488110.1/1-285 -DIF-LL------QLCSGVDENAV-GTCAELVFAPIDASFA---------------D---
XP_016537761.1/1-285 -DIF-LL------QLCNGVDDNAV-GTCAELMFAPIDASFA---------------D---
XP_006359741.1/1-285 -DIF-LL------QLCNGVDENAV-GSCAELMFAPIDASFA---------------D---
XP_010325078.1/1-288 -DIF-LL------QLCNGVDENAI-GTCAELMFAPIDASFA---------------D---
XP_004245155.1/1-285 -DIF-LL------QLCNGVDENAI-GTCAELMFAPIDASFA---------------D---
XP_015085523.1/1-285 -DIF-LL------QLCNGVDENAI-GTCAELMFAPIDASFA---------------D---
XP_022868182.1/1-285 -DIF-FV------QVCSGVDENAV-GSCAELIFAPIDASLS---------------D---
XP_022868181.1/1-285 -DIF-FV------QVCSGVDENAV-GSCAELIFAPIDASLS---------------D---
XP_022854559.1/1-285 -DIF-FL------QVCSGVDEKAV-GSCAELIFAPIDASFS---------------D---
XP_011081776.1/1-285 -DIF-LL------QVCNGVDESAV-GTCAELIFAPIDASFS---------------D---
XP_012857948.1/1-282 -DIF-LL------QVCNGVDENAM-GTCAELIFAPIDASFS---------------D---
XP_021847759.1/1-284 -DIF-ML------QLCSGVDENAV-GACAELIFAPIDASFS---------------D---
XP_010668539.1/1-284 -DIF-LL------QLCSGVDENAV-GACAELVFAPIDASFS---------------D---
XP_021739137.1/1-284 -DIF-ML------QLCSGVDENAV-GACAELVFAPIDASFS---------------D---
XP_021737291.1/1-284 -DIF-ML------QLCSGVDENAV-GACAELVFAPIDASFS---------------D---
XP_010047286.1/1-285 -DIF-LL------QLCNGVDENAV-GTCAELLFAPIDASFS---------------D---
XP_003603630.2/1-285 -DIF-LL------QLCSGVDEHAV-GTSAELIFAPIDASFS---------------D---
XP_004501146.1/1-286 -DIF-LL------QLCSGVDEHAV-GTSAELIFAPIDASFS---------------D---
XP_012571693.1/1-286 -DIF-LL------QLCSGVDEHAV-GTSAELIFAPIDASFS---------------D---
XP_012571692.1/1-286 -DIF-LL------QLCSGVDEHAV-GTSAELIFAPIDASFS---------------D---
XP_019417530.1/1-285 -DVF-LL------QLCSGVDEHGV-GTSAELVFAPIDASFS---------------D---
XP_019417531.1/1-285 -DVF-LL------QLCSGVDEHGV-GTSAELVFAPIDASFS---------------D---
XP_016180268.1/1-286 -DVF-LL------QLCSGVDENAV-GTSAELIFAPIDASFS---------------D---
XP_025623723.1/1-286 -DVF-LL------QLCSGVDENAV-GTSAELIFAPIDASFS---------------D---
XP_015945672.1/1-286 -DVF-LL------QLCSGVDENAV-GTSAELIFAPIDASFS---------------D---
XP_007137231.1/1-287 -DVF-LL------QLCSGVDEHAV-GTSAELVFAPIDASFS---------------D---
XP_022636494.1/1-293 -DVF-LLQCRKCSQLCSGVDEHAV-GTSAELVFAPIDASFS---------------D---
XP_017409217.1/1-287 -DVF-LL------QLCSGVDEHAV-GTSAELVFAPIDASFS---------------D---
XP_014501941.1/1-287 -DVF-LL------QLCSGVDEHAV-GTSAELVFAPIDASFS---------------D---
XP_003526496.1/1-286 -DVF-LL------QLCSGVDEHAV-GTSAELVFAPIDASFS---------------D---
XP_003522716.1/1-286 -DVF-LL------QLCSGVDEHAV-GTSAELVFAPIDASFS---------------D---
XP_020217689.1/1-286 -DVF-LL------QLCSGVDEHAV-GTSAELVFAPIDASFS---------------D---
XP_016683069.1/1-283 -DIF-LL------QLCTGVDENAV-STCAELIVAPIDGSFS---------------E---
XP_016677926.1/1-283 -DIF-LL------QLCTGVDENAV-STCAELIFAPIDGSFS---------------E---
XP_017643110.1/1-283 -DIF-LL------QLCTGVDENAV-STCAELIFAPIDGSFS---------------E---
XP_017643111.1/1-283 -DIF-LL------QLCTGVDENAV-STCAELIFAPIDGSFS---------------E---
XP_012451775.1/1-283 -DIF-LL------QLCTGVDENAV-STCAELIFAPIDGSFS---------------E---
XP_016744766.1/1-285 -DIF-LL------QLCSGVDENAV-GSCAELIFAPIDASFS---------------D---
XP_017643921.1/1-285 -DIF-LL------QLCSGVDENAV-GSCAELIFAPIDASFS---------------D---
XP_012436119.1/1-285 -DIF-LL------QLCSGVDENAV-GSCAELIFAPIDASFS---------------D---
XP_016681632.1/1-285 -DIF-LL------QLCSGVDENAV-GSCAELIFAPIDASFS---------------D---
XP_016689618.1/1-285 -DIF-LL------QLCSGVDENAV-GTCAELTFAPIDASFS---------------D---
XP_016689617.1/1-285 -DIF-LL------QLCSGVDENAV-GTCAELTFAPIDASFS---------------D---
XP_012445947.1/1-285 -DIF-LL------QLCSGVDENAV-GTCAELTFAPIDASFS---------------D---
XP_012445944.1/1-285 -DIF-LL------QLCSGVDENAV-GTCAELTFAPIDASFS---------------D---
XP_012445945.1/1-285 -DIF-LL------QLCSGVDENAV-GTCAELTFAPIDASFS---------------D---
XP_017614652.1/1-285 -DIF-LL------QLCSGVDENAV-GTCAELIFAPIDASFS---------------D---
XP_016732732.1/1-285 -DIF-LL------QLCSGVDENAV-GACAELIFAPIDASFS---------------D---
XP_016732731.1/1-285 -DIF-LL------QLCSGVDENAV-GACAELIFAPIDASFS---------------D---
XP_022754596.1/1-285 -DIF-LL------QLCSGVDENAA-GSCAELIFAPIDVSFS---------------D---
XP_022750159.1/1-285 -DIF-LL------QLCSGVDENAV-GTCAELIFAPIDASFS---------------D---
XP_022729737.1/1-285 -DIF-LL------QLCSGVDENAV-GTCAELIFAPIDASFS---------------D---
XP_022729738.1/1-285 -DIF-LL------QLCSGVDENAV-GTCAELIFAPIDASFS---------------D---
XP_021276132.1/1-285 -DIF-LL------QLCSGVDENAV-GTCAELIFAPIDASFS---------------D---
XP_017983822.1/1-285 -DIF-LL------QLCSGVDENAV-GTCAELIFAPIDASFS---------------D---
XP_002283717.3/1-285 -DVF-LL------QLCSGVDDNAV-GTCSELIFAPIDASFS---------------D---
XP_024446213.1/1-284 -DVF-LL------QLCSGVDENAV-GTCAELIFAPIDASFS---------------D---
XP_024446215.1/1-284 -DVF-LL------QLCSGVDENAV-GTCAELIFAPIDASFS---------------D---
XP_011006428.1/1-284 -DVF-LL------QLCSGVDENAV-GTCAELIFAPIDASFS---------------D---
XP_024459734.1/1-284 -DVF-LL------QLCSGVDENAV-GTCAELIFAPIDASFS---------------D---
XP_011019595.1/1-284 -DVF-LL------QLCSGVDENAV-GTCAELIFAPIDASFS---------------D---
XP_015573174.1/1-284 -DIF-LL------QLCSGVDENAV-GTCAELVFAPIDASFS---------------D---
XP_012077376.1/1-284 -DIF-LL------QLCSGVDENSI-GTCAELIFAPIDASFS---------------D---
XP_021608403.1/1-284 -DIF-LL------QFCTGVDENAV-GTCAELIFAPIDASFS---------------D---
XP_021608402.1/1-284 -DIF-LL------QFCTGVDENAV-GTCAELIFAPIDASFS---------------D---
XP_021597909.1/1-284 -DIF-LL------QLCSGVDENAV-GTCAELIFAPIDASFS---------------D---
XP_021642839.1/1-284 -DIF-LL------QLCSGVDENAV-GTCAELIFAPIDASFS---------------D---
XP_021689146.1/1-284 -DIF-LL------QLCSGVDENAV-GTCAELIFAPIDASFS---------------D---
XP_018805752.1/1-285 -DIF-LL------QLCSGVDENAA-GTCAELIFAPIDSSFS---------------D---
XP_023918167.1/1-285 -DIF-LL------QLCSGVDENAV-GNCAELIFAPIDASFS---------------D---
XP_018845499.1/1-285 -DIF-LL------QLCSGVDESAV-GTCAELIFAPIDASFS---------------D---
XP_018845500.1/1-285 -DIF-LL------QLCSGVDESAV-GTCAELIFAPIDASFS---------------D---
NP_001280750.1/1-285 ADIF-LL------QLCSGVDENAV-GTCAELVFAPIDASFS---------------D---
NP_001280796.1/1-285 ADIF-LL------QLCSGVDENAV-GTCSELVFAPIDASFS---------------D---
XP_009367440.1/1-285 ADIF-LL------QLCSGVDENAV-GTCSELVFAPIDASFS---------------D---
XP_017182108.1/1-285 ADIF-LL------QLCSGVDENAV-GTCSELVFAPIDASFS---------------D---
XP_004291167.1/1-285 ADIF-LL------QLCSGVDENAV-GTCAELVFAPIDASFS---------------D---
XP_024162655.1/1-285 ADIF-LL------QLCSGVDENAV-GTCAELVFAPIDASFS---------------D---
XP_021819449.1/1-284 ADIF-LL------QLCSGVDENSV-GTCAELVFAPIDASFS---------------D---
XP_007225281.1/1-285 ADIF-LL------QLCSGVDENSV-GTCAELVFAPIDASFS---------------D---
XP_008220092.1/1-285 ADIF-LL------QLCSGVDENSV-GTCAELVFAPIDASFS---------------D---
XP_006450706.1/1-285 -DIF-LL------QLCSGVDENAV-GNCAELVFAPIDASFS---------------D---
XP_006476026.1/1-285 -DIF-LL------QLCSGVDENAV-GNCAELVFAPIDASFS---------------D---
XP_024930942.1/1-285 -DIF-LL------QLCSGVDENAI-GTCAELVFAPIDASFS---------------D---
XP_010102406.2/1-285 -DIF-LL------QLCSGVDENAV-GTCAELIFAPIDASFS---------------D---
XP_024540686.1/1-258 -EIY-LL------QLCSGIEDEDI-DGCSQLIFAPVDANLS---------------D---
XP_024399220.1/1-283 -DSF-LL------QLCSGIEEGAV-GAGAQLVFAPIDAAVS---------------E---
XP_024399219.1/1-283 -DSF-LL------QLCSGIEEGAV-GAGAQLVFAPIDAAVS---------------E---
XP_024399215.1/1-283 -DSF-LL------QLCSGIEEGAV-GAGAQLVFAPIDAAVS---------------E---
XP_024390746.1/1-284 -DTF-LL------QLCSGVDESAV-GACAQLVFAPVDVALA---------------D---
XP_024390752.1/1-284 -DTF-LL------QLCSGVDESAV-GACAQLVFAPVDVALA---------------D---
XP_024373354.1/1-283 -DIF-LL------QLCSGIDESAL-GACSQLIFAPVDVALA---------------D---
XP_024373357.1/1-283 -DIF-LL------QLCSGIDESAL-GACSQLIFAPVDVALA---------------D---
XP_024370817.1/1-284 -DSF-LL------QLCSGIDENAV-GASAQLIFAPVDVALA---------------E---
XP_024370815.1/1-284 -DSF-LL------QLCSGIDENAV-GASAQLIFAPVDVALA---------------E---
XP_024370814.1/1-284 -DSF-LL------QLCSGIDENAV-GASAQLIFAPVDVALA---------------E---
XP_024386785.1/1-285 -DSF-LL------QLCSGIDENAV-GASAQLIFAPVDVALT---------------E---
XP_024386782.1/1-285 -DSF-LL------QLCSGIDENAV-GASAQLIFAPVDVALT---------------E---
XP_024386791.1/1-285 -DSF-LL------QLCSGIDENAV-GASAQLIFAPVDVALT---------------E---
XP_002961227.1/1-277 -ETV-LL------QLCSGHDDNAM-GVCAQLVFAPVDAAVS---------------E---
XP_002969458.1/1-277 -ETV-LL------QLCSGHDDNAT-GVCAQLVFAPVDAAVS---------------E---
XP_024533017.1/1-265 -DTV-LL------QLCTGYEDTAS-GTCAQLVFAPVDPAVS---------------N---
XP_011076983.1/1-261 -DLY-HL------QICNGMEDSGV-GACSELIFAPIDRTIT---------------N---
XP_011076980.1/1-264 -DLY-HL------QICNGMEDSGV-GACSELIFAPIDRTIT---------------N---
XP_011076981.1/1-263 -DLY-HL------QICNGMEDSGV-GACSELIFAPIDRTIT---------------N---
XP_011084330.1/1-264 -DLY-HL------QVITGMDDTGF-GACSELIFAPIDRTIP---------------N---
XP_011084333.1/1-264 -DLY-HL------QVITGMDDTGF-GACSELIFAPIDRTIP---------------N---
XP_011084331.1/1-264 -DLY-HL------QVITGMDDTGF-GACSELIFAPIDRTIP---------------N---
XP_012834933.1/1-260 -DIY-HL------QMSNGMEDNGF-GACSELIFAPVDRTIP---------------D---
XP_012834932.1/1-260 -DIY-HL------QMSNGMEDNGF-GACSELIFAPVDRTIP---------------D---
XP_012834931.1/1-260 -DIY-HL------QMSNGMEDNGF-GACSELIFAPVDRTIP---------------D---
XP_012834934.1/1-260 -DIY-HL------QMSNGMEDNGF-GACSELIFAPVDRTIP---------------D---
XP_002274194.1/1-284 -NIH-LL------QICNGVDDNAG-EACSELVFSPIDEMFP---------------D---
XP_019079956.1/1-284 -NIH-LL------QICNGVDDNAG-EACSELVFSPIDEMFP---------------D---
XP_023759583.1/1-283 -DIF-FL------QLCNGVDENAI-GTSAELVFAPIDASFT---------------D---
XP_020087984.1/1-275 -ELL-LL------QLCTGLDENAV-GTWSELIFAPIDASLA---------------D---
XP_015693848.1/1-289 -DLF-LL------QMYNGVEESTA-GTCSELIFAPIDASFS---------------D---
XP_015624690.1/1-290 -DLF-LL------QMYNGVEESSA-GTCSELIFAPIDASFS---------------D---
XP_008655743.1/1-292 -DLF-LL------QMYNGVDENMA-GTCSELIFAPIDASFS---------------D---
XP_020183693.1/1-281 -DLF-LL------QMYNGIDENTM-GTCSELIFAPIDASFG---------------D---
XP_020183687.1/1-281 -DLF-LL------QMYNGIDENTM-GTCSELIFAPIDASFG---------------D---
XP_003565084.1/1-284 -DLF-LL------QMYNGVDENTV-GSCSELIFAPIDASFS---------------D---
XP_002454995.1/1-286 -DLF-LL------QMYNGVDENMV-GTCSELIFAPIDASFS---------------D---
XP_012701181.1/1-286 -DLF-LL------QMYNGVDENTV-GTCSELIFAPIDASFS---------------D---
XP_012701180.1/1-286 -DLF-LL------QMYNGVDENTV-GTCSELIFAPIDASFS---------------D---
XP_012701179.1/1-286 -DLF-LL------QMYNGVDENTV-GTCSELIFAPIDASFS---------------D---
XP_025816436.1/1-287 -DLF-LL------QMYNGVDENTV-GTCSELIFAPIDASFS---------------D---
XP_018726848.1/1-227 SRML-ML------QE-SCT-DS-A-G--SFVIFAPVARFAM-NE----VLN-GM--D---
XP_018726849.1/1-227 SRML-ML------QE-SCT-DS-A-G--SFVIFAPVARFAM-NE----VLN-GM--D---
XP_019193052.1/1-216 SNML-IL------QE-SST-DP-T-A--SFVIYAPVDIVAM-NG----IMG-GG--D---
XP_019193050.1/1-216 SNML-IL------QE-SST-DP-T-A--SFVIYAPVDIVAM-NG----IMG-GG--D---
XP_021834047.1/1-194 SNML-IL------QA-SCT-DQ-T-A--SFVIYAPVDIVAM-NV----VLN-GS--D---
XP_010522330.1/1-229 SNML-IL------QE-SCT-DA-T-A--SFVIYAPVDIVAM-NV----VLN-GG--D---
XP_009802524.1/1-219 NNML-IL------QE-SST-DP-T-G--SFVVYAPVDVPAM-NT----VIN-GG--D---
XP_009802526.1/1-219 NNML-IL------QE-SST-DP-T-G--SFVVYAPVDVPAM-NT----VIN-GG--D---
XP_009802525.1/1-219 NNML-IL------QE-SST-DP-T-G--SFVVYAPVDVPAM-NT----VIN-GG--D---
XP_009802527.1/1-219 NNML-IL------QE-SST-DP-T-G--SFVVYAPVDVPAM-NT----VIN-GG--D---
XP_019250148.1/1-219 NNML-IL------QE-SSR-DP-T-G--SFVVYAPVDVPAM-NT----VIN-GG--D---
XP_019250149.1/1-219 NNML-IL------QE-SSR-DP-T-G--SFVVYAPVDVPAM-NT----VIN-GG--D---
XP_016449923.1/1-219 NNML-IL------QE-SST-DP-T-G--SFVVYAPVDVPAM-NT----VIN-GG--D---
XP_009629760.1/1-219 NNML-IL------QE-SST-DP-T-G--SFVVYAPVDVPAM-NT----VIN-GG--D---
XP_009629762.1/1-219 NNML-IL------QE-SST-DP-T-G--SFVVYAPVDVPAM-NT----VIN-GG--D---
XP_010234287.2/1-230 SNML-IL------QE-SST-DE-E-GGWSYVVYAPVDVVAM-NV----VLN-GG--D---
XP_021806177.1/1-204 SNML-IL------QE-SCT-DQ-T-A--SFVIYVPVDIVAM-NV----VLN-GS--D---
XP_016580642.1/1-229 SNML-IL------QE-CST-DL-T-G--SFVIYAPIDINSM-NV----VLN-GG--D---
XP_008667763.1/1-266 SNML-IL------QE-SCT-DA-S-G--SYVIYAPVDVVAM-NV----VLN-GG--D---
XP_006352040.1/1-227 SNMI-IL------QE-SST-DP-T-G--SYVIYAPVDISAM-NG----VLN-GG--D---
XP_009611638.1/1-223 SNML-IL------QE-SST-DP-T-N--SYVVYAPVDIGAM-NI----VLG-GG--D---
XP_009611623.1/1-223 SNML-IL------QE-SST-DP-T-N--SYVVYAPVDIGAM-NI----VLG-GG--D---
XP_016510654.1/1-223 SNML-IL------QE-SST-DP-T-N--SYVIYAPVDIGAM-NI----VLG-GG--D---
XP_009766038.1/1-223 SNML-IL------QE-SST-DP-T-N--SYVIYAPVDIAAM-NI----VLG-GG--D---
XP_020210648.1/1-236 SNMI-IL------QE-CWS-DA-A-G--SYVVYAPVDIVAM-NL----VLG-GG--N---
XP_007143210.1/1-219 SNMI-IL------QE-SCT-DA-T-G--SFVVYAPVDLMSM-NA----VLK-GG--N---
XP_014491869.1/1-226 SNMT-ML------QE-TCT-DA-T-G--SFVVYAPVDLISM-NA----VLR-GG--N---
XP_022633652.1/1-226 SNMT-ML------QE-TCT-DA-T-G--SFVVYAPVDLISM-NA----VLR-GG--N---
XP_017412341.1/1-227 SNMT-ML------QE-TCT-DA-T-G--SFVVYAPVDLISM-NA----VLR-GG--N---
XP_022011155.1/1-226 HNMI-ML------QE-SLT-DS-T-G--SYVVYAPIDVASL-NV----ILS-GG--D---
XP_003555330.2/1-228 NNML-IL------QE-SFI-DA-T-G--SFVIYAPIDVAAI-NV----VLG-GG--N---
XP_014628518.1/1-229 NNML-IL------QE-SFI-DA-T-G--SFVIYAPIDVAAI-NV----VLG-GG--N---
XP_017647009.1/1-225 SNML-IL------QE-SSN-DA-T-G--SYVIYAPVDFAAM-NI----VLN-GG--D---
XP_016711277.1/1-225 SNML-IL------QE-SSN-DA-T-G--SYVIYAPVDFAAM-NI----VLN-GG--D---
XP_012457027.1/1-225 SNML-IL------QE-SSN-DA-T-G--SYVIYAPVDFAAM-NI----VLT-GG--D---
XP_016723454.1/1-225 SNML-IL------QE-SSN-DA-T-G--SYVIYAPVDFAAM-NI----VLT-GG--D---
XP_020978973.1/1-214 SNML-IL------QE-SCTD-C-S-G--SYVVYAPVDMAAM-NI----ILS-GG--D---
XP_016202355.1/1-214 SNML-IL------QE-SCTD-C-S-G--SYVVYAPVDMAAM-NI----ILS-GG--D---
XP_015964876.1/1-214 SNML-IL------QE-SCTD-C-S-G--SYVVYAPVDMAAM-NI----ILS-GG--D---
XP_025699645.1/1-214 SNML-IL------QE-SCTD-C-S-G--SYVVYAPVDMAAM-NI----ILS-GG--D---
XP_008441375.1/1-228 SNML-IL------QE-SCSDDI-S-G--SYIIYAPVDTVAM-NM----VLS-GG--D---
XP_004154226.2/1-228 SNML-IL------QE-SCSDDI-S-G--SYIIYAPVDTAAM-NM----VLS-GG--D---
XP_023519981.1/1-226 SNML-IL------QE-SCS-DI-S-G--CYITYAPVDTVAM-NM----VLS-GG--D---
XP_023001231.1/1-226 SNML-IL------QE-SCS-DI-S-G--CYITYAPVDTVAM-NM----VLS-GG--D---
XP_022933178.1/1-226 SNML-IL------QE-SCS-DI-S-G--CYITYAPVDTVAM-NM----VLS-GG--D---
XP_004511340.1/1-228 SNML-IL------QE-SST-DI-T-G--SYVIYAPVDMAAM-NV----VLN-GG--D---
XP_002979015.2/1-232 SNML-IL------QE-SCT-DD-S-G--SLVVYAPVDIPAM-NL----VMQ-GG--D---
XP_002988402.1/1-232 SNML-IL------QE-SCT-DD-S-G--SLVVYAPVDIPAM-NL----VMQ-GG--D---
XP_024390340.1/1-238 SNML-IL------QE-SCT-DV-S-G--SLVIYAPVDIPAM-NL----VLQ-GG--D---
XP_024368074.1/1-235 SNML-IL------QE-SCT-DV-S-G--SLVIYAPVDIPAM-NL----VLQ-GG--D---
XP_024401280.1/1-235 SNML-IL------QE-SCT-DV-S-G--SLVIYAPVDIPAM-NL----VLQ-GG--D---
XP_024394840.1/1-235 SNML-IL------QE-SCT-DV-S-G--SLVIYAPVDIPAM-NL----VLQ-GG--D---
XP_024394838.1/1-235 SNML-IL------QE-SCT-DV-S-G--SLVIYAPVDIPAM-NL----VLQ-GG--D---
XP_017256650.1/1-227 STML-IL------QE-SYS-DP-T-A--SFIVYAPVDIMSM-NV----VIN-GG--D---
XP_019186546.1/1-227 GNMLMIL------QE-SCT-DS-T-G--CFVMYAPVDIVAM-RV----VLS-GG--D---
XP_024988375.1/1-226 SHML-IL------QE-SSC-DS-T-G--AYVIYAPVDIASM-NV----ALS-GA--D---
XP_017231769.1/1-226 SNML-IL------QE-SCA-DP-T-G--SYVIYAPVDMGAM-NL----VLS-GG--D---
XP_009108529.1/1-235 SNML-IL------QE-SCT-DA-S-G--SYVIYAPVDMMAM-NV----VLS-GG--D---
XP_018469534.1/1-236 SNML-IL------QE-SCT-DA-S-G--SYVIYAPVDVAAM-NV----VMN-GG--D---
XP_018467466.1/1-236 SNML-IL------QE-NCT-DA-S-G--SYVIYAPVDVAAM-NV----VMS-GG--D---
XP_022543253.1/1-234 SNML-IL------QE-SCT-DV-S-G--SYVIYAPVDMTAM-DV----VLS-GG--D---
XP_009135181.1/1-234 SNML-IL------QE-SCT-DV-S-G--SYVIYAPVDMTAM-DV----VLS-GG--D---
XP_013642975.1/1-234 SNML-IL------QE-SCT-DV-S-G--SYVIYAPVDMTAM-DV----VLS-GG--D---
XP_009135189.1/1-234 SNML-IL------QE-SCT-DV-S-G--SYVIYAPVDMTAM-DV----VLS-GG--D---
XP_018515487.1/1-234 SNML-IL------QE-SCT-DV-S-G--SYVIYAPVDMTAM-DV----VLS-GG--D---
XP_013623861.1/1-234 GNML-IL------QE-SCT-DV-S-G--SYVIYAPVDVAAM-DV----VLS-GG--D---
XP_013623905.1/1-234 GNML-IL------QE-SCT-DV-S-G--SYVIYAPVDVAAM-DV----VLS-GG--D---
XP_013623953.1/1-236 GNML-IL------QE-SCT-DV-S-G--SYVIYAPVDVAAM-DV----VLS-GG--D---
XP_013623587.1/1-236 GNML-IL------QE-SCT-DV-S-G--SYVIYAPVDVAAM-DV----VLS-GG--D---
XP_013623995.1/1-236 GNML-IL------QE-SCT-DV-S-G--SYVIYAPVDVAAM-DV----VLS-GG--D---
XP_013623820.1/1-236 GNML-IL------QE-SCT-DV-S-G--SYVIYAPVDVAAM-DV----VLS-GG--D---
XP_022926381.1/1-227 SNML-IL------QE-SCT-DS-T-G--SYVVYAPVDTVAM-NV----VLS-GC--D---
XP_023517485.1/1-227 SNML-IL------QE-SCT-DS-T-G--SYVVYAPVDTVAM-NV----VLS-GC--D---
XP_023002930.1/1-227 SNML-IL------QE-SCT-DS-T-G--SYVVYAPVDTVAM-NV----VLS-GC--D---
XP_004134971.1/1-229 SNML-IL------QE-SCT-DS-T-G--SYVIYAPVDTVAM-NV----VLS-GC--D---
XP_008439968.1/1-229 SNML-IL------QE-SCT-DS-T-G--SYVIYAPVDTVAM-NV----VLS-GC--D---
XP_022950176.1/1-227 SNML-IL------QE-SCT-DC-T-G--SYVIYAPVDTVAM-NV----VLS-GC--D---
XP_022979090.1/1-227 SNML-IL------QE-SCT-DS-T-G--SYVIYAPVDTVAM-NV----VLT-GC--D---
XP_023543388.1/1-249 SNML-IL------QE-SCT-DS-T-G--SYVIYAPVDTVAM-NV----VLS-GC--D---
XP_023543387.1/1-227 SNML-IL------QE-SCT-DS-T-G--SYVIYAPVDTVAM-NV----VLS-GC--D---
XP_009134323.1/1-228 SNML-IL------QE-SCT-DA-S-G--SYVIYAPVDIVAM-NV----VLS-GG--D---
XP_019176002.1/1-227 SNML-IL------QE-SCTDST----G-SYVIYAPVDIVAM-NV----VLS-GG--D---
XP_015070655.1/1-221 SNML-IL------QE-SSTDST----G-SYVIYAPVDIVAM-NV----VLS-GG--D---
XP_015070652.1/1-221 SNML-IL------QE-SSTDST----G-SYVIYAPVDIVAM-NV----VLS-GG--D---
XP_025885589.1/1-221 SNML-IL------QE-SSTDST----G-SYVIYAPVDIVAM-NV----VLS-GG--D---
XP_019422236.1/1-227 SNML-IL------QE-CCTDSS----G-SYVVYAPVDIVAM-NV----VLS-GG--D---
XP_007157166.1/1-226 SNML-IL------QE-SCTDST----G-SYVVYAPVDIVAM-NV----VLG-GG--D---
XP_003538765.1/1-227 SNML-IL------QE-SCTDST----G-SYVVYAPVDIVAM-NV----VLS-GG--D---
XP_003517589.1/1-227 SNML-IL------QE-SCTDST----G-SYVVYAPVDIVAM-NV----VLS-GG--D---
XP_006573890.1/1-227 SNML-IL------QE-SCTDST----G-SYVVYAPVDIVAM-NV----VLS-GG--D---
XP_011000687.1/1-226 SNML-IL------QE-SCTDST----G-SYVVYAPVDISAM-NI----VLS-GR--D---
XP_024436817.1/1-226 SNML-IL------QE-SCTDST----G-SYVIYAPVDISAM-NI----VLS-GG--D---
XP_024436820.1/1-226 SNML-IL------QE-SCTDST----G-SYVIYAPVDISAM-NI----VLS-GG--D---
XP_002518520.2/1-226 SNML-TL------QE-SCTDST----G-SYVIYAPVDIAAM-NI----VLS-GG--D---
XP_015574304.1/1-226 SNML-TL------QE-SCTDST----G-SYVIYAPVDIAAM-NI----VLS-GG--D---
XP_021642058.1/1-227 SNML-IL------QE-SCTDST----G-SYVIYAPVDISAM-NI----VLS-GG--D---
XP_012091395.1/1-226 SNML-IL------QE-SCTDST----G-SYVIYAPVDIAAM-NI----VLS-GG--D---
XP_021635824.1/1-226 SNML-IL------QE-SCTDST----G-SYVIYAPVDIAAM-NI----VLS-GG--D---
XP_021596887.1/1-226 SNML-IL------QE-SCTDST----G-SYVIYAPVDISAM-NI----VLS-GG--D---
XP_010057969.1/1-228 SNML-IL------QE-SCTDSV----G-AYVIYAPVDIVAM-NV----VLN-GG--D---
XP_006413738.1/1-245 SNML-IL------QE-SCTDAS----G-SYVIYAPVDTMAM-NV----VLS-GG--D---
XP_013630219.1/1-235 SNML-IL------QE-SCTDAS----G-SYVIYAPVDMMAM-NV----VLS-GG--D---
XP_013656136.1/1-235 SNML-IL------QE-SCTDAS----G-SYVIYAPVDMMAM-NV----VLS-GG--D---
XP_013685757.1/1-235 SNML-IL------QE-SCTDAS----G-SYVIYAPVDMMAM-NV----VLS-GG--D---
XP_013696499.1/1-240 SNML-IL------QE-SCTDAS----G-SYVIYAPVDIMAM-NV----VLS-GG--D---
XP_009137213.1/1-240 SNML-IL------QE-SCTDAS----G-SYVIYAPVDIMAM-NV----VLS-GG--D---
XP_013598423.1/1-241 SNML-IL------QE-SCTDAS----G-SYVIYAPVDIMAM-NV----VLS-GG--D---
XP_013717184.1/1-241 SNML-IL------QE-SCTDAS----G-SYVIYAPVDIMAM-NV----VLS-GG--D---
XP_018480642.1/1-241 SNML-IL------QE-SCTDAS----G-SYVIYAPVDIMAM-NV----VLS-GG--D---
XP_018477464.1/1-241 SNML-IL------QE-SCTDAS----G-SYVIYAPVDIMAM-NV----VLS-GG--D---
NP_193906.2/1-248    SNML-IL------QE-SCTDAS----G-SYVIYAPVDIIAM-NV----VLS-GG--D---
XP_020873301.1/1-248 SNML-IL------QE-SCTDAS----G-SYVIYAPVDILAM-NV----VLS-GG--D---
XP_006285476.1/1-249 SNML-IL------QE-SCTDAS----G-SYVIYAPVDIMAM-NV----VLS-GG--D---
XP_010434132.1/1-252 SNML-IL------QE-SCTDAS----G-SYVIYAPVDIMAM-NV----VLS-GG--D---
XP_010439429.1/1-252 SNML-IL------QE-SCTDAS----G-SYVIYAPVDIMAM-NV----VLS-GG--D---
XP_010449009.1/1-254 SNML-IL------QE-SCTDAS----G-SYVIYAPVDIMAM-NV----VLS-GG--D---
XP_010432053.1/1-234 SNML-IL------QE-SCTDAS----G-SYVIYAPVDIVAM-NV----VLS-GG--D---
XP_020876375.1/1-234 SNML-IL------QE-SCTDAS----G-SYVIYAPVDIVAM-NV----VLS-GG--D---
XP_023636874.1/1-234 SNML-IL------QE-SCTDAS----G-SYVIYAPVDIVAM-NV----VLS-GG--D---
XP_010422328.1/1-234 SNML-IL------QE-SCTDAS----G-SYVIYAPVDIVAM-NV----VLS-GG--D---
XP_010455798.1/1-234 SNML-IL------QE-SCTDAS----G-SYVIYAPVDIVAM-NV----VLS-GG--D---
NP_567274.1/1-235    SNML-IL------QE-SCTDAS----G-SYVIYAPVDIVAM-NV----VLS-GG--D---
XP_024009884.1/1-236 SNML-IL------QE-SCTDAS----G-SYVIYAPVDIVAM-NV----VLS-GG--D---
XP_018452346.1/1-236 SNML-IL------QE-SCTDAS----G-SYVIYAPVDIVAM-NV----VLS-GG--D---
XP_013611160.1/1-236 SNML-IL------QE-SCTDAS----G-SYVIYAPVDIVAM-NV----VLS-GG--D---
XP_009114536.1/1-235 SNML-IL------QE-SCTDAS----G-SYVIYAPVDIVAM-NV----VLS-GG--D---
XP_013662000.1/1-235 SNML-IL------QE-SCTDAS----G-SYVIYAPVDIVAM-NV----VLS-GG--D---
XP_018472664.1/1-233 SNML-IL------QE-SCTDAS----G-SYVIYAPVDIVAM-NV----VLS-GG--D---
XP_022555047.1/1-234 SNML-IL------QE-SCTDAS----G-SYVIYAPVDIVAM-NV----VLS-GG--D---
XP_013735469.1/1-234 SNML-IL------QE-SCTDAS----G-SYVIYAPVDIVAM-NV----VLS-GG--D---
XP_013629800.1/1-234 SNML-IL------QE-SCTDAS----G-SYVIYAPVDIVAM-NV----VLS-GG--D---
XP_013735464.1/1-234 SNML-IL------QE-SCTDAS----G-SYVIYAPVDIVAM-NV----VLS-GG--D---
XP_022571893.1/1-234 SNML-IL------QE-SCTDAS----G-SYVIYAPVDIVAM-NV----VLS-GG--D---
XP_010538642.1/1-236 SNML-IL------QE-SCTDAT----G-SYVIYAPVDIVAM-NV----VLS-GG--D---
XP_019057303.1/1-229 SNML-IL------QE-SCADAT----G-SYVIYAPVDIVAM-NV----VLS-GG--D---
XP_016545426.1/1-226 NNML-IL------QE-SCLDST----G-CYVIYAPVDIAAM-NV----VLS-GG--D---
XP_009793614.1/1-227 NNML-IL------QE-SCTDST----G-SYVIYAPVDIAAM-NV----VLS-GG--D---
XP_016508926.1/1-227 NNML-IL------QE-SCTDST----G-SYVIYAPVDIAAM-NV----VLS-GG--D---
XP_016560096.1/1-235 SNML-IL------QE-SSTDPT----G-SYVIYAPVDIVAM-NV----VLS-GG--D---
XP_021993721.1/1-227 GNML-IL------QE-SSNDST----G-SYVIYAPVDIAAM-NV----VLS-GG--D---
XP_022036928.1/1-226 SNML-IL------QE-SSSDPT----G-SYVIYAPVDIASM-NV----VLS-GG--D---
XP_023742870.1/1-226 SNML-IL------QE-SSTDST----G-SYVIYAPVDINAM-NV----ALN-GG--D---
XP_006352605.1/1-223 NNML-IL------QE-SCLDTT----G-SYVIYAPVDIAAM-NV----VLS-GG--D---
XP_010327237.1/1-223 NNML-IL------QE-SCLDIT----G-SYVIYAPVDIAAM-NV----VLS-GG--D---
XP_015055984.1/1-223 NNML-IL------QE-SCLDIT----G-SYVIYAPVDIAAM-NV----VLS-GG--D---
XP_009609606.1/1-229 NNML-IL------QE-SCTDST----G-SYVIYAPVDIAAM-NV----VLS-GG--D---
XP_009609608.1/1-229 NNML-IL------QE-SCTDST----G-SYVIYAPVDIAAM-NV----VLS-GG--D---
XP_018628742.1/1-229 NNML-IL------QE-SCTDST----G-SYVIYAPVDIAAM-NV----VLS-GG--D---
XP_019237831.1/1-229 NNML-IL------QE-SCTDST----G-SYVIYAPVDIAAM-NV----VLS-GG--D---
XP_018849836.1/1-227 SNML-IL------QE-SCTDST----G-SYVIYAPVDIVAM-NV----VLS-GG--D---
XP_007214541.1/1-230 SNML-IL------QE-SCTDST----G-SYVIYAPVDIVAM-NV----VLS-GG--D---
XP_021821565.1/1-230 SNML-IL------QE-SCTDST----G-SYVIYAPVDIVAM-NV----VLS-GG--D---
XP_008224792.1/1-230 SNML-IL------QE-SCTDST----G-SYVIYAPVDIVAM-NV----VLS-GG--D---
XP_024198966.1/1-227 SNML-IL------QE-SCTDST----G-SYVIYAPVDIVAM-NV----VLS-GG--D---
XP_004293268.1/1-227 SNML-IL------QE-SCTDST----G-SYVIYAPVDIVAM-NV----VLS-GG--D---
XP_009364098.1/1-227 SNML-IL------QE-SCTDST----G-SYVIYAPVDIVAM-NV----VLS-GG--D---
XP_008371114.1/1-227 SNML-IL------QE-SCTDST----G-SYVIYAPVDIVAM-NV----VLS-GG--D---
XP_008384034.1/1-227 SNML-IL------QE-SCTDST----G-SYVIYAPVDIVAM-NV----VLS-GG--D---
XP_009340946.1/1-227 SNML-IL------QE-SCTDST----G-SYVIYAPVDIVAM-NV----VLS-GG--D---
XP_009357415.1/1-227 SNML-IL------QE-SCTDST----G-SYVIYAPVDIVAM-NV----VLS-GG--D---
XP_011037708.1/1-226 SNML-IL------QE-SCTDST----G-SYVIYAPVDISAM-NI----VLS-GG--D---
XP_024454819.1/1-226 SNML-IL------QE-SCTDST----G-SYVIYAPVDISAM-NI----VLS-GG--D---
XP_015884145.1/1-226 SNML-IL------QE-SCTDRT----G-SYVIYAPVDIVAM-NM----VLS-GG--D---
XP_024024693.1/1-228 SNML-IL------QE-SCTDST----G-SYVIYAPVDIVAM-NV----VLS-GG--D---
XP_024024692.1/1-228 SNML-IL------QE-SCTDST----G-SYVIYAPVDIVAM-NV----VLS-GG--D---
XP_018820351.1/1-226 SNML-IL------QE-SCTDSI----G-SYVIYAPVDIVAM-NV----VLS-GG--D---
XP_023904570.1/1-226 SNML-IL------QE-SCTDST----G-SYVIYAPVDIVAM-NM----VLS-GG--D---
XP_023904569.1/1-226 SNML-IL------QE-SCTDST----G-SYVIYAPVDIVAM-NM----VLS-GG--D---
XP_006449076.1/1-226 SNML-VL------QE-SCTDST----G-SYVIYAPVDIVAM-NM----VLS-GG--D---
XP_006468235.1/1-226 SNML-VL------QE-SCTDST----G-SYVIYAPVDIVAM-NM----VLS-GG--D---
XP_022721034.1/1-226 SNML-IL------QE-SCSDVT----G-SYVIYAPVDIISM-NV----VLS-GG--D---
XP_022721035.1/1-226 SNML-IL------QE-SCSDVT----G-SYVIYAPVDIISM-NV----VLS-GG--D---
XP_022724746.1/1-226 SNML-IL------QE-SCSDAT----G-SYVIYAPVDIAAM-NV----VLS-GG--D---
XP_007026002.1/1-226 SNML-IL------QE-SCSDAT----G-SYVIYAPVDIVAM-NV----VLS-GG--D---
XP_021294392.1/1-226 SNML-IL------QE-SCSDAT----G-SYVIYAPVDIVAM-NV----VLS-GG--D---
XP_022777405.1/1-226 SNML-IL------QE-SCSDAT----G-SYVIYAPVDIGAM-NV----VLS-GG--D---
XP_017615807.1/1-226 SNML-IL------QE-SCTDAT----G-SYVIYAPVDIVAM-NV----VLS-GG--D---
XP_016753649.1/1-226 SNML-IL------QE-SCTDAT----G-SYVIYAPVDIVAM-NV----VLS-GG--D---
XP_016683607.1/1-226 SNML-IL------QE-SCTDAK----G-SYVIYAPVNIVAM-NI----VLS-GG--D---
XP_012451363.1/1-226 SNML-IL------QE-SCTDAT----G-SYVIYAPVDIVAM-NV----VLS-GG--D---
XP_002266688.1/1-226 SNML-IL------QE-SCTDPT----G-SYVIYAPVDIVAM-NV----VLS-GG--D---
XP_021853797.1/1-224 SNML-IL------QE-SSTDST----G-SYVIYAPVDIVAM-NV----VLN-GG--D---
XP_021719656.1/1-227 SNML-IL------QE-SSTDPT----G-SYVIYAPVDIVAM-NV----VLN-GG--D---
XP_021766927.1/1-227 SNML-IL------QE-SSTDPT----G-SYVIYAPVDIVAM-NV----VLN-GG--D---
XP_010665524.1/1-227 SNML-IL------QE-SSTDST----G-SYVIYAPVDIVAM-NV----VLN-GG--D---
XP_009605864.1/1-234 SNML-IL------QE-SSTDST----G-SYVIYAPVDIVAM-NV----VLS-GG--D---
XP_019223778.1/1-233 SNML-IL------QE-SSTDST----G-SYVIYAPVDIVAM-NV----VLS-GG--D---
XP_016440385.1/1-233 SNML-IL------QE-SSTDST----G-SYVIYAPVDIVAM-NV----VLS-GG--D---
XP_009790281.1/1-233 SNML-IL------QE-SSTDST----G-SYVIYAPVDIVAM-NV----VLS-GG--D---
XP_016570434.1/1-222 SNML-IL------QE-SSTDST----G-SYVIYAPVDIVAM-NV----VLS-GG--D---
XP_016486479.1/1-229 SNML-IL------QE-SSTDST----G-SYVIYAPVDIVAM-NV----VLS-GG--D---
XP_009597904.1/1-229 SNML-IL------QE-SSTDST----G-SYVIYAPVDIVAM-NV----VLS-GG--D---
XP_009799795.1/1-229 SNML-IL------QE-SSTDST----G-SYVIYAPVDIVAM-NV----VLS-GG--D---
XP_019249652.1/1-229 SNML-IL------QE-SSTDST----G-SYVIYAPVDIVAM-NV----VLS-GG--D---
XP_006343296.1/1-221 SNML-IL------QE-SSTDST----G-SYVIYAPVDIVAM-NV----VLS-GG--D---
XP_020217012.1/1-226 SNML-IL------QE-SSTDST----G-SYVVYAPVDIVAM-NV----VLS-GG--D---
XP_017406677.1/1-226 SNML-IL------QE-SCTDST----G-SYVVYAPVDIVAM-NV----VLG-GG--D---
XP_014519998.1/1-226 SNML-IL------QE-SCTDST----G-SYVVYAPVDIVAM-NV----VLG-GG--D---
XP_014519997.1/1-226 SNML-IL------QE-SCTDST----G-SYVVYAPVDIVAM-NV----VLG-GG--D---
XP_012841999.1/1-239 SNML-IL------QE-SCSDST----G-SFVVYAPVDIIAM-NV----VLS-GG--D---
XP_024984347.1/1-227 GNML-IL------QE-SSNDST----G-SYVIYAPVDIAAM-NV----VLS-GG--D---
XP_023751202.1/1-227 GNML-IL------QE-SSNDST----G-SYVIYAPVDIAAM-NV----VLS-GG--D---
XP_023751208.1/1-227 GNML-IL------QE-SSNDST----G-SYVIYAPVDIAAM-NV----VLS-GG--D---
XP_022041933.1/1-227 GNML-IL------QE-SSNDST----G-SYVIYAPVDIAAM-NV----VLS-GG--D---
XP_024988632.1/1-226 SNML-IL------QE-SSSDST----G-SYVIYAPVDIVAM-NV----VLS-GG--D---
XP_022018253.1/1-226 SNML-IL------QE-SSSDST----G-SYVIYAPVDIQAM-NV----VLS-GG--D---
XP_023771293.1/1-226 SNML-IL------QE-SSSDST----G-SYVIYAPVDIVAM-NV----VLS-GG--D---
XP_019170324.1/1-226 SNML-IL------QE-SCADST----G-SFVIYAPVDIVAM-NV----VLS-GG--D---
XP_017230122.1/1-226 SNML-IL------QE-SCADST----G-SYVIYAPVDIVAM-NV----VLS-GG--D---
XP_011096101.1/1-226 SNML-IL------QE-SSTDST----A-SYVIYAPVDIVAM-NV----VLS-GG--D---
XP_012848972.1/1-227 SNML-IL------QE-SSTDST----G-SYVIYAPVDIVAM-NV----VLS-GG--D---
XP_022876975.1/1-227 SNML-IL------QE-SCTDST----G-SYVIYAPVDIIAM-NV----VLS-GG--D---
XP_022863158.1/1-227 SNML-IL------QE-SCTDST----G-SYVIYAPVDIVAM-NV----VLS-GG--D---
XP_022877991.1/1-227 SNML-IL------QE-SCTDST----G-SYVIYAPVDIVAM-NV----VLS-GG--D---
XP_019223745.1/1-223 SNML-IL------QE-SSTDPT----N-SYVIYAPVDIAAM-NI----VLG-GG--D---
XP_021800220.1/1-221 SNML-IL------QE-SCTDKT----T-SFVIYAPVDIVAM-NV----VLI-GS--D---
XP_021684589.1/1-235 SNML-IL------QE-SCTDQT----A-SFVIYAPVDTVAM-NV----LLN-GG--D---
XP_021684519.1/1-235 SNML-IL------QE-SCTDQT----A-SFVIYAPVDTVAM-NV----LLN-GG--D---
XP_021684722.1/1-235 SNML-IL------QE-SCTDQT----A-SFVIYAPVDTVAM-NV----LLN-GG--D---
XP_021633498.1/1-230 SNML-IL------QE-SCTDPT----A-SFVIYAPVDVVSM-NV----VLN-GG--D---
XP_009420270.1/1-216 SNML-IL------QE-SCTDPV----V-SYVIYAPVDVVAM-NV----VLN-GG--D---
XP_003573403.1/1-232 SNML-IL------QE-SCTDES----GSSYVVYAPVDIVAM-NV----VLN-GG--D---
XP_006659894.1/1-229 SNML-IL------QE-SCTDAS----G-SYVVYAPVDIVAM-NV----VLN-GG--D---
XP_015650022.1/1-263 SNML-IL------QE-SCTDAS----G-SYVVYAPVDIVAM-NV----VLN-GG--D---
XP_015650023.1/1-230 SNML-IL------QE-SCTDAS----G-SYVVYAPVDIVAM-NV----VLN-GG--D---
XP_020407343.1/1-231 SNML-IL------QE-SCTDAS----G-SYVVYAPVDVVAM-NV----VLN-GG--D---
NP_001105127.2/1-231 SNML-IL------QE-SCTDAS----G-SYVVYAPVDVVAM-NV----VLN-GG--D---
XP_008661805.1/1-239 SNML-IL------QE-SCTDAS----G-SYVVYAPVDVVAM-NV----VLN-GG--D---
NP_001145750.1/1-239 SNML-IL------QE-SCTDAS----G-SYVVYAPVDVVAM-NV----VLN-GG--D---
XP_025821536.1/1-230 SNML-IL------QE-SCTDAS----G-SYVVYAPVDVVAM-NV----VLN-GG--D---
XP_002443965.1/1-229 SNML-IL------QE-SCTDAS----G-SYVVYAPVDVVAM-NV----VLN-GG--D---
XP_004972697.1/1-229 SNML-IL------QE-SCTDAS----G-SYVVYAPVDVVAM-NV----VLN-GG--D---
XP_008667765.1/1-230 SNML-IL------QE-SCTDAS----G-SYVIYAPVDVVAM-NV----VLN-GG--D---
NP_001347891.1/1-231 SNML-IL------QE-SCTDAS----G-SYVIYAPVDVVAM-NV----VLN-GG--D---
XP_015636340.1/1-229 SNML-IL------QE-SCTDAS----G-SYVIYAPVDVVAM-NV----VLN-GG--D---
XP_015636338.1/1-229 SNML-IL------QE-SCTDAS----G-SYVIYAPVDVVAM-NV----VLN-GG--D---
XP_006652859.1/1-228 SNML-IL------QE-SCTDAS----G-SYVIYAPVDVVAM-NV----VLN-GG--D---
XP_003580598.1/1-233 SNML-IL------QE-SCTDAS----G-SYVIYAPVDIVAM-NV----VLN-GG--D---
XP_020199198.1/1-227 SNML-IL------QE-SCTDAS----G-SYVIYAPVDVVAM-NV----VLN-GG--D---
XP_020400772.1/1-230 SSML-IL------QE-SCTDMS----G-SYVIYAPVDVVAM-NV----VLN-GG--D---
NP_001334663.1/1-230 SSML-IL------QE-SCTDMS----G-SYVIYAPVDVVAM-NV----VLN-GG--D---
XP_008662197.1/1-270 SSML-IL------QE-SCTDMS----G-SYVIYAPVDVVAM-NV----VLN-GG--D---
XP_008662196.1/1-270 SSML-IL------QE-SCTDMS----G-SYVIYAPVDVVAM-NV----VLN-GG--D---
XP_025823505.1/1-227 SNML-IL------QE-SCTDSS----G-SYVIYAPVDVVAM-NV----VLN-GG--D---
XP_004976894.1/1-227 SNML-IL------QE-SCTDAS----G-SYVIYAPVDVVAM-NV----VLN-GG--D---
XP_002448563.2/1-227 SNML-IL------QE-SCTDAS----G-SYVIYAPVDVVAM-NV----VLN-GG--D---
XP_019414154.1/1-227 GNMS-IL------QE-SCSDST----C-SFVIYAPVDIVAM-NV----VLN-GG--D---
XP_006348085.1/1-234 SNML-IL------QE-STTDIT----G-SYVIYAPVDIAAM-NV----VLG-GG--D---
XP_004232734.1/1-234 SNML-IL------QE-STTDVT----G-SYVIYAPVDIAAM-NV----VLG-GG--D---
XP_015066898.1/1-234 SNML-IL------QE-STTDVT----G-SYVIYAPVDIAAM-NV----VLG-GG--D---
XP_003605900.1/1-232 SNML-IL------QE-SVTDAT----G-SFVIYAPVDMVAM-NV----VLN-GG--D---
XP_022988693.1/1-229 SNML-IL------QE-NITDQT----A-AFVIYAPVDIVAI-NV----VLN-GG--D---
XP_022930609.1/1-229 SNML-IL------QE-NITDQT----A-AFVIYAPVDIVAI-NV----VLN-GG--D---
XP_023530197.1/1-229 SNML-IL------QE-NITDQT----A-AFVIYAPVDIVAI-NV----VLN-GG--D---
XP_016188415.1/1-237 SNML-IL------QE-SCTDST----G-SFVVYAPVDIVAM-NV----VLN-GG--D---
XP_011628991.1/1-236 SNML-IL------QE-SCTDSS----G-SYVIYAPVDIVAM-NV----VLN-GG--D---
XP_006826512.2/1-236 SNML-IL------QE-SCTDSS----G-SYVIYAPVDIVAM-NV----VLN-GG--D---
XP_020177398.1/1-220 SNML-IL------QE-CCTDTT----G-SYVVYAPVDVVAM-NV----VLN-GG--D---
XP_015695486.1/1-219 SNML-IL------QE-CCTDAT----G-SYVIYAPVDVVAM-NV----VLN-GG--D---
XP_015648539.1/1-217 SNML-IL------QE-CCTDAT----G-SYVIYAPVDVVAM-NV----VLN-GG--D---
XP_020401599.1/1-218 GNML-IL------QE-CCTDAT----G-SYVIYAPVDVVAM-NV----VLN-GG--D---
XP_020401598.1/1-218 GNML-IL------QE-CCTDAT----G-SYVIYAPVDVVAM-NV----VLN-GG--D---
XP_020401597.1/1-218 GNML-IL------QE-CCTDAT----G-SYVIYAPVDVVAM-NV----VLN-GG--D---
XP_021319954.1/1-239 SNML-IL------QE-CCTDAT----G-SYVIYAPVDVVAM-NV----VLN-GG--D---
XP_002445014.2/1-239 SNML-IL------QE-CCTDAT----G-SYVIYAPVDVVAM-NV----VLN-GG--D---
XP_025819913.1/1-218 SNML-IL------QE-CCTDAT----G-SYVIYAPVDVVAM-NV----VLN-GG--D---
XP_004972909.1/1-218 SNML-IL------QE-CCTDKT----G-SYVIYAPVDVVAM-NV----VLN-GG--D---
XP_020100432.1/1-229 SNML-IL------QE-SCTDAT----G-SYVIYAPVDVVAM-NV----VLN-GG--D---
XP_009385059.1/1-228 SNML-IL------QE-SCTDST----G-SYVIYAPVDVIAM-NV----VLN-GG--D---
XP_018678797.1/1-226 SNML-IL------QE-SCTDAT----G-SYVIYAPVDVIAM-NV----VLN-GG--D---
XP_009412012.1/1-233 SNML-IL------QE-SCTDAT----G-SYVIYAPVDVIAM-NV----VLN-GG--D---
XP_018685149.1/1-236 SNML-IL------QE-SCTDAT----G-SYVIYAPVDVIAM-NV----VLN-GG--D---
XP_020591278.1/1-230 SNML-IL------QE-SCTDPT----G-SYVIYAPVDVVAM-NV----VLN-GG--D---
XP_020702932.1/1-229 SNML-IL------QE-SCTDPT----G-SYVIYAPVDVVAM-NV----VLN-GG--D---
XP_020702933.1/1-230 SNML-IL------QE-SCTDPT----G-SYVIYAPVDVVAM-NV----VLN-GG--D---
XP_020702931.1/1-230 SNML-IL------QE-SCTDPT----G-SYVIYAPVDVVAM-NV----VLN-GG--D---
XP_020692781.1/1-229 SNML-IL------QE-SCTDMT----G-SYVIYAPVDVVAM-NV----VLN-GG--D---
XP_020692780.1/1-229 SNML-IL------QE-SCTDMT----G-SYVIYAPVDVVAM-NV----VLN-GG--D---
XP_020591591.1/1-229 SNML-IL------QE-SCTDLT----G-SYVIYAPVDVVAM-NV----VLN-GG--D---
XP_020591590.1/1-229 SNML-IL------QE-SCTDLT----G-SYVIYAPVDVVAM-NV----VLN-GG--D---
XP_020109359.1/1-227 SNML-IL------QE-SCTDPT----G-SYVIYAPVDVVAM-NV----VLN-GG--D---
XP_020270114.1/1-229 SNML-IL------QE-SCTDST----G-SYVIYAPVDVVAM-NV----VLN-GG--D---
XP_010908855.1/1-227 SNML-IL------QE-SCTDPT----G-SYVIYAPVDMVAM-NV----VLS-GG--D---
XP_008808987.1/1-227 SNML-IL------QE-SCTDPT----G-SYVIYAPVDMVAM-NV----VLS-GG--D---
XP_008784552.1/1-223 SNML-IL------QE-SCTDPT----G-SYVIYAPVDFAAM-NV----VLN-GG--D---
XP_010931820.1/1-223 SNML-IL------QE-SCTDPT----G-SYVIYAPVDFVAM-NV----VLN-GG--D---
XP_003540308.2/1-226 SNML-IL------QE-SCADST----G-SFVIYAPVDIVAM-NV----VLN-GG--D---
XP_006592818.1/1-226 SNML-IL------QE-SCADST----G-SFVIYAPVDIVAM-NV----VLN-GG--D---
XP_006592816.1/1-226 SNML-IL------QE-SCADST----G-SFVIYAPVDIVAM-NV----VLN-GG--D---
XP_003543387.1/1-226 SNML-IL------QE-SCADST----G-SFVIYAPVDIVAM-NV----VLN-GG--D---
XP_020223651.1/1-226 SNML-IL------QE-SCADST----G-SFVIYAPVDIVAM-NV----VLN-GG--D---
XP_020223648.1/1-226 SNML-IL------QE-SCADST----G-SFVIYAPVDIVAM-NV----VLN-GG--D---
XP_007149968.1/1-226 SNML-IL------QE-SCADST----G-SFVIYAPVDIVAM-NV----VLN-GG--D---
XP_017425582.1/1-226 SNML-IL------QE-SCADST----G-SFVIYAPVDIVAM-NV----VLN-GG--D---
XP_014501634.1/1-226 SNML-IL------QE-SCADST----G-SFVIYAPVDIVAM-NV----VLN-GG--D---
XP_022776632.1/1-226 SNML-IL------QE-SCLDPT----A-SFVIYAPVDIVAM-NV----VLN-GG--D---
XP_022776631.1/1-226 SNML-IL------QE-SCLDPT----A-SFVIYAPVDIVAM-NV----VLN-GG--D---
XP_022776629.1/1-226 SNML-IL------QE-SCLDPT----A-SFVIYAPVDIVAM-NV----VLN-GG--D---
XP_022733641.1/1-226 SNML-IL------QE-SCTDPT----A-SFVIYAPVDIVAM-NV----VLN-GG--D---
XP_022733640.1/1-226 SNML-IL------QE-SCTDPT----A-SFVIYAPVDIVAM-NV----VLN-GG--D---
XP_022733638.1/1-226 SNML-IL------QE-SCTDPT----A-SFVIYAPVDIVAM-NV----VLN-GG--D---
XP_007045612.1/1-226 SNML-IL------QE-SCADPT----A-SFVIYAPVDIVAM-NV----VLN-GG--D---
XP_007045611.1/1-226 SNML-IL------QE-SCADPT----A-SFVIYAPVDIVAM-NV----VLN-GG--D---
XP_017971478.1/1-226 SNML-IL------QE-SCADPT----A-SFVIYAPVDIVAM-NV----VLN-GG--D---
XP_021292769.1/1-226 SNML-IL------QE-SCADPT----A-SFVIYAPVDIVAM-NV----VLN-GG--D---
XP_021292767.1/1-226 SNML-IL------QE-SCADPT----A-SFVIYAPVDIVAM-NV----VLN-GG--D---
XP_021292765.1/1-226 SNML-IL------QE-SCADPT----A-SFVIYAPVDIVAM-NV----VLN-GG--D---
XP_010657309.1/1-226 SNML-IL------QE-SCTDST----A-SFVIYAPVDVVAM-NM----VLN-GG--D---
XP_010657313.1/1-226 SNML-IL------QE-SCTDST----A-SFVIYAPVDVVAM-NM----VLN-GG--D---
XP_010657310.1/1-226 SNML-IL------QE-SCTDST----A-SFVIYAPVDVVAM-NM----VLN-GG--D---
XP_010657311.1/1-226 SNML-IL------QE-SCTDST----A-SFVIYAPVDVVAM-NM----VLN-GG--D---
XP_012086347.1/1-228 SNML-IL------QE-SCTDPT----A-SFVIYAPVDIVAM-NV----VLN-GG--D---
XP_012086344.1/1-228 SNML-IL------QE-SCTDPT----A-SFVIYAPVDIVAM-NV----VLN-GG--D---
XP_024029967.1/1-226 SNML-IL------QE-SCTDPT----A-SFVIYAPVDIVAM-NV----VLN-GG--D---
XP_010109517.1/1-226 SNML-IL------QE-SCTDPT----A-SFVIYAPVDIVAM-NV----VLN-GG--D---
XP_006469697.1/1-226 SNML-IL------QE-SCTDPT----A-SFVIYAPVDIVAM-NV----VLN-GG--D---
XP_024046029.1/1-226 SNML-IL------QE-SCTDPT----A-SFVIYAPVDIVAM-NV----VLN-GG--D---
XP_020234199.1/1-226 SNML-IL------QE-SCTDTT----G-SFVIYAPVDIVAM-NV----VLN-GG--D---
XP_020234201.1/1-226 SNML-IL------QE-SCTDTT----G-SFVIYAPVDIVAM-NV----VLN-GG--D---
XP_021630856.1/1-226 SNML-IL------QE-SCTDPT----A-SFVIYAPVDIVAM-NV----VLN-GG--D---
XP_021630853.1/1-226 SNML-IL------QE-SCTDPT----A-SFVIYAPVDIVAM-NV----VLN-GG--D---
XP_021907367.1/1-221 SNML-IL------QE-SGTDAT----G-SFVVYAPVDIVAM-NV----VLN-GG--D---
XP_010275996.1/1-221 SNML-IL------QE-SCTDST----G-SLVIYAPVDVVAM-NV----VLN-GG--D---
XP_020870784.1/1-223 SNML-IL------QE-SCTDPT----A-SFVIYAPVDIVAM-NI----VLN-GG--D---
NP_172015.1/1-223    SNML-IL------QE-SCTDPT----A-SFVIYAPVDIVAM-NI----VLN-GG--D---
XP_006418050.1/1-223 SNML-IL------QE-SCTDPT----A-SFVIYAPVDIVAM-NI----VLN-GG--D---
XP_018447753.1/1-223 SNML-IL------QE-SCTDPT----A-SFVIYAPVDIVAM-NI----VLN-GG--D---
XP_010457605.1/1-218 SNML-IL------QE-SCTDPT----A-SFVIYAPVDIVAM-NI----VLN-GG--D---
XP_023632765.1/1-218 SNML-IL------QE-SCTDPT----A-SFVIYAPVDIVAM-NI----VLN-GG--D---
XP_010484829.1/1-218 SNML-IL------QE-SCTDPT----A-SFVIYAPVDIVAM-NI----VLN-GG--D---
XP_010475208.1/1-218 SNML-IL------QE-SCTDPT----A-SFVIYAPVDIVAM-NI----VLN-GG--D---
XP_018487097.1/1-220 SNML-IL------QE-SCTDPT----A-SFVIYAPVDIVAM-NI----VLN-GG--D---
XP_013664795.1/1-220 SNML-IL------QE-SCTDPT----A-SFVIYAPVDIVAM-NI----VLN-GG--D---
XP_013716253.1/1-220 SNML-IL------QE-SCTDPT----A-SFVIYAPVDIVAM-NI----VLN-GG--D---
XP_009118607.1/1-220 SNML-IL------QE-SCTDPT----A-SFVIYAPVDIVAM-NI----VLN-GG--D---
XP_013605632.1/1-220 SNML-IL------QE-SCTDPT----A-SFVIYAPVDIVAM-NI----VLN-GG--D---
XP_010475204.1/1-220 SNML-IL------QE-SCTDPT----A-SFVIYAPVDIVAM-NI----VLN-GG--D---
XP_010457604.1/1-220 SNML-IL------QE-SCTDPT----A-SFVIYAPVDIVAM-NI----VLN-GG--D---
XP_023632762.1/1-220 SNML-IL------QE-SCTDPT----A-SFVIYAPVDIVAM-NI----VLN-GG--D---
XP_010484823.1/1-220 SNML-IL------QE-SCTDPT----A-SFVIYAPVDIVAM-NI----VLN-GG--D---
XP_022558401.1/1-220 SNML-IL------QE-SCTDPT----A-SFVIYAPVDIVAM-NI----VLN-GG--D---
XP_013586641.1/1-220 SNML-IL------QE-SCTDPT----A-SFVIYAPVDIVAM-NI----VLN-GG--D---
XP_022558399.1/1-220 SNML-IL------QE-SCTDPT----A-SFVIYAPVDIVAM-NI----VLN-GG--D---
XP_022558400.1/1-220 SNML-IL------QE-SCTDPT----A-SFVIYAPVDIVAM-NI----VLN-GG--D---
XP_013586642.1/1-220 SNML-IL------QE-SCTDPT----A-SFVIYAPVDIVAM-NI----VLN-GG--D---
XP_013696832.1/1-220 SNML-IL------QE-SCTDPT----A-SFVIYAPVDIVAM-NI----VLN-GG--D---
XP_022548878.1/1-220 SNML-IL------QE-SCTDPT----A-SFVIYAPVDIVAM-NI----VLN-GG--D---
XP_013666552.1/1-220 SNML-IL------QE-SCTDPT----A-SFVIYAPVDIVAM-NI----VLN-GG--D---
XP_009119177.1/1-220 SNML-IL------QE-SCTDPT----A-SFVIYAPVDIVAM-NI----VLN-GG--D---
XP_020964430.1/1-225 SNML-IL------QE-SCTDST----G-SFVIYAPVDIVAM-NV----VLN-GG--D---
XP_015936069.1/1-225 SNML-IL------QE-SCTDST----G-SFVIYAPVDIVAM-NV----VLN-GG--D---
XP_020964429.1/1-225 SNML-IL------QE-SCTDST----G-SFVIYAPVDIVAM-NV----VLN-GG--D---
XP_015936068.1/1-225 SNML-IL------QE-SCTDST----G-SFVIYAPVDIVAM-NV----VLN-GG--D---
XP_015577880.1/1-228 SNML-IL------QE-SCTDPT----A-SFVIYAPVDIVAM-NV----VLN-GG--D---
XP_002524172.2/1-228 SNML-IL------QE-SCTDPT----A-SFVIYAPVDIVAM-NV----VLN-GG--D---
XP_007132557.1/1-221 SNML-IL------QE-SCTDST----G-SFVIYAPVDIVAM-NV----VLN-GG--D---
XP_017433694.1/1-221 SNML-IL------QE-SCTDST----G-SFVIYAPVDIVAM-NV----VLN-GG--D---
XP_014494419.1/1-221 SNML-IL------QE-SCTDST----G-SFVIYAPVDIVAM-NV----VLN-GG--D---
XP_022634310.1/1-221 SNML-IL------QE-SCTDST----G-SFVIYAPVDIVAM-NV----VLN-GG--D---
XP_010267735.1/1-225 SNML-IL------QE-SCTDLT----G-SFVIYAPVDIVAM-NV----VLN-GG--D---
XP_010267734.1/1-225 SNML-IL------QE-SCTDLT----G-SFVIYAPVDIVAM-NV----VLN-GG--D---
XP_024185199.1/1-224 SNML-IL------QE-SCTDST----A-SYVIYAPVDIVAM-NV----VLN-GS--D---
XP_024185197.1/1-224 SNML-IL------QE-SCTDST----A-SYVIYAPVDIVAM-NV----VLN-GS--D---
XP_009342246.1/1-222 SNML-IL------QE-SCTDPT----A-SFVIYAPVDIVAM-NV----VLN-GS--D---
XP_009342244.1/1-222 SNML-IL------QE-SCTDPT----A-SFVIYAPVDIVAM-NV----VLN-GS--D---
XP_009342240.1/1-222 SNML-IL------QE-SCTDPT----A-SFVIYAPVDIVAM-NV----VLN-GS--D---
XP_020416503.1/1-221 SNML-IL------QE-SCTDQT----A-SFVIYAPVDIVAM-NV----VLN-GS--D---
XP_021822854.1/1-221 SNML-IL------QE-SCTDQT----A-SFVIYAPVDIVAM-NV----VLN-GS--D---
XP_007217584.1/1-221 SNML-IL------QE-SCTDQT----A-SFVIYAPVDIVAM-NV----VLN-GS--D---
XP_021822852.1/1-221 SNML-IL------QE-SCTDQT----A-SFVIYAPVDIVAM-NV----VLN-GS--D---
XP_018730806.1/1-228 SNML-IL------QE-SCTDSA----G-SFVIYAPVDIVAM-NV----VLN-GG--D---
XP_010051064.1/1-228 SNML-IL------QE-SCTDSA----G-SFVIYAPVDIVAM-NV----VLN-GG--D---
XP_015881130.2/1-229 SNML-IL------QE-SCTDST----A-SFVIYAPVDIVAM-NV----VLN-GG--D---
XP_023534533.1/1-219 SNML-IL------QE-SCTDPT----A-SFVIYAPVDIVAM-NV----VLN-GG--D---
XP_022947095.1/1-219 SNML-IL------QE-SCTDPT----A-SFVIYAPVDIVAM-NV----VLN-GG--D---
XP_022947096.1/1-219 SNML-IL------QE-SCTDPT----A-SFVIYAPVDIVAM-NV----VLN-GG--D---
XP_022970950.1/1-219 SNML-IL------QE-SCTDPT----A-SFVIYAPVDIVAM-NV----VLN-GG--D---
XP_022970951.1/1-219 SNML-IL------QE-SCTDPT----A-SFVIYAPVDIVAM-NV----VLN-GG--D---
XP_022140692.1/1-217 SNML-IL------QE-SCTDPT----A-SFVIYAPVDIVAM-NV----VLN-GG--D---
XP_022140691.1/1-217 SNML-IL------QE-SCTDPT----A-SFVIYAPVDIVAM-NV----VLN-GG--D---
XP_008455853.1/1-220 SNML-IL------QE-SCTDPT----A-SFVIYAPVDVVAM-NL----VLN-GG--D---
XP_011650012.1/1-220 SNML-IL------QE-SCTDPT----A-SFVIYAPVDVVAM-NL----VLN-GG--D---
XP_008455850.1/1-220 SNML-IL------QE-SCTDPT----A-SFVIYAPVDVVAM-NL----VLN-GG--D---
XP_011650011.1/1-220 SNML-IL------QE-SCTDPT----A-SFVIYAPVDVVAM-NL----VLN-GG--D---
XP_023513403.1/1-220 SNML-IL------QE-SCTDPT----V-SFVIYAPVDIVAM-NV----VLN-GG--D---
XP_022944761.1/1-220 SNML-IL------QE-SCTDPT----V-SFVIYAPVDIVAM-NV----VLN-GG--D---
XP_023513401.1/1-220 SNML-IL------QE-SCTDPT----V-SFVIYAPVDIVAM-NV----VLN-GG--D---
XP_022944759.1/1-220 SNML-IL------QE-SCTDPT----V-SFVIYAPVDIVAM-NV----VLN-GG--D---
XP_022986270.1/1-220 SNML-IL------QE-SCTDPT----V-SFVIYAPVDIVAM-NV----VLN-GG--D---
XP_022986268.1/1-220 SNML-IL------QE-SCTDPT----V-SFVIYAPVDIVAM-NV----VLN-GG--D---
XP_022136006.1/1-224 SNML-IL------QE-SSTDET----A-SFVIYAPVDIVAI-NV----VLN-GG--D---
XP_008455423.1/1-229 SNML-IL------QE-SSTDQT----A-SFVIYAPVDIVSI-NV----VLN-GG--D---
XP_011658704.1/1-229 SNML-IL------QE-SSTDQT----A-SFVIYAPVDIVSI-NV----VLN-GG--D---
XP_004144488.1/1-229 SNML-IL------QE-SSTDQT----A-SFVIYAPVDIVSI-NV----VLN-GG--D---
XP_022968990.1/1-229 SNML-IL------QE-SSTDQT----A-SFVVYAPVDIVAI-NV----VLN-GG--D---
XP_023554403.1/1-229 SNML-IL------QE-SSTDQT----A-SFVVYAPVDIVAI-NV----VLN-GG--D---
XP_022952948.1/1-229 SNML-IL------QE-SSTDET----A-SFVVYAPVDIVAI-NV----VLN-GG--D---
XP_019447751.1/1-228 SNML-IL------QE-SCTDST----G-SFVIYAPVDIAAM-NV----VLN-GG--H---
XP_019447749.1/1-228 SNML-IL------QE-SCTDST----G-SFVIYAPVDIAAM-NV----VLN-GG--H---
XP_004515074.1/1-228 SNML-IL------QE-SLTDST----G-SFVIYAPVDIVAM-NV----VLN-GG--D---
XP_012463792.1/1-235 SNML-IL------QE-SCTDPT----A-SFVIYAPVDIVAM-NV----VLN-GG--D---
XP_012463790.1/1-235 SNML-IL------QE-SCTDPT----A-SFVIYAPVDIVAM-NV----VLN-GG--D---
XP_016678783.1/1-235 SNML-IL------QE-SCTDPT----A-SFVIYAPVDIVAM-NV----VLN-GG--D---
XP_016678782.1/1-235 SNML-IL------QE-SCTDPT----A-SFVIYAPVDIVAM-NV----VLN-GG--D---
XP_017631648.1/1-235 SNML-IL------QE-SCADPT----A-SFVIYAPVDIVAM-NV----VLN-GG--D---
XP_016732503.1/1-235 SNML-IL------QE-SCADPT----A-SFVIYAPVDIVAM-NV----VLN-GG--D---
XP_017631647.1/1-235 SNML-IL------QE-SCADPT----A-SFVIYAPVDIVAM-NV----VLN-GG--D---
XP_012438430.1/1-229 TNML-IL------QE-SCTDPT----A-SFVIYAPVDIVAM-NV----VLN-GG--D---
XP_012438427.1/1-229 TNML-IL------QE-SCTDPT----A-SFVIYAPVDIVAM-NV----VLN-GG--D---
XP_012438429.1/1-229 TNML-IL------QE-SCTDPT----A-SFVIYAPVDIVAM-NV----VLN-GG--D---
XP_017616162.1/1-229 TNML-IL------QE-SCTDPT----A-SFVIYAPVDIVAM-NV----VLN-GG--D---
XP_017616160.1/1-229 TNML-IL------QE-SCTDPT----A-SFVIYAPVDIVAM-NV----VLN-GG--D---
XP_017616161.1/1-229 TNML-IL------QE-SCTDPT----A-SFVIYAPVDIVAM-NV----VLN-GG--D---
XP_016726347.1/1-229 TNML-IL------QE-SCTDPT----A-SFVIYAPVDIVAM-NV----VLN-GG--D---
XP_016726344.1/1-229 TNML-IL------QE-SCTDPT----A-SFVIYAPVDIVAM-NV----VLN-GG--D---
XP_016726345.1/1-229 TNML-IL------QE-SCTDPT----A-SFVIYAPVDIVAM-NV----VLN-GG--D---
XP_019448933.1/1-227 SNML-IL------QE-SCTDST----G-SFVIYAPVDIVAM-NV----VLS-GG--D---
XP_004487400.1/1-228 SNML-IL------QE-SCTDTT----G-SFVIYAPVDIVAM-NV----VLN-GG--D---
XP_003596999.2/1-227 SNML-IL------QE-SCTDTT----G-SFVIYAPVDIVAM-NV----VLN-GG--D---
XP_024631890.1/1-227 SNML-IL------QE-SCTDTT----G-SFVIYAPVDIVAM-NV----VLN-GG--D---
XP_024631891.1/1-227 SNML-IL------QE-SCTDTT----G-SFVIYAPVDIVAM-NV----VLN-GG--D---
XP_004287935.1/1-224 SNML-IL------QE-SCTDST----A-SYVIYAPVDIVAM-NV----VLN-GS--D---
XP_018808969.1/1-228 SNML-IL------QE-SCTDQT----A-SFVIYAPVDIVAM-NL----VLN-GG--D---
XP_018808967.1/1-228 SNML-IL------QE-SCTDQT----A-SFVIYAPVDIVAM-NL----VLN-GG--D---
XP_018817598.1/1-227 SNML-IL------QE-SCNDPT----A-SFVIYAPVDIVAM-NL----VLN-GG--D---
XP_018817597.1/1-227 SNML-IL------QE-SCNDPT----A-SFVIYAPVDIVAM-NL----VLN-GG--D---
XP_018817594.1/1-227 SNML-IL------QE-SCNDPT----A-SFVIYAPVDIVAM-NL----VLN-GG--D---
XP_018817599.1/1-227 SNML-IL------QE-SCNDPT----A-SFVIYAPVDIVAM-NL----VLN-GG--D---
XP_023915056.1/1-227 SNML-IL------QE-SCTDPT----A-SFVIYAPVDIVAM-NL----VLN-GG--D---
XP_023915054.1/1-227 SNML-IL------QE-SCTDPT----A-SFVIYAPVDIVAM-NL----VLN-GG--D---
XP_023915053.1/1-227 SNML-IL------QE-SCTDPT----A-SFVIYAPVDIVAM-NL----VLN-GG--D---
XP_021685023.1/1-228 SNML-IL------QE-SCTDQT----A-SFVIYAPVDTVAM-NV----LLN-GG--D---
XP_021684657.1/1-228 SNML-IL------QE-SCTDQT----A-SFVIYAPVDTVAM-NV----LLN-GG--D---
XP_021685089.1/1-228 SNML-IL------QE-SCTDQT----A-SFVIYAPVDTVAM-NV----LLN-GG--D---
XP_015383009.1/1-229 SNML-IL------QE-SCTDPT----A-SFVIYAPVDIVAM-NV----VLN-GG--D---
XP_015383007.1/1-229 SNML-IL------QE-SCTDPT----A-SFVIYAPVDIVAM-NV----VLN-GG--D---
XP_020539441.1/1-235 SNML-IL------QE-SCTDPT----A-SFVIYAPVDIVAM-NV----VLN-GG--D---
XP_020539439.1/1-235 SNML-IL------QE-SCTDPT----A-SFVIYAPVDIVAM-NV----VLN-GG--D---
XP_015577881.1/1-233 SNML-IL------QE-SCTDPT----A-SFVIYAPVDIVAM-NV----VLN-GG--D---
XP_015577879.1/1-233 SNML-IL------QE-SCTDPT----A-SFVIYAPVDIVAM-NV----VLN-GG--D---
XP_015577877.1/1-233 SNML-IL------QE-SCTDPT----A-SFVIYAPVDIVAM-NV----VLN-GG--D---
XP_021630854.1/1-231 SNML-IL------QE-SCTDPT----A-SFVIYAPVDIVAM-NV----VLN-GG--D---
XP_021630849.1/1-231 SNML-IL------QE-SCTDPT----A-SFVIYAPVDIVAM-NV----VLN-GG--D---
XP_021652390.1/1-229 SNML-IL------QE-SCTDPT----A-SFVIYAPVDIVAM-NV----VLN-GG--D---
XP_021652388.1/1-229 SNML-IL------QE-SCTDPT----A-SFVIYAPVDIVAM-NV----VLN-GG--D---
XP_021652389.1/1-234 SNML-IL------QE-SCTDPT----A-SFVIYAPVDIVAM-NV----VLN-GG--D---
XP_021652386.1/1-234 SNML-IL------QE-SCTDPT----A-SFVIYAPVDIVAM-NV----VLN-GG--D---
XP_006592380.1/1-228 SNML-IL------QE-SCTNST----G-SFVIYAPVDIVAM-NV----VLN-GG--D---
XP_003539872.2/1-228 SNML-IL------QE-SCTNST----G-SFVIYAPVDIVAM-NV----VLN-GG--D---
XP_025984806.1/1-230 SNML-IL------QE-SCTDST----G-SFVIYAPVDIVAM-NV----VLN-GG--D---
XP_014632311.1/1-230 SNML-IL------QE-SCTDST----G-SFVIYAPVDIVAM-NV----VLN-GG--D---
XP_024450362.1/1-227 SNML-IL------QE-SCTDQT----A-SFVIYAPVDIVAM-NV----VLN-GG--D---
XP_002302880.2/1-227 SNML-IL------QE-SCTDQT----A-SFVIYAPVDIVAM-NV----VLN-GG--D---
XP_024450361.1/1-227 SNML-IL------QE-SCTDQT----A-SFVIYAPVDIVAM-NV----VLN-GG--D---
XP_024450360.1/1-227 SNML-IL------QE-SCTDQT----A-SFVIYAPVDIVAM-NV----VLN-GG--D---
XP_011011493.1/1-227 SNML-IL------QE-SCTDQT----A-SFVIYAPVDIVAM-NV----VLN-GG--D---
XP_011011491.1/1-227 SNML-IL------QE-SCTDQT----A-SFVIYAPVDIVAM-NV----VLN-GG--D---
XP_011011492.1/1-227 SNML-IL------QE-SCTDQT----A-SFVIYAPVDIVAM-NV----VLN-GG--D---
XP_011011489.1/1-227 SNML-IL------QE-SCTDQT----A-SFVIYAPVDIVAM-NV----VLN-GG--D---
XP_011011488.1/1-227 SNML-IL------QE-SCTDQT----A-SFVIYAPVDIVAM-NV----VLN-GG--D---
XP_011005116.1/1-225 SNML-IL------QE-SCTDQT----A-SFVIYAPVDIVAM-NV----VLN-GG--D---
XP_011005113.1/1-225 SNML-IL------QE-SCTDQT----A-SFVIYAPVDIVAM-NV----VLN-GG--D---
XP_011005112.1/1-225 SNML-IL------QE-SCTDQT----A-SFVIYAPVDIVAM-NV----VLN-GG--D---
XP_024440391.1/1-225 SNML-IL------QE-SCADQT----A-SFVIYAPVDIVAM-NV----VLN-GG--D---
XP_002320458.2/1-225 SNML-IL------QE-SCADQT----A-SFVIYAPVDIVAM-NV----VLN-GG--D---
XP_024440389.1/1-225 SNML-IL------QE-SCADQT----A-SFVIYAPVDIVAM-NV----VLN-GG--D---
XP_019453767.1/1-227 SNML-IL------QE-SCMDST----G-SFVIYAPVDIVAM-NV----VLN-GG--D---
XP_012478857.1/1-227 SNML-IL------QE-SCTDPT----A-SFVIYAPVDIVAM-NV----VLN-GG--D---
XP_012478855.1/1-227 SNML-IL------QE-SCTDPT----A-SFVIYAPVDIVAM-NV----VLN-GG--D---
XP_012478856.1/1-227 SNML-IL------QE-SCTDPT----A-SFVIYAPVDIVAM-NV----VLN-GG--D---
XP_012478851.1/1-227 SNML-IL------QE-SCTDPT----A-SFVIYAPVDIVAM-NV----VLN-GG--D---
XP_016724999.1/1-227 SNML-IL------QE-SCTDPT----A-SFVIYAPVDIVAM-NV----VLN-GG--D---
XP_017638204.1/1-227 SNML-IL------QE-SCTDPT----A-SFVIYAPVDIVAM-NV----VLN-GG--D---
XP_017638203.1/1-227 SNML-IL------QE-SCTDPT----A-SFVIYAPVDIVAM-NV----VLN-GG--D---
XP_016667113.1/1-227 SNML-IL------QE-SCTDPT----A-SFVIYAPVDIVAM-NV----VLN-GG--D---
XP_016667112.1/1-227 SNML-IL------QE-SCTDPT----A-SFVIYAPVDIVAM-NV----VLN-GG--D---
XP_015881131.2/1-227 SNML-IL------QE-SCTDST----A-SFVIYAPVDIVAM-NV----VLN-GG--D---
XP_008348131.1/1-222 SNML-IL------QE-SCSDPT----A-SFVIYAPVDIVSM-NV----VLN-GS--D---
XP_008348133.1/1-220 SNML-IL------QE-SCSDPT----A-SFVIYAPVDIVSM-NV----VLN-GS--D---
XP_018502305.1/1-220 SNML-IL------QE-SFSDPT----A-SFVIYAPVDIVSM-NV----VLN-GS--D---
XP_009342243.1/1-220 SNML-IL------QE-SCTDPT----A-SFVIYAPVDIVAM-NV----VLN-GS--D---
XP_017179321.1/1-222 SNML-IL------QE-SCTDPT----A-SFVIYAPVDIVAM-NV----VLN-GS--D---
XP_017179320.1/1-222 SNML-IL------QE-SCTDPT----A-SFVIYAPVDIVAM-NV----VLN-GS--D---
XP_008341986.1/1-222 SNML-IL------QE-SCTDPT----A-SFVIYAPVDIVAM-NV----VLN-GS--D---
XP_017179319.1/1-220 SNML-IL------QE-SCTDPT----A-SFVIYAPVDIVAM-NV----VLN-GS--D---
XP_024185198.1/1-222 SNML-IL------QE-SCTDST----A-SYVIYAPVDIVAM-NV----VLN-GS--D---
XP_011463209.1/1-222 SNML-IL------QE-SCTDST----A-SYVIYAPVDIVAM-NV----VLN-GS--D---
XP_020416501.1/1-219 SNML-IL------QE-SCTDQT----A-SFVIYAPVDIVAM-NV----VLN-GS--D---
XP_021822853.1/1-219 SNML-IL------QE-SCTDQT----A-SFVIYAPVDIVAM-NV----VLN-GS--D---
XP_008230079.1/1-219 SNML-IL------QE-SCTDQT----A-SFVIYAPVDIVAM-NV----VLN-GS--D---
XP_020870786.1/1-221 SNML-IL------QE-SCTDPT----A-SFVIYAPVDIVAM-NI----VLN-GG--D---
NP_001117231.1/1-221 SNML-IL------QE-SCTDPT----A-SFVIYAPVDIVAM-NI----VLN-GG--D---
XP_018817596.1/1-225 SNML-IL------QE-SCNDPT----A-SFVIYAPVDIVAM-NL----VLN-GG--D---
XP_019079350.1/1-224 SNML-IL------QE-SCTDST----A-SFVIYAPVDVVAM-NM----VLN-GG--D---
XP_022733639.1/1-224 SNML-IL------QE-SCTDPT----A-SFVIYAPVDIVAM-NV----VLN-GG--D---
XP_021292768.1/1-224 SNML-IL------QE-SCADPT----A-SFVIYAPVDIVAM-NV----VLN-GG--D---
XP_021292766.1/1-224 SNML-IL------QE-SCADPT----A-SFVIYAPVDIVAM-NV----VLN-GG--D---
XP_017971480.1/1-224 SNML-IL------QE-SCADPT----A-SFVIYAPVDIVAM-NV----VLN-GG--D---
XP_018721484.1/1-222 SNML-IL------QE-SCTDAT----G-SFVVYAPVDIVAM-NV----VLN-GG--D---
XP_018721483.1/1-222 SNML-IL------QE-SCTDAT----G-SFVVYAPVDIVAM-NV----VLN-GG--D---
XP_018721482.1/1-222 SNML-IL------QE-SCTDAT----G-SFVVYAPVDIVAM-NV----VLN-GG--D---
XP_025687365.1/1-237 SNML-IL------QE-SCTDST----G-SFVVYAPVDIVAM-NV----VLN-GG--D---
XP_006851770.2/1-243 SSML-IL------QE-TCT-DA-A-G--SLVVYAPVDIPAM-HV----VMS-GG--D---
XP_003623826.1/1-230 SSML-IL------QE-TST-DA-S-G--SLVVYAPVDIPAM-HV----VMN-GG--D---
XP_004492678.1/1-230 SSML-IL------QE-TCT-DA-S-G--SLVVYAPVDIPAM-HV----VMN-GG--D---
XP_004492677.1/1-230 SSML-IL------QE-TCT-DA-S-G--SLVVYAPVDIPAM-HV----VMN-GG--D---
XP_012847897.1/1-236 SSML-IL------QE-TCI-DA-A-G--ALVVYAPVDIPAM-HL----VMN-GG--D---
XP_012843673.1/1-229 SSML-IL------QE-TCI-DA-A-G--ALVVYAPVDIPAM-HL----VMN-GG--D---
XP_021279608.1/1-239 SSML-IL------QE-TCI-DA-A-G--SLVVYAPVDIPAM-HV----VMN-GG--D---
XP_025641241.1/1-241 SSML-IL------QE-TCI-DA-A-G--SLVVYAPVDIPAM-HV----VMN-GG--D---
XP_025641246.1/1-241 SSML-IL------QE-TCI-DA-A-G--SLVVYAPVDIPAM-HV----VMN-GG--D---
XP_020991942.1/1-241 SSML-IL------QE-TCI-DA-A-G--SLVVYAPVDIPAM-HV----VMN-GG--D---
XP_025632115.1/1-241 SSML-IL------QE-TCI-DA-A-G--SLVVYAPVDIPAM-HV----VMN-GG--D---
XP_025632116.1/1-241 SSML-IL------QE-TCI-DA-A-G--SLVVYAPVDIPAM-HV----VMN-GG--D---
XP_017442222.1/1-246 SSML-IL------QE-TCI-DE-A-G--SLVVYAPVDIPAM-HV----VMN-GG--D---
XP_017442225.1/1-246 SSML-IL------QE-TCI-DE-A-G--SLVVYAPVDIPAM-HV----VMN-GG--D---
XP_006576359.1/1-244 SSML-IL------QE-TCI-DA-A-G--SLVVYAPVDIPAM-HV----VMN-GG--D---
XP_003521284.1/1-244 SSML-IL------QE-TCI-DA-A-G--SLVVYAPVDIPAM-HV----VMN-GG--D---
XP_024174849.1/1-237 NSML-IL------QE-TCI-DA-A-G--SLVVYAPVDIPAM-HV----VMN-GG--D---
XP_024174854.1/1-237 NSML-IL------QE-TCI-DA-A-G--SLVVYAPVDIPAM-HV----VMN-GG--D---
XP_004306832.1/1-241 NSML-IL------QE-TCI-DA-A-G--SLVVYAPVDIPAM-HV----VMN-GG--D---
XP_011469062.1/1-241 NSML-IL------QE-TCI-DA-A-G--SLVVYAPVDIPAM-HV----VMN-GG--D---
XP_009340632.2/1-243 GSML-IL------QE-TCI-DA-A-G--SLVVYAPVDIPAM-HV----VMN-GG--D---
XP_008385703.1/1-243 GSML-IL------QE-TCI-DA-A-G--SLVVYAPVDIPAM-HV----VMN-GG--D---
XP_008385704.1/1-243 GSML-IL------QE-TCI-DA-A-G--SLVVYAPVDIPAM-HV----VMN-GG--D---
XP_008232741.1/1-243 SSML-IL------QE-TCI-DA-A-G--GLVVYAPVDIPAM-HV----VMN-GG--D---
XP_008232742.1/1-243 SSML-IL------QE-TCI-DA-A-G--GLVVYAPVDIPAM-HV----VMN-GG--D---
XP_007220256.1/1-243 SSML-IL------QE-TCI-DS-A-G--GLVVYAPVDIPAM-HV----VMN-GG--D---
XP_020413144.1/1-243 SSML-IL------QE-TCI-DS-A-G--GLVVYAPVDIPAM-HV----VMN-GG--D---
XP_021817103.1/1-243 SSML-IL------QE-TCI-DA-A-G--GLVVYAPVDIPAM-HV----VMN-GG--D---
XP_021817105.1/1-243 SSML-IL------QE-TCI-DA-A-G--GLVVYAPVDIPAM-HV----VMN-GG--D---
XP_022872559.1/1-230 SSML-IL------QE-TCI-DE-A-G--SLAVYAPVDIPAM-HV----VMN-GG--D---
XP_016575742.1/1-238 SSML-IL------QE-TCI-DA-A-G--ALVVYAPVDIPAM-HV----VMN-GG--D---
XP_017608938.1/1-232 SSML-IL------QE-TCM-DA-G-G--SLVVYAPVDIPAV-QV----VMN-GG--D---
XP_017608947.1/1-232 SSML-IL------QE-TCM-DA-G-G--SLVVYAPVDIPAV-QV----VMN-GG--D---
XP_010255501.1/1-245 SNML-IL------QE-TCF-DA-S-G--SLVVYAPVDIPAM-HV----VMN-GG--D---
XP_011023407.1/1-238 SSML-IL------QE-TCM-DA-A-G--SLVVYAPVDTPAM-HV----VMN-GG--D---
XP_011023404.1/1-238 SSML-IL------QE-TCM-DA-A-G--SLVVYAPVDTPAM-HV----VMN-GG--D---
XP_011023405.1/1-238 SSML-IL------QE-TCM-DA-A-G--SLVVYAPVDTPAM-HV----VMN-GG--D---
XP_019152252.1/1-243 SSML-IL------QE-TCI-NA-A-G--SLVVYAPVDIPAM-HV----VMN-GG--D---
XP_022870029.1/1-235 RSML-IL------QE-TCI-DA-A-G--SLVVYAPVDIPAM-HV----VMN-GG--D---
XP_017699285.1/1-225 SSML-IL------QE-TCT-DA-S-G--SMVVYAPVDIPAM-HV----VMN-GG--D---
XP_008795402.1/1-225 SSML-IL------QE-TCT-DA-S-G--SMVVYAPVDIPAM-HV----VMN-GG--D---
XP_010933869.1/1-224 SSML-IL------QE-TCT-DA-S-G--SMVVYAPVDIPAM-HV----VMN-GG--D---
XP_008799528.1/1-222 SSML-IL------QE-TCT-DA-S-G--SMVVYAPVDIPAM-HV----VMN-GG--D---
XP_010919252.1/1-223 SSML-IL------QE-TCT-DA-S-G--SMVVYAPVDIPAM-HV----VMN-GG--D---
XP_010919250.1/1-223 SSML-IL------QE-TCT-DA-S-G--SMVVYAPVDIPAM-HV----VMN-GG--D---
XP_007139955.1/1-229 SSML-IL------QE-TCT-DA-S-G--SLVVYAPVDIPAM-HV----VMN-GG--D---
XP_017418814.1/1-230 SSML-IL------QE-TCT-DA-S-G--SLVVYAPVDIPAM-HV----VMN-GG--D---
XP_014490274.1/1-230 SSML-IL------QE-TCT-DA-S-G--SLVVYAPVDIPAM-HV----VMN-GG--D---
XP_003534596.1/1-229 SSML-IL------QE-TCT-DA-S-G--SLVVYAPVDIPAM-HV----VMN-GG--D---
XP_006587871.1/1-229 SSML-IL------QE-TCT-DA-S-G--SLVVYAPVDIPAM-HV----VMN-GG--D---
XP_003552359.1/1-228 SSML-IL------QE-TCT-DA-S-G--SLVVYAPVDIPAM-HV----VMN-GG--D---
XP_015961616.1/1-228 SSML-IL------QE-TCT-DA-S-G--SLVVYAPVDIPAM-HL----VMN-GG--D---
XP_015961617.1/1-228 SSML-IL------QE-TCT-DA-S-G--SLVVYAPVDIPAM-HL----VMN-GG--D---
XP_025696060.1/1-228 SSML-IL------QE-TCT-DA-S-G--SLVVYAPVDIPAM-HL----VMN-GG--D---
XP_025696061.1/1-228 SSML-IL------QE-TCT-DA-S-G--SLVVYAPVDIPAM-HL----VMN-GG--D---
XP_016198931.1/1-228 SSML-IL------QE-TCT-DA-S-G--SLVVYAPVDIPAM-HL----VMN-GG--D---
XP_016198932.1/1-228 SSML-IL------QE-TCT-DA-S-G--SLVVYAPVDIPAM-HL----VMN-GG--D---
XP_025649261.1/1-228 SSML-IL------QE-TCT-DA-S-G--SLVVYAPVDIPAM-HL----VMN-GG--D---
XP_025649262.1/1-228 SSML-IL------QE-TCT-DA-S-G--SLVVYAPVDIPAM-HL----VMN-GG--D---
XP_021849370.1/1-228 SSML-IL------QE-TCT-DS-S-G--SLVVYAPVDIPAM-HV----VMN-GG--D---
XP_021767325.1/1-228 SSML-IL------QE-TCT-DS-S-G--SLVVYAPVDIPAM-HV----VMN-GG--D---
XP_021748881.1/1-228 SSML-IL------QE-TCT-DS-S-G--SLVVYAPVDIPAM-HV----VMN-GG--D---
XP_019461451.1/1-231 SSML-IL------QE-TSI-DA-A-G--SLVVYAPVDIPAM-HV----VMN-GG--D---
XP_012574211.1/1-233 SSML-IL------QE-TCI-DE-A-G--SLVVYAPVDIPAM-HV----VMN-GG--D---
XP_004510857.1/1-233 SSML-IL------QE-TCI-DE-A-G--SLVVYAPVDIPAM-HV----VMN-GG--D---
XP_020237185.1/1-234 SSML-IL------QE-TCI-DA-A-G--SLVVYAPVDIPAM-HV----VMN-GG--D---
XP_020237261.1/1-234 SSML-IL------QE-TCI-DA-A-G--SLVVYAPVDIPAM-HV----VMN-GG--D---
XP_019456357.1/1-238 SSML-IL------QE-TCI-DA-A-G--SLVVYAPVDIPAM-HV----VMN-GG--D---
XP_013444922.1/1-235 SSML-IL------QE-TCI-DE-A-G--SLVVYAPVDIPAM-HV----VMN-GG--D---
XP_003528880.1/1-238 SSML-IL------QE-TCI-DA-A-G--SLVVYAPVDIPAM-HV----VMN-GG--D---
XP_006583340.1/1-238 SSML-IL------QE-TCI-DA-A-G--SLVVYAPVDIPAM-HV----VMN-GG--D---
XP_014516480.1/1-238 SSML-IL------QE-TCI-DE-A-G--SLVVYAPVDIPAM-HV----VMN-GG--D---
XP_014516483.1/1-238 SSML-IL------QE-TCI-DE-A-G--SLVVYAPVDIPAM-HV----VMN-GG--D---
XP_007134961.1/1-238 SSML-IL------QE-TCI-DE-A-G--SLVVYAPVDIPAM-HV----VMN-GG--D---
XP_010671720.1/1-229 SSML-IL------QE-TCT-DA-S-G--SLVVYAPVDIPAM-HV----VMN-GG--D---
XP_012482732.1/1-232 SSML-IL------QE-TCM-DA-G-G--SLVVYAPVDIPAM-QV----VMS-GG--D---
XP_012482742.1/1-232 SSML-IL------QE-TCM-DA-G-G--SLVVYAPVDIPAM-QV----VMS-GG--D---
XP_016745638.1/1-232 SSML-IL------QE-TCM-DA-G-G--SLVVYAPVDIPAM-QV----VMS-GG--D---
XP_016745639.1/1-232 SSML-IL------QE-TCM-DA-G-G--SLVVYAPVDIPAM-QV----VMS-GG--D---
XP_010278578.1/1-242 SSML-IL------QE-TCI-DA-A-G--SLVVYAPVDIPAM-HL----VMN-GG--D---
XP_010278577.1/1-242 SSML-IL------QE-TCI-DA-A-G--SLVVYAPVDIPAM-HL----VMN-GG--D---
XP_006445143.1/1-238 SSML-IL------QE-TCT-DA-A-G--SLVVYAPVDIPAM-HV----VMN-GG--D---
XP_006445141.1/1-238 SSML-IL------QE-TCT-DA-A-G--SLVVYAPVDIPAM-HV----VMN-GG--D---
XP_022770361.1/1-238 SSML-IL------QE-TCI-DA-A-G--SLVVYAPVDIPAM-HV----VMN-GG--D---
XP_022757219.1/1-237 SSML-IL------QE-TCI-DA-A-G--SLVVYAPVDIPAM-HV----VMN-GG--D---
XP_022757220.1/1-237 SSML-IL------QE-TCI-DA-A-G--SLVVYAPVDIPAM-HV----VMN-GG--D---
NP_001314420.1/1-237 SSML-IL------QE-TCI-DA-A-G--SLVVYAPVDIPAM-HV----VMN-GG--D---
XP_016735217.1/1-237 SSML-IL------QE-TCI-DA-A-G--SLVVYAPVDIPAM-HV----VMN-GG--D---
XP_012437660.1/1-237 SSML-IL------QE-TCI-DA-A-G--SLVVYAPVDIPAM-HV----VMN-GG--D---
XP_012437661.1/1-237 SSML-IL------QE-TCI-DA-A-G--SLVVYAPVDIPAM-HV----VMN-GG--D---
XP_017615505.1/1-237 SSML-IL------QE-TCI-DA-A-G--SLVVYAPVDIPAM-HV----VMN-GG--D---
XP_017615506.1/1-237 SSML-IL------QE-TCI-DA-A-G--SLVVYAPVDIPAM-HV----VMN-GG--D---
XP_016723220.1/1-237 SSML-IL------QE-TCI-DA-A-G--SLVVYAPVDIPAM-HV----VMN-GG--D---
XP_016723229.1/1-237 SSML-IL------QE-TCI-DA-A-G--SLVVYAPVDIPAM-HV----VMN-GG--D---
XP_021904309.1/1-244 SSML-IL------QE-TCI-DA-A-G--SLVVYAPVDIPAM-HV----VMN-GG--D---
XP_021904311.1/1-244 SSML-IL------QE-TCI-DA-A-G--SLVVYAPVDIPAM-HV----VMN-GG--D---
XP_007051912.2/1-241 SSML-IL------QE-TCI-DA-A-G--SLVVYAPVDIPAM-HV----VMN-GG--D---
XP_007051913.2/1-241 SSML-IL------QE-TCI-DA-A-G--SLVVYAPVDIPAM-HV----VMN-GG--D---
XP_012489878.1/1-239 SSML-IL------QE-TCI-DA-A-G--SLVVYAPVDIPAM-HV----VMN-GG--D---
XP_016748231.1/1-239 SSML-IL------QE-TCI-DA-A-G--SLVVYAPVDIPAM-HV----VMN-GG--D---
XP_017631289.1/1-239 SSML-IL------QE-TCI-DA-A-G--SLVVYAPVDIPAM-HV----VMN-GG--D---
XP_023006686.1/1-236 SSML-IL------QE-TCI-DA-A-G--SLVVYAPVDIPAM-HV----VMN-GG--D---
XP_022958954.1/1-236 SSML-IL------QE-TCI-DA-A-G--SLVVYAPVDIPAM-HV----VMN-GG--D---
XP_023548045.1/1-236 TSML-IL------QE-TCI-DA-A-G--SLVVYAPVDIPAM-HV----VMN-GG--D---
XP_008460172.1/1-241 SSML-IL------QE-TCI-DA-A-G--SLVVYAPVDIPAM-HV----VMN-GG--D---
XP_004144982.1/1-241 SSML-IL------QE-TCI-DA-A-G--SLVVYAPVDIPAM-HV----VMN-GG--D---
XP_023514722.1/1-242 SSML-IL------QE-TCI-DA-A-G--SLVVYAPVDIPAM-HV----VMN-GG--D---
XP_022999814.1/1-242 SSML-IL------QE-TCI-DA-A-G--SLVVYAPVDIPAM-HV----VMN-GG--D---
XP_022964504.1/1-242 SSML-IL------QE-TCI-DA-A-G--SLVVYAPVDIPAM-HV----VMN-GG--D---
XP_010053664.1/1-245 SSML-IL------QE-TCI-DA-A-G--SLVVYAPVDIPAM-HV----VMN-GG--D---
XP_019189515.1/1-235 SSML-IL------QE-TCI-DA-A-G--SLVVYAPVDIPAM-HV----VMN-GG--D---
XP_023730775.1/1-234 SSML-IL------QE-TCI-DA-A-G--SLVVYAPVDIPAM-HV----VMN-GG--D---
XP_023730774.1/1-234 SSML-IL------QE-TCI-DA-A-G--SLVVYAPVDIPAM-HV----VMN-GG--D---
XP_022020543.1/1-230 SSML-IL------QE-TCI-DS-A-G--SLVVYAPVDIPAM-HV----VMN-GG--D---
XP_024962572.1/1-236 SSML-IL------QE-TCI-DA-A-G--SLVVYAPVDIPAM-HV----VMN-GG--D---
XP_022017696.1/1-236 SSML-IL------QE-TCI-DS-A-G--SLVVYAPVDIPAM-HV----VMN-GG--D---
XP_024018220.1/1-245 SSML-IL------QE-TCI-DA-A-G--SLVVYAPVDIPAM-HV----VMN-GG--D---
XP_011093166.1/1-234 SSML-IL------QE-TCI-DA-A-G--SLVVYAPVDIPAM-HV----VMN-GG--D---
XP_011093167.1/1-234 SSML-IL------QE-TCI-DA-A-G--SLVVYAPVDIPAM-HV----VMN-GG--D---
XP_006339457.1/1-238 SSML-IL------QE-TCI-DA-A-G--ALVVYAPVDIPAM-HV----VMN-GG--D---
XP_015059298.1/1-238 SSML-IL------QE-TCI-DA-A-G--ALVVYAPVDIPAM-HV----VMN-GG--D---
NP_001234657.2/1-238 SSML-IL------QE-TCI-DA-A-G--ALVVYAPVDIPAM-HV----VMN-GG--D---
XP_009602857.1/1-241 SSML-IL------QE-TCI-DA-A-G--ALVVYAPVDIPAM-HV----VMN-GG--D---
XP_009602856.1/1-241 SSML-IL------QE-TCI-DA-A-G--ALVVYAPVDIPAM-HV----VMN-GG--D---
XP_009782444.1/1-245 SSML-IL------QE-TCI-DA-A-G--ALVVYAPVDIPAM-HV----VMN-GG--D---
XP_009782443.1/1-245 SSML-IL------QE-TCI-DA-A-G--ALVVYAPVDIPAM-HV----VMN-GG--D---
XP_019259406.1/1-245 SSML-IL------QE-TCI-DA-A-G--ALVVYAPVDIPAM-HV----VMN-GG--D---
XP_015867375.1/1-242 SSML-IL------QE-TCI-DA-A-G--SLVVYAPVDIPAM-HI----VMN-GG--D---
XP_015867374.1/1-242 SSML-IL------QE-TCI-DA-A-G--SLVVYAPVDIPAM-HI----VMN-GG--D---
XP_015888672.1/1-242 SSML-IL------QE-TCI-DA-A-G--SLVVYAPVDIPAM-HI----VMN-GG--D---
XP_018810322.1/1-233 SSML-IL------QE-TCI-DA-A-G--SLVVYAPVDIPAM-HV----VMN-GG--D---
XP_018859074.1/1-233 SSML-IL------QE-TCI-DA-A-G--SLVVYAPVDIPAM-HV----VMN-GG--D---
XP_017231243.1/1-235 SSML-IL------QE-TCI-DA-A-G--SLVVYAPVDIPAM-NV----VMN-GG--D---
XP_017231244.1/1-235 SSML-IL------QE-TCI-DA-A-G--SLVVYAPVDIPAM-NV----VMN-GG--D---
XP_017231245.1/1-235 SSML-IL------QE-TCI-DA-A-G--SLVVYAPVDIPAM-NV----VMN-GG--D---
XP_017236322.1/1-235 SSML-IL------QE-TCI-DA-A-G--SLVVYAPVDIPAM-HV----VMN-GG--D---
XP_002301331.2/1-238 SSML-IL------QE-TCI-DA-A-G--SLVVYAPVDTPAM-HV----VMN-GG--D---
XP_024451706.1/1-238 SSML-IL------QE-TCI-DA-A-G--SLVVYAPVDTPAM-HV----VMN-GG--D---
XP_002320755.1/1-241 SSML-IL------QE-TCI-DA-A-G--SLVVYAPVDIPAM-HV----VMN-GG--D---
XP_011035097.1/1-241 SSML-IL------QE-TCI-DA-A-G--SLVVYAPVDIPAM-HV----VMN-GG--D---
XP_018813891.1/1-238 SSML-IL------QE-TCI-DA-A-G--SLVVYAPVDIPAM-HV----VMN-GG--D---
XP_012083470.1/1-239 SSML-IL------QE-TCI-DA-A-G--SLVVYAPVDIPAM-HV----VMN-GG--D---
XP_021651338.1/1-240 SSML-IL------QE-TCI-DA-A-G--SLVVYAPVDIPAM-HV----VMN-GG--D---
XP_021613538.1/1-241 SSML-IL------QE-TCI-DA-A-G--SLVVYAPVDIPAM-HV----VMN-GG--D---
XP_021686862.1/1-241 SSML-IL------QE-TCI-DA-A-G--SLVVYAPVDIPAM-HV----VMN-GG--D---
XP_021686863.1/1-241 SSML-IL------QE-TCI-DA-A-G--SLVVYAPVDIPAM-HV----VMN-GG--D---
XP_021612685.1/1-241 SSML-IL------QE-TCI-DA-A-G--SLVVYAPVDIPAM-HV----VMN-GG--D---
XP_023879166.1/1-242 SSML-IL------QE-TCI-DA-A-G--SLVVYAPVDIPAM-HV----VMN-GG--D---
XP_002272264.2/1-234 SSML-IL------QE-TCI-DA-A-G--SLVVYAPVDIPAM-HV----VMN-GG--D---
XP_010661562.1/1-234 SSML-IL------QE-TCI-DA-A-G--SLVVYAPVDIPAM-HV----VMN-GG--D---
XP_002511801.1/1-237 SSML-IL------QE-TCI-DA-A-G--SLVVYAPVDIPAM-HV----VMN-GG--D---
XP_015584500.1/1-237 SSML-IL------QE-TCI-DA-A-G--SLVVYAPVDIPAM-HV----VMN-GG--D---
XP_022141257.1/1-238 STML-IL------QE-TWT-DI-S-G--SLVVYAPVDTASV-NL----VMR-GG--D---
XP_023885612.1/1-244 SNML-IL------QE-TWT-DA-S-G--SVVVYAPVDAPSI-NV----VMS-GG--D---
XP_023885611.1/1-244 SNML-IL------QE-TWT-DA-S-G--SVVVYAPVDAPSI-NV----VMS-GG--D---
XP_018830842.1/1-239 NNML-IL------QE-TWS-DA-S-G--SVVVYAPVDIPSI-NV----VMG-GG--D---
XP_018830843.1/1-239 NNML-IL------QE-TWS-DA-S-G--SVVVYAPVDIPSI-NV----VMG-GG--D---
XP_018830840.1/1-239 NNML-IL------QE-TWS-DA-S-G--SVVVYAPVDIPSI-NV----VMG-GG--D---
XP_018830841.1/1-239 NNML-IL------QE-TWS-DA-S-G--SVVVYAPVDIPSI-NV----VMG-GG--D---
XP_012090293.1/1-240 SNML-IL------QE-TWN-DA-S-N--SLVVYAPVDIPSM-SV----VMN-GG--D---
XP_012090292.1/1-240 SNML-IL------QE-TWN-DA-S-N--SLVVYAPVDIPSM-SV----VMN-GG--D---
XP_021614581.1/1-239 NSMI-IL------QE-TWN-DA-S-S--SLVVYAPVDIQSI-SV----VMN-GG--D---
XP_021614580.1/1-239 NSMI-IL------QE-TWN-DA-S-S--SLVVYAPVDIQSI-SV----VMN-GG--D---
XP_015581247.1/1-240 SSML-IL------QE-TWH-DV-A-S--SLVVYAPVDIPSM-SV----VMN-GG--D---
XP_002510822.1/1-240 SSML-IL------QE-TWH-DV-A-S--SLVVYAPVDIPSM-SV----VMN-GG--D---
XP_021648646.1/1-240 NSML-IL------QE-TWN-DA-S-S--SLVVYAPVDIPSM-SV----VMN-GG--D---
XP_021634593.1/1-234 NNML-IL------QE-TWN-DA-S-S--SLIVYAPVDVPSM-SV----VMN-GG--D---
XP_021634592.1/1-234 NNML-IL------QE-TWN-DA-S-S--SLIVYAPVDVPSM-SV----VMN-GG--D---
XP_006490348.1/1-238 SGML-IL------QE-TWN-DS-S-G--SLVVYAPVDVPSM-SM----VMN-GG--D---
XP_006490346.1/1-238 SGML-IL------QE-TWN-DS-S-G--SLVVYAPVDVPSM-SM----VMN-GG--D---
XP_024958654.1/1-238 SGML-IL------QE-TWN-DS-S-G--SLVVYAPVDVPSM-SM----VMN-GG--D---
XP_006490345.1/1-238 SGML-IL------QE-TWN-DS-S-G--SLVVYAPVDVPSM-SM----VMN-GG--D---
XP_006421879.2/1-238 SGML-IL------QE-TWN-DS-S-G--SLVVYAPVDVPSM-SM----VMN-GG--D---
XP_021892212.1/1-221 NSML-IL------QE-TWS-DA-S-G--AVVVYAPVDISCI-SV----VMG-GG--D---
XP_021892211.1/1-221 NSML-IL------QE-TWS-DA-S-G--AVVVYAPVDISCI-SV----VMG-GG--D---
XP_021892210.1/1-221 NSML-IL------QE-TWS-DA-S-G--AVVVYAPVDISCI-SV----VMG-GG--D---
XP_022765148.1/1-244 NNML-IL------QE-TWS-DV-S-G--ALVVYAPIDISSM-SV----VMN-GG--D---
XP_022765158.1/1-244 NNML-IL------QE-TWS-DV-S-G--ALVVYAPIDISSM-SV----VMN-GG--D---
XP_022765120.1/1-244 NNML-IL------QE-TWS-DV-S-G--ALVVYAPIDISSM-SV----VMN-GG--D---
XP_022765114.1/1-244 NNML-IL------QE-TWS-DV-S-G--ALVVYAPIDISSM-SV----VMN-GG--D---
XP_022736693.1/1-237 NNML-IL------QE-TWS-DA-S-G--ALVVYAPVDMSSM-SV----VMN-GG--D---
XP_022736692.1/1-237 NNML-IL------QE-TWS-DA-S-G--ALVVYAPVDMSSM-SV----VMN-GG--D---
XP_022736689.1/1-237 NNML-IL------QE-TWS-DA-S-G--ALVVYAPVDMSSM-SV----VMN-GG--D---
XP_022734775.1/1-244 NNML-IL------QE-TWC-DP-S-G--ALVVYAPVDVSSM-NV----VMN-GG--D---
XP_016743353.1/1-244 NNML-IL------QE-TWS-DA-S-G--ALVVYAPVDISSM-SV----VMN-GG--D---
XP_012474605.1/1-244 NNML-IL------QE-TWS-DA-S-G--ALVVYAPVDISSM-SV----VMN-GG--D---
XP_012474606.1/1-244 NNML-IL------QE-TWS-DA-S-G--ALVVYAPVDISSM-SV----VMN-GG--D---
XP_016743352.1/1-244 NNML-IL------QE-TWS-DA-S-G--ALVVYAPVDISSM-SV----VMN-GG--D---
XP_016743351.1/1-244 NNML-IL------QE-TWS-DA-S-G--ALVVYAPVDISSM-SV----VMN-GG--D---
XP_012474604.1/1-244 NNML-IL------QE-TWS-DA-S-G--ALVVYAPVDISSM-SV----VMN-GG--D---
XP_012474603.1/1-244 NNML-IL------QE-TWS-DA-S-G--ALVVYAPVDISSM-SV----VMN-GG--D---
XP_017646297.1/1-244 NNML-IL------QE-TWS-DA-S-G--ALVVYAPVDISSM-SV----VMN-GG--D---
XP_016736315.1/1-244 NNML-IL------QE-TWS-DA-S-G--ALVVYAPVDISSM-SV----VMN-GG--D---
XP_017646296.1/1-244 NNML-IL------QE-TWS-DA-S-G--ALVVYAPVDISSM-SV----VMN-GG--D---
XP_017646298.1/1-244 NNML-IL------QE-TWS-DA-S-G--ALVVYAPVDISSM-SV----VMN-GG--D---
XP_016736306.1/1-244 NNML-IL------QE-TWS-DA-S-G--ALVVYAPVDISSM-SV----VMN-GG--D---
XP_017646295.1/1-244 NNML-IL------QE-TWS-DA-S-G--ALVVYAPVDISSM-SV----VMN-GG--D---
XP_017972896.1/1-244 NNML-IL------QE-TWS-DA-S-G--ALVVYAPVDISSI-GV----VMN-GG--D---
XP_017972895.1/1-244 NNML-IL------QE-TWS-DA-S-G--ALVVYAPVDISSI-GV----VMN-GG--D---
XP_017972893.1/1-244 NNML-IL------QE-TWS-DA-S-G--ALVVYAPVDISSI-GV----VMN-GG--D---
XP_021274823.1/1-244 NNML-IL------QE-TWS-DA-S-G--GIVVYAPVDISSI-SV----VMN-GG--D---
XP_024536343.1/1-231 SNML-IL------QE-SST-DE-S-G--SLIVYAPVDIPAM-NL----VMQ-GG--D---
XP_006366174.1/1-244 -NML-IL------QE-TCT-DA-S-G--SLVVYAPVDIPSM-HL----VMN-GG--D---
XP_020219711.1/1-212 SSML-IL------QE-TCT-DA-S-G--SLVVYAPVDIPAM-HV----VMN-GG--D---
XP_022897056.1/1-222 NSML-IL------QE-TCI-DE-T-G--SLAVYAPVDIPAM-HV----VMN-GG--D---
NP_001281015.1/1-243 GSML-IL------QE-TRI-DA-A-G--SLVVYAPVDIPAM-HV----VMN-GG--D---
XP_008383260.1/1-243 GSML-IL------QE-TRI-DA-A-G--SLVVYAPVDIPAM-HV----VMN-GG--D---
XP_022896229.1/1-235 RSML-IL------QE-TCI-GP-A-G--SLVVYAPVDIPAM-QV----VMN-GG--D---
XP_022896230.1/1-235 RSML-IL------QE-TCI-GP-A-G--SLVVYAPVDIPAM-QV----VMN-GG--D---
XP_025816451.1/1-225 SSML-IL------QE-TCT-DA-S-G--SMVVYAPVDIPAM-QL----VMN-GG--D---
XP_004968001.1/1-225 SSML-IL------QE-TCT-DA-S-G--SMVVYAPVDIPAM-QL----VMN-GG--D---
XP_004968002.1/1-225 SSML-IL------QE-TCT-DA-S-G--SMVVYAPVDIPAM-QL----VMN-GG--D---
XP_002457484.1/1-226 SSML-IL------QE-TCT-DA-S-G--SMVVYAPVDIPAM-QL----VMN-GG--D---
XP_009400446.1/1-224 SSML-IL------QE-TCT-DA-S-G--SLVVYAPVDVPAM-HL----VMS-GG--D---
XP_018676535.1/1-225 SSML-IL------QE-TCT-DA-S-G--SLVVYAPVDIPAM-HL----VMS-GG--D---
XP_009401771.1/1-232 SSML-IL------QE-TCT-DT-S-G--SLVVYAPVDIPAM-HL----VMS-GG--D---
XP_009384534.1/1-228 SSML-IL------QE-TCT-DA-S-G--SLVVYAPVDTPAM-HL----VMS-GG--D---
XP_020672447.1/1-217 NSML-IL------QE-TCT-DA-S-G--SMVVYAPVDVPAM-HL----VMN-GG--D---
XP_020582116.1/1-218 SSML-IL------QE-TCT-DA-S-G--SMVVYAPVDVPAM-HH----VMN-GG--D---
XP_020675647.1/1-231 SSML-IL------QE-TCT-DA-S-G--SMVVYAPVDVPAM-HL----VMN-GG--D---
XP_020675648.1/1-231 SSML-IL------QE-TCT-DA-S-G--SMVVYAPVDVPAM-HL----VMN-GG--D---
XP_020585632.1/1-228 SSML-IL------QE-TCT-DE-S-G--SMVVYAPVDVPAM-HV----VMN-GG--D---
XP_016176402.1/1-228 SSMV-IL------QE-TWT-DA-S-C--SVVVYAPVDVPSL-NV----VMS-GG--D---
XP_016176403.1/1-228 SSMV-IL------QE-TWT-DA-S-C--SVVVYAPVDVPSL-NV----VMS-GG--D---
XP_016176400.1/1-228 SSMV-IL------QE-TWT-DA-S-C--SVVVYAPVDVPSL-NV----VMS-GG--D---
XP_016176401.1/1-228 SSMV-IL------QE-TWT-DA-S-C--SVVVYAPVDVPSL-NV----VMS-GG--D---
XP_010523042.1/1-245 SSML-IL------QE-TRI-DA-S-G--ALVVYAPVDIPAM-HV----VMN-GG--D---
XP_010523041.1/1-240 SSML-IL------QE-TRI-DA-S-G--ALVVYAPVDIPAM-HV----VMN-GG--D---
XP_020878511.1/1-224 SSML-IL------QE-TCI-DA-S-G--ALVVYAPVDIPAM-HV----VMN-GG--D---
NP_567183.2/1-228    SSML-IL------QE-TCI-DA-S-G--ALVVYAPVDIPAM-HV----VMN-GG--D---
XP_006287095.1/1-228 SCML-IL------QE-TCI-DS-S-G--ALVVYAPVDIPAM-NV----VMN-GG--D---
XP_010456250.1/1-228 SSML-IL------QE-TCI-DA-S-G--ALVVYAPVDIPAM-HV----VMN-GG--D---
XP_010419627.1/1-228 SSML-IL------QE-TCI-DA-S-G--ALVVYAPVDIPAM-HV----VMN-GG--D---
XP_010427345.1/1-228 SSML-IL------QE-TCI-DA-S-G--ALVVYAPVDIPAM-HV----VMN-GG--D---
XP_009111469.1/1-229 SSML-IL------QE-TCI-DA-S-G--ALVVYAPVDIPAM-HV----VMN-GG--D---
XP_018487039.1/1-229 SSML-II------QE-TCI-DA-S-G--AMVVYAPVDIPAM-HV----VMN-GG--D---
XP_018453129.1/1-229 SSML-IL------QE-TCI-DA-S-G--AMVVYAPVDIPAM-HV----VMN-GG--D---
XP_013612299.1/1-229 SSML-IL------QE-TCI-DA-S-G--ALVVYAPVDIPAM-HV----VMN-GG--D---
XP_006396268.1/1-225 SSML-IL------QE-TCI-DA-S-G--ALVVYAPVDIPAM-NV----VMN-GG--E---
XP_018475317.1/1-221 SSML-IL------QE-TCI-DA-S-G--ALVVYAPVDIPAM-NV----VMN-GG--D---
XP_013629750.1/1-221 SSML-IL------QE-TCI-DA-S-G--ALVVYAPVDIPAM-NV----VMN-GG--D---
XP_009134533.1/1-221 SSML-IL------QE-TCI-DA-S-G--ALVVYAPVDIPAM-NV----VMN-GG--D---
XP_020531377.1/1-244 SSML-IL------QE-TCT-DP-S-G--ALVVYAPVDIPAM-HV----VMS-GG--D---
XP_006857866.1/1-244 SSML-IL------QE-TCT-DP-S-G--ALVVYAPVDIPAM-HV----VMS-GG--D---
XP_018434263.1/1-219 SSML-IL------QE-TSI-DA-S-G--ATVVYAPVDIPAM-HS----VMN-GG--D---
XP_018434260.1/1-219 SSML-IL------QE-TSI-DA-S-G--ATVVYAPVDIPAM-HS----VMN-GG--D---
XP_018434262.1/1-219 SSML-IL------QE-TSI-DA-S-G--ATVVYAPVDIPAM-HS----VMN-GG--D---
XP_013688926.1/1-219 SSML-IL------QE-TSI-DA-S-G--AVVVYAPVDIPAM-HA----VMN-GG--D---
XP_013637558.1/1-219 SSML-IL------QE-TSI-DA-S-G--AVVVYAPVDIPAM-HA----VMN-GG--D---
XP_013637556.1/1-219 SSML-IL------QE-TSI-DA-S-G--AVVVYAPVDIPAM-HA----VMN-GG--D---
XP_013637557.1/1-219 SSML-IL------QE-TSI-DA-S-G--AVVVYAPVDIPAM-HA----VMN-GG--D---
XP_009143168.1/1-219 SSML-IL------QE-TSI-DA-S-G--AVVVYAPVDIPAM-HS----VMN-GG--D---
XP_009143167.1/1-219 SSML-IL------QE-TSI-DA-S-G--AVVVYAPVDIPAM-HS----VMN-GG--D---
XP_009143166.1/1-219 SSML-IL------QE-TSI-DA-S-G--AVVVYAPVDIPAM-HS----VMN-GG--D---
XP_013692686.1/1-219 SSML-IL------QE-TSI-DA-S-G--AVVVYAPVDIPAM-HS----VMN-GG--D---
XP_022574820.1/1-219 SSML-IL------QE-TSI-DA-S-G--AVVVYAPVDIPAM-HS----VMN-GG--D---
XP_013692684.1/1-219 SSML-IL------QE-TSI-DA-S-G--AVVVYAPVDIPAM-HS----VMN-GG--D---
XP_013748127.1/1-219 SSML-IL------QE-TSI-DA-S-G--AVVVYAPVDIPAM-HS----VMN-GG--D---
XP_013692685.1/1-219 SSML-IL------QE-TSI-DA-S-G--AVVVYAPVDIPAM-HS----VMN-GG--D---
XP_013748124.1/1-219 SSML-IL------QE-TSI-DA-S-G--AVVVYAPVDIPAM-HS----VMN-GG--D---
XP_013748126.1/1-219 SSML-IL------QE-TSI-DA-S-G--AVVVYAPVDIPAM-HS----VMN-GG--D---
XP_022574821.1/1-219 SSML-IL------QE-TSI-DA-S-G--AVVVYAPVDIPAM-HS----VMN-GG--D---
XP_006290613.1/1-229 SSML-IL------QE-TSI-DA-A-G--AVVVYAPVDIPAM-QA----VMN-GG--D---
XP_013592301.1/1-230 SSML-IL------QE-TSI-DA-A-G--AVVVYAPVDIPAM-QT----VMN-GG--D---
XP_018442581.1/1-230 SSML-IL------QE-TSI-DA-A-G--AVVVYAPVDIPAM-QA----VMN-GG--D---
XP_013750213.1/1-232 SSML-IL------QE-TSI-DA-V-G--AVVVYAPVDIPAM-QA----VMN-GG--D---
XP_013648430.1/1-232 SSML-IL------QE-TSI-DA-A-G--AVVVYAPVDIPAM-QA----VMN-GG--D---
XP_009104292.1/1-232 SSML-IL------QE-TSI-DA-A-G--AVVVYAPVDIPAM-QA----VMN-GG--D---
XP_019058514.1/1-223 SSML-IL------QE-TSI-DP-A-G--ALVVYAPVDIPAM-HV----VMN-GG--D---
XP_010523728.1/1-220 SSML-VL------QE-TCI-DT-A-G--ALVVYAPVDIPAM-HV----VMN-GG--D---
XP_024514927.1/1-242 SNML-IL------QE-SCT-DA-S-G--SLIVYAPVDIPAM-NL----VMQ-GG--D---
XP_002985242.1/1-242 SNML-IL------QE-SCT-DA-S-G--SLIVYAPVDIPAM-NL----VMQ-GG--D---
XP_024530500.1/1-242 SNML-IL------QE-SCT-DA-S-G--SLIVYAPVDIPAM-NL----VMQ-GG--D---
XP_024530499.1/1-242 SNML-IL------QE-SCT-DA-S-G--SLIVYAPVDIPAM-NL----VMQ-GG--D---
XP_024530501.1/1-242 SNML-IL------QE-SCT-DA-S-G--SLIVYAPVDIPAM-NL----VMQ-GG--D---
XP_010107411.1/1-242 SSML-IL------QE-TWN-DA-S-G--SMVVYAPVDRASI-NV----VMH-GG--D---
XP_024028635.1/1-242 SSML-IL------QE-TWN-DA-S-G--SMVVYAPVDRASI-NV----VMH-GG--D---
XP_015877999.1/1-244 STML-IL------QE-TWS-DA-S-C--SLVVYAPVDTASI-NV----VMH-GG--E---
XP_019427294.1/1-247 SNML-IL------QE-TWM-DS-S-C--SVVVYAPVDVQSL-NV----VMS-GG--D---
XP_019427292.1/1-247 SNML-IL------QE-TWM-DS-S-C--SVVVYAPVDVQSL-NV----VMS-GG--D---
XP_019452814.1/1-239 SSML-IL------QE-TWT-DS-S-C--SVVVYAPVDVQSL-NV----VMS-GG--D---
XP_019452815.1/1-239 SSML-IL------QE-TWT-DS-S-C--SVVVYAPVDVQSL-NV----VMS-GG--D---
XP_003592295.2/1-235 SSML-IL------QE-TWM-DT-S-C--SVVVYAPVDGQSL-NV----VMS-GG--D---
XP_024625328.1/1-235 SSML-IL------QE-TWM-DT-S-C--SVVVYAPVDGQSL-NV----VMS-GG--D---
XP_012570175.1/1-236 SSML-IL------QE-TWM-DT-S-C--SVVVYAPVDAQSL-NV----VMS-GG--D---
XP_012570176.1/1-236 SSML-IL------QE-TWM-DT-S-C--SVVVYAPVDAQSL-NV----VMS-GG--D---
XP_004496593.1/1-236 SSML-IL------QE-TWM-DT-S-C--SVVVYAPVDAQSL-NV----VMS-GG--D---
XP_025620154.1/1-234 SSMV-IL------QE-TWT-DA-S-C--SVVVYAPVDVPSL-NV----VMS-GG--D---
XP_015941562.1/1-234 SSMV-IL------QE-TWT-DA-S-C--SVVVYAPVDVPSL-NV----VMS-GG--D---
XP_025620153.1/1-234 SSMV-IL------QE-TWT-DA-S-C--SVVVYAPVDVPSL-NV----VMS-GG--D---
XP_020239673.1/1-232 SSML-IL------QE-TWM-DA-S-C--SVVVYAPVDVQSL-NV----VMS-GG--D---
XP_020239648.1/1-232 SSML-IL------QE-TWM-DA-S-C--SVVVYAPVDVQSL-NV----VMS-GG--D---
XP_007143377.1/1-235 SSML-IL------QE-TWM-DA-S-C--SVVVYAPVDVQSL-NV----VMS-GG--D---
XP_007143378.1/1-235 SSML-IL------QE-TWM-DA-S-C--SVVVYAPVDVQSL-NV----VMS-GG--D---
XP_017415467.1/1-234 SSML-IL------QE-TWM-DA-S-C--SVVVYAPVDVQSL-NV----VMS-GG--D---
XP_017415466.1/1-234 SSML-IL------QE-TWM-DA-S-C--SVVVYAPVDVQSL-NV----VMS-GG--D---
XP_014514421.1/1-236 SSML-IL------QE-TWM-DA-S-C--SVVVYAPVDVQSL-NV----VMS-GG--D---
XP_014514419.1/1-236 SSML-IL------QE-TWM-DA-S-C--SVVVYAPVDVQSL-NV----VMS-GG--D---
XP_006606097.1/1-235 SSML-IL------QE-TWM-DA-S-C--SVVVYAPVDVQSL-NV----VMS-GG--D---
XP_006606096.1/1-235 SSML-IL------QE-TWM-DA-S-C--SVVVYAPVDVQSL-NV----VMS-GG--D---
XP_003536477.1/1-234 SSML-IL------QE-TWM-DA-S-C--SVVVYAPVDVQSL-NV----VMS-GG--D---
XP_006589557.1/1-234 SSML-IL------QE-TWM-DA-S-C--SVVVYAPVDVQSL-NV----VMS-GG--D---
XP_006402516.1/1-230 SSML-IL------QE-TSI-DA-A-G--AIVVYAPVDIPAM-QA----VMN-GG--D---
NP_191674.1/1-237    SSML-IL------QE-TSI-DA-A-G--AVVVYAPVDIPAM-QA----VMN-GG--D---
XP_002876596.1/1-239 SSML-IL------QE-TSI-DA-A-G--ALVVYAPVDIPAM-QA----VMN-GG--D---
XP_010413451.1/1-238 SSMV-IL------QE-TSI-DA-A-G--AVVVYAPVDILAM-QA----VMN-GG--D---
XP_010413452.1/1-238 SSMV-IL------QE-TSI-DA-A-G--AVVVYAPVDILAM-QA----VMN-GG--D---
XP_010413453.1/1-238 SSMV-IL------QE-TSI-DA-A-G--AVVVYAPVDILAM-QA----VMN-GG--D---
XP_010469036.1/1-242 SSML-IL------QE-TSI-DA-A-G--ALVVYAPVDIPAM-QA----VMN-GG--D---
XP_010469037.1/1-242 SSML-IL------QE-TSI-DA-A-G--ALVVYAPVDIPAM-QA----VMN-GG--D---
XP_010469038.1/1-242 SSML-IL------QE-TSI-DA-A-G--ALVVYAPVDIPAM-QA----VMN-GG--D---
XP_010512448.1/1-238 SSML-IL------QE-TSI-DA-A-G--AVVVYAPVDIPAM-QA----VMN-GG--D---
XP_010512449.1/1-238 SSML-IL------QE-TSI-DA-A-G--AVVVYAPVDIPAM-QA----VMN-GG--D---
XP_006397757.1/1-219 SCML-IL------QE-TSI-DA-S-G--AFVVYAPVDIPAM-HS----VMN-GA--D---
XP_024010837.1/1-220 SCML-IL------QE-TSI-DA-S-G--AFVVYAPVDIPAM-HS----VMN-GA--D---
XP_023640300.1/1-220 SSML-IL------QE-TSI-DA-S-G--AVVVYAPVDIPAM-HS----VMK-GA--D---
XP_006293718.1/1-220 SSML-IL------QE-TSI-DA-S-G--AVVVYAPVDIPAM-HS----VMK-GA--D---
XP_010506553.1/1-219 SSML-IL------QE-TSI-DA-S-G--AVVVHAPVDIPAM-HS----VMK-GA--D---
XP_010508114.1/1-219 SSML-IL------QE-TSI-DA-S-G--AVVVYAPVDIPAM-HS----VMK-GA--D---
XP_010508115.1/1-219 SSML-IL------QE-TSI-DA-S-G--AVVVYAPVDIPAM-HS----VMK-GA--D---
XP_022147276.1/1-219 SSML-IL------QE-TCT-DP-S-G--SLVVYAPVDIPAM-QV----VMN-GG--D---
XP_023527464.1/1-222 SSML-IL------QE-TCS-DS-S-G--SLVVYAPVDIPAM-QV----VMN-GG--D---
XP_022980233.1/1-222 SSML-IL------QE-TCS-DA-S-G--SLVVYAPVDIPAM-QV----VMN-GG--D---
XP_022980231.1/1-222 SSML-IL------QE-TCS-DA-S-G--SLVVYAPVDIPAM-QV----VMN-GG--D---
XP_022924366.1/1-223 SSML-IL------QE-TCS-DA-S-G--SLVVYAPVDIPAM-QV----VMN-GG--D---
XP_022924364.1/1-223 SSML-IL------QE-TCS-DA-S-G--SLVVYAPVDIPAM-QV----VMN-GG--D---
XP_023527467.1/1-221 SSML-IL------QE-TCS-DS-S-G--SLVVYAPVDIPAM-QV----VMN-GG--D---
XP_023527463.1/1-221 SSML-IL------QE-TCS-DS-S-G--SLVVYAPVDIPAM-QV----VMN-GG--D---
XP_023527465.1/1-221 SSML-IL------QE-TCS-DS-S-G--SLVVYAPVDIPAM-QV----VMN-GG--D---
XP_022980232.1/1-221 SSML-IL------QE-TCS-DA-S-G--SLVVYAPVDIPAM-QV----VMN-GG--D---
XP_022924365.1/1-222 SSML-IL------QE-TCS-DA-S-G--SLVVYAPVDIPAM-QV----VMN-GG--D---
XP_009412599.1/1-224 TSML-IL------QE-TCT-DA-S-G--AMVVYAPVDIPAM-HL----VMS-GG--D---
XP_018677084.1/1-231 NSML-IL------QE-TCT-DA-S-G--SVVVYAPVDVPAM-HL----VMS-GG--D---
XP_020114860.1/1-246 SSML-IL------QE-TCT-DA-S-G--SLVVYAPVDVPAM-HL----VMN-GG--D---
XP_020114857.1/1-246 SSML-IL------QE-TCT-DA-S-G--SLVVYAPVDVPAM-HL----VMN-GG--D---
XP_020114859.1/1-246 SSML-IL------QE-TCT-DA-S-G--SLVVYAPVDVPAM-HL----VMN-GG--D---
XP_020084803.1/1-234 SSML-IL------QE-TCT-DA-S-G--SMVVYAPVDIPAM-HL----LMN-GG--D---
XP_015625616.1/1-226 SSML-IL------QE-TCT-DA-S-G--SIVVYAPVDIPAM-QL----VMN-GG--D---
XP_006647677.1/1-226 SSML-IL------QE-TCT-DA-S-G--SIVVYAPVDIPAM-QL----VMN-GG--D---
NP_001105493.1/1-226 SSML-IL------QE-TCT-DA-S-G--SMVVYAPVDIPAM-QL----VMN-GG--D---
XP_020178635.1/1-225 SSML-IL------QE-TCT-DA-S-G--SIVVYAPVDIPAM-QL----VMN-GG--D---
XP_003570085.1/1-223 SSML-IL------QE-TCT-DA-S-G--SIVVYAPVDIPAM-QL----VME-GR--D---
XP_020247871.1/1-226 SVEP-LL------QE-SSTHD--S-GG-SIIVYAAIDVHGV-QV---A-MS--G-ED---
XP_020247870.1/1-226 SVEP-LL------QE-SSTHD--S-GG-SIIVYAAIDVHGV-QV---A-MS--G-ED---
XP_024004764.1/1-211 NVEL-VL------QE-TFT-D--I-SG-SLLVYSTVDVKTV-QL---V-LN--G-KD---
XP_018449374.1/1-217 NVEW-ML------QE-TCT-D--S-SG-SLVVYSTVDASAV-HL---A-MN--G-ED---
XP_013602839.1/1-215 NVEW-ML------QE-TCT-D--N-SG-SLVVYSTVHANAV-QL---A-MN--G-ED---
XP_013655966.1/1-215 NVEW-ML------QE-TCT-D--N-SG-SLVVYSTVHANAV-QL---A-MN--G-ED---
XP_009108342.1/1-215 NVEW-ML------QE-TCT-D--N-SG-SLVVYSTVHANAV-QL---A-MS--G-ED---
XP_013655964.1/1-215 NVEW-ML------QE-TCT-D--N-SG-SLVVYSTVHANAV-QL---A-MS--G-ED---
XP_020872468.1/1-222 NVEL-ML------QE-TCT-D--N-SG-SLLVYSTVEPDAV-QL---AMMN--G-ED---
NP_193506.2/1-221    NVEL-ML------QE-TCT-D--N-SG-SLLVYSTVDPVAV-QL---A-MN--G-ED---
XP_023633855.1/1-222 NVEL-ML------QE-TCT-D--S-SG-SLLVYSTVDADAV-QL---A-MN--G-ED---
XP_010449572.1/1-221 NVEL-ML------QE-TCT-D--N-SG-SLLVFSTVDADAV-QL---A-MN--G-KD---
XP_010439957.1/1-223 NVEL-ML------QE-TCT-D--D-SG-SLLVFSTVDADAF-QL---A-MN--G-KD---
XP_010439959.1/1-223 NVEL-ML------QE-TCT-D--D-SG-SLLVFSTVDADAF-QL---A-MN--G-KD---
XP_010439958.1/1-223 NVEL-ML------QE-TCT-D--D-SG-SLLVFSTVDADAF-QL---A-MN--G-KD---
XP_006854056.1/1-234 NVEL-ML------QE-SCTDP--S--A-SLVIYAPIDISSV-QM---A-VT--S-ED---
XP_020529072.1/1-234 NVEL-ML------QE-SCTDP--S--A-SLVIYAPIDISSV-QM---A-VT--S-ED---
NP_199499.3/1-224    NVEL-ML------QE-SCIDN--S--G-SLIVYSTVDVDSI-QQ---A-MN--G-ED---
XP_020871346.1/1-224 NVEL-ML------QE-SCIDN--S--G-SLIVYSTVDVDSI-QL---A-MN--G-ED---
XP_006281890.1/1-224 NVEL-ML------QE-SSIDN--S--G-SLIVYSTVDVDSV-QL---A-MN--G-ED---
XP_010481400.1/1-224 NVEL-ML------QE-SSIDN--S--G-SLIVYSTVDVDSI-QL---A-MN--G-ED---
XP_010494885.1/1-224 NVEL-ML------QE-SSIDN--S--G-SLIVYSTVDVDSI-QL---A-MN--G-ED---
XP_018455882.1/1-227 NVEL-ML------QE-SCVDN--S--G-GLIVYSSVNVDSI-QH---V-MN--G-ED---
XP_013713146.1/1-227 NVEL-ML------QE-SCVDN--S--G-GLIVYSSVDVVPI-QH---V-MN--I-ED---
XP_013607209.1/1-227 NVEL-ML------QE-SCVDN--S--G-GLIVYSSVDVVPI-QH---V-MN--I-ED---
XP_009114317.1/1-227 NVEL-ML------QE-SCIDN--S--G-GLIVYSTVGVDSI-QH---V-MN--G-ED---
XP_013661958.1/1-227 NVEL-ML------QE-SCIDN--S--G-GLIVYSTVGVESI-QH---V-MN--G-ED---
XP_006398384.1/1-227 NVEL-ML------QE-SCIDN--S--G-SLIVYSSVDVDSI-QH---A-MN--G-ED---
XP_024011152.1/1-227 NVEL-ML------QE-SCIDN--S--G-SLIVYSSVDVDSI-QH---A-MN--G-ED---
XP_018482944.1/1-227 NVEL-ML------QE-SCIDN--S--G-SLIVYSSVDVDSI-QH---A-MN--G-ED---
XP_009101472.1/1-227 NVEL-ML------QE-SCIDN--S--G-SLIVYSSVDVDSI-KH---A-MN--G-ED---
XP_013693979.1/1-227 NVEL-ML------QE-NCIDN--S--G-SLIVYSSVDVDSI-KH---A-MN--G-KD---
XP_013615089.1/1-227 NVDL-ML------QE-SCIDN--S--G-SLIVYSSVDVDYI-QH---A-MN--G-ED---
XP_013704013.1/1-227 NVDL-ML------QE-SCIDN--S--G-SLIVYSSVDVDYI-QH---A-MN--G-ED---
XP_009373477.1/1-246 NIEI-ML------QE-SCTDE----SG-SLVVYATMDVDGI-QL---A-MS--G-ED---
XP_006662631.2/1-256 NVEL-LL------QE-SSTHP--D-GG-SLVVFATVDVDAI-QV---T-MS--G-ED---
XP_015613461.1/1-259 NVEL-LL------QE-SSTHP--D-GG-SLVVFATVDVDAI-QV---T-MS--G-ED---
NP_001337805.1/1-240 NVEL-ML------QE-SSTHP--D-GG-SLVVFATVDVDAI-QV---T-MS--G-ED---
XP_020163828.1/1-239 NVEL-LL------QE-SSIHP--D-GG-SLVVFATVDVDAI-QV---T-MS--G-ED---
XP_010235052.1/1-238 NVEL-LL------QE-TSTHP--D-GG-SLVVFATVDVDAI-QV---T-MS--G-ED---
XP_025798256.1/1-238 NVEL-LL------QE-SSTHP--N-GG-SLVVFATVDVDAI-QV---T-MS--G-ED---
XP_004983555.1/1-238 NVEL-LL------QE-SSTHP--D-GG-SLVVFATVDVDAI-QV---T-MS--G-ED---
XP_002464880.2/1-238 NVEL-ML------QE-TSTHP--D-GG-SLVVFATVDVDAI-QV---T-MS--G-ED---
XP_020262583.1/1-231 SVEL-LL------QE-SSIHP--S-AE-STVVYATVDVDAV-QV---A-MS--G-ED---
XP_020677410.1/1-231 NVEL-LL------QE-SCTHP--S-GG-SLVVYATIDVDAV-QV---A-MN--G-DD---
XP_020577604.1/1-229 KVEL-LL------QE-SRTHP--S-GG-SLIVYATIDVGAV-QL---V-TN--G-ED---
XP_020577606.1/1-229 KVEL-LL------QE-SRTHP--S-GG-SLIVYATIDVGAV-QL---V-TN--G-ED---
XP_020577607.1/1-229 KVEL-LL------QE-SRTHP--S-GG-SLIVYATIDVGAV-QL---V-TN--G-ED---
XP_019709148.1/1-245 NVEL-LL------QE-SSTHP--S-GG-SLVVYATVDVDAV-QV---A-MS--G-ED---
XP_008803567.1/1-241 NMEL-LL------QE-SSTHP--S-GG-SHVVYATIDVDAV-QV---A-MS--G-ED---
XP_008803570.2/1-241 NMEL-LL------QE-SSTHP--S-GG-SHVVYATIDVDAV-QV---A-MS--G-ED---
XP_018681950.1/1-231 SVDL-LL------QE-SSTHP--S-GG-SIVVYAAIDVDAV-QV---A-MS--S-ED---
XP_018681951.1/1-231 SVDL-LL------QE-SSTHP--S-GG-SIVVYAAIDVDAV-QV---A-MS--S-ED---
XP_010919976.1/1-237 NVEL-LL------QE-SSTHP--S-GG-SLVVYATIDVDAV-QV---A-MS--G-ED---
XP_008800084.1/1-237 NVEL-LL------QE-SSTHP--S-GG-SLVVYATIDVDAV-QV---A-MS--G-ED---
XP_018679997.1/1-229 SVEL-LL------QE-SSTHP--S-GG-SIVVYATIDVDAV-QV---A-MS--G-ED---
XP_009397149.1/1-229 SVEL-LL------QE-SSTHP--S-GG-SIVVYATIDVDAV-QV---A-MS--G-ED---
XP_024995523.1/1-235 NVEL-VL------QE-SFTDD--S--G-SLVAYSIVDVDAI-KL---T-MN--G-ED---
XP_023736252.1/1-233 NVEL-VL------QE-SCTDD--S--G-SLVVYSVVDVDAI-QH---T-MS--G-ED---
XP_023736253.1/1-233 NVEL-VL------QE-SCTDD--S--G-SLVVYSVVDVDAI-QH---T-MS--G-ED---
XP_022022041.1/1-231 NVEL-VL------QE-SCTDD--S--G-SLVVYSTVEVDAI-KL---T-MN--G-GD---
XP_021969857.1/1-226 NVEL-VL------QE-SCTDD--S--G-SLVVYSVVDVDAI-KL---T-MS--G-ED---
XP_010245213.1/1-236 NVEL-ML------QE-SCTDP--S--G-SLIVYTTMDVDAV-QL---A-MS--G-ED---
XP_010245210.1/1-236 NVEL-ML------QE-SCTDP--S--G-SLIVYTTMDVDAV-QL---A-MS--G-ED---
XP_010276832.1/1-235 NVEL-ML------QE-SCTDT--S--G-SLIVYSTMDVDAV-QL---A-MS--G-ED---
XP_010276837.1/1-235 NVEL-ML------QE-SCTDT--S--G-SLIVYSTMDVDAV-QL---A-MS--G-ED---
XP_019155823.1/1-230 SVEL-ML------QE-SCADD--S--G-SLVVYATMDVDAI-QL---A-MS--G-DD---
XP_022881662.1/1-238 NVEL-ML------QE-SCTDD--S--G-SILVYTTIDVDSI-QL---A-MN--G-ED---
XP_022877113.1/1-234 NVEL-ML------QE-SCTDD--S--G-SIVVYTTIDVDSI-QL---A-MN--G-ED---
XP_016450789.1/1-246 SVEL-ML------QE-SCTDD--S--G-SLVVYTTVDVDAI-QL---A-MN--G-ED---
XP_009597691.1/1-246 SVEL-ML------QE-SCTDD--S--G-SLVAYTTVDVDAI-QL---A-MN--G-ED---
XP_009786380.1/1-246 SVEL-ML------QE-SCTDD--S--G-SLVVYTTVDVDAI-QL---A-MN--G-ED---
XP_016494823.1/1-246 SVEL-ML------QE-SCTDD--S--G-SLVVYTTVDVDAI-QL---A-MN--G-ED---
XP_016494822.1/1-246 SVEL-ML------QE-SCTDD--S--G-SLVVYTTVDVDAI-QL---A-MN--G-ED---
XP_019230103.1/1-246 SVEL-LL------QE-SCTDD--S--G-SLVVYTTVDVDAI-QL---A-MN--G-EA---
XP_016573090.1/1-250 SVEL-ML------QE-SCTDD--S--G-SLVAYTTVEVDAI-QL---A-MN--G-ED---
XP_016573102.1/1-250 SVEL-ML------QE-SCTDD--S--G-SLVAYTTVEVDAI-QL---A-MN--G-ED---
XP_006352911.1/1-250 SVEL-ML------QE-SCTDD--S--G-SLVVYTTVDVDAI-QL---A-MN--G-ED---
XP_015166604.1/1-250 SVEL-ML------QE-SCTDD--S--G-SLVVYTTVDVDAI-QL---A-MN--G-ED---
XP_015085651.1/1-250 SVEL-ML------QE-SCTDD--S--G-SLVVYTTVDVDAI-QL---A-MN--G-ED---
XP_015085654.1/1-250 SVEL-ML------QE-SCTDD--S--G-SLVVYTTVDVDAI-QL---A-MN--G-ED---
XP_004245919.1/1-250 SVEL-ML------QE-SCTDD--S--G-SLVVYTTVDVDAI-QL---A-MN--G-ED---
XP_025888165.1/1-250 SVEL-ML------QE-SCTDD--S--G-SLVVYTTVDVDAI-QL---A-MN--G-ED---
XP_011070327.1/1-236 SVEL-VL------QE-SCSDD--S--G-SIVVYTTIDVDAI-QM---A-MN--G-ED---
XP_011070329.1/1-236 SVEL-VL------QE-SCSDD--S--G-SIVVYTTIDVDAI-QM---A-MN--G-ED---
XP_012843107.1/1-236 SVEL-VL------QE-SCSDD--S--G-SVVVYTTIDVDAI-QM---A-MN--G-ED---
XP_019423575.1/1-234 NVEL-ML------QE-SCTDQ--S--G-SLVVYTTIDVDSI-QL---A-MS--G-ED---
XP_019423571.1/1-234 NVEL-ML------QE-SCTDQ--S--G-SLVVYTTIDVDSI-QL---A-MS--G-ED---
XP_019435331.1/1-234 NVEL-ML------QE-SCTDQ--S--G-SIVVYTTIDVDSI-QL---A-MN--G-ED---
XP_003612811.1/1-239 NVEL-ML------QE-SCTDQ--S--G-SLVVYTTVDVDSV-QL---A-MS--G-QD---
XP_020978415.1/1-227 NVEL-ML------QE-SCTDQ--S--G-SVVVYTTIAVDSI-QV---A-MS--G-ED---
XP_015963259.1/1-227 NVEL-ML------QE-SCTDQ--S--G-SVVVYTTIAVDSI-QV---A-MS--G-ED---
XP_025697880.1/1-227 NVEL-ML------QE-SCTDQ--S--G-SVVVYTTIAVDSI-QV---A-MS--G-ED---
XP_025697882.1/1-227 NVEL-ML------QE-SCTDQ--S--G-SVVVYTTIAVDSI-QV---A-MS--G-ED---
XP_025697881.1/1-227 NVEL-ML------QE-SCTDQ--S--G-SVVVYTTIAVDSI-QV---A-MS--G-ED---
XP_020217931.1/1-233 NVEL-ML------QE-SCTDQ--S--G-SLVVYTTIDVDSI-QL---A-MS--G-ED---
XP_007158253.1/1-232 NVEL-ML------QE-SCTDQ--S--G-SLVVYTTIDVDSI-QL---A-MS--G-ED---
XP_017427665.1/1-232 NVEL-ML------QE-SCTDQ--S--G-SLVVYTTIDVDSI-QL---A-MS--G-ED---
XP_017427666.1/1-232 NVEL-ML------QE-SCTDQ--S--G-SLVVYTTIDVDSI-QL---A-MS--G-ED---
XP_014521371.1/1-232 NVEL-ML------QE-SCTDQ--S--G-SLVVYTTIDVDSI-QL---A-MS--G-ED---
XP_014521373.1/1-232 NVEL-ML------QE-SCTDQ--S--G-SLVVYTTIDVDSI-QL---A-MS--G-ED---
XP_006587604.1/1-242 NVEL-ML------QE-NCTDQ--S--G-SIVVYTTIDVDSI-QL---A-MS--G-ED---
NP_001352051.1/1-233 NVEL-ML------QE-SCTDQ--S--G-SLVVYTTIDVDAI-QL---A-MS--G-ED---
XP_018840870.1/1-239 HVEL-ML------QE-SCTDD--S--G-SLVVYTTIDVDSI-QL---A-MS--G-ED---
XP_018852246.1/1-240 HVEL-ML------QE-SCTDP--S--G-SLVVYTTIDVDSI-QH---A-MS--G-ED---
XP_010053633.1/1-234 HVEL-ML------QE-SCTDA--S--G-SLVVYSTIDVDSI-QL---A-MN--G-ED---
XP_021675948.1/1-236 NVEL-ML------QE-SCTDQ--S--G-SLVVYTTVNVDSI-QL---A-MS--G-ED---
XP_012081064.1/1-228 HVEL-ML------QE-SCTDQ--S--G-SLIVYTTVNVDSI-QL---A-MN--G-ED---
XP_011028126.1/1-235 HVEL-ML------QE-CCTDQ--S--G-SLVVYTTVDVESI-QL---A-MS--G-ED---
XP_002303433.2/1-235 HVEL-ML------QE-CCTDQ--S--G-SLVVYTTVDVESI-QL---A-MS--G-ED---
XP_015897558.1/1-247 HVEL-ML------QE-SCTDQ--S--G-SLVVYTTIDVDAI-QL---A-MS--G-ED---
XP_002277673.1/1-238 NVEL-ML------QE-SCTDQ--S--G-SHVVYTTIDVDAI-QL---A-MS--G-ED---
XP_022996123.1/1-236 HVEL-ML------QE-SCTDQ--S--G-SLVVFAMIDVDSI-QL---A-MS--G-ED---
XP_023534245.1/1-236 HVEL-ML------QE-SCTDQ--S--G-SLVVFATIDVDSI-QL---A-MS--G-ED---
XP_022931244.1/1-236 HVEL-ML------QE-SCTDQ--S--G-SLVVFATIDVDSI-QL---A-MS--G-ED---
XP_022931242.1/1-236 HVEL-ML------QE-SCTDQ--S--G-SLVVFATIDVDSI-QL---A-MS--G-ED---
XP_022159691.1/1-235 HVEL-ML------QE-SCTDQ--S--G-SLVVYSTIDVDSI-QL---A-MS--G-ED---
XP_011652640.1/1-232 HVEL-ML------QE-SCTDQ--S--G-SLVVYATIDVDSI-QL---A-MS--G-ED---
XP_011652639.1/1-232 HVEL-ML------QE-SCTDQ--S--G-SLVVYATIDVDSI-QL---A-MS--G-ED---
XP_008466007.1/1-232 HVEL-ML------QE-SCTDQ--S--G-SLVVYATIDVDSI-QL---A-MS--G-ED---
XP_023511589.1/1-230 HVEL-ML------QE-SCTDQ--S--G-SLVVYATIDVDSI-QL---A-MS--G-ED---
XP_022963906.1/1-230 HVEL-ML------QE-SCTDQ--S--G-SLVVYATIDVDSI-QL---A-MS--G-ED---
XP_023511591.1/1-230 HVEL-ML------QE-SCTDQ--S--G-SLVVYATIDVDSI-QL---A-MS--G-ED---
XP_022963908.1/1-230 HVEL-ML------QE-SCTDQ--S--G-SLVVYATIDVDSI-QL---A-MS--G-ED---
XP_022967184.1/1-230 HVEL-ML------QE-SCTDQ--S--G-SLVVYTTIDVDSI-QL---A-MS--G-ED---
XP_022967186.1/1-230 HVEL-ML------QE-SCTDQ--S--G-SLVVYTTIDVDSI-QL---A-MS--G-ED---
XP_010518669.1/1-225 NVEL-ML------QE-SCTDE--S--G-SLVIYSTVDVDAV-QL---T-MS--G-ED---
XP_010677365.1/1-235 NVEL-ML------QE-SCTDP--S--G-SLVVYATMEVDYI-QL---V-MS--G-ED---
XP_010677364.1/1-235 NVEL-ML------QE-SCTDP--S--G-SLVVYATMEVDYI-QL---V-MS--G-ED---
XP_021852084.1/1-226 NVEL-ML------QE-SCTDP--S--G-SLVVYATMEVDYI-QL---V-MS--G-ED---
XP_021852076.1/1-226 NVEL-ML------QE-SCTDP--S--G-SLVVYATMEVDYI-QL---V-MS--G-ED---
XP_021765591.1/1-233 NVEL-ML------QE-SCTDP--S--G-SLVVYATMEVDYI-QL---V-MS--G-ED---
XP_021768254.1/1-233 NVEL-ML------QE-SCTDP--S--G-SLVVYATMEVDYI-QL---V-MS--G-ED---
XP_017232690.1/1-221 TVEF-ML------QE-SCTDE--S--G-SIVAYTTLDVDAV-QL---A-MN--G-ED---
XP_017232692.1/1-221 TVEF-ML------QE-SCTDE--S--G-SIVAYTTLDVDAV-QL---A-MN--G-ED---
XP_024021697.1/1-248 HVEL-ML------QE-SCTDQ--S--G-SLVVYSTMEVDAI-QL---A-MS--G-ED---
XP_010096931.1/1-248 HVEL-ML------QE-SCTDQ--S--G-SLVVYSTMEVDAI-QL---A-MS--G-ED---
XP_024021696.1/1-248 HVEL-ML------QE-SCTDQ--S--G-SLVVYSTMEVDAI-QL---A-MS--G-ED---
XP_017621310.1/1-230 HVDL-ML------QE-SCTNK--S--G-SLIVYSTVDVDSV-QL---A-MS--G-ED---
XP_016731150.1/1-230 HVDL-ML------QE-SCTNK--S--G-SLVVYSTVDVDSV-QL---A-MS--G-ED---
XP_012472199.1/1-230 HVDL-ML------QE-SCTDK--S--G-SLVVYSTVDVDSV-QL---A-MS--G-ED---
XP_016727305.1/1-230 HVDL-ML------QE-SCTDK--S--G-SLVVYSTVDVDSV-QL---A-MS--G-ED---
XP_016731145.1/1-230 HVDL-ML------QE-SCTDK--S--G-SLVVYSTVDVDSV-QL---A-MS--G-ED---
XP_012472200.1/1-230 HVDL-ML------QE-SCTNK--S--G-SLVVYSTVDVDSV-QL---A-MS--G-ED---
XP_012472201.1/1-230 HVDL-ML------QE-SCTNK--S--G-SLVVYSTVDVDSV-QL---A-MS--G-ED---
NP_001314348.1/1-230 HVDL-ML------QE-SCTNK--S--G-SLVVYSTVDVDSV-QL---A-MS--G-ED---
XP_016727300.1/1-230 HVDL-ML------QE-SCTNK--S--G-SLVVYSTVDVDSV-QL---A-MS--G-ED---
XP_022738572.1/1-235 HVDL-IL------QE-SCTDR--S--G-SLVVYSTVDVASV-QL---E-MS--G-ED---
XP_021297648.1/1-235 HVEL-ML------QE-SCTDR--S--G-SLVVYSTVDVDSV-QL---A-MS--G-ED---
XP_017973778.1/1-235 HVEL-ML------QE-SCTDR--S--G-SLVVYSTVDVDSV-QL---A-MS--G-ED---
XP_017973780.1/1-235 HVEL-ML------QE-SCTDR--S--G-SLVVYSTVDVDSV-QL---A-MS--G-ED---
XP_022717458.1/1-235 HVEL-ML------QE-SCTDR--S--G-SLVVYSTVDVDSV-QL---A-MS--G-ED---
XP_022717453.1/1-235 HVEL-ML------QE-SCTDR--S--G-SLVVYSTVDVDSV-QL---A-MS--G-ED---
XP_022717437.1/1-235 HVEL-ML------QE-SCTDR--S--G-SLVVYSTVDVDSV-QL---A-MS--G-ED---
XP_022756982.1/1-232 HVEL-ML------QE-SCTDR--S--G-SLVVYSTVGVDSI-QL---A-MS--G-ED---
XP_004300207.1/1-230 NIEL-ML------QE-SCTDQ--S--G-SLVVYATMDVNDI-QL---A-MS--G-ED---
XP_024169578.1/1-230 NIEL-ML------QE-SCTDQ--S--G-SLVVYATMDVNDI-QL---A-MS--G-ED---
XP_008238183.1/1-249 NVEL-ML------QE-SCTDE--S--G-SLVVYTTMDVDGI-QL---A-MS--G-ED---
XP_016651445.1/1-249 NVEL-ML------QE-SCTDE--S--G-SLVVYTTMDVDGI-QL---A-MS--G-ED---
XP_020418924.1/1-249 NVEL-ML------QE-SCTDE--S--G-SLVVYTTMDVDGI-QL---A-MS--G-ED---
XP_021804146.1/1-249 NVEL-ML------QE-SCTDE--S--G-SLVVYTTMDVDGI-QL---A-MS--G-ED---
XP_008238184.1/1-248 NVEL-ML------QE-SCTDE--S--G-SLVVYTTMDVDGI-QL---A-MS--G-ED---
XP_016651446.1/1-248 NVEL-ML------QE-SCTDE--S--G-SLVVYTTMDVDGI-QL---A-MS--G-ED---
XP_007209851.1/1-248 NVEL-ML------QE-SCTDE--S--G-SLVVYTTMDVDGI-QL---A-MS--G-ED---
XP_021804154.1/1-248 NVEL-ML------QE-SCTDE--S--G-SLVVYTTMDVDGI-QL---A-MS--G-ED---
XP_017179076.1/1-239 NAEL-ML------QE-SCTDE--S--G-SLVVYATMDVDGI-QL---A-MS--G-ED---
XP_008356445.1/1-246 NIEI-ML------QE-SCTDE--S--G-SLVVYATMDVDGI-QL---A-MS--G-ED---
XP_008356446.1/1-240 NIEI-ML------QE-SCTDE--S--G-SLVVYATMDVDGI-QL---A-MS--G-ED---
XP_008356447.1/1-239 NIEI-ML------QE-SCTDE--S--G-SLVVYATMDVDGI-QL---A-MS--G-ED---
XP_009373485.1/1-239 NIEI-ML------QE-SCTDE--S--G-SLVVYATMDVDGI-QL---A-MS--G-ED---
XP_009373484.1/1-240 NIEI-ML------QE-SCTDE--S--G-SLVVYATMDVDGI-QL---A-MS--G-ED---
XP_004512486.1/1-245 NVEL-ML------QE-SCTDQ--S--G-SLVVYTTIDVDSI-QL---A-MS--G-ED---
XP_023898631.1/1-242 HVEL-ML------QE-SCTDQ--S--G-SLVVYTTVDVDSI-QL---A-MN--S-ED---
XP_024465375.1/1-238 HVDL-ML------QE-SCTDQ--S--G-SLVVFTTVDVESI-QL---A-MS--G-ED---
XP_011018502.1/1-238 HVDL-ML------QE-SCTDQ--S--G-GLVVFTTVDVESI-QL---A-MS--G-ED---
XP_011018503.1/1-238 HVDL-ML------QE-SCTDQ--S--G-GLVVFTTVDVESI-QL---A-MS--G-ED---
XP_021893615.1/1-233 HVEL-ML------QE-SCTDQ--S--G-SLVVFSNVDVDSI-QL---A-MS--G-ED---
XP_024952893.1/1-247 HVEL-ML------QE-SCTDQ--S--G-SLVVYTTVDVDSI-QL---A-MS--G-ED---
XP_006471522.1/1-248 HVEL-ML------QE-SCTDQ--S--G-SLVVYTTVDVDSI-QL---A-MS--G-ED---
XP_006432745.2/1-248 HVEL-ML------QE-SCTDQ--S--G-SLVVYTTVDVDSI-QL---A-MS--G-ED---
XP_021640807.1/1-241 HVDL-ML------QE-SCTDQ--S--G-SFVVYTTINVESI-QL---A-MG--G-ED---
XP_002518976.1/1-243 HVEL-ML------QE-SCTDQ--S--G-SLIVYTTVNVDSI-QL---A-MS--G-ED---
XP_021598537.1/1-237 HVEL-ML------QE-SCTDQ--S--G-SLIVYTTVNVDSI-QL---A-MS--G-ED---
XP_021598535.1/1-239 HVEL-ML------QE-SCTDQ--S--G-SLIVYTTVNVDSI-QL---A-MS--G-ED---
XP_006659280.1/1-226 SSTL-LL------QE-ARTDL--S--G-SLIVYAGIDVNTM-HS----IMT-GD-LD---
XP_025801379.1/1-254 DRTL-IL------QE-ARTDA--S--G-SLVVYTPIGEEAM-DA----AMG-GG-D----
XP_023156326.1/1-229 KKTL-IL------QE-ACTDA--S--C-SMVVYAPVEEDSM-RA----VMN-GG-D----
NP_001349156.1/1-229 KKTL-IL------QE-ACTDA--S--C-SMVVYAPVEEDSM-RA----VMN-GG-D----
XP_021310588.1/1-246 KKVL-ML------QQ-ACTDA--S--R-SMVVYAPVEEDFM-RA----VMN-GG-D----
XP_020404516.1/1-224 NAML-FL------QQ-TSIDV--S--R-ALVVYSVVEETML-RS----VLD-VS-DD---
XP_022684239.1/1-229 SNML-IL------QE-ATSDL--S--C-SLVVYSFVEKNMM-CA----VMD-GA-D----
XP_020404892.1/1-233 SKVL-IL------QE-TTTDV--S--C-SLVVYSLVEENLM-RG----IMD-GR-E----
XP_022684238.1/1-237 NNML-IL------QQ-SSTDA--S--G-SLVVYSLIEENVM-RG----IMA-GA-E----
XP_025825251.1/1-229 NDML-IL------QQ-SSTDA--S--G-SLVVYSLVEENVM-RG----IMG-GA-D----
XP_020183090.1/1-242 NKKL-IL------QQ-ACSDA--P--C-MVVAYAPIDAGEL-KD----VMH-GG-S----
XP_020194819.1/1-240 YGKL-IL------QE-SCIDA--S--C-MVLAYAQIDDQTI-QD---V--I-NG-T----
XP_024314575.1/1-244 NDKL-IL------QE-AYADA--S--C-MFMAYAPIDKQSM-HQ----VMN-GG-G----
XP_024314342.1/1-235 NNKL-IL------QE-AYTDT--S--C-MLVVYVPVDEQSM-KD---ASMN-GG-G----
XP_024959674.1/1-192 DNMK-CL------QA-CFTDS--I--G-MYIVWTFLHNATV-YA----MIE--G-DD---
XP_019196338.1/1-215 AAKV-YL------QE-SFSDS--V--G-LYVVYAAVDVATA-QY----LST---------
XP_006646400.1/1-226 ERTM-IL------QN-NGYDV--S--G-SFIVYSPIDSKLM-DT----MVLGPS-ELPSG
XP_015632244.1/1-226 GRTM-IL------QN-NGYDA--S--G-SFMVYSQIDSELM-NT----MVLSPS-DLPPG
XP_024314625.1/1-231 GNLM-IL------QN-SWYDV--S--G-SFIVYAPVDSMLI-NR----IIG-PG-DV-A-
XP_020164122.1/1-228 DNIM-IL------QN-SGYDV--S--G-AFMVYCPVNIQLM-NE----IMI-PS-DT-AQ
XP_025814677.1/1-221 ETMM-IL------QN-SCYDA--S--G-SFMVYSSLDKHVM-EM----ITS-PG-GE-EA
XP_022682466.1/1-225 EIMM-IL------QN-SCYDA--S--G-SFMVYSSLDKNVM-EM----ITS-PG-GE-HA
XP_002456467.1/1-226 DTVM-IL------QN-TFYDA--S--G-AFMVYSSLDKQLL-EI----I----G-DN-QA
XP_008674944.1/1-228 DTMM-IL------QN-SSYDA--S--G-SFMVYSSLDKQLV-ET----ITN-PG-DS-QA
XP_010523539.1/1-228 NEKY-VL------QE-TYHEA--S--A-TMVIHSAVDLEAA-AI----AAT-HG-EE---
XP_020873847.1/1-203 TEVF-LL------QE-AYNEA--S--S-SMVIHSILDESSL--R----KII-NG-DS---
NP_001190248.1/1-203 KEWY-LI------QE-TYYDE--S--G-AMIIHTCVEAPYF--A----AAI-NG-GD---
NP_196343.2/1-203    KEWY-LI------QE-TYYDE--S--G-AMIIHTCVEAPYF--A----AAI-NG-GD---
XP_020876233.1/1-195 KEWY-LI------QE-TYYDE--T--G-AMIIHACLEAPYF--A----AVI-ND-GD---
XP_010423235.1/1-188 KEWY-LI------QE-TYYEA--S--G-AMIIYACIEAPCF--T----AII-NG-ED---
XP_010452682.1/1-218 KEWY-LI------QE-TYYEA--S--G-AMIIYACIEAPCF--T----AII-NG-ED---
XP_010491330.1/1-188 KEWY-LI------QE-TYYEA--F--G-AMIICACIEAPCF--T----AII-NG-ED---
XP_006413300.2/1-224 DGPA-VL------QE-TWNDA--S--G-ALLVFAPLEEQSV-KG----LM--RG-GD---
XP_022008499.1/1-216 QPLK-VL------QE-ASSDT--T--G-SLIVYAIVDTPTV-AL----VM--QG-GD---
XP_022008502.1/1-215 QPLM-VL------QE-ASSDT--T--G-SLIVYAIVDTPTV-AL----VM--QG-GD---
XP_022007635.1/1-226 DQIM-VL------QE-TTSDM--T--G-SLLVYATVDIPTV-SV----VM--NG-DD---
XP_022008501.1/1-224 DQVM-VL------QE-TTSDM--T--G-SLIVYATVDFPTV-SM----VM--NG-ED---
XP_021986984.1/1-226 DQVM-VL------QE-TICDM--T--G-SLIVYATIDFPTI-SV----VM--NG-ED---
XP_021996009.1/1-224 DQVM-VL------QE-TISDM--T--G-SLIVYATIDFPTV-SV----VM--NG-ED---
XP_021996011.1/1-224 DQVM-VL------QE-TISDM--T--G-SLIVYATIDFPTV-SV----VM--NG-ED---
XP_022026050.1/1-224 DQVM-VL------QE-TISDM--T--G-SLIVYATIDFPTV-SV----VM--NG-ED---
XP_022026083.1/1-224 DQVM-VL------QE-TISDM--T--G-SLIVYATIDFPTV-SV----VM--NG-ED---
XP_021722389.1/1-209 IDTF-FL------QE-KHESA--V--A-SYVLFSPMTNDSL-TT----L---SN-HG---
XP_021847315.1/1-219 IDTF-FL------QE-KHENA--V--A-SYVMFSPITYDSL-SS----LSS-NN-HG---
XP_020224300.1/1-216 NDVM-IF------QD-SYTNP--L--G-SYVVYAPISMKNA-CL----IM--NG-DD---
XP_021765248.1/1-224 RSEM-IF------QE-IMTGP--Y--E-SLIVYAAINEQNM-IG----VT--NG-MD---
XP_004510020.1/1-219 RMIH-IV------QE-SCMDP--V--G-AYVVFASVDTRKM-YR----IL--NG-EN---
XP_004487953.1/1-231 NIIL-YL------KE-SCIDI--L--G-AYVIYAPIIQSQV-KR----AL--SG-VD---
XP_008244376.1/1-218 IKAL-YM------QE-SYAAS--T--G-SYIVYAPFDYKHA-EN----KL--MK-DE---
XP_008244380.1/1-218 IKAL-YM------QE-SYAAS--T--G-SYIVYAPFDYKHA-EN----KL--MK-DE---
XP_012569676.1/1-224 TGMV-IL------HE-SFTSP--F--G-SYLVYAPMDTALM-NV----AI--SG-EE---
XP_019418393.1/1-229 ERVI-II------QE-NFVNH--L--G-SYIVFSPIKEGDL-GV----TL--RG-HD---
XP_019440118.1/1-223 ERGL-II------QE-IFMTP--L--G-SYIVYSPINVDDL-SM----TV--RG-HD---
XP_014517363.2/1-226 NNTA-II------QE-SYIDA--L--G-SYVVYSPVNISDV-KM----AV--NG-ED---
XP_012569675.1/1-231 EGVV-II------QE-CFINP--L--G-SYVVYSPVNIPEL-NI----AF--NG-QD---
XP_004486570.1/1-223 DSVV-IM------QE-CFIEP--L--G-SYVVFSPVNVPQL-KM----FL--DG-HD---
XP_003594569.3/1-220 DGVK-II------QE-CFIDP--L--G-SYVVYSPLNTQEL-NM----AI--NG-HD---
XP_024632692.1/1-220 DGVK-II------QE-CFIDP--L--G-SYVVYSPLNTQEL-NM----AI--NG-HD---
XP_025887127.1/1-229 -SRV-IL------QD-TCTDE--S--G-SILVYATIGSQEM-DK----VID-GG--D---
XP_015078684.1/1-230 -SRV-IL------QD-TSTDE--S--G-SILVYATIGSQEM-DK----VID-GG--D---
XP_025887126.1/1-230 -SRV-IL------QD-TCTDE--S--G-SILVYATIGSQEM-DK----VID-GG--D---
XP_015078401.1/1-220 NNMM-IL------QD-TFTDA--T--G-SLVVYARMDSLAM-NM----VKK-IG--D---
XP_015158871.1/1-217 NNMM-IL------QD-TCTDA--T--G-SLLVYATMDSLAM-NV----VKK-KG--D---
XP_015078778.1/1-220 NNMM-IF------QD-TCTDA--T--G-SLLVYAIVDSSKM-NT----VMK-GG--D---
XP_010321992.1/1-216 NNMM-IF------QD-TCTDA--T--G-SLLVYAIVDSSKM-NT----VMK-GG--D---
XP_004240735.1/1-216 NNMM-IF------QD-TCTDA--T--G-SLLVYAIVDSSKM-NT----VMK-GG--D---
XP_015169793.1/1-213 NNML-IF------QD-TCTDT--T--G-SLLVYATVGSQDI-NM----VMK-EG--D---
XP_006285631.1/1-212 NDML-VL------QE-VWNDA--S--G-AVLVYAPVETSSI-EA----VNR-GE--N---
XP_023635531.1/1-198 NDML-VL------QE-VWNDA--S--G-AVLVYAPVETSSI-EA----VNR-GE--N---
NP_567722.1/1-217    NGML-VL------QE-IWNDA--S--G-AMVVYAPVETNSI-EL----VKR-GE--N---
XP_020873337.1/1-219 NGML-VL------QE-IWNDA--S--G-AMVVYSPVETNPI-EW----VKR-GG--D---
XP_020873338.1/1-219 NGML-VL------QE-IWNDA--S--G-AMVVYSPVETNPI-EW----VKR-GG--D---
XP_023000325.1/1-225 PNKW-II------QD-SLTND--Y--E-SSVIYAQIDITSM-QS----VMA-GC--D---
XP_023000326.1/1-214 PNKW-II------QD-SLTND--Y--E-SSVIYAQIDITSM-QS----VMA-GC--D---
XP_022963997.1/1-214 SNKW-II------QD-SLTND--Y--E-STVIYAQIDITSM-QS----VMA-GC--D---
XP_023513617.1/1-208 SNKW-II------QD-TLTND--Y--E-STVIYAQIDITSM-QS----VMV-GC--D---
XP_021312124.1/1-225 SGKW-IL------QD-SSASP--C--E-STVAYAPIDAAVL-RP----VID-GH--D---
XP_012701492.1/1-243 GSEW-IL------QD-SSTNP--C--E-CTVAYAPVDAAAL-RP----VID-GH--D---
XP_025815339.1/1-229 DGVW-IL------QD-SSTSP--C--E-STVAYAAVDAAAL-RP----VID-GH--D---
XP_015644459.1/1-237 GGKW-VL------QD-VCTNP--C--E-STIAYAAIDAAAL-QP----VIA-GH--D---
XP_024314016.1/1-228 GGKW-IV------QDSSCTSS--C--E-AIVAYAPVDAAVL-QP----VIS-GH--D---
XP_020168333.1/1-225 GGEW-VV------QD-SCTSP--C--E-SIVAYAPVDAAVL-QP----VIS-GH--D---
XP_013589242.1/1-206 --NS-VL------QE-TWNDA--S--G-ALLVYAPVDMEKI--------LA-SE--N---
XP_022548456.1/1-204 --NS-VL------QE-TWNDA--S--G-ALLVYAPVDMEKI--------LA-SE--N---
XP_024632691.1/1-227 RQMV-IL------QE-SFTSS--V--G-SYVIYAPIDRKTM-DV----ALR-GE--D---
XP_017256377.1/1-220 TLKF-YL------QE-SYTDA--T--G-YYIVYAPVDDESL-KA----LLK-GG--S---
XP_009632057.1/1-224 SEMF-YL------QE-SFTDS--T--V-MYIIYAPVDASAV-QH----IME-GK--Y---
XP_016502932.1/1-224 SEMF-YL------QE-SFTDT--T--V-MYIIYAPVDASAV-QH----IME-GK--Y---
XP_019258997.1/1-221 SEMF-YL------QE-SFTDS--T--V-MYIIYAPVDASAV-QY----IME-GK--D---
XP_009792862.1/1-224 SEMF-YL------QE-SFTDS--T--V-MYIIYAPVDASAV-QY----IME-GK--D---
XP_016544784.1/1-213 SEIF-YL------QE-SFTDS--T--A-MYVIYAPIDTPAV-HY----IME-GK--D---
XP_015089689.1/1-213 SEMF-YL------QE-SFTDS--T--G-MYIIFAPVDGSAV-KY----IME-GK--N---
XP_025883585.1/1-213 SEMF-YL------QE-SFTDS--T--G-MYIIFAPVDGSAV-KY----IME-GK--N---
XP_011095790.1/1-221 REIT-YL------QE-SFSDS--T--A-FYIVYAPVDVPAM-HH----ILQ-GG--C---
XP_020553953.1/1-221 REIT-YL------QE-SFSDS--T--A-FYIVYAPVDVPAM-HH----ILQ-GG--C---
XP_022857758.1/1-208 NAVA-YL------HE-SFYDS--T--G-YYVVYAPIDISAM-DH----IME-GK--N---
XP_004487739.1/1-224 TEIF-YI------QE-SYADT--T--I-SYVTYAPLDESAV-AY----LAK-GS--N---
XP_003596663.1/1-221 NEIF-YL------QE-SYKDT--T--A-SYVIYCPLDEQKL-TH----LAT-GS--N---
XP_003596665.1/1-227 TEIF-YL------QE-SYADI--T--A-SYVIYAPLDEPAL-TS----LAT-GS-SN---
XP_020201972.1/1-216 LEIF-YL------QE-SYTDS--T--G-SYVIYAPLDEGTL-SS----LSK-GS--D---
XP_025980734.1/1-220 LQIF-YL------QE-SYTDS--T--G-SYVVYAPLDESAI-TA----IVK-GT--N---
XP_007149715.1/1-218 LVIC-FV------QE-SYTDS--T--G-SYVVYAPLDKAAV-AG----LIH-GS--N---
XP_017423636.1/1-218 LVIC-FL------QE-SYTDS--T--G-SYIVYAPLDASAV-AA----LVH-GA--N---
XP_022631965.1/1-218 LVIC-FL------QE-SYTDS--T--G-SYIVYAPLDESAV-AA----LIH-GA--N---
XP_014522034.2/1-218 LVIC-FL------QE-SYTDS--T--G-SYIVYAPLDESAV-AA----LIH-GA--N---
XP_025669173.1/1-223 IEIF-YI------QE-SYTDT--T--G-SYIVYAPLDEFAL-SS----LAN-GA--N---
XP_020963786.1/1-223 IEIF-YI------QE-SYTDT--T--G-SYIVYAPLDEFAL-SS----LAN-GA--N---
XP_025614692.1/1-223 IEIF-YI------QE-SYIDT--T--G-SYIVYAPLDEFAL-SS----LAN-GA--N---
XP_015936351.1/1-223 IEIF-YI------QE-SYIDT--T--G-SYIVYAPLDEFAL-SS----LAN-GA--N---
XP_019451675.1/1-226 TEIF-YI------QK-SYTDS--M--A-SYVVYAPLDESSL-KG----LGN-GS--N---
XP_019465428.1/1-219 TDIY-YI------QK-SYTDS--I--A-SYVVYAPLDESAL-KS----LSN-GS--N---
XP_004295798.2/1-220 IEIF-YL------QE-SYTDS--N--G-SYVVYAPIDQFAM-SA----LLD-GS--N---
XP_021807338.1/1-219 IKAL-YM------QE-SYTAS--T--G-SYIVYAPFDYKHAENK----LMK-DE--W---
XP_021807340.1/1-219 IKAL-YM------QE-SYTAS--T--G-SYIVYAPFDYKHAENK----LMK-DE--W---
XP_020417197.1/1-219 IKAL-YM------QE-SCAAS--T--G-SYIVYAPFDYKDAENK----LMK-DE--W---
XP_020417200.1/1-219 IKAL-YM------QE-SCAAS--T--G-SYIVYAPFDYKDAENK----LMK-DE--W---
XP_009333923.1/1-206 VGIF-YL------QA-SYTDT--T--G-SYVVYAPIDMYAM-TE----LIN-GG--N---
XP_017184363.1/1-206 VEIF-YL------QA-SYTDA--T--G-SYVVYAPIDMYAM-TE----LIN-GG--N---
XP_021892911.1/1-224 PAMV-YL------QK-IYKHE--T--G-SYVIHAPMDPVSV-AL----MLN-GG--N---
XP_002278971.2/1-210 IVVI-LL------QE-SYTTF--T--S-SYVTFAPVEACTL-DM----ILN-GG--S---
XP_010644412.1/1-211 TIKT-LL------QE-SFTTS--D--S-SYVTFTPVEASSF-SM----TLN-GG--D---
XP_024450660.1/1-210 IEIL-YL------QE-SYSHP--T--G-SYVVYAPIDILTM-GM----MLG-GG--N---
XP_002522867.2/1-214 KEIL-YL------QE-SYTDP--F--A-SYVVYAPFDFDSM-AT----ILK-GG--N---
XP_020534605.1/1-220 IEIL-YL------QE-SYSDP--T--G-FYIVYAPMDVISM-AT----LVD-GG--N---
XP_021685850.1/1-217 IEIL-YL------QE-SYSDP--T--A-FYIVYAPMDITSV-AT----ILK-GG--N---
XP_021594464.1/1-220 IEIL-YL------QE-SYSDP--T--A-LYVVYAPMDIFSV-AA----ILK-GG--S---
XP_018731177.1/1-223 SSVL-YL------QN-SYSDP--T--G-SYIVYAPVDLGAM-TS----LLN-GG--N---
XP_024018593.1/1-217 IAIL-YL------QQ-SCTDA--T--G-SYVVFAPLDILSV-SR----VLN-GG--N---
XP_022139420.1/1-219 VEIF-YL------QE-SFFDT--T--G-SYIVYAPIDVFAM-SA----VFH-GG--N---
XP_022981426.1/1-220 VEVF-YL------QE-SYFDA--T--G-SYIVYAPVDLFAM-SI----LLR-GG--N---
XP_022981427.1/1-220 VEVF-YL------QE-SYFDA--T--G-SYIVYAPVDLFAM-SI----LLR-GG--N---
XP_022941489.1/1-220 VEIF-YL------QE-SYFDA--T--G-SYIVYAPVDLFAM-SI----LLR-GG--N---
XP_022941488.1/1-220 VEIF-YL------QE-SYFDA--T--G-SYIVYAPVDLFAM-SI----LLR-GG--N---
XP_023525599.1/1-220 VEIF-YL------QE-SYFDA--T--G-SYIVYAPVDLFAM-SI----LLR-GG--N---
XP_023525597.1/1-220 VEIF-YL------QE-SYFDA--T--G-SYIVYAPVDLFAM-SI----LLR-GG--N---
XP_008454681.2/1-220 IEIF-YL------QE-SFFDA--S--G-SYIVFAPVDIYAM-AV----VLR-GG--N---
XP_011648553.1/1-223 IEIF-YL------QE-SFFDE--S--G-SYVVFAPVDIYAM-AV----VLR-GG--N---
XP_018822340.1/1-211 --VL-YL------QE-SYTDS--T--G-SYVVYAPLDAYAI-SM----VLS-GG--N---
XP_018822367.1/1-211 --VL-YL------QE-SYTDS--T--G-SYVVYAPLDAYAI-SM----VLS-GG--N---
XP_023876961.1/1-210 --VL-YL------QE-SYSDS--T--G-SYVVYAPMDAYAM-SI----VLN-GG--D---
XP_023876962.1/1-210 --VL-YL------QE-SYSDS--T--G-SYVVYAPMDAYAM-SI----VLN-GG--D---
XP_024034242.1/1-215 VEIF-YL------QE-SFSDD--I--G-SYVIYAPVDALAM-SG----VLN-GG--S---
XP_015381349.2/1-215 VEIF-YL------QE-SFSDD--I--G-SYVIYAPVDALAM-SG----VLN-GG--S---
XP_022717683.1/1-216 IEIL-YL------QE-SFTDE--T--G-SYVIYAPIDIFAM-SM----ILN-GG--N---
XP_016729588.1/1-216 IEIP-YL------QE-SYTDE--T--G-SYIVYAPMDIMAM-SK----ILN-GG--N---
XP_016729589.1/1-216 IEIP-YL------QE-SYTDE--T--G-SYIVYAPMDIMAM-SK----ILN-GG--N---
XP_016702140.1/1-208 IEIP-YL------QE-SYTDE--T--G-SYIVYAPMDIMAM-SK----ILN-GG--N---
XP_012460158.1/1-216 IEIL-YL------QE-SYTDE--T--G-SYIVYAPMDIMAM-SK----ILN-GG--N---
XP_012460145.1/1-216 IEIL-YL------QE-SYTDE--T--G-SYIVYAPMDIMAM-SK----ILN-GG--N---
XP_017245631.1/1-197 QNKL-IL------QE-SWTDP--S--G-ALVVYCPVPLPAI-KA----VMS-GE--D---
XP_004956045.3/1-247 NKML-IL------QE-ARTDV--S--G-SLVVYAPIRENTM-HA----VMN-SG--N---
XP_014660176.1/1-240 SKML-IL------QE-ARTDA--S--C-SIVVYAPVEEERM-RA----VMN-GG--D---
XP_017239609.1/1-213 PVHI-LL------QE-SWSDS--V--A-SHIVFASTEIGNI-DV----ILG-GS--S---
XP_023740378.1/1-206 NPVM-IV------QE-TTSDM--T--G-SLIVYAPVDLPTI-SV----MMN-NG--D---
XP_020095129.1/1-235 NNIM-IL------QQ-SCYNT--S--G-SFVIYAPIDATAL-NM----VLS-GA--D---
XP_020258373.1/1-212 KKKM-FI------QE-SCFDN--T--C-SYLIYAPVSKRSV-NV----CIH-GG--D---
XP_008812148.1/1-175 SHVM-IL------QE-NCSHA--T--G-SYVIYAPIDIVAL-NA----ILT-GQ--E---
XP_010930674.1/1-230 SHVM-IL------QE-NCSHA--T--G-SYVIYAPIDIVAL-NA----ILT-GQ--E---
XP_010931877.1/1-230 SHVM-IL------QE-NCSHA--T--G-SYVIYAPIDVVAL-NA----ILT-GQ--E---
XP_009380061.1/1-225 HNML-IL------QE-SCTDA--S--G-SVVVYSPIDLPAM-NT----IMS-G--ED---
XP_010939971.1/1-229 SNML-IL------QE-SCIDA--S--G-SLVVYASIDLPSL-ND----VMS-G--ED---
XP_019710626.1/1-218 SNML-IL------QE-SCIDA--S--G-SLVVYASIDLPSL-ND----VMS-G--ED---
XP_008787891.1/1-229 SNML-IL------QE-SCIDA--S--G-SLVVYTSVDLPSL-NN----VMS-G--ED---
XP_008787892.1/1-218 SNML-IL------QE-SCIDA--S--G-SLVVYTSVDLPSL-NN----VMS-G--ED---
XP_008799024.1/1-230 SNML-IL------QE-SCIDA--S--G-SLIAYASVDLPSL-NM----VMS-G--ED---
XP_010936845.1/1-230 SNML-IL------QE-SCIDA--S--V-SLTAYATVDLPSL-DM----VMS-G--VD---
XP_003570542.1/1-223 NSML-IL------QE-SSTDA--T--G-SQVVYAPIDLPAA-NV----VMS-G--ED---
XP_004964922.1/1-229 NSML-IL------QE-SCTDA--S--G-SLVVYSPIDLPAA-NV----VMS-G--ED---
XP_002438026.1/1-230 NSML-IL------QE-SCTDA--S--G-ALVVYSPIDIPAA-NV----VMS-G--ED---
XP_020399989.1/1-232 NSML-IL------QE-SCADA--S--G-ALVVYSPIDIPAA-NV----VMS-G--ED---
XP_008649207.1/1-222 NSML-IL------QE-SCTDA--S--G-ALVVYSPIDIPAA-NV----VMS-G--ED---
XP_020256822.1/1-228 NSML-IL------QE-SCTDA--S--G-SIVVYAPVNLQSM-DI----VMS-G--ED---
XP_009403550.1/1-228 NTML-LL------QE-CCTDA--S--G-SVVVYSPIDLPAI-NI----VMS-G--ED---
XP_020582158.1/1-232 RSML-IL------QE-SCTDP--S--G-SLVVYASIEVPSI-NI----AIN-G--ED---
XP_020699118.1/1-240 RNML-IL------QE-SCTDS--S--G-SLVVYASVDLPSM-NI----AMS-G--ED---
XP_020600375.1/1-232 GNML-IL------QE-SCTDS--S--G-SLVVYASVDLPSI-NI----AMS-G--ED---
XP_020600376.1/1-232 GNML-IL------QE-SCTDS--S--G-SLVVYASVDLPSI-NI----AMS-G--ED---
XP_020684394.1/1-232 GNML-IL------QE-SCTDP--S--G-SLVVYASIDLASI-NI----AMS-G--ED---
XP_017257147.1/1-219 NNML-IL------QE-SCIDS--S--G-ALVVYCPVDLPAI-TI----AMS-S--ED---
XP_010244428.1/1-230 NNML-LL------QE-SCIDS--S--E-SLVVYSPVDLPAI-NI----AMS-G--ED---
XP_010244355.1/1-230 NNML-LL------QE-SCIDS--S--E-SLVVYSPVDLPAI-NI----AMS-G--ED---
XP_017240757.1/1-236 SNML-VL------QE-SCIDS--S--G-SLVVYSPVDLHSI-NI----AMS-G--ED---
XP_017245021.1/1-233 SNML-VL------QE-SCIDS--S--G-SLVVYSPVDLPAI-NI----AMS-G--ED---
XP_012829269.1/1-233 SNTL-IL------QE-SCMDS--S--G-STVVYCPVDLQAV-NI----AMS-G--ED---
XP_022889938.1/1-236 -NML-IL------QE-CCIDS--S--G-SLVVYGPVDFQSI-NI----VMS-G--AD---
XP_022897471.1/1-232 -NML-IL------QE-SCIDS--S--G-SLVVYSPVDLPSI-NI----AMS-G--AD---
XP_009606250.1/1-235 NNML-IL------QE-SCIDS--S--G-SLVIYSPVDLPSI-NI----AMS-G--ED---
XP_016468230.1/1-235 NNML-IL------QE-SCIDS--S--G-SLVIYSPVDLPSI-NI----AMS-G--ED---
XP_019250773.1/1-235 NNML-IL------QE-SCIDS--S--G-SLVIYSPVDLPSI-NV----AMS-G--ED---
XP_009778533.1/1-235 NNML-IL------QE-SCIDS--S--G-SLVIYSPVDLPSI-NM----AMS-G--ED---
XP_016451582.1/1-235 NNML-IL------QE-SCIDS--S--G-SLVIYSPVDLPSI-NM----AMS-G--ED---
XP_016578176.1/1-240 NNML-IL------QE-SCIDS--S--G-SLVVYSPVDLQSI-NI----AMS-G--ED---
XP_006347370.1/1-231 NNML-IL------QE-SCIDS--S--G-SLVVYSPVDLQSI-NI----AMS-G--ED---
XP_004241474.1/1-230 NNML-IL------QE-SCIDS--S--G-SLVVYSPVDLQSI-NI----AMS-G--ED---
XP_015077748.1/1-230 NNML-IL------QE-SCIDS--S--G-SLVVYSPVDLQSI-NI----AMS-G--ED---
XP_022037776.1/1-241 NNML-IL------QE-SSTDA--S--G-SLVVYCPVDLPAI-NI----AMN-G--ED---
XP_024992062.1/1-237 NSML-IL------QE-SCTDA--S--G-SLVIYCPVDLPAI-NI----AMN-G--ED---
XP_023772270.1/1-245 NNML-IL------QE-SCTDA--S--G-SLVVYCPVDLPAI-NI----AMN-G--ED---
XP_012855673.1/1-237 NNML-IL------QE-SCIDS--S--G-SIVVHCPVDLPSI-NV----AMS-G--ED---
XP_022858328.1/1-236 SNML-IL------QE-SCIDS--S--G-SLVVYCPVDLHHI-NI----AMS-G--ED---
XP_011072039.1/1-232 NNML-IL------QE-SCIDS--S--G-SLVVYCPIDLPAI-NI----AMS-G--ED---
XP_006306921.1/1-215 NNML-IL------QE-SCIDS--S--SAALVIYTPVDLPAL-NI----AMS-G--QD---
NP_564041.2/1-215    NNML-IL------QE-SCIDS--S--SAALVIYTPVDLPAL-NI----AMS-G--QD---
XP_020866823.1/1-216 NNML-IL------QE-SCIDS--T--SAALVIYTPVDLPAL-NI----AMS-G--QD---
XP_010476956.1/1-215 NNML-IL------QE-SCIDS--S--GAALVIYTPVDIPAL-NI----AMS-G--QD---
XP_010498167.1/1-217 NNML-IL------QE-SCIDS--S--GAALVIYTPVDIPAL-NI----AMS-G--QD---
XP_019091528.1/1-217 NNML-IL------QE-SCIDS--S--GAALVIYTPVDIPAL-NI----AMS-G--QD---
XP_013613976.1/1-227 NNML-IL------QE-SCVDS--S--G-SLVVYTPADLSAL-NM----AMT-G--QD---
XP_018432674.1/1-227 NNML-IL------QE-SCIDS--S--G-SLVVYTPVDLSAL-KM----AMT-G--QD---
XP_018432667.1/1-227 NNML-IL------QE-SCIDS--S--G-SLVVYTPVDLSAL-KM----AMT-G--QD---
XP_013641731.1/1-225 NNML-IL------QE-SCVDS--S--G-SLVVYTPVDLSAL-NM----AMT-G--QD---
XP_009149243.1/1-227 NNML-IL------QE-SCVDS--S--G-SLVVYTPVDLSAL-NM----AMT-G--QD---
XP_006416656.2/1-224 NNML-IL------QE-SCVDS--S--G-SLVVYTPVDLPAL-NM----AMS-G--QD---
XP_018451633.1/1-224 NNML-IL------QE-SCVDS--S--G-SLVVYTPVDLPAL-NM----AMS-G--QD---
XP_018451632.1/1-225 NNML-IL------QE-SCVDS--S--G-SLVVYTPVDLPAL-NM----AMS-G--QD---
XP_018451634.1/1-195 NNML-IL------QE-SCVDS--S--G-SLVVYTPVDLPAL-NM----AMS-G--QD---
XP_013690958.1/1-228 NNML-IL------QE-SCVDS--S--G-SLVVYTPLDLPAL-NM----AMS-G--QD---
XP_009110376.1/1-227 NNML-IL------QE-SCVDS--S--G-SLVVYTPLDLPAL-NM----AMS-G--QD---
XP_009110377.1/1-227 NNML-IL------QE-SCVDS--S--G-SLVVYTPLDLPAL-NM----AMS-G--QD---
XP_013601964.1/1-227 NNML-IL------QE-SCVDS--S--G-SLVVYTPLDLPAL-NM----AMS-G--QD---
XP_013653874.1/1-227 NNML-IL------QE-SCVDS--S--G-SLVVYTPLDLPAL-NM----AMS-G--QD---
XP_018445004.1/1-237 NNML-IL------QE-SSIDS--S--G-ALVVYSPVDLSAL-NI----AMN-G--DD---
XP_018445005.1/1-237 NNML-IL------QE-SSIDS--S--G-ALVVYSPVDLSAL-NI----AMN-G--DD---
XP_013648946.1/1-228 NNML-IL------QE-TSIDS--S--G-ALVVYSPVDLSAL-NI----AMN-G--DD---
XP_009104812.1/1-228 NNML-IL------QE-TSIDS--S--G-ALVVYSPVDLSAL-NI----AMN-G--DD---
XP_013588523.1/1-228 NNML-IL------QE-TSIDS--S--G-ALVVYSPVDLSAL-NI----AMN-G--DD---
XP_013699248.1/1-228 NNML-IL------QE-TSIDS--S--G-ALVVYSPVDLSAL-NI----AMN-G--DD---
XP_018457296.1/1-228 NNML-IL------QE-TSIDS--S--G-ALVVYSPVDLSAL-NI----AMS-G--DD---
XP_009105998.1/1-230 NNML-IL------QE-TSIDS--S--V-ALVVYSPVDLPAL-NI----AMS-G--DD---
NP_001302850.1/1-231 NNML-IL------QE-TSIDS--S--G-ALVVYSPVDLPAL-NI----AMS-G--DD---
XP_013698656.1/1-231 NNML-IL------QE-TSIDS--S--G-ALVVYSPVDLPAL-NI----AMS-G--DD---
XP_013649619.1/1-231 NNML-IL------QE-TSIDS--S--G-ALVVYSPVDLPAL-NI----AMS-G--DD---
XP_013592873.1/1-231 NNML-IL------QE-TSIDS--S--G-ALVVYSPVDLPAL-NI----AMS-G--DD---
XP_006390565.1/1-233 NNML-IL------QE-SSIDS--S--G-ALVVYSPVDLAAL-NI----AMS-G--ED---
XP_006301097.1/1-230 NNML-IL------QE-SSTDS--A--G-ALVIYSPVDLAAL-NI----AMS-G--ED---
XP_010428260.1/1-231 NNML-IL------QE-SSTDS--S--G-ALVVYSPVDLSAL-NI----AMS-G--ED---
XP_010471396.1/1-230 NNML-IL------QE-SSTDS--S--G-ALVVYSPVDLAAL-NI----AMS-G--ED---
XP_010416135.1/1-230 NNML-IL------QE-SSTDS--S--G-ALVVYSPVDLAAL-NI----AMS-A--ED---
NP_177479.1/1-227    NNML-IL------QE-SSTDS--S--G-AFVVYSPVDLAAL-NI----AMS-G--ED---
XP_020890987.1/1-228 NNML-IL------QE-SSTDS--S--G-ALVVYSPVDLAAL-NI----AMS-G--ED---
XP_010537520.1/1-232 NNML-IL------QE-SCIDS--S--G-ALVVYSPVDLSAL-HI----AMS-G--ED---
XP_010540042.1/1-226 NNML-IL------QE-SCIDS--S--G-ALVVYSPLELPAL-QI----AMS-G--ED---
XP_010540046.1/1-196 NNML-IL------QE-SCIDS--S--G-ALVVYSPLELPAL-QI----AMS-G--ED---
XP_019432660.1/1-235 NDML-IL------QE-SFIDS--S--G-AIVVYCPVDLPSI-NI----AMN-G--ED---
XP_019420678.1/1-233 NGML-IL------QE-SCIDS--S--G-ALVVYCPIDLPSI-NK----VMS-G--ED---
XP_013452816.1/1-243 NNML-IL------QE-SCVDS--S--G-SIVVYCPIELTSM-NV----AMS-G--ED---
XP_020215886.1/1-221 -NML-IL------QE-SCIDS--C--G-SLVVYCPVDLASI-NI----AMS-G--ED---
XP_020215888.1/1-221 -NML-IL------QE-SCIDS--C--G-SLVVYCPVDLASI-NI----AMS-G--ED---
XP_007160572.1/1-230 -NML-IL------QE-SCIDS--S--G-SLVVYCPVDLPSM-NI----AMS-G--ED---
XP_017424661.1/1-212 -NML-IL------QE-SCIDS--S--G-SLVVYCPVDLPSI-NI----AMS-G--ED---
XP_017424660.1/1-230 -NML-IL------QE-SCIDS--S--G-SLVVYCPVDLPSI-NI----AMS-G--ED---
XP_014508329.1/1-230 -NML-IL------QE-SCIDS--S--G-SLVVYCPVDLPSI-NI----AMS-G--ED---
XP_022632295.1/1-230 -NML-IL------QE-SCIDS--S--G-SLVVYCPVDLPSI-NI----AMS-G--ED---
XP_019225984.1/1-234 NNML-IL------QE-SCIDS--S--G-ALVVYSPVDVPAI-NI----AMS-G--ED---
XP_016441514.1/1-233 NNML-IL------QE-SCIDS--S--G-ALVVYSPVDVPAI-NI----AMS-G--ED---
XP_009587298.1/1-233 NNML-IL------QE-SCIDS--S--G-ALVVYSPVDVPAI-NI----AMS-G--ED---
XP_016441515.1/1-233 NNML-IL------QE-SCIDS--S--G-ALVVYSPVDVPAI-NI----AMS-G--ED---
XP_009786509.1/1-233 NNML-IL------QE-SCIDS--S--G-ALVVYSPVDVPAI-NI----AMS-G--ED---
XP_016563952.1/1-234 NNML-IL------QE-SCIDS--S--G-ALVVYSPVDVPVI-NI----AMS-G--ED---
XP_004235382.1/1-234 NNML-IL------QE-SCIDS--S--G-ALVVYSPVDIPAI-NI----AMS-G--ED---
XP_015068940.1/1-234 NNML-IL------QE-SCIDS--S--G-ALVVYSPVDIPAI-NI----AMS-G--ED---
XP_006364383.1/1-234 NNML-IL------QE-SCIDS--S--G-ALVVYSPVDIPAI-NI----AMS-G--ED---
XP_019167409.1/1-245 NNML-IL------QE-SCIDS--S--G-CLVVYSPVDLPAI-NM----AMS-G--ED---
XP_011086973.1/1-238 NNML-IL------QE-SCIDS--S--G-SLVVYCPVDLPAI-NI----AMS-G--ED---
XP_010268321.1/1-230 NNML-IL------QE-SCTDS--S--G-SLVVYSPVDLTAI-NI----AMS-G--ED---
XP_010268322.1/1-230 NNML-IL------QE-SCTDS--S--G-SLVVYSPVDLTAI-NI----AMS-G--ED---
XP_010069492.1/1-236 NNML-IL------QE-SCIDS--S--G-SLIVYCPVDLPAI-NL----AMS-G--ED---
XP_002527933.1/1-229 NNML-IL------QE-SCIDS--S--G-SLVVYCPVDLPAI-NI----AMS-G--ED---
XP_021678368.1/1-229 NNML-IL------QE-SCIDA--S--G-SLVVYCPVDLPAV-NI----AMS-G--ED---
XP_021678369.1/1-229 NNML-IL------QE-SCIDA--S--G-SLVVYCPVDLPAV-NI----AMS-G--ED---
XP_021602738.1/1-229 NNML-IL------QE-SCIDA--S--G-SLVVYCPVDLPAV-NI----AMS-G--ED---
XP_012079215.1/1-230 NNML-IL------QE-SCIDS--S--G-SLVVYCPVDLPAI-NI----AMS-G--ED---
XP_021687701.1/1-229 NNML-IL------QE-SCMDE--S--G-SLVVYCPVDLPAI-NI----VMS-G--ED---
XP_006374212.1/1-231 NNML-IL------QE-SCVDS--S--G-SLVVYCPVDLPAI-NI----AMS-G--ED---
XP_011035552.1/1-231 NNML-IL------QE-SCVDS--S--G-SLVVYCPVDLPAI-NI----AMS-G--ED---
XP_002317722.3/1-231 NNML-IL------QE-SCMDS--S--G-SLVVYCPVDLPAI-NI----AMS-G--ED---
XP_011000766.1/1-231 NNML-IL------QE-SCIDS--S--A-ALVVYCPVDLPAI-NI----AMS-G--ED---
XP_021907827.1/1-230 NNML-IL------QE-SCIDS--S--G-SLVVYCPVDLHAI-NI----AMS-G--ED---
XP_017635401.1/1-230 NNML-IL------QE-SCIDS--S--G-SLVVYCPVDLPAI-NV----AMS-G--ED---
XP_016686699.1/1-230 NNML-IL------QE-SCIDS--S--G-SLVVYCPVDLPAI-NV----AMS-G--ED---
XP_012440361.1/1-230 NNML-IL------QE-SCIDS--S--G-SLVVYCPVDLPAI-NV----AMS-G--ED---
NP_001313868.1/1-230 NNML-IL------QE-SCIDS--S--G-SLVVYCPVDLPAI-NV----AMS-G--ED---
XP_023919022.1/1-230 NNML-IL------QE-SCMDS--S--G-SLIVYCPVDLPAI-NI----AMS-G--ED---
XP_006478112.1/1-232 NNML-IL------QE-SCIDS--S--G-SLVVYCPVDLPAI-NI----AMS-G--ED---
XP_024042646.1/1-232 NNML-IL------QE-SCIDS--S--G-SLVVYCPVDLPAI-NI----AMS-G--ED---
XP_022715403.1/1-231 NNML-IL------QE-SCIDG--S--G-SLVVYCPVDLPAI-NE----AMS-G--ED---
XP_022767009.1/1-255 NNML-IL------QE-SCIDR--S--G-SLVVYCPVDLPAI-NL----AMS-G--ED---
XP_022737050.1/1-231 NNML-IL------QE-SCIDN--S--G-SLVVYCPVDLPAI-NV----AMS-G--ED---
XP_017973835.1/1-231 NNML-IL------QE-SCIDS--S--G-SLVVYCPVDLPAI-NV----AMS-G--ED---
XP_015967008.1/1-238 NNTL-IL------QE-SCIDP--S--G-SIVVYCPVELPSI-NI----AMS-G--ED---
XP_025701434.1/1-239 NNTL-IL------QE-SCIDP--S--G-SIVVYCPVELPSI-NI----AMS-G--ED---
XP_020980110.1/1-239 NNTL-IL------QE-SCIDP--S--G-SIVVYCPVELPSI-NI----AMS-G--ED---
XP_019434881.1/1-231 NNML-IL------QE-SCIDS--S--G-ALVVYCPVDIPSI-NI----AMN-G--ED---
XP_019458275.1/1-234 NNML-IL------QE-SCIDS--S--G-ALVVYCPIDLPSI-NI----AMN-G--ED---
XP_019458276.1/1-234 NNML-IL------QE-SCIDS--S--G-ALVVYCPIDLPSI-NI----AMN-G--ED---
XP_006584922.1/1-234 QNML-IL------QE-SCIDS--Y--G-SFVVYCPVDLPSI-NL----AMS-G--ED---
XP_003524332.1/1-231 QNML-IL------QE-SCIDS--Y--G-SLVVYCPVDLPSI-NT----AVS-G--ED---
XP_004515249.1/1-229 NNML-IL------QE-SCVDS--S--G-SLVVYCPVDLPAI-NI----AMS-G--ED---
XP_019431381.1/1-229 NSML-IL------QE-SCIDS--S--G-SLVVYCPVELSAI-NI----AMS-G--ED---
XP_019436771.1/1-231 NSML-IL------QE-SCIDS--S--G-SIVVYCPVELPAI-NI----AMS-G--ED---
XP_019430342.1/1-233 NSML-IL------QE-SCIDS--S--G-SLVVYCPVELPAI-NI----AMS-G--ED---
XP_006587436.1/1-240 NNML-IL------QE-SCVDS--S--G-SLVVYCPVDLPAI-NI----AMS-G--ED---
XP_003549189.1/1-236 NNML-IL------QE-SCVDS--S--G-SLVVYCPVDLPAI-NI----AMS-G--ED---
XP_020221986.1/1-232 NNML-FL------QE-SCVDS--S--G-SLVVYCPVDLPAI-NI----AMS-G--ED---
XP_007152598.1/1-234 NNML-IL------QE-SCVDS--S--G-SLVVYCPVDLPAI-NI----AMS-G--ED---
XP_017437542.1/1-234 NNML-IL------QE-SCVDS--S--G-SLVVYCPVDLPAI-NI----AMS-G--ED---
XP_014505752.1/1-234 NNML-IL------QE-SCVDS--S--G-SLVVYCPVDLPAI-NI----AMS-G--ED---
XP_015881991.1/1-234 NNML-IL------QE-SCIDS--S--G-SLVVYCPVDLPAI-NI----AMS-G--ED---
XP_004299483.1/1-232 NNML-IL------QE-SCIDS--S--G-SLVVYCPVDLPAI-NI----AMS-G--ED---
XP_024174647.1/1-232 NNML-IL------QE-SCIDS--S--G-SVVVYCPVDLPAI-NI----AMS-G--ED---
XP_008375041.1/1-227 NNML-IL------QQ-SCIDS--S--G-SLVVYSPVDLPSI-NI----AMS-G--ED---
XP_017188172.1/1-199 NNML-IL------QQ-SCIDS--S--G-SLVVYSPVDLPSI-NI----AMS-G--ED---
XP_009355964.1/1-227 NNML-IL------QQ-SCIDS--S--G-SLVVYSPVDLPAI-NI----AMS-G--ED---
XP_009355965.1/1-199 NNML-IL------QQ-SCIDS--S--G-SLVVYSPVDLPAI-NI----AMS-G--ED---
XP_023523434.1/1-228 NNML-IL------QE-SCIDS--S--G-SLVVYCPVDLPAM-NL----AMS-G--ED---
XP_022949138.1/1-227 NNML-IL------QE-SCIDS--S--G-SLVVYCPVDLPAM-NL----AMS-G--ED---
XP_022998050.1/1-227 NNML-IL------QE-SCIDS--S--G-SLVVYCPVDLPAM-NL----AMS-G--ED---
XP_002271012.1/1-231 NNML-IL------QE-SCIDS--S--G-SLVIYCPVDLPAI-NI----AMS-G--ED---
XP_010663934.1/1-231 NNML-IL------QE-SCIDS--S--G-SLVIYCPVDLPAI-NI----AMS-G--ED---
XP_010099679.1/1-237 NNML-IL------QE-SSIDA--S--G-SVVVYCPVDLPAI-NI----AMS-G--ED---
XP_018823334.1/1-236 NNML-IL------QE-SCIDS--S--G-ALVVYCPVDLPAI-NI----AMS-G--ED---
XP_023001145.1/1-228 NNML-IL------QE-SCIDS--S--G-SLVVYCPVDLPAM-NV----VMS-G--ED---
XP_023519889.1/1-228 NNML-IL------QE-SCIDS--S--G-SLVVYCPVDLPAM-NV----VMS-G--ED---
XP_022924191.1/1-228 NNML-IL------QE-SCIDS--S--G-SLVVYCPVDLPAM-NV----VMS-G--ED---
XP_018849090.1/1-235 NNML-IL------QE-SCVDS--S--G-ALVVYCPVDLPAI-NI----AMS-G--ED---
XP_008240378.1/1-228 NNML-ML------QE-SCIDS--S--G-SLVVYSPVDLPSI-NI----AMS-G--ED---
XP_007210460.2/1-228 NNML-ML------QE-SCIDS--S--G-SLVVYSPVDLPSI-NI----AMS-G--ED---
XP_021801797.1/1-228 NNML-ML------QE-SCIDS--S--G-SLVVYSPVDLPSI-NI----AMS-G--ED---
XP_022136988.1/1-228 NNML-IL------QE-SCIDS--S--G-SLVVYCPVDLPAM-NV----AMS-G--ED---
XP_016898817.1/1-228 NNML-IL------QE-SCIDS--S--G-SLVVYCPVDLPAM-NV----AMS-G--ED---
XP_004137568.1/1-228 NNML-IL------QE-SCIDS--S--G-SLVVYCPVDLPAM-NV----AMS-G--ED---
XP_015642098.1/1-238 NSML-IL------QE-SCTDA--S--G-SLVVYSPIDIPAA-NV----VMS-G--ED---
XP_020186741.1/1-220 NSML-IL------QE-SCADA--S--G-SLVVYAPIDLPAA-NV----VMS-G--ED---
XP_025828617.1/1-229 NSML-IL------QE-SCTDA--S--G-SLVVYAPIDIPAA-NV----VMS-G--ED---
XP_004954072.1/1-226 NSIL-IL------QE-SCTDA--S--G-SLVVYAPIDVPAA-NV----VMS-G--ED---
XP_002452898.1/1-231 NSML-IL------QE-SCTDA--S--G-SLVVYAPIDIPAA-NV----VMS-G--ED---
NP_001142912.2/1-230 NSML-IL------QE-SCTDG--T--G-SLVVYAPIDIPAA-NV----VMS-G--ED---
XP_020089796.1/1-231 SSML-IL------QE-SCTDA--S--G-SLVVYAPIDIPAI-NI----VMS-G--ED---
XP_020089795.1/1-241 SSML-IL------QE-SCTDA--S--G-SLVVYAPIDIPAI-NI----VMS-G--ED---
XP_019708739.1/1-228 NNML-IL------QE-SCTDA--S--G-SLVVYAPIDLPAI-NI----VMS-G--ED---
XP_008789232.1/1-228 NSML-IL------QE-SCTDA--S--G-SLVVYAPIDLPAI-NI----VMS-G--ED---
XP_008789231.1/1-228 NSML-IL------QE-SCTDA--S--G-SLVVYAPIDLPAI-NI----VMS-G--ED---
XP_008789233.1/1-200 NSML-IL------QE-SCTDA--S--G-SLVVYAPIDLPAI-NI----VMS-G--ED---
XP_019704584.1/1-228 NSML-IL------QE-SCTDA--W--G-SLVVYAPIDLPAI-NI----VMS-G--ED---
XP_010917295.1/1-228 NSML-IL------QE-SCTDA--W--G-SLVVYAPIDLPAI-NI----VMS-G--ED---
XP_008806630.1/1-228 NNML-IL------QE-SCTDA--S--G-SLVIYAPIDLPSI-NI----VMS-G--ED---
XP_009388746.1/1-226 NTML-IL------QE-CCTDA--Y--G-SVIVYSPVDLPAI-NI----VMS-G--ED---
XP_018675467.1/1-228 NTML-IL------QE-CCTDA--S--G-SVVVYSPVDLPAI-NI----VMS-G--ED---
XP_018675466.1/1-228 NTML-IL------QE-CCTDA--S--G-SVVVYSPVDLPAI-NI----VMS-G--ED---
XP_009409108.1/1-228 NTML-IL------QE-CCTDA--S--G-SVVVYSPIDLPAI-NI----VMS-G--ED---
XP_024625408.1/1-220 NNML-IL------QE-SCVDS--S--GS-LVVSCPVDSSIM-----------S-EVD---
XP_010550261.1/1-219 SGML-ML------QE-SFMDS--L--GG-MMVYAPIDIPTM-HV----AVN-G-D-D---
XP_018816719.1/1-228 SNML-ML------QE-SWIDS--L--GS-LVVYAPIDMPSI-SM----AMS-G-E-D---
XP_008365756.2/1-218 DNML-IL------QE-CYIDP--L--GS-FVIYAPVDMQIL-NV----AVX-G-E-D---
XP_008381522.1/1-218 DNML-IL------QE-CYIDP--L--GS-FXIYAPVDMQIL-NV----AVS-G-E-D---
XP_018506060.1/1-218 DNML-ML------QE-CHIDP--L--GS-FIIYAPVDIQSL-NV----AIS-G-E-D---
XP_008362571.1/1-218 DNML-XL------QE-CHIDP--L--GS-XIIYAPVDIQTL-NV----AVS-G-E-D---
XP_023870350.1/1-229 NNML-ML------QE-TCTDT--T--GS-LVVYAPMDIPAI-NI----AMN-G-D-D---
XP_016649213.1/1-222 NNMV-ML------QE-SYIDS--L--GA-LVIYAPVDIPAL-NI----AVC-G-E-D---
XP_020415996.1/1-222 NNMV-ML------QE-SYIDS--L--GA-LVIYAPVDIPAL-NI----AVC-G-E-D---
XP_021823813.1/1-222 NNML-ML------QE-SYIDS--L--GA-LVIYAPVDIPAL-NI----AVC-G-E-D---
XP_009367126.1/1-222 NNML-ML------QE-SYTDP--L--GS-LFVYAPVDVPAL-NV----AVS-G-E-D---
XP_008348615.1/1-222 NNML-ML------QE-SYTDP--L--GS-LFVYAPVDVPAL-NV----AVS-G-E-D---
XP_017179578.1/1-222 NNML-ML------QE-SYTDP--L--GS-LVVYAPVDMPAL-NV----AVS-G-K-D---
XP_009370612.1/1-224 NNML-ML------QE-SYTDP--L--GS-IFVYAPVDMPAL-NV----AVS-G-E-D---
XP_012845089.1/1-228 N-IL-IL------QE-SIIDS--L--GA-TIVYAPIDLPAV-SS----VVN-G-E-D---
XP_012845088.1/1-228 N-IL-IL------QE-SIIDS--L--GA-TIVYAPIDLPAV-SS----VVN-G-E-D---
XP_012845087.1/1-228 N-IL-IL------QE-SIIDS--L--GA-TIVYAPIDLPAV-SS----VVN-G-E-D---
XP_011085930.1/1-227 NNML-ML------QE-SSIDP--L--GA-IMIYAPIDLQAI-IS----VVN-G-E-D---
XP_020551334.1/1-222 NNML-ML------QE-SSIDP--L--GA-IMIYAPIDLQAI-IS----VVN-G-E-D---
XP_021297998.1/1-224 NILM-LQ------EM-SCTES--L--GS-MVVYAPMGIAAM-NT----AIN-G-G-D---
XP_017974480.1/1-224 NILM-LQ------EM-SCTES--L--GS-MVVYAPMGIAAM-HT----AIN-G-G-D---
XP_024448785.1/1-229 NNML-IL------QE-SCTDS--S--GS-MVVYAPLDIPAM-NM----VIG-G-A-D---
XP_024455385.1/1-229 NNML-IL------QE-SCTDS--S--GS-MVVYAPLDIPAM-NM----VIG-G-A-D---
XP_011005937.1/1-269 NNML-IL------QE-SCTDS--S--GS-MVVYAPLDIPAM-NM----VID-G-A-D---
XP_011038413.1/1-229 NNML-IL------QE-SCTDS--S--VS-MVVYAPVGIPAM-NV----AIS-G-E-D---
XP_018731153.1/1-226 ENML-ML------QE-SCIDP--L--TC-MVVYAPIDSTAM-TI----ALS-G-E-D---
XP_024190515.1/1-228 GNML-LL------QE-SYIDT--L--GS-MVIYAPVEVATI-NI----AIG-G-EVD---
XP_012074722.2/1-231 NNML-LL------QE-SCTDA--L--GS-LVVYAPIDITSL-NI----AMT-S-E-D---
XP_010089514.2/1-229 NNML-ML------QE-SCIDR--L--GS-LIVYAPIDVTTL-NM----AIT-G-Q-D---
XP_024949330.1/1-235 NNML-ML------QE-SCIDQ--L--GS-MVVYAPIDIPSM-NL----AIS-G-D-D---
XP_006421085.1/1-235 NNML-ML------QE-SCIDQ--L--GS-MVVYAPIDIPSM-NL----AIS-G-D-D---
XP_002268272.1/1-229 NNML-ML------QE-SCVEP--L--GG-LVVYAPIDIPAI-NV----AIR-G-E-E---
XP_004140733.2/1-228 NNML-IL------QE-SCIDP--L--GS-LVIYAPIDMPAM-NI----ATS-G-Q-D---
XP_008457114.1/1-228 NNML-IL------QE-SCIDP--L--GS-LVIYAPIDMPAM-NI----ATS-G-Q-D---
XP_008457113.1/1-229 NNML-IL------QE-SCIDP--L--GS-LVIYAPIDMPAM-NI----ATS-G-Q-D---
XP_011651208.1/1-229 NNML-IL------QE-SCIDP--L--GS-LVIYAPIDMPAM-NI----ATS-G-Q-D---
XP_022143367.1/1-228 NNML-IL------QE-SCTDP--L--GS-LVIYAPVDMPAM-NV----AVS-G-Q-D---
XP_022935490.1/1-228 NNML-IL------QE-SCIDP--L--GS-LVIYAPIDMPAM-NI----ATS-G-Q-D---
XP_022984101.1/1-228 NNML-IL------QE-SCIDP--L--GS-LVIYAPIDMPAM-NI----ATS-G-Q-D---
XP_022935488.1/1-229 NNML-IL------QE-SCIDP--L--GS-LVIYAPIDMPAM-NI----ATS-G-Q-D---
XP_022984098.1/1-229 NNML-IL------QE-SCIDP--L--GS-LVIYAPIDMPAM-NI----ATS-G-Q-D---
XP_022984102.1/1-229 NNML-IL------QE-SCIDP--L--GS-LVIYAPIDMPAM-NI----ATS-G-Q-D---
XP_015579295.1/1-234 NNML-ML------QE-SCTDP--S--GS-LVVYAPVDIPSM-NI----AIS-G-E-D---
XP_002526595.2/1-234 NNML-ML------QE-SCTDP--S--GS-LVVYAPVDIPSM-NI----AIS-G-E-D---
XP_015579294.1/1-234 NNML-ML------QE-SCTDP--S--GS-LVVYAPVDIPSM-NI----AIS-G-E-D---
XP_021643520.1/1-232 NNMV-ML------QE-SCTDP--L--GS-FIVYAPIDVPTM-NI----AIS-G-E-D---
XP_021605433.1/1-231 NNMV-IL------QE-SCTDP--L--GS-LLVYAPIDIPTM-NM----AIN-G-E-D---
XP_023739098.1/1-228 TNIL-IL------QE-SCTDS--S--GS-LVVYYPVDLPAI-NM----AMN-G-E-E---
XP_024966239.1/1-227 TNIL-IL------QE-SCTDS--S--GS-LVVYCPVDLPVI-NI----ALS-G-E-D---
XP_025813558.1/1-232 DGML-IL------QE-SCTDA--S--GSSLVVYSPIDLLTA-NV----VMS-G-E-D---
XP_020150479.1/1-234 ATML-VL------QE-SCTDT--S--GCSMVVHSPIDIPAA-NM----MMS-G-A-D---
XP_003564122.2/1-224 DSML-VL------QE-SCADP--S--GS-MLVYSPIDMPAV-NL----VTS-G-E-D---
XP_006585049.2/1-225 NNMI-VL------QE-SYADG--L--G-SMMVYSAFDTETM-NF----AMR-G--ED---
XP_007143783.1/1-226 NNIV-VL------QE-SYVDA--L--G-CVMLYAPFDMKAL-NY----AMN-G--ED---
XP_007158859.1/1-226 NNIV-VL------QE-SYVDA--L--G-CVMLYAPFDMKAL-NY----VMN-G--DD---
XP_020230815.1/1-227 NNVV-MI------QE-SSFDP--L--G-SYVVYSPLNILDI-RR----AIN-G--ED---
XP_006586851.1/1-227 NNVV-II------QE-SCTDP--L--G-SYVVYSSTNILDI-KR----TIN-G--ED---
XP_021888058.1/1-234 EGMV-LA------QE-SFIDS--L--G-GMVVYAPIDVPAM-NM---AITS-D--DI---
XP_020886111.1/1-226 DDML-ML------QD-CYMDA--L--G-GMIVYAPMDMATM-NF---AVSG-E--VD---
XP_020886110.1/1-228 DDML-ML------QD-CYMDA--L--G-GMIVYAPMDMATM-NF---AVSG-E--VD---
XP_011469296.1/1-224 SNML-IL------QE-SYIDP--L--G-SMIIYAPIEVATI-NI---TVGG-E--LN---
XP_009367128.2/1-218 DNML-IL------QE-CYIDP--L--G-SFIIYAPVDMQI--LN---VAVS-G--ED---
XP_008348673.1/1-198 DNML-ML------XE-CYIDP--L--G-SFIIYAPVDMQII-LN---VAVS-G--ED---
XP_012832506.1/1-227 NKML-VL------QE-SSIDS--L--G-AAIIYAPMDLKAL-LS----VVN-G--DD---
XP_012832508.1/1-227 NKML-VL------QE-SSIDS--L--G-AAIIYAPMDLKAL-LS----VVN-G--DD---
XP_012832516.1/1-227 NKMV-VL------QE-SSIDS--L--G-AAIIYAPMDLKAL-LS----VVN-G--ED---
XP_012832518.1/1-227 NKMV-VL------QE-SSIDS--L--G-AAIIYAPMDLKAL-LS----VVN-G--ED---
XP_012835569.1/1-227 NKMV-VL------QE-SSIDS--L--G-AAIIYAPMDLKAL-LS----VVN-G--ED---
XP_012835571.1/1-226 NKMV-VL------QE-SSIDS--L--G-AAIIYAPMDLKAL-LS----VVN-G--ED---
XP_019172066.1/1-226 SSML-ML------QE-NCVNS--L--E-GHVVYAPMDLPVI-TA----TMN-G--DD---
XP_009631364.2/1-226 NNML-LL------QE-TNIDP--M--G-AFIIYAPIDLPTV-TS----IIN-G--ED---
XP_016435660.1/1-229 NNML-LL------QE-TNIDP--M--G-AFIIYAPIDLPTV-TS----IIN-G--AD---
XP_009771801.1/1-226 NNML-LL------QE-TNIDP--M--G-AFIIYAPIDLPAV-TS----IIN-G--AD---
XP_016493115.1/1-226 NNML-LL------QE-TNIDP--M--G-AFIIYAPIDLPAV-TS----IIN-G--AD---
XP_019248352.1/1-226 NNML-LL------QE-TNIDP--T--G-AFIIYAPIDLATV-TS----IIN-G--AD---
XP_025691463.1/1-225 NNTL-LL------QE-CCTDF--L--G-SVVVYAPVDVGTI-TA----AIN-G--ED---
XP_020995109.1/1-225 NNTL-LL------QE-CCTDF--L--G-SVVVYAPVDVGTI-TA----AIN-G--ED---
XP_016190415.2/1-225 NNTL-LL------QE-CCTDF--L--G-SVVVYAPVDVGTI-TA----AIN-G--ED---
XP_004486568.1/1-232 NHTL-IL------QE-SFTSP--V--G-SYVIYAATDTTSM-NV----AIR-G--ED---
XP_024632687.1/1-226 RQMM-IL------QE-SFTSR--V--G-SYIIYAPSDRQTM-DV----ALR-G--ED---
XP_020977993.1/1-241 NNAV-II------QE-SFIDP--V--GSSYIVYAPVDVEAI-NV----AIS-G--DD---
XP_025647253.1/1-241 NNAV-II------QE-SFIDP--V--GSSYIVYAPVDVEAI-NV----AIS-G--DD---
XP_020997095.1/1-240 NNAV-II------QE-SFIDP--V--GSSYIVYAPADVEAI-HV----AIS-G--ED---
XP_025692389.1/1-228 NNAV-II------QE-SFIDP--V--GSSYIVYAPADVEAI-HV----AIS-G--ED---
XP_019418389.1/1-226 N-MV-IL------QE-NFTDS--T--G-SYFIYAPLDVASS-NV----AIK-G--ED---
XP_007147460.1/1-227 NNAL-IL------QE-SFTTP--M--G-TYVIYAPTDVATM-TS----AIM-G--ED---
XP_014517364.1/1-227 NNAL-IL------QE-SFTTP--M--G-SYVIYAPTDVATM-TA----AIM-G--EN---
XP_017435438.1/1-226 NNAL-IL------QE-SFTTP--M--G-SYVIYAPTDVATM-TA----AIM-G--EN---
XP_017435439.1/1-217 NNAL-IL------QE-SFTTP--M--G-SYVIYAPTDVATM-TA----AIM-G--EN---
XP_025981664.1/1-227 NNAL-IL------QE-SFTTP--M--G-SYVVYAPTDVASM-IS----AIN-G--ED---
XP_006586853.1/1-227 NNAL-VL------QE-SFTTP--M--G-SYVVYAPTDVAAM-NS----AIN-G--ED---
XP_020230831.1/1-226 NNAL-IL------QE-SFTNP--M--G-SYVVYAPTDVATM-NL----AIK-G--ED---
XP_004489663.1/1-217 KNVL-IL------QE-NFTSH--V--G-SYVIFAGIDIASM-NV----AIR-G--ED---
XP_018489833.1/1-227 KKMM-LL------QE-CYMDA--L--G-GMIVYAPLDMALM-SI----AAS-G-KVD---
XP_013584487.1/1-219 KKMM-VL------QE-CYMDA--L--G-GMIVYSPLDMATM-SI----AAS-G-EVD---
XP_009130351.1/1-219 KKMM-VL------QE-CYMDA--L--G-GMIVYSPLDMATM-SI----AAS-G-EVD---
XP_013747906.2/1-219 KKMM-VL------QE-CYMDA--L--G-GMIVYSPLDMATM-SI----AAA-G-EVD---
XP_013747661.1/1-219 KKMM-VL------QE-CYMDA--L--G-GMIVYSPLDMATM-SI----AAA-G-EVD---
XP_013619678.1/1-219 KKMM-VL------QE-CYMDA--L--G-GMIVYSPLDMATM-SI----AAS-G-EVD---
XP_022551084.1/1-219 KKMM-VL------QE-CYMDA--L--G-GMIVYSPLDMATM-SI----AAS-G-EVD---
XP_009126392.1/1-219 KTMM-VL------QE-CYMDA--L--G-GMIVYSPLDMATM-SI----AAS-G-EVD---
XP_018437612.1/1-222 KNMM-ML------QD-CYMDE--L--G-GMIVYAPLDMASM-SL----AAS-G-EVD---
XP_013591191.1/1-225 RNMM-ML------QE-CYMDA--L--G-GMIVYAPLDMASM-SL----ATS-G-EVD---
XP_022567005.1/1-225 RNMM-ML------QE-CYMDA--L--G-GMIVYAPLDMASM-SL----ATS-G-EVD---
XP_022547796.1/1-225 RNMM-ML------QE-CYMDA--L--G-GMIVYAPLDMASM-SL----ATS-G-EVD---
XP_009119205.1/1-225 RNMM-ML------QE-CYMDA--L--G-GMIVYAPLDMASM-SL----ATS-G-EVD---
XP_009119199.1/1-225 RNMM-ML------QE-CYMDA--L--G-GMIVYAPLDMASM-SL----ATS-G-EVD---
XP_006408322.1/1-232 DNML-ML------QD-CYMDA--L--G-GMIVYAPLDETTM-NI----AAS-G-EVD---
XP_023642295.1/1-229 GDML-ML------QD-SYMDA--L--G-GMIVYAPMDMATM-NM----ALS-T-NVE---
XP_023642294.1/1-229 GDML-ML------QD-SYMDA--L--G-GMIVYAPMDMATM-NM----ALS-T-NVE---
NP_186976.2/1-228    DDML-ML------QD-CYMDA--L--G-GMIVYAPMDMATM-HF----AVS-G-EVD---
XP_010507998.1/1-229 GDML-ML------QD-CYMDA--L--G-GMLVYAPMDMTTM-DT----TLT-GADVE---
XP_010485661.1/1-229 DDIL-ML------QD-CYMDA--L--G-GMLVYAPMDMATM-DT----TLS-GADVK---
XP_010450732.1/1-230 DDML-ML------QD-CYMDA--L--G-GMLVYAPMDMATM-DT----TLS-GADVK---
XP_010463776.1/1-230 DDML-ML------QD-CYMDA--L--G-GMLVYAPMDMATM-DT----TLS-GADVK---
XP_010463774.1/1-230 DDML-ML------QD-CYMDA--L--G-GMLVYAPMDMATM-DT----TLS-GADVK---
XP_019092960.1/1-229 DDML-ML------QD-CYMDA--L--G-GMLVYAPMDMATM-DT----TLS-GADVK---
XP_022551077.1/1-228 ADMM-II------QD-SFRDA--L--G-GMVVYAPVDLNTA-SA----AVS-G-QVN---
XP_013619236.1/1-228 ADMM-II------QD-SFRDA--L--G-GMVVYAPVDLNTA-SA----AVS-G-QVN---
XP_009126177.1/1-226 ADMM-II------QD-SFRDA--L--G-GMVVYAPVDLNTA-SA----AVS-G-QVN---
XP_013652421.1/1-226 ADMM-II------QD-SFRDA--L--G-GMVVYAPVDLNTA-SA----AVS-G-QVN---
XP_013652420.1/1-226 ADMM-II------QD-SFRDA--L--G-GMVVYAPVDLNTA-SA----AVS-G-QVN---
XP_006400265.1/1-228 GELM-IL------QD-SFIDA--L--G-GMVVYAPMDLNTT-YA----AVS-G-QVD---
XP_013590801.1/1-226 GDLM-II------QD-SFIDA--L--G-GMVVYAPVDLNTA-HA----AVS-G-NVN---
XP_013651910.1/1-222 GDLM-II------QD-SFIDA--L--G-GMVVYAPVDLSTA-HA----AVS-G-NVN---
XP_009103958.1/1-222 GDLM-II------QD-SFIDA--L--G-GMVVYAPVDLSTA-HA----AVS-G-NVN---
XP_018444485.1/1-226 GDLM-II------QD-SFIDA--L--G-GMVVYAPVDLMTA-LA----AFS-G-NVD---
XP_009121397.1/1-226 GALM-II------QD-SFIDA--L--G-GMVVYAPVDLRTA-HA----AFL-G-NVD---
XP_013659428.1/1-226 GALM-II------QD-SFIDA--L--G-GMVVYAPVDLRTA-HA----AFL-G-NVD---
XP_022565333.1/1-226 GALM-II------QD-SFIDA--L--G-GMVVYAPVDLRTA-HA----AFL-G-NVD---
XP_013610327.1/1-226 GALM-II------QD-SFIDA--L--G-GMVVYAPVDLRTA-HA----AFL-G-NVD---
XP_006286655.1/1-225 NELM-IL------QD-SFIDA--L--G-GMVVYAPMDLETA-AS----AIS-G-QID---
XP_010492677.1/1-224 SKLM-IL------QD-GFIDA--L--G-GMVVYAPMDLKTA-AA----AIS-G-QVD---
XP_010420445.1/1-224 SNLM-IL------QD-GSIDA--L--G-GMVVYAPMGLETA-AI----ASA-G-QVD---
XP_010453918.1/1-224 SKLM-IL------QD-GFIDA--L--G-GMVVYAPMDLDTA-AA----AIS-G-QVD---
XP_006303739.1/1-224 TEMM-IL------QD-SFIDE--L--G-GMVVYAPMDLKTA-YA----AIS-G-DVD---
XP_020876074.1/1-222 NKLM-IL------QD-SFKDA--L--G-GMVVYAPMDLNTA-FA----AIS-G-EVV---
NP_197234.1/1-222    TKLM-IL------QD-SFKDA--L--G-GMVAYAPMDLNTA-CA----AIS-G-DID---
NP_174724.1/1-221    YKLM-IL------QD-GFIDA--L--G-GMVVYAPMNLNTA-YS----AIS-G-QVD---
XP_020869876.1/1-222 SELM-IL------QD-GFIDA--L--G-GMVVYAPMDIKTA-YS----AIS-G-QVD---
XP_019091098.1/1-222 SELM-IL------QD-GFIDA--L--G-GMFVYAPVDLNTA-YS----AIS-G-QVD---
XP_010478910.1/1-223 SELM-IL------QD-GFIDA--L--G-GMFVYAPVDLNTA-YS----AIS-G-QVD---
XP_010501781.1/1-221 SELM-IL------QD-GFIDA--L--G-GMFVYAPVDLNTA-YS----AIS-G-QVD---
XP_015089790.1/1-222 NNML-LL------EE-SSIDE--M--G-AFLIYGPIDLPIV-TS----ITN-E--GD---
XP_015089784.1/1-222 NNML-LL------EE-SSIDE--M--G-AFLIYGPIDLPIV-TS----IAI-G--GD---
XP_016549855.1/1-224 SNML-MI------QE-SNIDE--M--G-AFIIYAPIDLPTV-TS----VIN-G--GD---
XP_015057403.1/1-221 KEKL-VL------QE-SSIDE--M--G-AFLIFAPIELPTM-TS----IFN-G--HD---
XP_019066399.1/1-221 MEKL-VL------QE-SSIDE--M--G-AFLIFAPLELPTM-TS----IFN-G--HD---
XP_015166059.1/1-216 KEML-VL------QE-TSIDS--M--G-AFLVYAPIKLPTV-TS----IVN-G--GD---
XP_019071012.2/1-216 KEML-VI------QE-TSIDS--M--G-AFLVYAPIDLQAI-TS----IVN-G--GD---
XP_015086709.1/1-216 KEML-VL------QE-TSIDS--M--G-AFLVYAPIDLRAI-TS----IVN-G--GD---
XP_015087432.1/1-219 KEML-VL------EE-TSIDE--M--G-AFLVYAPIEIRAI-TS----IVN-G--VD---
XP_004247342.2/1-219 KEML-VL------EE-TSIDE--M--G-AFLVYAPIEIRAI-TS----IVN-G--VD---
XP_025888717.1/1-219 KEML-VL------EE-TSIDE--M--G-AFLVYAPIEIRAI-TS----IVN-G--VD---
XP_015087599.1/1-221 KEML-VL------EE-KSINE--M--G-AFLVYAPIELRAI-TS----IVN-G--CD---
XP_019071156.1/1-221 KEML-VL------EE-KSINE--M--G-AFLVYAPIELRVI-TS----IVN-G--CD---
XP_019070762.2/1-220 KEML-VL------EE-TSIDE--M--G-AFLVYAPIDLRAI-NS----IVN-G--GD---
XP_015087071.1/1-220 KEML-LL------EE-TGIDE--M--G-AFLVYAPIDLRAI-NS----IVN-G--GD---
XP_025888845.1/1-217 KEML-VL------EE-TSIDE--M--G-AFLVYAPIDLRAI-NS----IVN-G--GD---
XP_015170367.1/1-232 NNIL-VL------QE-SGIDE--I--G-AFLIYAPIDAPTV-NL----IIN-G--GD---
XP_025886268.1/1-232 KNML-IL------QE-SAIDE--L--G-AFLIYAPIDLSTV-NS----IFN-G--GD---
XP_015072491.1/1-224 KKML-IL------QE-SAIDE--L--G-AFLIYAPIDLSTV-NS----IFK-G--SD---
XP_010327374.2/1-219 NNMV-LL------QE-SGIDK--M--G-AFLIYGLIDSTAY-KS----FLS-G--GN---
XP_015089749.1/1-219 NNMV-LL------QE-SGIDK--M--G-AFLIYGLIDSTAY-KS----FLS-G--GN---
XP_015170971.1/1-209 NNML-VL------QE-SNIDD--M--G-AFLIYGTIDLSTY-NS----IVN-G--SD---
XP_025887726.1/1-223 NK-L-LL------QE-SNIDE--M--G-AFLIYGLIDLSTY-NS----IVD-G--SD---
XP_006586358.1/1-227 NNMV-IL------QE-SYVDA--L--G-SMLVFAPFYMEGL-DL----IMK-G--ED---
XP_014617351.1/1-228 NNVV-II------QE-SCTDP--L--G-SYVVYSSTNILDI-KR----TIN-G--ED---
XP_007147461.1/1-227 NNMA-II------QE-SCIDP--L--G-SYVVYSPINISDI-KM----AIN-G--EE---
XP_017435408.1/1-226 NNMA-II------QE-SFIDA--L--G-SYVVYSPVNISDI-KM----AVN-G--KD---
XP_017215828.1/1-223 KSVR-IL------QD-CCTNA--F--E-STVVYAPVDISSM-QS---A-MQ--G-CD---
XP_012856110.1/1-229 QSVL-VL------QD-SCTNA--Y--E-SMVVYAPVDIKGL-QS---V-MT--G-CD---
XP_023738891.1/1-226 -NMW-MI------QD-TSTNT--Y--E-SMVVCAPVCVTNM-QS---V-MG--G-CD---
XP_022017964.1/1-227 -NMW-MV------QD-SCTNA--Y--E-SMVVCARVAVSAM-QS---V-MA--G-CD---
XP_022154996.1/1-226 TNVW-VL------QD-SLTNE--Y--E-SMVVYAQVDITSM-KS---V-MA--G-CD---
XP_008462169.1/1-225 SNKW-IL------QD-SLTNE--Y--E-STVVYAEVDMNSM-KS---V-MA--G-FD---
XP_004141841.1/1-226 TNKW-IL------QD-SLTNE--Y--E-STVVYAQVDMNGM-KS---V-MA--G-FD---
XP_020098514.1/1-228 TNKW-LL------QD-SSTNA--Y--E-SIVVYTELDADSL-QS---V-MT--G-CD---
XP_020098513.1/1-228 TNKW-LL------QD-SSTNA--Y--E-SIVVYTELDADSL-QS---V-MT--G-CD---
XP_020263950.1/1-229 NCMW-IL------QD-SCTNA--Y--E-SMIVYAPVDSNGM-QK---V-LT--G-CD---
XP_020263948.1/1-255 NCMW-IL------QD-SCTNA--Y--E-SMIVYAPVDSNGM-QK---V-LT--G-CD---
XP_009415234.2/1-232 TNIW-VL------QD-CSTNS--Y--E-SMVVFAPVEIDGT-QS---V-MN--G-CD---
XP_008789333.1/1-230 TSMW-VL------QD-SSTNA--Y--E-SMIVYAPVDIDEM-QT---V-MT--G-RD---
XP_010920420.1/1-235 TSMW-VL------QD-SSTNA--Y--E-SMIVYAPVDIEEM-QS---V-MT--G-CD---
XP_010920411.1/1-235 TSMW-VL------QD-SSTNA--Y--E-SMIVYAPVDIEEM-QS---V-MT--G-CD---
XP_010920404.1/1-235 TSMW-VL------QD-SSTNA--Y--E-SMIVYAPVDIEEM-QS---V-MT--G-CD---
XP_010521515.1/1-219 KSMW-VL------QD-SCTNS--Y--E-SAVIYAPVDINAT-QV---A-MG--G-RD---
XP_013652056.1/1-227 KSTW-VL------QD-SCTNS--Y--E-SVVVYAPVDINTT-QM---V-IA--G-HD---
XP_009104428.1/1-227 KSTW-VL------QD-SCTNS--Y--E-SVVVYAPVDINTT-QM---V-IA--G-HD---
XP_018445476.1/1-226 KSTW-VL------QD-SCTNS--Y--E-SVVVYAPVDINTT-QL---V-IA--G-HD---
XP_013590784.1/1-226 KSTW-VL------QD-SCTNS--Y--E-SVVVYAPVDINTT-QL---V-IA--G-HD---
XP_013680822.1/1-226 KSTW-VL------QD-SCTNS--Y--E-SVVVYAPVDINTT-QL---V-IA--G-HD---
XP_006389720.2/1-226 KSIW-VL------QD-SCTNS--Y--E-SVVVYAPVDINTT-QL---V-IA--G-HD---
XP_023642973.1/1-226 KSIW-VL------QD-SCTNS--F--E-SVVVYAPVDINTT-QL---V-LA--G-HD---
XP_020891640.1/1-226 KSIW-VL------QD-SNTNS--Y--E-SVVVYAPVDINTT-QL---V-LA--G-HD---
NP_001320267.1/1-226 KSIW-VL------QD-SSTNS--Y--E-SVVVYAPVDINTT-QL---V-LA--G-HD---
NP_565223.1/1-226    KSIW-VL------QD-SSTNS--Y--E-SVVVYAPVDINTT-QL---V-LA--G-HD---
NP_001185443.1/1-226 KSIW-VL------QD-SSTNS--Y--E-SVVVYAPVDINTT-QL---V-LA--G-HD---
XP_010473021.1/1-226 KSIW-VL------QD-SCTNS--Y--E-SVVVYAPVDINTT-QL---V-LA--G-HD---
XP_010430051.1/1-226 KSIW-VL------QD-SSTNS--Y--E-SVVVYAPVDINTT-QL---V-LA--G-HD---
XP_025617667.1/1-241 IKWW-IL------QD-SSTNA--Y--E-SMVVYAPVDIPSM-KN---V-MN--G-CD---
XP_015938429.1/1-242 IKWW-IL------QD-SSTNA--Y--E-SMVVYAPVDIPSM-KN---V-MN--G-CD---
XP_016176999.1/1-241 IKWW-IL------QD-SSTNA--Y--E-SMVVYAPVDIPSM-KN---V-MN--G-CD---
XP_021862846.1/1-225 KITW-VL------QD-SSTNS--Y--E-SMVVYAPVEIPGL-QS---V-MT--G-CD---
XP_022979290.1/1-231 SSMW-IL------QD-SSTNS--S--E-SMVVYSGVDVTGM-QS---V-MT--G-CD---
XP_022955725.1/1-233 SGMW-IL------QD-SSTNS--S--E-SMVVYSGVDVTGM-QS---V-MT--G-CD---
XP_022998610.1/1-231 SSMW-IL------QD-TSTNS--S--E-SMVVYSGVDVSGM-QS---V-MT--G-CD---
XP_022998611.1/1-227 SSMW-IL------QD-TSTNS--S--E-SMVVYSGVDVSGM-QS---V-MT--G-CD---
XP_023523626.1/1-229 SSMW-IL------QD-ASTNS--S--E-SMVVYSGVDVAGM-QS---V-MT--G-CD---
XP_022948296.1/1-231 SSMW-IL------QD-ASTNS--S--E-SMVVYSGVDVSGM-QS---V-MT--G-CD---
XP_023523628.1/1-225 SSMW-IL------QD-ASTNS--S--E-SMVVYSGVDVAGM-QS---V-MT--G-CD---
XP_022948295.1/1-227 SSMW-IL------QD-ASTNS--S--E-SMVVYSGVDVSGM-QS---V-MT--G-CD---
XP_010249693.1/1-228 SSVW-IL------QD-SCTNA--Y--E-SMVVYAPVDITGT-QS---V-IT--G-CD---
XP_010254081.1/1-230 SSVW-VL------QD-SCTNA--Y--E-SMVVYAPVDIHGM-QT---V-MS--G-CD---
XP_003529763.1/1-227 NSVW-IL------QD-SCTSA--Y--E-STVVYAPVEFAGI-QS---V-LT--G-CD---
XP_003533048.1/1-226 NSVW-IL------QD-SCTSA--Y--E-SMVVYAPVEFAGI-QS---V-LT--G-CD---
XP_014634625.1/1-226 NSVW-IL------QD-SCTSA--Y--E-SMVVYAPVEFAGI-QS---V-LT--G-CD---
XP_007150635.1/1-226 KSVW-IL------QD-SCTNP--Y--E-SMVVYASVDITGI-QS---V-MT--G-CD---
XP_014498860.1/1-226 NSVW-IL------QD-SCTNP--Y--E-SMVVYASVDISGI-QS---V-MT--G-CD---
XP_017425661.1/1-226 NSVW-IL------QD-SCTNP--Y--E-SMVVYASVDITGI-QS---V-MT--G-CD---
XP_020207725.1/1-226 NSVW-IL------QD-SCTNP--Y--E-SMVVYASVDITGI-QS---V-MT--G-CD---
XP_003546423.2/1-226 NSVW-IL------QD-SYTNP--Y--E-SMVVYASVDITGT-QS---V-MT--G-CD---
XP_003543638.1/1-226 NSVW-IL------QD-SCTNL--Y--E-SMVAYACVDITGI-QS---V-MT--G-CD---
XP_019457980.1/1-226 NNMW-IL------QD-NSTNP--Y--E-SMVVYAPVDIAGI-QS---V-MT--G-CD---
XP_003597736.1/1-226 NNMW-IL------QD-SCTNS--Y--E-SMVVYAPADITGI-QS---V-MT--G-CD---
XP_004486751.1/1-226 NNMW-IL------QD-SCTNS--Y--E-SMVVYAPVDITGI-QS---V-MT--G-CD---
XP_016169615.1/1-226 NSMW-VV------QD-CCTNA--Y--E-SMVVYAPVDITGI-QS---A-MT--G-CD---
XP_025611006.1/1-226 NSMW-VV------QD-CCTNA--Y--E-SMVVYAPVDITGI-QS---A-MT--G-CD---
XP_020982478.1/1-227 NSMW-VV------QD-CCTNA--Y--E-SMVVYAPVDITGI-QS---A-MT--G-CD---
XP_021892835.1/1-225 NSLW-VL------QD-SSINA--Y--E-SMVVYAPVDITGM-QS---V-IT--G-CD---
XP_012442963.1/1-227 NSMW-VL------QD-SCTNA--F--E-SMVVFAHVDVTGI-QS---V-IT--G-CD---
NP_001314068.1/1-227 NSMW-VL------QD-SCTNA--F--E-SMVVFAHVDVTGI-QS---V-IT--G-CD---
XP_016684379.1/1-227 NSMW-VL------QD-SCTNA--F--E-SMVVFAHVDVTGI-QS---V-IT--G-CD---
XP_016730082.1/1-227 NSMW-VL------QD-SCTNA--F--E-SMVVFAHVDVTGI-QS---V-IT--G-CD---
NP_001316938.1/1-227 NSMW-IL------QD-SCTNA--F--E-SMVVFAHVDVTGI-QS---V-IT--G-CD---
XP_017606636.1/1-227 NSMW-IL------QD-SCTNA--F--E-SMVVFAHVDVTGI-QS---V-IT--G-CD---
XP_022714992.1/1-229 NSIW-VL------QD-SCTNA--F--E-SMVVFAPVDITAM-QS---V-IT--G-CD---
XP_022721436.1/1-227 NSMW-VL------QD-SCTNA--F--E-SMVVFAPVDITGM-QS---V-IT--G-CD---
XP_022721435.1/1-227 NSMW-VL------QD-SCTNA--F--E-SMVVFAPVDITGM-QS---V-IT--G-CD---
XP_007024189.1/1-227 NSMW-VL------QD-SCTNA--F--E-SMVIFAPVDIAGM-QS---V-IT--G-CD---
XP_021298731.1/1-227 NSTW-VL------QD-SCTNA--F--E-SMVIFAHVDIAGM-QS---V-IT--G-CD---
XP_004302894.1/1-228 NSMW-IL------QD-SCINA--Y--E-SMVVYAPVDITGM-QS---V-MT--G-CD---
XP_011466687.1/1-235 NSMW-IL------QD-SCINA--Y--E-SMVVYAPVDITGM-QS---V-MT--G-CD---
XP_024180978.1/1-228 NSMW-IL------QD-SCINA--Y--E-SMVVYAPVDITGM-QS---V-MT--G-CD---
XP_024180977.1/1-235 NSMW-IL------QD-SCINA--Y--E-SMVVYAPVDITGM-QS---V-MT--G-CD---
XP_009353605.1/1-226 KSMW-IL------QD-TCINS--Y--E-SMVVYAPVDITGM-QS---V-MT--G-CA---
XP_008380909.1/1-226 KSMW-IL------QD-TCINS--Y--E-SMVVYAPVDTTGM-QS---V-MT--G-CG---
NP_001280927.1/1-227 NSMW-IL------QD-TCINS--Y--E-SMVVYAPVDIPGM-QS---V-MT--G-CD---
XP_009338062.1/1-226 NSMW-IL------QD-TCINS--Y--E-SMVVYAPVDITGM-QS---V-MT--G-CD---
XP_009338046.1/1-226 NSMW-IL------QD-TCINS--Y--E-SMVVYAPVDITGM-QS---V-MT--G-CD---
XP_021829136.1/1-226 NSMW-IL------QD-TCINS--Y--E-SMVVYAPVDITGM-QS---V-MT--G-CD---
XP_008228560.1/1-226 NSMW-IL------QD-TCINS--Y--E-SMVVYAPVDITGM-QS---V-MT--G-CD---
XP_007217160.1/1-226 NSMW-IL------QD-TCINS--Y--E-SMVVYAPVDITGM-QS---V-MT--G-CD---
XP_022891611.1/1-228 QNMW-IL------QD-SCTNA--Y--E-SMVVYAPVDINGM-QS---V-IT--G-CD---
XP_002304207.2/1-228 NNMW-VL------QD-SCTNA--Y--E-SMVIYAPVDTNGM-QS---V-IN--G-CD---
XP_011010522.1/1-228 NNMW-VL------QD-SGTNA--Y--E-SMVIYAPVDTNGM-QS---V-IN--G-CD---
XP_011009828.1/1-227 -NMW-VL------QD-SCTNA--Y--E-SMVVYAPVDTNGM-QS---V-IT--G-CD---
XP_024462179.1/1-228 NNMW-VL------QD-SCTNA--Y--E-SMIVYAPVDTNGM-QS---V-IT--G-CD---
XP_018837483.1/1-226 NNMW-II------QD-SCTNA--Y--E-SMVVFAPVDITGM-QS---V-MT--G-CD---
XP_018856342.1/1-226 NSTW-II------QD-SCTNA--Y--E-SMVVYAPVDITGM-QS---V-MT--G-CD---
XP_018856994.1/1-226 NSTW-II------QD-SCTNA--Y--E-SMVVYAPVDITGM-QS---V-MT--G-CD---
XP_018858360.1/1-226 NSTW-II------QD-SCTNA--Y--E-SMVVYAPVDITGM-QS---V-MT--G-CD---
XP_015867693.1/1-226 NSMW-IL------QD-CCTNA--Y--E-STVVYAQVDITGM-QT---V-MT--G-CD---
XP_010062490.1/1-226 NSMW-VL------QD-CCTNA--Y--E-SMVVYAPVDITGM-QA---V-MT--G-CD---
XP_010098082.1/1-228 NSMW-IL------QD-CCTNA--Y--E-SMVVYAPVDITGM-QS---V-MT--G-CD---
XP_002284502.1/1-228 NSMW-VV------QD-TCTNA--Y--E-SMVVYAPVDIPGM-QS---V-MT--G-CD---
XP_023875609.1/1-226 NSMW-VL------QD-GCTNA--Y--E-SMVVYAPVDITGM-QS---V-MT--G-CD---
XP_002516023.1/1-226 NNMW-VL------QD-CCTNA--Y--E-SIVVYAPVDINGM-QS---V-IT--G-CD---
XP_021652755.1/1-228 NPMW-VL------QD-CCTNA--Y--E-SLVVYAPVDINGM-QS---V-IT--G-CD---
XP_021654904.1/1-226 NPMW-VL------QD-CCTNA--Y--E-SMVVYASVDINGM-QS---V-IT--G-CD---
XP_021611342.1/1-229 NPLW-VL------QD-CCTNA--Y--E-SMVVYAPVDINGM-QS---V-IT--G-CD---
XP_012073376.1/1-226 NTMW-IL------QD-SCTNA--Y--E-SMVVYAAVDISGM-QS---V-IT--G-CD---
XP_006465622.1/1-226 NSMW-LL------QD-SYTNA--Y--E-SMVVYAPVDITGM-QS---V-IT--G-CD---
XP_006426950.1/1-226 NSMW-LL------QD-SYTNA--Y--E-SMVVYAPVDITGM-QS---V-IT--G-CD---
XP_009392419.1/1-229 TNIW-VL------QD-SSTNA--Y--E-SMVVFAPVDIDGM-QS---V-MT--G-CD---
XP_020518333.1/1-227 SSVW-IL------QD-CSTNS--Y--E-SMMVYAPVDIVGM-QS---V-MS--G-CD---
XP_018629167.1/1-227 -DMW-IL------QD-TCTNA--Y--E-STVVYAPIDIGGM-KS---V-MT--G-RD---
XP_009611250.1/1-227 -DMW-IL------QD-TCTNA--Y--E-STVVYAPIDIGGM-KS---V-MT--G-RD---
XP_009611249.1/1-227 -DMW-IL------QD-TCTNA--Y--E-STVVYAPIDIGGM-KS---V-MT--G-RD---
XP_009611248.1/1-227 -DMW-IL------QD-TCTNA--Y--E-STVVYAPIDIGGM-KS---V-MT--G-RD---
XP_016479156.1/1-227 -DMW-IL------QD-TCTNA--Y--E-STVVYAPIDIGGM-KS---V-MT--G-RD---
XP_016479155.1/1-227 -DMW-IL------QD-TCTNA--Y--E-STVVYAPIDIGGM-KS---V-MT--G-RD---
XP_016479154.1/1-227 -DMW-IL------QD-TCTNA--Y--E-STVVYAPIDIGGM-KS---V-MT--G-RD---
XP_009778213.1/1-229 NKMW-IL------QD-TCTNA--Y--E-STVVYAPVDIAGM-QS---V-IT--G-CD---
XP_016501674.1/1-229 NKMW-IL------QD-TCTNA--Y--E-STVVYAPVDIAGM-QS---V-IT--G-CD---
XP_019224885.1/1-229 NNMW-IL------QD-TCTNA--Y--E-SAVVYAPVDIAGM-QS---V-IT--G-CD---
XP_019224886.1/1-229 NNMW-IL------QD-TCTNA--Y--E-SAVVYAPVDIAGM-QS---V-IT--G-CD---
XP_009592772.1/1-229 NNMW-IL------QD-TCTNA--Y--E-SAVVYAPVDIAGM-QS---V-IT--G-CD---
XP_016496403.1/1-229 NNMW-IL------QD-TCTNA--Y--E-SAVVYAPVDIAGM-QS---V-IT--G-CD---
XP_016562690.1/1-228 -NMW-IL------QD-TCTNA--Y--E-SAVVYAPVDIAGM-QS---V-IT--G-CD---
XP_006343080.1/1-229 NNMW-IL------QD-TCTNA--Y--E-SAVVYAPVDIAGM-QS---V-IT--G-CD---
XP_004235676.1/1-229 NNMW-IL------QD-TSTNA--Y--E-SAVVYAPVDIAGM-QS---V-IT--G-CD---
XP_020227921.1/1-228 NSVW-IL------QD-SCTSA--Y--E-SMVVYTPVDIAGI-KS---V-LT--G-CD---
XP_007135673.1/1-226 KSVW-IV------QD-SCTSA--Y--E-SMVAYAAVEFGGL-QS---V-LT--G-CD---
XP_014515030.1/1-226 NSVW-LL------QD-SCTSG--Y--E-SMVAYALVDFASL-QS---V-LT--G-CD---
XP_017407449.1/1-226 NSVW-VL------QD-SCTSG--Y--E-SMVAYALVDFAGL-QS---V-LT--G-CD---
XP_010691342.1/1-224 NMNW-VL------QD-SSINT--F--E-SMVVYAPVDISSI-QS---V-MT--G-CD---
XP_021731998.1/1-222 NMNW-VL------QD-SSTNA--Y--E-SMVVYAPVEISGL-QS---V-MT--G-CD---
XP_021736870.1/1-224 NANW-VL------QD-NSTNA--Y--E-SMVVYAPVEISGL-QS---V-MT--G-CD---
XP_024969955.1/1-229 -NMW-ML------ED-SCTNS--Y--E-SMVVCAPVSVSSM-QS---V-MA--G-CD---
XP_024969954.1/1-229 -NMW-ML------ED-SCTNS--Y--E-SMVVCAPVSVSSM-QS---V-MA--G-CD---
XP_024969953.1/1-229 -NMW-ML------ED-SCTNS--Y--E-SMVVCAPVSVSSM-QS---V-MA--G-CD---
XP_021984354.1/1-227 -NMW-MI------QD-SCTNA--Y--E-SMVVCAPVAVTAM-QS---I-MA--G-CD---
XP_017232370.1/1-228 KSMW-IL------QD-SCTNA--Y--E-STIVYAPVDIGSM-QS---A-MT--G-CD---
XP_017216573.1/1-228 KSVW-IL------QD-SCTNA--Y--E-STIVYAPVDISSM-ES---A-MT--G-CD---
XP_017248777.1/1-227 NSVW-IL------QD-SCTNV--Y--E-STIVYAPVDISGM-QS---V-IT--G-CD---
XP_021627148.1/1-226 NPTW-VI------QD-CCTNA--Y--E-STVVYAPVDVNGM-QS---V-IT--G-CD---
XP_021627146.1/1-226 NPTW-VI------QD-CCTNA--Y--E-STVVYAPVDVNGM-QS---V-IT--G-CD---
XP_021627145.1/1-226 NPTW-VI------QD-CCTNA--Y--E-STVVYAPVDVNGM-QS---V-IT--G-CD---
XP_019196965.1/1-228 NNMW-LL------QD-TCTNV--Y--E-SMVVYAPVDIGSM-QA---V-MT--G-CD---
XP_019196967.1/1-228 NNMW-LL------QD-TCTNV--Y--E-SMVVYAPVDIGSM-QA---V-MT--G-CD---
XP_022848789.1/1-228 QNMW-IL------QD-SCTNA--Y--E-SMVVYAPVDVNGM-QS---V-IA--G-CD---
XP_011072373.1/1-228 HKMW-LL------QD-SSTNA--Y--E-SMVVYGPVDINGM-QS---V-MT--G-CD---
XP_021907286.1/1-215 SNTV-IL------QE-NSIDA--T--G-SFVVYAPMDTTKA-YN---I-LD--G-GD---
XP_020885309.1/1-217 TKMM-IV------QE-TSADP--T--A-SFVLYAPVDMKSM-EI---S-LH-GG-GD---
XP_020885307.1/1-217 TKMM-IV------QE-TSADP--T--A-SFVLYAPVDMKSM-EI---S-LH-GG-GD---
NP_180796.2/1-218    SKMM-IV------QE-TSTDP--T--A-SFVLYAPVDMTSM-DI---T-LH-GG-GD---
XP_006296541.1/1-217 NNMM-VI------QE-TTTDP--T--A-SFVIYAPVETEAI-EA---V-LN-GG-GD---
XP_010510028.1/1-215 KNMM-II------QE-TSTDP--T--A-SFVVYAPVDTASI-EG---V-LS-EG-QD---
XP_010414006.1/1-214 KKMM-II------QE-TSTDP--T--A-SFVIYAPVDTASI-EG---V-LS-EG-QD---
XP_010469602.1/1-219 KKMM-II------QE-TSTDP--T--A-SFVIYAPVDTESI-EG---V-LS-AG-QD---
XP_010417818.1/1-218 TKKM-MI------QE-TFTDP--T--A-SFVIYAPVDTASI-EG---L-LN-LG-TD---
XP_010510869.1/1-220 MNKM-II------QE-TFTDP--T--A-SFVIYAPVDTASI-EG---L-LN-LG-TD---
XP_010510870.1/1-218 MNKM-II------QE-TFTDP--T--A-SFVIYAPVDTASI-EG---L-LN-LG-TD---
XP_018484769.1/1-219 NNKM-MI------QE-TSTDP--T--A-SFVIYAPLDVTLI-EN---V-LK--G-GD---
XP_018484754.1/1-219 NNKM-MI------QE-TSTDP--T--A-SFVIYAPLDVTLI-EN---V-LK--G-GD---
XP_013634882.1/1-219 KKMM-MI------QE-TSTDP--T--A-SFVIYAPLDVTLT-EN---V-LM--G-GD---
XP_018513778.1/1-219 KKMM-MI------QE-TSTDP--T--A-SFVIYAPLDVTLT-EN---V-LM--G-GD---
XP_009141217.1/1-219 KKMM-MI------QE-TSTDP--T--A-SFVIYAPLDVTLT-EN---V-LM--G-GD---
XP_013747219.1/1-219 KKMM-MI------QE-TSTDP--T--A-SFVIYAPLDVTLT-EN---V-LM--G-GD---
XP_024003849.1/1-217 KNMI-IV------QE-TSTDP--A--A-SFVISAPIDVSAM-DL---V-FK--G-GD---
XP_018437853.1/1-211 SEMM-MI------EE-SSIEP--T--A-SFLVYAPLDVPSI-EK---V-LN--G-GD---
XP_009143975.1/1-211 SEMM-MI------EK-SSTEP--A--A-SFLVYAPVSVPSM-EK---V-LN--G-GD---
XP_009143974.1/1-211 SEMM-MI------EK-SSTEP--A--A-SFLVYAPVSVPSM-EK---V-LN--G-GD---
XP_022575029.1/1-211 SEMM-MI------EE-SSTEP--A--A-SFLVYAPVDIPSM-EK---V-LN--G-GD---
XP_013748684.1/1-211 SEMM-MI------EE-SSTEP--A--A-SFLVYAPVDIPSM-EK---V-LN--G-GD---
XP_013694194.1/1-211 SEMM-MI------EE-SSTEP--A--A-SFLVYAPVDVPSM-EK---V-LN--G-GD---
XP_013634261.1/1-211 SEMM-MI------EE-SSTEP--A--A-SFLVYAPVDVPSM-EK---V-LN--G-GD---
XP_013741430.1/1-210 SEMM-MI------EE-SSTEP--A--A-SFLVYAPVDVPSM-EK---V-LN--G-GD---
XP_017254373.1/1-221 GNVH-IL------QE-SCADP--T--V-SYVIYAPVNIDSV-NV---V-FC--G-GD---
XP_017254240.1/1-222 SNTH-IL------KE-TRVDS--T--A-SYVIYTSVDTTPL-NV---V-LS--G-GD---
XP_017221582.1/1-224 SNKY-IL------QE-SCADP--T--A-SYVIYAPVDRISM-NV---V-LS--G-GD---
XP_017221584.1/1-224 SNKY-IL------QE-SCADP--T--A-SYVIYAPVDRISM-NV---V-LS--G-GD---
XP_015941575.1/1-215 NGMI-IV------QE-SSSDS--T--G-SFVIYAPVDMNSI-NA---V-IG--G-RD---
XP_025620358.1/1-215 NGMI-IV------QE-SSSDS--T--G-SFVIYAPVDMNSI-NA---V-IG--G-RD---
XP_016176493.1/1-215 NGMI-IV------QE-SSSDS--T--G-SFVIYAPVDMNSI-NA---V-IG--G-RD---
XP_003610661.2/1-228 SDVV-VL------QE-NCTDV--T--G-SYVVYAPVQIPTM-HE---I-LN--G-GD---
XP_004496853.1/1-220 NNMV-LL------QE-NSTDS--T--G-SYVIYAPVDYGAM-NV---V-MN--G-GD---
XP_006353342.1/1-230 NSML-TL------QE-SCTDV--S--G-SIIAYTSLNSGDM-NV---V-MS--G-GD---
XP_015070591.1/1-229 NSLL-TL------QE-SCTDV--S--G-SIIAYSSLNTGDM-NA---V-MN--G-GD---
XP_019068356.1/1-229 NGLL-TL------QE-SCTDV--S--G-SIIAYTSLNTGDM-NG---V-MN--G-GD---
XP_004234441.1/1-229 NGLL-TL------QE-SCTDV--S--G-SIIAYTSLNTGDM-NG---V-MN--G-GD---
XP_024926606.1/1-217 VHML-YL------QE-SRTDN--T--G-SYVVYAPVEVSAM-SM---V-FN--G-GN---
XP_024525400.1/1-231 NQML-FL------QE-SCTDA--S--G-SLVVYAPVELTMI-NM---M-IQ--G-GD---
XP_002965186.2/1-231 NQML-FL------QE-SCTDA--S--G-SLVVYAPVELTMI-NM---M-IQ--G-GD---
XP_002962272.2/1-231 NQML-FL------QE-SCTDT--S--G-SLVVYAPVELTMI-NM---M-IQ--G-GD---
XP_019441536.1/1-226 QNMF-IL------QE-SFTDA--T--G-SYAIYAPIDVGPM-NE---A-LC--G-GD---
XP_019441537.1/1-226 QNMF-IL------QE-SFTDA--T--G-SYAIYAPIDVGPM-NE---A-LC--G-GD---
XP_019441535.1/1-226 QNMF-IL------QE-SFTDA--T--G-SYAIYAPIDVGPM-NE---A-LC--G-GD---
XP_019451758.1/1-229 HNMF-IL------QE-SCSDC--T--G-SYVVYAPIGINPM-HK---I-LS--G-GE---
XP_012850835.1/1-238 GKMI-LL------QE-SRTDQ--T--A-SYVVYAPMDPVYV-SA---T-IG--G-GD---
XP_020552079.1/1-225 RQ-T-TL------LE-SRTDP--T--A-SYVVYAPIDMVCL-NA---I-LG--G-GD---
XP_020552078.1/1-225 RQ-T-TL------LE-SRTDP--T--A-SYVVYAPIDMVCL-NA---I-LG--G-GD---
XP_020552080.1/1-223 RQ-T-TL------LE-SRTDP--T--A-SYVVYAPIDMVCL-NA---I-LG--G-GD---
XP_009408807.2/1-226 TNMM-IL------QE-SCSDP--T--C-SYVIYAPVDAAAI-AA---V-LS--G-GD---
XP_006342404.1/1-225 NDML-IF------QE-SITDP--T--G-SFIVYAPIDIRAI-DM---V-LC--G-GN---
XP_025887998.1/1-218 NGML-II------QE-SISDP--T--G-SFIVYAPIDIRAI-DM---I-LC--G-GN---
XP_015081481.1/1-225 NGML-II------QE-SITDP--T--G-SFIVYAPIDIRAI-DI---I-LC--G-GN---
XP_022000230.1/1-232 NSVL-VL------QD-STTDV--T--G-SLMVYAAVDVQAM-TV---V-MS--G-GD---
XP_022000229.1/1-232 NSVL-VL------QD-STTDV--T--G-SLMVYAAVDVQAM-TV---V-MS--G-GD---
XP_022037790.1/1-222 NSVL-VL------QD-SATDV--T--G-SLMVYAAVDVEAI-TV---V-MN--G-GD---
XP_022037789.1/1-222 NSVL-VL------QD-SATDV--T--G-SLMVYAAVDVEAI-TV---V-MN--G-GD---
XP_024991925.1/1-226 NTVL-VL------QE-STTDV--T--G-SLIVYAAVDVQSI-TV---V-MN--G-GD---
XP_023760005.1/1-229 NTVL-VL------QE-SSTDV--S--G-SLIVYAAVDINSI-DV---V-MR--G-GD---
XP_023760001.1/1-229 NTVL-VL------QE-SSTDV--S--G-SLIVYAAVDINSI-DV---V-MR--G-GD---
XP_017251723.1/1-236 NNML-RL------QA-TSTDI--S--G-SLIVYAAVDMSSM-TL---V-LN--G-GD---
XP_017243321.1/1-239 SNVL-IL------QE-TSNDA--F--G-SLIVSTAVDMASM-NV---V-MN--G-GE---
XP_017243319.1/1-239 SNVL-IL------QE-TSNDA--F--G-SLIVSTAVDMASM-NV---V-MN--G-GE---
XP_017243320.1/1-239 SNVL-IL------QE-TSNDA--F--G-SLIVSTAVDMASM-NV---V-MN--G-GE---
XP_017255563.1/1-234 SNVL-IL------QE-TSSDA--F--G-SLIVSAAVDMSSM-NV---V-MN--G-GD---
XP_017255562.1/1-234 SNVL-IL------QE-TSSDA--F--G-SLIVSAAVDMSSM-NV---V-MN--G-GD---
XP_012852237.1/1-226 NGML-IL------QE-TCSDE--S--G-SMIVHAGVEAAYM-NA---V-VG--G-GD---
XP_012852238.1/1-226 NGML-IL------QE-TCSDE--S--G-SMIVHAGVEAAYM-NA---V-VG--G-GD---
XP_012852236.1/1-226 NGML-IL------QE-TCSDE--S--G-SMIVHAGVEAAYM-NA---V-VG--G-GD---
XP_012852235.1/1-226 NGML-IL------QE-TCSDE--S--G-SMIVHAGVEAAYM-NA---V-VG--G-GD---
XP_011076208.1/1-227 SSVL-IL------QD-TCTDT--S--G-SLIVHAAVETTSM-NI---V-VN--S-GD---
XP_019200484.1/1-228 NSML-IL------QE-SCNDI--S--G-SLIAYASVGAPEM-TM---V-MN--G-GD---
XP_019200481.1/1-228 NSML-IL------QE-SCNDI--S--G-SLIAYASVGAPEM-TM---V-MN--G-GD---
XP_022879034.1/1-223 ISML-IL------QE-TCMDV--S--G-SLVVHAAVNSEAM-NL---V-MS--G-GD---
XP_018623115.1/1-238 NNML-IL------QD-TCTDA--T--G-SLIVYATIDAADM-DV---V-MN--G-GD---
XP_018623116.1/1-238 NNML-IL------QD-TCTDA--T--G-SLIVYATIDAADM-DV---V-MN--G-GD---
XP_016449095.1/1-238 NNML-IL------QD-TCTDA--T--G-SLIVYATIDAADM-DV---V-MN--G-GD---
XP_009771835.1/1-238 NNML-IL------QD-TCTDA--T--G-SLIVYATIDAVDM-DV---V-MN--G-GD---
XP_009771833.1/1-238 NNML-IL------QD-TCTDA--T--G-SLIVYATIDAVDM-DV---V-MN--G-GD---
XP_019248454.1/1-225 NNML-IL------QD-TCTDA--T--G-SLIVYATIDAADM-DV---V-MN--G-GD---
XP_016564677.1/1-235 SSML-YL------QD-SCTDV--S--G-SIVSYAAVDTAQM-NV---V-MS--G-GD---
XP_016564676.1/1-235 SSML-YL------QD-SCTDV--S--G-SIVSYAAVDTAQM-NV---V-MS--G-GD---
XP_009606534.1/1-237 NSML-IL------QD-SCTDV--S--G-SIVAYAAVDTAEM-NV---V-MS--G-GD---
XP_016457239.1/1-237 NSML-IL------QD-SCTDV--S--G-SIVAYAAVDTAEM-NV---V-MS--G-GD---
XP_009606533.1/1-237 NSML-IL------QD-SCTDV--S--G-SIVAYAAVDTAEM-NV---V-MS--G-GD---
XP_009606532.1/1-237 NSML-IL------QD-SCTDV--S--G-SIVAYAAVDTAEM-NV---V-MS--G-GD---
XP_016457238.1/1-237 NSML-IL------QD-SCTDV--S--G-SIVAYAAVDTAEM-NV---V-MS--G-GD---
XP_016468064.1/1-237 NSML-IL------QD-SCTDV--S--G-SIVAYAAVDTAEM-NV---V-MS--G-GD---
XP_009774404.1/1-237 NSML-IL------QD-SCTDV--S--G-SIVAYAAVDTAEM-NV---V-MS--G-GD---
XP_016468062.1/1-237 NSML-IL------QD-SCTDV--S--G-SIVAYAAVDTAEM-NV---V-MS--G-GD---
XP_016468061.1/1-237 NSML-IL------QD-SCTDV--S--G-SIVAYAAVDTAEM-NV---V-MS--G-GD---
XP_009774402.1/1-237 NSML-IL------QD-SCTDV--S--G-SIVAYAAVDTAEM-NV---V-MS--G-GD---
XP_009774401.1/1-237 NSML-IL------QD-SCTDV--S--G-SIVAYAAVDTAEM-NV---V-MS--G-GD---
XP_019251817.1/1-237 NSML-IL------QD-SCTDV--S--G-SIVAYAAVDTAEM-NV---V-MS--G-GD---
XP_019251815.1/1-237 NSML-IL------QD-SCTDV--S--G-SIVAYAAVDTAEM-NV---V-MS--G-GD---
XP_019251816.1/1-237 NSML-IL------QD-SCTDV--S--G-SIVAYAAVDTAEM-NV---V-MS--G-GD---
XP_011470395.1/1-233 NNML-MF------QE-TMTDA--S--G-SIIVYAPITSTSS-DIMRDVVYE--G-GD---
XP_004309219.2/1-233 NNML-MF------QE-TMTDA--S--G-SIIVYAPITSTSS-DIMRDVVYE--G-GD---
XP_024180881.1/1-233 NNML-MF------QE-TRTDA--S--G-SIVVYAPITSTSS-DIMRDVVYE--G-GD---
XP_024180911.1/1-233 NNML-MF------QE-TRTDA--S--G-SIVVYAPITSTSS-DIMRDVVYE--G-GD---
XP_019450525.1/1-216 SSML-VL------QD-SWTDI--S--S-SVVVYVAIDVDSM-NL---V-VN--G-GE---
XP_019465255.1/1-230 SRML-VL------QD-AWTDS--S--S-SVMVYVAVDMDSL-NL---V-MS--G-GE---
XP_014502466.1/1-227 GNVL-YL------QE-SWTDA--S--G-SMVVYSPINMQSL-NM---MMMN--T-GD---
XP_017438850.1/1-222 GNVL-YL------QE-SWTDA--S--G-SMVVYSPINMQSL-NM---MMMN--S-GD---
XP_006599623.1/1-221 CNVL-YL------QE-SWSDA--S--G-SMVVYSPINMQAL-QM---V-MS--C-GD---
XP_006599622.1/1-221 CNVL-YL------QE-SWSDA--S--G-SMVVYSPINMQAL-QM---V-MS--C-GD---
XP_006599621.1/1-221 CNVL-YL------QE-SWSDA--S--G-SMVVYSPINMQAL-QM---V-MS--C-GD---
XP_004515730.1/1-240 GNVL-YL------QD-SWTDS--S--G-SMIVYSPINMQSL-NM---V-MN--G-GD---
XP_013442370.1/1-239 GSNL-YL------QD-SWTDS--S--G-SMVVYSPIDSQSL-DI---L-IS--G-GD---
XP_004252035.1/1-225 SNIV-IL------QE-NSTDP--T--G-SYVIYAPVEICTM-NT---I-LN--G-GD---
XP_015060726.1/1-225 SHIV-IL------QE-SSTDP--T--G-SYVIYAPVEICTM-NT---I-LN--G-GD---
XP_006589608.1/1-229 NNML-IL------QE-SCIDA--T--G-SFVVYAPIDLASM-NL---V-LG--G-GN---
XP_006589605.1/1-230 NNML-IL------QE-SCIDA--T--G-SFVVYAPIDLASM-NL---V-LG--G-GN---
XP_006849468.1/1-231 NNML-IL------QE-SCTDS--S--G-SLVVYAPIDVTAA-NI---A-MS--G-ED---
XP_020526173.1/1-231 NNML-IL------QE-SCTDS--S--G-SLVVYAPIDVTAA-NI---A-MS--G-ED---
XP_020109515.1/1-228 NSML-IL------QE-SCTDA--S--G-SLVIYAPIDIPAV-NV---V-MS--G-ED---
NP_001310555.1/1-224 NSML-IL------QE-SCTDA--S--G-SLVVYAPIDIPAA-NV---V-MS--G-ED---
XP_010544375.1/1-225 SSML-IL------QE-TCIDA--T--E-ALVVYAPVDVPAM-HV---V-MN--G-GD---
NP_001314001.1/1-230 TNML-IL------RE-TWSDA--C--G-ALIVYAPVDASSI-RV---V-MN--G-GD---
XP_016732454.1/1-230 TNML-IL------QE-TWSDA--C--G-ALIVYAPVDASSI-RV---V-MN--G-GD---
XP_016732453.1/1-230 TNML-IL------QE-TWSDA--C--G-ALIVYAPVDASSI-RV---V-MN--G-GD---
XP_012484067.1/1-230 TNML-IL------QE-TWSDA--C--G-ALIVYAPVDASSI-RV---V-MN--G-GD---
XP_012484066.1/1-230 TNML-IL------QE-TWSDA--C--G-ALIVYAPVDASSI-RV---V-MN--G-GD---
XP_017631791.1/1-230 TNML-IL------QE-TWSDD--C--G-ALIVYAPVDASSI-RV---V-MN--G-GD---
XP_017631789.1/1-230 TNML-IL------QE-TWSDD--C--G-ALIVYAPVDASSI-RV---V-MN--G-GD---
XP_016728129.1/1-230 TNML-IL------QE-TWSVD--C--G-ALIVYAPVDASSI-RV---V-MN--G-GD---
XP_016728128.1/1-230 TNML-IL------QE-TWSVD--C--G-ALIVYAPVDASSI-RV---V-MN--G-GD---
XP_021671684.1/1-240 SSMI-IL------QE-TWNDA--S--S-SLVVYAPIDIASM-TL---V-MN--G-GD---
XP_021671683.1/1-240 SSMI-IL------QE-TWNDA--S--S-SLVVYAPIDIASM-TL---V-MN--G-GD---
XP_021671685.1/1-240 SSMI-IL------QE-TWNDA--S--S-SLVVYAPIDIASM-TL---V-MN--G-GD---
XP_002318231.3/1-240 NNML-IL------QE-TRNDV--S--G-SQVVYAPVDIQSM-SV---V-TS--G-GD---
XP_024438602.1/1-240 NNML-IL------QE-TRNDV--S--G-SQVVYAPVDIQSM-SV---V-TS--G-GD---
XP_024442055.1/1-239 DNML-IL------QE-TWNDV--S--G-SLVVYAPVDVQSV-SV---V-MN--G-GD---
XP_002322460.1/1-239 DNML-IL------QE-TWNDV--S--G-SLVVYAPVDVQSV-SV---V-MN--G-GD---
XP_011048682.1/1-239 DNML-IL------QE-TWNDV--S--G-SLVVYAPVDVQSV-SV---V-MN--G-GD---
XP_011048684.1/1-239 DNML-IL------QE-TWNDV--S--G-SLVVYAPVDVQSV-SV---V-MN--G-GD---
XP_011048683.1/1-239 DNML-IL------QE-TWNDV--S--G-SLVVYAPVDVQSV-SV---V-MN--G-GD---
XP_019081404.1/1-234 NTML-IL------QE-TWADA--S--G-SLIVYAPLDVASM-RA---V-MT--G-GD---
XP_003634081.1/1-234 NTML-IL------QE-TWADA--S--G-SLIVYAPLDVASM-RA---V-MT--G-GD---
XP_008377249.1/1-231 SNML-IL------QE-TWTDP--S--G-SLVVYAPVDTESI-NV---V-MR--G-GD---
XP_017189459.1/1-231 SNML-IL------QE-TWTDP--S--G-SLVVYAPVDTESI-NV---V-MR--G-GD---
XP_008377244.1/1-231 SNML-IL------QE-TWTDP--S--G-SLVVYAPVDTESI-NV---V-MR--G-GD---
XP_009371302.1/1-231 SNML-IL------QE-TWTDP--T--G-SLVVYAPVDTESI-NV---V-MR--G-GD---
XP_009371299.1/1-231 SNML-IL------QE-TWTDP--T--G-SLVVYAPVDTESI-NV---V-MR--G-GD---
XP_008376545.1/1-231 SNVL-IL------QE-TWTDP--T--G-SLVVYAPVDTESI-NV---V-MR--G-GD---
XP_008376544.1/1-231 SNVL-IL------QE-TWTDP--T--G-SLVVYAPVDTESI-NV---V-MR--G-GD---
XP_017188609.1/1-231 SNVL-IL------QE-TWTDP--T--G-SLVVYAPVDTESI-NV---V-MR--G-GD---
XP_022945426.1/1-238 NTML-IL------QE-SWTDI--S--G-SLIVYAPVDAASI-NL---V-MR--G-GD---
XP_022945425.1/1-238 NTML-IL------QE-SWTDI--S--G-SLIVYAPVDAASI-NL---V-MR--G-GD---
XP_023541229.1/1-238 NTML-IL------QE-SWTDI--S--G-SLIVYAPVDAASV-NL---V-MR--G-GD---
XP_023541228.1/1-238 NTML-IL------QE-SWTDI--S--G-SLIVYAPVDAASV-NL---V-MR--G-GD---
XP_022968414.1/1-238 NTML-IL------QE-SWTDI--S--G-SLVVYAPVDTASV-NL---V-MR--G-ED---
XP_022968413.1/1-238 NTML-IL------QE-SWTDI--S--G-SLVVYAPVDTASV-NL---V-MR--G-ED---
XP_023552381.1/1-232 STMF-IL------QE-TWTDI--S--G-SLVVYAPVDTASV-SL---M-MR--G-GD---
XP_022922479.1/1-232 STMF-IL------QE-TWTDI--S--G-SLVVYAPVDTASV-SL---M-MR--G-GD---
XP_023552380.1/1-232 STMF-IL------QE-TWTDI--S--G-SLVVYAPVDTASV-SL---M-MR--G-GD---
XP_022922478.1/1-232 STMF-IL------QE-TWTDI--S--G-SLVVYAPVDTASV-SL---M-MR--G-GD---
XP_022984644.1/1-232 STMF-IL------QE-TWTDI--S--G-SLVVYTPVDTASV-SL---M-MR--G-GD---
XP_022984643.1/1-232 STMF-IL------QE-TWTDI--S--G-SLVVYTPVDTASV-SL---M-MR--G-GD---
XP_008439185.1/1-242 NTMF-IL------QE-TWTDV--S--G-SLVVFAPVDTSSV-NL---V-MR--G-GD---
XP_008439184.1/1-242 NTMF-IL------QE-TWTDV--S--G-SLVVFAPVDTSSV-NL---V-MR--G-GD---
XP_011651140.1/1-242 NTMF-IL------QE-TWTDV--S--G-SLVVFAPVDTSSV-NL---V-MR--G-GD---
XP_004140784.1/1-242 NTMF-IL------QE-TWTDV--S--G-SLVVFAPVDTSSV-NL---V-MR--G-GD---
XP_009391954.1/1-234 TSMM-LL------QE-TYTDA--S--G-AMLVYAPVDIPAM-HL---V-MN--G-GD---
XP_003581542.2/1-227 NGIL-IL------QE-TCTDA--S--G-SMVVYAPVDIPAM-HL---V-MS--G-GD---
XP_006652658.1/1-224 NSML-IL------QE-TCADA--S--G-LMVVYAPVDIPAM-HL---V-MS--G-GD---
XP_015637147.1/1-225 NSML-IL------QE-TCADA--S--G-SMVVYAPVDIPAM-HL---V-MS--G-GD---
XP_025823430.1/1-225 NSIL-IL------QE-TCTDK--S--G-AMVVYAPVDFPAM-QL---V-MG--G-GD---
XP_004978185.1/1-225 NSIL-IL------QE-TCTDK--S--G-AMVVYAPVDFPAM-QL---V-MG--G-GD---
XP_021317935.1/1-228 NSIL-IL------QE-TCTDR--S--G-AMVVYAPVDFPAM-QL---V-IG--G-GD---
NP_001336434.1/1-226 NSIL-IL------QE-TCTDR--S--G-AMVVYTPVDFPAM-QL---V-LG--G-GD---
XP_019709552.1/1-234 TSML-IL------QD-TCTDA--S--V-SLVVYAPVDVSAM-HV---V-MN--G-GD---
XP_015696802.1/1-257 NNML-IL------QE-TCTDS--S--G-SLVVYAPVDVQSM-HV---V-MN--G-GD---
XP_015612621.1/1-258 NNML-IL------QE-TCTDS--S--G-SLVVYAPVDVQSM-HV---V-MN--G-GD---
XP_004957413.1/1-253 NNML-IL------QE-TCTDS--S--G-SLVVYAPVDVQSM-HV---V-MN--G-GD---
XP_025801167.1/1-251 NNML-IL------QE-TCTDS--S--G-SLVVYAPVDVQSM-HV---V-MN--G-GD---
NP_001105125.2/1-256 NNML-IL------QE-TCTDP--S--G-SLVVYAPVDVQSM-HV---V-MN--G-GD---
XP_002462701.1/1-258 NNML-IL------QE-TCTDP--S--G-SLVVYAPVDVQSM-HV---V-MN--G-GD---
XP_020262629.1/1-222 NSML-IL------QE-TCTDS--S--G-SMVVYAPVDVPAM-HL---V-MN--G-GD---
XP_020699782.1/1-235 ESML-IL------QE-TSTDA--A--G-SLVVYAPVDAPAI-NL---V-MN--G-GD---
XP_020699776.1/1-235 ESML-IL------QE-TSTDA--A--G-SLVVYAPVDAPAI-NL---V-MN--G-GD---
XP_020573166.1/1-235 ESML-IL------QE-TSTDA--A--G-SLVVYAPVDAPAI-NL---V-MN--G-GD---
XP_020187432.1/1-228 SSIL-IL------QE-TCTDA--S--G-SMVVYTPVDIPAM-RR---V-MG--GEGD---
XP_025656795.1/1-233 NNTL-FL------QE-TWNDT--S--C-SALLYSPVEVESL-KV---V-MS--G-GD---
XP_025656792.1/1-233 NNTL-FL------QE-TWNDT--S--C-SALLYSPVEVESL-KV---V-MS--G-GD---
XP_025656794.1/1-233 NNTL-FL------QE-TWNDT--S--C-SALLYSPVEVESL-KV---V-MS--G-GD---
XP_020979736.1/1-233 NNTL-FL------QE-TWNDT--S--C-SALLYSPVEVESL-KV---V-MS--G-GD---
XP_020999151.1/1-233 NNTL-FL------QE-TWNDT--S--C-SALLYSPVEVESL-KV---V-MS--G-GD---
XP_015967451.1/1-233 NNTL-FL------QE-TWNDT--S--C-SALLYSPVEVESL-KV---V-MS--G-GD---
XP_020999150.1/1-233 NNTL-FL------QE-TWNDT--S--C-SALLYSPVEVESL-KV---V-MS--G-GD---
XP_025701970.1/1-233 NNTL-FL------QE-TWNDT--S--C-SALLYSPVEVESL-KV---V-MS--G-GD---
XP_025701968.1/1-233 NNTL-FL------QE-TWNDT--S--C-SALLYSPVEVESL-KV---V-MS--G-GD---
XP_025701969.1/1-233 NNTL-FL------QE-TWNDT--S--C-SALLYSPVEVESL-KV---V-MS--G-GD---
XP_020221720.1/1-222 SNVQ-YL------QE-SWTDA--S--G-SMVVYSPINMQSL-NV---V-MS--C-GD---
XP_007152327.1/1-222 SNMF-YL------QE-SWTDA--S--G-SMVVYSPINMQSL-NM---M-MN--C-GD---
XP_006587345.1/1-222 CNVL-YL------QE-SWTDA--S--G-SLVVYSPINMQAL-NM---V-MN--C-GD---
XP_006587344.1/1-222 CNVL-YL------QE-SWTDA--S--G-SLVVYSPINMQAL-NM---V-MN--C-GD---
XP_010035104.1/1-239 TSMV-IL------QE-TWTDA--S--G-SLIAYAPVDITVI-ES---V-MS--G-GD---
XP_021765162.1/1-234 SNML-IL------QE-SWTDC--T--S-SFIVYAPVDSTSM-ET---V-LR--G-GD---
XP_021765160.1/1-234 SNML-IL------QE-SWTDC--T--S-SFIVYAPVDSTSM-ET---V-LR--G-GD---
XP_021730123.1/1-234 SNML-IL------QE-SWTDR--T--S-SFIVYAPVDSTSM-ET---V-LR--G-GD---
XP_021842103.1/1-232 SNML-IL------QE-SWTDR--T--G-SFIVYAPVDSTSM-ET---V-LR--G-GD---
XP_021842106.1/1-232 SNML-IL------QE-SWTDR--T--G-SFIVYAPVDSTSM-ET---V-LR--G-GD---
XP_021842105.1/1-232 SNML-IL------QE-SWTDR--T--G-SFIVYAPVDSTSM-ET---V-LR--G-GD---
XP_021842104.1/1-232 SNML-IL------QE-SWTDR--T--G-SFIVYAPVDSTSM-ET---V-LR--G-GD---
XP_010693471.1/1-235 SNML-IL------QE-SWTDR--T--G-SFIVYAPVDSTSM-ES---V-LR--G-GD---
XP_019107938.1/1-235 SNML-IL------QE-SWTDR--T--G-SFIVYAPVDSTSM-ES---V-LR--G-GD---
XP_010693469.1/1-235 SNML-IL------QE-SWTDR--T--G-SFIVYAPVDSTSM-ES---V-LR--G-GD---
XP_019107937.1/1-235 SNML-IL------QE-SWTDR--T--G-SFIVYAPVDSTSM-ES---V-LR--G-GD---
XP_020163116.1/1-258 NNML-IL------QE-TCTDA--S--G-SLVVYAPVDVQSM-HV---V-MG--G-GD---
XP_020163109.1/1-258 NNML-IL------QE-TCTDA--S--G-SLVVYAPVDVQSM-HV---V-MG--G-GD---
XP_003578475.1/1-267 NNML-IL------QE-TCTDS--S--G-SLVVYAPVDVQSM-HI---V-MN--G-GD---
XP_011012369.1/1-242 NNML-IL------QE-TWNDV--S--G-SLVVYAPVDLQSM-SV---V-TS--G-GD---
XP_011012370.1/1-242 NNML-IL------QE-TWNDV--S--G-SLVVYAPVDLQSM-SV---V-TS--G-GD---
XP_008234278.1/1-230 NTML-ML------QE-SWSDA--S--G-SLVVYAPVDPASM-SA---V-MR--G-GD---
XP_021827117.1/1-230 NTML-ML------QE-SWSDA--S--G-SLVVYAPVDPASM-SA---V-MR--G-GD---
XP_021827116.1/1-230 NTML-ML------QE-SWSDA--S--G-SLVVYAPVDPASM-SA---V-MR--G-GD---
XP_020413581.1/1-230 NTML-ML------QE-SWSDA--S--G-ALVVYAPVDPASM-SA---V-MR--G-GD---
XP_007219555.2/1-230 NTML-ML------QE-SWSDA--S--G-ALVVYAPVDPASM-SA---V-MR--G-GD---
XP_018456926.1/1-230 NSML-IL------QE-TWSDA--S--G-ALVVYAPVDVPSM-NM---V-MS--G-GD---
XP_013613235.1/1-226 SSML-IL------QE-TWSDA--S--G-ALVVYAPVDVPSM-NM---V-MS--G-GD---
XP_013686466.1/1-227 SSML-IL------QE-TWSDA--S--G-ALVVYAPVDVPSM-NM---V-MS--G-GD---
XP_009106897.1/1-227 SSML-IL------QE-TWSDA--S--G-ALVVYAPVDVPST-NM---V-MS--G-GD---
XP_006401857.1/1-229 SSML-IL------QE-TWNDA--S--G-ALVVYAPVDVPSM-NV---V-MS--G-GD---
XP_020871109.1/1-230 SSML-IL------QE-TWNDT--S--G-ALVVYAPVDIPSM-NV---V-MS--G-GD---
NP_001332093.1/1-229 SSML-IL------QE-TWNDV--S--G-ALVVYAPVDIPSM-NT---V-MS--G-GD---
NP_200030.1/1-229    SSML-IL------QE-TWNDV--S--G-ALVVYAPVDIPSM-NT---V-MS--G-GD---
XP_006282099.1/1-225 SSML-IL------QE-TWNDA--S--G-ALVVYAPVDFPSM-NV---V-MS--G-GD---
XP_019095386.1/1-229 SSML-IL------QE-TWNDA--S--G-AMVVYAPVDIPSM-NV---V-MS--G-GD---
XP_019095387.1/1-229 SSML-IL------QE-TWNDA--S--G-AMVVYAPVDIPSM-NV---V-MS--G-GD---
XP_019091174.1/1-228 SSML-IL------QE-TWNDA--S--G-ALVVYAPVDIPSM-NV---V-MS--G-GD---
XP_019091179.1/1-228 SSML-IL------QE-TWNDA--S--G-ALVVYAPVDIPSM-NV---V-MS--G-GD---
XP_019087173.1/1-228 SSML-IL------QE-TWNDA--S--G-ALVVYAPVDIPSM-NV---V-MS--G-GD---
XP_010555654.1/1-234 STML-IL------QE-TWTDA--S--G-SLVMYAPVDVSSM-NV---V-MN--G-GD---
XP_010555656.1/1-234 STML-IL------QE-TWTDA--S--G-SLVMYAPVDVSSM-NV---V-MN--G-GD---
XP_010555655.1/1-234 STML-IL------QE-TWTDA--S--G-SLVMYAPVDVSSM-NV---V-MN--G-GD---
XP_010537199.1/1-242 SSML-IL------QE-TWTDP--S--G-AMVVYAPVDVSSM-NV---V-MN--G-GD---
XP_010537197.1/1-242 SSML-IL------QE-TWTDP--S--G-AMVVYAPVDVSSM-NV---V-MN--G-GD---
XP_010537198.1/1-241 SSML-IL------QE-TWTDP--S--G-AMVVYAPVDVSSM-NV---V-MN--G-GD---
Con_90%              .shh.hL......Q..o.h.........u.llauslD...................D...
Con_80%              .shh.lL......Q..oshst.....t.u.llaAPlDhshh...............D...
Con_70%              .shh.lL......Q-.oshDt.....s.uhllaAPlDhsth.p......ht.....D...
Con_60%              ssMh.lL......QE.SssDs..s..u.ShlVYAPlDhsuh.ph.....hs.....D...
Con_50%              ssML.IL......QE.SssDss.s..u.ShVVYAPVDlsuh.pl....hhs.sG..D...


XP_020582785.1/1-284 --D-AP-LLPSGFRV-IPLDLKA------------DSTPATRTLDLASTLEVSSGGT--S
XP_020682623.1/1-284 --D-AP-LLPSGFRV-IPLDVKA------------DSTAATKTLDLASTLEVGSGGA--S
XP_020682624.1/1-284 --D-AP-LLPSGFRV-IPLDVKA------------DSTAATKTLDLASTLEVGSGGA--S
XP_006846015.1/1-286 --D-AP-LLPSGFRV-IPLDSKS------------DGPGTNRTLDLASSIDVGSTGS---
NP_001142394.1/1-289 --D-AP-LLPSGFRV-IPLDAKTD-----------VPTATTRTLDLASALEVGSGGS--G
NP_001146215.1/1-288 --D-AP-LLPSGFRV-IPLDAKTD-----------VPSA-TRTLDLASALEVGSGGG--L
XP_020405718.1/1-288 --D-AP-LLPSGFRV-IPLDAKTD-----------VPSA-TRTLDLASALEVGSGGG--L
XP_002443548.1/1-287 --D-AP-LLPSGFRV-IPLDAKTD-----------VPSA-TRTLDLASALEVGSGGG--L
XP_025808876.1/1-287 --D-AP-LLPSGFRV-IPLDDKMD-----------MPSA-TRTLDLASALEVGSGAG--S
XP_004963232.1/1-287 --D-AP-LLPSGFRV-IPLDDKMD-----------VPSA-TRTLDLASALEVGSGAG--S
XP_020192001.1/1-285 --D-AP-LLPSGFRV-IPLDAKTD-----------APSA-TRTLDLASTLEVGPGGT--R
XP_003562005.1/1-285 --D-AP-LLPSGFRV-IPLDAKTD-----------APSA-TRTLDLASTLEVGSGGT--R
XP_004982339.1/1-286 --D-AP-LLPSGFRV-IPLDAKTD-----------PPSG-TRTLDLASTLEVGSGGT--T
XP_025796542.1/1-286 --D-AP-LLPSGFRV-IPLDAKTD-----------PPSG-TRTLDLASTLEVGSGGT--T
NP_001347456.1/1-286 --D-AP-LLPSGFRV-IPLDAKTD-----------PPSG-TRTLDLASTLEVGSGGT--T
NP_001352464.1/1-286 --D-AP-LLPSGFRV-IPLDAKTD-----------PPSG-TRTLDLASTLEVGSGGT--T
XP_002464180.1/1-286 --D-AP-LLPSGFRV-IPLDAKTD-----------PPSG-TRTLDLASTLEVGSGGT--T
XP_015689987.1/1-286 --D-AP-LLPSGFRV-IPLDAKAD-----------APSA-TRTLDLASTLEVGSGGT--T
XP_015632484.1/1-286 --D-AP-LLPSGFRV-IPLDGKTD-----------APSA-TRTLDLASTLEVGSGGT--T
XP_015620816.1/1-285 --D-AP-LLPSGFRV-IPLDTKMD-----------GPSA-TRTLDLASALEVGPGGA--S
XP_006664738.1/1-285 --D-AP-LLPSGFRV-IPLDTKMD-----------VPSA-TRTLDLASALEVGPGGA--S
XP_020190954.1/1-286 --D-AP-LLPSGFRV-IPLDTKTD-----------VPSA-TRTLDLASALEVGSGGA--L
XP_020163543.1/1-286 --D-AP-LLPSGFRV-IPLDTKTD-----------VPSA-TRTLDLASALEVGSGGA--L
XP_003577453.1/1-286 --D-AP-LLPSGFRV-IPLDTKTD-----------VPSA-TRTLDLASALEVGSGGA--L
XP_020575104.1/1-285 --D-AF-LLPSGFRV-IPLDAKT------------NPPSTTRTLDLASALEVGGGGT--G
XP_020696902.1/1-286 --D-AF-LLPSGFRV-IPLDVKT------------DPPSATRTLDLASALEVGGGGT--G
XP_020578108.1/1-284 --D-AP-LLPSGFRV-TLLESKT------------EANVAARTLDLASALEVGSGGA--T
XP_020698923.1/1-284 --D-AP-LLPSGFRV-IPLESKT------------EVHAAARTLDLASALEVGSGGA--T
XP_020096610.1/1-279 --D-VP-LLASGFRV-IPLDAKT------------DGPGTTRTLDLASTLEVGSGVG--S
XP_009412508.1/1-287 --D-TP-LLTSGFRI-IPLDSRP------------DGPKATRTLDLASTLEVGSGAA--A
XP_020271156.1/1-284 --D-AP-LLSSGFRV-IPLDQKM------------DSPAATKTLDLASTLEVGRGVS--T
XP_020273236.1/1-287 --D-AP-LLPSGFRI-IPLDPKT------------DSPAATRTLDLASTLEVGPGSA--A
XP_020092733.1/1-287 --D-AP-LLPSGFRV-IPLDAKT------------DAPTATRTLDLASTLEVGSGGA--A
XP_019701934.1/1-288 --D-AP-LLPSGFRV-IPLDPKT------------DSPTVNRTLDLASTLEVGSGAA--A
XP_010905421.1/1-288 --D-AP-LLPSGFRV-IPLDPKT------------DSPTVNRTLDLASTLEVGSGAA--A
XP_010905420.1/1-289 --D-AP-LLPSGFRV-IPLDPKTQ-----------DSPTVNRTLDLASTLEVGSGAA--A
XP_010923678.1/1-288 --D-AP-LLPSGFRV-IPLDPKT------------DAPTANRTLDLASTLEVGSGAA--A
XP_008794452.1/1-288 --D-AP-LLPSGFRV-IPLDPKT------------DAPTANRTLDLASTLEVGSGAA--A
XP_009397391.1/1-288 --D-VP-LLPSGFRV-IPLDPKT------------DSSTATRTLDLASTLEIGSGAT--A
XP_009408551.1/1-288 --D-VP-LLPSGFRV-IPLEPKT------------DSPVASRTLDLASTLEIGSGAA--S
XP_009399045.1/1-288 --D-VP-LLPSGFRV-TPLDPKT------------DSPAATRTLDLASTLEIGSGVT--A
XP_009388964.1/1-288 --D-LP-LLPSGFRV-IPLDPKT------------DSPATTRTLDLASMLEIGSGAT--A
XP_009397717.1/1-288 --N-LP-LLPSGFRV-IPLDPKT------------DSPATTRTLDLASTLEVGSGAT--S
XP_018488876.1/1-280 --D-AP-LLPSGFRV-IPLDQKTS------P---SDHISVNRTRDLASSLDGSNK-----
XP_013587486.1/1-280 --D-AP-LLPSGFRV-IPLDHKTS------P---NDHLSVNRTRDLASSLDGSTK-----
XP_009115139.1/1-280 --D-AP-LLPSGFRV-IPLDHKTS------P---SDHLSVNRTRDLASSLDGSTK-----
XP_013662459.1/1-280 --D-AP-LLPSGFRV-IPLDHKTS------P---SDHLSVNRTRDLASSLDGSTK-----
XP_013707293.1/1-280 --D-AP-LLPSGFRV-IPLDHKTS------P---NDHLSVNRTRDLASSLDGSTK-----
XP_006415465.2/1-280 --D-AP-LLPSGFRV-IPLEQKTT------P---SDHVSANRTRDLASSLDGSTK-----
XP_019099288.1/1-280 --D-AP-LLPSGFRV-IPLEQKVN------P---NDHQSTNRTRDLASSLDGSTK-----
NP_174337.1/1-280    --D-AP-LLPSGFRV-IPLDQKTN------P---NDHQSASRTRDLASSLDGSTK-----
XP_020867051.1/1-280 --D-AP-LLPSGFRV-IPLDQKTN------P---NDHQSASRTRDLASSLDGSTK-----
XP_020867052.1/1-280 --D-AP-LLPSGFRV-IPLDQKTN------P---NDHQSASRTRDLASSLDGSTK-----
XP_010460847.1/1-280 --D-AP-LLPSGFRV-IPLEQKVN------P---NDHQSASRTRDLASSLDGSTK-----
XP_019094482.1/1-280 --D-AP-LLPSGFRV-IPLEQKIN------P---NDHQSASRTRDLASSLDGSTK-----
XP_006306764.1/1-280 --D-AP-LLPSGFRV-IPLEQKTN------P---NDHQSASRTRDLASSLDGSTK-----
XP_010538268.1/1-285 --D-AA-LLPSGFCI-IPLEHKTT------P---DGP-LATRTLDLASALEGSTR-----
XP_010519070.1/1-284 --D-AP-LLPSGFRV-IPLEHKTI------P---NGS-SASRTLDLASALEGPSR-----
XP_018436356.1/1-280 --D-AP-LLPSGFRV-IPLEYKST------V---NGA-SANRTLDLASALEGSTR-----
XP_013637594.1/1-286 --D-AP-LLPSGFRV-IPLEHKST------P---NGA-SGNRTLDLASALEGSTR-----
XP_013692700.1/1-286 --D-AP-LLPSGFRV-IPLEHKST------P---NGA-SGNRTLDLASALEGSTR-----
XP_009143747.1/1-286 --D-AP-LLPSGFRV-IPLEHKST------P---NGA-SANRTLDLASALEGSTR-----
XP_018484893.1/1-285 --D-AP-LLPSGFRV-IPLEQKST------P---NGA-SVNRTLDLASALEGSTR-----
XP_013637264.1/1-280 --D-AP-LLPSGFRV-IPLEPKST------P---NGA-SVNRTLDLASALEGSTR-----
XP_013637263.1/1-280 --D-AP-LLPSGFRV-IPLEPKST------P---NGA-SVNRTLDLASALEGSTR-----
XP_022557131.1/1-280 --D-AP-LLPSGFRV-IPLEPKST------P---NGA-SVNRTLDLASALEGSTR-----
XP_013729051.1/1-280 --D-AP-LLPSGFRV-IPLEPKST------P---NGA-SVNRTLDLASALEGSTR-----
XP_013744548.1/1-280 --D-AP-LLPSGFRV-IPLEPKST------S---NNA-SVNRTLDLASALEGSTR-----
XP_009141396.1/1-280 --D-AP-LLPSGFRV-IPLEPKST------S---NNA-SVNRTLDLASALEGSTR-----
XP_006410621.1/1-284 --D-AP-LLPSGFRI-IPLEHKST------P---NSA-SANRTLDLASSLEGSTR-----
NP_181018.1/1-284    --D-AP-LLPSGFRI-IPLEQKST------P---NGA-SANRTLDLASALEGSTR-----
XP_020884308.1/1-284 --D-AP-LLPSGFRI-IPLEQKST------P---NGA-STNRTLDLASALEGSTR-----
XP_006293359.1/1-284 --D-AP-LLPSGFRV-IPLEHKST------P---TGP-SANRTLDLASALEGSTR-----
XP_010516626.1/1-284 --D-AP-LLPSGFRV-IPLEHKST------P---NGA-SANRTLDLASALEGSTR-----
XP_010509720.1/1-284 --D-AP-LLPSGFRV-IPLEHKST------P---NGA-TANRTLDLASALEGSTR-----
XP_010504942.1/1-284 --D-AP-LLPSGFRV-IPLEHKST------P---NGA-SANRTLDLASALEGSTR-----
XP_017223336.1/1-283 --D-AP-LLPSGFRV-IPLDPESD------------KSSANRTLDLASALEVRHGGA---
XP_016559371.1/1-285 --D-AP-LLPSGFSV-IPLEPKSD------------SPIAARTLDLASTLEARTGGT---
XP_016559374.1/1-285 --D-AP-LLPSGFSV-IPLEPKSD------------SPIAARTLDLASTLEARTGGT---
XP_012830207.1/1-281 --D-AP-MLPSGFRI-ISLDPKSN------------GPGPTQTLDLASTLDVG-------
XP_012827547.1/1-287 --D-AP-LLPSGFRV-IPLEPKSD-----------GGPTANRTLDLASALEVGHGGA---
XP_022866806.1/1-284 --D-AP-LLPSGFRL-IPLDSKT------------DGQGATRTLDLASALEVGPGGA---
XP_022866805.1/1-285 --D-AP-LLPSGFRL-IPLDSKTQ-----------DGQGATRTLDLASALEVGPGGA---
XP_023001017.1/1-285 --D-AP-LLPSGFRV-IPLESKA------------DASAATRTLDLASTLEVRPGSA--R
XP_023001016.1/1-285 --D-AP-LLPSGFRV-IPLESKA------------DASAATRTLDLASTLEVRPGSA--R
XP_023519837.1/1-285 --D-AP-LLPSGFRV-IPLESKT------------DASAATRTLDLASTLEVRPGSA--R
XP_023519836.1/1-285 --D-AP-LLPSGFRV-IPLESKT------------DASAATRTLDLASTLEVRPGSA--R
XP_022927139.1/1-285 --D-AP-LLPSGFRV-IPLESKT------------DASAATRTLDLASTLEVRPGSA--R
XP_022927138.1/1-285 --D-AP-LLPSGFRV-IPLESKT------------DASAATRTLDLASTLEVRPGSA--R
XP_022139982.1/1-285 --D-AP-LLPSGFRV-IPLESKT------------DAPAATRTLDLASTLEVRPGTA--R
XP_004135550.1/1-285 --D-AP-LLPSGFRV-IPLESKA------------EMPGATRTLDLASTLEVRPGTN--R
XP_008445207.1/1-285 --D-AP-LLPSGFRV-IPLESKT------------EMPGATRTLDLASTLEVRPGTN--R
XP_017230337.1/1-286 --D-AP-LLPSGFRV-IPLDPKS------------DKPGASRTLDLASTLEVGPGGA--R
XP_017230338.1/1-286 --D-AP-LLPSGFRV-IPLDPKS------------DKPGASRTLDLASTLEVGPGGA--R
XP_010316959.1/1-285 --D-AP-LLPSGFSV-IPLEPKS------------DSPSAARTLDLASTLEARTGGT--R
XP_004233170.1/1-285 --D-AP-LLPSGFSV-IPLEPKS------------DSPSAARTLDLASTLEARTGGT--R
XP_015066839.1/1-285 --D-AP-LLPSGFSV-IPLEPKS------------DSPSAARTLDLASTLEARTGGT--R
XP_015066842.1/1-285 --D-AP-LLPSGFSV-IPLEPKS------------DSPSAARTLDLASTLEARTGGT--R
XP_015066843.1/1-285 --D-AP-LLPSGFSV-IPLEPKS------------DSPSAARTLDLASTLEARTGGT--R
XP_006353018.1/1-285 --D-AP-LLPSGFSV-IPLEPKS------------DSPSAARTLDLASTLEARTGGT--R
XP_006353020.1/1-285 --D-AP-LLPSGFSV-IPLEPKS------------DSPSAARTLDLASTLEARTGGT--R
XP_019178964.1/1-287 --D-AP-LLPSGFRV-IPVEPKS------------DAPGTTRTLDLASTLEAGNGGS--T
XP_019178963.1/1-287 --D-AP-LLPSGFRV-IPVEPKS------------DAPGTTRTLDLASTLEAGNGGS--T
XP_019228489.1/1-285 --D-AP-LLPSGFRV-IPLEPKS------------DGPAATRTLDLASTLEAGTGG----
XP_019228488.1/1-285 --D-AP-LLPSGFRV-IPLEPKS------------DGPAATRTLDLASTLEAGTGG----
NP_001289498.1/1-285 --D-AP-LLPSGFRV-IPLEPKS------------DGPAATRTLDLASTLEAGTGG----
XP_009777911.1/1-285 --D-AP-LLPSGFRV-IPLEPKS------------DGPAATRTLDLASTLEAGTGG----
XP_009612342.1/1-285 --D-AP-LLPSGFRV-IPLEPKS------------DGPAATRTLDLASTLEAGTGG----
XP_009612341.1/1-285 --D-AP-LLPSGFRV-IPLEPKS------------DGPAATRTLDLASTLEAGTGG----
XP_006363649.1/1-287 --D-AP-LIPSGFRV-IPLEPKS------------DVPAATRTLDLASTLEAGTGGS--G
XP_015066689.1/1-287 --D-AP-LLPSGFRV-IPLEPKS------------DVPAATRTLDLASTLEAGTGGS--G
XP_010315636.1/1-287 --D-AP-LLPSGFRV-IPLEPKS------------DVPAATRTLDLASTLEAGTGGS--G
XP_022879368.1/1-285 --D-AP-LLPSGFRV-IPLDAKT------------DGDGATRTLDLASTLEVGPSGA--R
XP_020554026.1/1-285 --D-AP-VLPSGFRV-IPLEPKS------------DGPTATRTLDLASALEVGHSGA--R
XP_011096678.1/1-285 --D-AP-VLPSGFRV-IPLEPKS------------DGPTATRTLDLASALEVGHSGA--R
XP_011085207.1/1-285 --D-AH-MLPSGFRV-IPLDPKS------------DEPAATRTLDLASALEVRHSGG--R
XP_023003193.1/1-285 --D-AP-LLPSGFRV-IPLDPKT------------DEPTANRTLDLASTLEVGANGA--R
XP_022926723.1/1-285 --D-VP-LLPSGFRV-IPLDPKT------------DEPTANRTLDLASTLEVGANGA--R
XP_023518237.1/1-285 --D-AP-LLPSGFRV-IPLDPKT------------DEPTANRTLDLASTLEVGANGA--R
XP_022146537.1/1-286 --D-AP-LLPSGFRV-IPLDPKT------------DEPTAARTLDLASTLEVGANAS--R
XP_011658319.1/1-286 --D-AP-LLPSGFRV-IPLDPKT------------DEPTAARTLDLASTLEVGANAA--R
XP_008439554.1/1-286 --D-AP-LLPSGFRV-IPLDPKT------------DEPTAARTLDLASTLEVGANAA--R
XP_022978372.1/1-286 --D-AP-LLPSGFRV-IPLDPKT------------DDPTAARTLDLASTLEVGANAA--R
XP_023543833.1/1-286 --D-AP-LLPSGFRV-IPLDPKT------------DDPTAARTLDLASTLEVGANAA--R
XP_022926042.1/1-286 --D-AP-LLPSGFRV-IPLDPKT------------DDPTAARTLDLASTLEVGANAA--R
XP_010247499.1/1-286 --D-AP-LLPSGFRV-IPLDPKT------------DGPATTRTLDLASALEVGAGGT--R
XP_016717556.1/1-285 --N-AP-LLPSGFRV-IPLDSKP------------DGPGAARTLDLASTLEVGPGSN--R
XP_012455108.1/1-285 --N-AP-LLPSGFRV-IPLDSKP------------DGPGAARTLDLASTLEVGPGSN--R
XP_016717554.1/1-287 --N-AP-LLPSGFRV-IPLDSKPVM----------DGPGAARTLDLASTLEVGPGSN--R
XP_012455107.1/1-287 --N-AP-LLPSGFRV-IPLDSKPVM----------DGPGAARTLDLASTLEVGPGSN--R
XP_016711412.1/1-287 --N-AP-LLPSGFRV-IPLDSKPVM----------DGPGAARTLDLASTLEVGPGSN--R
XP_017649973.1/1-287 --N-AP-LLPSGFRV-IPLDSKPVM----------DGPGAARTLDLASTLEVGPGSN--R
XP_022716449.1/1-285 --D-AP-LLPSGFRV-IPLYPKTVT----------DGPGATRTLDLASTLEVGPGSN--R
XP_022724664.1/1-287 --D-AP-LLPSGFRV-IPLDPKTVM----------DGPGATRTLDLASTLEVGPASN--R
XP_021298192.1/1-285 --D-AP-LLPSGFRV-IPLDPKT------------DGAGATRTLDLASALEVGTGSN--R
XP_021298193.1/1-294 --D-AP-LLPSGFRV-IPLDPKTVTLTCQSS---ADGAGATRTLDLASALEVGTGSN--R
XP_021298191.1/1-294 --D-AP-LLPSGFRV-IPLDPKTVTLTCQSS---ADGAGATRTLDLASALEVGTGSN--R
XP_007022874.2/1-285 --D-AP-LLPSGFRV-IPLDPKT------------DGAGATRTLDLASALEVGPGSN--R
XP_017979205.1/1-294 --D-AP-LLPSGFRV-IPLDPKTVTLTCQSS---ADGAGATRTLDLASALEVGPGSN--R
XP_017979204.1/1-294 --D-AP-LLPSGFRV-IPLDPKTVTLTCQSS---ADGAGATRTLDLASALEVGPGSN--R
XP_017236680.1/1-286 --D-AP-LLPSGFRV-IPLDPKS------------DKPSANRTLDLASTLEGGPGGA--R
XP_010051959.1/1-285 --D-AP-LLPSGFRV-IQLDPKT------------DGPAPTRTLDLASTLEVGSGGA--R
XP_010051960.1/1-285 --D-AP-LLPSGFRV-IQLDPKT------------DGPAPTRTLDLASTLEVGSGGA--R
XP_015882975.1/1-284 --E-AP-LLPSGFRV-IPLDPKS------------DGPAATRTLDLASTLEVGPGST--R
XP_015882797.1/1-284 --E-AP-LLPSGFRV-IPLDPKS------------DGPAATRTLDLASTLEVGPGST--R
XP_010098570.1/1-284 --E-AP-LLPSGFRV-IPLDPKA------------DTPAATRTLDLASTLEVGSGGA--R
XP_011012084.1/1-284 --D-AP-LLPSGFRV-IPLDPKT------------DAPAATRTLDLASTLEVGPGGA--R
XP_011012083.1/1-284 --D-AP-LLPSGFRV-IPLDPKT------------DAPAATRTLDLASTLEVGPGGA--R
XP_006377667.2/1-284 --D-AP-LLPSGFRV-IPLDPKT------------DAPAATRTLDLASTLEVGPGGA--R
XP_002281868.2/1-285 --D-AP-LLPSGFRV-IPLDPKT------------DGPAATRTLDLASTLEVGAGGA--R
XP_018847863.1/1-285 --D-AP-LLPSGFRV-IPLDPKT------------DGPAATRTLDLASTLEVGAGGA--R
XP_018847862.1/1-285 --D-AP-LLPSGFRV-IPLDPKT------------DGPAATRTLDLASTLEVGAGGA--R
XP_023919942.1/1-285 --D-AP-LLPSGFRV-IPLDPKP------------DGPAATRTLDLASTLEVGAGGA--R
XP_023919941.1/1-285 --D-AP-LLPSGFRV-IPLDPKP------------DGPAATRTLDLASTLEVGAGGA--R
XP_018828101.1/1-285 --D-AP-LLPSGFRV-IPLDPKT------------DGPAATRTLDLASTLEVRAGGA--R
XP_018828100.1/1-285 --D-AP-LLPSGFRV-IPLDPKT------------DGPAATRTLDLASTLEVRAGGA--R
XP_002298892.1/1-285 --D-AP-LLSSGFRV-IPLDPKT------------DAPATTRTLDLASTLEVGPGGT--R
XP_011038887.1/1-285 --D-AP-LLSSGFRV-IPLDPKT------------DAPATTRTLDLASTLEVGPGGT--R
XP_011038886.1/1-285 --D-AP-LLSSGFRV-IPLDPKT------------DAPATTRTLDLASTLEVGPGGT--R
XP_021657817.1/1-285 --D-AP-LLPSGFRV-IPLDPKA------------DGPAPTRTLDLASTLEVGAGCA--R
XP_021657818.1/1-285 --D-AP-LLPSGFRV-IPLDPKA------------DGPAPTRTLDLASTLEVGAGCA--R
XP_015577248.1/1-260 --D-AP-LLPSGFRV-IPLDPKT------------DGPAPTRTLDLASTLEVGAGGA--R
XP_021616343.1/1-285 --D-AP-LLPSGFRV-IPLDPKA------------DGPAATRTLDLASTLEVGAGGA--R
XP_021645103.1/1-285 --D-AP-LLPSGFRV-IPLDPKT------------DGPAATRTLDLASTLEVGAGGA--R
XP_021592762.1/1-285 --D-AP-LLPSGFRV-IPLDVKT------------DGPAATRTLDLASTLEVGGGGA--R
XP_012075627.1/1-285 --D-AL-LLPSGFRV-IPLDPKT------------DGPAATRTLDLASTLEVGPGGA--R
XP_008372224.1/1-285 --D-AP-LLPSGFHV-IPLDPKA------------DGPTASRTLDLASTLEIGTSGA--R
XP_009362238.1/1-285 --D-AP-LLPSGFHV-IPLDPKA------------DGPTASRTLDLASTLEIGTSGA--R
XP_009344405.1/1-284 --D-AP-LLPSGFHV-IPLDPKA------------DGPTATRTLDLASTLEVGTSGA--R
XP_008349150.1/1-284 --D-AP-LLPSGFHV-IPLDPKA------------DGPTATRTLDLASTLEVGTSGA--R
XP_007214622.1/1-285 --D-AP-LLPSGFHV-IPLDPKT------------DGPTANRTLDLASTLEVGPSGS--R
XP_021812393.1/1-285 --D-AP-LLPSGFHV-IPLDPKT------------DGPTANRTLDLASTLEVGPSGS--R
XP_024161164.1/1-285 --D-AP-LLPSGFHV-IPLDAKT------------DGPTANRTLDLASTLEVGASSA--R
XP_004293989.1/1-285 --D-AP-LLPSGFHV-IPLDAKT------------DGPATNRTLDLASTLEVGASSA--R
XP_019419795.1/1-282 --D-AP-LLPSGFHV-IPLAPK------------TDAPANTRTLDLASTLEVGSGSI--R
XP_019426301.1/1-282 --D-AP-LLASGFRV-IPLAPK------------PDDPATSRTLDLASTLEVGSSIT--R
XP_003524993.1/1-285 --D-AL-LLPSGFRI-IPLDPK------------TDGLASTRTLDLASTLETGSGNA--R
XP_003532788.1/1-285 --D-AL-LLPSGFRI-IPLDPK------------TDGPASTRTLDLASTLETGSGNA--R
XP_020215009.1/1-285 --D-AL-LLPSGFRI-IPLDPK------------TDGLASTRTLDLASTMEIGSSNV--R
XP_017409410.1/1-285 --D-AL-LLPSGFRI-IPLDPK------------TDGPASTRTLDLASALDIGSGNA--R
XP_017409418.1/1-285 --D-AL-LLPSGFRI-IPLDPK------------TDGPASTRTLDLASALDIGSGNA--R
XP_014509941.1/1-285 --D-AL-LLPSGFRI-IPLDPK------------TDGPASTRTLDLASALDIGSGNA--R
XP_007159548.1/1-285 --D-AL-LLPSGFCI-IPLDPK------------TDGPASTRTLDLASALDIGSGNA--R
XP_024047818.1/1-288 --D-AP-LLASGFRV-IPLDSKAAM-----Q---QDGPAASRTLDLASALEVGSGGA--R
XP_006448915.2/1-288 --D-AP-LLASGFRV-IPLDSKAAM-----Q---QDGPAASRTLDLASALEVGSGGA--R
XP_006468302.1/1-288 --D-AP-LLASGFRV-IPLDSKAAM-----Q---QDGPAASRTLDLASALEVGSGGT--R
XP_006468303.1/1-287 --D-AP-LLASGFRV-IPLDSKAAM---------QDGPAASRTLDLASALEVGSGGT--R
XP_024047817.1/1-287 --D-AP-LLASGFRV-IPLDSKAAM---------QDGPAASRTLDLASALEVGSGGA--R
XP_004504217.1/1-285 --D-AL-LLPSGFRI-IPLDPK------------ADAPAATRTLDLASALEIGSGNS--H
XP_003594520.2/1-281 --D-AL-LLPSGFRV-IPLDPK------------SDGPTTSRTLDLLE----GSRNA--R
XP_024632601.1/1-281 --D-AL-LLPSGFRV-IPLDPK------------SDGPTTSRTLDLLE----GSRNA--R
XP_004486450.1/1-285 --D-AL-LLPSGFRV-IPLDPK------------SDGPAATRTLDLASTLEVGSGNA--R
XP_007147487.1/1-285 --D-AL-LLPSGFRV-IPLDPK------------SDGPAATRTLDLASTMEVGSANA--R
XP_017437412.1/1-285 --D-AL-LLPSGFRV-IPLDPK------------SDGPAATRTLDLASTMEVGSANA--R
XP_014518680.1/1-285 --D-AL-LLPSGFRV-IPLDPK------------SDGPAATRTLDLASTMEVGSANA--R
XP_003546255.1/1-285 --D-AL-LLPSGFRV-IPLDPK------------SDGPAPTRTLDLASTMEVGSGNA--R
XP_020233213.1/1-285 --D-AL-LLPSGFRV-IPLDPK------------SDGPAATRTLDLASTMEVGSGNA--R
XP_003535078.1/1-285 --D-AL-LLPSGFRV-IPLDPK------------SDGPAATRTLDLASTMEVGSGNA--R
XP_014617344.1/1-285 --D-AL-LLPSGFRV-IPLDPK------------SDGPAATRTLDLASTMEVGSGNA--R
XP_015959291.1/1-283 --D-AL-LLPSGFRV-IPLDPK------------TDTPTTTRTLDLASTLEVGSGNT--R
XP_016197724.1/1-283 --D-AL-LLPSGFRV-IPLDPK------------TDTPTTTRTLDLASTLEVGSGNT--R
XP_025694278.1/1-283 --D-AL-LLPSGFCV-IPLDPK------------TDTPTTTRTLDLASTLEVGSGNT--R
XP_006846777.1/1-281 --D-AP-LLPSGFRV-IPLDSKTNP-------QDN-TAAAIRTLDLTSSLDIGSATN--R
XP_003574071.1/1-280 --D-AP-LISSGFRV-IPLDMKT-----------D-GTPTGRTLDLASSLEVGSTTL--Q
XP_020174778.1/1-278 --D-AP-LISSGFRV-IPLDMKT-----------D-GAPAGRTLDLASSLEAGSTTL--Q
XP_006661867.1/1-281 --D-AP-LISSGFRV-IPLDMKT-----------D-GAPTGRTLDLASSLEVGSTTQ---
XP_015614901.1/1-281 --D-AP-LISSGFRV-IPLDMKT-----------D-GTPAGRTLDLASSLEVGSTAQ---
XP_004982838.1/1-282 --D-AP-LISSGFRV-IPLDIKS-----------D-GVPSGRTLDLASSLEVGTTTQ--H
XP_025796396.1/1-282 --D-AP-LISSGFRV-IPLDIKT-----------D-GVPSGRTLDLASSLEVGTTAQ--H
NP_001316502.1/1-284 --D-AP-LVSSGFRV-IPLDIKT-----------D-GPPSGRTLDLASSLEVGATTQ--Q
XP_021306737.1/1-282 --D-AP-LISSGFRV-IPLDIKT-----------D-GLSSGRTLDLASSLEVGATTQ--Q
XP_021306733.1/1-293 --D-AP-LISSGFRV-IPLDIKTVECFLLVYLFED-GLSSGRTLDLASSLEVGATTQ--Q
XP_020162053.1/1-281 --D-AP-LVSSGFRV-IPLDMKT-----------D-VVSSGRTLDLASSLEVGSTAA--Q
XP_006649248.1/1-282 --E-TP-LISSGFRV-IPLDMKT-----------D-GASSGRTLDLASSLEVGSTTT--Q
XP_015632709.1/1-282 --E-TP-LISSGFRV-IPLDMKT-----------D-GASSGRTLDLASSLEVGSATA--Q
NP_001296150.1/1-282 --D-AP-LISSGFRV-IPLDVKT-----------D-GVSSGRTLDLASSLDVGSAAP--Q
XP_008665074.1/1-283 --D-AP-LISSGFRV-IPLDMKT-----------D-GVSSGRTLDLASSLDVGSAAP--Q
NP_001105994.1/1-283 --D-AP-LISSGFRV-IPLDMKT-----------D-GVSSGRTLDLASSLDVGSAAP--Q
XP_008660404.1/1-283 --D-AP-LISSGFRV-IPLDMKT-----------D-GVSSGRTLDLASSLDVGSAAP--Q
XP_002468669.1/1-283 --D-AP-LISSGFRV-IPLDMKT-----------D-GVASGRTLDLASSLDVGSAAP--Q
XP_021309716.1/1-285 --D-AP-LISSGFRV-IPLDMKT-----------D-GVASGRTLDLASSLDVGSAAP--Q
XP_004986037.1/1-283 --D-AP-LISSGFRV-IPLDMKT-----------D-GVPSGRTLDLASSLDVGSATP--Q
XP_025795001.1/1-283 --D-AP-LISSGFRV-IPLDMKT-----------D-GVSSGRTLDLASSLDVGSAAP--Q
XP_020093893.1/1-283 --D-AP-LISSGFRV-IPLDTKA-----------D-AITSTRTLDLASSLEVGNATQ--R
XP_020093894.1/1-282 --D-AP-LISSGFRV-IPLDTK------------D-AITSTRTLDLASSLEVGNATQ--R
XP_020259754.1/1-283 --D-AP-LLPSGFRV-IPLDCRP-----------D-GLNSNRTLDLASSLEVGSAVN--R
XP_009401955.1/1-282 --D-AP-LLPSGFRV-IPLDAKT-----------N-GLSSTRTLDLASSLGVGSTVN--R
XP_008791541.1/1-283 --D-AP-LLPSGFRV-IPLDTKS-----------E-GLSSSRTLDLASSLEVGSATN--R
XP_017698637.1/1-283 --D-AP-LLPSGFRV-IPLDTKS-----------E-GLSSSRTLDLASSLEVGSATN--R
XP_010920422.1/1-283 --D-AP-LLPSGFRV-IPLDMKS-----------E-GLASSRTLDLASSLEVGSATN--R
XP_010920423.1/1-283 --D-AP-LLPSGFRV-IPLDMKS-----------E-GLASSRTLDLASSLEVGSATN--R
XP_010920419.1/1-283 --D-AP-LLPSGFRV-IPLDMKS-----------E-GLASSRTLDLASSLEVGSATN--R
XP_010920426.1/1-283 --D-AP-LLPSGFRV-IPLDMKS-----------E-GLASSRTLDLASSLEVGSATN--R
XP_019709326.1/1-287 --D-AP-LLPSGFRV-IPLDTKK-----------E-GLASSRTLDLASSLEVGSATN--H
XP_008785281.1/1-283 --D-AA-LLPSGFRV-IPLDANK-----------D-GFASCRTLDLASSLEVGSATN--R
XP_008462593.1/1-288 --D-AV-LLASGFRI-IPLHSK------TSDPKDTWN--SNRTLDLTSSLEVGSSAT--H
XP_022969629.1/1-286 --D-AV-LLPSGFRI-IPLHSE------TGDSKHTWK-NLNSTMDLTSNLEVGSSAS--Q
XP_023536970.1/1-286 --D-AV-LLPSGFRI-IPLHSE------TGDSKHTWK-NSNRTMDLTSNLEVGSSAS--Q
XP_022937855.1/1-288 --D-AV-LLPSGFRI-IPLHSE------TGDSKHTWK-NSNRTMDLTSNLEVGSSAS--Q
XP_022937858.1/1-288 --D-AV-LLPSGFRI-IPLHSE------TGDSKHTWK-NSNRTMDLTSNLEVGSSAS--Q
XP_012827869.1/1-285 --D-AQ-LLPSGFRI-IPLDSK------PVDLQDSLT-SHHKTLDLTSSLEVGPTVG--N
XP_022874815.1/1-279 --D-AP-LLPSGFRV-NPLDPK------S----DPLT--SFKTLDLTSSLEVRPAAN--H
XP_022874814.1/1-283 --D-AP-LLPSGFRV-NPLDPK------SGDLQDPLT--SFKTLDLTSSLEVRPAAN--H
XP_018434000.1/1-280 --D-AP-LVPSGFRV-IPLDSK------TGDAQDLLT-ANHRTLDSTSSQYVGSTTS--E
XP_013642496.1/1-279 --D-AP-LVPSGFRV-IPVDSK------TGDAQDLLT-ANHRTLDLTSSQDVGST-P--E
XP_009150087.1/1-279 --D-AP-LVPSGFRV-IPVDSK------TGDAQDLLT-ANHRTLDLTSSQDVGST-P--E
XP_009150088.1/1-279 --D-AP-LVPSGFRV-IPVDSK------TGDAQDLLT-ANHRTLDLTSSQDVGST-P--E
XP_022563181.1/1-279 --D-AP-LVPSGFRV-IPVDAK------TGDAQDLLT-ANHQTLDLTSSQDVGST-P--E
XP_013675790.1/1-280 --D-AP-LVPSGFRV-IPVDAKT-----VGDAQDLLT-ANHQTLDLTSSQDVGST-P--E
XP_013623090.1/1-279 --D-AP-LVPSGFRV-IPVDAK------TGDAQDLLT-ANHQTLDLTSSQDVGST-P--E
XP_013623089.1/1-280 --D-AP-LVPSGFRV-IPVDAKT-----VGDAQDLLT-ANHQTLDLTSSQDVGST-P--E
XP_020885782.1/1-285 --D-AP-LVPSGFRV-IPVDAK------TGDAQDLLT-ANHRTLDLTSSLEVGPSPE--N
XP_006280004.1/1-285 --D-AP-LVPSGFRV-IPVDAK------TGDAQDLLT-ANHRTLDLTSSLEVGPSPE--N
XP_019088563.1/1-285 --D-AP-LVPSGFRV-IPVDAK------TGDAQDLLT-ANHRTLDLTSSLEVGPSPE--N
XP_010483719.1/1-285 --D-AP-LVPSGFRV-IPVDAK------TGDAQDLLT-ANHRTLDLTSSLEVGPSPE--N
XP_010456679.1/1-285 --D-AP-LVPSGFRV-IPVDAK------TGDAQDLLT-ANHRTLDLTSSLEVGPSPE--N
XP_006400821.1/1-284 --D-AP-LVPSGFRV-IPVDAK------TGDSQDLLT-ANHRTLDLTSSLEVGPSPE--N
NP_200877.1/1-284    --D-AP-LVPSGFRV-IPVDAK------TGDVQDLLT-ANHRTLDLTSSLEVGPSPE--N
XP_013611543.1/1-282 --D-AP-LVPSGFRV-IPVDAK------TGDAQDLLT-ANHRTLDLTSSLEVGPTPE--N
XP_009120605.1/1-284 --D-AP-LVPSGFRV-IPVDAK------TGDAQDLLT-ANHRTLDLTSSLEVGPTPE--N
XP_013667152.1/1-284 --D-AP-LVPSGFRV-IPVDAK------TGDAQDLLT-ANHRTLDLTSSLEVGPTPE--N
XP_018446535.1/1-284 --D-AP-LVPSGFRV-IPVDAK------TGDAQDLLT-ANHRTLDLTSSLEVGPSPE--N
XP_018454374.1/1-284 --D-AP-LVPSGFRV-IPIDAK------TGDGQDLIT-SNHRTLDLTSSLEVGPSPE--N
XP_009126588.1/1-285 --D-AP-LVPSGFRV-IPIDAK------TGDAQDLIT-SNHRTLDLTSSLEVGPSPE--T
XP_013680521.1/1-285 --D-AP-LVPSGFRV-IPIDAK------TGDGQDLIT-SNHRTLDLTSSLEVGPSPE--T
XP_013612427.1/1-285 --D-AP-LVPSGFRV-IPIDAK------TGDGQDLIT-SNHRTLDLTSSLEVGPSPE--T
XP_010535608.1/1-283 --D-AP-LVPSGFRV-IPVDAK------TGDGQDLLA-A-NRTLDLTSSLEVGPASE--N
XP_010520372.1/1-276 --D-AP-LVPSGFRV-IPIDAK------TGGGSDLPT-A-NRTLDLTSSLEVGPASE--N
XP_004487103.1/1-286 --D-VP-LVPSGLRI-VLLNSQ------TSDKKNAV--TTKRTLDLTSGLEVGPTTT--H
XP_003597252.1/1-283 --D-AP-LVPSGFRI-VLLNSQ------PGDTKNTT--TANRTLDLTSGLEVSPATA--H
XP_024455683.1/1-286 --D-AP-LLPSGFRI-ISLESK------AKDTQEVL--TTNCTLDLTSSLEAGLAIN--H
XP_022026352.1/1-288 --D-AP-LVPSGFRI-IPLDPK------SGDGKNALA-TTHRTLDLTSSLDVAPTTN--H
XP_022000486.1/1-288 --D-AP-LIPSGFRI-IPLEPK------SGDAKNALV-TTHRTLDLTSSLDVTPTAN--Q
XP_016717477.1/1-281 --D-AA-LLPSGFRI-IPLESKP----------DSLA--TNRTLDLTSSLEVGPATS--Q
XP_017643014.1/1-281 --D-AA-FLPSGFRI-IPLESKP----------DSLA--TNRTLDLTSSLEVGPATS--Q
XP_016724213.1/1-281 --D-AA-LLPSGFRI-IPLESKP----------DSSA--TNRTLDLTSSLEVGPATS--Q
XP_012462938.1/1-281 --D-AA-LLPSGFRI-ILLESKP----------DSSA--TNRTLDLTSSLEVGPATS--Q
XP_012471509.1/1-292 --D-AP-LIPSGFRI-IPLESKPGVIIIQADTQDSLT--TNRTLDLTSSLEVGPATN--H
XP_017642742.1/1-292 --D-AP-LIPSGFRI-IPLESKPGVIIIQSDTKDSLT--TNRTLDLTSSLEVGPATN--H
XP_016755876.1/1-292 --D-AP-LIPSGFRI-IPLESKPGVIIIQSDTKDSLT--TNRTLDLTSSLEVGPATN--H
XP_012471510.1/1-286 --D-AP-LIPSGFRI-IPLESKP------ADTQDSLT--TNRTLDLTSSLEVGPATN--H
XP_016753711.1/1-286 --D-AP-LIPSGFRI-IPLESKP------SDTQDSLT--TNRTLDLTSSLEVGPATN--H
XP_017642744.1/1-286 --D-AP-LIPSGFRI-IPLESKP------SDTKDSLT--TNRTLDLTSSLEVGPATN--H
XP_016755877.1/1-286 --D-AP-LIPSGFRI-IPLESKP------SDTKDSLT--TNRTLDLTSSLEVGPATN--H
XP_016750468.1/1-286 --D-GP-LLPSGFRV-IPLDSKS------SDTQDSLT--TNRTLDLTSSLEVGPATN--H
XP_016688657.1/1-286 --D-AP-LLPSGFRV-IPLDSKS------SDTQDSLT--TNRTLDLTSSLEVGPATN--H
XP_012443272.1/1-286 --D-AP-LLPSGFRV-IPLDSKS------SDTQDSLT--TNRTLDLTSSLEVGPATN--H
XP_017603001.1/1-286 --D-GP-LLPSGFRV-IPLDSKS------SDTQDSLT--TNRTLDLTSSLEVGPATN--H
XP_022755632.1/1-285 --D-AP-LLPSGFHI-IPLESKS------SDTQDSLT--TNRTLDLTSSLEVGPATN--H
XP_022755633.1/1-285 --D-AP-LLPSGFHI-IPLESKS------SDTQDSLT--TNRTLDLTSSLEVGPATN--H
XP_022729815.1/1-286 --D-AP-LLPSGFRI-IPLESKP------SDTQDSLT--TNRTLDLTSSLEVGPATN--H
XP_022729816.1/1-286 --D-AP-LLPSGFRI-IPLESKP------SDTQDSLT--TNRTLDLTSSLEVGPATN--H
XP_022752857.1/1-286 --D-AP-LLPSGFRV-IPLDSKP------SDAQDSLT--TNRTLDLTSSLEVGPATN--H
XP_022752856.1/1-286 --D-AP-LLPSGFRV-IPLDSKP------SDAQDSLT--TNRTLDLTSSLEVGPATN--H
XP_021277981.1/1-286 --D-AP-LLPSGFRI-IPLDSKP------SDTQDSLT--TNRTLDLTSSLEVGTATN--H
XP_017983554.1/1-286 --D-AP-LLPSGFRI-IPLDSKP------SDTQDSLT--TNRTLDLTSSLEVGTATN--H
XP_011089912.1/1-283 --D-AP-LLPSGFRI-IPLDSKP------GDSQDVLT--SHKTLDLASSLEVGPATS--H
XP_007208090.1/1-286 --D-AP-LVPSGFRI-IPLDSKT------SDSKDTLA--THRTLDLTSSLEVGSTTN--N
XP_008225704.1/1-286 --D-AP-LVPSGFRI-IPLDSKT------SDSKDTLA--THRTLDLTSSLEVGSTTN--N
XP_021830853.1/1-286 --D-AP-LVPSGFRI-IPLDSKT------SDSKDTLA--THRTLDLTSSLEVGSTTN--N
XP_008349754.1/1-286 --D-AP-LLPSGFRI-IPLDSKT------GDSKDTLN--THRTLDLTSSLEVGSTTS--N
NP_001315783.1/1-288 --D-AP-LLPSGFRI-IPLDSKT------GDSKDTLN--THRTLDLTSSLEVGSTTS--N
XP_009367069.1/1-286 --D-AP-LLPSGFRI-IPLDSKT------SDSKATLN--THRTLDLASSLEVGSTTS--N
XP_008344872.1/1-286 --D-AP-LLPSGFRI-IPLDSKT------SDSRATLN--THRTLDLASSLEVGSTTS--N
XP_015952399.1/1-286 --D-AP-LVPSGFRI-IPLDSKP------GDKKDATT--SNRTLDLTSGLEVGPATT--Q
XP_020982118.1/1-286 --D-AP-LVPSGFRI-IPLDSKP------GDKKDATT--SNRTLDLTFGLEVGPATT--Q
XP_025685402.1/1-286 --D-AP-LVPSGFRI-IPLDSKP------GDKKDATT--SNRTLDLTSGLEVGPATT--Q
XP_016187430.1/1-286 --D-AP-LVPSGFRI-IPLDSKP------GDKKDATT--SNRTLDLTSGLEVGPPTT--Q
XP_020214107.1/1-284 --D-AP-LVPSGFRI-IPLDSKP------GDKKDTVA--TNRTLDLTSGFEVSPATT--Q
XP_006592258.1/1-284 --D-AP-LIPSGFRI-IPLDSKP------GDKKE-VA--TNRTLDLTSGFEVGPATT--A
XP_007132262.1/1-285 --D-AP-LVPSGFRI-IPLDSKP------GDKKDIVA--TNRTLDLTSGFEVGPATT--A
XP_003538150.1/1-285 --D-AP-LVPSGFRI-IPLDSKP------GDKKDAVA--TNRTLDLTSGFEVGPATT--A
XP_017433493.1/1-285 --D-AP-LVPSGFRI-IPLDSKP------GDKKDIVA--TNRTLDLTSGFEVGPATT--T
XP_014494018.1/1-285 --D-AP-LVPSGFRI-IPLDSKP------GDKKDIVA--SNRTLDLTSGFEVGPATT--T
XP_010089983.2/1-286 --D-AP-LLPSGFRI-IPLESKT------SDAKDALT--THRTLDLTSSLEVGSTTS--L
XP_006378974.2/1-285 --D-AP-LLPSGFRV-IPLESKT------KDAQEALT--TNRTLDLTSSLEVGPVTN--H
XP_011010051.1/1-285 --D-AP-LLPSGFRV-IPLESKT------KDAQEALT--TNRTLDLTSSLEVGPVTN--H
XP_011010050.1/1-285 --D-AP-LLPSGFRV-IPLESKT------KDAQEALT--TNRTLDLTSSLEVGPVTN--H
XP_021905657.1/1-285 --D-AP-LLPSGFRI-IPLDSKT------SDPQDTLN--RHRTLDLTSSLEVGPATN--T
XP_021610391.1/1-286 --D-AP-LLPSGFRV-IPLDSKT------KDAQTAVN--TSRTLDLTTSLEVGPATN--H
XP_002529946.1/1-286 --D-AP-LLPSGFRI-IPLDSKT------KDTQDALT--TSRTLDLTSSLEVGPAAN--N
XP_021658728.1/1-286 --D-AP-LLPSGFRV-IPLDSKT------RDAQDAVN--TSRTLDLTTSLEVGPATN--H
XP_012064816.1/1-286 --D-AP-LLPSGFRI-IPLDSKT------KDTQDALN--TSRTLDLTSSLEVGPATN--H
XP_021628343.1/1-286 --D-AP-LLPSGFRV-IPLDSKT------KDAPDAVN--ASRTLDLTSSLEVGPATN--H
XP_021657859.1/1-286 --D-AP-LLPSGFRV-IPLDSKT------KDAPDVMN--TSRTLDLTSSLEVGPATN--H
XP_015874442.1/1-285 --D-AP-LLPSGFRI-IPLDSKT------SDTKDALP--THRTLDLTSSLEVGSATN--L
XP_023926843.1/1-286 --D-AP-LLPSGFRI-IPLDSKT------SDAKDSLS--THRTLDLTSSLEVGPATN--H
XP_018819529.1/1-286 --D-AP-LLPSGFRI-IPLDSKT------CDAKDSLT--THRTLDLTSSLEVGPAIN--H
XP_010651164.1/1-286 --D-AP-LLPSGFRI-IPLDSKS------GDTQETLT--THRTLDLTSSLEVGPATN--Q
XP_024034672.1/1-286 --D-GP-LLPSGFRI-IPLDSKT------PDTPDTLT--AHRTLDLTSSLEVGPATN--P
XP_006488084.1/1-286 --D-GP-LLPSGFRI-IPLDSKT------PDTPDTLT--AHRTLDLTSSLEVGPATN--P
XP_024034673.1/1-286 --D-GP-LLPSGFRI-IPLDSKT------PDTPDTLT--AHRTLDLTSSLEVGPATN--P
XP_010264677.1/1-287 --D-SP-LLPSGFRI-IPLDSKT------CEKQDVLT--ANRTLDLASSLEVGPATN--R
XP_010253680.1/1-288 --D-AP-LLPSGFRI-IPLDSKS------CEKQDVLN--AHRTLDLASNLEAGPATN--R
XP_022005829.1/1-282 --D-AP-LIPSGFRI-IPLDPKS------GDPKDAIGTTHR-TLDLTSSLEVGQSTN--H
XP_023751537.1/1-278 --D-AP-LIPSGFRI-IPLDPKS------GDLKDALATTHRTTLDLTSSLEVGPSTN--H
XP_024971452.1/1-283 --D-AP-LIPSGFRI-IPLDPKS----------DALVTTHR-TLDLTSSLEVGPSTN--Q
XP_024971444.1/1-287 --D-AP-LIPSGFRI-IPLDPKS------GDSKDALVTTHR-TLDLTSSLEVGPSTN--Q
XP_024985647.1/1-287 --D-AP-LVPSGFRI-IPLDPKS------NDVKNA-LATPHRTLDLTSSLDVSPATN--H
XP_023749448.1/1-288 --D-AP-LVPSGFRI-IPLDPKS------NDVKNNTMVNAHRTLDLTSSLDASNNHG--S
XP_010689538.1/1-281 --D-AP-LLPSGFRI-IPLDSKP------EA-----A--TNRTLDLTSSLEVGPSSN--H
XP_021858740.1/1-280 --D-AP-LVPSGFRI-IPLDSKP------ET-----A--TNRTLDLTSSLEVGPSTN--H
XP_021773317.1/1-281 --D-AP-LLPSGFRI-IPLDSKP------ET-----A--TNRTLDLTSSLEVGPSTN--N
XP_021745941.1/1-281 --D-AP-LLPSGFRI-IPLDSKP------ET-----A--TNRTLDLTSSLEVGPSTN--N
XP_016729039.1/1-286 --D-AP-LLPSGFRV-IPLDTKS------SDTQDSLA--TNRTLDLTSSLEPGPATN--H
XP_017623828.1/1-286 --D-AP-LLPSGFRV-IPLDTKS------SDTQDSLA--TNRTLDLTSSLEPGPATN--H
XP_012476406.1/1-286 --D-AP-LLPSGFRV-IPLDTKS------SDTQDSLA--TNRTLDLTSSLEPGPATN--H
XP_016754776.1/1-286 --D-AP-LLPSGFRV-IPLDTKS------SDTQDSLA--TNRTLDLTSSLEPGPATN--H
XP_022152944.1/1-286 --D-AV-LLPSGFRI-IPLHSKT------GDPKDAWN--SNRTLDLTSSLELGSYAP--Q
XP_011040884.1/1-286 --D-AP-LLPSGFRI-IPLESKA------KDTQEVVT--TNCTLDLTSSLEAGLAIN--H
XP_010043753.1/1-286 --D-AP-LLPSGFRI-IPLDSKS------SDVQDSLT--TNRTLDLTSSLEVGPAST--N
XP_024158223.1/1-285 --D-AP-LLPSGFRI-IPLESKT------SDSKDAMA--THRTLDLTSSLEVGSTTN--H
XP_004291573.1/1-285 --D-AP-LLPSGFRI-IPLESKT------SDSKDALA--THRTLDLTSSLEVGSTTN--H
XP_018808765.1/1-285 --D-AP-LLPSGFRI-IPLDSKT------CDAKDSLP--THRTLDLTSSLEVGPATD--H
XP_023512455.1/1-283 --D-AP-LLPSGFRI-IPLDSRS------SDAKGS-----QRTLDLTSSLEVGCGTS--N
XP_023512456.1/1-283 --D-AP-LLPSGFRI-IPLDSRS------SDAKGS-----QRTLDLTSSLEVGCGTS--N
XP_022944186.1/1-283 --D-AP-LLPSGFRI-IPLDSRS------SDAKGS-----QRTLDLTSSLEVGCGTS--N
XP_022944185.1/1-283 --D-AP-LLPSGFRI-IPLDSRS------SDAKGS-----QRTLDLTSSLEVGCGTS--N
XP_022985614.1/1-283 --D-AP-LLPSGFRI-IPLDSRS------SDAKGS-----QRTLDLTSSLEVGCGTS--N
XP_022985615.1/1-283 --D-AP-LLPSGFRI-IPLDSRS------SDAKGS-----QRTLDLTSSLEVGCGTS--N
XP_011656996.1/1-283 --D-AP-LLPSGFRI-IPLDSRT------SDAKGS-----QRTLDLTSSLEVGSGTS--N
XP_011656997.1/1-283 --D-AP-LLPSGFRI-IPLDSRT------SDAKGS-----QRTLDLTSSLEVGSGTS--N
XP_008463945.1/1-283 --D-AP-LLPSGFRI-IPLDSRT------SDAKGS-----QRTLDLTSSLEVGSGTS--N
XP_008463946.1/1-283 --D-AP-LLPSGFRI-IPLDSRT------SDAKGS-----QRTLDLTSSLEVGSGTS--N
XP_022140363.1/1-286 --D-AP-LLPSGFRI-IPLDSKT------NDAKGTLT--TQRTLDLTSSLEVGSGTS--N
XP_022995706.1/1-286 --D-AP-LLPSGFRI-IPLDSRT------SDAQGALT--TQRTLDLTSSLEVGSGTS--N
XP_023534373.1/1-286 --D-AP-LLPSGFRI-IPLDSRT------SDAKGALT--TQRTLDLTSSLEVGSGTS--N
XP_022958713.1/1-286 --D-AP-LLPSGFRI-IPLDSRT------SDAKGALT--TQRTLDLTSSLEVGSGTS--N
XP_025614062.1/1-285 --V-AP-LLPSGFRI-IPLDSKP------GDKKDSMT--GSQTLDLTSGFEVGPTTN---
XP_025614063.1/1-285 --V-AP-LLPSGFRI-IPLDSKP------GDKKDSMT--GSQTLDLTSGFEVGPTTN---
XP_016170485.1/1-285 --D-AP-LLPSGFRI-IPLDSKP------GDKKDSMT--GSQTLDLTSGFEVGPTTN---
XP_016170486.1/1-285 --D-AP-LLPSGFRI-IPLDSKP------GDKKDSMT--GSQTLDLTSGFEVGPTTN---
XP_015935757.1/1-285 --D-AP-LLPSGFRI-IPLDSKP------GDKKDSMT--GSQTLDLTSGFEVGPTTN---
XP_015935758.1/1-285 --D-AP-LLPSGFRI-IPLDSKP------GDKKDSMT--GSQTLDLTSGFEVGPTTN---
XP_004505999.1/1-286 --D-AP-LVPSGFRI-IPLDSKP------GDKKDAVN--SNRTLDLASGIDMSPATT--H
XP_013456006.1/1-286 --D-AP-LVPSGFRI-IPLDSKP------GDKKDAVN--TNRTLDLTSGLEVSPATT--H
XP_019453966.1/1-285 --D-AL-LVPSGFRV-IPLASKP------GDKKDTIS--TNRTLDLTSGLEVGPMTA--H
XP_019448740.1/1-279 --D-AP-LVASGFRI-IPLASKQ------GDKKV-------ETLDLTSGLEVVPMTV--H
XP_019448741.1/1-279 --D-AP-LVASGFRI-IPLASKQ------GDKKV-------ETLDLTSGLEVVPMTV--H
XP_019458095.1/1-286 --D-AP-LVPSGFRI-IPLDSKP------GDKKDVTT--ANQTLNLTSGLDVGPSAN--N
XP_019447870.1/1-286 --D-AP-LVPSGFRI-IPLDSKP------GDKKDATT--GNRTLDLTSGLEVGLATS--H
XP_012838629.1/1-280 --D-SP-LLPSGFRI-IPLHSKP----------DVLN--THKTLDLTSSLEVGPTSG--S
XP_012838630.1/1-280 --D-SP-LLPSGFRI-IPLHSKP----------DVLN--THKTLDLTSSLEVGPTSG--S
XP_012838627.1/1-280 --D-SP-LLPSGFRI-IPLHSKP----------DVLN--THKTLDLTSSLEVGPTSG--S
XP_012838628.1/1-280 --D-SP-LLPSGFRI-IPLHSKP----------DVLN--THKTLDLTSSLEVGPTSG--S
XP_017214964.1/1-286 --D-AP-LLPSGFRI-IPLDSKS------GDVQDSLT--TNRTLDLASSLEVGPGTN--H
XP_017234817.1/1-286 --D-AP-LLPSGFRI-IPLESKS------GDVQDSIT--TNRTLDLASSLEVGPAAN--H
XP_019178153.1/1-282 --D-AP-LLPSGFRI-IPLDSIS------NDAQDTLN--AQRTLDLTSSLEVGPATN--T
XP_019166562.1/1-283 --D-AL-LIPSGFRI-IPLESKS------NDVQDALP--AQRTLDLTSSLEVGLATT--N
XP_011089294.1/1-282 --D-AQ-LLPSGFRL-IPLDSKP------GDFQDSLT--SHKTLDLTSSLEVGPATS--N
XP_022892122.1/1-274 --D-AP-LVPSGFRI-IPLDSKP------GDSQDTLT--AQRTLDLTSSLEVGPETN--H
XP_022883393.1/1-279 --D-VP-LVPSGFRI-IPLDSKP----------DTLT--AQRTLDLTSNLEVDPETN--H
XP_022883398.1/1-279 --D-VP-LVPSGFRI-IPLDSKP----------DTLT--AQRTLDLTSNLEVDPETN--H
XP_022883384.1/1-283 --D-VP-LVPSGFRI-IPLDSKP------GDSQDTLT--AQRTLDLTSNLEVDPETN--H
XP_022883389.1/1-283 --D-VP-LVPSGFRI-IPLDSKP------GDSQDTLT--AQRTLDLTSNLEVDPETN--H
XP_009776589.1/1-283 --D-AP-LLPSGFRI-IPLESKS------SDPQDTSN--AHRTLDLASSLEVGPATN--P
NP_001312504.1/1-283 --D-AP-LLPSGFRI-IPLESKS------SDPQDTSN--AHRTLDLASSLEVGPATN--P
XP_016472209.1/1-283 --D-AP-LLPSGFRI-IPLESKS------SDPQDTSN--AHRTLDLASSLEVGPATN--P
XP_019255429.1/1-283 --D-AP-LLPSGFRI-IPLESKS------SDPQDTSN--AHRTLDLASSLEVGPATN--P
XP_009624234.1/1-283 --D-AP-LLPSGFRI-IPLESKS------SDPQDTSN--AHRTLDLASSLEVGPATN--P
XP_016504967.1/1-283 --D-AP-LLPSGFRI-IPLESKS------SDPQDTSN--AHRTLDLASSLEVGPATN--P
XP_016547131.1/1-283 --D-AP-LLPSGFRI-IPLESKS------SDSHDTLN--THRTLDLASSLEVGAATN--P
XP_004251138.1/1-283 --D-AP-LLPSGFRI-IPLESKS------GDAQDTLN--AHRTLDLASSLEVGPATN--S
XP_015058512.1/1-283 --D-AP-LLPSGFRI-IPLESKS------GDALDTLN--AHRTLDLASSLEVGPATN--S
XP_006340193.1/1-283 --D-AP-LLPSGFRI-IPLESKS------GDAQDTLN--AHRTLDLASSLEVGPARN--S
XP_004235650.1/1-283 --D-AP-LLPSGFRI-ISLESGK------------EASSPNRTLDLTSALETGPAEN--K
XP_006343047.1/1-283 --D-AP-LLPSGFRI-ISLESGK------------EASSPNRTLDLTSALETGPAEN--K
XP_009774805.1/1-285 --D-AP-LLPSGFRI-ISLESGK------------ESSSPNRTLDLTSALETGPAEN--K
XP_016498616.1/1-285 --D-AP-LLPSGFRI-ISLESGK------------ESSSPNRTLDLTSALETGPAEN--K
XP_016434103.1/1-285 --D-AP-LLPSGFRI-ISLESGK------------ESSSPNRTLDLTSALETGPAEN--K
XP_009593967.1/1-285 --D-AP-LLPSGFRI-ISLESGK------------ESSSPNRTLDLTSALETGPAEN--K
XP_004510297.1/1-285 --D-AP-LLPSGFRI-IPLESGK-------E-----ASSPNRTLDLASALDIGPTGN--R
XP_004510296.1/1-285 --D-AP-LLPSGFRI-IPLESGK-------E-----ASSPNRTLDLASALDIGPTGN--R
XP_003627005.1/1-284 --D-AP-LLPSGFRI-IPLESGK-------E-----SSSPNRTLDLASALDIGPTGN--R
XP_003531653.1/1-284 --D-AP-LLPSGFRI-IPLESGK------------EASSPNRTLDLASSLDVGPSGN--R
XP_006585576.1/1-288 --D-AP-LLPSGFRI-IPLESGK-------KVL-QEASSPNRTLDLASSLDVGPSGN--R
XP_014634618.1/1-288 --D-AP-LLPSGFRI-IPLESGK-------KVL-QEASSPNRTLDLASSLDVGPSGN--R
XP_014634619.1/1-288 --D-AP-LLPSGFRI-IPLESGK-------KVL-QEASSPNRTLDLASSLDVGPSGN--R
XP_003531652.1/1-285 --D-AP-LLPSGFRI-IPLESGK------------EASSPNRTLDLASSLDVGPSGN--R
XP_003530109.1/1-284 --D-AP-LLPSGFRI-IPLESGK------------EASSPNRTLDLASALDVGPSGN--R
XP_007135643.1/1-285 --D-AP-LLPSGFRI-IPLESGK------------EASSPNRTLDLASALDIGPSGN--R
XP_003530112.1/1-285 --D-AP-LLPSGFRI-IPLESGK------------EASSPNRTLDLASALDIGSSGN--R
XP_020226142.1/1-285 --D-AP-LLPSGFRI-IPLESGK------------EASSPNRTLDLASALDVGPSGN--R
XP_014516466.1/1-285 --D-AP-LLPSGFRI-IPLESGK------------EASSPNRTLDLASALDIGPSGN--R
XP_017407594.1/1-285 --D-AP-LLPSGFRI-IPLESGK------------EASSPNRTLDLASALDIGPSGN--R
XP_019448178.1/1-285 --D-AP-LLPSGFRI-IPLDSGK-------EA-----SNPNRTLDLASALDIGPTGN--K
XP_019429328.1/1-285 --D-AP-LLPSGFRI-IPLQSGK-------EA-----SSPNRTLDLTSALDIGPTKN--K
XP_004486776.1/1-285 --D-AP-LLPSGFRI-IPLDSGK-------EV----V-NPNRTLDLASALDIGPAGN--K
XP_003597690.1/1-286 --D-AP-LLPSGFRI-IPLDSGK-------EV----ANNPNRTLDLTSALDIGPAGN--K
XP_015932679.1/1-285 --D-AP-LLPSGFRI-IPLDSGK-------EA-----SNPNRTLDLASALDIGPTGN--R
XP_025674177.1/1-285 --D-AP-LLPSGFRI-IPLDSGK-------EA-----SNPNRTLDLASALDIGPTGN--R
XP_025610204.1/1-285 --D-AP-LLPSGFRI-IPLDSGK-------EA-----SNPNRTLDLASALDIGPTGN--R
XP_020226055.1/1-285 --D-AP-LLPSGFRI-IPLDSGK-------EA-----SNPNRTLDLASALDVGPTGN--R
XP_007150614.1/1-285 --D-AP-LLPSGFRI-IPLDSAK-------EA-----SNPNRTLDLTSALDVGPTGN--R
XP_014501306.1/1-285 --D-AP-LLPSGFRI-IPLDSAK-------EA-----SNPNRTLDLASALDIGPTGN--R
XP_017425270.1/1-285 --D-AP-LLPSGFRI-IPLDSAK-------EA-----SNPNRTLDLASALDIGPTGN--R
XP_021836126.1/1-285 --D-AP-LLPSGFRI-IPLDSVK------------EASSPNRTLDLASALEVGPSAN--R
XP_010691423.1/1-284 --D-AP-LLPSGFRI-IPLDSAK------------EASSPNRTLDLASALEVGPSAN--R
XP_010691424.1/1-284 --D-AP-LLPSGFRI-IPLDSAK------------EASSPNRTLDLASALEVGPSAN--R
XP_021765330.1/1-284 --D-AP-LLPSGFRI-IPLDSVK------------ETSSPNRTLDLASALEVGSSAN--R
XP_021749885.1/1-284 --D-AP-LLPSGFRI-IPLDSVK------------ETSSPNRTLDLASALEVGSSAN--R
XP_010267183.1/1-285 --D-AP-LLPSGFRI-IPLDTGV------------DASSPNRTLDLASALEVGTAGN--R
XP_017258627.1/1-285 --D-AP-LLPSGFRI-IPLDTGK------------EASSPNRTLDLASALEIGSAGN--K
XP_023000315.1/1-284 --D-AP-LLPSGFRI-IPLDSGK------------ETSSPNRTLDLASALEIGPTGN--R
XP_023000313.1/1-284 --D-AP-LLPSGFRI-IPLDSGK------------ETSSPNRTLDLASALEIGPTGN--R
XP_023514554.1/1-284 --D-AP-LLPSGFRI-IPLDSGK------------ETSSPNRTLDLASALEIGPAGN--R
XP_023514552.1/1-284 --D-AP-LLPSGFRI-IPLDSGK------------ETSSPNRTLDLASALEIGPAGN--R
XP_022964458.1/1-284 --D-AP-LLPSGFRI-IPLDSGK------------ETSSPNRTLDLASALEIGPAGN--R
XP_022964454.1/1-284 --D-AP-LLPSGFRI-IPLDSGK------------ETSSPNRTLDLASALEIGPAGN--R
XP_022147627.1/1-284 --D-AP-LLPSGFRI-IPLDSGK------------EASSPNRTLDLASALEIGPAGN--R
XP_022147619.1/1-284 --D-AP-LLPSGFRI-IPLDSGK------------EASSPNRTLDLASALEIGPAGN--R
XP_008462437.1/1-284 --D-AP-LLPSGFRI-IPLDSGK------------EASSPNRTLDLASALEIGPAGN--R
XP_004141659.1/1-284 --D-AP-LLPSGFRI-IPLDSGK------------EASSPNRTLDLASALEIGPAGN--R
XP_008462436.1/1-284 --D-AP-LLPSGFRI-IPLDSGK------------EASSPNRTLDLASALEIGPAGN--R
XP_011659682.1/1-284 --D-AP-LLPSGFRI-IPLDSGK------------EASSPNRTLDLASALEIGPAGN--R
XP_022992111.1/1-284 --D-AP-LLPSGFRI-IPLDSGK------------EASSPNRTLDLASALEIGPAGN--R
XP_023548534.1/1-284 --D-AP-LLPSGFRI-IPLDSGK------------EASSPNRTLDLASALEIGPAGN--R
XP_022953371.1/1-284 --D-AP-LLPSGFRI-IPLDSGK------------EASSPNRTLDLASALEIGPAGN--R
XP_002304217.1/1-285 --D-AP-LLPSGFRI-IPLDSGK------------EASSPNRTLDLAAALEVGPAGN--R
XP_011013041.1/1-285 --D-AP-LLPSGFRI-IPLDSGK------------EASSPNRTLDLAAALEVGPAGN--R
XP_011013042.1/1-285 --D-AP-LLPSGFRI-IPLDSGK------------EASSPNRTLDLAAALEVGPAGN--R
XP_011046759.1/1-284 --D-AP-LLPSGFRI-IPLDSGK------------EASSPNRTLDLASALEVG-AGN--R
XP_024464085.1/1-284 --D-AP-LLPSGFRI-IPLDSGK------------EASSPNRTLDLASALEVG-AGN--R
XP_024183498.1/1-286 --D-AP-LLPSGFRI-IPLDSGK------------EASSPNRTLDLASALEVGPTGN--R
XP_004302866.1/1-286 --D-AP-LLPSGFRI-IPLDSGK------------EASSPNRTLDLASALEIGPTGN--R
NP_001280898.1/1-285 --D-AP-LLPSGFRI-IPLDYGK------------EASSPNRTLDLASALEIGPTGN--K
XP_007214960.1/1-285 --D-AP-LLPSGFRI-IPLDYGK------------EASSPNRTLDLASALEIGPTGN--K
XP_008228437.1/1-285 --D-AP-LLPSGFRI-IPLDYGK------------EASSPNRTLDLASALEIGPTGN--K
XP_021818763.1/1-285 --D-AP-LLPSGFRI-IPLDYGK------------EASSPNRTLDLASALEIGPTGN--K
XP_022723515.1/1-285 --D-AP-LLPSGFRI-IPLDSRK------------EASSPNRTLDLASALEIGPAGN--K
XP_016688508.1/1-285 --D-AP-LLPSGFRI-IPLDSGK------------EASSPNRTLDLASALEIGPAGN--K
XP_012443274.1/1-285 --D-AP-LLPSGFRI-IPLDSGK------------EASSPNRTLDLASALEIGPAGN--K
XP_022721457.1/1-285 --D-AP-LLPSGFRI-IPLDSGK------------EVSSPNRTLDLASALEIGPAGN--K
XP_022715018.1/1-285 --D-AP-LLPSGFRI-IPLDSGK------------EASSPNRTLDLASALDIGPAGN--K
XP_017979082.1/1-285 --D-AP-LLPSGFRI-IPLDSGK------------EASSPNRTLDLASALEIGPTGN--K
XP_021293768.1/1-285 --D-AP-LLPSGFRI-IPLDSGK------------EASSPNRTLDLASALEIGPTGN--K
XP_010062518.1/1-285 --D-AP-LLPSGFRI-IPLDSVK------------EASSPNRTLDLASSLEIGPAGN--R
XP_006426893.1/1-285 --D-AP-LLPSGFRI-IPLDSGK------------ETSSPNRTLDLASALEIGPAGN--R
XP_021610318.1/1-285 --D-AP-LLPSGFRI-IPLDSAK------------EASSPNRTLDLASALETGPAGN--K
XP_010098130.1/1-285 --D-AP-LLPSGFRI-IPLDSGK------------EASSPNRTLDLASALEIGPNGN--K
XP_021635282.1/1-296 --D-AP-LLPSGFRI-IPLDSAKVFILMHYKIM-QEASSPNRTLDLASALEIGPAGN--K
XP_002284003.2/1-285 --D-AP-LLPSGFRI-IPLDSGK------------EASSPNRTLDLASALEIGPAGN--R
XP_002515977.1/1-285 --D-AP-LLPSGFRI-IPLDSAK------------EASSPNRTLDLASALEIGPAGN--K
XP_021643260.1/1-285 --D-AP-LLPSGFRI-IPLDSAK------------EASSPNRTLDLASALETGPAGN--K
XP_021628194.1/1-285 --D-AP-LLPSGFRI-IPLDSGK------------EASSPNRTLDLASALEIGPAGN--K
XP_015894591.1/1-285 --D-AP-LLPSGFRI-IPLDSGK------------EASSPNRTLDLASALEIGPSGN--K
XP_012069098.1/1-285 --D-AP-LLPSGFRI-IPLDSAK------------EASSPNRTLDLASALEIGPAGN--K
XP_018847857.1/1-285 --D-AP-LLPSGFRI-IPLDSGK------------EASSPNRTLDLASALEIGPAGN--R
XP_023898511.1/1-285 --D-AP-LLPSGFRI-IPLDSGK------------EASSPNRTLDLASALEIGPAGN--K
XP_010545673.1/1-285 --D-AP-LLPSGFRI-IPLDLGK------------EVPSPNRTLDLASALEVGSAGT--K
XP_010545670.1/1-286 --D-AP-LLPSGFRI-IPLDLGKQ-----------EVPSPNRTLDLASALEVGSAGT--K
XP_010535179.1/1-285 --D-AP-LLPSGFRI-IPLDSGK------------EVSSPNRTLDLASALEVGPTGT--K
XP_018436929.1/1-283 --D-AP-LLPSGFRI-IPLDSTK------------EVSSPNRTLDLASALEIGSAGT--T
XP_013592012.1/1-283 --D-AP-LLPSGFRI-IPLDSTK------------EVSSPNRTLDLASALEIGPAGT--T
XP_013640375.1/1-284 --D-AP-LLPSGFRI-IPLDSTKQ-----------EVSSPNRTLDLASALEIGPAGT--T
XP_013640376.1/1-283 --D-AP-LLPSGFRI-IPLDSTK------------EVSSPNRTLDLASALEIGPAGT--T
XP_009147709.1/1-283 --D-AP-LLPSGFRI-IPLDSTK------------EVSSPNRTLDLASALEIGPAGT--T
XP_006306297.1/1-281 --D-AP-LLPSGFRI-IPLDSAK------------EVSSPNRTLDLASALEIGSAGT--K
XP_010462051.1/1-281 --D-AP-LLPSGFRI-IPLDSAK------------EVSSPNRTLDLASALEIGSAGT--K
XP_010479714.1/1-281 --D-AP-LLPSGFRI-IPLDSAK------------EVSSPNRTLDLASALEIGSAGT--K
XP_010462052.1/1-281 --D-AP-LLPSGFRI-IPLDSAK------------EVSSPNRTLDLASALEIGSAGT--K
XP_010500807.1/1-281 --D-AP-LLPSGFRI-IPLDSAK------------EVSSPNRTLDLASALEVGSAGT--K
NP_849795.1/1-282    --D-AP-LLPSGFRI-IPLDSAKQ-----------EVSSPNRTLDLASALEIGSAGT--K
NP_001077702.1/1-281 --D-AP-LLPSGFRI-IPLDSAK------------EVSSPNRTLDLASALEIGSAGT--K
NP_175627.1/1-281    --D-AP-LLPSGFRI-IPLDSAK------------EVSSPNRTLDLASALEIGSAGT--K
XP_020868586.1/1-281 --D-AP-LLPSGFRI-IPLDSAK------------EVSSPNRTLDLASALEIGSAGT--K
XP_006392953.1/1-282 --D-AP-LLPSGFRI-IPLDSAK------------EVSSPNRTLDLASALEIGSAGT--T
XP_018460807.1/1-282 --D-AP-LLPSGFRI-IPLDSTK------------EVSSPNRTLDLASALEIGSAGT--A
XP_013621760.1/1-283 --D-AP-LLPSGFRI-IPLDSTK------------EASSPNRTLDLASALEIGPAGT--T
XP_018451524.1/1-282 --D-AP-LLPSGFRI-IPLDSTK------------EVSSPNRTLDLASALEIGSAGT--T
XP_009107148.1/1-282 --D-AP-LLPSGFRI-IPLDSTK------------EASSPNRTLDLASALEIGPAGT--T
XP_009107147.1/1-283 --D-AP-LLPSGFRI-IPLDSTKQ-----------EASSPNRTLDLASALEIGPAGT--T
XP_009600774.1/1-284 --D-AP-LLPSGFRI-IPLDSAK------------EASSPNRTLDLTSALEIGPAES--K
XP_016459079.1/1-284 --D-AP-LLPSGFRI-IPLDSAK------------EASSPNRTLDLTSALEIGPAES--K
XP_019236029.1/1-285 --D-AP-LLPSGFRI-IPLDSAK------------EASSPNRTLDLTSALEIGPAES--K
XP_009771098.1/1-285 --D-AP-LLPSGFRI-IPLDSAK------------EASSPNRTLDLTSALEIGPAES--K
XP_016550962.1/1-285 --D-AP-LLPSGFRI-IPLDSAK------------EASSPNRTLDLTSALETGPVGS--E
XP_016550960.1/1-285 --D-AP-LLPSGFRI-IPLDSAK------------EASSPNRTLDLTSALETGPVGS--E
XP_016550961.1/1-285 --D-AP-LLPSGFRI-IPLDSAK------------EASSPNRTLDLTSALETGPVGS--E
XP_006359469.1/1-285 --D-AP-LLPSGFRI-IPLDSAK------------EASSPNRTLDLTSALETGPVGS--K
XP_004252354.1/1-285 --D-TP-LLPSGFRI-IPLDSAK------------EASSPNRTLDLTSALETGPVGS--K
XP_015061298.1/1-285 --D-TP-LLPSGFRI-IPLDSAK------------EASSPNRTLDLTSALETGPVGS--K
XP_019198775.1/1-285 --D-AP-LLPSGFRI-IPLDAGK------------EASSPSRTLDLTSALETGTAEN--K
XP_019194412.1/1-285 --D-AP-LLPSGFRI-IPLDFGK------------ESSSPNRTLDLTSALETGTAEN--K
XP_016562656.1/1-283 --D-AP-LLPSGFRI-ISLESGK------------EASSPNRTLDLTSALETGPAEN--K
XP_019267274.1/1-285 --D-AP-LLPSGFRI-ISLESGK------------ESSSPNRTLDLTSALETGPAEN--K
XP_019267275.1/1-282 --D-AP-LLPSGFRI-ISLESGK------------ESSSPNRTLDLTSALETGPAEN--K
XP_022889649.1/1-285 --D-AP-LLPSGFRI-IPLDSCE------------ENSSPSRTLDLASALETKTTGN--K
XP_022889648.1/1-285 --D-AP-LLPSGFRI-IPLDSCE------------ENSSPSRTLDLASALETKTTGN--K
XP_012852689.1/1-281 --D-AP-LLPSGFRI-IPLDPGK------------DASSPNRTLDLASALET---GN--K
XP_012856468.1/1-279 --D-VP-LLPSGFRI-IPLDSCK------------ETGSPNRTLDLASALEAGTAGA--K
XP_011080516.1/1-285 --D-AP-LLPSGFRI-IPLDSSK------------EASSPNRTLDLASALESGTAGN--K
XP_011072422.1/1-285 --D-AP-LLPSGFRI-IPLDSGK------------EASSPNRTLDLASALETGTAGN--K
XP_022851148.1/1-285 --D-AP-LLPSGFRI-IPLDSGK------------EASSPNRTLDLASALEIGTAGN--K
XP_022844668.1/1-285 --D-AP-LLPSGFRI-IPLDSGK------------ESPSPNRTLDLASALEIGTAGN--R
XP_006853643.2/1-288 --D-AP-LLPSGFRI-IPLDNGV------------DGSSPNRTLDLASALEVGPAGN--R
XP_010249545.1/1-285 --D-AP-LLPSGFRI-IPLDSGV------------DASSPNRTLDLASALEVGATGS--R
XP_010249539.1/1-285 --D-AP-LLPSGFRI-IPLDSGV------------DASSPNRTLDLASALEVGATGS--R
XP_010915409.1/1-284 --D-AP-LLPSGFRI-IPLDSVM------------ENSSPNRTLDLASALEIGSTGN--R
XP_008791633.1/1-284 --D-AP-LLPSGFRI-IPLDSVM------------ESSSPNRTLDLASTLEIGSTGN--R
XP_017235966.1/1-285 --D-AP-LLPSGFRI-ICLDSKK------------ESSSPNRTLDLASALEVGSAVS--K
XP_023761873.1/1-288 --D-AP-LLASGFRI-IPLDSGKE------------ASSPNRTLDLASALEVKGSGN--K
XP_021997125.1/1-286 --D-AP-LLPSGFRI-IPLDSCKG------------DSSPNRTLDLASALEVRGAGN--R
XP_021990052.1/1-285 --E-AP-LLASGFRI-IPLDSCKG------------DSSPNRTLDLASALDVRGTGN--K
XP_024979339.1/1-285 --D-AP-LLPSGFRI-IPLDFCKE------------ASSPSRTLDLASALEVRGPGN--K
XP_024979341.1/1-290 --D-AP-LLPSGFRI-IPLDFCKVRS-----SL-N-ASSPSRTLDLASALEVRGPGN--K
XP_024979340.1/1-291 --D-AP-LLPSGFRI-IPLDFCKVRS-----SL-NEASSPSRTLDLASALEVRGPGN--K
XP_024979342.1/1-291 --D-AP-LLPSGFRI-IPLDFCKVRS-----SL-NEASSPSRTLDLASALEVRGPGN--K
XP_022016208.1/1-285 --D-AP-LLPSGFRI-IPLDSVK------------EKSSPNRTLDLASALEIGGSGN--K
XP_022016207.1/1-311 --D-AP-LLPSGFRI-IPLDSVK------------EKSSPNRTLDLASALEIGGSGN--K
XP_022009819.1/1-285 --D-AP-LLPSGFRI-IPLDPIK------------GGSSPNRTLDLASALDIGGSRT--R
XP_022009815.1/1-285 --D-AP-LLPSGFRI-IPLDPIK------------GGSSPNRTLDLASALDIGGSRT--R
XP_022009809.1/1-285 --D-AP-LLPSGFRI-IPLDPIK------------GGSSPNRTLDLASALDIGGSRT--R
XP_023759500.1/1-286 --D-AP-LLPSGFRI-IPLDSTKE-----------TSGTPNRTLDLASALEIGGSGN--K
XP_023751740.1/1-285 --D-AP-LLPSGFRI-IPLDPIK------------DTSTPNRTLDLASALEIRGSGS--K
XP_024971803.1/1-285 --D-AP-LLPSGFRI-IPLDSGK------------ETSSPNRTLDLASALEIGGSGN--K
XP_022033649.1/1-285 --D-AP-LLPSGFRI-IPLDPTK------------DGSTPNRTLDLASALEIGGSGT--K
XP_024972195.1/1-285 --D-AP-LLASGFRI-IPLDPIK------------EASSPNRTLDLASALEIGGSGT--K
XP_024972196.1/1-289 --D-AP-LLASGFRI-IPLDPIKVRI--------DEASSPNRTLDLASALEIGGSGT--K
XP_020248539.1/1-284 --D-AP-LLPSGFRI-IPLDSVT------------ENSSPNRTLDLASALEVGPTKS--R
XP_020269197.1/1-284 --D-AP-LLPSGFRI-IPLDSVN------------ENSSPNRTLDLASALEVGQTKS--K
XP_020269196.1/1-284 --D-AP-LLPSGFRI-IPLDSVN------------ENSSPNRTLDLASALEVGQTKS--K
XP_009399813.1/1-284 --D-AP-LLPSGFRI-IPLDFKM------------DTNSPNRTLDLASALEVGPTGS--R
XP_009399812.1/1-284 --D-AP-LLPSGFRI-IPLDFKM------------DTNSPNRTLDLASALEVGPTGS--R
XP_009413853.1/1-284 --E-AP-LLPSGFRV-IPLDFKM------------DPTSPNRTLDLASVLEVGPTGS--R
XP_024985513.1/1-286 --N-AP-LLPSGFRI-IPIDNTA------------IYPSQNPTRDLASALEVGPLVN--R
XP_023729871.1/1-286 --D-AP-LLPSGFRI-IPINNVT------------NHPSQNPTRDLASALEVGPPGS--R
XP_022000974.1/1-282 --D-AP-LLPSGFRI-IPINNN----------------SQNPTRDLASTLEVGPPGK--R
XP_022023495.1/1-282 --D-AP-LLPSGFRI-IPVNNN----------------SQNPTRDLASTLEVGPPGK--R
XP_010526102.1/1-286 --D-AP-IIPSGFRI-IPVDSKS------------EGSSPNRTLDLASALEVGGPGN--R
XP_019086035.1/1-282 --D-AP-IIPSGFRI-IPLDSK-------------SGLSPNRTLDLASALDVG---S--R
XP_010437837.1/1-288 --D-AP-IIPSGFRI-IPLDSKSVR------LV-NEGLSPNRTLDLASALDVG---S--R
XP_010437838.1/1-288 --D-AP-IIPSGFRI-IPLDSKSVR------LV-NEGLSPNRTLDLASALDVG---S--R
XP_022549566.1/1-283 --D-AP-IIPSGFRI-IPLDSKS------------EGLSPNRTLDLASALDIG---S--R
XP_013670651.1/1-283 --D-AP-IIPSGFRI-IPLDSKS------------EGLSPNRTLDLASALDIG---S--R
XP_020874560.1/1-283 --D-AP-IIPSGFRI-IPLDSKS------------EGLSPNRTLDLASALDVG---S--R
XP_020874561.1/1-283 --D-AP-IIPSGFRI-IPLDSKS------------EGLSPNRTLDLASALDVG---S--R
XP_020874562.1/1-283 --D-AP-IIPSGFRI-IPLDSKS------------EGLSPNRTLDLASALDVG---S--R
NP_195014.1/1-283    --D-AP-IIPSGFRI-IPLDSKS------------EGLSPNRTLDLASALDVG---S--R
XP_006412419.1/1-283 --D-AP-IIPSGFRI-IPLESKS------------EGLSPNRTLDLASALDVG---S--R
XP_006412418.1/1-283 --D-AP-IIPSGFRI-IPLESKS------------EGLSPNRTLDLASALDVG---S--R
XP_018471819.1/1-283 --D-AP-IIPSGFRI-IPLDSKS------------EGLSPNRTLDLASALDIG---S--R
XP_009125299.1/1-283 --D-AP-IIPSGFRI-IPLDSKS------------EGLSPNRTLDLASALDIG---S--R
XP_010432643.1/1-283 --D-AP-IIPSGFRI-IPLDSKS------------EGLSPNRTLDLASALDVG---S--R
XP_010432644.1/1-283 --D-AP-IIPSGFRI-IPLDSKS------------EGLSPNRTLDLASALDVG---S--R
XP_006283109.1/1-283 --D-AP-IIPSGFRI-IPLDSKS------------EGLSPNRTLDLASALDVG---S--R
XP_010437839.1/1-283 --D-AP-IIPSGFRI-IPLDSKS------------EGLSPNRTLDLASALDVG---S--R
XP_010437840.1/1-283 --D-AP-IIPSGFRI-IPLDSKS------------EGLSPNRTLDLASALDVG---S--R
XP_010447325.1/1-283 --D-AP-IIPSGFRI-IPLDSKS------------EGLSPNRTLDLASALDVG---S--R
XP_010447326.1/1-283 --D-AP-IIPSGFRI-IPLDSKS------------EGLSPNRTLDLASALDVG---S--R
XP_013622315.1/1-283 --D-AP-IIPSGFRI-IPLDTKS------------EGVSPNRTLALASALDVG---S--R
XP_013656273.1/1-283 --D-AP-IIPSGFRI-IPLDTKS------------EGVSPNRTLDLASALDVG---S--R
XP_009108824.1/1-283 --D-AP-IIPSGFRI-IPLDTKS------------EGVSPNRTLDLASALDVG---S--R
XP_013730326.1/1-283 --D-AP-IIPSGFRI-IPLDTKS------------EGVSPNRTLDLASALDVG---S--R
XP_013730325.1/1-283 --D-AP-IIPSGFRI-IPLDTKS------------EGVSPNRTLDLASALDVG---S--R
XP_018447492.1/1-283 --D-AP-IIPSGFRI-IPLDTKS------------EGVSPNRTLDLASALDVG---S--R
XP_019415259.1/1-283 --D-PP-ILPSGFRI-IPLDSGR------------DAASPNRTLDLASALDVGTIGN--K
XP_022980940.1/1-286 --D-AP-ILASGFRI-IPLDSGM------------DASSPNRTLDLASALDVGPAGN--R
XP_023523539.1/1-286 --D-AP-ILASGFRI-IPLDSGM------------DASSPNRTLDLASALDVGPAGN--R
XP_022941184.1/1-286 --D-AP-ILASGFRI-IPLDSGL------------DASSPNRTLDLASALDVGPAGN--R
XP_022134366.1/1-286 --D-AP-ILPSGFRI-IPLDSGM------------DASSPNRTLDLASALDVGPAGN--K
XP_004136224.1/1-286 --D-AP-ILPSGFRI-IPLDSGM------------DASSPNRTLDLASALDVGPAGN--R
XP_008466070.1/1-286 --D-AP-ILPSGFRI-IPLDSGM------------DASSPNRTLDLASALDVGPAGN--R
XP_022976315.1/1-286 --D-AP-ILPSGFRI-IPLDSGM------------DASSPYRTLDLASALDVGPAGN--R
XP_022937345.1/1-286 --D-AP-ILPSGFRI-IPLDSGM------------DASSPYRTLDLASALDVGPAGN--R
XP_023534956.1/1-286 --D-AP-ILPSGFRI-IPLDSGM------------DASSPYRTLDLASALDVGPAGN--R
XP_023535295.1/1-286 --D-AP-ILPSGFRI-IPLDSGM------------DASSPYRTLDLASALDVGPAGN--R
XP_019175908.1/1-285 --D-AP-LLPSGFRI-IPLDSRM------------DASSPNRTLDLASTLEVGTGGT--R
XP_019175907.1/1-285 --D-AP-LLPSGFRI-IPLDSRM------------DASSPNRTLDLASTLEVGTGGT--R
XP_009626659.1/1-285 --D-AP-LLPSGFRI-IPLDSKA------------DASSPNRTLDLASTLEVGPAGS--R
XP_019266312.1/1-285 --D-AP-LLPSGFRI-IPLDSKA------------DASSPNRTLDLASTLEVGPAGS--R
XP_009781184.1/1-285 --D-AP-LLPSGFRI-IPLDSKA------------DASSPNRTLDLASTLEVGPAGS--R
XP_016488110.1/1-285 --D-AP-LLPSGFRI-IPLDSKA------------DASSPNRTLDLASTLEVGPAGS--R
XP_016537761.1/1-285 --D-AP-LLPSGFRI-IPLDSKS------------DASSPNRTLDLASTLEVGPAGS--R
XP_006359741.1/1-285 --D-AP-LLPSGFRI-IPLDAKA------------DASSPNRTLDLASTLEVGPAGS--R
XP_010325078.1/1-288 --D-AP-LLPSGFRI-IPLDSKAS--------M-QDASSPNRTLDLASTLEVGPAGS--R
XP_004245155.1/1-285 --D-AP-LLPSGFRI-IPLDSKA------------DASSPNRTLDLASTLEVGPAGS--R
XP_015085523.1/1-285 --D-AP-LLPSGFRI-IPLDSKA------------DASSPNRTLDLASTLEVGPAGS--R
XP_022868182.1/1-285 --D-AP-LLPSGFRI-IPLDSKA------------NTSSPNRTLDLASTLEVGQAGN--R
XP_022868181.1/1-285 --D-AP-LLPSGFRI-IPLDSKA------------NTSSPNRTLDLASTLEVGQAGN--R
XP_022854559.1/1-285 --D-AP-LLPSGFRI-IPLDSKA------------DTSSPNRTLDLASTLEVGPVGN--K
XP_011081776.1/1-285 --D-AP-LLPSGFRI-IPLDSKT------------DASTPNRTLDLASTLEVGPTGN--R
XP_012857948.1/1-282 --D-AP-LLPSGFRI-IPLDSKS------------DASSPNRTLDLASTLEVGPNNK--Q
XP_021847759.1/1-284 --D-AP-LLPSGFRI-IPIDSSK------------DVSSPKCTLDLTSALEVGHAGN--K
XP_010668539.1/1-284 --D-AP-LLPSGFRI-IPVDSSK------------DVSSPKCTLDLTSALEVGPAGN--K
XP_021739137.1/1-284 --D-AP-LLPSGFRI-IPIDSSK------------DVSSPKCTLDLTSALEVGAAGN--K
XP_021737291.1/1-284 --D-AP-LLPSGFRI-IPIDSSK------------DVSSPKCTLDLTSALEVGAAGN--K
XP_010047286.1/1-285 --D-AP-IIPSGFRI-IPLDPGS------------DASSPNRTLDLASALDVGPTGN--K
XP_003603630.2/1-285 --D-AP-ILPSGFRI-IPLESGT------------DAASPNRTLDLASALEVGTTGN--K
XP_004501146.1/1-286 --D-AP-ILSSGFRI-IPLDSCT------------DAASPNRTLDLASALEVGSAGN--K
XP_012571693.1/1-286 --D-AP-ILSSGFRI-IPLDSCT------------DAASPNRTLDLASALEVGSAGN--K
XP_012571692.1/1-286 --D-AP-ILSSGFRI-IPLDSCT------------DAASPNRTLDLASALEVGSAGN--K
XP_019417530.1/1-285 --D-AP-ILSSGFRI-IPLDSGT------------DAVSPNRTLDLASALDVGTTGN--K
XP_019417531.1/1-285 --D-AP-ILSSGFRI-IPLDSGT------------DAVSPNRTLDLASALDVGTTGN--K
XP_016180268.1/1-286 --D-AP-ILPSGFRI-IPLDSTS------------DAASPNRTLDLASALEVGTAGS--K
XP_025623723.1/1-286 --D-AP-ILPSGFRI-IPLDSTS------------DAASPNRTLDLASALEVGTAGS--K
XP_015945672.1/1-286 --D-AP-ILPSGFRI-IPLDSTS------------DAASPNRTLDLASALEVGTAGS--K
XP_007137231.1/1-287 --D-AP-ILPSGFRI-IPLDSSS------------DAASPNRTLDLASALEVGATAN--K
XP_022636494.1/1-293 --D-AP-ILPSGFRI-IPLDSSS------------DAASPNRTLDLASALEVGTTAN--K
XP_017409217.1/1-287 --D-AP-ILPSGFRI-IPLDSSS------------DAASPNRTLDLASALEVGTTAN--K
XP_014501941.1/1-287 --D-AP-ILPSGFRI-IPLDSSS------------DAASPNRTLDLASALEVGTTAN--K
XP_003526496.1/1-286 --D-AP-ILPSGFRI-IPLDSGT------------DAASPNRTLDLASALEVGTTAN--K
XP_003522716.1/1-286 --D-AP-ILPSGFRI-IPLDSGT------------DAASPNRTLDLASALEVGTTAN--K
XP_020217689.1/1-286 --D-AP-ILPSGFRI-IPLDSST------------GAASPNRTLDLASALEVGTTAN--K
XP_016683069.1/1-283 --D-SP-IIPSGFHI-IPLDSGM------------DASSPNRTLDLASTLEVGAAGN--R
XP_016677926.1/1-283 --D-AP-IIPSGFRI-IPLDSGM------------DASSPNRTLDLASTLEVGVAGN--R
XP_017643110.1/1-283 --D-AP-IIPCGFRI-IPLDSGM------------DASSPNRTLDLASTLEVGAAGN--R
XP_017643111.1/1-283 --D-AP-IIPCGFRI-IPLDSGM------------DASSPNRTLDLASTLEVGAAGN--R
XP_012451775.1/1-283 --D-SP-IIPSGFRI-IPLDSGM------------DASSPNRTLDLASTLEVGAAGN--R
XP_016744766.1/1-285 --D-AL-IIPSGFRI-IPLDSGT------------DASSPSRTLDLASTLEVGTAGN--Q
XP_017643921.1/1-285 --D-AL-IIPSGFRI-IPLDSGT------------DASSPSRTLDLASTLEVGTAGN--R
XP_012436119.1/1-285 --D-AL-IIPSGFRI-IPLDSGT------------DASSPSRTLDLASTLEVGTAGN--Q
XP_016681632.1/1-285 --D-AL-IIPSGFRI-IPLDSGT------------DASSPSRTLDLASTLEVGTAGN--Q
XP_016689618.1/1-285 --D-AP-IIPSGFRI-IPLDSGM------------DASSPNRTLDLASTLEVGAAGN--R
XP_016689617.1/1-285 --D-AP-IIPSGFRI-IPLDSGM------------DASSPNRTLDLASTLEVGAAGN--R
XP_012445947.1/1-285 --D-AP-IIPSGFRI-IPLDSGM------------DASSPNRTLDLASTLEVGAAGN--R
XP_012445944.1/1-285 --D-AP-IIPSGFRI-IPLDSGM------------DASSPNRTLDLASTLEVGAAGN--R
XP_012445945.1/1-285 --D-AP-IIPSGFRI-IPLDSGM------------DASSPNRTLDLASTLEVGAAGN--R
XP_017614652.1/1-285 --D-AP-IIPSGFRI-IPLDSGM------------DASSPNRTLDLASTLEVGAAGN--R
XP_016732732.1/1-285 --D-AP-IIPSGFRI-IPLDSGM------------DASSPNRTLDLASTLEVGAAGN--R
XP_016732731.1/1-285 --D-AP-IIPSGFRI-IPLDSGM------------DASSPNRTLDLASTLEVGAAGN--R
XP_022754596.1/1-285 --N-AA-IIPSGFRI-IPLDSGM------------DASSPNRTLDLASTLEVGATRN--R
XP_022750159.1/1-285 --D-AP-IIPSGFRI-IPLDSGM------------DASTPNRTLDLASTLEVGATGN--I
XP_022729737.1/1-285 --D-AP-IIPSGFRI-IPLDSGM------------DASGPNRTLDLASTLEVGASGN--R
XP_022729738.1/1-285 --D-AP-IIPSGFRI-IPLDSGM------------DASGPNRTLDLASTLEVGASGN--R
XP_021276132.1/1-285 --D-AP-IIPSGFRI-IPLDSGM------------DASSPNRTLDLASTLEVGAAGN--R
XP_017983822.1/1-285 --D-AP-IIPSGFRI-IPLDSGM------------DASSPNRTLDLASTLEVGAAGN--R
XP_002283717.3/1-285 --D-AP-LLPSGFRI-IPLDSGV------------DGSSPNRTLDLASSLEVGPAGN--K
XP_024446213.1/1-284 --D-AP-IIPSGFRI-IPLDSGM------------DASSPNRTLDLASALEVGPAGN--R
XP_024446215.1/1-284 --D-AP-IIPSGFRI-IPLDSGM------------DASSPNRTLDLASALEVGPAGN--R
XP_011006428.1/1-284 --D-AP-IIPSGFRI-IPLDSGM------------DASSPNRTLDLASSLEVGPAGN--R
XP_024459734.1/1-284 --D-AP-IIPSGFRI-IPLDSGM------------DASSPNRTLDLASALEVGPAGN--R
XP_011019595.1/1-284 --D-AP-IIPSGFRI-IPLDSGL------------DASSPNRTLDLASALEVGPAGN--R
XP_015573174.1/1-284 --D-AP-IIPSGFRI-IPLDSKM------------DVTSPNRTLDLASALEVGPAGN--K
XP_012077376.1/1-284 --D-AP-IIPSGFRI-IPLDSRV------------DASSPNRTLDLASALEIGPSGN--K
XP_021608403.1/1-284 --D-AP-IIPSGFRI-IPLDSRM------------DASSPNRTLDLASALDVGPTGN--K
XP_021608402.1/1-284 --D-AP-IIPSGFRI-IPLDSRM------------DASSPNRTLDLASALDVGPTGN--K
XP_021597909.1/1-284 --D-AP-IIPSGFRI-IPLDSRM------------DATSPNRTLDLTSALDVGPTGN--K
XP_021642839.1/1-284 --D-AP-IIPSGFRI-IPLDSRM------------DATSPNRTLDLASALDVGPAGN--K
XP_021689146.1/1-284 --D-AP-IIPSGFRI-IPLDSRM------------DAASPNRTLDLASALDVGPTGN--K
XP_018805752.1/1-285 --D-AP-IIPSGFRI-IPIDSGI------------DSSSPNRTLDLASALEVGPTGN--K
XP_023918167.1/1-285 --D-AP-IIPSGFRI-IPLDSGI------------DSSSPNRTLDLASALEVGPTGN--R
XP_018845499.1/1-285 --D-VP-IIPSGFRI-IPIESGI------------DASSPNRTLDLASALEVGPTGN--R
XP_018845500.1/1-285 --D-VP-IIPSGFRI-IPIESGI------------DASSPNRTLDLASALEVGPTGN--R
NP_001280750.1/1-285 --D-AP-ILPSGFRI-IPLDSRM------------DTPSPNRTLDLASALEVGPAGS--R
NP_001280796.1/1-285 --D-AP-ILPSGFRI-IPLDSRM------------DTPSPNRTLDLASALEVGPAGS--R
XP_009367440.1/1-285 --D-AP-ILPSGFRI-IPLDSRM------------DTPSPNRTLDLASALEVGPAGS--R
XP_017182108.1/1-285 --D-AP-ILPSGFRI-IPLDSRM------------DTPSPNRTLDLASALEVGPAGS--R
XP_004291167.1/1-285 --D-AP-ILPSGFRI-IPLDSHM------------DAPSPNRTLDLASALEVGPTGN--R
XP_024162655.1/1-285 --D-AP-ILPSGFRI-IPLDSHM------------DAPSPNRTLDLASALEIGPTGN--R
XP_021819449.1/1-284 --D-GP-ILPSGFRI-IPLDSRM------------DAPSPNRTLDLASALEVGPAGS--R
XP_007225281.1/1-285 --D-GP-ILPSGFRI-IPLDSRM------------DAPSPNRTLDLASALEVGPAGS--R
XP_008220092.1/1-285 --D-GP-ILPSGFRI-IPLDSRM------------DAPSPNRTLDLASALEVGPAGS--R
XP_006450706.1/1-285 --D-AP-IIPSGFRI-IPLDSGK------------DTPSPNRTLDLASALEVGPTGN--K
XP_006476026.1/1-285 --D-AP-IIPSGFRI-IPLDSGK------------DTPSPNRTLDLASALEVGPTGN--K
XP_024930942.1/1-285 --D-AP-ILPSGFRI-IPLDSGM------------DTPSPNRTLDLASALEVGPTGN--R
XP_010102406.2/1-285 --D-AP-ILPSGFRI-IPLDSGV------------DAPSPNRTLDLASALEVGHTGN--R
XP_024540686.1/1-258 --D-MP-LLSSGFRV-LPLCDDMD----------DIV---KRQ-------SDSEELR--S
XP_024399220.1/1-283 --D-IP-LLPSGFRV-IPVDSSVV-----------DGIGLNRTLDLASTLEDH-EAG--L
XP_024399219.1/1-283 --D-IP-LLPSGFRV-IPVDSSVV-----------DGIGLNRTLDLASTLEDH-EAG--L
XP_024399215.1/1-283 --D-IP-LLPSGFRV-IPVDSSVV-----------DGIGLNRTLDLASTLEDH-EAG--L
XP_024390746.1/1-284 --D-IP-LLPSGFCV-SPIDTNVV-----------DGFGLDRTLDLASTLEGGNDLR--L
XP_024390752.1/1-284 --D-IP-LLPSGFCV-SPIDTNVV-----------DGFGLDRTLDLASTLEGGNDLR--L
XP_024373354.1/1-283 --G-IP-LLPSGFCV-SPIDTNVV-----------DGFGLDRTLDLASTLEGGNDLR--L
XP_024373357.1/1-283 --G-IP-LLPSGFCV-SPIDTNVV-----------DGFGLDRTLDLASTLEGGNDLR--L
XP_024370817.1/1-284 --D-IP-LLPSGFCV-SPIDASVV-----------GGFDLDRTLDLASTLEGGSDLR--L
XP_024370815.1/1-284 --D-IP-LLPSGFCV-SPIDASVV-----------GGFDLDRTLDLASTLEGGSDLR--L
XP_024370814.1/1-284 --D-IP-LLPSGFCV-SPIDASVV-----------GGFDLDRTLDLASTLEGGSDLR--L
XP_024386785.1/1-285 --D-IP-LLPSGFCI-SPVDANVL-----------DEFDLDHTLDLASTLEGGSDLR--L
XP_024386782.1/1-285 --D-IP-LLPSGFCI-SPVDANVL-----------DEFDLDHTLDLASTLEGGSDLR--L
XP_024386791.1/1-285 --D-IP-LLPSGFCI-SPVDANVL-----------DEFDLDHTLDLASTLEGGSDLR--L
XP_002961227.1/1-277 --D-VP-LLPSGFRV-IPLDSGVD----------SS--GLSRTLDLASSLEGGADIG--K
XP_002969458.1/1-277 --D-VP-LLPSGFRV-IPLDSGVD----------SS--GLSRTLDLASSLEGGADIG--K
XP_024533017.1/1-265 --D-IP-LLPSGFRV-IPLDNGLD----------AS--ALSRTLDLTSTLEGSADGG--K
XP_011076983.1/1-261 --D-AV-LLSSGFRI-ISLPSNA-------S----------------S-ANMNSTGP--S
XP_011076980.1/1-264 --D-AV-LLSSGFRI-ISLPSNAGLQ----S----------------S-ANMNSTGP--S
XP_011076981.1/1-263 --D-AV-LLSSGFRI-ISLPSNAGLQ----S----------------S-ANMNSTGP--S
XP_011084330.1/1-264 --D-AA-LLCSGFRI-FLLGSNTGED----T----------------GSSNIAPNEA--L
XP_011084333.1/1-264 --D-AA-LLCSGFRI-FLLGSNTGED----T----------------GSSNIAPNEA--L
XP_011084331.1/1-264 --D-AA-LLCSGFRI-FLLGSNTGED----T----------------GSSNIAPNEA--L
XP_012834933.1/1-260 --D-AV-LFASGFRI-YLLGSHTDTN----S----------------S-TDLTSNEA--S
XP_012834932.1/1-260 --D-AV-LFASGFRI-YLLGSHTDTN----S----------------S-TDLTSNEA--S
XP_012834931.1/1-260 --D-AV-LFASGFRI-YLLGSHTDTN----S----------------S-TDLTSNEA--S
XP_012834934.1/1-260 --D-AV-LFASGFRI-YLLGSHTDTN----S----------------S-TDLTSNEA--S
XP_002274194.1/1-284 --D-AP-ILSSGFRI-LELDAKTCD-------R-QDMLAAKRMMNLASNLEVRSSDA--T
XP_019079956.1/1-284 --D-AP-ILSSGFRI-LELDAKTCD-------R-QDMLAAKRMMNLASNLEVRSSDA--T
XP_023759583.1/1-283 --D-AP-LLPSGFRI-IPINNKLTQ---------------NPTRDLASTLEVGVGPP--R
XP_020087984.1/1-275 --D-AP-LLPSGFRI-IPIDSIMDT-----------CSPSNRTLDLASTLEVGPTGT--R
XP_015693848.1/1-289 --D-SP-LLPSGFRI-IPIDSPLDT-----------SSP-NCTLDLASTLEAATPRS--R
XP_015624690.1/1-290 --D-SP-LLPSGFRI-IPIDSPLDT-----------SSP-NCTLDLASTLEAATPRS--R
XP_008655743.1/1-292 --D-SP-LLPSGFRI-IPIDAPLDT-----------SSP-KCTLDLASTLEAGTPRS--R
XP_020183693.1/1-281 --D-SP-LLPSGFRI-IPMESPLDT-----------SSP-NCTLDLASTLEVGTPGS--R
XP_020183687.1/1-281 --D-SP-LLPSGFRI-IPMESPLDT-----------SSP-NCTLDLASTLEVGTPGS--R
XP_003565084.1/1-284 --D-SP-LLPSGFRI-IPIDSPLDT-----------SSP-NCTLDLASTLEVGTPRG--R
XP_002454995.1/1-286 --D-SP-LLPSGFRI-IPIDAPLDT-----------SSP-KCTLDLASTLEVGTPRS--R
XP_012701181.1/1-286 --D-SP-LLPSGFRI-IPIDSPLDT-----------SSP-NCTLDLASTLEVGTPRS--R
XP_012701180.1/1-286 --D-SP-LLPSGFRI-IPIDSPLDT-----------SSP-NCTLDLASTLEVGTPRS--R
XP_012701179.1/1-286 --D-SP-LLPSGFRI-IPIDSPLDT-----------SSP-NCTLDLASTLEVGTPRS--R
XP_025816436.1/1-287 --D-SP-LLPSGFRI-IPIDSPLDT-----------PSP-KCTLDLASTLEVGTPRR--R
XP_018726848.1/1-227 P-DYVG-LLPSGFAI-LPDGSAMQGG------------N---------------------
XP_018726849.1/1-227 P-DYVG-LLPSGFAI-LPDGSAMQGG------------N---------------------
XP_019193052.1/1-216 P-DYVG-LLPSGFAI-LPDGA---------------------------------------
XP_019193050.1/1-216 P-DYVG-LLPSGFAI-LPDGA---------------------------------------
XP_021834047.1/1-194 P-DYVA-LLPSGFAI-LPDGG---------------------------------------
XP_010522330.1/1-229 P-DYVA-LLPSGFAI-LPDGTSPHPA------------P-AGA------------S----
XP_009802524.1/1-219 P-EYVA-LLPSGFAI-LPDGPPGRI-----------------------------------
XP_009802526.1/1-219 P-EYVA-LLPSGFAI-LPDGPPGRI-----------------------------------
XP_009802525.1/1-219 P-EYVA-LLPSGFAI-LPDGPPGRI-----------------------------------
XP_009802527.1/1-219 P-EYVA-LLPSGFAI-LPDGPPGRI-----------------------------------
XP_019250148.1/1-219 P-DYVA-LLPSGFAI-LPDGPPGRI-----------------------------------
XP_019250149.1/1-219 P-DYVA-LLPSGFAI-LPDGPPGRI-----------------------------------
XP_016449923.1/1-219 P-DYVA-LLPSGFAI-LPDGPPGRI-----------------------------------
XP_009629760.1/1-219 P-DYVA-LLPSGFAI-LPDGPPGRI-----------------------------------
XP_009629762.1/1-219 P-DYVA-LLPSGFAI-LPDGPPGRI-----------------------------------
XP_010234287.2/1-230 P-DYVA-LLPSGFAI-LPDGTPPDSG------------G---------------------
XP_021806177.1/1-204 P-DYVA-LLPSGFAI-LPDE----------------------------------------
XP_016580642.1/1-229 P-DFVA-LLPSGFAI-LPDGPPAGN-----------------------------------
XP_008667763.1/1-266 P-DYVA-LLPSGFAI-LPDGPSGSSN------------MQ--------------------
XP_006352040.1/1-227 P-DYVA-LLPSGFAI-LPDGPSGGR-----------------------------------
XP_009611638.1/1-223 P-DFVA-LLPSGFAI-LPDGPPGNS-----------------------------------
XP_009611623.1/1-223 P-DFVA-LLPSGFAI-LPDGPPGNS-----------------------------------
XP_016510654.1/1-223 P-DFVA-LLPSGFAI-LPDGPPGNS-----------------------------------
XP_009766038.1/1-223 P-DFVA-LLPSGFAI-LPDGPPGNS-----------------------------------
XP_020210648.1/1-236 P-DYVA-LLPSGFAV-LPDGSGMKNG------------PG--------------------
XP_007143210.1/1-219 P-DCVA-LLPSGFAV-LPDGPEPM------------------------------------
XP_014491869.1/1-226 P-DCVA-LLPSGFAV-LPDGPEPMNN------------GG--------------------
XP_022633652.1/1-226 P-DCVA-LLPSGFAV-LPDGPEPMNN------------GG--------------------
XP_017412341.1/1-227 P-DCVA-LLPSGFAV-LPDGPEPMNN------------EG--------------------
XP_022011155.1/1-226 P-DYVA-LLPSGFAI-LPDGPGRYE-----------------------------------
XP_003555330.2/1-228 P-DYVA-LLPSGFAV-LPDGPGLNGG------------PG--------------------
XP_014628518.1/1-229 P-DYVA-LLPSGFAV-LPDGPGLNGG------------PG--------------------
XP_017647009.1/1-225 P-DYVA-LLPSGFAI-LPDR--EGPN------------RG--------------------
XP_016711277.1/1-225 P-DYVA-LLPSGFAI-LPDR--EGPN------------RG--------------------
XP_012457027.1/1-225 P-DYVA-LLPSGFAI-LPDC--EGPN------------RG--------------------
XP_016723454.1/1-225 P-DYVS-LLPSGFAI-LPDC--EGPN------------RG--------------------
XP_020978973.1/1-214 P-DYLA-LLPSGFAI-LPDGLA--------------------------------------
XP_016202355.1/1-214 P-DYLA-LLPSGFAI-LPDGLA--------------------------------------
XP_015964876.1/1-214 P-DYLA-LLPSGFAI-LPDGLA--------------------------------------
XP_025699645.1/1-214 P-DYLA-LLPSGFAI-LPDGLA--------------------------------------
XP_008441375.1/1-228 P-DYVA-LLPSGFAI-LPDGPPIGPD------------GP--------------------
XP_004154226.2/1-228 P-DYVA-LLPSGFAI-LPDGPPIGPE------------GP--------------------
XP_023519981.1/1-226 P-DYVA-LLPSGFAI-LPDGP-SGPD------------AP--------------------
XP_023001231.1/1-226 P-DYVA-LLPSGFAI-LPDGP-SGPD------------AP--------------------
XP_022933178.1/1-226 P-DYVA-LLPSGFAI-LPDGP-SGPD------------AP--------------------
XP_004511340.1/1-228 S-NCVA-LLPSGFAI-VPDGSGPNGG----------------------------------
XP_002979015.2/1-232 P-AYVA-LLPSGFAI-LPDGQDHC-----------------G------------SL----
XP_002988402.1/1-232 P-AYVA-LLPSGFAI-LPDGQDHC-----------------G------------SL----
XP_024390340.1/1-238 P-AYVA-LLPSGFAI-LPDGPGGGER------------SGCGG-----------EQ----
XP_024368074.1/1-235 P-AYVA-LLPSGFAI-LPDGPGGGDR------------VYMG------------EQ----
XP_024401280.1/1-235 P-AYVA-LLPSGFAI-LPDGPGGDRG------------ALGN------------EQ----
XP_024394840.1/1-235 P-AYVA-LLPSGFAI-LPDGPGGERG------------SLGV------------DQ----
XP_024394838.1/1-235 P-AYVA-LLPSGFAI-LPDGPGGERG------------SLGV------------DQ----
XP_017256650.1/1-227 P-DYVA-LLPSGFAI-LPDGSPMQSH------------E---------------------
XP_019186546.1/1-227 P-DYVA-LLPSGFAI-LPDGPPGNGG----------------------------------
XP_024988375.1/1-226 P-DCFA-LLPSGFAI-LPDGPGQYEG----------------------------------
XP_017231769.1/1-226 P-DYVA-LLPSGFAI-LPDGPVQNV-----------------------------------
XP_009108529.1/1-235 P-DYVA-LLPSGFAI-LPDGSTR-GG------------NASVV-----------------
XP_018469534.1/1-236 P-EYVV-LFPSGFAI-LPDGSTREGG------------NASAV-----------------
XP_018467466.1/1-236 P-EYVV-LFPSGFAI-LPDGSTREGG------------NASAV-----------------
XP_022543253.1/1-234 P-DYVA-LLPSGFAI-LPDGG--EGG------------NASAG-----------------
XP_009135181.1/1-234 P-DYVA-LLPSGFAI-LPDGG--EGG------------NASAG-----------------
XP_013642975.1/1-234 P-DYVA-LLPSGFAI-LPDGG--EGG------------NASAG-----------------
XP_009135189.1/1-234 P-DYVA-LLPSGFAI-LPDGG--EGG------------NASAG-----------------
XP_018515487.1/1-234 P-DYVA-LLPSGFAI-LPDGG--EGG------------NASAG-----------------
XP_013623861.1/1-234 P-DYVA-LLPSGFAI-LPDGG--EGG------------DASAG-----------------
XP_013623905.1/1-234 P-DYVA-LLPSGFAI-LPDGG--EGG------------DASAG-----------------
XP_013623953.1/1-236 P-DYVA-LLPSGFAI-LPDGG--EGG------------DASAG-----------------
XP_013623587.1/1-236 P-DYVA-LLPSGFAI-LPDGG--EGG------------DASAG-----------------
XP_013623995.1/1-236 P-DYVA-LLPSGFAI-LPDGG--EGG------------DASAG-----------------
XP_013623820.1/1-236 P-DYVA-LLPSGFAI-LPDGG--EGG------------DASAG-----------------
XP_022926381.1/1-227 P-DYVA-LLPSGFAI-LPDGPA--------------------------------------
XP_023517485.1/1-227 P-DYVA-LLPSGFAI-LPDGPA--------------------------------------
XP_023002930.1/1-227 P-DYVA-LLPSGFAI-LPDGPA--------------------------------------
XP_004134971.1/1-229 P-DYVA-LLPSGFAI-LPDGPGGG------------------------------------
XP_008439968.1/1-229 P-DYVA-LLPSGFAI-LPDGPGGG------------------------------------
XP_022950176.1/1-227 P-DYVA-LLPSGFAI-LPDGPAG-------------------------------------
XP_022979090.1/1-227 P-DYVA-LLPSGFAI-LPDGPAG-------------------------------------
XP_023543388.1/1-249 P-DYVA-LLPSGFAI-LPDGPAGANG------------GEILELGSGFAILPDGPA----
XP_023543387.1/1-227 P-DYVA-LLPSGFAI-LPDGPAG-------------------------------------
XP_009134323.1/1-228 P-DYVA-LLPSGFAI-LPDGSVGGGG------------DGNQEVVSS-------------
XP_019176002.1/1-227 P-DYVA-LLPSGFAI-LPDGPSSNGA------------VN--------------------
XP_015070655.1/1-221 P-DYVA-LLPSGFAI-LPDGGGGIN-----------------------------------
XP_015070652.1/1-221 P-DYVA-LLPSGFAI-LPDGGGGIN-----------------------------------
XP_025885589.1/1-221 P-DYVA-LLPSGFAI-LPDGGGGIN-----------------------------------
XP_019422236.1/1-227 P-DYVA-LLPSGFAI-LPDGPGLNNG------------GGI-------------------
XP_007157166.1/1-226 P-DYVA-LLPSGFAI-LPDGP-GINR------------GSI-------------------
XP_003538765.1/1-227 P-DYVA-LLPSGFAI-LPDGPPALNG------------GPM-------------------
XP_003517589.1/1-227 P-DYVA-LLPSGFAI-LPDGPPALNG------------GPI-------------------
XP_006573890.1/1-227 P-DYVA-LLPSGFAI-LPDGPPALNG------------GPI-------------------
XP_011000687.1/1-226 P-DYVA-LLPSGFAI-LPDGPG-NGH------------AGI-------------------
XP_024436817.1/1-226 P-DYVA-LLPSGFAI-LPDGPG-YGP------------AGI-------------------
XP_024436820.1/1-226 P-DYVA-LLPSGFAI-LPDGPG-YGP------------AGI-------------------
XP_002518520.2/1-226 P-DYVA-LLPSGFAI-LPDGP-GFSP------------GII-------------------
XP_015574304.1/1-226 P-DYVA-LLPSGFAI-LPDGP-GFSP------------GII-------------------
XP_021642058.1/1-227 A-DYVA-LLPSGFAI-LPDGPAGFSP------------GGI-------------------
XP_012091395.1/1-226 P-DYVA-LLPSGFAI-LPDGPE-YNP------------GGS-------------------
XP_021635824.1/1-226 P-DYVA-LLPSGFAI-LPDGPG-FNP------------GGI-------------------
XP_021596887.1/1-226 P-DYVA-LLPSGFAI-LPDGQG-FNA------------GGI-------------------
XP_010057969.1/1-228 P-DYVA-LLPSGFAI-LPDGPEFGGG------------GGI-------------------
XP_006413738.1/1-245 P-DYFA-LLPSGFAI-LPDGSVREGG------------NASVGAEG--------GG----
XP_013630219.1/1-235 P-DYVA-LLPSGFAI-LPDGSTRGGN------------ASVVA-----------------
XP_013656136.1/1-235 P-DYVA-LLPSGFAI-LPDGSARGGN------------ASVVA-----------------
XP_013685757.1/1-235 P-DYVA-LLPSGFAI-LPDGSIRGGN------------ASVVA-----------------
XP_013696499.1/1-240 P-DYVA-LLPSGFAI-LPDGSSRVNA------------SA------------GAEG----
XP_009137213.1/1-240 P-DYVA-LLPSGFAI-LPDGSSRVNA------------SA------------GAEG----
XP_013598423.1/1-241 P-DYVA-LLPSGFAI-LPDGSSRVNA------------SA------------GAEG----
XP_013717184.1/1-241 P-DYVA-LLPSGFAI-LPDGSSRVNA------------SA------------GAEG----
XP_018480642.1/1-241 P-DYVA-LLPSGFAV-LPDGSSKANA------------SA------------GAEG----
XP_018477464.1/1-241 P-DYVA-LLPSGFAV-LPDGSSRVNA------------SA------------GAEG----
NP_193906.2/1-248    P-DYVA-LLPSGFAI-LPDGSARGGG------------GSANASAGA-----GVEG----
XP_020873301.1/1-248 P-DYVA-LLPSGFAI-LPDGSARGGG------------SAG-------GGTGAGVE----
XP_006285476.1/1-249 P-DYVA-LLPSGFAI-LPDGSARGGG------------GSASAGAGA----GAEGG----
XP_010434132.1/1-252 P-DYVA-LLPSGFAI-LPDGSARVGG------------GSASTGAGGAGTEGG--G----
XP_010439429.1/1-252 P-DYVA-LLPSGFAI-LPDGSARIGG------------GSASTGAGGAGAEGG--G----
XP_010449009.1/1-254 P-DYVA-LLPSGFAI-LPDGSARVGG------------GSASTGAGGSGAEGGGGG----
XP_010432053.1/1-234 P-DYVA-LLPSGFAI-LPDGSVGGGD------------GS-H------------------
XP_020876375.1/1-234 P-DYVA-LLPSGFAI-LPDGSVGGGD------------GN-H------------------
XP_023636874.1/1-234 P-DYVA-LLPSGFAI-LPDGSVGGGD------------GN-H------------------
XP_010422328.1/1-234 P-DYVA-LLPSGFAI-LPDGSVGGGD------------GN-H------------------
XP_010455798.1/1-234 P-DYVA-LLPSGFAI-LPDGSVGGGD------------GN-H------------------
NP_567274.1/1-235    P-DYVA-LLPSGFAI-LPDGSVGGGD------------GNQH------------------
XP_024009884.1/1-236 P-DYVA-LLPSGFAI-LPDGSVGGGT------------GDGN------------------
XP_018452346.1/1-236 P-DYVA-LLPSGFAI-LPDGSGGGGG------------GDGN------------------
XP_013611160.1/1-236 P-DYVA-LLPSGFAI-LPDGSVGGGG------------GDGN------------------
XP_009114536.1/1-235 P-DYVA-LLPSGFAI-LPDGSVGGGD------------GN-D------------------
XP_013662000.1/1-235 P-DYVA-LLPSGFAI-LPDGSVGGGD------------GN-D------------------
XP_018472664.1/1-233 P-DYVA-LLPSGFAI-LPDGSVGG-G------------DG-N------------------
XP_022555047.1/1-234 P-DYVA-LLPSGFAI-LPDGSVGGGG------------DG-N------------------
XP_013735469.1/1-234 P-DYVA-LLPSGFAI-LPDGSVGGGG------------DG-N------------------
XP_013629800.1/1-234 P-DYVA-LLPSGFAI-LPDGSVGGGG------------DG-N------------------
XP_013735464.1/1-234 P-DYVA-LLPSGFAI-LPDGSVGGGG------------DG-N------------------
XP_022571893.1/1-234 P-DYVA-LLPSGFAI-LPDGSVGGGG------------DG-N------------------
XP_010538642.1/1-236 P-DYVA-LLPSGFAI-LPDGSGGNGV------------GAGG------------------
XP_019057303.1/1-229 P-DYVA-LLPSGFAI-LPDGSGGGGE------------G---------------------
XP_016545426.1/1-226 P-DYVA-LLPSGFAI-LPDGSSNASS----------------------------------
XP_009793614.1/1-227 P-DYVA-LLPSGFAI-LPDGSTTTNG------------VGI-------------------
XP_016508926.1/1-227 P-DYVA-LLPSGFAI-LPDGSTTTNG------------VGI-------------------
XP_016560096.1/1-235 P-DYVA-LLPSGFAI-LPDGSTNHHG------------GSGS------------------
XP_021993721.1/1-227 P-DYVA-LLPSGFAI-LPDGAGKHQG------------GRI-------------------
XP_022036928.1/1-226 P-DYVA-LLPSGFAI-LPDGPGQYEG------------G-V-------------------
XP_023742870.1/1-226 P-DYVA-LLPSGFAI-LPDGPVHYEG------------G-V-------------------
XP_006352605.1/1-223 P-DYVA-LLPSGFAI-LPDGSSNAE-----------------------------------
XP_010327237.1/1-223 P-DYVA-LLPSGFAI-LPDGSSNAE-----------------------------------
XP_015055984.1/1-223 P-DYVA-LLPSGFAI-LPDGSSNAE-----------------------------------
XP_009609606.1/1-229 P-DYVA-LLPSGFAI-LPDGSATTNG------------VGI-------------------
XP_009609608.1/1-229 P-DYVA-LLPSGFAI-LPDGSATTNG------------VGI-------------------
XP_018628742.1/1-229 P-DYVA-LLPSGFAI-LPDGSATTNG------------VGI-------------------
XP_019237831.1/1-229 P-DYVA-LLPSGFAI-LPDGSTTTHG------------VGI-------------------
XP_018849836.1/1-227 P-DYVA-LLPSGFAI-LPDGPAG------------------------VNGG---------
XP_007214541.1/1-230 P-DYVA-LLPSGFAI-LPDGPTGPA---------------------GGGGG---------
XP_021821565.1/1-230 P-DYVA-LLPSGFAI-LPDGPTGPA---------------------GGGGG---------
XP_008224792.1/1-230 P-DYVA-LLPSGFAI-LPDGPTGPA---------------------GGGGG---------
XP_024198966.1/1-227 P-DYVA-LLPSGFAI-LPDGP---P---------------------AGGAG---------
XP_004293268.1/1-227 P-DYVA-LLPSGFAI-LPDGP---P---------------------AGGAG---------
XP_009364098.1/1-227 P-DYVA-LLPSGFAI-LPDGA---A---------------------GSAGG---------
XP_008371114.1/1-227 P-DYVA-LLPSGFAI-LPDGA---A---------------------GSAGG---------
XP_008384034.1/1-227 P-DYVA-LLPSGFAI-LPDGP---A---------------------GSAGG---------
XP_009340946.1/1-227 P-DYVA-LLPSGFAI-LPDGP---A---------------------GSAGG---------
XP_009357415.1/1-227 P-DYVA-LLPSGFAI-LPDGP---A---------------------GSAGG---------
XP_011037708.1/1-226 P-DYVA-LLPSGFAI-LPDGPGY-------------------------GPA---------
XP_024454819.1/1-226 P-DYVA-LLPSGFAI-LPDGPGY-------------------------GSA---------
XP_015884145.1/1-226 P-DYVA-LLPSGFAI-LPDGPGL-------------------------DAE---------
XP_024024693.1/1-228 P-DYVA-LLPSGFAI-LPDGPTNFN-----------------------AGG---------
XP_024024692.1/1-228 P-DYVA-LLPSGFAI-LPDGPTNFN-----------------------AGG---------
XP_018820351.1/1-226 P-DYVA-LLPSGFAI-LPDGPGL-------------------------NGG---------
XP_023904570.1/1-226 P-DYVA-LLPSGFAI-LPDGPGL-------------------------NGG---------
XP_023904569.1/1-226 P-DYVA-LLPSGFAI-LPDGPGL-------------------------NGG---------
XP_006449076.1/1-226 P-DYVA-LLPSGFAI-LPDGPGL-------------------------NGG---------
XP_006468235.1/1-226 P-DYVA-LLPSGFAI-LPDGPGL-------------------------NGG---------
XP_022721034.1/1-226 P-DYVA-LLPSGFAI-LPDGPGL-------------------------NGG---------
XP_022721035.1/1-226 P-DYVA-LLPSGFAI-LPDGPGL-------------------------NGG---------
XP_022724746.1/1-226 P-DYVA-LLPSGFAI-LPDGPGL-------------------------NSE---------
XP_007026002.1/1-226 P-DYVA-LLPSGFAI-LPDGPGL-------------------------NGG---------
XP_021294392.1/1-226 P-DYVA-LLPSGFAI-LPDGPGL-------------------------NGG---------
XP_022777405.1/1-226 P-DYVA-LLPSGFAI-LPDGPGL-------------------------NGG---------
XP_017615807.1/1-226 P-DYLA-LLPSGFAI-LPDGPGV-------------------------NGG---------
XP_016753649.1/1-226 P-DYLA-LLPSGFAI-LPDGPGV-------------------------NGG---------
XP_016683607.1/1-226 P-DYVA-LLPSGFAI-LPDGPGV-------------------------NGG---------
XP_012451363.1/1-226 P-DYVA-LLPSGFAI-LPDGPGV-------------------------NGG---------
XP_002266688.1/1-226 P-DYVA-LLPSGFAI-LPDGAV-LH----------------------GGGI---------
XP_021853797.1/1-224 P-DYVA-LLPSGFAI-LPDGPVG-------------------------GGG---------
XP_021719656.1/1-227 P-DYVA-LLPSGFAI-LPDGPVGAH----------------------GGGP---------
XP_021766927.1/1-227 P-DYVA-LLPSGFAI-LPDGPVGAH----------------------GGGP---------
XP_010665524.1/1-227 P-DYVA-LLPSGFAI-LPDGPVAGH----------------------GGGG---------
XP_009605864.1/1-234 P-DYVA-LLPSGFAI-LPDGSLTNH----------------------SGDN---------
XP_019223778.1/1-233 P-DYVA-LLPSGFAI-LPDGST-IH----------------------SGDN---------
XP_016440385.1/1-233 P-DYVA-LLPSGFAI-LPDGST-NH----------------------SGDN---------
XP_009790281.1/1-233 P-DYVA-LLPSGFAI-LPDGST-NH----------------------SGDN---------
XP_016570434.1/1-222 P-DYVA-LLPSGFAI-LPDGGGG-------------------------------------
XP_016486479.1/1-229 P-DYVA-LLPSGFAI-LPDGSTSSH----------------------CGGN---------
XP_009597904.1/1-229 P-DYVA-LLPSGFAI-LPDGSTSSH----------------------CGGN---------
XP_009799795.1/1-229 P-DYVA-LLPSGFAI-LPDGSTSGH----------------------CGGN---------
XP_019249652.1/1-229 P-DYVA-LLPSGFAI-LPDGSTSSH----------------------CGGN---------
XP_006343296.1/1-221 P-DYVA-LLPSGFAI-LPDGGG--------------------------------------
XP_020217012.1/1-226 P-DYVA-LLPSGFAI-LPDGPGLN-------------------------EG---------
XP_017406677.1/1-226 P-DYVA-LLPSGFAI-LPDGPGMN-------------------------GG---------
XP_014519998.1/1-226 P-DYVA-LLPSGFAI-LPDGPGMN-------------------------GG---------
XP_014519997.1/1-226 P-DYVA-LLPSGFAI-LPDGPGMN-------------------------GG---------
XP_012841999.1/1-239 P-DYVA-LLPSGFAI-LPDGPENNNN------------IIKNNNNNDEGGS---------
XP_024984347.1/1-227 P-DYVA-LLPSGFAI-LPDGPGKQQ------------------------GS---------
XP_023751202.1/1-227 P-DYVA-LLPSGFAI-LPDGPGKHQ------------------------GG---------
XP_023751208.1/1-227 P-DYVA-LLPSGFAI-LPDGPGKHQ------------------------GG---------
XP_022041933.1/1-227 P-DYVA-LLPSGFAI-LPDGGVKHQ------------------------GG---------
XP_024988632.1/1-226 P-DYVA-LLPSGFAI-LPDGPGQHQ------------------------G----------
XP_022018253.1/1-226 P-DYVA-LLPSGFAI-LPDGPVQYQ------------------------G----------
XP_023771293.1/1-226 P-DYVA-LLPSGFAI-LPDGPGQDQ------------------------G----------
XP_019170324.1/1-226 P-DYVA-LLPSGFAI-LPDGPPGNG-------------------------V---------
XP_017230122.1/1-226 P-DYVA-LLPSGFAI-LPDGPGQ-N------------------------VG---------
XP_011096101.1/1-226 P-DYVA-LLPSGFAI-LPDGPTNQA-------------------------G---------
XP_012848972.1/1-227 P-DYVA-LLPSGFAI-LPDGPTDPT------------------------VG---------
XP_022876975.1/1-227 P-DYVA-LLPSGFAI-LPDGPINPA------------------------GG---------
XP_022863158.1/1-227 P-DYVA-LLPSGFAI-LPDGPTNLA------------------------GG---------
XP_022877991.1/1-227 P-DYVA-LLPSGFAI-LPDGPINLA------------------------GG---------
XP_019223745.1/1-223 P-DFVA-LLPSGFAI-LPDGPPGNSR----------------------------------
XP_021800220.1/1-221 P-DYVA-LLPSGFAI-LPDGGGM-------------------------------------
XP_021684589.1/1-235 P-DYMA-LLPSGFAI-LPDGTT--TT------------HG---------G----------
XP_021684519.1/1-235 P-DYMA-LLPSGFAI-LPDGTT--TT------------HG---------G----------
XP_021684722.1/1-235 P-DYMA-LLPSGFAI-LPDGTT--TT------------HG---------G----------
XP_021633498.1/1-230 P-DYVA-LLPSGFAI-LPDGTTTNTV------------HG---------G----------
XP_009420270.1/1-216 P-DYVA-LLPSGFAI-LPDGAS--------------------------------------
XP_003573403.1/1-232 P-DYVA-LLPSGFAI-LPDGPSAPLS------------GINEEGG---------------
XP_006659894.1/1-229 P-DYVA-LLPSGFAI-LPDGPPSGNG------------QAVG------------------
XP_015650022.1/1-263 P-DYVA-LLPSGFAI-LPDGPSGNAQ------------AAVG------------------
XP_015650023.1/1-230 P-DYVA-LLPSGFAI-LPDGPSGNAQ------------AAVG------------------
XP_020407343.1/1-231 P-DYVA-LLPSGFAI-LPDGPPPAGA------------APSHGEGLDTG-----------
NP_001105127.2/1-231 P-DYVA-LLPSGFAI-LPDGPPPAGA------------APSHGEGLDTG-----------
XP_008661805.1/1-239 P-DYVA-LLPSGFAI-LPDGPPAPGM------------APHHGGEGAAGG----------
NP_001145750.1/1-239 P-DYVA-LLPSGFAI-LPDGPPAPGM------------APHHGGEGAAGG----------
XP_025821536.1/1-230 P-DYVA-LLPSGFAI-LPDGPPGAS--------------PH-------GE----------
XP_002443965.1/1-229 P-DYVA-LLPSGFAI-LPDGPPGMA--------------PH-------GE----------
XP_004972697.1/1-229 P-DYVA-LLPSGFAI-LPDGPPGAA--------------PH-------GE----------
XP_008667765.1/1-230 P-DYVA-LLPSGFAI-LPDGPSGSSN------------MQ-----GGGGG----------
NP_001347891.1/1-231 P-DYVA-LLPSGFAI-LPDGPSGSSN------------MQ-----GGGGG----------
XP_015636340.1/1-229 P-DYVA-LLPSGFAI-LPDGPAHDGG------------D------GDGGV----------
XP_015636338.1/1-229 P-DYVA-LLPSGFAI-LPDGPAHDGG------------D------GDGGV----------
XP_006652859.1/1-228 P-DYVA-LLPSGFAI-LPDGPGHGGG------------X------XXXGV----------
XP_003580598.1/1-233 P-DYVA-LLPSGFAI-LPDGPAGNIH------------TG--GGPSVSDG----------
XP_020199198.1/1-227 P-DYVA-LLPSGFAI-LPDGPAGT--------------------MHAAAG----------
XP_020400772.1/1-230 P-DYVA-LLPSGFAI-LPDGPSSGSS-----------------SMLQGDG----------
NP_001334663.1/1-230 P-DYVA-LLPSGFAI-LPDGPSSGSS-----------------SMLQGDG----------
XP_008662197.1/1-270 P-DYVA-LLPSGFAI-LPDGPSSGSS-----------------SMLQGDG----------
XP_008662196.1/1-270 P-DYVA-LLPSGFAI-LPDGPSSGSS-----------------SMLQGDG----------
XP_025823505.1/1-227 P-DYVA-LLPSGFAI-LPDGPARGN--------------------MQGEG----------
XP_004976894.1/1-227 P-DYVA-LLPSGFAI-LPDGPAGGN--------------------MQGDG----------
XP_002448563.2/1-227 P-DYVA-LLPSGFAI-LPDGPAGSN--------------------MQGDG----------
XP_019414154.1/1-227 P-DYVA-LLPSGFAI-LPDGSATHGN----------------------------------
XP_006348085.1/1-234 P-DYVA-LLPSGFAI-LPDGPMNHHG------------GG--------------------
XP_004232734.1/1-234 P-DYVA-LLPSGFAI-LPDGPMNYHG------------GG--------------------
XP_015066898.1/1-234 P-DYVA-LLPSGFAI-LPDGPINYHG------------GG--------------------
XP_003605900.1/1-232 P-DYVA-LLPSGFAI-LSDGNGNGVG------------GETG-------G----------
XP_022988693.1/1-229 P-DYVA-LLPSGFAI-LPDGTAASND------------GA--------------------
XP_022930609.1/1-229 P-DYVA-LLPSGFAI-LPDGTAASND------------GA--------------------
XP_023530197.1/1-229 P-DYVA-LLPSGFAI-LPDGTAASND------------GA--------------------
XP_016188415.1/1-237 P-DYVA-LLPSGFAI-LPDGSSGGCG------------T-GIGGETNGPT----------
XP_011628991.1/1-236 P-DYVA-LLPSGFAI-LPDGGLQPPS------------PSP---QSAPPA----------
XP_006826512.2/1-236 P-DYVA-LLPSGFAI-LPDGGLQPPS------------PSP---QSAPPA----------
XP_020177398.1/1-220 P-DYVA-LLPSGFAI-LPDGPAG-------------------------------------
XP_015695486.1/1-219 P-DYVA-LLPSGFAI-LPDGPA--------------------------------------
XP_015648539.1/1-217 P-DYVA-LLPSGFAI-LPDGPD--------------------------------------
XP_020401599.1/1-218 P-DYVA-LLPSGFAI-LPDG----------------------------------------
XP_020401598.1/1-218 P-DYVA-LLPSGFAI-LPDG----------------------------------------
XP_020401597.1/1-218 P-DYVA-LLPSGFAI-LPDG----------------------------------------
XP_021319954.1/1-239 P-DYVA-LLPSGFAI-LPDGSGAGGA------------PPGFAVLPDGPG----------
XP_002445014.2/1-239 P-DYVA-LLPSGFAI-LPDGSGAGGA------------PPGFAVLPDGPG----------
XP_025819913.1/1-218 P-DYVA-LLPSGFAI-LPDGPGA-------------------------------------
XP_004972909.1/1-218 P-DYVA-LLPSGFAI-LPDGPGA-------------------------------------
XP_020100432.1/1-229 P-DYVA-LLPSGFAI-LPDGPVAVPA------------GGA-------------------
XP_009385059.1/1-228 P-DYVA-LLPSGFAI-LPDGPCGGQG------------GE--------------------
XP_018678797.1/1-226 P-DYVA-LLPSGFAI-LPDGPCGSGG----------------------------------
XP_009412012.1/1-233 P-DYVA-LLPSGFSI-LPDGPTGGGP------------GGGRI------N----------
XP_018685149.1/1-236 P-DYVA-LLPSGFSI-LPDGPTGGGP------------GGGRI------N----------
XP_020591278.1/1-230 P-DYVA-LLPSGFAI-LPDGSNGVHG------------GGSGI------G----------
XP_020702932.1/1-229 P-DYVA-LLPSGFAI-LPDGSNGVQG------------GGSGI------G----------
XP_020702933.1/1-230 P-DYVA-LLPSGFAI-LPDGSNGVQG------------GGSGI------G----------
XP_020702931.1/1-230 P-DYVA-LLPSGFAI-LPDGSNGVQG------------GGSGI------G----------
XP_020692781.1/1-229 P-DYVA-LLPSGFAI-LPDGYGGVNG------------AGSGI------G----------
XP_020692780.1/1-229 P-DYVA-LLPSGFAI-LPDGYGGVNG------------AGSGI------G----------
XP_020591591.1/1-229 P-DYVA-LLPSGFAI-LPDGYDGVNG------------VGSGI------E----------
XP_020591590.1/1-229 P-DYVA-LLPSGFAI-LPDGYDGVNG------------VGSGI------E----------
XP_020109359.1/1-227 P-DCVA-LLPSGFAI-LPDGPSAV--------------PGGTT------V----------
XP_020270114.1/1-229 P-DYVA-LLPSGFAI-LPDGPTGSVP------------GGGPI------S----------
XP_010908855.1/1-227 P-DYVA-LLPSGFAI-LPDGPTGMP--------------GGGV------G----------
XP_008808987.1/1-227 P-DYVA-LLPSGFAI-LPDGPTGMQ--------------GGGV------G----------
XP_008784552.1/1-223 P-NYVA-LLPSGFAI-LPDGPTG------------------SI------G----------
XP_010931820.1/1-223 P-NYVA-LLPSGFAI-LPDGPTG------------------SI------G----------
XP_003540308.2/1-226 P-DYVA-LLPSGFAI-LPDGTTAHGG------------G---------------------
XP_006592818.1/1-226 P-DYVA-LLPSGFAI-LPDGTTAHGG------------G---------------------
XP_006592816.1/1-226 P-DYVA-LLPSGFAI-LPDGTTAHGG------------G---------------------
XP_003543387.1/1-226 P-DYVA-LLPSGFAI-LPDGTTAHGG------------G---------------------
XP_020223651.1/1-226 P-DYVA-LLPSGFAI-LPDGTTAHGG------------G---------------------
XP_020223648.1/1-226 P-DYVA-LLPSGFAI-LPDGTTAHGG------------G---------------------
XP_007149968.1/1-226 P-DYVA-LLPSGFAI-LPDGTTAHGG------------S---------------------
XP_017425582.1/1-226 P-DYVA-LLPSGFAI-LPDGTTAHGG------------S---------------------
XP_014501634.1/1-226 P-DYVA-LLPSGFAI-LPDGTTAHGG------------S---------------------
XP_022776632.1/1-226 P-DYVA-LLPSGFAI-LPDGTTSSTG------------G---------------------
XP_022776631.1/1-226 P-DYVA-LLPSGFAI-LPDGTTSSTG------------G---------------------
XP_022776629.1/1-226 P-DYVA-LLPSGFAI-LPDGTTSSTG------------G---------------------
XP_022733641.1/1-226 P-DYVA-LLPSGFAI-LPDGTTATAG------------G---------------------
XP_022733640.1/1-226 P-DYVA-LLPSGFAI-LPDGTTATAG------------G---------------------
XP_022733638.1/1-226 P-DYVA-LLPSGFAI-LPDGTTATAG------------G---------------------
XP_007045612.1/1-226 P-DYVA-LLPSGFAI-LPDGTTASAG------------G---------------------
XP_007045611.1/1-226 P-DYVA-LLPSGFAI-LPDGTTASAG------------G---------------------
XP_017971478.1/1-226 P-DYVA-LLPSGFAI-LPDGTTASAG------------G---------------------
XP_021292769.1/1-226 P-DYVA-LLPSGFAI-LPDGTPASAG------------G---------------------
XP_021292767.1/1-226 P-DYVA-LLPSGFAI-LPDGTPASAG------------G---------------------
XP_021292765.1/1-226 P-DYVA-LLPSGFAI-LPDGTPASAG------------G---------------------
XP_010657309.1/1-226 P-DYVA-LLPSGFAI-LPDGTTAHG---------------G-------------------
XP_010657313.1/1-226 P-DYVA-LLPSGFAI-LPDGTTAHG---------------G-------------------
XP_010657310.1/1-226 P-DYVA-LLPSGFAI-LPDGTTAHG---------------G-------------------
XP_010657311.1/1-226 P-DYVA-LLPSGFAI-LPDGTTAHG---------------G-------------------
XP_012086347.1/1-228 P-DYVA-LLPSGFAI-LPDGTTTHDG------------G-G-------------------
XP_012086344.1/1-228 P-DYVA-LLPSGFAI-LPDGTTTHDG------------G-G-------------------
XP_024029967.1/1-226 P-DYVA-LLPSGFAI-LPDGSAAHG-------------D-G-------------------
XP_010109517.1/1-226 P-DYVA-LLPSGFAI-LPDGSAAHG-------------D-G-------------------
XP_006469697.1/1-226 P-DYVA-LLPSGFAI-LPDGTSLHG-------------A-N-------------------
XP_024046029.1/1-226 P-DYVA-LLPSGFAI-LPDGTSLHG-------------A-N-------------------
XP_020234199.1/1-226 P-DYVA-LLPSGFAI-LPDGTTTHG-------------S-G-------------------
XP_020234201.1/1-226 P-DYVA-LLPSGFAI-LPDGTTTHG-------------S-G-------------------
XP_021630856.1/1-226 P-DYVA-LLPSGFAI-LPDGTTTHG-------------G-G-------------------
XP_021630853.1/1-226 P-DYVA-LLPSGFAI-LPDGTTTHG-------------G-G-------------------
XP_021907367.1/1-221 P-DYVA-LLPSGFAI-LPDGAGVNQ-----------------------------------
XP_010275996.1/1-221 P-DYVA-LLPSGFAI-LPDGAPVQS-----------------------------------
XP_020870784.1/1-223 P-DYVA-LLPSGFAI-LPDGNANSG-----------------------------------
NP_172015.1/1-223    P-DYVA-LLPSGFAI-LPDGNANSG-----------------------------------
XP_006418050.1/1-223 P-DYVA-LLPSGFAI-LPDGNANGG-----------------------------------
XP_018447753.1/1-223 P-DYVA-LLPSGFAI-LPDGNANGG-----------------------------------
XP_010457605.1/1-218 P-DYVA-LLPSGFAI-LPDGNANG------------------------------------
XP_023632765.1/1-218 P-DYVA-LLPSGFAI-LPDGNANG------------------------------------
XP_010484829.1/1-218 P-DYVA-LLPSGFAI-LPDGNANG------------------------------------
XP_010475208.1/1-218 P-DYVA-LLPSGFAI-LPDGNANG------------------------------------
XP_018487097.1/1-220 P-DYVA-LLPSGFAI-LPDGNTNG------------------------------------
XP_013664795.1/1-220 P-DYVA-LLPSGFAI-LPDGNANG------------------------------------
XP_013716253.1/1-220 P-DYVA-LLPSGFAI-LPDGNANG------------------------------------
XP_009118607.1/1-220 P-DYVA-LLPSGFAI-LPDGNANG------------------------------------
XP_013605632.1/1-220 P-DYVA-LLPSGFAI-LPDGNANG------------------------------------
XP_010475204.1/1-220 P-DYVA-LLPSGFAI-LPDGNANG------------------------------------
XP_010457604.1/1-220 P-DYVA-LLPSGFAI-LPDGNANG------------------------------------
XP_023632762.1/1-220 P-DYVA-LLPSGFAI-LPDGNANG------------------------------------
XP_010484823.1/1-220 P-DYVA-LLPSGFAI-LPDGNANG------------------------------------
XP_022558401.1/1-220 P-DYVA-LLPSGFAI-LPDGNANG------------------------------------
XP_013586641.1/1-220 P-DYVA-LLPSGFAI-LPDGNANG------------------------------------
XP_022558399.1/1-220 P-DYVA-LLPSGFAI-LPDGNANG------------------------------------
XP_022558400.1/1-220 P-DYVA-LLPSGFAI-LPDGNANG------------------------------------
XP_013586642.1/1-220 P-DYVA-LLPSGFAI-LPDGNANG------------------------------------
XP_013696832.1/1-220 P-DYVA-LLPSGFAI-LPDGNANG------------------------------------
XP_022548878.1/1-220 P-DYVA-LLPSGFAI-LPDGNATG------------------------------------
XP_013666552.1/1-220 P-DYVA-LLPSGFAI-LPDGNATG------------------------------------
XP_009119177.1/1-220 P-DYVA-LLPSGFAI-LPDGNATG------------------------------------
XP_020964430.1/1-225 P-DYVA-LLPSGFAI-LPDGTTPDGG------------SEA-------------------
XP_015936069.1/1-225 P-DYVA-LLPSGFAI-LPDGTTPDGG------------SEA-------------------
XP_020964429.1/1-225 P-DYVA-LLPSGFAI-LPDGTTPDGG------------SEA-------------------
XP_015936068.1/1-225 P-DYVA-LLPSGFAI-LPDGTTPDGG------------SEA-------------------
XP_015577880.1/1-228 P-DYVA-LLPSGFAI-LPDGTTAHGG------------GIG-------------------
XP_002524172.2/1-228 P-DYVA-LLPSGFAI-LPDGTTAHGG------------GIG-------------------
XP_007132557.1/1-221 P-DYVA-LLPSGFAI-LPDGTISHET------------G---------------------
XP_017433694.1/1-221 P-DYVA-LLPSGFAI-LPDGTISHET------------G---------------------
XP_014494419.1/1-221 P-DYVA-LLPSGFAI-LPDGTISHET------------G---------------------
XP_022634310.1/1-221 P-DYVA-LLPSGFAI-LPDGTISHET------------G---------------------
XP_010267735.1/1-225 P-DYVA-LLPSGFAI-LPDGSTVHAG----------------------------------
XP_010267734.1/1-225 P-DYVA-LLPSGFAI-LPDGSTVHAG----------------------------------
XP_024185199.1/1-224 P-DYVA-LLPSGFAI-LPDGTSG-----------------S-------------------
XP_024185197.1/1-224 P-DYVA-LLPSGFAI-LPDGTSG-----------------S-------------------
XP_009342246.1/1-222 P-DYVA-LLPSGFAI-LPDGAGI-----------------G-------------------
XP_009342244.1/1-222 P-DYVA-LLPSGFAI-LPDGAGI-----------------G-------------------
XP_009342240.1/1-222 P-DYVA-LLPSGFAI-LPDGAGI-----------------G-------------------
XP_020416503.1/1-221 P-DYVA-LLPSGFAI-LPDGGGM-----------------G-------------------
XP_021822854.1/1-221 P-DYVA-LLPSGFAI-LPDGGGM-----------------G-------------------
XP_007217584.1/1-221 P-DYVA-LLPSGFAI-LPDGGGM-----------------G-------------------
XP_021822852.1/1-221 P-DYVA-LLPSGFAI-LPDGGGM-----------------G-------------------
XP_018730806.1/1-228 P-DYVA-LLPSGFAI-LPDGSAMQGG------------ND--------------------
XP_010051064.1/1-228 P-DYVA-LLPSGFAI-LPDGSAMQGG------------ND--------------------
XP_015881130.2/1-229 P-DYVA-LLPSGFAI-LPDGTSIHGG------------GGG-------------------
XP_023534533.1/1-219 P-DYVA-LLPSGFAI-LPDGT---------------------------------------
XP_022947095.1/1-219 P-DYVA-LLPSGFAI-LPDGT---------------------------------------
XP_022947096.1/1-219 P-DYVA-LLPSGFAI-LPDGT---------------------------------------
XP_022970950.1/1-219 P-DYVA-LLPSGFAI-LPDGT---------------------------------------
XP_022970951.1/1-219 P-DYVA-LLPSGFAI-LPDGT---------------------------------------
XP_022140692.1/1-217 P-DYVA-LLPSGFAI-LPDGG---------------------------------------
XP_022140691.1/1-217 P-DYVA-LLPSGFAI-LPDGG---------------------------------------
XP_008455853.1/1-220 P-DYVA-LLPSGFAI-LPDGG---------------------------------------
XP_011650012.1/1-220 P-DYVA-LLPSGFAI-LPDGG---------------------------------------
XP_008455850.1/1-220 P-DYVA-LLPSGFAI-LPDGG---------------------------------------
XP_011650011.1/1-220 P-DYVA-LLPSGFAI-LPDGG---------------------------------------
XP_023513403.1/1-220 P-DYVA-LLPSGFAI-LPDGG---------------------------------------
XP_022944761.1/1-220 P-DYVA-LLPSGFAI-LPDGG---------------------------------------
XP_023513401.1/1-220 P-DYVA-LLPSGFAI-LPDGG---------------------------------------
XP_022944759.1/1-220 P-DYVA-LLPSGFAI-LPDGG---------------------------------------
XP_022986270.1/1-220 P-DYVA-LLPSGFAI-LPDGG---------------------------------------
XP_022986268.1/1-220 P-DYVA-LLPSGFAI-LPDGG---------------------------------------
XP_022136006.1/1-224 P-DYVA-LLPSGFAI-LPDGTAAA------------------G-----------------
XP_008455423.1/1-229 P-DYVA-LLPSGFAI-LPDGSTAS------------------S-G--GAN----------
XP_011658704.1/1-229 P-DYVA-LLPSGFAI-LPDGSTAS------------------S-G--GAN----------
XP_004144488.1/1-229 P-DYVA-LLPSGFAI-LPDGSTAS------------------S-G--GAN----------
XP_022968990.1/1-229 P-DYVA-LLPSGFAI-LPDGSTTS------------------D-G--GAN----------
XP_023554403.1/1-229 P-DYVA-LLPSGFAI-LPDGSTTS------------------D-G--GAN----------
XP_022952948.1/1-229 P-DYVA-LLPSGFAI-LPDGSTTS------------------D-G--GAN----------
XP_019447751.1/1-228 P-DYVA-LLPSGFAI-LPDGTTTHGS------------SIVE------------------
XP_019447749.1/1-228 P-DYVA-LLPSGFAI-LPDGTTTHGS------------SIVE------------------
XP_004515074.1/1-228 P-DYVA-LLPSGFAI-LSDGNG-------------------NG-I--GDE----------
XP_012463792.1/1-235 P-DYVA-LLPSGFAI-LPDGSTITAT------------TSSAG-G--GID----------
XP_012463790.1/1-235 P-DYVA-LLPSGFAI-LPDGSTITAT------------TSSAG-G--GID----------
XP_016678783.1/1-235 P-DYVA-LLPSGFAI-LPDGSTITAT------------TSSAG-G--GID----------
XP_016678782.1/1-235 P-DYVA-LLPSGFAI-LPDGSTITAT------------TSSAG-G--GID----------
XP_017631648.1/1-235 P-DYVA-LLPSGFAI-LPDGSTITAT------------TSSAG-G--GID----------
XP_016732503.1/1-235 P-DYVA-LLPSGFAI-LPDGSTITAT------------TSSAG-G--GID----------
XP_017631647.1/1-235 P-DYVA-LLPSGFAI-LPDGSTITAT------------TSSAG-G--GID----------
XP_012438430.1/1-229 P-DYVA-LLPSGFAI-LPDGSSGST-------------------G--SGM----------
XP_012438427.1/1-229 P-DYVA-LLPSGFAI-LPDGSSGST-------------------G--SGM----------
XP_012438429.1/1-229 P-DYVA-LLPSGFAI-LPDGSSGST-------------------G--SGM----------
XP_017616162.1/1-229 P-DYVA-LLPSGFAI-LPDGSSGSS-------------------G--SGV----------
XP_017616160.1/1-229 P-DYVA-LLPSGFAI-LPDGSSGSS-------------------G--SGV----------
XP_017616161.1/1-229 P-DYVA-LLPSGFAI-LPDGSSGSS-------------------G--SGV----------
XP_016726347.1/1-229 P-DYVA-LLPSGFAI-LPDGSSGSS-------------------G--SGV----------
XP_016726344.1/1-229 P-DYVA-LLPSGFAI-LPDGSSGSS-------------------G--SGV----------
XP_016726345.1/1-229 P-DYVA-LLPSGFAI-LPDGSSGSS-------------------G--SGV----------
XP_019448933.1/1-227 P-DYVA-LLPSGFAI-LPDGTTTHG----------------NG-----------------
XP_004487400.1/1-228 P-DYVA-LLPSGFAI-LPDGTTTTT----------------TN-G--SA-----------
XP_003596999.2/1-227 P-DYVA-LLPSGFAI-LPDGTTT-N----------------GG-G--VG-----------
XP_024631890.1/1-227 P-DYVA-LLPSGFAI-LPDGTTT-N----------------GG-G--VG-----------
XP_024631891.1/1-227 P-DYVA-LLPSGFAI-LPDGTTT-N----------------GG-G--VG-----------
XP_004287935.1/1-224 P-DYVA-LLPSGFAI-LPDGTSG-------------------------S-----------
XP_018808969.1/1-228 P-DYVA-LLPSGFAI-LPDGTSSTL-------------------G--AG-----------
XP_018808967.1/1-228 P-DYVA-LLPSGFAI-LPDGTSSTL-------------------G--AG-----------
XP_018817598.1/1-227 P-DYVA-LLPSGFAI-LPDGTS-DL-------------------G--GG-----------
XP_018817597.1/1-227 P-DYVA-LLPSGFAI-LPDGTS-DL-------------------G--GG-----------
XP_018817594.1/1-227 P-DYVA-LLPSGFAI-LPDGTS-DL-------------------G--GG-----------
XP_018817599.1/1-227 P-DYVA-LLPSGFAI-LPDGTS-DL-------------------G--GG-----------
XP_023915056.1/1-227 P-DYVA-LLPSGFAI-LPDGTTA-------------------H-E--GG-----------
XP_023915054.1/1-227 P-DYVA-LLPSGFAI-LPDGTTA-------------------H-E--GG-----------
XP_023915053.1/1-227 P-DYVA-LLPSGFAI-LPDGTTA-------------------H-E--GG-----------
XP_021685023.1/1-228 P-DYMA-LLPSGFAI-LPDGTTTT------------------H-G--GG-----------
XP_021684657.1/1-228 P-DYMA-LLPSGFAI-LPDGTTTT------------------H-G--GG-----------
XP_021685089.1/1-228 P-DYMA-LLPSGFAI-LPDGTTTT------------------H-G--GG-----------
XP_015383009.1/1-229 P-DYVA-LLPSGFAI-LPDGTSLH--------------------G--AN-----------
XP_015383007.1/1-229 P-DYVA-LLPSGFAI-LPDGTSLH--------------------G--AN-----------
XP_020539441.1/1-235 P-DYVA-LLPSGFAI-LPDGTTT------------------HD-G--GG-----------
XP_020539439.1/1-235 P-DYVA-LLPSGFAI-LPDGTTT------------------HD-G--GG-----------
XP_015577881.1/1-233 P-DYVA-LLPSGFAI-LPDGTTA------------------HG-G--GI-----------
XP_015577879.1/1-233 P-DYVA-LLPSGFAI-LPDGTTA------------------HG-G--GI-----------
XP_015577877.1/1-233 P-DYVA-LLPSGFAI-LPDGTTA------------------HG-G--GI-----------
XP_021630854.1/1-231 P-DYVA-LLPSGFAI-LPDGTT--T----------------HG-G--GI-----------
XP_021630849.1/1-231 P-DYVA-LLPSGFAI-LPDGTT--T----------------HG-G--GI-----------
XP_021652390.1/1-229 P-DYVA-LLPSGFAI-LPDGTTTIT----------------HG-G--GI-----------
XP_021652388.1/1-229 P-DYVA-LLPSGFAI-LPDGTTTIT----------------HG-G--GI-----------
XP_021652389.1/1-234 P-DYVA-LLPSGFAI-LPDGTTTIT----------------HG-G--GI-----------
XP_021652386.1/1-234 P-DYVA-LLPSGFAI-LPDGTTTIT----------------HG-G--GI-----------
XP_006592380.1/1-228 P-DYVA-LLPSGFAI-LPDGTTSHN----------------GS-G--G------------
XP_003539872.2/1-228 P-DYVA-LLPSGFAI-LPDGTTSHN----------------GS-G--G------------
XP_025984806.1/1-230 P-DYVA-LLPSGFAI-LPDGTTSHG----------------SG-G--GV-----------
XP_014632311.1/1-230 P-DYVA-LLPSGFAI-LPDGTTSHG----------------SG-G--GV-----------
XP_024450362.1/1-227 P-DYVA-LLPSGFAI-FPDGTAAHG----------------VG-----------------
XP_002302880.2/1-227 P-DYVA-LLPSGFAI-FPDGTAAHG----------------VG-----------------
XP_024450361.1/1-227 P-DYVA-LLPSGFAI-FPDGTAAHG----------------VG-----------------
XP_024450360.1/1-227 P-DYVA-LLPSGFAI-FPDGTAAHG----------------VG-----------------
XP_011011493.1/1-227 P-DYVA-LLPSGFAI-FPDGTAAHG----------------VG-----------------
XP_011011491.1/1-227 P-DYVA-LLPSGFAI-FPDGTAAHG----------------VG-----------------
XP_011011492.1/1-227 P-DYVA-LLPSGFAI-FPDGTAAHG----------------VG-----------------
XP_011011489.1/1-227 P-DYVA-LLPSGFAI-FPDGTAAHG----------------VG-----------------
XP_011011488.1/1-227 P-DYVA-LLPSGFAI-FPDGTAAHG----------------VG-----------------
XP_011005116.1/1-225 P-DYVA-LLPSGFAV-LPDGTAAHG----------------GG-----------------
XP_011005113.1/1-225 P-DYVA-LLPSGFAV-LPDGTAAHG----------------GG-----------------
XP_011005112.1/1-225 P-DYVA-LLPSGFAV-LPDGTAAHG----------------GG-----------------
XP_024440391.1/1-225 P-DYVA-LLPSGFAV-LPDGTGAHV----------------GG-----------------
XP_002320458.2/1-225 P-DYVA-LLPSGFAV-LPDGTGAHV----------------GG-----------------
XP_024440389.1/1-225 P-DYVA-LLPSGFAV-LPDGTGAHV----------------GG-----------------
XP_019453767.1/1-227 P-EYVA-LLPSGFAI-LPDGTTTHGS----------------------------------
XP_012478857.1/1-227 P-DYVA-LLPSGFAI-LPDGMTVTDV------------G---------MA----------
XP_012478855.1/1-227 P-DYVA-LLPSGFAI-LPDGMTVTDV------------G---------MA----------
XP_012478856.1/1-227 P-DYVA-LLPSGFAI-LPDGMTVTDV------------G---------MA----------
XP_012478851.1/1-227 P-DYVA-LLPSGFAI-LPDGMTVTDV------------G---------MA----------
XP_016724999.1/1-227 P-DYVA-LLPSGFAI-LPDGMTVTDV------------G---------MA----------
XP_017638204.1/1-227 P-DYVA-LLPSGFAI-LPDGMTVTDV------------G---------MA----------
XP_017638203.1/1-227 P-DYVA-LLPSGFAI-LPDGMTVTDV------------G---------MA----------
XP_016667113.1/1-227 P-DYVA-LLPSGFAI-LPDGMTVTDV------------G---------MA----------
XP_016667112.1/1-227 P-DYVA-LLPSGFAI-LPDGMTVTDV------------G---------MA----------
XP_015881131.2/1-227 P-DYVA-LLPSGFAI-LPDGTSIHGG------------G---------GG----------
XP_008348131.1/1-222 P-DYVA-LLPSGFAI-LPDGAGIGD-----------------------------------
XP_008348133.1/1-220 P-DYVA-LLPSGFAI-LPDGAGIGD-----------------------------------
XP_018502305.1/1-220 P-DYVA-LLPSGFAI-LPDGAGIGD-----------------------------------
XP_009342243.1/1-220 P-DYVA-LLPSGFAI-LPDGAGIGD-----------------------------------
XP_017179321.1/1-222 P-DYVA-LLPSGFAI-LPDGADIGD-----------------------------------
XP_017179320.1/1-222 P-DYVA-LLPSGFAI-LPDGADIGD-----------------------------------
XP_008341986.1/1-222 P-DYVA-LLPSGFAI-LPDGADIGD-----------------------------------
XP_017179319.1/1-220 P-DYVA-LLPSGFAI-LPDGADIGD-----------------------------------
XP_024185198.1/1-222 P-DYVA-LLPSGFAI-LPDGTSGSN-----------------------------------
XP_011463209.1/1-222 P-DYVA-LLPSGFAI-LPDGTSGSN-----------------------------------
XP_020416501.1/1-219 P-DYVA-LLPSGFAI-LPDGGG--------------------------------------
XP_021822853.1/1-219 P-DYVA-LLPSGFAI-LPDGGG--------------------------------------
XP_008230079.1/1-219 P-DYVA-LLPSGFAI-LPDGGG--------------------------------------
XP_020870786.1/1-221 P-DYVA-LLPSGFAI-LPDGNAN--S------------G---------A-----------
NP_001117231.1/1-221 P-DYVA-LLPSGFAI-LPDGNAN--S------------G---------A-----------
XP_018817596.1/1-225 P-DYVA-LLPSGFAI-LPDGTSD--L------------G---------GG----------
XP_019079350.1/1-224 P-DYVA-LLPSGFAI-LPDGTTA--H----------------------GG----------
XP_022733639.1/1-224 P-DYVA-LLPSGFAI-LPDGTTA--T----------------------AG----------
XP_021292768.1/1-224 P-DYVA-LLPSGFAI-LPDGTPA--S----------------------AG----------
XP_021292766.1/1-224 P-DYVA-LLPSGFAI-LPDGTPA--S----------------------AG----------
XP_017971480.1/1-224 P-DYVA-LLPSGFAI-LPDGTTA--S----------------------AG----------
XP_018721484.1/1-222 P-DYVA-LLPSGFSI-LPDGSQGS------------------------------------
XP_018721483.1/1-222 P-DYVA-LLPSGFSI-LPDGSQGS------------------------------------
XP_018721482.1/1-222 P-DYVA-LLPSGFSI-LPDGSQGS------------------------------------
XP_025687365.1/1-237 P-DYVA-LLPSGFAI-LPDGSSGGCG------------SGIGG-ETNGPT----------
XP_006851770.2/1-243 S-AYVA-LLPSGFAI-LPDGNNSNNN------------KDGGGGG---------------
XP_003623826.1/1-230 S-AYVA-LLPSGFAV-LPDGHSNGS-----------------------------------
XP_004492678.1/1-230 S-AYVA-LLPSGFAV-LPDGQCQES-----------------------------------
XP_004492677.1/1-230 S-AYVA-LLPSGFAV-LPDGQCQES-----------------------------------
XP_012847897.1/1-236 S-AYVA-LLPSGFSI-MPDGTGGGGP------------VSGDGSGG--------------
XP_012843673.1/1-229 S-AYVA-LLPSGFSI-MPDGTGGGGP------------VSGDGSGG--------------
XP_021279608.1/1-239 S-AYVA-LLPSGFAI-VPDGPGSRGP------------XSNGHVNG--------------
XP_025641241.1/1-241 S-AYVA-LLPSGFAI-VPDGPAPRGA------------QNGGADAN--------------
XP_025641246.1/1-241 S-AYVA-LLPSGFAI-VPDGPAPRGA------------QNGGADAN--------------
XP_020991942.1/1-241 S-AYVA-LLPSGFAI-VPDGPAPRGA------------QNGGADAN--------------
XP_025632115.1/1-241 S-AYVA-LLPSGFAI-VPDGPGPRGA------------QNGGAEAN--------------
XP_025632116.1/1-241 S-AYVA-LLPSGFAI-VPDGPGPRGA------------QNGGAEAN--------------
XP_017442222.1/1-246 S-AYVA-LLPSGFAI-VPDGPGSRGS------------QN-GP-----------------
XP_017442225.1/1-246 S-AYVA-LLPSGFAI-VPDGPGSRGS------------QN-GP-----------------
XP_006576359.1/1-244 S-AYVA-LLPSGFAI-VPDGPGSRGP------------HQNGPTSS--------------
XP_003521284.1/1-244 S-AYVA-LLPSGFAI-VPDGPGSRGP------------HQNGPTSS--------------
XP_024174849.1/1-237 S-AYVA-LLPSGFAI-VPDGPGSRGS---------------EAKAGQG------------
XP_024174854.1/1-237 S-AYVA-LLPSGFAI-VPDGPGSRGS---------------EAKAGQG------------
XP_004306832.1/1-241 S-AYVA-LLPSGFAI-VPDGPGSRGP------------GGAEGKAGQG------------
XP_011469062.1/1-241 S-AYVA-LLPSGFAI-VPDGPGSRGP------------GGAEGKAGQG------------
XP_009340632.2/1-243 S-AYVA-LLPSGFAI-VPDGPGSRGP------------MFGKGGS-HG------------
XP_008385703.1/1-243 S-AYVA-LLPSGFAI-VPDGPGSRGP------------MFGKGGS-HG------------
XP_008385704.1/1-243 S-AYVA-LLPSGFAI-VPDGPGSRGP------------MFGKGGS-HG------------
XP_008232741.1/1-243 S-AYVA-LLPSGFAI-VPDGPGSRGP------------MTVKGGG-HG------------
XP_008232742.1/1-243 S-AYVA-LLPSGFAI-VPDGPGSRGP------------MTVKGGG-HG------------
XP_007220256.1/1-243 S-AYVA-LLPSGFAI-VPDGPGSRGP------------MTVKGGG-HG------------
XP_020413144.1/1-243 S-AYVA-LLPSGFAI-VPDGPGSRGP------------MTVKGGG-HG------------
XP_021817103.1/1-243 S-AYVA-LLPSGFAI-VPDGPGSRGA------------MTVKGGG-HG------------
XP_021817105.1/1-243 S-AYVA-LLPSGFAI-VPDGPGSRGA------------MTVKGGG-HG------------
XP_022872559.1/1-230 S-TYVA-LLPSGFAI-VPDGSGSHGP------------PSNR------------------
XP_016575742.1/1-238 S-AYVA-LLPSGFSI-VPDGPGSRGS------------STGNCGS-NG------------
XP_017608938.1/1-232 S-AYVA-LLPSGFSI-IPDGTASPGP------------TTSNG-----------------
XP_017608947.1/1-232 S-AYVA-LLPSGFSI-IPDGTASPGP------------TTSNG-----------------
XP_010255501.1/1-245 S-AYVA-LLPSGFSI-VPDGPGSRGP------------TSNHHHG-P-------------
XP_011023407.1/1-238 S-AYVA-LLPSGFAI-VPDGPGSSDH------------QTTN-GG-P-------------
XP_011023404.1/1-238 S-AYVA-LLPSGFAI-VPDGPGSSDH------------QTTN-GG-P-------------
XP_011023405.1/1-238 S-AYVA-LLPSGFAI-VPDGPGSSDH------------QTTN-GG-P-------------
XP_019152252.1/1-243 S-AYVA-LLPSGFAI-VPDGPGSRGP------------TSGT-NG-P-------------
XP_022870029.1/1-235 S-AYVA-LLPSGFAI-MPDGPGSRGP------------AMDG------------------
XP_017699285.1/1-225 S-AYVA-LLPSGFAI-LPDGGGGGGG----------------------------------
XP_008795402.1/1-225 S-AYVA-LLPSGFAI-LPDGGGGGGG----------------------------------
XP_010933869.1/1-224 S-AYVA-LLPSGFAI-LPDGGGG-GG----------------------------------
XP_008799528.1/1-222 S-AYVA-LLPSGFAI-LPDGGGG-------------------------------------
XP_010919252.1/1-223 S-AYVA-LLPSGFAI-LPDGGGG--G----------------------------------
XP_010919250.1/1-223 S-AYVA-LLPSGFAI-LPDGGGG--G----------------------------------
XP_007139955.1/1-229 S-AYVA-LLPSGFAI-VPDGSVSGGE-------------H--------------------
XP_017418814.1/1-230 S-AYVA-LLPSGFAI-VPDGSAAGGE------------QH--------------------
XP_014490274.1/1-230 S-AYVA-LLPSGFAI-VPDGSAPGGE------------QH--------------------
XP_003534596.1/1-229 S-AYVA-LLPSGFAI-VPDGSVEE---------------N--------------------
XP_006587871.1/1-229 S-AYVA-LLPSGFAI-VPDGSVEE---------------N--------------------
XP_003552359.1/1-228 S-AYVA-LLPSGFAI-VPDGSGEE---------------Q--------------------
XP_015961616.1/1-228 S-AYVA-LLPSGFAV-APDGSGGRND------------HN--------------------
XP_015961617.1/1-228 S-AYVA-LLPSGFAV-APDGSGGRND------------HN--------------------
XP_025696060.1/1-228 S-AYVA-LLPSGFAV-APDGSGGRND------------HN--------------------
XP_025696061.1/1-228 S-AYVA-LLPSGFAV-APDGSGGRND------------HN--------------------
XP_016198931.1/1-228 S-AYVA-LLPSGFAV-APDGSSGRND------------HN--------------------
XP_016198932.1/1-228 S-AYVA-LLPSGFAV-APDGSSGRND------------HN--------------------
XP_025649261.1/1-228 S-AYVA-LLPSGFAV-APDGSGGRND------------HN--------------------
XP_025649262.1/1-228 S-AYVA-LLPSGFAV-APDGSGGRND------------HN--------------------
XP_021849370.1/1-228 S-AYVA-LLPSGFAI-VPDGPGSRGT------------NN--------------------
XP_021767325.1/1-228 S-AYVA-LLPSGFAI-VPDGPGLRGP------------SN--------------------
XP_021748881.1/1-228 S-AYVA-LLPSGFAI-VPDGPGSRGP------------SN--------------------
XP_019461451.1/1-231 S-AYVA-LLPSGFAI-VPDGPHNGGA------------N---------------------
XP_012574211.1/1-233 S-AYVA-LLPSGFAV-VPDGPGSRGP------------ENET------------------
XP_004510857.1/1-233 S-AYVA-LLPSGFAV-VPDGPGSRGP------------ENET------------------
XP_020237185.1/1-234 S-AYVA-LLPSGFAI-VPDGPGSRGP------------TA----T---------------
XP_020237261.1/1-234 S-AYVA-LLPSGFAI-VPDGPGSRGP------------TA----T---------------
XP_019456357.1/1-238 S-AYVA-LLPSGFAI-VPDGPGSRGT------------QNGGATT---------------
XP_013444922.1/1-235 S-AYVA-LLPSGFAI-VPDGPGSRGL------------ENGDATA---------------
XP_003528880.1/1-238 S-AYVA-LLPSGFAI-VPDGPGSRGP------------PNGPTST---------------
XP_006583340.1/1-238 S-AYVA-LLPSGFAI-VPDGPGSRGP------------PNGPTST---------------
XP_014516480.1/1-238 S-AYVA-LLPSGFAI-VPDGPGSRGS------------QNGPTAA---------------
XP_014516483.1/1-238 S-AYVA-LLPSGFAI-VPDGPGSRGS------------QNGPTAA---------------
XP_007134961.1/1-238 S-AYVA-LLPSGFAI-VPDGPGSRGS------------QNGTTTT---------------
XP_010671720.1/1-229 S-AYVA-LLPSGFAI-TPDGPSTRGP----------------------------------
XP_012482732.1/1-232 S-AYVA-LLPSGFAI-IPDGPGSPGP------------TTS-------------------
XP_012482742.1/1-232 S-AYVA-LLPSGFAI-IPDGPGSPGP------------TTS-------------------
XP_016745638.1/1-232 S-AYVA-LLPSGFAI-IPDGPGSPGP------------TTS-------------------
XP_016745639.1/1-232 S-AYVA-LLPSGFAI-IPDGPGSPGP------------TTS-------------------
XP_010278578.1/1-242 S-AYVA-LLPSGFAI-VPDGPGSRGP------------INSNHH----------------
XP_010278577.1/1-242 S-AYVA-LLPSGFAI-VPDGPGSRGP------------INSNHH----------------
XP_006445143.1/1-238 S-AYVA-LLPSGFAI-VPDGPDSRGP------------LANGPTS---------------
XP_006445141.1/1-238 S-AYVA-LLPSGFAI-VPDGPDSRGP------------LANGPTS---------------
XP_022770361.1/1-238 S-AYVA-LLPSGFAI-VPDGPGSRGP------------ISNGHIN---------------
XP_022757219.1/1-237 S-AYVA-LLPSGFAI-VPDGPGSRGP------------ASNEHIN---------------
XP_022757220.1/1-237 S-AYVA-LLPSGFAI-VPDGPGSRGP------------ASNEHIN---------------
NP_001314420.1/1-237 S-AYVA-LLPSGFAI-VPDGPRSHGP------------ISNGHVN---------------
XP_016735217.1/1-237 S-AYVA-LLPSGFAI-VPDGPRSHGP------------ISNGHVN---------------
XP_012437660.1/1-237 S-AYVA-LLPSGFAI-VPDGPGSHGP------------ISNGHVN---------------
XP_012437661.1/1-237 S-AYVA-LLPSGFAI-VPDGPGSHGP------------ISNGHVN---------------
XP_017615505.1/1-237 S-AYVA-LLPSGFAI-VPDGPGSHGP------------ISNGHVN---------------
XP_017615506.1/1-237 S-AYVA-LLPSGFAI-VPDGPGSHGP------------ISNGHVN---------------
XP_016723220.1/1-237 S-AYVA-LLPSGFAI-VPDGPGSHGP------------ISNGHVS---------------
XP_016723229.1/1-237 S-AYVA-LLPSGFAI-VPDGPGSHGP------------ISNGHVS---------------
XP_021904309.1/1-244 S-AYVA-LLPSGFAI-VPDGPGSRGP------------TVPNGPTSCN------------
XP_021904311.1/1-244 S-AYVA-LLPSGFAI-VPDGPGSRGP------------TVPNGPTSCN------------
XP_007051912.2/1-241 S-AYVA-LLPSGFAI-VPDGPGSRGP------------TSNGHV---N------------
XP_007051913.2/1-241 S-AYVA-LLPSGFAI-VPDGPGSRGP------------TSNGHV---N------------
XP_012489878.1/1-239 S-AYVA-LLPSGFAI-VPDGPGSRGP------------TSNGQV---N------------
XP_016748231.1/1-239 S-AYVA-LLPSGFAI-VPDGPGSRGP------------TSNGQV---N------------
XP_017631289.1/1-239 S-AYVA-LLPSGFAI-VPDGPGSRGP------------ISNGQV---N------------
XP_023006686.1/1-236 S-AYVA-LLPSGFAI-VADGAVTGG--------------------RSP------------
XP_022958954.1/1-236 S-AYVA-LLPSGFAI-VADGAVTGG--------------------RSP------------
XP_023548045.1/1-236 S-AYVA-LLPSGFAI-VADGAVTGG--------------------RSP------------
XP_008460172.1/1-241 S-AYVA-LLPSGFAI-VPDGAVTGGL------------TATN--GSSP------------
XP_004144982.1/1-241 S-AYVA-LLPSGFAI-VPDGAVTGGL------------TATN--GSSP------------
XP_023514722.1/1-242 S-AYVA-LLPSGFAI-VPDGAVSRGL------------TTTN--GSSP------------
XP_022999814.1/1-242 S-AYVA-LLPSGFAI-VPDGAVSRGL------------TTTN--GSSP------------
XP_022964504.1/1-242 S-AYVA-LLPSGFAI-VPDGAVSRGL------------TTTN--GSSP------------
XP_010053664.1/1-245 S-AYVA-LLPSGFAI-VPDGPDGPGS------------HG----GGAA------------
XP_019189515.1/1-235 S-AYVA-LLPSGFAI-VPDGPGCRGG------------PNAENGGVP-------------
XP_023730775.1/1-234 S-AYVA-LLPSGFAI-VPDGTGGRVG------------SETDNN----------------
XP_023730774.1/1-234 S-AYVA-LLPSGFAI-VPDGTGGRVG------------SETDNN----------------
XP_022020543.1/1-230 S-AYVA-LLPSGFAI-VPDGPGDRGV------------SETDNGG---------------
XP_024962572.1/1-236 S-AYVA-LLPSGFAI-VPDGPGNRGV------------SETDNGG---------------
XP_022017696.1/1-236 S-AYVA-LLPSGFAI-VPDGPGNRVG------------SETENGG---------------
XP_024018220.1/1-245 S-AYVA-LLPSGFSI-VPDGPGSRGS------------VSATTNGGGN------------
XP_011093166.1/1-234 S-AYVA-LLPSGFAI-VPDGPGSRGG--------------------DD------------
XP_011093167.1/1-234 S-AYVA-LLPSGFAI-VPDGPGSRGG--------------------DD------------
XP_006339457.1/1-238 S-AYVA-LLPSGFSI-VPDGPGSRG---------------------SN------------
XP_015059298.1/1-238 S-AYVA-LLPSGFSI-VPDGPGSRG---------------------SN------------
NP_001234657.2/1-238 S-AYVA-LLPSGFSI-VPDGPGSRG---------------------SN------------
XP_009602857.1/1-241 S-AYVA-LLPSGFSI-VPDGPGSRGS------------STAN--CGSN------------
XP_009602856.1/1-241 S-AYVA-LLPSGFSI-VPDGPGSRGS------------STAN--CGSN------------
XP_009782444.1/1-245 S-AYVA-LLPSGFSI-VPDGPGSRGS------------NTAN--CGSN------------
XP_009782443.1/1-245 S-AYVA-LLPSGFSI-VPDGPGSRGS------------NTAN--CGSN------------
XP_019259406.1/1-245 S-AYVA-LLPSGFSI-VPDGPGSRGS------------NTAN--CGSN------------
XP_015867375.1/1-242 S-AYVA-LLPSGFAI-VPDGPGSRGP------------MATTTSNGNN------------
XP_015867374.1/1-242 S-AYVA-LLPSGFAI-VPDGPGSRGP------------MATTTSNGNN------------
XP_015888672.1/1-242 S-AYVA-LLPSGFAI-VPDGPGSRGP------------MATTTSNGNN------------
XP_018810322.1/1-233 S-AYVA-LLPSGFAI-VPDGPGSRGS------------GET-------------------
XP_018859074.1/1-233 S-AYVA-LLPSGFAI-VPDGPGSRGS------------GET-------------------
XP_017231243.1/1-235 S-AYVA-LLPSGFAI-LPDGPDPRGP------------SGNGPLSN--------------
XP_017231244.1/1-235 S-AYVA-LLPSGFAI-LPDGPDPRGP------------SGNGPLSN--------------
XP_017231245.1/1-235 S-AYVA-LLPSGFAI-LPDGPDPRGP------------SGNGPLSN--------------
XP_017236322.1/1-235 S-AYVA-LLPSGFAI-VPDGQGSRGP------------SGNGTVSN--------------
XP_002301331.2/1-238 S-AYVA-LLPSGFAI-VPDGPGSRDP------------PSTNGGPTAN------------
XP_024451706.1/1-238 S-AYVA-LLPSGFAI-VPDGPGSRDP------------PSTNGGPTAN------------
XP_002320755.1/1-241 S-AYVA-LLPSGFAI-VPDGPGSRGP------------PTTNGGPTAN------------
XP_011035097.1/1-241 S-AYVA-LLPSGFAI-VPDGPGSRGP------------PTTNGGPTAN------------
XP_018813891.1/1-238 S-AYVA-LLPSGFAI-VPDGPGSRGP------------GTATA----N------------
XP_012083470.1/1-239 S-AYVA-LLPSGFSI-VPDGPGSRGS------------PSTNANGPSS------------
XP_021651338.1/1-240 S-AYVA-LLPSGFAV-VPDGPGSRGS------------PTTP-NGPTG------------
XP_021613538.1/1-241 S-AYVA-LLPSGFAI-VPDGPGSRGS------------PGTA-NDPTS------------
XP_021686862.1/1-241 S-AYVA-LLPSGFAI-VPDGPGSRGS------------LANP-NGPTS------------
XP_021686863.1/1-241 S-AYVA-LLPSGFAI-VPDGPGSRGS------------LANP-NGPTS------------
XP_021612685.1/1-241 S-AYVA-LLPSGFAI-VPDGPGSRGS------------LSTP-NGPTG------------
XP_023879166.1/1-242 S-AYVA-LLPSGFAI-VPDGPGSRGP------------TGNGNNSNNG------------
XP_002272264.2/1-234 S-AYVA-LLPSGFAI-VPDGPGSRGP------------NS--------------------
XP_010661562.1/1-234 S-AYVA-LLPSGFAI-VPDGPGSRGP------------NS--------------------
XP_002511801.1/1-237 S-AYVA-LLPSGFAI-VPDGPGSRGS------------PTNQ-----N------------
XP_015584500.1/1-237 S-AYVA-LLPSGFAI-VPDGPGSRGS------------PTNQ-----N------------
XP_022141257.1/1-238 S-TYVS-LLPSGFAI-LPDGLSSYAC-------------TN-NGKDAS-VTSDI------
XP_023885612.1/1-244 S-TYVA-LLPSGFSI-LPDDQ-SFFG------------GPQ-NCNGTV-IKEDN------
XP_023885611.1/1-244 S-TYVA-LLPSGFSI-LPDDQ-SFFG------------GPQ-NCNGTV-IKEDN------
XP_018830842.1/1-239 S-AYVA-LLPSGFAI-LPDEP-SSYG------------GPH-NFNRTM-VKAGD------
XP_018830843.1/1-239 S-AYVA-LLPSGFAI-LPDEP-SSYG------------GPH-NFNRTM-VKAGD------
XP_018830840.1/1-239 S-AYVA-LLPSGFAI-LPDEP-SSYG------------GPH-NFNRTM-VKAGD------
XP_018830841.1/1-239 S-AYVA-LLPSGFAI-LPDEP-SSYG------------GPH-NFNRTM-VKAGD------
XP_012090293.1/1-240 S-TYVA-LLPSGFVI-VPDDS-LRQG------------GPN-FCNGNL-VKRDS------
XP_012090292.1/1-240 S-TYVA-LLPSGFVI-VPDDS-LRQG------------GPN-FCNGNL-VKRDS------
XP_021614581.1/1-239 S-TYVA-LLPSGFVI-LPDDT-SSQG------------GPN-FS-STL-VKRDS------
XP_021614580.1/1-239 S-TYVA-LLPSGFVI-LPDDT-SSQG------------GPN-FS-STL-VKRDS------
XP_015581247.1/1-240 S-TYVA-LLPSGFVI-LPDDS-SSQG------------GSN-FCNGTL-VKRDS------
XP_002510822.1/1-240 S-TYVA-LLPSGFVI-LPDDS-SSQG------------GSN-FCNGTL-VKRDS------
XP_021648646.1/1-240 S-TYVA-LLPSGFVI-HPYDS-IGQG------------GPN-FCKGTL-VKRNG------
XP_021634593.1/1-234 S-TYVA-LLPSGFVI-H-------QG------------GPS-FSNGTL-VERDN------
XP_021634592.1/1-234 S-TYVA-LLPSGFVI-H-------QG------------GPS-FSNGTL-VERDN------
XP_006490348.1/1-238 S-TCVA-LLPSGFAI-LPDGY-FGHG------------GLI-NSNGTM-VKTEG------
XP_006490346.1/1-238 S-TCVA-LLPSGFAI-LPDGY-FGHG------------GLI-NSNGTM-VKTEG------
XP_024958654.1/1-238 S-TCVA-LLPSGFAI-LPDGY-FGHG------------GLI-NSNGTM-VKTEG------
XP_006490345.1/1-238 S-TCVA-LLPSGFAI-LPDGY-FGHG------------GLI-NSNGTM-VKTEG------
XP_006421879.2/1-238 S-TCVA-LLPSGFAI-LPDGY-FGHG------------GLI-NSNGTM-VKTEG------
XP_021892212.1/1-221 S-TYVA-LLPSGFGI-VPLPPNKC------------------------------------
XP_021892211.1/1-221 S-TYVA-LLPSGFGI-VPLPPNKC------------------------------------
XP_021892210.1/1-221 S-TYVA-LLPSGFGI-VPLPPNKC------------------------------------
XP_022765148.1/1-244 S-TYVA-LLPSGFAI-LPCISPNNHV------------GQS-NSNGPL-VKPDI------
XP_022765158.1/1-244 S-TYVA-LLPSGFAI-LPCISPNNHV------------GQS-NSNGPL-VKPDI------
XP_022765120.1/1-244 S-TYVA-LLPSGFAI-LPCISPNNHV------------GQS-NSNGPL-VKPDI------
XP_022765114.1/1-244 S-TYVA-LLPSGFAI-LPCISPNNHV------------GQS-NSNGPL-VKPDI------
XP_022736693.1/1-237 S-AYVA-LLPSGFAI-LPGISPSYRG------------GQS-NSNGPL-MKPDI------
XP_022736692.1/1-237 S-AYVA-LLPSGFAI-LPGISPSYRG------------GQS-NSNGPL-MKPDI------
XP_022736689.1/1-237 S-AYVA-LLPSGFAI-LPGISPSYRG------------GQS-NSNGPL-MKPDI------
XP_022734775.1/1-244 S-AYVA-LLPSGFAI-FPGISPSYHG------------GQS-NSNEPS-VKPDI------
XP_016743353.1/1-244 S-AYVA-LLPSGFAI-LPGISPSYHG------------GRS-ESNGAL-VKPEI------
XP_012474605.1/1-244 S-AYVA-LLPSGFAI-LPGISPSYHG------------GRS-ESNGAL-VKPEI------
XP_012474606.1/1-244 S-AYVA-LLPSGFAI-LPGISPSYHG------------GRS-ESNGAL-VKPEI------
XP_016743352.1/1-244 S-AYVA-LLPSGFAI-LPGISPSYHG------------GRS-ESNGAL-VKPEI------
XP_016743351.1/1-244 S-AYVA-LLPSGFAI-LPGISPSYHG------------GRS-ESNGAL-VKPEI------
XP_012474604.1/1-244 S-AYVA-LLPSGFAI-LPGISPSYHG------------GRS-ESNGAL-VKPEI------
XP_012474603.1/1-244 S-AYVA-LLPSGFAI-LPGISPSYHG------------GRS-ESNGAL-VKPEI------
XP_017646297.1/1-244 S-TYVA-LLPSGFAI-LPGISPSYHG------------GRS-ESNGAL-VKPEI------
XP_016736315.1/1-244 S-TYVA-LLPSGFAI-LPGISPSYHG------------GRS-ESNGAL-VKPEI------
XP_017646296.1/1-244 S-TYVA-LLPSGFAI-LPGISPSYHG------------GRS-ESNGAL-VKPEI------
XP_017646298.1/1-244 S-TYVA-LLPSGFAI-LPGISPSYHG------------GRS-ESNGAL-VKPEI------
XP_016736306.1/1-244 S-TYVA-LLPSGFAI-LPGISPSYHG------------GRS-ESNGAL-VKPEI------
XP_017646295.1/1-244 S-TYVA-LLPSGFAI-LPGISPSYHG------------GRS-ESNGAL-VKPEI------
XP_017972896.1/1-244 S-AYVA-LLPSGFAI-LPGISPSYHG------------GQS-NSNGPM-VKPDI------
XP_017972895.1/1-244 S-AYVA-LLPSGFAI-LPGISPSYHG------------GQS-NSNGPM-VKPDI------
XP_017972893.1/1-244 S-AYVA-LLPSGFAI-LPGISPSYHG------------GQS-NSNGPM-VKPDI------
XP_021274823.1/1-244 S-AYVA-LLPSGFAI-LPGISPSYHG------------GQS-NSNGPL-VKPDI------
XP_024536343.1/1-231 P-AYVA-LLPSGFAI-LPEGPRSIGT------------TPETSS----------------
XP_006366174.1/1-244 S-AYVA-LLPSGFSI-VPDGPGSRGP------------NLVKSLNN-------GPG----
XP_020219711.1/1-212 S-AYVA-LLPSGFSI-VPDGSGGGED------------QG---------------G----
XP_022897056.1/1-222 S-TYVA-LLPSGFAI-VPDG--SRGP------------T---------------SN----
NP_001281015.1/1-243 S-AYVA-LLPSGFAI-VPDGPGSRGP------------MSGKGATHGS-SNGGGCG----
XP_008383260.1/1-243 S-AYVA-LLPSGFAI-VPDGPGSRGP------------MSGKGATHGS-SNGGGCG----
XP_022896229.1/1-235 S-AYVA-LLPSGFAI-MPAGPGSRGP------------T---------------MD----
XP_022896230.1/1-235 S-AYVA-LLPSGFAI-MPAGPGSRGP------------T---------------MD----
XP_025816451.1/1-225 S-TYVA-LLPSGFAI-LPDGPSA-AT------------G---------------------
XP_004968001.1/1-225 S-TYVA-LLPSGFAI-LPDGPS-TGA------------E---------------------
XP_004968002.1/1-225 S-TYVA-LLPSGFAI-LPDGPS-TGA------------E---------------------
XP_002457484.1/1-226 S-TYVA-LLPSGFAI-LPDGPSGVGA------------E---------------------
XP_009400446.1/1-224 S-SYVA-LLPSGFAI-LPDG----------------------------------------
XP_018676535.1/1-225 S-AYVS-LLPSGFAI-LPDG----------------------------------------
XP_009401771.1/1-232 S-AYVA-LLPSGFAV-LPDGLPSGSV------------G---------------------
XP_009384534.1/1-228 S-AYVA-LLPSGFAI-LPDG----SG------------G---------------------
XP_020672447.1/1-217 S-AYVA-LLPSGFAV-MPDGA---------------------------------------
XP_020582116.1/1-218 S-TYVA-LLPSGFAV-MPDGG---------------------------------------
XP_020675647.1/1-231 S-AYVA-LLPSGFAV-MPDGPVAAGG------------E---------------------
XP_020675648.1/1-231 S-AYVA-LLPSGFAV-MPDGPVAAGG------------E---------------------
XP_020585632.1/1-228 S-AYVA-LLPSGFAV-MPDGQVAEDG------------E---------------------
XP_016176402.1/1-228 S-AYVA-LLPSGFAI-LPDGHSSSGS------------KGG-------------------
XP_016176403.1/1-228 S-AYVA-LLPSGFAI-LPDGHSSSGS------------KGG-------------------
XP_016176400.1/1-228 S-AYVA-LLPSGFAI-LPDGHSSSGS------------KGG-------------------
XP_016176401.1/1-228 S-AYVA-LLPSGFAI-LPDGHSSSGS------------KGG-------------------
XP_010523042.1/1-245 S-SYVA-LLPSGFAI-LPDGGGPSSP------------HGGGGSPRGP-ASPHGGG----
XP_010523041.1/1-240 S-SYVA-LLPSGFAI-LPDGGGPSSP------------HGGG-----P-ASPHGGG----
XP_020878511.1/1-224 S-SYVA-LLPSGFAV-LPDGGID-----------------G----DGE-Q----------
NP_567183.2/1-228    S-SYVA-LLPSGFAV-LPDGGID-----------------GGGSGDGD-Q----------
XP_006287095.1/1-228 S-SYVA-LLPSGFAV-LPDGGMD-----------------GGSGGDGE-Q----------
XP_010456250.1/1-228 S-SYVA-LLPSGFAV-LPDGGMD-----------------GGSSGDSE-Q----------
XP_010419627.1/1-228 S-SYVA-LLPSGFAV-LPDGGMD-----------------GGSSGDSE-Q----------
XP_010427345.1/1-228 S-SYVA-LLPSGFAV-LPDGGMD-----------------GGSSGDGE-Q----------
XP_009111469.1/1-229 S-SYVA-LLPSGFAV-LPDGGFN-----------------G-GSGDGE-Q----------
XP_018487039.1/1-229 S-SYVA-LLPSGFAV-LPDGGFD-----------------G-GSRDGE-Q----------
XP_018453129.1/1-229 S-SYVA-LLPSGFAV-LPDGGFD-----------------G-GSRDGE-Q----------
XP_013612299.1/1-229 S-SYVA-LLPSGFAV-LPDGGFD-----------------G-VSGDGE-Q----------
XP_006396268.1/1-225 S-SYVA-LLPSGFAI-LPDGGID-----------------G-GSGDGE-Q----------
XP_018475317.1/1-221 S-SYVA-LLPSGFVV-LPDG----------------------GSGDVE-Q----------
XP_013629750.1/1-221 S-SYVA-LLPSGFAV-LPDG----------------------GSGDVE-Q----------
XP_009134533.1/1-221 S-SYVA-LLPSGFAV-LPDG----------------------GSGDVE-Q----------
XP_020531377.1/1-244 S-TYVA-LLPSGFAI-LPDGPGSPHQ------------LGWPPQPG--------------
XP_006857866.1/1-244 S-TYVA-LLPSGFAI-LPDGPGSPHQ------------LGWPPQPG--------------
XP_018434263.1/1-219 S-AYVA-LLPSGFAI-LPDVQG--------------------------------------
XP_018434260.1/1-219 S-AYVA-LLPSGFAI-LPDVQG--------------------------------------
XP_018434262.1/1-219 S-AYVA-LLPSGFAI-LPDVQG--------------------------------------
XP_013688926.1/1-219 S-AYVA-LLPSGFAI-LPDGQG--------------------------------------
XP_013637558.1/1-219 S-AYVA-LLPSGFAI-LPDDQG--------------------------------------
XP_013637556.1/1-219 S-AYVA-LLPSGFAI-LPDDQG--------------------------------------
XP_013637557.1/1-219 S-AYVA-LLPSGFAI-LPDDQG--------------------------------------
XP_009143168.1/1-219 S-AYVA-LLPSGFAI-LPDGQG--------------------------------------
XP_009143167.1/1-219 S-AYVA-LLPSGFAI-LPDGQG--------------------------------------
XP_009143166.1/1-219 S-AYVA-LLPSGFAI-LPDGQG--------------------------------------
XP_013692686.1/1-219 S-AYVA-LLPSGFAI-LPDGQG--------------------------------------
XP_022574820.1/1-219 S-AYVA-LLPSGFAI-LPDGQG--------------------------------------
XP_013692684.1/1-219 S-AYVA-LLPSGFAI-LPDGQG--------------------------------------
XP_013748127.1/1-219 S-AYVA-LLPSGFAI-LPDGQG--------------------------------------
XP_013692685.1/1-219 S-AYVA-LLPSGFAI-LPDGQG--------------------------------------
XP_013748124.1/1-219 S-AYVA-LLPSGFAI-LPDGQG--------------------------------------
XP_013748126.1/1-219 S-AYVA-LLPSGFAI-LPDGQG--------------------------------------
XP_022574821.1/1-219 S-AYVA-LLPSGFAI-LPDGQG--------------------------------------
XP_006290613.1/1-229 S-AYVA-LLPSGFAI-LPNAGTQREE------------SN----GG--------------
XP_013592301.1/1-230 S-AYVA-LLPSGFAI-LPNAPQRLEE------------RN-G--NG--------------
XP_018442581.1/1-230 S-AYVA-LLPSGFAI-LPNAPQGSEE------------RN-G--NG--------------
XP_013750213.1/1-232 S-AYVA-LLPSGFAI-LPSAPQRSEE------------RN-GNGSG--------------
XP_013648430.1/1-232 S-AYVA-LLPSGFAI-LPSAPQLSEE------------RN-GNGSG--------------
XP_009104292.1/1-232 S-AYVA-LLPSGFAI-LPSAPQLSEE------------RN-GNGSG--------------
XP_019058514.1/1-223 S-AYVA-LLPSGFAI-LPDSGAG-------------------------------------
XP_010523728.1/1-220 S-AYVA-LLPSGFAI-VPDSGQM-------------------------------------
XP_024514927.1/1-242 P-ATVA-LLPSGFAI-SPDGAVSQRT------------TQ----------EGGGAG----
XP_002985242.1/1-242 P-ATVA-LLPSGFAI-SPDGAVSQRT------------TQ----------EGGGAG----
XP_024530500.1/1-242 P-ATVA-LLPSGFAI-SPDGAVSQRT------------TQ----------EGGGAG----
XP_024530499.1/1-242 P-ATVA-LLPSGFAI-SPDGAVSQRT------------TQ----------EGGGAG----
XP_024530501.1/1-242 P-ATVA-LLPSGFAI-SPDGAVSQRT------------TQ----------EGGGAG----
XP_010107411.1/1-242 S-AYVA-LLPSGFAI-LPDGSSQPN-----------------YCSGPS-VKGEASN----
XP_024028635.1/1-242 S-AYVA-LLPSGFAI-LPDGSSQPN-----------------YCSGPS-VKGEASN----
XP_015877999.1/1-244 S-AYVA-LLPSGFAI-LPDGRSSGIG------------GST-SYNGSS-NKGQGDG----
XP_019427294.1/1-247 S-EYVA-LLPSGFAI-VPDGQSSGYG------------VAN-SSNGTI-PKGGGGG----
XP_019427292.1/1-247 S-EYVA-LLPSGFAI-VPDGQSSGYG------------VAN-SSNGTI-PKGGGGG----
XP_019452814.1/1-239 S-AYVA-LLPSGFAI-LPDCQSSRYS------------TSNNSCNGNI-PKGG-------
XP_019452815.1/1-239 S-AYVA-LLPSGFAI-LPDCQSSRYS------------TSNNSCNGNI-PKGG-------
XP_003592295.2/1-235 S-AYVA-LLPSGFAI-VPDGNDP---------------SGYGMSNGTL-QKGG-------
XP_024625328.1/1-235 S-AYVA-LLPSGFAI-VPDGNDP---------------SGYGMSNGTL-QKGG-------
XP_012570175.1/1-236 S-AYVA-LLPSGFAI-LPDGNS----------------SDYGASNGTL-QKGG-------
XP_012570176.1/1-236 S-AYVA-LLPSGFAI-LPDGNS----------------SDYGASNGTL-QKGG-------
XP_004496593.1/1-236 S-AYVA-LLPSGFAI-LPDGNS----------------SDYGASNGTL-QKGG-------
XP_025620154.1/1-234 S-AYVA-LLPSGFAI-LPDGHSS---------------S---GSNGNL-AKGG-------
XP_015941562.1/1-234 S-AYVA-LLPSGFAI-LPDGHSS---------------S---GSNGNL-AKGG-------
XP_025620153.1/1-234 S-AYVA-LLPSGFAI-LPDGHSS---------------S---GSNGNL-AKGG-------
XP_020239673.1/1-232 S-AYVA-LLPSGFAI-LPDGHSS---------------N---SCNGTM-AKGG-------
XP_020239648.1/1-232 S-AYVA-LLPSGFAI-LPDGHSS---------------N---SCNGTM-AKGG-------
XP_007143377.1/1-235 S-AYVA-LLPSGFAI-LPDGHCN---------------NNS-SCNGTM-QKGG-------
XP_007143378.1/1-235 S-AYVA-LLPSGFAI-LPDGHCN---------------NNS-SCNGTM-QKGG-------
XP_017415467.1/1-234 S-AYVA-LLPSGFAI-VPDGHCN---------------NS--TCNGTM-QKGG-------
XP_017415466.1/1-234 S-AYVA-LLPSGFAI-VPDGHCN---------------NS--TCNGTM-QKGG-------
XP_014514421.1/1-236 S-AYVA-LLPSGFAI-VPDGHCN---------------NSSSSCNGTM-QKGG-------
XP_014514419.1/1-236 S-AYVA-LLPSGFAI-VPDGHCN---------------NSSSSCNGTM-QKGG-------
XP_006606097.1/1-235 S-AYVA-LLPSGFAI-LPDGHCN---------------DN--GCNGSL-QKGR-------
XP_006606096.1/1-235 S-AYVA-LLPSGFAI-LPDGHCN---------------DN--GCNGSL-QKGR-------
XP_003536477.1/1-234 S-AYVA-LLPSGFAI-LPDGHCN---------------DN--GCNGTL-QKGG-------
XP_006589557.1/1-234 S-AYVA-LLPSGFAI-LPDGHCN---------------DN--GCNGTL-QKGG-------
XP_006402516.1/1-230 S-AYVA-LLPSGFAI-LPNAP---RR------------CAAEE-----------------
NP_191674.1/1-237    S-AYVA-LLPSGFAI-LPNGQAGTQR------------CAAEER-------NSI------
XP_002876596.1/1-239 S-AYVA-LLPSGFAI-LPNAQAGTQR------------CAAEER-------NANGN----
XP_010413451.1/1-238 S-AYVA-LLPSGFAI-LPNAQTGTQR------------CTTEEP-------NGS------
XP_010413452.1/1-238 S-AYVA-LLPSGFAI-LPNAQTGTQR------------CTTEEP-------NGS------
XP_010413453.1/1-238 S-AYVA-LLPSGFAI-LPNAQTGTQR------------CTTEEP-------NGS------
XP_010469036.1/1-242 S-AYVA-LLPSGFAI-LPNAQAGTQR------------CATEEP-------NGNGN----
XP_010469037.1/1-242 S-AYVA-LLPSGFAI-LPNAQAGTQR------------CATEEP-------NGNGN----
XP_010469038.1/1-242 S-AYVA-LLPSGFAI-LPNAQAGTQR------------CATEEP-------NGNGN----
XP_010512448.1/1-238 S-AYVA-LLPSGFAI-LPNAQAGTQR------------CATEE-----------GN----
XP_010512449.1/1-238 S-AYVA-LLPSGFAI-LPNAQAGTQR------------CATEE-----------GN----
XP_006397757.1/1-219 S-AYVA-LLPSGFAI-LPN-G---------------------------------------
XP_024010837.1/1-220 S-AYVA-LLPSGFAI-LPN-G---------------------------------------
XP_023640300.1/1-220 S-AHVA-LLPSGFGI-LPDDG---------------------------------------
XP_006293718.1/1-220 S-AHVA-LLPSGFGI-LPDDG---------------------------------------
XP_010506553.1/1-219 S-AYVA-LLPSGFGI-LPD-G---------------------------------------
XP_010508114.1/1-219 S-AYVA-LLPSGFGI-LPD-G---------------------------------------
XP_010508115.1/1-219 S-AYVA-LLPSGFGI-LPD-G---------------------------------------
XP_022147276.1/1-219 S-AYVA-LLPSGFAM-VPD--GG-------------------------------------
XP_023527464.1/1-222 S-AYVA-LLPSGFAV-VPAAEDC-------------------------------------
XP_022980233.1/1-222 S-AYVA-LLPSGFAV-VPAAEDC-------------------------------------
XP_022980231.1/1-222 S-AYVA-LLPSGFAV-VPAAEDC-------------------------------------
XP_022924366.1/1-223 S-AYVA-LLPSGFAV-VPAAEDC-------------------------------------
XP_022924364.1/1-223 S-AYVA-LLPSGFAV-VPAAEDC-------------------------------------
XP_023527467.1/1-221 S-AYVA-LLPSGFAV-VPAAEDC-------------------------------------
XP_023527463.1/1-221 S-AYVA-LLPSGFAV-VPAAEDC-------------------------------------
XP_023527465.1/1-221 S-AYVA-LLPSGFAV-VPAAEDC-------------------------------------
XP_022980232.1/1-221 S-AYVA-LLPSGFAV-VPAAEDC-------------------------------------
XP_022924365.1/1-222 S-AYVA-LLPSGFAV-VPAAEDC-------------------------------------
XP_009412599.1/1-224 S-AYVA-LLPSGFAI-LPDRSVEPHQ----------------------------------
XP_018677084.1/1-231 S-TYVT-LLPSGFAI-LPDGRGSGAG------------VT--------------------
XP_020114860.1/1-246 S-AYVA-LLPSGFAI-LPDAPRSNPN------------IPNSNP------PTSAAP----
XP_020114857.1/1-246 S-AYVA-LLPSGFAI-LPDAPRSNPN------------IPNSNP------PTSAAP----
XP_020114859.1/1-246 S-AYVA-LLPSGFAI-LPDAPRSNPN------------IPNSNP------PTSAAP----
XP_020084803.1/1-234 A-AYVA-LLPSGFAI-LPDANRS-------------------------------------
XP_015625616.1/1-226 S-TYVA-LLPSGFAI-LPDGPRIGAT----------------------------------
XP_006647677.1/1-226 S-TYVA-LLPSGFAI-LPDGPRIGAS----------------------------------
NP_001105493.1/1-226 S-TYVA-LLPSGFAI-LPDGPSSVGA----------------------------------
XP_020178635.1/1-225 S-TYVA-LLPSGFAI-LPDGPS-IGS----------------------------------
XP_003570085.1/1-223 S-TCVA-LLPSGFAI-LPDGPS-I------------------------------------
XP_020247871.1/1-226 T-SFIP-L-------------LPTG-------------F-VLLPAPS-------------
XP_020247870.1/1-226 T-SFIP-L-------------LPTG-------------F-VLLPAPS-------------
XP_024004764.1/1-211 L-SSIP-LLPLGFSV-VPVNPP------------------------E-------------
XP_018449374.1/1-217 P-SRIP-LLPLGFSV-VPVNQP------------------------H-------------
XP_013602839.1/1-215 P-SRIS-LLPLGFSV-VPVNQP------------------------H-------------
XP_013655966.1/1-215 P-SRIS-LLPLGFSV-VPVNQP------------------------H-------------
XP_009108342.1/1-215 P-SRIP-LLPLGFSV-VPVNQP------------------------H-------------
XP_013655964.1/1-215 P-SRIP-LLPLGFSV-VPVNQP------------------------H-------------
XP_020872468.1/1-222 P-FKLP-LLPVGFSV-VPVNPS------------------------D-------------
NP_193506.2/1-221    P-SEIP-LLPVGFSV-VPVNPS------------------------D-------------
XP_023633855.1/1-222 P-SKVP-LLPVGFSI-VPVNPS------------------------D-------------
XP_010449572.1/1-221 P-SNIP-LLPVGFSV-VPVNPS------------------------D-------------
XP_010439957.1/1-223 P-SNIP-LLPVGFSV-VPVNPS------------------------D-------------
XP_010439959.1/1-223 P-SNIP-LLPVGFSV-VPVNPS------------------------D-------------
XP_010439958.1/1-223 P-SNIP-LLPVGFSV-VPVNPS------------------------D-------------
XP_006854056.1/1-234 H-SYVP-LLPSGFII-TPRRQPNDPK------------HAY-------------------
XP_020529072.1/1-234 H-SYVP-LLPSGFII-TPRRQPNDPK------------HAY-------------------
NP_199499.3/1-224    S-SNIP-ILPLGFSI-VPVNPP---E------------G---------------------
XP_020871346.1/1-224 S-SSIP-ILPLGFSI-VPVNPP---E------------G---------------------
XP_006281890.1/1-224 S-SNIP-ILPLGFSI-VPVNPP---E------------G---------------------
XP_010481400.1/1-224 S-SNIP-ILPLGFSI-VPVIPP---E------------G---------------------
XP_010494885.1/1-224 S-SNIP-ILPLGFSI-VPVNPP---E------------G---------------------
XP_018455882.1/1-227 P-SSIP-LLPLGFSV-VPANDLGEVE------------G---------------------
XP_013713146.1/1-227 P-SNVP-LLPLGFSV-VPVNPPGEVE------------G---------------------
XP_013607209.1/1-227 P-SNVP-LLPLGFSV-VPVNPPGEVE------------G---------------------
XP_009114317.1/1-227 P-SNVP-VLPLGFSV-VPVNPPGEVE------------G---------------------
XP_013661958.1/1-227 P-SNVP-FLPLGFSV-VPVNPPGEVE------------G---------------------
XP_006398384.1/1-227 P-SGIP-LLPLGFSV-VPVDPPDHVE------------G---------------------
XP_024011152.1/1-227 P-SGIP-LLPLGFSV-VPVDPPDHVE------------G---------------------
XP_018482944.1/1-227 P-SGIP-LLPLGFSI-IPVNYPDQVE------------G---------------------
XP_009101472.1/1-227 P-SGMP-LLPLGFSV-VPVNYPDQVE------------G---------------------
XP_013693979.1/1-227 P-SGIP-LLPLGFSV-VPVNYPDQVE------------G---------------------
XP_013615089.1/1-227 P-SGIP-LLPLGFSV-VPVNYPDQVE------------G---------------------
XP_013704013.1/1-227 P-SGIP-LLPLGFSV-VPVNYPDQVE------------G---------------------
XP_009373477.1/1-246 P-SCIP-LLPLGFVI-VPVQPIESSA------------ATAVD----------HGTSSPD
XP_006662631.2/1-256 P-SYIP-LLPLGFAI-FPATSPSPAA------------ATISSSTTTTTTTTGNGESSST
XP_015613461.1/1-259 P-SYIP-LLPLGFAI-FPATSPSPAA------------APTISSSTTTTTGNGNGETSST
NP_001337805.1/1-240 P-SYIP-LLPLGFAI-FPAANPSAAA------------TSAAG--------SGDGQSSP-
XP_020163828.1/1-239 P-SYIP-LLPMGFAI-FPATSPSPGA------------TSSDT--------TSNGETSP-
XP_010235052.1/1-238 P-SYIP-LLPLGFAI-FPATNPSPSA------------TRAN---------TGDGESTP-
XP_025798256.1/1-238 P-SYIP-LLPLGFAI-FPATNPLPAA------------TSAS---------SGNGESSP-
XP_004983555.1/1-238 P-SYIP-LLPLGFAI-FPATNPSPAA------------TSTS---------SGNGESSP-
XP_002464880.2/1-238 P-SYIP-LLPLGFAI-FPATNPSPAA------------TSTS---------SGNGESSP-
XP_020262583.1/1-231 T-SFIP-LLPTGFIL-LPSPPPSSIS------------SPDD------------------
XP_020677410.1/1-231 P-SYIP-LLPTGFAL-FPAEQPASVS------------CSD-------------------
XP_020577604.1/1-229 T-SYIP-LLPTGFVL-FPAEQPASAS------------CSN-------------------
XP_020577606.1/1-229 T-SYIP-LLPTGFVL-FPAEQPASAS------------CSN-------------------
XP_020577607.1/1-229 T-SYIP-LLPTGFVL-FPAEQPASAS------------CSN-------------------
XP_019709148.1/1-245 S-SFIP-LLPTGFII-SPADPPTFSG------------VTDAFPSPSTDV----------
XP_008803567.1/1-241 P-SFIP-LLPTGFII-SPADPPTT-G------------ATDALPSPSTD-----------
XP_008803570.2/1-241 P-SFIP-LLPTGFII-SPADPPTT-G------------ATDALPSPSTD-----------
XP_018681950.1/1-231 P-SYIP-LLPTGFVI-SPAARQPNAG------------T---------------------
XP_018681951.1/1-231 P-SYIP-LLPTGFVI-SPAARQPNAG------------T---------------------
XP_010919976.1/1-237 P-SFIP-LLPTGFVI-SPPGPSTTGV------------TNA--TAPTAE-----------
XP_008800084.1/1-237 P-SFIP-LLPTGFVI-SPPSPPATGV------------TNA--PPPTTE-----------
XP_018679997.1/1-229 P-SYIP-LLPTGFVI-SPAAPPNGVI------------S---------------------
XP_009397149.1/1-229 P-SYIP-LLPTGFVI-SPAAPPNGVI------------S---------------------
XP_024995523.1/1-235 P-SCIP-LLPLGFVI-VPAGQNPAGN------------LSSTS-----------------
XP_023736252.1/1-233 P-SCIP-LLPLGFVI-VPAGQNPNDN------------ITST------------------
XP_023736253.1/1-233 P-SCIP-LLPLGFVI-VPAGQNPNDN------------ITST------------------
XP_022022041.1/1-231 P-SCIP-LLPLGFVI-IPLGQNPNSN------------ITSTS-----------------
XP_021969857.1/1-226 P-SRIP-LLPLGFVI-VPMGQNPNGN------------ITA-------------------
XP_010245213.1/1-236 P-SYIP-LLPIGFVV-FPVGSAGVGG------------SISCN----------------G
XP_010245210.1/1-236 P-SYIP-LLPIGFVV-FPVGSAGVGG------------SISCN----------------G
XP_010276832.1/1-235 P-SYIP-LLPIGFVV-VPAGHSSVSC------------N-NGN----------------G
XP_010276837.1/1-235 P-SYIP-LLPIGFVV-VPAGHSSVSC------------N-NGN----------------G
XP_019155823.1/1-230 P-SSVP-LLPLGFVI-TPMAAAATAT------------A---------------------
XP_022881662.1/1-238 P-SCIP-LLPLGFII-SPMGHTINDS------------SITI------E----------A
XP_022877113.1/1-234 P-SCIP-LLPLGFII-SPMGHAINDS------------SITS------E-----------
XP_016450789.1/1-246 P-SCIP-LLPLGFVV-TPVGQINN-L--------------DTNISPSEA----------N
XP_009597691.1/1-246 P-SCIP-LLPLGFVI-TPVGQINN-L--------------DTNISPSEA----------N
XP_009786380.1/1-246 P-SCIP-LLPLGFVV-TPVGQINN-C--------------GTNINPSEA----------N
XP_016494823.1/1-246 P-SCIP-LLPLGFVV-TPVGQINN-C--------------GTNINPSEA----------N
XP_016494822.1/1-246 P-SCIP-LLPLGFVV-TPVGQINN-C--------------GTNINPSEA----------N
XP_019230103.1/1-246 P-SCIP-LLPLGFVV-TPVGQINN-C--------------DTNINPSEA----------N
XP_016573090.1/1-250 P-SCIP-LLPLGFVI-TPVNGQTNNI------------NNSDNISSSEA----------N
XP_016573102.1/1-250 P-SCIP-LLPLGFVI-TPVNGQTNNI------------NNSDNISSSEA----------N
XP_006352911.1/1-250 P-SCIP-LLPLGFVI-TPINNGQANI------------NNCDNNVSGIE----------A
XP_015166604.1/1-250 P-SCIP-LLPLGFVI-TPINNGQANI------------NNCDNNVSGIE----------A
XP_015085651.1/1-250 P-SCIP-LLPLGFVI-TPINNGQVNM------------NNSDNVVSGTE----------A
XP_015085654.1/1-250 P-SCIP-LLPLGFVI-TPINNGQVNM------------NNSDNVVSGTE----------A
XP_004245919.1/1-250 P-SCIP-LLPLGFVI-TPINNGQVNM------------NNSDNNVSGTE----------A
XP_025888165.1/1-250 P-SCIP-LLPLGFVI-TPINNGQVNM------------NNSDNNVSGTE----------A
XP_011070327.1/1-236 P-SCIP-LLPLGFVI-VPMGQIANND------------SGI-A-----------------
XP_011070329.1/1-236 P-SCIP-LLPLGFVI-VPMGQIANND------------SGI-A-----------------
XP_012843107.1/1-236 P-SCIP-LLPLGFVV-IPLGQTNNYE------------GTNVI-----------------
XP_019423575.1/1-234 P-SCIA-LLPQGFMI-VPMDSPNIGT------------SEPVTGP---------------
XP_019423571.1/1-234 P-SCIA-LLPQGFMI-VPMDSPNIGT------------SEPVTGP---------------
XP_019435331.1/1-234 P-SCIA-LLPQGFII-VPMDTPNVGT------------SSEAVKG---------------
XP_003612811.1/1-239 P-SCIA-LLPQGFMI-VPMVSSNADT------------SSEQGVTGT-------------
XP_020978415.1/1-227 P-SCIA-LLPQGFII-TPMPPSTNNS------------SS--------------------
XP_015963259.1/1-227 P-SCIA-LLPQGFII-TPMPPSSNNS------------SS--------------------
XP_025697880.1/1-227 P-SCIA-LLPQGFII-TPMPPSSNNS------------SS--------------------
XP_025697882.1/1-227 P-SCIA-LLPQGFII-TPMPPSSNNS------------SS--------------------
XP_025697881.1/1-227 P-SCIA-LLPQGFII-TPMPPSSNNS------------SS--------------------
XP_020217931.1/1-233 P-SCIA-LLPQGFMI-VPMISSPNVD------------TS-----TDA------------
XP_007158253.1/1-232 P-SCIA-LLPQGFTI-VPMLSSPNVD------------TP-----INA------------
XP_017427665.1/1-232 P-SCIA-LLPQGFMI-VPMLSSPNVD------------TP-----INA------------
XP_017427666.1/1-232 P-SCIA-LLPQGFMI-VPMLSSPNVD------------TP-----INA------------
XP_014521371.1/1-232 P-SCIA-LLPQGFMI-VPMLSSPNVD------------TP-----ING------------
XP_014521373.1/1-232 P-SCIA-LLPQGFMI-VPMLSSPNVD------------TP-----ING------------
XP_006587604.1/1-242 P-SCIA-LLPQGFKI-VPMSSPPNNV------------DTPIIDAATN------------
NP_001352051.1/1-233 P-SCIA-LLPQGFKI-VPMLSSPITD--------------------TT------------
XP_018840870.1/1-239 P-SCIP-LLPMGFVI-VPVGSTGETT------------------NISGD----------G
XP_018852246.1/1-240 P-SCIP-LLPIGFVI-VPVGSTD-TT------------------NISGD----------G
XP_010053633.1/1-234 P-SHLP-LLPIGFSI-VPFGSGSPGS------------DSGA------------------
XP_021675948.1/1-236 P-SCIP-LLPLGFVI-VPVESTCNSN------------TVNE-----------------G
XP_012081064.1/1-228 P-SFIP-LLPLGFVI-LPVESTNATN----------------------------------
XP_011028126.1/1-235 P-SCIP-LLPLGFVI-VPVESSSAV----------------------SE----------G
XP_002303433.2/1-235 P-SCIP-LLPLGFVI-VPVESSSAV----------------------SE----------G
XP_015897558.1/1-247 P-SCIP-LLPLGFVI-VPVESTSVTT------------TPTTTATMATD----------G
XP_002277673.1/1-238 P-SCIP-LLPMGFAI-VPVVPNNDCN------------IMTT-----TD----------D
XP_022996123.1/1-236 S-SSIP-LLPIGFSI-VPVVGSTAD----------------------------------G
XP_023534245.1/1-236 S-SSIP-LLPIGFSI-VPVVDSTAD----------------------------------G
XP_022931244.1/1-236 S-SSIP-LLPIGFSI-VPVVDSTAD----------------------------------G
XP_022931242.1/1-236 S-SSIP-LLPIGFSI-VPVVDSTAD----------------------------------G
XP_022159691.1/1-235 P-SCIP-LLPIGFSI-IPVVGLTAD----------------------------------G
XP_011652640.1/1-232 P-SCIP-LLPIGFSI-VPIIGSTID----------------------------------G
XP_011652639.1/1-232 P-SCIP-LLPIGFSI-VPIIGSTID----------------------------------G
XP_008466007.1/1-232 P-SCIP-LLPIGFSI-VPILGSTVD----------------------------------G
XP_023511589.1/1-230 P-SCIP-LLPIGFSI-VPVVGSTVD----------------------------------G
XP_022963906.1/1-230 P-SCIP-LLPIGFSI-VPVVGSTVD----------------------------------G
XP_023511591.1/1-230 P-SCIP-LLPIGFSI-VPVVGSTVD----------------------------------G
XP_022963908.1/1-230 P-SCIP-LLPIGFSI-VPVVGSTVD----------------------------------G
XP_022967184.1/1-230 P-SCIP-LLPIGFSI-VPVVGSTID----------------------------------G
XP_022967186.1/1-230 P-SCIP-LLPIGFSI-VPVVGSTID----------------------------------G
XP_010518669.1/1-225 P-STIP-LLPLGFSV-VPVNPSDADA------------D---------------------
XP_010677365.1/1-235 P-CCIP-LLPTGFSI-IPLGQTNTTT------------NATNAEA---------------
XP_010677364.1/1-235 P-CCIP-LLPTGFSI-IPLGQTNTTT------------NATNAEA---------------
XP_021852084.1/1-226 P-SCIP-LLPTGFSI-IPLGRNNITP------------DATNTAE---------------
XP_021852076.1/1-226 P-SCIP-LLPTGFSI-IPLGRNNITP------------DATNTAE---------------
XP_021765591.1/1-233 P-SCIP-LLPTGFSI-IPLGQNNITP------------DATNNTD---------------
XP_021768254.1/1-233 P-SCIP-LLPTGFSI-IPLGRNNITP------------DAKNSTD---------------
XP_017232690.1/1-221 P-SCIP-ILPLGFVI-VPVNQNNGI-----------------------------------
XP_017232692.1/1-221 P-SCIP-ILPLGFVI-VPVNQNNGI-----------------------------------
XP_024021697.1/1-248 P-SCIR-ILPIGFSI-IPVEPTAAVA------------SPADGSS-SSA----------A
XP_010096931.1/1-248 P-SCIR-ILPIGFSI-IPVEPTAAVA------------SPADGSS-SSA----------A
XP_024021696.1/1-248 P-SCIR-ILPIGFSI-IPVEPTAAVA------------SPADGSS-SSA----------A
XP_017621310.1/1-230 P-SCIP-LLPLGFFI-TPMELMNDGG------------CKDE------------------
XP_016731150.1/1-230 P-SCIP-LLPLGFFI-TPMELMNDGG------------CKDE------------------
XP_012472199.1/1-230 P-SCIP-LLPLGFFI-TPMELMNDGG------------CKDE------------------
XP_016727305.1/1-230 P-SCIP-LLPLGFFI-TPMELMNDGG------------CKDE------------------
XP_016731145.1/1-230 P-SCIP-LLPLGFFI-TPMELMNDGG------------CKDE------------------
XP_012472200.1/1-230 P-SCIP-LLPLGFFI-TPMELVNDGG------------CKDE------------------
XP_012472201.1/1-230 P-SCIP-LLPLGFFI-TPMELVNDGG------------CKDE------------------
NP_001314348.1/1-230 P-SCIP-LLPLGFFI-TPMGLVNDGG------------CKDE------------------
XP_016727300.1/1-230 P-SCIP-LLPLGFFI-TPMELVNDGG------------CKDE------------------
XP_022738572.1/1-235 P-SHIP-LLPLGFSI-TPVELIRDAS------------SNEGKS----------------
XP_021297648.1/1-235 P-SCIP-LLPLGFFI-TPVELIRDAG------------DDQGKS----------------
XP_017973778.1/1-235 P-SCIP-LLPLGFFI-TPVELIRDAS------------DDQGKS----------------
XP_017973780.1/1-235 P-SCIP-LLPLGFFI-TPVELIRDAS------------DDQGKS----------------
XP_022717458.1/1-235 P-SCIP-LLPIGFYL-TPVELIRDAS-------------GEGKS----------------
XP_022717453.1/1-235 P-SCIP-LLPIGFYL-TPVELIRDAS-------------GEGKS----------------
XP_022717437.1/1-235 P-SCIP-LLPIGFYL-TPVELIRDAS-------------GEGKS----------------
XP_022756982.1/1-232 P-SCIP-LLPLGFFI-TPVELIRD----------------EGKS----------------
XP_004300207.1/1-230 P-SCIP-LLPLGFAI-VPVESPNKST------------TTT-------------------
XP_024169578.1/1-230 P-SCIP-ILPLGFAI-VPVESTTEST------------TTT-------------------
XP_008238183.1/1-249 P-SCIP-LLPLGFVI-VPLHPMESTC------------PTPNHLT-SSS----------P
XP_016651445.1/1-249 P-SCIP-LLPLGFVI-VPLHPMESTC------------PTPNHLT-SSS----------P
XP_020418924.1/1-249 P-SCIP-LLPLGFVI-VPLHPMESTG------------PTPNHLT-SSS----------P
XP_021804146.1/1-249 P-SCIP-LLPLGFVI-VPLHPMESTG------------PTPNHLT-SSS----------P
XP_008238184.1/1-248 P-SCIP-LLPLGFVI-VPLHPMESTC------------PTPNHLT-SSS----------P
XP_016651446.1/1-248 P-SCIP-LLPLGFVI-VPLHPMESTC------------PTPNHLT-SSS----------P
XP_007209851.1/1-248 P-SCIP-LLPLGFVI-VPLHPMESTG------------PTPNHLT-SSS----------P
XP_021804154.1/1-248 P-SCIP-LLPLGFVI-VPLHPMESTG------------PTPNHLT-SSS----------P
XP_017179076.1/1-239 P-SCIP-LLLLGFVI-VPVLPMQSST------------STAADHG-SSS----------P
XP_008356445.1/1-246 P-SCIP-LLPLGFVI-VPVQPMESSA------------ATAADHG-TSS----------P
XP_008356446.1/1-240 P-SCIP-LLPLGFVI-VPVQPMESSA------------ATAADHG-TSS----------P
XP_008356447.1/1-239 P-SCIP-LLPLGFVI-VPVQPMESSA------------ATAADHG-TSS----------P
XP_009373485.1/1-239 P-SCIP-LLPLGFVI-VPVQPIESSA------------ATAVDHG-TSS----------P
XP_009373484.1/1-240 P-SCIP-LLPLGFVI-VPVQPIESSA------------ATAVDHG-TSS----------P
XP_004512486.1/1-245 P-SCIA-LLPQGFMI-VPMVSSNIDT------------LSCLGNS-SDQ----------G
XP_023898631.1/1-242 P-SCIP-LLPIGFVI-IPVRSTTDTT------------NTTSG----DG----------N
XP_024465375.1/1-238 P-SCIP-LLPLGFVI-VPVESSSSTV------------SEGNS-----------------
XP_011018502.1/1-238 P-SCIP-LLPLGFVI-VPVESSSSNV------------SEGNS-----------------
XP_011018503.1/1-238 P-SCIP-LLPLGFVI-VPVESSSSNV------------SEGNS-----------------
XP_021893615.1/1-233 P-SCIP-LLPLGFSI-IPVETINSPA------------TVGDS-----------------
XP_024952893.1/1-247 P-SCIP-LLPLGFVI-VPVEPIKESN------------TTSGDGN-GNG----------N
XP_006471522.1/1-248 P-SCIP-LLPLGFVI-VPVEPIKESN------------TTSGDGN-GNG----------N
XP_006432745.2/1-248 P-SCIP-LLPLGFVI-VPVEPIKESN------------TTSGDGN-GNG----------N
XP_021640807.1/1-241 P-SCIP-LLPLGFVI-IPMESTCKTN------------TTV--NE-GNS----------I
XP_002518976.1/1-243 P-SCIP-LLPLGFVI-VPVESITSTS------------KDTGGNE-GNS----------I
XP_021598537.1/1-237 P-SCIP-ILPLGFVI-IPVESTCNTN------------TPSEGNS-AHS----------S
XP_021598535.1/1-239 P-SCIP-ILPLGFVI-IPVESTCNTN------------TPSEGNS-AHS----------S
XP_006659280.1/1-226 P-ATVF-LVSSGCVI-LPDCLDSCPL------------LPPPTAD---------------
XP_025801379.1/1-254 G-ASDF-PTPSGFAI-LPDGRGKACH------------APSP------------------
XP_023156326.1/1-229 H-ASVF-LLPSGFAV-LPDGHGRARH------------APSS------------------
NP_001349156.1/1-229 H-ASVF-LLPSGFAV-LPDGHGRARH------------APSS------------------
XP_021310588.1/1-246 H-ASVF-LMPSGFAV-LPDGHGRVRD------------APSS------------------
XP_020404516.1/1-224 T-SNLV-LLPSGFAI-LPDGHGRAHH------------AAAS------------------
XP_022684239.1/1-229 N-SAVF-LLPSGFAI-LPDGHAKAHR------------AAAA------------------
XP_020404892.1/1-233 R-SNVF-VLPSGFAI-LPDGHGRAHA------------GDTA------------------
XP_022684238.1/1-237 --NNIF-LVPSGFAI-LPDGHGKARY------------NAAS------------------
XP_025825251.1/1-229 --SSIF-LLPSGFAI-LPDGHGKAHY------------TAVS------------------
XP_020183090.1/1-242 R-ASLS-LLPSGFAI-LPDGVGDMQN------------DPLD------------------
XP_020194819.1/1-240 N-TSFS-LLPSGVVV-LPDGNAEPGA------------PPTS------------------
XP_024314575.1/1-244 H-TSFS-LSPCGFIV-LPDGQGQQSV------------QTIS------------------
XP_024314342.1/1-235 C-ASFS-TPPSGFAV-IPDGLNQQTS------------PWS-------------------
XP_024959674.1/1-192 P-DIVP-ILVFGAAI-LPWNP---------------------------------------
XP_019196338.1/1-215 --DNVP-IVPCGFAI-MPAEKPAKLG------------LS--------------------
XP_006646400.1/1-226 A-SGLS-LYPTGFSL-LPDGEAAR--------------ES--------------------
XP_015632244.1/1-226 R-GGPS-LYPTGFSL-LPDVEAAQ--------------DS--------------------
XP_024314625.1/1-231 E-GELP-LFPTGLAL-LPVGGTA---------------LQ--------------------
XP_020164122.1/1-228 S-NKVS-LYPTGFHL-LPVEDTA-------------------------------------
XP_025814677.1/1-221 M-SNIA-LFPAGFSL-VPLADPANA-----------------------------------
XP_022682466.1/1-225 I-NNIS-LFPAGFSL-VPLPDPANL-----------------------------------
XP_002456467.1/1-226 M-SNIS-LFPAGFSL-VPLTDPAGHD----------------------------------
XP_008674944.1/1-228 M-SSIN-LFPAGFSL-VPLTNPDPAP------------QE--------------------
XP_010523539.1/1-228 Y-SGCD-LLPSGFTI-MPGCQVGTAE------------AEAE------------------
XP_020873847.1/1-203 S--FSI-TYPCGFTI-MPGQNSG-D-----------------------------------
NP_001190248.1/1-203 L-SGVE-LLPSGFTI-IPCESQECF-----------------------------------
NP_196343.2/1-203    L-SGVE-LLPSGFTI-IPCESQECF-----------------------------------
XP_020876233.1/1-195 L-SGIE-LLPCGFTI-MPHGSQECF-----------------------------------
XP_010423235.1/1-188 F-SGVE-ILPFGFTI-IPNGLRESF-----------------------------------
XP_010452682.1/1-218 L-SRIE-ILPFGFTI-IPNGLQEIF-----------------------------------
XP_010491330.1/1-188 L-SGVE-ILPFGFTI-IPNGLRESF-----------------------------------
XP_006413300.2/1-224 S-YSMP-ILPSGFSI-LPDGGDTE------------------------------------
XP_022008499.1/1-216 S-SRVG-LLPIGLSI-VPYHG---------------------------------------
XP_022008502.1/1-215 S-SRVG-LLPIGFSI-VPYHG---------------------------------------
XP_022007635.1/1-226 I-SSVA-LLPSGFYI-VPGHGKGN------------------------------------
XP_022008501.1/1-224 I-SSVA-LLPSGLCI-VPGY--GE------------------------------------
XP_021986984.1/1-226 I-ASVA-LLPSGLCI-VPGYGYGE------------------------------------
XP_021996009.1/1-224 I-SLVA-LLPSGLCI-VPGY--GE------------------------------------
XP_021996011.1/1-224 I-SSVA-LLPSGLCI-VPGY--GE------------------------------------
XP_022026050.1/1-224 I-SSVA-LLPSGLCI-VPGY--GE------------------------------------
XP_022026083.1/1-224 I-SSVA-LLPSGLCI-VPGY--GE------------------------------------
XP_021722389.1/1-209 N-IDEIPLLPSGFAI-LSNKDSS-------------------------------------
XP_021847315.1/1-219 N-VDEIPLMPSGFAI-LSNKDHPN------------------------------------
XP_020224300.1/1-216 P-MVPK-VLPSGFLI-SKDNRTIVES------------SS--------------------
XP_021765248.1/1-224 C-SSIP-ILASGFAI-TPNVIGGRAA------------PS--------------------
XP_004510020.1/1-219 S-STIPL-FPSGFMI-SRDGQF-DHF------------DT--------------------
XP_004487953.1/1-231 S-STMPMIIPSGFMI-SSHDGGTINV------------EA--------------------
XP_008244376.1/1-218 W-PDHIPILPSGFSI-LPDGPILR------------------------------------
XP_008244380.1/1-218 W-PDHIPILPSGFSI-LPDGPILR------------------------------------
XP_012569676.1/1-224 D-LTLLEILPCGFVV-SPICPSNE------------------------------------
XP_019418393.1/1-229 L-SMVS-LLPSGITI-SEAVSTTNVA------------GSGE------------------
XP_019440118.1/1-223 S-SKVF-VLPSGITI-SELPESTVQ---------------EA------------------
XP_014517363.2/1-226 P-ARMS-IFPSGIVI-SKDVQSITHA------------SA--------------------
XP_012569675.1/1-231 S-STVS-LLSSGFVI-TEDSHSLFNA------------RI--------------------
XP_004486570.1/1-223 S-SMVS-LIPSGFFI-SEESHSLLND------------ETTS------------------
XP_003594569.3/1-220 L-SNVSLIIPSGFLI-SEDSKSLSKD----------------------------------
XP_024632692.1/1-220 L-SNVSLIIPSGFLI-SEDSKSLSKD----------------------------------
XP_025887127.1/1-229 S-SWVA-LFSNGIAI-APDCNRNLLA------------AND-------------------
XP_015078684.1/1-230 S-SWVA-LFSNGIAI-APDCNRNLFA------------AND-------------------
XP_025887126.1/1-230 S-SWVA-LFSNGIAI-APDCNRNLLA------------AND-------------------
XP_015078401.1/1-220 P-SSVA-LFPCGIAI-VPDSFQDS------------------------------------
XP_015158871.1/1-217 P-SGVA-LFPCGIAI-VPDSFQDYNT------------ANNF------------------
XP_015078778.1/1-220 P-SCVE-LLPNGISI-LPD----LSA------------NNNK------------------
XP_010321992.1/1-216 P-SCVE-LLPNGISI-LPD----LSA------------NNNK------------------
XP_004240735.1/1-216 P-SCVE-LLPNGISI-LPD----LSA------------NNNK------------------
XP_015169793.1/1-213 S-SFVG-LLPNGISI-VQD----YSA------------VDND------------------
XP_006285631.1/1-212 S-NYVP-LLPSGFSI-VPDG----------------------------------------
XP_023635531.1/1-198 S-NYVP-LLPSGFSI-VPDG----------------------------------------
NP_567722.1/1-217    S-DSVK-FLPSGFSI-VPDGVNGSYH----------------------------------
XP_020873337.1/1-219 S-DYVQ-LLPSGFSI-MPDGVPDRKG----------------------------------
XP_020873338.1/1-219 S-DYVQ-LLPSGFSI-MPDGVPDRKG----------------------------------
XP_023000325.1/1-225 P-STIT-TLPMGFSI-LPDGHPPRA-------------SVISK-----------------
XP_023000326.1/1-214 P-STIT-TLPMGFSI-LPDGHPPRA-------------SVISK-----------------
XP_022963997.1/1-214 P-STIT-TLPMGFSI-LPDGHPPRA-------------SVISK-----------------
XP_023513617.1/1-208 P-STIT-TLPMGFSI-LPDGHPPRA-------------SVISK-----------------
XP_021312124.1/1-225 S-SAVA-VLPFGFVV-MP--------------------ALITS-----------------
XP_012701492.1/1-243 S-SGVA-LLPCGFAV-MPDGLEPR-P------------SVITS-----------------
XP_025815339.1/1-229 S-SGVA-VLPCGFAV-MPDGLESR-P------------AVFTS-----------------
XP_015644459.1/1-237 S-SGVH-LLPCGFISVMPDGLESK-P------------AVITA-----------------
XP_024314016.1/1-228 S-SGVA-VLPCGFAV-VPDGLEYSRP------------AVITS-----------------
XP_020168333.1/1-225 S-SGVA-LLPCGFAV-VPDGLEAR-P------------AVIRS-----------------
XP_013589242.1/1-206 S-DSVQ-ILPSGFSI-LPDGGGET------------------------------------
XP_022548456.1/1-204 S-DSVQ-ILPSGFSI-LPDGGGET------------------------------------
XP_024632691.1/1-227 S-KELP-ILPYGLIV-CSKNQANLNA------------PF--G-----------------
XP_017256377.1/1-220 S-TDMA-ILGSGFTV-LPENPVE-------------------------------------
XP_009632057.1/1-224 S-DDVV-MLASGFAI-LPFSPEKTNF------------P---------------------
XP_016502932.1/1-224 S-DDVV-MLASGFAI-LPFSPEKTNF------------P---------------------
XP_019258997.1/1-221 S-DDVV-MLASGFAV-LPLSPEKTNF------------P---------------------
XP_009792862.1/1-224 S-DDVV-MLASGFAV-LPFSPEKTNF------------P---------------------
XP_016544784.1/1-213 S-DRVD-ILASGFAV-LPCTPR--------------------------------------
XP_015089689.1/1-213 T-DNVD-MLASGFAV-LPCTSE--------------------------------------
XP_025883585.1/1-213 T-DNVG-MLASGFAV-LPCTSE--------------------------------------
XP_011095790.1/1-221 A-DSVA-ILPSGFAV-LPEGSPD-------------------------------------
XP_020553953.1/1-221 A-DSVA-ILPSGFAV-LPEGSPD-------------------------------------
XP_022857758.1/1-208 S-DNVS-ILGSGFAV-LPGRTD--------------------------------------
XP_004487739.1/1-224 P-DNVI-VFPSGFAI-IPGGLPKDGD------------DQGK------------------
XP_003596663.1/1-221 P-DDVV-AFPSGFAI-IPGGLPRDGD------------KGKGN-----------------
XP_003596665.1/1-227 P-DNVV-AYPSGFAI-IPGGLPRDGD------------KGKGN-----------------
XP_020201972.1/1-216 P-DKVI-ILPSGFSI-LPGPLPGK---------------EDG------------------
XP_025980734.1/1-220 P-DRVM-ILPSGFSI-LPGRLQGD---------------EDR------------------
XP_007149715.1/1-218 P-DKVM-ILPSGFSI-LPERLEGD---------------EDR------------------
XP_017423636.1/1-218 P-DKVM-ILPSGFSI-LPERLQGD---------------EDR------------------
XP_022631965.1/1-218 P-DKVM-ILPSGFSI-LPERLHGD---------------EDR------------------
XP_014522034.2/1-218 P-DKVM-ILPSGFSI-LPERLHGD---------------EDR------------------
XP_025669173.1/1-223 P-NNVM-VLPSGFSI-LPCGFPSDYI------------DEK-------------------
XP_020963786.1/1-223 P-NNVM-VLPSGFSI-LPCGFPSDYI------------DEK-------------------
XP_025614692.1/1-223 P-NKVM-VLPSGFSI-LPCGFPSDYI------------DEK-------------------
XP_015936351.1/1-223 P-NKVM-VLPSGFSI-LPCGFPSDYI------------DEK-------------------
XP_019451675.1/1-226 P-NIVM-ILPSGFVI-CPSGFPRNDV------------DDKN------------------
XP_019465428.1/1-219 P-EIVM-MLPSGFTI-LPARFSD-----------------NN------------------
XP_004295798.2/1-220 P-DEFA-ILASGFSI-LPDRPMA-------------------------------------
XP_021807338.1/1-219 P-DHIP-ILPSGFSI-LPDRPIH-------------------------------------
XP_021807340.1/1-219 P-DHIP-ILPSGFSI-LPDRPIH-------------------------------------
XP_020417197.1/1-219 P-DHIP-ILPSGFSI-LPDRPIH-------------------------------------
XP_020417200.1/1-219 P-DHIP-ILPSGFSI-LPDRPIH-------------------------------------
XP_009333923.1/1-206 P-DNLA-ILASGFSI-LPDKPMH-------------------------------------
XP_017184363.1/1-206 P-DNLA-ILASGFSI-LPDKPKH-------------------------------------
XP_021892911.1/1-224 S-DWIS-ALPSGFVI-LPDRLSM-------------------------------------
XP_002278971.2/1-210 P-DHVP-ILPSGFSI-LPDGPTR-------------------------------------
XP_010644412.1/1-211 P-DNVP-VMPSGFSI-SPDGPTG-------------------------------------
XP_024450660.1/1-210 S-DLVN-ILPSGFVI-HPDGPLR-------------------------------------
XP_002522867.2/1-214 S-DDMN-ILPSGFVI-HPDKQAS-------------------N-----------------
XP_020534605.1/1-220 P-DYVN-ILPSGFVI-HPSHKPITSN---------------YN-----------------
XP_021685850.1/1-217 P-DYVN-ILPSGFVI-HPDRQVM-------------------N-----------------
XP_021594464.1/1-220 P-DYVN-ILPSGFVI-HPATQVM-------------------N-----------------
XP_018731177.1/1-223 P-DSVV-MLPSGFAI-LPEKPTV-------------------P-----------------
XP_024018593.1/1-217 P-DFVR-ILSSGFVI-LPDVSKM-------------------F-----------------
XP_022139420.1/1-219 P-DNVA-ILPSGFAV-LPDKPNM-------------------N-----------------
XP_022981426.1/1-220 P-DNVA-ILSSGFVV-LPDNLKM-------------------N-----------------
XP_022981427.1/1-220 P-DNVA-ILSSGFVV-LPDNLKM-------------------N-----------------
XP_022941489.1/1-220 P-DNVA-ILSSGFVV-LPDNLKM-------------------N-----------------
XP_022941488.1/1-220 P-DNVA-ILSSGFVV-LPDNLKM-------------------N-----------------
XP_023525599.1/1-220 P-DNVA-ILSSGFVV-LPDNLKM-------------------N-----------------
XP_023525597.1/1-220 P-DNVA-ILSSGFVV-LPDNLKM-------------------N-----------------
XP_008454681.2/1-220 P-DYVA-ILPSGFAI-LPDRPRM-------------------N-----------------
XP_011648553.1/1-223 P-DYVA-ILPSGFAI-LPDSPRM-------------------N-----------------
XP_018822340.1/1-211 P-DHVV-ILPSGFAI-LPDKATL-------------------E-----------------
XP_018822367.1/1-211 P-DHVV-ILPSGFAI-LPDKATL-------------------E-----------------
XP_023876961.1/1-210 P-DTVV-ILPSGFAI-LPDKPTP-------------------E-----------------
XP_023876962.1/1-210 P-DTVV-ILPSGFAI-LPDKPTP-------------------E-----------------
XP_024034242.1/1-215 P-EIVN-ILPCGFSI-LPDRPPL-------------------N-----------------
XP_015381349.2/1-215 P-EIVN-IFPCGFSI-LPDRPPL-------------------N-----------------
XP_022717683.1/1-216 P-KFAA-VLPSGFAI-LPDKPPG-------------------Q-----------------
XP_016729588.1/1-216 P-KFVS-ILPSGFSI-MPDKAPG-------------------Q-----------------
XP_016729589.1/1-216 P-KFVS-ILPSGFSI-MPDKAPG-------------------Q-----------------
XP_016702140.1/1-208 P-KFVS-ILPSGFSI-MPDKAPG-------------------Q-----------------
XP_012460158.1/1-216 P-KFVS-ILPSGFSI-MPDKAPG-------------------Q-----------------
XP_012460145.1/1-216 P-KFVS-ILPSGFSI-MPDKAPG-------------------Q-----------------
XP_017245631.1/1-197 PSILS--LSPSGFVI-SGAMSGGALV------------TVLLQ-----------------
XP_004956045.3/1-247 NTFVSF--LPSGFAI-LPDGHGKASQ------------ALVTA-----------------
XP_014660176.1/1-240 HASVDF-LLPSGFAV-LPDGHGKARR------------HAP-------------------
XP_017239609.1/1-213 G-SSLP-ILPSGFTI-LPHGSSLFTE----------------------------------
XP_023740378.1/1-206 T-SGVG-LLPCGLYI-VPTGS---------------------------------------
XP_020095129.1/1-235 PTSAIA-LLPSGFAI-HPDMKSGAEN------------GMA-------------------
XP_020258373.1/1-212 Q-ERIQ-LLPSGVTI-VPDGPYWMTN----------------------------------
XP_008812148.1/1-175 A-DYVA-LLPCGFTI-FPDLPHGMQD------------GID-------------------
XP_010930674.1/1-230 A-DYVA-LLPCGFTI-FPDLPYGMQD------------GLV-------------------
XP_010931877.1/1-230 A-DYVA-LLPCGFTI-FPDLPYGMQD------------GLV-------------------
XP_009380061.1/1-225 P-SYIH-ILPSGFTI-LPDGRFGVGG----------------------------GAST--
XP_010939971.1/1-229 P-SKIP-LLPSGFAV-LPDGQPMGG-----------------------------GAST--
XP_019710626.1/1-218 P-SKIP-LLPSGFAV-LPDGQPMGG-----------------------------GAST--
XP_008787891.1/1-229 P-SNVP-LLPSGFAI-LPDGQPVGG-----------------------------GAST--
XP_008787892.1/1-218 P-SNVP-LLPSGFAI-LPDGQPVGG-----------------------------GAST--
XP_008799024.1/1-230 P-SSIP-LLPSGFAI-LPDGQPTGG-----------------------------GAST--
XP_010936845.1/1-230 P-SKIP-LLPSGFAI-LPDGQPNGE-----------------------------GAST--
XP_003570542.1/1-223 P-SAIP-LLPSGFTI-LPDGSGGPG-----------------------------------
XP_004964922.1/1-229 P-SGIP-LLPSGFAI-LPDGRPGSGA---------------------------AGASS--
XP_002438026.1/1-230 P-SGIP-LLPSGFAI-LPDGRPGSGA---------------------------GASSS--
XP_020399989.1/1-232 P-SGIP-LLPSGFAI-LPDGRPGSSG---------------------------AGASS--
XP_008649207.1/1-222 P-SGIP-LLPSGFAI-LPGSGAGAS--------------------------------S--
XP_020256822.1/1-228 T-SYIP-LLPSGFAI-LPDGRPSNG----------------------------LAAST--
XP_009403550.1/1-228 P-SYVP-LLPSGFAI-LPDGRSAGGQ--------------------------G--ASS--
XP_020582158.1/1-232 P-SFIP-LLPSGFVV-FPDGRSGA----------------------------GINATS--
XP_020699118.1/1-240 S-SFIP-LLPSGFVI-LPDGRPGTGT--------------------------GINAAS--
XP_020600375.1/1-232 P-SFIP-LLPSGFIV-LPDGRPGTGS----------------------------DASS--
XP_020600376.1/1-232 P-SFIP-LLPSGFIV-LPDGRPGTGS----------------------------DASS--
XP_020684394.1/1-232 T-SYIP-LLPSGFTV-LPDGRPGTGS----------------------------NASS--
XP_017257147.1/1-219 S-SFIP-VLSSGFAI-LPDAGKKLG-----------------------------------
XP_010244428.1/1-230 T-SYIN-ILPSGFAI-YSDGRRDQAA------------EGA-----------SSSSL---
XP_010244355.1/1-230 T-SYIN-ILPSGFAI-YSDGRRDQAA------------EGA-----------SSSSL---
XP_017240757.1/1-236 P-SFIP-LLPSGFTI-SPDGKTDISQ------------HSK-----------SHDQGA--
XP_017245021.1/1-233 T-SYIP-LLPSGFTI-SPDGRTSTLQ------------T--------------VHEGA--
XP_012829269.1/1-233 T-SYIP-ILPSGFTI-SPDGRDAGGG------------GG--------------DAAA--
XP_022889938.1/1-236 P-SCIE-LLPSGFAI-SPDGRPNDQF------------QAR-----------GSDHGA--
XP_022897471.1/1-232 P-SSIA-LLPSGFTI-SPDGRQQDQL------------QAG-----------GIDYGA--
XP_009606250.1/1-235 T-TYIP-LLPSGFTI-SPDGRQGQRS---------------------------NEASY--
XP_016468230.1/1-235 T-TYIP-LLPSGFTI-SPDGRQGQRS---------------------------NEASY--
XP_019250773.1/1-235 T-TYIP-LLPSGFTI-SPDGRQGQRS---------------------------NEASC--
XP_009778533.1/1-235 T-TYIP-LLPSGFTI-SPDGRQGRRS---------------------------NEASC--
XP_016451582.1/1-235 T-TYIP-LLPSGFTI-SPDGRQGRRS---------------------------NEASC--
XP_016578176.1/1-240 T-TYIP-LLPSGFTI-LRDGRHDGGS---------------------------NEASM--
XP_006347370.1/1-231 T-TYIP-LLPSGFTI-SQDGRGSN------------------------------EALS--
XP_004241474.1/1-230 T-TYIP-LLPSGFTI-SPDGRGSD------------------------------EALS--
XP_015077748.1/1-230 T-TYIP-LLPSGFTI-SPDGRGSD------------------------------EALS--
XP_022037776.1/1-241 P-SYIP-LLPSGFVI-TTDGHQEIIS------------KSG-----------VASAST--
XP_024992062.1/1-237 P-SYIP-LLPSGFII-TTDGHQEITS------------AGG-----------ASTSNM--
XP_023772270.1/1-245 P-SYIP-LLPSGFII-TTDGRHEITS------------QGQ-----------GGASTS--
XP_012855673.1/1-237 P-TFLP-LLPSGFTI-SPDGGPHQLH------------HGQ-----------YDVAST--
XP_022858328.1/1-236 P-SYIP-LLPSGLTI-SPDGRPDQER------------GVN-----------G--AST--
XP_011072039.1/1-232 P-SYIP-LLPSGFTI-TSDGRADHLQ------------ASG-----------GDGAST--
XP_006306921.1/1-215 T-SCIP-ILPSGFAI-SPDGSS--------------------------------------
NP_564041.2/1-215    T-SYIP-ILPSGFAI-SPDGSS--------------------------------------
XP_020866823.1/1-216 T-SYIP-ILPSGFAI-SPDGSS--------------------------------------
XP_010476956.1/1-215 T-SCIP-ILPSGFAI-TPDGSS--------------------------------------
XP_010498167.1/1-217 T-SCIP-ILPSGFAI-TPDGSS--------------------------------------
XP_019091528.1/1-217 T-SCIP-ILPSGFAI-TPDGSS--------------------------------------
XP_013613976.1/1-227 T-SYIP-ILPSGFAV-LPEGGRNNQN------------AEI----------KAE------
XP_018432674.1/1-227 T-SYIP-ILPSGFAV-LPEGGRNNQT------------AEI----------KAE------
XP_018432667.1/1-227 T-SYIP-ILPSGFAV-LPEGGRNNQT------------AEI----------KAE------
XP_013641731.1/1-225 T-SYIP-ILPSGFAV-LPEGGRNNQN------------AEI----------KAE------
XP_009149243.1/1-227 T-SYIP-ILPSGFAV-LPEGGRNNQT------------AEI----------KAE------
XP_006416656.2/1-224 T-SYVP-ILPSGFAV-SPDGTRNNQL------------AEH----------KVE------
XP_018451633.1/1-224 T-SYIP-ILPSGFAI-LPDGSRNNQT------------PEL----------KVE------
XP_018451632.1/1-225 T-SYIP-ILPSGFAI-LPDGSRNNQT------------PEL----------KVE------
XP_018451634.1/1-195 T-SYIP-ILPSGFAI-LPDGSRNNQT------------PEL----------KVE------
XP_013690958.1/1-228 T-SYIP-ILPSGFAI-SPDGSRSSQI------------PEL----------KVE------
XP_009110376.1/1-227 T-SYIP-ILPSGFAI-SPDGSRSSQI------------PEL----------KVE------
XP_009110377.1/1-227 T-SYIP-ILPSGFAI-SPDGSRSSQI------------PEL----------KVE------
XP_013601964.1/1-227 T-SYIP-ILPSGFAV-SPDGSKNNQI------------PEL----------KVE------
XP_013653874.1/1-227 T-SYIP-ILPSGFAV-SPDGSRNDQI------------PEL----------KVE------
XP_018445004.1/1-237 T-SYIP-LLSSGFAI-SPDGNHNSLT------------AEQ----------GGGGAST--
XP_018445005.1/1-237 T-SYIP-LLSSGFAI-SPDGNHNSLT------------AEQ----------GGGGAST--
XP_013648946.1/1-228 T-SYIP-LLSSGFAI-SPDGNRNSPS------------AEQ----------GGA------
XP_009104812.1/1-228 T-SYIP-LLSSGFAI-SPDGNRNSPS------------AEQ----------GGA------
XP_013588523.1/1-228 T-SYIP-ILSSGFAI-SPDGNRNSPS------------AEQ----------GGA------
XP_013699248.1/1-228 T-SYIP-ILSSGFAI-SPDGNRNSPS------------AEQ----------GGA------
XP_018457296.1/1-228 T-SYIP-LLSSGFAI-SPDGNHSSGE-------------QG----------GGGA-----
XP_009105998.1/1-230 T-SYIP-LLSSGFAI-SPDGNHSSST------------TEQ----------G-GA-----
NP_001302850.1/1-231 T-SYIP-LLSSGFAI-SPDGNHSSST------------TEQ----------GGGA-----
XP_013698656.1/1-231 T-SYIP-LLSSGFAI-SPDGNHSSST------------TEQ----------GGGA-----
XP_013649619.1/1-231 T-SYIP-LLSSGFAI-SPDGNHSSST------------TEQ----------GGGA-----
XP_013592873.1/1-231 T-SYIP-LLSSGFAI-SPDGNHSSST------------TEQ----------GGGA-----
XP_006390565.1/1-233 T-SYIP-LLSSGFTI-SPDGSRTIE-------------QGG----------GA---ST--
XP_006301097.1/1-230 P-SYIP-LLSSGFAI-SPDGNGSTS-------------EQG----------GVGGAST--
XP_010428260.1/1-231 P-SYIP-LLSSGFAI-SPDGNGSTS-------------EQG----------GGA--ST--
XP_010471396.1/1-230 P-SYIP-LLSSGFAI-SPDGNGSTS-------------EQG----------GGA--ST--
XP_010416135.1/1-230 P-SYIP-LLSSGFAI-SPDGNGSTS-------------EQG----------GGA--ST--
NP_177479.1/1-227    P-SYIP-LLSSGFTI-SPDGNGSNS-------------EQG----------G-A--ST--
XP_020890987.1/1-228 P-SYIP-LLSSGFTI-SPDGNGSTS-------------EQG----------GGA--ST--
XP_010537520.1/1-232 P-SYIP-LLPSGFTI-SPDCSSSRIA------------TDD----------ENCA-ST--
XP_010540042.1/1-226 P-LFIP-LLPSGFTI-SPDGNSRITT------------MDQ----------GDCG-SS--
XP_010540046.1/1-196 P-LFIP-LLPSGFTI-SPDGNSRITT------------MDQ----------GDCG-SS--
XP_019432660.1/1-235 P-SSIP-LLPSGFSI-TPDGQDEQ---------------------------GGDGAST--
XP_019420678.1/1-233 P-SFIP-LMPSGFSI-SPDGQKHHG--------------------------GGDGAST--
XP_013452816.1/1-243 T-SHIP-VTPSGFTI-TSDGNTLDNN------------NNLQGSGNV----NADGAST--
XP_020215886.1/1-221 P-SHIP-LLPNGFSI-LPDGQGEQ---------------------------EGDGAST--
XP_020215888.1/1-221 P-SHIP-LLPNGFSI-LPDGQGEQ---------------------------EGDGAST--
XP_007160572.1/1-230 T-SCIP-LLPNGFTI-LPDGQAEQ---------------------------EGDGAST--
XP_017424661.1/1-212 T-SCIP-LLPNGFII-LPDGETE-------------------------------------
XP_017424660.1/1-230 T-SCIP-LLPNGFII-LPDGETEQ---------------------------EGDGAST--
XP_014508329.1/1-230 T-SCIP-LLPNGFTI-LPDGETEQ---------------------------EGDGAST--
XP_022632295.1/1-230 T-SCIP-LLPNGFTI-LPDGETEQ---------------------------EGDGAST--
XP_019225984.1/1-234 P-TYIP-LLPSGFTI-SPDGHQLDQD------------AAS-------------------
XP_016441514.1/1-233 P-TYIP-LLPSGFTI-SPDGHQLDQD------------AAS-------------------
XP_009587298.1/1-233 P-TYIP-LLPSGFTI-SPDGHQLDQD------------AAS-------------------
XP_016441515.1/1-233 P-TYIP-LLPSGFTI-SPDGHQLDQD------------AAS-------------------
XP_009786509.1/1-233 P-TYIP-LLPSGFTI-LPDGHQLDQD------------AAS-------------------
XP_016563952.1/1-234 P-TYIP-LLPSGFTI-SPDGRQLNAE------------AAS-------------------
XP_004235382.1/1-234 P-TYIP-LLPSGFTI-SPDGRQLDTD------------AAS-------------------
XP_015068940.1/1-234 P-TYIP-LLPSGFTI-SPDGRQLDTD------------AAS-------------------
XP_006364383.1/1-234 P-TYIP-LLPSGFTI-SPDGRQLDAD------------AAS-------------------
XP_019167409.1/1-245 P-TYIP-LLPSGFTI-SPDGRSDRGN------------STKINIDQA----S-----S--
XP_011086973.1/1-238 P-SYIP-LLPSGFTI-SPDGRPDHNQ------------AGGVTGG---------------
XP_010268321.1/1-230 P-SHIS-LLPSGFTI-SPDGQLDQVM------------GASTSS----------------
XP_010268322.1/1-230 P-SHIS-LLPSGFTI-SPDGQLDQVM------------GASTSS----------------
XP_010069492.1/1-236 P-SYIP-LLPSGFTI-SPDGGPDPGD------------GASSSS----------------
XP_002527933.1/1-229 P-SYIP-LLPSGFTI-CPDGRPDHGD------------GASTSS----------------
XP_021678368.1/1-229 P-AYIP-LLPSGFTI-SPDGHPDHGD------------GASSSS----------------
XP_021678369.1/1-229 P-AYIP-LLPSGFTI-SPDGHPDHGD------------GASSSS----------------
XP_021602738.1/1-229 P-SYIP-LLPSGFTI-SPDGGADHGD------------GASSSS----------------
XP_012079215.1/1-230 P-SYIP-LLPSGFTI-SPDGRLDHGD------------GASTSS----------------
XP_021687701.1/1-229 P-SYIP-LLPSGFTI-SPDGRPDNGN------------GASSSS----------------
XP_006374212.1/1-231 P-SYIP-LLPSGFAI-SPDGRPDQGD------------GASTSS----------------
XP_011035552.1/1-231 P-SYIP-LLPSGFTI-SPDGRPDQGD------------GASTSS----------------
XP_002317722.3/1-231 P-SYIP-LLPSGFTI-SPDGYPDQGD------------GASTSS----------------
XP_011000766.1/1-231 P-SYIP-LLPSGFTI-SPDGYPDQGD------------GASTSS----------------
XP_021907827.1/1-230 P-SYIP-LLPSGFTI-SSDGQSQQGD------------GASSSS----------------
XP_017635401.1/1-230 P-SYIP-LLPSGFTI-TPDGHLEQGD------------GASTSS----------------
XP_016686699.1/1-230 P-SYIP-LLPSGFTI-TPDGHLEQGD------------GASTSS----------------
XP_012440361.1/1-230 P-SYIP-LLPSGFTI-TPDGHLEQGD------------GASTSS----------------
NP_001313868.1/1-230 P-SYIP-LLPSGFTI-TPDGHLEQGD------------GASTSS----------------
XP_023919022.1/1-230 P-SYIP-LLPSGFTI-SPDGRAEQGD------------GASTSS----------------
XP_006478112.1/1-232 P-SYIP-LLPSGFTI-SPDGHLDQGD------------GASTSS----------------
XP_024042646.1/1-232 P-SYIP-LLPSGFTI-SPDGHLDQGD------------GASTSS----------------
XP_022715403.1/1-231 P-SYIP-LLPSGFTI-SPDGHPDQGD------------GPSTSS----------------
XP_022767009.1/1-255 P-SYIP-LLPSGFTI-SPDGHPDQGD------------GASTSS----------------
XP_022737050.1/1-231 P-SYIP-LLPSGFTI-SPDGHPDQGD------------GASTSS----------------
XP_017973835.1/1-231 P-SYIP-LLPSGFTI-SPDGHPDQGD------------GASTSS----------------
XP_015967008.1/1-238 P-SYIP-LVPSGFTI-TPDGNHHDHQ------------GGGDGA----------------
XP_025701434.1/1-239 P-SYIP-LVPSGFTI-TPDGNHHDHQ------------AGGDGA----------------
XP_020980110.1/1-239 P-SYIP-LVPSGFTI-TPDGNHHDHQ------------AGGDGA----------------
XP_019434881.1/1-231 P-SYIP-LLPSGFSI-SHDGQHD-QG------------G-VDGA----------------
XP_019458275.1/1-234 P-SYIP-LLPSGFSI-SPDGQQD-QG------------GGEDGA----------------
XP_019458276.1/1-234 P-SYIP-LLPSGFSI-SPDGQQD-QG------------GGEDGA----------------
XP_006584922.1/1-234 P-SYIP-LLPNGFTI-LPDGQPDQEG------------DDGAST----------------
XP_003524332.1/1-231 P-SYIP-LLPNGFTI-LPDGQPDQEG------------DGAST-----------------
XP_004515249.1/1-229 P-SYIP-LLPSGFTI-TSDGNQNDNN------------NNNNNN----------------
XP_019431381.1/1-229 P-SYIP-LLPSGFTI-APDGQTDQGH--------------GDG-----------------
XP_019436771.1/1-231 P-SYIP-LLPSGFTI-SPDGQSDEGG--------------GDGA----------------
XP_019430342.1/1-233 P-SYIP-LLPSGFTI-SPDGQNDQGG------------VGGDGA----------------
XP_006587436.1/1-240 P-SYIP-LLPSGFTI-SPDGQADQDG------------GGASTS----------------
XP_003549189.1/1-236 P-SYIP-LLPSGFTI-SPDGQADQDG------------GGASTS----------------
XP_020221986.1/1-232 P-SYIP-LLPSGFTI-SPDGQGEQDG------------AGASTS----------------
XP_007152598.1/1-234 P-SYIP-LLPSGFTI-SPDGQGEQDG------------GGASTS----------------
XP_017437542.1/1-234 P-SYIP-LLPSGFTI-SPDGQGEQDG------------GGASSS----------------
XP_014505752.1/1-234 P-SYIP-LLPSGFTI-SPDGQGEQDG------------GGASSS----------------
XP_015881991.1/1-234 P-SYIP-LLPSGFTI-TPDGRPDQGG------------GDGAST----------------
XP_004299483.1/1-232 P-SYIP-LLPSGFTI-TPDGRQDQGD------------TSASTS----------------
XP_024174647.1/1-232 P-SYIP-LLPSGFTI-SPDGPQDQGD------------TSASTS----------------
XP_008375041.1/1-227 P-SYIP-LLPSGFSI-SPDGQADQGD------------CASSSS----------------
XP_017188172.1/1-199 P-SYIP-LLPSGFSI-SPDGQADQGD------------CASSSS----------------
XP_009355964.1/1-227 P-SYIP-LLPSGFSI-SPDGRADQGD------------CASSSS----------------
XP_009355965.1/1-199 P-SYIP-LLPSGFSI-SPDGRADQGD------------CASSSS----------------
XP_023523434.1/1-228 P-SNIP-LLPSGFAI-LPDGRQDQGE------------GASSSS----------------
XP_022949138.1/1-227 P-SSIP-LLPSGFAI-LPDGRQDQGE------------GASSSS----------------
XP_022998050.1/1-227 P-SSIP-LLPSGFAI-LPDGRQDQGE------------GASSSS----------------
XP_002271012.1/1-231 P-SYIP-LLPSGFTI-SPDGRLDQGD------------GASSSS----------------
XP_010663934.1/1-231 P-SYIP-LLPSGFTI-SPDGRLDQGD------------GASSSS----------------
XP_010099679.1/1-237 P-SYIP-LLPSGFTI-SPDGRTEPGG------------GGDGAS----------------
XP_018823334.1/1-236 P-SYIP-LLPSGFAI-SPDGRPDLLP------------ADGAST----------------
XP_023001145.1/1-228 P-SSIP-LLPSGFTI-LPDGRRDQGE------------GTSSSL----------------
XP_023519889.1/1-228 P-SSIP-LLPSGFTI-LPDGRRDQGE------------GTSSSL----------------
XP_022924191.1/1-228 P-SSIP-LLPSGFTI-LPDGRRDQGE------------GTSSSL----------------
XP_018849090.1/1-235 P-SYIP-LLPSGFTI-SPDGRPDHMQ------------GGDVAS----------------
XP_008240378.1/1-228 P-SYIP-LLPSGFTI-SPDGRPEQGD------------GASTSS----------------
XP_007210460.2/1-228 P-SYIP-LLPSGFTI-SPDGRPEQGD------------GASTSS----------------
XP_021801797.1/1-228 P-SYIP-LLPSGFTI-SPDGRPEQGD------------GASTSS----------------
XP_022136988.1/1-228 P-SSIP-LLPSGFTI-LPDGRRDPGE------------GASSSS----------------
XP_016898817.1/1-228 P-SSIP-LLPSGFTI-LPDGRRDQGE------------GASSSS----------------
XP_004137568.1/1-228 P-SSIP-LLPSGFTI-LPDGRRDQGE------------GASSSS----------------
XP_015642098.1/1-238 P-SSIP-LLPSGFTI-LPDGRPGSAA------------GASTSSAGP----L-----A--
XP_020186741.1/1-220 P-SAIP-LLPSGFTI-LPDGRAS-------------------------------------
XP_025828617.1/1-229 P-SAIP-LLPSGFTI-LPDGRPGASP------------SSSSSAGP--------------
XP_004954072.1/1-226 P-SAIP-LLPSGFTI-LPDGRHGAS---------------SSSAGP--------------
XP_002452898.1/1-231 P-SAIP-LLPSGFTI-LPDGRPGASS------------SSSSAAGGT-------------
NP_001142912.2/1-230 P-SAIP-LLPSGFTI-LPDGRPGAPS------------SS-SAGGPL-------------
XP_020089796.1/1-231 P-SNIA-LLPSGFTI-LPDGRAGGVR------------GASTSSSAH-------------
XP_020089795.1/1-241 P-SNIA-LLPSGFTI-LPDGRAGGVR------------GASTSSSAH-------------
XP_019708739.1/1-228 P-SYIP-LLPSGFTI-LPDGRPGGGG------------GATTSSNP--------------
XP_008789232.1/1-228 P-SYIP-LLPSGFTI-LADGRPGGGG------------GATTSSNP--------------
XP_008789231.1/1-228 P-SYIP-LLPSGFTI-LADGRPGGGG------------GATTSSNP--------------
XP_008789233.1/1-200 P-SYIP-LLPSGFTI-LADGRPGGGG------------GATTSSNP--------------
XP_019704584.1/1-228 P-SYIP-LLPSGFTI-LPDGRPGGGG------------GATTSSNP--------------
XP_010917295.1/1-228 P-SYIP-LLPSGFTI-LPDGRPGGGG------------GATTSSNP--------------
XP_008806630.1/1-228 P-SYIP-LLPSGFTI-LPDGRAGGGG------------GATTSSNP--------------
XP_009388746.1/1-226 P-SYIP-ILPSGFTI-LPDGRAAA--------------GASSSSNP--------------
XP_018675467.1/1-228 P-SYVP-ILPSGFTI-LPDGRSAGGY------------GASSSSNP--------------
XP_018675466.1/1-228 P-SYVP-ILPSGFTI-LPDGRSAGGY------------GASSSSNP--------------
XP_009409108.1/1-228 P-SYVP-ILPSGFTI-LPDGRSAGGQ------------GASSSSNP--------------
XP_024625408.1/1-220 P-SYIQ-LLPSGFII-TSDGKQNDNN------------IQGGNSGND-------------
XP_010550261.1/1-219 P-SAVP-ILPSGFII-SRDGRSSPVT------------VE--------------------
XP_018816719.1/1-228 P-SNIP-ILPSGFVI-SSDGHPETGS------------AGAST--SK-------------
XP_008365756.2/1-218 S-SNMS-ILPSGFMI-SSDGRPEMGG------------A---------------------
XP_008381522.1/1-218 S-SNMS-ILPSGFMI-SSDGRPEMGG------------A---------------------
XP_018506060.1/1-218 S-SNIA-ILPSGFLI-SGDGRPEMGD------------A---------------------
XP_008362571.1/1-218 S-SNIA-ILPSGFLI-SGDGRPEMGD------------A---------------------
XP_023870350.1/1-229 S-SEVP-ILPSGFII-SGDGRPETGT------------IDAST--SA-------------
XP_016649213.1/1-222 S-SKIP-ILPSGFVI-SGDGHPETGP------------AA--------------------
XP_020415996.1/1-222 S-SKIP-ILPSGFVI-SGDGHPETGP------------AA--------------------
XP_021823813.1/1-222 S-SKIP-ILPSGFVI-SGDGHPETGP------------AA--------------------
XP_009367126.1/1-222 S-SNIP-ILPSGFVI-SGDGRAEVTG------------DS--------------------
XP_008348615.1/1-222 S-SNIP-ILPSGFVI-SGDGRAEVTG------------DS--------------------
XP_017179578.1/1-222 S-SNIP-ILPSGFVI-SGDGRAEITG------------DS--------------------
XP_009370612.1/1-224 S-SNIP-ILPSGFVI-SGDGRAETTG------------DS--------------------
XP_012845089.1/1-228 T-MKIP-ILPSGFII-SSDGRFTSNK------------GSGAS--AA-------------
XP_012845088.1/1-228 T-MKIP-ILPSGFII-SSDGRFTSNK------------GSGAS--AA-------------
XP_012845087.1/1-228 T-MKIP-ILPSGFII-SSDGRFTSNK------------GSGAS--AA-------------
XP_011085930.1/1-227 T-MRIP-ILPSGYVI-SSDGRA--NK------------GMGAS--SS-------------
XP_020551334.1/1-222 T-MRIP-ILPSGYVI-SSDGRA--NK------------GMGAS--SS-------------
XP_021297998.1/1-224 S-SNIP-ILPSGFII-TGDGRSELGV------------GANSS-----------------
XP_017974480.1/1-224 S-SNIP-ILPSGFIV-SGDGRSELGV------------GANST-----------------
XP_024448785.1/1-229 S-SIIP-ILPSGFVI-SGDGRPDTGG------------DSSTS--TS-------------
XP_024455385.1/1-229 S-SIIP-ILPSGFVI-SGDGRPDTGG------------DSSTS--TS-------------
XP_011005937.1/1-269 S-SIIP-ILPSGFVI-SGDGHPDTGG------------DSSTS--TS-------------
XP_011038413.1/1-229 S-SIIP-ILPSGFVI-SGDGRMDTRG------------NNSSF--TS-------------
XP_018731153.1/1-226 T-STVQ-ILPSGFTI-SSDGRANPGS------------KPSTS--TS-------------
XP_024190515.1/1-228 S-SDIP-ILPSGFII-SDDGRPDTGT------------SSNTN--P--------------
XP_012074722.2/1-231 S-LIIP-ILPSGFVI-SEDGRPDNSR------------ASTSA-----------------
XP_010089514.2/1-229 S-SAIP-ILPSGFII-SADGRGTNHR------------STKAA--ST-------------
XP_024949330.1/1-235 S-SNIP-ILPSGFVI-SSDGRQRDHY------------RASTS--TE-------------
XP_006421085.1/1-235 S-SNIP-ILPSGFVI-SRDGRQRDHY------------RASTS--TE-------------
XP_002268272.1/1-229 S-GNIP-ILPSGFIV-SGDGRSDSGA------------GCTSD--GN-------------
XP_004140733.2/1-228 P-SEIP-ILPSGFVI-TGDGRTHSGI------------GASTS--AT-------------
XP_008457114.1/1-228 P-SEIP-ILPSGFVI-TGDGRTHSGI------------GASTS--ST-------------
XP_008457113.1/1-229 P-SEIP-ILPSGFVI-TGDGRTHSGI------------GASTS--ST-------------
XP_011651208.1/1-229 P-SEIP-ILPSGFVI-TGDGRTHSGI------------GASTS--AT-------------
XP_022143367.1/1-228 S-SEIP-ILPSGFVI-SGDGRAHLGG------------GASTS--AT-------------
XP_022935490.1/1-228 P-SEIP-ILPSGFVI-TGDGRSHSGV------------GASTS--AT-------------
XP_022984101.1/1-228 P-SEIL-ILPSGFVI-TGDGRSHSGV------------GASTS--AT-------------
XP_022935488.1/1-229 P-SEIP-ILPSGFVI-TGDGRSHSGV------------GASTS--AT-------------
XP_022984098.1/1-229 P-SEIL-ILPSGFVI-TGDGRSHSGV------------GASTS--AT-------------
XP_022984102.1/1-229 P-SEIL-ILPSGFVI-TGDGRSHSGV------------GASTS--AT-------------
XP_015579295.1/1-234 S-SIIP-ILPSGFVI-SGDGRPDAGN------------VASTS--AN-------------
XP_002526595.2/1-234 S-SIIP-ILPSGFVI-SGDGRPDAGN------------VASTS--AN-------------
XP_015579294.1/1-234 S-SIIP-ILPSGFVI-SGDGRPDAGN------------VASTS--AN-------------
XP_021643520.1/1-232 S-SIIP-ILPSGFVI-SGDGRPDTGN------------GASTS--AN-------------
XP_021605433.1/1-231 S-SMIP-VLPSGFVI-SGDGRPDTGN------------GASTS--AN-------------
XP_023739098.1/1-228 --VCVP-LLPSGFVI-TTDGRHRSTM------------STSRKEGC--------------
XP_024966239.1/1-227 P-LYVP-LLPSGFAI-TTDGRQSSTT------------SPATGDSS--------------
XP_025813558.1/1-232 A-SGIP-LLPSGFAI-LPDARPGSGA------------GATSSSSV--------------
XP_020150479.1/1-234 P-SGIP-LLPSGFAI-WPAGVGG---------------GASTSAAA--------------
XP_003564122.2/1-224 T-ANIP-LLPSGFII-LPDGRNSVSA------------GGGAGSSG--------------
XP_006585049.2/1-225 T-SQLL-VLPSGFTI-SGDGHSNAFE------------GQ--------------------
XP_007143783.1/1-226 T-SLFP-ILPSGFTI-SRDGKANVPE------------GE--------------------
XP_007158859.1/1-226 T-SLFP-ILPSGFTI-SREGKANVPE------------GE--------------------
XP_020230815.1/1-227 S-STTS-IFPSGIVI-LDNEQSITNA------------PT--------------------
XP_006586851.1/1-227 S-STMP-FFPSGIVI-SEEGQSITNA------------RA--------------------
XP_021888058.1/1-234 T-MSIP-ILPSGFVI-STDGHQNNTG------------GASTS-----------------
XP_020886111.1/1-226 P-SHIP-ILPSGFII-SSDGRRSTVE------------D---------------------
XP_020886110.1/1-228 P-SHIP-ILPSGFII-SSDGRRSTVE------------D---------------------
XP_011469296.1/1-224 S-SRIP-ILPSGFII-SDDGCLGTGT------------S---------------------
XP_009367128.2/1-218 S-SNMS-ILPSGYMI-SRDGRPEMG-----------------------------------
XP_008348673.1/1-198 S-SNMS-ILPSGFMI-SRDGRPEMG-----------------------------------
XP_012832506.1/1-227 T-MKIP-ILPSGFVI-STDGRASSRG------------T---------------------
XP_012832508.1/1-227 T-MKIP-ILPSGFVI-STDGRASSRG------------T---------------------
XP_012832516.1/1-227 A-TKIP-ILPSGFVI-STDGRASSRG------------T---------------------
XP_012832518.1/1-227 A-TKIP-ILPSGFVI-STDGRASSRG------------T---------------------
XP_012835569.1/1-227 A-TKIP-ILPSGFVI-STDGRASSRG------------T---------------------
XP_012835571.1/1-226 A-TKIP-ILPSGFVI-STDGRA-SRG------------T---------------------
XP_019172066.1/1-226 P-IKFP-MLPSGFVI-SGDGRLEKRS------------S---------------------
XP_009631364.2/1-226 T-TQIP-ILPSGFII-SPDGRLAADR------------D---------------------
XP_016435660.1/1-229 T-TQIP-ILPSGFII-SPDGRLAADR------------D---------------------
XP_009771801.1/1-226 T-TQIP-ILPSGFII-SPDGRLAADR------------D---------------------
XP_016493115.1/1-226 T-TQIP-ILPSGFII-SPDGRLAADR------------D---------------------
XP_019248352.1/1-226 T-TQIP-ILPSGLII-SPDGRLAADR------------D---------------------
XP_025691463.1/1-225 T-TTMP-ILPSGFTI-CGDGSPPRGA------------F---------------------
XP_020995109.1/1-225 T-TTMP-ILPSGFTI-CGDGSPPRGA------------F---------------------
XP_016190415.2/1-225 T-TTMP-ILPSGFTI-CGDGSPPRGA------------F---------------------
XP_004486568.1/1-232 S-KELQ-ILPSGFVV-CSKGQPNVTL------------EE--------------------
XP_024632687.1/1-226 S-KELP-ILPYGFVV-CSKSQPNLNA------------P---------------------
XP_020977993.1/1-241 S-STIP-ILPSGFVV-CGDGRASMMS------------NSTTIIAPS----V--------
XP_025647253.1/1-241 S-STIP-ILPSGFVV-CGDGRASMMS------------NSTTIIAPS----V--------
XP_020997095.1/1-240 S-STIP-ILPSGFVV-CGDGRASMMS------------NS-TIIAPS----V--------
XP_025692389.1/1-228 S-STIP-ILPSGFVV-CGDGRASMMS------------NS-TIIAPS----V--------
XP_019418389.1/1-226 S-TLIP-ILPSGFVI-CGDGKPNATF------------G---------------------
XP_007147460.1/1-227 S-SMLP-VLPSGFVI-SPEGDPNAAL------------G---------------------
XP_014517364.1/1-227 S-SMLP-VLPSGFVI-CPDGEPNSAM------------G---------------------
XP_017435438.1/1-226 S-SMLP-VLPSGFVI-CPDGEPNSAM----------------------------------
XP_017435439.1/1-217 S-SMLP-VLPSGFVI-CPDGEPNSAM----------------------------------
XP_025981664.1/1-227 S-SMLP-VLPSGFVI-SADGEPNAAL------------E---------------------
XP_006586853.1/1-227 S-SMLP-VLPSGFVI-SADGEPNAAL------------G---------------------
XP_020230831.1/1-226 S-SVLP-ILPSGFVI-SADGEPNATL------------G---------------------
XP_004489663.1/1-217 T-VKMH-ILPSGFVV-CPNDQKSN--------------AT--------------------
XP_018489833.1/1-227 P-LKIP-ILPSGFTI-SSDNSGGS--------------MA--------------------
XP_013584487.1/1-219 P-LKIP-ILPSGFTI-SSDNSE--------------------------------------
XP_009130351.1/1-219 P-LNIP-ILPSGFTI-SSDNSE--------------------------------------
XP_013747906.2/1-219 P-LNIP-ILPSGFTI-SSDNSE--------------------------------------
XP_013747661.1/1-219 P-LNIP-ILPSGFTI-SSDNSE--------------------------------------
XP_013619678.1/1-219 P-LNIP-ILPSGFTI-SSDNNR--------------------------------------
XP_022551084.1/1-219 P-LNIP-ILPSGFTI-SSDNNR--------------------------------------
XP_009126392.1/1-219 P-LNIP-ILPSGFTI-SSDNNR--------------------------------------
XP_018437612.1/1-222 P-MNIA-ILASGFTI-SNDGRRSM--------------GA--------------------
XP_013591191.1/1-225 P-LKIP-ILSSGFTI-SNDGRRSM--------------VA--------------------
XP_022567005.1/1-225 P-LKIP-ILSSGFTI-SNDGRRSM--------------VA--------------------
XP_022547796.1/1-225 P-SKIP-ILSSGFTI-SNDGRRSM--------------VA--------------------
XP_009119205.1/1-225 P-LKIP-ILSSGFTI-SNDGRRSM--------------VA--------------------
XP_009119199.1/1-225 P-LKIP-ILSSGFTI-SNDGRRSM--------------VA--------------------
XP_006408322.1/1-232 A-SKIP-ILPSGFTI-SGDGRRSM--------------AA--------------------
XP_023642295.1/1-229 A-PNIP-ILPSGFII-SSDRRPST--------------T---------------------
XP_023642294.1/1-229 A-PNIP-ILPSGFII-SSDRRPST--------------T---------------------
NP_186976.2/1-228    P-SHIP-ILPSGFVI-SSDGRRST--------------V---------------------
XP_010507998.1/1-229 I-SDIP-ILPSGFII-SSDGRRST--------------V---------------------
XP_010485661.1/1-229 I-SDIP-ILPSGFII-SSDGRRST--------------V---------------------
XP_010450732.1/1-230 I-SDIP-ILPSGFII-SSDGRRST--------------V---------------------
XP_010463776.1/1-230 I-SDIP-ILPSGFII-SSDGRRST--------------V---------------------
XP_010463774.1/1-230 I-SDIP-ILPSGFII-SSDGRRST--------------V---------------------
XP_019092960.1/1-229 I-SDIP-ILPSGFII-SSDGRRST--------------V---------------------
XP_022551077.1/1-228 P-SGIP-ILPSGFII-SRDGHHFT--------------SP--------------------
XP_013619236.1/1-228 P-SGIP-ILPSGFII-SRDGHHFT--------------SP--------------------
XP_009126177.1/1-226 P-SGIP-ILPSGFII-SRDGHHFT--------------SP--------------------
XP_013652421.1/1-226 P-SGIP-ILPSGFII-SRDGHHFT--------------SP--------------------
XP_013652420.1/1-226 P-SGIP-ILPSGFII-SRDGHHFT--------------SP--------------------
XP_006400265.1/1-228 P-SGIA-ILPSGFII-SRDGRPSSTP------------AA--------------------
XP_013590801.1/1-226 P-SGIP-ILPSGFVI-SRDGRPSA--------------TA--------------------
XP_013651910.1/1-222 P-SDIP-ILPSGFVI-SRDGRPSA--------------TA--------------------
XP_009103958.1/1-222 P-SDIP-ILPSGVVI-SRDGRPSA--------------TA--------------------
XP_018444485.1/1-226 P-SGIP-ILPSGFII-SSDGRPSA--------------AA--------------------
XP_009121397.1/1-226 P-SSIP-ILPSGFVI-SRDGRPPS--------------AA--------------------
XP_013659428.1/1-226 P-SSIP-ILPSGFVI-SRDGRPPS--------------AA--------------------
XP_022565333.1/1-226 P-SSIP-ILPSGFVI-SRDGRPPS--------------AA--------------------
XP_013610327.1/1-226 P-SSIP-ILPSGFVI-SRDGRPPS--------------AA--------------------
XP_006286655.1/1-225 S-STIP-ILPSGFII-SCDGRPLS--------------AD--------------------
XP_010492677.1/1-224 P-SAIP-ILPSGFII-SRDGRPSS--------------AE--------------------
XP_010420445.1/1-224 P-STIP-ILPSGFII-SRDGRPSS--------------AE--------------------
XP_010453918.1/1-224 P-SAIP-ILPSGFII-SRDGRPNS--------------AE--------------------
XP_006303739.1/1-224 P-STIP-ILPSGFII-SRDS-RSF--------------PG--------------------
XP_020876074.1/1-222 P-NTIP-ILPSGFII-SRDG-RPS--------------EG--------------------
NP_197234.1/1-222    P-TTIP-ILPSGFMI-SRDG-RPS--------------EG--------------------
NP_174724.1/1-221    P-STIP-ILPSGFII-SRDS-HPS--------------SS--------------------
XP_020869876.1/1-222 P-STIP-ILPSGFII-SRDS-RPS--------------VG--------------------
XP_019091098.1/1-222 P-STIP-VLPSGFII-SRDS-RPS--------------VG--------------------
XP_010478910.1/1-223 P-STIP-VLPSGFII-SRDSRRPS--------------VG--------------------
XP_010501781.1/1-221 P-SSIP-VLPSGFII-SRDS-RPS--------------VG--------------------
XP_015089790.1/1-222 A-TKVD-IFPLGIII-SPDGRLASER------------G---------------------
XP_015089784.1/1-222 A-TKVD-IFPLGIII-SPDGRLASER------------G---------------------
XP_016549855.1/1-224 A-TKVS-ILPSGITI-SPDGQLASNR------------K---------------------
XP_015057403.1/1-221 A-TRVP-ILPSGIII-SPDGRLVSDR------------G---------------------
XP_019066399.1/1-221 A-TKVP-ILPSGIII-SPDGRLVSDR------------G---------------------
XP_015166059.1/1-216 A-TKVP-ILPSGIII-SPDGRLSSDR------------D---------------------
XP_019071012.2/1-216 V-KRVP-ILPSGITI-SPDGRLSSDR------------D---------------------
XP_015086709.1/1-216 V-KRVP-ILPSGITI-SPDGRLSSDR------------D---------------------
XP_015087432.1/1-219 A-TKVP-ILPSGIII-SPDGRLSSNS------------D---------------------
XP_004247342.2/1-219 A-TKVP-ILPSGIII-SPDGRLSSNS------------D---------------------
XP_025888717.1/1-219 A-TKVP-ILPSGIII-SPDGRLSSNS------------D---------------------
XP_015087599.1/1-221 A-TKVP-ILPSGIII-SPDGRLSSNR------------Y---------------------
XP_019071156.1/1-221 A-TKVP-ILPSGIII-SPDGRLSSNR------------Y---------------------
XP_019070762.2/1-220 A-TKVP-FLPSGIII-SPDGRLSSNR------------D---------------------
XP_015087071.1/1-220 A-TKVP-ILPSGIII-SPDGRLSSNR------------D---------------------
XP_025888845.1/1-217 A-MKVP-ILPSGIII-SPDGRLSSNR------------D---------------------
XP_015170367.1/1-232 A-KKVT-TLPSGFII-SPYGHYSLGR------------D---------------------
XP_025886268.1/1-232 V-NKVS-ILPSGFII-CPDGRHNLDT------------Y---------------------
XP_015072491.1/1-224 V-NKVS-ILPSGFII-CPDGRHNLDT------------Y---------------------
XP_010327374.2/1-219 A-EEVF-ILPSGIII-SPDGRLTD------------------------------------
XP_015089749.1/1-219 A-EEVF-ILPSGIII-SPDGRLTN------------------------------------
XP_015170971.1/1-209 A-KEIA-ILPSGIMI-SHDGRLGSDR------------N---------------------
XP_025887726.1/1-223 A-KEFA-ILPSGIII-SHDGRLGSDR------------N---------------------
XP_006586358.1/1-227 S-SLFP-ILPSGLTI-SWDYQSNKVP------------EG--------------------
XP_014617351.1/1-228 S-STMP-FFPSGIVI-SEEGQSITNA------------RA--------------------
XP_007147461.1/1-227 S-TTLA-IFPSGIVI-SKDEQSITNA------------SA--------------------
XP_017435408.1/1-226 S-AKMS-IFPSGIVI-SKDVQSITHA------------SA--------------------
XP_017215828.1/1-223 T-SDIA-ILPSGFSI-IPDGVESGPL------------LLSS------------------
XP_012856110.1/1-229 S-SKIP-VLPSGFAI-ISDGIESRPF------------VFTS------------------
XP_023738891.1/1-226 S-SNIA-ILPSGFAI-LPDGVETRPS------------LIRS------------------
XP_022017964.1/1-227 S-RSIS-ILPSGFSI-LPDGIESRPL------------VITS------------------
XP_022154996.1/1-226 P-GNIT-TLPTGFSI-LPDGHQSRPM------------VISS------------------
XP_008462169.1/1-225 S-GNIT-TLPTGFSI-LPDGHPTRPL------------VISS------------------
XP_004141841.1/1-226 S-GNIT-TLPTGFSI-LPDGHPTRPL------------VISS------------------
XP_020098514.1/1-228 S-SKVA-VLPYGFSI-LPDGLETKPL------------LITM------------------
XP_020098513.1/1-228 S-SKVA-VLPYGFSI-LPDGLETKPL------------LITM------------------
XP_020263950.1/1-229 S-SDMA-ILPSGFSI-LPDGIDSRPV------------VITS------------------
XP_020263948.1/1-255 S-SDMA-ILPSGFSI-LPDGIDSRPV------------VITS------------------
XP_009415234.2/1-232 S-SSLA-ILPSGFSI-LPDGLETRPL------------VITS------------------
XP_008789333.1/1-230 S-SNMA-ILPSGFSI-LPDELETRPL------------VITS------------------
XP_010920420.1/1-235 S-SNMA-ILPSGFSI-LPDELETRPL------------VITS------------------
XP_010920411.1/1-235 S-SNMA-ILPSGFSI-LPDELETRPL------------VITS------------------
XP_010920404.1/1-235 S-SNMA-ILPSGFSI-LPDELETRPL------------VITS------------------
XP_010521515.1/1-219 P-SDIQ-ILPSGFSI-IPDGEESRPL------------VIRS------------------
XP_013652056.1/1-227 P-SNIQ-ILPCGFSI-IPDGVESRQL------------VISS------------------
XP_009104428.1/1-227 P-SNIQ-ILPCGFSI-IPDGVESRQL------------VISS------------------
XP_018445476.1/1-226 P-SNIQ-ILPCGFSI-IPDGVESRQL------------VISS------------------
XP_013590784.1/1-226 P-SNIQ-ILPCGFSI-IPDGVESRQL------------VISS------------------
XP_013680822.1/1-226 P-SNIQ-ILPCGFSI-IPDGVESRQL------------VISS------------------
XP_006389720.2/1-226 P-SNIQ-ILPSGFSI-IPDGVESRPL------------VITS------------------
XP_023642973.1/1-226 P-SNIQ-ILPSGFSI-IPDGVESRPL------------VITT------------------
XP_020891640.1/1-226 P-SNIQ-ILPSGFSI-IPDGVESRPL------------VITS------------------
NP_001320267.1/1-226 P-SNIQ-ILPSGFSI-IPDGVESRPL------------VITS------------------
NP_565223.1/1-226    P-SNIQ-ILPSGFSI-IPDGVESRPL------------VITS------------------
NP_001185443.1/1-226 P-SNIQ-ILPSGFSI-IPDGVESRPL------------VITS------------------
XP_010473021.1/1-226 S-SSIQ-ILPCGFSI-IPDGVESRPL------------VITT------------------
XP_010430051.1/1-226 P-SNIQ-ILPCGFSI-IPDGVESRPL------------VITT------------------
XP_025617667.1/1-241 S-TNIP-ILPSGFSI-LPDGVDSRPL------------ITSTT-----------------
XP_015938429.1/1-242 S-TNIP-ILPSGFSI-LPDGVDSRPL------------ITSTT-----------------
XP_016176999.1/1-241 S-TNIP-ILPSGFSI-LPDGVDSRPL------------ITTST-----------------
XP_021862846.1/1-225 S-SNVP-VLPSGFSI-LPDGSESRPL------------VLTS------------------
XP_022979290.1/1-231 S-SSLT-ILPSGFSI-LPDGAVSRPP------------LLIT------------------
XP_022955725.1/1-233 S-SSLT-ILPSGFSI-LPDGAVSRPP------------LLIT------------------
XP_022998610.1/1-231 S-GSLT-ILPSGFSI-LPDGAESRS-------------LLIT------------------
XP_022998611.1/1-227 S-GSLT-ILPSGFSI-LPDGAESRS-------------LLIT------------------
XP_023523626.1/1-229 S-GSLT-ILPSGFSI-LPDGAESRS-------------LLIT------------------
XP_022948296.1/1-231 S-GSLT-ILPSGFSI-LPDGAESRS-------------LLIT------------------
XP_023523628.1/1-225 S-GSLT-ILPSGFSI-LPDGAESRS-------------LLIT------------------
XP_022948295.1/1-227 S-GSLT-ILPSGFSI-LPDGAESRS-------------LLIT------------------
XP_010249693.1/1-228 S-SNVA-ILPSGFSI-LPDGLETRPL------------VLTS------------------
XP_010254081.1/1-230 S-SNIA-ILPSGFSI-LPDGLETRSL------------VLTS------------------
XP_003529763.1/1-227 S-SNLA-ILPSGFSI-LPDGIEGRPL------------VITS------------------
XP_003533048.1/1-226 S-SNLA-ILPSGFSI-LPDGIEGRPL------------VISS------------------
XP_014634625.1/1-226 S-SNLA-ILPSGFSI-LPDGIEGRPL------------VISS------------------
XP_007150635.1/1-226 S-SNLA-ILPSGFSI-IPDGLESRPL------------VITS------------------
XP_014498860.1/1-226 S-SNLA-ILPSGFSI-IPDGLESRPL------------VITS------------------
XP_017425661.1/1-226 S-SNLA-ILPSGFSI-IPDGLESRPL------------VITS------------------
XP_020207725.1/1-226 S-SNLA-ILPSGFSI-IPDGLESRPL------------VITS------------------
XP_003546423.2/1-226 S-SNLA-ILPSGFSI-IPDGLESRPL------------VISS------------------
XP_003543638.1/1-226 S-SNLA-ILPSGFSI-IPDGLESRPL------------VISS------------------
XP_019457980.1/1-226 S-SNLA-ILPSGFSI-IPDGFESRPL------------LITS------------------
XP_003597736.1/1-226 S-SNLA-ILPSGFSI-VSDGLESRQM------------VITS------------------
XP_004486751.1/1-226 S-SNLV-ILPSGFSI-VPDGLESRPM------------VITS------------------
XP_016169615.1/1-226 S-SNLA-VLPSGFSI-VPDGMESRPV------------VITS------------------
XP_025611006.1/1-226 S-SNLA-VLPSGFSI-VPDGMESRPM------------VITS------------------
XP_020982478.1/1-227 S-SNLA-VLPSGFSI-VPDGMESRPM------------VITS------------------
XP_021892835.1/1-225 S-SSIA-ILPSGFSI-LPDGLESRPL------------VITS------------------
XP_012442963.1/1-227 S-SNMA-ILPSGFSI-LPDGLESRPL------------VISS------------------
NP_001314068.1/1-227 S-SNMA-ILPSGFSI-LPDGLESRPL------------VISS------------------
XP_016684379.1/1-227 S-SNMA-ILPSGFSI-LPDGLESRPL------------VISS------------------
XP_016730082.1/1-227 S-SNMA-ILPSGFSI-LPDGLESRPL------------VISS------------------
NP_001316938.1/1-227 S-SNMA-ILPSGFSI-LPDGLESRPL------------VISS------------------
XP_017606636.1/1-227 S-SNMA-ILPSGFSI-LPDGLESRPL------------VISS------------------
XP_022714992.1/1-229 S-SNMA-ILPSGFSI-LPDGLESRPL------------VITS------------------
XP_022721436.1/1-227 S-SSMA-ILPSGFSI-LPDGLESRPL------------VITS------------------
XP_022721435.1/1-227 S-SSMA-ILPSGFSI-LPDGLESRPL------------VITS------------------
XP_007024189.1/1-227 S-SNMA-ILPSGFSI-LPDGLESRPL------------VITS------------------
XP_021298731.1/1-227 S-SNMA-ILPSGFSI-LPDGLESRPL------------VITS------------------
XP_004302894.1/1-228 S-SNMA-VLSSGFSI-LPDGLESRPM------------VITS------------------
XP_011466687.1/1-235 S-SNMA-VLSSGFSI-LPDGLESRPM------------VITS------------------
XP_024180978.1/1-228 S-SNMA-VLSSGFSI-LPDGLESRPM------------VITS------------------
XP_024180977.1/1-235 S-SNMA-VLSSGFSI-LPDGLESRPM------------VITS------------------
XP_009353605.1/1-226 A-SNIA-ILPSGFSI-LPDGLESRPM------------VITS------------------
XP_008380909.1/1-226 A-SNIA-ILPSGFSI-LPDGLESRPM------------VITS------------------
NP_001280927.1/1-227 A-SNIA-ILPSGFSI-LPDGLESRPM------------VLTS------------------
XP_009338062.1/1-226 A-SNIA-ILPSGFSI-LPDGLESRPM------------VLTS------------------
XP_009338046.1/1-226 A-SNIA-ILPSGFSI-LPDGLESRPM------------VLTS------------------
XP_021829136.1/1-226 A-SNIA-ILPSGFSI-LPDGLESRPM------------VITS------------------
XP_008228560.1/1-226 A-SNIA-ILPSGFSI-LPDGLESRPM------------VITS------------------
XP_007217160.1/1-226 A-SNIA-ILPSGFSI-LPDGLESRPM------------VITS------------------
XP_022891611.1/1-228 S-SDIA-LLSSGFSV-LPDGIESRSL------------VITS------------------
XP_002304207.2/1-228 S-SNLA-ILPSGFSI-LPDGHESRPL------------VITS------------------
XP_011010522.1/1-228 S-SNLA-ILPSGFSI-LPDGHESRPL------------VITS------------------
XP_011009828.1/1-227 S-SNLA-ILPSGFSI-LPDGHESRPL------------VITS------------------
XP_024462179.1/1-228 S-SNLA-ILPSGFSI-LPDGHESRPL------------VITS------------------
XP_018837483.1/1-226 S-SNIA-ILSSGFSI-LPDGLESRPL------------VITS------------------
XP_018856342.1/1-226 S-SNIA-ILPSGFSI-LPDGLESRPL------------VITS------------------
XP_018856994.1/1-226 S-SNIA-ILPSGFSI-LPDGLESRPL------------VITS------------------
XP_018858360.1/1-226 S-SNIA-ILPSGFSI-LPDGLESRPL------------VITS------------------
XP_015867693.1/1-226 S-SNLA-ILPSGFSI-LPDGLDSRPL------------VITS------------------
XP_010062490.1/1-226 S-SNIA-ALPSGFSI-LPDGIESRPL------------VISS------------------
XP_010098082.1/1-228 S-SNLT-VLPSGFSI-LPDGVESRPL------------VITS------------------
XP_002284502.1/1-228 S-SSIA-ILPSGFSI-LPDGVESRPL------------VITS------------------
XP_023875609.1/1-226 S-SNMA-ILPSGFSI-LPDGVESRPL------------VITS------------------
XP_002516023.1/1-226 S-SSTA-ILPSGFAI-LPDGLETRAL------------VITS------------------
XP_021652755.1/1-228 S-SNVA-VLPSGFSI-LPDGLESRPL------------VITS------------------
XP_021654904.1/1-226 S-SNIA-VLPSGFSI-LPDGLESRPL------------VITS------------------
XP_021611342.1/1-229 S-SNIA-VLPSGFSI-LPDGLESRPL------------VITS------------------
XP_012073376.1/1-226 S-SNIA-VLPSGFSI-LPDGLESRPL------------VITS------------------
XP_006465622.1/1-226 S-SNIA-ILPSGFSI-LPDGLESRPL------------VITS------------------
XP_006426950.1/1-226 S-SNIA-ILPSGFSI-LPDGLESRPL------------VITS------------------
XP_009392419.1/1-229 S-SSLA-ILPSGFSI-LPDGLETRPL------------VITS------------------
XP_020518333.1/1-227 S-SNLP-ILPSGFSV-VSDGMESRPL------------VITS------------------
XP_018629167.1/1-227 S-SNVA-VLPSGFSI-LPDGLESRSL------------VITS------------------
XP_009611250.1/1-227 S-SNVA-VLPSGFSI-LPDGLESRSL------------VITS------------------
XP_009611249.1/1-227 S-SNVA-VLPSGFSI-LPDGLESRSL------------VITS------------------
XP_009611248.1/1-227 S-SNVA-VLPSGFSI-LPDGLESRSL------------VITS------------------
XP_016479156.1/1-227 S-SNVA-VLPSGFSI-LPDGLESRSL------------VITS------------------
XP_016479155.1/1-227 S-SNVA-VLPSGFSI-LPDGLESRSL------------VITS------------------
XP_016479154.1/1-227 S-SNVA-VLPSGFSI-LPDGLESRSL------------VITS------------------
XP_009778213.1/1-229 S-SNTA-VLPSGFSI-LPDGIESRGL------------VITS------------------
XP_016501674.1/1-229 S-SNTA-VLPSGFSI-LPDGIESRGL------------VITS------------------
XP_019224885.1/1-229 S-SNTA-VLPSGFSI-LSDGIESRGL------------VITS------------------
XP_019224886.1/1-229 S-SNTA-VLPSGFSI-LSDGIESRGL------------VITS------------------
XP_009592772.1/1-229 S-SNTA-VLPSGFSI-LPDGIESRGL------------VITS------------------
XP_016496403.1/1-229 S-SNTA-VLPSGFSI-LPDGIESRGL------------VITS------------------
XP_016562690.1/1-228 S-SNIA-MLPSGFSI-LPDGLESRPF------------VITS------------------
XP_006343080.1/1-229 S-SNIA-VLPSGFSI-LPDGLESRPF------------VITS------------------
XP_004235676.1/1-229 S-SNIA-ALPSGFSI-LPDGLESRPF------------VITS------------------
XP_020227921.1/1-228 S-SNLT-VLPSGFSI-VPDGIEARPF------------VITS------------------
XP_007135673.1/1-226 S-SNVA-ILPSGFSI-LPDGIEGRPM------------LIRS------------------
XP_014515030.1/1-226 S-SNLA-ILPSGFSI-LPDGVEERPL------------VIRS------------------
XP_017407449.1/1-226 S-SNLT-ILPSGFSI-LPDGVEERPL------------VIRS------------------
XP_010691342.1/1-224 S-SNVA-VLPSGFSI-LPDGTESRPL------------VLTS------------------
XP_021731998.1/1-222 S-GNVA-VLPSGFSI-LPDGSESRPL------------VLTS------------------
XP_021736870.1/1-224 S-GNVA-VLPSGFSI-LPDGSESRPL------------VLTS------------------
XP_024969955.1/1-229 S-SNIS-ILPSGFSI-LPDGVETRPL------------VITS------------------
XP_024969954.1/1-229 S-SNIS-ILPSGFSI-LPDGVETRPL------------VITS------------------
XP_024969953.1/1-229 S-SNIS-ILPSGFSI-LPDGVETRPL------------VITS------------------
XP_021984354.1/1-227 S-SNIA-ILPLGFSI-LPDGIETRPL------------VITS------------------
XP_017232370.1/1-228 S-SNIA-ILPSGFSI-LPDGLESRPL------------VISS------------------
XP_017216573.1/1-228 P-SDIA-ILPSGFSI-LPDGVESRPL------------LISS------------------
XP_017248777.1/1-227 S-SNVS-IMPSGFAI-LPEGIESRPL------------VISS------------------
XP_021627148.1/1-226 S-SNVA-VLPSGFSI-LPDGLESRPL------------VISS------------------
XP_021627146.1/1-226 S-SNVA-VLPSGFSI-LPDGLESRPL------------VISS------------------
XP_021627145.1/1-226 S-SNVA-VLPSGFSI-LPDGLESRPL------------VISS------------------
XP_019196965.1/1-228 S-SNTA-VLASGFSI-LPDGLDSRPL------------LITS------------------
XP_019196967.1/1-228 S-SNTA-VLASGFSI-LPDGLDSRPL------------LITS------------------
XP_022848789.1/1-228 S-SNIA-VLSSGFSI-LPDGIESRSL------------VITS------------------
XP_011072373.1/1-228 S-SNIA-VLPSGFSI-LPDGIESRPL------------VITS------------------
XP_021907286.1/1-215 P-QFTA-FLPSGFAI-FPNGPN--------------------------------------
XP_020885309.1/1-217 P-DFVA-LLPYGFAI-LPDGTG-QPG------------G---------------------
XP_020885307.1/1-217 P-DFVA-LLPYGFAI-LPDGTG-QPG------------G---------------------
NP_180796.2/1-218    P-DFVV-ILPSGFAI-FPDGTG-KPG------------G---------------------
XP_006296541.1/1-217 P-DAVA-LLPCGFAI-LPDGTV-QPG------------G---------------------
XP_010510028.1/1-215 P-DYVP-LLPSGIAI-LPDGMGDEHG----------------------------------
XP_010414006.1/1-214 P-DYVA-LLPSGFAI-LPDGMGDQPG----------------------------------
XP_010469602.1/1-219 P-DYVA-LLPSGFAI-LPDGMGDQPG------------G---------------------
XP_010417818.1/1-218 P-DYVA-LLPSGFAI-LPDGIGDQPG------------G---------------------
XP_010510869.1/1-220 P-DYVA-LLPSGFAI-LPDGMGDQPG------------G---------------------
XP_010510870.1/1-218 P-DYVA-LLPSGFAI-LPDGMGDQPG------------G---------------------
XP_018484769.1/1-219 P-DYVS-LLPMGFAI-LPDGTA-QHG------------R---------------------
XP_018484754.1/1-219 P-DYVS-LLPMGFAI-LPDGTA-QHG------------R---------------------
XP_013634882.1/1-219 P-GNVS-LLPMGFAI-LPDGTA-QPG------------R---------------------
XP_018513778.1/1-219 P-GNVS-LLPMGFAI-LPDGTA-QPG------------R---------------------
XP_009141217.1/1-219 P-GNVS-LLPMGFAI-LPDGTA-QPG------------R---------------------
XP_013747219.1/1-219 P-GNVS-LLPMGFAI-LPDGTA-QPG------------R---------------------
XP_024003849.1/1-217 S-DYVP-MLPSGFAI-LPDGMA-RPG------------R---------------------
XP_018437853.1/1-211 P-DYVP-LLPSGFAI-LPDGMA-QPG------------K---------------------
XP_009143975.1/1-211 P-KYVP-LLPSGFAI-LPDGTA-QPG------------K---------------------
XP_009143974.1/1-211 P-KYVP-LLPSGFAI-LPDGTA-QPG------------K---------------------
XP_022575029.1/1-211 P-KYVP-LLPSGFAI-LPDGTA-QPG------------K---------------------
XP_013748684.1/1-211 P-KYVP-LLPSGFAI-LPDGTA-QPG------------K---------------------
XP_013694194.1/1-211 P-KYVP-LLPSGFAI-LPDGTA-QPG------------K---------------------
XP_013634261.1/1-211 P-KCVP-LLPSGFAI-LPDGTA-QPG------------K---------------------
XP_013741430.1/1-210 P-KCVP-LLPSGFAI-LPDGTA-QPG------------K---------------------
XP_017254373.1/1-221 P-GNVR-ILPCGFAI-LPDGPRPNAG------------G---------------------
XP_017254240.1/1-222 P-TYVA-LLPSGFVI-HPDGPRHNVG------------G---------------------
XP_017221582.1/1-224 P-NYVD-VLPWGFAI-LPDGPGKNLG------------G---------------------
XP_017221584.1/1-224 P-NYVD-VLPWGFAI-LPDGPGKNLG------------G---------------------
XP_015941575.1/1-215 P-DELP-ILPSGMAI-LPDG----------------------------------------
XP_025620358.1/1-215 P-DELP-ILPSGMAI-LPDG----------------------------------------
XP_016176493.1/1-215 P-DELP-ILPSGMAI-LPDG----------------------------------------
XP_003610661.2/1-228 S-SRLT-LLPSGFAI-FPDGCITNGG------------P---------------------
XP_004496853.1/1-220 P-DCVV-MLPSGFVI-LPDGVPLNN-----------------------------------
XP_006353342.1/1-230 S-SCVT-FLPSGFAI-IPDCYENSNG------------VAAGN-----------------
XP_015070591.1/1-229 S-SCVT-FLPSGFAI-VPDCYENSNG------------VA-GI-----------------
XP_019068356.1/1-229 S-SCVT-FLPSGFAM-VPDCYENSNG------------VA-GI-----------------
XP_004234441.1/1-229 S-SCVT-FLPSGFAM-VPDCYENSNG------------VA-GI-----------------
XP_024926606.1/1-217 P-DYVR-ILPSGFAI-LPDTDINQMN------------G---------------------
XP_024525400.1/1-231 P-AHVA-VLPSGFVI-LPDGSEPHST---------------T------------------
XP_002965186.2/1-231 P-AHVA-VLPSGFVI-LPDGSEPHST---------------T------------------
XP_002962272.2/1-231 P-AHVA-VLPSGFVI-LPDGSEPHST---------------T------------------
XP_019441536.1/1-226 P-DFIS-MLPSGFAI-VPDEPIFSSP------------PQGG------------------
XP_019441537.1/1-226 P-DFIS-MLPSGFAI-VPDEPIFSSP------------PQGG------------------
XP_019441535.1/1-226 P-DFIS-MLPSGFAI-VPDEPIFSSP------------PQGG------------------
XP_019451758.1/1-229 Q-DCIG-LLPSGFAI-LPFVPNLSSS------------PLGG------------------
XP_012850835.1/1-238 P-NHVP-LLPSGFAI-LPDGPTGGQG------------R--G------------------
XP_020552079.1/1-225 P-SSIP-LLPSGFAI-LPDGPTGSQG------------R--G------------------
XP_020552078.1/1-225 P-SSIP-LLPSGFAI-LPDGPTGSQG------------R--G------------------
XP_020552080.1/1-223 P-SSIP-LLPSGFAI-LPDGPTGSQG------------R--G------------------
XP_009408807.2/1-226 P-NHVA-LLPSGFAI-LPDRPSSAVD------------H--D------------------
XP_006342404.1/1-225 P-DGVP-LLPSGFAI-FPDGPSSSTN------------Y--E------------------
XP_025887998.1/1-218 P-DVVP-LLPSGFAI-LPDGPSGSTN------------H--E------------------
XP_015081481.1/1-225 P-DVVP-LLPSGFAI-FPDGPSGSTN------------H--E------------------
XP_022000230.1/1-232 S-SCVA-LLPSGFAI-VPDCMAEP------------------------------------
XP_022000229.1/1-232 S-SCVA-LLPSGFAI-VPDCMAEP------------------------------------
XP_022037790.1/1-222 S-SYVA-LLPSGFAI-VPDCVAESGV----------------------------------
XP_022037789.1/1-222 S-SYVA-LLPSGFAI-VPDCVAESGV----------------------------------
XP_024991925.1/1-226 S-SCVA-LLPSGFAI-VPDCVAESGA------------PVT-------------------
XP_023760005.1/1-229 S-SCVA-LLPSGFAI-MPDCVGEAGA------------PVT-------------------
XP_023760001.1/1-229 S-SCVA-LLPSGFAI-MPDCVGEAGA------------PVT-------------------
XP_017251723.1/1-236 S-SSVA-LLPSGFAV-VPDCVPRSEG------------GSSCN-----------------
XP_017243321.1/1-239 S-ACIA-ILPSGFAI-VPDCFDSAGP------------SS--S-----------------
XP_017243319.1/1-239 S-ACIA-ILPSGFAI-VPDCFDSAGP------------SS--S-----------------
XP_017243320.1/1-239 S-ACIA-ILPSGFAI-VPDCFDSAGP------------SS--S-----------------
XP_017255563.1/1-234 S-SCVA-ILPSGFAI-VPDCFPDST--------------G--P-----------------
XP_017255562.1/1-234 S-SCVA-ILPSGFAI-VPDCFPDST--------------G--P-----------------
XP_012852237.1/1-226 P-SGVA-ILPSGFAI-APDCFPDSGE------------AHEGR-----------------
XP_012852238.1/1-226 P-SGVA-ILPSGFAI-APDCFPDSGE------------AHEGR-----------------
XP_012852236.1/1-226 P-SGVA-ILPSGFAI-APDCFPDSGE------------AHEGR-----------------
XP_012852235.1/1-226 P-SGVA-ILPSGFAI-APDCFPDSGE------------AHEGR-----------------
XP_011076208.1/1-227 S-SSIA-ILPSGFAI-VPDCYSNSNN------------SENGN-----------------
XP_019200484.1/1-228 S-SCVA-LLPSGFSI-VPDCFNSSGW------------PDDHP-----------------
XP_019200481.1/1-228 S-SCVA-LLPSGFSI-VPDCFNSSGW------------PDDHP-----------------
XP_022879034.1/1-223 P-SCVA-LLPSGFAI-LPDCSPVSDG------------HINYN-----------------
XP_018623115.1/1-238 S-SSVA-FLPSGIAI-VPDCFQDYSG------------ANNCN-----------------
XP_018623116.1/1-238 S-SSVA-FLPSGIAI-VPDCFQDYSG------------ANNCN-----------------
XP_016449095.1/1-238 S-SSVA-FLPSGIAI-VPDCFQDYSG------------ANNCN-----------------
XP_009771835.1/1-238 S-SSVA-FLPSGIAI-VPDCFQDYSG------------ANNCN-----------------
XP_009771833.1/1-238 S-SSVA-FLPSGIAI-VPDCFQDYSG------------ANNCN-----------------
XP_019248454.1/1-225 S-SSVA-FLPSGIAI-VPDCFQDYTG------------ANNCD-----------------
XP_016564677.1/1-235 S-SCVT-FLPSGFAI-VPDCFGNSNG------------VTS-------------------
XP_016564676.1/1-235 S-SCVT-FLPSGFAI-VPDCFGNSNG------------VTS-------------------
XP_009606534.1/1-237 S-SCVA-FLPSGFAI-VPDCFQNSN-----------------------------------
XP_016457239.1/1-237 S-SCVA-FLPSGFAI-VPDCFQNSN-----------------------------------
XP_009606533.1/1-237 S-SCVA-FLPSGFAI-VPDCFQNSN-----------------------------------
XP_009606532.1/1-237 S-SCVA-FLPSGFAI-VPDCFQNSN-----------------------------------
XP_016457238.1/1-237 S-SCVA-FLPSGFAI-VPDCFQNSN-----------------------------------
XP_016468064.1/1-237 S-SCVA-FLPSGFAI-VPDCFQNSN-----------------------------------
XP_009774404.1/1-237 S-SCVA-FLPSGFAI-VPDCFQNSN-----------------------------------
XP_016468062.1/1-237 S-SCVA-FLPSGFAI-VPDCFQNSN-----------------------------------
XP_016468061.1/1-237 S-SCVA-FLPSGFAI-VPDCFQNSN-----------------------------------
XP_009774402.1/1-237 S-SCVA-FLPSGFAI-VPDCFQNSN-----------------------------------
XP_009774401.1/1-237 S-SCVA-FLPSGFAI-VPDCFQNSN-----------------------------------
XP_019251817.1/1-237 S-SCVA-FLPSGFAI-VPDCFQNSN-----------------------------------
XP_019251815.1/1-237 S-SCVA-FLPSGFAI-VPDCFQNSN-----------------------------------
XP_019251816.1/1-237 S-SCVA-FLPSGFAI-VPDCFQNSN-----------------------------------
XP_011470395.1/1-233 S-TYVE-LLPSGFAI-LPCVTSEYVK----------------------------------
XP_004309219.2/1-233 S-TYVE-LLPSGFAI-LPCVTSEYVK----------------------------------
XP_024180881.1/1-233 S-TYVE-LLPSGFAI-LPCVTSDHVK----------------------------------
XP_024180911.1/1-233 S-TYVE-LLPSGFAI-LPCVTSDHVK----------------------------------
XP_019450525.1/1-216 S-AYLP-LLPSGFII-HPDGTG--------------------------------------
XP_019465255.1/1-230 S-AYLH-LLPSGFII-HPDGRSNNGS----------------S-----------------
XP_014502466.1/1-227 S-SFVS-LRPSGFAI-LPDGHSNNS-----------------------------------
XP_017438850.1/1-222 S-SFVS-LRPSGFAI-LPDGHSNNS-----------------------------------
XP_006599623.1/1-221 S-SFVP-LRPSGFAI-LPDGTSNNG-----------------------------------
XP_006599622.1/1-221 S-SFVP-LRPSGFAI-LPDGTSNNG-----------------------------------
XP_006599621.1/1-221 S-SFVP-LRPSGFAI-LPDGTSNNG-----------------------------------
XP_004515730.1/1-240 S-SFIS-LLPSGFSI-LPDGHSNNNC-------------VGTS-----------------
XP_013442370.1/1-239 S-SVIP-LLPSGFSI-LPDGHSNVNN------------IVGTS-----------------
XP_004252035.1/1-225 P-EHVS-LLPSGFAI-LPDGPPG-----------------VR------------------
XP_015060726.1/1-225 P-ESVS-LLPSGFAI-LPDGPPG-----------------VR------------------
XP_006589608.1/1-229 P-DYVA-LLPSGFAV-LPDGPALNVV------------PGPV------------------
XP_006589605.1/1-230 P-DYVA-LLPSGFAV-LPDGPALNVV------------PGPV------------------
XP_006849468.1/1-231 P-SYIA-LLPSGFTI-LPDGRPPDHN------------LIGGP-----------------
XP_020526173.1/1-231 P-SYIA-LLPSGFTI-LPDGRPPDHN------------LIGGP-----------------
XP_020109515.1/1-228 P-SNVA-LLPSGFTI-LPDGRPGGG---------------GGA-----------------
NP_001310555.1/1-224 P-SAIP-LLPSGFSI-LPDGRPGAS---------------SSR-----------------
XP_010544375.1/1-225 S-SFVA-LLPSGFAI-LPDGGGPHDG------------AGE-------------------
NP_001314001.1/1-230 S-SHVA-LLPSGFAI-LPGVQT--------------------D-----------------
XP_016732454.1/1-230 S-SHVA-LLPSGFAI-LPGVQT--------------------D-----------------
XP_016732453.1/1-230 S-SHVA-LLPSGFAI-LPGVQT--------------------D-----------------
XP_012484067.1/1-230 S-SHVA-LLPSGFAI-LPGVQT--------------------D-----------------
XP_012484066.1/1-230 S-SHVA-LLPSGFAI-LPGVQT--------------------D-----------------
XP_017631791.1/1-230 S-SHVA-LLPSGFAI-LPGVQT--------------------D-----------------
XP_017631789.1/1-230 S-SHVA-LLPSGFAI-LPGVQT--------------------D-----------------
XP_016728129.1/1-230 S-SHVA-LLPSGFAI-LPGVQT--------------------D-----------------
XP_016728128.1/1-230 S-SHVA-LLPSGFAI-LPGVQT--------------------D-----------------
XP_021671684.1/1-240 S-NYVA-LLPSGFVI-LSDDPISQGG------------ANFCN-----------------
XP_021671683.1/1-240 S-NYVA-LLPSGFVI-LSDDPISQGG------------ANFCN-----------------
XP_021671685.1/1-240 S-NYVA-LLPSGFVI-LSDDPISQGG------------ANFCN-----------------
XP_002318231.3/1-240 S-TYVA-LLPSGFVI-LPDNSFSNGE------------PSNSD-----------------
XP_024438602.1/1-240 S-TYVA-LLPSGFVI-LPDNSFSNGE------------PSNSD-----------------
XP_024442055.1/1-239 S-TYVA-LLPSGFVI-LPGNSFSNGE------------PNNCN-----------------
XP_002322460.1/1-239 S-TYVA-LLPSGFVI-LPGNSFSNGE------------PNNCN-----------------
XP_011048682.1/1-239 S-TYVA-LLPSGFVI-LPGNSFSNGE------------PNNCN-----------------
XP_011048684.1/1-239 S-TYVA-LLPSGFVI-LPGNSFSNGE------------PNNCN-----------------
XP_011048683.1/1-239 S-TYVA-LLPSGFVI-LPGNSFSNGE------------PNNCN-----------------
XP_019081404.1/1-234 S-SFVA-LLPSGFAI-VPDGSSGYGD------------DWSGK-----------------
XP_003634081.1/1-234 S-SFVA-LLPSGFAI-VPDGSSGYGD------------DWSGK-----------------
XP_008377249.1/1-231 S-AYVA-LLPSGFAI-LPGGPPGYEV------------VKSEG-----------------
XP_017189459.1/1-231 S-AYVA-LLPSGFAI-LPGGPPGYEV------------VKSEG-----------------
XP_008377244.1/1-231 S-AYVA-LLPSGFAI-LPGGPPGYEV------------VKSEG-----------------
XP_009371302.1/1-231 S-AYVA-LLPSGFAI-LPGGPPGYEV------------VKSEG-----------------
XP_009371299.1/1-231 S-AYVA-LLPSGFAI-LPGGPPGYEV------------VKSEG-----------------
XP_008376545.1/1-231 S-AYVA-LLPSGFAI-LPGGPPGYGV------------VKSEG-----------------
XP_008376544.1/1-231 S-AYVA-LLPSGFAI-LPGGPPGYGV------------VKSEG-----------------
XP_017188609.1/1-231 S-AYVA-LLPSGFAI-LPGGPPGYGV------------VKSEG-----------------
XP_022945426.1/1-238 S-AYVS-LLPSGFAI-LPDGPSIYAS------------TNNDK-----------------
XP_022945425.1/1-238 S-AYVS-LLPSGFAI-LPDGPSIYAS------------TNNDK-----------------
XP_023541229.1/1-238 S-AYVS-LLPSGFAI-LPDGPSNYAS------------TNNDK-----------------
XP_023541228.1/1-238 S-AYVS-LLPSGFAI-LPDGPSNYAS------------TNNDK-----------------
XP_022968414.1/1-238 S-AYVS-LLPSGFAI-LPDGPSNYAC------------TNNDK-----------------
XP_022968413.1/1-238 S-AYVS-LLPSGFAI-LPDGPSNYAC------------TNNDK-----------------
XP_023552381.1/1-232 S-AYVS-LLPSGFAI-LPNA-----T-------------NNDK-----------------
XP_022922479.1/1-232 S-AYVS-LLPSGFAI-LPNA-----T-------------NNDK-----------------
XP_023552380.1/1-232 S-AYVS-LLPSGFAI-LPNA-----T-------------NNDK-----------------
XP_022922478.1/1-232 S-AYVS-LLPSGFAI-LPNA-----T-------------NNDK-----------------
XP_022984644.1/1-232 S-AYVS-LLPSGFAI-LPNA-----T-------------NNDK-----------------
XP_022984643.1/1-232 S-AYVS-LLPSGFAI-LPNA-----T-------------NNDK-----------------
XP_008439185.1/1-242 S-AYVS-LLPSGFAI-LPIDQSNYAC-------------TNDQ-----------------
XP_008439184.1/1-242 S-AYVS-LLPSGFAI-LPIDQSNYAC-------------TNDQ-----------------
XP_011651140.1/1-242 S-AYVS-LLPSGFAI-LPIDQSNYAC-------------TNGE-----------------
XP_004140784.1/1-242 S-AYVS-LLPSGFAI-LPIDQSNYAC-------------TNGE-----------------
XP_009391954.1/1-234 S-AHVA-LLPSGFAI-VPDGAGYRGE------------LTE-------------------
XP_003581542.2/1-227 S-ASVP-LLPSGFVI-LPDGPT--------------------------------------
XP_006652658.1/1-224 S-TCVA-LLPSGFAI-LPAGPN--------------------------------------
XP_015637147.1/1-225 S-SCVA-LLPSGFAI-LPAGPS--------------------------------------
XP_025823430.1/1-225 S-TYVA-LLPSGFAI-LPGGS--S------------------------------------
XP_004978185.1/1-225 S-TNVA-LLPSGFAI-LPGRS--C------------------------------------
XP_021317935.1/1-228 S-TNVA-LLPSGFVI-LPDGSSSS------------------------------------
NP_001336434.1/1-226 S-KNVA-LLPSGFVI-LPAGST--------------------------------------
XP_019709552.1/1-234 P-AYVA-FLPSGFAI-LPDGSGGRAT------------TVKSR-----------------
XP_015696802.1/1-257 S-AYVS-LLPSGFAI-LPDGHNS-AS------------PSPVDV-----------GSA--
XP_015612621.1/1-258 S-AYVS-LLPSGFAI-LPDGHNNGAS------------PSPAEV-----------GSG--
XP_004957413.1/1-253 S-AYVS-LLPSGFAI-LPDGHSQPSS------------NTAQG-------------S---
XP_025801167.1/1-251 S-AYVS-LLPSGFAI-LPDGHSPPS-------------NAAQG-------------S---
NP_001105125.2/1-256 S-AYVS-LLPSGFAI-LPDGHCQSPN------------TAHQG-------------S---
XP_002462701.1/1-258 S-AYVS-LLPSGFAI-LPDGHCQSSN------------P-AQG-------------S---
XP_020262629.1/1-222 S-AYVA-LLPSGFSI-VPDGAHNK------------------------------------
XP_020699782.1/1-235 S-SYVA-LLPSGFAI-LPDGSGG-------------------------------------
XP_020699776.1/1-235 S-SYVA-LLPSGFAI-LPDGSGG-------------------------------------
XP_020573166.1/1-235 S-SYVA-LLPSGFSI-LPDGSGG-------------------------------------
XP_020187432.1/1-228 P-ASIM-LLPSGFAI-LPGGPSMSGD----------------------------------
XP_025656795.1/1-233 S-TYVA-LIPSGFTI-HPDGHSGTQD----------------------------------
XP_025656792.1/1-233 S-TYVA-LIPSGFTI-HPDGHSGTQD----------------------------------
XP_025656794.1/1-233 S-TYVA-LIPSGFTI-HPDGHSGTQD----------------------------------
XP_020979736.1/1-233 S-TYVA-LIPSGFTI-HPDGHSGTQD----------------------------------
XP_020999151.1/1-233 S-TYVA-LIPSGFTI-HPDGHSGTQD----------------------------------
XP_015967451.1/1-233 S-TYVA-LIPSGFTI-HPDGHSGTQD----------------------------------
XP_020999150.1/1-233 S-TYVA-LIPSGFTI-HPDGHSGTQD----------------------------------
XP_025701970.1/1-233 S-TYVA-LIPSGFTI-HPDGHSGTQD----------------------------------
XP_025701968.1/1-233 S-TYVA-LIPSGFTI-HPDGHSGTQD----------------------------------
XP_025701969.1/1-233 S-TYVA-LIPSGFTI-HPDGHSGTQD----------------------------------
XP_020221720.1/1-222 S-SFVA-LRPSGFAI-LPDGHPVNSD----------------------------------
XP_007152327.1/1-222 S-SFVA-LRPSGFAI-LPDGHSNNSD----------------------------------
XP_006587345.1/1-222 S-SFVA-LRPSGFAI-LPDGASNNGD----------------------------------
XP_006587344.1/1-222 S-SFVA-LRPSGFAI-LPDGASNNGD----------------------------------
XP_010035104.1/1-239 P-NSVA-LLPSGFAL-LPDSPSHIDG----------------------------------
XP_021765162.1/1-234 S-DYLA-LLPSGFAV-ADAPIHPNVL----------------------------------
XP_021765160.1/1-234 S-DYLA-LLPSGFAV-ADAPIHPNVL----------------------------------
XP_021730123.1/1-234 S-DYLS-LLPSGFAV-ADAPIHPNVL----------------------------------
XP_021842103.1/1-232 S-DYLA-LLPSGFAI-ADAPINPNGL----------------------------------
XP_021842106.1/1-232 S-DYLA-LLPSGFAI-ADAPINPNGL----------------------------------
XP_021842105.1/1-232 S-DYLA-LLPSGFAI-ADAPINPNGL----------------------------------
XP_021842104.1/1-232 S-DYLA-LLPSGFAI-ADAPINPNGL----------------------------------
XP_010693471.1/1-235 S-DYLA-LLPSGFAI-ADAAILPNAL----------------------------------
XP_019107938.1/1-235 S-DYLA-LLPSGFAI-ADAAILPNAL----------------------------------
XP_010693469.1/1-235 S-DYLA-LLPSGFAI-ADAAILPNAL----------------------------------
XP_019107937.1/1-235 S-DYLA-LLPSGFAI-ADAAILPNAL----------------------------------
XP_020163116.1/1-258 S-AYVS-LLPSGFAI-LPDGHTAPAA------------ATDPSP-----------QGS--
XP_020163109.1/1-258 S-AYVS-LLPSGFAI-LPDGHTAPAA------------ATDPSP-----------QGS--
XP_003578475.1/1-267 S-AYVS-LLPSGFAI-LPDGHNTPPG------------AVVD-P-----------AGS--
XP_011012369.1/1-242 S-TYVA-LLPSGFVI-LPDNSFSNGE----------------------------------
XP_011012370.1/1-242 S-TYVA-LLPSGFVI-LPDNSFSNGE----------------------------------
XP_008234278.1/1-230 S-AYVA-LLPSGFAI-LPGGPPGYGM----------------------------------
XP_021827117.1/1-230 S-AYVA-LLPSGFAI-LPGGPPGYGM----------------------------------
XP_021827116.1/1-230 S-AYVA-LLPSGFAI-LPGGPPGYGM----------------------------------
XP_020413581.1/1-230 S-AYVA-LLPSGFAI-LPGGPPGYGM----------------------------------
XP_007219555.2/1-230 S-AYVA-LLPSGFAI-LPGGPPGYGM----------------------------------
XP_018456926.1/1-230 S-ANVA-LLPSGFSI-SPDGSSSSSS----------------------------------
XP_013613235.1/1-226 S-ANVA-LLPSGFSI-SPDGS---------------------------------------
XP_013686466.1/1-227 S-ANVA-LLPSGFSI-SPDGS----S----------------------------------
XP_009106897.1/1-227 S-ANVA-LLPSGFSI-SPDGS----S----------------------------------
XP_006401857.1/1-229 S-ANVP-LLPSGFSI-LPDGSSSS------------------------------------
XP_020871109.1/1-230 S-AYVA-LLPSGFSI-LPDGSSSSS-----------------------------------
NP_001332093.1/1-229 S-AYVA-LLPSGFSI-LPDGSSS-S-----------------------------------
NP_200030.1/1-229    S-AYVA-LLPSGFSI-LPDGSSS-S-----------------------------------
XP_006282099.1/1-225 S-GYVA-LLPSGFSI-LPDGS-S-L-----------------------------------
XP_019095386.1/1-229 S-VYVA-LLPSGFSI-FPDGSSS-L-----------------------------------
XP_019095387.1/1-229 S-VYVA-LLPSGFSI-FPDGSSS-L-----------------------------------
XP_019091174.1/1-228 S-AYVA-LLPSGFSI-LPDGS-S-L-----------------------------------
XP_019091179.1/1-228 S-AYVA-LLPSGFSI-LPDGS-S-L-----------------------------------
XP_019087173.1/1-228 S-AYVA-LLPSGFSI-LPDGS-S-L-----------------------------------
XP_010555654.1/1-234 S-AYVA-LLPSGFSI-LPDDGG-MMK----------------------------------
XP_010555656.1/1-234 S-AYVA-LLPSGFSI-LPDDGG-MMK----------------------------------
XP_010555655.1/1-234 S-AYVA-LLPSGFSI-LPDDGG-MMK----------------------------------
XP_010537199.1/1-242 S-AYVA-LLPSGFWI-SPDGSKGKVK----------------------------------
XP_010537197.1/1-242 S-AYVA-LLPSGFWI-SPDGSKGKVK----------------------------------
XP_010537198.1/1-241 S-AYVA-LLPSGFWI-SPDGSKGKVK----------------------------------
Con_90%              ..s.hs.lLPSGF.l..P.t........................................
Con_80%              ..s.hs.lLPSGFtI.lP.s........................................
Con_70%              s.s.ls.LLPSGFtI.lP.s.tt.....................................
Con_60%              s.s.ls.LLPSGFsI.lPsssts...............s.....................
Con_50%              s.chls.LLPSGFuI.lPDGssst..............ssst..................


XP_020582785.1/1-284 RIVTDT---------TISDGRSVLTIAFQ------------FIFEN-H------------
XP_020682623.1/1-284 RIASDT---------TTSDMRSVLTIAFQ------------FIFEN-H------------
XP_020682624.1/1-284 RIASDT---------TTSDMRSVLTIAFQ------------FIFEN-H------------
XP_006846015.1/1-286 RPSGES-------GANGYTMRSVLTIAFQ------------FTYET-H------------
NP_001142394.1/1-289 MRASCD-------GSGTCATRSVLTIAFQ------------FSFEN-H------------
NP_001146215.1/1-288 CALSDS-------GSGTRSTRSVLTIAFQ------------FSFEN-H------------
XP_020405718.1/1-288 CALSDS-------GSGTRSTRSVLTIAFQ------------FSFEN-H------------
XP_002443548.1/1-287 RALSDS--------SGTCTTRSVLTIAFQ------------FSFEN-H------------
XP_025808876.1/1-287 RAPSDA--------SGACTTRSVLTIAFQ------------FSFEN-H------------
XP_004963232.1/1-287 RSPSDA--------SGSCTTRSVLTIAFQ------------FSFEN-H------------
XP_020192001.1/1-285 -APS-D-------ASSTSNTRSVLTIAFQ------------FSYEN-H------------
XP_003562005.1/1-285 -AAS-D-------APSTSNTRSVLTIAFQ------------FSYEN-H------------
XP_004982339.1/1-286 RASS-D-------ASSTCNTRSVLTIAFQ------------FSYEN-H------------
XP_025796542.1/1-286 RASS-D-------ASSTCNTRSVLTIAFQ------------FSYEN-H------------
NP_001347456.1/1-286 RTSS-D-------ASSTCNTRSVLTIAFQ------------FSYEN-H------------
NP_001352464.1/1-286 RASS-D-------ASSTCNTRSVLTIAFQ------------FSYEN-H------------
XP_002464180.1/1-286 RASS-D-------ASSTCNTRSVLTIAFQ------------FSYEN-H------------
XP_015689987.1/1-286 RASS-D-------TSGTCNTRSVLTIAFQ------------FSYEN-H------------
XP_015632484.1/1-286 RASS-D-------TSSTCNTRSVLTIAFQ------------FSYEN-H------------
XP_015620816.1/1-285 RASV-E-------ASGTC-NRSVLTIAFQ------------FSYEN-H------------
XP_006664738.1/1-285 RAST-D-------ASGVC-NRSVLTIAFQ------------FSYEN-H------------
XP_020190954.1/1-286 RGSG-D-------SPGGCSTRSVLTIAFQ------------FSFEN-H------------
XP_020163543.1/1-286 RGSG-D-------SPGGCSTRSVLTIAFQ------------FSFEN-H------------
XP_003577453.1/1-286 RGSS-D-------ASGTCNMRSVLTIAFQ------------FSFEN-H------------
XP_020575104.1/1-285 RVANDS-------GLNSGNSRSVLTIAFQ------------FTFEN-H------------
XP_020696902.1/1-286 RAPNDP-------VSYPSNSRSVLTIAFQ------------FTFEN-H------------
XP_020578108.1/1-284 RPSLEA-------ASNMSNLRSVLTIAFQ------------FTYEN-H------------
XP_020698923.1/1-284 HPATEG-------ASNMSNLRSVLTIAFQ------------FTYEN-H------------
XP_020096610.1/1-279 RPK-SN-------PSNSYNQRSVLTIAFQ------------FMYEN-H------------
XP_009412508.1/1-287 HTANKT-------TCSAFNSRSVMTVAFQ------------FTYEN-H------------
XP_020271156.1/1-284 RSASDS-------ASNAYNLRSVLTIAFQ------------FTFEN-H------------
XP_020273236.1/1-287 RSASDN-------ASNAYNLRSVLTIAFQ------------FTFEN-H------------
XP_020092733.1/1-287 RSPND--------TSNAYNSRSVLTIAFQ------------FMYEN-H------------
XP_019701934.1/1-288 RSSGEN-------SSNTYNLRSVLTIAFQ------------FTYEN-H------------
XP_010905421.1/1-288 RSSGEN-------SSNTYNLRSVLTIAFQ------------FTYEN-H------------
XP_010905420.1/1-289 RSSGEN-------SSNTYNLRSVLTIAFQ------------FTYEN-H------------
XP_010923678.1/1-288 RSSSEN-------ASNTYNLRSVLTIAFQ------------FTYEN-H------------
XP_008794452.1/1-288 RSSSEN-------ASDTYNLRSVLTIAFQ------------FTYEN-H------------
XP_009397391.1/1-288 CSVKET-------ASNAYNLRSVLTIAFQ------------FTCEN-H------------
XP_009408551.1/1-288 RSVNET-------ASSSYNLRSVLTIAFQ------------FTYEN-H------------
XP_009399045.1/1-288 RAVNET-------ASSTYNLRSVLTIAFQ------------FTYEN-H------------
XP_009388964.1/1-288 RSVNET-------ASSTYNLRSVLTIAFQ------------FTYEN-H------------
XP_009397717.1/1-288 RSVNET-------ASTTYNLRSVLTIAFQ------------FTYEN-H------------
XP_018488876.1/1-280 TD-------------AETNSRLVLTIAFQ------------FTFDN-H------------
XP_013587486.1/1-280 TD-------------TNTNSRLVLTIAFQ------------FTFDN-H------------
XP_009115139.1/1-280 TD-------------AETNSRLVLTIAFQ------------FTFDN-H------------
XP_013662459.1/1-280 TD-------------AETNSRLVLTIAFQ------------FTFDN-H------------
XP_013707293.1/1-280 TD-------------AETNSRLVLTIAFQ------------FTFDN-H------------
XP_006415465.2/1-280 TD-------------SETNSRLVLTIAFQ------------FTFDN-H------------
XP_019099288.1/1-280 TD-------------SETNSRLVLTIAFQ------------FTFDN-H------------
NP_174337.1/1-280    TD-------------SETNSRLVLTIAFQ------------FTFDN-H------------
XP_020867051.1/1-280 TD-------------SETNSRLVLTIAFQ------------FTFDN-H------------
XP_020867052.1/1-280 TD-------------SETNSRLVLTIAFQ------------FTFDN-H------------
XP_010460847.1/1-280 TD-------------SETNSRLVLTIAFQ------------FTYDN-H------------
XP_019094482.1/1-280 TD-------------TETNSRLVLTIAFQ------------FTFDN-H------------
XP_006306764.1/1-280 TD-------------SETNSRLVLTIAFQ------------FTFDN-H------------
XP_010538268.1/1-285 EASAEA-------ERSGYNHRSVLTIAFQ------------FTFDS-H------------
XP_010519070.1/1-284 AT-GEA-------DPNGYNHRSVLTIAFQ------------FTFDS-H------------
XP_018436356.1/1-280 QG-GEA-------DTNGCNFRSVVTIAFQ------------FTFDN-H------------
XP_013637594.1/1-286 QG-SEA-------GPNGCNFRSVVTIAFQ------------FTFDN-H------------
XP_013692700.1/1-286 QG-SEA-------GPNGCNFRSVVTIAFQ------------FTFDN-H------------
XP_009143747.1/1-286 QG-SEA-------DTNGCNFRSVVTIAFQ------------FTFDN-H------------
XP_018484893.1/1-285 QA-GEA-------DPNGCNFRSVLTIAFQ------------FTFDN-H------------
XP_013637264.1/1-280 QG-GEA-------DPNGCNFRSVLTIAFQ------------FTFDN-H------------
XP_013637263.1/1-280 QG-GEA-------DPNGCNFRSVLTIAFQ------------FTFDN-H------------
XP_022557131.1/1-280 QG-GEA-------DPNGCNFRSVLTIAFQ------------FTFDN-H------------
XP_013729051.1/1-280 QG-GEA-------DPNGCNFRSVLTIAFQ------------FTFDN-H------------
XP_013744548.1/1-280 QG-GEA-------DPNGCNFRSVLTIAFQ------------FTFDN-H------------
XP_009141396.1/1-280 QG-GEA-------DPNGCNFRSVLTIAFQ------------FTFDN-H------------
XP_006410621.1/1-284 QA-GEA-------DQNDCNFRSVLTIAFQ------------FTFDN-H------------
NP_181018.1/1-284    QA-GEA-------DPNGCNFRSVLTIAFQ------------FTFDN-H------------
XP_020884308.1/1-284 QA-GEA-------DPNGCNFRSVLTIAFQ------------FTFDN-H------------
XP_006293359.1/1-284 QE-GEA-------DPNGCNFRSVITIAFQ------------FTFDN-H------------
XP_010516626.1/1-284 QA-GEA-------DPNGCNFRSVITIAFQ------------YTFDN-H------------
XP_010509720.1/1-284 QA-GEA-------DPNGCNFRSVITIAFQ------------FTFDN-H------------
XP_010504942.1/1-284 QA-GEA-------DPNGCNFRSVITIAFQ------------FTFDN-H------------
XP_017223336.1/1-283 HPHGED-------NAGNYNLRSVLTIAFQ------------FPFEN-H------------
XP_016559371.1/1-285 RPAGEV-------EGSKYNHRSVLTIAFQ------------FTFEN-H------------
XP_016559374.1/1-285 RPAGEV-------EGSKYNHRSVLTIAFQ------------FTFEN-H------------
XP_012830207.1/1-281 RPAGGI-------DSTNQNVRSVLSIAFQ------------FTFES-H------------
XP_012827547.1/1-287 RSSGEA-------DVANQNVRSVLTIAFQ------------FTFEN-H------------
XP_022866806.1/1-284 RPAGEI-------DASNQNLRSVLTIAFQ------------FAYEN-H------------
XP_022866805.1/1-285 RPAGEI-------DASNQNLRSVLTIAFQ------------FAYEN-H------------
XP_023001017.1/1-285 --PGGET------DATNYNLRSVLTIAFQ------------FTFEN-H------------
XP_023001016.1/1-285 --PGGET------DATNYNLRSVLTIAFQ------------FTFEN-H------------
XP_023519837.1/1-285 --PGGET------DATNYNLRSVLTIAFQ------------FTFEN-H------------
XP_023519836.1/1-285 --PGGET------DATNYNLRSVLTIAFQ------------FTFEN-H------------
XP_022927139.1/1-285 --PGGET------DATNYNLRSVLTIAFQ------------FTFEN-H------------
XP_022927138.1/1-285 --PGGET------DATNYNLRSVLTIAFQ------------FTFEN-H------------
XP_022139982.1/1-285 --PGSET------DVTNYNLRSVLTIAFQ------------FTFEN-H------------
XP_004135550.1/1-285 --PGCET------DVTNYNLRSVLTIAFQ------------FTFEN-H------------
XP_008445207.1/1-285 --PGGET------DVTNYNLRSVLTIAFQ------------FTFEN-H------------
XP_017230337.1/1-286 --PPGEA------DLTNYNVRSVLTIAFQ------------FTYEN-H------------
XP_017230338.1/1-286 --PPGEA------DLTNYNVRSVLTIAFQ------------FTYEN-H------------
XP_010316959.1/1-285 --PAGEV------EGSSYNHRSVLTIAFQ------------FAFEN-H------------
XP_004233170.1/1-285 --PAGEV------EGSSYNHRSVLTIAFQ------------FAFEN-H------------
XP_015066839.1/1-285 --PAGEV------EGSSYNHRSVLTIAFQ------------FAFEN-H------------
XP_015066842.1/1-285 --PAGEV------EGSSYNHRSVLTIAFQ------------FAFEN-H------------
XP_015066843.1/1-285 --PAGEV------EGSSYNHRSVLTIAFQ------------FAFEN-H------------
XP_006353018.1/1-285 --LAGEV------EGRSYNHRSVLTIAFQ------------FAFEN-H------------
XP_006353020.1/1-285 --LAGEV------EGRSYNHRSVLTIAFQ------------FAFEN-H------------
XP_019178964.1/1-287 RPAGGEV------ESNNYNLRSVLTIAFQ------------FTFEN-H------------
XP_019178963.1/1-287 RPAGGEV------ESNNYNLRSVLTIAFQ------------FTFEN-H------------
XP_019228489.1/1-285 TRPAGEI------EASNYNHRSVLTIAFQ------------FIFES-H------------
XP_019228488.1/1-285 TRPAGEI------EASNYNHRSVLTIAFQ------------FIFES-H------------
NP_001289498.1/1-285 TRPAGEI------EASNYNHRSVLTIAFQ------------FTFES-H------------
XP_009777911.1/1-285 TRPAGEI------EASNYNHRSVLTIAFQ------------FTFES-H------------
XP_009612342.1/1-285 TRPAGEI------EASNYNHRSVLTIAFQ------------FTFES-H------------
XP_009612341.1/1-285 TRPAGEI------EASNYNHRSVLTIAFQ------------FTFES-H------------
XP_006363649.1/1-287 TRPAGEI------EAGNYNHRSVLTIAFQ------------FTFES-H------------
XP_015066689.1/1-287 TRPAGEI------EAGNYNHRSVLTISFQ------------FTFES-H------------
XP_010315636.1/1-287 TRPAGEI------EAGNYNHRSVLTISFQ------------FTFES-H------------
XP_022879368.1/1-285 --TVGEV------DSCKQSLRSVLTIAFQ------------FTFEN-H------------
XP_020554026.1/1-285 --SGGEA------DTSNQNFRSVLTIAFQ------------FTFEN-H------------
XP_011096678.1/1-285 --SGGEA------DTSNQNFRSVLTIAFQ------------FTFEN-H------------
XP_011085207.1/1-285 --PPGEV------DTSNQNLRSVLTIAFQ------------FTFEN-H------------
XP_023003193.1/1-285 -SGGEA-------DLSTYNLRSVLTIAFQ------------FTFEN-H------------
XP_022926723.1/1-285 -SGGEA-------DLSTYNLRSVLTIAFQ------------FTFEN-H------------
XP_023518237.1/1-285 -SGGEA-------DLSTYNLRSVLTIAFQ------------FTYEN-H------------
XP_022146537.1/1-286 -PAGET-------DLSSYNLRSVLTIAFQ------------FTFEN-H------------
XP_011658319.1/1-286 -SAGET-------DLSNYNLRSVLTIAFQ------------FTFEN-H------------
XP_008439554.1/1-286 -SAGET-------DLSSYNLRSVLTIAFQ------------FTFEN-H------------
XP_022978372.1/1-286 -SAGQT-------DLSSYNLRSVLTIAFQ------------FTFEN-H------------
XP_023543833.1/1-286 -SAGQT-------DLSSYNLRSVLTIAFQ------------FTFEN-H------------
XP_022926042.1/1-286 -SAGQT-------DLSSYNLRSVLTIAFQ------------FTFEN-H------------
XP_010247499.1/1-286 -SDGDS-------SSNGYNLRSVLTIAFQ------------FTYEN-H------------
XP_016717556.1/1-285 -STGDG-------EPNSYNLRSVLTIAFQ------------FTFEN-H------------
XP_012455108.1/1-285 -STGDG-------EPNSYNLRSVLTIAFQ------------FTFEN-H------------
XP_016717554.1/1-287 -STGDG-------EPNSYNLRSVLTIAFQ------------FTFEN-H------------
XP_012455107.1/1-287 -STGDG-------EPNSYNLRSVLTIAFQ------------FTFEN-H------------
XP_016711412.1/1-287 -STGDG-------ETNSYNLRSVLTIAFQ------------FTFEN-H------------
XP_017649973.1/1-287 -STGDG-------ETNSYNLRSVLTIAFQ------------FTFEN-H------------
XP_022716449.1/1-285 -TAGDG-------QPNNYNLRSVLTIAFQ------------FTFEN-H------------
XP_022724664.1/1-287 -PAGDG-------EPNNYNLRSVLTIAFQ------------FTFEN-H------------
XP_021298192.1/1-285 -LAGDG-------DPSNYNLRSVLTIAFQ------------FTFEN-H------------
XP_021298193.1/1-294 -LAGDG-------DPSNYNLRSVLTIAFQ------------FTFEN-H------------
XP_021298191.1/1-294 -LAGDG-------DPSNYNLRSVLTIAFQ------------FTFEN-H------------
XP_007022874.2/1-285 -LAGDG-------DPSNYNLRSVLTIAFQ------------FTFEN-H------------
XP_017979205.1/1-294 -LAGDG-------DPSNYNLRSVLTIAFQ------------FTFEN-H------------
XP_017979204.1/1-294 -LAGDG-------DPSNYNLRSVLTIAFQ------------FTFEN-H------------
XP_017236680.1/1-286 -PPGEA-------DINSYNLRSVLTIAFQ------------FTFEN-H------------
XP_010051959.1/1-285 -PTCEA-------DASTYNLRSVLTIAFQ------------FVFEN-H------------
XP_010051960.1/1-285 -PTCEA-------DASTYNLRSVLTIAFQ------------FVFEN-H------------
XP_015882975.1/1-284 -PTSEA-------NTNSDNLRSVLTIAFQ------------FTFEN-H------------
XP_015882797.1/1-284 -PTSEA-------NTNSDNLRSVLTIAFQ------------FTFEN-H------------
XP_010098570.1/1-284 -PSSEA-------DTNSYNLRSVLTIAFQ------------FTFEN-H------------
XP_011012084.1/1-284 -PASEA-------DTNSYNLRSVLTIAFQ------------FTFEN-H------------
XP_011012083.1/1-284 -PASEA-------DTNSYNLRSVLTIAFQ------------FTFEN-H------------
XP_006377667.2/1-284 -PASEA-------DTNSYNLRSVLTIAFQ------------FTFEN-H------------
XP_002281868.2/1-285 -PANES-------DLNNYNLRSVLTIAFQ------------FTFEN-H------------
XP_018847863.1/1-285 -PASEA-------DLNSYNLRSVLTIAFQ------------FTFEN-H------------
XP_018847862.1/1-285 -PASEA-------DLNSYNLRSVLTIAFQ------------FTFEN-H------------
XP_023919942.1/1-285 -PAGEA-------DLNGYNLRSVLTIAFQ------------FTFES-H------------
XP_023919941.1/1-285 -PAGEA-------DLNGYNLRSVLTIAFQ------------FTFES-H------------
XP_018828101.1/1-285 -PAGEA-------DLNSYNLRSVLTIAFQ------------FTFEN-N------------
XP_018828100.1/1-285 -PAGEA-------DLNSYNLRSVLTIAFQ------------FTFEN-N------------
XP_002298892.1/1-285 -PASEA-------DTNSYNLRSVLTIAFQ------------FSFEN-H------------
XP_011038887.1/1-285 -PASEA-------DTNSYNLRSVLTIAFQ------------FSFEN-H------------
XP_011038886.1/1-285 -PASEA-------DTNSYNLRSVLTIAFQ------------FSFEN-H------------
XP_021657817.1/1-285 -PSTEA-------DTNNYNLRSVLTIAFQ------------FTFDN-H------------
XP_021657818.1/1-285 -PSTEA-------DTNNYNLRSVLTIAFQ------------FTFDN-H------------
XP_015577248.1/1-260 -PPSEV-------DTNNYNLRSVLTIAFQ------------FTLRT--------------
XP_021616343.1/1-285 -PSSEA-------DTSSYNLRSVLTIAFQ------------FTFEN-H------------
XP_021645103.1/1-285 -PSSEA-------DANNCNLRSVLTIAFQ------------FTFEN-H------------
XP_021592762.1/1-285 -PSSEV-------DTNNCNLRSVLTIAFQ------------FTFEN-H------------
XP_012075627.1/1-285 -PSGEA-------DTSSYNLRSVLTIAFQ------------FTFEN-H------------
XP_008372224.1/1-285 -AVNDP-------DGNGYNLRSVLTIAFQ------------FTFEN-H------------
XP_009362238.1/1-285 -AVNDP-------DGNGYNLRSVLTIAFQ------------FTFEN-H------------
XP_009344405.1/1-284 -AINDA-------DGNSYNRRSVLTIAFQ------------FTFEN-H------------
XP_008349150.1/1-284 -AINDA-------DGNSYNRRSVLTIAFQ------------FTFEN-H------------
XP_007214622.1/1-285 -PVNEA-------DGNSYNLRSVLTIAFQ------------FTFEN-H------------
XP_021812393.1/1-285 -PVNEA-------DGNSYNLRSVLTIAFQ------------FTFEN-H------------
XP_024161164.1/1-285 -RVNEG-------DANSYNLRSVLTIAFQ------------FTFEN-H------------
XP_004293989.1/1-285 -RVNEG-------DANSYNLRSVLTIAFQ------------FTFEN-H------------
XP_019419795.1/1-282 -PAGET----------DYNLRSVLTIAFQ------------FTFEN-H------------
XP_019426301.1/1-282 -PAGEA----------DYNLRSVLTIAFQ------------FTFEN-H------------
XP_003524993.1/1-285 -SAGES-------DSNNYNLRSVLTIAFQ------------FTFEN-H------------
XP_003532788.1/1-285 -SAGES-------DLNNYNLRSVLTIAFQ------------FTFEN-H------------
XP_020215009.1/1-285 -PAGES-------DLNDYNLRSVLTIAFQ------------FTFEN-H------------
XP_017409410.1/1-285 -PAGET-------DLNNYNLRSVLTIAFQ------------FTFEN-H------------
XP_017409418.1/1-285 -PAGET-------DLNNYNLRSVLTIAFQ------------FTFEN-H------------
XP_014509941.1/1-285 -PAGET-------DLNNYNLRSVLTIAFQ------------FTFEN-H------------
XP_007159548.1/1-285 -PAGES-------DLSNYNLRSVLTIAFQ------------FTFEN-H------------
XP_024047818.1/1-288 -PAGGT-------ELSNYNSRSVLTIAFQ------------FTFEN-H------------
XP_006448915.2/1-288 -PAGGT-------ELSNYNSRSVLTIAFQ------------FTFEN-H------------
XP_006468302.1/1-288 -PAGGT-------ELSNYNSRSVLTIAFQ------------FTFEN-H------------
XP_006468303.1/1-287 -PAGGT-------ELSNYNSRSVLTIAFQ------------FTFEN-H------------
XP_024047817.1/1-287 -PAGGT-------ELSNYNSRSVLTIAFQ------------FTFEN-H------------
XP_004504217.1/1-285 -QAGEG-------DLNSYNIRSVLTIAFQ------------FTFEN-H------------
XP_003594520.2/1-281 -PSGEGA------GSNGYNLRSVLTIAFQ------------FTFEN-H------------
XP_024632601.1/1-281 -PSGEGA------GSNGYNLRSVLTIAFQ------------FTFEN-H------------
XP_004486450.1/1-285 -LSGEG-------DLNGYNLRSVLTIAFQ------------FTFEN-H------------
XP_007147487.1/1-285 -PAGEA-------DLNGYNLRSVLTIAFQ------------FTFEN-H------------
XP_017437412.1/1-285 -PSGEA-------DLSGYNLRSVLTIAFQ------------FTFEN-H------------
XP_014518680.1/1-285 -PAGEA-------DLNGYNLRSVLTIAFQ------------FTFEN-H------------
XP_003546255.1/1-285 -PAGEA-------DLNGYNLRSVLTIAFQ------------FTFEN-H------------
XP_020233213.1/1-285 -PAGEA-------DLNGYNLRSVLTIAFQ------------FTFEN-H------------
XP_003535078.1/1-285 -PAGEA-------DLNGYNLRSVLTIAFQ------------FTFEN-H------------
XP_014617344.1/1-285 -PAGEA-------DLNGYNLRSVLTIAFQ------------FTFEN-H------------
XP_015959291.1/1-283 -AAGEA---------DDYNLRSVLTIAFQ------------FTFEN-H------------
XP_016197724.1/1-283 -AAGEA---------DDYNLRSVLTIAFQ------------FTFEN-H------------
XP_025694278.1/1-283 -AAGEA---------DDYNLRSVLTIAFQ------------FTFEN-H------------
XP_006846777.1/1-281 ASGDGGG------PNSCNNLRSVLTIAFQ------------FTFEP-H------------
XP_003574071.1/1-280 APGNTS--------LDDSNLRSVLTIAFQ------------FPYEM-H------------
XP_020174778.1/1-278 ASGG----------ADDCNLRSVLTIAFQ------------FPYEM-H------------
XP_006661867.1/1-281 PTGDAT--------LDDCNLRSVLTIAFQ------------FPYEM-H------------
XP_015614901.1/1-281 PTGDAS--------MDDCNLRSVLTIAFQ------------FPYEM-H------------
XP_004982838.1/1-282 ASGDGS--------PDDCNLRSVLTIAFQ------------FPYEI-H------------
XP_025796396.1/1-282 ASGDGS--------QDDCNLRSVLTIAFQ------------FPYEI-H------------
NP_001316502.1/1-284 VAGNGS--------QDVCNLRSVLTIAFQ------------FPYET-H------------
XP_021306737.1/1-282 ASADGS--------QDACNLRSVLTIAFQ------------FPYEI-H------------
XP_021306733.1/1-293 ASADGS--------QDACNLRSVLTIAFQ------------FPYEI-H------------
XP_020162053.1/1-281 ASG-AS--------PDDCNVRSVLTIAFQ------------FPYEL-H------------
XP_006649248.1/1-282 ASGDAS--------PDDCNMRSVLTIAFQ------------FPYEM-H------------
XP_015632709.1/1-282 ASGDAS--------ADDCNLRSVLTIAFQ------------FPYEL-H------------
NP_001296150.1/1-282 ASGE-S--------PDDCSLRSVLTIAFQ------------FPYEM-H------------
XP_008665074.1/1-283 ASGDAP--------PDDCNLRSVLTIAFQ------------FPYEM-H------------
NP_001105994.1/1-283 ASGDAP--------PDDCNLRSVLTIAFQ------------FPYEM-H------------
XP_008660404.1/1-283 ASGDAP--------PDDCNLRSVLTIAFQ------------FPYEM-H------------
XP_002468669.1/1-283 ASGDAS--------PDDCNLRSVLTIAFQ------------FPYEM-H------------
XP_021309716.1/1-285 ASGDAS--------PDDCNLRSVLTIAFQ------------FPYEM-H------------
XP_004986037.1/1-283 ASGDAS--------PDDCNLRSVLTIAFQ------------FPYEM-H------------
XP_025795001.1/1-283 ASGDAS--------PDDCNLRSVLTIAFQ------------FPYEM-H------------
XP_020093893.1/1-283 TSTEKS--------PCDYSLRSVLTIAFQ------------FPYEM-H------------
XP_020093894.1/1-282 TSTEKS--------PCDYSLRSVLTIAFQ------------FPYEM-H------------
XP_020259754.1/1-283 TGGEAS--------PSDYSLRSVLTIAFQ------------FPYEF-H------------
XP_009401955.1/1-282 SAGDAS--------TDDYNLRSVLTIAFQ------------FPYEV-H------------
XP_008791541.1/1-283 CAGDTP--------TIDHNLRSVLTIAFQ------------FPYEV-N------------
XP_017698637.1/1-283 CAGDTP--------TIDHNLRSVLTIAFQ------------FPYEV-N------------
XP_010920422.1/1-283 CAGDTS--------TNDHSLRSVLTIAFQ------------FPYEV-N------------
XP_010920423.1/1-283 CAGDTS--------TNDHSLRSVLTIAFQ------------FPYEV-N------------
XP_010920419.1/1-283 CAGDTS--------TNDHSLRSVLTIAFQ------------FPYEV-N------------
XP_010920426.1/1-283 CAGDTS--------TNDHSLRSVLTIAFQ------------FPYEV-N------------
XP_019709326.1/1-287 SASDKS--------SNGHKLRSVLTIAFQ------------FPYEV-H------------
XP_008785281.1/1-283 SAGDKS--------TDGHNVRSVLTIAFQ------------FPYEL-H------------
XP_008462593.1/1-288 DAAVA-A------AASTSNFRSVLTIAFQ------------FPFGG-N------------
XP_022969629.1/1-286 DAA----------AASTSNFRSVLTIAFQ------------FSFGS-N------------
XP_023536970.1/1-286 DAA----------AASTSNFRSVLTIAFQ------------FSFGS-N------------
XP_022937855.1/1-288 DAAA--A------AASTSNFRSVLTIAFQ------------FSFGS-N------------
XP_022937858.1/1-288 DAAA--A------AASTSNFRSVLTIAFQ------------FSFGS-N------------
XP_012827869.1/1-285 NNGK--N--------AAYSGRSILTIAFQ------------FPFES-S------------
XP_022874815.1/1-279 AAGY--A------A-SSFSARSVLTLAFQ------------FPFER-N------------
XP_022874814.1/1-283 AAGY--A------A-SSFSARSVLTLAFQ------------FPFER-N------------
XP_018434000.1/1-280 TG-------------SNPSTRCILTIAFQ------------FPFEN-N------------
XP_013642496.1/1-279 TG-------------SSPSSRCILTIAFQ------------FPFEN-N------------
XP_009150087.1/1-279 TG-------------SSPSSRCILTIAFQ------------FPFEN-N------------
XP_009150088.1/1-279 TG-------------SSPSSRCILTIAFQ------------FPFEN-N------------
XP_022563181.1/1-279 TG-------------SNLSTRCILTIAFQ------------FPFEN-N------------
XP_013675790.1/1-280 TG-------------SNLSTRCILTIAFQ------------FPFEN-N------------
XP_013623090.1/1-279 TG-------------SNPSTQCILTIAFQ------------FPFEN-N------------
XP_013623089.1/1-280 TG-------------SNPSTQCILTIAFQ------------FPFEN-N------------
XP_020885782.1/1-285 ASGN--S------SSSSSSSRCILTIAFQ------------FPFEN-N------------
XP_006280004.1/1-285 ASGN--S------TSSSSSSRCILTIAFQ------------FPFEN-N------------
XP_019088563.1/1-285 ASGN--P------SSSSSSSRCILTIAFQ------------FPFEN-N------------
XP_010483719.1/1-285 ASGN--P------SSSSSSSRCILTIAFQ------------FPFEN-N------------
XP_010456679.1/1-285 ASGN--P------SSSSSSSRCILTIAFQ------------FPFEN-N------------
XP_006400821.1/1-284 ASGN--S------SA-NSSSRCILTIAFQ------------FPFEN-N------------
NP_200877.1/1-284    ASGN--S------FS-SSSSRCILTIAFQ------------FPFEN-N------------
XP_013611543.1/1-282 ASGN--S------S---SSSRCILTIAFQ------------FPFES-N------------
XP_009120605.1/1-284 ASGN--S------SS-SSSSRCILTIAFQ------------FPFEN-N------------
XP_013667152.1/1-284 ASGN--S------SS-SSSSRCILTIAFQ------------FPFEN-N------------
XP_018446535.1/1-284 ASGN--S------SS-GSNSRCILTIAFQ------------FPFEN-N------------
XP_018454374.1/1-284 ASGN--C------SS-SSSSRCILTIAFQ------------FPFEP-N------------
XP_009126588.1/1-285 ASGN--S------SSSCSSSRCILTIAFQ------------FPFEP-N------------
XP_013680521.1/1-285 ASGD--S------SSSCSSSRCILTIAFQ------------FPFEP-N------------
XP_013612427.1/1-285 ASGN--S------SSSCSSSRCILTIAFQ------------FPFEP-N------------
XP_010535608.1/1-283 SSGN--P------TT-SSSSRSILTIAFQ------------FPFET-N------------
XP_010520372.1/1-276 SS----------------RSRSILTIAFQ------------FPFET-D------------
XP_004487103.1/1-286 AAGDM---------SLCPNNRSVLTVAFQ------------FPFEN-G------------
XP_003597252.1/1-283 ANGDA----------SCPNNRCVLTVAFQ------------FPFES-G------------
XP_024455683.1/1-286 TAVDG---------SSCHSLRSVLTIAFQ------------FPFES-N------------
XP_022026352.1/1-288 GSTDM---------STCHTSRSVLTIAFQ------------FPFEN-N------------
XP_022000486.1/1-288 GSTDM---------STCQTTRSVLTITFQ------------FPFEN-S------------
XP_016717477.1/1-281 AAG---D------SPS-QNARSVLTIAFQ------------FPFDT-N------------
XP_017643014.1/1-281 AAG---D------SPS-QNARSVLTIAFQ------------FPFDT-N------------
XP_016724213.1/1-281 AAG---D------SPC-QNARSVLTIAFQ------------FPFDT-N------------
XP_012462938.1/1-281 AAG---D------SPC-QNARSVLTIAFQ------------FPFDT-N------------
XP_012471509.1/1-292 SSG---D------VPSSQNSRSVLTIAFQ------------FPFDS-S------------
XP_017642742.1/1-292 SSG---D------VPSSQNSRSVLTIAFQ------------FPFDS-S------------
XP_016755876.1/1-292 SSG---D------VPSSQNSRSVLTIAFQ------------FPFDS-S------------
XP_012471510.1/1-286 SSG---D------VPSSQNSRSVLTIAFQ------------FPFDS-S------------
XP_016753711.1/1-286 SSG---D------VPSSQNSRSVLTIAFQ------------FPFDS-S------------
XP_017642744.1/1-286 SSG---D------VPSSQNSRSVLTIAFQ------------FPFDS-S------------
XP_016755877.1/1-286 SSG---D------VPSSQNSRSVLTIAFQ------------FPFDS-S------------
XP_016750468.1/1-286 AAG---D------TSSCRNTRSVLTIAFQ------------FPFES-N------------
XP_016688657.1/1-286 AAG---D------TSSCRNTRSVLTIAFQ------------FPFES-N------------
XP_012443272.1/1-286 AAG---D------TSSCRNTRSVLTIAFQ------------FPFES-N------------
XP_017603001.1/1-286 AAG---D------TSSCRNTRSVLTIAFQ------------FPFES-N------------
XP_022755632.1/1-285 AAG---D------A-PCQNTRSVLTIAFQ------------FPFDS-N------------
XP_022755633.1/1-285 AAG---D------A-PCQNTRSVLTIAFQ------------FPFDS-N------------
XP_022729815.1/1-286 SAG---D------ALSCQNTRSVLTIAFQ------------FPFDS-N------------
XP_022729816.1/1-286 SAG---D------ALSCQNTRSVLTIAFQ------------FPFDS-N------------
XP_022752857.1/1-286 AAG---D------ATSCQNTRSVLTIAFQ------------FPFDI-N------------
XP_022752856.1/1-286 AAG---D------ATSCQNTRSVLTIAFQ------------FPFDI-N------------
XP_021277981.1/1-286 AAG---D------APSCQNSRSVLTIAFQ------------FPFDS-N------------
XP_017983554.1/1-286 AAG---D------APSCQNSRSVLTIALQ------------FPFDS-N------------
XP_011089912.1/1-283 SAE---N------ASTSYNARSVLTIAFQ------------FPFES-N------------
XP_007208090.1/1-286 AAG---E------LSSFHNTRSVLTIAFQ------------FPFEN-S------------
XP_008225704.1/1-286 AAG---E------LSSFHNTRSVLTIAFQ------------FPFES-S------------
XP_021830853.1/1-286 AAG---E------LSSFHNTRSVLTIAFQ------------FPFES-S------------
XP_008349754.1/1-286 AAG---E------LTTFHNTRSVLTIAFQ------------FPFDN-S------------
NP_001315783.1/1-288 AAG---E------LTTFHNTRSVLTIAFQ------------FPFDN-S------------
XP_009367069.1/1-286 AAG---D------LTTFHNTRSVLTIAFQ------------FPLDN-S------------
XP_008344872.1/1-286 AAG---D------LTTFHNTRSVLTIAFQ------------FPLDN-X------------
XP_015952399.1/1-286 GAG---D------ASSSQNTRSVLTIAFQ------------FPFDS-N------------
XP_020982118.1/1-286 GAG---D------ASSSQNTRSVLTIAFQ------------FPFDS-N------------
XP_025685402.1/1-286 GAG---D------ASSSQNTRSVLTIAFQ------------FPFDS-N------------
XP_016187430.1/1-286 GAG---D------ASSSQNTRSVLTIAFQ------------FPFDS-N------------
XP_020214107.1/1-284 GA----D------AS-SHNTRSVLTIAFQ------------FPFDS-S------------
XP_006592258.1/1-284 GT----D------ASSSQNTRSVLTIAFQ------------FPFDS-S------------
XP_007132262.1/1-285 GA----D------ASSSQNTRSVLTIAFQ------------FPFDS-S------------
XP_003538150.1/1-285 GA----D------ASSSQNTRSVLTIAFQ------------FPFDS-S------------
XP_017433493.1/1-285 GA----D------ASSSQNTRSVLTIAFQ------------FPFDS-S------------
XP_014494018.1/1-285 GA----D------ASSSQNTRSVLTIAFQ------------FPFDS-S------------
XP_010089983.2/1-286 AAG---D------LSPCNNARSLLTIAFQ------------FPFES-S------------
XP_006378974.2/1-285 ASV---D------GSSCH-LRSVLTIAFQ------------FPFES-N------------
XP_011010051.1/1-285 ASV---D------GSSCH-LRSVLTIAFQ------------FPFES-N------------
XP_011010050.1/1-285 ASV---D------GSSCH-LRSVLTIAFQ------------FPFES-N------------
XP_021905657.1/1-285 -SG---D------GSSCNNTRSVLTIAFQ------------FPFES-N------------
XP_021610391.1/1-286 ATE---D------ASSSHSTRSVLTIAFQ------------FPFES-N------------
XP_002529946.1/1-286 TAG---D------ASSSQSTRSVLTIAFQ------------FPFES-N------------
XP_021658728.1/1-286 SAG---D------ALSSHSTRSVLTIAFQ------------FPFES-N------------
XP_012064816.1/1-286 VSG---D------ASSSHITRSVLTIAFQ------------FPFES-N------------
XP_021628343.1/1-286 VAG---D------ASSSHSTRSVLTIAFQ------------FPFES-N------------
XP_021657859.1/1-286 AAG---D------VSSSHSTRSVLTIAFQ------------FPFEN-N------------
XP_015874442.1/1-285 AAG---D-------SSCQNTRSVLTIAFQ------------FPFES-S------------
XP_023926843.1/1-286 AAG---D------VSSCHYSRSVLTIAFQ------------FPFDS-S------------
XP_018819529.1/1-286 AGG---D------APLAHNSRSVLTIAFQ------------FPFDS-T------------
XP_010651164.1/1-286 AAG---D------SSSCYNTRSVLTIAFQ------------FPFES-N------------
XP_024034672.1/1-286 AAG---D------SSSCHHTRSVLTIAFQ------------FPFES-N------------
XP_006488084.1/1-286 AAG---D------SSSCHHTRSVLTIAFQ------------FPFES-N------------
XP_024034673.1/1-286 AAG---D------SSSCHHTRSVLTIAFQ------------FPFES-N------------
XP_010264677.1/1-287 AAG--DT------PSNAYNARSVLTIAFQ------------FPFEN-H------------
XP_010253680.1/1-288 ASG--NA------SSNAINTRSVLTIAFQ------------FPFEN-H------------
XP_022005829.1/1-282 GPS--DS------------MRSVLTIAFQ------------FPFEN-N------------
XP_023751537.1/1-278 GPA--DT------L----TTRSVLTIAFQ------------FPFEN-N------------
XP_024971452.1/1-283 GTS--DT------LG-CQNTRSVLTIAFQ------------FPFEN-N------------
XP_024971444.1/1-287 GTS--DT------LG-CQNTRSVLTIAFQ------------FPFEN-N------------
XP_024985647.1/1-287 GST--DM------TM-CQNTRSVLTIAFQ------------FPFEN-N------------
XP_023749448.1/1-288 GGD--MS------MS-CQNMRSVLTIAFQ------------FPFEN-N------------
XP_010689538.1/1-281 GAA--DA------TS-SGSNRSVLTIAFQ------------FPFES-S------------
XP_021858740.1/1-280 GAA--DA------T--SGNTRSVLTIAFQ------------FPFEI-S------------
XP_021773317.1/1-281 GAA--DA------TS-SGSTRSVLTIAFQ------------FPFEI-S------------
XP_021745941.1/1-281 GAA--DA------TS-SGSTRSVLTIAFQ------------FPFEI-S------------
XP_016729039.1/1-286 IAV--DA------PS-CPNIRSVLTIAFQ------------FPFDS-N------------
XP_017623828.1/1-286 IAV--DA------PS-CPNIRSVLTIAFQ------------FPFDS-N------------
XP_012476406.1/1-286 IAV--DA------PS-CPNIRSVLTIAFQ------------FPFDS-N------------
XP_016754776.1/1-286 IAV--DA------PS-CPNIRSVLTIAFQ------------FPFDS-N------------
XP_022152944.1/1-286 DSA--AA------ST-SHNFRSVLTIAFQ------------FPFGS-N------------
XP_011040884.1/1-286 AAV--DG------SS-CHSLRSVLTIAFQ------------FPFES-N------------
XP_010043753.1/1-286 CVG--DV------AP-SHGARSVLTIAFQ------------FPFDA-N------------
XP_024158223.1/1-285 NAG--DL------NS-FHNARSVLTIAFQ------------FPFES-S------------
XP_004291573.1/1-285 NAG--DL------NS-FHNTRSVLTIAFQ------------FPFES-S------------
XP_018808765.1/1-285 SAG--DA------SS-CH-SRSVLTIAFQ------------FPFDS-S------------
XP_023512455.1/1-283 AAG--DA------SS-SQSSRSVLTIAFQ------------FPFES-S------------
XP_023512456.1/1-283 AAG--DA------SS-SQSSRSVLTIAFQ------------FPFES-S------------
XP_022944186.1/1-283 AAG--DA------SS-SQSSRSVLTIAFQ------------FPFES-S------------
XP_022944185.1/1-283 AAG--DA------SS-SQSSRSVLTIAFQ------------FPFES-S------------
XP_022985614.1/1-283 AAG--DA------SS-SPSSRSVLTIAFQ------------FPFES-S------------
XP_022985615.1/1-283 AAG--DA------SS-SPSSRSVLTIAFQ------------FPFES-S------------
XP_011656996.1/1-283 TAG--DA------SS-SQSARSVLTIAFQ------------FPFES-S------------
XP_011656997.1/1-283 TAG--DA------SS-SQSARSVLTIAFQ------------FPFES-S------------
XP_008463945.1/1-283 TAG--DA------SS-SQSARSVLTIAFQ------------FPFES-S------------
XP_008463946.1/1-283 TAG--DA------SS-SQSARSVLTIAFQ------------FPFES-S------------
XP_022140363.1/1-286 SAG--DV------SS-SHSPRSVLTIAFQ------------FPFES-S------------
XP_022995706.1/1-286 IAG--DA------SS-SQSPRSVLTIAFQ------------FPFES-S------------
XP_023534373.1/1-286 VAG--DA------SS-SQSPRSVLTIAFQ------------FPFES-S------------
XP_022958713.1/1-286 VAG--DA------SS-SQCPRSVLTIAFQ------------FPFES-S------------
XP_025614062.1/1-285 GAR--DA------VS-CHSARSVLTIAFQ------------FPFDS-S------------
XP_025614063.1/1-285 GAR--DA------VS-CHSARSVLTIAFQ------------FPFDS-S------------
XP_016170485.1/1-285 GAR--DA------VS-CHSARSVLTIAFQ------------FPFDS-S------------
XP_016170486.1/1-285 GAR--DA------VS-CHSARSVLTIAFQ------------FPFDS-S------------
XP_015935757.1/1-285 GAR--DA------VS-CHSARSVLTIAFQ------------FPFDS-S------------
XP_015935758.1/1-285 GAR--DA------VS-CHSARSVLTIAFQ------------FPFDS-S------------
XP_004505999.1/1-286 GGV--DS------SA-SQNTRSVLTIAFQ------------FPFDS-S------------
XP_013456006.1/1-286 GGA--DS------SS-SQNTRSVLTIAFQ------------FPFDS-T------------
XP_019453966.1/1-285 GA---DA------SS-SQNTRSVLTIAFQ------------FPFDS-N------------
XP_019448740.1/1-279 GA---DA------S--SQNTRSVLTIAFQ------------FPFDR-S------------
XP_019448741.1/1-279 GA---DA------S--SQNTRSVLTIAFQ------------FPFDR-S------------
XP_019458095.1/1-286 AAG--DA------SS-CYSNRSVLTIAFQ------------FPFDS-S------------
XP_019447870.1/1-286 AAG--DA------SS-CYTNRSVLTIAFQ------------FPFDS-S------------
XP_012838629.1/1-280 AAENAES------SSSSRNTRSVLTIAFQ------------FPLES-N------------
XP_012838630.1/1-280 AAENAES------SSSSRNTRSVLTIAFQ------------FPLES-N------------
XP_012838627.1/1-280 AAENAES------SSSSRNTRSVLTIAFQ------------FPLES-N------------
XP_012838628.1/1-280 AAENAES------SSSSRNTRSVLTIAFQ------------FPLES-N------------
XP_017214964.1/1-286 N-A--GD------SSTCYSMRSVLTIAFQ------------FPYEN-H------------
XP_017234817.1/1-286 A-A--SD------SSACYNMRSVLTIAFQ------------FPYEN-N------------
XP_019178153.1/1-282 A----QN------ASSSCAARSLLTIAFQ------------FPYED-N------------
XP_019166562.1/1-283 HAV--GS------ASASYTARSVLTIAFQ------------FPFEN-N------------
XP_011089294.1/1-282 N-G--KN------AAASCSARSILTIAFQ------------FPFEN-N------------
XP_022892122.1/1-274 G-A--GN------ASSSYSARSVLTIAFQ------------FPFES-N------------
XP_022883393.1/1-279 G-A--GN------ATSSYSARSVLTIAFQ------------FPFES-N------------
XP_022883398.1/1-279 G-A--GN------ATSSYSARSVLTIAFQ------------FPFES-N------------
XP_022883384.1/1-283 G-A--GN------ATSSYSARSVLTIAFQ------------FPFES-N------------
XP_022883389.1/1-283 G-A--GN------ATSSYSARSVLTIAFQ------------FPFES-N------------
XP_009776589.1/1-283 A-T--GD------VVSGYSARSVLTIAFQ------------FPFED-N------------
NP_001312504.1/1-283 A-T--GD------VVSGYSARSVLTIAFQ------------FPFED-N------------
XP_016472209.1/1-283 A-T--GD------VVSGYSARSVLTIAFQ------------FPFED-N------------
XP_019255429.1/1-283 A-T--GD------VVSGYSARSVLTIAFQ------------FPFED-N------------
XP_009624234.1/1-283 A-T--GD------VVSGYSARSVLTIAFQ------------FPFED-N------------
XP_016504967.1/1-283 A-T--GD------VVSGYSARSVLTIAFQ------------FPFED-N------------
XP_016547131.1/1-283 A-T--GD------AASCYSARSVLTIAFQ------------FPFED-N------------
XP_004251138.1/1-283 T-T--GD------AASCYSARSVLTIAFQ------------FPFED-N------------
XP_015058512.1/1-283 A-T--GD------AASCYSARSVLTIAFQ------------FPFED-N------------
XP_006340193.1/1-283 A-T--GD------MASCYSARSVLTIAFQ------------FPFED-N------------
XP_004235650.1/1-283 AA-NDLH-----TSG--GSSRSVMTIAFQ------------FAFES-H------------
XP_006343047.1/1-283 AA-NDLH-----TSG--GSSRSVMTIAFQ------------FAFES-H------------
XP_009774805.1/1-285 VG-NDLH-----TNG--GTSRSVMTIAFQ------------FAFES-H------------
XP_016498616.1/1-285 VG-NDLH-----TNG--GTSRSVMTIAFQ------------FAFES-H------------
XP_016434103.1/1-285 VG-NDLH-----TNG--GTSRSVMTIAFQ------------FAFES-H------------
XP_009593967.1/1-285 VG-NDLH-----TNG--GTSRSVMTIAFQ------------FAFES-H------------
XP_004510297.1/1-285 AS-SDNA-----GNS--GCVRSVMTIAFE------------FAFES-H------------
XP_004510296.1/1-285 AS-SDNA-----GNS--GCVRSVMTIAFE------------FAFES-H------------
XP_003627005.1/1-284 AS-SDNA-----GNS--GCVRSVMTIAFE------------FAYES-H------------
XP_003531653.1/1-284 AS-DECA-----GNS--SYMRSVMTIAFE------------FAFES-H------------
XP_006585576.1/1-288 AS-DECA-----GNS--SYMRSVMTIAFE------------FAFES-H------------
XP_014634618.1/1-288 AS-DECA-----GNS--SYMRSVMTIAFE------------FAFES-H------------
XP_014634619.1/1-288 AS-DECA-----GNS--SYMRSVMTIAFE------------FAFES-H------------
XP_003531652.1/1-285 AS-NGSA-----GNS--SCMRSVMTIAFE------------FAFES-H------------
XP_003530109.1/1-284 AS-NGCA------NS--SCMRSVMTIAFE------------FAFES-H------------
XP_007135643.1/1-285 AS-NECA-----GNS--SCMRSVMTIAFE------------FAFES-H------------
XP_003530112.1/1-285 AS-NECA-----GNS--SYMRSVMTIAFE------------FAFES-H------------
XP_020226142.1/1-285 AS-NEYA-----GNS--GCMRSVMTIAFE------------FAFES-H------------
XP_014516466.1/1-285 AS-NECA-----GNS--GCMRSVMTIAFE------------FAFES-H------------
XP_017407594.1/1-285 AS-NECA-----GNS--GCMRSVMTIAFE------------FAFES-H------------
XP_019448178.1/1-285 AS-NDYS-----GNS--GCMRSVMTIAFE------------FAFES-H------------
XP_019429328.1/1-285 AS-NDYS-----GNS--GCVRSVMTIAFE------------FAFES-H------------
XP_004486776.1/1-285 VS-NDYS-----GNS--GCMRSVMTIAFE------------FAFES-H------------
XP_003597690.1/1-286 AS-NDYS-----GNS--GCMRSVMTIAFE------------FAFES-H------------
XP_015932679.1/1-285 AS-NETS-----GNS--GSVRSVMTIAFE------------FAFES-H------------
XP_025674177.1/1-285 AS-NETS-----GNS--GSVRSVMTIAFE------------FAFES-H------------
XP_025610204.1/1-285 AS-NETS-----GNS--GSVRSVMTIAFE------------FAFES-H------------
XP_020226055.1/1-285 AS-NDYS-----GNS--GCMRSVMTIAFE------------FAFES-H------------
XP_007150614.1/1-285 AS-NDYA-----GNS--GSMRSVMTIAFE------------FAFES-H------------
XP_014501306.1/1-285 AS-NDYS-----GNS--GSMRSVMTIAFE------------FAFES-H------------
XP_017425270.1/1-285 AS-NDYS-----GNS--GSMRSVMTIAFE------------FAFES-H------------
XP_021836126.1/1-285 SS-SDNS-----GSG--NNMRSVMTIAFQ------------FAFES-H------------
XP_010691423.1/1-284 S--SENS-----GSG--NNMRSVMTIAFQ------------FAFES-H------------
XP_010691424.1/1-284 S--SENS-----GSG--NNMRSVMTIAFQ------------FAFES-H------------
XP_021765330.1/1-284 S--SDHS-----ASG--NSMRSVMTIAFQ------------FAFES-H------------
XP_021749885.1/1-284 S--SDHS-----ASG--NSMRSVMTIAFQ------------FAFES-H------------
XP_010267183.1/1-285 IS-GDYS-----GNT--GNMRSVMTIAFQ------------FAFES-H------------
XP_017258627.1/1-285 AC-SNYT-----ATG--GSTRSVMTIAFE------------FAFES-H------------
XP_023000315.1/1-284 AP-ND-T-----VNS--GCTRSVMTIAFE------------FAFES-H------------
XP_023000313.1/1-284 AP-ND-T-----VNS--GCTRSVMTIAFE------------FAFES-H------------
XP_023514554.1/1-284 AP-ND-A-----VNS--GCTRSVMTIAFE------------FAFES-H------------
XP_023514552.1/1-284 AP-ND-A-----VNS--GCTRSVMTIAFE------------FAFES-H------------
XP_022964458.1/1-284 AP-ND-A-----VNS--GCTRSVMTIAFE------------FAFES-H------------
XP_022964454.1/1-284 AP-ND-A-----VNS--GCTRSVMTIAFE------------FAFES-H------------
XP_022147627.1/1-284 AS-ND-C-----ANS--GCTRSVMTIAFE------------FAYES-H------------
XP_022147619.1/1-284 AS-ND-C-----ANS--GCTRSVMTIAFE------------FAYES-H------------
XP_008462437.1/1-284 TP-ND-S-----VNS--GCTRSVMTIAFE------------FAFES-H------------
XP_004141659.1/1-284 TP-ND-S-----VNS--GCTRSVMTIAFE------------FAFES-H------------
XP_008462436.1/1-284 TP-ND-S-----VNS--GCTRSVMTIAFE------------FAFES-H------------
XP_011659682.1/1-284 TP-ND-S-----VNS--GCTRSVMTIAFE------------FAFES-H------------
XP_022992111.1/1-284 AP-ND-S-----VNS--GCSRSVMTIAFE------------FAFES-H------------
XP_023548534.1/1-284 AP-ND-S-----VNS--GCSRSVMTIAFE------------FAFES-H------------
XP_022953371.1/1-284 AP-ND-S-----VNS--GCSRSVMTIAFE------------FAFES-H------------
XP_002304217.1/1-285 AS-SDHS-----ANS--GCTRSVMTIAFE------------FAFES-H------------
XP_011013041.1/1-285 AS-SDHS-----ANS--GCTRSVMTIAFE------------FAFES-H------------
XP_011013042.1/1-285 AS-SDHS-----ANS--GCTRSVMTIAFE------------FAFES-H------------
XP_011046759.1/1-284 AS-SDFS-----ANS--GCTRSVMTIAFE------------FAFES-H------------
XP_024464085.1/1-284 AS-SDFS-----ANS--GCTRSVMTIAFE------------FAFES-H------------
XP_024183498.1/1-286 AS-SEYS-----ANA--GCVRSVMTIAFE------------FAFES-H------------
XP_004302866.1/1-286 AS-SEYS-----ANA--GCVRSVMTIAFE------------FACES-H------------
NP_001280898.1/1-285 GS-SEYS-----ASA--GCVRSVMTIAFE------------FACET-H------------
XP_007214960.1/1-285 AS-SEFS-----ANT--GCVRSVMTIAFE------------FACES-H------------
XP_008228437.1/1-285 AS-SEFS-----ANT--GCVRSVMTIAFE------------FACES-H------------
XP_021818763.1/1-285 AS-SEFS-----ANT--GCVRSVMTIAFE------------FACES-H------------
XP_022723515.1/1-285 AS-SDYS-----ANS--GCMRSVMTIAFE------------FAFES-H------------
XP_016688508.1/1-285 AS-NDYS-----AAG--GCMRSVMTIAFE------------FAFES-H------------
XP_012443274.1/1-285 AS-NDYS-----AAG--GCMRSVMTIAFE------------FAFES-H------------
XP_022721457.1/1-285 SS-NDYS-----SNS--GCMRSVMTIAFE------------FAFEN-H------------
XP_022715018.1/1-285 AS-NDYW-----SNS--GCMRSVMTIAFE------------FALES-H------------
XP_017979082.1/1-285 AS-NDYS-----GNS--GCMRSVMTIAFE------------FAFES-H------------
XP_021293768.1/1-285 AS-NDYS-----GNS--GCMRSVMTIAFE------------FAFES-H------------
XP_010062518.1/1-285 SF-NDIN-----ANS--GCTRSVMTIAFE------------FAFES-H------------
XP_006426893.1/1-285 AT-NNYS-----TNS--TCMRSVMTIAFE------------FAFES-H------------
XP_021610318.1/1-285 SS-TDYS-----ANS--GCMRSVMTIAFE------------FAFES-H------------
XP_010098130.1/1-285 AT-NDYS-----ANS--GCVRSVMTIAFE------------FAFES-H------------
XP_021635282.1/1-296 SS-TDYS-----ANS--GCVRSVMTIAFE------------FAFES-H------------
XP_002284003.2/1-285 SS-NDYS-----VNG--GNTRSVMTIAFE------------FAFES-H------------
XP_002515977.1/1-285 SS-TDYS-----SNS--GCMRSVMTIAFE------------FAFES-H------------
XP_021643260.1/1-285 SS-TDYT-----ANS--GCMRSVMTIAFE------------FAFES-H------------
XP_021628194.1/1-285 SS-TDYS-----ANS--GCVRSVMTIAFE------------FAFES-H------------
XP_015894591.1/1-285 AS-SDYS-----ANN--GCMRSVMTIAFE------------FAFES-H------------
XP_012069098.1/1-285 SS-TEYS-----ANS--GCVRSVMTIAFE------------FAFES-H------------
XP_018847857.1/1-285 TS-TDYS-----ANA--GSVRSVMTIAFE------------FAYES-H------------
XP_023898511.1/1-285 AS-NDYS-----ANS--GCVRSVMTIAFE------------FAFES-H------------
XP_010545673.1/1-285 A--STDG-----SGN-SSCTRSVMTIAFE------------FAFES-H------------
XP_010545670.1/1-286 A--STDG-----SGN-SSCTRSVMTIAFE------------FAFES-H------------
XP_010535179.1/1-285 AL-SSDG-----SGNSG-VTRSVMTIAFE------------FAFES-Q------------
XP_018436929.1/1-283 KA-STDQ-----SGN-SACARSVMTIAFE------------FGIES-H------------
XP_013592012.1/1-283 KA-STDQ-----SGISGTCARSVMTIAFE------------FGIES-H------------
XP_013640375.1/1-284 KA-STDQ-----SGSSGTCARSVMTIAFE------------FGIES-H------------
XP_013640376.1/1-283 KA-STDQ-----SGSSGTCARSVMTIAFE------------FGIES-H------------
XP_009147709.1/1-283 KA-STDQ-----SGNSGTCARSVMTIAFE------------FGIES-H------------
XP_006306297.1/1-281 -A-SSDQ-----SGS-STCARSVMTIAFE------------FGIES-H------------
XP_010462051.1/1-281 -A-STDQ-----SGN-STCARSVMTIAFE------------FGIES-H------------
XP_010479714.1/1-281 -A-STDQ-----SGN-STCARSVMTIAFE------------FGIES-H------------
XP_010462052.1/1-281 -A-STDQ-----SGN-STCARSVMTIAFE------------FGIES-H------------
XP_010500807.1/1-281 -A-STDQ-----SGN-STCARSVMTIAFE------------FGIES-H------------
NP_849795.1/1-282    -A-STDQ-----SGN-STCARSVMTIAFE------------FGIES-H------------
NP_001077702.1/1-281 -A-STDQ-----SGN-STCARSVMTIAFE------------FGIES-H------------
NP_175627.1/1-281    -A-STDQ-----SGN-STCARSVMTIAFE------------FGIES-H------------
XP_020868586.1/1-281 -A-SADQ-----SGN-STCARSVMTIAFE------------FGIES-H------------
XP_006392953.1/1-282 KA-SSDQ-----SGNT-TCARSVMTIAFE------------FGIES-H------------
XP_018460807.1/1-282 KA-STDQ-----SGNS-TCARSVMTIAFE------------FGVES-H------------
XP_013621760.1/1-283 KA-STDQ-----SGNSGTCARSVMTIAFE------------FGVES-H------------
XP_018451524.1/1-282 KA-ATDQ-----SGNS-TCARSVMTIAFE------------FGVES-H------------
XP_009107148.1/1-282 KA-STDQ-----SGNS-TCARSVMTIAFE------------FGIES-H------------
XP_009107147.1/1-283 KA-STDQ-----SGNS-TCARSVMTIAFE------------FGIES-H------------
XP_009600774.1/1-284 VV-ND--------KSTGVTSKSIMTIAFQ------------FAFES-H------------
XP_016459079.1/1-284 VV-ND--------KSTGVTSKSIMTIAFQ------------FAFES-H------------
XP_019236029.1/1-285 VA-NDL-------KSTGVASKSIMTIAFQ------------FAFES-H------------
XP_009771098.1/1-285 VA-NDF-------KSTGVTSKSIMTVAFQ------------FAFES-H------------
XP_016550962.1/1-285 VA-NDL-------KATDGTSKCIMTMAFQ------------FAFES-H------------
XP_016550960.1/1-285 VA-NDL-------KATDGTSKCIMTMAFQ------------FAFES-H------------
XP_016550961.1/1-285 VA-NDL-------KATDGTSKCIMTMAFQ------------FAFES-H------------
XP_006359469.1/1-285 VA-NDL-------KSTGGTSKSIMTIAFQ------------FAFES-H------------
XP_004252354.1/1-285 VA-NDL-------KSTGGTSKSIMTIAFQ------------FAFES-H------------
XP_015061298.1/1-285 LA-NDL-------KSTGGTSKSIMTIAFQ------------FAFES-H------------
XP_019198775.1/1-285 AP-NSL-------GTNNSATRSVMTIAFQ------------FAYES-H------------
XP_019194412.1/1-285 VL-NDL-------NTNNGAARSVMTIAFQ------------FAFES-H------------
XP_016562656.1/1-283 VA-NDL-------HTVGGTSRSVMTIAFQ------------FAFES-H------------
XP_019267274.1/1-285 VG-NDL-------HTNGGTSRSVMTIAFQ------------FAFES-H------------
XP_019267275.1/1-282 VG-NDL-------HTNGGTSRSVMTIAFQ------------FAFES-H------------
XP_022889649.1/1-285 SL-NGD-------AVISGATRSVMTIAFQ------------FAFES-H------------
XP_022889648.1/1-285 SL-NGD-------AVISGATRSVMTIAFQ------------FAFES-H------------
XP_012852689.1/1-281 PS-SDL-------AVTNGNSRSVMTIAFQ------------FAFEN-H------------
XP_012856468.1/1-279 SL-NGV-------SMTGGATRSVMTIAFQ------------FGFES-H------------
XP_011080516.1/1-285 PS-NDL-------AVTSCTTRSVMTIAFQ------------FAFES-H------------
XP_011072422.1/1-285 PS-NDL-------SSTNGASRSVMTIAFQ------------FAFES-H------------
XP_022851148.1/1-285 AS-NDI-------VVTSGATRSVMTIAFE------------FAFES-H------------
XP_022844668.1/1-285 SS-NDL-------AVTSGTTRSVMTIAFE------------FAFES-H------------
XP_006853643.2/1-288 IS-GEFA-----GGS--GSLRSVLTIAFQ------------FSYEN-N-----------H
XP_010249545.1/1-285 VS-GDYS-----GNS--NSTRSVMTIAFQ------------FPFES-H------------
XP_010249539.1/1-285 VS-GDYS-----GNS--NSTRSVMTIAFQ------------FPFES-H------------
XP_010915409.1/1-284 VS-GDYS-----GNS--TSMRSVMTIAFQ------------FAFES-H------------
XP_008791633.1/1-284 VS-GNYS-----GNS--TSTRSVMTIAFQ------------FAFES-H------------
XP_017235966.1/1-285 GF-GEYN-----DGS--GSTRSVMTIAFE------------FACES-H------------
XP_023761873.1/1-288 LS-GDRN-----VGGGGGSARSVMTIAFE------------FAFES-H------------
XP_021997125.1/1-286 AS-GDRN-----VGN--NCARSVMTIALE------------FAFES-H------------
XP_021990052.1/1-285 LS-GDRD-----VGT--GSARSVMTIAFE------------FAFES-H------------
XP_024979339.1/1-285 TS-GDRS-----VSS--GNARSVMTIAFE------------FAFES-H------------
XP_024979341.1/1-290 TS-GDRS-----VSS--GNARSVMTIAFE------------FAFES-H------------
XP_024979340.1/1-291 TS-GDRS-----VSS--GNARSVMTIAFE------------FAFES-H------------
XP_024979342.1/1-291 TS-GDRS-----VSS--GNARSVMTIAFE------------FAFES-H------------
XP_022016208.1/1-285 LP-GDHN-----SGS--GTIRSVVTIAFE------------FAFDC-H------------
XP_022016207.1/1-311 LP-GDHN-----SGS--GTIRSVVTIAFE------------FAFDC-H------------
XP_022009819.1/1-285 PS-SNYY-----VAS--GSARSVMTIAFE------------FACES-H------------
XP_022009815.1/1-285 PS-SNYY-----VAS--GSARSVMTIAFE------------FACES-H------------
XP_022009809.1/1-285 PS-SNYY-----VAS--GSARSVMTIAFE------------FACES-H------------
XP_023759500.1/1-286 LS-NDQN-----TNN--GTSRSVMTIAFE------------FAFES-H------------
XP_023751740.1/1-285 IS-NDRN-----IAS--GNSRSLMTMAFE------------FAFES-H------------
XP_024971803.1/1-285 VS-GDPN-----TAS--NTARSVMTIAFE------------FAFET-H------------
XP_022033649.1/1-285 LS-SNHN-----VAS--GTARSVMTIAFE------------FACES-H------------
XP_024972195.1/1-285 IS-SDRN-----AAS--GTVRSVMTIAFE------------FAFES-H------------
XP_024972196.1/1-289 IS-SDRN-----AAS--GTVRSVMTIAFE------------FAFES-H------------
XP_020248539.1/1-284 VS-GNAI-----GS--CGSTKSVMTIAFQ------------FAFEN-H------------
XP_020269197.1/1-284 VG-GDSA-----GT--CGSMKSVMTIAFQ------------FAYEN-N------------
XP_020269196.1/1-284 VG-GDSA-----GT--CGSMKSVMTIAFQ------------FAYEN-N------------
XP_009399813.1/1-284 VH-NDYS-----GN--RGNIRSVMTIAFQ------------FAFES-H------------
XP_009399812.1/1-284 VH-NDYS-----GN--RGNIRSVMTIAFQ------------FAFES-H------------
XP_009413853.1/1-284 AS-NDYS-----GN--CGSTKSVMTIAFQ------------FAFES-H------------
XP_024985513.1/1-286 AS-ADYL-----GQ--RGPTKSVMTIAFQ------------FAFES-H------------
XP_023729871.1/1-286 TP-ADYL-----GQ--SGPTKSVMTIAFQ------------FAFEI-H------------
XP_022000974.1/1-282 TP-ADYL-----GQ--SGPTKSVMTIAFQ------------FAFEI-H------------
XP_022023495.1/1-282 TA-ADYV-----GQ--SGSTKSVMTIAFQ------------FAFEI-H------------
XP_010526102.1/1-286 GA-GDSC-----GT--RGSTKSVMTIAFQ------------LAFEV-H------------
XP_019086035.1/1-282 TA-GDSC-----GS--RGNTKSVMTIAFQ------------LAFEM-H------------
XP_010437837.1/1-288 TA-GDSC-----GS--RGNTKSVMTIAFQ------------LAFEM-H------------
XP_010437838.1/1-288 TA-GDSC-----GS--RGNTKSVMTIAFQ------------LAFEM-H------------
XP_022549566.1/1-283 TA-GDSC-----GS--RGNTKSVMTIAFQ------------LAFEM-H------------
XP_013670651.1/1-283 TA-GDSC-----GS--RGNTKSVMTIAFQ------------LAFEM-H------------
XP_020874560.1/1-283 TA-GDSC-----GS--RGNSKSVMTIAFQ------------LAFEM-H------------
XP_020874561.1/1-283 TA-GDSC-----GS--RGNSKSVMTIAFQ------------LAFEM-H------------
XP_020874562.1/1-283 TA-GDSC-----GS--RGNSKSVMTIAFQ------------LAFEM-H------------
NP_195014.1/1-283    TA-GDSC-----GS--RGNSKSVMTIAFQ------------LAFEM-H------------
XP_006412419.1/1-283 TA-GDSC-----GS--RGNTKSVMTIAFQ------------LAFEM-H------------
XP_006412418.1/1-283 TA-GDSC-----GS--RGNTKSVMTIAFQ------------LAFEM-H------------
XP_018471819.1/1-283 TA-GDSC-----GN--RGNTKSVMTIAFQ------------LAFEM-H------------
XP_009125299.1/1-283 TA-GDSC-----GS--RGNTKSVMTIAFQ------------LAFEM-H------------
XP_010432643.1/1-283 TA-GDSC-----GS--RGNTKSVMTIAFQ------------LAFEM-H------------
XP_010432644.1/1-283 TA-GDSC-----GS--RGNTKSVMTIAFQ------------LAFEM-H------------
XP_006283109.1/1-283 TA-GDSC-----GS--RGNTKSVMTIAFQ------------LAFEM-H------------
XP_010437839.1/1-283 TA-GDSC-----GS--RGNTKSVMTIAFQ------------LAFEM-H------------
XP_010437840.1/1-283 TA-GDSC-----GS--RGNTKSVMTIAFQ------------LAFEM-H------------
XP_010447325.1/1-283 TA-GDSC-----GS--RGNTKSVMTIAFQ------------LAFEM-H------------
XP_010447326.1/1-283 TA-GDSC-----GS--RGNTKSVMTIAFQ------------LAFEM-H------------
XP_013622315.1/1-283 TA-GDSC-----GS--RGNTKSVMTIAFQ------------LAFEM-H------------
XP_013656273.1/1-283 TA-GDSC-----GS--RGNTKSVMTIAFQ------------LAFEM-H------------
XP_009108824.1/1-283 TA-GDSC-----GS--RGNTKSVMTIAFQ------------LAFEM-H------------
XP_013730326.1/1-283 TA-GDSC-----GS--RGNTKSVMTIAFQ------------LAFEM-H------------
XP_013730325.1/1-283 TA-GDSC-----GS--RGNTKSVMTIAFQ------------LAFEM-H------------
XP_018447492.1/1-283 TA-GDSC-----GS--RGNTKSVMTIAFQ------------LAFEM-H------------
XP_019415259.1/1-283 AA-GDNS-----GH--S--TKSVMTIAFM------------FACEI-H------------
XP_022980940.1/1-286 AA-GDYA-----CQ--SGKSKSVMTIAFQ------------FVFDV-H------------
XP_023523539.1/1-286 AA-GDYA-----CQ--SGKSKSVMTIAFQ------------FVFDV-H------------
XP_022941184.1/1-286 AA-GDYA-----CQ--SGKSKSVMTIAFQ------------FVFDV-H------------
XP_022134366.1/1-286 AS-GDYA-----GQ--SGKSKSVMTIAFQ------------FVFDV-H------------
XP_004136224.1/1-286 AS-GDCA-----GQ--SGKSKSVMTIAFQ------------FVFDV-H------------
XP_008466070.1/1-286 AS-GDCA-----GQ--SGKSKSVMTIAFQ------------FVFDV-H------------
XP_022976315.1/1-286 AS-GNCA-----GQ--SGKSKSVMTIAFQ------------FVFDV-H------------
XP_022937345.1/1-286 AS-GDCA-----GQ--SGKSKSVMTIAFQ------------FVFDV-H------------
XP_023534956.1/1-286 AS-GDCA-----GQ--SGKSKSVMTIAFQ------------FVFDV-H------------
XP_023535295.1/1-286 AS-GDCA-----GQ--SGKSKSVMTIAFQ------------FVFDV-H------------
XP_019175908.1/1-285 QT-GDQS-----KQ--CSTSKSVMTIAFQ------------FAFEI-H------------
XP_019175907.1/1-285 QT-GDQS-----KQ--CSTSKSVMTIAFQ------------FAFEI-H------------
XP_009626659.1/1-285 PN-GDYS-----KN--SGSTKSVMTIAFQ------------FAFEI-H------------
XP_019266312.1/1-285 PN-GDYS-----KN--SGSTKSVMTIAFQ------------FAFEI-H------------
XP_009781184.1/1-285 PN-GDYS-----KN--SSSTKSVMTIAFQ------------FAFEI-H------------
XP_016488110.1/1-285 PN-GDYS-----KN--SSSTKSVMTIAFQ------------FAFEI-H------------
XP_016537761.1/1-285 PT-GDHS-----KS--SGSTKSVMTIAFQ------------FAFEI-H------------
XP_006359741.1/1-285 PT-GDHS-----KN--SGSTKSVMTIAFQ------------FAFEI-H------------
XP_010325078.1/1-288 PT-GDHS-----KN--SGSAKSVMTIAFQ------------FAFEI-H------------
XP_004245155.1/1-285 PT-GDHS-----KN--SGSAKSVMTIAFQ------------FAFEI-H------------
XP_015085523.1/1-285 PT-GDHS-----KN--SGSAKSVMTIAFQ------------FAFEI-H------------
XP_022868182.1/1-285 AS-GDYS-----RH--LGSAKSVMTIAFQ------------FAFEI-H------------
XP_022868181.1/1-285 AS-GDYS-----RH--LGSAKSVMTIAFQ------------FAFEI-H------------
XP_022854559.1/1-285 AS-GDCS-----RH--LGSAKSIMTIAFQ------------FAFEI-H------------
XP_011081776.1/1-285 SP-GDHS-----RN--AGTAKSVMTIAFQ------------FAFEI-H------------
XP_012857948.1/1-282 C----GD-----RN--NGSVKSVMTIAFQ------------FAFEI-H------------
XP_021847759.1/1-284 ALADNNG-----S----RSGKSVMTIAFQ------------FAFEV-H------------
XP_010668539.1/1-284 TLADNSG-----S----GGGKSVMTIAFQ------------FAFEI-H------------
XP_021739137.1/1-284 SLADNSG-----S----GNGKSVMTIAFQ------------FAFEI-H------------
XP_021737291.1/1-284 SLADNSG-----S----GSGKSVMTIAFQ------------FAFEI-H------------
XP_010047286.1/1-285 AV-GDNS-----GH--SGNTKSVMTIAFQ------------FAFEL-H------------
XP_003603630.2/1-285 AG-GDNS-----GH--SGITKSVMTIAFQ------------FAFEA-H------------
XP_004501146.1/1-286 AGGGANS-----GH--SGNTKSVMTIAFQ------------FAFEV-H------------
XP_012571693.1/1-286 AGGGANS-----GH--SGNTKSVMTIAFQ------------FAFEV-H------------
XP_012571692.1/1-286 AGGGANS-----GH--SGNTKSVMTIAFQ------------FAFEV-H------------
XP_019417530.1/1-285 AG-GDNS-----GY--LGSTKSVMTIAFQ------------FACEV-H------------
XP_019417531.1/1-285 AG-GDNS-----GY--LGSTKSVMTIAFQ------------FACEV-H------------
XP_016180268.1/1-286 AG-GENS-----SH--SGSTKSVMTIAFQ------------FAFEV-H------------
XP_025623723.1/1-286 AG-GENS-----SH--SGSTKSVMTIAFQ------------FAFEV-H------------
XP_015945672.1/1-286 AG-GENS-----SH--SGSTKSVMTIAFQ------------FAFEV-H------------
XP_007137231.1/1-287 AA-GDNS-----GH--SGSTKSVMTIAFQ------------FAFEV-H------------
XP_022636494.1/1-293 AA-GDNS-----GH--SGSTKSVMTIAFQ------------FAFEV-H------------
XP_017409217.1/1-287 AA-GDNA-----GH--SGSTKSVMTIAFQ------------FAFEV-H------------
XP_014501941.1/1-287 AA-GDNS-----GH--SGSTKSVMTIAFQ------------FAFEV-H------------
XP_003526496.1/1-286 AA-SDNS-----AH--SGSTKSVMTIAFQ------------FAFEV-H------------
XP_003522716.1/1-286 AA-GDNS-----GH--SGSTKSVMTIAFQ------------FAFEV-H------------
XP_020217689.1/1-286 AA-GDNS-----GH--SGSTKSVMTIAFQ------------FAFDV-H------------
XP_016683069.1/1-283 AT-GDKS-----SR---GSTKSVMTIAFQ------------FVYEM-Q------------
XP_016677926.1/1-283 AT-GDKS-----SR---GSTKSVMTIAFQ------------FVYEM-Q------------
XP_017643110.1/1-283 AT-GDKS-----SR---GSTKSVMTIAFQ------------FVYEM-Q------------
XP_017643111.1/1-283 AT-GDKS-----SR---GSTKSVMTIAFQ------------FVYEM-Q------------
XP_012451775.1/1-283 AT-GDKS-----SC---GSTKSVMTIAFQ------------FVHEM-Q------------
XP_016744766.1/1-285 TT-GDRS-----GR--CGNSKSVMTIAFQ------------FVYEI-H------------
XP_017643921.1/1-285 TT-GDRS-----GR--CGNSKSVMTIAFQ------------FVCEI-H------------
XP_012436119.1/1-285 TT-GDRS-----GR--CGNSKSVMTIAFQ------------FVYEI-H------------
XP_016681632.1/1-285 TT-GDRS-----GR--CGNSKSVMTIAFQ------------FVYEI-H------------
XP_016689618.1/1-285 ST-GDNS-----GR--NGSTKSVMTIAFQ------------FVYEI-H------------
XP_016689617.1/1-285 ST-GDNS-----GR--NGSTKSVMTIAFQ------------FVYEI-H------------
XP_012445947.1/1-285 ST-GDNS-----GR--NGSTKSVMTIAFQ------------FVYEI-H------------
XP_012445944.1/1-285 ST-GDNS-----GR--NGSTKSVMTIAFQ------------FVYEI-H------------
XP_012445945.1/1-285 ST-GDNS-----GR--NGSTKSVMTIAFQ------------FVYEI-H------------
XP_017614652.1/1-285 ST-GDNS-----GR--NGSTKSVMTIAFQ------------FVYEI-H------------
XP_016732732.1/1-285 ST-GDNS-----GR--NGSTKSVMTIAFQ------------FVYEI-H------------
XP_016732731.1/1-285 ST-GDNS-----GR--NGSTKSVMTIAFQ------------FVYEI-H------------
XP_022754596.1/1-285 AT-ADHS-----GR--CRSTKSVMTIAFQ------------FVYEI-H------------
XP_022750159.1/1-285 AT-ADNS-----GR--CGSTKSVMTIAFQ------------FLYEI-H------------
XP_022729737.1/1-285 AT-GDNS-----GR--CGSAKSVMTIAFQ------------FVYEI-H------------
XP_022729738.1/1-285 AT-GDNS-----GR--CGSAKSVMTIAFQ------------FVYEI-H------------
XP_021276132.1/1-285 AT-GDHS-----GR--CGSTKSVMTIAFQ------------FVYEI-H------------
XP_017983822.1/1-285 AT-GDHS-----GR--CGSTKSVMTIAFQ------------FVYEI-H------------
XP_002283717.3/1-285 AS-SDNS-----GH--TGSAKSVMTISFQ------------FAFEM-H------------
XP_024446213.1/1-284 AS-GDLS-----GR--SGCTKSVMTIAFQ------------FAFEM-H------------
XP_024446215.1/1-284 AS-GDLS-----GR--SGCTKSVMTIAFQ------------FAFEM-H------------
XP_011006428.1/1-284 AS-GDLS-----GR--RGCTKSVMTIAFQ------------FAFEM-H------------
XP_024459734.1/1-284 AS-GDLS-----GR--SGCTKSVMTIAFQ------------FAFEM-H------------
XP_011019595.1/1-284 AS-GDLS-----GR--SGCTKSVMTIAFQ------------FAFEM-H------------
XP_015573174.1/1-284 AS-GDLP-----GH--CGSTKSVMTIAFQ------------FAFEI-H------------
XP_012077376.1/1-284 AS-GDFS-----GH--CGSTKSVMTIAFQ------------FAFEM-H------------
XP_021608403.1/1-284 AS-GDVS-----GH--CGSTKSVMTIAFQ------------FAFEL-H------------
XP_021608402.1/1-284 AS-GDVS-----GH--CGSTKSVMTIAFQ------------FAFEL-H------------
XP_021597909.1/1-284 AS-GDLS-----GN--CGSTKSVMTIAFQ------------FAFEL-H------------
XP_021642839.1/1-284 AS-GDFS-----GH--CGSSKSVMTIAFQ------------FAFEL-H------------
XP_021689146.1/1-284 AS-SDFP-----GH--CGSTKSVMTIAFQ------------FAFEL-H------------
XP_018805752.1/1-285 AS-GENS-----GH--CGSTKSVMTIAFQ------------FAIEV-H------------
XP_023918167.1/1-285 AS-GENS-----GQ--CGSTKSVMTIAFQ------------FAFEI-H------------
XP_018845499.1/1-285 TS-SENS-----GK--SGSTKSVMTIAFQ------------FAFEI-H------------
XP_018845500.1/1-285 TS-SENS-----GK--SGSTKSVMTIAFQ------------FAFEI-H------------
NP_001280750.1/1-285 AS-GDNA-----GH--SGNTKSVMTIAFQ------------FAFEI-H------------
NP_001280796.1/1-285 AS-GDNA-----GH--SGNTKSVMTIAFQ------------FAFEI-H------------
XP_009367440.1/1-285 AS-GDNA-----GH--SGNTKSVMTIAFQ------------FAFEI-H------------
XP_017182108.1/1-285 AS-GDNA-----GH--SGNTKSVMTIAFQ------------FAFEI-H------------
XP_004291167.1/1-285 SS-GDIA-----GH--CGSTKSVMTIAFQ------------FAFEI-H------------
XP_024162655.1/1-285 SS-GDNA-----GH--CGNTKSVMTIAFQ------------FAFEI-H------------
XP_021819449.1/1-284 AS-GDNA-----GH--SGNTKSVMTIAFQ------------FAFEI-H------------
XP_007225281.1/1-285 AS-GDNA-----GH--SGNTKSVMTIAFQ------------FAFEI-H------------
XP_008220092.1/1-285 AS-GDNA-----GQ--SGNTKSVMTIAFQ------------FAFEI-H------------
XP_006450706.1/1-285 AS-GDSS-----TQ--CGSTKSVITIAFQ------------FAFEM-H------------
XP_006476026.1/1-285 AS-GDSS-----TQ--CGSTKSVITIAFQ------------FAFEM-H------------
XP_024930942.1/1-285 AS-GDNA-----SN--SGNRKSVMTIAFQ------------FAFEM-H------------
XP_010102406.2/1-285 TS-GDNA-----GN--SGNRKSVMTIAFQ------------FAFEM-H------------
XP_024540686.1/1-258 G----KR-------KNHKFARSILTIAFQ------------FMYEV-G------------
XP_024399220.1/1-283 NGESKSN-------GSSSQVRSVLTIAFQ------------FAYEV-H------------
XP_024399219.1/1-283 NGESKSN-------GSSSQVRSVLTIAFQ------------FAYEV-H------------
XP_024399215.1/1-283 NGESKSN-------GSSSQVRSVLTIAFQ------------FAYEV-H------------
XP_024390746.1/1-284 NGDAKSS-------NSPGQMRSVLTIAFQ------------FAYEV-H------------
XP_024390752.1/1-284 NGDAKSS-------NSPGQMRSVLTIAFQ------------FAYEV-H------------
XP_024373354.1/1-283 NGDVKSN-------SSSGQMRSVLTIAFQ------------FAYEV-H------------
XP_024373357.1/1-283 NGDVKSN-------SSSGQMRSVLTIAFQ------------FAYEV-H------------
XP_024370817.1/1-284 NGDTKSN-------GTSGQMRSVLTIAFQ------------FAYEV-H------------
XP_024370815.1/1-284 NGDTKSN-------GTSGQMRSVLTIAFQ------------FAYEV-H------------
XP_024370814.1/1-284 NGDTKSN-------GTSGQMRSVLTIAFQ------------FAYEV-H------------
XP_024386785.1/1-285 NGDSKSN-------GTSSHMRSVLTIAFQ------------FAYEV-H------------
XP_024386782.1/1-285 NGDSKSN-------GTSSHMRSVLTIAFQ------------FAYEV-H------------
XP_024386791.1/1-285 NGDSKSN-------GTSSHMRSVLTIAFQ------------FAYEV-H------------
XP_002961227.1/1-277 FPDE-----------SGCHLRSVLTLAFQ------------FLFEA-H------------
XP_002969458.1/1-277 FPDE-----------SGCHLRSVLTLAFQ------------FLFEA-H------------
XP_024533017.1/1-265 FPDD-FS-------GSGGNMRSVLTMAFQ------------FVFEA-H------------
XP_011076983.1/1-261 NATN-VG-------TAASKPSSVLILAFQ------------FPFET-H------------
XP_011076980.1/1-264 NATN-VG-------TAASKPSSVLILAFQ------------FPFET-H------------
XP_011076981.1/1-263 NATN-VG-------TAASKPSSVLILAFQ------------FPFET-H------------
XP_011084330.1/1-264 NARN-AQ-------AAISHPLSMLIVAFQ------------FPSEA-D------------
XP_011084333.1/1-264 NARN-AQ-------AAISHPLSMLIVAFQ------------FPSEA-D------------
XP_011084331.1/1-264 NARN-AQ-------AAISHPLSMLIVAFQ------------FPSEA-D------------
XP_012834933.1/1-260 NTRN-GA-----------QAPSMLIVAFQ------------FPFEA-H------------
XP_012834932.1/1-260 NTRN-GA-----------QAPSMLIVAFQ------------FPFEA-H------------
XP_012834931.1/1-260 NTRN-GA-----------QAPSMLIVAFQ------------FPFEA-H------------
XP_012834934.1/1-260 NTRN-GA-----------QAPSMLIVAFQ------------FPFEA-H------------
XP_002274194.1/1-284 GCTA-S----------SSDSRSVLIIAFQ------------FLFES-H------------
XP_019079956.1/1-284 GCTA-S----------SSDSRSVLIIAFQ------------FLFES-H------------
XP_023759583.1/1-283 NQRAS---------NQAGPTKSVMTIAFQ------------FGFEI-H------------
XP_020087984.1/1-275 VCGDPSG--------RYSATRSVMTIAFQ------------FAFES-H------------
XP_015693848.1/1-289 ISGVNGGGGGGACAA--ASPKAVMTIAFQ------------FAFES-H------------
XP_015624690.1/1-290 ISGVNGGGGGCAAAAASSSSKAVMTIAFQ------------FAFDG-H------------
XP_008655743.1/1-292 TSGSGGPG---TAAAACAGSKAVMTIVFQ------------FAFES-H------------
XP_020183693.1/1-281 IPGHSR-----------SSSKAVMTIAFQ------------FAFES-H------------
XP_020183687.1/1-281 IPGHSR-----------SSSKAVMTIAFQ------------FAFES-H------------
XP_003565084.1/1-284 MTGSGS--------VNGAGMKAVMTIAFQ------------FAFES-H------------
XP_002454995.1/1-286 INGS-GPG---N--AASAGSKAVMTIVFQ------------FAFES-H------------
XP_012701181.1/1-286 MP-VGGSG---N--AACAGSKAVMTIAFQ------------FAFES-H------------
XP_012701180.1/1-286 MP-VGGSG---N--AACAGSKAVMTIAFQ------------FAFES-H------------
XP_012701179.1/1-286 MP-VGGSG---N--AACAGSKAVMTIAFQ------------FAFES-H------------
XP_025816436.1/1-287 IHGAGGSG---N--AACAGSKAVMTIAFQ------------FAFES-H------------
XP_018726848.1/1-227 -----------DQQAVGSGGGSLLTVAFQ------------ILV-D-LVP--------TA
XP_018726849.1/1-227 -----------DQQAVGSGGGSLLTVAFQ------------ILV-D-LVP--------TA
XP_019193052.1/1-216 ------------------LAGSLLTISFQ------------ILV-D-SVP--------TA
XP_019193050.1/1-216 ------------------LAGSLLTISFQ------------ILV-D-SVP--------TA
XP_021834047.1/1-194 ------------GMGDSGFGGSLLTIAFQ------------ILV-D-YAP--------TA
XP_010522330.1/1-229 -NGG------SMGDGAGEAGGSLLTVAFQ------------ILV-D-SVP--------TA
XP_009802524.1/1-219 ------------------AGGSLLTIAFQ------------IFV-D-SVP--------TA
XP_009802526.1/1-219 ------------------AGGSLLTIAFQ------------IFV-D-SVP--------TA
XP_009802525.1/1-219 ------------------AGGSLLTIAFQ------------IFV-D-SVP--------TA
XP_009802527.1/1-219 ------------------AGGSLLTIAFQ------------IFV-D-SVP--------TA
XP_019250148.1/1-219 ------------------AGGSLLTIAFQ------------ILV-D-SVP--------TS
XP_019250149.1/1-219 ------------------AGGSLLTIAFQ------------ILV-D-SVP--------TS
XP_016449923.1/1-219 ------------------AGGSLLTIAFQ------------ILV-D-SVP--------TA
XP_009629760.1/1-219 ------------------AGGSLLTIAFQ------------ILV-D-SVP--------TA
XP_009629762.1/1-219 ------------------AGGSLLTIAFQ------------ILV-D-SVP--------TA
XP_010234287.2/1-230 -----------NGGCVGGGGGSLLTVAFQ------------ILV-D-SVP--------TA
XP_021806177.1/1-204 -----------GGMGDSGSGGSLLTVAFQ------------ILV-D-SAP--------TA
XP_016580642.1/1-229 ----------CRGAYNDGSGGSLLTVAFQ------------ILV-N-SVP--------TA
XP_008667763.1/1-266 ----------GGGGGGVGSGGSLLTVAFQ------------ILV-D-SVP--------TA
XP_006352040.1/1-227 ------------GAYNHGSGGSLLTVAFQ------------ILV-D-LVP--------VA
XP_009611638.1/1-223 ---------------RVGCGGSLLTVAFQ------------ILV-D-SIP--------TA
XP_009611623.1/1-223 ---------------RVGCGGSLLTVAFQ------------ILV-D-SIP--------TA
XP_016510654.1/1-223 ---------------RVGSGGSLLTVAFQ------------ILV-D-SIP--------TA
XP_009766038.1/1-223 ---------------RVGCGGSLLTVAFQ------------ILV-D-SIP--------TA
XP_020210648.1/1-236 -----------P-IIETGSGGCLLTVAFQ------------ILV-D-SAP--------TA
XP_007143210.1/1-219 ----------------VGSGGCLLTVAFQ------------ILV-D-SVP--------TS
XP_014491869.1/1-226 -----------S-IGEVGSGGCLLTVAFQ------------ILV-D-SVP--------TS
XP_022633652.1/1-226 -----------S-IGEVGSGGCLLTVAFQ------------ILV-D-SVP--------TS
XP_017412341.1/1-227 -----------P-IREVGSGGCLLTVAFQ------------ILV-D-SVP--------TS
XP_022011155.1/1-226 -----------SGVLEVGTGGSIITTGLQ------------ILF-D-SAP--------TA
XP_003555330.2/1-228 -----------PICEAGSGGGCLLTVAFQ------------ILV-D-SAP--------TS
XP_014628518.1/1-229 -----------PICEAGSGGGCLLTVAFQ------------ILV-D-SAP--------TS
XP_017647009.1/1-225 -----------IGITEIGSGGSLVTLAFQ------------ILV-D-SAP--------NS
XP_016711277.1/1-225 -----------IGITEIGSGGSLVTLAFQ------------ILV-D-SAP--------NS
XP_012457027.1/1-225 -----------IKITEIGSGGSLVTLAFQ------------ILV-D-SAP--------NS
XP_016723454.1/1-225 -----------IEITEIGSGGSLVTLAFQ------------ILV-D-SAP--------NS
XP_020978973.1/1-214 ----------------ASSGGSLLTVAFQ------------ILV-D-SAP--------TA
XP_016202355.1/1-214 ----------------ASSGGSLLTVAFQ------------ILV-D-SAP--------TA
XP_015964876.1/1-214 ----------------ASSGGSLLTVAFQ------------ILV-D-SAP--------TA
XP_025699645.1/1-214 ----------------ASSGGSLLTVAFQ------------ILV-D-SAP--------TA
XP_008441375.1/1-228 -----------PGILEFGSGGSLLTVAFQ------------ILV-D-SVP--------TA
XP_004154226.2/1-228 -----------PGILEFGAGGSLLTVAFQ------------ILV-D-SVP--------TA
XP_023519981.1/1-226 -----------AGILEFGSGGSLLTVAFQ------------ILV-D-SVP--------TA
XP_023001231.1/1-226 -----------AGILEFGSGGSLLTVAFQ------------ILV-D-SVP--------TA
XP_022933178.1/1-226 -----------AGILEFGSGGSLLTVAFQ------------ILV-D-SVP--------TA
XP_004511340.1/1-228 ------------PIPDLLSGGSLLTVAFH------------ILV-D-SVT--------NA
XP_002979015.2/1-232 -QHD------SLEVDRPRVPGSLLTVAFQ------------ILV-S-NVP--------SA
XP_002988402.1/1-232 -QHD------SLEVDRPRVPGSLLTVAFQ------------ILV-S-NVP--------SA
XP_024390340.1/1-238 -AGQ------GAESGRGAGRGSLVTVAFQ------------ILV-S-SVP--------SA
XP_024368074.1/1-235 -PGQ------LTESG--RGSGSLLTVAFQ------------ILV-S-SVP--------SA
XP_024401280.1/1-235 -GGQ------LTEIG--RGTGSLLTVAFQ------------ILV-S-SIP--------SA
XP_024394840.1/1-235 -GSQ------LTESS--RGTGSLLTVAFQ------------ILV-S-SIP--------SA
XP_024394838.1/1-235 -GSQ------LTESS--RGTGSLLTVAFQ------------ILV-S-SIP--------SA
XP_017256650.1/1-227 ------------PGSKPTSAGSLLTIAFQ------------ILV-D-SIP--------TA
XP_019186546.1/1-227 ------------LSPEIGSGGSLITVAFQ------------VLV-D-SVP--------TA
XP_024988375.1/1-226 ------------GDLDVGTGGSLLTIAFQ------------ILV-D-SVP--------TS
XP_017231769.1/1-226 -----------EQILEVGSGGSLITVAFQ------------ILV-D-SVA--------TA
XP_009108529.1/1-235 -AEG------EVVTSTASNCGSLLTVAFQ------------ILV-D-SVP--------TA
XP_018469534.1/1-236 -AGG------GGNNLEVVNSGSLLTVAFQ------------ILV-D-SVP--------TA
XP_018467466.1/1-236 -AGG------GGNNLEVVNSGSLLTVAFQ------------ILV-D-SVP--------TA
XP_022543253.1/1-234 -AEG------GGNNRVVVNAGSLLTVAFQ------------IIV-D-SVP--------TA
XP_009135181.1/1-234 -AEG------GGNNRVVVNAGSLLTVAFQ------------IIV-D-SVP--------TA
XP_013642975.1/1-234 -AEG------GGNNRVVVNAGSLLTVAFQ------------IIV-D-SVP--------TA
XP_009135189.1/1-234 -AEG------GGNNRVVVNAGSLLTVAFQ------------IIV-D-SVP--------TA
XP_018515487.1/1-234 -AEG------GGNNRVVVNAGSLLTVAFQ------------IIV-D-SVP--------TA
XP_013623861.1/1-234 -AEG------GGNNLEVVNSGSLLTVAFQ------------IIV-D-SVP--------TA
XP_013623905.1/1-234 -AEG------GGNNLEVVNSGSLLTVAFQ------------IIV-D-SVP--------TA
XP_013623953.1/1-236 -AEG------GGNNLEVVNSGSLLTVAFQ------------IIV-D-SVP--------TA
XP_013623587.1/1-236 -AEG------GGNNLEVVNSGSLLTVAFQ------------IIV-D-SVP--------TA
XP_013623995.1/1-236 -AEG------GGNNLEVVNSGSLLTVAFQ------------IIV-D-SVP--------TA
XP_013623820.1/1-236 -AEG------GGNNLEVVNSGSLLTVAFQ------------IIV-D-SVP--------TA
XP_022926381.1/1-227 -GSN------GGGILEVGSGGSLITVAFQ------------ILV-D-SVP--------TA
XP_023517485.1/1-227 -GSN------GGGILEVGSGGSLITVAFQ------------ILV-D-SVP--------TA
XP_023002930.1/1-227 -GTN------GRGILEVGSGGSLITVAFQ------------ILV-D-SVP--------TA
XP_004134971.1/1-229 -GNN------GGGILELGSGGSLITVAFQ------------ILV-D-SVP--------TA
XP_008439968.1/1-229 -GNN------GGGILELGSGGSLITVAFQ------------ILV-D-SVP--------TA
XP_022950176.1/1-227 --AN------GGEILELGSGGSLITVAFQ------------ILV-D-SVP--------TA
XP_022979090.1/1-227 --AN------GGEILELGSGGSLITVAFQ------------ILV-D-SVP--------TA
XP_023543388.1/1-249 -GAN------GGEILELGSGGSLITVAFQ------------ILV-D-SVP--------TA
XP_023543387.1/1-227 --AN------GGEILELGSGGSLITVAFQ------------ILV-D-SVP--------TA
XP_009134323.1/1-228 -------------STASESCGSLLTVAFQ------------ILV-D-SVP--------TA
XP_019176002.1/1-227 -------------PEVGSGGGSLLTVAFQ------------ILV-D-SVP--------TA
XP_015070655.1/1-221 ----------------AGTGGSLLTVAFQ------------ILV-D-SVP--------TA
XP_015070652.1/1-221 ----------------AGTGGSLLTVAFQ------------ILV-D-SVP--------TA
XP_025885589.1/1-221 ----------------VGTGGSLLTVAFQ------------ILV-D-SVP--------TA
XP_019422236.1/1-227 --------------VDVGSGGSLLTVAFQ------------ILV-D-SAP--------TA
XP_007157166.1/1-226 --------------HDVGSGGSLLTVAFQ------------ILV-D-SAP--------TA
XP_003538765.1/1-227 --------------HEVGSGGSLLTVGFQ------------ILV-D-SAP--------TA
XP_003517589.1/1-227 --------------HDVGSGGSLLTVAFQ------------ILV-D-SAP--------TA
XP_006573890.1/1-227 --------------HDVGSGGSLLTVAFQ------------ILV-D-SAP--------TA
XP_011000687.1/1-226 --------------LDVGSGGSLLTVAFQ------------ILV-D-SVP--------SV
XP_024436817.1/1-226 --------------LDVGSGGSLLTVAFQ------------ILV-D-SVP--------SV
XP_024436820.1/1-226 --------------LDVGSGGSLLTVAFQ------------ILV-D-SVP--------SV
XP_002518520.2/1-226 --------------LDVGSGGALVTVAFQ------------ILV-D-SIP--------TA
XP_015574304.1/1-226 --------------LDVGSGGALVTVAFQ------------ILV-D-SIP--------TA
XP_021642058.1/1-227 --------------VDVGSGGALLTVAFQ------------ILV-D-SVP--------TA
XP_012091395.1/1-226 --------------LDVGSGGALLTVAFQ------------ILV-D-SVP--------TA
XP_021635824.1/1-226 --------------HDVRSGGALLTVAFQ------------ILV-D-SVP--------TA
XP_021596887.1/1-226 --------------HDVRSGCALLTVAFQ------------ILV-D-SAP--------TA
XP_010057969.1/1-228 --------------LEIGSGGSLLTVAFQ------------ILV-D-TVP--------SA
XP_006413738.1/1-245 -GGEGSNLDL--VTSTASNCGSLLTVAFQ------------ILV-D-SVP--------TA
XP_013630219.1/1-235 --------EGEVVTSTASNCGSLLTVAFQ------------ILV-D-SVP--------TA
XP_013656136.1/1-235 --------EGEVVTSTASNCGSLLTVAFQ------------ILV-D-SVP--------TA
XP_013685757.1/1-235 --------EGEVVTSTASNCGSLLTVAFQ------------ILV-D-SVP--------TA
XP_013696499.1/1-240 --GDGNNLEVVTS--TASNCGSLLTVAFQ------------ILV-D-SVP--------TA
XP_009137213.1/1-240 --GDGNNLEVVTS--TASNCGSLLTVAFQ------------ILV-D-SVP--------TA
XP_013598423.1/1-241 -GGDGNNLEVVTS--TASNCGSLLTVAFQ------------ILV-D-SVP--------TA
XP_013717184.1/1-241 -GGDGNNLEVVTS--TASNCGSLLTVAFQ------------ILV-D-SVP--------TA
XP_018480642.1/1-241 -GVDGNNLEVVTS--TASNGGSLLTVAFQ------------ILV-D-SVP--------TA
XP_018477464.1/1-241 -GVDGNNLEVVTS--TASNGGSLLTVAFQ------------ILV-D-SVP--------TA
NP_193906.2/1-248    -GGEGNNLEVVT--TTGSCGGSLLTVAFQ------------ILV-D-SVP--------TA
XP_020873301.1/1-248 -GGEGNNLEVVTTTATGSSGGSLLTVAFQ------------ILV-D-SVP--------TA
XP_006285476.1/1-249 -GGEGNNLEVVTT--TENNGGSLLTVAFQ------------ILV-D-SVP--------TA
XP_010434132.1/1-252 -GGEGNNLEVVTT-TTGNNGGSLLTVAFQ------------ILV-D-SVP--------TA
XP_010439429.1/1-252 -GGEGNNLEVVTT-TTGNNGGSLLTVAFQ------------ILV-D-SVP--------TA
XP_010449009.1/1-254 -GGEGNNLEVVTT-TTGNNGGSLLTVAFQ------------ILV-D-SVP--------TA
XP_010432053.1/1-234 -----QEVASST--SFGSCGGSLLTVAFQ------------ILV-D-SVP--------TA
XP_020876375.1/1-234 -----QEVVSST--SSGGCGGSLLTVAFQ------------ILV-D-SVP--------TA
XP_023636874.1/1-234 -----QEVVSST--SAGSCGGSLLTVAFQ------------ILV-D-SVP--------TA
XP_010422328.1/1-234 -----QEVVSST--SFGSCGGSLLTVAFQ------------ILV-D-SVP--------TA
XP_010455798.1/1-234 -----QEVVSST--SFGSCGGSLLTVAFQ------------ILV-D-SVP--------TA
NP_567274.1/1-235    -----QEMVSTT--SSGSCGGSLLTVAFQ------------ILV-D-SVP--------TA
XP_024009884.1/1-236 -----QEVVSSSSSSSGSC-GSLLTVAFQ------------ILV-D-SVP--------TA
XP_018452346.1/1-236 -----DQEVVSSSNASGSC-GSLLTVAFQ------------ILV-D-SVP--------TA
XP_013611160.1/1-236 -----DQEVVSSSSASGSC-GSLLTVAFQ------------ILV-D-SVP--------TA
XP_009114536.1/1-235 -----QEVVSSSTTASGSC-GSLLTVAFQ------------ILV-D-SVP--------TA
XP_013662000.1/1-235 -----QEVVSSSTTASGSC-GSLLTVAFQ------------ILV-D-SVP--------TA
XP_018472664.1/1-233 -----QEVVAS-STASESC-GSLLTVAFQ------------ILV-D-SVP--------TA
XP_022555047.1/1-234 -----QEVVSS-STASESC-GSLLTVAFQ------------ILV-D-SVP--------TA
XP_013735469.1/1-234 -----QEVVSS-STASESC-GSLLTVAFQ------------ILV-D-SVP--------TA
XP_013629800.1/1-234 -----QEVVSS-STASESC-GSLLTVAFQ------------ILV-D-SVP--------TA
XP_013735464.1/1-234 -----QEVVSS-STASESC-GSLLTVAFQ------------ILV-D-SVP--------TA
XP_022571893.1/1-234 -----QEVVSS-STASESC-GSLLTVAFQ------------ILV-D-SVP--------TA
XP_010538642.1/1-236 -----GEENQEVASSSGGC-GSLLTVAFQ------------ILV-D-SVP--------TA
XP_019057303.1/1-229 --------NQE-VDSSRVC-GSLLTVAFQ------------ILV-D-SVP--------TA
XP_016545426.1/1-226 ------------AENTNVAGGSLLTVAFQ------------ILV-D-SVP--------TA
XP_009793614.1/1-227 ------------NPETNAAGGSLLTVAFQ------------ILV-D-SVP--------TA
XP_016508926.1/1-227 ------------NPETNAAGGSLLTVAFQ------------ILV-D-SVP--------TA
XP_016560096.1/1-235 ------------SSDVGSVGGSLLTVAFQ------------ILV-D-SVP--------TA
XP_021993721.1/1-227 --------------VEVGNGGSLLTVAFQ------------ILV-D-SVP--------TA
XP_022036928.1/1-226 --------------LEVGTGGSLITVAFQ------------ILV-D-SVP--------TA
XP_023742870.1/1-226 --------------LVGGTGGSLLTIAFQ------------ILV-D-SVP--------TS
XP_006352605.1/1-223 --------------NSNAAGGSLLTVAFQ------------ILV-D-SVP--------TA
XP_010327237.1/1-223 --------------NSNIAGGSLLTVAFQ------------ILV-D-SVP--------TA
XP_015055984.1/1-223 --------------NSNTAGGSLLTVAFQ------------ILV-D-SVP--------TA
XP_009609606.1/1-229 ------------NLETNSAGGSLLTVAFQ------------ILV-D-SVP--------TA
XP_009609608.1/1-229 ------------NLETNSAGGSLLTVAFQ------------ILV-D-SVP--------TA
XP_018628742.1/1-229 ------------NLETNSAGGSLLTVAFQ------------ILV-D-SVP--------TA
XP_019237831.1/1-229 ------------NPETNAAGGSLLTVAFQ------------ILV-D-SVP--------TA
XP_018849836.1/1-227 ------------GILGVGSGGSLLTVAFQ------------ILV-D-SVP--------TA
XP_007214541.1/1-230 ------------GILDVGSGGSLLTVAFQ------------ILV-D-SVP--------TA
XP_021821565.1/1-230 ------------GILDVGSGGSLLTVAFQ------------ILV-D-SVP--------TA
XP_008224792.1/1-230 ------------GILDVGSGGSLLTVAFQ------------ILV-D-SVP--------TA
XP_024198966.1/1-227 ------------GILDVGSGGSLLTVAFQ------------ILV-D-SVP--------TA
XP_004293268.1/1-227 ------------GILDVGSGGSLLTVAFQ------------ILV-D-SVP--------TA
XP_009364098.1/1-227 ------------GILDVGSGGSLLTVAFQ------------ILV-D-SVP--------TA
XP_008371114.1/1-227 ------------GILDVGSGGSLLTVAFQ------------ILV-D-SVP--------TA
XP_008384034.1/1-227 ------------GTLDVGSGGSLLTVAFQ------------ILV-D-SVP--------TA
XP_009340946.1/1-227 ------------GALDVGSGGSLLTVAFQ------------ILV-D-SIP--------TA
XP_009357415.1/1-227 ------------GALDVGSGGSLLTVAFQ------------ILV-D-SIP--------TA
XP_011037708.1/1-226 ------------GILDVASGGSLLTVAFQ------------ILV-D-SVP--------TA
XP_024454819.1/1-226 ------------GILDVGSGGSLLTVAFQ------------ILV-D-SVP--------TA
XP_015884145.1/1-226 ------------AILEVGSGGSLLTVAFQ------------ILV-D-SAP--------TA
XP_024024693.1/1-228 ------------GILEVGSGGSLLTVAFQ------------ILV-D-SVP--------TA
XP_024024692.1/1-228 ------------GILEVGSGGSLLTVAFQ------------ILV-D-SVP--------TA
XP_018820351.1/1-226 ------------GIHEVGSCGSLLTVAFQ------------ILV-D-SVP--------SA
XP_023904570.1/1-226 ------------GIVEVGSGGSLLTVAFQ------------ILV-D-SVP--------TA
XP_023904569.1/1-226 ------------GIVEVGSGGSLLTVAFQ------------ILV-D-SVP--------TA
XP_006449076.1/1-226 ------------GILEVGSGGSLLTVAFQ------------ILV-D-SVP--------TA
XP_006468235.1/1-226 ------------GILEVGSGGSLLTVAFQ------------ILV-D-SVP--------TA
XP_022721034.1/1-226 ------------GILEIGSGGSLLTVAFQ------------ILV-D-SVP--------TA
XP_022721035.1/1-226 ------------GILEIGSGGSLLTVAFQ------------ILV-D-SVP--------TA
XP_022724746.1/1-226 ------------GILEIGSGGSLLTVAFQ------------ILV-D-SVP--------TA
XP_007026002.1/1-226 ------------GILEIGSGGSLLTVAFQ------------ILV-D-SVP--------TA
XP_021294392.1/1-226 ------------GILEIGSGGSLLTVAFQ------------ILV-D-SVP--------TA
XP_022777405.1/1-226 ------------GILEIGSGGSLLTVAFQ------------ILV-D-SVP--------TA
XP_017615807.1/1-226 ------------GILEIGSGGSLLTVAFQ------------ILV-D-SVP--------TA
XP_016753649.1/1-226 ------------GILEIGSGGSLLTVAFQ------------ILV-D-SVP--------TA
XP_016683607.1/1-226 ------------GILEIGSGGSLLTVAFQ------------ILV-D-SVP--------TA
XP_012451363.1/1-226 ------------GILEIGSGGSLLTVAFQ------------ILV-D-SVP--------TA
XP_002266688.1/1-226 ------------LDV--GSGGSLLTVAFQ------------ILV-D-SAP--------TA
XP_021853797.1/1-224 ------------LDA--GSGGSLLTVAFQ------------ILV-D-SVP--------TA
XP_021719656.1/1-227 ------------LEV--GSGGSLLTVAFQ------------ILV-D-SVP--------TA
XP_021766927.1/1-227 ------------LEV--GSGGSLLTVAFQ------------ILV-D-SVP--------TA
XP_010665524.1/1-227 ------------MEV--GSGGSLLTVAFQ------------ILV-D-SVP--------TA
XP_009605864.1/1-234 ------------PEVIGNSGGSLLTVAFQ------------ILV-D-SVP--------TA
XP_019223778.1/1-233 ------------PEVISNGGGSLLTVAFQ------------ILV-D-SVP--------TA
XP_016440385.1/1-233 ------------PEVIGNGGGSLLTVAFQ------------ILV-D-SVP--------TA
XP_009790281.1/1-233 ------------PEVIGNVGGSLLTVAFQ------------ILV-D-SVP--------TA
XP_016570434.1/1-222 -------------INTTCTSGSLLTVAFQ------------ILV-D-SIP--------TA
XP_016486479.1/1-229 ------------NVVEVGNGGSLLTVAFQ------------ILV-D-SVP--------TA
XP_009597904.1/1-229 ------------NVVEVGNGGSLLTVAFQ------------ILV-D-SVP--------TA
XP_009799795.1/1-229 ------------NVAEVGNGGSLLTVAFQ------------ILV-D-SVP--------TA
XP_019249652.1/1-229 ------------SVAEVGNGGSLLTVAFQ------------ILV-D-SVP--------TA
XP_006343296.1/1-221 -------------GIDVGTRGSLLTVAFQ------------ILV-D-SVP--------TA
XP_020217012.1/1-226 ------------PILDVGSGGSLLTVAFQ------------ILV-D-SAP--------TA
XP_017406677.1/1-226 ------------SILDVGSGGSLLTVAFQ------------ILV-D-SAP--------TA
XP_014519998.1/1-226 ------------SILEVGSGGSLLTVAFQ------------ILV-D-SAP--------TA
XP_014519997.1/1-226 ------------SILEVGSGGSLLTVAFQ------------ILV-D-SAP--------TA
XP_012841999.1/1-239 ------------GGPDVGNGGSLLTVAFQ------------ILV-D-SVP--------TA
XP_024984347.1/1-227 ------------TILEVGNGGSLLTVAFQ------------ILV-D-SAP--------TA
XP_023751202.1/1-227 ------------RIPEVGDGGSLLTVAFQ------------ILV-D-SVP--------TA
XP_023751208.1/1-227 ------------RIPEVGDGGSLLTVAFQ------------ILV-D-SVP--------TA
XP_022041933.1/1-227 ------------TILEVGNGGSLLTVAFQ------------ILV-D-SVP--------TA
XP_024988632.1/1-226 ------------GIAEVGTGGSLLTVAFQ------------ILV-D-STP--------TA
XP_022018253.1/1-226 ------------GIPEVGTGGSLLTVAFQ------------ILV-D-SVP--------TA
XP_023771293.1/1-226 ------------GIPELGTGGSLLTVAFQ------------ILV-D-SVP--------TA
XP_019170324.1/1-226 ------------LNPEIGSGGSLLTVAFQ------------ILV-D-SVP--------TA
XP_017230122.1/1-226 ------------GILEVGSGGSLLTVAFQ------------ILV-D-SVP--------TA
XP_011096101.1/1-226 ------------GAPEVGSGGSLLTVAFQ------------ILV-D-SVP--------TA
XP_012848972.1/1-227 ------------INPEVGSGGSLLTVAFQ------------ILV-D-SVP--------TA
XP_022876975.1/1-227 ------------INPEVGSGGSLLTVAFQ------------ILV-D-SVP--------TA
XP_022863158.1/1-227 ------------INAEVGSGGSLLTVAFQ------------ILV-D-SVP--------TA
XP_022877991.1/1-227 ------------INPEVGSGGSLLTVAFQ------------ILV-D-SVP--------TA
XP_019223745.1/1-223 ----------------VGCGGSLLTVAFQ------------ILV-D-SIP--------TA
XP_021800220.1/1-221 --------------GDSGSGGSLLTVAFQ------------ILV-D-SGL--------TA
XP_021684589.1/1-235 ----G-------IGELGSAGGSLLTVAFQ------------ILV-D-SVP--------TA
XP_021684519.1/1-235 ----G-------IGELGSAGGSLLTVAFQ------------ILV-D-SVP--------TA
XP_021684722.1/1-235 ----G-------IGELGSAGGSLLTVAFQ------------ILV-D-SVP--------TA
XP_021633498.1/1-230 ----G-------IGEFNCASGSLLTVAFQ------------ILV-D-SVP--------TA
XP_009420270.1/1-216 -----------------AAQGSLVTVAFE------------ILV-D-SVP--------TA
XP_003573403.1/1-232 --------------VAAGKGGSLLTVAFQ------------ILV-D-SVP--------TA
XP_006659894.1/1-229 ------------ENG-GSGGGALLTVAFQ------------ILV-D-SVP--------TA
XP_015650022.1/1-263 ------------ENGSGSGGGSLLTVAFQ------------ILV-D-SVP--------TA
XP_015650023.1/1-230 ------------ENGSGSGGGSLLTVAFQ------------ILV-D-SVP--------TA
XP_020407343.1/1-231 ------------------GGGSLLTVAFQ------------ILV-D-SVP--------TA
NP_001105127.2/1-231 ------------------GGGSLLTVAFQ------------ILV-D-SVP--------TA
XP_008661805.1/1-239 ----G-------LEELEAAGGSLLTVAFQ------------ILV-D-SVP--------TA
NP_001145750.1/1-239 ----G-------LEELEAAGGSLLTVAFQ------------ILV-D-SVP--------TA
XP_025821536.1/1-230 ----I-------SAGLEAGGGSLLTVAFQ------------ILV-D-SVP--------TA
XP_002443965.1/1-229 ----G--------AALETGGGSLLTVAFQ------------ILV-D-SVP--------TA
XP_004972697.1/1-229 ----S--------GSLEAGGGSLLTVAFQ------------ILV-D-SVP--------TA
XP_008667765.1/1-230 ----G-------V----GSGGSLLTVAFQ------------ILV-D-SVP--------TA
NP_001347891.1/1-231 ----G-------V----GSGGSLLTVAFQ------------ILV-D-SVP--------TA
XP_015636340.1/1-229 ----G-------V----GSGGSLLTVAFQ------------ILV-D-SVP--------TA
XP_015636338.1/1-229 ----G-------V----GSGGSLLTVAFQ------------ILV-D-SVP--------TA
XP_006652859.1/1-228 ------------V----GSGGSLLTVAFQ------------ILV-D-SVP--------TA
XP_003580598.1/1-233 ----G-------V----GSGGSLLTVAFQ------------ILV-D-SVP--------TA
XP_020199198.1/1-227 ----A-------T----GTGGSLLTVAFQ------------ILV-D-SVP--------TA
XP_020400772.1/1-230 ----G-------V----GSGGSLLTVAFQ------------ILV-D-SVP--------TA
NP_001334663.1/1-230 ----G-------V----GSGGSLLTVAFQ------------ILV-D-SVP--------TA
XP_008662197.1/1-270 ----G-------V----GSGGSLLTVAFQ------------ILV-D-SVP--------TA
XP_008662196.1/1-270 ----G-------V----GSGGSLLTVAFQ------------ILV-D-SVP--------TA
XP_025823505.1/1-227 ----G-------V----GSGGSLLTVAFQ------------ILV-D-SVP--------TA
XP_004976894.1/1-227 ----G-------V----GSGGSLLTVAFQ------------ILV-D-SVP--------TA
XP_002448563.2/1-227 ----G-------V----GSGGSLLTVAFQ------------ILV-D-SVP--------TA
XP_019414154.1/1-227 ------------GIGETGSGGSLLTVAFQ------------ILV-D-SVP--------TA
XP_006348085.1/1-234 ------------NSDIDSPGGSLLTVAFQ------------ILV-D-SVP--------TA
XP_004232734.1/1-234 ------------NSEIDSPGGSLLTVAFQ------------ILV-D-SVP--------TA
XP_015066898.1/1-234 ------------NSEIDSPGGSLLTVAFQ------------ILV-D-SVP--------TA
XP_003605900.1/1-232 ----G-------VGAGAGGGGSLLTVAFQ------------ILV-D-STP--------TA
XP_022988693.1/1-229 ----N-------NIMEHGSGGSLLTVAFQ------------ILV-D-SIP--------TA
XP_022930609.1/1-229 ----N-------NIMEHGSGGSLLTVAFQ------------ILV-D-SIP--------TA
XP_023530197.1/1-229 ----N-------SIMEHGSGGSLLTVAFQ------------ILV-D-SIP--------TA
XP_016188415.1/1-237 ------------GGGGGGGGGSLLTVAFQ------------ILV-D-SVP--------TA
XP_011628991.1/1-236 ----A-------AIGEVGFGGSLLTVAFQ------------ILV-D-SAP--------TA
XP_006826512.2/1-236 ----A-------AIGEVGFGGSLLTVAFQ------------ILV-D-SAP--------TA
XP_020177398.1/1-220 ---------------APDAGGSLLTVAFQ------------ILV-D-SVP--------TA
XP_015695486.1/1-219 ---------------DGAAGGSLLTVAFQ------------ILV-D-SVP--------TA
XP_015648539.1/1-217 ---------------GG--GGSLLTVAFQ------------ILV-D-SVP--------TA
XP_020401599.1/1-218 --------------GGGGGGGSLLTVAFQ------------ILV-D-SVP--------TA
XP_020401598.1/1-218 --------------GGGGGGGSLLTVAFQ------------ILV-D-SVP--------TA
XP_020401597.1/1-218 --------------GGGGGGGSLLTVAFQ------------ILV-D-SVP--------TA
XP_021319954.1/1-239 ----A-------GGGGGGGGGSLLTVAFQ------------ILV-D-SVP--------TA
XP_002445014.2/1-239 ----A-------GGGGGGGGGSLLTVAFQ------------ILV-D-SVP--------TA
XP_025819913.1/1-218 -----------------GGGGSLLTVAFQ------------ILV-D-SVP--------TA
XP_004972909.1/1-218 -----------------GGGGSLLTVAFQ------------ILV-D-SVP--------TA
XP_020100432.1/1-229 ------------ADGGSASGGSLLTVAFQ------------ILV-D-SVP--------TA
XP_009385059.1/1-228 ------------MVDGVGSGGSLLTVAFQ------------ILV-D-SAP--------TA
XP_018678797.1/1-226 ------------RVEEVGSGGSLLTVAFQ------------ILV-D-SVP--------TA
XP_009412012.1/1-233 ----G-------GEEGGGSGGSLLTVAFQ------------ILV-D-SVP--------TA
XP_018685149.1/1-236 ----G-------GEEGGGSGGSLLTVAFQ------------ILV-D-SVP--------TA
XP_020591278.1/1-230 --------------EVGSGGGSLLTVAFQ------------ILV-D-SIP--------TA
XP_020702932.1/1-229 --------------EVGSG-GSLLTVAFQ------------ILV-D-SIP--------TA
XP_020702933.1/1-230 --------------EVGSG-GSLLTVAFQ------------ILV-D-SIP--------TA
XP_020702931.1/1-230 --------------EVGSG-GSLLTVAFQ------------ILV-D-SIP--------TA
XP_020692781.1/1-229 --------------EVGSG-GSLLTVAFQ------------ILV-D-SVP--------TA
XP_020692780.1/1-229 --------------EVGSG-GSLLTVAFQ------------ILV-D-SVP--------TA
XP_020591591.1/1-229 --------------EVGSG-GSLLTVAFQ------------ILV-D-SVP--------TA
XP_020591590.1/1-229 --------------EVGSG-GSLLTVAFQ------------ILV-D-SVP--------TA
XP_020109359.1/1-227 --------------EVGSS-GSLLTVAFQ------------ILV-D-SVP--------TA
XP_020270114.1/1-229 --------------EVGSG-GSLLTVAFQ------------ILV-D-SIP--------TA
XP_010908855.1/1-227 --------------EVGSG-GSLLTVAFQ------------ILV-D-SVP--------TA
XP_008808987.1/1-227 --------------EVGSG-GSLLTVAFQ------------ILV-D-SVP--------TA
XP_008784552.1/1-223 --------------EVGSG-GSLLTVAFQ------------ILV-D-SVP--------TA
XP_010931820.1/1-223 --------------EVGSG-GSLLTVAFQ------------ILV-D-SVP--------TA
XP_003540308.2/1-226 -------------IGDIGHGGSLLTVAFQ------------ILV-D-SVP--------TA
XP_006592818.1/1-226 -------------IGDIGHGGSLLTVAFQ------------ILV-D-SVP--------TA
XP_006592816.1/1-226 -------------IGDIGHGGSLLTVAFQ------------ILV-D-SVP--------TA
XP_003543387.1/1-226 -------------IGDTGHGGSLLTVAFQ------------ILV-D-SVP--------TA
XP_020223651.1/1-226 -------------IGDTGHGGSLLTVAFQ------------ILV-D-SVP--------TA
XP_020223648.1/1-226 -------------IGDTGHGGSLLTVAFQ------------ILV-D-SVP--------TA
XP_007149968.1/1-226 -------------IGDTGNGGSLLTVAFQ------------ILV-D-SVP--------TA
XP_017425582.1/1-226 -------------IGDTGNGGSLLTVAFQ------------ILV-D-SVP--------TA
XP_014501634.1/1-226 -------------IGDTGHGGSLLTVAFQ------------ILV-D-SVP--------TA
XP_022776632.1/1-226 -------------IADAGSGGSLLTVAFQ------------ILV-D-SVP--------TA
XP_022776631.1/1-226 -------------IADAGSGGSLLTVAFQ------------ILV-D-SVP--------TA
XP_022776629.1/1-226 -------------IADAGSGGSLLTVAFQ------------ILV-D-SVP--------TA
XP_022733641.1/1-226 -------------IADAGSGGSLLTVAFQ------------ILV-D-SVP--------TA
XP_022733640.1/1-226 -------------IADAGSGGSLLTVAFQ------------ILV-D-SVP--------TA
XP_022733638.1/1-226 -------------IADAGSGGSLLTVAFQ------------ILV-D-SVP--------TA
XP_007045612.1/1-226 -------------IGDAGSAGSLLTVAFQ------------ILV-D-SVP--------TA
XP_007045611.1/1-226 -------------IGDAGSAGSLLTVAFQ------------ILV-D-SVP--------TA
XP_017971478.1/1-226 -------------IGDAGSAGSLLTVAFQ------------ILV-D-SVP--------TA
XP_021292769.1/1-226 -------------IGDAGSAGSLLTVAFQ------------ILV-D-SVP--------TA
XP_021292767.1/1-226 -------------IGDAGSAGSLLTVAFQ------------ILV-D-SVP--------TA
XP_021292765.1/1-226 -------------IGDAGSAGSLLTVAFQ------------ILV-D-SVP--------TA
XP_010657309.1/1-226 ----V--------IGEVGSGGSLLTVAFQ------------ILV-D-SVP--------TA
XP_010657313.1/1-226 ----V--------IGEVGSGGSLLTVAFQ------------ILV-D-SVP--------TA
XP_010657310.1/1-226 ----V--------IGEVGSGGSLLTVAFQ------------ILV-D-SVP--------TA
XP_010657311.1/1-226 ----V--------IGEVGSGGSLLTVAFQ------------ILV-D-SVP--------TA
XP_012086347.1/1-228 ----I--------GEPASPGGSLLTVAFQ------------ILV-D-SVP--------TA
XP_012086344.1/1-228 ----I--------GEPASPGGSLLTVAFQ------------ILV-D-SVP--------TA
XP_024029967.1/1-226 ----I--------GE-AGSGGSLLTVAFQ------------ILV-D-SVP--------TA
XP_010109517.1/1-226 ----I--------GE-AGSGGSLLTVAFQ------------ILV-D-SVP--------TA
XP_006469697.1/1-226 ----I--------GE-AASGGSLLTVAFQ------------ILV-D-SVP--------TA
XP_024046029.1/1-226 ----I--------GE-AASGGSLLTVAFQ------------ILV-D-SVP--------TA
XP_020234199.1/1-226 ----I--------GE-SGPGGSLLTVAFQ------------ILV-D-SVP--------TA
XP_020234201.1/1-226 ----I--------GE-SGPGGSLLTVAFQ------------ILV-D-SVP--------TA
XP_021630856.1/1-226 ----I--------GE-SGSGGSLLTVAFQ------------ILV-D-SVP--------TA
XP_021630853.1/1-226 ----I--------GE-SGSGGSLLTVAFQ------------ILV-D-SVP--------TA
XP_021907367.1/1-221 ----------------GGSGGSLLTVAFQ------------ILV-D-SVP--------TA
XP_010275996.1/1-221 ----------------GVHCGSLLTVAFQ------------ILV-D-SVP--------TA
XP_020870784.1/1-223 -------------A-PGGDGGSLLTVAFQ------------ILV-D-SVP--------TA
NP_172015.1/1-223    -------------A-PGGDGGSLLTVAFQ------------ILV-D-SVP--------TA
XP_006418050.1/1-223 -------------A-SGGDGGSLLTVAFQ------------ILV-D-SVP--------TA
XP_018447753.1/1-223 -------------A-AGGEGGSLLTVAFQ------------ILV-D-SVP--------TA
XP_010457605.1/1-218 ----------------GGDGGSLLTVAFQ------------ILV-D-SVP--------TA
XP_023632765.1/1-218 ----------------GGDGGSLLTVAFQ------------ILV-D-SVP--------TA
XP_010484829.1/1-218 ----------------GGDGGSLLTVAFQ------------ILV-D-SVP--------TA
XP_010475208.1/1-218 ----------------GGDGGSLLTVAFQ------------ILV-D-SVP--------TA
XP_018487097.1/1-220 ----------------GGEGGSLLTVAFQ------------ILV-D-SVP--------TA
XP_013664795.1/1-220 ----------------RGEGGSLLTVAFQ------------ILV-D-SVP--------TA
XP_013716253.1/1-220 ----------------GGEGGSLLTVAFQ------------ILV-D-SVP--------TA
XP_009118607.1/1-220 ----------------GGEGGSLLTVAFQ------------ILV-D-SVP--------TA
XP_013605632.1/1-220 ----------------GGEGGSLLTVAFQ------------ILV-D-SVP--------TA
XP_010475204.1/1-220 ----------------GGDGGSLLTVAFQ------------ILV-D-SVP--------TA
XP_010457604.1/1-220 ----------------GGDGGSLLTVAFQ------------ILV-D-SVP--------TA
XP_023632762.1/1-220 ----------------GGDGGSLLTVAFQ------------ILV-D-SVP--------TA
XP_010484823.1/1-220 ----------------GGDGGSLLTVAFQ------------ILV-D-SVP--------TA
XP_022558401.1/1-220 ----------------GGEGGSLLTVAFQ------------ILV-D-SVP--------TA
XP_013586641.1/1-220 ----------------GGEGGSLLTVAFQ------------ILV-D-SVP--------TA
XP_022558399.1/1-220 ----------------GGEGGSLLTVAFQ------------ILV-D-SVP--------TA
XP_022558400.1/1-220 ----------------GGEGGSLLTVAFQ------------ILV-D-SVP--------TA
XP_013586642.1/1-220 ----------------GGEGGSLLTVAFQ------------ILV-D-SVP--------TA
XP_013696832.1/1-220 ----------------GGEGGSLLTVAFQ------------ILV-D-SVP--------TA
XP_022548878.1/1-220 ----------------GGEGGSLLTVAFQ------------ILV-D-SVP--------TA
XP_013666552.1/1-220 ----------------GGEGGSLLTVAFQ------------ILV-D-SVP--------TA
XP_009119177.1/1-220 ----------------GGEGGSLLTVAFQ------------ILV-D-SVP--------TA
XP_020964430.1/1-225 ----G------------GHGGSLLTVAFQ------------ILV-D-SVP--------TA
XP_015936069.1/1-225 ----G------------GHGGSLLTVAFQ------------ILV-D-SVP--------TA
XP_020964429.1/1-225 ----G------------GHGGSLLTVAFQ------------ILV-D-SVP--------TA
XP_015936068.1/1-225 ----G------------GHGGSLLTVAFQ------------ILV-D-SVP--------TA
XP_015577880.1/1-228 ----G--------E-SVSAGGSLLTVAFQ------------ILV-D-SVP--------TA
XP_002524172.2/1-228 ----G--------E-SVSAGGSLLTVAFQ------------ILV-D-SVP--------TA
XP_007132557.1/1-221 ------------------PGGSLLTVAFQ------------ILV-D-SVP--------TA
XP_017433694.1/1-221 ------------------PGGSLLTVAFQ------------ILV-D-SVP--------TA
XP_014494419.1/1-221 ------------------AGGSLLTVAFQ------------ILV-D-SVP--------TA
XP_022634310.1/1-221 ------------------AGGSLLTVAFQ------------ILV-D-SVP--------TA
XP_010267735.1/1-225 -------------IGGVGSGGSLLTVAFQ------------ILV-D-SVP--------TA
XP_010267734.1/1-225 -------------IGGVGSGGSLLTVAFQ------------ILV-D-SVP--------TA
XP_024185199.1/1-224 ----N--------NGEGESGGSLLTVAFQ------------ILV-D-SVP--------TA
XP_024185197.1/1-224 ----N--------NGEGESGGSLLTVAFQ------------ILV-D-SVP--------TA
XP_009342246.1/1-222 ----D----------SGSGGGSLLTVAFQ------------ILV-D-SVP--------TA
XP_009342244.1/1-222 ----D----------SGSGGGSLLTVAFQ------------ILV-D-SVP--------TA
XP_009342240.1/1-222 ----D----------SGSGGGSLLTVAFQ------------ILV-D-SVP--------TA
XP_020416503.1/1-221 ----D----------SGSG-GSLLTVAFQ------------ILV-D-SVP--------TA
XP_021822854.1/1-221 ----D----------SGSG-GSLLTVAFQ------------ILV-D-SVP--------TA
XP_007217584.1/1-221 ----D----------SGSG-GSLLTVAFQ------------ILV-D-SVP--------TA
XP_021822852.1/1-221 ----D----------SGSG-GSLLTVAFQ------------ILV-D-SVP--------TA
XP_018730806.1/1-228 ----Q--------QGVGSGGGSLLTVAFQ------------ILV-D-SVP--------TA
XP_010051064.1/1-228 ----Q--------QGVGSGGGSLLTVAFQ------------ILV-D-SVP--------TA
XP_015881130.2/1-229 ----G--------IGETGSGGSLLTVAFQ------------ILV-D-SVP--------TA
XP_023534533.1/1-219 -------------A-ETATGGSLLTVAFQ------------ILV-D-SVP--------TA
XP_022947095.1/1-219 -------------A-ETATGGSLLTVAFQ------------ILV-D-SVP--------TA
XP_022947096.1/1-219 -------------A-ETATGGSLLTVAFQ------------ILV-D-SVP--------TA
XP_022970950.1/1-219 -------------A-ETAAGGSLLTVAFQ------------ILV-D-SVP--------TA
XP_022970951.1/1-219 -------------A-ETAAGGSLLTVAFQ------------ILV-D-SVP--------TA
XP_022140692.1/1-217 -------------GGE---GGSLLTVAFQ------------ILV-D-SVP--------TA
XP_022140691.1/1-217 -------------GGE---GGSLLTVAFQ------------ILV-D-SVP--------TA
XP_008455853.1/1-220 -------------GGEGVSGGSLLTVAFQ------------ILV-D-SVP--------TA
XP_011650012.1/1-220 -------------GGEGVSGGSLLTVAFQ------------ILV-D-SVP--------TA
XP_008455850.1/1-220 -------------GGEGVSGGSLLTVAFQ------------ILV-D-SVP--------TA
XP_011650011.1/1-220 -------------GGEGVSGGSLLTVAFQ------------ILV-D-SVP--------TA
XP_023513403.1/1-220 -------------GGEGVSGGSLLTVAFQ------------ILV-D-SVP--------TA
XP_022944761.1/1-220 -------------GGEGVSGGSLLTVAFQ------------ILV-D-SVP--------TA
XP_023513401.1/1-220 -------------GGEGVSGGSLLTVAFQ------------ILV-D-SVP--------TA
XP_022944759.1/1-220 -------------GGEGVSGGSLLTVAFQ------------ILV-D-SVP--------TA
XP_022986270.1/1-220 -------------GGEGVSGGSLLTVAFQ------------ILV-D-SVP--------TA
XP_022986268.1/1-220 -------------GGEGVSGGSLLTVAFQ------------ILV-D-SVP--------TA
XP_022136006.1/1-224 -------------EPGSGGSGSLLTVAFQ------------ILV-D-SVP--------TA
XP_008455423.1/1-229 ----G--------AGEHGSGGSLLTVAFQ------------ILV-D-SVP--------TA
XP_011658704.1/1-229 ----G--------VGEHGSGGSLLTVAFQ------------ILV-D-SVP--------TA
XP_004144488.1/1-229 ----G--------VGEHGSGGSLLTVAFQ------------ILV-D-SVP--------TA
XP_022968990.1/1-229 ----G--------VAEHGSGGSLLTVAFQ------------ILV-D-SVP--------TA
XP_023554403.1/1-229 ----G--------VAEHGSGGSLLTVAFQ------------ILV-D-SVP--------TA
XP_022952948.1/1-229 ----G--------VAEHGSGGSLLTVAFQ------------ILV-D-SVP--------TA
XP_019447751.1/1-228 ----------------NDHGGSLLTVAFQ------------ILV-D-SVP--------TA
XP_019447749.1/1-228 ----------------NDHGGSLLTVAFQ------------ILV-D-SVP--------TA
XP_004515074.1/1-228 ----N--------GQAGVCGGSLLTVAFQ------------ILV-D-SVP--------TA
XP_012463792.1/1-235 ----T--------DAAGSSSGSLLTVAFQ------------ILV-D-SVP--------TA
XP_012463790.1/1-235 ----T--------DAAGSSSGSLLTVAFQ------------ILV-D-SVP--------TA
XP_016678783.1/1-235 ----T--------DAAGSSGGSLLTVAFQ------------ILV-D-SVP--------TA
XP_016678782.1/1-235 ----T--------DAAGSSGGSLLTVAFQ------------ILV-D-SVP--------TA
XP_017631648.1/1-235 ----T--------DAAGSSGGSLLTVAFQ------------ILV-D-SVP--------TA
XP_016732503.1/1-235 ----T--------DAAGSSGGSLLTVAFQ------------ILV-D-SVP--------TA
XP_017631647.1/1-235 ----T--------DAAGSSGGSLLTVAFQ------------ILV-D-SVP--------TA
XP_012438430.1/1-229 ----A--------DAGGSSGGSLLTVAFQ------------ILV-D-SVP--------TA
XP_012438427.1/1-229 ----A--------DAGGSSGGSLLTVAFQ------------ILV-D-SVP--------TA
XP_012438429.1/1-229 ----A--------DAGGSSGGSLLTVAFQ------------ILV-D-SVP--------TA
XP_017616162.1/1-229 ----A--------DAGGSSGGSLLTVAFQ------------ILV-D-SVP--------TA
XP_017616160.1/1-229 ----A--------DAGGSSGGSLLTVAFQ------------ILV-D-SVP--------TA
XP_017616161.1/1-229 ----A--------DAGGSSGGSLLTVAFQ------------ILV-D-SVP--------TA
XP_016726347.1/1-229 ----A--------DAGGSSGGSLLTVAFQ------------ILV-D-SVP--------TA
XP_016726344.1/1-229 ----A--------DAGGSSGGSLLTVAFQ------------ILV-D-SVP--------TA
XP_016726345.1/1-229 ----A--------DAGGSSGGSLLTVAFQ------------ILV-D-SVP--------TA
XP_019448933.1/1-227 ----I--------GET-VPSGSLLTVAFQ------------ILV-D-SVP--------TA
XP_004487400.1/1-228 --------------ESGHGGGSLLTVAFQ------------ILV-D-SVP--------TA
XP_003596999.2/1-227 --------------ETGHGGGSLLTVAFQ------------ILV-D-SVP--------TA
XP_024631890.1/1-227 --------------ETGHGGGSLLTVAFQ------------ILV-D-SVP--------TA
XP_024631891.1/1-227 --------------ETGHGGGSLLTVAFQ------------ILV-D-SVP--------TA
XP_004287935.1/1-224 ----N--------SGEAESGGSLLTVAFQ------------ILV-D-SVP--------TA
XP_018808969.1/1-228 ----C--------IGEAESGGSLLTVAFQ------------ILV-D-SVP--------TA
XP_018808967.1/1-228 ----C--------IGEAESGGSLLTVAFQ------------ILV-D-SVP--------TA
XP_018817598.1/1-227 ----A--------IGEADSGGSLLTVAFQ------------ILV-D-SVP--------TA
XP_018817597.1/1-227 ----A--------IGEADSGGSLLTVAFQ------------ILV-D-SVP--------TA
XP_018817594.1/1-227 ----A--------IGEADSGGSLLTVAFQ------------ILV-D-SVP--------TA
XP_018817599.1/1-227 ----A--------IGEADSGGSLLTVAFQ------------ILV-D-SVP--------TA
XP_023915056.1/1-227 ----I--------GEATTSGGSLLTVAFQ------------ILV-D-SVP--------TA
XP_023915054.1/1-227 ----I--------GEATTSGGSLLTVAFQ------------ILV-D-SVP--------TA
XP_023915053.1/1-227 ----I--------GEATTSGGSLLTVAFQ------------ILV-D-SVP--------TA
XP_021685023.1/1-228 ----I--------GELGSAGGSLLTVAFQ------------ILV-D-SVP--------TA
XP_021684657.1/1-228 ----I--------GELGSAGGSLLTVAFQ------------ILV-D-SVP--------TA
XP_021685089.1/1-228 ----I--------GELGSAGGSLLTVAFQ------------ILV-D-SVP--------TA
XP_015383009.1/1-229 ----I--------GE-AASGGSLLTVAFQ------------ILV-D-SVP--------TA
XP_015383007.1/1-229 ----I--------GE-AASGGSLLTVAFQ------------ILV-D-SVP--------TA
XP_020539441.1/1-235 ----I--------GEPASPGGSLLTVAFQ------------ILV-D-SVP--------TA
XP_020539439.1/1-235 ----I--------GEPASPGGSLLTVAFQ------------ILV-D-SVP--------TA
XP_015577881.1/1-233 ----G--------GESVSAGGSLLTVAFQ------------ILV-D-SVP--------TA
XP_015577879.1/1-233 ----G--------GESVSAGGSLLTVAFQ------------ILV-D-SVP--------TA
XP_015577877.1/1-233 ----G--------GESVSAGGSLLTVAFQ------------ILV-D-SVP--------TA
XP_021630854.1/1-231 -------------GESG-SGGSLLTVAFQ------------ILV-D-SVP--------TA
XP_021630849.1/1-231 -------------GESG-SGGSLLTVAFQ------------ILV-D-SVP--------TA
XP_021652390.1/1-229 -------------GESVSAGGSLLTVAFQ------------ILV-D-SVP--------TA
XP_021652388.1/1-229 -------------GESVSAGGSLLTVAFQ------------ILV-D-SVP--------TA
XP_021652389.1/1-234 -------------GESVSAGGSLLTVAFQ------------ILV-D-SVP--------TA
XP_021652386.1/1-234 -------------GESVSAGGSLLTVAFQ------------ILV-D-SVP--------TA
XP_006592380.1/1-228 ----I--------GETGP-SGSLLTVAFQ------------ILV-D-SVP--------TA
XP_003539872.2/1-228 ----I--------GETGP-SGSLLTVAFQ------------ILV-D-SVP--------TA
XP_025984806.1/1-230 ----I--------GETSPSSGSLLTVAFQ------------ILV-D-SVP--------TA
XP_014632311.1/1-230 ----I--------GETSPSSGSLLTVAFQ------------ILV-D-SVP--------TA
XP_024450362.1/1-227 ----M--------DESGSTGGSLLTVAFQ------------ILV-D-SVP--------TA
XP_002302880.2/1-227 ----M--------DESGSTGGSLLTVAFQ------------ILV-D-SVP--------TA
XP_024450361.1/1-227 ----M--------DESGSTGGSLLTVAFQ------------ILV-D-SVP--------TA
XP_024450360.1/1-227 ----M--------DESGSTGGSLLTVAFQ------------ILV-D-SVP--------TA
XP_011011493.1/1-227 ----M--------EESGSTGGSLLTVAFQ------------ILV-D-SVP--------TA
XP_011011491.1/1-227 ----M--------EESGSTGGSLLTVAFQ------------ILV-D-SVP--------TA
XP_011011492.1/1-227 ----M--------EESGSTGGSLLTVAFQ------------ILV-D-SVP--------TA
XP_011011489.1/1-227 ----M--------EESGSTGGSLLTVAFQ------------ILV-D-SVP--------TA
XP_011011488.1/1-227 ----M--------EESGSTGGSLLTVAFQ------------ILV-D-SVP--------TA
XP_011005116.1/1-225 ----M--------EEA--SGGSLLTVAFQ------------ILV-D-SVP--------TA
XP_011005113.1/1-225 ----M--------EEA--SGGSLLTVAFQ------------ILV-D-SVP--------TA
XP_011005112.1/1-225 ----M--------EEA--SGGSLLTVAFQ------------ILV-D-SVP--------TA
XP_024440391.1/1-225 ----M--------EEA--AGGSLLTVAFQ------------ILV-D-SVP--------TA
XP_002320458.2/1-225 ----M--------EEA--AGGSLLTVAFQ------------ILV-D-SVP--------TA
XP_024440389.1/1-225 ----M--------EEA--AGGSLLTVAFQ------------ILV-D-SVP--------TA
XP_019453767.1/1-227 ----G--------IGETGPGGSLLTVAFQ------------ILV-D-SAP--------TA
XP_012478857.1/1-227 --------------DSGGSSGSLLTVAFQ------------ILV-D-SVP--------TA
XP_012478855.1/1-227 --------------DSGGSSGSLLTVAFQ------------ILV-D-SVP--------TA
XP_012478856.1/1-227 --------------DSGGSSGSLLTVAFQ------------ILV-D-SVP--------TA
XP_012478851.1/1-227 --------------DSGGSSGSLLTVAFQ------------ILV-D-SVP--------TA
XP_016724999.1/1-227 --------------DSGGSSGSLLTVAFQ------------ILV-D-SVP--------TA
XP_017638204.1/1-227 --------------DSGGSSGSLLTVAFQ------------ILV-D-SVP--------TA
XP_017638203.1/1-227 --------------DSGGSSGSLLTVAFQ------------ILV-D-SVP--------TA
XP_016667113.1/1-227 --------------DSGGSSGSLLTVAFQ------------ILV-D-SVP--------TA
XP_016667112.1/1-227 --------------DSGGSSGSLLTVAFQ------------ILV-D-SVP--------TA
XP_015881131.2/1-227 ----G--------IGETGSGGSLLTVAFQ------------ILV-D-SVP--------TA
XP_008348131.1/1-222 ---------------SGSGGGSLLTVAFQ------------ILV-D-SVP--------TA
XP_008348133.1/1-220 ---------------SGSGGGSLLTVAFQ------------ILV-D-SVP--------TA
XP_018502305.1/1-220 ---------------SGSGGGSLLTVAFQ------------ILV-D-SVP--------TA
XP_009342243.1/1-220 ---------------SGSGGGSLLTVAFQ------------ILV-D-SVP--------TA
XP_017179321.1/1-222 ---------------SGSGGGSLLTVAFQ------------ILV-D-SIP--------TA
XP_017179320.1/1-222 ---------------SGSGGGSLLTVAFQ------------ILV-D-SIP--------TA
XP_008341986.1/1-222 ---------------SGSGGGSLLTVAFQ------------ILV-D-SIP--------TA
XP_017179319.1/1-220 ---------------SGSGGGSLLTVAFQ------------ILV-D-SIP--------TA
XP_024185198.1/1-222 -------------NGEGESGGSLLTVAFQ------------ILV-D-SVP--------TA
XP_011463209.1/1-222 -------------SGEAESGGSLLTVAFQ------------ILV-D-SVP--------TA
XP_020416501.1/1-219 -------------MGDSGSGGSLLTVAFQ------------ILV-D-SVP--------TA
XP_021822853.1/1-219 -------------MGDSGSGGSLLTVAFQ------------ILV-D-SVP--------TA
XP_008230079.1/1-219 -------------MGDSGSGGSLLTVAFQ------------ILV-D-SVP--------TA
XP_020870786.1/1-221 ---------------PGGDGGSLLTVAFQ------------ILV-D-SVP--------TA
NP_001117231.1/1-221 ---------------PGGDGGSLLTVAFQ------------ILV-D-SVP--------TA
XP_018817596.1/1-225 ----A--------IGEADSGGSLLTVAFQ------------ILV-D-SVP--------TA
XP_019079350.1/1-224 ----V--------IGEVGSGGSLLTVAFQ------------ILV-D-SVP--------TA
XP_022733639.1/1-224 ----G--------IADAGSGGSLLTVAFQ------------ILV-D-SVP--------TA
XP_021292768.1/1-224 ----G--------IGDAGSAGSLLTVAFQ------------ILV-D-SVP--------TA
XP_021292766.1/1-224 ----G--------IGDAGSAGSLLTVAFQ------------ILV-D-SVP--------TA
XP_017971480.1/1-224 ----G--------IGDAGSAGSLLTVAFQ------------ILV-D-SVP--------TA
XP_018721484.1/1-222 --------------GEVASGGSLLTVAFQ------------ILV-D-SVP--------TA
XP_018721483.1/1-222 --------------GEVASGGSLLTVAFQ------------ILV-D-SVP--------TA
XP_018721482.1/1-222 --------------GEVASGGSLLTVAFQ------------ILV-D-SVP--------TA
XP_025687365.1/1-237 ----G--------GGGGGGGGSLLTVAFQ------------ILV-D-SVP--------TA
XP_006851770.2/1-243 -------VG-WGQGEKLRTEGSLLTVAFQ------------ILV-S-SLP--------TA
XP_003623826.1/1-230 -------GN-HEDASQPRVSGSLLTVAFQ------------ILV-N-SLP--------TA
XP_004492678.1/1-230 -------NN-HGGASQQRASGSLLTVAFQ------------ILV-N-SLP--------TA
XP_004492677.1/1-230 -------NN-HGGASQQRASGSLLTVAFQ------------ILV-N-SLP--------TA
XP_012847897.1/1-236 -----------NGGSGHGGGGSLLTVGFQ------------ILV-N-SLP--------TA
XP_012843673.1/1-229 -----------NG-------GSLLTVGFQ------------ILV-N-SLP--------TA
XP_021279608.1/1-239 -NG-------GGGWRVAESGWVPSTVAFQ------------ILV-N-SLP--------TA
XP_025641241.1/1-241 -GGDD--L-----GGGARGSGSLLTVAFQ------------ILV-N-SLP--------TA
XP_025641246.1/1-241 -GGDD--L-----GGGARGSGSLLTVAFQ------------ILV-N-SLP--------TA
XP_020991942.1/1-241 -GGDD--L-----GGGARGSGSLLTVAFQ------------ILV-N-SLP--------TA
XP_025632115.1/1-241 -GGDD--L-----GGGARGSGSLLTVAFQ------------ILV-N-SLP--------TA
XP_025632116.1/1-241 -GGDD--L-----GGGARGSGSLLTVAFQ------------ILV-N-SLP--------TA
XP_017442222.1/1-246 --TAA--TN-GGDNGVARVSGSLLTVAFQ------------ILV-N-SLP--------TA
XP_017442225.1/1-246 --TAA--TN-GGDNGVARVSGSLLTVAFQ------------ILV-N-SLP--------TA
XP_006576359.1/1-244 -TTTT--TN-GGDNGVTRVSGSLLTVAFQ------------ILV-N-SLP--------TA
XP_003521284.1/1-244 -TTTT--TN-GGDNGVTRVSGSLLTVAFQ------------ILV-N-SLP--------TA
XP_024174849.1/1-237 -SSNG--------GGEARVSGSLLTMTFQ------------ILV-N-SLP--------SA
XP_024174854.1/1-237 -SSNG--------GGEARVSGSLLTMTFQ------------ILV-N-SLP--------SA
XP_004306832.1/1-241 -SSNG-------NGGEARVSGSLLTMTFQ------------ILV-N-SLP--------SA
XP_011469062.1/1-241 -SSNG-------NGGEARVSGSLLTMTFQ------------ILV-N-SLP--------SA
XP_009340632.2/1-243 -SGNS--GG-GVDDGVHRVSGSLLTMTFQ------------ILV-N-SLP--------TA
XP_008385703.1/1-243 -SGNS--GG-GVDDGGHRVSGSLLTMTFQ------------ILV-N-SLP--------TA
XP_008385704.1/1-243 -SGNS--GG-GVDDGGHRVSGSLLTMTFQ------------ILV-N-SLP--------TA
XP_008232741.1/1-243 -SNNG--GG-G-EDATHRVSGSLLTMTFQ------------ILV-N-SLP--------SA
XP_008232742.1/1-243 -SNNG--GG-G-EDATHRVSGSLLTMTFQ------------ILV-N-SLP--------SA
XP_007220256.1/1-243 -SSNG--GG-G-EDATHRVSGSLLTMTFQ------------ILV-N-SLP--------SA
XP_020413144.1/1-243 -SSNG--GG-G-EDATHRVSGSLLTMTFQ------------ILV-N-SLP--------SA
XP_021817103.1/1-243 -SSNG--GG-G-EDATHRVSGSLLTMTFQ------------ILV-N-SLP--------SA
XP_021817105.1/1-243 -SSNG--GG-G-EDATHRVSGSLLTMTFQ------------ILV-N-SLP--------SA
XP_022872559.1/1-230 -------------DPNQRVSGSLLTVAFQ------------ILV-N-SLP--------TA
XP_016575742.1/1-238 -P-----PS-CNGGPDQRISGSLLTVAFQ------------ILV-N-SLP--------TA
XP_017608938.1/1-232 ------------NGDSHRVGGSLLTVAFQ------------ILV-N-SLP--------TA
XP_017608947.1/1-232 ------------NGDSHRVGGSLLTVAFQ------------ILV-N-SLP--------TA
XP_010255501.1/1-245 -L-----AN-GNGSSQQRVGGSLLTVAFQ------------ILV-N-NLP--------TA
XP_011023407.1/1-238 -N-----AN-NV-GGQERVSGSLLTVAFQ------------ILV-N-SLP--------TA
XP_011023404.1/1-238 -N-----AN-NV-GGQERVSGSLLTVAFQ------------ILV-N-SLP--------TA
XP_011023405.1/1-238 -N-----AN-NV-GGQERVSGSLLTVAFQ------------ILV-N-SLP--------TA
XP_019152252.1/1-243 -C-----SN-GD-PYQQRVSGSLLTVAFQ------------ILV-N-SLP--------TA
XP_022870029.1/1-235 -------------VSGQRASGSLLTVAFQ------------ILV-N-SLP--------AA
XP_017699285.1/1-225 --------------GAHKVGGSLLTVAFQ------------ILV-N-SQP--------TA
XP_008795402.1/1-225 --------------GAHKVGGSLLTVAFQ------------ILV-N-SQP--------TA
XP_010933869.1/1-224 --------------GPQKVGGSLLTVAFQ------------ILV-N-SQP--------TA
XP_008799528.1/1-222 --------------GAQKVVGSLLTVAFQ------------ILV-N-SQP--------TA
XP_010919252.1/1-223 --------------GVQKVGGSLLTVAFQ------------ILV-N-SQP--------TA
XP_010919250.1/1-223 --------------GVQKVGGSLLTVAFQ------------ILV-N-SQP--------TA
XP_007139955.1/1-229 --------G-GASQK--RASGCLLTVAFQ------------ILV-N-SLP--------TA
XP_017418814.1/1-230 --------G-GASQK--RTGGCLLTVAFQ------------ILV-N-SLP--------TA
XP_014490274.1/1-230 --------G-GASQK--RASGCLLTVAFQ------------ILV-N-SLP--------TA
XP_003534596.1/1-229 --------G-GASQQRAASGGCLLTVAFQ------------ILV-N-SLP--------TA
XP_006587871.1/1-229 --------G-GASQQRAASGGCLLTVAFQ------------ILV-N-SLP--------TA
XP_003552359.1/1-228 --------G-GASQQRAA-SGCLLTVAFQ------------ILV-N-SLP--------TA
XP_015961616.1/1-228 --------T-V----SQSGGGSLLTVAFQ------------ILV-N-SLP--------TA
XP_015961617.1/1-228 --------T-V----SQSGGGSLLTVAFQ------------ILV-N-SLP--------TA
XP_025696060.1/1-228 --------T-V----PQSGGGSLLTVAFQ------------ILV-N-SLP--------TA
XP_025696061.1/1-228 --------T-V----PQSGGGSLLTVAFQ------------ILV-N-SLP--------TA
XP_016198931.1/1-228 --------T-V----SQPGGGSLLTVAFQ------------ILV-N-SLP--------TA
XP_016198932.1/1-228 --------T-V----SQPGGGSLLTVAFQ------------ILV-N-SLP--------TA
XP_025649261.1/1-228 --------T-V----SQPGGGSLLTVAFQ------------ILV-N-SLP--------TA
XP_025649262.1/1-228 --------T-V----SQPGGGSLLTVAFQ------------ILV-N-SLP--------TA
XP_021849370.1/1-228 --------G-S----PQRGGGSLLTVAFQ------------ILV-N-SLP--------TA
XP_021767325.1/1-228 --------G-G----PQRVGGSLLTVAFQ------------ILV-N-SLP--------TA
XP_021748881.1/1-228 --------G-G----PQRVGGSLLTVAFQ------------ILV-N-SLP--------TA
XP_019461451.1/1-231 --------D-SNNGGENRVSGSLLTVAFQ------------ILV-N-SLP--------TA
XP_012574211.1/1-233 ----------TTNGGETRVSGSLLTVAFQ------------ILV-N-SLP--------TA
XP_004510857.1/1-233 ----------TTNGGETRVSGSLLTVAFQ------------ILV-N-SLP--------TA
XP_020237185.1/1-234 ----T---N-GADNGGARVSGSLLTVAFQ------------ILV-N-SLP--------TA
XP_020237261.1/1-234 ----T---N-GADNGGARVSGSLLTVAFQ------------ILV-N-SLP--------TA
XP_019456357.1/1-238 ----N---S-GNNGDEARVSGSLLTVAFQ------------ILV-N-SLP--------TA
XP_013444922.1/1-235 ----N---G-GG---EARVSGSLLTVAFQ------------ILV-N-SLP--------TA
XP_003528880.1/1-238 ----T---N-GGDNGVTRVSGSLLTVAFQ------------ILV-N-SLP--------TA
XP_006583340.1/1-238 ----T---N-GGDNGVTRVSGSLLTVAFQ------------ILV-N-SLP--------TA
XP_014516480.1/1-238 ----T---N-GGDNGAARVSGSLLTVAFQ------------ILV-N-SLP--------TA
XP_014516483.1/1-238 ----T---N-GGDNGAARVSGSLLTVAFQ------------ILV-N-SLP--------TA
XP_007134961.1/1-238 ----A---N-GGDNGGARVSGSLLTVAFQ------------ILV-N-SLP--------TA
XP_010671720.1/1-229 ----------NGNAGPQRVGGSLLSVAFQ------------ILV-N-SLP--------TA
XP_012482732.1/1-232 ----------NGNGDSHRVGGSLLTVAFQ------------ILV-N-SLP--------TA
XP_012482742.1/1-232 ----------NGNGDSHRVGGSLLTVAFQ------------ILV-N-SLP--------TA
XP_016745638.1/1-232 ----------NGNGDSHMVGGSLLTVAFQ------------ILV-N-SLP--------TA
XP_016745639.1/1-232 ----------NGNGDSHMVGGSLLTVAFQ------------ILV-N-SLP--------TA
XP_010278578.1/1-242 ----H---T-NGNGSSQRVGGSLLTVAFQ------------ILV-N-NLP--------TA
XP_010278577.1/1-242 ----H---T-NGNGSSQRVGGSLLTVAFQ------------ILV-N-NLP--------TA
XP_006445143.1/1-238 ----G---N-GSNGGSQRVGGSLLTVAFQ------------ILV-N-SLP--------TA
XP_006445141.1/1-238 ----G---N-GSNGGSQRVGGSLLTVAFQ------------ILV-N-SLP--------TA
XP_022770361.1/1-238 ----V---N-GGAGESHRVGGSLLTVAFQ------------ILV-N-SLP--------TA
XP_022757219.1/1-237 ----G---N-AGG-GSQRVGGSLLTVAFQ------------ILV-N-SLP--------TA
XP_022757220.1/1-237 ----G---N-AGG-GSQRVGGSLLTVAFQ------------ILV-N-SLP--------TA
NP_001314420.1/1-237 ----G---N-TGG-GSSSVGGSPLTVAFQ------------ILV-N-SSP--------TA
XP_016735217.1/1-237 ----G---N-TGG-GSSSVGGSLLTVAFQ------------ILV-N-SLP--------TA
XP_012437660.1/1-237 ----G---N-TGG-GSSRVGGSLLTVAFQ------------ILV-N-SLP--------TA
XP_012437661.1/1-237 ----G---N-TGG-GSSRVGGSLLTVAFQ------------ILV-N-SLP--------TA
XP_017615505.1/1-237 ----G---N-TGG-GSSRVGGSLLTVAFQ------------ILV-N-SLP--------TA
XP_017615506.1/1-237 ----G---N-TGG-GSSRVGGSLLTVAFQ------------ILV-N-SLP--------TA
XP_016723220.1/1-237 ----G---N-TGG-GSSRVGGSLLTVAFQ------------ILV-N-SLP--------TA
XP_016723229.1/1-237 ----G---N-TGG-GSSRVGGSLLTVAFQ------------ILV-N-SLP--------TA
XP_021904309.1/1-244 -GAGS---D-GGVGGPQRVGGSLLTVAFQ------------ILV-N-SLP--------TA
XP_021904311.1/1-244 -GAGS---D-GGVGGPQRVGGSLLTVAFQ------------ILV-N-SLP--------TA
XP_007051912.2/1-241 -GN-G---G-GGGGGSQRVGGSLLTVAFQ------------ILV-N-SLP--------TA
XP_007051913.2/1-241 -GN-G---G-GGGGGSQRVGGSLLTVAFQ------------ILV-N-SLP--------TA
XP_012489878.1/1-239 -RN-G---G-G-GGGAQRVGGSLLTVAFQ------------ILV-N-SLP--------TA
XP_016748231.1/1-239 -GN-G---S-G-GGGAERVGGSLLTVAFQ------------ILV-N-SLP--------TA
XP_017631289.1/1-239 -GN-G---S-G-GGGAERVGGSLLTVAFQ------------ILV-N-SLP--------TA
XP_023006686.1/1-236 -SGGE---G-GRQGERGTGGGSLLTVAFQ------------ILV-N-SLP--------TA
XP_022958954.1/1-236 -SGGE---G-GRQGERGTGGGSLLTVAFQ------------ILV-N-SLP--------TA
XP_023548045.1/1-236 -SGGE---G-GRQGERGTGGGSLLTVAFQ------------ILV-N-SLP--------TA
XP_008460172.1/1-241 -SGGE---G-PQSQRAAG-GGSLLTVAFQ------------ILV-N-SLP--------TA
XP_004144982.1/1-241 -SGGE---G-PQSQRAAG-GGSLLTVAFQ------------ILV-N-SLP--------TA
XP_023514722.1/1-242 -SGGD---G-PQSQRVTGGGGSLLTVAFQ------------ILV-N-SLP--------TA
XP_022999814.1/1-242 -SGGD---G-PQSQRVTGGGGSLLTVAFQ------------ILV-N-SLP--------TA
XP_022964504.1/1-242 -SGGD---G-PQSQRVTGGGGSLLTVAFQ------------ILV-N-SLP--------TA
XP_010053664.1/1-245 -PNGP---A-ASNGGPPRVGGSLLTVAFQ------------ILV-N-SLP--------TA
XP_019189515.1/1-235 -------------EHRGGGGGSLLTVAFQ------------ILV-N-SLP--------TA
XP_023730775.1/1-234 -----------SGGSGGGAGGSLLTVAFQ------------ILV-N-SLP--------TA
XP_023730774.1/1-234 -----------SGGSGGGAGGSLLTVAFQ------------ILV-N-SLP--------TA
XP_022020543.1/1-230 ----------------GRAGGSLLTVAFQ------------ILV-N-SLP--------TA
XP_024962572.1/1-236 ----------VGGGSGGGAGGSLLTVAFQ------------ILV-N-SLP--------TA
XP_022017696.1/1-236 ----------VGGGSGGGAGGSLLTVAFQ------------ILV-N-SLP--------TA
XP_024018220.1/1-245 -NVNN---V-NGGDGPQRVGGSLLTVAFQ------------ILV-N-SLP--------TA
XP_011093166.1/1-234 -G--P---T-SNGGPGHRVSGSLLTVAFQ------------ILV-N-SLP--------TA
XP_011093167.1/1-234 -G--P---T-SNGGPGHRVSGSLLTVAFQ------------ILV-N-SLP--------TA
XP_006339457.1/1-238 -G--P---S-CNGGPDQRISGSLLTVAFQ------------ILV-N-SLP--------TA
XP_015059298.1/1-238 -G--P---S-CNGGPDQRISGSLLTVAFQ------------ILV-N-SLP--------TA
NP_001234657.2/1-238 -G--P---S-CNGGPDQRISGSLLTVAFQ------------ILV-N-SLP--------TA
XP_009602857.1/1-241 -----------NDGPDQRISGSLLTVAFQ------------ILV-N-SLP--------TA
XP_009602856.1/1-241 -----------NDGPDQRISGSLLTVAFQ------------ILV-N-SLP--------TA
XP_009782444.1/1-245 -G--P---S-CNGGPDQRISGSLLTVAFQ------------ILV-N-SLP--------TA
XP_009782443.1/1-245 -G--P---S-CNGGPDQRISGSLLTVAFQ------------ILV-N-SLP--------TA
XP_019259406.1/1-245 -G--P---S-CNGGPDQRISGSLLTVAFQ------------ILV-N-SLP--------TA
XP_015867375.1/1-242 -A--S---S-NGGDGPHRVSGSLLTVAFQ------------ILV-N-SLP--------TA
XP_015867374.1/1-242 -A--S---S-NGGDGPHRVSGSLLTVAFQ------------ILV-N-SLP--------TA
XP_015888672.1/1-242 -A--S---S-NGGDGPHRVSGSLLTVAFQ------------ILV-N-SLP--------TA
XP_018810322.1/1-233 --------A-SNGDGPLRVSGSLLTVAFQ------------ILV-N-SLP--------TA
XP_018859074.1/1-233 --------A-SNGDGPLRVSGSLLTVAFQ------------ILV-N-SLP--------TA
XP_017231243.1/1-235 ------------GGSGHRAGGSLLTVAFQ------------ILV-N-SLP--------TA
XP_017231244.1/1-235 ------------GGSGHRAGGSLLTVAFQ------------ILV-N-SLP--------TA
XP_017231245.1/1-235 ------------GGSGHRAGGSLLTVAFQ------------ILV-N-SLP--------TA
XP_017236322.1/1-235 ------------GGSGHRTGGSLLTVAFQ------------ILV-N-SLP--------TA
XP_002301331.2/1-238 ----N-------VGGQERVSGSLLTVAFQ------------ILV-N-SLP--------TA
XP_024451706.1/1-238 ----N-------VGGQERVSGSLLTVAFQ------------ILV-N-SLP--------TA
XP_002320755.1/1-241 ----N---N-SNGGGPERVSGSLLTVAFQ------------ILV-N-SLP--------TA
XP_011035097.1/1-241 ----N---N-SNGCGPDRVSGSLLTVAFQ------------ILV-N-SLP--------TA
XP_018813891.1/1-238 -G--G---G-GAGPGPHRVSGSLLTVAFQ------------ILV-N-SLP--------TA
XP_012083470.1/1-239 -N--N---G----GGQQRVSGSLLTVAFQ------------ILV-N-SLP--------TA
XP_021651338.1/1-240 -N--N-----GGAGGQHRVSGSLLTVAFQ------------ILV-N-SLP--------TA
XP_021613538.1/1-241 -N--N---G-GGPGGQHRVSGSLLTVAFQ------------ILV-N-SLP--------TA
XP_021686862.1/1-241 -N--N---G-GGAAGQHRVSGSLLTVAFQ------------ILV-N-SLP--------TA
XP_021686863.1/1-241 -N--N---G-GGAAGQHRVSGSLLTVAFQ------------ILV-N-SLP--------TA
XP_021612685.1/1-241 -N--N---G-GGTGGQQRVSGSLLTVAFQ------------ILV-N-SLP--------TA
XP_023879166.1/1-242 -G--G---G-GGGPGPHRVSGSLLTVAFQ------------ILV-N-SLP--------TA
XP_002272264.2/1-234 -G--V---H-TNSGGPNRVSGSLLTVAFQ------------ILV-N-SLP--------TA
XP_010661562.1/1-234 -G--V---H-TNSGGPNRVSGSLLTVAFQ------------ILV-N-SLP--------TA
XP_002511801.1/1-237 -G--G---G-NNGGGPNRVSGSLLTVAFQ------------ILV-N-SLP--------TA
XP_015584500.1/1-237 -G--G---G-NNGGGPNRVSGSLLTVAFQ------------ILV-N-SLP--------TA
XP_022141257.1/1-238 -------------KIGH-SGGCLLTVAFQ------------ILV-N-SLP--------TA
XP_023885612.1/1-244 -------------NGCDGSGGCLLTVGFQ------------ILI-N-SQP--------AA
XP_023885611.1/1-244 -------------NGCDGSGGCLLTVGFQ------------ILI-N-SQP--------AA
XP_018830842.1/1-239 -------------NGTDG-GGCLLTVGFQ------------ILL-D-SQP--------TA
XP_018830843.1/1-239 -------------NGTDG-GGCLLTVGFQ------------ILL-D-SQP--------TA
XP_018830840.1/1-239 -------------NGTDG-GGCLLTVGFQ------------ILL-D-SQP--------TA
XP_018830841.1/1-239 -------------NGTDG-GGCLLTVGFQ------------ILL-D-SQP--------TA
XP_012090293.1/1-240 -------------DGNDG-AGSLLTVGFQ------------ILV-N-NLP--------TA
XP_012090292.1/1-240 -------------DGNDG-AGSLLTVGFQ------------ILV-N-NLP--------TA
XP_021614581.1/1-239 -------------DGGDG-GGSILTVGFQ------------ILV-N-NLP--------TA
XP_021614580.1/1-239 -------------DGGDG-GGSILTVGFQ------------ILV-N-NLP--------TA
XP_015581247.1/1-240 -------------DGGDG-GGCILTVGFQ------------ILV-N-NLP--------TA
XP_002510822.1/1-240 -------------DGGDG-GGCILTVGFQ------------ILV-N-NLP--------TA
XP_021648646.1/1-240 -------------DGGDG-EGSLLTVGFQ------------ILV-N-NLP--------TA
XP_021634593.1/1-234 -------------DGGDG-GGSLLTVGFQ------------ILV-N-NLP--------TA
XP_021634592.1/1-234 -------------DGGDG-GGSLLTVGFQ------------ILV-N-NLP--------TA
XP_006490348.1/1-238 -------------SSNES-GGCLLTVGFQ------------ILV-N-SLP--------TA
XP_006490346.1/1-238 -------------SSNES-GGCLLTVGFQ------------ILV-N-SLP--------TA
XP_024958654.1/1-238 -------------SSNES-GGCLLTVGFQ------------ILV-N-SLP--------TA
XP_006490345.1/1-238 -------------SSNES-GGCLLTVGFQ------------ILV-N-SLP--------TA
XP_006421879.2/1-238 -------------SSNES-GGCLLTVGFQ------------ILV-N-SLP--------TA
XP_021892212.1/1-221 ---------------KGA-EGCILTVGFQ------------ILV-N-SLP--------TA
XP_021892211.1/1-221 ---------------KGA-EGCILTVGFQ------------ILV-N-SLP--------TA
XP_021892210.1/1-221 ---------------KGA-EGCILTVGFQ------------ILV-N-SLP--------TA
XP_022765148.1/1-244 -------------DGSIS-GGCLLTVGFQ------------ILV-N-SAP--------TA
XP_022765158.1/1-244 -------------DGSIS-GGCLLTVGFQ------------ILV-N-SAP--------TA
XP_022765120.1/1-244 -------------DGSIS-GGCLLTVGFQ------------ILV-N-SAP--------TA
XP_022765114.1/1-244 -------------DGSIS-GGCLLTVGFQ------------ILV-N-SAP--------TA
XP_022736693.1/1-237 -------------DGSIS-SGCLLTVGFQ------------ILV-N-SIP--------TA
XP_022736692.1/1-237 -------------DGSIS-SGCLLTVGFQ------------ILV-N-SIP--------TA
XP_022736689.1/1-237 -------------DGSIS-SGCLLTVGFQ------------ILV-N-SIP--------TA
XP_022734775.1/1-244 -------------DGSIN-SGCILTVGFQ------------ILV-N-SIP--------TA
XP_016743353.1/1-244 -------------DGSIV-SGCLLTVGFQ------------ILV-N-NVP--------TA
XP_012474605.1/1-244 -------------DGSIV-SGCLLTVGFQ------------ILV-N-NVP--------TA
XP_012474606.1/1-244 -------------DGSIV-SGCLLTVGFQ------------ILV-N-NVP--------TA
XP_016743352.1/1-244 -------------DGSIV-SGCLLTVGFQ------------ILV-N-NVP--------TA
XP_016743351.1/1-244 -------------DGSIV-SGCLLTVGFQ------------ILV-N-NVP--------TA
XP_012474604.1/1-244 -------------DGSIV-SGCLLTVGFQ------------ILV-N-NVP--------TA
XP_012474603.1/1-244 -------------DGSIV-SGCLLTVGFQ------------ILV-N-NVP--------TA
XP_017646297.1/1-244 -------------DGSIV-SGCLLTVGFQ------------ILV-N-NVP--------TA
XP_016736315.1/1-244 -------------DGSIV-SGCLLTVGFQ------------ILV-N-NVP--------TA
XP_017646296.1/1-244 -------------DGSIV-SGCLLTVGFQ------------ILV-N-NVP--------TA
XP_017646298.1/1-244 -------------DGSIV-SGCLLTVGFQ------------ILV-N-NVP--------TA
XP_016736306.1/1-244 -------------DGSIV-SGCLLTVGFQ------------ILV-N-NVP--------TA
XP_017646295.1/1-244 -------------DGSIV-SGCLLTVGFQ------------ILV-N-NVP--------TA
XP_017972896.1/1-244 -------------DGSIS-GGCLLTVGFQ------------ILV-N-SLP--------TA
XP_017972895.1/1-244 -------------DGSIS-GGCLLTVGFQ------------ILV-N-SLP--------TA
XP_017972893.1/1-244 -------------DGSIS-GGCLLTVGFQ------------ILV-N-SLP--------TA
XP_021274823.1/1-244 -------------DGSIS-GGCLLTVGFQ------------ILV-N-NLP--------TA
XP_024536343.1/1-231 -------------RASSGEPGCLLTVAFQ------------ILV-S-NVP--------TA
XP_006366174.1/1-244 -P-----------GPDMRVSGSLLTVAFQ------------ILV-N-SLP--------TA
XP_020219711.1/1-212 -V-----------ASQARAGGCLLTVAFQ------------ILV-N-SLP--------TA
XP_022897056.1/1-222 -G-----------YPNPRVSGSLLTVAFQ------------ILV-N-SLP--------TA
NP_001281015.1/1-243 -D-----------DGGNRVSGSLLTMTFQ------------ILV-N-SLP--------AG
XP_008383260.1/1-243 -D-----------DGGNRVSGSLLTMTFQ------------ILV-N-SLP--------AG
XP_022896229.1/1-235 -G-----------GPGNRTSGSLLTVAFQ------------ILV-N-SLP--------AA
XP_022896230.1/1-235 -G-----------GPGNRTSGSLLTVAFQ------------ILV-N-SLP--------AA
XP_025816451.1/1-225 ----------------HKTGGSLLTVAFQ------------ILV-N-SQP--------TA
XP_004968001.1/1-225 ----------------HKTGGSLLTVAFQ------------ILV-N-SQP--------TA
XP_004968002.1/1-225 ----------------HKTGGSLLTVAFQ------------ILV-N-SQP--------TA
XP_002457484.1/1-226 ----------------HKTGGSLLTVAFQ------------ILV-N-SQP--------TA
XP_009400446.1/1-224 ----------------RKAVGSLLTVAFQ------------ILV-N-SQP--------TA
XP_018676535.1/1-225 --------------GTHKAPGSLLTVAFQ------------ILV-N-SQP--------TA
XP_009401771.1/1-232 --------------GARKAGGSLLTVAFQ------------ILV-N-SQP--------TA
XP_009384534.1/1-228 --------------GARKAGGSLLTVAFQ------------ILV-N-SQP--------TA
XP_020672447.1/1-217 ------------------GVGSLLTVAFQ------------ILV-N-SLP--------TA
XP_020582116.1/1-218 ------------------AVGSLLTVAFQ------------ILV-N-SLP--------TA
XP_020675647.1/1-231 --------------GKIGG-GSLLTVAFQ------------ILV-N-SLP--------MA
XP_020675648.1/1-231 --------------GKIGG-GSLLTVAFQ------------ILV-N-SLP--------MA
XP_020585632.1/1-228 --------------GKMGGGGSLLTVAFQ------------ILV-N-SLP--------MA
XP_016176402.1/1-228 --------------SSDDGGGSLLTVGFQ------------ILV-N-SLP--------TA
XP_016176403.1/1-228 --------------SSDDGGGSLLTVGFQ------------ILV-N-SLP--------TA
XP_016176400.1/1-228 --------------SSDDGGGSLLTVGFQ------------ILV-N-SLP--------TA
XP_016176401.1/1-228 --------------SSDDGGGSLLTVGFQ------------ILV-N-SLP--------TA
XP_010523042.1/1-245 -V----------SPRPPAPRGSLLTVAFQ------------ILV-N-SLP--------TA
XP_010523041.1/1-240 -V----------SPRPPAPRGSLLTVAFQ------------ILV-N-SLP--------TA
XP_020878511.1/1-224 ---------------RPDGGGSLLTVAFQ------------ILV-N-NLP--------TA
NP_567183.2/1-228    ---------------RPVGGGSLLTVAFQ------------ILV-N-NLP--------TA
XP_006287095.1/1-228 ---------------RPVGGGSLLTVAFQ------------ILV-N-NLP--------TA
XP_010456250.1/1-228 ---------------RPVGGGSLLTVAFQ------------ILV-N-NLP--------TA
XP_010419627.1/1-228 ---------------RPVGGGSLLTVAFQ------------ILV-N-NLP--------TA
XP_010427345.1/1-228 ---------------RPVGGGSLLTVAFQ------------ILV-N-NLP--------TA
XP_009111469.1/1-229 ---------------RPVGGGSLLTVAFQ------------ILV-N-NLP--------TA
XP_018487039.1/1-229 ---------------CPVGGGSLLTVAFQ------------ILV-N-NLP--------TA
XP_018453129.1/1-229 ---------------CPVGGGSLLTVAFQ------------ILV-N-NLP--------TA
XP_013612299.1/1-229 ---------------RPVGGGSLLTVAFQ------------ILV-N-NLP--------TA
XP_006396268.1/1-225 ---------------RPVGGGSLLTVAFQ------------ILV-N-NLP--------TA
XP_018475317.1/1-221 ---------------RPVGGGSLLTVAFQ------------ILV-N-NLP--------TA
XP_013629750.1/1-221 ---------------RPVGGGSLLTVAFQ------------ILV-N-DLP--------TA
XP_009134533.1/1-221 ---------------RPVGGGSLLTVAFQ------------ILV-N-NLP--------TA
XP_020531377.1/1-244 --------------GPARVGGSLLTVAFQ------------ILV-N-SLP--------TA
XP_006857866.1/1-244 --------------GPARVGGSLLTVAFQ------------ILV-N-SLP--------TA
XP_018434263.1/1-219 ----------------TEETGSLFTVAFQ------------ILV-N-SLP--------TA
XP_018434260.1/1-219 ----------------TEETGSLFTVAFQ------------ILV-N-SLP--------TA
XP_018434262.1/1-219 ----------------TEETGSLFTVAFQ------------ILV-N-SLP--------TA
XP_013688926.1/1-219 ----------------TEETGSLLTVAFQ------------ILV-N-SLP--------TA
XP_013637558.1/1-219 ----------------TEETGSLLTVAFQ------------ILV-N-SLP--------TA
XP_013637556.1/1-219 ----------------TEETGSLLTVAFQ------------ILV-N-SLP--------TA
XP_013637557.1/1-219 ----------------TEETGSLLTVAFQ------------ILV-N-SLP--------TA
XP_009143168.1/1-219 ----------------TEETGSLLTVAFQ------------ILV-N-SLP--------TA
XP_009143167.1/1-219 ----------------TEETGSLLTVAFQ------------ILV-N-SLP--------TA
XP_009143166.1/1-219 ----------------TEETGSLLTVAFQ------------ILV-N-SLP--------TA
XP_013692686.1/1-219 ----------------TEETGSLLTVAFQ------------ILV-N-SLP--------TA
XP_022574820.1/1-219 ----------------TEETGSLLTVAFQ------------ILV-N-SLP--------TA
XP_013692684.1/1-219 ----------------TEETGSLLTVAFQ------------ILV-N-SLP--------TA
XP_013748127.1/1-219 ----------------TEETGSLLTVAFQ------------ILV-N-SLP--------TA
XP_013692685.1/1-219 ----------------TEETGSLLTVAFQ------------ILV-N-SLP--------TA
XP_013748124.1/1-219 ----------------TEETGSLLTVAFQ------------ILV-N-SLP--------TA
XP_013748126.1/1-219 ----------------TEETGSLLTVAFQ------------ILV-N-SLP--------TA
XP_022574821.1/1-219 ----------------TEETGSLLTVAFQ------------ILV-N-SLP--------TA
XP_006290613.1/1-229 --------------SWMEEGGSLLTVAFQ------------ILV-N-SLP--------TA
XP_013592301.1/1-230 --------------GCMAEGGSLLTVAFQ------------ILV-N-SLP--------TA
XP_018442581.1/1-230 --------------GCMEEGGSLLTVAFQ------------ILV-N-SLP--------TA
XP_013750213.1/1-232 --------------GCMEEGGSLLTVAFQ------------ILV-N-SLP--------TA
XP_013648430.1/1-232 --------------GCMEEGGSLLTVAFQ------------ILV-N-SLP--------TA
XP_009104292.1/1-232 --------------GCMEEGGSLLTVAFQ------------ILV-N-SLP--------TA
XP_019058514.1/1-223 --------------RTDGEGGSLLTVAFQ------------ILV-N-SLP--------TA
XP_010523728.1/1-220 -----------------EEGGSLLTVAFQ------------ILV-N-SLP--------TA
XP_024514927.1/1-242 -GGGG--AD-LAEASSSSSSGALLTVAFQ------------ILV-S-NVP--------TA
XP_002985242.1/1-242 -GGGG--AD-LAEASSSSSSGALLTVAFQ------------ILV-S-NVP--------TA
XP_024530500.1/1-242 -GGGA--AD-LAEASSSSSSGALLTVAFQ------------ILV-S-NVP--------TA
XP_024530499.1/1-242 -GGGA--AD-LAEASSSSSSGALLTVAFQ------------ILV-S-NVP--------TA
XP_024530501.1/1-242 -GGGA--AD-LAEASSSSSSGALLTVAFQ------------ILV-S-NVP--------TA
XP_010107411.1/1-242 --------------GEGGGGGCLLSVGFQ------------ILV-N-SLP--------TA
XP_024028635.1/1-242 --------------GEGGGGGCLLSVGFQ------------ILV-N-SLP--------TA
XP_015877999.1/1-244 ----G-------SGGGGDGGGSLLTVGFQ------------ILV-N-SLP--------SA
XP_019427294.1/1-247 -GGGG-------GGGGDDGGGSLLTVGFQ------------ILV-N-NLP--------TA
XP_019427292.1/1-247 -GGGG-------GGGGDDGGGSLLTVGFQ------------ILV-N-NLP--------TA
XP_019452814.1/1-239 -------------G-GKDEGGSLLTVGFQ------------ILV-N-NLP--------TA
XP_019452815.1/1-239 -------------G-GKDEGGSLLTVGFQ------------ILV-N-NLP--------TA
XP_003592295.2/1-235 -------------A--SDGGGSLLTVGFQ------------ILV-N-SLP--------TA
XP_024625328.1/1-235 -------------A--SDGGGSLLTVGFQ------------ILV-N-SLP--------TA
XP_012570175.1/1-236 -------------SIGSDGGGSLLTVGFQ------------ILV-N-SLP--------TA
XP_012570176.1/1-236 -------------SIGSDGGGSLLTVGFQ------------ILV-N-SLP--------TA
XP_004496593.1/1-236 -------------SIGSDGGGSLLTVGFQ------------ILV-N-SLP--------TA
XP_025620154.1/1-234 -------------SSSDEGGGSLLTVGFQ------------ILV-N-SLP--------TA
XP_015941562.1/1-234 -------------SSSDEGGGSLLTVGFQ------------ILV-N-SLP--------TA
XP_025620153.1/1-234 -------------SSSDEGGGSLLTVGFQ------------ILV-N-SLP--------TA
XP_020239673.1/1-232 -------------G--DNSGGSLLTVGFQ------------ILV-N-SLP--------TA
XP_020239648.1/1-232 -------------G--DNSGGSLLTVGFQ------------ILV-N-SLP--------TA
XP_007143377.1/1-235 -------------GG-EESGGSLLTVGFQ------------ILV-N-SLP--------TA
XP_007143378.1/1-235 -------------GG-EESGGSLLTVGFQ------------ILV-N-SLP--------TA
XP_017415467.1/1-234 -------------GG-EDSGGSLLTVGFQ------------ILV-N-SLP--------TA
XP_017415466.1/1-234 -------------GG-EDSGGSLLTVGFQ------------ILV-N-SLP--------TA
XP_014514421.1/1-236 -------------GG-EESGGSLLTVGFQ------------ILV-N-SLP--------TA
XP_014514419.1/1-236 -------------GG-EESGGSLLTVGFQ------------ILV-N-SLP--------TA
XP_006606097.1/1-235 -------------GSDDGSGGSLLTVGFQ------------ILV-N-SLP--------TA
XP_006606096.1/1-235 -------------GSDDGSGGSLLTVGFQ------------ILV-N-SLP--------TA
XP_003536477.1/1-234 -------------GGNDG-GGSLLTVGFQ------------ILV-N-SLP--------TA
XP_006589557.1/1-234 -------------GGNDG-GGSLLTVGFQ------------ILV-N-SLP--------TA
XP_006402516.1/1-230 -RNG----------GCMEEGGSLLTVAFQ------------ILV-N-SLP--------TA
NP_191674.1/1-237    -GNG----------GCMEEGGSLLTVAFQ------------ILV-N-SLP--------TA
XP_002876596.1/1-239 -GNG----------GCMEEGGSLLTVAFQ------------ILV-N-SLP--------TA
XP_010413451.1/1-238 ---GS-------GEMCMEEGGSLLTVAFQ------------ILV-N-SLP--------TA
XP_010413452.1/1-238 ---GS-------GEMCMEEGGSLLTVAFQ------------ILV-N-SLP--------TA
XP_010413453.1/1-238 ---GS-------GEMCMEEGGSLLTVAFQ------------ILV-N-SLP--------TA
XP_010469036.1/1-242 -GNGN-------GGMCMEEGGSLLTVAFQ------------ILV-N-SLP--------TA
XP_010469037.1/1-242 -GNGN-------GGMCMEEGGSLLTVAFQ------------ILV-N-SLP--------TA
XP_010469038.1/1-242 -GNGN-------GGMCMEEGGSLLTVAFQ------------ILV-N-SLP--------TA
XP_010512448.1/1-238 -GNGN-------GGMCMEEGGSLLTVAFQ------------ILV-N-SLP--------TA
XP_010512449.1/1-238 -GNGN-------GGMCMEEGGSLLTVAFQ------------ILV-N-SLP--------TA
XP_006397757.1/1-219 --------------QGMPENESLLTVAFQ------------ILV-N-SLP--------TA
XP_024010837.1/1-220 --------------QGMPENESLLTVAFQ------------ILV-N-SLP--------TA
XP_023640300.1/1-220 --------------QGMEETGSLLTVAFQ------------ILV-N-SLP--------TA
XP_006293718.1/1-220 --------------QGMEETGSLLTVAFQ------------ILV-N-SLP--------TA
XP_010506553.1/1-219 --------------QGMEETGSLLTVAFQ------------ILV-N-SLP--------TA
XP_010508114.1/1-219 --------------QGMEETGSLLTVAFQ------------ILV-N-SLP--------TA
XP_010508115.1/1-219 --------------QGMEETGSLLTVAFQ------------ILV-N-SLP--------TA
XP_022147276.1/1-219 ------------------GGGCLLTVAFQ------------ILV-N-SLP--------TE
XP_023527464.1/1-222 ------------------GGGSLLTVAFQ------------ILV-N-SLP--------TD
XP_022980233.1/1-222 ------------------GGGSLLTVAFQ------------ILV-N-SLP--------TD
XP_022980231.1/1-222 ------------------GGGSLLTVAFQ------------ILV-N-SLP--------TD
XP_022924366.1/1-223 ------------------GGGSLLTVAFQ------------ILV-N-SLP--------TD
XP_022924364.1/1-223 ------------------GGGSLLTVAFQ------------ILV-N-SLP--------TD
XP_023527467.1/1-221 ------------------GGGSLLTVAFQ------------ILV-N-SLP--------TD
XP_023527463.1/1-221 ------------------GGGSLLTVAFQ------------ILV-N-SLP--------TD
XP_023527465.1/1-221 ------------------GGGSLLTVAFQ------------ILV-N-SLP--------TD
XP_022980232.1/1-221 ------------------GGGSLLTVAFQ------------ILV-N-SLP--------TD
XP_022924365.1/1-222 ------------------GGGSLLTVAFQ------------ILV-N-SLP--------TD
XP_009412599.1/1-224 ---------------TSGSLGSLLTVGFQ------------ILV-N-SQP--------TA
XP_018677084.1/1-231 ----------------HKAGGSLLTVGFQ------------ILV-N-NQP--------TA
XP_020114860.1/1-246 -SDAN-------IDGSSVGGGSLVTVAFQ------------ILV-N-SLP--------TA
XP_020114857.1/1-246 -SDAN-------IDGSSVGGGSLVTVAFQ------------ILV-N-SLP--------TA
XP_020114859.1/1-246 -SDAN-------IDGSSVGGGSLVTVAFQ------------ILV-N-SLP--------TA
XP_020084803.1/1-234 ------------------SPGSLLTVAFQ------------ILV-N-SQP--------TA
XP_015625616.1/1-226 ---------------GYETGGSLLTVAFQ------------ILV-N-NQP--------TA
XP_006647677.1/1-226 ---------------GYETGGSLLTVAFQ------------ILV-N-NQP--------TA
NP_001105493.1/1-226 ---------------EHKTGGSLLTVAFQ------------ILV-N-SQP--------TA
XP_020178635.1/1-225 ---------------EHKTGGSLLTVAFQ------------ILV-N-SQP--------TA
XP_003570085.1/1-223 ---------------EQKTGGSLLTVAFQ------------ILV-N-SQP--------TA
XP_020247871.1/1-226 ------QISPSDENVDGSVIGCLLTVGVQ------------VLA---STVP-------SA
XP_020247870.1/1-226 ------QISPSDENVDGSVIGCLLTVGVQ------------VLA---STVP-------SA
XP_024004764.1/1-211 -------------GISANSPYCLLTVGIQ------------VLV---SNVA-------TA
XP_018449374.1/1-217 ----------VFEGISVNLASCLLTVAVQ------------VLV---SDVT-------TA
XP_013602839.1/1-215 ----------VVEGISVNLDSCLLTVAIQ------------VLV---SNAT-------TA
XP_013655966.1/1-215 ----------VVEGISVNLDSCLLTVAIQ------------VLV---SNAT-------TG
XP_009108342.1/1-215 ----------VVEGISVNLDSCLLTVAIQ------------VLV---SNAT-------TA
XP_013655964.1/1-215 ----------VVEGISVNLDSCLLTVAIQ------------VLV---SNAT-------TA
XP_020872468.1/1-222 ----------GVEGISVNLPSCFLTVAIQ------------VLG---SNV-------TTA
NP_193506.2/1-221    ----------GVEGSSVSSPSCLLTVAIQ------------VLG---SNV-------TTE
XP_023633855.1/1-222 ----------GVEGISVNLPSCLLTVAIQ------------VLG---SNA---V---AAE
XP_010449572.1/1-221 ----------GVEGTSVNLPSCLLTVAMQ------------VL-----GTS--A---ADA
XP_010439957.1/1-223 ----------GVKGTSVNLPSCFLTVAIQ------------VLV---KNTS--G---ADA
XP_010439959.1/1-223 ----------GVKGTSVNLPSCFLTVAIQ------------VLV---KNTS--G---ADA
XP_010439958.1/1-223 ----------GVKGTSVNLPSCFLTVAIQ------------VLV---KNTS--G---ADA
XP_006854056.1/1-234 -------NGNGISSSNNRGDGCLMTVGVQ------------MLA---SSNP-------TA
XP_020529072.1/1-234 -------NGNGISSSNNRGDGCLMTVGVQ------------MLA---SSNP-------TA
NP_199499.3/1-224    -----------ISVNSHSPPSCLLTVGIQ------------VLA---SNVP-------TA
XP_020871346.1/1-224 -----------VSVNSNSPPSCLLTVGIQ------------VLA---SNVP-------TA
XP_006281890.1/1-224 -----------VSVNSNSPQSCLLTVAIQ------------VLA---SNVP-------TA
XP_010481400.1/1-224 -----------ISVNSNSPPSCLLTVAIQ------------VLA---SNVP-------TA
XP_010494885.1/1-224 -----------ISVNSNSPPSCLLTVAIQ------------VLA---SNVP-------TA
XP_018455882.1/1-227 -----------ISVNLLPPPSCLLTVGIQ------------VLA---SNVP-------TA
XP_013713146.1/1-227 -----------ISVNSIPPPSCLLTVGIQ------------VLA---SNVP-------TA
XP_013607209.1/1-227 -----------ISVNSIPPPSCLLTVGIQ------------VLA---SNVP-------TA
XP_009114317.1/1-227 -----------ISVNSIPPPSCLLTVGIQ------------VLA---SNVP-------TA
XP_013661958.1/1-227 -----------ISVNSIPPPSCLLTVGIQ------------VLA---SNVP-------TA
XP_006398384.1/1-227 -----------NSVNSLSPPSCLLTVGIQ------------VLA---SNVP-------TA
XP_024011152.1/1-227 -----------NSVNSLSPPSCLLTVGIQ------------VLA---SNVP-------TA
XP_018482944.1/1-227 -----------ISVNSHPLPSCLLTVGIQ------------VLA---SNVP-------TA
XP_009101472.1/1-227 -----------VSVNSIPLPSCLLTVGIQ------------VLA---SNVP-------TA
XP_013693979.1/1-227 -----------VSVNSHPLPSCLLTVGIQ------------VLA---SNVP-------TA
XP_013615089.1/1-227 -----------VSVNSHPLPSCLLTVGIQ------------VLA---SNVP-------TA
XP_013704013.1/1-227 -----------VSVNSHPLPSCLLTVGIQ------------VLA---SNVP-------TA
XP_009373477.1/1-246 ----------QNQENRIMNSGCLLTVGFQ------------VLA---STSP-------SA
XP_006662631.2/1-256 PPPGNS----NADELPPAANGCLLTVGMQ------------VLA---SAVP-------SA
XP_015613461.1/1-259 PPRNSSSNNNNADELL-PPNGCLLTVGMQ------------VLA---SAVP-------SA
NP_001337805.1/1-240 -----------AGNADEPASGCLLTVGMQ------------VLA---SAVP-------SA
XP_020163828.1/1-239 ------------GNADEPATGCLLTVGMQ------------VLA---SAVP-------SA
XP_010235052.1/1-238 ------------GNADEPANGCLLTVGMQ------------VLA---SAVP-------SA
XP_025798256.1/1-238 ------------GKPDEPASGCLLTVGMQ------------VLA---SAVP-------SA
XP_004983555.1/1-238 ------------GNTDEPASGCLLTVGMQ------------VLA---SAVP-------SA
XP_002464880.2/1-238 ------------GNTDEPTSGCLLTVGMQ------------VLA---SAVP-------SA
XP_020262583.1/1-231 -----------VNIDVSAASGCLLTIGVQ------------VLV---SSVP-------SA
XP_020677410.1/1-231 ----------PAATNNSAAIGCLLTIGMQ------------VLA---SAVP-------SA
XP_020577604.1/1-229 ----------TA-TNNSVPVGCLLTVGMQ------------VLA---SAVP-------SA
XP_020577606.1/1-229 ----------TA-TNNSVPVGCLLTVGMQ------------VLA---SAVP-------SA
XP_020577607.1/1-229 ----------TA-TNNSVPVGCLLTVGMQ------------VLA---SAVP-------SA
XP_019709148.1/1-245 ------DGTATSCGGDGSATGCLLTVAMQ------------VLA---SAVP-------SA
XP_008803567.1/1-241 -------AGAASCGSDGSATGCLLTVAVQ------------VLA---STVP-------SA
XP_008803570.2/1-241 -------AGAASCGSDGSATGCLLTVAVQ------------VLA---STVP-------SA
XP_018681950.1/1-231 ----------GSGSDGHATVGCLLTVGMQ------------VLA---TAVP-------SA
XP_018681951.1/1-231 ----------GSGSDGHATVGCLLTVGMQ------------VLA---TAVP-------SA
XP_010919976.1/1-237 ----------AGASGDGSATGCLLTVGMQ------------VLA---SAVP-------SA
XP_008800084.1/1-237 ----------VGATGDGSAIGCLLTVGMQ------------VLA---SAVP-------SA
XP_018679997.1/1-229 -----------SSDGSASTIGCLLTIGMQ------------VLA---SAVP-------SA
XP_009397149.1/1-229 -----------SSDGSASTIGCLLTIGMQ------------VLA---SAVP-------SA
XP_024995523.1/1-235 -------ATTSIGNTTESGGGCLLTVGLQ------------VLA---NALP-------TA
XP_023736252.1/1-233 --------TTTTGNSTESGGGCLLTVGLQ------------VLA---NAIP-------TA
XP_023736253.1/1-233 --------TTTTGNSTESGGGCLLTVGLQ------------VLA---NAIP-------TA
XP_022022041.1/1-231 -------TT----TTAESGGGCLLTVGLQ------------VLA---NSMP-------TA
XP_021969857.1/1-226 --------------SSESGGGCLLTVGLQ------------VVA---NSMP-------TA
XP_010245213.1/1-236 SGI-------PSEGANGPSPGCLLTVGLQ------------VLA---STVP-------SA
XP_010245210.1/1-236 SGI-------PSEGANGPSPGCLLTVGLQ------------VLA---STVP-------SA
XP_010276832.1/1-235 VPS-------PEEGNGQAPAGCLLTVGLQ------------VLA---STIP-------SA
XP_010276837.1/1-235 VPS-------PEEGNGQAPAGCLLTVGLQ------------VLA---STIP-------SA
XP_019155823.1/1-230 --------VQSSESGCLPESGCLLTVALQ------------VLA---STMP-------TA
XP_022881662.1/1-238 DGT-----QNSENVNLQPDSGCLLTVGLQ------------VLA---STIP-------TA
XP_022877113.1/1-234 ---------DTENGNLQPDSGCLLTVGLQ------------VLA---STIP-------TA
XP_016450789.1/1-246 SGTQSAE-KRNLSIQESGSGGCLLTVGLQ------------VLA---STIP-------TA
XP_009597691.1/1-246 SGTQSAE-KRNLSIQESGSGGCLLTVGLQ------------VLA---STIP-------TA
XP_009786380.1/1-246 SGAQSSE-KRNLSIQESASGGCLLTVGLQ------------ALA---STIP-------TA
XP_016494823.1/1-246 SGAQSSE-KRNLSIQESASGGCLLTVGLQ------------ALA---STIP-------TA
XP_016494822.1/1-246 SGAQSSE-KRNLSIQESASGGCLLTVGLQ------------ALA---STIP-------TA
XP_019230103.1/1-246 SGAQSSE-KRNLSIQESGSGGCLLTVGLQ------------VLA---STIP-------TA
XP_016573090.1/1-250 SSAQSSEKRQNLSSIQEYSGGCLLTIGLQ------------VLA---STIP-------TA
XP_016573102.1/1-250 SSAQSSEKRQNLSSIQEYSGGCLLTIGLQ------------VLA---STIP-------TA
XP_006352911.1/1-250 NSSQSSEKRQNLSSIQEYSGGCLLTVGLQ------------VLA---STIP-------SA
XP_015166604.1/1-250 NSSQSSEKRQNLSSIQEYSGGCLLTVGLQ------------VLA---STIP-------SA
XP_015085651.1/1-250 NSSQSSEKRQNSSSIQEYSGGCLLTVGLQ------------VLA---STIP-------SA
XP_015085654.1/1-250 NSSQSSEKRQNSSSIQEYSGGCLLTVGLQ------------VLA---STIP-------SA
XP_004245919.1/1-250 NSSQSSEKRQNLSSIQEYSGGCLLTVGLQ------------VLA---STIP-------SA
XP_025888165.1/1-250 NSSQSSEKRQNLSSIQEYSGGCLLTVGLQ------------VLA---STIP-------SA
XP_011070327.1/1-236 ------DSKQASENGHLPDSGCLLTVGLQ------------VLA---STIP-------SA
XP_011070329.1/1-236 ------DSKQASENGHLPDSGCLLTVGLQ------------VLA---STIP-------SA
XP_012843107.1/1-236 ------TKQPSDQNGQLPDSGCLLTVGLQ------------VLA---STIP-------TA
XP_019423575.1/1-234 ----------LPNTTTSVNSGCLLTAGLQ------------VLA---STIP-------SA
XP_019423571.1/1-234 ----------LPNTTTSVNSGCLLTAGLQ------------VLA---STIP-------SA
XP_019435331.1/1-234 ----------KLPATTSVNSGCLLTVGMQ------------VLA---STNP-------SA
XP_003612811.1/1-239 --------PSSTASANAANSGCLLIMGMQ------------VLA---STIP-------SA
XP_020978415.1/1-227 ------------SEALINEHGCLLTVGLQ------------ILA---STIP-------SA
XP_015963259.1/1-227 ------------SEALINDHGCLLTVGLQ------------ILA---STIP-------SA
XP_025697880.1/1-227 ------------SEALINEHGCLLTVGLQ------------ILA---STIP-------SA
XP_025697882.1/1-227 ------------SEALINEHGCLLTVGLQ------------ILA---STIP-------SA
XP_025697881.1/1-227 ------------SEALINEHGCLLTVGLQ------------ILA---STIP-------SA
XP_020217931.1/1-233 -----NSSEAPPSV----NSGCLLTMGLQ------------VLA---STIP-------SA
XP_007158253.1/1-232 -----NSS-EPPSL----NSGCLLTMGLQ------------VLA---STIP-------SA
XP_017427665.1/1-232 -----NSS-ESPSL----NSGCLLTMGLQ------------VLA---STIP-------SA
XP_017427666.1/1-232 -----NSS-ESPSL----NSGCLLTMGLQ------------VLA---STIP-------SA
XP_014521371.1/1-232 -----NSS-ESPSL----NSGCLLTMGLQ------------VLA---STIP-------SA
XP_014521373.1/1-232 -----NSS-ESPSL----NSGCLLTMGLQ------------VLA---STIP-------SA
XP_006587604.1/1-242 -----SSSEPPPSLNNNNSGGCLLTMGLQ------------VLA---STIP-------SA
NP_001352051.1/1-233 -----NSSE-PPISLNNNSGGCLLTMGVQ------------VLA---STIP-------SA
XP_018840870.1/1-239 NPNI-PSSE-EGN--SPTSSGCLLTVVVQ------------DLV---STIP-------SA
XP_018852246.1/1-240 NQIP-SSED-QGGNAPSSSPGCLLTIVVQ------------VLA---STVP-------SA
XP_010053633.1/1-234 -------NDNLGLDACPQGEGCLLTVGLQ------------VLA---STIP-------TA
XP_021675948.1/1-236 NSIQ------SLQEGNCTNSGCLLTVGLQ------------VLA---STIP-------SA
XP_012081064.1/1-228 ----------SSQEGNNNSSGCLLTVGLQ------------VLA---STIP-------SA
XP_011028126.1/1-235 NSMP-SNSEDGN---GHNNSGCLLTVGLQ------------VLA---STIP-------SA
XP_002303433.2/1-235 NSMP-SNSEDGN---GHNNSGCLLTVGLQ------------VLA---STIP-------SA
XP_015897558.1/1-247 SSV--PSSEDATGSAATVSSSCLLTVGLQ------------VLA---STIP-------SA
XP_002277673.1/1-238 NPMP-PSG-----DGNGHNSGCLLTVGLQ------------VLA---STIP-------TA
XP_022996123.1/1-236 RLAS-SPPKDGATNAAVVNSGCLLTVGLQ------------VLA---STIP-------SA
XP_023534245.1/1-236 RLAS-SPPKDGATNAAVVNSGCLLTVGLQ------------VLA---STIP-------SA
XP_022931244.1/1-236 RLAS-SPPKDGATNAAVVNSGCLLTVGLQ------------VLA---STIP-------SA
XP_022931242.1/1-236 RLAS-SPPKDGATNAAVVNSGCLLTVGLQ------------VLA---STIP-------SA
XP_022159691.1/1-235 HPLP-PPDVDG-STAAVVNSGCLLTVGLQ------------VLA---STIP-------SA
XP_011652640.1/1-232 HPAP-PPEDGT----PNPNSGCLLTVGLQ------------VLA---STIP-------SA
XP_011652639.1/1-232 HPAP-PPEDGT----PNPNSGCLLTVGLQ------------VLA---STIP-------SA
XP_008466007.1/1-232 HPAP-PPDDGT----PNANSGCLLTVGLQ------------VLA---STIP-------SA
XP_023511589.1/1-230 HPSP-PPEDGV----A--NSGCLLTVGLQ------------VLA---STIP-------SA
XP_022963906.1/1-230 HPSP-PPEDGV----A--NSGCLLTVGLQ------------VLA---STIP-------SA
XP_023511591.1/1-230 HPSP-PPEDGV----A--NSGCLLTVGLQ------------VLA---STIP-------SA
XP_022963908.1/1-230 HPSP-PPEDGV----A--NSGCLLTVGLQ------------VLA---STIP-------SA
XP_022967184.1/1-230 HPSP-PQEDSV----A--NSGCLLTVGLQ------------VLA---STIP-------SA
XP_022967186.1/1-230 HPSP-PQEDSV----A--NSGCLLTVGLQ------------VLA---STIP-------SA
XP_010518669.1/1-225 --------------ADANTASCLLTVGIQ------------VLA---SSVP-------TA
XP_010677365.1/1-235 --------QGQTNMNHHNTGGCLLTIGIQ------------VHA---SAIP-------NA
XP_010677364.1/1-235 --------QGQTNMNHHNTGGCLLTIGIQ------------VHA---SAIP-------NA
XP_021852084.1/1-226 --------G---------GGGCLLTVGIQ------------VHA---SAIP-------NA
XP_021852076.1/1-226 --------G---------GGGCLLTVGIQ------------VHA---SAIP-------NA
XP_021765591.1/1-233 --------GQ--SSSSNMGGGCLLTVGIQ------------VHA---SASP-------NA
XP_021768254.1/1-233 --------GQ--SSSSNMDGGCLLTVGIQ------------VHA---SASP-------NA
XP_017232690.1/1-221 ---------------ATQDPGCLLTIGLQ------------VLA---STVP-------NA
XP_017232692.1/1-221 ---------------ATQDPGCLLTIGLQ------------VLA---STVP-------NA
XP_024021697.1/1-248 AASSSEAAAAAAAAAGSAKPSCLLTVGLH------------VLV---STIP-------SA
XP_010096931.1/1-248 AASSSEAAAAAAAAAGSAKPSCLLTVGLH------------VLV---STIP-------SA
XP_024021696.1/1-248 AASSSEAAAAAAAAAGSAKPSCLLTVGLH------------VLV---STIP-------SA
XP_017621310.1/1-230 ------------ANGHNITTGSLLTVGLQ------------VLA---STIP-------SA
XP_016731150.1/1-230 ------------ANGHNITTGSLLTVGLQ------------VLA---STIP-------SA
XP_012472199.1/1-230 ------------ANGHNITTGSLLTVGLQ------------VLA---STIP-------SA
XP_016727305.1/1-230 ------------ANGHNITTGSLLTVGLQ------------VLA---STIP-------SA
XP_016731145.1/1-230 ------------ANEHNITTGSLLTVGLQ------------VLA---STIP-------SA
XP_012472200.1/1-230 ------------ANGHNITAGSLLTVGLQ------------VLA---STIP-------SA
XP_012472201.1/1-230 ------------ANGHNITAGSLLTVGLQ------------VLA---STIP-------SA
NP_001314348.1/1-230 ------------ANGHNITTGSLLTVGLQ------------VLA---STIP-------SA
XP_016727300.1/1-230 ------------ANGHNITTGSLLTVGLQ------------VLA---STIP-------SA
XP_022738572.1/1-235 --------VPPSEKANGHISGSLLTVGLQ------------ILA---STIP-------SA
XP_021297648.1/1-235 --------VPPSEEASGHISGSLLTVGLQ------------VLA---STVP-------SA
XP_017973778.1/1-235 --------VPPSEEANGHISGSLLTVGLQ------------VLA---STVP-------SA
XP_017973780.1/1-235 --------VPPSEEANGHISGSLLTVGLQ------------VLA---STVP-------SA
XP_022717458.1/1-235 --------VPPSEEANGHISGSLLTVGLQ------------VLA---SSVP-------ST
XP_022717453.1/1-235 --------VPPSEEANGHISGSLLTVGLQ------------VLA---SSVP-------ST
XP_022717437.1/1-235 --------VPPSEEANGHISGSLLTVGLQ------------VLA---SSVP-------ST
XP_022756982.1/1-232 --------VPPSEEANGHIQGSLLTVGLQ------------VLA---STVP-------SA
XP_004300207.1/1-230 ----------SPSDQYSRNSGCLMTVGLQ------------VVA---SPNP-------SA
XP_024169578.1/1-230 ----------SPSAQDSRNSGCLMTVGLQ------------VVA---SPNP-------SA
XP_008238183.1/1-249 DHRQEDSTATTTTTSNVINSGCLLTVGLQ------------VLA---STSP-------SA
XP_016651445.1/1-249 DHRQEDSTATTTTTSNVINSGCLLTVGLQ------------VLA---STSP-------SA
XP_020418924.1/1-249 DHRQEDSTATTTTTSNVINSGCLLTVGLQ------------VLA---STSP-------SA
XP_021804146.1/1-249 DHRQENSTATTTTTSNVINSGCLLTVGLQ------------VLA---STSP-------SA
XP_008238184.1/1-248 DHRQEDSTATTTTTSNVINSGCLLTVGLQ------------VLA---STSP-------SA
XP_016651446.1/1-248 DHRQEDSTATTTTTSNVINSGCLLTVGLQ------------VLA---STSP-------SA
XP_007209851.1/1-248 DHRQEDSTATTTTTSNVINSGCLLTVGLQ------------VLA---STSP-------SA
XP_021804154.1/1-248 DHRQENSTATTTTTSNVINSGCLLTVGLQ------------VLA---STSP-------SA
XP_017179076.1/1-239 DQNHE---------SSIMNTGCLLTVGLQ------------VLA---STSP-------SA
XP_008356445.1/1-246 DQNQE---------KRIMNSGCLLTVGFQ------------VLA---STSP-------SA
XP_008356446.1/1-240 DQNQE---------KRIMNSGCLLTVGFQ------------VLA---STSP-------SA
XP_008356447.1/1-239 DQNQE---------KRIMNSGCLLTVGFQ------------VLA---STSP-------SA
XP_009373485.1/1-239 DQNQE---------NRIMNSGCLLTVGFQ------------VLA---STSP-------SA
XP_009373484.1/1-240 DQNQE---------NRIMNSGCLLTVGFQ------------VLA---STSP-------SA
XP_004512486.1/1-245 V-TGTP--SSTTTTTTTANSGCLLIMGMQ------------VLA---STIP-------SA
XP_023898631.1/1-242 P---MPSSEEGNASTTSSPAGCLLTVVLQ------------VVA---STIP-------SA
XP_024465375.1/1-238 --MQ--SNSEDGNGNGHNNSGCLLTVGLQ------------ALA---STIP-------SA
XP_011018502.1/1-238 --VQ--SNSEDGNGNGHNNSGCLLTVGLQ------------ALA---STIS-------SA
XP_011018503.1/1-238 --VQ--SNSEDGNGNGHNNSGCLLTVGLQ------------ALA---STIS-------SA
XP_021893615.1/1-233 ---------SSSPDQQQNSAGCLLTVGLQ------------ALA---STVP-------TS
XP_024952893.1/1-247 S-MALSPEEANNIGVQNNSAGCLLTVGLQ------------VLG---STIP-------SA
XP_006471522.1/1-248 S-MALSPEEANNIGVQNNSAGCLLTVGLQ------------VLG---STIP-------SA
XP_006432745.2/1-248 S-MALSPEEANNIGVQNNSAGCLLTVGLQ------------VLG---STIP-------SA
XP_021640807.1/1-241 ---Q--SSQDG--NNNNSNSGCLLTVGLQ------------VLA-NNSTIP-------SA
XP_002518976.1/1-243 ---K--SSEENGNTGHGCTSGCLLTIGLQ------------VLA---STIP-------SA
XP_021598537.1/1-237 --------Q---HGNCTTNPGCLLTVGLQ------------ILA---STIP-------SA
XP_021598535.1/1-239 --------Q---HGNCTTNPGCLLTVGLQ------------ILA---STIP-------SA
XP_006659280.1/1-226 -QASSSSSAGI--ASRSRTGGSFVTVAYQ------------MFV----------------
XP_025801379.1/1-254 -APSTSSSAPVL---RNDTGGSLVTMAYQ------------VLL-P-GPLP--DT---AA
XP_023156326.1/1-229 -S----SSTPVGC--DDTTAGSLLTVACQ------------ALV-P-GSSP--SDNRAA-
NP_001349156.1/1-229 -S----SSTPVGC--DDTTAGSLLTVACQ------------ALV-P-GSSP--SDNRAA-
XP_021310588.1/1-246 ------SSAPIGR--DNHTAGSILTMACQ------------ALL-P-GLSS--SDKHAAD
XP_020404516.1/1-224 -NSSSA----LAGLNG--TAGCLLTAAYQ------------VPV----------------
XP_022684239.1/1-229 -SSSYST--P---TGQNSTAGSLLTAAYQ------------QIL-P-NSIS--TH-----
XP_020404892.1/1-233 -ATNSSASAAIDDDHSSNAAGSLVTVAFQ------------TPL-P-GNP----------
XP_022684238.1/1-237 -S---STSAPI-DNNDDDGEGALLTVAFQ------------AML-P-SSPS--GD--HAT
XP_025825251.1/1-229 -S---SSSAP------NNGEGALLTVASL------------GML-S-SSPS--GG--PAA
XP_020183090.1/1-242 -ANPSGVDPMD----HQRSRGSLVSVLWQ------------TPL-V-GG-----N--LTA
XP_020194819.1/1-240 -AMCSSSSSAS----HRSNSGSLVSIMYQ------------TLL-S-GQPP--EH--LFK
XP_024314575.1/1-244 -AGS-SSSAGV----SATCAGSIVSALYQ------------TEL-S-APLQ--QS--VAI
XP_024314342.1/1-235 ------------------SSGAIVSAMYQ------------ILP-S-SASP--QN--LTL
XP_024959674.1/1-192 ---------------IAVSEGSILTLSFE------------IRDE---------------
XP_019196338.1/1-215 -----------GSSS-------ILSICFE------------LVDEK-LSST--DH--LPP
XP_006646400.1/1-226 -----------SGMDLGDVGGTLMTLGFQ------------IPV-K-LATG--NG--MYP
XP_015632244.1/1-226 -----------SGIALGEVGGTLMTMGFQ------------IPV-K-LASG--DR--MYS
XP_024314625.1/1-231 -----------GQAPLGDDGETIVTVGFQ------------ILV-R-HAQD---D--VFS
XP_020164122.1/1-228 -------------LGLGEGGATLVTVGFQ------------IML-K-LARG--TG--LYP
XP_025814677.1/1-221 ------------GSPIGEAGGTVMTAGFQ------------ILM-K-LARG--TG--LCP
XP_022682466.1/1-225 ------------GAPMGEAGRTMMTAGFQ------------ILM-K-LARG--TG--LCP
XP_002456467.1/1-226 ------------GAGIAQPGATVMTAGFQ------------ILM-K-LARG--TG--LCS
XP_008674944.1/1-228 -----------GSGSIAQPGATVMTAGFQ------------ILM-K-MARG--TG--LCP
XP_010523539.1/1-228 --------AGS--SRSGGDEWCVVTAGFQ------------MVA-G-DAAL--TG--GVS
XP_020873847.1/1-203 -----------------EEAGCVVSVGFQ------------AIV-T-EAIV--AN--TMM
NP_001190248.1/1-203 -----------------VTASCYVKADQ-------------TMV-T-SPNE--LG--SYM
NP_196343.2/1-203    -----------------VTASCYVKADQ-------------TMV-T-SPNE--LG--SYM
XP_020876233.1/1-195 -----------------VSAGCHVRADQ-------------AMV-T-SPNE---------
XP_010423235.1/1-188 -----------------LSAACQVKINQ-------------T------------------
XP_010452682.1/1-218 -----------------LSAACQVKINQ-------------TIL-T-DSDK--LM--EFM
XP_010491330.1/1-188 -----------------LSVACQVKINQ-------------T------------------
XP_006413300.2/1-224 -------------DADELGEGCLLTIGYQ------------FLF-T-KNLT--TE--EVD
XP_022008499.1/1-216 ---------------ESGESGSMVTVGFH------------RLL-R-NQVI--SN---IT
XP_022008502.1/1-215 ---------------ESGESGSMVTVGFH------------RLL-R-NQVI--SN---IT
XP_022007635.1/1-226 -------------GNAGGERGSMVTVGFQ------------LLH-P-DIAT--SN--LIT
XP_022008501.1/1-224 -------------DGANGERGSMVTVGFQ------------LLH-P-DIAT--SN--LVT
XP_021986984.1/1-226 -------------DGAGGERGSMVTVGLQ------------LLH-P-DITT--SN--MIT
XP_021996009.1/1-224 -------------DHAGGERGSMVTVGLQ------------LLH-P-DITT--SN--MIT
XP_021996011.1/1-224 -------------DGAGGERGSMVTVGLQ------------LLH-P-DITT--SN--MIT
XP_022026050.1/1-224 -------------DGAGGERGSMVTVGLQ------------LLH-P-DITT--SN--MIT
XP_022026083.1/1-224 -------------DGAGGERGSMVTVGLQ------------LLH-P-DITT--SN--MIT
XP_021722389.1/1-209 --------------QEEEDAGSIVTLSFQ------------LLD-K-SSRA--QN--VPK
XP_021847315.1/1-219 --------------QEEENEGSIVTVLFQ------------VLD-K-SSKA--QD--IPK
XP_020224300.1/1-216 -----------RQHPNNRSGGSFLTVVYQ------------LLL-C-SNEE--LI--GDI
XP_021765248.1/1-224 -----------ASSSVTHLGGSLITVAFQ------------VLA-C-SSLD--TI--YVG
XP_004510020.1/1-219 --------P----GSHHTQKGTLLTFSYQ------------VLV-S-DQTS--GA--Q--
XP_004487953.1/1-231 --------SSNIENATSSRGKSLLTMAFQ------------MVY-V-QVEP--TI--Q--
XP_008244376.1/1-218 ----------------GETGGSLLTIAFH------------VVA-K-SPTD--EH--EPS
XP_008244380.1/1-218 ----------------GETGGSLLTIAFH------------VVA-K-SPTD--EH--EPS
XP_012569676.1/1-224 ---------GNNNNNNTSGAGSLLTLAYQ------------IFV-A-DHYE--EN--VNN
XP_019418393.1/1-229 --------ASTSR-NHGGTRGSLVTIALQ------------IQM-S-NYIN--SD--F--
XP_019440118.1/1-223 --------SSSSG-NHGATRGSLVTVALQ------------IEI-S-AAIN--SD--S--
XP_014517363.2/1-226 ---------WSSGSGDKRTHGTLMTMAFQ------------ILM-N-GPTT--MV--AES
XP_012569675.1/1-231 --------SNRNGGTSAKPRGSLLTVAFQ------------ILM-S-NAEV--VN--LEH
XP_004486570.1/1-223 --------SGSNGNGGVKTRGSLLTVALQ------------ILM-H-GEI---MS--LES
XP_003594569.3/1-220 ----------------SKSRGSLLTVAFQ------------MHM-P-APLI--TN--FES
XP_024632692.1/1-220 ----------------SKSRGSLLTVAFQ------------MHM-P-APLI--TN--FES
XP_025887127.1/1-229 ---------TCEEMDNGFEDGSMVTINFQ------------MMG-N-MLPD--T--TLSM
XP_015078684.1/1-230 ---------TSEEMDNGFEDGSMVTINFQ------------MMG-N-MLPD--T--TLSM
XP_025887126.1/1-230 ---------TCEEMDNGFEDGSMVTINFQ------------MMG-N-MLPD--T--TLSM
XP_015078401.1/1-220 -------------NDNGFCGGSLVTIGLQ------------MLV-K-PFQN--K--THTI
XP_015158871.1/1-217 -------NRILGENDNGFCGGSLVTIGFQ------------MLV-K-TIHS--K--KHTI
XP_015078778.1/1-220 -------EF---------GSGSLVTIMFQ------------MLV-D-NIST--A--DLPQ
XP_010321992.1/1-216 -------EF---------GSGSLVTIMFQ------------MLV-D-NIST--A--DLPQ
XP_004240735.1/1-216 -------EF---------GSGSLVTIMFQ------------MLV-D-NIST--A--DLPQ
XP_015169793.1/1-213 -------NDIFEKNDNGVYGGSLEALSLA------------IQN-D-------G--DFRD
XP_006285631.1/1-212 ----------------STGGGCLLTLGLQ------------ILA-S-TNPT--A--QLTQ
XP_023635531.1/1-198 ----------------STGGGCLLTLGLQ------------ILA-S-TNPT--A--QLTQ
NP_567722.1/1-217    ------------RG--NTGGGCLLTFGLQ------------ILV-G-INPT--A--ALIQ
XP_020873337.1/1-219 ------------KSKTGGGGGCLLTFGLQ------------LLF-S-SNPT--A--ELPQ
XP_020873338.1/1-219 ------------KSKTGGGGGCLLTFGLQ------------LLF-S-SNPT--A--ELPQ
XP_023000325.1/1-225 ------SK-E----ERVTEGGSLLTIATQ------------IPV-S-SSGT--A--EKTS
XP_023000326.1/1-214 ------SK-E----ERVTEGGSLLTIATQ-----------------------------TS
XP_022963997.1/1-214 ------SK-E----ERVTEGGSLLTIATQ-----------------------------TS
XP_023513617.1/1-208 ------SK-E----ERVTEGGSLLTIATQ-----------------------------TS
XP_021312124.1/1-225 ------R---EQDRTAVDSAGSLVTMVFQ------------ALF-S-SSAT---DAALSA
XP_012701492.1/1-243 ------RKGEEESRAAAEAGGSLVTVAFQ------------ALA-S-SSPT--ADDALPP
XP_025815339.1/1-229 ------CRKEEEDRAAAEAGGALVTVAFQ------------ALA-S-PSPP--DAA----
XP_015644459.1/1-237 ------SRRGGEA----SGAGSLVTVAFQ------------VPA-S-PSAA---AATLSP
XP_024314016.1/1-228 ------SSRKG-D----VAAGSLVTVAFQ------------VLA-S-SS-L---TATLSP
XP_020168333.1/1-225 -------RRED-G----AAAGSLVTVAFQ------------VLA-S-SS-P---AAALSP
XP_013589242.1/1-206 -------------DPNRSRVGCLLTLGYQ------------MLH-S-VKP----GEDVDQ
XP_022548456.1/1-204 -------------DPN---VGCLLTLGYQ------------MLH-S-VKP----TEDVDQ
XP_024632691.1/1-227 -------------ASKSIEDGSLITLAAQ------------TYA-I-GQV-------LNV
XP_017256377.1/1-220 --------------QTTGTGGSILTIVFQ------------IAD-E-NLS---SPEYLPP
XP_009632057.1/1-224 --------------EDILTGGSVLTIAFQ------------LMD-E-ELS---TPEYLPP
XP_016502932.1/1-224 --------------EDILTGGSVLTIAFQ------------LMD-E-ELS---TPEYLPP
XP_019258997.1/1-221 --------------EEIVTGGSVLTMAFQ------------LMD-E-ELS---TPEYLPP
XP_009792862.1/1-224 --------------EEIVTGGSVLTIALQ------------LMD-E-ELS---TPEYLPP
XP_016544784.1/1-213 --------------------GSILTIAFH------------IMD-E-ELS---TPEYLPP
XP_015089689.1/1-213 --------------------GSILTMAFQ------------LMD-E-EIS---TPEYLPP
XP_025883585.1/1-213 --------------------GSILTMAFQ------------LMD-E-EIS---TPEYLPP
XP_011095790.1/1-221 --------------DDAATQNTILTIGFQ------------IMD-E-QLT---TPEELPP
XP_020553953.1/1-221 --------------DDAATQNTILTIGFQ------------IMD-E-QLT---TPEELPP
XP_022857758.1/1-208 -------------------RESILTIGFQ------------IMD-E-ELT---TPEYLPP
XP_004487739.1/1-224 ---------------NKSNNESLLTISFH------------LID-K-TNN----SSSISP
XP_003596663.1/1-221 -------------ANSTANDESLLTISFH------------IIG-K-ANN----AASIPP
XP_003596665.1/1-227 -------------ANSTANNESLLTMSFH------------IID-N-ASN----VASIAP
XP_020201972.1/1-216 -----------------AT-ASFLTVAFH------------IVD-G-AST----RPFISS
XP_025980734.1/1-220 -----------------GTTGSLLTVAFH------------VFE-S-VTN----KSHILP
XP_007149715.1/1-218 -----------------ST-RSLLTIAFH------------IVE-S-CTT----RPYIAP
XP_017423636.1/1-218 -----------------NS-RSLLTIAFH------------IVE-S-TTS----KPYLAP
XP_022631965.1/1-218 -----------------NS-RSLLTIAFH------------IVE-S-TTN----EPYLAP
XP_014522034.2/1-218 -----------------NS-RSLLTIAFH------------IVE-S-TTN----EPYLAP
XP_025669173.1/1-223 -----------------GEGGSILTIAFH------------TIE-S-HNI----KQFFSL
XP_020963786.1/1-223 -----------------GEGGSILTIAFH------------TIE-S-HNI----KQFFSL
XP_025614692.1/1-223 -----------------GEGGSILTIAFH------------TIE-S-HNI----KQFFSL
XP_015936351.1/1-223 -----------------GEGGSILTIAFH------------TIE-S-HNI----KQFFSL
XP_019451675.1/1-226 -----------------NSGGSILTIAFH------------IVE-S-SSI----RSFIPS
XP_019465428.1/1-219 -----------------NGDGSLLTIAFH------------SIE-S-TSI----RSSIPS
XP_004295798.2/1-220 ---------------NDESGGSLLTLALH------------ITE-E-GAT----EDFIPP
XP_021807338.1/1-219 ---------------WDETGGSLLTIAFH------------VVA-K-SPT----DEREPS
XP_021807340.1/1-219 ---------------WDETGGSLLTIAFH------------VVA-K-SPT----DEREPS
XP_020417197.1/1-219 ---------------RDETGGSLLTIAFH------------VVA-K-SPT----DEHEPS
XP_020417200.1/1-219 ---------------RDETGGSLLTIAFH------------VVA-K-SPT----DEHEPS
XP_009333923.1/1-206 ---------------RDDTGGSLLTIAFH------------VAA-G-TST----QEHVSA
XP_017184363.1/1-206 ---------------RDDTGGSLLTIXFH------------VAA-G-TST----QEHVSA
XP_021892911.1/1-224 --------------SNEKVGGTILTLVFH------------IID-N-SGR----LGNIPA
XP_002278971.2/1-210 --------------DGGS--GSLVTMAFQ------------ILD-N-SSS----ATYIPP
XP_010644412.1/1-211 --------------D--E--GSLVTIVFQ------------ILD-G-TAS----PMHFPS
XP_024450660.1/1-210 --------------NGEETRGSLLTLAFH------------IMD-G-SSTE--DYLP-AA
XP_002522867.2/1-214 -------------YGGHEGDGCVLTLAFH------------IID-G-SSIK--DI--ISP
XP_020534605.1/1-220 -------------GHGHQVDGSVLTLFFH------------ILD-G-CSTE--DSIYIPR
XP_021685850.1/1-217 -------------NNGKEVGGSLLTLAFH------------ILD-G-SSME--DSQFIPR
XP_021594464.1/1-220 -------------YNGEEVGGSLLTVAFH------------IVD-G-FSME--DSLYIPR
XP_018731177.1/1-223 -------------GGDSR--RSLLTVVFH------------IMD-V-SARV--QSNILPR
XP_024018593.1/1-217 -------------GGVAH--GCLLTLALH------------IVD-D-RTS----KERITK
XP_022139420.1/1-219 -------------GEEYETDGSILTVALN------------IID-H-SIT-----ERIPY
XP_022981426.1/1-220 -------------GQEIEADGSIVTVALN------------IVD-H-SIT-----ERIPF
XP_022981427.1/1-220 -------------GQEIEADGSIVTVALN------------IVD-H-SIT-----ERIPF
XP_022941489.1/1-220 -------------GQEVEADGSIVTVALN------------IVD-H-SIT-----ERIPF
XP_022941488.1/1-220 -------------GQEVEADGSIVTVALN------------IVD-H-SIT-----ERIPF
XP_023525599.1/1-220 -------------GQEIEADGSIVTVALN------------IVD-H-SIT-----ERIPF
XP_023525597.1/1-220 -------------GQEIEADGSIVTVALN------------IVD-H-SIT-----ERIPF
XP_008454681.2/1-220 -------------GEEDVADGSILTVALN------------IID-H-SVT-----QRVPF
XP_011648553.1/1-223 -------------GEEDVADGSILTVALN------------IID-H-SVT-----QRVPF
XP_018822340.1/1-211 -------------GQDSS--GSLLSVTFH------------IID-R-ASM----DGYVPP
XP_018822367.1/1-211 -------------GQDSS--GSLLSVTFH------------IID-R-ASM----DGYVPP
XP_023876961.1/1-210 -------------GEEYS--ASLLTLGFH------------IID-G-AST----KSFIPS
XP_023876962.1/1-210 -------------GEEYS--ASLLTLGFH------------IID-G-AST----KSFIPS
XP_024034242.1/1-215 -------------GHGDV-PGSLLTIAFH------------IID-L-AST----EEYIPP
XP_015381349.2/1-215 -------------GHGDV-PGSLLTIAFH------------IID-L-AST----EEYIPP
XP_022717683.1/1-216 -------------GEEPV--GSILTIAFH------------TAD-R-SST----EEYIPP
XP_016729588.1/1-216 -------------GDGAV--GSILTLAFQ------------SVD-R-LSN----KEYMPQ
XP_016729589.1/1-216 -------------GDGAV--GSILTLAFQ------------SVD-R-LSN----KEYMPQ
XP_016702140.1/1-208 -------------GDGAV--GSILTLAFQ------------SVD-R-LSN----KEYMPQ
XP_012460158.1/1-216 -------------GDGAG--GSILTLAFQ------------SVD-R-LSN----KEYMRP
XP_012460145.1/1-216 -------------GDGAG--GSILTLAFQ------------SVD-R-LSN----KEYMRP
XP_017245631.1/1-197 -----MSE-------NSATPGSMSLE-SA------------NLL-N-T------------
XP_004956045.3/1-247 -----ASTSRAPVCRHNNTEGSLLTMAYQ------------VLL-P-DNL--------TA
XP_014660176.1/1-240 ------CTSEAPV-GDGNTAGALLTVACQ------------ALL-P-GSP----SGDHTA
XP_017239609.1/1-213 -----------------GSSGTLLTVSFQ------------VLT-KEINP----VDAGNC
XP_023740378.1/1-206 -----------------EADGSMVTVGFQ------------MSL-QDLVT----PNLITM
XP_020095129.1/1-235 --------------CDGENGGSLLTVVFQ------------IIA-DRSGP----EPGLPA
XP_020258373.1/1-212 --------------------GSLVTVSIQ------------ILS-D-SNP----QTDVNP
XP_008812148.1/1-175 --------------GEGGSGGSLLTVSFQ------------ILA-D-SNP----HSNISA
XP_010930674.1/1-230 --------------GEGGSGGSLLTVSFQ------------ILV-D-SNP----DSNISA
XP_010931877.1/1-230 --------------GEGGSGGSLLTVSFQ------------ILV-D-SNP----DSNISA
XP_009380061.1/1-225 -SSCPT-------G---RPSGSLVTVAFQ------------MLM-S-SS-------P-GA
XP_010939971.1/1-229 -SSNPM-------G---VASGSLVTVLFQ------------LLA-S-SS-------P-SA
XP_019710626.1/1-218 -SSNPM-------G---VASGSLVTVLFQ------------LLA-S-SS-------P-SA
XP_008787891.1/1-229 -SSNPM-------G---VTSGSLVTVLFQ------------LLV-S-SS-------P-SA
XP_008787892.1/1-218 -SSNPM-------G---VTSGSLVTVLFQ------------LLV-S-SS-------P-SA
XP_008799024.1/1-230 -SSNPM-------G---GASGSLVTVLFQ------------LLV-S-SS-------P-SA
XP_010936845.1/1-230 -SSNPM-------G---VASGSLVTVLFQ------------LLV-S-SS-------P-SA
XP_003570542.1/1-223 -------------------SGSLVTVAFQ------------ILV-S-SL-------P-SS
XP_004964922.1/1-229 -SAAPL-------A---SPPGCVVTVAFQ------------ILV-S-NL-------P-SS
XP_002438026.1/1-230 -VVLPL-------A---SQPGCVVTVAFQ------------ILV-S-NL-------P-SS
XP_020399989.1/1-232 -SAVPL-------AAAPPPPGCVVTVAFQ------------ILV-S-NL-------P-SS
XP_008649207.1/1-222 -------------SAVVPPPGCVVTVAFQ------------ILV-S-NL-------P-SS
XP_020256822.1/1-228 -SSNPI-------S---GSSGSLLTITFQ------------ILM-S-SL-------P-TA
XP_009403550.1/1-228 -SSTPM-------V---GSSGSLVTVAYQ------------IHL-S-SL-------P-SA
XP_020582158.1/1-232 -SLNPL-------R---VPSGSVLTVAFQ------------IMV-S-SL-------P-SA
XP_020699118.1/1-240 -SSNPL-------G---GSSGTVLTIAFQ------------IMV-S-SL-------P-SA
XP_020600375.1/1-232 -SSNPM-------G---GSSGSVLTVAFQ------------IMV-S-SL-------P-SA
XP_020600376.1/1-232 -SSNPM-------G---GSSGSVLTVAFQ------------IMV-S-SL-------P-SA
XP_020684394.1/1-232 -SSNPM-------G---GSSGSVLTVAFQ------------IMV-S-SL-------P-SA
XP_017257147.1/1-219 --------------AGEMAVGSIVTVVVQ------------IIV-S-NL--------TSG
XP_010244428.1/1-230 ------------QGNTSRSSGSLITVAYQ------------ILV-S-SL-------P-TA
XP_010244355.1/1-230 ------------QGNTSRSSGSLITVAYQ------------ILV-S-SL-------P-TA
XP_017240757.1/1-236 -SSS----------SATTTSGSLLTIVIQ------------IMV-S-HNM-----ASSGP
XP_017245021.1/1-233 -SSS----------SSTTTTGSLLTIVIQ------------IMV-S-H-M-----ASTTT
XP_012829269.1/1-233 -STS-------NGPSSAAATGSLMTVVFQ------------ILV-S-NL-------P-NA
XP_022889938.1/1-236 -ST------SLNTNIGDGSSGSLITIVFQ------------ILV-S-SL-------T-SA
XP_022897471.1/1-232 -ST------S----TGGSSSGSLITIVFQ------------ILV-S-SL-------P-SA
XP_009606250.1/1-235 -SSTNN-----GTMGDNGSEGSLVTVVFQ------------ILV-S-SL-------SSSA
XP_016468230.1/1-235 -SSTNN-----GTMGDNGSEGSLVTVVFQ------------ILV-S-SL-------SSSA
XP_019250773.1/1-235 -SSTNN-----GTMGDNRSEGSLVTVVFQ------------ILV-S-SL-------SSSA
XP_009778533.1/1-235 -SSTNN-----GTMGDNGSEGSLVTVVFQ------------ILV-S-SL-------SSSA
XP_016451582.1/1-235 -SSTNN-----GTMGDNGSEGSLVTVVFQ------------ILV-S-SL-------SSSA
XP_016578176.1/1-240 -NNNNDTMGEHNRTGGGRGGGSLVTVAFQ------------ILV-S-SL-------SSSA
XP_006347370.1/1-231 -------MNNGSTMGGAGAGGSLVTVVFQ------------ILV-S-SL-------SSSA
XP_004241474.1/1-230 -------MNNGSTMRA-GGGGSLVTVVFQ------------ILV-S-SL-------SSSA
XP_015077748.1/1-230 -------MNNGSTMGA-GGGGSLVTVVFQ------------ILV-S-SL-------SSSA
XP_022037776.1/1-241 -SNT--TTSDLAGDQGGTSSGSLVTVVFQ------------IQT-N-SL-------P-SA
XP_024992062.1/1-237 -TD----HG--ADHRGSTSTGSLITVVFQ------------IQV-S-SL-------P-AA
XP_023772270.1/1-245 -SNNNSDLGAADHCGGGTSTGSLVTVVFQ------------IQV-S-SL-------P-SA
XP_012855673.1/1-237 -SSNSTTV------DRSSSSGSLVTVVFQ------------ILV-S-NS-------P-SA
XP_022858328.1/1-236 -SSNTGGVG-----GRLPSAGSLITVVFQ------------ILL-S-NL-------P-SA
XP_011072039.1/1-232 -SSN-----------LDRSSGSLITVVFQ------------ILV-S-NL-------P-TA
XP_006306921.1/1-215 ---------------KG-G-GSLITVGFQ------------IMV-S-GL-------Q-PA
NP_564041.2/1-215    ---------------KG-G-GSLITVGFQ------------IMV-S-GL-------Q-PA
XP_020866823.1/1-216 ---------------KGGG-GSLITVGFQ------------IMV-S-GL-------Q-PA
XP_010476956.1/1-215 ---------------KG---GSLITVGFQ------------IMV-S-GM-------Q-PA
XP_010498167.1/1-217 ---------------KGGGGGSLITVGFQ------------IMV-S-GL-------Q-PA
XP_019091528.1/1-217 ---------------KGGVGGSLITVGFQ------------IMV-S-GL-------Q-PA
XP_013613976.1/1-227 -------------GGGG-GGGSLITVGFQ------------IMV-S-SL-------Q-SG
XP_018432674.1/1-227 -------------GGGG-GGGSLITVGFQ------------IMV-S-SL-------Q-SG
XP_018432667.1/1-227 -------------GGGG-GGGSLITVGFQ------------IMV-S-SL-------Q-SG
XP_013641731.1/1-225 -------------GG---GGGSLITVGFQ------------IMV-S-SL-------Q-SG
XP_009149243.1/1-227 -------------GGGE-GGGSLITVGFQ------------IMV-S-SL-------Q-SG
XP_006416656.2/1-224 -------------G----GGGSLITVGFQ------------IMV-S-SL-------Q-SA
XP_018451633.1/1-224 -------------GGG--GGGSLITVGFQ------------IMV-T-SL-------Q-SG
XP_018451632.1/1-225 -------------GGGG-GGGSLITVGFQ------------IMV-T-SL-------Q-SG
XP_018451634.1/1-195 -------------GG----GGSLITVGFQ------------IMV----------------
XP_013690958.1/1-228 -------------GGGSGGGGSLITVGFQ------------IMV-S-SL-------Q-SG
XP_009110376.1/1-227 -------------GG-SGGGGSLITVGFQ------------IMV-S-SL-------Q-SG
XP_009110377.1/1-227 -------------GG-SGGGGSLITVGFQ------------IMV-S-SL-------Q-SG
XP_013601964.1/1-227 -------------GGGGGGGGSLITVGFQ------------IMV-S-SL-------Q-SG
XP_013653874.1/1-227 -------------GRGSGGGGSLITVGFQ------------IMV-S-SL-------Q-SG
XP_018445004.1/1-237 -SSGRS-----SSSSGFGGGGSLITVGFQ------------IMV-S-NL-------P-SA
XP_018445005.1/1-237 -SSGRS-----SSSSGFGGGGSLITVGFQ------------IMV-S-NL-------P-SA
XP_013648946.1/1-228 -----------SSSSGYGGGGSLITVGFQ------------IMV-S-NL-------P-SA
XP_009104812.1/1-228 -----------SSSSGYGGGGSLITVGFQ------------IMV-S-NL-------P-SA
XP_013588523.1/1-228 -----------SSSSGYGGGGSLITVGFQ------------IMV-S-NL-------P-SA
XP_013699248.1/1-228 -----------SSSSGYGGGGSLITVGFQ------------ITV-S-NL-------P-SA
XP_018457296.1/1-228 -----------STSSGFAGGGSLITVGFQ------------IMV-S-NL-------P-SA
XP_009105998.1/1-230 -----------STSSGFGGGGSLITVGFQ------------IMV-S-NL-------P-SA
NP_001302850.1/1-231 -----------STSSGFGGGGSLITVGFQ------------IMV-S-NL-------P-SA
XP_013698656.1/1-231 -----------STSSGFGGGGSLITVGFQ------------IMV-S-NL-------P-SA
XP_013649619.1/1-231 -----------STSSGFGGGGSLITVGFQ------------IMV-S-NL-------P-SA
XP_013592873.1/1-231 -----------STSSGFGGGGSLITVGFQ------------IMV-S-NL-------P-SA
XP_006390565.1/1-233 -SSGRS-----SSSSGFGGGGSLITVGFQ------------IMV-S-NL-------P-SA
XP_006301097.1/1-230 -SSGR-----------SSSSGSLITVGFQ------------IMV-S-NL-------P-SA
XP_010428260.1/1-231 -SSGRS-----S---SSNSSGSLITVGFQ------------IMV-S-NL-------P-SA
XP_010471396.1/1-230 -SSGRS-----S----SNSSGSLITVGFQ------------IMV-S-NL-------P-SA
XP_010416135.1/1-230 -SSGRS-----S----SNSSGSLITVGFQ------------IMV-S-NL-------P-SA
NP_177479.1/1-227    -SSGRA-----S------ASGSLITVGFQ------------IMV-S-NL-------P-TA
XP_020890987.1/1-228 -SSGRS-----S------SSGSLITVGFQ------------IMV-S-NL-------P-SA
XP_010537520.1/1-232 -SSGRS-----S---SGGVGGSLITVAFQ------------IMV-S-SL-------P-SA
XP_010540042.1/1-226 -S--------------SFGGGSLITVAFQ------------IMV-S-SL-------S-SA
XP_010540046.1/1-196 -S--------------SFGGGSLITVAFQ------------IMV-S-SL-------S-SA
XP_019432660.1/1-235 -SSNTG-----RS-IGRCSGGSLITVALQ------------IMM-N-NL-------P-SA
XP_019420678.1/1-233 -SSSLT-----RS-IGG-SGGSLITVSFQ------------ILV-S-NI-------P-SA
XP_013452816.1/1-243 -SS-NL-----GN-NGG-SGGSLITVAFQ------------IMV-S-SL-------P-SA
XP_020215886.1/1-221 -SSNSI--------RSG-SGGSLVTIALQ------------ILV-S-SL-------P-SA
XP_020215888.1/1-221 -SSNSI--------RSG-SGGSLVTIALQ------------ILV-S-SL-------P-SA
XP_007160572.1/1-230 -SSNGN-----RN-KGR-SGGSLVTVAFQ------------ILV-S-SS-------P-SA
XP_017424661.1/1-212 ------------------SGGSLVTVAFQ------------ILV-S-SS-------P-SA
XP_017424660.1/1-230 -SSNAN-----RN-KAR-SGGSLVTVAFQ------------ILV-S-SS-------P-SA
XP_014508329.1/1-230 -SSNAN-----RN-KAR-CGGSLVTVAFQ------------ILV-S-SS-------P-SA
XP_022632295.1/1-230 -SSNAN-----RN-KAR-CGGSLVTVAFQ------------ILV-S-SS-------P-SA
XP_019225984.1/1-234 ----CSSSSSNASTIGGRSGGSLITVVFQ------------ILV-S-SL-------P-SA
XP_016441514.1/1-233 ----CSSS-SNASTIGGRSGGSLITVVFQ------------ILV-S-SL-------P-SA
XP_009587298.1/1-233 ----CSSS-SNASTIGGRSGGSLITVVFQ------------ILV-S-SL-------P-SA
XP_016441515.1/1-233 ----CSSS-SNASTIGGRSGGSLITVVFQ------------ILV-S-SL-------P-SA
XP_009786509.1/1-233 ----CSSS-SNASTIGGRSGGSLITVVFQ------------ILV-S-SL-------P-SA
XP_016563952.1/1-234 ----CSSSSNASTIGGGRSGGSLITVVFQ------------ILV-S-SL-------P-SA
XP_004235382.1/1-234 ----CSSSSNASTIGGGRSGGSLITVVFQ------------ILV-S-SL-------P-SA
XP_015068940.1/1-234 ----CSSSSNASTIGGGRSGGSLITVVFQ------------ILV-S-SL-------P-SA
XP_006364383.1/1-234 ----CSSSSNASTIGGGRSGGSLITVVFQ------------ILV-S-SL-------P-SA
XP_019167409.1/1-245 -STSNPASSTSTSAAGGGEGGSLITVVFQ------------ILV-S-SL-------P-SA
XP_011086973.1/1-238 ----DGASTSSNTGIGGNSSGSLITVVFQ------------ILV-S-NL-------P-SA
XP_010268321.1/1-230 -L----------QGSMSKSSGSLITVAFQ------------ILV-S-SL-------P-SA
XP_010268322.1/1-230 -L----------QGSMSKSSGSLITVAFQ------------ILV-S-SL-------P-SA
XP_010069492.1/1-236 -AAA------AGQGSTSRSAGSLMTVAFQ------------ILV-S-SL-------P-SA
XP_002527933.1/1-229 -NAH------GS--MCR-SSGSLITVSFQ------------ILV-S-SL-------P-SA
XP_021678368.1/1-229 -NTH------GS--IGR-SSGSLITVSFQ------------ILV-S-SL-------P-SA
XP_021678369.1/1-229 -NTH------GS--IGR-SSGSLITVSFQ------------ILV-S-SL-------P-SA
XP_021602738.1/1-229 -NTH------GS--MSR-SSGSLITVSFQ------------ILV-S-SL-------P-SA
XP_012079215.1/1-230 -STH------VS--MGRRSSGSLITVSFQ------------ILV-S-SL-------P-SA
XP_021687701.1/1-229 -STH------AS--MGR-SSGSLITVSFQ------------ILV-S-SL-------P-SA
XP_006374212.1/1-231 -NTQ------GS--TA-RLSGSLITVAFQ------------ILV-S-SL-------P-SA
XP_011035552.1/1-231 -NTQ------GS--TA-RLSGSLITVAFQ------------ILV-S-SL-------P-SA
XP_002317722.3/1-231 -NTQ------GR--MA-RSSGSLITVAFQ------------ILV-S-SL-------P-SA
XP_011000766.1/1-231 -NTQ------GR--MT-RSSGSLITVAFQ------------ILV-S-SL-------P-SA
XP_021907827.1/1-230 -S--------NV--LGNSSSGSLITVAFQ------------ILV-S-SL-------P-SA
XP_017635401.1/1-230 -STG----------HGRSSGGSLITVAFQ------------ILV-S-SL-------P-SA
XP_016686699.1/1-230 -STG----------HGRSSGGSLITVAFQ------------ILV-S-SL-------P-SA
XP_012440361.1/1-230 -STG----------HGRSSGGSLITVAFQ------------ILV-S-SL-------P-SA
NP_001313868.1/1-230 -STG----------HGRSSGGSLITVAFQ------------ILV-S-SL-------P-SA
XP_023919022.1/1-230 -STH------GH----GRSGGSLITVAFQ------------ILV-S-SL-------P-SA
XP_006478112.1/1-232 -NVH------GN--MGSRSSGSLISVAFQ------------ILV-S-SL-------P-SA
XP_024042646.1/1-232 -NVH------GN--MGSRSSGSLISVAFQ------------ILV-S-SL-------P-SA
XP_022715403.1/1-231 -SAH------GN--MG-RSGGSLITVAFQ------------ILV-S-SL-------P-SA
XP_022767009.1/1-255 -SAN------GN--MG-RSGGSLITIAFQ------------ILV-S-SL-------P-SA
XP_022737050.1/1-231 -SSH------GN--MG-KSGGSLITVAFQ------------ILV-S-SL-------P-SA
XP_017973835.1/1-231 -TVH------GN--MG-RSGGSLITVAFQ------------ILV-S-SL-------P-SA
XP_015967008.1/1-238 -STSSTPSRSSSTMVGMRSGGSLVTVAFQ------------ILV-S-SL-------P-SA
XP_025701434.1/1-239 -STSSTPSRSSSTMVGMRSGGSLVTVAFQ------------ILV-S-SL-------P-SA
XP_020980110.1/1-239 -STSSTPSRSSSTMVGMRSGGSLVTVAFQ------------ILV-S-SL-------P-SA
XP_019434881.1/1-231 -STSSNTST-----NIGRSGGSLITVAFQ------------ILV-S-NL-------P-SA
XP_019458275.1/1-234 -STSSSTSK-----NIGRCSGSLVTVAFQ------------ILV-S-NL-------P-SA
XP_019458276.1/1-234 -STSSSTSK-----NIGRCSGSLVTVAFQ------------ILV-S-NL-------P-SA
XP_006584922.1/1-234 -S--S----NNANRNIVRSGGSLVTIAFQ------------ILV-S-SL-------P-SA
XP_003524332.1/1-231 ----S----SNTNRNIARSGGSLVTIAFQ------------ILV-S-SL-------P-SA
XP_004515249.1/1-229 ----NVGDGASTSSNTNLGGGSLITVAFQ------------IMV-S-SL-------P-SA
XP_019431381.1/1-229 ----AS-----TSTNKNSSGGSLVTVAFQ------------ILV-S-SL-------P-SS
XP_019436771.1/1-231 -S--TS-----SNTNRNTSSGSLVTVAFQ------------ILV-S-SL-------P-SA
XP_019430342.1/1-233 -S--TS-----SNTNRNTSGGSLVTVAFQ------------ILV-S-TL-------P-SA
XP_006587436.1/1-240 -T--STGSRVMGGGSGPGSGGSLITVAFQ------------ILV-S-SL-------P-SA
XP_003549189.1/1-236 -T----SSRVMGGGS--GSGGSLITVAFQ------------ILV-S-SL-------P-SA
XP_020221986.1/1-232 -T--STSRA-M-----GGSGGSLITVAFQ------------ILV-S-SL-------P-SA
XP_007152598.1/1-234 -T--SSGRV-M-----GGSGGSLITVAFQ------------ILV-S-SL-------P-SA
XP_017437542.1/1-234 -T--SSGRV-M-----GGSGGSLITVAFQ------------ILV-S-SL-------P-SA
XP_014505752.1/1-234 -T--SSGRV-M-----GGSGGSLITVAFQ------------ILV-S-SL-------P-SA
XP_015881991.1/1-234 -S------SNTHGNLLGRSSGSLITVAFQ------------ILI-S-SL-------P-NA
XP_004299483.1/1-232 -S--CS-NI-----NHIGGSGSLITVAFQ------------ILV-S-SL-------P-SA
XP_024174647.1/1-232 -S--CS-NV-----NHIGGGGSLITVAFQ------------ILV-S-SL-------P-SA
XP_008375041.1/1-227 --------------CNATGSGSLITVAFQ------------ILV-S-SL-------P-ST
XP_017188172.1/1-199 --------------CNATGSGSLITVAFQ------------IL-----------------
XP_009355964.1/1-227 --------------CNATGSGSLITVAFQ------------ILV-S-SL-------P-AT
XP_009355965.1/1-199 --------------CNATGSGSLITVAFQ------------IL-----------------
XP_023523434.1/1-228 -D------------VHNRSGGSLVTVAFQ------------ILV-S-SL-------P-SG
XP_022949138.1/1-227 -D------------VQ-RSGGSLVTVAFQ------------ILV-S-SL-------P-SG
XP_022998050.1/1-227 -D------------VQ-RSGGSLVTVAFQ------------ILV-S-SL-------P-SG
XP_002271012.1/1-231 -S--TT-------ASMGRSGGSLITVVFQ------------ILV-S-SL-------P-SA
XP_010663934.1/1-231 -S--TT-------ASMGRSGGSLITVVFQ------------ILV-S-SL-------P-SA
XP_010099679.1/1-237 -T--SS-AAVPPGNAMGRSSGSLITVAFQ------------ILV-S-SL-------P-SA
XP_018823334.1/1-236 -S---S-SSQHGGTMMSRFGGSLITVAFQ------------ILV-S-SL-------P-SA
XP_023001145.1/1-228 -D------------VHSRSGGSLLTVAFQ------------ILV-S-SL-------P-SG
XP_023519889.1/1-228 -D------------VHSRSGGSLLTVAFQ------------ILV-S-SL-------P-SG
XP_022924191.1/1-228 -D------------VHSRSGGSLLTVAFQ------------ILV-S-SL-------P-SG
XP_018849090.1/1-235 -T---S-SS-THGSMARLGSGSLITVAFQ------------ILV-S-SL-------P-SA
XP_008240378.1/1-228 -C------------NVHGSGGSLVTVAFQ------------ILV-S-SL-------P-SA
XP_007210460.2/1-228 -C------------NVHGSGGSLVTVAFQ------------ILV-S-SL-------P-SA
XP_021801797.1/1-228 -C------------NVHGSGGSLVTVAFQ------------ILV-S-SL-------P-SA
XP_022136988.1/1-228 -D------------VHSRSRGSLVTVAFQ------------ILV-S-SL-------P-SG
XP_016898817.1/1-228 -D------------VHNRSGGSLVTVAFQ------------ILV-S-SL-------P-SG
XP_004137568.1/1-228 -D------------VHNRSGGSLVTVAFQ------------ILV-S-SL-------P-SG
XP_015642098.1/1-238 --------AARGGGGGGAGGGSVVTVAFQ------------ILV-S-SL-------P-SS
XP_020186741.1/1-220 -----------------SSTGSVVTVAFQ------------ILV-S-SL-------P-SS
XP_025828617.1/1-229 -------------LG-SPPAGSLVTVAFQ------------ILV-S-SL-------P-SS
XP_004954072.1/1-226 -------------LG-SPAAGSLVTVAFQ------------ILV-S-SL-------P-SS
XP_002452898.1/1-231 -------------LGATAAAGSLVTVAFQ------------ILV-S-SL-------P-SS
NP_001142912.2/1-230 -------------VGSPAAAGSLVTVAFQ------------ILV-S-SL-------P-SS
XP_020089796.1/1-231 -------------QTGFNSSGSLLTVAFQ------------ILV-S-SL-------P-SS
XP_020089795.1/1-241 -------------QTGFNSSGSLLTVAFQ------------ILV-S-SL-------P-SS
XP_019708739.1/1-228 ---------------MGGSSGSLITVAFQ------------ILV-S-SL-------P-SA
XP_008789232.1/1-228 ---------------MGGSSGSLITVAFQ------------ILV-S-SL-------P-SA
XP_008789231.1/1-228 ---------------MGGSSGSLITVAFQ------------ILV-S-SL-------P-SA
XP_008789233.1/1-200 ---------------MGGSSGSLITVAFQ------------IL-----------------
XP_019704584.1/1-228 ---------------MGGSSGSLITVAFQ------------ILV-S-SL-------P-SA
XP_010917295.1/1-228 ---------------MGGSSGSLITVAFQ------------ILV-S-SL-------P-SA
XP_008806630.1/1-228 ---------------MGGSSGSLITVAFQ------------ILV-S-SL-------P-SA
XP_009388746.1/1-226 ---------------MVGSSGSLLTVAFQ------------ILM-S-GL-------P-SA
XP_018675467.1/1-228 ---------------MGGSAGSLVTVAFQ------------ILM-S-SL-------P-SA
XP_018675466.1/1-228 ---------------MGGSAGSLVTVAFQ------------ILM-S-SL-------P-SA
XP_009409108.1/1-228 ---------------LGGSSGSLVTVAFQ------------ILM-S-SL-------P-SA
XP_024625408.1/1-220 --------DVASTSSNTNIGGSLLTVAFQ------------IMV-N-S-----------L
XP_010550261.1/1-219 -------------------GSTLLTVAFQ------------ILV-S-GPT-------YSR
XP_018816719.1/1-228 --------------NAGMSGGSLLTVAFQ------------ILV-S-SPS--------SK
XP_008365756.2/1-218 -------------------CGSLLTVAFH------------ILV-S-SPT-------TSK
XP_008381522.1/1-218 -------------------CGSLLTVAFH------------ILV-S-SPT-------TSK
XP_018506060.1/1-218 -------------------CGSLVTVAFH------------ILV-S-SPT-------TSE
XP_008362571.1/1-218 -------------------CXSLVTVXFH------------ILV-S-SPT-------TSE
XP_023870350.1/1-229 --------------NAVKSGGSLLTVAFQ------------ILV-S-SPS-------SLK
XP_016649213.1/1-222 --------------SNGRSGGSLLTVAFQ------------ILV-S-CPA-------SSK
XP_020415996.1/1-222 --------------SNGRSGGSLLTVAFQ------------ILV-S-SPT-------SSK
XP_021823813.1/1-222 --------------SNGRSGGSLLTVAFQ------------ILV-S-CPT-------SSK
XP_009367126.1/1-222 --------------GHSTAGGSLLTVAFQ------------ILV-S-SPS-------TSK
XP_008348615.1/1-222 --------------GHSTAGGSLLTVAFQ------------ILV-S-SPS-------TSK
XP_017179578.1/1-222 --------------GHSKXGGSLLTVAFQ------------ILV-S-SPS-------TSK
XP_009370612.1/1-224 --------------GHSRAGGSLLTVAFQ------------ILV-S-TPS-------TSK
XP_012845089.1/1-228 -------------TSSSTPSGSLLTVAFQ------------ILV-C-CSS-------RSK
XP_012845088.1/1-228 -------------TSSSTPSGSLLTVAFQ------------ILV-C-CSS-------RSK
XP_012845087.1/1-228 -------------TSSSTPSGSLLTVAFQ------------ILV-C-CSS-------RSK
XP_011085930.1/1-227 -------------SSSNSSSGSLLTVAFQ------------ILV-C-HST-------LTK
XP_020551334.1/1-222 -------------SSSNSSSGSLLTVAFQ------------ILV-C-HST-------LTK
XP_021297998.1/1-224 -----------------RSSGSLLTVAFQ------------IMA-C-SPS-------SSM
XP_017974480.1/1-224 -----------------RSSGSLLTVAYQ------------IMA-C-SPS-------SSM
XP_024448785.1/1-229 -------------STGADSGGSLLTVAFQ------------ILV-A-GPNVT-----SST
XP_024455385.1/1-229 -------------STGADSGGSLLTVAFQ------------ILV-A-GPNVT-----SST
XP_011005937.1/1-269 -------------STGADSGGSLLTVAFQ------------ILV-A-GPNAT-----SST
XP_011038413.1/1-229 -------------YTGSNSGGSLLTIAFQ------------ILV-S-GPNSS-----SSA
XP_018731153.1/1-226 ---------------SSKPAGTLLTVAFQ------------ILV-S-SHS-------GPE
XP_024190515.1/1-228 ----------------AKSGGSLLTVAFQ------------ILV-P-FPS-------PSQ
XP_012074722.2/1-231 ----------------GGGGGSLVTIAFQ------------ILG-S-NPLTSSASSSTPT
XP_010089514.2/1-229 --------SSSGTSGSSSSGGSLLTVAFQ------------ILV-------------SCK
XP_024949330.1/1-235 --------TGFGTGTGIGTGGSLLTVAFQ------------ILV-S-SPS-------SSK
XP_006421085.1/1-235 --------TGFGTGTGIGTGGSLLTVAFQ------------ILV-S-SPS-------SSK
XP_002268272.1/1-229 --------------IM-GSGGSLLTVAFQ------------VLV-C-GPAA------PQQ
XP_004140733.2/1-228 --------------LGRP-SGSLLTIAFQ------------ILV-S-SVS-------SSK
XP_008457114.1/1-228 --------------LGRP-SGSLLTIAFQ------------ILV-S-SVS-------SSK
XP_008457113.1/1-229 --------------LGRP-SGSLLTIAFQ------------ILV-S-SVS-------SSK
XP_011651208.1/1-229 --------------LGRP-SGSLLTIAFQ------------ILV-S-SVS-------SSK
XP_022143367.1/1-228 --------------LGRP-SGSLLTVAFQ------------ILV-S-SVS-------SSR
XP_022935490.1/1-228 --------------SGRP-SGSLLTVAFQ------------ILV-S-SVS-------SSK
XP_022984101.1/1-228 --------------SGRP-SGSLLTVAFQ------------ILV-S-SVS-------SSK
XP_022935488.1/1-229 --------------SGRP-SGSLLTVAFQ------------ILV-S-SVS-------SSK
XP_022984098.1/1-229 --------------SGRP-SGSLLTVAFQ------------ILV-S-SVS-------SSK
XP_022984102.1/1-229 --------------SGRP-SGSLLTVAFQ------------ILV-S-SVS-------SSK
XP_015579295.1/1-234 --------------AGRT-GGSLLTVAFQ------------ILV-S-TPTSTSS-SFSTK
XP_002526595.2/1-234 --------------AGRT-GGSLLTVAFQ------------ILV-S-TPTSTSS-SFSTK
XP_015579294.1/1-234 --------------AGRT-GGSLLTVAFQ------------ILV-S-TPTSTSS-SFSTK
XP_021643520.1/1-232 --------------PGRSSGGSLLTVAFQ------------ILV-S-TPTSSS----SSK
XP_021605433.1/1-231 --------------AGRSSGGSLLTVAFQ------------ILV-S-TPCST-----SSK
XP_023739098.1/1-228 ------------S--AASTAGSLVTLVLQ------------MVV-S-SQP--------GE
XP_024966239.1/1-227 ------------G--QNPTAGSLVTLMVQ------------SVI-G-GPA--------WG
XP_025813558.1/1-232 ------------GPLGKISPGCVVAVAFQ------------ILV-S-SLP--------SS
XP_020150479.1/1-234 ------------T---PMAAGCIVSVAFQ------------ILI-S-SLP--------SS
XP_003564122.2/1-224 ------------G---GVLAGCVVTVAFQ------------ILI-S-SLP--------AC
XP_006585049.2/1-225 ------------SRQVV-SKGSLVTLMLQ------------VLA-S-S-------TPSMD
XP_007143783.1/1-226 ------------SGEVGKSGGSLVTLMFQ------------ILA-S-S-------PSRMS
XP_007158859.1/1-226 ------------SGEVGKSGGSLVTLMFQ------------ILA-S-S-------PSRMS
XP_020230815.1/1-227 ---------SSSGNGILRTRGSLLTVAFQ------------ILM-N-S----------PT
XP_006586851.1/1-227 ---------SSSGNGDVRTRGSLLTVAFQ------------ILM-N-S----------SP
XP_021888058.1/1-234 ---------ADTSASIRSG-GSLLTVIFQ------------ATV-S-S-------PTTTK
XP_020886111.1/1-226 -------------------GGTLLTVAFQ------------ILV-S-G------KAARSR
XP_020886110.1/1-228 -------------------GGTLLTVAFQ------------ILV-S-G------KAARSR
XP_011469296.1/1-224 -----------SYTNPNRSGGSLLTVAFQ------------ILV-P--------FSSHSQ
XP_009367128.2/1-218 -----------------GTCGSLLTVAFH------------ILV-S--------SPTTSK
XP_008348673.1/1-198 -----------------DASGSLLTVAFH------------ILV-S--------IGRSRK
XP_012832506.1/1-227 --------NGASTSSNTGSGGSLLTVAFQ------------ILI-C--------HSQLTS
XP_012832508.1/1-227 --------NGASTSSNTGSGGSLLTVAFQ------------ILI-C--------HSQLTS
XP_012832516.1/1-227 --------NGASTSSNTGSGGSLLTVAFQ------------ILI-C--------HSQLTT
XP_012832518.1/1-227 --------NGASTSSNTGSGGSLLTVAFQ------------ILI-C--------HSQLTT
XP_012835569.1/1-227 --------NGASTSSNTGSGGSLLTVAFQ------------ILI-C--------HSQLTT
XP_012835571.1/1-226 --------NGASTSSNTGSGGSLLTVAFQ------------ILI-C--------HSQLTT
XP_019172066.1/1-226 -----------TSSSKKGQGGSLLTVAFQ------------ILI-S--------NGSLSK
XP_009631364.2/1-226 -------------SNGILQTGSLLTVAFQ------------ILI-S-ANNN---NNPMSK
XP_016435660.1/1-229 -------------SNGISQTGSLLTVAFQ------------ILV-S-ANNNNNNNNLISK
XP_009771801.1/1-226 -------------SNGISQTGSLLTVAFQ------------ILV-S-ANNN---NNSISK
XP_016493115.1/1-226 -------------SNGISQTGSLLTVAFQ------------ILV-S-ANNN---NNSISK
XP_019248352.1/1-226 -------------GNGISQTGSLLTVAFQ------------ILV-S-ANTN---TNSISK
XP_025691463.1/1-225 --------FGCALPESRHYGGSILTVTVQ------------VLV-S-N-------QNAVG
XP_020995109.1/1-225 --------FGCALPESRHYGGSILTVTVQ------------VLV-S-N-------QNAVG
XP_016190415.2/1-225 --------FGCALPESRHYGGSILTVTVQ------------VLV-S-N-------QNAVG
XP_004486568.1/1-232 -------TFNNGNSSVDGSGGTLLTLAYQ------------ILT-S-S------TNGIDK
XP_024632687.1/1-226 --------FGASN---NIEDGSLLTLAAQ------------ILS-T-S------PHEIDQ
XP_020977993.1/1-241 -----LGGVGSYGYQHRSEGGSLLTVAFQ------------ILV-N-T------ITCGPN
XP_025647253.1/1-241 -----LGGVGSYGYQHRSEGGSLLTVAFQ------------ILV-N-T------ITCGPN
XP_020997095.1/1-240 -----LGGVGSYGYQHRSEGGSLLTVAFQ------------ILV-N-T------ITCGPN
XP_025692389.1/1-228 -----L------------EGGSLLTVAFQ------------ILV-N-T------ITCGPN
XP_019418389.1/1-226 -------ASS--SRYAER-SGSLLTIAFQ------------TLV-C-S-------LPGIV
XP_007147460.1/1-227 -------AFD--CSDGERLGGSLLTVAFQ------------ILA-S---------ADGRN
XP_014517364.1/1-227 -------AFD--GADTERSGGSLLTVAFQ------------ILA-S---------ADGRN
XP_017435438.1/1-226 -------AFD--GADTERLGGSLLTVAFQ------------ILA-S---------ADGRN
XP_017435439.1/1-217 -------AFD--GADTERLGGSLLTVAFQ------------ILA-S---------ADGRN
XP_025981664.1/1-227 -------AFN--SSDIERLGGSLLTVAFQ------------ILA-S-S-------PDGIN
XP_006586853.1/1-227 -------AFN--SSDIERLGGSLLTVAFQ------------ILA-S-S-------PDGIN
XP_020230831.1/1-226 -------AFN--DVNIERIGGSLLTVAFQ------------ILA-S-S-------PDGIN
XP_004489663.1/1-217 -----------NS--EGFKGGSLLTLAYE------------ILS-C-SSE------RTTN
XP_018489833.1/1-227 ----------------SENGGTVLTLAFQ------------ILI-T-DKN------SKIK
XP_013584487.1/1-219 --------------------GTLLMLAFQ------------ILN-S-DEN------SKTR
XP_009130351.1/1-219 --------------------GTLLMLAFQ------------ILN-S-DEN------SKTR
XP_013747906.2/1-219 --------------------GTLLMLAFQ------------ILN-S-DEN------SKTR
XP_013747661.1/1-219 --------------------GTLLMLAFQ------------ILN-S-DEN------SKTR
XP_013619678.1/1-219 --------------------GTVLMLAFQ------------ILI-S-DEN------SKTR
XP_022551084.1/1-219 --------------------GTVLMLAFQ------------ILI-S-DEN------SKTR
XP_009126392.1/1-219 --------------------GTVLMLAFQ------------ILI-S-DEN------SKTR
XP_018437612.1/1-222 -----------------AEGGTILTVVFQ------------ILV-S-AED------NRTR
XP_013591191.1/1-225 -----------------EEGGTILTVVFQ------------ILV-S-E-D------KRIS
XP_022567005.1/1-225 -----------------EEGGTILTVVFQ------------ILV-S-E-D------KRIS
XP_022547796.1/1-225 -----------------EEGGTILTVVFQ------------ILV-S-E-D------RRIR
XP_009119205.1/1-225 -----------------EEGGTILTVVFQ------------ILV-S-E-D------RRIR
XP_009119199.1/1-225 -----------------EEGGTILTVVFQ------------ILV-S-E-D------RRIR
XP_006408322.1/1-232 ---------EDGG--VGHDGGTLLTVAFQ------------ILV-S-GKI------TRSR
XP_023642295.1/1-229 -----------------EDGGTLLTVAFQ------------ILV-S-GKT------TSSI
XP_023642294.1/1-229 -----------------EDGGTLLTVAFQ------------ILV-S-GKT------TSSI
NP_186976.2/1-228    -----------------EDGGTLLTVAFQ------------ILV-S-GKA------NRSR
XP_010507998.1/1-229 -----------------EDGGTLLTLAFQ------------ILV-S-GNT------NRAR
XP_010485661.1/1-229 -----------------EDGGTLLTLAFQ------------ILV-S-GNT------NRAR
XP_010450732.1/1-230 -----------------EDGGTLLTLAFQ------------ILV-S-GKT------SRSR
XP_010463776.1/1-230 -----------------EDGGTLLTLAFQ------------ILV-S-GRT------SRSR
XP_010463774.1/1-230 -----------------EDGGTLLTLAFQ------------ILV-S-GRT------SRSR
XP_019092960.1/1-229 -----------------EDGETLLTLAFQ------------ILV-S-GKT------STSR
XP_022551077.1/1-228 ---------DLED--GSDYSTSLLTVAFQ------------ILV-S-GPT------LSGD
XP_013619236.1/1-228 ---------DLED--GSDYSTSLLTVAFQ------------ILV-S-GPT------LSGD
XP_009126177.1/1-226 ---------ELED--GSDYSKSLLTVAFQ------------ILV-S-GPT------LSGD
XP_013652421.1/1-226 ---------ELED--GSDYSKSLLTVAFQ------------ILV-S-GPT------LSGD
XP_013652420.1/1-226 ---------ELED--GSDYSKSLLTVAFQ------------ILV-S-GPT------LSGD
XP_006400265.1/1-228 ---------ELDG--GRDYCKTLLTVAFQ------------ILV-C-GPN------LSAD
XP_013590801.1/1-226 ---------ELGG--GRDNCKTLLTVAFQ------------ILV-T-GPT------ISEE
XP_013651910.1/1-222 ---------ELGG--GRDNCKTLLTVAFQ------------ILV-T-GPT------IYEE
XP_009103958.1/1-222 ---------ELDG--GCDNCKTLLTVAFQ------------ILV-T-GPT------IYEE
XP_018444485.1/1-226 ---------ELDG--GLDDCKTLLTVAFQ------------ILA-T-GST------NFDE
XP_009121397.1/1-226 ---------ELDG--GLDNCKTLLTVAFQ------------ILV-T-GPA------MFEE
XP_013659428.1/1-226 ---------ELDG--GLDNCKTLLTVAFQ------------ILV-T-GPA------MFEE
XP_022565333.1/1-226 ---------ELDG--GLDNCKTLLTVAFQ------------ILV-T-GPA------MFEE
XP_013610327.1/1-226 ---------ELDG--GLDNCKTLLTVAFQ------------ILV-T-GPT------MFEE
XP_006286655.1/1-225 ---------EQDG--GSS--STLLTVAFQ------------ILV-S-DPR------YSTN
XP_010492677.1/1-224 ---------DPDG--GS---STLLTVAFQ------------ILV-C-DPD------NCTN
XP_010420445.1/1-224 ---------DPDG--GS---STLLTVAFQ------------ILV-C-DPN------HSAA
XP_010453918.1/1-224 ---------DPDG--GS---STLLTVAFQ------------ILV-C-DPN------YSAA
XP_006303739.1/1-224 ---------EQDG--RC---MTLLTLAFQ------------ILV-S-GSI------SSTE
XP_020876074.1/1-222 ---------EIEG--GS---STLLTVAFQ------------ILV-S-GPS------YSPD
NP_197234.1/1-222    ---------EAEG--GS---YTLLTVAFQ------------ILV-S-GPS------YSPD
NP_174724.1/1-221    ---------EVDG--GS---MTLLTLAFQ------------IFV-T-GPS------YYTD
XP_020869876.1/1-222 ---------ELNG--GR---MTLLTVAFQ------------ILV-S-GPS------YSTH
XP_019091098.1/1-222 ---------ELDS--ER---MTLLTVAFQ------------ILV-S-GSS------QSTN
XP_010478910.1/1-223 ---------ELDS--GR---MTLLTVAFQ------------ILV-S-GSS------HSID
XP_010501781.1/1-221 ---------ELDT--GR---MTLLTVAFQ------------ILV-S-GSS------HSID
XP_015089790.1/1-222 -------------NNGSEKDGSILTVAFQ------------KLI-C-TN----NNP-ISQ
XP_015089784.1/1-222 -------------NNGSEKDGSILTVAFQ------------KLI-C-AN----NNP-VSQ
XP_016549855.1/1-224 -------------NTGNAQDGSILTVAFQ------------ILI-S-GD----NYP-MNQ
XP_015057403.1/1-221 -------------NTQNAQNGSILTVTFQ------------ILI-C-DN----TNISISQ
XP_019066399.1/1-221 -------------NTENAQNGSILTVTFQ------------ILI-C-DN----NNISISQ
XP_015166059.1/1-216 -------------STANAENGSILTVTFQ------------ILI-S-GH----NNP-TSQ
XP_019071012.2/1-216 -------------STANAENGSILTVTFQ------------ILV-S-GH----NNP-TSQ
XP_015086709.1/1-216 -------------STANAENGSILTVTFQ------------ILI-S-GH----NNP-TSQ
XP_015087432.1/1-219 -------------GTANAQNGSILTVAFQ------------ILI-C-GD-----NP-TSK
XP_004247342.2/1-219 -------------NTANAQNGSILTVAFQ------------ILI-C-GD-----NP-TSK
XP_025888717.1/1-219 -------------NTANAQNGSILTVAFQ------------ILI-C-GD-----NP-TSK
XP_015087599.1/1-221 -------------NTANAQNGSILTVVFQ------------ILI-C-DN----NNP-TSK
XP_019071156.1/1-221 -------------NTANAQNGSILTVVFQ------------ILI-C-DN----NNP-TSK
XP_019070762.2/1-220 -------------STPNAQNGSILTVTFQ------------IMI-C-GN----NNP-TSR
XP_015087071.1/1-220 -------------NTANAQNGSILTVTFQ------------IMI-C-GN----NNP-TSR
XP_025888845.1/1-217 -------------NTASAQNGSILTVTFQ------------IMI-C-GN----NNP-TSR
XP_015170367.1/1-232 -------------NNGNAQNGSILTIAFQ------------ILI-I-GHP---NDNSISQ
XP_025886268.1/1-232 -------------ITKNAQNGSILTMAFQ------------ILI-C--HA---DNASISL
XP_015072491.1/1-224 -------------ITKNAQNGSILTMTFQ------------ILI-C--HA---NNASISL
XP_010327374.2/1-219 -------------NNENVKNGSILTVAFQ------------KLN-F---A---NN-SISQ
XP_015089749.1/1-219 -------------NNENAKNGSILTVAFQ------------KLI-F---A---NN-SISQ
XP_015170971.1/1-209 -------------NNGNVQNGSILTVALQ------------KLI-C---A---DNNLISQ
XP_025887726.1/1-223 -------------NNGNVQNGSILTVALQ------------ELI-C---A---NNHLISQ
XP_006586358.1/1-227 ---------QSGQ--VGQSRGSLVTLMFQ------------LLA-S-ST-------SKID
XP_014617351.1/1-228 ---------SSSGNGDVRTRGSLLTVAFQ------------ILM-N-SS-------PTMM
XP_007147461.1/1-227 ---------WSSGSDDTRTHSTLLTVAFQ------------IMM-N-GP-------TTMI
XP_017435408.1/1-226 ---------WSSGSDDTRRHGTLLTVTFQ------------ILM-N-GP-------TTMV
XP_017215828.1/1-223 --------G--------TEGGSLLTIAFQ------------ILT-S-NS-------PT-E
XP_012856110.1/1-229 --------TRQDE--KSTDGGSLLTVAFQ------------ILI-S-NA-------TS-A
XP_023738891.1/1-226 --------KGQDQ--SLEEGGSLLTVGFQ------------ILT-T-DD------STG-G
XP_022017964.1/1-227 --------KADKQ--SWE-NGSLLTVGFQ------------ILT-S-DS-------PT-S
XP_022154996.1/1-226 --------SKEEK---GAEGGSLLTMASQ------------ILA-S-AS-------QT-A
XP_008462169.1/1-225 --------SKEER---ET-GGSLLTVASQ------------ILV-S-PS-------PT-A
XP_004141841.1/1-226 --------SKEER---ETRGGSLLTVASQ------------ILV-S-PS-------PT-A
XP_020098514.1/1-228 --------RKEEK--SSE-GGSLLTVAYQ------------FHA-N-SS-------PL-A
XP_020098513.1/1-228 --------RKEEK--SSE-GGSLLTVAYQ------------FHA-N-SS-------PL-A
XP_020263950.1/1-229 --------RPEER--STE-GGSLLTVAFQ------------ILA-S-AS-------PS-A
XP_020263948.1/1-255 --------RPEER--STE-GGSLLTVAFQ------------ILA-S-AS-------PS-A
XP_009415234.2/1-232 --------RPQER--TME-GGSLLTVAFQ------------ILA-D-AS-------PV-A
XP_008789333.1/1-230 --------KPEEK--TTEGGGSLLTVGVQ------------ILA-N-TS-------PL-A
XP_010920420.1/1-235 --------KPEEK--ATDGGGSLLTVGVQ------------VLA-N-TS-------PL-A
XP_010920411.1/1-235 --------KPEEK--ATDGGGSLLTVGVQ------------VLA-N-TS-------PL-A
XP_010920404.1/1-235 --------KPEEK--ATDGGGSLLTVGVQ------------VLA-N-TS-------PL-A
XP_010521515.1/1-219 --------SRQDEK--NSEGGSLLTVALQ------------SPV-V-HA------SIS-A
XP_013652056.1/1-227 --------AQEADR--NTQGGSLLTMALQ------------TLV-N--Q------SPA-A
XP_009104428.1/1-227 --------AQEADR--NTQGGSLLTMALQ------------TLV-N--Q------SPA-A
XP_018445476.1/1-226 --------AQE-DR--NTQGGCLLTLALQ------------TLI-N--Q------SPA-A
XP_013590784.1/1-226 --------AQE-DK--NTQGGSLLTLALQ------------TLV-N--Q------SPA-A
XP_013680822.1/1-226 --------AQE-DR--NTQGGSLLTLALQ------------TLV-N--Q------SPA-A
XP_006389720.2/1-226 --------TR-EDR--NTQGGSLLTLALQ------------TLI-N--P------SPA-A
XP_023642973.1/1-226 --------TQ-DDR--NSQGGSLLTVALQ------------TLI-N--P------SPA-T
XP_020891640.1/1-226 --------TQ-DDR--NSQGGSLLTLALQ------------TLI-N--P------SPA-A
NP_001320267.1/1-226 --------TQ-DDR--NSQGGSLLTLALQ------------TLI-N--P------SPA-A
NP_565223.1/1-226    --------TQ-DDR--NSQGGSLLTLALQ------------TLI-N--P------SPA-A
NP_001185443.1/1-226 --------TQ-DDR--NSQGGSLLTLALQ------------TLI-N--P------SPA-A
XP_010473021.1/1-226 --------TQ-DDR--NSQGGSLLTLALQ------------TLI-N--P------SPA-A
XP_010430051.1/1-226 --------TQ-DDR--NSQGGSLLTLALQ------------TLI-N--P------SPA-A
XP_025617667.1/1-241 ---MMQQ-QQEENMNTEEGGGSLLTMAFQ------------VIT-N-NNS--NGSPSS-A
XP_015938429.1/1-242 ---MMQQQQQEENMNTEEGGGSLLTMAFQ------------VIT-N-NNS--NGSPSS-A
XP_016176999.1/1-241 ---TMMQQQQQENMNTVEG-GSLLTMAFQ------------VIT-N-NNS--NGSPSS-A
XP_021862846.1/1-225 --------RQEEEKS--SEGGSLLTIALQ------------VVT-N-SS-------PA-A
XP_022979290.1/1-231 --------RQKDDKTADTNGGVLLTAAVQ------------ILT-D-AS-------PS-A
XP_022955725.1/1-233 --------RQKDDKTADTNGGVLLTVAVQ------------ILT-D-AS-------PS-A
XP_022998610.1/1-231 --------RQKDHKSSDTHGGALLTAAVQ------------IVT-D-AS-------PS-A
XP_022998611.1/1-227 --------RQKDHKSSDTHGGALLTAAVQ------------IVT-D-AS-------PS-A
XP_023523626.1/1-229 --------RQKDHKSSDTHGGALLTAAVQ------------IVT-D-AS-------PS-A
XP_022948296.1/1-231 --------RQKDHKSSDTHGGALLTAAVQ------------IVT-D-AS-------PS-A
XP_023523628.1/1-225 --------RQKDHKSSDTHGGALLTAAVQ------------IVT-D-AS-------PS-A
XP_022948295.1/1-227 --------RQKDHKSSDTHGGALLTAAVQ------------IVT-D-AS-------PS-A
XP_010249693.1/1-228 --------RPE--E-KTTEGGSLLTIAFQ------------ILA-N-TS-------PT-A
XP_010254081.1/1-230 --------KPDDEE-KTMEGGSLLTVAFQ------------ILA-K-SS-------PT-A
XP_003529763.1/1-227 --------RQEEKY--T-EGGSLFTMAFQ------------ILA-N-PS-------PT-T
XP_003533048.1/1-226 --------RQEEKY--T-EGGSLFTMAFQ------------ILV-N-PS-------PT-V
XP_014634625.1/1-226 --------RQEEKY--T-EGGSLFTMAFQ------------ILV-N-PS-------PT-V
XP_007150635.1/1-226 --------RQEEKY--T-EGGSLFTMAFQ------------ILT-N-TS-------PT-A
XP_014498860.1/1-226 --------RQEEKY--T-EGGSLFTMAFQ------------ILT-N-AS-------PT-A
XP_017425661.1/1-226 --------RQEEKY--T-EGGSLFTMAFQ------------ILT-N-AS-------PT-A
XP_020207725.1/1-226 --------RQEEKN--T-EGGSLFTMAFQ------------ILT-N-AS-------PT-A
XP_003546423.2/1-226 --------RQEEKN--T-EGGSLFTMAFQ------------ILT-N-AS-------PA-A
XP_003543638.1/1-226 --------RQEEKN--T-EGGSLFTMAFQ------------ILT-N-AS-------PT-A
XP_019457980.1/1-226 --------KQDEKN--T-EGGSLFTIALQ------------ILT-N-VS-------PT-A
XP_003597736.1/1-226 --------RREEKN--T-EGGSLFTIAFQ------------ILT-N-AS-------PT-A
XP_004486751.1/1-226 --------RQEEKN--T-EGGSLFTVAFQ------------ILT-N-AS-------PT-A
XP_016169615.1/1-226 --------RQEEKN--T-EGGSLFTIAFQ------------ILT-N-AS-------PT-A
XP_025611006.1/1-226 --------RQEEKN--T-EGGSLFTIAFQ------------ILT-N-AS-------PT-A
XP_020982478.1/1-227 --------RQEEKN--TAEGGSLFTIAFQ------------ILT-N-AS-------PT-A
XP_021892835.1/1-225 --------RPE-KS-N--EGGSLLTIAFQ------------ILT-N-TS-------PN-A
XP_012442963.1/1-227 --------RHE-KS-NDTEGGSLLTVAFQ------------ILT-N-SS-------PT-A
NP_001314068.1/1-227 --------RHE-KS-NDTEGGSLLTVAFQ------------ILT-N-SS-------PT-A
XP_016684379.1/1-227 --------RHE-KS-NDTEGGSLLTVAFQ------------ILT-T-SS-------PT-A
XP_016730082.1/1-227 --------RHE-KS-NDTEGGSLLTVAFQ------------ILT-T-SS-------PT-A
NP_001316938.1/1-227 --------RHE-KS-NDTEGGSLLTVAFQ------------ILT-N-SS-------PT-A
XP_017606636.1/1-227 --------RHE-KS-NDTEGGSLLTVAFQ------------ILT-N-SS-------PT-A
XP_022714992.1/1-229 --------KHE-KS-NDTEGGSLLTIAFQ------------ILT-N-SS-------PT-A
XP_022721436.1/1-227 --------RHE-KS-NDTEGGSLLTIAFQ------------ILT-N-SS-------PT-A
XP_022721435.1/1-227 --------RHE-KS-NDTEGGSLLTIAFQ------------ILT-N-SS-------PT-A
XP_007024189.1/1-227 --------RQE-KS-NDTEGGSLLTIAFQ------------ILT-N-SS-------PT-A
XP_021298731.1/1-227 --------RQE-KS-NDTEGGSLLTIAFQ------------ILT-N-SS-------PT-A
XP_004302894.1/1-228 --------RQEDRG-SDSEGGTLLTVAFQ------------VLT-N-TS-------PT-A
XP_011466687.1/1-235 --------RQEDRG-SDSEGGTLLTVAFQ------------VLT-N-TS-------PT-A
XP_024180978.1/1-228 --------RQEDRG-SDSEGGTLLTVAFQ------------VLT-N-TS-------PT-A
XP_024180977.1/1-235 --------RQEDRG-SDSEGGTLLTVAFQ------------VLT-N-TS-------PT-A
XP_009353605.1/1-226 --------RQEDRS---SEGGTLLTAAFQ------------VLT-N-SS-------PT-A
XP_008380909.1/1-226 --------RQEDRS---SEGGTLLTAAFQ------------VLT-N-SS-------PT-G
NP_001280927.1/1-227 --------SQEDRS---SEGGTLLTAAFQ------------VLT-N-SS-------TTAN
XP_009338062.1/1-226 --------SQEDRS---SEGGTLLTAAFQ------------VLT-N-SS-------PT-A
XP_009338046.1/1-226 --------SQEDRS---SEGGTLLTAAFQ------------VLT-N-SS-------PT-A
XP_021829136.1/1-226 --------RQEDRS---SEGGTLLTAAFQ------------VLT-N-SS-------PT-A
XP_008228560.1/1-226 --------RQEDRS---SEGGTLLTAAFQ------------VLT-N-SS-------PT-A
XP_007217160.1/1-226 --------KQEDRS---SEGGTLLTAAFQ------------VLT-N-SS-------PT-A
XP_022891611.1/1-228 --------KPEEKS---TEGGSLLTIAFQ------------ILT-S-NS-------PT-A
XP_002304207.2/1-228 --------RQEEKS---TEGGSLLTIAFQ------------ILT-N-TS-------PT-A
XP_011010522.1/1-228 --------RQEEKS---TEGGSLLTIAFQ------------ILT-N-TS-------PT-A
XP_011009828.1/1-227 --------RQEERS---TEGGCLLTIAFQ------------ILT-N-TS-------PT-A
XP_024462179.1/1-228 --------RQEERS---TEGGCLLTIAFQ------------ILT-N-TS-------PT-A
XP_018837483.1/1-226 --------RQEEKS---TEAGSLLTIAFQ------------ILT-S-TS-------TT-A
XP_018856342.1/1-226 --------RQDEKS---TEAGSLLTIAFQ------------ILT-S-TS-------TT-A
XP_018856994.1/1-226 --------RQDEKS---TEAGSLLTIAFQ------------ILT-S-TS-------TT-A
XP_018858360.1/1-226 --------RQDEKS---TEAGSLLTIAFQ------------ILT-S-TS-------TT-A
XP_015867693.1/1-226 --------RREEKT---AEGGSLLTVAFQ------------ILT-N-TS-------PT-A
XP_010062490.1/1-226 --------RHEEKS---SEGGSLLTIAFQ------------ILT-N-TS-------PT-A
XP_010098082.1/1-228 --------RPEEKT---SEGGSLLTVAFQ------------ILT-N-TS-------PT-A
XP_002284502.1/1-228 --------RPEEKS---TEGGSLLTIAFQ------------VLT-N-TS-------PT-A
XP_023875609.1/1-226 --------RQEEKS---TEGGSLLTIAIQ------------ILT-N-TS-------PT-A
XP_002516023.1/1-226 --------RREEKR---TEGGSLLTVAFQ------------ILT-N-TS-------PT-A
XP_021652755.1/1-228 --------RQEEKG---TEGGSLLTMAVQ------------IIT-S-TS-------PT-A
XP_021654904.1/1-226 --------RQEEKG---TEGGSLLTMAFQ------------ILT-N-TS-------PT-A
XP_021611342.1/1-229 --------RQEEKG---TEGGSLLTIAFQ------------ILT-N-TS-------PT-A
XP_012073376.1/1-226 --------RQEEKG---TEGGSLLTMAFQ------------ILT-N-TS-------PT-A
XP_006465622.1/1-226 --------RQEEKI---TEGGSLLSIAFQ------------ILT-N-NS-------PA-A
XP_006426950.1/1-226 --------RQEEKI---TEGGSLLSIAFQ------------ILT-N-NS-------PA-A
XP_009392419.1/1-229 --------RPEERT-MEG--GSLLTIAFQ------------ILA-N-SS-------PM-A
XP_020518333.1/1-227 --------RPKNRA-SEG--GSLLTIAFQ------------ILA-N-TS-------PS-A
XP_018629167.1/1-227 --------RPEEKC-TGG--GSLLTVLYQ------------ILT-S-NS-------PA-V
XP_009611250.1/1-227 --------RPEEKC-TGG--GSLLTVLYQ------------ILT-S-NS-------PA-V
XP_009611249.1/1-227 --------RPEEKC-TGG--GSLLTVLYQ------------ILT-S-NS-------PA-V
XP_009611248.1/1-227 --------RPEEKC-TGG--GSLLTVLYQ------------ILT-S-NS-------PA-V
XP_016479156.1/1-227 --------RPEEKC-TGG--GSLLTVLYQ------------ILT-S-NS-------PA-V
XP_016479155.1/1-227 --------RPEEKC-TGG--GSLLTVLYQ------------ILT-S-NS-------PA-V
XP_016479154.1/1-227 --------RPEEKC-TGG--GSLLTVLYQ------------ILT-S-NS-------PA-V
XP_009778213.1/1-229 --------KPEDRS-SEG--GSLLTVAFQ------------ILT-S-NS-------PT-A
XP_016501674.1/1-229 --------KPEDRS-SEG--GSLLTVAFQ------------ILT-S-NS-------PT-A
XP_019224885.1/1-229 --------KPEDRS-SEG--GSLLTIAFQ------------ILT-S-NS-------PT-A
XP_019224886.1/1-229 --------KPEDRS-SEG--GSLLTIAFQ------------ILT-S-NS-------PT-A
XP_009592772.1/1-229 --------KPEDRS-SEG--GSLLTIAFQ------------ILT-S-NS-------PT-A
XP_016496403.1/1-229 --------KPEDRS-SEG--GSLLTIAFQ------------ILT-S-NS-------PT-A
XP_016562690.1/1-228 --------KPEDRS-SEG--GSLLTVAFQ------------ILT-S-SS-------PT-A
XP_006343080.1/1-229 --------RPEDRS-SEG--GSLLTVAFQ------------ILT-S-NS-------PT-T
XP_004235676.1/1-229 --------RPEDRS-SEG--GSLLTVAFQ------------ILT-S-NS-------TT-A
XP_020227921.1/1-228 --------RQEEKY-TEG--GSLLTMAFQ------------ILA-N-PS-------TT-T
XP_007135673.1/1-226 --------RQEEKG-TEG--GSLFTMLFQ------------MLV-D-AS-------PN-G
XP_014515030.1/1-226 --------KQDEKG-TEG--GSLFTMLFQ------------MLV-N-AS-------PT-A
XP_017407449.1/1-226 --------KQDEKG-TEG--GSLFTMLLQ------------MLV-N-AS-------PT-A
XP_010691342.1/1-224 --------VQEEKS-SEG--GCLLTIALQ------------VVT-N-SS-------PA-A
XP_021731998.1/1-222 --------RQEERS-SEG--GSLLTIALQ------------VVT-N-SS-------PA-A
XP_021736870.1/1-224 --------RQEERS-SEG--GSLLTIALQ------------VVT-N-SS-------PA-A
XP_024969955.1/1-229 --------KAQDKS-WEGLGGSLLTVGFQ------------ILT-S-NS-------PT-G
XP_024969954.1/1-229 --------KAQDKS-WEGLGGSLLTVGFQ------------ILT-S-NS-------PT-G
XP_024969953.1/1-229 --------KAQDKS-WEGLGGSLLTVGFQ------------ILT-S-NS-------PT-G
XP_021984354.1/1-227 --------KADDQS-WEY--GSLLTVGFQ------------ILT-S-DS-------PI-S
XP_017232370.1/1-228 --------RQEDKS-ADG--GSLLTIAFQ------------ILT-S-NS-------PS-E
XP_017216573.1/1-228 --------RSEDKS-TEG--GSLLTIAFQ------------ILT-T-NS-------PT-E
XP_017248777.1/1-227 --------RADDKS--TG--GSLLTIAFQ------------VLI-S-ES-------PT-A
XP_021627148.1/1-226 --------RQEEKG-TEG--GSLLTMAVQ------------ILT-N-TS-------PA-A
XP_021627146.1/1-226 --------RQEEKG-TEG--GSLLTMAVQ------------ILT-N-TS-------PA-A
XP_021627145.1/1-226 --------RQEEKG-TEG--GSLLTMAVQ------------ILT-N-TS-------PA-A
XP_019196965.1/1-228 --------KPEEKS-TEG--GSLLTIAFQ------------ILT-S-NS-------PT-A
XP_019196967.1/1-228 --------KPEEKS-TEG--GSLLTIAFQ------------ILT-S-NS-------PT-A
XP_022848789.1/1-228 --------RPEEKS-TEG--GSLLTIAFQ------------ILA-S-NS-------LT-A
XP_011072373.1/1-228 --------RPAEKC-TEG--GSLLTVVFQ------------ILI-S-NS-------PT-A
XP_021907286.1/1-215 --------------------RSLLTISFQ------------IQL-H-KI-------PT-A
XP_020885309.1/1-217 ----------------NEG-GSLLTVSFQ------------MLV-E-SG-------PS-G
XP_020885307.1/1-217 ----------------NEG-GSLLTVSFQ------------MLV-E-SG-------PS-G
NP_180796.2/1-218    ----------------KEG-GSLLTISFQ------------MLV-E-SG-------PE-A
XP_006296541.1/1-217 ----------------NAG-GSLLTVAYQ------------MLL-E-TV-------PT-D
XP_010510028.1/1-215 --------------------GSLLTVSFQ------------MLV-E-EV-------HM-G
XP_010414006.1/1-214 --------------------GSLLTVSFQ------------MLV-E-EV-------HS-G
XP_010469602.1/1-219 ----------------NGGGGSLLTVSFQ------------MLV-E-GV-------DL-G
XP_010417818.1/1-218 ----------------NGG-GSLLTVSLQ------------MLV-E-EV-------PL-G
XP_010510869.1/1-220 ----------------NGG-GSLLTVSLQ------------MLV-E-EV-------SL-A
XP_010510870.1/1-218 ----------------NGG-GSLLTVSLQ------------MLV-E-EV-------SL-A
XP_018484769.1/1-219 ----------------EG--GSLVSASFQ------------VLV-E-TD-------PL-A
XP_018484754.1/1-219 ----------------EG--GSLVSASFQ------------VLV-E-TD-------PL-A
XP_013634882.1/1-219 ----------------EG--GSLVSAAFQ------------VLA-E-KD-------PS-A
XP_018513778.1/1-219 ----------------EG--GSLVSTAFQ------------VLA-E-KD-------PS-T
XP_009141217.1/1-219 ----------------EG--GSLVSTAFQ------------VLA-E-KD-------PS-T
XP_013747219.1/1-219 ----------------EG--GSLVSTAFQ------------VLA-E-KD-------PS-T
XP_024003849.1/1-217 ----------------EG--GSLLSVAFQ------------ILV-E-SV-------HS-A
XP_018437853.1/1-211 ----------------AG--GTLVNVSFQ------------MLV-D-SS-------PS-G
XP_009143975.1/1-211 ----------------AG--GSLVNVAFQ------------MLV-D-SY-------PS-G
XP_009143974.1/1-211 ----------------AG--GSLVNVAFQ------------MLV-D-SY-------PS-G
XP_022575029.1/1-211 ----------------AG--GSLVNVAFQ------------MLV-D-SY-------PS-G
XP_013748684.1/1-211 ----------------AG--GSLVNVAFQ------------MLV-D-SY-------PS-G
XP_013694194.1/1-211 ----------------AG--GSLVNVAFQ------------MLV-D-SF-------PS-G
XP_013634261.1/1-211 ----------------AG--GSLVNVSFQ------------MLV-D-SS-------PS-G
XP_013741430.1/1-210 ----------------AG--GSLVNVSFQ------------MLV-D-SS-------PS-G
XP_017254373.1/1-221 -------------IVGVGSGGSLLTVAFQ------------MLN-D-AI-------PT-K
XP_017254240.1/1-222 -------------IIEVGSGGSLLTVAFQ------------IMV-H-SA-------LP-E
XP_017221582.1/1-224 -------------ILEVGSGGSLLTVAFQ------------IKI-R-SV-------PP-E
XP_017221584.1/1-224 -------------ILDVGSGGSLLTVAFQ------------IKI-R-SV-------PP-E
XP_015941575.1/1-215 ------------------GGGCLVTLAFQ------------ILI-E-SD-------PT-K
XP_025620358.1/1-215 ------------------GGGCLVTLAFQ------------ILI-E-SD-------PT-K
XP_016176493.1/1-215 ------------------GGGCLVTLAFQ------------ILI-E-SD-------PT-K
XP_003610661.2/1-228 -------------IMNVGSGGSLVTVAFQ------------IIV-D-SI-------PH-A
XP_004496853.1/1-220 ------------------GGGCLLTISFQ------------ILI-D-SN-------PN-A
XP_006353342.1/1-230 ----------GILENGGKINGCLLTMGFQ------------ILM-T-NP-------PT-G
XP_015070591.1/1-229 ----------GTLENGGKINGCLLTMGYQ------------VLM-S-NP-------PT-G
XP_019068356.1/1-229 ----------GTLENGGKMNGCLLTMGYQ------------VLM-S-NP-------PT-G
XP_004234441.1/1-229 ----------GTLENGGKMNGCLLTMGYQ------------VLM-S-NP-------PT-G
XP_024926606.1/1-217 ---------------ETAGCGSLLTVAFHVVDRASTLDFI--------------------
XP_024525400.1/1-231 ------------SILQNDATGTLLTVAVQ------------ILI-S-TL-------PS-A
XP_002965186.2/1-231 ------------SILQNDATGTLLTVAVQ------------ILI-S-TL-------PS-A
XP_002962272.2/1-231 ------------SILQNDATGTLLTVAVQ------------ILI-S-TL-------PS-A
XP_019441536.1/1-226 ------------SIVKVGPRGSLITVAFQ------------ILV-H-NK-------PT-G
XP_019441537.1/1-226 ------------SIVKVGPRGSLITVAFQ------------ILV-H-NK-------PT-G
XP_019441535.1/1-226 ------------SIVKVGPRGSLITVAFQ------------ILV-H-NK-------PT-G
XP_019451758.1/1-229 ------------YMLDDGSEGSLITVAFQ------------ILV-D-NN-------PT-S
XP_012850835.1/1-238 ------------TAAQVDSGGSLLTVAHQVSSPSFSLFFFVILV-D-SS-------PN-A
XP_020552079.1/1-225 ------------MAAEADSGGSLLTVALQ------------VLV-D-PS-------PN-A
XP_020552078.1/1-225 ------------MAAEADSGGSLLTVALQ------------VLV-D-PS-------PN-A
XP_020552080.1/1-223 ------------MAAEADSGGSLLTVALQ------------VLV-D-PS-------PN-A
XP_009408807.2/1-226 ------------GE-TDLGGGSLLTVAFQ------------ILV-F-SG-------PG-S
XP_006342404.1/1-225 ------------I---SDYSGSFLTISFQ------------ILV-H-NV-------PT-A
XP_025887998.1/1-218 ------------I---SDYSGSFLTIAFQ------------ILV-D-NV-------PT-A
XP_015081481.1/1-225 ------------I---SDYSGSFLTIAFQ------------ILV-D-TV-------PT-T
XP_022000230.1/1-232 --------------KAGGEGGSLLTVGFQ------------ILV-N-DL-------PS-S
XP_022000229.1/1-232 --------------KAGGEGGSLLTVGFQ------------ILV-N-DL-------PS-S
XP_022037790.1/1-222 --------------KAEGEGGSLLTVGFQ------------ILV-N-DL-------PS-S
XP_022037789.1/1-222 --------------KAEGEGGSLLTVGFQ------------ILV-N-DL-------PS-S
XP_024991925.1/1-226 --------------KAGSEGGSLLTVGFQ------------ILV-N-DL-------PS-S
XP_023760005.1/1-229 --------------KPGSEGGSLLTVGFQ------------ILV-N-DL-------PS-S
XP_023760001.1/1-229 --------------KPGSEGGSLLTVGFQ------------ILV-N-DL-------PS-S
XP_017251723.1/1-236 -----GGVPE-----DNTSSGSLLTVSFQ------------ILL-N-NL-------PM-A
XP_017243321.1/1-239 -----LEMMGKESGCDGVGGGCLLTVGFQ------------IMI-N-DF-------PA-S
XP_017243319.1/1-239 -----LEMMGKESGCDGVGGGCLLTVGFQ------------IMI-N-DF-------PA-S
XP_017243320.1/1-239 -----LEMMGKESGCDGVGGGCLLTVGFQ------------IMI-N-DF-------PA-S
XP_017255563.1/1-234 -----NEMVGKESSCQ--GGGSLLTVGFQ------------IMV-S-DL-------PA-A
XP_017255562.1/1-234 -----NEMVGKESSCQ--GGGSLLTVGFQ------------IMV-S-DL-------PA-A
XP_012852237.1/1-226 -----L------GGG--GGGQSLLTVVFQ------------MLV-S-NT-------PA-A
XP_012852238.1/1-226 -----L------GGG--GGGQSLLTVVFQ------------MLV-S-NT-------PA-A
XP_012852236.1/1-226 -----L------GGG--GGGQSLLTVVFQ------------MLV-S-NT-------PA-A
XP_012852235.1/1-226 -----L------GGG--GGGQSLLTVVFQ------------MLV-S-NT-------PA-A
XP_011076208.1/1-227 -----E------GHRGGGRSGSLLTVGFQ------------ILV-N-NL-------PA-A
XP_019200484.1/1-228 -----KGTLPKEGGPGNNSTGALLTVGFQ------------MLV-S-SL-------PG-A
XP_019200481.1/1-228 -----KGTLPKEGGPGNNSTGALLTVGFQ------------MLV-S-SL-------PG-A
XP_022879034.1/1-223 -----RGS-------DEGNSGSLLTIGFQ------------ILV-N-SS-------PA-A
XP_018623115.1/1-238 -----VGTSWEKD-NGFSGTGSLVTIGFQ------------VLV-T-SS-------PA-A
XP_018623116.1/1-238 -----VGTSWEKD-NGFSGTGSLVTIGFQ------------VLV-T-SS-------PA-A
XP_016449095.1/1-238 -----VGTSWEKD-NGFSGTGSLVTIGFQ------------VLV-N-SS-------PA-A
XP_009771835.1/1-238 -----VETSWEKD-NGFSGTGSLVTIGFQ------------VLV-N-SS-------PA-E
XP_009771833.1/1-238 -----VETSWEKD-NGFSGTGSLVTIGFQ------------VLV-N-SS-------PA-E
XP_019248454.1/1-225 -----IGTSWEKD-NGFSGTGSLVTIGFQ------------VLV-N-SS-------PA-E
XP_016564677.1/1-235 -----NGMLEKED-NGGRNNGSFLTVGYQ------------ILV-N-NL-------PG-G
XP_016564676.1/1-235 -----NGMLEKED-NGGRNNGSFLTVGYQ------------ILV-N-NL-------PG-G
XP_009606534.1/1-237 -----NGMLEKED-NGGSSNGSLLTLGFQ------------ILV-N-SL-------PA-A
XP_016457239.1/1-237 -----NGMLEKED-NGGSSNGSLLTLGFQ------------ILV-N-SL-------PA-A
XP_009606533.1/1-237 -----NGMLEKED-NGGSSNGSLLTLGFQ------------ILV-N-SL-------PA-A
XP_009606532.1/1-237 -----NGMLEKED-NGGSSNGSLLTLGFQ------------ILV-N-SL-------PA-A
XP_016457238.1/1-237 -----NGMLEKED-NGGSSNGSLLTLGFQ------------ILV-N-SL-------PA-A
XP_016468064.1/1-237 -----NGMLEKED-NGGSSNGSLLTLGFQ------------ILV-N-SL-------PA-A
XP_009774404.1/1-237 -----NGMLEKED-NGGSSNGSLLTLGFQ------------ILV-N-SL-------PA-A
XP_016468062.1/1-237 -----NGMLEKED-NGGSSNGSLLTLGFQ------------ILV-N-SL-------PA-A
XP_016468061.1/1-237 -----NGMLEKED-NGGSSNGSLLTLGFQ------------ILV-N-SL-------PA-A
XP_009774402.1/1-237 -----NGMLEKED-NGGSSNGSLLTLGFQ------------ILV-N-SL-------PA-A
XP_009774401.1/1-237 -----NGMLEKED-NGGSSNGSLLTLGFQ------------ILV-N-SL-------PA-A
XP_019251817.1/1-237 -----NGMLEKED-NGGSSNGSLLTLGFQ------------ILV-N-SL-------PA-A
XP_019251815.1/1-237 -----NGMLEKED-NGGSSNGSLLTLGFQ------------ILV-N-SL-------PA-A
XP_019251816.1/1-237 -----NGMLEKED-NGGSSNGSLLTLGFQ------------ILV-N-SL-------PA-A
XP_011470395.1/1-233 -----------EEGNGSDEGGCLLTVGFQ------------IVC-D-GG------QPT-S
XP_004309219.2/1-233 -----------EEGNGSDEGGCLLTVGFQ------------IVC-D-GG------QPT-S
XP_024180881.1/1-233 -----------GEGNGSDDGGCLLTVGFQ------------IVC-D-GG------QPT-S
XP_024180911.1/1-233 -----------GEGNGSDDGGCLLTVGFQ------------IVC-D-GG------QPT-S
XP_019450525.1/1-216 -----------------GSGGSLLTVGLQ------------ILL-S-TL------SNG-N
XP_019465255.1/1-230 -----SNGVGLGG---SESGGSLLTFGLQ------------ILS-S-NL------PND-N
XP_014502466.1/1-227 ------------DNNDG-GGSCLLTVGLQ------------MVQ-N-DN-------PS-A
XP_017438850.1/1-222 ------------DNNDGGGGSCLLTVGLQ------------MLQ-N-DN-------PS-A
XP_006599623.1/1-221 ------------DGSDG-GGSCLLTVGLQ------------MLP-N-GN------HQS-A
XP_006599622.1/1-221 ------------DGSDG-GGSCLLTVGLQ------------MLP-N-GN------HQS-A
XP_006599621.1/1-221 ------------DGSDG-GGSCLLTVGLQ------------MLP-N-GN------HQS-A
XP_004515730.1/1-240 -----NDGSGNGGSDNNDSGGCLLTAGIQ------------MLL-N-NL-------QT-S
XP_013442370.1/1-239 -----SDGS-SGNGEGGDNGGCLLTFGLQ------------MLL-N-NT-------PT-S
XP_004252035.1/1-225 ------------GTYNHGSGGSLLTVAFQ------------ILV-E-FV-------PV-A
XP_015060726.1/1-225 ------------GTYNHGSGGSLLTVAFQ------------IMV-E-FV-------PV-A
XP_006589608.1/1-229 ------------CEVVGSGRGCLLTVAFQ------------ILV-D-ST-------PT-A
XP_006589605.1/1-230 ------------CEVVGSGRGCLLTVAFQ------------ILV-D-ST-------PT-A
XP_006849468.1/1-231 ---------STSSSSSSGSSGSLITVAFQ------------ILV-S-SL-------PS-A
XP_020526173.1/1-231 ---------STSSSSSSGSSGSLITVAFQ------------ILV-S-SL-------PS-A
XP_020109515.1/1-228 ---------STSSNPMGASLGSLITVAFQ------------ILV-S-SL-------PS-S
NP_001310555.1/1-224 -------------AGQAPSAGSLVTVAFQ------------ILV-S-SL-------PS-A
XP_010544375.1/1-225 ---------M---KTPAAGCGSLLTVAFQ------------ILV-N-SL-------PT-A
NP_001314001.1/1-230 ------GPSMQPDIDENTSDGCILTVGFQ------------ILV-N-SV-------PT-A
XP_016732454.1/1-230 ------GPSMQPDIDENTSDGCILTVGFQ------------ILV-N-SV-------PT-A
XP_016732453.1/1-230 ------GPSMQPDIDENTSDGCILTVGFQ------------ILV-N-SV-------PT-A
XP_012484067.1/1-230 ------GPSMQPDIDENTSDGCILTVGFQ------------ILV-N-SV-------PT-A
XP_012484066.1/1-230 ------GPSMQPDIDENTSDGCILTVGFQ------------ILV-N-SV-------PT-A
XP_017631791.1/1-230 ------GPSMQPDIDENTSDGCILTVGFQ------------ILV-N-SV-------PT-A
XP_017631789.1/1-230 ------GPSMQPDIDENTSDGCILTVGFQ------------ILV-N-SV-------PT-A
XP_016728129.1/1-230 ------GPSMQPDIDENTSDGCILTVGFQ------------ILV-N-SV-------PT-A
XP_016728128.1/1-230 ------GPSMQPDIDENTSDGCILTVGFQ------------ILV-N-SV-------PT-A
XP_021671684.1/1-240 ------GTLIKRDSDGGDGGGSLLTVGFQ------------ILV-N-NL-------RT-A
XP_021671683.1/1-240 ------GTLIKRDSDGGDGGGSLLTVGFQ------------ILV-N-NL-------RT-A
XP_021671685.1/1-240 ------GTLIKRDSDGGDGGGSLLTVGFQ------------ILV-N-NL-------RT-A
XP_002318231.3/1-240 ------GNPVKRDSDSNNGGGSFFTVGFQ------------ILA-S-NL-------PS-A
XP_024438602.1/1-240 ------GNPVKRDSDSNNGGGSFFTVGFQ------------ILA-S-NL-------PS-A
XP_024442055.1/1-239 ------GNPAKRDCDGNSGGGSFLTVGFQ------------ILA-S-NL-------PS-A
XP_002322460.1/1-239 ------GNPAKRDCDGNSGGGSFLTVGFQ------------ILA-S-NL-------PS-A
XP_011048682.1/1-239 ------GNPAKRDCDGNSGGGSFLTVGFQ------------ILA-S-NL-------PS-A
XP_011048684.1/1-239 ------GNPAKRDCDGNSGGGSFLTVGFQ------------ILA-S-NL-------PS-A
XP_011048683.1/1-239 ------GNPAKRDCDGNSGGGSFLTVGFQ------------ILA-S-NL-------PS-A
XP_019081404.1/1-234 ------LA----RGSSNKGSGSLLTVAFQ------------ILV-N-SL-------PM-A
XP_003634081.1/1-234 ------LA----RGSSNKGSGSLLTVAFQ------------ILV-N-SL-------PM-A
XP_008377249.1/1-231 ------N-------GSDGSNGCLLTVAFQ------------ILV-S-SL-------PT-A
XP_017189459.1/1-231 ------N-------GSDGSNGCLLTVAFQ------------ILV-S-SL-------PT-A
XP_008377244.1/1-231 ------N-------GSDGSNGCLLTVAFQ------------ILV-S-SL-------PT-A
XP_009371302.1/1-231 ------N-------GYDGSGGCLLTVAFQ------------ILV-S-SL-------PT-A
XP_009371299.1/1-231 ------N-------GYDGSGGCLLTVAFQ------------ILV-S-SL-------PT-A
XP_008376545.1/1-231 ------N-------GYGGGGGCLLTVAFQ------------ILV-S-SL-------PT-A
XP_008376544.1/1-231 ------N-------GYGGGGGCLLTVAFQ------------ILV-S-SL-------PT-A
XP_017188609.1/1-231 ------N-------GYGGGGGCLLTVAFQ------------ILV-S-SL-------PT-A
XP_022945426.1/1-238 ------DGSIMSDINSGYAGGCLLTVAFQ------------ILV-N-NL-------PT-A
XP_022945425.1/1-238 ------DGSIMSDINSGYAGGCLLTVAFQ------------ILV-N-NL-------PT-A
XP_023541229.1/1-238 ------DGPIMSDINSGYAGGCLLTVAFQ------------ILV-N-NL-------PT-A
XP_023541228.1/1-238 ------DGPIMSDINSGYAGGCLLTVAFQ------------ILV-N-NL-------PT-A
XP_022968414.1/1-238 ------DGSIMSDINSGYASGCLLTVAFQ------------ILV-N-NL-------PT-A
XP_022968413.1/1-238 ------DGSIMSDINSGYASGCLLTVAFQ------------ILV-N-NL-------PT-A
XP_023552381.1/1-232 ------DASMKSGMNSGHDNGCLLTVAFQ------------ILV-N-NL-------PT-A
XP_022922479.1/1-232 ------DASMKSGMNSGHDNGCLLTVAFQ------------ILV-N-NL-------PT-A
XP_023552380.1/1-232 ------DASMKSGMNSGHDNGCLLTVAFQ------------ILV-N-NL-------PT-A
XP_022922478.1/1-232 ------DASMKSGMNSGHDNGCLLTVAFQ------------ILV-N-NL-------PT-A
XP_022984644.1/1-232 ------DASMKSGMNSGHDNGCLLTVAFQ------------ILV-N-SL-------PT-A
XP_022984643.1/1-232 ------DASMKSGMNSGHDNGCLLTVAFQ------------ILV-N-SL-------PT-A
XP_008439185.1/1-242 ------DASVKSSLNSGHGGACLLTVAFQ------------ILV-N-SL-------PT-A
XP_008439184.1/1-242 ------DASVKSSLNSGHGGACLLTVAFQ------------ILV-N-SL-------PT-A
XP_011651140.1/1-242 ------DATVKSSINSGHGGACLLTVAFQ------------ILV-N-SL-------PT-A
XP_004140784.1/1-242 ------DATVKSSINSGHGGACLLTVAFQ------------ILV-N-SL-------PT-A
XP_009391954.1/1-234 --------EHHSDGAATAGSGSLLTIGFQ------------ILV-N-GQ-------PT-E
XP_003581542.2/1-227 -----------IPGDGHKTCGSLLTFAFQ------------ILVKN-SE-------PT-A
XP_006652658.1/1-224 ------------TGADHKMG-SLLTVAFQ------------ILV-N-SQ-------PT-A
XP_015637147.1/1-225 ------------IGADHKMGGSLLTVAFQ------------ILA-N-SQ-------PS-A
XP_025823430.1/1-225 -----------AGGVGHKTSGSLLTVAFQ------------ILV-N-SQ-------PT-A
XP_004978185.1/1-225 -----------TGGVGHKTSGALLTVAFQ------------ILV-N-SQ-------PT-A
XP_021317935.1/1-228 -----------AGGVGHKTCGSLLTVAFQ------------ILV-N-SQ-------PT-A
NP_001336434.1/1-226 -----------ASGLGHKARGSLLTVAFQ------------ILV-N-SQ-------PT-A
XP_019709552.1/1-234 ----------AGELAESPAGGSLLTVAFQ------------ILV-S-SN-------LN-D
XP_015696802.1/1-257 -SPSSTAGAGATGGGGSNNTGSLVTVAFQ------------ILV-N-NL-------PT-A
XP_015612621.1/1-258 -ASP---NSAAGGGGGSNNTGSLVTVAFQ------------ILV-N-NL-------PT-A
XP_004957413.1/1-253 -------PNSQSSTVGSNSAGSLVTVAFQ------------ILV-N-NL-------PT-A
XP_025801167.1/1-251 -------PSVQS---ASGSAGSLVTVAFQ------------ILV-N-NL-------PT-A
NP_001105125.2/1-256 -------PSCGGSS-SSSSTGSLVTVAFQ------------ILV-N-NL-------PT-A
XP_002462701.1/1-258 -------PNCSGGG-NSSTGGSLVTVAFQ------------ILV-N-NL-------PT-A
XP_020262629.1/1-222 ---------------AGTSGGSLLTVAFQ------------ILV-N-TL-------PS-A
XP_020699782.1/1-235 ---------------SGSVGGSLLTVAFQ------------ILV-T-TL-------ST-A
XP_020699776.1/1-235 ---------------SGSVGGSLLTVAFQ------------ILV-T-TL-------ST-A
XP_020573166.1/1-235 ---------------SGNVGGSLLTVAFQ------------ILV-S-TL-------ST-A
XP_020187432.1/1-228 --------------DGHKTCGSLLTVAFQ------------ILG-N-RQ-------PT-G
XP_025656795.1/1-233 -------------GDDGGSSGCLLTVGLQ------------ILL-N-SL-------PT-A
XP_025656792.1/1-233 -------------GDDGGSSGCLLTVGLQ------------ILL-N-SL-------PT-A
XP_025656794.1/1-233 -------------GDDGGSSGCLLTVGLQ------------ILL-N-SL-------PT-A
XP_020979736.1/1-233 -------------GDDGGSSGCLLTVGLQ------------ILL-N-SL-------PT-A
XP_020999151.1/1-233 -------------GDDGGSSGCLLTVGLQ------------ILL-N-SL-------PT-A
XP_015967451.1/1-233 -------------GDDGGSSGCLLTVGLQ------------ILL-N-SL-------PT-A
XP_020999150.1/1-233 -------------GDDGGSSGCLLTVGLQ------------ILL-N-SL-------PT-A
XP_025701970.1/1-233 -------------VDDGGSSGCLLTVGLQ------------ILL-N-SL-------PT-A
XP_025701968.1/1-233 -------------VDDGGSSGCLLTVGLQ------------ILL-N-SL-------PT-A
XP_025701969.1/1-233 -------------VDDGGSSGCLLTVGLQ------------ILL-N-SL-------PT-A
XP_020221720.1/1-222 -------------GSDGGG-SCLLTVGLQ------------MLQ-N-GN-------QT-S
XP_007152327.1/1-222 -------------NNDGGG-SCLLTVGLQ------------MLQ-N-DN-------QS-A
XP_006587345.1/1-222 -------------GSDGGG-SCLLTVGLQ------------MLP-N-GD-------QS-T
XP_006587344.1/1-222 -------------GSDGGG-SCLLTVGLQ------------MLP-N-GD-------QS-T
XP_010035104.1/1-239 -----LKNPDESLIGTADSRGCLMTIGFQ------------ILV-S-ST-------PA-A
XP_021765162.1/1-234 ----TLANDNGAGGSG-SGGASALTLGFQ------------ILV-N-SL-------PT-A
XP_021765160.1/1-234 ----TLANDNGAGGSG-SGGASALTLGFQ------------ILV-N-SL-------PT-A
XP_021730123.1/1-234 ----TLANDNGAGGSG-GGGASALTLGFQ------------ILV-N-SL-------PT-A
XP_021842103.1/1-232 ----TLAHDSGPGA---GTGASALTLGFQ------------ILV-N-SL-------PT-A
XP_021842106.1/1-232 ----TLAHDSGPGA---GTGASALTLGFQ------------ILV-N-SL-------PT-A
XP_021842105.1/1-232 ----TLAHDSGPGA---GTGASALTLGFQ------------ILV-N-SL-------PT-A
XP_021842104.1/1-232 ----TLAHDSGPGA---GTGASALTLGFQ------------ILV-N-SL-------PT-A
XP_010693471.1/1-235 ----TLANDNGDGGGTVGRGASALTLGFQ------------ILV-N-SL-------PT-A
XP_019107938.1/1-235 ----TLANDNGDGGGTVGRGASALTLGFQ------------ILV-N-SL-------PT-A
XP_010693469.1/1-235 ----TLANDNGDGGGTVGRGASALTLGFQ------------ILV-N-SL-------PT-A
XP_019107937.1/1-235 ----TLANDNGDGGGTVGRGASALTLGFQ------------ILV-N-SL-------PT-A
XP_020163116.1/1-258 -SPNA------HGGSSNNSPGSLVTVAFQ------------ILV-N-NL-------PT-A
XP_020163109.1/1-258 -SPNA------HGGSSNNSPGSLVTVAFQ------------ILV-N-NL-------PT-A
XP_003578475.1/1-267 -SQQSQQGSSESAAHGNNNTGSLVTVAFQ------------ILV-N-NL-------PT-A
XP_011012369.1/1-242 -PSNSDGNPVKRDSDSNNGGGSFFTVGFQ------------ILA-S-NL-------PS-A
XP_011012370.1/1-242 -PSNSDGNPVKRDSDSNNGGGSFFTVGFQ------------ILA-S-NL-------PS-A
XP_008234278.1/1-230 -V-KTEG-------NGCDDGGCFLTVGFQ------------ILG-S-NY-------PA-A
XP_021827117.1/1-230 -V-KTEG-------NGCDDGGCFLTVGFQ------------ILG-S-NY-------PA-A
XP_021827116.1/1-230 -V-KTEG-------NGCDDGGCFLTVGFQ------------ILG-S-NY-------PA-A
XP_020413581.1/1-230 -V-KTEG-------NGCDDGGCFLTVGFQ------------ILG-S-NY-------PA-A
XP_007219555.2/1-230 -V-KTEG-------NGCDDGGCFLTVGFQ------------ILG-S-NY-------PA-A
XP_018456926.1/1-230 -S-WYDQIDTHGGFVNHESKGCLLTVGFQ------------ILV-N-SV-------PT-T
XP_013613235.1/1-226 -S-WSDQIDTNGRLVNHESKGCLLTVGFQ------------ILV-N-SV-------PT-T
XP_013686466.1/1-227 -S-WSDQIDKNGGLVNHESKGCLLTVGFQ------------ILV-N-SV-------PT-T
XP_009106897.1/1-227 -S-WSDQIDKNGGLVNHESKGCLLTVGFQ------------ILV-N-SV-------PT-T
XP_006401857.1/1-229 ----SGQVDSNGGLVNHESKGCLLTLGFQ------------ILV-N-SL-------PT-S
XP_020871109.1/1-230 ----SDQFDTDGGLVNHESKGCLLTVGFQ------------ILV-N-SL-------PT-A
NP_001332093.1/1-229 ----SDQFDTDGGLVNQESKGCLLTVGFQ------------ILV-N-SL-------PT-A
NP_200030.1/1-229    ----SDQFDTDGGLVNQESKGCLLTVGFQ------------ILV-N-SL-------PT-A
XP_006282099.1/1-225 ----SDQIDTNG---NQESKGCLLTVGFQ------------ILV-N-SL-------PT-A
XP_019095386.1/1-229 ----SDQIDTNGGLVNQESKGCLLTVGFQ------------ILV-N-SL-------PT-A
XP_019095387.1/1-229 ----SDQIDTNGGLVNQESKGCLLTVGFQ------------ILV-N-SL-------PT-A
XP_019091174.1/1-228 ----SDQINTNGGLVNQESKGCLLTVGFQ------------ILV-N-SL-------PS-A
XP_019091179.1/1-228 ----SDQINTNGGLVNQESKGCLLTVGFQ------------ILV-N-SL-------PS-A
XP_019087173.1/1-228 ----SDQINTNGGMVNQESKGCLLTLGFQ------------ILV-N-CL-------PT-A
XP_010555654.1/1-234 -P-ESDG-------FGGGGSGCLLSIGFQ------------ILV-N-SL-------PN-A
XP_010555656.1/1-234 -P-ESDG-------FGGGGSGCLLSIGFQ------------ILV-N-SL-------PN-A
XP_010555655.1/1-234 -P-ESDG-------FGGGGSGCLLSIGFQ------------ILV-N-SL-------PN-A
XP_010537199.1/1-242 -N-EVDGNT--TTSTSNNNGGCILTVGFQ------------ILV-N-SL-------PN-A
XP_010537197.1/1-242 -N-EVDGNT--TTSTSNNNGGCILTVGFQ------------ILV-N-SL-------PN-A
XP_010537198.1/1-241 -N-EVDGNT--TTSTSNNNGGCILTVGFQ------------ILV-N-SL-------PN-A
Con_90%              ....................tslhTluhQ............hhh...p............
Con_80%              ..................tstSllTluFQ............hhh.s.p............
Con_70%              ................ttssuSlLTlAFQ............hhh.s.s............
Con_60%              ...............tsssuGSLLTlAFQ............lLs.s.s...........s
Con_50%              .............ssssssuGSLLTVAFQ............ILV.s.Sh.........sA


XP_020582785.1/1-284 --LQESVVAMARQYTRSVVASVQRVA---
XP_020682623.1/1-284 --LQESVVAMARQYIRSIVASVQRVA---
XP_020682624.1/1-284 --LQESVVAMARQYIRSIVASVQRVA---
XP_006846015.1/1-286 --LRDNVVAMARQYVRSVMASVQRVA---
NP_001142394.1/1-289 --LRESVAAMAKQYVRGVMASVQRVA---
NP_001146215.1/1-288 --LRESVAAMARQYVRAVMAIVQRVA---
XP_020405718.1/1-288 --LRESVAAMARQYVRAVMAIVQRVA---
XP_002443548.1/1-287 --LRDSVAAMARQYVRGVMASVQRVA---
XP_025808876.1/1-287 --LRESVAAMARQYVRGVMASVQRVA---
XP_004963232.1/1-287 --LRESVAAMARQYVRGVMASVQRVA---
XP_020192001.1/1-285 --LRESVAAMARQYVRTVVASVQRVA---
XP_003562005.1/1-285 --LRESVASMARQYVRTVVASVQRVA---
XP_004982339.1/1-286 --LRESVAAMARQYVRTVVASVQRVA---
XP_025796542.1/1-286 --LRESVAAMARQYVRTVVASVQRVA---
NP_001347456.1/1-286 --LRESVAAMARQYVRTVVASVQRVA---
NP_001352464.1/1-286 --LRESVAAMARQYVRTVVASVQRVA---
XP_002464180.1/1-286 --LRESVAAMARQYVRTVVASVQRVA---
XP_015689987.1/1-286 --LRESVAAMARQYVRTVVASVQRVA---
XP_015632484.1/1-286 --LRESVAAMARQYVRTVVASVQRVA---
XP_015620816.1/1-285 --LRESVAAMARSYVRAVMASVQRVA---
XP_006664738.1/1-285 --LRDSVAAMARNYVRAVMASVQRVA---
XP_020190954.1/1-286 --LRESVAAMARQYVRAVMASVQRVA---
XP_020163543.1/1-286 --LRESVAAMARQYVRAVMASVQRVA---
XP_003577453.1/1-286 --LRESVAAMARQYVRAVMASVQRVA---
XP_020575104.1/1-285 --LRENVTAMARQYVRSVVASVQRVA---
XP_020696902.1/1-286 --LRENVTAMARQYVRSVVASVQRVA---
XP_020578108.1/1-284 --FRENMAAMARQYVRSIVTSVQRVA---
XP_020698923.1/1-284 --FRENMAAMARQYVRSVVASVQRVA---
XP_020096610.1/1-279 --LRENVATMARQYVRSVMASIQRVA---
XP_009412508.1/1-287 --LRENVAAMARQYVRSVVTSVQRVA---
XP_020271156.1/1-284 --LRDNVAAMARQYVRSVVASVQRVA---
XP_020273236.1/1-287 --LRDNVAAMARQYVRSVVASVQRVA---
XP_020092733.1/1-287 --LRENVAAMARQYVRSVVASVQRVA---
XP_019701934.1/1-288 --LQESVAAMARQYVRSVVASVQRVA---
XP_010905421.1/1-288 --LQESVAAMARQYVRSVVASVQRVA---
XP_010905420.1/1-289 --LQESVAAMARQYVRSVVASVQRVA---
XP_010923678.1/1-288 --LRESVAAMARQYVRSVVASVQRVA---
XP_008794452.1/1-288 --LRESVAAMARQYVRSVVASVQRVA---
XP_009397391.1/1-288 --LQGNVAAMARQYVRSVVASVQRVA---
XP_009408551.1/1-288 --LQDNVAAMARQYVRSVVASVQRVA---
XP_009399045.1/1-288 --LRDSVAEMARQYVRNVVASVQRVA---
XP_009388964.1/1-288 --LRDNVAAMARQYVRSVVASVQRVA---
XP_009397717.1/1-288 --LRDSVAAMARQYVRSVVTSVQRVA---
XP_018488876.1/1-280 --SRDNVATMARQYVRNVVGSIQRVA---
XP_013587486.1/1-280 --SRDNVATMARQYVRNVVGSIQRVA---
XP_009115139.1/1-280 --SRDNVATMARQYVRNVVGSIQRVA---
XP_013662459.1/1-280 --SRDNVATMARQYVRNVVGSIQRVA---
XP_013707293.1/1-280 --SRDNVATMARQYVRNVVGSIQRVA---
XP_006415465.2/1-280 --SRDNVATMARQYVRNVVGSIQRVA---
XP_019099288.1/1-280 --SRDNVATMARHYVRNVVGSIQRVA---
NP_174337.1/1-280    --SRDNVATMARQYVRNVVGSIQRVA---
XP_020867051.1/1-280 --SRDNVATMARQYVRNVVGSIQRVA---
XP_020867052.1/1-280 --SRDNVATMARQYVRNVVGSIQRVA---
XP_010460847.1/1-280 --SRDNVATMARQYVRNVVGSIQRVA---
XP_019094482.1/1-280 --SRDNVATMARQYVRNVVGSIQRVA---
XP_006306764.1/1-280 --SRDNVATMARQYVRNVVGSIQRVA---
XP_010538268.1/1-285 --SRDNVASMARQYVRSVVGSIQRVA---
XP_010519070.1/1-284 --SRDNVAAMARPYVRGIVGSIQRVA---
XP_018436356.1/1-280 --TRDNVASMAREYVRSIVGSIQRVA---
XP_013637594.1/1-286 --TRDSVASMARQYVRSIVGSIQRVA---
XP_013692700.1/1-286 --TRDSVASMARQYVRSIVGSIQRVA---
XP_009143747.1/1-286 --TRDNVASMARQYVRSIVGSIQRVA---
XP_018484893.1/1-285 --TRDNVASMARQYVRNIVGSIQRVA---
XP_013637264.1/1-280 --TRDNVASMARQYVRNIVGSIQRVA---
XP_013637263.1/1-280 --TRDNVASMARQYVRNIVGSIQRVA---
XP_022557131.1/1-280 --TRDNVASMARQYVRNIVGSIQRVA---
XP_013729051.1/1-280 --TRDNVASMARQYVRNIVGSIQRVA---
XP_013744548.1/1-280 --TRDNVASMARQYVRNIVGSIQRVA---
XP_009141396.1/1-280 --TRDNVASMARQYVRNIVGSIQRVA---
XP_006410621.1/1-284 --TRENVASMARQYVRSIVGSIQRVA---
NP_181018.1/1-284    --SRDSVASMARQYVRSIVGSIQRVA---
XP_020884308.1/1-284 --SRDSVASMARQYVRSIVGSIQRVA---
XP_006293359.1/1-284 --SRDSVASMARQYVRSIVGSIQRVA---
XP_010516626.1/1-284 --SRDNVASMARQYVRSIVGSIQRVS---
XP_010509720.1/1-284 --SRDSVASMARQYVRSIVGSIQRVA---
XP_010504942.1/1-284 --SRDSVASMARQYVRSIVGSIQRVA---
XP_017223336.1/1-283 --FRDNVASMARQYVRSVVGSVQRVA---
XP_016559371.1/1-285 --YRENVAAMARQYVRNIVGSVQRVA---
XP_016559374.1/1-285 --YRENVAAMARQYVRNIVGSVQRVA---
XP_012830207.1/1-281 --YQDSVAAMARQYVRSIVASVQRVA---
XP_012827547.1/1-287 --YRDSVVAMARQYVRSIVGSVQRVA---
XP_022866806.1/1-284 --YRDSVAAMARHYLRSIVGSVQRVA---
XP_022866805.1/1-285 --YRDSVAAMARHYLRSIVGSVQRVA---
XP_023001017.1/1-285 --MRDSVAAMARQYVRTVVASVQRVA---
XP_023001016.1/1-285 --MRDSVAAMARQYVRTVVASVQRVA---
XP_023519837.1/1-285 --MRDNVAAMARQYVRTVVASVQRVA---
XP_023519836.1/1-285 --MRDNVAAMARQYVRTVVASVQRVA---
XP_022927139.1/1-285 --MRDSVAAMARQYVRTVVASVQRVA---
XP_022927138.1/1-285 --MRDSVAAMARQYVRTVVASVQRVA---
XP_022139982.1/1-285 --MRDSVAAMARQYVRTVVGSVQRVA---
XP_004135550.1/1-285 --MRDSVAAMARQYVRTVVGSVQRVA---
XP_008445207.1/1-285 --MRDSVAAMARQYVQTVVGSVQRVA---
XP_017230337.1/1-286 --LRDNVAVMARQYVRSVVGSVQRVA---
XP_017230338.1/1-286 --LRDNVAVMARQYVRSVVGSVQRVA---
XP_010316959.1/1-285 --YRENVAAMARQYVRSIVGSVQRVA---
XP_004233170.1/1-285 --YRENVAAMARQYVRSIVGSVQRVA---
XP_015066839.1/1-285 --YRENVAAMARQYVRSIVGSVQRVA---
XP_015066842.1/1-285 --YRENVAAMARQYVRSIVGSVQRVA---
XP_015066843.1/1-285 --YRENVAAMARQYVRSIVGSVQRVA---
XP_006353018.1/1-285 --YRENVAAMARQYVRSIVGSVQRVA---
XP_006353020.1/1-285 --YRENVAAMARQYVRSIVGSVQRVA---
XP_019178964.1/1-287 --YRDNVAAMARQYVRSIVSSVQRVA---
XP_019178963.1/1-287 --YRDNVAAMARQYVRSIVSSVQRVA---
XP_019228489.1/1-285 --YRDNVAAMARQYVRSIVGSVQRVA---
XP_019228488.1/1-285 --YRDNVAAMARQYVRSIVGSVQRVA---
NP_001289498.1/1-285 --YRDNVAAMARQYVRSIVGSVQRVA---
XP_009777911.1/1-285 --YRDNVAAMARQYVRSIVGSVQRVA---
XP_009612342.1/1-285 --YRDNVAAMARQYVRSIVGSVQRVA---
XP_009612341.1/1-285 --YRDNVAAMARQYVRSIVGSVQRVA---
XP_006363649.1/1-287 --YQDNVAAMARQYVRSIVGSVQRVA---
XP_015066689.1/1-287 --YQDNVAAMARQYVRSIVGSVQRVA---
XP_010315636.1/1-287 --YQDNVAAMARQYVRSIVGSVQRVA---
XP_022879368.1/1-285 --YQDTVAAMARRYVRNIVSSVQRVA---
XP_020554026.1/1-285 --YRDSVAAMARQYVRSIVGSVQRVA---
XP_011096678.1/1-285 --YRDSVAAMARQYVRSIVGSVQRVA---
XP_011085207.1/1-285 --YQDSVAAMARQYVRSIVASVQRVA---
XP_023003193.1/1-285 --LQENVAAMARQYVRSVVGSVQRVA---
XP_022926723.1/1-285 --LQENVAAMARQYVRSIVGSVQRVA---
XP_023518237.1/1-285 --LQENVAAMARQYVRSVVGSVQRVA---
XP_022146537.1/1-286 --MRDNVAAMARQYVRSVVGSVQRVA---
XP_011658319.1/1-286 --LQENVAAMARQYVRSVVGSVQRVA---
XP_008439554.1/1-286 --LQENVAAMARQYVRSVVGSVQRVA---
XP_022978372.1/1-286 --LQENVAAMARQYVRSVVGSVQRVS---
XP_023543833.1/1-286 --LQENVAAMARQYVRSVVGSVQRVA---
XP_022926042.1/1-286 --LQENVAAMARQYVRSVVGSVQRVA---
XP_010247499.1/1-286 --FRDNVAAMARQYVRSVVGSVQRVA---
XP_016717556.1/1-285 --LRDDVASMARQYVRSVVGSVQRVA---
XP_012455108.1/1-285 --LRDDVASMARQYVRSVVGSVQRVA---
XP_016717554.1/1-287 --LRDDVASMARQYVRSVVGSVQRVA---
XP_012455107.1/1-287 --LRDDVASMARQYVRSVVGSVQRVA---
XP_016711412.1/1-287 --LRDDVASMARQYVRSVVGSVQRVA---
XP_017649973.1/1-287 --LRDDVASMARQYVRSVVGSVQRVA---
XP_022716449.1/1-285 --LRDNVAAMARQYVRSVVGSVQRVA---
XP_022724664.1/1-287 --MRDNVAAMARQYVRSVVGSVQRVA---
XP_021298192.1/1-285 --SRENVAAMARQYVRSVVGSVQRVA---
XP_021298193.1/1-294 --SRENVAAMARQYVRSVVGSVQRVA---
XP_021298191.1/1-294 --SRENVAAMARQYVRSVVGSVQRVA---
XP_007022874.2/1-285 --LRDNVAAMARQYVRSVVGSVQRVA---
XP_017979205.1/1-294 --LRDNVAAMARQYVRSVVGSVQRVA---
XP_017979204.1/1-294 --LRDNVAAMARQYVRSVVGSVQRVA---
XP_017236680.1/1-286 --FRDNVAVMARQYVRSVVGSVQRVA---
XP_010051959.1/1-285 --LRDTVAIMARQYVRSVVGSVQRVA---
XP_010051960.1/1-285 --LRDTVAIMARQYVRSVVGSVQRVA---
XP_015882975.1/1-284 --LRDNVAAMARQYVRSVVGSVQRVA---
XP_015882797.1/1-284 --LRDNVAAMARQYVRSVVGSVQRVA---
XP_010098570.1/1-284 --TRDNVAAMARQYVRSVVGSVQRVA---
XP_011012084.1/1-284 --FRDNVAAMARQYVRGVVASVQRVA---
XP_011012083.1/1-284 --FRDNVAAMARQYVRGVVASVQRVA---
XP_006377667.2/1-284 --FRDNVAAMARQYVRGVVASVQRVA---
XP_002281868.2/1-285 --VRDNVAAMARQYVRSVMASVQRVA---
XP_018847863.1/1-285 --LRDNVAAMARQYVRSVVGSVQRVA---
XP_018847862.1/1-285 --LRDNVAAMARQYVRSVVGSVQRVA---
XP_023919942.1/1-285 --LRDNVAAMARQYVRSVVGSVQRVA---
XP_023919941.1/1-285 --LRDNVAAMARQYVRSVVGSVQRVA---
XP_018828101.1/1-285 --LRDNVAAMARQYVRSVVGSVQRVA---
XP_018828100.1/1-285 --LRDNVAAMARQYVRSVVGSVQRVA---
XP_002298892.1/1-285 --MRDNVAAMARQYVRGVVGSVQRVA---
XP_011038887.1/1-285 --MRDNVAAMARQYVRGVVGSVQRVA---
XP_011038886.1/1-285 --MRDNVAAMARQYVRGVVGSVQRVA---
XP_021657817.1/1-285 --LRDNVAAMARQYVRSVVGSVQRVA---
XP_021657818.1/1-285 --LRDNVAAMARQYVRSVVGSVQRVA---
XP_015577248.1/1-260 -----------------------------
XP_021616343.1/1-285 --LRDNVAAMARQYVRSVVGSVQRVA---
XP_021645103.1/1-285 --LRDNVAAMARQYVRSVVGSVQRVA---
XP_021592762.1/1-285 --LRDNVAAMARQYVRSVVGSVQRVA---
XP_012075627.1/1-285 --LRDNVAAMARQYVRSVVGSVQRVA---
XP_008372224.1/1-285 --LRDNVAAMARQYVRSVVSSVQRVA---
XP_009362238.1/1-285 --LRDNVAAMARQYVRSVVSSVQRVA---
XP_009344405.1/1-284 --LRDNVAAMARQYVRSVVSSVQRVA---
XP_008349150.1/1-284 --LRDNVAAMARQYVRSVVSSVQRVA---
XP_007214622.1/1-285 --LRDNVAAMARQYVRSVVSSVQRVA---
XP_021812393.1/1-285 --LRDNVAAMARQYVRSVVSSVQRVA---
XP_024161164.1/1-285 --LRDSVAAMARQYVRGVVSSVQRVA---
XP_004293989.1/1-285 --MRDSVAAMARQYVRGVVSSVQRVA---
XP_019419795.1/1-282 --LRDNVAAMARQYVRSVVGSVQRVA---
XP_019426301.1/1-282 --LRDNVAAMARQYVRSVVGSVQRVA---
XP_003524993.1/1-285 --LRDNVAVMARQYVRNVVRSVQRVA---
XP_003532788.1/1-285 --LRDNVAVMARQYVRNVVRSVQRVA---
XP_020215009.1/1-285 --LRDNVAVMARQYVRNVVRSVQRVA---
XP_017409410.1/1-285 --LRDNVAVMARQYVRNVVRSVQRVA---
XP_017409418.1/1-285 --LRDNVAVMARQYVRNVVRSVQRVA---
XP_014509941.1/1-285 --LRDNVAVMARQYVRNVVRSVQRVA---
XP_007159548.1/1-285 --LRDNVAVMARQYVRNVVRSVQRVA---
XP_024047818.1/1-288 --MRDNVAAMARQYVRSVVGSVQRVA---
XP_006448915.2/1-288 --MRDNVAAMARQYVRSVVGSVQRVA---
XP_006468302.1/1-288 --MRDNVAAMARQYVRSVVGSVQRVA---
XP_006468303.1/1-287 --MRDNVAAMARQYVRSVVGSVQRVA---
XP_024047817.1/1-287 --MRDNVAAMARQYVRSVVGSVQRVA---
XP_004504217.1/1-285 --LRDNVAAMARQYVRSVVRSVQRVA---
XP_003594520.2/1-281 --LRDNVASMARQYVRSVVASVQRVA---
XP_024632601.1/1-281 --LRDNVASMARQYVRSVVASVQRVA---
XP_004486450.1/1-285 --MRDNVAAMARQYVRSVVGSVQRVA---
XP_007147487.1/1-285 --TRDNVAAMARQYVRSVVGSVQRVA---
XP_017437412.1/1-285 --TRDNVAAMARQYVRSVVGSVQRVA---
XP_014518680.1/1-285 --TRDNVAAMARQYVRSVVGSVQRVA---
XP_003546255.1/1-285 --TRDNVAAMARQYVRSVVGSVQRVA---
XP_020233213.1/1-285 --TRDNVAAMARQYVRSVVGSVQRVA---
XP_003535078.1/1-285 --TRDNVAAMARQYVRSVVGSVQRVA---
XP_014617344.1/1-285 --TRDNVAAMARQYVRSVVGSVQRVA---
XP_015959291.1/1-283 --LRDNVAAMARQYVRSVVGSVQRVA---
XP_016197724.1/1-283 --LRDNVAAMARQYVRSVVGSVQRVA---
XP_025694278.1/1-283 --LRDNVAAMARQYVRSVVGSVQRVA---
XP_006846777.1/1-281 --MRDNVVAMARQYVRGVVGSVQRVA---
XP_003574071.1/1-280 --LQDTVATMARQYVRSIVSAVQRVS---
XP_020174778.1/1-278 --LQDSVATMARQYVRSIVSAVQRVS---
XP_006661867.1/1-281 --LQDSVATMARQYVRSIVSSVQRVS---
XP_015614901.1/1-281 --LQDSVATMARQYVRSIVSSVQRVS---
XP_004982838.1/1-282 --LQDSVAAMARQYVRSIVSAVQRVS---
XP_025796396.1/1-282 --LQDSVAAIARQYVRSIVSAVQRVS---
NP_001316502.1/1-284 --LQDTVAAMARQYVRSIVSAVQRVS---
XP_021306737.1/1-282 --LQDTVAAMARQYVRSIVSAVQRVS---
XP_021306733.1/1-293 --LQDTVAAMARQYVRSIVSAVQRVS---
XP_020162053.1/1-281 --LQDSVAAMARQYVRSIVSAVQRVS---
XP_006649248.1/1-282 --LQDSVAAMARQYVRSIVSAVQRVS---
XP_015632709.1/1-282 --LQDSVAAMARQYVRSIVSAVQRVS---
NP_001296150.1/1-282 --LQDSVAAMARQYVRSVISAVQRVS---
XP_008665074.1/1-283 --LQDSVATMARQYVRSVVSAVQRVS---
NP_001105994.1/1-283 --LQDSVATMARQYVRSVVSAVQRVS---
XP_008660404.1/1-283 --LQDSVATMARQYVRSVVSAVQRVS---
XP_002468669.1/1-283 --LQDSVATMARQYVRGVVSAVQRVS---
XP_021309716.1/1-285 --LQDSVATMARQYVRGVVSAVQRVS---
XP_004986037.1/1-283 --LQDSVATMARQYVRSVVSAVQRVS---
XP_025795001.1/1-283 --LQDSVATMARQYVRSVVSAVQRVS---
XP_020093893.1/1-283 --LQESIAATARQYVRSIVSAVQRVA---
XP_020093894.1/1-282 --LQESIAATARQYVRSIVSAVQRVA---
XP_020259754.1/1-283 --LQESVAGMARQYVRNIVSAVQRVS---
XP_009401955.1/1-282 --LQDSIAAMARQYVRSIVSAIQRVS---
XP_008791541.1/1-283 --LQDSVAAMARQYVRSIVSSVQRVS---
XP_017698637.1/1-283 --LQDSVAAMARQYVRSIVSSVQRVS---
XP_010920422.1/1-283 --LQDSVAAMARQYVRSIVSAVQRVS---
XP_010920423.1/1-283 --LQDSVAAMARQYVRSIVSAVQRVS---
XP_010920419.1/1-283 --LQDSVAAMARQYVRSIVSAVQRVS---
XP_010920426.1/1-283 --LQDSVAAMARQYVRSIVSAVQRVS---
XP_019709326.1/1-287 --LQDNVAAMARQYVRSIISAVQRVS---
XP_008785281.1/1-283 --LQDNVVAMARQYVRSIISAVQRVS---
XP_008462593.1/1-288 --LQDNVAAMARQYVRSVVSAVQRVA---
XP_022969629.1/1-286 --LQDDVAAMARQYVRSVVSAVQRVA---
XP_023536970.1/1-286 --LQDDVAAMARQYVRSVVSAVQRVA---
XP_022937855.1/1-288 --LQDDVAAMARQYVRSVVSAVQRVA---
XP_022937858.1/1-288 --LQDDVAAMARQYVRSVVSAVQRVA---
XP_012827869.1/1-285 --LQDNVATMARQYVRSVVSSVQRVA---
XP_022874815.1/1-279 --LQESVATFACQYVRSVISSVQRVS---
XP_022874814.1/1-283 --LQESVATFACQYVRSVISSVQRVS---
XP_018434000.1/1-280 --LQENVANMACQYLRSVISSVQRVA---
XP_013642496.1/1-279 --LQENVANMACQYVRSVISSVQRVA---
XP_009150087.1/1-279 --LQENVANMACQYVRSVISSVQRVA---
XP_009150088.1/1-279 --LQENVANMACQYVRSVISSVQRVA---
XP_022563181.1/1-279 --LQDNVANMACQYVRSVISSVQRVA---
XP_013675790.1/1-280 --LQDNVANMACQYVRSVISSVQRVA---
XP_013623090.1/1-279 --LQENVANMACQYVRSVISSVQRVA---
XP_013623089.1/1-280 --LQENVANMACQYVRSVISSVQRVA---
XP_020885782.1/1-285 --LQENVAGMACQYVRSVISSVQRVA---
XP_006280004.1/1-285 --LQENVAGMACQYVRSVISSVQRVA---
XP_019088563.1/1-285 --LQDNVAGMACQYVRSVISSVQRVA---
XP_010483719.1/1-285 --LQDNVAGMACQYVRSVISSVQRVA---
XP_010456679.1/1-285 --LQDNVAGMACQYVRSVISSVQRVA---
XP_006400821.1/1-284 --LQENVAGMACQYVRSVISSVQRVA---
NP_200877.1/1-284    --LQENVAGMACQYVRSVISSVQRVA---
XP_013611543.1/1-282 --LQDNVAGMACQYVRSVISSVQRVA---
XP_009120605.1/1-284 --LQDNVAGMACQYVRSVISSVQRVA---
XP_013667152.1/1-284 --LQDNVAGMACQYVRSVISSVQRVA---
XP_018446535.1/1-284 --LQDNVAGMACQYVRSVISSVQRVA---
XP_018454374.1/1-284 --LQENVAGMACQYVRSVISSVQRVA---
XP_009126588.1/1-285 --LQENVAGMACQYVRSVISSVQRVA---
XP_013680521.1/1-285 --LQENVAGMACQYVRSVISSVQRVA---
XP_013612427.1/1-285 --LQENVAGMACQYVRSVISSVQRVA---
XP_010535608.1/1-283 --LQENVATMACQYVRSVISSVQRVA---
XP_010520372.1/1-276 --LRENVAIMACQYVRSVISSVQRVA---
XP_004487103.1/1-286 --LQDNVAAMARQYVRRVVSSVQAVA---
XP_003597252.1/1-283 --LQDNVAAMARQYVRRVVSAVQAVA---
XP_024455683.1/1-286 --LQDNVATMARQYVRSVISSVQRVA---
XP_022026352.1/1-288 --LADSVATMARQYVRSVINSVQRVA---
XP_022000486.1/1-288 --LAESVATMARQYVRSVINSVQRVA---
XP_016717477.1/1-281 --LQDNVATMARQYVRSVISSVQRVA---
XP_017643014.1/1-281 --LQDNVATMARQYVRSVISSVQRVA---
XP_016724213.1/1-281 --LQDNVLTMARQYVRSVISSVQRIA---
XP_012462938.1/1-281 --LQDNVLTMARQYVRSVISSVQRIA---
XP_012471509.1/1-292 --LQDNVATMARQYVRSVISSVQRVA---
XP_017642742.1/1-292 --LQDNVATMARQYVRSVISSVQRVA---
XP_016755876.1/1-292 --LQDNVATMARQYVRSVISSVQRVA---
XP_012471510.1/1-286 --LQDNVATMARQYVRSVISSVQRVA---
XP_016753711.1/1-286 --LQDNVATRARQYVRSVISSVQRVA---
XP_017642744.1/1-286 --LQDNVATMARQYVRSVISSVQRVA---
XP_016755877.1/1-286 --LQDNVATMARQYVRSVISSVQRVA---
XP_016750468.1/1-286 --LRDNVATMARQYVRSVISSVQRVA---
XP_016688657.1/1-286 --LRDNVATMARQYVRSVISSVQRVA---
XP_012443272.1/1-286 --LRDNVATMARQYVRSVISSVQRVA---
XP_017603001.1/1-286 --LRDNVATMARQYVRSVISSVQRVA---
XP_022755632.1/1-285 --LQDNVATMARQYVRSVISSVQRVA---
XP_022755633.1/1-285 --LQDNVATMARQYVRSVISSVQRVA---
XP_022729815.1/1-286 --LQDNVANMARQYVRSVISSFQRVA---
XP_022729816.1/1-286 --LQDNVANMARQYVRSVISSFQRVA---
XP_022752857.1/1-286 --LQDHVATMARQYVRSVISSVQRVA---
XP_022752856.1/1-286 --LQDHVATMARQYVRSVISSVQRVA---
XP_021277981.1/1-286 --LQDNVAAMARQYVRSVIASVQRVA---
XP_017983554.1/1-286 --LQDNVAAMARQYVRSVIASVQRVA---
XP_011089912.1/1-283 --LQDNVATMARQYVRSVVSSVQRVA---
XP_007208090.1/1-286 --LQENVATMARQYVRSVISSVQRVA---
XP_008225704.1/1-286 --LQENVATMARQYVRSVISSVQRVA---
XP_021830853.1/1-286 --LQENVATMARQYVRSVISSVQRVA---
XP_008349754.1/1-286 --LQENVANMARQYVRSVISSVQRVA---
NP_001315783.1/1-288 --LQENVANMARQYVRSVISSVQRVA---
XP_009367069.1/1-286 --LQENVANMARQYVRSVISSVQRVA---
XP_008344872.1/1-286 --LQENVANMARQYVRSVISSVQRVA---
XP_015952399.1/1-286 --LQDNVAVMARQYVRSVISSVQRVA---
XP_020982118.1/1-286 --LQDNVAVMARQYVRCVISSVQRVA---
XP_025685402.1/1-286 --LQDNVAVMARQYVRSVISSVQRVA---
XP_016187430.1/1-286 --LQDNVAVMARQYVRSVISSVQRVA---
XP_020214107.1/1-284 --LQDNVAVMARQYVRSVISSVQRVA---
XP_006592258.1/1-284 --LQDNVAVMARQYVRSVISSVQRVA---
XP_007132262.1/1-285 --LQDNVAVMARQYVRSVISSVQRVA---
XP_003538150.1/1-285 --LQDNVAVMARQYVRSVISSVQRVA---
XP_017433493.1/1-285 --LQDNVAVMARQYVRSVISSVQRVA---
XP_014494018.1/1-285 --LQDNVAVMARQYVRSVISSVQRVA---
XP_010089983.2/1-286 --LQDNVATMARQYVRSVISSVQRVA---
XP_006378974.2/1-285 --LQDNVATMARQYVRSVISSVQRVA---
XP_011010051.1/1-285 --LQDNVATMARQYVRSVISSVQRVA---
XP_011010050.1/1-285 --LQDNVATMARQYVRSVISSVQRVA---
XP_021905657.1/1-285 --LQENVATMARQYVRSVISSVQRVA---
XP_021610391.1/1-286 --LQDNVANMARQYVRSVISSVQRVA---
XP_002529946.1/1-286 --LQENVATMARQYVRSVISSVQRVA---
XP_021658728.1/1-286 --LQDNVATMARQYVRSVISSVQRVA---
XP_012064816.1/1-286 --LQDNVATMARQYVRSVISSVQRVA---
XP_021628343.1/1-286 --LQDNVSTMARQYVRSVISAVQRVA---
XP_021657859.1/1-286 --LQDNVATMARQYVRSVISSVQRVA---
XP_015874442.1/1-285 --LQENVAAMARQYVCSVISSVQRVA---
XP_023926843.1/1-286 --LQDNVATMARQYVRSVISSVQRVA---
XP_018819529.1/1-286 --LQDNVANMACQYVRSVISSVQRVA---
XP_010651164.1/1-286 --LQDNVATMARQYVRSVISSVQRVA---
XP_024034672.1/1-286 --LQDNVATMARQYVRSVISSVQRVA---
XP_006488084.1/1-286 --LQDNVATMARQYVRSVISSVQRVA---
XP_024034673.1/1-286 --LQDNVATMARQYVRSVISSVQRVA---
XP_010264677.1/1-287 --LQENVAAMARQYVRSVISSVQRVA---
XP_010253680.1/1-288 --LQDNVATMARQYIRSVISSVQRVS---
XP_022005829.1/1-282 --LADSVATMARQYVRSVINSVQRVA---
XP_023751537.1/1-278 --LADSVATMARQYVRSVINSVQRVA---
XP_024971452.1/1-283 --LADSVATMARQYVRSVINSVQRVA---
XP_024971444.1/1-287 --LADSVATMARQYVRSVINSVQRVA---
XP_024985647.1/1-287 --LAESVATMARQYVRSVINSVQRVA---
XP_023749448.1/1-288 --LAESVATMARQYVRSVINSVQRVA---
XP_010689538.1/1-281 --LQENVATMARQYVRSVISSVQRVA---
XP_021858740.1/1-280 --LQENVATMARQYVRTVISSVQRVA---
XP_021773317.1/1-281 --LQENVATMARQYVRTVISSVQRVA---
XP_021745941.1/1-281 --LQENVATMARQYVRTVISSVQRVA---
XP_016729039.1/1-286 --LEDNVANMAHQYVRSIISSVQRIA---
XP_017623828.1/1-286 --LEDNVANMAHQYVRSIISSVQRIA---
XP_012476406.1/1-286 --LEDNVANMAHQYVRSVISSVQRIA---
XP_016754776.1/1-286 --LEDNVANMAHQYVRSVISSVQRIA---
XP_022152944.1/1-286 --LQDDVAAMARQYVRSVVSAVQRVA---
XP_011040884.1/1-286 --LQDNVATMARQYVRSVISSVQRVA---
XP_010043753.1/1-286 --TQDNVAVMARQYVRSVISSVQRVA---
XP_024158223.1/1-285 --LQDNVASMARQYVRNVISSVQRVA---
XP_004291573.1/1-285 --LQDNVASMARQYVRNVISSVQRVA---
XP_018808765.1/1-285 --LQDNVANMARQYVRSVISSVQRVA---
XP_023512455.1/1-283 --MQDNVTNMAHQYVRSVISSVQRVA---
XP_023512456.1/1-283 --MQDNVTNMAHQYVRSVISSVQRVA---
XP_022944186.1/1-283 --MQDNVTNMAHQYVRSVISSVQRVA---
XP_022944185.1/1-283 --MQDNVTNMAHQYVRSVISSVQRVA---
XP_022985614.1/1-283 --MQDNVTNMAHQYVRSVISSVQRVA---
XP_022985615.1/1-283 --MQDNVTNMAHQYVRSVISSVQRVA---
XP_011656996.1/1-283 --MQDNVANMAHQYVRSVISSVQRVA---
XP_011656997.1/1-283 --MQDNVANMAHQYVRSVISSVQRVA---
XP_008463945.1/1-283 --LQDNVANMAHQYVRSVISSVQRVA---
XP_008463946.1/1-283 --LQDNVANMAHQYVRSVISSVQRVA---
XP_022140363.1/1-286 --MQDNVANMAHQYVRSVISSVQRVA---
XP_022995706.1/1-286 --MQDNVMNMAQQYVRSVISSVQRVA---
XP_023534373.1/1-286 --MQDNIMNMAQQYVRSVISSVQRVA---
XP_022958713.1/1-286 --MQDNVMNMAQQYVRSVISSVQRVA---
XP_025614062.1/1-285 --LQDNVAIMARQYIRSVISSVQRVA---
XP_025614063.1/1-285 --LQDNVAIMARQYIRSVISSVQRVA---
XP_016170485.1/1-285 --LQDNVAVMARQYIRSVISSVQRVA---
XP_016170486.1/1-285 --LQDNVAVMARQYIRSVISSVQRVA---
XP_015935757.1/1-285 --LQDNVAIMARQYIRSVISSVQRVA---
XP_015935758.1/1-285 --LQDNVAIMARQYIRSVISSVQRVA---
XP_004505999.1/1-286 --MQENVAIMARQYVRSVISSVQRVA---
XP_013456006.1/1-286 --MQDNVVIMARQYVRSVIASVQRVA---
XP_019453966.1/1-285 --LQDNVAIMARQYVRSVISSVQRVA---
XP_019448740.1/1-279 --LQDNVAIMARQYVRSVISSVQRVA---
XP_019448741.1/1-279 --LQDNVAIMARQYVRSVISSVQRVA---
XP_019458095.1/1-286 --LQDNIALMAIQYVRSVISSVQRIA---
XP_019447870.1/1-286 --LQDNVAGMALQYVRSVISSVQRVA---
XP_012838629.1/1-280 --LHENVAAMARQYVRSVISSVQRAA---
XP_012838630.1/1-280 --LHENVAAMARQYVRSVISSVQRAA---
XP_012838627.1/1-280 --LHENVAAMARQYVRSVISSVQRAA---
XP_012838628.1/1-280 --LHENVAAMARQYVRSVISSVQRAA---
XP_017214964.1/1-286 --QADSVATMARQYVRTVVSSVQRVA---
XP_017234817.1/1-286 --QPENVATMARQYVRSVVSSVQRVA---
XP_019178153.1/1-282 --LQENVATMARQYVRSVISSVQRVA---
XP_019166562.1/1-283 --LQDNVATMARQYVRSVISSVQRVA---
XP_011089294.1/1-282 --LQENVATMARQYVRSVISSVQRVA---
XP_022892122.1/1-274 --LQDNVAAMARQYVRSVISSVQRVA---
XP_022883393.1/1-279 --LQDNVATMARQYVRSVISSVQRVS---
XP_022883398.1/1-279 --LQDNVATMARQYVRSVISSVQRVS---
XP_022883384.1/1-283 --LQDNVATMARQYVRSVISSVQRVS---
XP_022883389.1/1-283 --LQDNVATMARQYVRSVISSVQRVS---
XP_009776589.1/1-283 --LQDNVATMARQYVRSVVSSVQRVA---
NP_001312504.1/1-283 --LQDNVATMARQYVRSVVSSVQRVA---
XP_016472209.1/1-283 --LQDNVATMARQYVRSVVSSVQRVA---
XP_019255429.1/1-283 --LQDNVATMARQYVCSVVSSVQRVA---
XP_009624234.1/1-283 --LQDNVATMARQYVRSVVSSVQRVA---
XP_016504967.1/1-283 --LQDNVATMARQYVRSVVSSVQRVA---
XP_016547131.1/1-283 --LQENVATMARQYVRSVVSSVQRVA---
XP_004251138.1/1-283 --LQDNVATMARQYVRSVVSSVQRVA---
XP_015058512.1/1-283 --LQDNVATMARQYVRSVVSSVQRVA---
XP_006340193.1/1-283 --LQDNVATMARQYVRSVVSSVQRVA---
XP_004235650.1/1-283 --MQESVASMARQYVRSIISSVQRVA---
XP_006343047.1/1-283 --MQESVASMARQYVRSIISSVQRVA---
XP_009774805.1/1-285 --MQENVASMARQYVRSIISSVQRVA---
XP_016498616.1/1-285 --MQENVASMARQYVRSIISSVQRVA---
XP_016434103.1/1-285 --MQENVASMARQYVRSIISSVQRVA---
XP_009593967.1/1-285 --MQENVASMARQYVRSIISSVQRVA---
XP_004510297.1/1-285 --MQENVACMARQYVRSIISSVQRVA---
XP_004510296.1/1-285 --MQENVACMARQYVRSIISSVQRVA---
XP_003627005.1/1-284 --MQENVACMARQYVRSIISSVQRVA---
XP_003531653.1/1-284 --MQEHVAAMARQYVRSIISSVQRV----
XP_006585576.1/1-288 --MQEHVAAMARQYVRSIISSVQRV----
XP_014634618.1/1-288 --MQEHVAAMARQYVRSIISSVQRV----
XP_014634619.1/1-288 --MQEHVAAMARQYVRSIISSVQRV----
XP_003531652.1/1-285 --MQEHVTSMARQYVRSIISSVQRVA---
XP_003530109.1/1-284 --MQEHVASMARQYVRSIISSVQRVA---
XP_007135643.1/1-285 --MQDHVASMARQYVRSIISSVQRVA---
XP_003530112.1/1-285 --MQEHVASMARQYVRSIISSVQRVA---
XP_020226142.1/1-285 --MQEHVASMARQYVRSIISSVQRVA---
XP_014516466.1/1-285 --MQEHVASMARQYVRSIISSVQRVA---
XP_017407594.1/1-285 --MQEHVASMARQYVRSIISSVQRVA---
XP_019448178.1/1-285 --MQEHVASMARQYVRSIISSVQRVA---
XP_019429328.1/1-285 --MQDHVASMARQYVRSIISSVQRVA---
XP_004486776.1/1-285 --MQEHVASMARQYVRSIISSVQRVA---
XP_003597690.1/1-286 --MQDHVASMARQYVRSIISSVQRVA---
XP_015932679.1/1-285 --MQEHVASMARQYVRSIISSVQRVA---
XP_025674177.1/1-285 --MQEHVASMARQYVRSIISSVQRVA---
XP_025610204.1/1-285 --MQEHVASMARQYVRSIISSVQRVA---
XP_020226055.1/1-285 --MQEHVASMARQYVRSIISSVQRVA---
XP_007150614.1/1-285 --MQDHVASMARQYVRSIISSVQRVA---
XP_014501306.1/1-285 --MQDHVASMARQYVRSIISSVQRVA---
XP_017425270.1/1-285 --MQDHVASMARQSVRSIISSVQRVA---
XP_021836126.1/1-285 --LQDNVASMARQYVRSIISSVQRVA---
XP_010691423.1/1-284 --LQDNVATMARQYVRSIISSVQRVA---
XP_010691424.1/1-284 --LQDNVATMARQYVRSIISSVQRVA---
XP_021765330.1/1-284 --LQDNVASMARQYVRSIISSVQRVA---
XP_021749885.1/1-284 --LQDNVASMARQYVRSIISSVQRVA---
XP_010267183.1/1-285 --LQENVASMARQYVRSIISSVQRVA---
XP_017258627.1/1-285 --MQENVASMARQYVRSIISSVQRVA---
XP_023000315.1/1-284 --MQEHVASMARQYVRSIISSVQRVA---
XP_023000313.1/1-284 --MQEHVASMARQYVRSIISSVQRVA---
XP_023514554.1/1-284 --MQEHVASMARQYVRSIISSVQRVA---
XP_023514552.1/1-284 --MQEHVASMARQYVRSIISSVQRVA---
XP_022964458.1/1-284 --MQEHVASMARQYVRSIISSVQRVA---
XP_022964454.1/1-284 --MQEHVASMARQYVRSIISSVQRVA---
XP_022147627.1/1-284 --MQEHVASMARQYVRSIISSVQRVA---
XP_022147619.1/1-284 --MQEHVASMARQYVRSIISSVQRVA---
XP_008462437.1/1-284 --MQEHVASMARQYVRSIISSVQRVA---
XP_004141659.1/1-284 --MQEHVASMARQYVRSIISSVQRVA---
XP_008462436.1/1-284 --MQEHVASMARQYVRSIISSVQRVA---
XP_011659682.1/1-284 --MQEHVASMARQYVRSIISSVQRVA---
XP_022992111.1/1-284 --MQEHVASMARQYVRSIISSVQRVA---
XP_023548534.1/1-284 --MQEHVASMARQYVRSIISSVQRVA---
XP_022953371.1/1-284 --MQEHVASMARQYVRSIISSVQRVA---
XP_002304217.1/1-285 --MQEHVASMTRQYIRSIISSVQRVA---
XP_011013041.1/1-285 --MQEHVASMTRQYIRSIISSVQRVA---
XP_011013042.1/1-285 --MQEHVASMTRQYIRSIISSVQRVA---
XP_011046759.1/1-284 --MQEHVASMARQYIRSIISSVQRVA---
XP_024464085.1/1-284 --MQEHVASMARQYIRSIISSVQRVA---
XP_024183498.1/1-286 --MQEHVASMARQYVRSIISSVQRVA---
XP_004302866.1/1-286 --MQEHVASMARQYVRSIISSVQRVA---
NP_001280898.1/1-285 --MQEHVASMARQYVRSIISSVQRVA---
XP_007214960.1/1-285 --MQEHVASMARQYVRSIISSVQRVA---
XP_008228437.1/1-285 --MQEHVASMARQYVRSIISSVQRVA---
XP_021818763.1/1-285 --MQEHVASMARQYVRSIISSVQRVA---
XP_022723515.1/1-285 --MQEHVASMARQYVRSIISSFQRVA---
XP_016688508.1/1-285 --LQEHVASMARQYVRSIISSVQRVA---
XP_012443274.1/1-285 --LQEHVASMARQYVRSIISSVQRVA---
XP_022721457.1/1-285 --MQEHVASMARQYVRSIISSVQRVA---
XP_022715018.1/1-285 --MQEHVASMARQYVRSIISSVQRVA---
XP_017979082.1/1-285 --MQEHVASMARQYVRSIISSVQRVA---
XP_021293768.1/1-285 --MQEHVASMARQYVRSIISSVQRVA---
XP_010062518.1/1-285 --MQEHVASMARQYVRSIISSVQRVA---
[truncated: 112,204 more chars]
